# Dysadherin awakens mechanical forces and promotes colorectal cancer progression

**DOI:** 10.7150/thno.72354

**Published:** 2022-05-21

**Authors:** So-Yeon Park, Choong-Jae Lee, Jang-Hyun Choi, Jee-Heun Kim, Won-Jae Lee, Tae-Young Jang, So-El Jeon, Jae-Hyun Kim, Sang-Hee Cho, Ji-Shin Lee, Jeong-Seok Nam

**Affiliations:** 1 School of Life Sciences, Gwangju Institute of Science and Technology, Gwangju, 61005, Republic of Korea; 2 Cell Logistics Research Center, Gwangju Institute of Science and Technology, Gwangju, 61005, Republic of Korea; 3 Department of Hemato-oncology, Chonnam National University Medical School, Gwangju, 61469, Republic of Korea; 4 Department of Pathology, Chonnam National University Medical School, Gwangju, 61469, Republic of Korea

**Keywords:** Dysadherin, Mouse Model, Colorectal Cancer, Fibronectin, Yes-associated Protein 1

## Abstract

**Rationale**: Dysadherin is a tumor-associated, membrane-embedded antigen found in multiple types of cancer cells, and associated with malignant behavior of cancer cells; however, the fundamental molecular mechanism by which dysadherin drives aggressive phenotypes of cancer is not yet fully determined.

**Methods**: To get a mechanistic insight, we explored the physiological relevance of dysadherin on intestinal tumorigenesis using dysadherin knockout mice and investigated its impact on clinicopathological features in patients with advanced colorectal cancer (CRC). Next, to discover the downstream signaling pathways of dysadherin, we applied bioinformatic analysis using gene expression data of CRC patient tumors and dysadherin knockout cancer cells. Additionally, comprehensive proteomic and molecular analyses were performed to identify dysadherin-interacting proteins and their functions.

**Results**: Dysadherin deficiency suppressed intestinal tumorigenesis in both genetic and chemical mouse models. Moreover, increased dysadherin expression in cancer cells accounted for shorter survival in CRC patients. Comprehensive bioinformatics analyses suggested that the effect of dysadherin deletion is linked to a reduction in the extracellular matrix receptor signaling pathway. Mechanistically, the extracellular domain of dysadherin bound fibronectin and enhanced cancer cell adhesion to fibronectin, facilitating the activation of integrin-mediated mechanotransduction and leading to yes-associated protein 1 activation. Dysadherin-fibronectin interaction promoted cancer cell growth, survival, migration, and invasion, effects collectively mediated the protumor activity of dysadherin.

**Conclusion**: Our results highlight a novel function of dysadherin as a driver of mechanotransduction that stimulates CRC progression, providing a potential therapy strategy for CRC.

## Introduction

Dysadherin is a cancer-associated antigen and cell membrane glycoprotein with an FXYD motif. While dysadherin is frequently overexpressed in a broad range of human cancers, including thyroid, esophageal, gastric, colorectal, pancreatic, cervical, testicular, breast, and head and neck tumors, dysadherin surface expression is limited in normal cells, and dysadherin is rarely expressed on non-neoplastic cells 1-11. Dysadherin has been identified as a strong inducer of cancer invasion and metastasis. More specifically, clinical studies have indicated that high dysadherin expression in tumor tissues is significantly correlated with clinicopathological variables such as distant metastasis, recurrence, and low survival rate 2, 3, 5, 6, 8. Consistent with these studies, ectopic overexpression (OE) and gene silencing of dysadherin in cancer cells demonstrated that dysadherin promotes both single-cell and collective cell migration through down-regulation of cell-cell adhesion proteins and up-regulation of cytokine production 7, 12, 13. However, dysadherin has a short cytoplasmic tail without enzymatic activity; thus, the associated molecular mechanism remains ambiguous. Moreover, the potential physiological relevance of dysadherin to tumorigenesis has not yet been determined.

Extracellular matrix (ECM) molecules trigger a variety of critical signaling cues and play a key role in the regulation of cellular phenotype and behavior 14. During tumor development, reciprocal interaction between ECM and cancer cells constantly occurs and leads to changes in cell structure, adhesion properties, and response to signals from ECM proteins 15. Some of these changes of cellular phenotype are accomplished by the cells' availability to sense mechanical forces, which are then converted into biochemical signals within the cell, leading to a number of cellular mechanisms being activated, including cell adhesion, proliferation, survival, and migration 16. Abnormalities in mechanotransduction lead to aberrant cell behavior and activation of key signaling pathways to drive phenotype switching from normal cells into more mesenchymal-like cancer cells, and can allow cancer cells to achieve further malignant transformation and overcome stromal challenges during invasion and metastasis 17. Thus, a better understanding the conversion of mechanical cues into biochemical signals is required to discover new therapeutic targets for cancer treatment.

Mouse models play a vital role in understanding gene function. A large number of cancer researchers have relied on knockout (KO) mouse models to determine the biological functions of genes or to identify target genes with phenotypes of interest. In this study, we established dysadherin-KO mice for the first time and examined the significance of dysadherin in adenomatous polyposis coli mutation-induced (*Apc*^Min/+^) and chemically induced mouse models of intestinal tumorigenesis 18, 19. To establish the mechanism of dysadherin in intestinal cancer, we conducted comprehensive bioinformatics analyses and found an association between dysadherin and ECM-cell signaling. We thus investigated dysadherin-interacting ECM proteins and downstream signaling pathways. This study provides strong evidence for a novel role of dysadherin in aberrant signal transduction that fuels tumorigenesis.

## Methods

### Ethical approval

Prior approval for animal studies was obtained from the Institutional Animal Care and Use Committee (IACUC) of the Gwangju Institute of Science and Technology (GIST, No. GIST2018-049). Analysis of dysadherin expression in patients with colorectal cancer (CRC) was preapproved by the Institutional Review Board at GIST (No. 20200108-BR-50-07-02). All work related to human tissues was conducted in accordance with the Helsinki Declaration. Written informed consent was obtained from all participants prior to the study. *In vitro* experiments were all performed on at least three separate occasions. Exclusion criteria were not applied in this study; thus outliers were included in all experiments.

### Animal models

Establishment of dysadherin-KO mice and the detailed methods are described in in the Supplementary Materials and Methods. Briefly, the incidence of tumor-bearing mice was determined by intestinal observation under a stereomicroscope after methylene blue staining. Mouse intestinal tumors were divided into 3 groups based on tumor diameter: small tumors, < 3 mm; medium tumors, ≥ 3 mm and < 5 mm; and large tumors, ≥ 5 mm. Tumor load was calculated according to the following formula described in a previous report 20: tumor load = (number of small tumors) × 1 + (number of medium tumors) × 2 + (number of large tumors) × 3. To compare the tumor-forming potential of empty vector-transfected control cells and dysadherin-KO cells, an *in vivo* limiting dilution assay was performed as described in a previous report 21. A splenic injection experiment was performed to estimate metastasis and distant organ colonization 22. In this model, empty vector-transfected control cells or dysadherin-KO cells were tagged with luciferase and inoculated into the spleens of NSG mice (NOD.Cg-Prkdc^scid^ Il2rg^tm1Wjl^/SzJ, #005557, Jackson Laboratory, Bar Harbor, ME, USA) followed by splenectomy. Detailed information is provided in the Supplementary Materials and Methods. The exact number of mice for each experiment is noted in the figure legends.

### *Apc*^Min/+^ mouse polyp-derived tumoroid culture

Single cells were isolated from the intestinal polyps of 20-week-old *Apc*^Min/+^ mice and cultured as described in a previous report with slight modifications 23. The detailed methods are described in in the Supplementary Materials and Methods.

### Clinical analysis and statistics

Tissue microarray slides from 123 patients with CRC were immunostained to detect dysadherin using a specific monoclonal antibody (M53) as previously reported 2. The slides contained 3 tumor tissue cores and 2 matched normal tissue cores from each patient. Immunohistochemistry and scoring were performed blindly as described in the Supplementary Materials and Methods. Patients were divided into two group according to the integrated optical density values; dysadherin-high (≥ 75%, n = 27) and dysadherin-low (< 75%, n = 96). Survival was calculated using the Kaplan-Meier method, and comparisons were made using log-rank tests. Factors associated with recurrence-free survival (RFS) and overall survival (OS) were identified by univariate and multivariate Cox proportional hazards regression models with hazard ratios and 95% confidence intervals. Statistical analyses were performed using SPSS version 21.0 (IBM Corporation, Armonk, NY, USA). Normal and tumor tissue samples from 187 patients with CRC were used to compare the dysadherin mRNA levels. Protein lysates of normal and tumor tissues from 7 patients with CRC were subjected to Western blot analyses to compare dysadherin protein levels.

### Establishment of dysadherin-KO or -OE cell lines and *in vitro* studies

Vector transfection, cell line generation, and validation were performed as described in the Supplementary Materials and Methods. *In vitro* assays, including cell growth and apoptosis assays and the Boyden chamber assay, were performed as described in the Supplementary Materials and Methods.

### Bioinformatics analyses

To compare the *FXYD5* mRNA expression levels between normal and tumor tissues, gene expression data were obtained from an open-source database (R2: Genomic analysis and visualization platform, https://hgserver1.amc.nl/cgi-bin/r2/main.cgi). Gene set enrichment analysis (GSEA) was conducted as described in our previous study 24 using the transcriptomic data of CRC patients (GSE21510). Patients were divided into two group according to the dysadherin expression level; dysadherin-high (≥ 50%, n = 52) and dysadherin-low (< 50%, n = 52). Ingenuity Pathway Analysis was performed as described in the Supplementary Materials and Methods using RNA-sequencing data from dysadherin-KO SW480 cells to identify the potential diseases, functions, and upstream regulators that are significantly associated with dysadherin.

### Identification of dysadherin-interacting proteins

Potential dysadherin-interacting proteins were identified in whole-protein lysates of SW480 cells (KCB Cat# KCB 200848YJ) using M53 monoclonal antibody-based co-immunoprecipitation (co-IP) followed by liquid chromatography with tandem mass spectrometry (LC-MS).

### Statistical analyses

All results are expressed as the mean ± standard error of the mean. Statistical comparisons of data from 2 groups were carried out by Student's t-test or two-way ANOVA with the Bonferroni multiple comparison test, and statistical comparisons of 3 or more groups were carried out by one-way ANOVA with the Dunnett's multiple comparison test using GraphPad Prism (GraphPad Software Inc, San Diego, California, USA). The log-rank test was used for Kaplan-Meier analysis using SPSS version 21.0 (IBM Corp., Armonk, NY, USA). The specific numbers of biological replicates are provided in the figure legends. Asterisks are used to indicate statistical significance. *, **, and *** indicate *p* < 0.05, < 0.01, and < 0.001, respectively.

Other detailed materials and methods are described in supplementary materials. The list of primers used for real-time polymerase chain reaction, small interfering RNA (siRNA) sequence and antibodies are provided in table S1-S3, respectively.

## Results

### Deletion of dysadherin attenuates intestinal tumorigenesis

**Fig 1:**
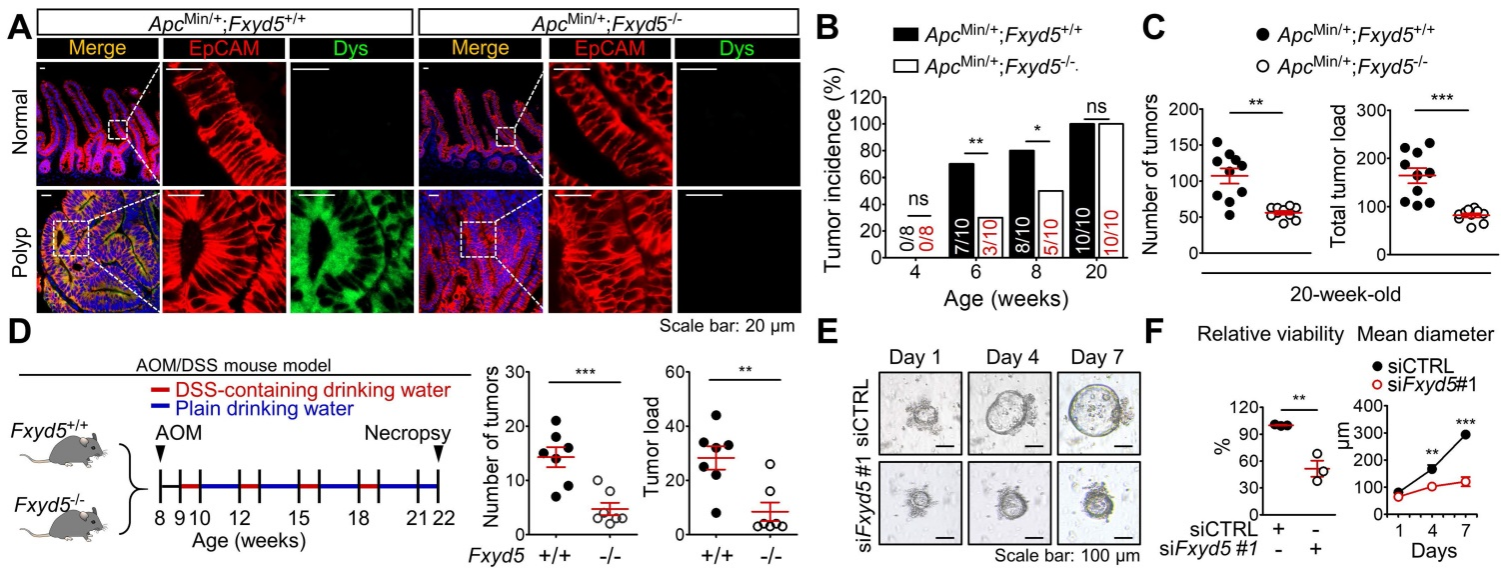
** Dysadherin deficiency inhibits intestinal tumorigenesis.** (**A**) IF showing the acquisition of dysadherin expression in the intestinal tumor epithelium (EpCAM^+^) of *Apc*^Min/+^;*Fxyd5*^+/+^ mice and complete elimination of dysadherin expression in the intestinal tumor epithelium of *Apc*^Min/+^;*Fxyd5*^-/-^mice. (**B**) Tumor incidence in *Apc*^Min/+^;*Fxyd5*^+/+^ and *Apc*^Min/+^;*Fxyd5*^-/-^ mice at the indicated ages (# of tumor-bearing mice/# of examined mice). (**C**) Number of intestinal tumors and total tumor load per mouse (*Apc*^Min/+^;*Fxyd5*^+/+^, n = 12; *Apc*^Min/+^;*Fxyd5*^-/-^, n = 20). (**D**) Left: schematic view of AOM/DSS-induced intestinal tumorigenesis model. Right: number of colonic tumors and total tumor load in 22-week-old AOM/DSS mice (*Fxyd5*^+/+^, n = 7; *Fxyd5*^-/-^, n = 7). (**E**) Representative images showing intestinal tumoroids derived from *Apc*^Min/+^ mice subjected to knockdown of *Fxyd5*. (**F**) Effects of dysadherin knockdown on tumoroid viability and size (n = 3/group). In all panels, data are reported as means ± SEMs; *, **, and *** indicate *p* < 0.05, < 0.01, and < 0.001, respectively; ns indicates no significance. Statistical comparisons between 2 groups were performed using Student's t-test or two-way ANOVA with the Bonferroni multiple comparison test.

To verify the role of dysadherin in intestinal tumorigenesis, we established dysadherin-KO (*Fxyd5*^-/-^) mice by deleting exons 2-7 of *Fxyd5* with CRISPR/Cas9 technology (Figure S1A-D). *Fxyd5*^-/-^ mice were viable without any discernible phenotype, were born at a Mendelian ratio (Figure S1E), and did not present with any intestinal abnormalities (Figure. S1F). In the *Apc*^Min/+^ mouse model 18, 25, histological assessment of *Apc*^Min/+^ mice revealed the acquisition of dysadherin expression in epithelial cell adhesion molecule-positive (EpCAM^+^) epithelial cells residing within intestinal tumors, whereas no dysadherin expression was observed in epithelial cells within normal intestines (**Figure 1A**). The complete absence of dysadherin expression in the intestinal tumor epithelium of *Apc*^Min/+^;*Fxyd5^-^*^/-^ mice was confirmed (**Figure 1A**). Of note, dysadherin deletion did not completely block *Apc*^Min/+^-driven intestinal tumorigenesis but delayed tumor development, as indicated by a decrease in tumor incidence in younger mice (6- and 8-week-old; **Figure 1B**). Consistently, in older (20-week-old) mice, the number of tumors and total tumor load were significantly decreased by dysadherin deletion (**Figure 1C** and Figure S2A). Further histological analyses showed a reduction in cell proliferation and an increase in cell death in dysadherin-deficient tumor cells (Supplementary Figure S2B). Approximately 6.3% of tumors from the 20-week-old *Apc*^Min/+^ mice showed local invasion through a fissure of the mucosal muscle layer; however, all tumors in the *Apc*^Min/+^;*Fxyd5*^-/-^ mice remained above the mucosal muscle layer and thus presented with a noninvasive phenotype (Figure S2C,D). In the azoxymethane/dextran sodium sulfate (AOM/DSS)-induced intestinal tumorigenesis mouse model^19^, ^26^, we also detected the presence of dysadherin expression in EpCAM^+^ intestinal tumor epithelial cells, whereas no dysadherin expression was observed in epithelial cells within normal intestines (Figure S2E). Additionally, we repeatedly confirmed the absence of dysadherin expression in the tumor epithelium of *Fxyd5*^-/-^ mice (Figure S2E). Of note, dysadherin deletion also significantly reduced both the number of tumors and total tumor load (**Figure 1D** and Figure S2F), suggesting that dysadherin expression contributes to intestinal tumorigenesis in both the genetically and chemically induced mouse models of intestinal cancer.

Conversely, when we explored the stromal effect of dysadherin depletion on tumorigenesis by inoculating murine intestinal tumor cells (MC38) 27 into *Fxyd5*^+/+^ and *Fxyd5*^-/-^ mice, we found that dysadherin-KO physiological environment did not affect tumor seeding and growth (Figure S3A-C). Additionally, blood analyses did not show hematological differences between *Fxyd5*^+/+^ and *Fxyd5*^-/-^ mice (Figure S3D). Although these data cannot completely exclude the potential role of dysadherin in stromal cellular compartments during tumorigenesis, they inspired us to focus on the role of dysadherin in tumor epithelial cells in further mechanistic studies. Moreover, the majority of CRC cells are known to originate from the intestinal epithelial cells of the colorectal mucosa that acquire advantages of clonal growth and expansion during tumor development 28. Thus, to validate the effect of dysadherin deficiency on the growth of the tumor epithelium, we cultured intestinal tumoroids derived from the polyps of 20-week-old *Apc*^Min/+^ mice and silenced dysadherin expression using siRNAs. First, we tested the effectiveness of siRNAs targeting mouse dysadherin (si*Fxyd5*) in MC38 cells and chose the most effective sequence (Figure S4A,B). We found that dysadherin silencing significantly suppressed the growth of intestinal tumoroids (**Figure 1E**,**F**), confirming the importance of acquired dysadherin expression during growth of tumor epithelium.

**Fig 2:**
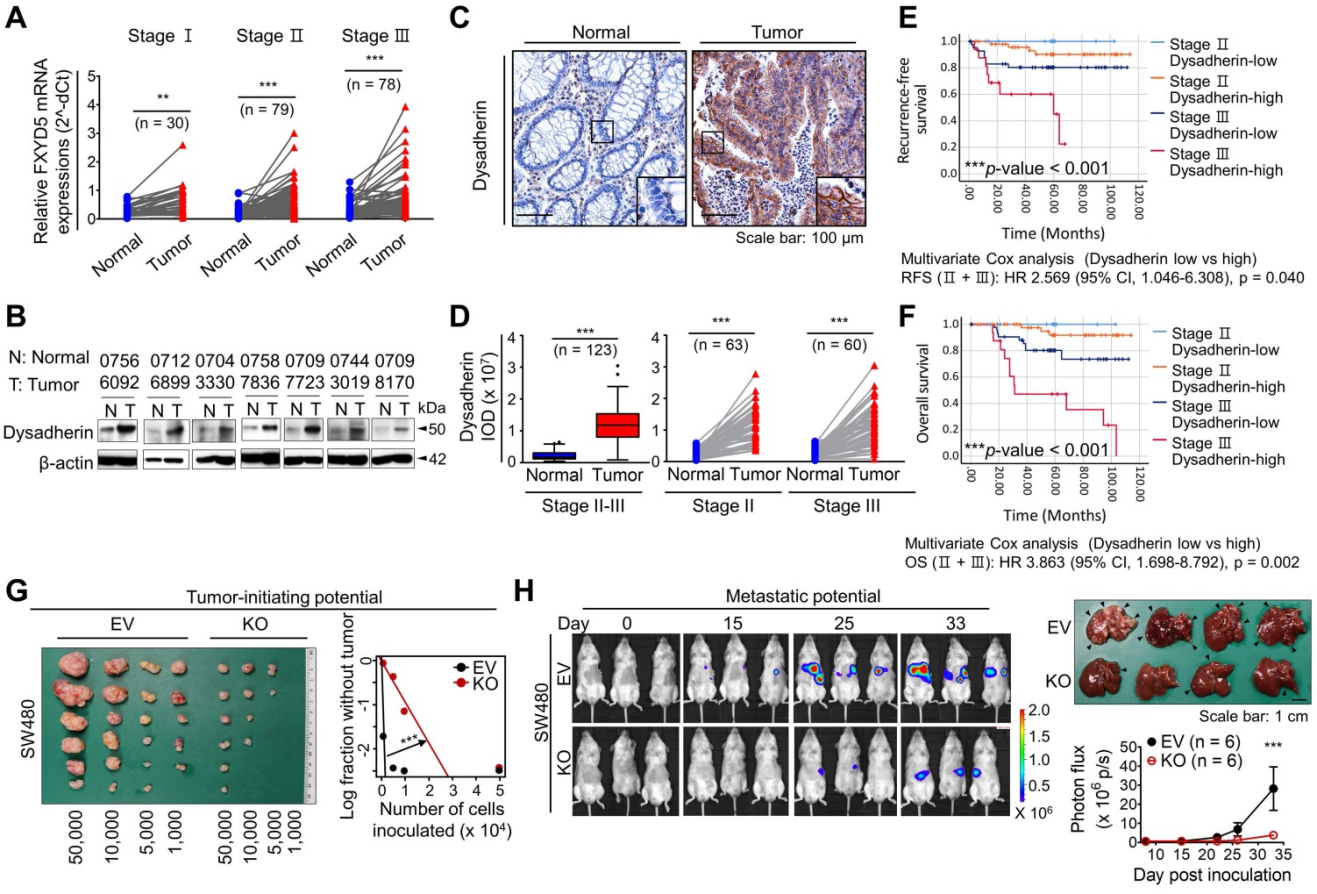
** Clinical implications of dysadherin expression in CRC patients and it pleiotropic role within CRC cells.** (**A**) mRNA expression of dysadherin (FXYD5) was measured by real-time RT-qPCR in tumor tissues and matched adjacent normal tissues (total n = 187, Stage I n = 30, Stage II n = 79, Stage III n = 78). Statistical significance was determined by a paired Student's t-test. (**B**) Representative immunoblots showing dysadherin protein expression in tumor and matched normal adjacent tissues from 7 patients with CRC. (**C**) Representative immunohistochemical staining of dysadherin in tumors and matched normal tissues from patients with CRC. (**D**) Graphs showing the integrated optical density (IOD) of dysadherin protein levels within the epithelium in the indicated group. Statistical significance was determined by a paired Student's t-test. (**E**,**F**) Kaplan-Meier survival analysis of patients with CRC. Patients were divided into four groups according to stage (stage II and III) and dysadherin expression (high and low). Statistical significance was determined by log-rank tests. (**G**) LDA was performed to compare the tumor-forming potential. Different numbers of SW480 cells with and without KO of dysadherin were inoculated *s.c.* into NOD.Cg-*Prkdc*^scid^/J mice (n = 6/group). (**H**) Luciferase-labeled SW480 cells with and without KO of dysadherin were inoculated into the spleens of NOD.Cg-*Prkdc*^scid^/J mice (n = 6/group). Metastatic tumor formation was observed via bioluminescence and necropsy. In all panels, data are reported as means ± SEMs; *, **, and *** indicate *p* < 0.05, < 0.01, and < 0.001, respectively. Statistical comparisons between 2 groups were performed using Student's t-test or two-way ANOVA with the Bonferroni multiple comparison test, or using one-way ANOVA with Dunnett's multiple comparison tests for 3 or more groups. EV: empty vector.

Consistent with the mouse experiments, our clinical investigation of 187 patients with CRC revealed an increase in dysadherin at both the mRNA and protein levels in tumor tissues versus matched normal tissues (**Figure 2A,B**). Analysis of an open-source genomic database (R2) further confirmed the increase in dysadherin mRNA levels in tumors of CRC patients (Figure S5). Histopathological analyses of tissues from 123 patients with CRC confirmed that dysadherin was not expressed in the normal epithelium, but its expression was increased in the tumor epithelium (**Figure 2C,D**). Interestingly, the extent of the increase in dysadherin expression in the tumor epithelium was significantly correlated with tumor T stage and recurrence (Table S4), and dysadherin expression was an independent and significant prognostic marker of poor clinical outcomes, short OS and RFS in patients with CRC (**Figure 2E,F**).

### Dysadherin plays a pleiotropic role in CRC cells

The combination of increased dysadherin expression and reduced E-cadherin expression is known to reflect tumor aggressiveness and is considered to be a prognostic marker of poor clinical outcomes in patients with a broad range of cancers 1, 2, 4. Consistent with these clinical observations, immunoblot analysis of a human CRC cell line panel showed a significant tendency towards increased dysadherin expression and decreased membrane E-cadherin expression (Figure S6A). Additionally, we repeatedly confirmed a significant decrease in E-cadherin expression in a normal human colonic epithelial cell line (NCM460D) after dysadherin overexpression (Figure S6B), suggesting the importance and biological relevance of dysadherin to E-cadherin. For our mechanistic study, we deleted dysadherin in SW480 cells, which express the highest levels of dysadherin among CRC cell lines. We also overexpressed dysadherin in HCT116 cells, which express the lowest levels of dysadherin. Dysadherin KO attenuated the growth of SW480 cells, and dysadherin OE promoted the growth of HCT116 cells (Figure S6C). In clonogenic assays, dysadherin KO reduced the survival potential of SW480 cells, whereas dysadherin OE increased the survival potential of HCT116 cells (Figure S6D). Also, apoptosis was increased upon dysadherin deletion and reduced upon dysadherin OE (Figure S6E), consistent with our previous observations in breast and liver cancer cells 12, 13, 29. Moreover, Boyden chamber assays with or without Matrigel coating revealed that dysadherin OE promotes the invasive and chemotactic migration of CRC cells, whereas dysadherin KO suppresses these aggressive phenotypes (Figure S6F,G). Next, we examined the *in vivo* function of dysadherin in CRC xenograft mouse models. A limiting dilution assay confirmed the reduction in the tumor-initiating potential of CRC cells upon dysadherin deletion (**Figure 2G**). This result may reflect that dysadherin plays a critical role in determining the tumorigenic capacity of CRC cells, as observed in genetically and chemically induced CRC mouse models (**Figure 1B-D**). In addition, a splenic injection mouse model confirmed that dysadherin deficiency reduced the metastatic potential of injected CRC cells to the liver (**Figure 2H**). Collectively, these data suggest that dysadherin expression is required for diverse processes in CRC cells, including growth, survival, migration, and invasion.

### The ECM-integrin pathway is a key dysadherin signaling pathway

**Fig 3:**
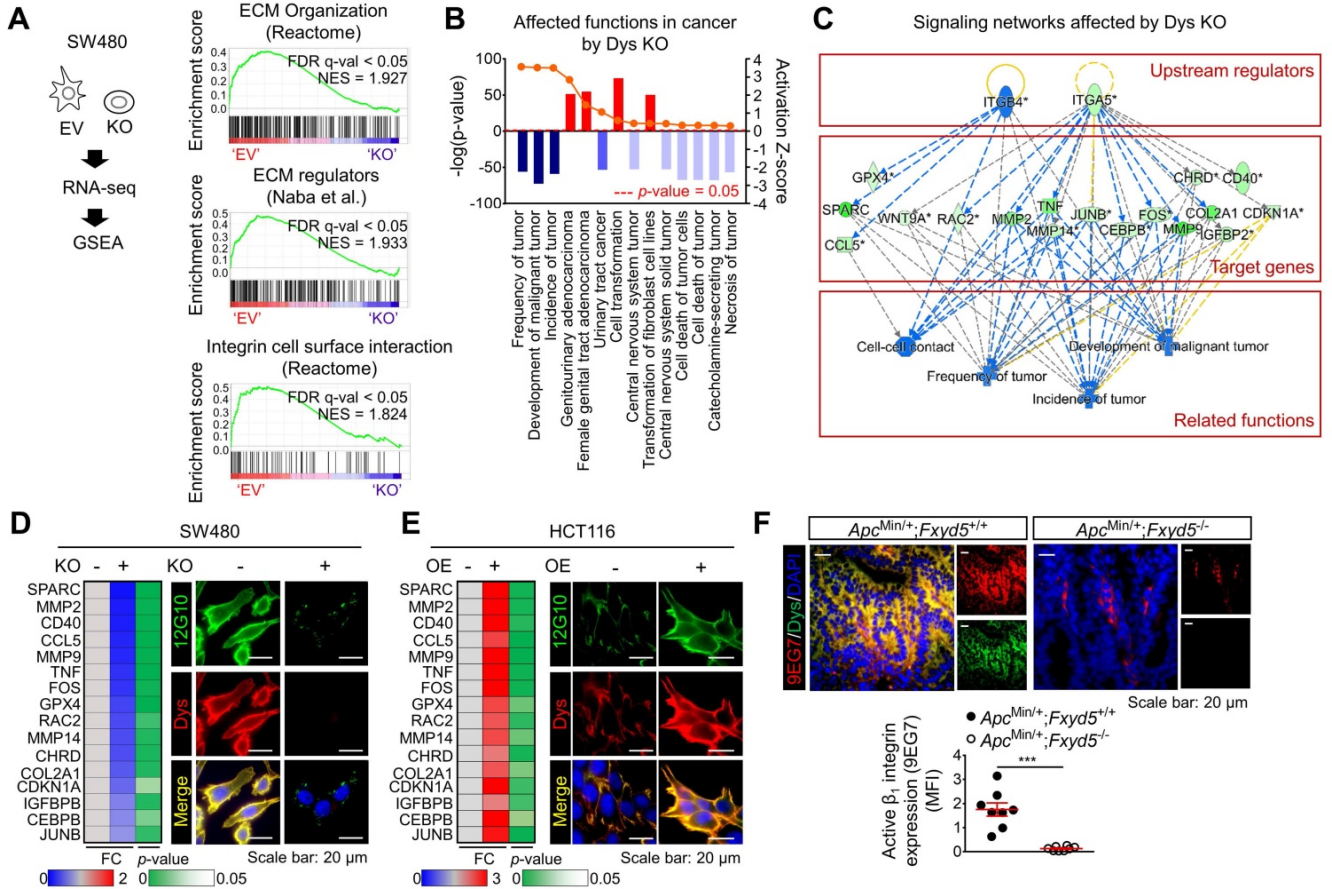
** The ECM-integrin pathway is a potential downstream mediator of dysadherin with tumorigenesis.** (**A**) GSEA was performed using the mRNA sequencing profiles of dysadherin-KO and control (EV-transfected) cells. Gene signatures associated with the ECM-integrin pathway were significantly enriched in control cells compared with dysadherin-KO cells. (**B**,**C**) Ingenuity Pathway Analysis was performed with the list of differentially expressed genes in dysadherin-KO cells to reveal the dysadherin-associated mechanism. (**B**) Disease and function analyses show significant reductions in tumor frequency, tumor incidence, and malignant tumor development upon dysadherin KO. Categories with *p* < 0.05 and |z-score| > 2 were considered statistically significant. (**C**) Upstream analysis indicates the potential link between integrin pathways and reduced cancer-related functions, with significant reductions in integrin target gene expression upon dysadherin KO, which collectively led to a decrease in tumor development. (**D**,**E**) The potential relationship between dysadherin and the integrin signaling pathway, validated by RT-qPCR analyses of integrin target genes and by IF in SW480 (**D**) and HCT116 (**E**) cell lines. RT-qPCR heatmaps show changes in integrin signaling target genes upon dysadherin KO (**D**) and OE (**E**). IF staining for human active β_1_ integrin (12G10) and dysadherin in dysadherin-KO or -OE CRC cells. (**F**) IF staining for murine active β_1_ integrin (9EG7) and dysadherin in intestinal tumor tissues of *Apc*^Min/+^;*Fxyd*5^+/+^ and *Apc*^Min/+^;*Fxyd*5^-/-^ mice (n = 8/ group).*** indicates *p* < 0.001. Dys: dysadherin, FC: fold change, FDR: false discovery rate, MFI: mean fluorescence intensity, NES: normalized enrichment score, EV: empty vector.

To obtain further mechanistic insights, we compared the gene expression profiles of tumors from 104 CRC patients (GSE21510) 30 with higher dysadherin expression (dysadherin^high^) versus lower dysadherin expression (dysadherin^low^). The list of differentially expressed genes in the dysadherin^high^ tumors (Table S5) was subjected to GSEA (Figure S7A). Malignant gene signatures associated with poor clinical outcome, metastasis, and migration clusters were significantly enriched in the dysadherin^high^ tumors (Figure S7B). Intriguingly, gene signatures related to ECM receptor pathways such as ECM organization, ECM regulators, and integrin signaling, were also significantly up-regulated in the dysadherin^high^ tumors (Figure S7C). Consistent with the clinical data, GSEA of RNA-sequencing data from dysadherin-KO SW480 cells (n = 4,437, *p* < 0.05, Table S6) repeatedly confirmed the link between dysadherin and the ECM receptor pathway, as indicated by enrichment gene signatures related to ECM organization, ECM regulators, and integrin-cell surface interactions (**Figure 3A**). Next, we explored the potential molecular network involved in the relationship between dysadherin and ECM receptor pathways by Ingenuity Pathway Analysis of the differentially expressed genes in dysadherin-KO SW480 cells. The data revealed the diseases and functions most affected by dysadherin KO (Figure S7D). As expected, cancer was the disease most affected by dysadherin KO, which led to robust decreases in tumor frequency, tumor incidence, and malignant tumor development (**Figure 3B**). Analyses of upstream regulators further revealed that the reductions in these cancer-related functions are presumably modulated by a set of integrin signaling target genes (**Figure 3C**). Validation with CRC cells confirmed that integrin target gene expression tended to decrease upon dysadherin KO and increase upon dysadherin OE (**Figure 3D,E**). Next, we assessed integrin activation by immunofluorescence staining using an antibody that specifically recognizes the active conformation of β1 integrin (clone 12G10) 31. The extent of active β1 integrin was strikingly reduced by dysadherin KO but increased by dysadherin OE (**Figure 3D,E**). Using an antibody that specifically recognizes the active conformation of murine β_1_ integrin (clone 9EG7) 31, we confirmed the role of dysadherin in regulating β1 integrin activation in intestinal tumors of *Apc*^Min/+^;*Fxyd*5^-/-^ mice and *Apc*^Min/+^;*Fxyd*5^+/+^ mice, showing a striking reduction in β1 integrin activation in the absence of dysadherin (**Figure 3F**). Collectively, these comprehensive analyses suggest the potential involvement of ECM-integrin signaling in dysadherin-mediated intestinal tumorigenesis.

### Dysadherin directly binds fibronectin through its extracellular domain

**Fig 4:**
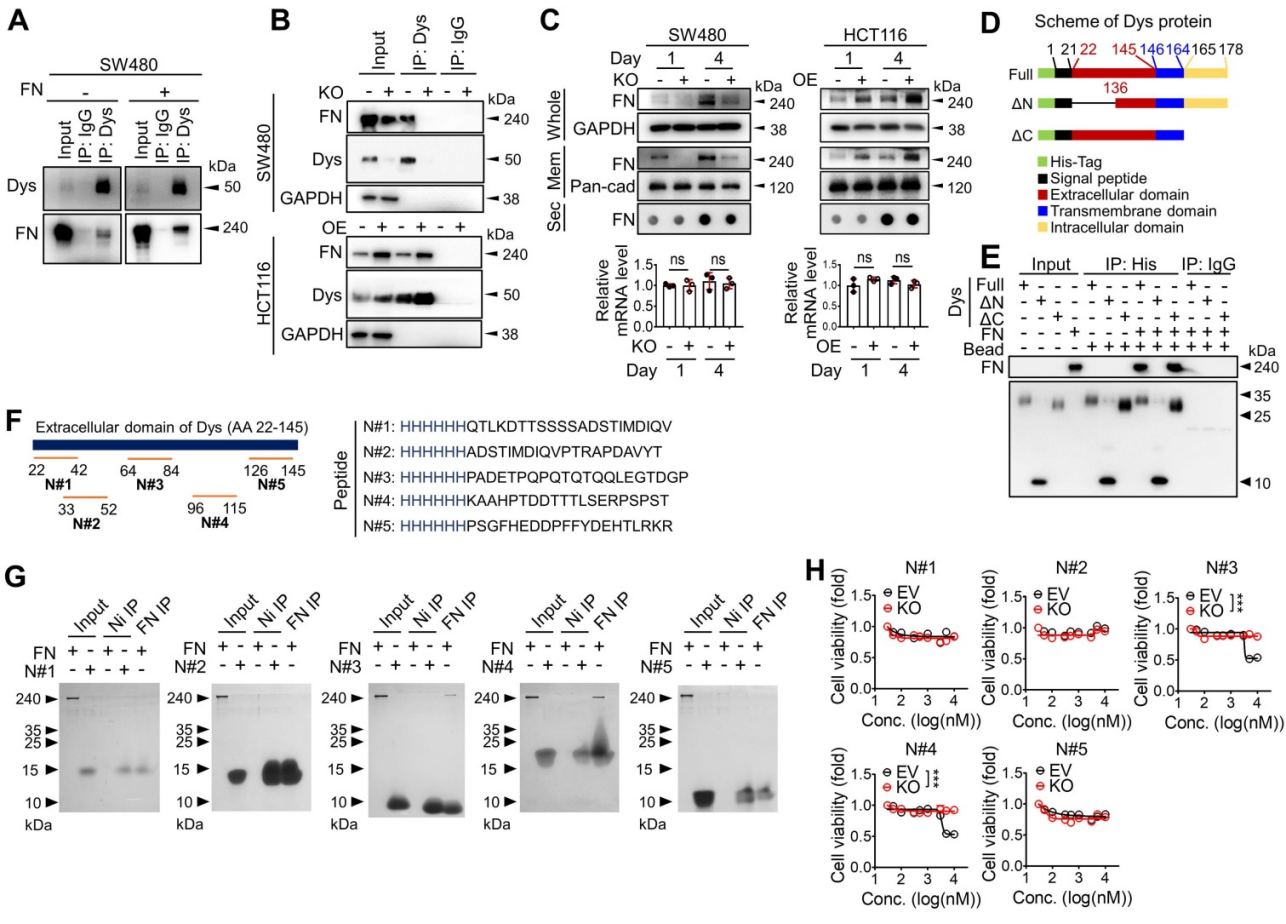
** The extracellular domain of dysadherin directly binds fibronectin.** (**A**) Co-IP with anti-dysadherin antibody (M53) and subsequent immunoblot analyses validate the binding of dysadherin to endogenous fibronectin in CRC cells. (**B**) Co-IP with M53 and subsequent immunoblot analyses confirm that the quantity of dysadherin-bound fibronectin is decreased by dysadherin KO but increased by dysadherin OE. (**C**) Whole-protein lysates and membrane fractions were extracted from cells at the indicated time points. Culture media were collected and applied to dot blot assays to compare the quantities of secreted fibronectin. RT-qPCR analyses were performed to compare fibronectin mRNA transcript levels (n = 3/group). (**D**) Schematic of full-length (wild-type) and mutant dysadherin proteins purified from *E. coli*. (**E**) Pull-down assay using various forms of purified His-tagged dysadherin proteins and purified fibronectin protein to determine direct protein-protein interactions. (**F**) The schematic shows the candidate sites and the sequences of peptides used to verify which could be bound with fibronectin. (**G**) Pull-down assay using purified fibronectin and purified His-tagged synthesized peptides to determine the specific sequence with fibronectin-binding activity. (**H**) Cell viability were measured by an MTT assay at 48 hours after the treatment of annotated peptide in SW480 cells with and without KO of dysadherin. In all panels, data are reported as means ± SEMs; ns indicates no significance. Statistical comparisons between 2 groups were performed using Student's t-test. Dys: dysadherin, FN: fibronectin, Mem: membrane, Sec: secreted, ΔC**:** ΔC-mutant, ΔN: ΔN-mutant.

To identify the downstream mechanisms through which dysadherin regulates intestinal tumorigenesis, potential dysadherin-interacting proteins in SW480 cell extracts were identified through co-IP with a monoclonal anti-dysadherin antibody (M53) 2 followed by LC-MS. A total of 301 proteins were pulled down with the M53 antibody and were identified as potential dysadherin-interacting proteins (Figure S8A and Table S7). Gene ontology analysis with DAVID, a web-based functional annotation platform 32, revealed that ECM proteins make up one of the top clusters of dysadherin-interacting proteins. Particularly among ECM proteins, fibronectin is known to act as a ligand for various integrin receptors, linking the ECM with intracellular signaling cascades. Fibronectin was significantly enriched among the proteins co-IPed with anti-dysadherin (Table S8). Thus, we conducted further investigation on the potential interaction between dysadherin and fibronectin. We confirmed a dysadherin-fibronectin interaction in SW480 cells by immunoblot analysis of anti-dysadherin co-IP samples (**Figure 4A**). Additionally, when we added exogenous fibronectin prior to anti-dysadherin co-IP, the quantity of dysadherin-bound fibronectin increased (**Figure 4A**). The dysadherin-fibronectin interaction was decreased upon dysadherin KO in SW480 cells, and was increased by dysadherin OE in HCT116 cells (**Figure 4B**).

To obtain deeper insights into this phenomenon, we compared the quantity of fibronectin in total cell protein extracts, membrane fraction extracts, and the secretome in the culture media and mRNA transcripts in dysadherin-KO and dysadherin-OE CRC cells (**Figure 4C**). Dysadherin-induced alteration of fibronectin protein levels was not due to changes in mRNA or secreted protein levels; rather, membrane-bound fibronectin was decreased upon dysadherin KO and increased upon dysadherin OE, suggesting that the dysadherin-fibronectin interaction enriches fibronectin at the cellular membrane. Thus, fibronectin became more abundant in CRC cell protein extracts over time (**Figure 4C**).

Dysadherin is a membrane protein that consists of a short C-terminal cytoplasmic tail, a transmembrane domain, and a long extracellular domain 33. This unusually long extracellular domain may facilitate interactions with other membrane proteins or ECM components, leading to the alteration of signaling dynamics. Thus, we further investigated the interaction between dysadherin and fibronectin by generating purified recombinant His-tagged dysadherin protein (Figure S8B). We generated 2 mutant forms of dysadherin (**Figure 4D**): one without the extracellular N-terminal sequence (amino acids 22-135, ΔN-mutant), and the other without the intracellular C-terminal sequence (amino acids 165-178, ΔC-mutant). A pull-down assay of these purified His-tagged proteins validated the direct binding of dysadherin to fibronectin; while the ΔC-mutant was still able to bind fibronectin, the ΔN-mutant lost the ability to bind fibronectin (**Figure 4E**). Next, we generated HCT116 cell lines that overexpressed 3 different forms of dysadherin conjugated with a His-tag: full length, ΔN-mutant, or ΔC-mutant (Figure S8C). IF revealed binding of fibronectin to full-length dysadherin and the ΔC-mutant form, as indicated by the colocalization of fibronectin and dysadherin on the cellular membrane, but the ΔN-mutant form did not interact with fibronectin at the cellular level (Figure S8D). In further analyses, to find the specific amino acid sequence involved in dysadherin-fibronectin binding, we synthesized several His-tagged peptides (N#1~#5) mimicking the amino acid sequences of dysadherin found in five different regions of extracellular domain (**Figure 4F**) and performed a pull-down assay with purified fibronectin. Of note, we found that a peptide N#3 (PADETPQPQTQTQQLEGTDGP) and N#4 (KAAHPTDDTTTLSERPSPST) displayed fibronectin-binding activity, while N#1, #2 and #5 did not (**Figure 4G**). In line with this result, only peptide N#3 and N#4 displayed a selective cytotoxicity against SW480 cells, while showing a limited effect against dysadherin KO SW480 cells (**Figure 4H**). Although further studies are required to determine the exact physicochemical interaction between dysadherin and fibronectin, our data suggest that the middle sequences within the extracellular domain of dysadherin may be important for its binding activity to fibronectin.

Dysadherin KO resulted in significant reduction of fibronectin on the cellular membrane in SW480 cells (Figure S8E). Consistent with these *in vitro* data, fibronectin was significantly enriched on the membranes of dysadherin-expressing tumor cells within *Apc*^Min/+^;*Fxyd5*^+/+^ mouse intestinal tumors, and the enrichment of fibronectin was significantly decreased in dysadherin-deficient mouse tumors (Figure S8F). These results suggest that dysadherin and fibronectin interact in tumor cells and that the extracellular domain of dysadherin is a key regulatory element involved in fibronectin binding.

### The dysadherin-fibronectin interaction facilitates sustained activation of the fibronectin-integrin-FAK axis

Fibronectin is a primary ECM component that mediates a wide variety of cellular interactions with the ECM and plays pleiotropic roles in diverse processes in cancer, such as cancer cell growth, migration, and invasion 15. Furthermore, fibronectin is an adhesive glycoprotein primarily involved in cellular adhesive interactions 34; thus, we investigated whether dysadherin expression affects fibronectin-mediated cell adhesion. First, we compared the adhesive capacity of dysadherin-OE HCT116 cells with various ECM protein coatings. We observed a significant tendency towards greater adhesive capacity with increasing concentrations of all tested ECM proteins (fibronectin, laminin, collagen type I and collagen type IV); however, dysadherin OE enhanced the adhesive capacity of HCT116 cells under fibronectin-coated conditions (Figure S9A). Similarly, dysadherin KO reduced the capacity of SW480 cells to adhere to fibronectin without affecting their capacity to adhere to laminin, collagen type I or collagen type IV (Figure S9B), suggesting the specific role of dysadherin in cell-to-fibronectin adhesion.

**Fig 5:**
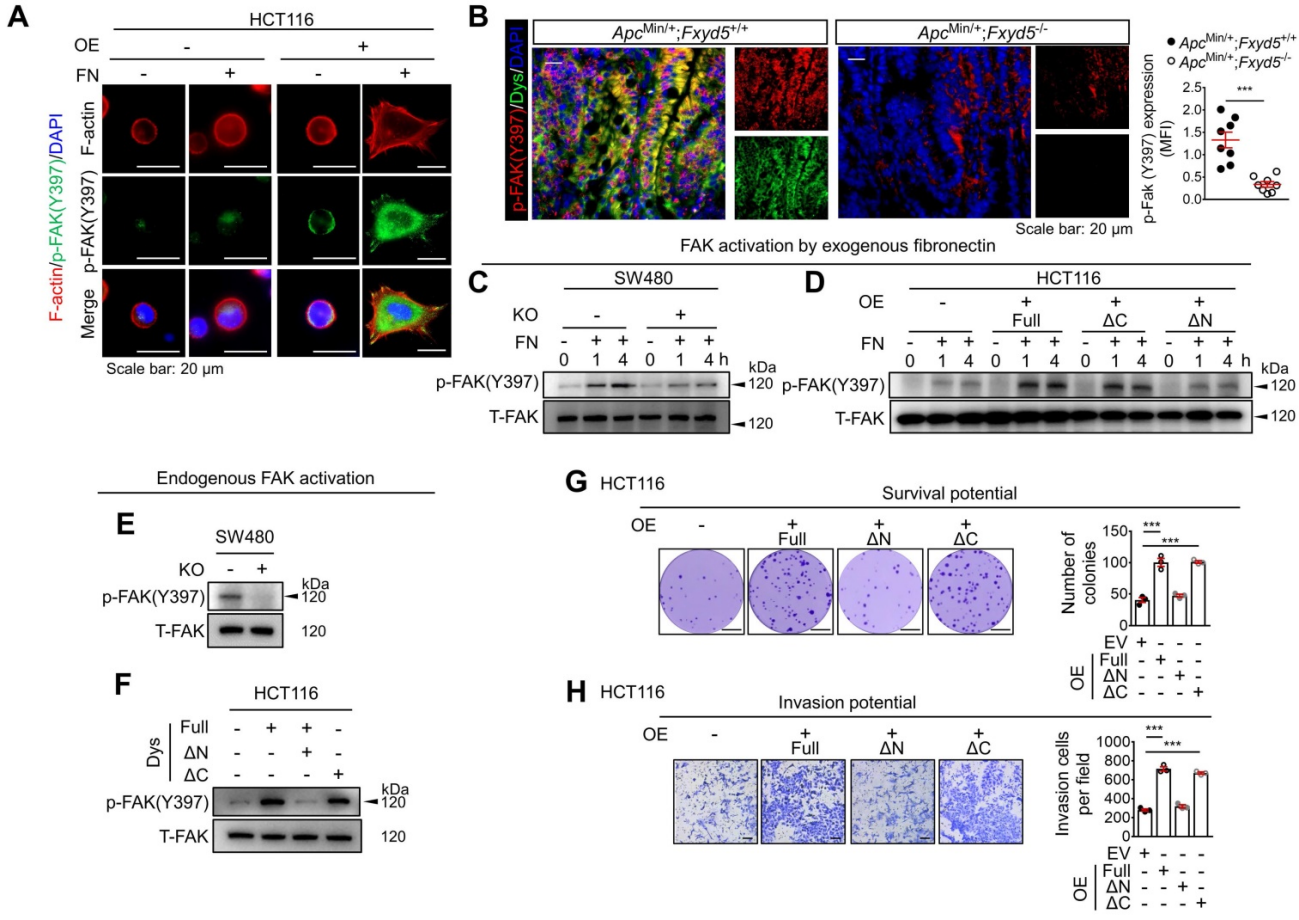
** Dysadherin facilitates CRC adhesion to fibronectin and activates the fibronectin-integrin-FAK axis, leading to pro-tumor activity.** (**A**) Activation of FAK (p-FAK) in HCT116 cells with and without OE of dysadherin 1 h after cell seeding on culture plates with or without fibronectin coating, visualized by IF. (**B**) IF staining for p-FAK and dysadherin in intestinal tumor tissues from *Apc*^Min/+^;*Fxyd5*^+/+^ and *Apc*^Min/+^;*Fxyd5*^-/-^ mice. Graph shows fibronectin protein levels in the indicated groups (n = 8/group). (**C**,**D**) The extent of FAK activation was determined in CRC cells with and without KO (SW480 cells) and OE (HCT116 cells) of dysadherin at the indicated time points after cell seeding on fibronectin-coated culture dishes. (**E**,**F**) FAK activation was measured in 4-day cultures of CRC cells without fibronectin coating. (**G**) The survival potential of HCT116 cells overexpressing wild-type (full-length) or mutant dysadherin compared in clonogenic assays (n = 3/group). (**H**) Comparison of invasion potential of HCT116 cells overexpressing wild-type or mutant dysadherin by Boyden chamber assay. In all panels, data are reported as means ± SEMs; *** indicates *p* < 0.001. Statistical comparisons between 2 groups were performed using Student's t-test. Dys: dysadherin, FN: fibronectin, MFI: mean fluorescence intensity, ΔC: ΔC-mutant, ΔN: ΔN-mutant, T-FAK: [definition], EV: empty vector.

Fibronectin serves as a ligand for numerous integrins that activate the focal adhesion kinase (FAK) signaling pathway through the autophosphorylation of FAK at tyrosine 397 (Y397) 35. Thus, we investigated the potential link between dysadherin and the fibronectin-integrin-FAK pathway upon cell adhesion to exogenous fibronectin coated on culture plates. Consistent with the adhesion assay, dysadherin-OE HCT116 cells showed a greater adhesive phenotype under fibronectin-coated conditions than control cells, with increased cell spreading, polymerized F-actin in their protrusions, more obvious spike-like filopodia and intercellular filaments, and an increase in FAK phosphorylation (p-FAK, **Figure 5A**), while dysadherin-KO SW480 cells showed a lower adhesive phenotype with a decrease in p-FAK (Figure S9C). Consistently, p-FAK was elevated in dysadherin-positive cells within the intestinal tumors of *Apc*^Min/+^/*Fxyd5*^+/+^ mice and this increase in p-FAK was significantly decreased by dysadherin deletion (**Figure 5B**). An immunoblot analysis of SW480 cells showed that p-FAK was induced by cellular adhesion to fibronectin; however, the extent of p-FAK was significantly decreased by dysadherin KO (**Figure 5C**). In HCT116 cells, dysadherin OE enhanced the levels of p-FAK following cell attachment to fibronectin (**Figure 5D**). Of note, in this experiment, we found that deletion of the extracellular domain of dysadherin (ΔN-mutant) eliminated the effect of dysadherin on FAK activation during cell adhesion to fibronectin (**Figure 5D**).

Because we observed fibronectin secretion and adhesion to CRC cell membranes during 4 days of culture *in vitro* (**Figure 4C**), we next examined the potential effect of dysadherin on the status of endogenous FAK activation. Immunoblot analysis of CRC cells cultured for 4 days showed that the endogenous p-FAK level was attenuated or enhanced by dysadherin KO or OE, respectively (**Figure 5E,F**). In this experiment, we repeatedly observed that the OE of the ΔN-mutant dysadherin did not increase FAK activation, indicating the indispensable role of the extracellular domain of dysadherin in FAK activation (**Figure 5F**). Next, to determine whether fibronectin is involved in the dysadherin-mediated increase in FAK activation, we silenced fibronectin expression in both wild-type and dysadherin-OE HCT116 cells (Figure S9D,E). The results showed that fibronectin silencing diminished the dysadherin-induced increase in p-FAK levels (Figure S9F), suggesting that fibronectin mediates dysadherin-induced FAK activation. Collectively, these data provide evidence that the dysadherin-fibronectin interaction through the extracellular domain of dysadherin contributes to sustained activation of the fibronectin-integrin-FAK axis in CRC cells.

### Inhibition of the dysadherin-fibronectin-FAK axis attenuates the protumor activity of dysadherin

To investigate whether dysadherin-fibronectin interaction and subsequent FAK activation are critical for dysadherin-mediated protumor activity, we disrupted the dysadherin-fibronectin-FAK axis in 3 different ways and examined the effects on diverse cellular functions: CRC growth and CRC cell survival, migration, and invasion. First, using dysadherin with the extracellular domain deleted (ΔN-mutant), we examined whether the fibronectin-binding domain is a key regulatory element that mediates the biological function of dysadherin. Second, by silencing fibronectin expression, we determined whether fibronectin binding mediates the critical step in dysadherin function. Finally, by treating cells with a FAK inhibitor, VS-4718 36, we examined whether the biological function of dysadherin is dependent on FAK activation. In the context of CRC growth, deletion of the fibronectin-binding domain (ΔN-mutant) abrogated the dysadherin-induced increase in tumor growth (Figure S10A). Notably, fibronectin knockdown and FAK inhibition diminished dysadherin-induced CRC growth and also attenuated the growth of wild-type HCT116 cells (Figure S10B). This result is consistent with the FAK activation status shown in Figure S9F and S10C, which show that both fibronectin knockdown and treatment with VS-4718 diminished basal p-FAK levels. Similarly, disruption of the dysadherin-fibronectin interaction by deletion of the extracellular domain of dysadherin (ΔN-mutant) abrogated the dysadherin-induced increases in CRC survival, migration, and invasion (**Figure 5G,H** and Figure S10D**)**; this dysadherin-induced aggressive phenotype was attenuated by fibronectin knockdown or FAK inhibition** (**Figure S10D-F). Collectively, these results suggest that the fibronectin-integrin-FAK pathway is a key mechanism of dysadherin-induced intestinal tumorigenesis. Consistently, IF staining of CRC tissues from patients revealed that the extent of fibronectin and p-FAK within the tumor epithelium are significantly increased in dysadherin^high^ tumors compared to dysadherin^low^ tumors (Figure S10G).

### Dysadherin is responsible for the generation of mechanical forces and promotes YAP signal activation

**Fig 6:**
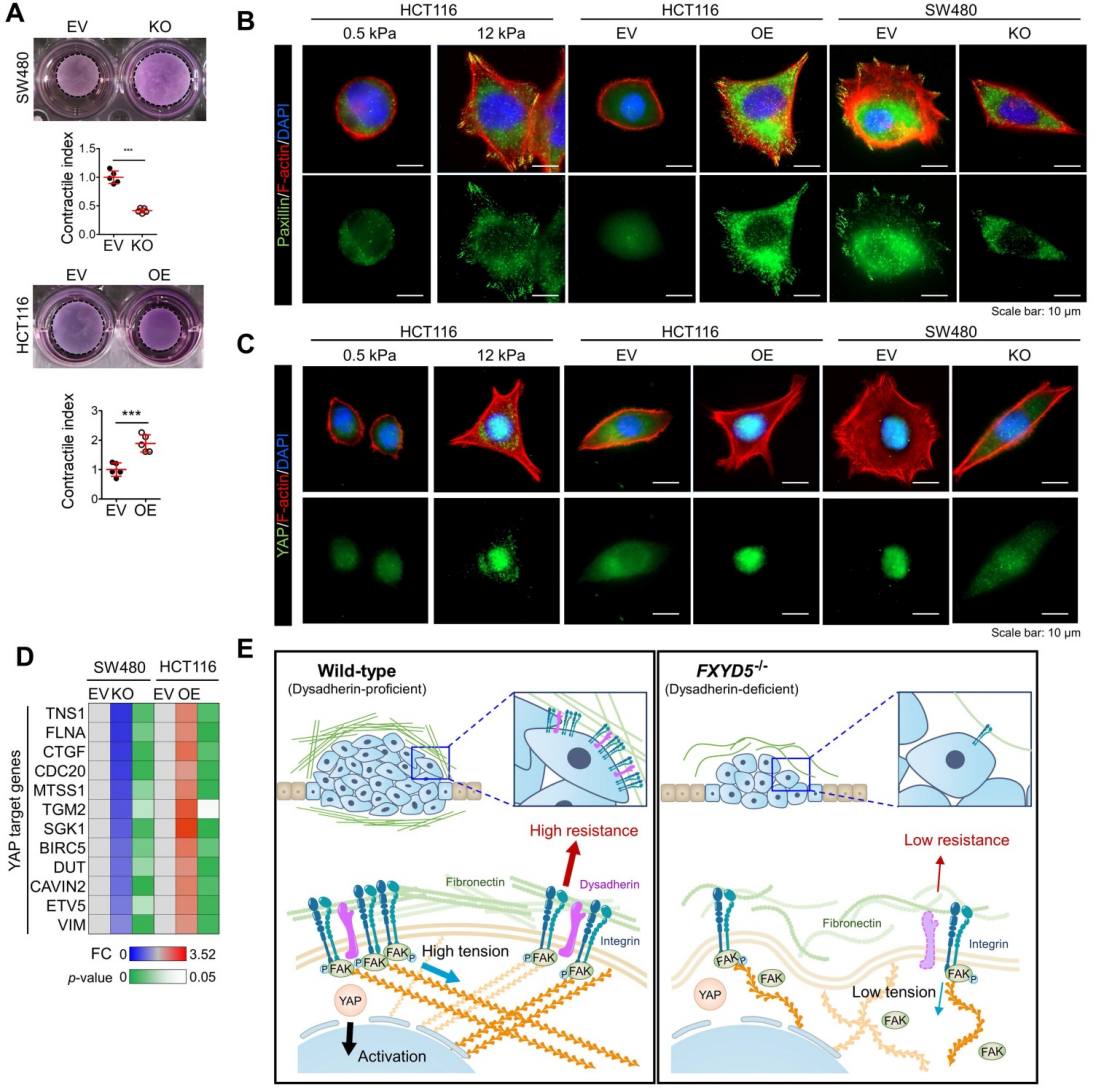
** Dysadherin enhances mechanical force in CRC cells and facilitates YAP mechanotransduction.** (**A**) The extent of mechanical force exerted by CRC cells was measured by collagen gel contraction assays. Contractile index implies the percentage gel contraction^perturbation^/percentage gel contraction^control^. Thus, an increase in the contractile index is an increase in contraction. Images show CRC cell-induced gel contraction after 48 h of cell seeding. Graph shows the extent of dysadherin KO- or OE-induced gel contraction relative to control cells. (**B**,**C**) IF analysis of mechanotransduction by visualizing paxillin-positive focal adhesions (**B**) and YAP (**C**) in CRC cells with dysadherin OE or KO. Hydrogels with a defined elastic modulus (0.5 kPa and 12 kPa) were used as a positive control for mechanical force. (**D**) RT-qPCR validation for YAP target gene expression in CRC cells upon dysadherin OE or KO. (**E**) Schematic summary of the study findings, indicating the potential role of dysadherin-fibronectin interaction in cancer cells to promote mechanical force and CRC tumorigenesis. Statistical comparisons between 2 groups were performed using Student's t-test. EV: empty vector

During cell adhesion, an integrin-FAK axis at focal adhesions (FAs) integrates biomechanical signals by connecting the ECM with the actin cytoskeleton to generate mechanical force in cells; this promotes reciprocal ECM remodeling 16. We therefore wondered whether dysadherin serves as a regulator of mechanical force. The collagen gel contraction assay 37, 38 has served as a classic tool in the field of mechanobiology to study cell-induced contraction of the ECM, which plays an important role in tumor progression and aggression 39. We analyzed the extent of gel contraction upon dysadherin OE and KO using this assay. Dysadherin OE significantly increased the gel contraction compared with the control-transfected cells, while dysadherin KO reduced the extent of gel contraction (**Figure 6A**). In line with these data, we also confirmed that dysadherin expression is a positive regulator of cytoskeletal tension, indicated by an increase or decrease in F-actin staining intensity over the nucleus 40, 41 upon dysadherin OE or KO, respectively (Figure S11A). In parallel, a recent study has demonstrated that cancer cell binding to fibronectin promotes integrin activation, which augments myosin IIa localization at FAs to facilitate cell migration and intracellular force generation 42, 43. Dysadherin augmented cancer cell binding to fibronectin, resulting in increased integrin activation; hence, we sought to determine the role of dysadherin in myosin IIa organization using dysadherin-OE or dysadherin-KO CRC cells plated on fibronectin-coated coverslips. The results of immunolocalization analyses in wild-type HCT116 cells, which expressed a lower level of dysadherin, revealed that myosin IIa was generally localized along the stress fibers (SFs) in the cell center and to actin arches. Intriguingly, dysadherin OE caused an additional accumulation of myosin IIa toward the distal ends of the radial SFs that emanated from FAs (Supplementary Figure S11B, left panel). Consistently, in SW480 cells, which expressed a higher level of dysadherin, myosin IIa was localized at FAs, and this localization was diminished by dysadherin KO (Supplementary Figure S11B, right panel). Additionally, dysadherin OE augmented the myosin IIa phosphorylation at serine 1943 (S1943), indicating an increase in myosin IIa filament dynamics at the leading edge of the cells during cancer cell migration and invasion 44-46, and dysadherin-KO attenuated the myosin IIa phosphorylation (Supplementary Figure S11C). Overall, these data suggested that dysadherin promoted myosin IIa localization at FAs, supporting the notion that dysadherin generates mechanical forces in cells. To confirm whether dysadherin-driven mechanical forces activate downstream biochemical signals, we visualized signal transductions such as FA assembly and yes-associated protein 1 (YAP) activation 47, 48. FA assembly was visualized by staining a FA adapter protein, paxillin 49. In these experiments, we used hydrogels with a defined elastic modulus as a positive control for mechanical force. Consequently, dysadherin-OE cells displayed larger cell spreading areas and greater size and number of FAs, similar to how cells grown on stiff hydrogel (12 kPa) showed greater cell spreading and FA assembly than cells grown on soft hydrogel (0.5 kPa) (**Figure 6B**). Consistently, the nuclear translocation of YAP was up-regulated in cells grown on stiff hydrogel, confirming that mechanical stress enhances YAP signal activation. In line with this result, dysadherin OE significantly increased the nuclear YAP ratio during cell adhesion to fibronectin, while dysadherin KO decreased it (**Figure 6C**). Moreover, validation with CRC cells confirmed that YAP target gene expression tended to increase upon dysadherin OE and decrease upon dysadherin deletion (**Figure 6D**), and immunoblots confirmed that dysadherin OE facilitated activation of YAP by dephosphorylation (Figure S12). In this context, we sought to confirm whether dysadherin expression alters the activation status of mechanotransduction under the same mechanical stress. Hence, we compared the state of mechanotransduction activation in dysadherin-KO or dysadherin-OE CRC cells to that in wild-type CRC cells cultured on the plates coated with a matrix of a certain stiffness (0.5 kPa or 12 kPa). The results indicated that dysadherin-KO SW480 cells were capable of cell spreading, FA assembly and YAP activation on a 12 kPa hydrogel was similar to those detected in wild-type SW480 cells (Figure S13A, left panel). This finding implied that dysadherin KO could not reduce the degree of stiffness-induced mechanotransduction. However, in the case of a 0.5 kPa hydrogel, we detected a significant decrease in cell spreading, FA assembly, and YAP activation upon dysadherin KO (Figure S13A, right panel). Similarly, we were unable to detect the differences in cell spreading, FA assembly, and YAP activation between wild-type and dysadherin-OE HCT116 cells on a 12 kPa matrix (Figure S13B, left panel), implying that dysadherin OE did not alter the degree of stiffness-induced mechanotransduction. However, cell spreading, FA assembly, and YAP activation were increased on a 0.5 kPa hydrogel upon dysadherin OE (Figure S13B, middle panel). Since autonomous secretion of fibronectin from CRC cells induced fibronectin binding to dysadherin-expressing CRC cells to generate phenotypic changes in the cells (Figure 4C and Figure S9 and S10), we decided to further confirm whether phenotypic changes detected on a 0.5 kPa hydrogel are caused by fibronectin binding to dysadherin. Thus, we silenced fibronectin in dysadherin-OE cells and demonstrated that fibronectin depletion did not downregulate mechanical stress-induced cell spreading, FA assembly, or YAP activation in HCT116 cells on a stiff hydrogel (12 kPa, Figure S13B, right panel). However, an increase in cell spreading, FA assembly, and YAP activation by dysadherin OE were significantly diminished by fibronectin depletion in the cells on a soft hydrogel (0.5 kPa, Figure S13B, right panel). Overall, these data suggested that dysadherin promoted the activation of mechanotransduction by facilitating adhesion of the cells to fibronectin, which generated mechanical force in CRC cells (Figure 6E), rather than amplifying mechanical stress-induced intracellular signals at the downstream levels.

## Discussion

Most studies have focused on the importance of dysadherin in cancer invasion and metastasis, and its potential role in tumorigenesis remains elusive. This study confirmed for the first time that dysadherin expression is elevated in CRC patient tumors compared to matched normal tissues. Moreover, we demonstrated that dysadherin is a promising independent prognostic biomarker of CRC that could improve risk stratification of patients, inform clinical management, and avoid unnecessary over-treatment (**Figure 2A-F**). We also confirmed that genetic depletion of dysadherin attenuated intestinal tumorigenesis in both *Apc*^Min/+^ mice and AOM/DSS-treated mice (**Figure 1**). Consistently, in CRC cells, dysadherin expression was found to play a critical role in not only migration and invasion, but also cancer cell growth and survival (Figure S6C,D). Therefore, our findings represent an important step towards unraveling the functional importance of dysadherin in CRC tumorigenesis.

Through gene expression profiling, we found that dysadherin expression was associated with enriched ECM receptor signaling in CRC (Figure S7C and **Figure 3A**). We further identified a possible link between dysadherin and integrin signal transduction; this link may be a critical mechanism of dysadherin-induced intestinal tumorigenesis (**Figure 3C-F**). Thus far, the notion that ECM-mediated cell signaling alterations play a pivotal role in tumor development and progression is widely accepted 15. In particular, integrins are frequently up-regulated in multiple types of solid cancer, including CRC, and exist as 24 combinations of different α- and β-subunits 50. Integrins play a critical role in ECM-cell interactions by acting as a signaling hub that receives signals from outside the cell and activating complex intracellular signal cascades 51. Components of the ECM-integrin axis play multifaceted roles as signaling molecules, mechanotransducers, and key components of the cellular machinery in nearly every step of tumorigenesis, from primary tumor development to metastasis 51. In this study, comprehensive analyses confirmed that dysadherin contributes to sustained activation of the ECM-integrin axis through its extracellular domain, providing an explanation for how dysadherin enforces aberrant signaling to fuel intestinal tumorigenesis. Also, we also identified fibronectin as a binding partner for dysadherin. Fibronectin, a major core component of the tumor microenvironment, can be produced by multiple cellular compartments in the stroma and tumor cells. Fibronectin binds the extracellular domain of dysadherin; thus, dysadherin facilitates tumor cell adhesion to fibronectin, which enhances sustained activation of the fibronectin-integrin axis. Dysadherin contributes to sustained integrin-FAK activation by interacting with fibronectin through its extracellular domain, which mediates protumor activities of dysadherin (**Figure 5**, Figure S10). Further, this study revealed a unique sequence within the extracellular domain of dysadherin, amino acids 64-115, which displayed the fibronectin-binding ability (**Figure 4G**), providing a basic understanding of the binding mechanism between dysadherin and fibronectin. This novel finding will be valuable for future development of therapeutic strategies to target dysadherin-driven tumor progression and aggression. However, further peptidomimetic studies will be required to develop this amino acid sequence to function* in vitro* and *in vivo* for disruption of dysadherin-fibronectin binding.

Cells are known to detect and react to the biophysical properties of the extracellular environment through integrin-based adhesion sites, the so-called FAs 16, 52. At these adhesion sites, integrins connect the ECM with the F-actin cytoskeleton to generate mechanical force in cells, and this promotes reciprocal ECM remodeling. High mechanical stress in solid tumors can drive tumor progression and promote malignant cell behavior through FA assembly and downstream mechanotransduction 39. One prominent mechanism by which mechanical forces regulate cell behavior is the regulation of YAP activation 53. YAP is a transcriptional coactivator that shuttles between the cytoplasm and the nucleus and plays a prominent role as an oncogenic factor in multiple tissues 48. Moreover, fibronectin-integrin-FAK axis is a key mediator of mechanotransduction of signals responsible for aberrant YAP activation 54, 55. In this context, we confirmed that dysadherin is a positive regulator of mechanical forces and downstream mechanotransduction during cell adhesion to fibronectin (**Figure 6**). Additionally, by deleting the extracellular region of dysadherin which is responsible for dysadherin-fibronectin binding, we found that the interaction between dysadherin and fibronectin is critical for dysadherin-mediated YAP activation during cell adhesion to fibronectin (Figure S12). Therefore, our results propose that dysadherin may serve as a promising target to potentially perturb the mechanical stress implicated in cancer progression and aggression.

During metastasis, cell adhesion to the ECM proteins has dual functions of either inhibiting or promoting metastasis 9. For instance, several genetic alterations, which inhibit cell adhesion to the ECM proteins, upregulate the migratory and invasive potentials of cancer cells 56-59. In contrast to these reports, a great number of studies have shown that cancer cell adhesion to the ECM proteins enhances the malignant behaviors of cancer cells by activating multiple signaling cascades associated with cell migration, such as the FA pathway 60-63. In this context, the present study sheds light on a novel function of dysadherin in CRC tumorigenesis and metastasis by providing fundamental evidence that dysadherin facilitated CRC cell adhesion to fibronectin and thereby activated the integrin/FAK signaling axis, which collectively contributed to the multifaceted role of dysadherin in CRC cells. Intriguingly, we presented additional evidence that dysadherin was involved in ECM remodeling, as indicated by the data of GSEA (Figure S14), suggesting potential involvement of dysadherin in the dynamics of ECM remodeling, which occurs during cancer progression 15, 64. Thus, verification of a putative link between dysadherin and ECM remodeling and relevance of this link to CRC progression will be an important subject of future research.

Collectively, our results reveal that dysadherin and fibronectin interact and subsequently distort integrin-mediated mechanotransduction, establishing dysadherin as a new driver of mechanotransduction that drives intestinal tumorigenesis. Our findings represent a key step in understanding the complexity of mechanobiology and may provide a basis for further development of new therapeutics to overcome the mechanics of cancer progression and aggression.

## Conclusions

This study demonstrated that dysadherin is a key driver of mechanotransduction that contributes to CRC development and accelerates progression to the malignant phenotype, paving the way for new molecular and biological insight as well as therapeutic implications.

## Supplementary Material

Supplementary materials and methods, figures, and tables.

## Abbreviations

OE: Overexpression
ECM: Extracellular matrix
KO: Knockout
APC: Adenomatous polyposis coli
CRC: Colorectal cancer
RFS: Recurrence-free survival
OS: Overall survival
GSEA: Gene set enrichment analysis
IP: Immunoprecipitation
siRNA: small interfering ribonucleic acid
CRISPR: Clustered regularly interspaced short palindromic repeats
LC-MS: Liquid chromatography - Mass spectrometry
DAVID: Database for annotation, visualization and integrated discovery
EpCAM: Epithelial cell adhesion molecule
AOM: Azoxymethane
DSS: Dextran sodium sulfate
FAK: Focal adhesion kinase
FA: Focal adhesion
SF: Stress fiber
YAP: Yes-associated protein 1
