# Supplementary material for: Dysadherin awakens mechanical forces and promotes colorectal cancer progression
Source: Theranostics. 2022 May 21;12(9):4399–414. doi: 10.7150/thno.72354 (PMC9169357; doi:10.7150/thno.72354)
Supplement: Supplementary file 1 — Supplementary materials and methods, figures, and tables. [file thnov12p4399s1.pdf]

SUPPLEMENTARY MATERIALS for

Dysadherin awakens mechanical forces and promotes colorectal cancer progression

So-Yeon Park[1,2,#]†, Choong-Jae Lee[1,]†, Jang-Hyun Choi[1], Jee-Heun Kim[1], Won-Jae Lee[1], Tae-Young Jang[1], So-El Jeon[1], Jae-Hyun Kim[1], Sang-Hee Cho[3], Ji-Shin Lee[4], Jeong-Seok Nam[1,2,]*

[1]School of Life Sciences, Gwangju Institute of Science and Technology, Gwangju, 61005, Republic of Korea

[2]Cell Logistics Research Center, Gwangju Institute of Science and Technology, Gwangju, 61005, Republic of Korea

[3]Department of Hemato-oncology, Chonnam National University Medical School, Gwangju, 61469, Republic of Korea

[4]Department of Pathology, Chonnam National University Medical School, Gwangju, 61469, Republic of Korea

***Corresponding author**: Jeong-Seok Nam (phone: +82-62-715-2893; fax: +82-62-715-2484, address: School of Life Sciences, Gwangju Institute of Science and Technology, Gwangju, 61005, Republic of Korea; e-mail: namje@gist.ac.kr)

†These authors contributed equally to this work.

# Current address: Institute for Basic Science, Center for Genome Engineering, 55, Expo-ro, Yuseong-gu, Daejeon, Korea, 34126

SUPPLEMENTARY MATERIALS AND METHODS

Study design

The object of our study was to elucidate the biological role and mechanistic basis of dysadherin in intestinal tumorigenesis. Prior approval for animal studies was obtained from the Institutional Animal Care and Use Committee (IACUC) of the Gwangju Institute of Science and Technology (GIST, No. GIST2018-049). For all analyses, sample sizes were determined in accordance with the 3 Rs (replacement, reduction, and refinement). Humane end points were predefined as impaired breathing and/or hunching with 10% or more weight loss. Outcome assessment was performed blindly without reference to genotype. Reporting is consistent with the ARRIVE guidelines [1]. *In vitro* experiments were all performed on three separate occasions. *In vitro* analyses were not blinded. Outliers were included in all experiments. Analysis of dysadherin expression in patients with CRC was preapproved by the Institutional Review Board at GIST (No. 20200108-BR-50-07-02). All work related to human tissues was conducted in accordance with the Helsinki Declaration. Written informed consent was obtained from all participants prior to the study. This was a retrospective study using tissue microarray (TMA) slides, and therefore, study size calculation was not performed. Histological assessment was performed blindly and scored automatically using Image-Pro Premier 9.2 software (Media Cybernetics Inc., Rockville, MD, USA) in a blinded manner without any clinical information. The data of clinical relevance are reported consistently with the REMARK guidelines [2].

Chemicals and reagents

10X cell lysis buffer (#9803), 3X sodium dodecyl sulfate (SDS) sample buffer (#7722), and Protein A agarose beads (#9863) were purchased from Cell Signaling Technology (Beverly, MA, USA). Fluorescence-activated cell sorting (FACS) Lysing^{TM} Solution was purchased from BD Biosciences (San Diego, CA, USA). TRIzol reagent was purchased from Ambion (Austin, TX, USA). VS-4718 was purchased from Tocris Bioscience (Ellisville, MO, USA). Anti-dysadherin monoclonal antibody (M53) [3] was kindly gifted by Dr. Yoshinori Ino (National Cancer Center Research Institute, Tokyo, Japan).

Generation of *Fxyd5* knockout (KO) mice

The *Fxyd5*$^{-/-}$ mice were purchased from Vitalstar (Beijing, China). Ear tagging was used for identification of mice and recordkeeping throughout the research project. Briefly, *Fxyd5*$^{-/-}$ mice were generated using CRISPR/Cas9 technology. Three guide RNAs (gRNA sequences: AGGCTGCTAGGCATCTCGGGGGG, TCTTCCTGGGCTCGGTCACGTGG, and CCCCGATGAGCGATACAGAGACA) were used to cut the genomic DNA at *Fxyd5* introns 1 and 7, which resulted in the deletion of exons 2-7, which contain the ATG start codon and most of the coding sequences. Four strains harboring the *Fxyd5* mutation (gene targeting efficiency: 4/6 = 66.66%) were identified. Strain 661, a heterozygote strain carrying 5740-bp deletions of *Fxyd5* (Fxyd5 null), was selected as a founder. Using strain 661, at least 5 backcrosses with C57BL/6J were conducted to minimize off-target effects. No sex-specific differences were observed. The list of primers used for genotyping is provided in Table S1.

Animal models

All mice used in this study were housed under specific pathogen-free conditions and cared for in accordance with international guidelines preapproved by the IACUC at GIST. The exact number of mice for each experiment is noted in the figure legends. To examine the impact of dysadherin deficiency on intestinal tumor development, we developed a new murine model by crossing female dysadherin-KO (*Fxyd5*$^{-/-}$) mice with male *Apc*$^{Min/+}$ mice on a C57BL/6J background. *Apc*$^{Min/+}$ mice were purchased from Jackson Laboratory (Bar Harbor, ME, USA) and the *Fxyd5*$^{-/-}$ mice were purchased from Vitalstar (Beijing, China). Ear tagging was used for identification of mice and record keeping throughout the research project.

To compare the status of tumor development between *Apc*$^{Min/+}$;*Fxyd5*$^{-/-}$ and *Apc*$^{Min/+}$;*Fxyd5*$^{+/+}$ mice, male mice were sacrificed at the ages of 4, 6, 8, and 20 weeks, and the intestinal tracts were opened longitudinally and carefully examined for tumors in a blinded manner without any genotype information.

To prepare the chemically induced intestinal tumor mouse model, 8-week-old male wild-type (*Fxyd5*$^{+/+}$) and *Fxyd5*$^{-/-}$ mice were treated with a single intraperitoneal injection of 10 mg/kg AOM (day 0). One week later, the mice were treated with 2.0% DSS in drinking water for 1 week, and then the DSS-containing drinking water was

exchanged for plain water for 2 weeks. This 3-week DSS treatment course was performed a total of 4 times for 12 weeks. After 1 additional week, the mice were sacrificed, and the status of CRC development was evaluated as described above.

Apc$^{Min/+}$ mouse polyp-derived tumoroid culture

Single cells were isolated from the intestinal polyps of 20-week-old *Apc*$^{Min/+}$ mice and cultured as described in a previous report with slight modifications [4]. Briefly, mouse intestines containing polyps were incubated with ethylenediamine tetraacetic acid (EDTA) chelation buffer for 60 min on ice. After chelation, the detached normal intestinal epithelial cells were removed by centrifugation, while tumor cells remained attached to the mesenchyme. Intestinal fragments with tumor cells were then dissociated with collagenase as described in a previous report [4]. The isolated tumor cells were counted and pelleted and a total of 20,000 cells or 100 cells were then mixed with 50 µL or 10 µL of Matrigel (Corning Matrigel® Growth Factor Reduced Basement Membrane Matrix, #356231, Corning, NY, USA) and plated in 24-well plates or 96-well plates, respectively. After the polymerization of Matrigel, 500 mL of IntestiCult™ Organoid Growth Medium (Mouse, #06005, STEMCELL Technology) was added. Beginning on the day of seeding, growth and morphology of organoids were observed daily, and the viability of organoids was evaluated by performing a resazurin-based Cell Titer Blue assay (Promega, Leiden, The Netherlands) on the seventh day of organoid culture. The mean diameter of tumoroids from each well was measured using Image-Pro Premier 9.2 software (Media Cybernetics Inc., Rockville, MD, USA). For generation of the *Fxyd5* knockdown tumoroids, isolated tumor cells were transfected with siRNA against the *Fxyd5* gene using a NEPA21 superelectroporator (NEPAGENE, Chiba, Japan). The transfected cells were then cultured and monitored as described above. The list of siRNA sequences used for *Fxyd5* knockdown is provided in Table S2.

Histological assessment

All tissue samples were formalin-fixed and paraffin-embedded or frozen in optimal cutting temperature compound (Leica Microsystems, Buffalo Grove, IL, USA) within 30 min after removal from mice. Paraffin-embedded or compound-embedded tissue blocks were manually sectioned with a microtome to obtain 4-5 µm

thick sections. Paraffin sections were dewaxed and stained with hematoxylin (Dako, Carpinteria, CA, USA) and eosin (Millipore, Billerica, MD, USA) according to the supplier's instructions. Target proteins were visualized by immunohistochemistry (IHC) and immunofluorescence (IF). Proteins were visualized using the specific antibodies described in Table S3. Nuclei were counterstained with hematoxylin (Dako, Carpinteria, CA, USA) for IHC or 4′,6-diamidino-2-phenylindole (DAPI, Sigma-Aldrich) for IF. Secondary antibodies conjugated with horseradish peroxidase (HRP, Dako) or with fluorescent dyes (Life Technologies, Carlsbad, CA, USA) were used to visualize target proteins. For IHC, target proteins were visualized by the DAB reaction [5] and observed with light microscopy (Leica Microsystems) at 400x magnification. DAB intensity was automatically quantified with Image-Pro Premier 9.2 software (Media Cybernetics Inc., Rockville, MD, USA) in 3 random spots per every tissue sample in a blinded manner without any genotype information. The integrated optical density (IOD) of the target protein was calculated by multiplying the area and average density. Fluorescence signals were visualized using an Axio Imager 2 (Carl Zeiss, Oberkochen, Germany) or confocal LSM880 microscope (Carl Zeiss) at a total magnification of 400x or 1000x. Relative expression levels of the target protein were measured based on fluorescence intensity as described above and normalized to DAPI intensity.

Real-time reverse transcriptase quantitative polymerase chain reaction (RT-qPCR)

Total RNA was isolated using TRIzol reagent (Invitrogen). The purity of RNA was verified by measuring 260/280 and 260/230 absorbance ratios. cDNA templates were synthesized from 0.5 μg of total RNA using the PrimeScript^{TM} 1st strand cDNA Synthesis Kit (Takara Biomedicals, Kusatsu, Japan) with random primers. Power SYBR Green PCR Master Mix and Step-One Real-time PCR systems (Applied Biosystems, Foster City, CA, USA) were used for the PCR amplification of cDNAs. The list of primers used for RT-qPCR is provided in Table S1.

Clinical analysis and statistics

TMA slides from 123 patients with CRC were immunostained to detect dysadherin using a specific monoclonal antibody (M53) as previously reported [3]. The TMA slides contained 3 tumor tissue cores and 2 matched normal tissue cores from each patient. After heat-induced epitope retrieval, the slides were permeabilized

and incubated with primary antibodies (1:500) at 4 °C overnight. Following repeated washing steps, the slides were incubated with anti-mouse biotinylated antibody (Vector ABC Kit, Vector Laboratories, Burlingame, CA, USA) for 30 min at room temperature, and dysadherin expression was visualized by the DAB reaction and observed under light microscopy (Leica Microsystems, Buffalo Grove, IL, USA). The expression of dysadherin in the CRC epithelium was automatically quantified with Image-Pro Premier 9.2 software (Media Cybernetics Inc., Rockville, MD, USA) in a blinded manner without any clinical information. Three spots per core were randomly selected, and the IOD of dysadherin was calculated by multiplying the area and average density. The ratio of dysadherin expression in tumor tissues versus normal tissues (average IOD of 3 tumor tissue cores/average IOD of 2 normal tissue cores) was used to determine the association of dysadherin expression with clinicopathological variables. Patients were divided into two group according to the IOD values; dysadherin-high (≥ 75%, n = 27) and dysadherin-low (< 75%, n = 96). The statistical significance of differences was assessed using the chi-square test or Fisher's exact test for categorical data, and continuous variables were compared using the independent samples t-test. Recurrence-free survival (RFS) was defined as the time from the date of surgery to the date of recurrence or death, whichever occurred first, and patients who were alive at the last follow-up were recorded at that time If neither event had occurred at the time of analysis, the patient was censored. Overall survival (OS) was calculated from the diagnosis of disease to death from any cause, and patients who were alive at last follow-up were recorded at that time. Survival was calculated using the Kaplan-Meier method, and comparisons were made using log-rank tests. Factors associated with RFS and OS were identified by univariate and multivariate Cox proportional hazards regression models with hazard ratios and 95% confidence intervals. Statistical analyses were performed using SPSS version 21.0 (IBM Corporation, Armonk, NY, USA); all p-values were two-sided, and $p < 0.05$ was used as an indicator of statistical significance.

Cell lines

HCT116 (KCLB Cat# 10247, RRID:CVCL_0291), SW480 (KCLB Cat# 10228, RRID:CVCL_0546), LoVo (KCLB Cat# 10229, RRID:CVCL_0399), HCT15 (KCLB Cat# 10225, RRID:CVCL_0292), LS174T (KCLB Cat# 10188, RRID:CVCL_1384), and HT29 (KCLB Cat# 30038, RRID:CVCL_0320) cell lines were purchased from the Korean Cell Line Bank (Seoul, Republic of Korea). The SW48 cell line (ATCC Cat# CCL-231,

RRID:CVCL_1724) was purchased from American Type Culture Collection (Rockville, MD, USA). The NCM460D cell (INCELL cat# NCM460D, RRID:CVCL_IS47) line was purchased from INCELL (San Antonio, TX, USA). The MC38 C57BL6 murine intestinal tumor cell line (RRID:CVCL_B288) was purchased from Kerafast (Boston, MA, USA). All cells were cultured according to the supplier's instructions. The cells were routinely tested for mycoplasma contamination every 6 months using the e-Myco^{TM} Mycoplasma detection kit (iNtron Biotechnology, Seongnam, Republic of Korea), and all experiments were performed within 20 passages from the first thaw.

Syngeneic mouse model using murine colon carcinoma cell line

Luciferase-labeled MC38 cells (C57BL/6J mice-derived colon carcinoma cell line) were inoculated subcutaneously (s.c.) into 8-week-old male *Fxyd5*$^{+/+}$ or *Fxyd5*$^{-/-}$ C57BL/6J mice (5 x 10^4/mouse). Tumor growth was monitored by measuring luciferase activity for 18 days from cell inoculation until necropsy (*Fxyd5*$^{+/+}$, n = 6; *Fxyd5*$^{-/-}$, n = 6). After necropsy, tumor volumes were calculated according to the following formula: tumor volume = length x width^2/2. Blood was collected from the abdominal vena cava, stored in EDTA blood collection tubes, and subjected to hematological test using an Exigo Veterinary Hematology Analyzer (Boule Medical, Stockholm, Sweden). Age-matched naïve C57BL/6J mice were used as the control group (*Fxyd5*$^{+/+}$, n = 3; *Fxyd5*$^{-/-}$, n = 3).

Protein isolation and immunoblot analysis

Tissues or cells were homogenized in RIPA buffer for 20 min on ice. Protein concentrations were determined based on bicinchoninic acid (BCA) assay using the BCA Protein Assay kit (Thermo Fisher Scientific, Waltham, MA, USA). Proteins were denatured with SDS (Sigma-Aldrich) by boiling at 95 °C for 5 min. Equal amounts of total protein (4-15 μg) were separated by 8% or 10% polyacrylamide gel electrophoresis (PAGE), and separated proteins were transferred to a polyvinylidene difluoride membrane (Millipore, Billerica, MA, USA). Membranes were blocked with 5% bovine serum albumin (Sigma-Aldrich) and incubated overnight at 4 °C with the indicated primary antibodies. Membranes were then incubated with HRP-conjugated secondary antibodies. Chemiluminescence of HRP was developed with ECL reagent (Atto, Tokyo, Japan) and detected with a digital

imaging system (ProteinSimple, San Jose, CA). Antibodies used for immunoblot analyses are listed in Table S3.

Establishment of dysadherin-knockout (KO) or -overexpressing (OE) cell lines

A dysadherin-KO (*FXYD5*$^{-/-}$) SW480 cell line was generated by Transomic Technologies (Huntsville, AL, USA). Specific gRNA targeting different regions of the human *FXYD5* gene was designed and cloned into the pCLIP-All (EFS-Puro) expression vector. SW480 cells were infected with lentiviral particles and selected with puromycin for 1 week. Dilution cloning was performed to obtain different monoclonal cell populations. The KO efficiencies of multiple clones were estimated by real-time RT-qPCR and immunoblotting, and a single clone showing the most potent KO efficiency was selected (gRNA sequence: GAGATGGGTCTTACCTC TGG) and used for further experiments. For establishment of the dysadherin-OE cell line, HCT116 cells were transfected with dysadherin expression vector (pcDNA-L3HSV) [6] and selected with G418 for 1 week. Dilution cloning was performed and OE of the dysadherin gene was confirmed by RT-qPCR and immunoblot analysis. For generation of HCT116 cell lines overexpressing mutant forms of dysadherin, coding sequences were synthesized and cloned into pcDNA™4/HisMax vector by Thermo Fisher Scientific. Vector transfection, stable cell line generation, and validation were performed as described above.

Clonogenic assay

A clonogenic assay was performed as described in our previous report [5]. Briefly, cells were seeded in 12-well plates (200 cells/well) and cultured for 14 days. The numbers of colonies greater than 50 μm in size were counted after staining with crystal violet (n = 3/group).

Cell growth assay

Cells were seeded in 6-well or 96-well plates and incubated for various time points. The numbers of viable cells were measured with an automated cell counter (Countess II, ThermoFisher Scientific, Waltham, MA, USA) or

measured by staining with thiazolyl blue tetrazolium bromide (MTT, Sigma-Aldrich, St. Louis, MO, USA), and the absorbance was measured using a microplate spectrophotometer (Bio-Tek Instruments Inc., Winooski, VT, USA).

Wound healing assay

Cells were seeded in culture inserts (Ibidi, GmbH, Martinsried, Germany). Cells were incubated for 48 h (endpoint) after wound scratch. Phase-contrast images of cells were captured using a camera attached to a microscope (Carl Zeiss) at a total magnification of 100x. The wound area at time zero or the endpoint was measured using Image-Pro Premier 9.2. The area of wound closure was calculated as a percentage of the initial wound area. To minimize the effect of differences in cell growth rate, cycloheximide, which inhibits protein synthesis and blocks mitotic entry [7], was added to the culture media during the period of wound closure as described in a previous report [8].

Apoptosis assay (Annexin V+)

The rate of cell apoptosis was quantitatively analyzed by performing apoptosis assays using an Annexin V-Fluorescein Isothiocyanate (FITC) Apoptosis Detection Kit I (BD Biosciences). Cell suspensions (1 x 10^6/mL) were prepared by washing cells twice with cold PBS. Then, 100 μL of the suspension was transferred to a tube to which 5 μL of FITC, annexin V, and propidium iodide were added. The mixture was incubated at room temperature for 15 min in the dark after gentle vortexing. After incubation, 400 μL of 1X binding buffer was added before analysis using flow cytometry. FACS analysis was performed using a BD Accuri™ flow cytometer (BD Biosciences). FACS data were analyzed using FlowJo software (TreeStar, San Carlos, CA, USA) as described in our previous report [5].

Boyden chamber assay

The Transwell system (8 μm pore size, Corning) was employed for migration and invasion assays. For the migration assay, 3 x 10^5 cells were seeded on the upper chambers in serum-free medium. For the invasion assay, 3 x 10^5 cells were seeded on the upper chamber of a Matrigel-coated Transwell system (8 μm pore size, Corning) in serum-free medium. The bottom chamber was filled with medium supplemented with 20% fetal bovine serum. After incubation for 24 h at 37 °C, the cells that migrated or invaded the Matrigel through to the bottom of the insert membrane were fixed, stained with crystal violet, and counted under a phase-contrast microscope (Carl Zeiss, biological triplicates).

CRC xenograft mouse model

To compare the tumor-forming potential of empty vector (EV)-transfected control cells and dysadherin-KO cells, an *in vivo* limiting dilution assay (LDA) was performed. Cells were inoculated s.c. into male NSG mice (NOD.Cg-Prkdc^{scid} Il2rg^{tm1Wjl}/SzJ, #005557, Jackson Laboratory, Bar Harbor, ME, USA) at various cell dilutions (50000, 10000, 5000, 1000 cells/mouse, n = 6/group). After 56 days, the incidence of tumors in mice was determined by definitive necropsy. LDA graphs were generated, and statistical values were calculated using online software provided by Walter+Eliza Hall Bioinformatics (http://bioinf.wehi.edu.au/softwar e/elda/) as described in a previous report [9].

A splenic injection experiment was performed to estimate metastasis and distant organ colonization [10]. In this model, EV-transfected control cells or dysadherin-KO cells were tagged with luciferase and inoculated into the spleens of NSG mice followed by splenectomy (1 x 10^6 cells/mouse); surviving cells that had migrated out of the spleen and grew in distant organs would then contribute to the formation of liver metastases. We routinely monitored liver metastasis weekly by visualizing luciferase activity for 33 days. After sacrifice, the livers were removed to verify and quantify liver metastasis.

Bioinformatics analyses using an open-source database

To compare the *FXYD5* mRNA expression levels between normal and tumor tissues, gene expression data were

obtained from an open-source database (R2: Genomic analysis and visualization platform, https://hgserver1.amc.nl/cgi-bin/r2/main.cgi). Gene set enrichment analysis (GSEA) was conducted as described in our previous study [11]. Briefly, the differentially expressed gene (DEG) list was obtained using the R2 platform (GSE21510 dataset) by comparing two groups of patients with CRC (divided according to median dysadherin level; n = 52/group). Total DEG lists ($p < 0.001$) were applied as a ranked gene list. The GSEA of the ranked gene list was conducted using the Java implementation of GSEA obtained from http://www.broadinstitue.org/gsea/ (1,000 permutations; minimum term size: 15; maximum term size: 500, C2: all curated genes). The normalized enrichment score accounts for the differences in gene set size. The false discovery rate q-value was used to set the significance threshold.

RNA-sequencing and Ingenuity Pathway Analysis (IPA)

RNA-sequencing was performed to find the altered gene expression signature between dysadherin-KO and control (EV-transfected) SW480 cells. Samples were prepared in biological triplicates, and an RNA-sequencing analysis was performed by LAS science (Seoul, Republic of Korea). Briefly, the mRNA sequencing library was prepared using the TruSeq Stranded mRNA Sample Preparation Kit and the Illumina NextSeq platform. The list of DEGs (n = 4,437, $p < 0.05$) altered in dysadherin-KO SW480 cells was subjected to IPA (Qiagen, Redwood City, CA, USA) to identify the potential diseases, functions, and upstream regulators that are significantly associated with dysadherin KO.

Identification of dysadherin-interacting proteins

Potential dysadherin-interacting proteins were identified in whole-protein lysates of SW480 cells (KCB Cat# KCB 200848YJ) using M53 monoclonal antibody-based co-immunoprecipitation (co-IP). Incubation with isotype IgG control antibody was used as a negative control. Co-IP proteins were separated by sodium dodecyl sulfate-polyacrylamide gel electrophoresis (SDS-PAGE) and digested in-gel with trypsin. The peptide samples were purified and concentrated by using columns containing C18 reverse-phase resin. The peptides were identified by LC-MS. Raw mass spectrometric data were processed with Sorcerer 2-SEQUEST (Sage-N Research, Milpitas,

CA, USA). A list of proteins enriched in the anti-dysadherin co-IP samples versus the IgG control IP samples was obtained by estimating the protein abundance through Top3 TIC method used in Scaffold 4 Q + S program (version 4.6.1, Proteome Software Inc., Portland, OR, USA).

Pull-down assay for determination of direct protein-protein interaction

The coding sequences of wild-type or mutant forms of dysadherin were synthesized and cloned into pET151/D-TOPO vector by Thermo Fisher Scientific. OE of His-tagged dysadherin was achieved using *E. coli* grown in Luria-Bertani (LB) broth medium, supplemented with ampicillin. Isopropyl β-D-1-thiogalactopyranoside (IPTG, 0.5 mM) was added into the LB broth medium to induce the expression of the protein of interest, and the mixture was incubated for 5 h at 37 °C. The cells were then lysed using RIPA buffer and purified using a Ni-NTA column (R90101, Thermo Fisher Scientific). The concentrated purified protein was measured using the BCA Protein Assay kit (Thermo Fisher Scientific). Purified proteins were visualized by separating in 10% SDS-polyacrylamide gels and staining with Coomasie Brilliant Blue. Purified fibronectin protein was purchased from Sigma Aldrich. The mixture of purified His-tagged dysadherin proteins (1 μg) and purified fibronectin protein (0.5 μg) was incubated with anti-His mouse monoclonal antibody (MA1-21315, Thermo Fisher Scientific) at 4 °C overnight. Protein G agarose beads (#37478, Cell Signaling Technology, Beverly, MA, USA) were added and samples were incubated for 6 h at 4 °C. After the bead binding step, samples were centrifuged and the pellets were boiled in 3X SDS-PAGE loading buffer for immunoblotting. Presence of His-tagged dysadherin and fibronectin proteins were determined by immunoblot analysis using anti-His rabbit monoclonal antibody (#12698, Cell Signaling Technology) and anti-fibronectin rabbit polyclonal antibody (ab2413, Abcam, Cambridge, MA, USA).

Pull-down assays of peptides were performed using Ni-NTA agarose beads (R90101, Thermo Fisher Scientific). His-tagged dysadherin protein was purchased from Abcam (ab140573) and peptides were synthesized by ANYGEN (Cheongju, Republic of Korea). The purity of each peptide was determined to be over 95% by LC-MS. The mixtures of purified fibronectin protein (0.5 μg, Abcam, F2006) and His-tagged dysadherin proteins (2 μg) or peptides (20 μg) were incubated in 500 μL of buffer containing 50 mM NaH_2PO_4 (pH 7.4), 250 mM NaCl, and 10 mM imidazole for 1 h on ice, followed by the addition of 15 μL of Ni-NTA agarose beads (R90101, Thermo

Fisher Scientific) with end-to-end rotation for 1 h at 4 °C. The beads were precipitated by centrifugation at 2,000 × g for 3 min and then washed five times with 500 μL of binding buffer containing 30 mM imidazole. The beads were boiled in 3X SDS-PAGE loading buffer, and the proteins were separated by 15% SDS-PAGE. The presence of His-tagged dysadherin, dysadherin peptides, and fibronectin was determined by staining with Coomassie Brilliant Blue.

Immunofluorescence staining and quantification

Cells were cultured on fibronectin-coated coverslip or on the plates coated with a matrix with a certain stiffness (0.5 kPa or 12 kPa) and fixed with 4% paraformaldehyde for fluorescent staining. Cells were permeabilized with 0.3% Triton X-100, and nonspecific binding was blocked with 3% bovine serum albumin. Proteins were visualized using the specific antibodies described in Table S3. Samples were examined by fluorescence microscopy. Quantitative analysis of F-actin distribution was performed using ImageJ

Cell adhesion assay

To examine the cell adhesion capacity to diverse ECM molecules, 6-well and 96-well plates were coated with ECM molecules (Fibronectin, Laminin, Collagen type I and Collagen type IV). Cells were seeded in each ECM molecule-coated 6-well plates. After incubation for 1 h, cells were washed with PBS and stained with crystal violet. Stained cells observed with light microscopy (Leica Microsystems). Additionally, when seeded in 96-well plates. After incubation for 1 h, the cells were then quantified by absorbance readings (570 nm), after MTT staining.

Collagen gel contraction assay

Force-mediated gel contraction was performed as previously described with slight modification [12]. Briefly, CRC cells (1 x 10^6 cells) were embedded in 1 mL collagen-I, yielding a final collagen I concentration of 1.5 mg/mL, and seeded in 12-well plates. When performing the gel contraction assay, the wells were pre-coated with

1% bovine serum albumin. We used high-concentration Corning collagen-I purified from rat tail (#354249). After 1 h of gelation at 37 °C, the cells were washed once in normal medium for 1 h and reimmersed in fresh medium. The gel contraction was monitored after 48 h by taking photographs of the gels. The percentage of gel contraction was quantified using the formula: percentage gel contraction = 100 x ((well areal-gel area)/well area)). To normalize the data points, we generated a contractile index using the formula: contractile index = (percentage gel contraction^{perturbation}/percentage gel contraction^{control}). Thus, an increase in the contractile index is an increase in contraction.

SUPLEMENTARY FIGURES

Fig. S1

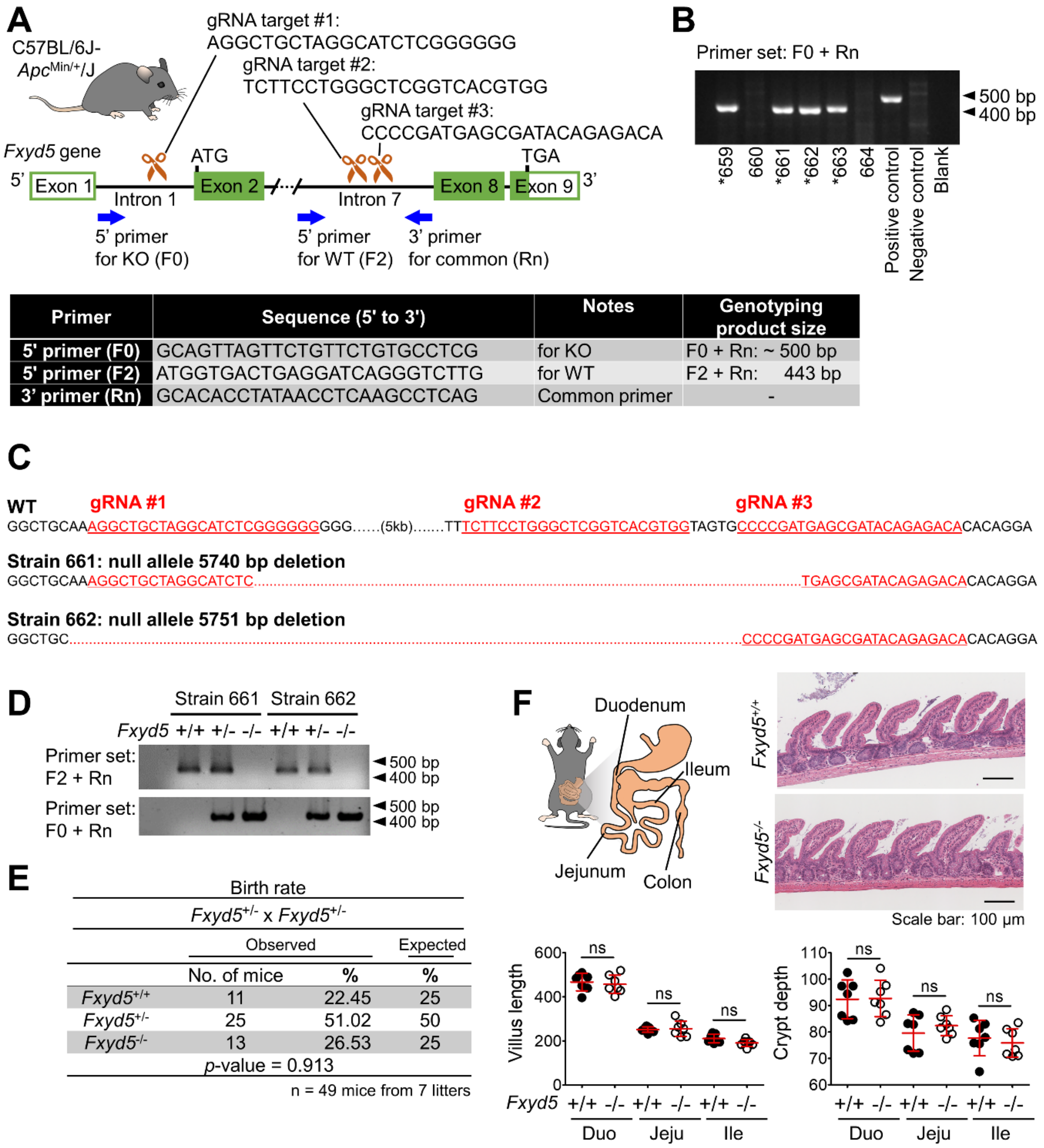

Primer	Sequence (5' to 3')	Notes	Genotyping product size
5' primer (F0)	GCAGTTAGTTCTGTTCTGTGCCTCG	for KO	F0 + Rn: ~ 500 bp
5' primer (F2)	ATGGTGACTGAGGATCAGGGTCTTG	for WT	F2 + Rn: 443 bp
3' primer (Rn)	GCACACCTATAACCTCAAGCCTCAG	Common primer	-

Birth rate

Fxyd5$^{+/-}$ x *Fxyd5*$^{+/-}$

	Observed		Expected
	No. of mice	%	%
Fxyd5$^{+/+}$	11	22.45	25
Fxyd5$^{+/-}$	25	51.02	50
Fxyd5$^{-/-}$	13	26.53	25
p-value = 0.913			

n = 49 mice from 7 litters

Fig. S1. Genetic ratio of pups born in *Fxyd5*$^{+/-}$ litters and analysis of intestines of wild-type and *Fxyd5*$^{-/-}$ mice. (**A**) Schematic view showing the targeting of exons 2-7 of the *Fxyd5* gene using 3 guide RNAs (gRNAs). Arrows indicate the 5'- and 3'- primer pairs used for genotyping. (**B**) Detection of the *Fxyd5*-deleted chromosome by PCR. Among the six pups from zygote injection, four showed deletion bands. Asterisks indicate pups harboring deleted alleles. (**C**) Sequencing analysis of strains 661 and 662. Strains 661 and 662 carried 5740-bp and 5751-bp deletions between the gRNA #1- and gRNA #3-targeted regions. Because the deleted region contained the first ATG sequence at exon 2 and most of the coding region, chromosomes harboring deletions in strains 661 and 662 were null. (**D**) Generation of *Fxyd5*-KO founder from strain 661. At least 5 successive breedings with C57BL/6J mice were conducted for the experiments. (**E**) The pups born from *Fxyd5*$^{+/-}$ litters followed the Mendelian ratio.

Statistical values were estimated by the chi-square test. (**F**) H&E-stained images of wild-type or *Fxyd5*$^{-/-}$ mouse intestines. The lengths of crypts and villi were measured in mice with the indicated genotype (n = 7/group). Data indicate the means ± SEMs. Statistical comparisons of the two groups were carried out by Student's t-test. ns indicates no significance.

Fig. S2

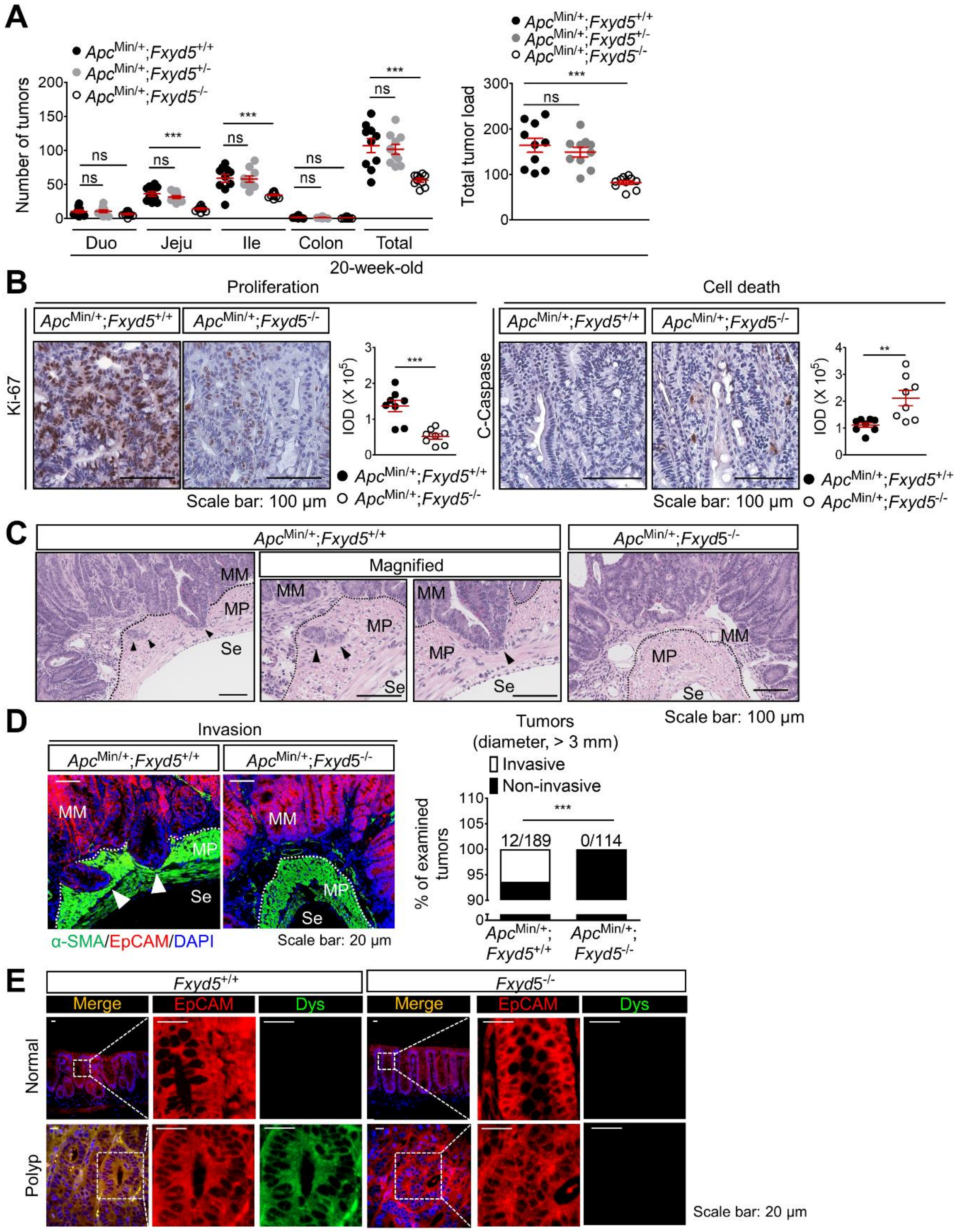
A
ApcMin/+;Fxyd5+/+
ApcMin/+;Fxyd5+/-
ApcMin/+;Fxyd5-/-
Number of tumors
Duo
Jeju
Ile
Colon
Total
20-week-old
Total tumor load
ns
***
B
Proliferation
Cell death
Ki-67
C-Caspase
IOD (X 10^5)
Scale bar: 100 μm
C
Magnified
MM
MP
Se
Scale bar: 100 μm
D
Invasion
α-SMA/EpCAM/DAPI
Scale bar: 20 μm
Tumors
(diameter, > 3 mm)
Invasive
Non-invasive
% of examined tumors
12/189
0/114
E
Fxyd5+/+
Fxyd5-/-
Merge
EpCAM
Dys
Normal
Polyp
Scale bar: 20 μm

Fig. S2 (Continued)

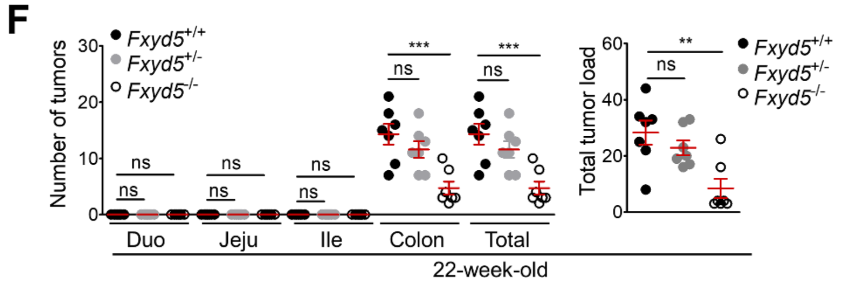

Fig. S2. The impact of dysadherin deficiency on intestinal tumorigenesis in *Apc*$^{Min/+}$ mice and AOM/DSS-treated mice. (**A**) Number of intestinal tumors and total tumor load per mouse were quantified in 20-week-old *Apc*$^{Min/+}$ mice (*Apc*$^{Min/+}$;*Fxyd5*$^{+/+}$, n = 12; *Apc*$^{Min/+}$;*Fxyd5*$^{+/-}$, n = 14; *Apc*$^{Min/+}$;*Fxyd5*$^{-/-}$, n = 20). Total tumor number and load from *Apc*$^{Min/+}$;*Fxyd5*$^{+/+}$ and *Apc*$^{Min/+}$;*Fxyd5*$^{-/-}$ mice are also reported in Fig. 1C. (**B**) Representative images of immunohistochemical detection of Ki67 and cleaved-caspase 3 in the intestinal tumors of 20-week-old *Apc*$^{Min/+}$ mice. Nuclei were counterstained with hematoxylin. Graphs show the protein levels of Ki67 and cleaved-caspase 3 in the intestinal tumors of 20-week-old ApcMin/+ mice (*Apc*$^{Min/+}$;*Fxyd5*$^{+/+}$, n = 8; *Apc*$^{Min/+}$;*Fxyd5*$^{-/-}$, n = 8). (**C**) Representative images of hematoxylin and eosin stained intestinal tumor tissues from 20-week-old *Apc*$^{Min/+}$ mice. In control mice (*Apc*$^{Min/+}$;*Fxyd5*$^{+/+}$), tumor cells (arrowheads) invaded the muscularis mucosae (MM, dotted line) and muscularis propria (MP), reaching the serosa (Se). In dysadherin-deficient mice (*Apc*$^{Min/+}$;*Fxyd5*$^{-/-}$), tumor cells remained above the MM. (**D**) IF analysis of the intestines of 20-week-old *Apc*$^{Min/+}$ mice labeled for the epithelial marker EpCAM and α-smooth muscle actin (SMA). The stacked bar graph shows the percentage of invasive or noninvasive tumors among examined tumors per group. . (**E**) IF showing the acquisition of dysadherin expression in the intestinal tumor epithelium (EpCAM^{+}) of *Fxyd5*$^{+/+}$ mice and complete elimination of dysadherin expression in the intestinal tumor epithelium of *Fxyd5*$^{-/-}$ mice in the AOM/DSS-induced intestinal tumorigenesis mouse model. (**F**) The number of colonic tumors and total tumor load in 22-week-old AOM/DSS mice (*Fxyd5*$^{+/+}$, n = 7; *Fxyd5*$^{+/-}$, n = 7; *Fxyd5*$^{-/-}$, n = 7). Mice were divided into 3 groups according to the diameter of the tumors in the intestines: small tumors, < 3 mm; medium tumors, > 3 mm and < 5 mm; and large tumors, > 5 mm. Tumor load was calculated according to the following formula: tumor load = (number of small tumors) × 1 + (number of medium tumors) × 2 + (number of large tumors) × 3. Total tumor number and load from *Fxyd5*$^{+/+}$ and *Fxyd5*$^{-/-}$ mice are also reported in Fig. 1D. In all panels, data indicate the means ± SEMs; *, **, and *** indicate $p < 0.05$, < 0.01, and < 0.001, respectively; ns indicates no significance. Statistical comparisons were performed using one-way ANOVA with Dunnett's multiple comparison tests for 3 or more groups. AOM: azoxymethane, DSS: dextran sulfate sodium. C-Caspase3: cleaved-Caspase 3, Dys: dysadherin, IOD: integrated optical density.

Fig. S3

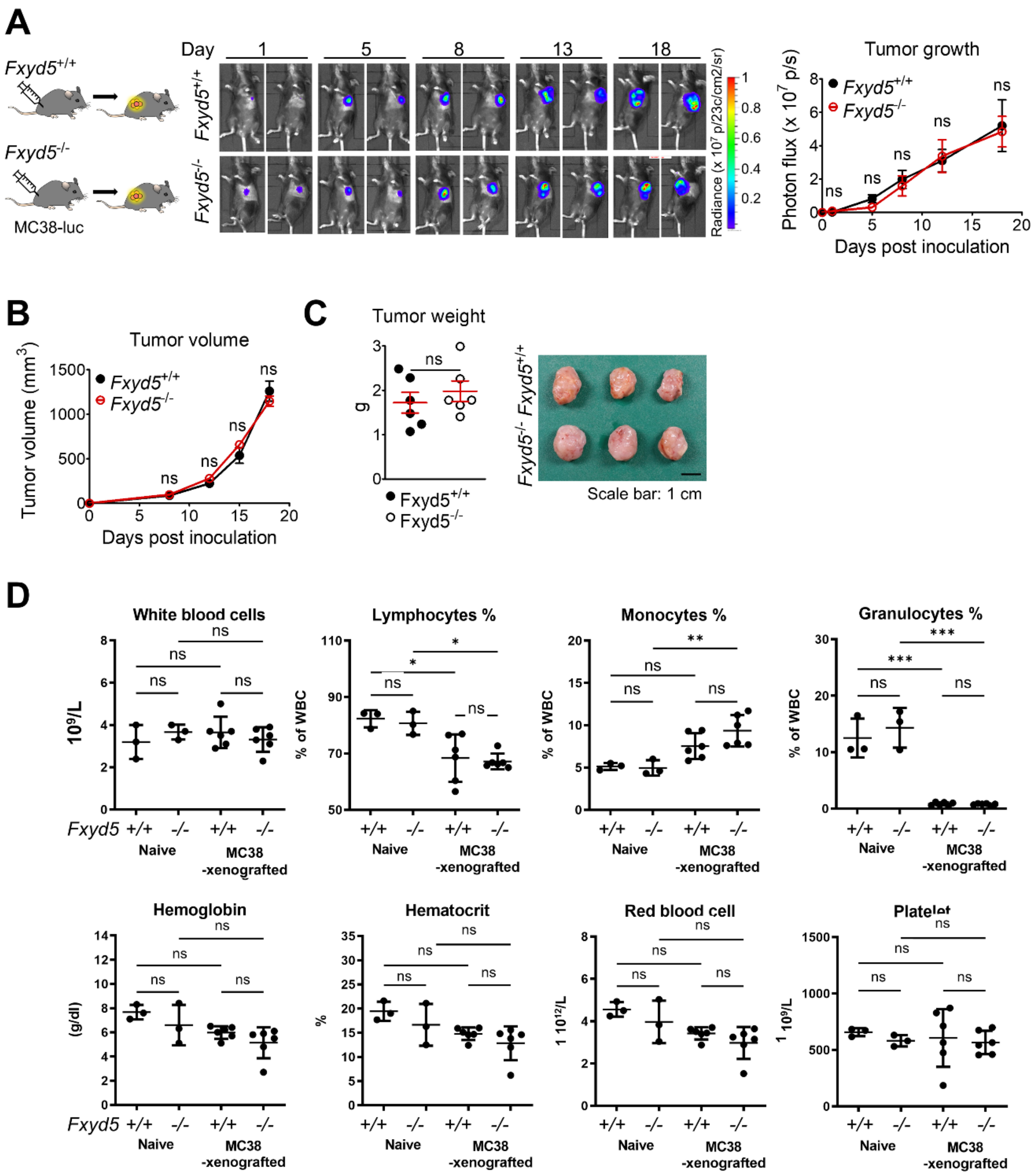

Fig. S3. Effect of stromal depletion of dysadherin in a syngeneic mouse model. (**A**) Left: schematic view of syngeneic mouse model. Luciferase-labeled MC38 cells (C57BL/6 mice-derived colon carcinoma cell line) were inoculated s.c. into C57BL/6J mice (5 x 10^4/mouse). Middle and right: tumor growth was monitored by measuring luciferase activity until necropsy (*Fxyd5*$^{+/+}$, n = 6; *Fxyd5*$^{-/-}$, n = 6). (**B**) Tumor volumes were calculated according to the following formula: tumor volume = length x width^2/2. (**C**) Left: Tumor weights were measured after necropsy. Right: representative images of MC38 tumors grown in *Fxyd5*$^{+/+}$ or *Fyxd5*$^{-/-}$ mice. (**D**) Immune cell and

hematological parameters in naive control and MC38-inoculated mice with the indicated genotypes were compared. In all panels, data indicate the means ± SEMs; *, **, and *** indicate $p < 0.05$, < 0.01, and < 0.001, respectively; ns indicates no significance. Statistical comparisons between 2 groups were performed using Student's t-test or two-way ANOVA with the Bonferroni multiple comparison test, and for 3 or more groups using one-way ANOVA with Dunnett's multiple comparison tests.

Fig. S4

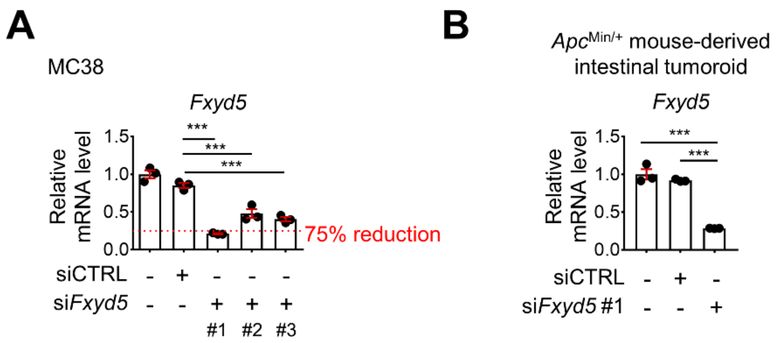

Fig. S4. **Validation of siRNA efficacy using MC38 cells and intestinal tumoroids derived from *Apc*$^{\mathrm{Min/+}}$ mice.** (**A**) MC38 cells were transfected with scrambled siRNAs (siCTRL) and siRNAs targeting mouse *Fxyd5* (si*Fxyd5*). The *Fxyd5* transcript level was determined by real-time RT-qPCR 96 h after transfection. Relative *Fxyd5* levels were normalized to endogenous *Hprt*. (**B**) Intestinal tumoroids from *Apc*$^{\mathrm{Min/+}}$ mice were dissociated into single cells, and siCTRL or si*Fxyd5* was transfected by electroporation (NEPA Gene, Chiba, Japan). The level of mRNA expression was determined 96 h after transfection. In all panels, data indicate the means ± SEMs; *** indicates $p < 0.001$. Statistical comparisons were performed using one-way ANOVA with Dunnett's multiple comparison tests for 3 or more groups.

Fig. S5

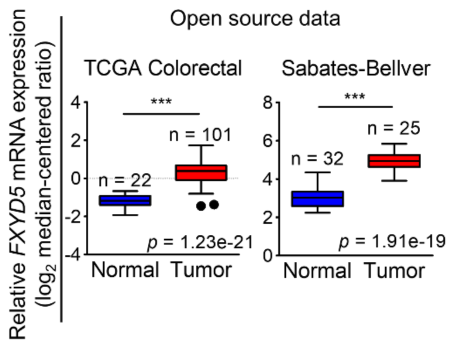

Fig. S5. Evaluation of dysadherin expression in CRC patients. mRNA expression data obtained from an open-source database (R2: Genomic analysis and visualization platform) showing *FXYD5* expression in normal tissues and tumor tissues. Box plots display the median values with upper and lower quartiles, and Tukey whisker plots show the ranges. Statistical significance was determined by non-paired Student's t-tests.

Fig. S6

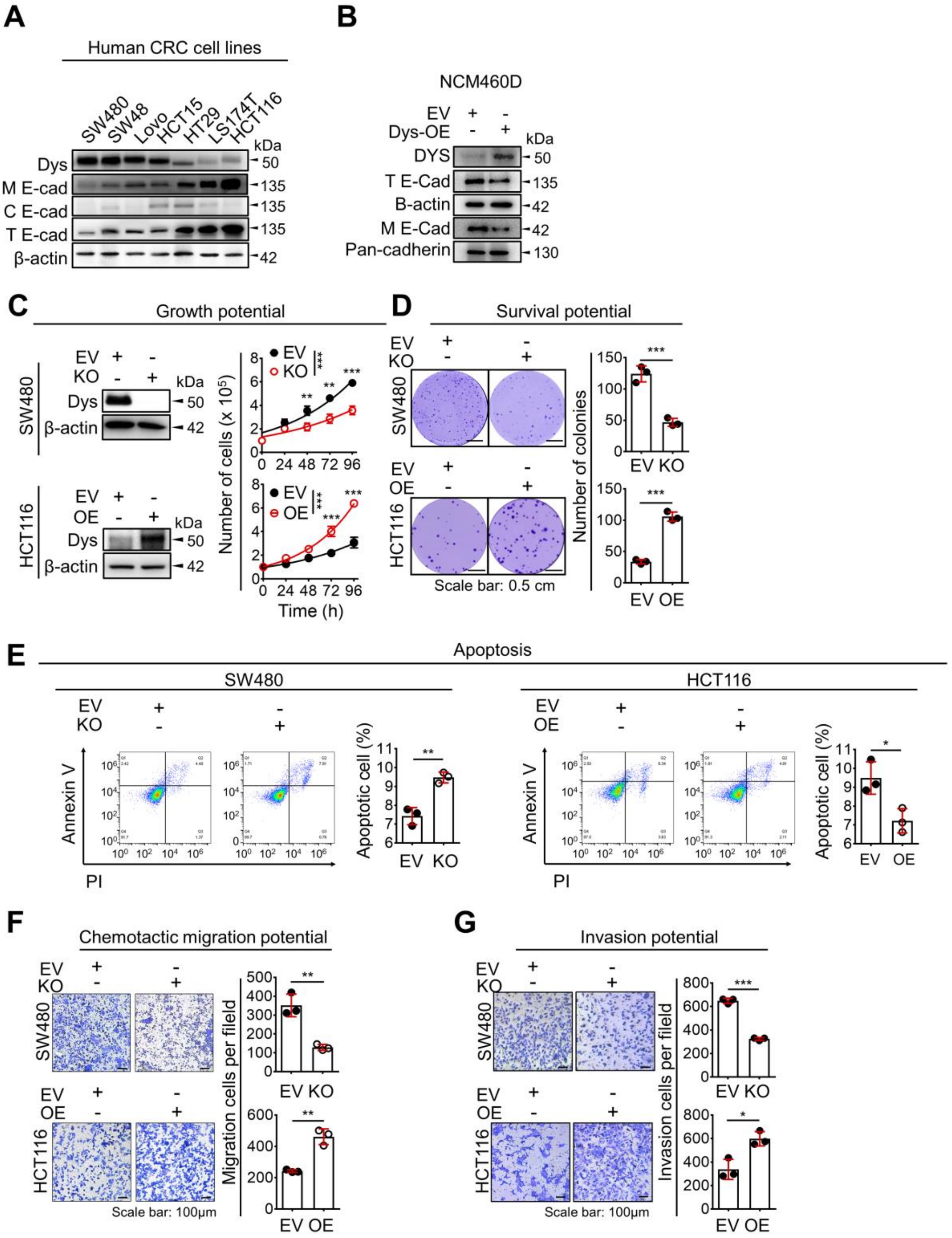

Fig. S6. Effect of dysadherin expression on CRC cell behavior including cell growth, survival, apoptosis, migration, and invasion potential. (**A**) Immunoblot analyses in a panel of human CRC cell lines. (**B**)

Immunoblot analyses in a panel of human intestinal epithelial cell line, NCM460D. (**C**) Immunoblots confirming the establishment of dysadherin-KO and dysadherin-OE CRC cell lines. Numbers of viable cells at the indicated time points measured with an automated cell counter (n = 5/group). (**D**) Survival potential measured by clonogenic assays (n = 3/group). (**E**) Dysadherin-KO and dysadherin-OE cells were cultured in serum-free media for 72 h. Annexin V/PI staining and subsequent FACS analyses were performed to determine the population of apoptotic cells (n = 3/group). (**F**) Boyden chamber assays without Matrigel matrix-coated membranes were performed to compare the chemotactic migration potential of dysadherin-KO or -OE cells (n = 3/group). (**G**) Boyden chamber assays with Matrigel matrix-coated membranes were performed to compare the invasion potential (n = 3/group). In all panels, data are reported as means ± SEMs; *, **, and *** indicate $p < 0.05$, < 0.01, and < 0.001, respectively. Statistical comparisons between 2 groups were performed using Student's t-test. EV: empty vector, PI: propidium iodide.

Fig. S7

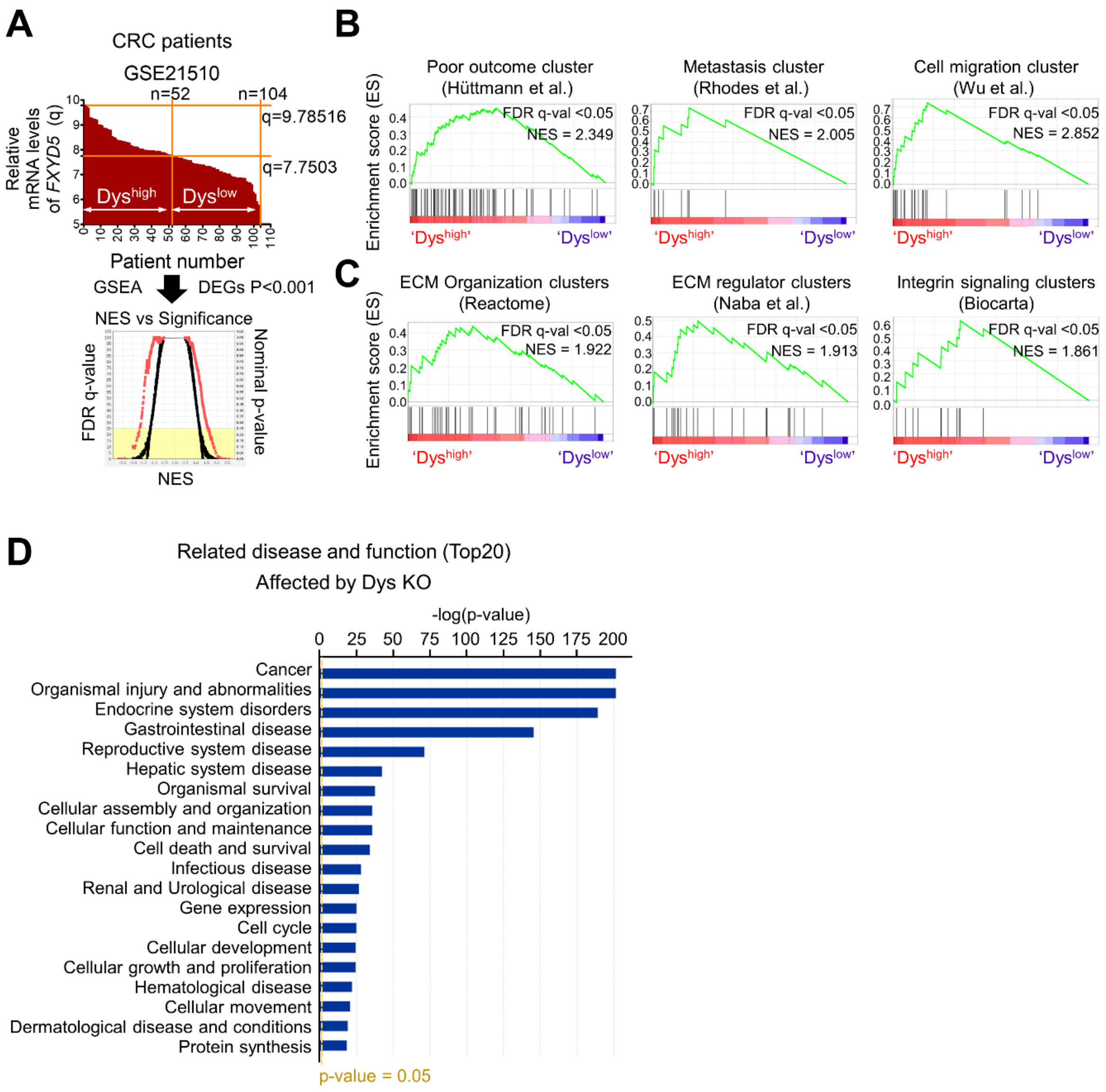

Fig. S7. Bioinformatics analyses to identify dysadherin-associated mechanisms. (**A**) Scheme showing groups of patients with CRC according to dysadherin expression. mRNA expression data from patient tumors were obtained from the GEO database (GSE21510), and patients were divided into 2 groups according to median dysadherin level (dysadherin^{high}, n = 52; dysadherin^{low}, n = 52). The list of differentially expressed genes (DEGs) was obtained through R2 analyses with the GEO platform ($p < 0.001$) and used for gene set enrichment analyses (GSEA) to determine the associated gene signatures. (**B**) Gene signatures related to features of cancer malignancy, such as poor outcome clusters, metastatic clusters, and cell migration clusters, were significantly enriched in the dysadherin^{high} tumors. (**C**) Significant enrichment of ECM receptor pathway genes, such as genes involved in ECM organization, ECM regulators, and integrin signaling, was observed in the dysadherin^{high} tumors of patients with CRC. (**D**) The list of DEGs (n = 4,437, $p < 0.05$) in dysadherin-KO SW480 cells was subjected to IPA. The bar graph shows the top 20 significant diseases and functions related to dysadherin KO. Cancer is most significantly affected by dysadherin KO. Dys: dysadherin, FDR: false discovery rate, NES: normalized enrichment score.

Fig. S8

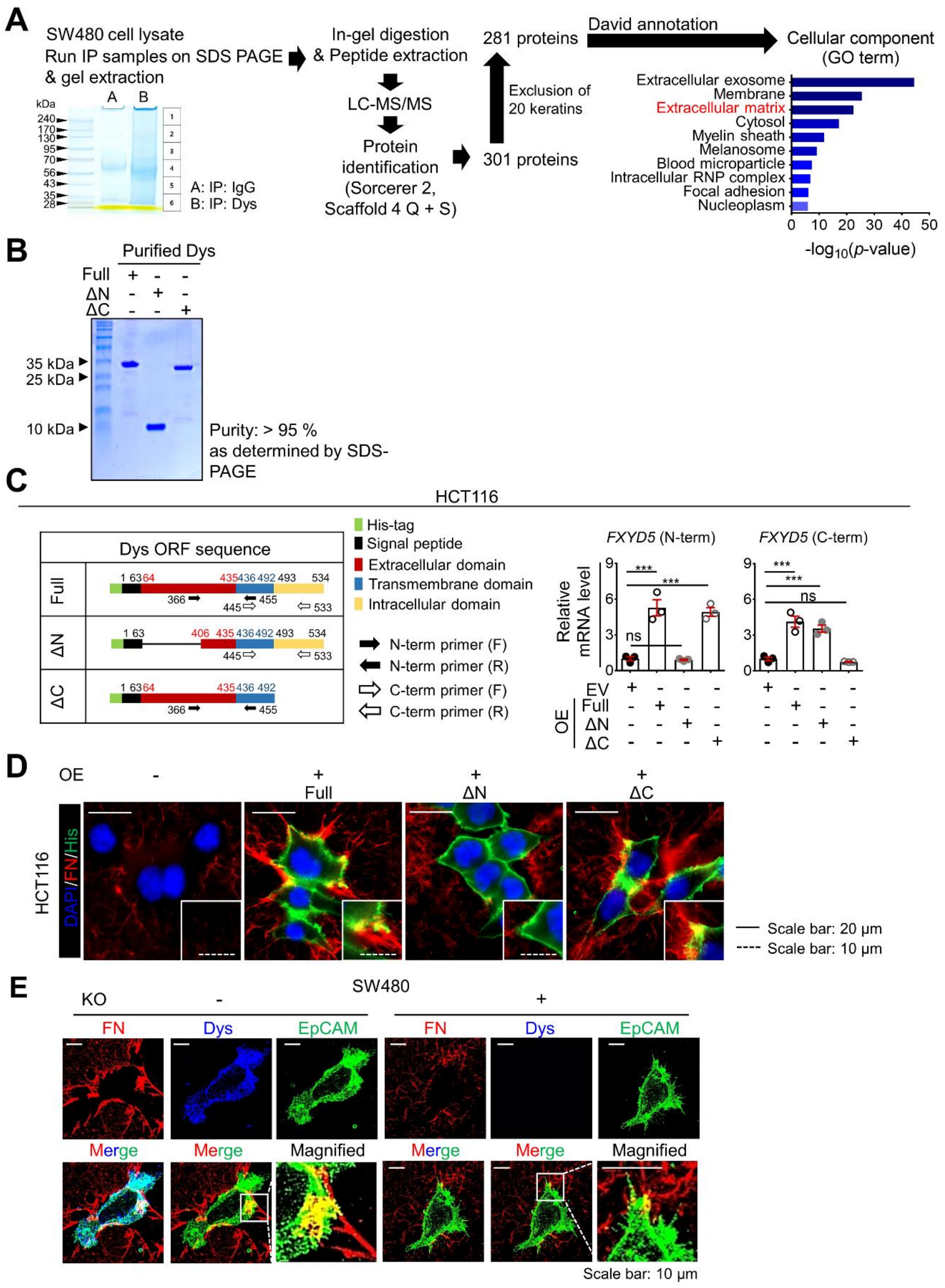
A
SW480 cell lysate
Run IP samples on SDS PAGE
& gel extraction
A: IP: IgG
B: IP: Dys
In-gel digestion
& Peptide extraction
LC-MS/MS
Protein identification (Sorcerer 2, Scaffold 4 Q + S)
301 proteins
Exclusion of 20 keratins
281 proteins
David annotation
Cellular component (GO term)
Extracellular exosome
Membrane
Extracellular matrix
Cytosol
Myelin sheath
Melanosome
Blood microparticle
Intracellular RNP complex
Focal adhesion
Nucleoplasm
-log10(p-value)
B
Purified Dys
Full
ΔN
ΔC
35 kDa
25 kDa
10 kDa
Purity: > 95 % as determined by SDS-PAGE
C
HCT116
Dys ORF sequence
His-tag
Signal peptide
Extracellular domain
Transmembrane domain
Intracellular domain
N-term primer (F)
N-term primer (R)
C-term primer (F)
C-term primer (R)
FXYD5 (N-term)
FXYD5 (C-term)
Relative mRNA level
EV
OE
D
HCT116
DAPI/FN/His
Scale bar: 20 μm
Scale bar: 10 μm
E
SW480
KO
FN
Dys
EpCAM
Merge
Magnified
Scale bar: 10 μm

Fig. S8 (Continued)

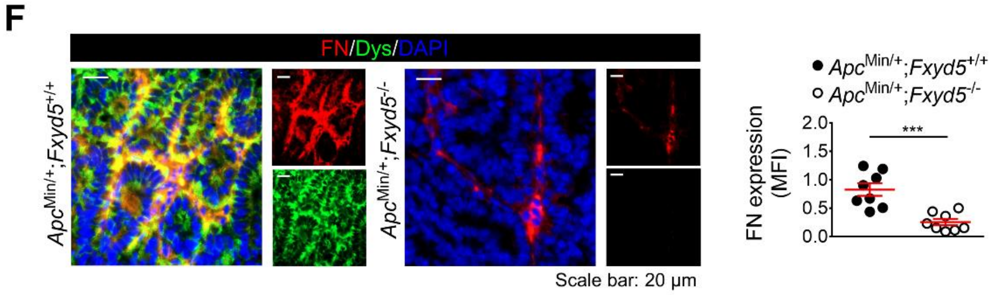

Fig. S8. Identification and validation of binding between dysadherin and fibronectin. (**A**) Schematic flow for identification of dysadherin-binding proteins based on co-immunoprecipitation (co-IP) with anti-dysadherin (M53) monoclonal antibody and subsequent liquid chromatography with tandem mass spectrometry (LC-MS). LC-MS identified a total of 301 proteins in the co-IP samples. After exclusion of 20 keratins as accidental or unavoidable contaminants of proteomics assays, functional annotation of the remaining 281 proteins was performed by DAVID functional annotation analysis (https://david.ncifcrf.gov/), which showed the significant enrichment of the ECM proteins. Fibronectin was one of the ECM proteins significantly enriched by co-IP. (**B**) Confirmation of purified recombinant dysadherin constructs generated from *E. coli*. (**C**) Schematic of the dysadherin open reading frame (ORF) sequence used to establish HCT116 cell lines overexpressing wild-type (full length) or mutant dysadherin. Arrows indicate the binding sites of primer sets that were designed to detect mutant forms of dysadherin. Right: confirmation of wild-type or mutant dysadherin OE by RT-qPCR analysis (n = 3/group). (**D**) IF analyses for visualizing His-tagged dysadherin (green) and fibronectin (red). His-tagged wild-type or mutant dysadherin was transfected into HCT116 cells, and the localization of dysadherin was visualized by staining with an anti-His antibody. OE of wild-type or ΔC-mutant dysadherin increased the colocalization of dysadherin and fibronectin on the cellular membrane, while OE of ΔN-mutant dysadherin did not. (**E**) IF staining for fibronectin and dysadherin in SW480 cells with and without dysadherin KO. (**F**) IF staining for fibronectin and dysadherin in intestinal tumor tissues from $Apc^{Min/+}$;$Fxyd5^{+/+}$ and $Apc^{Min/+}$;$Fxyd5^{-/-}$ mice. Data indicate the means ± SEMs; *** indicates $p < 0.001$ and ns indicates no significance. Statistical comparisons between 3 groups were performed using one-way ANOVA with Dunnett's multiple comparison tests. Statistical comparisons between 2 groups were performed using Student's t-test. Dys: dysadherin, FN, fibronectin, Full: full length, GO: gene ontology, IgG: immunoglobulin G, MFI: mean fluorescence intensity, SDS-PAGE: sodium dodecyl sulfate polyacrylamide gel electrophoresis, ΔC: ΔC-mutant, ΔN: ΔN-mutant.

Fig. S9

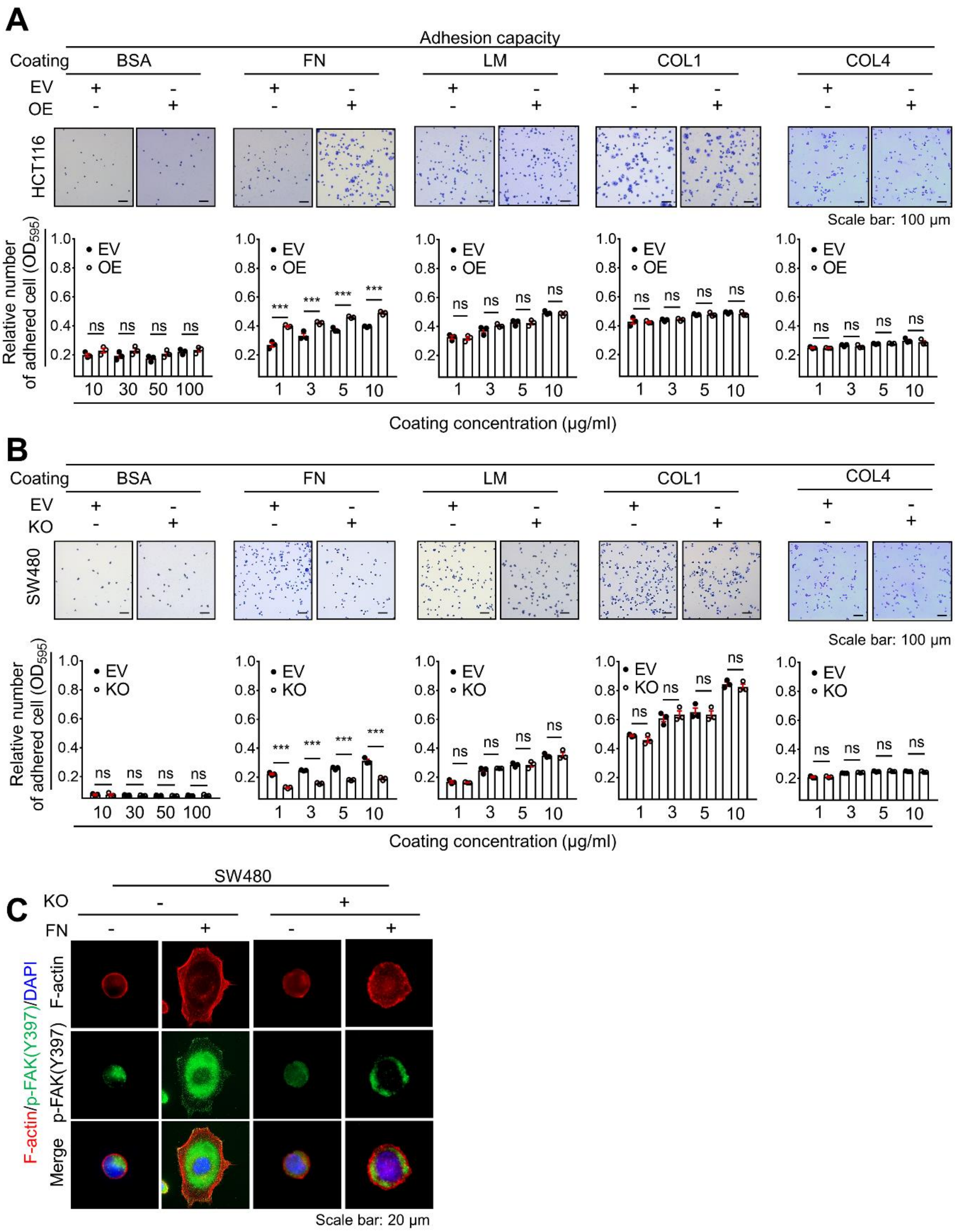
A
Adhesion capacity
Coating
BSA
FN
LM
COL1
COL4
EV
OE
HCT116
Scale bar: 100 μm
Relative number of adhered cell (OD_{595})
Coating concentration (μg/ml)
B
KO
SW480
C
F-actin/p-FAK(Y397)/DAPI
F-actin
p-FAK(Y397)
Merge
Scale bar: 20 μm

Fig. S9 (Continued)

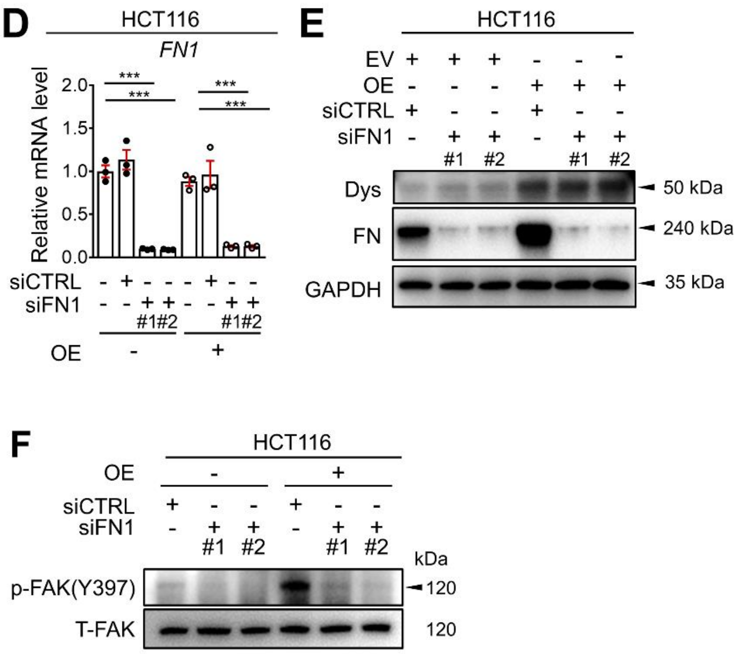

Fig. S9. Effect of dysadherin on cancer cell adhesion capacity to fibronectin and confirmation of fibronectin knockdown with siRNAs. (**A,B**) Cancer cell adhesion to various ECM proteins was determined by measuring the relative number of bound cells on culture plates coated with the indicated proteins 1 h after cell seeding. Both HCT116 and SW480 cells showed greater adhesive capacity with higher concentrations of ECM coating. Dysadherin OE increased the number of cells that adhered to fibronectin, while dysadherin KO reduced the number of adherent cells. The extent of cell adhesion to laminin or collagen was not affected by dysadherin OE or KO. Upper: representative images of adhered cells stained with crystal violet. Bottom: graphs show the relative cell number determined by measuring absorbance at OD 595 nm (n = 3/group). (**C**) Activation of FAK (p-FAK) in SW480 cells 1 h after cell seeding on culture plates with or without fibronectin coating, visualized by IF. (**D**) The gene silencing effects of siRNAs targeting fibronectin (si*FN1*) were confirmed by RT-qPCR analyses 96 h after transfection. Cells transfected with scrambled siRNAs (siCTRL) were used as the control. (**E**) Immunoblot analyses show a reduction in fibronectin protein levels in si*FN1*-transfected cells without an effect on dysadherin protein levels. (**F**) FAK activation in 4-day cultures of CRC cells after gene silencing of fibronectin. In all panels, data indicate the means ± SEMs; *** indicates $p < 0.001$ and ns indicates no significance. Statistical comparisons were performed using two-way ANOVA with the Bonferroni multiple comparison test for 2 groups, or using one-way ANOVA with Dunnett's multiple comparison tests for 3 or more groups. BSA: bovine serum albumin, COL1: collagen type 1, Dys: dysadherin, FN: fibronectin, LM: laminin, OD: optical density.

Fig. S10

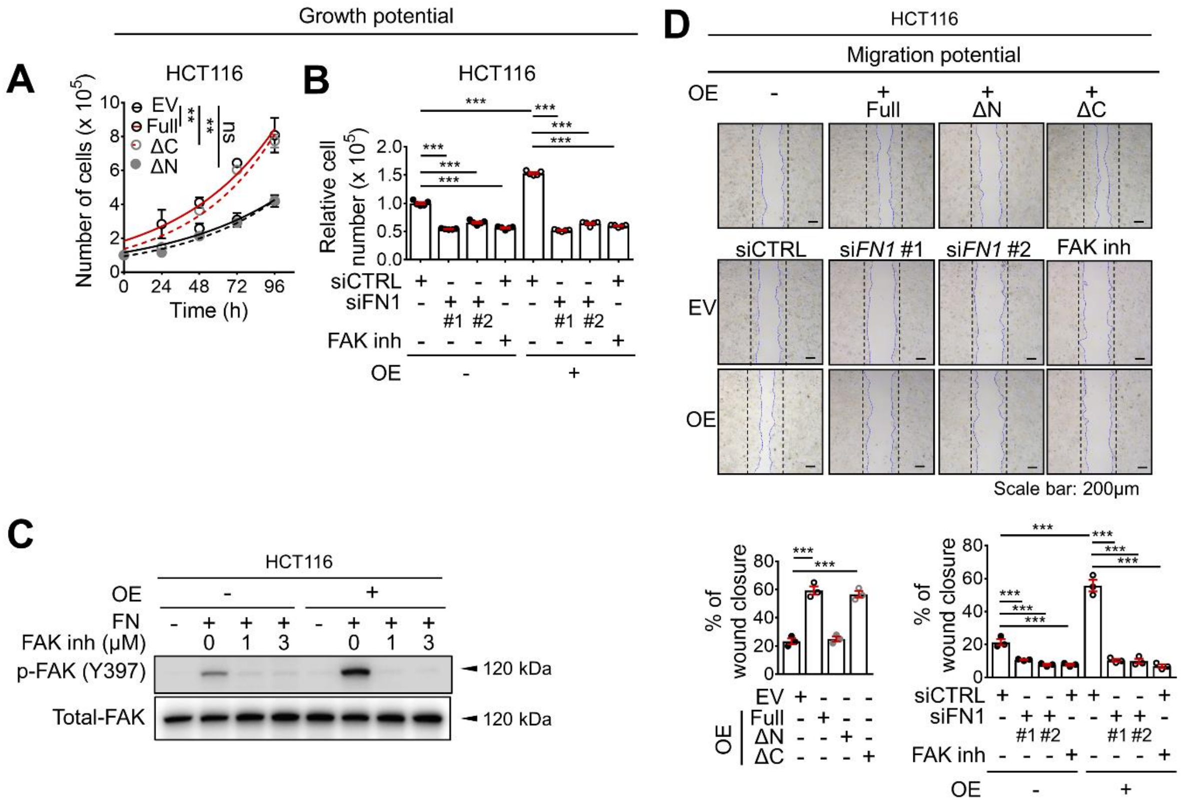

Growth potential
A
HCT116
Number of cells (x 10^5)
Time (h)
EV
Full
ΔC
ΔN
**
**
ns
B
HCT116
Relative cell number (x 10^5)
siCTRL
siFN1
FAK inh
OE
C
HCT116
OE
FN
FAK inh (μM)
p-FAK (Y397)
Total-FAK
120 kDa
120 kDa
D
HCT116
Migration potential
OE
Full
ΔN
ΔC
siCTRL
siFN1 #1
siFN1 #2
FAK inh
EV
OE
Scale bar: 200μm
% of wound closure
EV
Full
ΔN
ΔC
siCTRL
siFN1
FAK inh
OE

Fig. S10Continued)

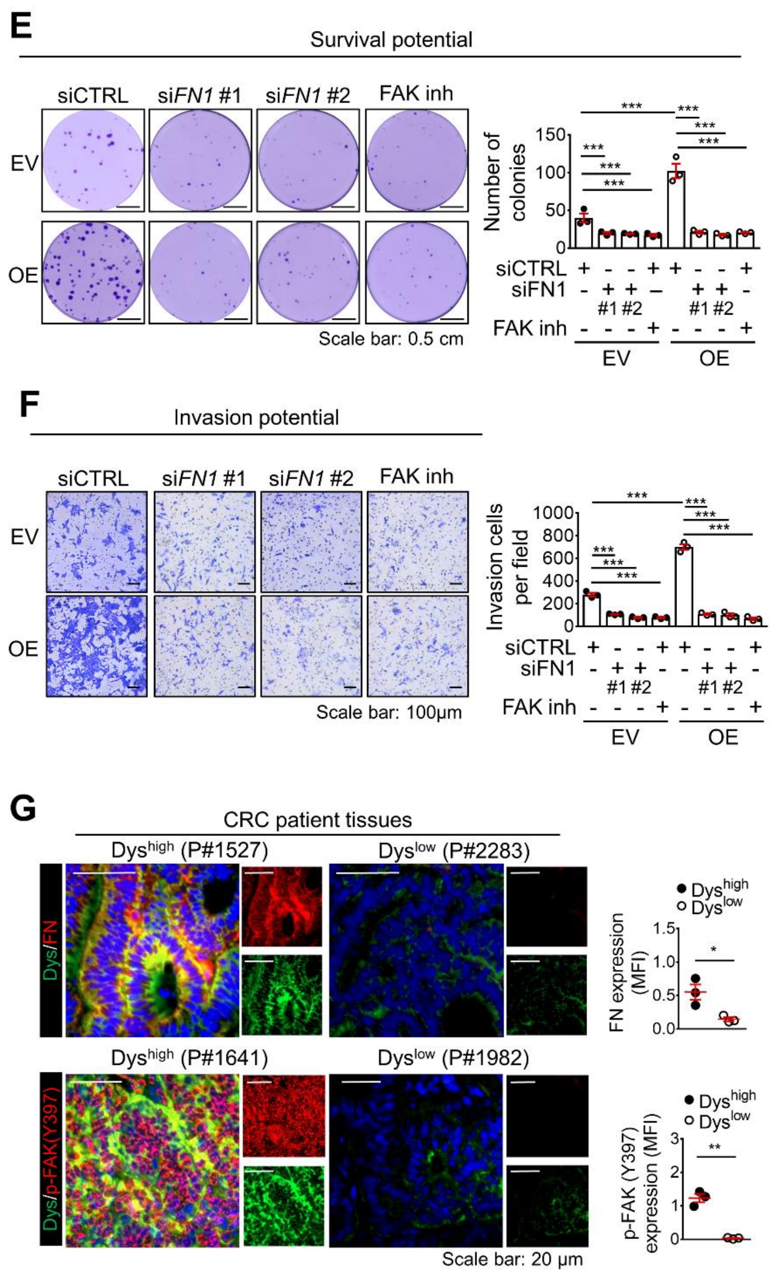

Fig. S10. Functional involvement of fibronectin/integrin/FAK axis in dysadherin-mediated protumor activity. (**A**) The number of viable cells was measured with an automated cell counter (Countess II, ThermoFisher Scientific, Waltham, MA, USA) at the indicated time points (n = 3/group) in HCT116 cells overexpressing wild-type (full length) or mutant dysadherin. EV-transfected cells were used as controls. Deletion of the extracellular domain of dysadherin (ΔN-mutant) abrogated the dysadherin-induced increase in tumor growth, while the ΔC-mutant did not. (**B**) To determine the effect of fibronectin and FAK signaling on cancer cell growth, dysadherin-OE or control cells were transfected with control siRNA (siCTRL) or siRNAs targeting fibronectin (si*FN1*). At 24 h after transfection, cells were detached, reseeded, and incubated for 12 h for cell attachment. Then cells were incubated for 36 h with or without FAK inhibitor treatment (VS-4718, 3 μM). The relative cell numbers were

determined by MTT assays (n = 3/group). (**C**) Immunoblot confirming the inhibitory effects of FAK inhibitor (VS-4718) on fibronectin-induced FAK activation. Cells were seeded on fibronectin-coated culture plates with or without VS-4718, at 1 or 3 μM. After 4 h, the cells were lysed and whole-cell extracts were subjected to immunoblot analyses to visualize the FAK activation status. (**D**) The migration potential of HCT116 cells overexpressing wild-type or mutant dysadherin were compared by wound healing assays. To determine the effect of fibronectin and FAK signaling on cancer cell migration, cells were transfected as described in (B). At 24 h after transfection, cells were detached and reseeded for a wound healing assay. The black dotted line indicates initial wound area, and the blue line indicates the cell boundaries determined after a 48-h incubation in serum-free media. FAK inhibitor (VS-4718, 1 μM) was added at the initial time point of the wound healing assay. Bar graphs show the percentage of wound closure (n = 3/group). (**E**) The survival potential of HCT116 cells overexpressing wild-type dysadherin or EV were compared. Cells were transfected with control siRNA (siCTRL) or siRNA targeting fibronectin (si*FN1*). The next day, cells were incubated for 24 h with or without FAK inhibitor (VS-4718, 3 μmol/L) then detached and reseeded for clonogenic assays (n = 3/group). (**F**) Comparison of invasion potential of HCT116 cells overexpressing wild-type dysadherin or EV by Boyden chamber assays. Fibronectin knockdown was performed as described in **e**. The transfected cells were detached and reseeded on the upper chamber with or without FAK inhibitor (3 μmol/L) in serum-free media. After a 24-h incubation, the cells that invaded through to the bottom of the membrane were fixed, stained with crystal violet, and counted under a phase-contrast microscope (n = 3/group). (**G**) IF staining for dysadherin and fibronectin or p-FAK in CRC patient tumors. Patients were classified as high or low expressors of dysadherin. Graphs show the protein levels of fibronectin or p-FAK within tumor epithelium in the indicated groups (n = 3/group). Statistical comparisons between 2 groups were performed using Student's t-test. EV: empty vector, FN: fibronectin, Full: full length, ΔC: ΔC-mutant, ΔN: ΔN-mutant.

Fig. S11

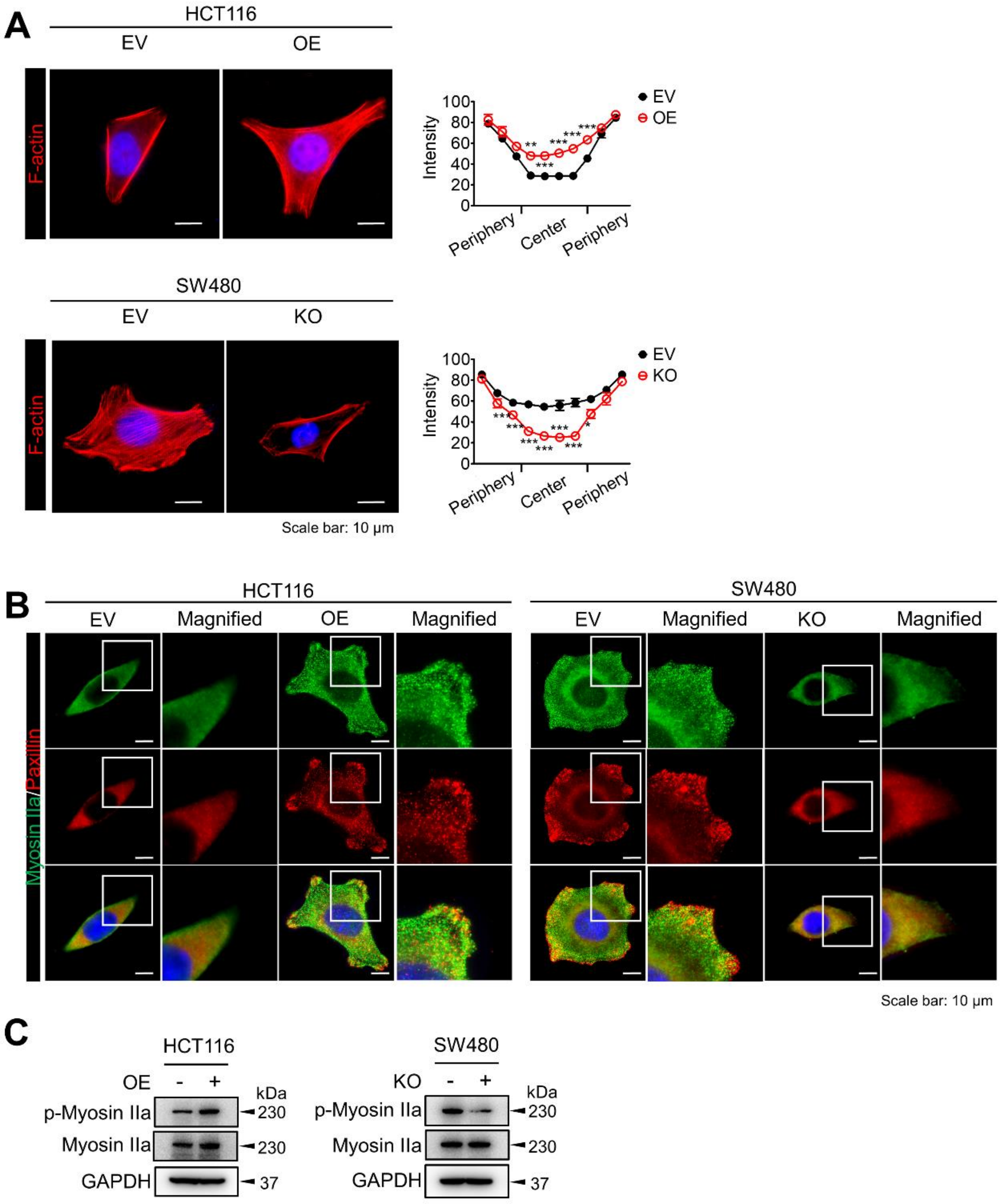

Fig. S11. Effect of dysadherin on F-actin stress fibers. (**A**) IF analysis of cytoskeletal tension by visualization of F-actin in CRC cells after dysadherin OE (in HCT116 cells) or KO (in SW480 cells). CRC cells were seeded on fibronectin-coated glass slides and stained with Alexa Fluor™ 555-conjugated phalloidin. Quantitative analysis of F-actin distribution was performed. (**B**) IF analysis of myosin IIa and paxillin in CRC cells after dysadherin OE (in HCT116 cells) or KO (in SW480 cells). (**C**) Immunoblot showing the expression of myosin II and phospho-myosin II in dysadherin OE (in HCT116 cells) or KO (in SW480 cells) cells.

Fig. S12

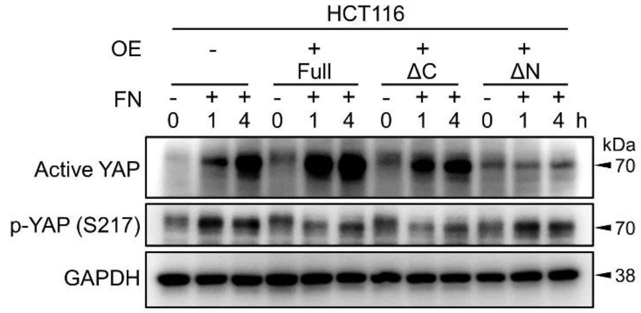

Fig. S12. Potential involvement of dysadherin in YAP activation during cell adhesion to fibronectin. Immunoblot analysis was performed in HCT116 cells overexpressing (OE) wild-type (full length) or mutant dysadherin to visualize the status of YAP activation. Cells were starved for 24 h in serum-free media, then detached and reseeded onto fibronectin-coated culture plates. After the indicated times, the cells were lysed, and whole-cell extracts were subjected to immunoblot analyses to visualize YAP activation status. EV-transfected cells were used as the control group. The extent of active YAP was increased and the extent of inactive YAP (phosphorylated-YAP; p-YAP) was decreased upon cell adhesion to fibronectin, suggesting that YAP activation occurs during cell adhesion to fibronectin. Dysadherin OE enhanced fibronectin-induced YAP activation. Deletion of the extracellular domain of dysadherin (ΔN-mutant) attenuated the dysadherin-mediated increase in YAP activation, while deletion of the intracellular domain (ΔC-mutant) did not. EV: empty vector, ΔC: ΔC-mutant, ΔN: ΔN-mutant.

Fig. S13

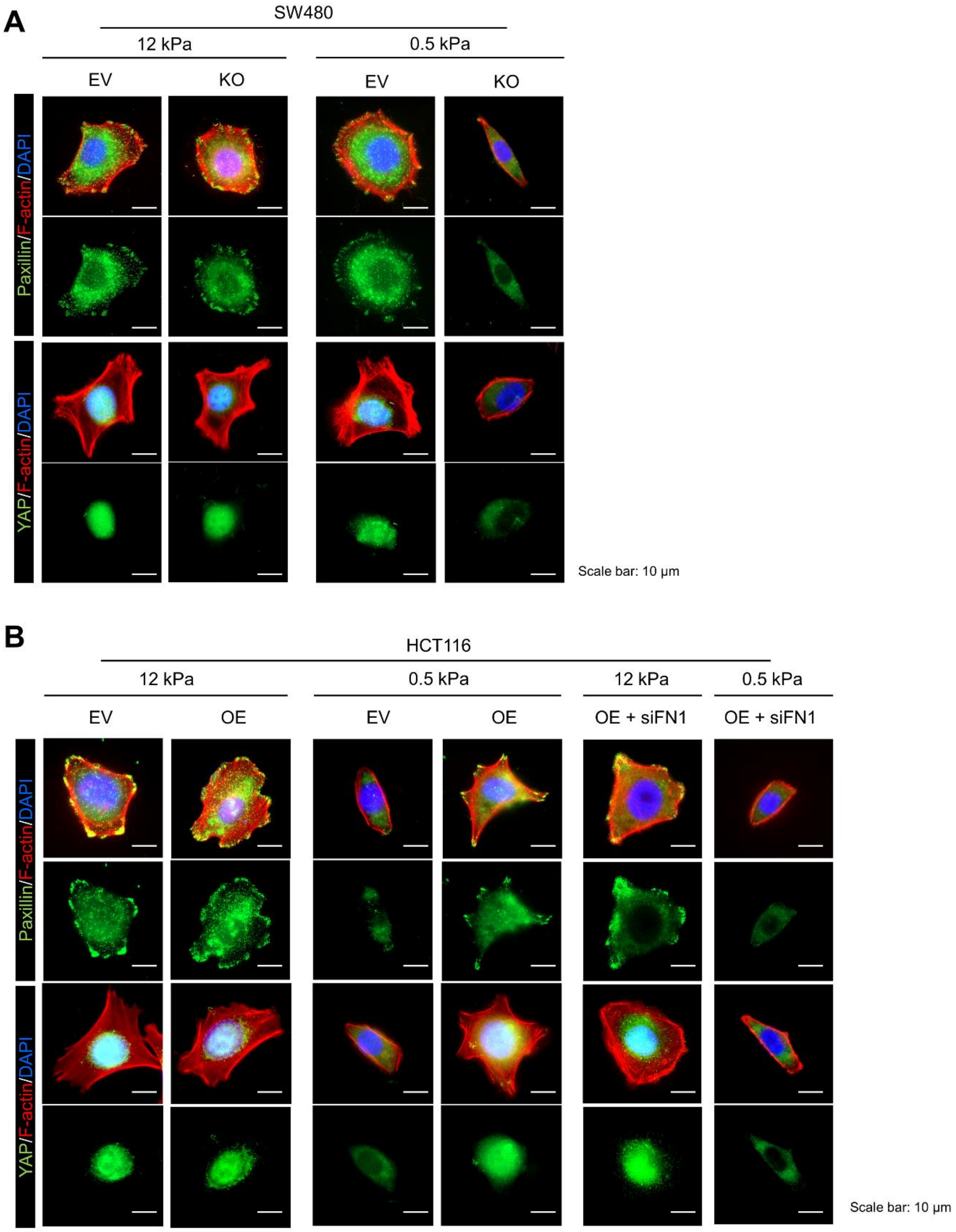

Fig. S13. Dysadherin facilitates cell adhesion to fibronectin, transduces mechanical force in CRC cells, and facilitates YAP mechanotransduction. (**A, B**) IF analysis of mechanotransduction by detected by staining of paxillin-positive focal adhesions and YAP in CRC cells with dysadherin OE (A) and dysadherin KO (B). The cells

were cultured on the plates coated with a matrix with a certain stiffness (0.5 kPa or 12 kPa) and stained with Alexa Fluor^{TM} 555-conjugated phalloidin or a nonconjugated anti-YAP antibody. There were no differences in cell spreading, FA assembly or YAP activation between WT, dysadherin-OE, and dysadherin-KO cells on the 12 kPa matrix. However, on the 0.5 kPa matrix, the changes in cell spreading, FA assembly, and YAP activation between WT, dysadherin-OE, and dysadherin-KO cells were detected. To confirm that the changes on the 0.5 kPa matrix were due to autonomous secretion of fibronectin from CRC cells, cell spreading, FA assembly, and YAP activation were evaluated in similar experiments after siRNA-induced silencing of fibronectin in dysadherin-OE cells.

Fig. S14

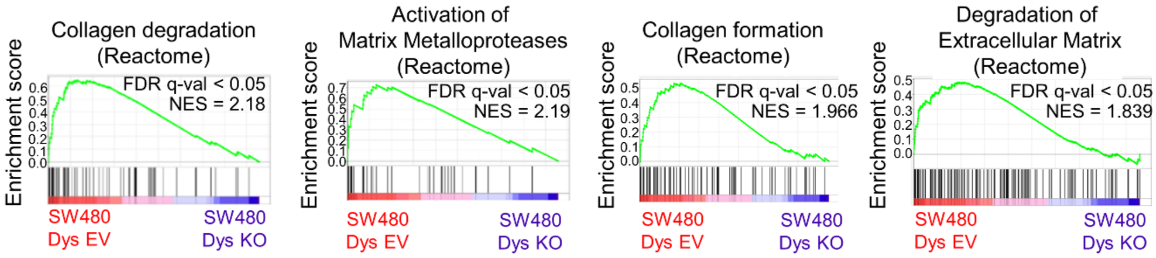

Fig. S14. ECM remodeling is a potential downstream mediator of dysadherin. GSEA was performed using the mRNA sequencing profiles of dysadherin-KO and control (EV-transfected) cells. Gene signatures associated with ECM remodeling were significantly enriched in control cells compared with those in dysadherin-KO cells.

SUPPLEMENTARY TABLES

Table S1. List of primers used for real-time RT-qPCR or genotyping

Target	Sequence	Application
Mouse *Fxyd5*	GCAGTTAGTTCTGTTCTGTGCCTCG	5' primer (F0) for KO, Genotyping
Mouse *Fxyd5*	ATGGTGACTGAGGATCAGGGTCTTG	5' primer (F2) for WT, Genotyping
Mouse *Fxyd5*	GCACACCTATAACCTCAAGCCTCAG	3’ primer (Rn) for KO and WT, Genotyping
Mouse *HPRT*	F: GCCTAAGATGAGCGCAAGTTG<br>R: TACTAGGCAGATGGCCACAGG	Real-time RT-qPCR, Endogenous control
Mouse *Fxyd5*	F: GAAAGGTACCCCTGCAGTCT<br>R: ACCAGCAGTCCCCGTTTC	Real-time RT-qPCR
Human FXYD5 (N-term)	F: TCCCACTGATGACACCACGA<br>R: AAACCAGATGGCTTGAGGG	For WT and ΔC, Real-time RT-qPCR
Human FXYD5 (C-term)	F: GTCGCAGCTGTGCTGTTCAT<br>R: CTGCAATGATTCCGGCATAACC	For WT and ΔN, Real-time RT-qPCR
Human *SPARC*	F: AGACAGGGGTACCTGTGGG<br>R: CACATGGGGGTGTTGCTCTC	Real-time RT-qPCR
Human *MMP2*	F: AAGGATGGCAAGTACGGCTT<br>R: AAACTTGCAGGGCTGTCCTT	Real-time RT-qPCR
Human *CD40*	F: AATGCCTTCCTTGCGGTGAA<br>R: TCTCACAGGCCTCACTCGTA	Real-time RT-qPCR
Human *CCL5*	F: TCAAGACAGCACGTGGACCT<br>R: CGGGCAATGTAGGCAAAGCA	Real-time RT-qPCR

Human *MMP9*	F: GTCGAAATCTCTGGGGCCTG<br>R: ATGTTGTGGTGGTGCCACTT	Real-time RT-qPCR
Human *TNF*	F: CAGGCAGGTTCTCTTCCTCTCA<br>R: AGGAGAAGAGGCTGAGGAACAA	Real-time RT-qPCR
Human *FOS*	F: CAGACTACGAGGCGTCATCC<br>R: CGTGGGAATGAAGTTGGCAC	Real-time RT-qPCR
Human *GPX4*	F: ACGCCCGATACGCTGAGT<br>R: TCACGCAGATCTTGCTGAACATA	Real-time RT-qPCR
Human *RAC2*	F: ATCAGCTACACCACCAACGC<br>R: ACGTCCGTCTGTGGATAGGA	Real-time RT-qPCR
Human *MMP14*	F: CCTTGGACTGTCAGGAATGAGG<br>R: TTCTCCGTGTCCATCCACTGGT	Real-time RT-qPCR
Human *CHRD*	F: CGCATCAGTGGACACATTGC<br>R: CCTCACTGCTTGTCCCTACC	Real-time RT-qPCR
Human *COL2A1*	F: CATCCCACCCTCTCACAGTT<br>R: GTCTCTGCCTTGACCCAAAG	Real-time RT-qPCR
Human *CDKN1A*	F: ACTTTGTCACCGAGACACCA<br>R: CAGCAGAGCAGGTGAGGTG	Real-time RT-qPCR
Human *IGFBP2*	F: TGCACATCCCCAACTGTGAC<br>R: TGTAGAAGAGATGACACTCGGG	Real-time RT-qPCR
Human *CEBPB*	F: TGATAAACTCTCTGCTTCTCCCT<br>R: GTTGCGTCAGTCCCGTGT	Real-time RT-qPCR
Human *JUNB*	F: ACCACGACGACTCATACACAG<br>R: CGAGCCCTGACCAGAAAAGT	Real-time RT-qPCR

Human *FN1*	F: AGCAAGCCCGGTTGTTATGA<br>R: CCCACTCGGTAAGTGTTCCC	Real-time RT-qPCR
Human *PPIA*	F: TGCCATCGCCAAGGAGTAG<br>R: TGCACAGACGGTCACTCAAA	Real-time RT-qPCR, Endogenous control
Human SGK1	F: CGGAATGTTCTGTTGAAGAATGTG<br>R: TGTCAGCAGTCTGGAAAGAGAAGT	Real-time RT-qPCR
Human BIRC5	F: AGCCCTTTCTCAAGGACCACC<br>R: TTGAAGCAGAAGAAACACTGGGC	Real-time RT-qPCR
Human ETV5	F: CAGTCAACTTCAAGAGGCTTGG<br>R: TGCTCATGGCTACAAGACGAC	Real-time RT-qPCR
Human MTSS1	F: ACCATCATCAGCGACATGAA<br>R: CACATCCTGGTGAGAGCAGA	Real-time RT-qPCR
Human VIM	F: ACCCGCACCAACGAGAAGGT<br>R: ATTCTGCTGCTCCAGGAAGCG	Real-time RT-qPCR
Human NDRG1	F: AAGATGGCGGACTGTGGC<br>R: TCAGGCGGGTCATGCTA	Real-time RT-qPCR
Human TNS1	F: TCAAGTGGAAGAACTTGTTTGCTT<br>R: CACGACAATATAGTGGAGGCACA	Real-time RT-qPCR
Human CDC20	F: GCCCACCAAGAAGGAACATC<br>R: TTTTCCACTGAGCCGAAGGA	Real-time RT-qPCR
Hunan DUT	F: GTCTCCTCGCTCGCCTTCT<br>R: GGTGAAATGGCGGGTGTCT	Real-time RT-qPCR
Human TGM2	F: AGAAGAGCGAAGGGACGTACTG<br>R: AGTCTACCACGTCGGCATTGAC	Real-time RT-qPCR

Human CTGF	F: CCAATGACAACGCCTCCTG	Real-time RT-qPCR
	R: TGGTGCAGCCAGAAAGCTC	
Human CAVIN2	F: AAGAGCGCATGGATAGGCAG	Real-time RT-qPCR
	R: AAGAGCGCATGGATAGGCAG	
Human FLNA	F: CATCAAGTACGGTGGTGACG	Real-time RT-qPCR
	R: ACATCCACCTCTGAGCCATC	

Table S2. List of siRNAs

Target		Sequence
Mouse *Fxyd5*	siRNA #1	Sense: CUC ACU AGU GGG AAG UGU A(dTdT)
		Antisense: UAC ACU UCC CAC UAG UGA G(dTdT)
Mouse *Fxyd5*	siRNA #2	Sense: CAC AUG GUC UCU CUU CCA U(dTdT)
		Antisense: AUG GAA GAG AGA CCA UGU G(dTdT)
Mouse *Fxyd5*	siRNA #3	Sense: CUG GAU UCG AAU GAG AAC A(dTdT)
		Antisense: UGU UCU CAU UCG AAU CCA G(dTdT)
Human *FN1*	siRNA#1	Sense: CUCCAUGAUCUGGGACUG(dTdT)
		Antisense: ACAGUCCCAGAUCAUGGA(dTdT)
Human *FN1*	siRNA#2	Sense: CAGACUUACGGUGGCAACU(dTdT)
		Antisense: AGUUGCCACCGUAAGUCU(dTdT)

Table S3. List of antibodies used for immunoblot analysis, co-IP, IHC, and IF staining

Target	Conjugate	Catalog # (company/provider)	Application
Mouse Ki-67	-	MA5-14520	IHC
Mouse cleaved-Caspase 3	-	9661S (Cell Signaling Technology)	IHC
Human Dysadherin	-	M53 (Dr. Ino)	IHC
VECTASTAIN® ABC Kit, Rabbit IgG	-	PK-6101 (Vector Laboratories)	IHC
VECTASTAIN® ABC Kit, Mouse IgG	-	PK-6102 (Vector Laboratories)	IHC
Human Dysadherin	-	M53 (Dr. Ino)	Immunoblot
Human E-cadherin	-	14472S (Cell Signaling Technology)	Immunoblot
Human β-actin	-	A5316 (Sigma-Aldrich)	Immunoblot
Human Fibronectin	-	ab2413 (Abcam)	Immunoblot
Human GAPDH	-	5174S (Cell Signaling Technology)	Immunoblot
Human p-FAK(Y397)	-	700255 (ThermoFisher)	Immunoblot
Human total FAK	-	3285S (Cell Signaling Technology)	Immunoblot
Human Pan-cadherin	-	4068S (Cell Signaling Technology)	Immunoblot
Human YAP	-	ab205270 (Abcam)	Immunoblot
Human p-YAP (S217)	-	ab76252 (Abcam)	Immunoblot
His-Tag	-	MA1-21315 (ThermoFisher)	Immunoblot

Goat anti-mouse,<br>Light chain-specific	HRP	91196S (Cell Signaling Technology)	Immunoblot
Goat anti-mouse	HRP	554002 (BD Pharmigen™)	Immunoblot
Goat anti-rabbit	HRP	554021 (BD Pharmigen™)	Immunoblot
Annexin V	FITC	556547 (BD Pharmigen™)	FACS
Human Dysadherin	-	M53 (Dr. Ino)	IF
Human Dysadherin	APC	M53 (Dr. Ino)	IF
Human active integrin β1 (12G10)	-	ab202641 (Abcam)	IF
Human Fibronectin	-	ab2413 (Abcam)	IF
Human EpCAM	-	93790S (Cell Signaling Technology)	IF
Human EpCAM	Alexa488	14-9326-82 (eBioscience™)	IF
Human F-actin	Alexa555	A34055 (ThermoFisher)	IF
Human p-FAK(Y397)	-	700255 (ThermoFisher)	IF
Human Paxillin	-	AHO0492(ThermoFisher)	IF
Human YAP	-	14074 (Cell Signaling Technology)	IF
Mouse active integrin β1(9EG7)	-	550531 (BD Pharmigen™)	IF
Mouse Dysadherin	-	SC-30606 (SantaCruz)	IF
Mouse Fibronectin	-	ab2413 (Abcam)	IF
Mouse a-SMA	-	A2547 (Sigma-Aldrich)	IF
Mouse EpCAM	-	93790S (Cell Signaling Technology)	IF
Mouse p-FAK(Y397)	-	700255 (ThermoFisher)	IF

Human Myosin IIa	-	3403 (Cell Signaling Technology)	Immunoblot & IF
Human Phosphor-Myosin IIa (Ser1943)	-	14611 (Cell Signaling Technology)	Immunoblot & IF
His-tag	-	MA1-21315 (ThermoFisher)	IF
Donkey anti-mouse	Alexa488	A21202 (ThermoFisher)	IF
Donkey anti-rabbit	Alexa555	A31572 (ThermoFisher)	IF
Donkey anti-goat	Alexa488	A11055 (ThermoFisher)	IF
Goat anti-mouse	Alexa555	A21422 (ThermoFisher)	IF
Donkey anti-rat	Alexa555	A48270 (ThermoFisher)	IF
Donkey anti-rabbit	Alexa488	A21206 (ThermoFisher)	IF
Phalloidin	Alexa555	A34055 (ThermoFisher)	IF

IHC, immunohistochemistry; IF, immunofluorescence; FACS, fluorescence-activated cell sorting; HRP, horseradish peroxidase; FITC, fluorescein isothiocyanate; APC, adenomatous polyposis coli

Table S4. Association of clinicopathologic characteristics and dysadherin expression in patients with resected colon cancer (n = 123)

	Dysadherin expression		
Variable	Low (< 75%, n = 96)	High (≥ 75%, n = 27)	*p*-value
Age (mean ± SD)	64.7±9.92	67.3±9.03	0.362
Sex			
Male	57 (59.4%)	14 (51.9%)	0.484
Female	39 (40.6%)	13 (48.1%)	
T stage			
T2-3	86 (89.6%)	19 (70.4%)	**0.013**
T4	10 (10.4%)	8 (29.6%)	
N stage			
N0	52 (54.2%)	11 (40.7%)	0.435
N1	27 (28.1%)	9 (33.3%)	
N2	17 (17.7%)	7 (25.9%)	
TNM stage			
II	52 (54.2%)	11 (40.7%)	0.218
III	44 (45.8%)	16 (59.3%)	
Tumor differentiation			
Well	33 (34.4%)	8 (29.6%)	0.770
Moderately	56 (58.3%)	16 (59.3%)	
Poorly	7 (7.3%)	3 (11.1%)	
LVI			
Positive	19 (19.8%)	9 (33.3%)	0.138
Negative	77 (80.2%)	18 (66.7%)	
PNI			
Positive	43 (44.8%)	17 (63.0%)	0.095
Negative	53 (55.2%)	10 (37.0%)	
Recurrence			
Yes	12 (12.5%)	8 (29.6%)	**0.033**
No	84 (87.5%)	19 (70.4%)	

Multivariate Cox analysis (low dysadherin group vs. high dysadherin group)
OS (II + III): HR 3.863 (95% CI, 1.698-8.792), $p = 0.002$
RFS (II + III): HR 2.569 (95% CI, 1.046-6.308), $p = 0.040$

Supplementary Table 5. List of DEGs in dysadherin-high versus dysadherin-low tumors from patients with CRC.

ID	P.Value	logFC	Gene.symbol	Gene.title
224252_s_at	3.72E-23	1.31246158	FXYD5	FXYD domain containing ion transport regulator 5
218084_x_at	4.95E-23	1.32376803	FXYD5	FXYD domain containing ion transport regulator 5
217655_at	1.58E-10	0.40578691	LOC100127972	uncharacterized LOC100127972
208190_s_at	2.88E-09	0.62960605	LSR	lipolysis stimulated lipoprotein receptor
219513_s_at	1.14E-08	0.5287249	SH2D3A	SH2 domain containing 3A
208916_at	3.60E-08	0.46217828	SLC1A5	solute carrier family 1 member 5
214062_x_at	3.66E-08	0.33091234	NFKBIB	NFKB inhibitor beta
205460_at	1.33E-07	0.34382327	NPAS2	neuronal PAS domain protein 2
200601_at	2.33E-07	0.42276669	ACTN4	actinin alpha 4
214448_x_at	2.97E-07	0.28760986	NFKBIB	NFKB inhibitor beta
223003_at	5.49E-07	0.48900836	C19orf43	chromosome 19 open reading frame 43
208611_s_at	7.76E-07	0.49771028	SPTAN1	spectrin alpha, non-erythrocytic 1
202415_s_at	8.81E-07	0.42708143	HSPBP1	HSPA (Hsp70) binding protein 1
202417_at	9.82E-07	0.4142642	KEAP1	kelch like ECH associated protein 1
210715_s_at	1.14E-06	0.39030856	SPINT2	serine peptidase inhibitor, Kunitz type 2
232079_s_at	1.34E-06	0.52574233	NECTIN2	nectin cell adhesion molecule 2
208336_s_at	1.59E-06	0.53677077	TECR	trans-2,3-enoyl-CoA reductase
200660_at	1.92E-06	0.62914447	S100A11	S100 calcium binding protein A11
200001_at	2.04E-06	0.57432037	CAPNS1	calpain small subunit 1
58994_at	2.34E-06	0.55897357	CC2D1A	coiled-coil and C2 domain containing 1A
206284_x_at	2.45E-06	0.67511116	CLTB	clathrin light chain B
211136_s_at	2.82E-06	0.37397428	CLPTM1	CLPTM1, transmembrane protein
35148_at	3.44E-06	0.45225734	TJP3	tight junction protein 3
208540_x_at	3.67E-06	0.5424753		
200695_at	3.86E-06	0.53362027	PPP2R1A	protein phosphatase 2 scaffold subunit Aalpha
203718_at	4.05E-06	0.42024948	PNPLA6	patatin like phospholipase domain containing 6
218855_at	4.19E-06	0.35900389	TPRA1	transmembrane protein adipocyte associated 1
50314_i_at	4.26E-06	0.34690105	C20orf27	chromosome 20 open reading frame 27
201264_at	4.30E-06	0.50313996	COPE	coatomer protein complex subunit epsilon
200649_at	4.41E-06	0.39029679	NUCB1	nucleobindin 1
221853_s_at	4.53E-06	0.43998312	LOC101060373///NOMO3///NOMO2///NOMO1	uncharacterized LOC101060373///NODAL modulator 3///NODAL modulator 2///NODAL modulator 1
213669_at	4.65E-06	0.24022197	FCHO1	FCH domain only 1
205459_s_at	4.73E-06	0.28367794	NPAS2	neuronal PAS domain protein 2
223385_at	4.84E-06	0.72531897	CYP2S1	cytochrome P450 family 2 subfamily S member 1
200707_at	4.86E-06	0.51964587	PRKCSH	protein kinase C substrate 80K-H
220956_s_at	4.86E-06	0.27224496	RAB4B-EGLN2///EGLN2	RAB4B-EGLN2 readthrough (NMD candidate)///egl-9 family hypoxia inducible factor 2
205740_s_at	5.04E-06	0.39404961	RBM42	RNA binding motif protein 42
229862_x_at	5.09E-06	0.25416373	ZBTB45	zinc finger and BTB domain containing 45
219476_at	6.28E-06	0.70892605	C1orf116	chromosome 1 open reading frame 116
207525_s_at	6.81E-06	0.2817994	GIPC1	GIPC PDZ domain containing family member 1
200666_s_at	7.20E-06	0.64499322	DNAJB1	DnaJ heat shock protein family (Hsp40) member B1
203287_at	7.39E-06	0.397909	LAD1	ladinin 1
224375_at	7.69E-06	-0.31996403	LOC102724870	uncharacterized LOC102724870
208751_at	8.03E-06	0.48747814	NAPA	NSF attachment protein alpha
202734_at	8.32E-06	0.34838452	TRIP10	thyroid hormone receptor interactor 10
203904_x_at	8.48E-06	0.21764731	CD82	CD82 molecule
202045_s_at	9.24E-06	0.211491	ARHGAP35	Rho GTPase activating protein 35
205172_x_at	9.61E-06	0.59477119	CLTB	clathrin light chain B
200990_at	9.63E-06	0.51795887	TRIM28	tripartite motif containing 28
200752_s_at	9.85E-06	0.43931194	CAPN1	calpain 1
227951_s_at	1.14E-05	0.39572568	FAM98C	family with sequence similarity 98 member C
234929_s_at	1.19E-05	-0.3129059	SPATA7	spermatogenesis associated 7
201039_s_at	1.21E-05	0.46263154	RAD23A	RAD23 homolog A, nucleotide excision repair protein
91826_at	1.27E-05	0.4390381	EPS8L1	EPS8 like 1
225434_at	1.32E-05	0.41478705	DEDD2	death effector domain containing 2
205019_s_at	1.39E-05	0.48748988	VIPR1	vasoactive intestinal peptide receptor 1
1562776_at	1.43E-05	0.13952328	LOC339807	uncharacterized LOC339807
202253_s_at	1.53E-05	0.42108237	DNM2	dynamin 2

206491_s_at	1.62E-05	0.37240224	NAPA	NSF attachment protein alpha
202120_x_at	1.67E-05	0.40987485	AP2S1	adaptor related protein complex 2 sigma 1 subunit
217225_x_at	1.70E-05	0.34369215	LOC101060373///NOMO3///NOMO2///NOMO1	uncharacterized LOC101060373///NODAL modulator 3///NODAL modulator 2///NODAL modulator 1
221665_s_at	1.75E-05	0.35321015	EPS8L1	EPS8 like 1
202525_at	1.81E-05	0.59728954	PRSS8	protease, serine 8
232977_x_at	1.92E-05	0.36851152	MYH14	myosin, heavy chain 14, non-muscle
207713_s_at	1.95E-05	0.47259995	RBCK1	RANBP2-type and C3HC4-type zinc finger containing 1
1555735_a_at	1.97E-05	0.25785681	BAP1	BRCA1 associated protein 1
210926_at	2.06E-05	0.3904306	POTEKP	POTE ankyrin domain family member K, pseudogene
200646_s_at	2.11E-05	0.27385917	NUCB1	nucleobindin 1
202910_s_at	2.15E-05	0.40275203	ADGRE5	adhesion G protein-coupled receptor E5
225156_at	2.20E-05	0.34602036	ELOF1	elongation factor 1 homolog
227003_at	2.24E-05	-0.3176165	RAB28	RAB28, member RAS oncogene family
224609_at	2.25E-05	0.40789216	SLC44A2	solute carrier family 44 member 2
226917_s_at	2.26E-05	-0.29712739	ANAPC4	anaphase promoting complex subunit 4
218913_s_at	2.28E-05	0.27787144	GMIP	GEM interacting protein
208110_x_at	2.28E-05	0.18028087	MED25	mediator complex subunit 25
219583_s_at	2.32E-05	-0.49268741	SPATA7	spermatogenesis associated 7
213412_at	2.47E-05	0.34763802	TJP3	tight junction protein 3
219075_at	2.51E-05	0.26043909	YIPF2	Yip1 domain family member 2
201841_s_at	2.68E-05	0.94000023	HSPB1	heat shock protein family B (small) member 1
201046_s_at	2.71E-05	0.28590103	RAD23A	RAD23 homolog A, nucleotide excision repair protein
201480_s_at	2.71E-05	0.31257456	SUPT5H	SPT5 homolog, DSIF elongation factor subunit
202067_s_at	2.82E-05	0.44730983	LDLR	low density lipoprotein receptor
208074_s_at	2.86E-05	0.46767529	AP2S1	adaptor related protein complex 2 sigma 1 subunit
201050_at	2.90E-05	0.40866806	PLD3	phospholipase D family member 3
211237_s_at	3.03E-05	0.33337072	FGFR4	fibroblast growth factor receptor 4
AFFX-HSAC07/X00	3.06E-05	0.38071936	ACTB	actin beta
215235_at	3.17E-05	0.42977788	SPTAN1	spectrin alpha, non-erythrocytic 1
221418_s_at	3.20E-05	0.35308099	MED16	mediator complex subunit 16
209953_s_at	3.22E-05	0.40607645	CDC37	cell division cycle 37
1555730_a_at	3.28E-05	0.53372781	CFL1	cofilin 1
224434_s_at	3.32E-05	0.26195777	WDR83	WD repeat domain 83
217354_s_at	3.41E-05	0.1878345	HPS1	HPS1, biogenesis of lysosomal organelles complex 3 subunit 1
211047_x_at	3.54E-05	0.41412917	AP2S1	adaptor related protein complex 2 sigma 1 subunit
220074_at	3.54E-05	0.42735806	CDHR5	cadherin related family member 5
234290_x_at	3.55E-05	0.36809841	MYH14	myosin, heavy chain 14, non-muscle
91952_at	3.56E-05	0.28705639	DCAF15	DDB1 and CUL4 associated factor 15
232053_x_at	3.65E-05	0.34504521	RHBDD2	rhomboid domain containing 2
224749_at	3.67E-05	0.44261527	FAM234A	family with sequence similarity 234 member A
229920_at	3.69E-05	0.28691516	RBCK1	RANBP2-type and C3HC4-type zinc finger containing 1
1556336_at	3.82E-05	-0.36333186	LOC101928747///RBMX///SNORD61	uncharacterized LOC101928747///RNA binding motif protein, X-linked///small nucleolar RNA, C/D box 61
221764_at	3.87E-05	0.47291531	R3HDM4	R3H domain containing 4
208757_at	3.95E-05	0.31698364	TMED9	transmembrane p24 trafficking protein 9
202356_s_at	3.97E-05	0.36458374	GTF2F1	general transcription factor IIF subunit 1
225869_s_at	4.21E-05	0.3136463	UNC93B1	unc-93 homolog B1 (C. elegans)
209872_s_at	4.23E-05	0.24213973	PKP3	plakophilin 3
225020_at	4.33E-05	0.30842725	DAB2IP	DAB2 interacting protein
204401_at	4.39E-05	0.8253872	KCNN4	potassium calcium-activated channel subfamily N member 4
225252_at	4.47E-05	0.49223041	SRXN1	sulfiredoxin 1
221091_at	4.49E-05	-0.16854054	INSL5	insulin like 5
217930_s_at	4.52E-05	0.26576552	TOLLIP	toll interacting protein
221849_s_at	4.54E-05	0.32417786	DCAF15	DDB1 and CUL4 associated factor 15
200664_s_at	4.61E-05	0.61381822	DNAJB1	DnaJ heat shock protein family (Hsp40) member B1
221711_s_at	4.62E-05	0.31801806	BABAM1	BRISC and BRCA1 A complex member 1
217903_at	4.64E-05	0.26835947	STRN4	striatin 4
202201_at	4.70E-05	0.50599452	BLVRB	biliverdin reductase B
241830_at	4.72E-05	-0.13912245	NOL4L	nucleolar protein 4 like
202117_at	4.77E-05	0.34094513	ARHGAP1	Rho GTPase activating protein 1

36994_at	4.77E-05	0.3656266	ATP6V0C	ATPase H+ transporting V0 subunit c
203109_at	4.91E-05	0.50362208	UBE2M	ubiquitin conjugating enzyme E2 M
218317_x_at	4.94E-05	0.41818427	SLX1A///SLX1B	SLX1 homolog A, structure-specific endonuclease subunit///SLX1 homolog B, structure-specific endonuclease subunit
202545_at	5.08E-05	0.42957142	PRKCD	protein kinase C delta
202942_at	5.26E-05	0.42985929	ETFB	electron transfer flavoprotein beta subunit
214687_x_at	5.32E-05	0.45703831	ALDOA	aldolase, fructose-bisphosphate A
232018_at	5.41E-05	0.17957484	LENG1	leukocyte receptor cluster member 1
200801_x_at	5.43E-05	0.31877167	ACTB	actin beta
1555788_a_at	5.46E-05	0.32310533	TRIB3	tribbles pseudokinase 3
239144_at	5.49E-05	-0.33670037	B3GAT2	beta-1,3-glucuronyltransferase 2
40359_at	5.52E-05	0.22040893	RASSF7	Ras association domain family member 7
200922_at	6.03E-05	0.41127263	KDELR1	KDEL endoplasmic reticulum protein retention receptor 1
226516_at	6.03E-05	0.21632988	MFSD12	major facilitator superfamily domain containing 12
219922_s_at	6.05E-05	0.61251652	LTBP3	latent transforming growth factor beta binding protein 3
228261_at	6.19E-05	0.56305528	MIB2	mindbomb E3 ubiquitin protein ligase 2
224594_x_at	6.31E-05	0.26973106	ACTB	actin beta
AFFX-HSAC07/X00	6.33E-05	0.50914187	ACTB	actin beta
205546_s_at	6.42E-05	0.27798488	TYK2	tyrosine kinase 2
212647_at	6.44E-05	0.59506164	RRAS	related RAS viral (r-ras) oncogene homolog
34478_at	6.45E-05	0.10979398	RAB11B	RAB11B, member RAS oncogene family
214080_x_at	6.51E-05	0.24052662	PRKCSH	protein kinase C substrate 80K-H
217831_s_at	6.56E-05	0.48238481	NSFL1C	NSFL1 cofactor
241514_at	6.63E-05	0.13404963		
1555575_a_at	6.64E-05	0.32285131	KDELR1	KDEL endoplasmic reticulum protein retention receptor 1
220947_s_at	6.70E-05	0.31709087	TBC1D10B	TBC1 domain family member 10B
220248_x_at	6.78E-05	0.51971124	NSFL1C	NSFL1 cofactor
223425_at	6.81E-05	0.24874434	RAVER1	ribonucleoprotein, PTB binding 1
231193_s_at	6.91E-05	-0.19861153	TAOK1	TAO kinase 1
91703_at	6.98E-05	0.29821572	EHBP1L1	EH domain binding protein 1 like 1
215735_s_at	7.15E-05	0.43639358	TSC2	tuberous sclerosis 2
200820_at	7.17E-05	0.41725244	PSMD8	proteasome 26S subunit, non-ATPase 8
232244_at	7.23E-05	0.36304132	KIAA1161	KIAA1161
211043_s_at	7.43E-05	0.48300269	CLTB	clathrin light chain B
1566342_at	7.48E-05	-0.40363024	SOD2	superoxide dismutase 2, mitochondrial
226988_s_at	7.51E-05	0.33639272	MYH14	myosin, heavy chain 14, non-muscle
219088_s_at	7.79E-05	0.26664743	ZNF576	zinc finger protein 576
223637_s_at	7.83E-05	0.28749467	FAM160A2	family with sequence similarity 160 member A2
221888_at	7.86E-05	0.27238148	CC2D1A	coiled-coil and C2 domain containing 1A
179_at	8.06E-05	0.15502422	DTX2P1-UPK3BP1-PMS2P11	DTX2P1-UPK3BP1-PMS2P11 readthrough, transcribed pseudogene
208308_s_at	8.14E-05	0.52703568	GPI	glucose-6-phosphate isomerase
203252_at	8.20E-05	0.39227039	CDK2AP2	cyclin dependent kinase 2 associated protein 2
215737_x_at	8.35E-05	0.20140898	USF2	upstream transcription factor 2, c-fos interacting
210624_s_at	8.51E-05	0.37176477	ILVBL	ilvB acetolactate synthase like
202993_at	8.53E-05	0.45137506	ILVBL	ilvB acetolactate synthase like
213867_x_at	8.55E-05	0.26631628	ACTB	actin beta
218688_at	8.58E-05	0.37229887	TKFC	triokinase and FMN cyclase
238778_at	8.62E-05	-0.84938852	MPP7	membrane palmitoylated protein 7
203452_at	8.66E-05	0.2903231	B3GAT3	beta-1,3-glucuronyltransferase 3
218996_at	8.71E-05	0.40286867	TFPT	TCF3 fusion partner
201850_at	8.87E-05	0.58862421	CAPG	capping actin protein, gelsolin like
1561503_at	8.92E-05	0.13750757	MYLK4	myosin light chain kinase family member 4
200966_x_at	9.00E-05	0.45518315	ALDOA	aldolase, fructose-bisphosphate A
1555821_a_at	9.13E-05	0.17924258	AKT1S1	AKT1 substrate 1
200021_at	9.19E-05	0.23963059	CFL1	cofilin 1
219622_at	9.42E-05	0.36876409	RAB20	RAB20, member RAS oncogene family
217784_at	9.44E-05	0.31932147	YKT6	YKT6 v-SNARE homolog (S. cerevisiae)
200613_at	9.48E-05	0.36971223	AP2M1	adaptor related protein complex 2 mu 1 subunit

219354_at	9.61E-05	0.27602728	KLHL26	kelch like family member 26
235009_at	9.67E-05	-0.46288442	BOD1L1	biorientation of chromosomes in cell division 1 like 1
235517_at	9.69E-05	-0.19014097	PACRGL	PARK2 coregulated like
225175_s_at	9.72E-05	0.38244668	SLC44A2	solute carrier family 44 member 2
1552664_at	9.78E-05	-0.39496524	FLCN	folliculin
221293_s_at	9.96E-05	0.20297195	DEF6	DEF6, guanine nucleotide exchange factor
AFFX-HSAC07/X00	9.97E-05	0.48049636	ACTB	actin beta
213211_s_at	1.01E-04	0.22828263	TAF6L	TATA-box binding protein associated factor 6 like
210398_x_at	1.02E-04	0.41766245	FUT6	fucosyltransferase 6
217173_s_at	1.02E-04	0.23279553	LDLR	low density lipoprotein receptor
1564166_s_at	1.02E-04	0.2962804	LOC100630923	LOC100289561-PRKRIP1 readthrough
211885_x_at	1.04E-04	0.37426159	FUT6	fucosyltransferase 6
232524_x_at	1.05E-04	-0.28130938	ANAPC4	anaphase promoting complex subunit 4
220587_s_at	1.05E-04	0.3620182	MLST8	MTOR associated protein, LST8 homolog
202161_at	1.05E-04	0.32266649	PKN1	protein kinase N1
226437_at	1.05E-04	0.47478354	YIF1B	Yip1 interacting factor homolog B, membrane trafficking protein
218474_s_at	1.07E-04	0.37485421	KCTD5	potassium channel tetramerization domain containing 5
202328_s_at	1.07E-04	0.20937121	LOC101930075///PKD1	polycystin-1-like///polycystin 1, transient receptor potential channel interacting
204856_at	1.08E-04	0.34082084	B3GNT3	UDP-GlcNAc:betaGal beta-1,3-N-acetylglucosaminyltransferase 3
202711_at	1.08E-04	0.23116677	EFNB1	ephrin B1
213677_s_at	1.08E-04	-0.31795795	PMS1	PMS1 homolog 1, mismatch repair system component
1563565_at	1.09E-04	0.11028193	INPP5B	inositol polyphosphate-5-phosphatase B
200065_s_at	1.09E-04	0.3955563	MIR3620///ARF1	microRNA 3620///ADP ribosylation factor 1
203175_at	1.09E-04	0.27764706	RHOG	ras homolog family member G
214992_s_at	1.14E-04	0.17306683	DNASE2	deoxyribonuclease 2, lysosomal
217716_s_at	1.15E-04	0.29533248	SEC61A1	Sec61 translocon alpha 1 subunit
209795_at	1.16E-04	-0.90621472	CD69	CD69 molecule
224792_at	1.16E-04	0.20853081	TNKS1BP1	tankyrase 1 binding protein 1
232520_s_at	1.18E-04	0.49577466	NSFL1C	NSFL1 cofactor
201204_s_at	1.18E-04	0.62233247	RRBP1	ribosome binding protein 1
65517_at	1.19E-04	0.38948311	AP1M2	adaptor related protein complex 1 mu 2 subunit
51200_at	1.19E-04	0.38491088	C19orf60	chromosome 19 open reading frame 60
200954_at	1.20E-04	0.30776158	ATP6V0C	ATPase H+ transporting V0 subunit c
229883_at	1.21E-04	0.35695264	GRIN2D	glutamate ionotropic receptor NMDA type subunit 2D
202424_at	1.21E-04	0.39773926	MAP2K2	mitogen-activated protein kinase kinase 2
203552_at	1.21E-04	-0.32977118	MAP4K5	mitogen-activated protein kinase kinase kinase kinase 5
218215_s_at	1.21E-04	0.33035499	NR1H2	nuclear receptor subfamily 1 group H member 2
210407_at	1.21E-04	-0.39689845	PPM1A	protein phosphatase, Mg2+/Mn2+ dependent 1A
209950_s_at	1.21E-04	0.55911367	VILL	villin like
221755_at	1.22E-04	0.30613309	EHBP1L1	EH domain binding protein 1 like 1
202445_s_at	1.23E-04	-0.15026742	NOTCH2	notch 2
212662_at	1.24E-04	0.39426571	PVR	poliovirus receptor
55705_at	1.24E-04	0.29185352	R3HDM4	R3H domain containing 4
225454_at	1.25E-04	0.3922383	CCDC124	coiled-coil domain containing 124
204757_s_at	1.27E-04	0.18906674	C2CD2L	C2CD2 like
222090_at	1.27E-04	0.16240653	NDUFB2-AS1	NDUFB2 antisense RNA 1
202631_s_at	1.28E-04	-0.32074027	APPBP2	amyloid beta precursor protein binding protein 2
224982_at	1.29E-04	0.27252046	AKT1S1	AKT1 substrate 1
209808_x_at	1.29E-04	0.28826087	ING1	inhibitor of growth family member 1
203143_s_at	1.30E-04	0.45478279	KIAA0040	KIAA0040
225650_at	1.32E-04	0.24040364	SAMD1	sterile alpha motif domain containing 1
201360_at	1.33E-04	0.68507382	CST3	cystatin C
222647_at	1.33E-04	0.25646455	SLC35C1	solute carrier family 35 member C1
206050_s_at	1.38E-04	0.37593085	RNH1	ribonuclease/angiogenin inhibitor 1
209282_at	1.39E-04	0.37339027	PRKD2	protein kinase D2
241608_at	1.39E-04	-0.15555812		
32837_at	1.40E-04	0.40409513	AGPAT2	1-acylglycerol-3-phosphate O-acyltransferase 2
216641_s_at	1.42E-04	0.28874303	LAD1	ladinin 1
236580_at	1.42E-04	-0.20609969		

225995_x_at	1.44E-04	0.2667323	WASH1///WASH2P	WAS protein family homolog 1///WAS protein family homolog 2 pseudogene
211924_s_at	1.45E-04	0.51626735	PLAUR	plasminogen activator, urokinase receptor
233337_s_at	1.45E-04	0.57518354	SEZ6L2	seizure related 6 homolog like 2
218779_x_at	1.47E-04	0.31412754	EPS8L1	EPS8 like 1
205590_at	1.48E-04	-0.73582578	RASGRP1	RAS guanyl releasing protein 1
218261_at	1.49E-04	0.38297574	AP1M2	adaptor related protein complex 1 mu 2 subunit
231370_at	1.50E-04	-0.49091202	PPM1A	protein phosphatase, Mg2+/Mn2+ dependent 1A
220638_s_at	1.51E-04	0.51837375	CBLC	Cbl proto-oncogene C
203220_s_at	1.51E-04	0.13889239	TLE1	transducin like enhancer of split 1
226927_at	1.52E-04	-0.33044657	C12orf73	chromosome 12 open reading frame 73
213499_at	1.53E-04	0.31586156	CLCN2	chloride voltage-gated channel 2
226839_at	1.53E-04	0.27780399	NR2C2AP	nuclear receptor 2C2 associated protein
213521_at	1.53E-04	0.32473136	PTPN18	protein tyrosine phosphatase, non-receptor type 18
227864_s_at	1.57E-04	0.50817672	MVB12A	multivesicular body subunit 12A
213402_at	1.57E-04	0.24705706	ZNF787	zinc finger protein 787
202621_at	1.58E-04	0.32294965	IRF3	interferon regulatory factor 3
223133_at	1.58E-04	-0.29295957	TMEM14B	transmembrane protein 14B
213175_s_at	1.59E-04	0.61508632	SNRPB	small nuclear ribonucleoprotein polypeptides B and B1
205462_s_at	1.60E-04	0.32695897	HPCAL1	hippocalcin like 1
202068_s_at	1.60E-04	0.51378354	LDLR	low density lipoprotein receptor
211975_at	1.67E-04	0.35925171	ARFGAP2	ADP ribosylation factor GTPase activating protein 2
205443_at	1.68E-04	-0.37601455	SNAPC1	small nuclear RNA activating complex polypeptide 1
211902_x_at	1.68E-04	-0.16720506	YME1L1	YME1 like 1 ATPase
211759_x_at	1.69E-04	0.44772539	TBCB	tubulin folding cofactor B
218105_s_at	1.73E-04	0.35963206	MRPL4	mitochondrial ribosomal protein L4
224695_at	1.75E-04	0.24954013	CNOT11	CCR4-NOT transcription complex subunit 11
216602_s_at	1.75E-04	0.49844513	FARSA	phenylalanyl-tRNA synthetase alpha subunit
236890_at	1.75E-04	-0.14912141	MTG2	mitochondrial ribosome associated GTPase 2
204096_s_at	1.76E-04	0.14429508	ELL	elongation factor for RNA polymerase II
212695_at	1.79E-04	0.22518612	CRY2	cryptochrome circadian clock 2
39548_at	1.82E-04	0.28307828	NPAS2	neuronal PAS domain protein 2
201221_s_at	1.82E-04	0.33458885	SNRNP70	small nuclear ribonucleoprotein U1 subunit 70
224308_s_at	1.85E-04	-0.43360537	INTS2	integrator complex subunit 2
222865_x_at	1.85E-04	0.12556332	TMEM204	transmembrane protein 204
221256_s_at	1.86E-04	0.43431856	HDHD3	haloacid dehalogenase like hydrolase domain containing 3
1555321_at	1.90E-04	0.15451801	ACOT11	acyl-CoA thioesterase 11
210054_at	1.90E-04	-0.33432101	HAUS3	HAUS augmin like complex subunit 3
1554152_a_at	1.90E-04	0.17244378	OGDH	oxoglutarate dehydrogenase
226130_at	1.90E-04	0.28670143	RPS16	ribosomal protein S16
219150_s_at	1.91E-04	0.38835055	ADAP1	ArfGAP with dual PH domains 1
205748_s_at	1.91E-04	0.37404962	RNF126	ring finger protein 126
202475_at	1.91E-04	0.37134451	TMEM147	transmembrane protein 147
632_at	1.92E-04	0.18079743	GSK3A	glycogen synthase kinase 3 alpha
204331_s_at	1.92E-04	0.59276757	MRPS12	mitochondrial ribosomal protein S12
219360_s_at	1.93E-04	0.57793651	TRPM4	transient receptor potential cation channel subfamily M member 4
224352_s_at	1.94E-04	-0.48768077	CFL2	cofilin 2
223469_at	1.94E-04	0.34797639	PGPEP1	pyroglutamyl-peptidase I
233851_s_at	1.95E-04	0.20500104	TOR3A	torsin family 3 member A
223743_s_at	1.96E-04	0.51079546	MRPL4	mitochondrial ribosomal protein L4
210344_at	1.97E-04	0.14589781	OSBPL7	oxysterol binding protein like 7
208947_s_at	2.03E-04	0.25183549	UPF1	UPF1, RNA helicase and ATPase
234478_at	2.06E-04	-0.10839618		
226689_at	2.09E-04	-0.33644607	CISD2	CDGSH iron sulfur domain 2
227346_at	2.09E-04	-0.67493273	IKZF1	IKAROS family zinc finger 1
220848_x_at	2.10E-04	0.13997338	OBP2A	odorant binding protein 2A
216464_x_at	2.11E-04	0.10318428	PTGDR2	prostaglandin D2 receptor 2
212625_at	2.15E-04	0.27339742	STX10	syntaxin 10
235332_at	2.18E-04	0.14716216	NUTM2B///NUTM2A	NUT family member 2B///NUT family member 2A
213076_at	2.19E-04	0.27294134	ITPKC	inositol-trisphosphate 3-kinase C
218064_s_at	2.20E-04	0.43457487	AKAP8L	A-kinase anchoring protein 8 like

226083_at	2.20E-04	-0.30990517	TMEM70	transmembrane protein 70
209873_s_at	2.21E-04	0.28667839	PKP3	plakophilin 3
210350_x_at	2.22E-04	0.2457572	ING1	inhibitor of growth family member 1
222645_s_at	2.24E-04	0.19964267	KCTD5	potassium channel tetramerization domain containing 5
227471_at	2.26E-04	-0.50949527	HACE1	HECT domain and ankyrin repeat containing E3 ubiquitin protein ligase 1
213526_s_at	2.30E-04	0.34157394	LIN37	lin-37 DREAM MuvB core complex component
225467_s_at	2.35E-04	0.28850029	RDH13	retinol dehydrogenase 13
222203_s_at	2.37E-04	-0.23635822	NT5C1B-RDH14///RDH14	NT5C1B-RDH14 readthrough///retinol dehydrogenase 14 (all-trans/9-cis/11-cis)
215807_s_at	2.39E-04	0.29086714	PLXNB1	plexin B1
243310_at	2.39E-04	-0.34704976		
204989_s_at	2.40E-04	0.23331629	ITGB4	integrin subunit beta 4
243425_at	2.40E-04	0.14644069	LOC101928557	uncharacterized LOC101928557
221270_s_at	2.43E-04	0.30906652	QTRT1	queuine tRNA-ribosyltransferase catalytic subunit 1
224927_at	2.46E-04	0.30739268	PPP1R18	protein phosphatase 1 regulatory subunit 18
1554553_s_at	2.47E-04	0.3504245	YIF1B	Yip1 interacting factor homolog B, membrane trafficking protein
202135_s_at	2.48E-04	0.28791012	ACTR1B	ARP1 actin-related protein 1 homolog B, centractin beta
221664_s_at	2.48E-04	0.34614307	F11R	F11 receptor
224673_at	2.52E-04	0.30475431	LENG8	leukocyte receptor cluster member 8
203942_s_at	2.54E-04	0.27816399	MARK2	microtubule affinity regulating kinase 2
219243_at	2.56E-04	-0.4225894	GIMAP4	GTPase, IMAP family member 4
202185_at	2.57E-04	0.37313914	PLOD3	procollagen-lysine,2-oxoglutarate 5-dioxygenase 3
207225_at	2.59E-04	0.11249026	AANAT	aralkylamine N-acetyltransferase
238996_x_at	2.59E-04	0.52843425	ALDOA	aldolase, fructose-bisphosphate A
200740_s_at	2.60E-04	0.26041181	SUMO3	small ubiquitin-like modifier 3
221422_s_at	2.62E-04	-0.11778226	MIR600///MIR600HG	microRNA 600///MIR600 host gene
214101_s_at	2.68E-04	-0.46951051	NPEPPS	aminopeptidase puromycin sensitive
203555_at	2.68E-04	0.22387989	PTPN18	protein tyrosine phosphatase, non-receptor type 18
1556058_s_at	2.70E-04	0.12632592	SPEN	spen family transcriptional repressor
209179_s_at	2.71E-04	0.28211635	MBOAT7	membrane bound O-acyltransferase domain containing 7
219946_x_at	2.71E-04	0.31383448	MYH14	myosin, heavy chain 14, non-muscle
216488_s_at	2.72E-04	0.21939602	ATP11A	ATPase phospholipid transporting 11A
224674_at	2.72E-04	0.27098725	TTYH3	tweety family member 3
212193_s_at	2.73E-04	0.43011381	LARP1	La ribonucleoprotein domain family member 1
203419_at	2.78E-04	0.29931876	KMT2B	lysine methyltransferase 2B
215399_s_at	2.82E-04	0.31740714	OS9	OS9, endoplasmic reticulum lectin
227540_at	2.83E-04	0.17349128	EEFSEC	eukaryotic elongation factor, selenocysteine-tRNA specific
218186_at	2.83E-04	0.43737555	RAB25	RAB25, member RAS oncogene family
218958_at	2.85E-04	0.29662531	C19orf60	chromosome 19 open reading frame 60
223473_at	2.85E-04	0.26492245	MPV17L2	MPV17 mitochondrial inner membrane protein like 2
232032_x_at	2.87E-04	0.33437062	SDF4	stromal cell derived factor 4
212046_x_at	2.90E-04	0.26171091	MAPK3	mitogen-activated protein kinase 3
240486_at	2.92E-04	-0.3580404	HELZ	helicase with zinc finger
221603_at	2.92E-04	0.17696821	PEX16	peroxisomal biogenesis factor 16
227266_s_at	2.96E-04	-0.74410257	FYB	FYN binding protein
223921_s_at	2.96E-04	0.18610484	GBA2	glucosylceramidase beta 2
220585_at	2.96E-04	0.18665541	HKDC1	hexokinase domain containing 1
209200_at	2.98E-04	-0.51055503	MEF2C	myocyte enhancer factor 2C
200808_s_at	2.99E-04	0.37371182	ZYX	zyxin
200805_at	3.02E-04	0.34185983	LMAN2	lectin, mannose binding 2
226992_at	3.02E-04	0.57286879	NOSTRIN	nitric oxide synthase trafficking
209428_s_at	3.03E-04	0.25198294	ZFPL1	zinc finger protein like 1
213879_at	3.05E-04	-0.30122699		
210749_x_at	3.08E-04	0.3255412	MIR4640///DDR1	microRNA 4640///discoidin domain receptor tyrosine kinase 1
233550_s_at	3.08E-04	0.175566	SLC4A11	solute carrier family 4 member 11
1557065_at	3.08E-04	-0.38055043	YLPM1	YLP motif containing 1
206452_x_at	3.10E-04	0.22775383	PTPA	protein phosphatase 2 phosphatase activator
213827_at	3.12E-04	0.13699732	ARHGAP33	Rho GTPase activating protein 33

211543_s_at	3.12E-04	0.24614031	GRK6	G protein-coupled receptor kinase 6
241771_at	3.12E-04	0.14892929	RIMBP2	RIMS binding protein 2
234192_s_at	3.15E-04	-0.44622335	GKAP1	G kinase anchoring protein 1
201126_s_at	3.15E-04	0.24347248	MGAT1	mannosyl (alpha-1,3-)-glycoprotein beta-1,2-N-acetylglucosaminyltransferase
233334_x_at	3.15E-04	0.32375219	SLX1B-SULT1A4///SLX1A-SULT1A3///SLX1A///SULT1A4///SLX1B///SULT1A3	SLX1B-SULT1A4 readthrough (NMD candidate)///SLX1A-SULT1A3 readthrough (NMD candidate)///SLX1 homolog A, structure-specific endonuclease subunit///sulfotransferase family 1A member 4///SLX1 homolog B, structure-specific endonuclease subunit///sulfotransferase family 1A member 3
32836_at	3.17E-04	0.27060874	AGPAT1	1-acylglycerol-3-phosphate O-acyltransferase 1
221432_s_at	3.17E-04	0.30537764	SLC25A28	solute carrier family 25 member 28
214853_s_at	3.19E-04	0.28498307	SHC1	SHC adaptor protein 1
226419_s_at	3.23E-04	-0.60246269	SRSF1	serine and arginine rich splicing factor 1
240883_at	3.25E-04	-0.1203643	PFKFB1	6-phosphofructo-2-kinase/fructose-2,6-biphosphatase 1
219152_at	3.28E-04	0.29569902	PODXL2	podocalyxin like 2
214704_at	3.29E-04	0.15267411	TCF25	transcription factor 25
204572_s_at	3.30E-04	-0.39646087	PIN4	peptidylprolyl cis/trans isomerase, NIMA-interacting 4
211518_s_at	3.32E-04	0.79490019	BMP4	bone morphogenetic protein 4
223019_at	3.34E-04	0.38088372	FAM129B	family with sequence similarity 129 member B
221490_at	3.35E-04	0.20220695	UBAP1	ubiquitin associated protein 1
220189_s_at	3.36E-04	0.40602135	MGAT4B	mannosyl (alpha-1,3-)-glycoprotein beta-1,4-N-acetylglucosaminyltransferase, isozyme B
202779_s_at	3.37E-04	0.56456063	UBE2S	ubiquitin conjugating enzyme E2 S
228632_at	3.38E-04	0.09750054	MEG9	maternally expressed 9 (non-protein coding)
200714_x_at	3.40E-04	0.26124019	OS9	OS9, endoplasmic reticulum lectin
233787_at	3.41E-04	-0.191348	C6orf163	chromosome 6 open reading frame 163
1555738_at	3.41E-04	0.1511092	CMTM1	CKLF like MARVEL transmembrane domain containing 1
223199_at	3.43E-04	0.40729071	MKNK2	MAP kinase interacting serine/threonine kinase 2
218132_s_at	3.45E-04	0.22818221	TSEN34	tRNA splicing endonuclease subunit 34
210865_at	3.49E-04	-0.20242919	FASLG	Fas ligand
228472_at	3.51E-04	0.23437597	CCDC61	coiled-coil domain containing 61
234350_at	3.52E-04	-0.1249699	IGLC1	immunoglobulin lambda constant 1
1564165_at	3.53E-04	0.12248521	LOC100630923	LOC100289561-PRKRIP1 readthrough
238115_at	3.56E-04	-0.22132974	DNAJC18	DnaJ heat shock protein family (Hsp40) member C18
218052_s_at	3.59E-04	0.31995471	ATP13A1	ATPase 13A1
212439_at	3.59E-04	0.19762229	IP6K1	inositol hexakisphosphate kinase 1
200919_at	3.60E-04	0.28325718	PHC2	polyhomeotic homolog 2
206723_s_at	3.61E-04	0.29069344	LPAR2	lysophosphatidic acid receptor 2
227378_x_at	3.63E-04	0.34712381	METTL26	methyltransferase like 26
206956_at	3.64E-04	0.25295056	PMF1-BGLAP///PMF1///BGLAP	PMF1-BGLAP readthrough///polyamine-modulated factor 1///bone gamma-carboxyglutamate protein
1554448_at	3.65E-04	-0.14935861	JPX	JPX transcript, XIST activator (non-protein coding)
212329_at	3.65E-04	0.31677799	SCAP	SREBF chaperone
201770_at	3.65E-04	0.38564183	SNRPA	small nuclear ribonucleoprotein polypeptide A
203365_s_at	3.67E-04	0.33973621	MMP15	matrix metallopeptidase 15
201052_s_at	3.67E-04	0.50029109	PSMF1	proteasome inhibitor subunit 1
230712_at	3.73E-04	-0.42763297	LOC102724250///LOC101930059///NBPF15///NBPF11///NBPF1	neuroblastoma breakpoint family member 1///uncharacterized LOC101930059///neuroblastoma breakpoint family member 15///neuroblastoma breakpoint family member 11///neuroblastoma breakpoint family member 1
215116_s_at	3.75E-04	0.59798565	DNM1	dynamin 1
215004_s_at	3.76E-04	0.26328489	SUGP1	SURP and G-patch domain containing 1
203559_s_at	3.79E-04	0.66264442	AOC1	amine oxidase, copper containing 1
223265_at	3.81E-04	0.18429514	SH3BP5L	SH3 binding domain protein 5 like
220323_at	3.82E-04	0.19571186	CNTD2	cyclin N-terminal domain containing 2
223054_at	3.82E-04	0.31984383	DNAJB11	DnaJ heat shock protein family (Hsp40) member B11
1564729_at	3.82E-04	-0.1174426	LOC101928666	uncharacterized LOC101928666
220948_s_at	3.85E-04	0.40903507	ATP1A1	ATPase Na+/K+ transporting subunit alpha 1
231128_at	3.85E-04	0.11835696	FAM181B	family with sequence similarity 181 member B

216010_x_at	3.85E-04	0.3995334	FUT3	fucosyltransferase 3 (Lewis blood group)
1567014_s_at	3.87E-04	0.46227337	NFE2L2///CSNK2A1	nuclear factor, erythroid 2 like 2///casein kinase 2 alpha 1
208906_at	3.88E-04	0.35515281	HNRNPUL2-BSCL2///BSCL2	HNRNPUL2-BSCL2 readthrough (NMD candidate)///BSCL2, seipin lipid droplet biogenesis associated
213104_at	3.88E-04	0.33589406	TSR3	TSR3, acp transferase ribosome maturation factor
231697_s_at	3.89E-04	-0.55592426	MIR21///VMP1	microRNA 21///vacuole membrane protein 1
1566833_x_at	3.90E-04	0.08357038	TOP1P2	topoisomerase (DNA) I pseudogene 2
205723_at	3.93E-04	0.16422569	CNTFR	ciliary neurotrophic factor receptor
219491_at	3.94E-04	0.2766312	LRFN4	leucine rich repeat and fibronectin type III domain containing 4
224611_s_at	3.97E-04	0.39437698	DNAJC5	DnaJ heat shock protein family (Hsp40) member C5
208759_at	3.98E-04	0.26417712	NCSTN	nicastrin
227621_at	3.99E-04	-0.45083388	WTAP	Wilms tumor 1 associated protein
202477_s_at	4.00E-04	0.27975195	TUBGCP2	tubulin gamma complex associated protein 2
228973_at	4.01E-04	0.13455734	DLG2	discs large MAGUK scaffold protein 2
229512_at	4.01E-04	-0.63914958	FAM120C	family with sequence similarity 120C
1552528_at	4.02E-04	0.12709488	LINC01547	long intergenic non-protein coding RNA 1547
204566_at	4.03E-04	-0.36679144	PPM1D	protein phosphatase, Mg2+/Mn2+ dependent 1D
204701_s_at	4.05E-04	0.22710195	STOML1	stomatin like 1
232159_at	4.08E-04	-0.11520422		
232078_at	4.12E-04	0.27179807	NECTIN2	nectin cell adhesion molecule 2
203500_at	4.13E-04	0.29537799	GCDH	glutaryl-CoA dehydrogenase
209998_at	4.15E-04	0.33417376	PIGO	phosphatidylinositol glycan anchor biosynthesis class O
201804_x_at	4.16E-04	0.40919446	TBCB	tubulin folding cofactor B
222631_at	4.17E-04	-0.33972585	SEPSECS-AS1///PI4K2B	SEPSECS antisense RNA 1 (head to head)///phosphatidylinositol 4-kinase type 2 beta
232163_at	4.18E-04	-0.09641771	WDR19	WD repeat domain 19
203522_at	4.21E-04	0.3173824	CCS	copper chaperone for superoxide dismutase
228017_s_at	4.26E-04	0.12084749	NKAIN4	Na+/K+ transporting ATPase interacting 4
212588_at	4.30E-04	-0.707502	PTPRC	protein tyrosine phosphatase, receptor type C
218693_at	4.30E-04	0.33361389	TSPAN15	tetraspanin 15
222003_s_at	4.35E-04	0.15608256	DOCK6	dedicator of cytokinesis 6
229051_at	4.35E-04	0.11464155		
64486_at	4.37E-04	0.28115965	CORO1B	coronin 1B
202292_x_at	4.40E-04	0.24138239	LYPLA2	lysophospholipase II
205086_s_at	4.43E-04	0.33423013	NCAPH2	non-SMC condensin II complex subunit H2
224846_at	4.44E-04	0.29141351	SHKBP1	SH3KBP1 binding protein 1
203254_s_at	4.46E-04	0.21031901	TLN1	talin 1
230815_at	4.48E-04	-0.36529026	LOC389765	kinesin family member 27 pseudogene
239717_at	4.53E-04	0.12047637	CHRNA10	cholinergic receptor nicotinic alpha 10 subunit
227821_at	4.54E-04	0.17511356	LGI4	leucine rich repeat LGI family member 4
212955_s_at	4.56E-04	0.39001745	POLR2I	RNA polymerase II subunit I
203655_at	4.56E-04	0.24135508	XRCC1	X-ray repair cross complementing 1
1554466_a_at	4.58E-04	0.30410967	METTL26	methyltransferase like 26
201234_at	4.59E-04	0.30267412	ILK	integrin linked kinase
240531_at	4.60E-04	-0.09674805		
222131_x_at	4.61E-04	0.21117228	RHOT2	ras homolog family member T2
209373_at	4.62E-04	0.50900719	MALL	mal, T-cell differentiation protein like
202492_at	4.63E-04	0.36509366	ATG9A	autophagy related 9A
233110_s_at	4.65E-04	0.27169517	BCL2L12	BCL2 like 12
210443_x_at	4.65E-04	0.19815508	OGFR	opioid growth factor receptor
217805_at	4.67E-04	0.41694394	ILF3	interleukin enhancer binding factor 3
201252_at	4.69E-04	0.33158497	PSMC4	proteasome 26S subunit, ATPase 4
209878_s_at	4.74E-04	0.21759201	RELA	RELA proto-oncogene, NF-kB subunit
206075_s_at	4.75E-04	0.41848122	CSNK2A1	casein kinase 2 alpha 1
203515_s_at	4.75E-04	0.3678903	PMVK	phosphomevalonate kinase
226131_s_at	4.78E-04	0.12692458	RPS16	ribosomal protein S16
231853_at	4.78E-04	-0.42502056	TUBD1	tubulin delta 1
220881_at	4.78E-04	-0.12711127		
228968_at	4.79E-04	-0.37304531	ZNF449	zinc finger protein 449
231291_at	4.80E-04	0.14040587	GIPR	gastric inhibitory polypeptide receptor

215714_s_at	4.81E-04	0.48990685	SMARCA4	SWI/SNF related, matrix associated, actin dependent regulator of chromatin, subfamily a, member 4
202996_at	4.84E-04	0.26684796	POLD4	DNA polymerase delta 4, accessory subunit
212358_at	4.86E-04	0.15209604	CLIP3	CAP-Gly domain containing linker protein 3
235546_at	4.86E-04	0.27204039	SPINT1	serine peptidase inhibitor, Kunitz type 1
206960_at	4.87E-04	-0.10257276	LPAR4	lysophosphatidic acid receptor 4
210417_s_at	4.87E-04	0.27899459	PI4KB	phosphatidylinositol 4-kinase beta
204605_at	4.88E-04	-0.36953553	CGRRF1	cell growth regulator with ring finger domain 1
205182_s_at	4.88E-04	0.23121032	ZNF324	zinc finger protein 324
221995_s_at	4.90E-04	-0.32515346	MRPL57	mitochondrial ribosomal protein L57
1555896_a_at	4.92E-04	0.14703135	ADAM15	ADAM metallopeptidase domain 15
203966_s_at	4.92E-04	-0.2848668	PPM1A	protein phosphatase, Mg2+/Mn2+ dependent 1A
226495_at	4.96E-04	0.39842908	MAVS	mitochondrial antiviral signaling protein
240413_at	4.96E-04	-0.15794471	PYHIN1	pyrin and HIN domain family member 1
210678_s_at	4.97E-04	0.34633461	AGPAT2	1-acylglycerol-3-phosphate O-acyltransferase 2
225058_at	4.97E-04	0.25919409	MIR6791///GPR108	microRNA 6791///G protein-coupled receptor 108
205217_at	5.04E-04	-0.44463041	TIMM8A	translocase of inner mitochondrial membrane 8 homolog A (yeast)
226206_at	5.05E-04	0.35405419	MAFK	MAF bZIP transcription factor K
217787_s_at	5.08E-04	0.34899561	GALNT2	polypeptide N-acetylgalactosaminyltransferase 2
1554447_at	5.08E-04	-0.48394292	JPX	JPX transcript, XIST activator (non-protein coding)
212520_s_at	5.15E-04	0.34711695	SMARCA4	SWI/SNF related, matrix associated, actin dependent regulator of chromatin, subfamily a, member 4
202855_s_at	5.17E-04	0.2449271	MIR6787///SLC16A3	microRNA 6787///solute carrier family 16 member 3
201979_s_at	5.17E-04	0.18120159	PPP5C	protein phosphatase 5 catalytic subunit
1553728_at	5.18E-04	0.20568055	LRRC43	leucine rich repeat containing 43
217934_x_at	5.23E-04	0.3251959	STUB1	STIP1 homology and U-box containing protein 1
202737_s_at	5.25E-04	0.3641782	LSM4	LSM4 homolog, U6 small nuclear RNA and mRNA degradation associated
209415_at	5.26E-04	0.12919638	FZR1	fizzy/cell division cycle 20 related 1
213746_s_at	5.34E-04	0.30910422	FLNA	filamin A
218538_s_at	5.34E-04	-0.36017706	MRS2	MRS2, magnesium transporter
221267_s_at	5.40E-04	0.41478434	ABHD17A	abhydrolase domain containing 17A
202318_s_at	5.43E-04	-0.27059548	SENP6	SUMO1/sentrin specific peptidase 6
1557470_at	5.46E-04	0.17419285	SPATA13	spermatogenesis associated 13
225035_x_at	5.47E-04	0.38196857	LOC102723897///MIR6859-1///MIR6859-2///LOC101930154///LOC100288778///WASH1///WASH7P///WASH2P///WASH3P	WAS protein family homolog 2-like///microRNA 6859-1///microRNA 6859-2///WAS protein family homolog 6-like///WAS protein family homolog 1 pseudogene///WAS protein family homolog 1///WAS protein family homolog 7 pseudogene///WAS protein family homolog 2 pseudogene///WAS protein family homolog 3 pseudogene
201907_x_at	5.50E-04	0.16088248	DVL3	dishevelled segment polarity protein 3
202826_at	5.53E-04	0.35511787	SPINT1	serine peptidase inhibitor, Kunitz type 1
1553594_a_at	5.58E-04	0.13162711	INSL3	insulin like 3
212838_at	5.61E-04	0.3012803	DNMBP	dynamin binding protein
203370_s_at	5.63E-04	0.22435544	PDLIM7	PDZ and LIM domain 7
233614_at	5.63E-04	0.09914895		
216194_s_at	5.65E-04	0.42663216	TBCB	tubulin folding cofactor B
231061_at	5.71E-04	-0.29590953	MIR302B	microRNA 302b
201258_at	5.71E-04	0.38836116	RPS16	ribosomal protein S16
208369_s_at	5.73E-04	0.29672806	GCDH	glutaryl-CoA dehydrogenase
222263_at	5.73E-04	0.193593	SLC35E1	solute carrier family 35 member E1
233881_s_at	5.74E-04	0.15206231	TOLLIP	toll interacting protein
209199_s_at	5.75E-04	-0.46047636	MEF2C	myocyte enhancer factor 2C
211716_x_at	5.76E-04	0.27506836	ARHGDIA	Rho GDP dissociation inhibitor alpha
217226_s_at	5.78E-04	0.22499589	SFXN3	sideroflexin 3
202394_s_at	5.83E-04	0.22609422	ABCF3	ATP binding cassette subfamily F member 3
208625_s_at	5.83E-04	0.36707013	EIF4G1	eukaryotic translation initiation factor 4 gamma 1
211623_s_at	5.83E-04	0.37310505	FBL	fibrillarin
37996_s_at	5.87E-04	0.20350338	DMPK	dystrophia myotonica protein kinase
214121_x_at	5.90E-04	0.18960058	PDLIM7	PDZ and LIM domain 7
231056_at	5.90E-04	0.22833922		

220477_s_at	5.91E-04	0.55161887	TMEM230	transmembrane protein 230
1555272_at	5.94E-04	-0.24696645	RSPH10B2///RSPH10B	radial spoke head 10 homolog B2///radial spoke head 10 homolog B
219937_at	5.97E-04	-0.91402039	TRHDE	thyrotropin releasing hormone degrading enzyme
55583_at	5.99E-04	0.28155265	DOCK6	dedicator of cytokinesis 6
208821_at	6.01E-04	0.54833686	SNRPB	small nuclear ribonucleoprotein polypeptides B and B1
208837_at	6.02E-04	0.39375784	TMED3	transmembrane p24 trafficking protein 3
38766_at	6.03E-04	0.24430288	TMEM265///SRCAP	transmembrane protein 265///Snf2-related CREBBP activator protein
222349_x_at	6.04E-04	0.20514242	RNF126P1	ring finger protein 126 pseudogene 1
224727_at	6.05E-04	0.21495175	EMC10	ER membrane protein complex subunit 10
227325_at	6.06E-04	0.30267422	INAFM1	InaF motif containing 1
202152_x_at	6.07E-04	0.17892412	USF2	upstream transcription factor 2, c-fos interacting
234858_at	6.09E-04	0.13263606		
233385_x_at	6.11E-04	0.2506457	MIA-RAB4B///RAB4B	MIA-RAB4B readthrough (NMD candidate)///RAB4B, member RAS oncogene family
204857_at	6.13E-04	0.50631568	MAD1L1	MAD1 mitotic arrest deficient like 1
65770_at	6.21E-04	0.240587	RHOT2	ras homolog family member T2
230124_at	6.22E-04	0.12357324	PRKCH	protein kinase C eta
218661_at	6.27E-04	0.23731771	NAA60	N(alpha)-acetyltransferase 60, NatF catalytic subunit
208291_s_at	6.28E-04	0.1967028	TH	tyrosine hydroxylase
207555_s_at	6.29E-04	0.12531495	TBXA2R	thromboxane A2 receptor
201281_at	6.30E-04	0.4051637	ADRM1	adhesion regulating molecule 1
227578_at	6.32E-04	-0.15779681	TMPO-AS1	TMPO antisense RNA 1
206722_s_at	6.33E-04	0.19107271	LPAR2	lysophosphatidic acid receptor 2
201286_at	6.33E-04	0.32818838	SDC1	syndecan 1
240393_at	6.33E-04	0.15621131		
215031_x_at	6.38E-04	0.2269133	RNF126	ring finger protein 126
207595_s_at	6.42E-04	0.15480658	BMP1	bone morphogenetic protein 1
1555895_at	6.42E-04	0.31021216	DNM2	dynamin 2
227096_at	6.45E-04	0.14404012	JOSD2	Josephin domain containing 2
227511_at	6.47E-04	0.18022122	SAMD4B	sterile alpha motif domain containing 4B
215732_s_at	6.48E-04	0.31818283	LOC102725292///DTX2	probable E3 ubiquitin-protein ligase DTX2-like///deltex E3 ubiquitin ligase 2
200003_s_at	6.52E-04	0.29494513	MIR6805///RPL28	microRNA 6805///ribosomal protein L28
225436_at	6.53E-04	0.29679052	ABHD17C	abhydrolase domain containing 17C
33814_at	6.53E-04	0.16780478	PAK4	p21 (RAC1) activated kinase 4
235922_at	6.53E-04	0.19364751	TPM4	tropomyosin 4
201102_s_at	6.59E-04	0.28456902	PFKL	phosphofructokinase, liver type
240715_at	6.59E-04	-0.15888361	TBX5	T-box 5
213885_at	6.59E-04	0.15098636	TRIM3	tripartite motif containing 3
218020_s_at	6.59E-04	0.22781291	ZFAND3	zinc finger AN1-type containing 3
225247_at	6.61E-04	0.28980609	TMEM259	transmembrane protein 259
203679_at	6.63E-04	0.23647641	TMED1	transmembrane p24 trafficking protein 1
231090_s_at	6.64E-04	-0.26240211	ARID2	AT-rich interaction domain 2
223790_at	6.69E-04	-0.21259383	KATNAL1	katanin catalytic subunit A1 like 1
1568408_x_at	6.73E-04	0.13563319		
1560477_a_at	6.77E-04	0.12135949	SAMD11	sterile alpha motif domain containing 11
200859_x_at	6.78E-04	0.30939973	FLNA	filamin A
212870_at	6.79E-04	-0.37866733	SOS2	SOS Ras/Rho guanine nucleotide exchange factor 2
219878_s_at	6.80E-04	0.32115265	KLF13	Kruppel like factor 13
219911_s_at	6.80E-04	0.53483213	SLCO4A1	solute carrier organic anion transporter family member 4A1
226405_s_at	6.81E-04	0.33100813	ARRDC1	arrestin domain containing 1
1561537_at	6.82E-04	0.12178505	OGFRP1	opioid growth factor receptor pseudogene 1
235110_at	6.82E-04	0.21343236	PLA2G16	phospholipase A2 group XVI
233049_x_at	6.83E-04	0.33906053	STUB1	STIP1 homology and U-box containing protein 1
1556017_at	6.84E-04	0.15521978	NBEAL2	neurobeachin like 2
217788_s_at	6.89E-04	0.34893823	GALNT2	polypeptide N-acetylgalactosaminyltransferase 2
226451_at	6.90E-04	0.23705758	FDX1L	ferredoxin 1 like
213986_s_at	6.91E-04	0.26050831	TMEM259	transmembrane protein 259
227845_s_at	6.93E-04	0.1529305	SHD	Src homology 2 domain containing transforming protein D

208659_at	6.96E-04	0.2196364	CLIC1	chloride intracellular channel 1
203706_s_at	6.96E-04	-0.89970035	FZD7	frizzled class receptor 7
203209_at	6.98E-04	-0.34491051	RFC5	replication factor C subunit 5
205812_s_at	6.99E-04	0.24821849	TMED9	transmembrane p24 trafficking protein 9
244143_at	7.08E-04	0.08726248		
217891_at	7.10E-04	0.35967469	C16orf58	chromosome 16 open reading frame 58
207985_at	7.11E-04	0.09507881		
232922_s_at	7.12E-04	0.1710208	SLC17A9	solute carrier family 17 member 9
202159_at	7.15E-04	0.30061874	FARSA	phenylalanyl-tRNA synthetase alpha subunit
210743_s_at	7.18E-04	-0.14983945	CDC14A	cell division cycle 14A
216905_s_at	7.18E-04	0.38678846	ST14	suppression of tumorigenicity 14
203110_at	7.19E-04	0.24958366	PTK2B	protein tyrosine kinase 2 beta
212443_at	7.20E-04	0.40268551	NBEAL2	neurobeachin like 2
226409_at	7.22E-04	0.37032992	TBC1D20	TBC1 domain family member 20
242376_at	7.22E-04	0.13126486		
1561006_at	7.23E-04	-0.10510306		
202354_s_at	7.25E-04	0.30853356	GTF2F1	general transcription factor IIF subunit 1
218961_s_at	7.32E-04	0.42435897	PNKP	polynucleotide kinase 3'-phosphatase
232126_at	7.35E-04	-0.19524254	COQ2	coenzyme Q2, polyprenyltransferase
216360_x_at	7.35E-04	0.10336027	RRP12	ribosomal RNA processing 12 homolog
205993_s_at	7.36E-04	0.11461722	TBX2	T-box 2
210655_s_at	7.38E-04	0.28813062	FOXO3B///FOXO3	forkhead box O3B pseudogene///forkhead box O3
204202_at	7.41E-04	0.36879163	IQCE	IQ motif containing E
31846_at	7.41E-04	0.19236884	RHOD	ras homolog family member D
210974_s_at	7.42E-04	0.28646993	AP3D1	adaptor related protein complex 3 delta 1 subunit
1555322_at	7.45E-04	0.09415477	PP2672	uncharacterized LOC100130249
237070_at	7.47E-04	-0.08042101	TRPM1	transient receptor potential cation channel subfamily M member 1
221794_at	7.48E-04	0.29025993	DOCK6	dedicator of cytokinesis 6
209171_at	7.48E-04	0.37127219	ITPA	inosine triphosphatase
210088_x_at	7.48E-04	0.14132502	MYL4	myosin light chain 4
212576_at	7.50E-04	0.19908671	MGRN1	mahogunin ring finger 1
222155_s_at	7.51E-04	0.54058583	SLC52A2	solute carrier family 52 member 2
1569283_at	7.53E-04	-0.31443439	ZNF891	zinc finger protein 891
205574_x_at	7.55E-04	0.22871872	BMP1	bone morphogenetic protein 1
218720_x_at	7.55E-04	0.4650324	SEZ6L2	seizure related 6 homolog like 2
217943_s_at	7.56E-04	0.23880709	MAP7D1	MAP7 domain containing 1
222976_s_at	7.56E-04	0.22789535	TPM3	tropomyosin 3
235344_at	7.59E-04	-0.25576098	PPM1A	protein phosphatase, Mg2+/Mn2+ dependent 1A
241938_at	7.59E-04	-0.36426108	QKI	QKI, KH domain containing RNA binding
203206_at	7.60E-04	0.20201679	FAM53B	family with sequence similarity 53 member B
230337_at	7.62E-04	-0.47757398	SOS1	SOS Ras/Rac guanine nucleotide exchange factor 1
209117_at	7.63E-04	0.32107808	WBP2	WW domain binding protein 2
238792_at	7.64E-04	-0.23718735	PCNX1	pecanex homolog 1 (Drosophila)
242181_at	7.64E-04	0.51491178		
214484_s_at	7.65E-04	0.36488903	SIGMAR1	sigma non-opioid intracellular receptor 1
244252_at	7.66E-04	0.10475429	LOC399884	uncharacterized LOC399884
209156_s_at	7.71E-04	0.56623443	COL6A2	collagen type VI alpha 2 chain
233929_x_at	7.72E-04	0.37079589	MIR6859-1///MIR6859-2///LOC101930154///LOC100288778///WASH1///WASH7P///WASH2P///WASH3P	microRNA 6859-1///microRNA 6859-2///WAS protein family homolog 6-like///WAS protein family homolog 1 pseudogene///WAS protein family homolog 1///WAS protein family homolog 7 pseudogene///WAS protein family homolog 2 pseudogene///WAS protein family homolog 3 pseudogene
239689_at	7.73E-04	0.21367939		
243702_at	7.78E-04	-0.31148361		
234521_at	7.81E-04	-0.13804245	OR51I2	olfactory receptor family 51 subfamily I member 2
240485_at	7.86E-04	-0.2959818		
218286_s_at	7.87E-04	0.27385289	RNF7	ring finger protein 7
219185_at	7.88E-04	-0.33438402	SIRT5	sirtuin 5
202262_x_at	7.95E-04	0.41821149	DDAH2	dimethylarginine dimethylaminohydrolase 2
1565254_s_at	8.00E-04	0.08506614	ELL	elongation factor for RNA polymerase II
204966_at	8.01E-04	0.1078032	ADGRB2	adhesion G protein-coupled receptor B2

1560620_at	8.02E-04	0.15371271	KCNK15	potassium two pore domain channel subfamily K member 15
205581_s_at	8.09E-04	0.30642938	NOS3	nitric oxide synthase 3
244861_at	8.11E-04	0.15673031	ZNF527	zinc finger protein 527
205288_at	8.12E-04	-0.41521971	CDC14A	cell division cycle 14A
226660_at	8.12E-04	-0.30983237	RPS6KB1	ribosomal protein S6 kinase B1
204927_at	8.16E-04	0.23357371	RASSF7	Ras association domain family member 7
223394_at	8.16E-04	0.29846703	SERTAD1	SERTA domain containing 1
213813_x_at	8.16E-04	-0.23414766		
219559_at	8.17E-04	0.42152144	SLC17A9	solute carrier family 17 member 9
1558306_at	8.19E-04	-0.10978188	THADA	THADA, armadillo repeat containing
222554_s_at	8.20E-04	0.15957297	NOL6	nucleolar protein 6
201654_s_at	8.23E-04	0.15109287	HSPG2	heparan sulfate proteoglycan 2
210212_x_at	8.25E-04	-0.37008605	CMC4	C-X9-C motif containing 4
204990_s_at	8.25E-04	0.35478541	ITGB4	integrin subunit beta 4
241530_at	8.36E-04	0.08367905		
201087_at	8.37E-04	0.24537934	PXN	paxillin
37028_at	8.39E-04	0.33400915	PPP1R15A	protein phosphatase 1 regulatory subunit 15A
215537_x_at	8.46E-04	0.43550768	DDAH2	dimethylarginine dimethylaminohydrolase 2
200846_s_at	8.50E-04	0.40496402	PPP1CA	protein phosphatase 1 catalytic subunit alpha
209461_x_at	8.50E-04	0.35887626	WDR18	WD repeat domain 18
90265_at	8.51E-04	0.40367943	ADAP1	ArfGAP with dual PH domains 1
215363_x_at	8.52E-04	-0.22803952	FOLH1B///FOLH1	folate hydrolase 1B///folate hydrolase (prostate-specific membrane antigen) 1
208641_s_at	8.56E-04	0.26761706	RAC1	ras-related C3 botulinum toxin substrate 1 (rho family, small GTP binding protein Rac1)
207396_s_at	8.61E-04	0.34561733	ALG3	ALG3, alpha-1,3- mannosyltransferase
219224_x_at	8.66E-04	0.15688382	ZNF408	zinc finger protein 408
201954_at	8.71E-04	0.41191201	ARPC1B	actin related protein 2/3 complex subunit 1B
210013_at	8.72E-04	0.14816787	HPX	hemopexin
217855_x_at	8.77E-04	0.25202653	SDF4	stromal cell derived factor 4
237167_at	8.79E-04	0.21976357	KIAA1217	KIAA1217
221867_at	8.79E-04	0.29618383	N4BP1	NEDD4 binding protein 1
201953_at	8.83E-04	0.4129708	CIB1	calcium and integrin binding 1
201206_s_at	8.83E-04	0.51514198	RRBP1	ribosome binding protein 1
202794_at	8.88E-04	0.35873847	INPP1	inositol polyphosphate-1-phosphatase
1556401_a_at	8.88E-04	0.16762522	LOC105378589	uncharacterized LOC105378589
221628_s_at	8.93E-04	0.14351168	GLYR1	glyoxylate reductase 1 homolog
228413_s_at	8.93E-04	0.10270256	SFRP1	secreted frizzled related protein 1
204661_at	8.99E-04	-0.45944556	CD52	CD52 molecule
212505_s_at	9.00E-04	0.19203911	MAU2	MAU2 sister chromatid cohesion factor
1554417_s_at	9.01E-04	0.26031559	APH1A	aph-1 homolog A, gamma-secretase subunit
221848_at	9.01E-04	0.40029507	ZGPAT	zinc finger CCCH-type and G-patch domain containing
233349_at	9.03E-04	-0.10481908		
210628_x_at	9.04E-04	0.22018598	LTBP4	latent transforming growth factor beta binding protein 4
204021_s_at	9.05E-04	0.2516116	PURA	purine rich element binding protein A
210042_s_at	9.07E-04	0.39574192	CTSZ	cathepsin Z
223389_s_at	9.07E-04	0.31799317	ZNF581	zinc finger protein 581
217804_s_at	9.09E-04	0.23326911	ILF3	interleukin enhancer binding factor 3
213462_at	9.11E-04	0.26535674	NPAS2	neuronal PAS domain protein 2
244882_at	9.11E-04	-0.13670274	TNRC18	trinucleotide repeat containing 18
209427_at	9.13E-04	0.29691791	SMTN	smoothelin
205015_s_at	9.23E-04	0.12623823	TGFA	transforming growth factor alpha
1559563_at	9.24E-04	0.11372792		
206878_at	9.27E-04	-0.12660947	DAO	D-amino acid oxidase
35179_at	9.28E-04	0.18481316	B3GAT3	beta-1,3-glucuronyltransferase 3
229170_s_at	9.39E-04	-0.42104463	CFAP70	cilia and flagella associated protein 70
228884_at	9.39E-04	-0.10520974	LRRC27	leucine rich repeat containing 27
243239_at	9.39E-04	-0.10436225	SAMM50	SAMM50 sorting and assembly machinery component
211598_x_at	9.39E-04	0.13930421	VIPR2	vasoactive intestinal peptide receptor 2
212045_at	9.40E-04	0.25183884	GLG1	golgi glycoprotein 1
1560488_at	9.44E-04	0.10398471	LCNL1	lipocalin like 1

228463_at	9.49E-04	0.59493252	FOXA3	forkhead box A3
219722_s_at	9.57E-04	0.46243045	GDPD3	glycerophosphodiester phosphodiesterase domain containing 3
215535_s_at	9.58E-04	0.2818883	AGPAT1	1-acylglycerol-3-phosphate O-acyltransferase 1
217729_s_at	9.68E-04	0.27876178	AES	amino-terminal enhancer of split
216298_at	9.69E-04	-0.08708168	TARP	TCR gamma alternate reading frame protein
209857_s_at	9.73E-04	0.2001338	SPHK2	sphingosine kinase 2
243948_at	9.74E-04	-0.45198069	ZC3H14	zinc finger CCCH-type containing 14
241017_at	9.75E-04	-0.81932653	TBC1D8///RPL31	TBC1 domain family member 8///ribosomal protein L31
1553730_x_at	9.78E-04	0.12833299	LRRC43	leucine rich repeat containing 43
241016_at	9.78E-04	-0.08742775		
220757_s_at	9.82E-04	0.24134161	MIR4746///UBXN6	microRNA 4746///UBX domain protein 6
203533_s_at	9.85E-04	-0.31858495	CUL5	cullin 5
1558331_at	9.86E-04	0.16885262	SIRT2	sirtuin 2
1554086_at	9.86E-04	0.35548261	TUBGCP3	tubulin gamma complex associated protein 3
204248_at	9.91E-04	0.33870988	GNA11	G protein subunit alpha 11
216642_at	9.93E-04	-0.1171636		
1555031_at	9.97E-04	0.09567317		
213384_x_at	1.00E-03	0.17344108	PLCB3	phospholipase C beta 3
227630_at	1.00E-03	-0.29152455	PPP2R5E	protein phosphatase 2 regulatory subunit B'epsilon
224833_at	1.01E-03	-0.32017371	ETS1	ETS proto-oncogene 1, transcription factor
202746_at	1.01E-03	-0.58907392	ITM2A	integral membrane protein 2A
211217_s_at	1.01E-03	0.15177392	KCNQ1	potassium voltage-gated channel subfamily Q member 1
244843_x_at	1.01E-03	-0.11848544	LOC105371226	uncharacterized LOC105371226
215707_s_at	1.01E-03	0.20524496	PRNP	prion protein
1563946_at	1.01E-03	0.10226108		
230955_s_at	1.02E-03	0.15707265	NOL4L	nucleolar protein 4 like
203859_s_at	1.02E-03	0.09978494	PALM	paralemmin
209345_s_at	1.02E-03	0.21724952	PI4K2A	phosphatidylinositol 4-kinase type 2 alpha
232883_at	1.03E-03	-0.19510919		
1568777_at	1.04E-03	-0.14630996	EML5	echinoderm microtubule associated protein like 5
207651_at	1.04E-03	-0.6763119	GPR171	G protein-coupled receptor 171
243078_at	1.04E-03	0.12253369		
201575_at	1.05E-03	-0.22665107	SNW1	SNW domain containing 1
200672_x_at	1.05E-03	0.26445551	SPTBN1	spectrin beta, non-erythrocytic 1
219010_at	1.06E-03	0.32783415	C1orf106	chromosome 1 open reading frame 106
213045_at	1.06E-03	0.267091	MAST3	microtubule associated serine/threonine kinase 3
1007_s_at	1.06E-03	0.22845852	MIR4640///DDR1	microRNA 4640///discoidin domain receptor tyrosine kinase 1
218028_at	1.06E-03	0.3274904	MIR6734///ELOVL1	microRNA 6734///ELOVL fatty acid elongase 1
1554600_s_at	1.07E-03	0.41726265	LMNA	lamin A/C
202198_s_at	1.07E-03	0.13192009	MTMR3	myotubularin related protein 3
215706_x_at	1.07E-03	0.25774529	ZYX	zyxin
226156_at	1.08E-03	0.36910429	AKT2	AKT serine/threonine kinase 2
232543_x_at	1.08E-03	-0.291746	ARHGAP9	Rho GTPase activating protein 9
216862_s_at	1.08E-03	-0.34685979	CMC4	C-X9-C motif containing 4
217146_at	1.08E-03	0.12175755	JRK	Jrk helix-turn-helix protein
244790_at	1.08E-03	-0.30908833	MTCP1	mature T-cell proliferation 1
203701_s_at	1.08E-03	0.36411559	TRMT1	tRNA methyltransferase 1
203422_at	1.09E-03	0.33028696	POLD1	DAN polymerase delta 1, catalytic subunit
227728_at	1.09E-03	-0.37246674	PPM1A	protein phosphatase, Mg2+/Mn2+ dependent 1A
202639_s_at	1.09E-03	0.14959302	RANBP3	RAN binding protein 3
1557226_a_at	1.10E-03	0.11728393	ASPG	asparaginase
212393_at	1.10E-03	0.20963305	SBF1	SET binding factor 1
243595_at	1.10E-03	0.12556528	SHB	SH2 domain containing adaptor protein B
229355_at	1.10E-03	-0.33123054	UBE2D3	ubiquitin conjugating enzyme E2 D3
212984_at	1.11E-03	-0.26529126	ATF2	activating transcription factor 2
230820_at	1.11E-03	-0.415699	SMURF2	SMAD specific E3 ubiquitin protein ligase 2
212144_at	1.11E-03	0.21266036	SUN2	Sad1 and UNC84 domain containing 2
218563_at	1.12E-03	0.32103035	NDUFA3	NADH:ubiquinone oxidoreductase subunit A3
218601_at	1.12E-03	0.24014864	URGCP	upregulator of cell proliferation
202790_at	1.13E-03	0.39983922	CLDN7	claudin 7

224814_at	1.13E-03	0.35317564	DPP7	dipeptidyl peptidase 7
223353_at	1.13E-03	0.22546862	MOB2	MOB kinase activator 2
221593_s_at	1.13E-03	-0.34649549	RPL31	ribosomal protein L31
226033_at	1.13E-03	-0.27712057	USP31	ubiquitin specific peptidase 31
211795_s_at	1.14E-03	-0.50937585	FYB	FYN binding protein
209377_s_at	1.14E-03	-0.47038823	HMGN3	high mobility group nucleosomal binding domain 3
224477_s_at	1.14E-03	0.22132674	NUDT16L1	nudix hydrolase 16 like 1
213795_s_at	1.14E-03	0.37412332	PTPRA	protein tyrosine phosphatase, receptor type A
201203_s_at	1.14E-03	0.42750717	RRBP1	ribosome binding protein 1
202125_s_at	1.14E-03	-0.32504048	TRAK2	trafficking kinesin protein 2
219791_s_at	1.15E-03	0.08686211	HAND2-AS1	HAND2 antisense RNA 1 (head to head)
205269_at	1.15E-03	-0.50848615	LCP2	lymphocyte cytosolic protein 2
1556257_at	1.15E-03	0.09610903	LOC645513	septin 7 pseudogene
208779_x_at	1.15E-03	0.26323629	MIR4640///DDR1	microRNA 4640///discoidin domain receptor tyrosine kinase 1
209084_s_at	1.15E-03	-0.28298742	RAB28	RAB28, member RAS oncogene family
206845_s_at	1.15E-03	0.2815549	RNF40	ring finger protein 40
52078_at	1.15E-03	0.16188375	TMEM222	transmembrane protein 222
203378_at	1.16E-03	-0.30843992	PCF11	PCF11 cleavage and polyadenylation factor subunit
213890_x_at	1.16E-03	0.14653758	RPS16	ribosomal protein S16
211184_s_at	1.16E-03	0.37582163	USH1C	USH1 protein network component harmonin
218762_at	1.16E-03	0.19374441	ZNF574	zinc finger protein 574
215420_at	1.17E-03	0.1845815	IHH	indian hedgehog
224886_at	1.17E-03	0.19455586	JMJD8	jumonji domain containing 8
204182_s_at	1.17E-03	0.19831329	ZBTB43	zinc finger and BTB domain containing 43
1566002_at	1.17E-03	0.12395427		
212360_at	1.18E-03	0.21400705	AMPD2	adenosine monophosphate deaminase 2
215631_s_at	1.18E-03	0.31950828	BRMS1	breast cancer metastasis suppressor 1
201677_at	1.18E-03	0.21083223	HMCES	5-hydroxymethylcytosine (hmC) binding, ES cell-specific
239019_at	1.18E-03	0.13275588	KLF12	Kruppel like factor 12
201073_s_at	1.18E-03	0.13637848	SMARCC1	SWI/SNF related, matrix associated, actin dependent regulator of chromatin subfamily c member 1
224868_at	1.18E-03	0.18705091	ZDHHC5	zinc finger DHHC-type containing 5
203932_at	1.19E-03	-0.4955601	HLA-DMB	major histocompatibility complex, class II, DM beta
1555634_a_at	1.19E-03	0.09575814	LILRA5	leukocyte immunoglobulin like receptor A5
38710_at	1.19E-03	0.2125759	LOC101927673///OTUB1	uncharacterized LOC101927673///OTU deubiquitinase, ubiquitin aldehyde binding 1
1561110_at	1.19E-03	-0.07349803	LOC102723648	uncharacterized LOC102723648
208791_at	1.20E-03	0.6801859	CLU	clusterin
238752_at	1.20E-03	-0.22332107	GPLD1	glycosylphosphatidylinositol specific phospholipase D1
212444_at	1.20E-03	0.47342053	GPRC5A	G protein-coupled receptor class C group 5 member A
203308_x_at	1.20E-03	0.1329227	HPS1	HPS1, biogenesis of lysosomal organelles complex 3 subunit 1
232526_at	1.20E-03	0.11502071	ITPKB	inositol-trisphosphate 3-kinase B
210927_x_at	1.20E-03	0.16807303	JTB	jumping translocation breakpoint
231226_at	1.20E-03	-0.13268166	MED14OS	MED14 opposite strand
227994_x_at	1.20E-03	0.31959925	PPDPF	pancreatic progenitor cell differentiation and proliferation factor
220767_at	1.20E-03	-0.07974411		
226494_at	1.21E-03	0.21351372	CAMSAP3	calmodulin regulated spectrin associated protein family member 3
236945_at	1.21E-03	-0.11286899	CCDC171	coiled-coil domain containing 171
1555751_a_at	1.21E-03	0.24847592	GEMIN7	gem nuclear organelle associated protein 7
200866_s_at	1.21E-03	0.42802604	PSAP	prosaposin
203136_at	1.21E-03	0.44226062	RABAC1	Rab acceptor 1
230713_at	1.21E-03	-0.35550449		
201781_s_at	1.22E-03	0.26347257	AIP	aryl hydrocarbon receptor interacting protein
222807_at	1.22E-03	-0.28075723	EMSY	EMSY, BRCA2 interacting transcriptional repressor
243989_at	1.22E-03	0.087342		
231189_at	1.23E-03	0.14061928	LINC01126	long intergenic non-protein coding RNA 1126
219777_at	1.25E-03	-0.52325139	GIMAP6	GTPase, IMAP family member 6
231910_at	1.25E-03	0.11012339	NUDT14	nudix hydrolase 14
212125_at	1.25E-03	0.29127452	RANGAP1	Ran GTPase activating protein 1

237683_s_at	1.25E-03	-0.11812902		
228123_s_at	1.26E-03	0.56244975	ABHD12	abhydrolase domain containing 12
244166_at	1.26E-03	-0.26467022	APLN	apelin
201439_at	1.26E-03	0.1690323	GBF1	golgi brefeldin A resistant guanine nucleotide exchange factor 1
206136_at	1.27E-03	0.25180141	FZD5	frizzled class receptor 5
229204_at	1.27E-03	-0.2897066	HP1BP3	heterochromatin protein 1 binding protein 3
1562408_at	1.27E-03	0.13244231	LOC101926984	leucine-rich repeat-containing protein 37A3
209468_at	1.27E-03	0.27745763	LRP5	LDL receptor related protein 5
205137_x_at	1.27E-03	0.35245908	USH1C	USH1 protein network component harmonin
212892_at	1.27E-03	0.26883855	ZNF282	zinc finger protein 282
202630_at	1.28E-03	-0.33883077	APPBP2	amyloid beta precursor protein binding protein 2
238122_at	1.28E-03	-0.30446891	RBM12B	RNA binding motif protein 12B
31807_at	1.29E-03	0.19433731	DDX49	DEAD-box helicase 49
229311_at	1.29E-03	-0.1498077	GKAP1	G kinase anchoring protein 1
217953_at	1.29E-03	-0.18326007	PHF3	PHD finger protein 3
61734_at	1.29E-03	0.29379013	RCN3	reticulocalbin 3
201908_at	1.30E-03	0.3294919	DVL3	dishevelled segment polarity protein 3
235263_at	1.30E-03	0.17800023	STAG3L2///STAG3L3///STAG3L1	stromal antigen 3-like 2 (pseudogene)///stromal antigen 3-like 3 (pseudogene)///stromal antigen 3-like 1 (pseudogene)
225648_at	1.30E-03	0.38180325	STK35	serine/threonine kinase 35
207546_at	1.31E-03	0.0972006	ATP4B	ATPase H+/K+ transporting beta subunit
211558_s_at	1.31E-03	0.35200294	DHPS	deoxyhypusine synthase
213906_at	1.31E-03	-0.70184241	MYBL1	MYB proto-oncogene like 1
231408_at	1.31E-03	0.32824173	TMEM92	transmembrane protein 92
223233_s_at	1.32E-03	0.31480839	CGN	cingulin
200766_at	1.32E-03	0.36354428	CTSD	cathepsin D
227544_at	1.32E-03	0.23600755	TMEM229B	transmembrane protein 229B
218081_at	1.33E-03	0.277188	C20orf27	chromosome 20 open reading frame 27
1553678_a_at	1.33E-03	0.30173163	ITGB1	integrin subunit beta 1
228114_x_at	1.33E-03	0.20916686	METTL26	methyltransferase like 26
203582_s_at	1.33E-03	0.39306602	SPHAR///RAB4A	S-phase response (cyclin related)///RAB4A, member RAS oncogene family
1554954_at	1.33E-03	0.11878822	TSPEAR-AS2	TSPEAR antisense RNA 2
238586_at	1.33E-03	0.08906975		
202913_at	1.34E-03	0.20621842	ARHGEF11	Rho guanine nucleotide exchange factor 11
236830_at	1.34E-03	-0.14673937		
217888_s_at	1.35E-03	0.34948197	ARFGAP1	ADP ribosylation factor GTPase activating protein 1
226340_x_at	1.35E-03	0.31919937	MIR6859-1///MIR6859-2///LOC101930154///LOC100288778///WASH1///WASH7P///WASH2P///WASH3P	microRNA 6859-1///microRNA 6859-2///WAS protein family homolog 6-like///WAS protein family homolog 1 pseudogene///WAS protein family homolog 1///WAS protein family homolog 7 pseudogene///WAS protein family homolog 2 pseudogene///WAS protein family homolog 3 pseudogene
47560_at	1.36E-03	0.42919301	ADGRL1	adhesion G protein-coupled receptor L1
235369_at	1.36E-03	-0.37201355	C14orf28	chromosome 14 open reading frame 28
238383_at	1.36E-03	0.16254486	C6orf58	chromosome 6 open reading frame 58
48659_at	1.36E-03	0.19344481	MIIP	migration and invasion inhibitory protein
208009_s_at	1.37E-03	0.2432536	ARHGEF16	Rho guanine nucleotide exchange factor 16
212437_at	1.37E-03	0.33072628	CENPB	centromere protein B
229420_at	1.37E-03	-0.25367942	LOC101243545///SNORD4A	uncharacterized LOC101243545///small nucleolar RNA, C/D box 4A
221938_x_at	1.37E-03	0.2379835	MED16	mediator complex subunit 16
206205_at	1.38E-03	-0.36902566	MPHOSPH9	M-phase phosphoprotein 9
214221_at	1.39E-03	-0.34551276	ALMS1	ALMS1, centrosome and basal body associated protein
231996_at	1.39E-03	-0.16726967	N4BP2	NEDD4 binding protein 2
208502_s_at	1.39E-03	0.30249199	PITX1	paired like homeodomain 1
228725_x_at	1.39E-03	0.28989267	PRMT2	protein arginine methyltransferase 2
210051_at	1.39E-03	0.17941953	RAPGEF3	Rap guanine nucleotide exchange factor 3
1554241_at	1.40E-03	-0.42434495	COCH	cochlin
206234_s_at	1.40E-03	0.14551841	MMP17	matrix metallopeptidase 17
32402_s_at	1.40E-03	0.15210587	SYMPK	symplekin

1553039_a_at	1.41E-03	0.12374619	ASB10	ankyrin repeat and SOCS box containing 10
1553993_s_at	1.41E-03	0.33040006	MIR6800///MED25	microRNA 6800///mediator complex subunit 25
1570529_at	1.41E-03	0.10146951	PUM3	pumilio RNA binding family member 3
202081_at	1.42E-03	0.36747193	IER2	immediate early response 2
205077_s_at	1.42E-03	-0.21394875	PIGF	phosphatidylinositol glycan anchor biosynthesis class F
219751_at	1.42E-03	-0.35997967	SETD6	SET domain containing 6
214638_s_at	1.43E-03	0.10088074	CCNT2	cyclin T2
212716_s_at	1.43E-03	0.30430097	EIF3K	eukaryotic translation initiation factor 3 subunit K
206223_at	1.43E-03	0.10856732	LMTK2	lemur tyrosine kinase 2
208874_x_at	1.43E-03	0.18848411	PTPA	protein phosphatase 2 phosphatase activator
225832_s_at	1.44E-03	0.2623968	DAGLB	diacylglycerol lipase beta
228852_at	1.44E-03	0.28088389	ENSA	endosulfine alpha
201673_s_at	1.45E-03	0.28281137	GYS1	glycogen synthase 1
211101_x_at	1.45E-03	0.11535233	LILRA2	leukocyte immunoglobulin like receptor A2
204899_s_at	1.46E-03	0.16197657	SAP30	Sin3A associated protein 30
221047_s_at	1.47E-03	-0.52388499	MARK1	microtubule affinity regulating kinase 1
203777_s_at	1.47E-03	0.21673468	RPS6KB2	ribosomal protein S6 kinase B2
209381_x_at	1.47E-03	0.24828682	SF3A2	splicing factor 3a subunit 2
203147_s_at	1.47E-03	0.25489438	TRIM14	tripartite motif containing 14
206405_x_at	1.47E-03	-0.25003227	USP32///USP6	ubiquitin specific peptidase 32///ubiquitin specific peptidase 6
218522_s_at	1.48E-03	0.27965038	MAP1S	microtubule associated protein 1S
215400_x_at	1.49E-03	-0.10042484	ADCY9	adenylate cyclase 9
207629_s_at	1.49E-03	0.2065293	ARHGEF2	Rho/Rac guanine nucleotide exchange factor 2
217817_at	1.49E-03	0.20762665	ARPC4	actin related protein 2/3 complex subunit 4
1564207_at	1.49E-03	0.22286727	LINC00957	long intergenic non-protein coding RNA 957
225708_at	1.49E-03	0.25422249	MED29	mediator complex subunit 29
210797_s_at	1.49E-03	0.20788602	OASL	2'-5'-oligoadenylate synthetase like
233140_s_at	1.49E-03	0.15892136	VIPAS39	VPS33B interacting protein, apical-basolateral polarity regulator, spe-39 homolog
222362_at	1.50E-03	0.21575284	AGFG2	ArfGAP with FG repeats 2
220438_at	1.50E-03	0.17078845	QPCTL	glutaminyl-peptide cyclotransferase like
227521_at	1.51E-03	-0.24763291	FBXO33	F-box protein 33
233804_at	1.51E-03	-0.08602732	LINC00544	long intergenic non-protein coding RNA 544
221566_s_at	1.51E-03	0.28240484	NOL3	nucleolar protein 3
208890_s_at	1.51E-03	0.29302558	PLXNB2	plexin B2
214041_x_at	1.51E-03	-0.41869042	RPL37A	ribosomal protein L37a
201863_at	1.52E-03	0.23163738	FAM32A	family with sequence similarity 32 member A
1559045_at	1.52E-03	-0.11437003	LOC100128288	uncharacterized LOC100128288
224817_at	1.52E-03	0.27116518	SH3PXD2A	SH3 and PX domains 2A
237019_at	1.52E-03	-0.14977462		
237020_at	1.53E-03	-0.08954042	CATSPERD	cation channel sperm associated auxiliary subunit delta
206309_at	1.53E-03	-0.11086206	LECT1	leukocyte cell derived chemotaxin 1
234339_s_at	1.53E-03	0.28635616	SNORD23///GLTSCR2	small nucleolar RNA, C/D box 23///glioma tumor suppressor candidate region gene 2
209083_at	1.54E-03	0.47362929	CORO1A	coronin 1A
235012_at	1.54E-03	0.23245491	LRCH1	leucine rich repeats and calponin homology domain containing 1
221373_x_at	1.54E-03	0.1417167	PSPN	persephin
224439_x_at	1.54E-03	0.26122035	RNF7	ring finger protein 7
221972_s_at	1.54E-03	0.42534244	SDF4	stromal cell derived factor 4
208887_at	1.55E-03	0.33161757	EIF3G	eukaryotic translation initiation factor 3 subunit G
237444_at	1.55E-03	-0.29930412	KIF13A	kinesin family member 13A
208938_at	1.55E-03	0.30941378	PRCC	papillary renal cell carcinoma (translocation-associated)
225546_at	1.56E-03	0.30405833	LOC101930123///EEF2K	eukaryotic elongation factor 2 kinase///eukaryotic elongation factor 2 kinase
244714_at	1.56E-03	-0.09513998		
214728_x_at	1.57E-03	0.33467816	SMARCA4	SWI/SNF related, matrix associated, actin dependent regulator of chromatin, subfamily a, member 4
200972_at	1.57E-03	0.35703064	TSPAN3	tetraspanin 3
238258_at	1.57E-03	0.11071634	WBSCR28	Williams-Beuren syndrome chromosome region 28
224500_s_at	1.58E-03	0.22012045	MON1A	MON1 homolog A, secretory trafficking associated
223748_at	1.58E-03	0.80636201	SLC4A11	solute carrier family 4 member 11

203849_s_at	1.59E-03	0.09939355	KIF1A	kinesin family member 1A
207169_x_at	1.59E-03	0.28117688	MIR4640///DDR1	microRNA 4640///discoidin domain receptor tyrosine kinase 1
218000_s_at	1.59E-03	0.23000586	PHLDA1	pleckstrin homology like domain family A member 1
221063_x_at	1.59E-03	0.22451333	RNF123	ring finger protein 123
44669_at	1.59E-03	0.20148997	SDHAF1	succinate dehydrogenase complex assembly factor 1
213882_at	1.59E-03	-0.33304601	TM2D1	TM2 domain containing 1
212073_at	1.60E-03	0.40288763	CSNK2A1	casein kinase 2 alpha 1
1563821_at	1.60E-03	-0.24646488	LINC00858	long intergenic non-protein coding RNA 858
221189_s_at	1.60E-03	0.19702735	MIR6878///TARS2	microRNA 6878///threonyl-tRNA synthetase 2, mitochondrial (putative)
212433_x_at	1.60E-03	0.1947511	SNORA64///RPS2	small nucleolar RNA, H/ACA box 64///ribosomal protein S2
214763_at	1.61E-03	0.22108041	ACOT11	acyl-CoA thioesterase 11
230213_at	1.61E-03	0.21423093	C19orf43	chromosome 19 open reading frame 43
218003_s_at	1.61E-03	-0.30283575	FKBP3	FK506 binding protein 3
222482_at	1.61E-03	0.31549522	SSBP3	single stranded DNA binding protein 3
226168_at	1.61E-03	0.22750637	ZFAND2B	zinc finger AN1-type containing 2B
212169_at	1.62E-03	0.29096734	FKBP9	FK506 binding protein 9
229027_at	1.62E-03	-0.35386975	PPM1A	protein phosphatase, Mg2+/Mn2+ dependent 1A
226088_at	1.62E-03	0.2286753	ZDHHC12	zinc finger DHHC-type containing 12
209270_at	1.63E-03	0.4204867	LAMB3	laminin subunit beta 3
218376_s_at	1.64E-03	0.31508934	MICAL1	microtubule associated monooxygenase, calponin and LIM domain containing 1
210972_x_at	1.64E-03	-0.15463236	TRAC///TRAJ17///TRAV20///TRDV2	T-cell receptor alpha constant///T cell receptor alpha joining 17///T cell receptor alpha variable 20///T cell receptor delta variable 2
201082_s_at	1.65E-03	0.33679918	SLC4A5///DCTN1	solute carrier family 4 member 5///dynactin subunit 1
231069_at	1.65E-03	-0.40813108		
225833_at	1.66E-03	0.25201371	DAGLB	diacylglycerol lipase beta
1555900_at	1.66E-03	0.37487418	DCTN5	dynactin subunit 5
201378_s_at	1.66E-03	0.3081474	UBAP2L	ubiquitin associated protein 2 like
234001_s_at	1.67E-03	0.26931438	ARFGAP1	ADP ribosylation factor GTPase activating protein 1
228297_at	1.67E-03	-0.45575266	CNN3	calponin 3
204398_s_at	1.67E-03	0.28062898	EML2	echinoderm microtubule associated protein like 2
1554987_at	1.67E-03	-0.134167	GOLGA3	golgin A3
203235_at	1.67E-03	0.33765018	THOP1	thimet oligopeptidase 1
241927_x_at	1.68E-03	0.10662729	CDC34	cell division cycle 34
240718_at	1.68E-03	-0.0678134	LRMP	lymphoid restricted membrane protein
235690_at	1.68E-03	-0.27238272	ZNF594	zinc finger protein 594
243121_x_at	1.68E-03	0.18674401		
210418_s_at	1.69E-03	0.46816919	IDH3B	isocitrate dehydrogenase 3 (NAD(+)) beta
240346_at	1.69E-03	0.09165538	LOC102724030	uncharacterized LOC102724030
223577_x_at	1.69E-03	-0.37877585	MALAT1	metastasis associated lung adenocarcinoma transcript 1 (non-protein coding)
204139_x_at	1.69E-03	0.22312718	MZF1	myeloid zinc finger 1
221789_x_at	1.69E-03	0.21005034	RHOT2	ras homolog family member T2
204579_at	1.70E-03	0.42627316	FGFR4	fibroblast growth factor receptor 4
228071_at	1.70E-03	-0.56395601	GIMAP7	GTPase, IMAP family member 7
228965_s_at	1.70E-03	-0.08868503	PANK2	pantothenate kinase 2
229901_at	1.70E-03	0.18503431	ZNF488	zinc finger protein 488
226846_at	1.71E-03	0.11410237	PHYHD1	phytanoyl-CoA dioxygenase domain containing 1
61874_at	1.72E-03	0.14818842	CACFD1	calcium channel flower domain containing 1
218159_at	1.72E-03	0.38679399	DDRGK1	DDRGK domain containing 1
204804_at	1.72E-03	0.36459561	TRIM21	tripartite motif containing 21
210014_x_at	1.73E-03	0.4511563	IDH3B	isocitrate dehydrogenase 3 (NAD(+)) beta
209035_at	1.73E-03	0.51521911	MDK	midkine (neurite growth-promoting factor 2)
201287_s_at	1.73E-03	0.26684289	SDC1	syndecan 1
229670_at	1.73E-03	-0.30472758		
209779_at	1.74E-03	-0.1031589	LLPH	LLP homolog, long-term synaptic facilitation
1557094_at	1.74E-03	0.14957706	LOC100996760	uncharacterized LOC100996760
233560_x_at	1.74E-03	0.11498672	MCM8	minichromosome maintenance 8 homologous recombination repair factor

230457_at	1.74E-03	-0.18356373	TMEM242	transmembrane protein 242
223747_x_at	1.74E-03	-0.13683233	WWOX	WW domain containing oxidoreductase
217133_x_at	1.75E-03	0.1151487	CYP2B6	cytochrome P450 family 2 subfamily B member 6
225025_at	1.75E-03	0.14844441	IGSF8	immunoglobulin superfamily member 8
202856_s_at	1.75E-03	0.55808465	MIR6787///SLC16A3	microRNA 6787///solute carrier family 16 member 3
201875_s_at	1.75E-03	0.28128758	MPZL1	myelin protein zero like 1
224976_at	1.75E-03	0.34068699	NFIA	nuclear factor I A
220262_s_at	1.76E-03	0.12702611	DLK2	delta like non-canonical Notch ligand 2
203154_s_at	1.76E-03	0.19833563	PAK4	p21 (RAC1) activated kinase 4
1563943_at	1.76E-03	0.11804704	PPARGC1B	PPARG coactivator 1 beta
233125_at	1.76E-03	0.08907474		
218115_at	1.77E-03	0.28837947	ASF1B	anti-silencing function 1B histone chaperone
220511_s_at	1.77E-03	0.10563403	DLC1	DLC1 Rho GTPase activating protein
236502_at	1.77E-03	-0.26584459		
1553565_s_at	1.78E-03	0.10073379	DDAH1	dimethylarginine dimethylaminohydrolase 1
215979_s_at	1.78E-03	-0.09388795	SLC7A1	solute carrier family 7 member 1
1553021_s_at	1.79E-03	0.09210428	BICD2	BICD cargo adaptor 2
1568796_at	1.79E-03	0.1405706	CCDC157	coiled-coil domain containing 157
222644_s_at	1.79E-03	0.2134595	COLGALT1	collagen beta(1-O)galactosyltransferase 1
1556670_at	1.79E-03	0.1262906	GTF3C2-AS1	GTF3C2 antisense RNA 1
238720_at	1.79E-03	0.08343595	LOC101927057///OMG	uncharacterized LOC101927057///oligodendrocyte myelin glycoprotein
237338_at	1.80E-03	0.29596299	B3GNT8	UDP-GlcNAc:betaGal beta-1,3-N-acetylglucosaminyltransferase 8
1567080_s_at	1.80E-03	0.32521916	CLN6	ceroid-lipofuscinosis, neuronal 6, late infantile, variant
214623_at	1.80E-03	0.18655785	FBXW4P1	F-box and WD repeat domain containing 4 pseudogene 1
1561301_at	1.80E-03	0.11894898	MAGI1-IT1	MAGI1 intronic transcript 1
206317_s_at	1.81E-03	0.14727883	ABCB8	ATP binding cassette subfamily B member 8
218608_at	1.81E-03	0.32511535	ATP13A2	ATPase 13A2
203531_at	1.81E-03	-0.27704999	CUL5	cullin 5
1560371_at	1.81E-03	0.2016571	LINC00997	long intergenic non-protein coding RNA 997
241534_at	1.81E-03	-0.23216455	LOC100505549	uncharacterized LOC100505549
225346_at	1.81E-03	-0.29974944	MTERF2	mitochondrial transcription termination factor 2
216383_at	1.81E-03	0.24170902		
209113_s_at	1.82E-03	0.3228643	HMG20B	high mobility group 20B
212138_at	1.82E-03	-0.23357711	PDS5A	PDS5 cohesin associated factor A
218336_at	1.82E-03	0.25144598	PFDN2	prefoldin subunit 2
221551_x_at	1.82E-03	0.26880091	ST6GALNAC4	ST6 N-acetylgalactosaminide alpha-2,6-sialyltransferase 4
235698_at	1.82E-03	-0.36185394	ZFP90	ZFP90 zinc finger protein
203826_s_at	1.83E-03	0.21123111	PITPNM1	phosphatidylinositol transfer protein membrane associated 1
223332_x_at	1.83E-03	0.24748981	RNF126	ring finger protein 126
200973_s_at	1.83E-03	0.47065085	TSPAN3	tetraspanin 3
231506_at	1.83E-03	0.07531142		
232788_at	1.83E-03	-0.25410321		
241006_at	1.83E-03	-0.11340201		
237513_at	1.84E-03	0.18751013	PRSS58	protease, serine 58
1555888_at	1.84E-03	-0.4342543	UBR5-AS1	UBR5 antisense RNA 1
214909_s_at	1.85E-03	0.38488907	DDAH2	dimethylarginine dimethylaminohydrolase 2
207660_at	1.86E-03	-0.16797652	DMD	dystrophin
233571_x_at	1.86E-03	0.3471987	PPDPF	pancreatic progenitor cell differentiation and proliferation factor
242983_at	1.86E-03	-0.16107558		
212862_at	1.87E-03	0.33795566	CDS2	CDP-diacylglycerol synthase 2
205455_at	1.87E-03	0.38333936	MST1R	macrophage stimulating 1 receptor
211882_x_at	1.88E-03	0.31428142	FUT6	fucosyltransferase 6
223326_s_at	1.88E-03	0.26913604	GOLGA2P10	golgin A2 pseudogene 10
209044_x_at	1.89E-03	0.21173804	SF3B4	splicing factor 3b subunit 4
226506_at	1.89E-03	0.2210636	THSD4	thrombospondin type 1 domain containing 4
239966_at	1.89E-03	-0.13861308		
217569_x_at	1.90E-03	-0.12373425		

224742_at	1.91E-03	0.31269472	ABHD12	abhydrolase domain containing 12
240986_at	1.91E-03	0.08417498	LOC101928943	uncharacterized LOC101928943
218725_at	1.91E-03	0.21361208	SLC25A22	solute carrier family 25 member 22
241950_at	1.91E-03	0.12694764	WWC1	WW and C2 domain containing 1
40273_at	1.92E-03	0.33298847	SPHK2	sphingosine kinase 2
211065_x_at	1.93E-03	0.27541458	PFKL	phosphofructokinase, liver type
214991_s_at	1.93E-03	0.19678376	PIGO	phosphatidylinositol glycan anchor biosynthesis class O
200652_at	1.93E-03	0.26618221	SSR2	signal sequence receptor subunit 2
225218_at	1.93E-03	0.20079952	ZFYVE27	zinc finger FYVE-type containing 27
1560999_a_at	1.93E-03	-0.09605278		
211934_x_at	1.94E-03	0.28466079	GANAB	glucosidase II alpha subunit
218302_at	1.94E-03	0.35458883	PSENEN	presenilin enhancer gamma-secretase subunit
228612_at	1.94E-03	-0.39406687	RAB30-AS1	RAB30 antisense RNA 1 (head to head)
203107_x_at	1.94E-03	0.12195674	SNORA64///RPS2	small nucleolar RNA, H/ACA box 64///ribosomal protein S2
201605_x_at	1.95E-03	0.22576697	CNN2	calponin 2
244593_at	1.95E-03	0.11319263	HID1	HID1 domain containing
219972_s_at	1.95E-03	-0.3169148	PCNX4	pecanex homolog 4 (Drosophila)
209671_x_at	1.95E-03	-0.13937946	TRAC	T-cell receptor alpha constant
216938_x_at	1.96E-03	0.14283119	DRD2	dopamine receptor D2
216128_at	1.96E-03	0.10929845	LOC101929597///TBCD	uncharacterized LOC101929597///tubulin folding cofactor D
200758_s_at	1.96E-03	0.2263824	NFE2L1	nuclear factor, erythroid 2 like 1
218466_at	1.96E-03	0.13407884	TBC1D17	TBC1 domain family member 17
232675_s_at	1.97E-03	0.18585927	UCKL1	uridine-cytidine kinase 1 like 1
214679_x_at	1.98E-03	0.24334096	GNA11	G protein subunit alpha 11
233335_at	1.98E-03	-0.08899093	ITGA11	integrin subunit alpha 11
234686_at	1.98E-03	0.08106106	SUGT1P1	SGT1 homolog, MIS12 kinetochore complex assembly cochaperone pseudogene 1
207831_x_at	1.99E-03	0.30989042	DHPS	deoxyhypusine synthase
212484_at	1.99E-03	0.21705899	FAM89B	family with sequence similarity 89 member B
236324_at	1.99E-03	0.1085423	MBP	myelin basic protein
214979_at	1.99E-03	0.10721918		
202263_at	2.00E-03	0.3165184	CYB5R1	cytochrome b5 reductase 1
214678_x_at	2.00E-03	-0.27703019	ZFX	zinc finger protein, X-linked
243302_at	2.00E-03	-0.36372521		
204447_at	2.01E-03	0.47100269	LZTS3	leucine zipper tumor suppressor family member 3
38269_at	2.01E-03	0.24180046	PRKD2	protein kinase D2
208732_at	2.02E-03	-0.31713395	RAB2A	RAB2A, member RAS oncogene family
218813_s_at	2.02E-03	0.28793062	SH3GLB2	SH3 domain containing GRB2 like endophilin B2
228582_x_at	2.03E-03	-0.53742498	MALAT1	metastasis associated lung adenocarcinoma transcript 1 (non-protein coding)
210627_s_at	2.03E-03	0.31783755	MOGS	mannosyl-oligosaccharide glucosidase
225377_at	2.03E-03	0.27942754	RABL6	RAB, member RAS oncogene family-like 6
240561_at	2.03E-03	-0.13026403	RAP2C-AS1	RAP2C antisense RNA 1
235876_at	2.03E-03	-0.1723711	TMEM218	transmembrane protein 218
241435_at	2.03E-03	-0.34904834		
223000_s_at	2.05E-03	0.20811356	F11R	F11 receptor
210264_at	2.05E-03	0.36525511	GPR35	G protein-coupled receptor 35
229317_at	2.06E-03	-0.43565535	KPNA5	karyopherin subunit alpha 5
231732_at	2.06E-03	0.18504052	SMPD3	sphingomyelin phosphodiesterase 3
212855_at	2.07E-03	-0.31626871	DCUN1D4	defective in cullin neddylation 1 domain containing 4
216850_at	2.07E-03	-0.09216615	LOC101930404///SNORD116-28///SNORD115-26///SNORD115-13///SNORD115-7///SNORD116-22///SNORD116-4///PWARSN///SNORD107///SNURF///SNRPN///IPW	uncharacterized LOC101930404///small nucleolar RNA, C/D box 116-28///small nucleolar RNA, C/D box 115-26///small nucleolar RNA, C/D box 115-13///small nucleolar RNA, C/D box 115-7///small nucleolar RNA, C/D box 116-22///small nucleolar RNA, C/D box 116-4///Prader Willi/Angelman region RNA, SNRPN neighbor///small nucleolar RNA, C/D box 107///SNRPN upstream reading frame///small nuclear ribonucleoprotein polypeptide N///imprinted in Prader-Willi syndrome (non-protein coding)
226053_at	2.07E-03	0.16144022	MAP2K7	mitogen-activated protein kinase kinase 7

203565_s_at	2.07E-03	-0.30487498	MNAT1	MNAT1, CDK activating kinase assembly factor
203782_s_at	2.07E-03	0.37497515	POLRMT	RNA polymerase mitochondrial
235762_at	2.07E-03	-0.39828809	TAS2R14	taste 2 receptor member 14
242475_at	2.07E-03	0.14137358		
202079_s_at	2.08E-03	0.27016382	TRAK1	trafficking kinesin protein 1
218965_s_at	2.08E-03	0.14689486	TUT1	terminal uridylyl transferase 1, U6 snRNA-specific
228244_at	2.09E-03	0.22695883	BLOC1S3	biogenesis of lysosomal organelles complex 1 subunit 3
201583_s_at	2.09E-03	0.48559379	SEC23B	Sec23 homolog B, coat complex II component
210811_s_at	2.10E-03	0.23706368	DDX49	DEAD-box helicase 49
222995_s_at	2.10E-03	0.4135294	RHBDD2	rhomboid domain containing 2
215860_at	2.10E-03	-0.11684594	SYT12	synaptotagmin 12
240085_at	2.10E-03	0.10688561		
221818_at	2.11E-03	0.18562574	INTS5	integrator complex subunit 5
204791_at	2.11E-03	-0.28278349	NR2C1	nuclear receptor subfamily 2 group C member 1
210475_at	2.11E-03	-0.13533708	POU3F1	POU class 3 homeobox 1
217529_at	2.12E-03	0.21121828	ORAI2	ORAI calcium release-activated calcium modulator 2
202093_s_at	2.12E-03	0.25717045	PAF1	PAF1 homolog, Paf1/RNA polymerase II complex component
202075_s_at	2.12E-03	0.64168443	PLTP	phospholipid transfer protein
226898_s_at	2.12E-03	-0.12088559	SFPQ	splicing factor proline and glutamine rich
229618_at	2.12E-03	-0.42643607	SNX16	sorting nexin 16
232842_at	2.13E-03	-0.13426532	DOCK8	dedicator of cytokinesis 8
237339_at	2.13E-03	-0.09027499	LINC00993	long intergenic non-protein coding RNA 993
1553715_s_at	2.13E-03	0.35942568	MCRIP2	MAPK regulated corepressor interacting protein 2
222175_s_at	2.13E-03	0.2638701	MED15	mediator complex subunit 15
200077_s_at	2.14E-03	0.17258777	OAZ1	ornithine decarboxylase antizyme 1
AFFX-M27830_5_at	2.14E-03	0.32990786		
226849_at	2.15E-03	0.22620689	DENND1A	DENN domain containing 1A
223950_s_at	2.15E-03	0.22226703	FLYWCH1	FLYWCH-type zinc finger 1
200885_at	2.15E-03	0.39256698	RHOC	ras homolog family member C
227625_s_at	2.15E-03	0.28673062	STUB1	STIP1 homology and U-box containing protein 1
1563560_at	2.16E-03	0.11726681	AHNAK	AHNAK nucleoprotein
209280_at	2.16E-03	0.10359794	MRC2	mannose receptor C type 2
204598_at	2.16E-03	0.17319264	UBOX5	U-box domain containing 5
208684_at	2.17E-03	0.26423099	COPA	coatomer protein complex subunit alpha
212861_at	2.17E-03	0.3097043	MFSD5	major facilitator superfamily domain containing 5
210362_x_at	2.17E-03	0.1294113	PML	promyelocytic leukemia
1563473_at	2.17E-03	-0.15565741		
225861_at	2.18E-03	0.3544522	MCRIP2	MAPK regulated corepressor interacting protein 2
236873_at	2.18E-03	0.21077276		
226584_s_at	2.19E-03	0.29595384	FAM110A	family with sequence similarity 110 member A
231055_at	2.19E-03	0.31023643		
1568617_a_at	2.20E-03	0.27508347	CAMSAP3	calmodulin regulated spectrin associated protein family member 3
219404_at	2.20E-03	0.28350146	EPS8L3	EPS8 like 3
208750_s_at	2.20E-03	0.36950735	MIR3620///ARF1	microRNA 3620///ADP ribosylation factor 1
212739_s_at	2.20E-03	0.38128449	NME4	NME/NM23 nucleoside diphosphate kinase 4
218180_s_at	2.21E-03	0.28298186	EPS8L2	EPS8 like 2
242711_x_at	2.21E-03	-0.29253006	FANCM	Fanconi anemia complementation group M
228658_at	2.21E-03	-0.20742416	MIAT	myocardial infarction associated transcript (non-protein coding)
209367_at	2.21E-03	0.25720062	STXBP2	syntaxin binding protein 2
217325_at	2.22E-03	-0.12175183	KRT3	keratin 3
212680_x_at	2.22E-03	0.30878568	PPP1R14B	protein phosphatase 1 regulatory inhibitor subunit 14B
38892_at	2.23E-03	-0.2630407	GLTSCR1L	GLTSCR1 like
226328_at	2.23E-03	0.18236283	KLF16	Kruppel like factor 16
222656_at	2.23E-03	-0.367162	UBE2W	ubiquitin conjugating enzyme E2 W (putative)
237849_at	2.23E-03	-0.30079138		
202367_at	2.24E-03	0.25216738	CUX1	cut like homeobox 1
203705_s_at	2.24E-03	-0.71245799	FZD7	frizzled class receptor 7
81737_at	2.24E-03	-0.13944316	LOC100505915	uncharacterized LOC100505915
202005_at	2.24E-03	0.2984199	ST14	suppression of tumorigenicity 14
238548_at	2.25E-03	0.13633403	PANK2	pantothenate kinase 2

65086_at	2.25E-03	0.18445116	YIPF2	Yip1 domain family member 2
201482_at	2.26E-03	0.46675027	QSOX1	quiescin sulfhydryl oxidase 1
1557382_x_at	2.27E-03	0.11947345	AGAP11	ArfGAP with GTPase domain, ankyrin repeat and PH domain 11
1557836_at	2.27E-03	0.14548769	ELMOD2	ELMO domain containing 2
1560115_a_at	2.27E-03	0.14366081	KIAA1217	KIAA1217
221790_s_at	2.27E-03	0.28697675	LDLRAP1	low density lipoprotein receptor adaptor protein 1
204442_x_at	2.27E-03	0.32071128	LTBP4	latent transforming growth factor beta binding protein 4
221269_s_at	2.27E-03	0.41048401	SH3BGRL3	SH3 domain binding glutamate rich protein like 3
212495_at	2.28E-03	0.17430618	KDM4B	lysine demethylase 4B
224231_at	2.28E-03	0.06793719	PRO0471	uncharacterized LOC28994
1564949_at	2.28E-03	0.08650611		
1556131_s_at	2.29E-03	-0.13023035	FBF1	Fas binding factor 1
204660_at	2.29E-03	-0.11273973	GFER	growth factor, augmenter of liver regeneration
228242_at	2.29E-03	-0.41068996	N4BP2	NEDD4 binding protein 2
227257_s_at	2.30E-03	0.22227222	CACUL1	CDK2 associated cullin domain 1
237805_at	2.30E-03	0.1154665	LOC729296	uncharacterized LOC729296
236874_at	2.30E-03	-0.10435233		
1559478_at	2.31E-03	0.10802866		
212950_at	2.32E-03	-0.44074219	ADGRF5	adhesion G protein-coupled receptor F5
221655_x_at	2.32E-03	0.23428306	EPS8L1	EPS8 like 1
224037_at	2.32E-03	-0.25357699	SDAD1	SDA1 domain containing 1
218672_at	2.32E-03	0.19901619	TNFAIP8L2-SCNM1///SCNM1	TNFAIP8L2-SCNM1 readthrough///sodium channel modifier 1
215952_s_at	2.33E-03	0.33667761	OAZ1	ornithine decarboxylase antizyme 1
203189_s_at	2.34E-03	0.40081512	MIR7113///MIR4691///NDUFS8	microRNA 7113///microRNA 4691///NADH:ubiquinone oxidoreductase core subunit S8
221450_x_at	2.34E-03	0.13948668	PCDHB13	protocadherin beta 13
202758_s_at	2.34E-03	0.24728379	RFXANK	regulatory factor X associated ankyrin containing protein
213888_s_at	2.34E-03	-0.4374146	TRAF3IP3	TRAF3 interacting protein 3
1570065_at	2.35E-03	-0.11460232	C22orf34	chromosome 22 open reading frame 34
229287_at	2.35E-03	-0.29912989	PCNX1	pecanex homolog 1 (Drosophila)
201251_at	2.35E-03	0.41891671	PKM	pyruvate kinase, muscle
211819_s_at	2.35E-03	0.13235859	SORBS1	sorbin and SH3 domain containing 1
232964_at	2.35E-03	-0.25943881	SPDYE2B///SPDYE6///SPDYE5///SPDYE2///SPDYE1	speedy/RINGO cell cycle regulator family member E2B///speedy/RINGO cell cycle regulator family member E6///speedy/RINGO cell cycle regulator family member E5///speedy/RINGO cell cycle regulator family member E2///speedy/RINGO cell cycle regulator family member E1
217223_s_at	2.36E-03	0.2572896	BCR	BCR, RhoGEF and GTPase activating protein
65884_at	2.36E-03	0.25760682	MAN1B1	mannosidase alpha class 1B member 1
1568666_at	2.36E-03	0.10377159	PLIN5	perilipin 5
200675_at	2.37E-03	0.32722152	CD81	CD81 molecule
213907_at	2.37E-03	-0.27462048	EEF1E1	eukaryotic translation elongation factor 1 epsilon 1
214709_s_at	2.37E-03	-0.2296795	KTN1	kinectin 1
220156_at	2.38E-03	0.11543492	EFCAB1	EF-hand calcium binding domain 1
1563035_x_at	2.38E-03	0.09086617	GPD1	glycerol-3-phosphate dehydrogenase 1
210982_s_at	2.38E-03	-0.73483812	HLA-DRA	major histocompatibility complex, class II, DR alpha
211534_x_at	2.38E-03	0.13692663	PTPRN2	protein tyrosine phosphatase, receptor type N2
221028_s_at	2.39E-03	0.19314796	GFOD2	glucose-fructose oxidoreductase domain containing 2
213173_at	2.39E-03	-0.20768208	PCNX1	pecanex homolog 1 (Drosophila)
226564_at	2.39E-03	0.1254097	ZFAT	zinc finger and AT-hook domain containing
217079_at	2.39E-03	-0.0970815		
200852_x_at	2.40E-03	0.3480287	GNB2	G protein subunit beta 2
224472_x_at	2.40E-03	0.28654575	SDF4	stromal cell derived factor 4
230383_x_at	2.40E-03	-0.46894153	SLFN5	schlafen family member 5
206280_at	2.41E-03	-0.1043937	CDH18	cadherin 18
210029_at	2.41E-03	-0.79249266	IDO1	indoleamine 2,3-dioxygenase 1
227924_at	2.41E-03	-0.10661006	INO80D	INO80 complex subunit D
219039_at	2.41E-03	0.2180893	SEMA4C	semaphorin 4C

209129_at	2.41E-03	0.64488547	TRIP6	thyroid hormone receptor interactor 6
229630_s_at	2.41E-03	-0.19844917	WTAP	Wilms tumor 1 associated protein
225531_at	2.42E-03	0.19455847	CABLES1	Cdk5 and Abl enzyme substrate 1
200785_s_at	2.42E-03	0.24579562	LRP1	LDL receptor related protein 1
209740_s_at	2.42E-03	-0.50906137	PNPLA4	patatin like phospholipase domain containing 4
220127_s_at	2.43E-03	0.1939172	FBXL12	F-box and leucine rich repeat protein 12
236597_at	2.43E-03	-0.07791673	UGT3A1	UDP glycosyltransferase family 3 member A1
1555221_at	2.44E-03	0.08308068	LOC101927122	uncharacterized LOC101927122
1555851_s_at	2.44E-03	0.38078433	SEPW1	selenoprotein W, 1
239838_at	2.44E-03	-0.08921575	ZNF776	zinc finger protein 776
221419_s_at	2.44E-03	-0.15529352		
212411_at	2.45E-03	0.29805474	IMP4	IMP4 homolog, U3 small nucleolar ribonucleoprotein
210357_s_at	2.45E-03	0.38657131	SMOX	spermine oxidase
212032_s_at	2.46E-03	0.36561645	PTOV1	prostate tumor overexpressed 1
32699_s_at	2.46E-03	0.10234644	PVR	poliovirus receptor
1560921_at	2.46E-03	0.13163244	ZNF169	zinc finger protein 169
213642_at	2.46E-03	-0.27285148		
1554837_a_at	2.47E-03	0.1156414	CYP4A22///CYP4A11	cytochrome P450 family 4 subfamily A member 22///cytochrome P450 family 4 subfamily A member 11
1557944_s_at	2.47E-03	0.15066331	TMX2-CTNND1///CTNND1	TMX2-CTNND1 readthrough (NMD candidate)///catenin delta 1
224385_s_at	2.48E-03	0.08539926	MOV10L1	Mov10 RISC complex RNA helicase like 1
234725_s_at	2.48E-03	0.36770417	SEMA4B	semaphorin 4B
214752_x_at	2.49E-03	0.27317053	FLNA	filamin A
219796_s_at	2.50E-03	0.38404882	CDHR5	cadherin related family member 5
219052_at	2.50E-03	0.15012859	HPS6	HPS6, biogenesis of lysosomal organelles complex 2 subunit 3
215566_x_at	2.50E-03	0.2338999	LYPLA2	lysophospholipase II
225449_at	2.50E-03	0.14046803	RDH13	retinol dehydrogenase 13
79005_at	2.50E-03	0.11606843	SLC35E1	solute carrier family 35 member E1
221679_s_at	2.52E-03	0.21844664	ABHD6	abhydrolase domain containing 6
207884_at	2.52E-03	-0.09761998	GUCY2D	guanylate cyclase 2D, retinal
202781_s_at	2.52E-03	0.20165684	INPP5K	inositol polyphosphate-5-phosphatase K
201755_at	2.52E-03	0.34077931	MCM5	minichromosome maintenance complex component 5
217765_at	2.52E-03	0.20250721	NRBP1	nuclear receptor binding protein 1
241618_at	2.52E-03	-0.17247864		
235522_at	2.53E-03	-0.19070171	CLEC2D	C-type lectin domain family 2 member D
46142_at	2.53E-03	0.26693716	LMF1	lipase maturation factor 1
200709_at	2.53E-03	0.3700556	LOC101929368///FKBP1A	uncharacterized LOC101929368///FK506 binding protein 1A
1560981_a_at	2.55E-03	0.15776528	PPARA	peroxisome proliferator activated receptor alpha
214951_at	2.55E-03	0.10176375	SLC26A10	solute carrier family 26 member 10
208428_at	2.55E-03	0.274794	TAP2	transporter 2, ATP binding cassette subfamily B member
239561_at	2.55E-03	-0.33143074		
205384_at	2.56E-03	0.11875399	FXYD1	FXYD domain containing ion transport regulator 1
202891_at	2.56E-03	0.21338948	NIT1	nitrilase 1
232834_at	2.56E-03	0.18015889		
34689_at	2.57E-03	0.17155878	ATRIP///TREX1	ATR interacting protein///three prime repair exonuclease 1
228774_at	2.57E-03	-0.27007882	CEP78	centrosomal protein 78
53968_at	2.57E-03	0.28452431	INTS5	integrator complex subunit 5
228387_at	2.58E-03	-0.37699854	LINC01355	long intergenic non-protein coding RNA 1355
236691_at	2.58E-03	-0.12949038		
208932_at	2.59E-03	0.27770482	PPP4C	protein phosphatase 4 catalytic subunit
210463_x_at	2.59E-03	0.33121807	TRMT1	tRNA methyltransferase 1
212265_at	2.60E-03	-0.575776	QKI	QKI, KH domain containing RNA binding
200916_at	2.60E-03	0.32918913	TAGLN2	transgelin 2
219146_at	2.60E-03	-0.30795471	TEFM	transcription elongation factor, mitochondrial
203839_s_at	2.60E-03	0.22465473	TNK2	tyrosine kinase non receptor 2
207428_x_at	2.61E-03	0.25041118	CDK11A///CDK11B	cyclin dependent kinase 11A///cyclin dependent kinase 11B
218161_s_at	2.61E-03	0.26514452	CLN6	ceroid-lipofuscinosis, neuronal 6, late infantile, variant
202802_at	2.61E-03	0.33008632	DHPS	deoxyhypusine synthase

225630_at	2.61E-03	0.33105672	EEPD1	endonuclease/exonuclease/phosphatase family domain containing 1
1562631_at	2.61E-03	-0.13183463	TEX26-AS1	TEX26 antisense RNA 1
202629_at	2.62E-03	-0.26729887	APPBP2	amyloid beta precursor protein binding protein 2
204495_s_at	2.62E-03	0.24221833	C15orf39	chromosome 15 open reading frame 39
34210_at	2.62E-03	-0.61523835	CD52	CD52 molecule
214224_s_at	2.62E-03	-0.2658095	PIN4	peptidylprolyl cis/trans isomerase, NIMA-interacting 4
209476_at	2.62E-03	-0.27378207	TMX1	thioredoxin related transmembrane protein 1
227466_at	2.63E-03	-0.255549	FAM200B	family with sequence similarity 200 member B
213063_at	2.63E-03	-0.29546015	ZC3H14	zinc finger CCCH-type containing 14
240653_at	2.63E-03	-0.08852701		
227152_at	2.64E-03	-0.427633	KIAA1551	KIAA1551
200772_x_at	2.65E-03	0.2399278	LOC100506248///MIR1244-2///MIR1244-3///MIR1244-1///LOC728026///PTMA	prothymosin alpha///microRNA 1244-2///microRNA 1244-3///microRNA 1244-1///prothymosin alpha-like///prothymosin, alpha
212929_s_at	2.65E-03	-0.19600211	LOC101930591///FAM21A///FAM21C	uncharacterized LOC101930591///family with sequence similarity 21 member A///family with sequence similarity 21 member C
204535_s_at	2.65E-03	-0.19449568	REST	RE1 silencing transcription factor
234194_at	2.65E-03	-0.06865881		
210380_s_at	2.66E-03	0.08098298	CACNA1G	calcium voltage-gated channel subunit alpha1 G
227820_at	2.66E-03	-0.14410668	TBC1D25	TBC1 domain family member 25
205932_s_at	2.67E-03	-0.71256421	MSX1	msh homeobox 1
204480_s_at	2.68E-03	0.41391367	C9orf16	chromosome 9 open reading frame 16
237651_x_at	2.68E-03	-0.0671075	LINC00518	long intergenic non-protein coding RNA 518
237294_at	2.68E-03	-0.11117288	RASSF4	Ras association domain family member 4
204986_s_at	2.68E-03	0.15429448	TAOK2	TAO kinase 2
243152_at	2.69E-03	0.1010815	COPS8	COP9 signalosome subunit 8
216606_x_at	2.69E-03	0.19075313	LYPLA2	lysophospholipase II
1563077_at	2.70E-03	0.1011717	LOC100289058	uncharacterized LOC100289058
237203_at	2.70E-03	0.08275455	LOC101929305	uncharacterized LOC101929305
223040_at	2.70E-03	0.39447565	NAA20	N(alpha)-acetyltransferase 20, NatB catalytic subunit
214125_s_at	2.70E-03	-0.14280823	NENF	neudesin neurotrophic factor
210845_s_at	2.70E-03	0.45806607	PLAUR	plasminogen activator, urokinase receptor
203459_s_at	2.70E-03	0.33954794	VPS16	VPS16, CORVET/HOPS core subunit
206994_at	2.71E-03	0.32060788	CST4	cystatin S
200703_at	2.71E-03	0.27263522	DYNLL1	dynein light chain LC8-type 1
201749_at	2.71E-03	0.21302929	ECE1	endothelin converting enzyme 1
231735_s_at	2.71E-03	-0.41823673	MALAT1	metastasis associated lung adenocarcinoma transcript 1 (non-protein coding)
218773_s_at	2.71E-03	0.4063252	MSRB2	methionine sulfoxide reductase B2
36907_at	2.72E-03	0.09201418	MVK	mevalonate kinase
233500_x_at	2.73E-03	-0.17791409	CLEC2D	C-type lectin domain family 2 member D
217872_at	2.73E-03	0.25307565	PIH1D1	PIH1 domain containing 1
207408_at	2.74E-03	0.13081858	SLC22A14	solute carrier family 22 member 14
225394_s_at	2.74E-03	-0.22770939	ZCRB1	zinc finger CCHC-type and RNA binding motif containing 1
243694_at	2.74E-03	-0.11069711		
206005_s_at	2.75E-03	-0.24152638	CEP162	centrosomal protein 162
210116_at	2.75E-03	-0.31575286	SH2D1A	SH2 domain containing 1A
223958_s_at	2.76E-03	-0.19086071	DNAL1	dynein axonemal light chain 1
215417_at	2.76E-03	-0.1792705	EXOC6B	exocyst complex component 6B
230265_at	2.76E-03	-0.40547864		
35974_at	2.77E-03	-0.479246	LRMP	lymphoid restricted membrane protein
224394_at	2.77E-03	0.17301263	RNF7	ring finger protein 7
204226_at	2.77E-03	-0.32023016	STAU2	staufen double-stranded RNA binding protein 2
221939_at	2.77E-03	0.13151897	YIPF2	Yip1 domain family member 2
216737_at	2.77E-03	-0.06811863		
225471_s_at	2.78E-03	0.16705937	AKT2	AKT serine/threonine kinase 2
238152_at	2.78E-03	0.12220146	C11orf95	chromosome 11 open reading frame 95
200076_s_at	2.78E-03	0.13460356	KXD1	KxDL motif containing 1
209166_s_at	2.78E-03	0.31619478	MAN2B1	mannosidase alpha class 2B member 1
230052_s_at	2.78E-03	0.16958615	NFKBID	NFKB inhibitor delta

210010_s_at	2.78E-03	0.3780873	SLC25A1	solute carrier family 25 member 1
229262_at	2.79E-03	0.12896751	PPP1R37	protein phosphatase 1 regulatory subunit 37
1566441_at	2.79E-03	0.07218204		
231412_at	2.81E-03	-0.34810614	LOC101929709	uncharacterized LOC101929709
208793_x_at	2.81E-03	0.23740428	SMARCA4	SWI/SNF related, matrix associated, actin dependent regulator of chromatin, subfamily a, member 4
221512_at	2.81E-03	0.26702401	TMEM222	transmembrane protein 222
210434_x_at	2.82E-03	0.15282899	JTB	jumping translocation breakpoint
201332_s_at	2.82E-03	0.15402587	STAT6	signal transducer and activator of transcription 6
222212_s_at	2.83E-03	0.30029074	CERS2	ceramide synthase 2
215498_s_at	2.83E-03	0.36633006	LOC100996792///MAP2K3	dual specificity mitogen-activated protein kinase kinase 3///mitogen-activated protein kinase kinase 3
201246_s_at	2.83E-03	0.21910945	LOC101927673///OTUB1	uncharacterized LOC101927673///OTU deubiquitinase, ubiquitin aldehyde binding 1
202180_s_at	2.83E-03	0.32996791	MVP	major vault protein
223649_s_at	2.83E-03	0.39325151	SLC25A39	solute carrier family 25 member 39
201511_at	2.84E-03	0.25425999	AAMP	angio associated migratory cell protein
208050_s_at	2.84E-03	0.1880624	CASP2	caspase 2
226207_at	2.84E-03	-0.23828706	RILPL1	Rab interacting lysosomal protein like 1
1561134_at	2.84E-03	-0.09923249		
225437_s_at	2.85E-03	0.23649566	BRAT1	BRCA1 associated ATM activator 1
203118_at	2.85E-03	0.29774826	PCSK7	proprotein convertase subtilisin/kexin type 7
206582_s_at	2.86E-03	0.12265081	ADGRG1	adhesion G protein-coupled receptor G1
215723_s_at	2.86E-03	0.3452958	PLD1	phospholipase D1
207490_at	2.86E-03	0.12838834	TUBA4B	tubulin alpha 4b
228864_at	2.86E-03	0.15470001	ZNF653	zinc finger protein 653
235905_at	2.86E-03	-0.27388991	ZNF704	zinc finger protein 704
202361_at	2.89E-03	0.28577385	SEC24C	SEC24 homolog C, COPII coat complex component
237195_at	2.89E-03	-0.16991671		
206411_s_at	2.90E-03	0.11020532	ABL2	ABL proto-oncogene 2, non-receptor tyrosine kinase
202014_at	2.90E-03	0.29798404	PPP1R15A	protein phosphatase 1 regulatory subunit 15A
224395_s_at	2.90E-03	0.25047098	RNF7	ring finger protein 7
1552623_at	2.91E-03	0.1750806	HSH2D	hematopoietic SH2 domain containing
225418_at	2.92E-03	0.32094848	NECTIN2	nectin cell adhesion molecule 2
201276_at	2.93E-03	0.22189225	RAB5B	RAB5B, member RAS oncogene family
203085_s_at	2.94E-03	0.16294629	TGFB1	transforming growth factor beta 1
243405_at	2.94E-03	-0.32725516		
207722_s_at	2.95E-03	0.18840481	BTBD2	BTB domain containing 2
213720_s_at	2.96E-03	0.25983789	SMARCA4	SWI/SNF related, matrix associated, actin dependent regulator of chromatin, subfamily a, member 4
203237_s_at	2.97E-03	0.11919883	NOTCH3	notch 3
212559_at	2.97E-03	0.17597758	PRKAR1B	protein kinase cAMP-dependent type I regulatory subunit beta
202024_at	2.98E-03	0.30176413	ASNA1	arsA arsenite transporter, ATP-binding, homolog 1 (bacterial)
237623_at	2.98E-03	0.37876433	CST3	cystatin C
52005_at	2.98E-03	0.17099885	WIZ	widely interspaced zinc finger motifs
203658_at	2.99E-03	0.31791082	SLC25A20	solute carrier family 25 member 20
215644_at	2.99E-03	-0.10711058	ZNF518A	zinc finger protein 518A
1564263_at	3.00E-03	0.11963253	LINC00330	long intergenic non-protein coding RNA 330
218199_s_at	3.00E-03	0.2219242	NOL6	nucleolar protein 6
211049_at	3.00E-03	0.11608111	TLX2	T-cell leukemia homeobox 2
212090_at	3.01E-03	0.2669648	GRINA	glutamate ionotropic receptor NMDA type subunit associated protein 1
218988_at	3.01E-03	-0.22644027	SLC35E3	solute carrier family 35 member E3
220421_at	3.03E-03	0.49271973	BTNL8	butyrophilin like 8
200784_s_at	3.03E-03	0.1733942	LRP1	LDL receptor related protein 1
223999_at	3.03E-03	-0.21733728	PPIL2	peptidylprolyl isomerase like 2
209158_s_at	3.04E-03	0.30153423	CYTH2	cytohesin 2
221856_s_at	3.04E-03	0.24973245	FAM63A	family with sequence similarity 63 member A
216483_s_at	3.05E-03	0.34710517	MYDGF	myeloid derived growth factor
208675_s_at	3.06E-03	0.3223922	DDOST	dolichyl-diphosphooligosaccharide--protein glycosyltransferase non-catalytic subunit

1553997_a_at	3.07E-03	0.28284192	ASPHD1	aspartate beta-hydroxylase domain containing 1
234356_at	3.07E-03	0.09864552	DKFZP434K028	uncharacterized LOC26070
204231_s_at	3.07E-03	0.31078334	FAAH	fatty acid amide hydrolase
228522_at	3.07E-03	0.1314212	FBRSL1	fibrosin like 1
204764_at	3.08E-03	0.13192195	CHURC1-FNTB///FNTB	CHURC1-FNTB readthrough///farnesyltransferase, CAAX box, beta
1559194_a_at	3.08E-03	0.09722203	CLEC4GP1	C-type lectin domain family 4 member G pseudogene 1
213466_at	3.08E-03	0.13247428	RAB40C	RAB40C, member RAS oncogene family
202264_s_at	3.08E-03	0.21155355	TOMM40	translocase of outer mitochondrial membrane 40
204241_at	3.09E-03	-0.28653568	ACOX3	acyl-CoA oxidase 3, pristanoyl
206707_x_at	3.09E-03	-0.18583821	FAM65B	family with sequence similarity 65 member B
1556553_at	3.09E-03	-0.0943064		
212552_at	3.10E-03	0.25108931	HPCAL1	hippocalcin like 1
201283_s_at	3.10E-03	0.12917395	TRAK1	trafficking kinesin protein 1
210501_x_at	3.11E-03	0.26261578	EIF3K	eukaryotic translation initiation factor 3 subunit K
234017_at	3.11E-03	-0.09759035	LINC00923	long intergenic non-protein coding RNA 923
212119_at	3.13E-03	-0.26534364	RHOQ	ras homolog family member Q
1568843_at	3.13E-03	0.10917305	TTLL13P	tubulin tyrosine ligase like 13, pseudogene
1438_at	3.14E-03	0.44998728	EPHB3	EPH receptor B3
233565_s_at	3.14E-03	0.45909021	FKBP1A-SDCBP2///SDCBP2	FKBP1A-SDCBP2 readthrough (NMD candidate)///syndecan binding protein 2
234106_s_at	3.14E-03	0.14642044	FLYWCH1	FLYWCH-type zinc finger 1
242379_at	3.14E-03	-0.16464596		
209931_s_at	3.15E-03	0.16685906	MFSD2B///FKBP1B	major facilitator superfamily domain containing 2B///FK506 binding protein 1B
218745_x_at	3.15E-03	0.20081552	TMEM161A	transmembrane protein 161A
213705_at	3.16E-03	-0.30829371	GGCX	gamma-glutamyl carboxylase
225337_at	3.17E-03	0.47051535	ABHD2	abhydrolase domain containing 2
209606_at	3.17E-03	-0.57538639	CYTIP	cytohesin 1 interacting protein
226076_s_at	3.17E-03	0.23004869	MBD6	methyl-CpG binding domain protein 6
229599_at	3.17E-03	0.27114615	SMIM22	small integral membrane protein 22
209195_s_at	3.18E-03	0.2177026	ADCY6	adenylate cyclase 6
201168_x_at	3.18E-03	0.20957456	ARHGDIA	Rho GDP dissociation inhibitor alpha
231926_at	3.18E-03	0.27351224	EPS15L1	epidermal growth factor receptor pathway substrate 15 like 1
201508_at	3.19E-03	0.33776661	IGFBP4	insulin like growth factor binding protein 4
229759_s_at	3.19E-03	0.11021763	VEPH1	ventricular zone expressed PH domain containing 1
243883_at	3.20E-03	0.12684056	MMP15	matrix metallopeptidase 15
210474_s_at	3.21E-03	0.27466505	CDK11A///CDK11B	cyclin dependent kinase 11A///cyclin dependent kinase 11B
202245_at	3.22E-03	0.37201577	LSS	lanosterol synthase (2,3-oxidosqualene-lanosterol cyclase)
202740_at	3.23E-03	0.38540936	ABHD14A-ACY1///ACY1	ABHD14A-ACY1 readthrough///aminoacylase 1
227409_at	3.23E-03	-0.32800592	PPP1R3E	protein phosphatase 1 regulatory subunit 3E
222778_s_at	3.23E-03	-0.30629961	WHSC1	Wolf-Hirschhorn syndrome candidate 1
222126_at	3.24E-03	0.26796507	AGFG2	ArfGAP with FG repeats 2
223814_at	3.24E-03	-0.32375203	TRNT1	tRNA nucleotidyl transferase 1
224807_at	3.25E-03	0.269366	GRAMD1A	GRAM domain containing 1A
205698_s_at	3.25E-03	-0.44805184	MAP2K6	mitogen-activated protein kinase kinase 6
216105_x_at	3.25E-03	0.14890294	PTPA	protein phosphatase 2 phosphatase activator
215831_at	3.25E-03	-0.09415416		
211433_x_at	3.26E-03	0.199526	FAM214B	family with sequence similarity 214 member B
242855_at	3.26E-03	0.13448772	KCP	kielin/chordin-like protein
213049_at	3.28E-03	-0.2963952	RALGAPA1	Ral GTPase activating protein catalytic alpha subunit 1
207714_s_at	3.28E-03	0.42403921	SERPINH1	serpin family H member 1
1560149_at	3.28E-03	0.10602789	SLC29A2	solute carrier family 29 member 2
229629_at	3.28E-03	-0.31783856		
226943_at	3.29E-03	-0.34028123	C12orf73	chromosome 12 open reading frame 73
204433_s_at	3.29E-03	0.30340562	SPATA2	spermatogenesis associated 2
205225_at	3.30E-03	-0.14558456	ESR1	estrogen receptor 1
1553361_x_at	3.30E-03	0.11027905	FBXL18	F-box and leucine rich repeat protein 18
202167_s_at	3.30E-03	0.2808412	MMS19	MMS19 homolog, cytosolic iron-sulfur assembly component

225921_at	3.30E-03	-0.40012476	NIN	ninein
222238_s_at	3.30E-03	0.22406519	POLM	DNA polymerase mu
203781_at	3.31E-03	-0.1974334	MRPL33	mitochondrial ribosomal protein L33
218058_at	3.32E-03	0.27473161	CXXC1	CXXC finger protein 1
200060_s_at	3.33E-03	0.17579526	RNPS1	RNA binding protein with serine rich domain 1
233151_s_at	3.33E-03	0.14226125	TTTY7B///TTTY7	testis-specific transcript, Y-linked 7B (non-protein coding)///testis-specific transcript, Y-linked 7 (non-protein coding)
213073_at	3.33E-03	-0.22821411	ZFYVE26	zinc finger FYVE-type containing 26
238683_at	3.33E-03	0.13054951	ZNF524	zinc finger protein 524
214225_at	3.36E-03	-0.31776687	PIN4	peptidylprolyl cis/trans isomerase, NIMA-interacting 4
228637_at	3.36E-03	0.22613238	ZDHHC1	zinc finger DHHC-type containing 1
1569250_at	3.36E-03	0.10136398	ZNF333	zinc finger protein 333
211141_s_at	3.37E-03	0.0812888	CNOT3	CCR4-NOT transcription complex subunit 3
200915_x_at	3.37E-03	-0.19892765	KTN1	kinectin 1
221758_at	3.38E-03	0.15849319	ARMC6	armadillo repeat containing 6
221545_x_at	3.38E-03	0.24672315	MED16	mediator complex subunit 16
223032_x_at	3.38E-03	0.36058309	PRELID1	PRELI domain containing 1
206949_s_at	3.38E-03	0.23942037	RUSC1	RUN and SH3 domain containing 1
214804_at	3.39E-03	-0.35967001	CENPI	centromere protein I
200048_s_at	3.39E-03	0.16528888	JTB	jumping translocation breakpoint
217014_s_at	3.40E-03	0.45659391	AZGP1P1///AZGP1	alpha-2-glycoprotein 1, zinc-binding pseudogene 1///alpha-2-glycoprotein 1, zinc-binding
200621_at	3.40E-03	0.27620191	CSRP1	cysteine and glycine rich protein 1
1553530_a_at	3.41E-03	0.26735663	ITGB1	integrin subunit beta 1
204981_at	3.41E-03	0.38927525	SLC22A18	solute carrier family 22 member 18
212491_s_at	3.42E-03	-0.23266954	DNAJC8	DnaJ heat shock protein family (Hsp40) member C8
229312_s_at	3.42E-03	-0.25994585	GKAP1	G kinase anchoring protein 1
203685_at	3.43E-03	-0.60954008	BCL2	BCL2, apoptosis regulator
1570470_at	3.43E-03	0.10410915	CATSPERB	cation channel sperm associated auxiliary subunit beta
212682_s_at	3.43E-03	0.15834466	LMF2	lipase maturation factor 2
210775_x_at	3.44E-03	0.1661367	CASP9	caspase 9
210990_s_at	3.44E-03	0.10060144	LAMA4	laminin subunit alpha 4
228550_at	3.44E-03	0.17146251	RTN4R	reticulon 4 receptor
212212_s_at	3.45E-03	0.20061977	INTS1	integrator complex subunit 1
230282_at	3.45E-03	0.16304429	TSPAN3	tetraspanin 3
239351_at	3.46E-03	-0.10760607	FKBP3	FK506 binding protein 3
206459_s_at	3.46E-03	-0.08660164	WNT2B	Wnt family member 2B
1861_at	3.47E-03	0.26530756	BAD	BCL2 associated agonist of cell death
1568600_at	3.47E-03	0.31904246	CALML4	calmodulin like 4
211012_s_at	3.47E-03	0.20742641	PML	promyelocytic leukemia
238331_at	3.47E-03	0.12319489	SPRN	shadow of prion protein homolog (zebrafish)
220917_s_at	3.47E-03	-0.3423581	WDR19	WD repeat domain 19
227892_at	3.48E-03	-0.8564519	PRKAA2	protein kinase AMP-activated catalytic subunit alpha 2
226683_at	3.48E-03	-0.26483298	SNX18	sorting nexin 18
208019_at	3.48E-03	-0.06801355	ZNF157	zinc finger protein 157
204275_at	3.49E-03	0.28884548	CAPN15	calpain 15
208534_s_at	3.49E-03	0.19174042	RASA4B///RASA4CP///RASA4	RAS p21 protein activator 4B///RAS p21 protein activator 4C, pseudogene///RAS p21 protein activator 4
1569106_s_at	3.49E-03	0.13116775	SETD5	SET domain containing 5
226135_at	3.49E-03	-0.20676575	UHRF1BP1	UHRF1 binding protein 1
241537_at	3.49E-03	-0.07318293		
222165_x_at	3.50E-03	0.23020041	C9orf16	chromosome 9 open reading frame 16
221335_x_at	3.50E-03	0.18645719	SMG9	SMG9, nonsense mediated mRNA decay factor
224913_s_at	3.50E-03	0.40190863	TIMM50	translocase of inner mitochondrial membrane 50
1557757_at	3.50E-03	0.10514843		
222246_at	3.50E-03	-0.07323827		
212015_x_at	3.51E-03	0.20402929	MIR4745///PTBP1	microRNA 4745///polypyrimidine tract binding protein 1
231369_at	3.51E-03	-0.19912955	ZNF333	zinc finger protein 333
205831_at	3.52E-03	-0.37748438	CD2	CD2 molecule
230343_at	3.53E-03	0.5215021	CST3	cystatin C
222978_at	3.53E-03	0.2467298	SURF4	surfeit 4
205660_at	3.54E-03	0.4018055	OASL	2'-5'-oligoadenylate synthetase like

204164_at	3.54E-03	0.18234569	SIPA1	signal-induced proliferation-associated 1
226807_at	3.54E-03	-0.27049057	ZFP1	ZFP1 zinc finger protein
221519_at	3.55E-03	0.1979571	FBXW4	F-box and WD repeat domain containing 4
1569986_x_at	3.55E-03	0.10172492	TNNT3	troponin T3, fast skeletal type
219363_s_at	3.56E-03	-0.35263939	MTERF3	mitochondrial transcription termination factor 3
244730_x_at	3.56E-03	-0.09130282	ZSCAN16-AS1	ZSCAN16 antisense RNA 1
212072_s_at	3.57E-03	0.36482861	CSNK2A1	casein kinase 2 alpha 1
212512_s_at	3.58E-03	0.22543081	CARM1	coactivator associated arginine methyltransferase 1
203751_x_at	3.59E-03	0.11676611	JUND	JunD proto-oncogene, AP-1 transcription factor subunit
1558211_s_at	3.59E-03	0.09748229	SRC	SRC proto-oncogene, non-receptor tyrosine kinase
212663_at	3.62E-03	0.157159	FKBP15	FK506 binding protein 15
231901_at	3.63E-03	0.23205451	C19orf52	chromosome 19 open reading frame 52
209864_at	3.63E-03	0.2268728	FRAT2	frequently rearranged in advanced T-cell lymphomas 2
203766_s_at	3.63E-03	0.15721659	LMOD1	leiomodin 1
206666_at	3.64E-03	-0.38794834	GZMK	granzyme K
1558675_s_at	3.64E-03	-0.26294414	NEMF	nuclear export mediator factor
241814_at	3.64E-03	-0.11336982		
229063_s_at	3.65E-03	0.19923574	CCDC107	coiled-coil domain containing 107
208975_s_at	3.65E-03	-0.23911994	KPNB1	karyopherin subunit beta 1
210336_x_at	3.65E-03	0.18064057	MZF1	myeloid zinc finger 1
202261_at	3.65E-03	0.25856387	VPS72	vacuolar protein sorting 72 homolog
234255_at	3.65E-03	-0.21799045		
227850_x_at	3.66E-03	0.56550218	CDC42EP5	CDC42 effector protein 5
225150_s_at	3.66E-03	0.31839521	RTKN	rhotekin
222143_s_at	3.69E-03	0.17862697	MTMR14	myotubularin related protein 14
219194_at	3.69E-03	0.32771866	SEMA4G	semaphorin 4G
228229_at	3.70E-03	0.20163819	ZNF526	zinc finger protein 526
205377_s_at	3.72E-03	0.13648857	ACHE	acetylcholinesterase (Cartwright blood group)
228426_at	3.73E-03	-0.08953067	CLEC2D	C-type lectin domain family 2 member D
209943_at	3.73E-03	-0.29882626	FBXL4	F-box and leucine rich repeat protein 4
224923_at	3.73E-03	0.13579026	TTC7A	tetratricopeptide repeat domain 7A
216484_x_at	3.73E-03	0.18899305		
209668_x_at	3.74E-03	0.60718473	CES2	carboxylesterase 2
206501_x_at	3.74E-03	0.13074342	ETV1	ETS variant 1
1562424_at	3.74E-03	0.09614966	LOC285889	uncharacterized LOC285889
216503_s_at	3.74E-03	0.24615996	MLLT10	myeloid/lymphoid or mixed-lineage leukemia; translocated to, 10
223293_at	3.74E-03	0.3049209	WDR24	WD repeat domain 24
244061_at	3.74E-03	-0.31922837		
222051_s_at	3.75E-03	-0.11166365	E2F5	E2F transcription factor 5
219724_s_at	3.75E-03	-0.07410845	TESPA1	thymocyte expressed, positive selection associated 1
200721_s_at	3.76E-03	0.22206203	ACTR1A	ARP1 actin-related protein 1 homolog A, centractin alpha
1554333_at	3.76E-03	0.14967448	DNAJA4	DnaJ heat shock protein family (Hsp40) member A4
208223_s_at	3.77E-03	0.14396439	ACVR1B	activin A receptor type 1B
226810_at	3.77E-03	-0.6955867	OGFRL1	opioid growth factor receptor like 1
220964_s_at	3.77E-03	0.23787594	RAB1B	RAB1B, member RAS oncogene family
226120_at	3.77E-03	-0.27236704	TTC8	tetratricopeptide repeat domain 8
231682_at	3.77E-03	-0.10018574		
236012_at	3.78E-03	0.30385213	PSMF1	proteasome inhibitor subunit 1
244194_at	3.79E-03	-0.26964174	ADAM22	ADAM metallopeptidase domain 22
226651_at	3.79E-03	-0.42095515	HOMER1	homer scaffolding protein 1
239534_at	3.79E-03	-0.18147274		
233384_at	3.79E-03	0.097867		
217117_x_at	3.80E-03	0.14749639	MUC3B///MUC3A	mucin 3B, cell surface associated///mucin 3A, cell surface associated
207435_s_at	3.80E-03	0.19403297	SRRM2	serine/arginine repetitive matrix 2
242941_x_at	3.80E-03	0.15280805	TBX1	T-box 1
224451_x_at	3.82E-03	-0.30516674	ARHGAP9	Rho GTPase activating protein 9
229876_at	3.82E-03	-0.51745	PHKA1	phosphorylase kinase regulatory subunit alpha 1
221295_at	3.83E-03	-0.11542436	CIDEA	cell death-inducing DFFA-like effector a
207116_s_at	3.83E-03	0.11929374	GAPDHS	glyceraldehyde-3-phosphate dehydrogenase, spermatogenic

218612_s_at	3.83E-03	0.21597047	TSSC4	tumor suppressing subtransferable candidate 4
1568938_at	3.84E-03	0.0771373	LINC01398	long intergenic non-protein coding RNA 1398
219134_at	3.85E-03	-0.42574413	ADGRL4	adhesion G protein-coupled receptor L4
201883_s_at	3.85E-03	0.2005733	B4GALT1	beta-1,4-galactosyltransferase 1
1556421_at	3.85E-03	-0.08179959	C8orf34-AS1	C8orf34 antisense RNA 1
216969_s_at	3.85E-03	0.16818873	KIF22	kinesin family member 22
229371_at	3.85E-03	-0.37479495	SLC20A2	solute carrier family 20 member 2
218077_s_at	3.85E-03	0.24142482	ZDHHC3	zinc finger DHHC-type containing 3
202756_s_at	3.86E-03	0.38372891	GPC1	glypican 1
234086_at	3.87E-03	-0.09065472		
212075_s_at	3.88E-03	0.37499656	CSNK2A1	casein kinase 2 alpha 1
204904_at	3.88E-03	-0.14056336	GJA4	gap junction protein alpha 4
564_at	3.88E-03	0.21770164	GNA11	G protein subunit alpha 11
208894_at	3.88E-03	-0.75673226	HLA-DRA	major histocompatibility complex, class II, DR alpha
207740_s_at	3.88E-03	0.24014951	NUP62	nucleoporin 62
1566577_at	3.88E-03	0.08137028		
1555407_s_at	3.89E-03	0.10991973	FGD3	FYVE, RhoGEF and PH domain containing 3
239846_at	3.90E-03	-0.14993211	MTHFD1	methylenetetrahydrofolate dehydrogenase, cyclohydrolase and formyltetrahydrofolate synthetase 1
216385_at	3.93E-03	0.13378245	LOC220077	dedicator of cytokinesis 1 pseudogene
233660_at	3.94E-03	0.14657908	EHD4	EH domain containing 4
221291_at	3.94E-03	0.15392821	ULBP2	UL16 binding protein 2
208657_s_at	3.95E-03	0.22166058	SEPT9	septin 9
223045_at	3.95E-03	0.09685025	EGLN1	egl-9 family hypoxia inducible factor 1
217202_s_at	3.95E-03	0.44293156	GLUL	glutamate-ammonia ligase
201201_at	3.96E-03	0.36978219	CSTB	cystatin B
222546_s_at	3.96E-03	0.13635916	EPS8L2	EPS8 like 2
204110_at	3.96E-03	-0.19050494	HNMT	histamine N-methyltransferase
239237_at	3.96E-03	-0.24757071	TRG-AS1	T cell receptor gamma locus antisense RNA 1
224114_at	3.96E-03	0.07481616		
1559863_a_at	3.97E-03	0.11368003	AFG3L1P	AFG3 like matrix AAA peptidase subunit 1, pseudogene
212150_at	3.97E-03	-0.25724959	EFR3A	EFR3 homolog A
230848_s_at	3.97E-03	-0.34176057	MGA	MGA, MAX dimerization protein
220289_s_at	3.98E-03	0.38171928	AIM1L	absent in melanoma 1-like
228769_at	3.98E-03	0.16943792	ZSCAN22	zinc finger and SCAN domain containing 22
1568488_at	3.98E-03	-0.07759415		
210100_s_at	3.99E-03	0.11341561	ABCA2	ATP binding cassette subfamily A member 2
213514_s_at	3.99E-03	0.15355713	DIAPH1	diaphanous related formin 1
224920_x_at	3.99E-03	0.22290489	MYADM	myeloid associated differentiation marker
233779_x_at	4.00E-03	-0.09081226		
200896_x_at	4.01E-03	0.19595889	HDGF	hepatoma-derived growth factor
202747_s_at	4.01E-03	-0.30410986	ITM2A	integral membrane protein 2A
229953_x_at	4.01E-03	-0.19208502	LCA5	LCA5, lebercilin
218370_s_at	4.01E-03	-0.21381232	S100PBP	S100P binding protein
233096_at	4.02E-03	-0.10013468	KIAA1109	KIAA1109
229511_at	4.02E-03	-0.55875347	SMARCE1	SWI/SNF related, matrix associated, actin dependent regulator of chromatin, subfamily e, member 1
213977_s_at	4.04E-03	0.22899768	CIZ1	CDKN1A interacting zinc finger protein 1
35160_at	4.04E-03	0.17507458	LDB1	LIM domain binding 1
218010_x_at	4.05E-03	0.27050599	PPDPF	pancreatic progenitor cell differentiation and proliferation factor
242189_at	4.05E-03	-0.19982886		
1556127_at	4.06E-03	0.13605522	DIP2A	disco interacting protein 2 homolog A
226448_at	4.06E-03	0.33996611	MIR1182///FAM89A	microRNA 1182///family with sequence similarity 89 member A
200824_at	4.07E-03	0.33242242	GSTP1	glutathione S-transferase pi 1
209208_at	4.08E-03	0.33349634	MPDU1	mannose-P-dolichol utilization defect 1
1552641_s_at	4.10E-03	0.22784651	ATAD3B///ATAD3A	ATPase family, AAA domain containing 3B///ATPase family, AAA domain containing 3A
208590_x_at	4.10E-03	0.11265044	GJA3	gap junction protein alpha 3
208917_x_at	4.10E-03	0.13559832	NADK	NAD kinase
221704_s_at	4.10E-03	0.32798115	VPS37B	VPS37B, ESCRT-I subunit
226691_at	4.11E-03	0.24822922	TNRC18	trinucleotide repeat containing 18

224347_x_at	4.11E-03	0.17354622	UBE2J2	ubiquitin conjugating enzyme E2 J2
210364_at	4.12E-03	-0.12434416	SCN2B	sodium voltage-gated channel beta subunit 2
200923_at	4.13E-03	0.44397328	LGALS3BP	galectin 3 binding protein
210008_s_at	4.13E-03	0.15425462	MRPS12	mitochondrial ribosomal protein S12
213922_at	4.13E-03	-0.32170505	TTBK2	tau tubulin kinase 2
243983_at	4.13E-03	0.11259339		
225384_at	4.14E-03	-0.2477087	DOCK7	dedicator of cytokinesis 7
223342_at	4.14E-03	-0.34902275	RRM2B	ribonucleotide reductase regulatory TP53 inducible subunit M2B
201459_at	4.14E-03	0.31676038	RUVBL2	RuvB like AAA ATPase 2
223105_s_at	4.14E-03	-0.15587264	TMEM14B///TMEM14C	transmembrane protein 14B///transmembrane protein 14C
211816_x_at	4.15E-03	0.09391341	FCAR	Fc fragment of IgA receptor
214326_x_at	4.15E-03	0.12624491	JUND	JunD proto-oncogene, AP-1 transcription factor subunit
1567068_at	4.15E-03	0.10644331	OR4D1	olfactory receptor family 4 subfamily D member 1
1552272_a_at	4.15E-03	0.1240606	PRR22	proline rich 22
217857_s_at	4.15E-03	0.26682877	RBM8A	RNA binding motif protein 8A
1558972_s_at	4.15E-03	-0.37974831	THEMIS	thymocyte selection associated
204576_s_at	4.16E-03	-0.20794501	CLUAP1	clusterin associated protein 1
213059_at	4.16E-03	0.42964599	CREB3L1	cAMP responsive element binding protein 3 like 1
232037_at	4.16E-03	0.15389462	IGDCC3	immunoglobulin superfamily DCC subclass member 3
202122_s_at	4.16E-03	0.27649169	PLIN3	perilipin 3
226600_at	4.16E-03	-0.33617895	TMTC3	transmembrane and tetratricopeptide repeat containing 3
239067_s_at	4.19E-03	0.09460556	PANX2	pannexin 2
202407_s_at	4.19E-03	0.27180966	PRPF31	pre-mRNA processing factor 31
219847_at	4.21E-03	0.10782543	HDAC11	histone deacetylase 11
219408_at	4.21E-03	0.29190774	PRMT7	protein arginine methyltransferase 7
227592_at	4.22E-03	0.27000844	ALDH16A1	aldehyde dehydrogenase 16 family member A1
222951_s_at	4.22E-03	0.20009472	ANKEF1	ankyrin repeat and EF-hand domain containing 1
210966_x_at	4.22E-03	0.22792732	LARP1	La ribonucleoprotein domain family member 1
208928_at	4.22E-03	0.33740117	POR	cytochrome p450 oxidoreductase
1554334_a_at	4.23E-03	0.38138718	DNAJA4	DnaJ heat shock protein family (Hsp40) member A4
232581_x_at	4.23E-03	0.09782999	HIVEP3	human immunodeficiency virus type I enhancer binding protein 3
236627_at	4.23E-03	-0.15680204	LOC101927085	uncharacterized LOC101927085
243366_s_at	4.24E-03	-0.40504736	ITGA4	integrin subunit alpha 4
231232_at	4.24E-03	-0.32886703	LOC100506125	uncharacterized LOC100506125
1559240_at	4.24E-03	-0.19795699	LOC100507053	uncharacterized LOC100507053
202736_s_at	4.24E-03	0.31299081	LSM4	LSM4 homolog, U6 small nuclear RNA and mRNA degradation associated
205267_at	4.24E-03	-0.8155242	POU2AF1	POU class 2 associating factor 1
237759_at	4.25E-03	-0.16142883	CD48	CD48 molecule
229887_at	4.26E-03	0.14985065	ALS2CL	ALS2 C-terminal like
1564308_a_at	4.26E-03	-0.21524806	MPP7	membrane palmitoylated protein 7
224981_at	4.26E-03	0.28369844	TMEM219	transmembrane protein 219
203027_s_at	4.27E-03	0.19109036	MVD	mevalonate diphosphate decarboxylase
225091_at	4.27E-03	0.34124332	ZCCHC3	zinc finger CCHC-type containing 3
239628_at	4.27E-03	-0.09583843		
211672_s_at	4.28E-03	0.20228645	ARPC4-TTLL3///ARPC4	ARPC4-TTLL3 readthrough///actin related protein 2/3 complex subunit 4
224253_at	4.28E-03	-0.07956626	EXOC5	exocyst complex component 5
238630_at	4.28E-03	0.16266944	OPA3	optic atrophy 3 (autosomal recessive, with chorea and spastic paraplegia)
236371_s_at	4.28E-03	-0.15850909	TGS1	trimethylguanosine synthase 1
229164_s_at	4.30E-03	0.2738368	ABTB1	ankyrin repeat and BTB domain containing 1
216590_at	4.30E-03	-0.14133057	GNAT3	G protein subunit alpha transducin 3
201797_s_at	4.30E-03	0.41691861	VARS	valyl-tRNA synthetase
1553990_at	4.31E-03	0.2152125	BRICD5	BRICHOS domain containing 5
226775_at	4.31E-03	-0.37422995	ENY2	ENY2, transcription and export complex 2 subunit
236702_at	4.31E-03	0.10791662	RTFDC1	replication termination factor 2 domain containing 1
223192_at	4.31E-03	0.19206879	SLC25A28	solute carrier family 25 member 28
208202_s_at	4.32E-03	0.12913607	JADE2	jade family PHD finger 2

204860_s_at	4.33E-03	-0.35564958	NAIP	NLR family apoptosis inhibitory protein
215836_s_at	4.33E-03	0.23098028	PCDHGA1///PCDHGA2///PCDHGA3///PCDHGA4///PCDHGA5///PCDHGA6///PCDHGA7///PCDHGA9///PCDHGA10///PCDHGA11///PCDHGB1///PCDHGB2///PCDHGB3///PCDHGB5///PCDHGB6///PCDHGB7///PCDHGC4///PCDHGC5///PCDHGA12///PCDHGA8///PCDHGB4///PCDHGC3	protocadherin gamma subfamily A, 1///protocadherin gamma subfamily A, 2///protocadherin gamma subfamily A, 3///protocadherin gamma subfamily A, 4///protocadherin gamma subfamily A, 5///protocadherin gamma subfamily A, 6///protocadherin gamma subfamily A, 7///protocadherin gamma subfamily A, 9///protocadherin gamma subfamily A, 10///protocadherin gamma subfamily A, 11///protocadherin gamma subfamily B, 1///protocadherin gamma subfamily B, 2///protocadherin gamma subfamily B, 3///protocadherin gamma subfamily B, 5///protocadherin gamma subfamily B, 6///protocadherin gamma subfamily B, 7///protocadherin gamma subfamily C, 4///protocadherin gamma subfamily C, 5///protocadherin gamma subfamily A, 12///protocadherin gamma subfamily A, 8///protocadherin gamma subfamily B, 4///protocadherin gamma subfamily C, 3
202082_s_at	4.33E-03	-0.28491503	SEC14L1	SEC14 like lipid binding 1
1561086_at	4.33E-03	-0.09100059		
214182_at	4.35E-03	-0.35934209	ARF6	ADP ribosylation factor 6
219501_at	4.35E-03	-0.14955556	ENOX1	ecto-NOX disulfide-thiol exchanger 1
206689_x_at	4.35E-03	0.18396035	KAT5	lysine acetyltransferase 5
221604_s_at	4.36E-03	0.26796823	PEX16	peroxisomal biogenesis factor 16
208880_s_at	4.36E-03	0.3878173	PRPF6	pre-mRNA processing factor 6
238371_s_at	4.36E-03	-0.22778665		
220075_s_at	4.37E-03	0.18163757	CDHR5	cadherin related family member 5
224271_x_at	4.37E-03	0.09986436	FRMD8P1	FERM domain containing 8 pseudogene 1
1569830_at	4.37E-03	-0.08624228	PTPRC	protein tyrosine phosphatase, receptor type C
1554594_at	4.38E-03	0.32565809	ARHGAP27	Rho GTPase activating protein 27
208027_s_at	4.38E-03	0.06911949	TLL2	tolloid like 2
201379_s_at	4.38E-03	0.3247514	TPD52L2	tumor protein D52 like 2
237054_at	4.39E-03	-0.53144518	ENPP5	ectonucleotide pyrophosphatase/phosphodiesterase 5 (putative)
221549_at	4.39E-03	0.14950041	GRWD1	glutamate rich WD repeat containing 1
212925_at	4.39E-03	0.27774915	MISP	mitotic spindle positioning
208699_x_at	4.39E-03	0.4173076	TKT	transketolase
206357_at	4.40E-03	0.12995845	OPA3	optic atrophy 3 (autosomal recessive, with chorea and spastic paraplegia)
225294_s_at	4.40E-03	0.28566922	TRAPPC1	trafficking protein particle complex 1
1560662_s_at	4.40E-03	-0.08980184	WHAMMP2///WHAMMP3	WAS protein homolog associated with actin, golgi membranes and microtubules pseudogene 2///WAS protein homolog associated with actin, golgi membranes and microtubules pseudogene 3
230580_at	4.40E-03	-0.17414694		
221683_s_at	4.41E-03	-0.29992577	CEP290	centrosomal protein 290
1557769_at	4.41E-03	-0.12867931	CHN2	chimerin 2
202669_s_at	4.41E-03	0.54569989	EFNB2	ephrin B2
227168_at	4.41E-03	-0.14810195	MIAT	myocardial infarction associated transcript (non-protein coding)
209212_s_at	4.42E-03	0.38841117	KLF5	Kruppel like factor 5
201282_at	4.42E-03	0.17677573	OGDH	oxoglutarate dehydrogenase
202098_s_at	4.42E-03	0.28418499	PRMT2	protein arginine methyltransferase 2
227999_at	4.42E-03	0.23159903	PWWP2B	PWWP domain containing 2B
230224_at	4.42E-03	-0.11523468	ZCCHC18	zinc finger CCHC-type containing 18
204896_s_at	4.43E-03	0.11141438	PTGER4	prostaglandin E receptor 4
238860_at	4.44E-03	-0.35453557	OARD1	O-acyl-ADP-ribose deacylase 1
206815_at	4.44E-03	0.12565151	SPAG8	sperm associated antigen 8
41397_at	4.44E-03	0.12343031	ZNF821	zinc finger protein 821

218448_at	4.45E-03	0.28120227	GID8	GID complex subunit 8 homolog
209337_at	4.45E-03	-0.30320206	PSIP1	PC4 and SFRS1 interacting protein 1
204949_at	4.49E-03	0.30094379	ICAM3	intercellular adhesion molecule 3
211230_s_at	4.49E-03	-0.15683049	PIK3CD	phosphatidylinositol-4,5-bisphosphate 3-kinase catalytic subunit delta
241807_x_at	4.49E-03	-0.09302		
240047_at	4.49E-03	0.07737511		
210011_s_at	4.50E-03	0.24463513	EWSR1	EWS RNA binding protein 1
244350_at	4.50E-03	0.31728547	MYO10	myosin X
218649_x_at	4.50E-03	-0.25848251	NEMF	nuclear export mediator factor
222137_at	4.51E-03	0.17887698	CC2D1A	coiled-coil and C2 domain containing 1A
206693_at	4.54E-03	-0.7583998	IL7	interleukin 7
235282_at	4.55E-03	-0.17829316	LOC102724532///SP2-AS1	uncharacterized LOC102724532///SP2 antisense RNA 1
206445_s_at	4.55E-03	0.33215761	PRMT1	protein arginine methyltransferase 1
203149_at	4.56E-03	0.2442307	NECTIN2	nectin cell adhesion molecule 2
239256_at	4.56E-03	0.09934062		
225562_at	4.57E-03	0.34340585	RASA3	RAS p21 protein activator 3
209499_x_at	4.57E-03	0.25635165	TNFSF12-TNFSF13///TNFSF13	TNFSF12-TNFSF13 readthrough///tumor necrosis factor superfamily member 13
241862_x_at	4.58E-03	0.19621853	C19orf45	chromosome 19 open reading frame 45
218451_at	4.58E-03	0.23643727	CDCP1	CUB domain containing protein 1
217187_at	4.58E-03	0.24565231	MUC5AC	mucin 5AC, oligomeric mucus/gel-forming
219695_at	4.58E-03	0.23008103	SMPD3	sphingomyelin phosphodiesterase 3
1570433_at	4.58E-03	0.12173994	TMPRSS2	transmembrane protease, serine 2
1554067_at	4.59E-03	-0.38532823	C12orf66	chromosome 12 open reading frame 66
223041_at	4.59E-03	0.24883088	CD99L2	CD99 molecule like 2
39549_at	4.59E-03	0.23494205	NPAS2	neuronal PAS domain protein 2
1553424_at	4.61E-03	-0.09480269	C12orf40	chromosome 12 open reading frame 40
202127_at	4.61E-03	-0.23235822	PRPF4B	pre-mRNA processing factor 4B
236808_at	4.62E-03	-0.41173886	FGFR1OP2	FGFR1 oncogene partner 2
210428_s_at	4.62E-03	0.27344049	HGS	hepatocyte growth factor-regulated tyrosine kinase substrate
230454_at	4.62E-03	-0.29333362	ICA1L	islet cell autoantigen 1 like
204171_at	4.62E-03	-0.25162616	RPS6KB1	ribosomal protein S6 kinase B1
1569431_at	4.63E-03	-0.07031293	PAFAH1B2	platelet activating factor acetylhydrolase 1b catalytic subunit 2
240072_at	4.64E-03	-0.22781064	ASXL2	additional sex combs like 2, transcriptional regulator
201172_x_at	4.64E-03	0.25038997	ATP6V0E1	ATPase H+ transporting V0 subunit e1
205488_at	4.64E-03	-0.70379319	GZMA	granzyme A
211865_s_at	4.65E-03	0.12341236	FZR1	fizzy/cell division cycle 20 related 1
221783_at	4.65E-03	0.11548978	WIZ	widely interspaced zinc finger motifs
1559566_at	4.66E-03	-0.0801573	FBXO42	F-box protein 42
234777_at	4.66E-03	0.09624299	OR51M1	olfactory receptor family 51 subfamily M member 1
224467_s_at	4.67E-03	0.33335875	PDCD2L	programmed cell death 2 like
1559911_at	4.67E-03	-0.10043455		
209435_s_at	4.68E-03	0.23506532	ARHGEF2	Rho/Rac guanine nucleotide exchange factor 2
240618_at	4.68E-03	0.10883919		
201441_at	4.69E-03	0.24654959	COX6B1	cytochrome c oxidase subunit 6B1
227344_at	4.69E-03	-0.18945872	IKZF1	IKAROS family zinc finger 1
201853_s_at	4.71E-03	0.43986769	CDC25B	cell division cycle 25B
1557278_s_at	4.71E-03	-0.22977286	TNPO1	transportin 1
203830_at	4.72E-03	-0.31192598	C17orf75	chromosome 17 open reading frame 75
218529_at	4.72E-03	0.35402129	CD320	CD320 molecule
236616_at	4.72E-03	0.35972869	PSD4	pleckstrin and Sec7 domain containing 4
241289_at	4.72E-03	-0.064313		
202410_x_at	4.73E-03	0.43464603	INS-IGF2///IGF2	INS-IGF2 readthrough///insulin like growth factor 2
238109_at	4.73E-03	0.32977539	ZNF782	zinc finger protein 782
244033_at	4.74E-03	-0.35588833	CEP128	centrosomal protein 128
202960_s_at	4.75E-03	-0.23445456	MUT	methylmalonyl-CoA mutase
218225_at	4.76E-03	0.2681108	ECSIT	ECSIT signalling integrator
236080_at	4.76E-03	-0.1618298	LOC100507670	uncharacterized LOC100507670
208854_s_at	4.76E-03	0.33230621	STK24	serine/threonine kinase 24
204341_at	4.76E-03	0.42101565	TRIM16	tripartite motif containing 16

200862_at	4.77E-03	0.41326383	DHCR24	24-dehydrocholesterol reductase
204034_at	4.77E-03	0.34623873	ETHE1	ETHE1, persulfide dioxygenase
201556_s_at	4.77E-03	0.139243	VAMP2	vesicle associated membrane protein 2
1567253_at	4.78E-03	0.09918187	OR10D3	olfactory receptor family 10 subfamily D member 3 (putative)
203228_at	4.79E-03	0.35343454	PAFAH1B3	platelet activating factor acetylhydrolase 1b catalytic subunit 3
202663_at	4.79E-03	-0.32134346	WIPF1	WAS/WASL interacting protein family member 1
200688_at	4.80E-03	0.2066621	SF3B3	splicing factor 3b subunit 3
202041_s_at	4.81E-03	0.31925523	FIBP	FGF1 intracellular binding protein
227317_at	4.81E-03	0.1125966	LMCD1	LIM and cysteine rich domains 1
234373_x_at	4.81E-03	0.12867825		
216587_s_at	4.82E-03	0.11428866	MIR4683///FZD8	microRNA 4683///frizzled class receptor 8
1558281_a_at	4.82E-03	0.29410615	TMEM184A	transmembrane protein 184A
243307_at	4.83E-03	-0.08100901		
214001_x_at	4.84E-03	-0.19966283	RPS10	ribosomal protein S10
227427_at	4.85E-03	0.1098698	ARHGEF25	Rho guanine nucleotide exchange factor 25
228249_at	4.85E-03	-0.42303631	C11orf74	chromosome 11 open reading frame 74
233563_s_at	4.85E-03	0.21644796	MIR6727///CPSF3L	microRNA 6727///cleavage and polyadenylation specific factor 3-like
227614_at	4.86E-03	0.4811909	HKDC1	hexokinase domain containing 1
213416_at	4.86E-03	-0.45022429	ITGA4	integrin subunit alpha 4
227450_at	4.87E-03	-0.93244423	ERP27	endoplasmic reticulum protein 27
222986_s_at	4.87E-03	0.26731315	SHISA5	shisa family member 5
243768_at	4.87E-03	-0.30980412		
201040_at	4.88E-03	0.22821412	GNAI2	G protein subunit alpha i2
1561706_at	4.88E-03	-0.09819748		
213618_at	4.89E-03	-0.3730418	ARAP2	ArfGAP with RhoGAP domain, ankyrin repeat and PH domain 2
217926_at	4.89E-03	0.23796052	C19orf53	chromosome 19 open reading frame 53
204503_at	4.89E-03	0.26081074	EVPL	envoplakin
1557009_a_at	4.89E-03	-0.06543849	LOC340107	uncharacterized LOC340107
212207_at	4.89E-03	-0.21874736	MED13L	mediator complex subunit 13 like
242570_at	4.89E-03	-0.08991891		
202111_at	4.90E-03	0.29253482	SLC4A2	solute carrier family 4 member 2
244144_at	4.90E-03	0.09478738	SYNE1	spectrin repeat containing nuclear envelope protein 1
1563431_x_at	4.90E-03	-0.24903847		
213539_at	4.92E-03	-0.39361678	CD3D	CD3d molecule
228677_s_at	4.92E-03	-0.15237328	RASAL3	RAS protein activator like 3
203615_x_at	4.92E-03	0.33945997	SULT1A1	sulfotransferase family 1A member 1
239897_at	4.93E-03	-0.21745584	BCLAF1	BCL2 associated transcription factor 1
222206_s_at	4.93E-03	0.29484534	NCLN	nicalin
57539_at	4.94E-03	0.34923567	ZGPAT///LIME1	zinc finger CCCH-type and G-patch domain containing///Lck interacting transmembrane adaptor 1
213350_at	4.95E-03	-0.30768617	RPS11	ribosomal protein S11
214061_at	4.95E-03	-0.34747387	TBC1D31	TBC1 domain family member 31
225104_at	4.95E-03	0.21993118	ZNF598	zinc finger protein 598
224612_s_at	4.96E-03	0.27130275	DNAJC5	DnaJ heat shock protein family (Hsp40) member C5
210378_s_at	4.96E-03	0.21061575	SSNA1	SS nuclear autoantigen 1
218732_at	4.97E-03	-0.29002122	PTRH2	peptidyl-tRNA hydrolase 2
219256_s_at	4.97E-03	0.27313992	SH3TC1	SH3 domain and tetratricopeptide repeats 1
1566700_at	4.97E-03	0.10576653	VRK3	vaccinia related kinase 3
203011_at	4.98E-03	-0.30996982	IMPA1	inositol monophosphatase 1
235871_at	4.98E-03	0.47063598	LIPH	lipase H
226037_s_at	4.98E-03	-0.39697805	TAF9B	TATA-box binding protein associated factor 9b
201142_at	4.99E-03	-0.24664291	EIF2S1	eukaryotic translation initiation factor 2 subunit alpha
224148_at	4.99E-03	-0.08141213	FYB	FYN binding protein
204512_at	4.99E-03	-0.25858615	HIVEP1	human immunodeficiency virus type I enhancer binding protein 1
229073_at	4.99E-03	-0.3521132	PRTG	protogenin
226664_at	5.00E-03	0.23512338	TBC1D20	TBC1 domain family member 20
211257_x_at	5.00E-03	-0.19074469	ZNF638	zinc finger protein 638
1561488_at	5.01E-03	-0.09934184	LOC105377938	uncharacterized LOC105377938

225103_at	5.01E-03	0.29218875	MRPL38	mitochondrial ribosomal protein L38
223934_at	5.01E-03	-0.09559543	SP140L	SP140 nuclear body protein like
1554351_a_at	5.01E-03	0.25621526	TIPRL	TOR signaling pathway regulator
229389_at	5.02E-03	-0.2045444	ATG16L2	autophagy related 16 like 2
220646_s_at	5.02E-03	-0.25223779	KLRF1	killer cell lectin like receptor F1
211271_x_at	5.02E-03	0.19937167	MIR4745///PTBP1	microRNA 4745///polypyrimidine tract binding protein 1
209529_at	5.02E-03	0.44965995	PLPP2	phospholipid phosphatase 2
209190_s_at	5.03E-03	0.23344996	DIAPH1	diaphanous related formin 1
232859_s_at	5.03E-03	0.16724097	MAGI1	membrane associated guanylate kinase, WW and PDZ domain containing 1
208114_s_at	5.04E-03	0.2501427	ISG20L2	interferon stimulated exonuclease gene 20 like 2
236778_at	5.04E-03	-0.44049468		
233177_s_at	5.05E-03	0.29301996	PNKD	paroxysmal nonkinesigenic dyskinesia
203728_at	5.07E-03	0.28216113	BAK1	BCL2 antagonist/killer 1
219012_s_at	5.07E-03	-0.23968997	EMSY	EMSY, BRCA2 interacting transcriptional repressor
1554006_a_at	5.07E-03	0.28934949	LLGL2	LLGL2, scribble cell polarity complex component
225502_at	5.08E-03	-0.50474703	DOCK8	dedicator of cytokinesis 8
231388_at	5.08E-03	-0.11116522		
208094_s_at	5.09E-03	0.21158651	CCDC130	coiled-coil domain containing 130
229244_at	5.09E-03	0.2120211	LSAMP	limbic system-associated membrane protein
222399_s_at	5.10E-03	0.22932209	TM9SF3	transmembrane 9 superfamily member 3
235986_at	5.10E-03	-0.08997095		
240092_at	5.10E-03	0.12245157		
206974_at	5.11E-03	-0.38042504	CXCR6	C-X-C motif chemokine receptor 6
1553859_at	5.12E-03	0.07907525	TPH1	tryptophan hydroxylase 1
203915_at	5.13E-03	-0.8579495	CXCL9	C-X-C motif chemokine ligand 9
224706_at	5.13E-03	0.22402456	LOC102724984///KIAA2013	uncharacterized protein KIAA2013///KIAA2013
232510_s_at	5.14E-03	0.22542358	DPP3	dipeptidyl peptidase 3
1557383_a_at	5.14E-03	-0.41765929		
202039_at	5.15E-03	0.32188614	MYO18A///TIAF1	myosin XVIIIA///TGFB1-induced anti-apoptotic factor 1
242707_at	5.16E-03	-0.1075503	MED23	mediator complex subunit 23
242984_at	5.16E-03	-0.21213652	MKLN1	muskelin 1
213328_at	5.16E-03	-0.24627095	NEK1	NIMA related kinase 1
239928_at	5.16E-03	0.10146998	TCTN2	tectonic family member 2
AFFX-r2-Ec-bioB-M	5.17E-03	-0.19062779		
239756_at	5.17E-03	0.15917356		
208679_s_at	5.18E-03	0.20391458	ARPC2	actin related protein 2/3 complex subunit 2
239942_at	5.18E-03	-0.0872074	GNG11	G protein subunit gamma 11
217308_at	5.18E-03	-0.10039718	OR1F2P	olfactory receptor family 1 subfamily F member 2 pseudogene
223706_at	5.19E-03	0.10075841	C22orf23	chromosome 22 open reading frame 23
216074_x_at	5.19E-03	0.23331362	WWC1	WW and C2 domain containing 1
216830_at	5.21E-03	-0.09638941	LOC101929047///HERC2P10///HERC2	uncharacterized LOC101929047///hect domain and RLD 2 pseudogene 10///HECT and RLD domain containing E3 ubiquitin protein ligase 2
218389_s_at	5.22E-03	0.21359331	APH1A	aph-1 homolog A, gamma-secretase subunit
212849_at	5.22E-03	0.21635655	AXIN1	axin 1
212618_at	5.22E-03	0.15731572	ZNF609	zinc finger protein 609
213833_x_at	5.24E-03	-0.10282635	NOP16	NOP16 nucleolar protein
50376_at	5.24E-03	0.18754344	ZNF444	zinc finger protein 444
207556_s_at	5.25E-03	0.24174878	DGKZ	diacylglycerol kinase zeta
235563_at	5.25E-03	0.36664301	GPRC5A	G protein-coupled receptor class C group 5 member A
221798_x_at	5.25E-03	0.0611276		
209260_at	5.26E-03	0.35308183	SFN	stratifin
214002_at	5.27E-03	0.1544733	MYL6	myosin light chain 6
228174_at	5.27E-03	-0.31125885	SCAI	suppressor of cancer cell invasion
1569675_at	5.28E-03	-0.18942621	POU2AF1	POU class 2 associating factor 1
223666_at	5.28E-03	0.4456826	SNX5	sorting nexin 5
201644_at	5.28E-03	0.43536298	TSTA3	tissue specific transplantation antigen P35B
1553968_a_at	5.29E-03	0.17295065	ADAT3	adenosine deaminase, tRNA specific 3
234984_at	5.29E-03	-0.25935459	NEDD1	neural precursor cell expressed, developmentally down-regulated 1
209777_s_at	5.29E-03	0.16617783	SLC19A1	solute carrier family 19 member 1

217416_x_at	5.29E-03	0.24980277		
219961_s_at	5.30E-03	0.50198532	KIZ	kizuna centrosomal protein
203057_s_at	5.30E-03	-0.17446333	PRDM2	PR/SET domain 2
244038_at	5.30E-03	-0.20554803	WDR89	WD repeat domain 89
1553282_at	5.31E-03	-0.09984201	UMODL1-AS1	UMODL1 antisense RNA 1
203530_s_at	5.32E-03	0.2799368	STX4	syntaxin 4
213644_at	5.33E-03	-0.39795976	CEP112	centrosomal protein 112
219997_s_at	5.33E-03	0.18948373	COPS7B	COP9 signalosome subunit 7B
1569127_at	5.33E-03	-0.14795695		
206006_s_at	5.35E-03	-0.27014122	CEP162	centrosomal protein 162
223150_s_at	5.35E-03	0.14683056	PTPN23	protein tyrosine phosphatase, non-receptor type 23
211823_s_at	5.35E-03	0.0965361	PXN	paxillin
221427_s_at	5.36E-03	0.23923435	CCNL2	cyclin L2
208792_s_at	5.37E-03	0.52037274	CLU	clusterin
219447_s_at	5.37E-03	0.18340312	SLC35C2	solute carrier family 35 member C2
221749_at	5.37E-03	-0.19772549	YTHDF3	YTH N6-methyladenosine RNA binding protein 3
212983_at	5.38E-03	0.30089951	HRAS	HRas proto-oncogene, GTPase
204056_s_at	5.39E-03	0.11578082	MVK	mevalonate kinase
229246_at	5.39E-03	-0.355686	SRSF1	serine and arginine rich splicing factor 1
239630_at	5.39E-03	-0.36563541		
230551_at	5.40E-03	-0.29661358	KSR2	kinase suppressor of ras 2
219807_x_at	5.40E-03	0.20723898	MIA-RAB4B///RAB4B	MIA-RAB4B readthrough (NMD candidate)///RAB4B, member RAS oncogene family
211950_at	5.40E-03	0.32176391	UBR4	ubiquitin protein ligase E3 component n-recognin 4
216208_s_at	5.41E-03	0.13126417	ATF6B	activating transcription factor 6 beta
207390_s_at	5.41E-03	0.25650107	SMTN	smoothelin
223053_x_at	5.41E-03	0.23216281	SSU72	SSU72 homolog, RNA polymerase II CTD phosphatase
217166_at	5.41E-03	0.15943885		
213411_at	5.43E-03	-0.57355612	ADAM22	ADAM metallopeptidase domain 22
204674_at	5.43E-03	-0.35417749	LRMP	lymphoid restricted membrane protein
209309_at	5.44E-03	0.6933282	AZGP1	alpha-2-glycoprotein 1, zinc-binding
215245_x_at	5.44E-03	-0.38493795	FMR1	fragile X mental retardation 1
1569097_at	5.44E-03	0.10127023	TP53BP1	tumor protein p53 binding protein 1
239317_at	5.44E-03	-0.07068		
239895_at	5.45E-03	-0.18415801	AQR	aquarius intron-binding spliceosomal factor
1553105_s_at	5.45E-03	0.29379129	DSG2	desmoglein 2
1563589_at	5.46E-03	-0.07241929	LOC340184	uncharacterized LOC340184
234265_at	5.46E-03	0.09605846	PPP1R12C	protein phosphatase 1 regulatory subunit 12C
209432_s_at	5.47E-03	0.29055249	CREB3	cAMP responsive element binding protein 3
205597_at	5.47E-03	0.49912722	SLC44A4	solute carrier family 44 member 4
209646_x_at	5.49E-03	-0.27958106	ALDH1B1	aldehyde dehydrogenase 1 family member B1
227803_at	5.50E-03	-0.60025112	ENPP5	ectonucleotide pyrophosphatase/phosphodiesterase 5 (putative)
203620_s_at	5.50E-03	-0.27819733	FCHSD2	FCH and double SH3 domains 2
208395_s_at	5.50E-03	0.13475044	URB1	URB1 ribosome biogenesis 1 homolog (S. cerevisiae)
233378_at	5.50E-03	0.11749798		
203689_s_at	5.51E-03	-0.4059832	FMR1	fragile X mental retardation 1
228988_at	5.52E-03	-0.67688938	ZNF711	zinc finger protein 711
200775_s_at	5.53E-03	0.16206731	HNRNPK	heterogeneous nuclear ribonucleoprotein K
228503_at	5.53E-03	-0.74254908	RPS6KA6	ribosomal protein S6 kinase A6
201015_s_at	5.55E-03	0.33943778	JUP	junction plakoglobin
231610_at	5.55E-03	-0.08285378	TTBK2	tau tubulin kinase 2
1553856_s_at	5.56E-03	-0.13451316	P2RY10	purinergic receptor P2Y10
226653_at	5.57E-03	-0.65192392	MARK1	microtubule affinity regulating kinase 1
211168_s_at	5.57E-03	0.25192272	UPF1	UPF1, RNA helicase and ATPase
32209_at	5.58E-03	0.20205509	FAM89B	family with sequence similarity 89 member B
241369_at	5.59E-03	-0.06498542	ADAMTS9-AS1	ADAMTS9 antisense RNA 1
207988_s_at	5.59E-03	0.14946268	ARPC2	actin related protein 2/3 complex subunit 2
209111_at	5.60E-03	0.24544568	RNF5P1///RNF5	ring finger protein 5 pseudogene 1///ring finger protein 5
228172_at	5.61E-03	0.12265512	TTLL11	tubulin tyrosine ligase like 11
230233_at	5.62E-03	-0.54028401		
223479_s_at	5.64E-03	0.32790764	CHCHD5	coiled-coil-helix-coiled-coil-helix domain containing 5

229723_at	5.64E-03	-0.38353298	TAGAP	T-cell activation RhoGTPase activating protein
1554589_at	5.65E-03	0.0980666	LENG9	leukocyte receptor cluster member 9
244579_at	5.65E-03	-0.34850302		
1563472_at	5.65E-03	0.10530993		
230892_at	5.65E-03	-0.41444102		
205211_s_at	5.66E-03	0.12940313	RIN1	Ras and Rab interactor 1
1566340_at	5.66E-03	-0.11361792	SNORD8	small nucleolar RNA, C/D box 8
202664_at	5.66E-03	-0.43574824	WIPF1	WAS/WASL interacting protein family member 1
228861_at	5.67E-03	0.24698595	CDS2	CDP-diacylglycerol synthase 2
201509_at	5.68E-03	0.38211007	IDH3B	isocitrate dehydrogenase 3 (NAD(+)) beta
234515_at	5.68E-03	0.11891219	PCGEM1	PCGEM1, prostate-specific transcript (non-protein coding)
213434_at	5.68E-03	-0.28133238	STX2	syntaxin 2
226269_at	5.70E-03	-0.48748388	GDAP1	ganglioside induced differentiation associated protein 1
223166_x_at	5.70E-03	0.22982571	RABL6	RAB, member RAS oncogene family-like 6
220974_x_at	5.70E-03	0.20544478	SFXN3	sideroflexin 3
215544_s_at	5.70E-03	0.14986866	UBOX5	U-box domain containing 5
231835_at	5.71E-03	0.27587833	FAM213B	family with sequence similarity 213 member B
238030_at	5.71E-03	-0.37545331	ZNF268	zinc finger protein 268
214788_x_at	5.72E-03	0.13033221	DDN	dendrin
218121_at	5.72E-03	0.15465244	HMOX2	heme oxygenase 2
211030_s_at	5.72E-03	0.25758139	SLC6A6	solute carrier family 6 member 6
213508_at	5.72E-03	-0.26817543	SPTSSA	serine palmitoyltransferase small subunit A
211945_s_at	5.73E-03	0.12636333	ITGB1	integrin subunit beta 1
217052_x_at	5.73E-03	-0.09653677		
222060_at	5.76E-03	0.24228983	KRT8P12	keratin 8 pseudogene 12
241636_x_at	5.76E-03	-0.15485624		
214946_x_at	5.77E-03	-0.17213619	LOC101930591///FAM21A///FAM21C	uncharacterized LOC101930591///family with sequence similarity 21 member A///family with sequence similarity 21 member C
229803_s_at	5.77E-03	-0.19560274	RPS10-NUDT3///NUDT3	RPS10-NUDT3 readthrough///nudix hydrolase 3
228236_at	5.78E-03	0.34874194	SLC52A3	solute carrier family 52 member 3
226580_at	5.79E-03	-0.24825698	BRMS1L	breast cancer metastasis-suppressor 1-like
226078_at	5.79E-03	0.13370249	RPUSD1	RNA pseudouridylate synthase domain containing 1
215260_s_at	5.79E-03	0.11849214	TCF3	transcription factor 3
232442_at	5.80E-03	0.10430685	BCAR1	BCAR1, Cas family scaffolding protein
1558214_s_at	5.80E-03	0.28622133	CTNNA1	catenin alpha 1
223029_s_at	5.80E-03	0.16445416	TRAF7	TNF receptor associated factor 7
215177_s_at	5.81E-03	0.36555952	ITGA6	integrin subunit alpha 6
220133_at	5.82E-03	0.75416855	ODAM	odontogenic, ameloblast asssociated
203364_s_at	5.83E-03	0.21349908	ATG13	autophagy related 13
1555907_at	5.84E-03	0.18203537	AGAP2-AS1	AGAP2 antisense RNA 1
231568_at	5.84E-03	0.08995382	CT47A12///CT47A1///CT47A2///CT47A3///CT47A4///CT47A5///CT47A6///CT47A8///CT47A9///CT47A10///CT47A7///CT47A11	cancer/testis antigen family 47, member A12///cancer/testis antigen family 47, member A1///cancer/testis antigen family 47, member A2///cancer/testis antigen family 47, member A3///cancer/testis antigen family 47, member A4///cancer/testis antigen family 47, member A5///cancer/testis antigen family 47, member A6///cancer/testis antigen family 47, member A8///cancer/testis antigen family 47, member A9///cancer/testis antigen family 47, member A10///cancer/testis antigen family 47, member A7///cancer/testis antigen family 47, member A11
242282_at	5.84E-03	0.18517566	ZFPM1	zinc finger protein, FOG family member 1
229625_at	5.85E-03	-0.31702983	GBP5	guanylate binding protein 5
225122_at	5.85E-03	0.09731631	RNF31	ring finger protein 31
201021_s_at	5.86E-03	0.35789137	DSTN	destrin, actin depolymerizing factor
242847_at	5.86E-03	-0.22104308	EMSY	EMSY, BRCA2 interacting transcriptional repressor
1568598_at	5.86E-03	0.14542097	KAZALD1	Kazal type serine peptidase inhibitor domain 1
235919_at	5.87E-03	-0.34661916	CEP78	centrosomal protein 78
222429_at	5.87E-03	0.24024601	MIR6837///DBNL	microRNA 6837///drebrin like
213785_at	5.88E-03	0.21325831	IPO9	importin 9

1554065_at	5.88E-03	0.07837595	MVB12B	multivesicular body subunit 12B
221855_at	5.88E-03	0.14711854	SDHAF1	succinate dehydrogenase complex assembly factor 1
216971_s_at	5.89E-03	0.12928219	PLEC	plectin
206113_s_at	5.91E-03	0.14950507	RAB5A	RAB5A, member RAS oncogene family
222652_s_at	5.92E-03	0.13693212	GLYR1	glyoxylate reductase 1 homolog
203163_at	5.92E-03	0.30851186	KATNB1	katanin regulatory subunit B1
208989_s_at	5.92E-03	0.18710671	KDM2A	lysine demethylase 2A
212269_s_at	5.92E-03	0.19366738	MCM3AP	minichromosome maintenance complex component 3 associated protein
221649_s_at	5.92E-03	0.21765374	PPAN-P2RY11///PPAN	PPAN-P2RY11 readthrough///peter pan homolog (Drosophila)
228520_s_at	5.94E-03	-0.28773886	APLP2	amyloid beta precursor like protein 2
242584_at	5.94E-03	-0.31858245	FAM161A	family with sequence similarity 161 member A
213887_s_at	5.94E-03	0.32115613	POLR2E	RNA polymerase II subunit E
202782_s_at	5.95E-03	0.15624575	INPP5K	inositol polyphosphate-5-phosphatase K
209008_x_at	5.95E-03	0.4187412	KRT8	keratin 8
207463_x_at	5.95E-03	0.30883527	PRSS3	protease, serine 3
227998_at	5.97E-03	0.40378372	S100A16	S100 calcium binding protein A16
232614_at	5.97E-03	-0.18464093		
238599_at	5.98E-03	-0.46984072	IRAK1BP1	interleukin 1 receptor associated kinase 1 binding protein 1
211026_s_at	5.98E-03	0.39435895	MGLL	monoglyceride lipase
231406_at	5.98E-03	0.24243347	ORAI2	ORAI calcium release-activated calcium modulator 2
45526_g_at	5.99E-03	0.17963074	NAA60	N(alpha)-acetyltransferase 60, NatF catalytic subunit
1562261_at	6.00E-03	-0.09027644	AMZ1	archaelysin family metallopeptidase 1
200041_s_at	6.00E-03	0.31350523	ATP6V1G2-DDX39B///SNORD84///DDX39B	ATP6V1G2-DDX39B readthrough (NMD candidate)///small nucleolar RNA, C/D box 84///DEAD-box helicase 39B
211396_at	6.00E-03	0.11445952	FCGR2C	Fc fragment of IgG receptor IIc (gene/pseudogene)
231577_s_at	6.01E-03	-0.51198174	GBP1	guanylate binding protein 1
235560_at	6.01E-03	-0.11770132	NOVA2	NOVA alternative splicing regulator 2
211796_s_at	6.01E-03	-0.46916349	TRBC1	T cell receptor beta constant 1
227389_x_at	6.02E-03	-0.11525886	IRF2BP2	interferon regulatory factor 2 binding protein 2
227458_at	6.03E-03	-0.41643638	CD274	CD274 molecule
1554574_a_at	6.03E-03	0.14506616	CYB5R3	cytochrome b5 reductase 3
1562022_s_at	6.03E-03	0.19043002	LOC100130987///RAD9A	uncharacterized LOC100130987///RAD9 checkpoint clamp component A
1565454_at	6.04E-03	0.17489585	XAGE-4	XAGE-4 protein
243819_at	6.04E-03	-0.26458504		
1554795_a_at	6.05E-03	0.18963635	FBLIM1	filamin binding LIM protein 1
210439_at	6.06E-03	-0.28654678	ICOS	inducible T-cell costimulator
205934_at	6.06E-03	-0.26930218	PLCL1	phospholipase C like 1
209670_at	6.06E-03	-0.30438928	TRAC	T-cell receptor alpha constant
214284_s_at	6.07E-03	-0.10807925	FGF18	fibroblast growth factor 18
236248_x_at	6.08E-03	-0.19460271	TADA2B	transcriptional adaptor 2B
228863_at	6.09E-03	-0.4165069	PCDH17	protocadherin 17
221827_at	6.09E-03	0.38812458	RBCK1	RANBP2-type and C3HC4-type zinc finger containing 1
232219_x_at	6.09E-03	0.26121986	USP21	ubiquitin specific peptidase 21
1553566_at	6.10E-03	0.10726043	PIANP	PILR alpha associated neural protein
230836_at	6.10E-03	-0.45464848	ST8SIA4	ST8 alpha-N-acetyl-neuraminide alpha-2,8-sialyltransferase 4
201871_s_at	6.10E-03	0.26197493	UBXN1	UBX domain protein 1
242884_at	6.11E-03	-0.09893484	LOC440570	uncharacterized LOC440570
228445_at	6.13E-03	0.20107239	AIFM2	apoptosis inducing factor, mitochondria associated 2
218139_s_at	6.13E-03	-0.25624884	AP5M1	adaptor related protein complex 5 mu 1 subunit
205874_at	6.14E-03	0.2628668	ITPKA	inositol-trisphosphate 3-kinase A
207238_s_at	6.14E-03	-0.43365035	PTPRC	protein tyrosine phosphatase, receptor type C
221494_x_at	6.15E-03	0.23946748	EIF3K	eukaryotic translation initiation factor 3 subunit K
221217_s_at	6.15E-03	0.14239892	RBFOX1	RNA binding protein, fox-1 homolog 1
201474_s_at	6.16E-03	0.28247741	ITGA3	integrin subunit alpha 3
200914_x_at	6.16E-03	-0.25725529	KTN1	kinectin 1
206107_at	6.16E-03	0.16596981	RGS11	regulator of G-protein signaling 11
244654_at	6.17E-03	-0.1597799	MYO1G	myosin IG

204928_s_at	6.18E-03	0.29778952	SLC10A3	solute carrier family 10 member 3
1569144_a_at	6.19E-03	0.11520477	CYSRT1	cysteine rich tail 1
204905_s_at	6.19E-03	-0.29300377	EEF1E1	eukaryotic translation elongation factor 1 epsilon 1
205860_x_at	6.19E-03	-0.19240113	FOLH1B///FOLH1	folate hydrolase 1B///folate hydrolase (prostate-specific membrane antigen) 1
239929_at	6.19E-03	0.09433203	PM20D1	peptidase M20 domain containing 1
1566722_a_at	6.19E-03	-0.08150101	SVEP1	sushi, von Willebrand factor type A, EGF and pentraxin domain containing 1
214365_at	6.19E-03	0.09277096	TPM3	tropomyosin 3
203442_x_at	6.20E-03	0.15890455	EML3	echinoderm microtubule associated protein like 3
217950_at	6.20E-03	0.25444787	NOSIP	nitric oxide synthase interacting protein
218481_at	6.21E-03	0.27474483	EXOSC5	exosome component 5
48030_i_at	6.22E-03	0.12308145	FAXDC2	fatty acid hydroxylase domain containing 2
210460_s_at	6.22E-03	0.23717887	PSMD4	proteasome 26S subunit, non-ATPase 4
1558689_a_at	6.22E-03	-0.19136819	STX17-AS1	STX17 antisense RNA 1
238441_at	6.23E-03	-0.83247463	PRKAA2	protein kinase AMP-activated catalytic subunit alpha 2
203021_at	6.23E-03	0.63966034	SLPI	secretory leukocyte peptidase inhibitor
205291_at	6.24E-03	-0.18265012	IL2RB	interleukin 2 receptor subunit beta
202906_s_at	6.24E-03	-0.37325134	NBN	nibrin
218749_s_at	6.24E-03	0.17279229	SLC8B1	solute carrier family 8 member B1
213736_at	6.26E-03	-0.22464254	COX5B	cytochrome c oxidase subunit 5B
218272_at	6.26E-03	0.28408605	TTC38	tetratricopeptide repeat domain 38
203551_s_at	6.27E-03	-0.26690272	COX11	COX11, cytochrome c oxidase copper chaperone
202205_at	6.27E-03	0.23773223	VASP	vasodilator-stimulated phosphoprotein
213860_x_at	6.28E-03	0.203445	CSNK1A1	casein kinase 1 alpha 1
226144_at	6.28E-03	0.11381563	MIR1909///REXO1	microRNA 1909///RNA exonuclease 1 homolog
1553535_a_at	6.29E-03	0.19529858	RANGAP1	Ran GTPase activating protein 1
242120_at	6.30E-03	0.09072219	USP49	ubiquitin specific peptidase 49
1564314_at	6.31E-03	0.09031102	LOC219690	uncharacterized LOC219690
237184_at	6.31E-03	-0.152672		
39249_at	6.32E-03	0.14657348	AQP3	aquaporin 3 (Gill blood group)
212263_at	6.32E-03	-0.40248326	QKI	QKI, KH domain containing RNA binding
223318_s_at	6.33E-03	0.35313446	ALKBH7	alkB homolog 7
230309_at	6.33E-03	-0.06846943	BHMT2	betaine--homocysteine S-methyltransferase 2
229316_at	6.33E-03	0.09665924	KXD1	KxDL motif containing 1
240890_at	6.33E-03	-0.42143187	LOC643733	caspase 4, apoptosis-related cysteine peptidase pseudogene
228719_at	6.33E-03	-0.23446715	ZSWIM7	zinc finger SWIM-type containing 7
229131_at	6.35E-03	-0.19116415	EXD2	exonuclease 3'-5' domain containing 2
200913_at	6.35E-03	0.28017794	PPM1G	protein phosphatase, Mg2+/Mn2+ dependent 1G
1559808_at	6.35E-03	0.13974123		
235577_at	6.36E-03	-0.31480461	ZNF652	zinc finger protein 652
214643_x_at	6.37E-03	0.18064566	BIN1	bridging integrator 1
1555212_at	6.37E-03	0.07427258	OR8B8	olfactory receptor family 8 subfamily B member 8
215657_at	6.37E-03	-0.59406844	SLC26A3	solute carrier family 26 member 3
238932_at	6.37E-03	0.22450268	TSC22D2	TSC22 domain family member 2
220330_s_at	6.38E-03	-0.5277474	SAMSN1	SAM domain, SH3 domain and nuclear localization signals 1
1556314_a_at	6.38E-03	-0.2220722	ZNF366	zinc finger protein 366
205089_at	6.38E-03	-0.29812058	ZNF7	zinc finger protein 7
216363_at	6.38E-03	0.10158705		
232242_at	6.39E-03	-0.17171052	PTPRG-AS1	PTPRG antisense RNA 1
227992_s_at	6.39E-03	0.16248803	SPACA6	sperm acrosome associated 6
244404_at	6.39E-03	-0.24773871	STXBP4	syntaxin binding protein 4
235860_at	6.39E-03	-0.20193488		
1569468_at	6.40E-03	-0.0720122	ZNF876P	zinc finger protein 876, pseudogene
202489_s_at	6.41E-03	0.48040872	FXYD3	FXYD domain containing ion transport regulator 3
203709_at	6.41E-03	0.21628535	PHKG2	phosphorylase kinase catalytic subunit gamma 2
212587_s_at	6.41E-03	-0.52280228	PTPRC	protein tyrosine phosphatase, receptor type C
218316_at	6.41E-03	-0.21933838	TIMM9	translocase of inner mitochondrial membrane 9
241235_at	6.42E-03	-0.0573991		
234335_s_at	6.43E-03	-0.29202752	FAM84A	family with sequence similarity 84 member A
203953_s_at	6.44E-03	0.48822806	CLDN3	claudin 3

200764_s_at	6.44E-03	0.22304099	CTNNA1	catenin alpha 1
222641_s_at	6.44E-03	0.1961412	FAM222B	family with sequence similarity 222 member B
241850_at	6.44E-03	-0.07756313		
212786_at	6.45E-03	0.17795535	CLEC16A	C-type lectin domain family 16 member A
213160_at	6.45E-03	-0.25382404	DOCK2	dedicator of cytokinesis 2
214014_at	6.46E-03	0.24004991	CDC42EP2	CDC42 effector protein 2
201401_s_at	6.46E-03	0.12521954	GRK2	G protein-coupled receptor kinase 2
203976_s_at	6.47E-03	0.20027756	CHAF1A	chromatin assembly factor 1 subunit A
241808_at	6.47E-03	-0.49651293	ZC2HC1A	zinc finger C2HC-type containing 1A
201704_at	6.48E-03	0.36531379	ENTPD6	ectonucleoside triphosphate diphosphohydrolase 6 (putative)
212120_at	6.48E-03	-0.22437409	RHOQ	ras homolog family member Q
227737_at	6.48E-03	0.23802503	SRPRB	SRP receptor beta subunit
222675_s_at	6.49E-03	0.28680943	BAIAP2L1	BAI1 associated protein 2 like 1
214277_at	6.49E-03	-0.32029166	COX11	COX11, cytochrome c oxidase copper chaperone
205954_at	6.49E-03	0.07951368	RXRG	retinoid X receptor gamma
244105_at	6.49E-03	-0.09268807	WHAMMP2///WHAMMP3	WAS protein homolog associated with actin, golgi membranes and microtubules pseudogene 2///WAS protein homolog associated with actin, golgi membranes and microtubules pseudogene 3
1564208_x_at	6.52E-03	0.10887523	LINC00957	long intergenic non-protein coding RNA 957
229736_at	6.52E-03	0.12224799	TMEM86B	transmembrane protein 86B
239803_at	6.52E-03	-0.09148862		
219346_at	6.53E-03	0.22530033	LRFN3	leucine rich repeat and fibronectin type III domain containing 3
200022_at	6.53E-03	0.25084111	RPL18	ribosomal protein L18
241057_x_at	6.53E-03	-0.12843032		
201782_s_at	6.54E-03	0.2101891	AIP	aryl hydrocarbon receptor interacting protein
1555742_at	6.54E-03	-0.24078795	ERVH-6	endogenous retrovirus group H member 6
203411_s_at	6.54E-03	0.31846828	LMNA	lamin A/C
217730_at	6.54E-03	0.31887738	MIR6513///TMBIM1	microRNA 6513///transmembrane BAX inhibitor motif containing 1
233813_at	6.54E-03	-0.21586708	PPP1R16B	protein phosphatase 1 regulatory subunit 16B
218624_s_at	6.55E-03	0.23918154	CENPBD1P1	CENPB DNA-binding domains containing 1 pseudogene 1
203592_s_at	6.55E-03	0.20901506	FSTL3	follistatin like 3
219762_s_at	6.55E-03	0.34879611	RPL36	ribosomal protein L36
204692_at	6.55E-03	-0.09826531	SAP25///LRCH4	Sin3A associated protein 25///leucine rich repeats and calponin homology domain containing 4
1554475_a_at	6.56E-03	0.09434549	C19orf47	chromosome 19 open reading frame 47
202841_x_at	6.56E-03	0.15555011	OGFR	opioid growth factor receptor
219141_s_at	6.57E-03	0.18514421	AMBRA1	autophagy and beclin 1 regulator 1
223226_x_at	6.57E-03	0.25631057	SSBP4	single stranded DNA binding protein 4
204196_x_at	6.59E-03	0.07569072	PKNOX1	PBX/knotted 1 homeobox 1
209124_at	6.60E-03	0.22641212	MYD88	myeloid differentiation primary response 88
1553442_a_at	6.61E-03	0.09192251	CNTNAP4	contactin associated protein like 4
223327_x_at	6.61E-03	0.21143225	GOLGA2P10	golgin A2 pseudogene 10
220553_s_at	6.61E-03	-0.28321336	PRPF39	pre-mRNA processing factor 39
225055_at	6.62E-03	-0.33135492	LINC00674	long intergenic non-protein coding RNA 674
239615_at	6.63E-03	0.12829342	SLC22A5	solute carrier family 22 member 5
220937_s_at	6.63E-03	0.29436792	ST6GALNAC4	ST6 N-acetylgalactosaminide alpha-2,6-sialyltransferase 4
219166_at	6.64E-03	-0.26278616	DNAAF2	dynein axonemal assembly factor 2
213944_x_at	6.64E-03	0.17688472	GNA11	G protein subunit alpha 11
201053_s_at	6.65E-03	0.30677104	PSMF1	proteasome inhibitor subunit 1
211098_x_at	6.65E-03	0.20437798	TMCO1	transmembrane and coiled-coil domains 1
242651_at	6.65E-03	-0.07198448		
237588_at	6.68E-03	-0.13012007		
207969_x_at	6.70E-03	0.10413136	ACRV1	acrosomal vesicle protein 1
239849_at	6.70E-03	-0.10745847		
209232_s_at	6.71E-03	0.19254725	DCTN5	dynactin subunit 5
202116_at	6.71E-03	0.29034	DPF2	double PHD fingers 2
215794_x_at	6.71E-03	0.24078984	GLUD2	glutamate dehydrogenase 2

236293_at	6.72E-03	-0.31652535	RHOH	ras homolog family member H
210978_s_at	6.72E-03	0.25651232	TAGLN2	transgelin 2
240429_at	6.72E-03	-0.09544921	ZNF546	zinc finger protein 546
229684_s_at	6.72E-03	-0.15751582	ZNF644	zinc finger protein 644
207667_s_at	6.73E-03	0.19543789	LOC100996792///MAP2K3	dual specificity mitogen-activated protein kinase kinase 3///mitogen-activated protein kinase kinase 3
205591_at	6.73E-03	-0.21279382	OLFM1	olfactomedin 1
1561234_at	6.73E-03	-0.0645224		
203108_at	6.74E-03	0.43860891	GPRC5A	G protein-coupled receptor class C group 5 member A
220232_at	6.74E-03	-0.18051792	SCD5	stearoyl-CoA desaturase 5
231960_at	6.76E-03	-0.27577386	BRWD1	bromodomain and WD repeat domain containing 1
200011_s_at	6.77E-03	0.28635255	ARF3	ADP ribosylation factor 3
217139_at	6.77E-03	0.11817537	VDAC1	voltage dependent anion channel 1
216455_at	6.78E-03	-0.08847834	LOC101927181	uncharacterized LOC101927181
211507_s_at	6.78E-03	0.10359102	MTMR3	myotubularin related protein 3
225705_at	6.79E-03	-0.25473135	CEP95	centrosomal protein 95
203255_at	6.79E-03	-0.26922831	FBXO11	F-box protein 11
241095_at	6.79E-03	0.09382875	LOC102723661	uncharacterized LOC102723661
207784_at	6.80E-03	-0.10734056	ARSD	arylsulfatase D
225880_at	6.80E-03	0.24335543	TOR1AIP2	torsin 1A interacting protein 2
1554378_a_at	6.81E-03	0.07314561	PDE1C	phosphodiesterase 1C
223152_at	6.81E-03	0.12249552	PPP1R12C	protein phosphatase 1 regulatory subunit 12C
221417_x_at	6.81E-03	0.09154274	S1PR5	sphingosine-1-phosphate receptor 5
1552477_a_at	6.82E-03	0.30878004	IRF6	interferon regulatory factor 6
221960_s_at	6.83E-03	-0.27548127	RAB2A	RAB2A, member RAS oncogene family
212316_at	6.84E-03	0.28857904	NUP210	nucleoporin 210
242820_at	6.84E-03	-0.05771557		
229113_s_at	6.87E-03	0.28658571	FAAP20	Fanconi anemia core complex associated protein 20
226500_at	6.88E-03	0.1198272	ZBTB47	zinc finger and BTB domain containing 47
211286_x_at	6.89E-03	-0.13013165	CSF2RA	colony stimulating factor 2 receptor alpha subunit
214460_at	6.90E-03	0.28045523	LSAMP	limbic system-associated membrane protein
1556582_at	6.90E-03	0.15139799	PVR	poliovirus receptor
212997_s_at	6.90E-03	-0.21500002	TLK2	tousled like kinase 2
220649_at	6.91E-03	-0.08159529	AGBL3	ATP/GTP binding protein like 3
202610_s_at	6.91E-03	-0.27706522	MED14	mediator complex subunit 14
241003_at	6.91E-03	0.08545144		
200789_at	6.92E-03	0.38046919	ECH1	enoyl-CoA hydratase 1
207364_at	6.92E-03	0.13146917	TEX28	testis expressed 28
213039_at	6.93E-03	0.24029486	ARHGEF18	Rho/Rac guanine nucleotide exchange factor 18
206761_at	6.93E-03	-0.14777017	CD96	CD96 molecule
208930_s_at	6.93E-03	0.28675656	ILF3	interleukin enhancer binding factor 3
207778_at	6.93E-03	0.23073656	REG1CP	regenerating family member 1 gamma, pseudogene
241491_at	6.93E-03	-0.14470781		
240235_at	6.94E-03	0.08345533	C10orf62	chromosome 10 open reading frame 62
207709_at	6.94E-03	-0.21009239	PRKAA2	protein kinase AMP-activated catalytic subunit alpha 2
240816_at	6.94E-03	-0.10634323		
228853_at	6.95E-03	-0.29139896	STYX	serine/threonine/tyrosine interacting protein
204655_at	6.96E-03	-0.48003306	CCL5	C-C motif chemokine ligand 5
216306_x_at	6.96E-03	0.21699418	MIR4745///PTBP1	microRNA 4745///polypyrimidine tract binding protein 1
36936_at	6.96E-03	0.43682537	TSTA3	tissue specific transplantation antigen P35B
203236_s_at	6.97E-03	0.28893901	LGALS9	galectin 9
229444_at	6.97E-03	-0.28296775	LOC101929243	uncharacterized LOC101929243
206105_at	7.01E-03	0.08754681	AFF2	AF4/FMR2 family member 2
205159_at	7.01E-03	-0.52046657	CSF2RB	colony stimulating factor 2 receptor beta common subunit
212795_at	7.01E-03	-0.24829708	KIAA1033	KIAA1033
216060_s_at	7.03E-03	-0.322039	DAAM1	dishevelled associated activator of morphogenesis 1
218780_at	7.03E-03	0.23690044	HOOK2	hook microtubule tethering protein 2
242045_at	7.03E-03	-0.30683648		
240044_x_at	7.04E-03	-0.29215836	TNRC6B	trinucleotide repeat containing 6B
234739_at	7.06E-03	-0.07890165		
220380_at	7.07E-03	0.11548333	DNASE2B	deoxyribonuclease 2 beta
223256_at	7.07E-03	-0.25508977	G2E3	G2/M-phase specific E3 ubiquitin protein ligase

225528_at	7.07E-03	-0.21691487	IPO8	importin 8
202028_s_at	7.07E-03	-0.23487542	RPL38	ribosomal protein L38
220463_at	7.07E-03	0.09133239	TRPM3	transient receptor potential cation channel subfamily M member 3
212517_at	7.09E-03	0.42994263	ATRN	attractin
209530_at	7.09E-03	0.16662336	CACNB3	calcium voltage-gated channel auxiliary subunit beta 3
205976_at	7.09E-03	-0.27385008	FASTKD2	FAST kinase domains 2
232575_at	7.09E-03	-0.07072752	PCA3	prostate cancer associated 3 (non-protein coding)
228070_at	7.09E-03	-0.21185847	PPP2R5E	protein phosphatase 2 regulatory subunit B'epsilon
217861_s_at	7.09E-03	0.24755286	PREB	prolactin regulatory element binding
1562910_at	7.09E-03	0.10146423	SH3PXD2B	SH3 and PX domains 2B
238498_at	7.09E-03	0.34402931		
1570255_s_at	7.10E-03	-0.11747506	ANKRD20A12P///ANKRD20A8P///ANKRD20A4///ANKRD20A2///ANKRD20A3///ANKRD20A5P///ANKRD20A1	ankyrin repeat domain 20 family member A12, pseudogene///ankyrin repeat domain 20 family member A8, pseudogene///ankyrin repeat domain 20 family member A4///ankyrin repeat domain 20 family member A2///ankyrin repeat domain 20 family member A3///ankyrin repeat domain 20 family member A5, pseudogene///ankyrin repeat domain 20 family member A1
203193_at	7.10E-03	0.12831787	ESRRA	estrogen related receptor alpha
229322_at	7.10E-03	-0.26765119	PPP2R5E	protein phosphatase 2 regulatory subunit B'epsilon
232609_at	7.11E-03	0.15898286	CRB3	crumbs 3, cell polarity complex component
226701_at	7.11E-03	-0.17645681	GJA5	gap junction protein alpha 5
225014_at	7.11E-03	-0.23971074	SMIM20	small integral membrane protein 20
241891_at	7.11E-03	-0.29929577		
218950_at	7.12E-03	0.31350224	ARAP3	ArfGAP with RhoGAP domain, ankyrin repeat and PH domain 3
220103_s_at	7.12E-03	-0.1073833	MRPS18C	mitochondrial ribosomal protein S18C
208649_s_at	7.12E-03	0.23261503	VCP	valosin containing protein
219171_s_at	7.12E-03	-0.22289155	ZNF236	zinc finger protein 236
242008_at	7.12E-03	-0.29758407		
217818_s_at	7.13E-03	0.15753377	ARPC4	actin related protein 2/3 complex subunit 4
236400_at	7.13E-03	-0.13004579	IDH1-AS1	IDH1 antisense RNA 1
230874_at	7.13E-03	-0.33310474	SLC36A4	solute carrier family 36 member 4
206775_at	7.14E-03	0.09275865	CUBN	cubilin
230592_at	7.14E-03	-0.23048299	NSL1	NSL1, MIS12 kinetochore complex component
226201_at	7.17E-03	0.21577792	DOT1L	DOT1 like histone lysine methyltransferase
211115_x_at	7.17E-03	-0.31676504	GEMIN2	gem nuclear organelle associated protein 2
216640_s_at	7.17E-03	0.31981265	PDIA6	protein disulfide isomerase family A member 6
231411_at	7.18E-03	-0.17768927	LHFP	lipoma HMGIC fusion partner
227225_at	7.18E-03	-0.09934535	ZNF503	zinc finger protein 503
225063_at	7.19E-03	0.23477883	UBL7	ubiquitin like 7
208770_s_at	7.20E-03	0.20446069	EIF4EBP2	eukaryotic translation initiation factor 4E binding protein 2
219798_s_at	7.20E-03	0.25042176	MEPCE	methylphosphate capping enzyme
1554774_at	7.20E-03	0.1925499	MINA	MYC induced nuclear antigen
214839_at	7.20E-03	-0.10891241	MIR124-1///LINC00599	microRNA 124-1///long intergenic non-protein coding RNA 599
1554503_a_at	7.20E-03	0.10120629	OSCAR	osteoclast associated, immunoglobulin-like receptor
244287_at	7.20E-03	0.16361781	SREK1	splicing regulatory glutamic acid and lysine rich protein 1
1487_at	7.21E-03	0.20983749	ESRRA	estrogen related receptor alpha
211031_s_at	7.22E-03	0.21403916	CLIP2	CAP-Gly domain containing linker protein 2
200869_at	7.22E-03	0.23865717	SNORA68///RPL18A	small nucleolar RNA, H/ACA box 68///ribosomal protein L18a
240014_at	7.23E-03	0.11300397	POLR2J4	RNA polymerase II subunit J4, pseudogene
228397_at	7.23E-03	-0.32153125	TUG1	taurine up-regulated 1 (non-protein coding)
227549_x_at	7.23E-03	0.15981853	ZDHHC24	zinc finger DHHC-type containing 24
213276_at	7.24E-03	0.10021363	CAMK2B	calcium/calmodulin dependent protein kinase II beta
216267_s_at	7.24E-03	0.22574995	TMEM115	transmembrane protein 115
239500_at	7.25E-03	-0.08966327	EFCAB1	EF-hand calcium binding domain 1
200736_s_at	7.25E-03	0.32970548	GPX1	glutathione peroxidase 1

202121_s_at	7.26E-03	0.27722379	CHMP2A	charged multivesicular body protein 2A
221706_s_at	7.26E-03	0.26417812	USE1	unconventional SNARE in the ER 1
234251_at	7.26E-03	0.09332715		
201763_s_at	7.27E-03	0.1957294	DAXX	death domain associated protein
202593_s_at	7.27E-03	0.30800415	GDE1	glycerophosphodiester phosphodiesterase 1
226850_at	7.27E-03	-0.30098875	SUMF1	sulfatase modifying factor 1
235211_at	7.27E-03	-0.2251346		
209674_at	7.28E-03	-0.21002694	CRY1	cryptochrome circadian clock 1
235025_at	7.28E-03	-0.30232301	WDR89	WD repeat domain 89
238595_at	7.28E-03	-0.37965092		
240232_at	7.29E-03	-0.24883436		
1568981_at	7.29E-03	0.08241904		
227646_at	7.30E-03	-0.2862076	EBF1	early B-cell factor 1
37408_at	7.31E-03	0.20982683	MRC2	mannose receptor C type 2
241666_at	7.32E-03	0.2033046	TCAIM	T-cell activation inhibitor, mitochondrial
228224_at	7.33E-03	0.11339265	PRELP	proline and arginine rich end leucine rich repeat protein
213566_at	7.33E-03	-0.48426099	RNASE6	ribonuclease A family member k6
232392_at	7.33E-03	-0.32754805	SRSF3	serine and arginine rich splicing factor 3
215419_at	7.33E-03	-0.07332151	ZFR2	zinc finger RNA binding protein 2
206656_s_at	7.34E-03	0.34080421	APMAP	adipocyte plasma membrane associated protein
212156_at	7.34E-03	0.19592389	VPS39	VPS39, HOPS complex subunit
238903_at	7.35E-03	-0.31769104	UBXN2B	UBX domain protein 2B
200965_s_at	7.36E-03	0.30778024	ABLIM1	actin binding LIM protein 1
229946_at	7.36E-03	0.15259527	FAM168B	family with sequence similarity 168 member B
204132_s_at	7.38E-03	0.36166283	FOXO3B///FOXO3	forkhead box O3B pseudogene///forkhead box O3
211821_x_at	7.38E-03	0.07102072	GYPA	glycophorin A (MNS blood group)
205229_s_at	7.39E-03	-0.71122167	COCH	cochlin
222718_at	7.39E-03	0.15840997	TMEM8A	transmembrane protein 8A
207765_s_at	7.43E-03	0.22752169	FAM214B	family with sequence similarity 214 member B
223601_at	7.43E-03	0.0874106	OLFM2	olfactomedin 2
238786_at	7.44E-03	-0.18334322	ANK3	ankyrin 3, node of Ranvier (ankyrin G)
241887_at	7.45E-03	-0.34029235	UBE2W	ubiquitin conjugating enzyme E2 W (putative)
202177_at	7.46E-03	0.40295878	GAS6	growth arrest specific 6
160020_at	7.46E-03	0.09381855	MMP14	matrix metallopeptidase 14
203421_at	7.46E-03	0.1749389	TP53I11	tumor protein p53 inducible protein 11
237439_at	7.46E-03	-0.20618394	USP43	ubiquitin specific peptidase 43
207986_x_at	7.47E-03	0.11014038	CYB561	cytochrome b561
239493_at	7.47E-03	-0.25215109	RPL7	ribosomal protein L7
241917_at	7.47E-03	-0.17058442		
208465_at	7.48E-03	0.0846707	GRM2	glutamate metabotropic receptor 2
235145_at	7.48E-03	0.12233984	ZBTB7B	zinc finger and BTB domain containing 7B
215130_s_at	7.49E-03	0.14110722	IQCK	IQ motif containing K
226531_at	7.50E-03	0.20592734	ORAI1	ORAI calcium release-activated calcium modulator 1
243868_at	7.50E-03	-0.34796772	RFX3	regulatory factor X3
209178_at	7.51E-03	0.1798641	DHX38	DEAH-box helicase 38
220150_s_at	7.51E-03	-0.31664338	FAM184A	family with sequence similarity 184 member A
214258_x_at	7.51E-03	0.17365235	KAT5	lysine acetyltransferase 5
217466_x_at	7.51E-03	0.24016833	SNORA64///RPS2	small nucleolar RNA, H/ACA box 64///ribosomal protein S2
207940_x_at	7.52E-03	0.08397826	CNR1	cannabinoid receptor 1
1555809_at	7.52E-03	0.0992329	CRISPLD2	cysteine rich secretory protein LCCL domain containing 2
205758_at	7.53E-03	-0.30818256	CD8A	CD8a molecule
1565694_at	7.53E-03	0.08511236	DTYMK	deoxythymidylate kinase
211609_x_at	7.53E-03	0.22452067	PSMD4	proteasome 26S subunit, non-ATPase 4
238764_at	7.54E-03	0.09648535	CSAD	cysteine sulfinic acid decarboxylase
206855_s_at	7.54E-03	0.20529351	HYAL2	hyaluronoglucosaminidase 2
214344_at	7.56E-03	0.09770823	LINC00950	long intergenic non-protein coding RNA 950
1560689_s_at	7.57E-03	0.16853806	AKT2	AKT serine/threonine kinase 2
203105_s_at	7.57E-03	-0.26902176	DNM1L	dynamin 1 like
210773_s_at	7.57E-03	0.10730967	FPR2	formyl peptide receptor 2
218928_s_at	7.57E-03	0.29821874	SLC37A1	solute carrier family 37 member 1

222086_s_at	7.57E-03	0.061445	WNT6	Wnt family member 6
225307_at	7.57E-03	0.31143616	ZNF511	zinc finger protein 511
1557316_at	7.57E-03	0.15805287		
1557242_at	7.57E-03	0.21179831		
230341_x_at	7.58E-03	-0.13749808	ADAMTS10	ADAM metallopeptidase with thrombospondin type 1 motif 10
228306_at	7.58E-03	0.21623471	CNIH4	cornichon family AMPA receptor auxiliary protein 4
206153_at	7.58E-03	0.14798628	CYP4F11	cytochrome P450 family 4 subfamily F member 11
212127_at	7.58E-03	0.22059669	RANGAP1	Ran GTPase activating protein 1
210202_s_at	7.59E-03	0.29175163	BIN1	bridging integrator 1
219928_s_at	7.59E-03	0.11053775	CABYR	calcium binding tyrosine phosphorylation regulated
1553262_a_at	7.61E-03	0.09178356	UTS2R	urotensin 2 receptor
1563333_at	7.61E-03	0.05922649		
227273_at	7.62E-03	-0.27038756	BMPR1A	bone morphogenetic protein receptor type 1A
1560818_at	7.63E-03	-0.13098106	LINC00944	long intergenic non-protein coding RNA 944
222385_x_at	7.63E-03	0.24145682	SEC61A1	Sec61 translocon alpha 1 subunit
218038_at	7.64E-03	0.18922185	ATP5SL	ATP5S like
232909_s_at	7.65E-03	-0.2243827	BPTF	bromodomain PHD finger transcription factor
205516_x_at	7.65E-03	0.19983149	CIZ1	CDKN1A interacting zinc finger protein 1
1553611_s_at	7.65E-03	0.34947473	KLHL35	kelch like family member 35
218277_s_at	7.66E-03	-0.33048935	DHX40	DEAH-box helicase 40
210594_x_at	7.67E-03	0.20586574	MPZL1	myelin protein zero like 1
239845_at	7.67E-03	0.16334241		
1570349_at	7.70E-03	-0.09048542		
204103_at	7.71E-03	-0.38812836	CCL4	C-C motif chemokine ligand 4
225780_at	7.71E-03	-0.19774924	DDI2///RSC1A1	DNA damage inducible 1 homolog 2///regulatory solute carrier protein, family 1, member 1
1556538_at	7.72E-03	0.13159421	MELTF	melanotransferrin
211113_s_at	7.74E-03	0.2551816	ABCG1	ATP binding cassette subfamily G member 1
211190_x_at	7.74E-03	-0.12304363	CD84	CD84 molecule
226171_at	7.74E-03	0.31854743	ZDHHC3	zinc finger DHHC-type containing 3
214738_s_at	7.75E-03	0.10785825	NEK9	NIMA related kinase 9
211240_x_at	7.76E-03	0.287071	TMX2-CTNND1///CTNND1	TMX2-CTNND1 readthrough (NMD candidate)///catenin delta 1
214117_s_at	7.77E-03	0.26129733	BTD	biotinidase
241068_at	7.77E-03	-0.12534676	IGSF6	immunoglobulin superfamily member 6
212714_at	7.77E-03	-0.21569686	LARP4	La ribonucleoprotein domain family member 4
230050_at	7.77E-03	0.11428006	NACC2	NACC family member 2
216340_s_at	7.79E-03	0.10608717	CYP2A7P1	cytochrome P450 family 2 subfamily A member 7 pseudogene 1
240229_at	7.79E-03	-0.1178845		
210576_at	7.80E-03	0.11546136	CYP4F8	cytochrome P450 family 4 subfamily F member 8
225970_at	7.81E-03	-0.32368316	DDHD1	DDHD domain containing 1
202726_at	7.81E-03	0.23831153	LIG1	DNA ligase 1
207383_s_at	7.81E-03	0.14914394	RHBDL1	rhomboid like 1
1553588_at	7.82E-03	0.08403531	SH3KBP1///ND3	SH3 domain containing kinase binding protein 1///NADH dehydrogenase, subunit 3 (complex I)
237766_at	7.83E-03	0.1195535	ATP9B	ATPase phospholipid transporting 9B (putative)
1557541_at	7.84E-03	-0.16999673	FAM201A	family with sequence similarity 201 member A
210692_s_at	7.84E-03	0.21788871	SLC43A3	solute carrier family 43 member 3
234753_x_at	7.84E-03	-0.15880824		
210620_s_at	7.85E-03	0.13476601	GTF3C2	general transcription factor IIIC subunit 2
1569193_at	7.85E-03	-0.07540881		
203032_s_at	7.86E-03	0.17102035	FH	fumarate hydratase
205692_s_at	7.87E-03	-0.34917522	CD38	CD38 molecule
226666_at	7.87E-03	-0.29964274	DAAM1	dishevelled associated activator of morphogenesis 1
211358_s_at	7.89E-03	0.16639886	CIZ1	CDKN1A interacting zinc finger protein 1
233328_x_at	7.89E-03	0.17101465	SLC17A9	solute carrier family 17 member 9
33736_at	7.89E-03	0.21911628	STOML1	stomatin like 1
1555724_s_at	7.89E-03	0.18998799	TAGLN	transgelin
207688_s_at	7.90E-03	-0.26657381	INHBC	inhibin beta C subunit
236280_at	7.90E-03	-0.4271512	P2RY10	purinergic receptor P2Y10
208995_s_at	7.90E-03	-0.18561366	PPIG	peptidylprolyl isomerase G

203260_at	7.92E-03	-0.28690209	HDDC2	HD domain containing 2
215139_at	7.93E-03	-0.09569299	ARHGEF10	Rho guanine nucleotide exchange factor 10
226306_at	7.93E-03	0.12073582	C6orf1	chromosome 6 open reading frame 1
203954_x_at	7.93E-03	0.34265399	CLDN3	claudin 3
212781_at	7.93E-03	-0.17690531	RBBP6	RB binding protein 6, ubiquitin ligase
232209_x_at	7.94E-03	0.13018448	HM13	histocompatibility minor 13
227129_x_at	7.94E-03	0.30095946	LINC01000	long intergenic non-protein coding RNA 1000
40640_at	7.94E-03	0.08786877	NCAPH2	non-SMC condensin II complex subunit H2
238328_at	7.95E-03	0.08230736	MPRIP	myosin phosphatase Rho interacting protein
204246_s_at	7.96E-03	0.23417435	DCTN3	dynactin subunit 3
1568787_at	7.97E-03	-0.12286786	LOC100506083	uncharacterized LOC100506083
211249_at	7.99E-03	0.11148689	GPR68	G protein-coupled receptor 68
228521_s_at	7.99E-03	0.20924914	MIA-RAB4B///RAB4B	MIA-RAB4B readthrough (NMD candidate)///RAB4B, member RAS oncogene family
1559496_at	7.99E-03	-0.3038537	PPA2	pyrophosphatase (inorganic) 2
244090_at	7.99E-03	-0.10281569		
209793_at	8.00E-03	-0.08541711	GRIA1	glutamate ionotropic receptor AMPA type subunit 1
228254_at	8.00E-03	-0.22455232	STAM2	signal transducing adaptor molecule 2
213667_at	8.01E-03	0.22635222	TMEM265///SRCAP	transmembrane protein 265///Snf2-related CREBBP activator protein
228279_s_at	8.01E-03	-0.11665034		
219223_at	8.02E-03	0.13504592	CACFD1	calcium channel flower domain containing 1
205012_s_at	8.02E-03	0.2053266	HAGH	hydroxyacylglutathione hydrolase
203933_at	8.02E-03	0.22179917	RAB11FIP3	RAB11 family interacting protein 3
205102_at	8.02E-03	0.17577564	TMPRSS2	transmembrane protease, serine 2
238055_at	8.03E-03	-0.21444566	LOC100505549	uncharacterized LOC100505549
222469_s_at	8.03E-03	0.19954299	TOLLIP	toll interacting protein
1559795_at	8.03E-03	0.09268298		
244408_at	8.04E-03	-0.09469732		
211099_s_at	8.05E-03	0.08830967	CNGB1	cyclic nucleotide gated channel beta 1
209416_s_at	8.05E-03	0.10447452	FZR1	fizzy/cell division cycle 20 related 1
223708_at	8.06E-03	0.0907709	C1QTNF4	C1q and tumor necrosis factor related protein 4
205247_at	8.06E-03	0.14468493	NOTCH4	notch 4
212165_at	8.06E-03	0.22120286	TMEM183B///TMEM183A	transmembrane protein 183B///transmembrane protein 183A
234735_s_at	8.06E-03	0.2134099	USP21	ubiquitin specific peptidase 21
202518_at	8.07E-03	0.24053135	BCL7B	BCL tumor suppressor 7B
228007_at	8.07E-03	-0.41057504	CEP85L	centrosomal protein 85 like
222164_at	8.07E-03	-0.15428648	FGFR1	fibroblast growth factor receptor 1
243640_x_at	8.09E-03	-0.14944364		
230921_s_at	8.10E-03	-0.35088928	MAP3K12	mitogen-activated protein kinase kinase kinase 12
234499_at	8.10E-03	0.09797766		
240292_x_at	8.11E-03	-0.067741	ANKS1B	ankyrin repeat and sterile alpha motif domain containing 1B
210399_x_at	8.11E-03	0.3245712	FUT6	fucosyltransferase 6
242776_at	8.11E-03	0.16284174	ZCCHC6	zinc finger CCHC-type containing 6
239817_at	8.11E-03	0.11968123		
212969_x_at	8.12E-03	0.16435968	EML3	echinoderm microtubule associated protein like 3
215923_s_at	8.12E-03	0.13599321	PSD4	pleckstrin and Sec7 domain containing 4
235911_at	8.13E-03	0.59395836	MELTF	melanotransferrin
226073_at	8.13E-03	-0.22526476	TMEM218	transmembrane protein 218
237009_at	8.15E-03	-0.30444033		
209807_s_at	8.17E-03	0.11689227	NFIX	nuclear factor I X
1552727_s_at	8.18E-03	0.39163145	ADAMTS17	ADAM metallopeptidase with thrombospondin type 1 motif 17
216283_s_at	8.18E-03	0.13844273	PVR	poliovirus receptor
212005_at	8.18E-03	0.23079911	SZRD1	SUZ RNA binding domain containing 1
232980_at	8.19E-03	-0.20849922	LMBRD1	LMBR1 domain containing 1
209214_s_at	8.20E-03	0.17966897	EWSR1	EWS RNA binding protein 1
205947_s_at	8.20E-03	0.08490175	VIPR2	vasoactive intestinal peptide receptor 2
227071_at	8.20E-03	0.09708676	ZNF414	zinc finger protein 414
237914_s_at	8.20E-03	-0.14637795		
222214_at	8.20E-03	-0.28097376		

201315_x_at	8.21E-03	0.34483636	IFITM2	interferon induced transmembrane protein 2
222598_s_at	8.21E-03	0.13918398	NAV2	neuron navigator 2
211097_s_at	8.21E-03	0.10020918	PBX2	PBX homeobox 2
201194_at	8.21E-03	0.38706325	SEPW1	selenoprotein W, 1
242041_at	8.22E-03	-0.248772	CSPP1	centrosome and spindle pole associated protein 1
201561_s_at	8.24E-03	0.24699407	CLSTN1	calsyntenin 1
200991_s_at	8.24E-03	0.23104114	SNX17	sorting nexin 17
244398_x_at	8.25E-03	-0.23382102	ZNF684	zinc finger protein 684
211624_s_at	8.26E-03	0.08419387	DRD2	dopamine receptor D2
230790_x_at	8.26E-03	-0.21941542	FOXN3	forkhead box N3
1569462_x_at	8.26E-03	0.08514636	KCNT1	potassium sodium-activated channel subfamily T member 1
233252_s_at	8.26E-03	-0.20558706	STRBP	spermatid perinuclear RNA binding protein
204133_at	8.27E-03	0.27020785	RRP9	ribosomal RNA processing 9, small subunit (SSU) processome component, homolog (yeast)
243352_at	8.30E-03	-0.41327933	ALPK1	alpha kinase 1
210277_at	8.30E-03	-0.40521402	AP4S1	adaptor related protein complex 4 sigma 1 subunit
209731_at	8.30E-03	0.28073498	NTHL1	nth like DNA glycosylase 1
220772_at	8.31E-03	-0.07081028	BPESC1	blepharophimosis, epicanthus inversus and ptosis, candidate 1 (non-protein coding)
218678_at	8.32E-03	0.46366999	NES	nestin
240176_at	8.32E-03	0.28692249		
229117_s_at	8.33E-03	0.09299953	JUND	JunD proto-oncogene, AP-1 transcription factor subunit
217507_at	8.33E-03	0.09628939	SLC11A1	solute carrier family 11 member 1
207326_at	8.35E-03	-0.20833313	BTC	betacellulin
223991_s_at	8.35E-03	0.25096486	GALNT2	polypeptide N-acetylgalactosaminyltransferase 2
244284_at	8.35E-03	0.0984391		
219560_at	8.36E-03	-0.21295878	C22orf29///GNB1L	chromosome 22 open reading frame 29///G protein subunit beta 1 like
208820_at	8.36E-03	-0.22609939	PTK2	protein tyrosine kinase 2
218686_s_at	8.36E-03	0.32398812	RHBDF1	rhomboid 5 homolog 1
229758_at	8.37E-03	-0.16367585	TIGD5	tigger transposable element derived 5
223131_s_at	8.38E-03	0.09665006	TRIM8	tripartite motif containing 8
1554703_at	8.39E-03	-0.30145952	ARHGEF10	Rho guanine nucleotide exchange factor 10
232202_at	8.39E-03	-0.50283892	FAM83B	family with sequence similarity 83 member B
238725_at	8.39E-03	-0.38450691	IRF1	interferon regulatory factor 1
229189_s_at	8.39E-03	-0.37980214	LOC105374366	uncharacterized LOC105374366
1556464_a_at	8.40E-03	0.10784361	C2orf72	chromosome 2 open reading frame 72
206920_s_at	8.40E-03	0.11621969	GLE1	GLE1, RNA export mediator
231365_at	8.40E-03	0.09541189	HOXA10-AS///MIR196B	HOXA10 antisense RNA///microRNA 196b
220178_at	8.40E-03	0.23577157	MFSD12	major facilitator superfamily domain containing 12
207226_at	8.41E-03	0.10445716	HIST1H2BN	histone cluster 1, H2bn
231613_at	8.41E-03	-0.12993402	MARK1	microtubule affinity regulating kinase 1
203918_at	8.41E-03	0.14845076	PCDH1	protocadherin 1
240547_at	8.41E-03	-0.14826869		
217543_s_at	8.43E-03	0.14804553	MBTPS1	membrane bound transcription factor peptidase, site 1
234749_s_at	8.43E-03	0.19068759	POC1A	POC1 centriolar protein A
219885_at	8.43E-03	-0.40553614	SLFN12	schlafen family member 12
234051_at	8.43E-03	0.13939904		
243882_at	8.43E-03	-0.22546727		
209840_s_at	8.44E-03	-0.17611746	LRRN3	leucine rich repeat neuronal 3
234835_at	8.45E-03	-0.09535225	LOC100506667	uncharacterized LOC100506667
216878_x_at	8.46E-03	0.08094319	HAB1	B1 for mucin
1553851_at	8.46E-03	-0.06381167	SPIC	Spi-C transcription factor
213435_at	8.47E-03	-0.64805764	SATB2	SATB homeobox 2
236472_at	8.47E-03	-0.28773283		
211658_at	8.48E-03	0.15031738	PRDX2	peroxiredoxin 2
232080_at	8.49E-03	-0.36027572	HECW2	HECT, C2 and WW domain containing E3 ubiquitin protein ligase 2
227874_at	8.50E-03	-0.27654196	EMCN	endomucin
204774_at	8.50E-03	-0.5226792	EVI2A	ecotropic viral integration site 2A
1552515_at	8.50E-03	0.08899221	HIPK1	homeodomain interacting protein kinase 1
234349_at	8.50E-03	0.07500188	SSPO	SCO-spondin

226852_at	8.51E-03	0.20142133	MTA3	metastasis associated 1 family member 3
209761_s_at	8.51E-03	0.18494925	SP110	SP110 nuclear body protein
241637_at	8.51E-03	-0.1435048		
203488_at	8.52E-03	0.14692616	ADGRL1	adhesion G protein-coupled receptor L1
209733_at	8.52E-03	-0.31722546	MID2	midline 2
1554148_a_at	8.52E-03	0.21519761	SLC33A1	solute carrier family 33 member 1
1565701_at	8.52E-03	-0.17851142		
200948_at	8.54E-03	0.28754322	MLF2	myeloid leukemia factor 2
212117_at	8.54E-03	-0.19906433	RHOQ	ras homolog family member Q
228786_at	8.54E-03	0.15781919	SVIL-AS1///PTCHD3P1	SVIL antisense RNA 1///patched domain containing 3 pseudogene 1
1558537_x_at	8.54E-03	0.07142547	ZNF844	zinc finger protein 844
243466_at	8.54E-03	0.0669112		
221297_at	8.55E-03	0.10506516	GPRC5D	G protein-coupled receptor class C group 5 member D
209989_at	8.55E-03	-0.35515599	ZNF268	zinc finger protein 268
1563139_at	8.55E-03	0.07352162		
201653_at	8.56E-03	-0.21441837	CNIH1	cornichon family AMPA receptor auxiliary protein 1
241928_at	8.56E-03	-0.22529335		
228678_at	8.57E-03	0.1259438	DENND6B	DENN domain containing 6B
207258_at	8.57E-03	0.09069333	DSCR4	Down syndrome critical region 4
215816_at	8.57E-03	-0.09455394	GUSBP11	glucuronidase, beta pseudogene 11
1569176_at	8.57E-03	-0.07342174	TMPRSS12	transmembrane protease, serine 12
221812_at	8.58E-03	-0.17467738	FBXO42	F-box protein 42
219779_at	8.58E-03	0.08268335	ZFHX4	zinc finger homeobox 4
204116_at	8.59E-03	0.47797749	IL2RG	interleukin 2 receptor subunit gamma
233947_s_at	8.60E-03	0.06710852	TBX5-AS1	TBX5 antisense RNA 1
217799_x_at	8.60E-03	0.16742488	UBE2H	ubiquitin conjugating enzyme E2 H
202323_s_at	8.61E-03	0.33966461	ACBD3	acyl-CoA binding domain containing 3
229467_at	8.62E-03	-0.24155716	PCBP2	poly(rC) binding protein 2
218145_at	8.62E-03	0.41199926	TRIB3	tribbles pseudokinase 3
1569202_x_at	8.63E-03	-0.14008339		
238101_at	8.64E-03	-0.08613686	BEND4	BEN domain containing 4
243236_at	8.64E-03	-0.24064785		
227653_at	8.66E-03	-0.26559656	TRMT5	tRNA methyltransferase 5
235373_at	8.67E-03	-0.37930128	LOC100506314	uncharacterized LOC100506314
38398_at	8.68E-03	0.13860684	MADD	MAP kinase activating death domain
234087_at	8.68E-03	-0.09313844		
203858_s_at	8.69E-03	0.18533194	COX10	COX10, heme A:farnesyltransferase cytochrome c oxidase assembly factor
240456_at	8.69E-03	0.1242743	LOC101928419	uncharacterized LOC101928419
213585_s_at	8.69E-03	-0.12141158	PDCD2	programmed cell death 2
243530_at	8.69E-03	-0.06757041		
221361_at	8.70E-03	0.10338462	OMP	olfactory marker protein
216583_x_at	8.71E-03	0.18759602		
1555829_at	8.72E-03	0.14515172	ESYT2	extended synaptotagmin 2
222152_at	8.73E-03	0.20133691	PDCD6	programmed cell death 6
232775_at	8.73E-03	0.07530628		
205260_s_at	8.74E-03	-0.22206876	ACYP1	acylphosphatase 1
1553279_at	8.75E-03	0.11134731	BTNL9	butyrophilin like 9
218938_at	8.75E-03	0.22008392	FBXL15	F-box and leucine rich repeat protein 15
234142_at	8.75E-03	-0.08397959		
37547_at	8.76E-03	-0.32805091	BBS9	Bardet-Biedl syndrome 9
214020_x_at	8.76E-03	0.16183334	ITGB5	integrin subunit beta 5
1558787_a_at	8.76E-03	0.07081176	TSPAN3	tetraspanin 3
231219_at	8.77E-03	0.19958858	CMTM1	CKLF like MARVEL transmembrane domain containing 1
222961_at	8.77E-03	-0.10185868	SERINC2	serine incorporator 2
201390_s_at	8.78E-03	0.33705472	CSNK2B	casein kinase 2 beta
219456_s_at	8.78E-03	0.0774527	RIN3	Ras and Rab interactor 3
213775_x_at	8.78E-03	-0.17971364	ZNF638-IT1///ZNF638	ZNF638 intronic transcript 1///zinc finger protein 638
224885_s_at	8.79E-03	0.22628665	KRTCAP2	keratinocyte associated protein 2
206606_at	8.81E-03	0.43189169	LIPC	lipase C, hepatic type
201526_at	8.82E-03	0.24888766	ARF5	ADP ribosylation factor 5
227162_at	8.82E-03	-0.25735159	ZBTB26	zinc finger and BTB domain containing 26

48580_at	8.83E-03	0.20075787	CXXC1	CXXC finger protein 1
229472_at	8.83E-03	0.08534469	SFT2D3///WDR33	SFT2 domain containing 3///WD repeat domain 33
227358_at	8.83E-03	0.12355109	ZBTB46	zinc finger and BTB domain containing 46
204220_at	8.84E-03	-0.33407529	GMFG	glia maturation factor gamma
204828_at	8.85E-03	0.19695096	RAD9A	RAD9 checkpoint clamp component A
231576_at	8.85E-03	-0.32090211		
203478_at	8.86E-03	0.20901508	NDUFC1	NADH:ubiquinone oxidoreductase subunit C1
235227_at	8.86E-03	-0.33295585	STXBP5	syntaxin binding protein 5
223153_x_at	8.86E-03	0.18118154	TMUB1	transmembrane and ubiquitin like domain containing 1
240855_at	8.86E-03	0.11539937		
229640_x_at	8.87E-03	-0.22860059	LINC00621	long intergenic non-protein coding RNA 621
1559343_at	8.87E-03	-0.23731392	LOC101930404///SNORD116-28///SNORD115-26///SNORD115-13///SNORD115-7///SNORD116-22///SNORD116-4///PWARSN///SNORD107///SNRPN///IPW	uncharacterized LOC101930404///small nucleolar RNA, C/D box 116-28///small nucleolar RNA, C/D box 115-26///small nucleolar RNA, C/D box 115-13///small nucleolar RNA, C/D box 115-7///small nucleolar RNA, C/D box 116-22///small nucleolar RNA, C/D box 116-4///Prader Willi/Angelman region RNA, SNRPN neighbor///small nucleolar RNA, C/D box 107///small nuclear ribonucleoprotein polypeptide N///imprinted in Prader-Willi syndrome (non-protein coding)
204346_s_at	8.87E-03	0.20499838	RASSF1	Ras association domain family member 1
216409_at	8.88E-03	0.12339966	ACSL6	acyl-CoA synthetase long-chain family member 6
212441_at	8.88E-03	-0.21772449	KIAA0232	KIAA0232
235818_at	8.88E-03	-0.07392789	VSTM1	V-set and transmembrane domain containing 1
229089_at	8.88E-03	-0.12536799	ZBTB49	zinc finger and BTB domain containing 49
213858_at	8.88E-03	-0.26017232	ZNF250	zinc finger protein 250
202153_s_at	8.89E-03	0.20584946	NUP62	nucleoporin 62
219448_at	8.89E-03	-0.23895406	TMEM70	transmembrane protein 70
226307_at	8.90E-03	0.17899825	CRTC2	CREB regulated transcription coactivator 2
1557051_s_at	8.90E-03	0.35550137	HOTAIRM1	HOXA transcript antisense RNA, myeloid-specific 1
204911_s_at	8.90E-03	0.07846858	TRIM3	tripartite motif containing 3
224153_s_at	8.91E-03	-0.08896253	DHRS4-AS1	DHRS4 antisense RNA 1
1559650_at	8.91E-03	-0.07290674	JAZF1-AS1	JAZF1 antisense RNA 1
207305_s_at	8.91E-03	-0.20932294	TRAPPC8	trafficking protein particle complex 8
220723_s_at	8.92E-03	-0.62962764	CWH43	cell wall biogenesis 43 C-terminal homolog
235052_at	8.93E-03	0.30120444	ZNF792	zinc finger protein 792
232913_at	8.95E-03	0.09003267	TMED8	transmembrane p24 trafficking protein family member 8
238274_at	8.95E-03	0.08001574		
201933_at	8.96E-03	0.21104539	CHMP1A	charged multivesicular body protein 1A
1553450_s_at	8.96E-03	0.06502545	LINC00304	long intergenic non-protein coding RNA 304
212309_at	8.98E-03	-0.2307844	CLASP2	cytoplasmic linker associated protein 2
200739_s_at	8.98E-03	0.2165836	SUMO3	small ubiquitin-like modifier 3
227392_at	8.99E-03	0.13343582	NISCH	nischarin
207196_s_at	8.99E-03	0.22784503	TNIP1	TNFAIP3 interacting protein 1
202997_s_at	9.00E-03	0.09425479	LOXL2	lysyl oxidase like 2
222154_s_at	9.00E-03	0.21682466	SPATS2L	spermatogenesis associated serine rich 2 like
238469_at	9.01E-03	-0.15551448	OGFRL1	opioid growth factor receptor like 1
210598_at	9.01E-03	-0.17917555		
202226_s_at	9.03E-03	0.15433914	CRK	CRK proto-oncogene, adaptor protein
212917_x_at	9.03E-03	-0.29729709	RECQL	RecQ like helicase
209182_s_at	9.04E-03	0.14756797	C10orf10	chromosome 10 open reading frame 10
202500_at	9.05E-03	0.22972104	DNAJB2	DnaJ heat shock protein family (Hsp40) member B2
240131_at	9.05E-03	0.08945133		
204844_at	9.06E-03	-0.46697771	ENPEP	glutamyl aminopeptidase
203752_s_at	9.06E-03	0.2244717	JUND	JunD proto-oncogene, AP-1 transcription factor subunit
231896_s_at	9.07E-03	-0.19191465	DENR	density regulated re-initiation and release factor
229190_at	9.07E-03	-0.39082196	LOC105374366	uncharacterized LOC105374366
234978_at	9.07E-03	-0.35670643	SLC36A4	solute carrier family 36 member 4
203668_at	9.08E-03	0.31106906	MAN2C1	mannosidase alpha class 2C member 1
223658_at	9.09E-03	0.28887912	KCNK6	potassium two pore domain channel subfamily K member 6
214470_at	9.11E-03	-0.56286608	KLRB1	killer cell lectin like receptor B1
231861_at	9.13E-03	0.26193684	LRP10	LDL receptor related protein 10

1558407_at	9.13E-03	0.06445201	PLEKHG2	pleckstrin homology and RhoGEF domain containing G2
1567213_at	9.13E-03	-0.2389845	PNN	pinin, desmosome associated protein
202102_s_at	9.14E-03	0.21111524	BRD4	bromodomain containing 4
1565483_at	9.14E-03	-0.44527461	EGFR	epidermal growth factor receptor
243611_at	9.14E-03	0.19281306	MICALCL	MICAL C-terminal like
235247_at	9.15E-03	-0.34630714		
211160_x_at	9.17E-03	0.26685553	ACTN1	actinin alpha 1
233688_at	9.17E-03	-0.06703512	KCNIP3	potassium voltage-gated channel interacting protein 3
220789_s_at	9.17E-03	0.24698603	SNORA5B///TBRG4	small nucleolar RNA, H/ACA box 5B///transforming growth factor beta regulator 4
229533_x_at	9.17E-03	-0.3362266	ZNF680	zinc finger protein 680
1561092_at	9.17E-03	0.08637043		
206593_s_at	9.18E-03	0.21322661	MED22	mediator complex subunit 22
1563608_a_at	9.19E-03	0.07340609	KCNT1	potassium sodium-activated channel subfamily T member 1
244804_at	9.20E-03	0.29114258	SQSTM1	sequestosome 1
212668_at	9.21E-03	0.12472817	SMURF1	SMAD specific E3 ubiquitin protein ligase 1
240548_at	9.21E-03	-0.08632075		
219654_at	9.23E-03	-0.58909729	HACD1	3-hydroxyacyl-CoA dehydratase 1
1557399_at	9.24E-03	0.12783206	LOC284009///METTL16	uncharacterized LOC284009///methyltransferase like 16
226178_at	9.24E-03	-0.22625163	SOCS4	suppressor of cytokine signaling 4
211715_s_at	9.25E-03	0.26288374	BDH1	3-hydroxybutyrate dehydrogenase, type 1
1564520_s_at	9.25E-03	-0.27762934	PRMT5	protein arginine methyltransferase 5
201419_at	9.26E-03	0.22683885	BAP1	BRCA1 associated protein 1
210656_at	9.26E-03	-0.35779757	EED	embryonic ectoderm development
218406_x_at	9.26E-03	-0.10494364	NENF	neudesin neurotrophic factor
202115_s_at	9.26E-03	0.20159487	NOC2L	NOC2 like nucleolar associated transcriptional repressor
1552708_a_at	9.28E-03	-0.10465764	DUSP19	dual specificity phosphatase 19
201125_s_at	9.29E-03	0.21655508	ITGB5	integrin subunit beta 5
227750_at	9.29E-03	0.31180629	KALRN	kalirin, RhoGEF kinase
232536_at	9.29E-03	0.13596729	LRRC3///LRRC3DN	leucine rich repeat containing 3///LRRC3 downstream neighbor (non-protein coding)
205409_at	9.30E-03	0.13151846	FOSL2	FOS like 2, AP-1 transcription factor subunit
217300_at	9.31E-03	-0.09652193		
210733_at	9.32E-03	-0.26894872	TRAM1	translocation associated membrane protein 1
209665_at	9.33E-03	0.23790132	CYB561D2	cytochrome b561 family member D2
1560665_at	9.33E-03	-0.09532682	SCPEP1	serine carboxypeptidase 1
AFFX-HUMRGE/M1	9.33E-03	0.17534348		
213948_x_at	9.34E-03	0.09986287	CADM3	cell adhesion molecule 3
206468_s_at	9.34E-03	0.1880591	METTL13	methyltransferase like 13
211119_at	9.35E-03	0.06327768	ESR2	estrogen receptor 2
230098_at	9.35E-03	-0.31641831	PHF20L1	PHD finger protein 20-like 1
209339_at	9.35E-03	0.23071308	SIAH2	siah E3 ubiquitin protein ligase 2
231157_at	9.35E-03	0.13777605	TTLL11	tubulin tyrosine ligase like 11
217297_s_at	9.36E-03	0.13890306	MYO9B	myosin IXB
1554286_at	9.37E-03	0.10365778	FLJ25758	microtubule affinity regulating kinase 1 pseudogene
209577_at	9.37E-03	0.23531447	PCYT2	phosphate cytidylyltransferase 2, ethanolamine
227980_at	9.37E-03	-0.25544683	ZNF322	zinc finger protein 322
235190_at	9.37E-03	0.24795283		
226785_at	9.39E-03	-0.34698133	ATP11C	ATPase phospholipid transporting 11C
209539_at	9.40E-03	-0.30559117	ARHGEF6	Rac/Cdc42 guanine nucleotide exchange factor 6
241629_at	9.40E-03	-0.09495769		
207253_s_at	9.42E-03	0.13878942	UBN1	ubinuclein 1
210320_s_at	9.43E-03	-0.27485659	DDX52	DEAD-box helicase 52
210827_s_at	9.43E-03	0.28177135	ELF3	E74 like ETS transcription factor 3
240798_at	9.43E-03	-0.34871619		
220998_s_at	9.46E-03	0.18022172	UNC93B1	unc-93 homolog B1 (C. elegans)
218541_s_at	9.47E-03	-0.70181039	C8orf4	chromosome 8 open reading frame 4
204635_at	9.47E-03	-0.37398859	RPS6KA5	ribosomal protein S6 kinase A5
235035_at	9.48E-03	0.17602975	SLC35E1	solute carrier family 35 member E1
241412_at	9.49E-03	-0.44204609	BTC	betacellulin
1569602_at	9.49E-03	0.08711359	KANK2	KN motif and ankyrin repeat domains 2

203169_at	9.49E-03	0.1648115	RGP1	RGP1 homolog, RAB6A GEF complex partner 1
223023_at	9.52E-03	0.18656338	BET1L	Bet1 golgi vesicular membrane trafficking protein like
1558827_a_at	9.52E-03	-0.07566035	ZNF831	zinc finger protein 831
238987_at	9.53E-03	0.09892491	B4GALT1	beta-1,4-galactosyltransferase 1
228577_x_at	9.54E-03	-0.3298038	ODF2L	outer dense fiber of sperm tails 2 like
229022_at	9.54E-03	-0.25327341	ZFX	zinc finger protein, X-linked
1552914_a_at	9.55E-03	0.11365518	CD276	CD276 molecule
204282_s_at	9.55E-03	-0.15138812	FARS2	phenylalanyl-tRNA synthetase 2, mitochondrial
208639_x_at	9.55E-03	0.26469222	PDIA6	protein disulfide isomerase family A member 6
208830_s_at	9.55E-03	0.16542607	SUPT6H	SPT6 homolog, histone chaperone
214755_at	9.55E-03	0.14838914	UAP1L1	UDP-N-acetylglucosamine pyrophosphorylase 1 like 1
221587_s_at	9.56E-03	0.20219386	C19orf24	chromosome 19 open reading frame 24
236191_at	9.56E-03	-0.21054119		
217476_at	9.57E-03	-0.09909552	NR1D1///THRA	nuclear receptor subfamily 1 group D member 1///thyroid hormone receptor, alpha
231252_at	9.58E-03	-0.39087147	KANSL1L	KAT8 regulatory NSL complex subunit 1 like
210665_at	9.59E-03	-0.5172846	TFPI	tissue factor pathway inhibitor
238654_at	9.59E-03	0.26207415	VSIG10L	V-set and immunoglobulin domain containing 10 like
224030_s_at	9.60E-03	-0.16938638	TCAF1	TRPM8 channel associated factor 1
236343_at	9.60E-03	0.22331336		
236674_at	9.62E-03	0.22025678	LOC388780	uncharacterized LOC388780
225955_at	9.63E-03	0.39497379	METRNL	meteorin like, glial cell differentiation regulator
206530_at	9.63E-03	0.08919731	RAB30	RAB30, member RAS oncogene family
213483_at	9.64E-03	-0.24742635	PPWD1	peptidylprolyl isomerase domain and WD repeat containing 1
205890_s_at	9.64E-03	-0.86102675	UBD///GABBR1	ubiquitin D///gamma-aminobutyric acid type B receptor subunit 1
1556065_at	9.68E-03	-0.10107955	CDPF1	cysteine rich DPF motif domain containing 1
203239_s_at	9.68E-03	0.14736605	CNOT3	CCR4-NOT transcription complex subunit 3
218687_s_at	9.68E-03	0.28646434	MUC13	mucin 13, cell surface associated
41856_at	9.68E-03	0.09928071	UNC5B	unc-5 netrin receptor B
233798_at	9.68E-03	-0.07110844		
203047_at	9.69E-03	0.17067677	STK10	serine/threonine kinase 10
224527_at	9.70E-03	0.0873277	LOC100653137///CDH23	cadherin-23-like///cadherin-related 23
219189_at	9.71E-03	0.35644394	FBXL6	F-box and leucine rich repeat protein 6
233405_at	9.71E-03	-0.38490398		
229018_at	9.72E-03	-0.28525412	METTL25	methyltransferase like 25
237744_at	9.72E-03	0.1182777		
1568754_at	9.73E-03	-0.10716247	LOC101927814	uncharacterized LOC101927814
228139_at	9.73E-03	0.19390397	RIPK3	receptor interacting serine/threonine kinase 3
217991_x_at	9.73E-03	0.15199171	SSBP3	single stranded DNA binding protein 3
203198_at	9.74E-03	0.25419681	CDK9	cyclin dependent kinase 9
235177_at	9.74E-03	-0.30759294	METTL21A	methyltransferase like 21A
228542_at	9.75E-03	-0.26449773	MRS2	MRS2, magnesium transporter
204571_x_at	9.75E-03	-0.19904813	PIN4	peptidylprolyl cis/trans isomerase, NIMA-interacting 4
221943_x_at	9.75E-03	-0.27062209	RPL38	ribosomal protein L38
229178_at	9.76E-03	-0.32090831	PRTG	protogenin
212918_at	9.76E-03	-0.27823358	RECQL	RecQ like helicase
222727_s_at	9.76E-03	0.10348149	SLC8B1	solute carrier family 8 member B1
216416_at	9.77E-03	0.09357807		
225341_at	9.78E-03	-0.250003	MTERF2	mitochondrial transcription termination factor 2
202959_at	9.78E-03	-0.25787516	MUT	methylmalonyl-CoA mutase
1569453_a_at	9.79E-03	0.1975362	LOC692247	uncharacterized LOC692247
234738_s_at	9.80E-03	0.17299855	KLHDC4	kelch domain containing 4
204623_at	9.80E-03	0.64398777	TFF3	trefoil factor 3
220341_s_at	9.82E-03	0.1294505	C5orf45	chromosome 5 open reading frame 45
218473_s_at	9.82E-03	0.18222479	COLGALT1	collagen beta(1-O)galactosyltransferase 1
201428_at	9.83E-03	0.34244901	CLDN4	claudin 4
213737_x_at	9.83E-03	-0.27033754	GOLGA8N	golgin A8 family member N
31637_s_at	9.83E-03	0.15850628	NR1D1///THRA	nuclear receptor subfamily 1 group D member 1///thyroid hormone receptor, alpha
211822_s_at	9.84E-03	0.10835713	NLRP1	NLR family pyrin domain containing 1
1558142_at	9.84E-03	-0.31263879	TNRC6B	trinucleotide repeat containing 6B

205801_s_at	9.86E-03	-0.31514413	RASGRP3	RAS guanyl releasing protein 3
220221_at	9.86E-03	-0.29262364	VPS13D	vacuolar protein sorting 13 homolog D
1559713_at	9.86E-03	0.08274046		
218429_s_at	9.87E-03	0.17964424	C19orf66	chromosome 19 open reading frame 66
236350_at	9.87E-03	-0.22556578		
217020_at	9.89E-03	-0.09246072	RARB	retinoic acid receptor beta
1553338_at	9.89E-03	0.29136224	SDE2	SDE2 telomere maintenance homolog
209100_at	9.90E-03	0.24345959	IFRD2	interferon related developmental regulator 2
237196_at	9.91E-03	0.08145976	LOC100506558///MATN2	uncharacterized LOC100506558///matrilin 2
217040_x_at	9.91E-03	-0.14029098	SOX15	SRY-box 15
219261_at	9.92E-03	0.19634853	C7orf26	chromosome 7 open reading frame 26
217349_s_at	9.92E-03	0.10590899	PRICKLE3	prickle planar cell polarity protein 3
205920_at	9.92E-03	0.13998169	SLC6A6	solute carrier family 6 member 6
211162_x_at	9.93E-03	0.28158165	SCD	stearoyl-CoA desaturase
242982_x_at	9.94E-03	-0.2413476	ITGB8	integrin subunit beta 8
1559131_a_at	9.94E-03	-0.11161693	LOC101928047	uncharacterized LOC101928047
211100_x_at	9.95E-03	0.08681383	LILRA2	leukocyte immunoglobulin like receptor A2
204765_at	9.96E-03	0.19739484	ARHGEF5	Rho guanine nucleotide exchange factor 5
209787_s_at	9.96E-03	-0.18205023	HMGN4	high mobility group nucleosomal binding domain 4
202757_at	9.96E-03	0.21539286	NELFB	negative elongation factor complex member B
224964_s_at	9.97E-03	-0.39615526	GNG2	G protein subunit gamma 2
203111_s_at	9.97E-03	0.10749583	PTK2B	protein tyrosine kinase 2 beta
225282_at	9.97E-03	0.19578065	SMAP2	small ArfGAP2
200096_s_at	9.99E-03	0.27078973	ATP6V0E1	ATPase H+ transporting V0 subunit e1
1557385_at	1.00E-02	-0.17378394	FAM161A	family with sequence similarity 161 member A
225151_at	1.00E-02	0.08321572	RTKN	rhotekin
204062_s_at	1.00E-02	-0.35542808	ULK2	unc-51 like autophagy activating kinase 2
205667_at	1.00E-02	-0.34675063	WRN	Werner syndrome RecQ like helicase
244666_at	1.00E-02	0.09537446		
206170_at	1.01E-02	-0.31068923	ADRB2	adrenoceptor beta 2
203258_at	1.01E-02	0.24108438	DRAP1	DR1 associated protein 1
223858_at	1.01E-02	0.08874032	ESRRB	estrogen related receptor beta
202945_at	1.01E-02	0.20703951	FPGS	folylpolyglutamate synthase
236001_at	1.01E-02	0.31871848	LINC00675	long intergenic non-protein coding RNA 675
1561096_at	1.01E-02	0.20844333	LINC01091	long intergenic non-protein coding RNA 1091
219965_s_at	1.01E-02	0.10667186	MAGIX	MAGI family member, X-linked
209973_at	1.01E-02	0.1088016	NFKBIL1	NFKB inhibitor like 1
204210_s_at	1.01E-02	0.16538006	PCYT1A	phosphate cytidylyltransferase 1, choline, alpha
1564031_a_at	1.01E-02	0.19673459	RELL2	RELT like 2
1554060_s_at	1.01E-02	-0.24595165	SETMAR	SET domain and mariner transposase fusion gene
227360_at	1.01E-02	0.09395541		
207204_at	1.02E-02	0.09468873	FSCN2	fascin actin-bundling protein 2, retinal
242807_at	1.02E-02	-0.42509678	FSD1L	fibronectin type III and SPRY domain containing 1 like
229343_at	1.02E-02	-0.10098313	GTSE1	G2 and S-phase expressed 1
226352_at	1.02E-02	-0.26213648	JMY	junction mediating and regulatory protein, p53 cofactor
1570234_at	1.02E-02	-0.07268272	LINC01467	long intergenic non-protein coding RNA 1467
213761_at	1.02E-02	-0.28950861	MDM1	Mdm1 nuclear protein
217796_s_at	1.02E-02	0.18005888	NPLOC4	NPL4 homolog, ubiquitin recognition factor
225813_at	1.02E-02	-0.17990422	RC3H2	ring finger and CCCH-type domains 2
224450_s_at	1.02E-02	-0.23258222	RIOK1	RIO kinase 1
224626_at	1.02E-02	0.20448215	SLC35A4	solute carrier family 35 member A4
1553599_a_at	1.02E-02	-0.09323597	SYCP3	synaptonemal complex protein 3
202124_s_at	1.02E-02	-0.25242011	TRAK2	trafficking kinesin protein 2
221999_at	1.02E-02	0.28820682	VRK3	vaccinia related kinase 3
1555192_at	1.02E-02	-0.12129494	ZNF277	zinc finger protein 277
1557658_at	1.02E-02	-0.12243245		
230248_x_at	1.02E-02	-0.15384668		
216376_x_at	1.02E-02	0.10812582		
221421_s_at	1.03E-02	0.10154559	ADAMTS12	ADAM metallopeptidase with thrombospondin type 1 motif 12
204533_at	1.03E-02	-0.72878584	CXCL10	C-X-C motif chemokine ligand 10
203733_at	1.03E-02	0.19302709	DEXI	Dexi homolog
1554468_s_at	1.03E-02	0.11571603	FBF1	Fas binding factor 1

201656_at	1.03E-02	0.1938174	ITGA6	integrin subunit alpha 6
1558477_at	1.03E-02	0.0663056	LOC100131496	uncharacterized LOC100131496
239294_at	1.03E-02	-0.48747778	PIK3CG	phosphatidylinositol-4,5-bisphosphate 3-kinase catalytic subunit gamma
1559946_s_at	1.03E-02	0.27142526	RUVBL2	RuvB like AAA ATPase 2
200831_s_at	1.03E-02	0.25890482	SCD	stearoyl-CoA desaturase
226051_at	1.03E-02	0.44698509	SELM	selenoprotein M
1554460_at	1.03E-02	-0.07448961	ST8SIA4	ST8 alpha-N-acetyl-neuraminide alpha-2,8-sialyltransferase 4
219950_s_at	1.03E-02	-0.12928728	TIAM2	T-cell lymphoma invasion and metastasis 2
240594_at	1.03E-02	-0.31872984		
242593_at	1.03E-02	-0.25589529		
1565887_at	1.03E-02	-0.10292119		
237396_at	1.03E-02	-0.09999699		
220749_at	1.04E-02	0.13312415	CCDC7	coiled-coil domain containing 7
210742_at	1.04E-02	-0.1859973	CDC14A	cell division cycle 14A
1560599_a_at	1.04E-02	0.13040172	CEP89	centrosomal protein 89
229390_at	1.04E-02	-0.61124804	FAM26F	family with sequence similarity 26 member F
204169_at	1.04E-02	0.19175699	IMPDH1	inosine monophosphate dehydrogenase 1
212592_at	1.04E-02	-1.19228485	JCHAIN	joining chain of multimeric IgA and IgM
225506_at	1.04E-02	-0.20691651	KIAA1468	KIAA1468
202042_at	1.04E-02	0.24447608	LOC101928623///HARS	uncharacterized LOC101928623///histidyl-tRNA synthetase
226929_at	1.04E-02	0.18206951	MTHFR	methylenetetrahydrofolate reductase (NAD(P)H)
1554300_a_at	1.04E-02	-0.18097259	SVOPL	SVOP like
207554_x_at	1.04E-02	-0.09375927	TBXA2R	thromboxane A2 receptor
1561243_at	1.04E-02	0.09198502	TMEM105	transmembrane protein 105
43977_at	1.04E-02	0.18894165	TMEM161A	transmembrane protein 161A
1552769_at	1.04E-02	0.09187439	ZNF625	zinc finger protein 625
212312_at	1.05E-02	0.24910919	BCL2L1	BCL2 like 1
226916_x_at	1.05E-02	0.13066134	DPP9	dipeptidyl peptidase 9
224627_at	1.05E-02	0.20393403	GBA2	glucosylceramidase beta 2
234452_at	1.05E-02	0.1385943	HIBADH	3-hydroxyisobutyrate dehydrogenase
218870_at	1.05E-02	-0.30784379	LOC101928361///ARHGAP15	uncharacterized LOC101928361///Rho GTPase activating protein 15
213899_at	1.05E-02	-0.27929882	METAP2	methionyl aminopeptidase 2
225530_at	1.05E-02	0.20196195	MOB3A	MOB kinase activator 3A
209751_s_at	1.05E-02	0.37632617	TRAPPC2B///TRAPPC2	trafficking protein particle complex 2B///trafficking protein particle complex 2
232721_at	1.05E-02	-0.07550434	TRIM55	tripartite motif containing 55
213535_s_at	1.05E-02	0.24311979	UBE2I	ubiquitin conjugating enzyme E2 I
1559449_a_at	1.05E-02	0.21703219	ZNF254	zinc finger protein 254
207781_s_at	1.05E-02	-0.13248619	ZNF711	zinc finger protein 711
237049_at	1.05E-02	-0.07580959		
242230_at	1.06E-02	-0.22971895	ATXN1	ataxin 1
208279_s_at	1.06E-02	0.08775299	CDRT1	CMT1A duplicated region transcript 1
212942_s_at	1.06E-02	0.55686004	CEMIP	cell migration inducing hyaluronan binding protein
242141_at	1.06E-02	-0.17968044	HDAC2	histone deacetylase 2
1569287_at	1.06E-02	-0.08071999	LINC00458	long intergenic non-protein coding RNA 458
243400_x_at	1.06E-02	0.10157808	LTBR	lymphotoxin beta receptor
207883_s_at	1.06E-02	0.1122025	TFR2	transferrin receptor 2
218381_s_at	1.06E-02	0.16713754	U2AF2	U2 small nuclear RNA auxiliary factor 2
37943_at	1.06E-02	-0.17538734	ZFYVE26	zinc finger FYVE-type containing 26
224011_at	1.06E-02	0.14799305		
242563_at	1.06E-02	-0.25257082		
239359_at	1.07E-02	0.08372528	MARCHF11	membrane associated ring-CH-type finger 11
220144_s_at	1.07E-02	0.37134636	ANKEF1	ankyrin repeat and EF-hand domain containing 1
1405_i_at	1.07E-02	-0.48959239	CCL5	C-C motif chemokine ligand 5
207386_at	1.07E-02	-0.20172655	CYP7B1	cytochrome P450 family 7 subfamily B member 1
200825_s_at	1.07E-02	0.28631643	HYOU1	hypoxia up-regulated 1
229771_at	1.07E-02	0.1847275	MAP3K13	mitogen-activated protein kinase kinase kinase 13
211873_s_at	1.07E-02	0.10114605	PCDHGA9	protocadherin gamma subfamily A, 9
234647_at	1.07E-02	0.08217808	PROM2	prominin 2

216470_x_at	1.07E-02	0.33553266	PRSS2	protease, serine 2
211974_x_at	1.07E-02	-0.21930704	RBPJ	recombination signal binding protein for immunoglobulin kappa J region
209776_s_at	1.07E-02	0.09823472	SLC19A1	solute carrier family 19 member 1
240700_at	1.07E-02	0.10690194	TOR1AIP2	torsin 1A interacting protein 2
208844_at	1.07E-02	-0.13821439	VDAC3	voltage dependent anion channel 3
1570424_at	1.07E-02	0.08421339		
243467_at	1.07E-02	0.0804922		
224882_at	1.08E-02	0.34193926	ACSS1	acyl-CoA synthetase short-chain family member 1
207809_s_at	1.08E-02	0.27046242	ATP6AP1	ATPase H+ transporting accessory protein 1
234811_at	1.08E-02	0.09514134	CENPN	centromere protein N
202493_x_at	1.08E-02	0.07390541	GH1///CSHL1///CSH1	growth hormone 1///chorionic somatomammotropin hormone like 1///chorionic somatomammotropin hormone 1
1557502_at	1.08E-02	0.09869624	PCCB	propionyl-CoA carboxylase beta subunit
205628_at	1.08E-02	-0.22089891	PRIM2	primase (DNA) subunit 2
211663_x_at	1.08E-02	0.11988901	PTGDS	prostaglandin D2 synthase
208794_s_at	1.08E-02	0.24658128	SMARCA4	SWI/SNF related, matrix associated, actin dependent regulator of chromatin, subfamily a, member 4
221769_at	1.08E-02	0.17745663	SPSB3	splA/ryanodine receptor domain and SOCS box containing 3
220803_at	1.08E-02	0.27475055	STAMBPL1	STAM binding protein like 1
221747_at	1.08E-02	0.12810505	TNS1	tensin 1
241919_x_at	1.08E-02	0.0650043	WDR31	WD repeat domain 31
226668_at	1.08E-02	-0.27802654	WDSUB1	WD repeat, sterile alpha motif and U-box domain containing 1
1559949_at	1.08E-02	-0.11403111		
223951_at	1.08E-02	-0.09819724		
206397_x_at	1.09E-02	0.09624867	CERS1///GDF1	ceramide synthase 1///growth differentiation factor 1
242092_at	1.09E-02	0.25615336	EPB41L2	erythrocyte membrane protein band 4.1 like 2
203720_s_at	1.09E-02	0.28164884	ERCC1	ERCC excision repair 1, endonuclease non-catalytic subunit
230596_at	1.09E-02	0.45542037	KALRN	kalirin, RhoGEF kinase
1564109_at	1.09E-02	0.0921661	LOC284865	uncharacterized LOC284865
203195_s_at	1.09E-02	0.20021067	NUP98	nucleoporin 98
1554593_s_at	1.09E-02	-0.05276043	SLC1A6	solute carrier family 1 member 6
233141_s_at	1.09E-02	-0.14036468	ST7L	suppression of tumorigenicity 7 like
223581_at	1.09E-02	-0.14699215	ZNF577	zinc finger protein 577
1560327_at	1.09E-02	0.13569844		
232982_at	1.09E-02	-0.07964117		
236630_at	1.10E-02	0.09050404	AQP2	aquaporin 2
229589_x_at	1.10E-02	-0.18447956	BIVM	basic, immunoglobulin-like variable motif containing
217720_at	1.10E-02	0.18854696	CHCHD2	coiled-coil-helix-coiled-coil-helix domain containing 2
212793_at	1.10E-02	0.10026176	DAAM2	dishevelled associated activator of morphogenesis 2
238324_at	1.10E-02	0.07437227	GATAD2A	GATA zinc finger domain containing 2A
65493_at	1.10E-02	-0.22093115	HEATR6	HEAT repeat containing 6
243176_at	1.10E-02	-0.29471403	LOC101929356///ARL5A	uncharacterized LOC101929356///ADP ribosylation factor like GTPase 5A
212567_s_at	1.10E-02	0.15955108	MAP4	microtubule associated protein 4
217830_s_at	1.10E-02	0.33042267	NSFL1C	NSFL1 cofactor
207939_x_at	1.10E-02	0.12650348	RNPS1	RNA binding protein with serine rich domain 1
209969_s_at	1.10E-02	-0.34697754	STAT1	signal transducer and activator of transcription 1
232426_at	1.10E-02	0.12840035	SV2B	synaptic vesicle glycoprotein 2B
224721_at	1.10E-02	-0.16232972	WDR75	WD repeat domain 75
242106_at	1.10E-02	-0.16461552		
235640_at	1.10E-02	0.19473552		
200996_at	1.11E-02	0.15283458	ACTR3	ARP3 actin related protein 3 homolog
224012_at	1.11E-02	-0.08555303	ANKRD20A4///ANKRD20A2///ANKRD20A3///ANKRD20A1	ankyrin repeat domain 20 family member A4///ankyrin repeat domain 20 family member A2///ankyrin repeat domain 20 family member A3///ankyrin repeat domain 20 family member A1
1559420_x_at	1.11E-02	-0.1035852	CACNB2	calcium voltage-gated channel auxiliary subunit beta 2
224707_at	1.11E-02	0.33281732	CYSTM1	cysteine rich transmembrane module containing 1

206173_x_at	1.11E-02	0.23561341	GABPB1	GA binding protein transcription factor beta subunit 1
240149_at	1.11E-02	-0.05409753	HEATR6	HEAT repeat containing 6
226878_at	1.11E-02	-0.25222315	HLA-DOA	major histocompatibility complex, class II, DO alpha
1557174_a_at	1.11E-02	-0.40353972	IRAK1BP1	interleukin 1 receptor associated kinase 1 binding protein 1
1563341_at	1.11E-02	0.08607086	LOC101927783	uncharacterized LOC101927783
227308_x_at	1.11E-02	0.16228584	LTBP3	latent transforming growth factor beta binding protein 3
1553277_at	1.11E-02	-0.0570319	RTTN	rotatin
227164_at	1.11E-02	-0.21916745	SRSF1	serine and arginine rich splicing factor 1
218834_s_at	1.11E-02	0.17165171	TMEM132A	transmembrane protein 132A
211495_x_at	1.11E-02	0.1708126	TNFSF12-TNFSF13///TNFSF13	TNFSF12-TNFSF13 readthrough///tumor necrosis factor superfamily member 13
237865_x_at	1.11E-02	-0.10814768		
228835_at	1.11E-02	0.41506355		
241300_at	1.11E-02	0.19952199		
1557775_a_at	1.11E-02	0.09341723		
244213_at	1.11E-02	-0.06215351		
209391_at	1.12E-02	0.21782274	DPM2	dolichyl-phosphate mannosyltransferase subunit 2, regulatory
212149_at	1.12E-02	-0.19887855	EFR3A	EFR3 homolog A
213053_at	1.12E-02	0.13079226	HAUS5	HAUS augmin like complex subunit 5
220420_at	1.12E-02	0.09350428	LMAN1L	lectin, mannose binding 1 like
239275_at	1.12E-02	0.08621495	LOC101930006///FRMPD2B///FRMPD2	putative protein FRMPD2-like///FERM and PDZ domain containing 2B, pseudogene///FERM and PDZ domain containing 2
229747_x_at	1.12E-02	-0.12540307	LOC146880	Rho GTPase activating protein 27 pseudogene
1558611_at	1.12E-02	-0.09147775	MGC57346///CRHR1-IT1	ADP-ribosylation factor pseudogene///CRHR1 intronic transcript 1
224324_at	1.12E-02	0.08550043	MRO	maestro
239100_x_at	1.12E-02	-0.11044048	PCNX1	pecanex homolog 1 (Drosophila)
235082_at	1.12E-02	-0.1054453	PCNX1	pecanex homolog 1 (Drosophila)
223123_s_at	1.12E-02	0.0766923	PITHD1	PITH domain containing 1
238691_at	1.12E-02	-0.11888086	SCARNA13///SNHG10	small Cajal body-specific RNA 13///small nucleolar RNA host gene 10
223031_s_at	1.12E-02	0.17031518	TRAF7	TNF receptor associated factor 7
213476_x_at	1.12E-02	0.28123438	TUBB3	tubulin beta 3 class III
222318_at	1.12E-02	-0.11042127	ZNF324B	zinc finger protein 324B
210062_s_at	1.12E-02	0.15339492	ZNF589	zinc finger protein 589
1561627_at	1.12E-02	-0.06926881		
234100_at	1.12E-02	-0.11724147		
239706_x_at	1.12E-02	0.10021177		
1561561_x_at	1.12E-02	0.08383908		
45828_at	1.13E-02	0.2164002	ATP5SL	ATP5S like
205250_s_at	1.13E-02	-0.23770245	CEP290	centrosomal protein 290
208302_at	1.13E-02	0.07235725	HMHB1	histocompatibility minor HB-1
206853_s_at	1.13E-02	-0.28287208	MAP3K7	mitogen-activated protein kinase kinase kinase 7
203783_x_at	1.13E-02	0.09714522	POLRMT	RNA polymerase mitochondrial
216257_at	1.13E-02	-0.07994194	SERPINB13	serpin family B member 13
201078_at	1.13E-02	0.22786705	TM9SF2	transmembrane 9 superfamily member 2
224852_at	1.13E-02	0.26660438	TTC17	tetratricopeptide repeat domain 17
208977_x_at	1.13E-02	0.27973735	TUBB4B	tubulin beta 4B class IVb
223633_s_at	1.13E-02	0.19604921		
220717_at	1.14E-02	0.05298807	ADAMTS20	ADAM metallopeptidase with thrombospondin type 1 motif 20
210185_at	1.14E-02	0.10375981	CACNB1	calcium voltage-gated channel auxiliary subunit beta 1
219761_at	1.14E-02	-0.10855135	CLEC1A	C-type lectin domain family 1 member A
236341_at	1.14E-02	-0.42263763	CTLA4	cytotoxic T-lymphocyte associated protein 4
232843_s_at	1.14E-02	-0.32558533	DOCK8	dedicator of cytokinesis 8
206478_at	1.14E-02	-0.2410865	FAM30A	family with sequence similarity 30, member A
208748_s_at	1.14E-02	0.08623183	FLOT1	flotillin 1
222821_s_at	1.14E-02	0.22990068	GEMIN7	gem nuclear organelle associated protein 7
229541_at	1.14E-02	-0.23854682	HMBOX1	homeobox containing 1

1558045_a_at	1.14E-02	0.21133741	LOC389906	zinc finger protein 839 pseudogene
225888_at	1.14E-02	-0.22563245	NAA25	N(alpha)-acetyltransferase 25, NatB auxiliary subunit
222894_x_at	1.14E-02	0.33996169	NDUFAF5	NADH:ubiquinone oxidoreductase complex assembly factor 5
209485_s_at	1.14E-02	0.54237301	OSBPL1A	oxysterol binding protein like 1A
227698_s_at	1.14E-02	0.24313567	RAB40C	RAB40C, member RAS oncogene family
220683_at	1.14E-02	0.09659623	RDH8	retinol dehydrogenase 8 (all-trans)
207107_at	1.14E-02	-0.05543077	RPE65	RPE65, retinoid isomerohydrolase
218737_at	1.14E-02	-0.25151809	SBNO1	strawberry notch homolog 1
207707_s_at	1.14E-02	0.23854553	SEC13	SEC13 homolog, nuclear pore and COPII coat complex component
205126_at	1.14E-02	-0.22240747	VRK2	vaccinia related kinase 2
236309_x_at	1.14E-02	0.15303699	ZMIZ2	zinc finger MIZ-type containing 2
241818_at	1.14E-02	-0.14011213		
237438_at	1.14E-02	0.09943456		
244503_at	1.14E-02	-0.07339054		
217579_x_at	1.14E-02	-0.18559904		
1565780_at	1.15E-02	0.07133189	ABCA8	ATP binding cassette subfamily A member 8
214626_s_at	1.15E-02	0.17170713	GANAB	glucosidase II alpha subunit
200800_s_at	1.15E-02	0.64213328	HSPA1L///HSPA1B///HSPA1A	heat shock protein family A (Hsp70) member 1 like///heat shock protein family A (Hsp70) member 1B///heat shock protein family A (Hsp70) member 1A
210354_at	1.15E-02	-0.33457133	IFNG	interferon gamma
211375_s_at	1.15E-02	0.21938481	ILF3	interleukin enhancer binding factor 3
202911_at	1.15E-02	-0.22566218	MSH6	mutS homolog 6
218143_s_at	1.15E-02	0.20177737	SCAMP2	secretory carrier membrane protein 2
206052_s_at	1.15E-02	-0.20678439	SLBP	stem-loop binding protein
223782_s_at	1.15E-02	-0.53132953	TINAG	tubulointerstitial nephritis antigen
1558602_a_at	1.15E-02	-0.07080626	TUSC7	tumor suppressor candidate 7 (non-protein coding)
1558837_a_at	1.15E-02	0.1244651		
216707_at	1.15E-02	0.11133821		
204636_at	1.16E-02	0.32507198	COL17A1	collagen type XVII alpha 1 chain
1554429_a_at	1.16E-02	0.09350905	DMWD	dystrophia myotonica, WD repeat containing
218665_at	1.16E-02	-0.1366876	FZD4	frizzled class receptor 4
203560_at	1.16E-02	-0.42490217	GGH	gamma-glutamyl hydrolase
244401_at	1.16E-02	-0.13537674	LCA5	LCA5, lebercilin
208594_x_at	1.16E-02	0.10422407	LILRA6	leukocyte immunoglobulin like receptor A6
244769_at	1.16E-02	0.13259047	LOC101929964///LINC01184	uncharacterized LOC101929964///long intergenic non-protein coding RNA 1184
222712_s_at	1.16E-02	0.3276557	MUC13	mucin 13, cell surface associated
237870_at	1.16E-02	0.08865338	NQO2	NAD(P)H quinone dehydrogenase 2
201270_x_at	1.16E-02	0.21670854	NUDCD3	NudC domain containing 3
219292_at	1.16E-02	-0.25241218	THAP1	THAP domain containing 1
235386_at	1.16E-02	-0.0951685		
220692_at	1.16E-02	0.16139554		
231484_at	1.16E-02	-0.77958376		
214671_s_at	1.17E-02	0.17570894	ABR	active BCR-related
1555448_at	1.17E-02	-0.16287457	AP5M1	adaptor related protein complex 5 mu 1 subunit
222191_s_at	1.17E-02	0.16595242	B4GALT7	beta-1,4-galactosyltransferase 7
243864_at	1.17E-02	0.08311529	CCDC80	coiled-coil domain containing 80
223978_s_at	1.17E-02	0.36667051	CRLS1	cardiolipin synthase 1
208505_s_at	1.17E-02	0.19121987	FUT2	fucosyltransferase 2
209786_at	1.17E-02	-0.20249908	HMGN4	high mobility group nucleosomal binding domain 4
203991_s_at	1.17E-02	-0.34441389	KDM6A	lysine demethylase 6A
237192_at	1.17E-02	0.14128613	LOC101927668	uncharacterized LOC101927668
1561427_at	1.17E-02	-0.07902251	LOC101928333	uncharacterized LOC101928333
218586_at	1.17E-02	0.24436748	MRGBP	MRG/MORF4L binding protein
1554126_at	1.17E-02	0.09473021	MSRB3	methionine sulfoxide reductase B3
220656_at	1.17E-02	-0.05236976	NAA16	N(alpha)-acetyltransferase 16, NatA auxiliary subunit
217299_s_at	1.17E-02	-0.38445467	NBN	nibrin
214226_at	1.17E-02	0.09897341	PRSS53	protease, serine 53
49327_at	1.17E-02	0.09213978	SIRT3	sirtuin 3
201796_s_at	1.17E-02	0.09951401	VARS	valyl-tRNA synthetase

211383_s_at	1.17E-02	0.21997542	WDR37	WD repeat domain 37
244150_at	1.17E-02	-0.11237598		
221860_at	1.17E-02	0.23080354		
217992_s_at	1.18E-02	0.20815114	EFHD2	EF-hand domain family member D2
208842_s_at	1.18E-02	0.22090956	GORASP2	golgi reassembly stacking protein 2
225390_s_at	1.18E-02	0.27635272	KLF13	Kruppel like factor 13
208083_s_at	1.18E-02	0.15849903	LOC100505984///ITGB6	uncharacterized LOC100505984///integrin subunit beta 6
1558534_at	1.18E-02	0.08879301	LOC101060604///SMG1P3///SMG1P1///SLC7A5P1	putative L-type amino acid transporter 1-like protein IMAA///SMG1P3, nonsense mediated mRNA decay associated PI3K related kinase pseudogene 3///SMG1P1, nonsense mediated mRNA decay associated PI3K related kinase pseudogene 1///solute carrier family 7 member 5 pseudogene 1
240142_at	1.18E-02	-0.14349433	LOC101929289	uncharacterized LOC101929289
235151_at	1.18E-02	-0.39290599	LOC283357	uncharacterized LOC283357
202556_s_at	1.18E-02	0.20202537	MCRS1	microspherule protein 1
1560116_a_at	1.18E-02	-0.2273504	NEDD1	neural precursor cell expressed, developmentally down-regulated 1
218860_at	1.18E-02	0.22951712	NOC4L	nucleolar complex associated 4 homolog
212782_x_at	1.18E-02	0.23052052	POLR2J	RNA polymerase II subunit J
1558217_at	1.18E-02	-0.26047123	SLFN13	schlafen family member 13
36742_at	1.18E-02	0.27876621	TRIM15	tripartite motif containing 15
227501_at	1.18E-02	0.27621763		
AFFX-r2-Ec-bioB-3	1.18E-02	-0.15373724		
219145_at	1.19E-02	0.16051646	ADGRL1	adhesion G protein-coupled receptor L1
226641_at	1.19E-02	-0.47447988	ANKRD44	ankyrin repeat domain 44
221161_at	1.19E-02	0.09460545	ASCL3	achaete-scute family bHLH transcription factor 3
228910_at	1.19E-02	0.16269086	CD82	CD82 molecule
1552868_at	1.19E-02	0.11753022	CIRBP-AS1	CIRBP antisense RNA 1
209831_x_at	1.19E-02	0.10710351	DNASE2	deoxyribonuclease 2, lysosomal
218991_at	1.19E-02	-0.19843926	HEATR6	HEAT repeat containing 6
213331_s_at	1.19E-02	-0.29378804	NEK1	NIMA related kinase 1
1566670_at	1.19E-02	0.08401424	PDXK	pyridoxal (pyridoxine, vitamin B6) kinase
205093_at	1.19E-02	0.24728678	PLEKHA6	pleckstrin homology domain containing A6
226389_s_at	1.19E-02	0.08765851	RAPGEF1	Rap guanine nucleotide exchange factor 1
205009_at	1.19E-02	0.97003149	TFF1	trefoil factor 1
235735_at	1.19E-02	-0.14960773	TNFSF8	tumor necrosis factor superfamily member 8
233275_at	1.19E-02	-0.08261924		
222550_at	1.20E-02	-0.22110456	ARMC1	armadillo repeat containing 1
229487_at	1.20E-02	-0.15136576	EBF1	early B-cell factor 1
219570_at	1.20E-02	0.34226418	KIF16B	kinesin family member 16B
1561411_at	1.20E-02	-0.09736021	LINC01222	long intergenic non-protein coding RNA 1222
214866_at	1.20E-02	0.31229851	PLAUR	plasminogen activator, urokinase receptor
228643_at	1.20E-02	-0.23843977	RAB30-AS1	RAB30 antisense RNA 1 (head to head)
223995_at	1.20E-02	0.14771024	SLC12A9	solute carrier family 12 member 9
209607_x_at	1.20E-02	0.32305111	SLX1B-SULT1A4///SLX1A-SULT1A3///SULT1A4///SULT1A3	SLX1B-SULT1A4 readthrough (NMD candidate)///SLX1A-SULT1A3 readthrough (NMD candidate)///sulfotransferase family 1A member 4///sulfotransferase family 1A member 3
1565347_s_at	1.20E-02	0.0791868	TFE3	transcription factor binding to IGHM enhancer 3
226954_at	1.20E-02	0.1963212	UBE2R2	ubiquitin conjugating enzyme E2 R2
204063_s_at	1.20E-02	-0.36698939	ULK2	unc-51 like autophagy activating kinase 2
214879_x_at	1.20E-02	0.10685808	USF2	upstream transcription factor 2, c-fos interacting
1556540_a_at	1.20E-02	0.07198678		
202025_x_at	1.21E-02	0.20296864	ACAA1	acetyl-CoA acyltransferase 1
226588_at	1.21E-02	-0.16165935	CWC22	CWC22 homolog, spliceosome-associated protein
212987_at	1.21E-02	-0.19916692	FBXO9	F-box protein 9
220291_at	1.21E-02	0.13545141	GDPD2	glycerophosphodiester phosphodiesterase domain containing 2
206865_at	1.21E-02	-0.08876793	HRK	harakiri, BCL2 interacting protein
225263_at	1.21E-02	0.19187663	HS6ST1	heparan sulfate 6-O-sulfotransferase 1
1554646_at	1.21E-02	0.11094364	OSBPL1A	oxysterol binding protein like 1A

237202_at	1.21E-02	0.10758421	PGPEP1	pyroglutamyl-peptidase I
1555852_at	1.21E-02	-0.36458512	PSMB8-AS1	PSMB8 antisense RNA 1 (head to head)
210568_s_at	1.21E-02	-0.26801481	RECQL	RecQ like helicase
212122_at	1.21E-02	-0.28277448	RHOQ	ras homolog family member Q
208354_s_at	1.21E-02	0.10289528	SLC12A3	solute carrier family 12 member 3
243816_at	1.21E-02	-0.28290165	ZNF70	zinc finger protein 70
218581_at	1.22E-02	0.1833104	ABHD4	abhydrolase domain containing 4
218534_s_at	1.22E-02	-0.22466636	AGGF1	angiogenic factor with G-patch and FHA domains 1
206430_at	1.22E-02	0.22898791	CDX1	caudal type homeobox 1
203267_s_at	1.22E-02	0.18580939	DRG2	developmentally regulated GTP binding protein 2
211303_x_at	1.22E-02	-0.10744811	FOLH1B	folate hydrolase 1B
237493_at	1.22E-02	-0.07974312	IL22RA2	interleukin 22 receptor subunit alpha 2
221111_at	1.22E-02	-0.16117438	IL26	interleukin 26
204864_s_at	1.22E-02	0.09517458	IL6ST	interleukin 6 signal transducer
229231_at	1.22E-02	-0.21262568	LRRC37B	leucine rich repeat containing 37B
206877_at	1.22E-02	0.17406975	MXD1	MAX dimerization protein 1
221739_at	1.22E-02	0.24514956	MYDGF	myeloid derived growth factor
205091_x_at	1.22E-02	-0.27806215	RECQL	RecQ like helicase
225713_at	1.22E-02	0.15883164	STK11IP	serine/threonine kinase 11 interacting protein
1560292_a_at	1.22E-02	0.08401167	TMCO4	transmembrane and coiled-coil domains 4
1553439_at	1.22E-02	0.08457945		
237479_at	1.22E-02	-0.05577743		
231302_at	1.22E-02	-0.15773359		
1556623_at	1.22E-02	0.10423846		
238267_s_at	1.22E-02	-0.12179703		
214998_at	1.23E-02	-0.09271262	AAK1	AP2 associated kinase 1
221589_s_at	1.23E-02	-0.39212585	ALDH6A1	aldehyde dehydrogenase 6 family member A1
206200_s_at	1.23E-02	0.25253438	ANXA11	annexin A11
231016_s_at	1.23E-02	-0.20478485	ARNT	aryl hydrocarbon receptor nuclear translocator
221160_s_at	1.23E-02	0.09363696	CABP5	calcium binding protein 5
217078_s_at	1.23E-02	0.07968651	CD300A	CD300a molecule
228516_at	1.23E-02	-0.18068427	CDAN1	codanin 1
227285_at	1.23E-02	0.19540133	CIART	circadian associated repressor of transcription
203653_s_at	1.23E-02	-0.165857	COIL	coilin
203028_s_at	1.23E-02	0.44606616	CYBA	cytochrome b-245 alpha chain
207786_at	1.23E-02	0.15996621	CYP2R1	cytochrome P450 family 2 subfamily R member 1
216389_s_at	1.23E-02	0.12992352	DCAF11	DDB1 and CUL4 associated factor 11
204355_at	1.23E-02	0.28401731	DHX30	DEAH-box helicase 30
220426_at	1.23E-02	0.17208355	FNDC11	fibronectin type III domain containing 11
201245_s_at	1.23E-02	0.2680266	LOC101927673///OTUB1	uncharacterized LOC101927673///OTU deubiquitinase, ubiquitin aldehyde binding 1
232899_at	1.23E-02	-0.22654386	LOC101929177	uncharacterized LOC101929177
230530_at	1.23E-02	0.09123885	MLYCD	malonyl-CoA decarboxylase
231941_s_at	1.23E-02	0.5217057	MUC20	mucin 20, cell surface associated
209229_s_at	1.23E-02	0.10933352	PPP6R1	protein phosphatase 6 regulatory subunit 1
39729_at	1.23E-02	0.25889412	PRDX2	peroxiredoxin 2
218345_at	1.23E-02	0.53412719	TMEM176A	transmembrane protein 176A
203838_s_at	1.23E-02	0.12057813	TNK2	tyrosine kinase non receptor 2
210954_s_at	1.23E-02	0.09691255	TSC22D2	TSC22 domain family member 2
233102_at	1.23E-02	-0.06118679		
229932_at	1.23E-02	-0.11762683		
1552304_at	1.24E-02	-0.27818979	ALG10	ALG10, alpha-1,2-glucosyltransferase
233912_x_at	1.24E-02	-0.12481047	ELMOD2	ELMO domain containing 2
208987_s_at	1.24E-02	0.13713922	KDM2A	lysine demethylase 2A
234617_at	1.24E-02	-0.09568855	OR52D1	olfactory receptor family 52 subfamily D member 1
1560138_at	1.24E-02	0.06434277	RUBCN	RUN and cysteine rich domain containing beclin 1 interacting protein
221920_s_at	1.24E-02	0.32000727	SLC25A37	solute carrier family 25 member 37
230623_x_at	1.24E-02	-0.24886831	USP28	ubiquitin specific peptidase 28
225926_at	1.24E-02	-0.2290217	VTI1B	vesicle transport through interaction with t-SNAREs 1B
235068_at	1.24E-02	-0.27795182	ZDHHC21	zinc finger DHHC-type containing 21
203410_at	1.25E-02	-0.26857492	AP3M2	adaptor related protein complex 3 mu 2 subunit
243433_at	1.25E-02	0.18286363	ARHGAP30	Rho GTPase activating protein 30

1554783_s_at	1.25E-02	0.07571022	ARHGEF2	Rho/Rac guanine nucleotide exchange factor 2
1567035_at	1.25E-02	0.11218475	C20orf181	chromosome 20 open reading frame 181
243093_at	1.25E-02	-0.11712557	C2orf49	chromosome 2 open reading frame 49
212677_s_at	1.25E-02	-0.30420794	CEP68	centrosomal protein 68
226800_at	1.25E-02	-0.3454664	EFCAB7	EF-hand calcium binding domain 7
224577_at	1.25E-02	0.24698589	ERGIC1	endoplasmic reticulum-golgi intermediate compartment 1
206774_at	1.25E-02	0.09784571	FRMPD1	FERM and PDZ domain containing 1
212370_x_at	1.25E-02	-0.17495438	LOC101930591///FAM21A///FAM21C	uncharacterized LOC101930591///family with sequence similarity 21 member A///family with sequence similarity 21 member C
1558680_s_at	1.25E-02	0.09375886	PDE1A	phosphodiesterase 1A
1570274_at	1.25E-02	0.07782168	PRSS55	protease, serine 55
200611_s_at	1.25E-02	0.25879284	WDR1	WD repeat domain 1
230827_at	1.25E-02	-0.148729		
229659_s_at	1.25E-02	-1.04683366		
244035_at	1.25E-02	-0.2559165		
210461_s_at	1.26E-02	0.24105708	ABLIM1	actin binding LIM protein 1
218274_s_at	1.26E-02	0.17251287	ANKZF1	ankyrin repeat and zinc finger domain containing 1
218214_at	1.26E-02	0.21881549	ATG101	autophagy related 101
210783_x_at	1.26E-02	0.12635935	CLEC11A	C-type lectin domain family 11 member A
207611_at	1.26E-02	-0.07618154	HIST1H2BL	histone cluster 1, H2bl
214098_at	1.26E-02	-0.32218415	KIAA1107	KIAA1107
235970_at	1.26E-02	-0.32525894	LCORL	ligand dependent nuclear receptor corepressor like
209215_at	1.26E-02	0.26601792	MFSD10	major facilitator superfamily domain containing 10
201173_x_at	1.26E-02	0.23441204	NUDC	nuclear distribution C, dynein complex regulator
219168_s_at	1.26E-02	0.21822298	PRR5	proline rich 5
238137_at	1.26E-02	-0.07894		
1565651_at	1.26E-02	0.17124454		
234273_at	1.26E-02	0.11100928		
205146_x_at	1.27E-02	0.12951396	APBA3	amyloid beta precursor protein binding family A member 3
203256_at	1.27E-02	0.40005082	CDH3	cadherin 3
222694_at	1.27E-02	0.11513039	CENPBD1P1	CENPB DNA-binding domains containing 1 pseudogene 1
214290_s_at	1.27E-02	0.3949796	HIST2H2AA4///HIST2H2AA3	histone cluster 2, H2aa4///histone cluster 2, H2aa3
1557787_at	1.27E-02	0.06687658	LOC101929153	uncharacterized LOC101929153
230027_s_at	1.27E-02	0.2734507	MRPL43	mitochondrial ribosomal protein L43
231469_at	1.27E-02	0.07775147	NTRK3-AS1	NTRK3 antisense RNA 1
201136_at	1.27E-02	0.3462223	PLP2	proteolipid protein 2
235758_at	1.27E-02	-0.17325948	PNMA6A	paraneoplastic Ma antigen family member 6A
225753_at	1.27E-02	0.22811252	ZNF513	zinc finger protein 513
237000_at	1.27E-02	-0.058619		
236590_at	1.27E-02	-0.232409		
236003_x_at	1.27E-02	-0.10897434		
215787_at	1.28E-02	-0.09348045	ACTA2	actin, alpha 2, smooth muscle, aorta
1557985_s_at	1.28E-02	-0.15527579	CEP78	centrosomal protein 78
1561590_a_at	1.28E-02	0.07050765	CNTFR-AS1	CNTFR antisense RNA 1
208735_s_at	1.28E-02	0.14476952	CTDSP2	CTD small phosphatase 2
238401_at	1.28E-02	0.1233174	ENDOV	endonuclease V
217118_s_at	1.28E-02	0.23368746	KIAA0930	KIAA0930
216624_s_at	1.28E-02	0.10015106	KMT2A	lysine methyltransferase 2A
216264_s_at	1.28E-02	0.26956068	LAMB2	laminin subunit beta 2
224559_at	1.28E-02	-0.40144899	MALAT1	metastasis associated lung adenocarcinoma transcript 1 (non-protein coding)
232102_at	1.28E-02	-0.09107508	METTL6	methyltransferase like 6
211708_s_at	1.28E-02	0.4091189	SCD	stearoyl-CoA desaturase
218367_x_at	1.28E-02	0.22440222	USP21	ubiquitin specific peptidase 21
244489_at	1.28E-02	0.12080877		
232743_at	1.28E-02	0.07681614		
206725_x_at	1.29E-02	0.13410812	BMP1	bone morphogenetic protein 1
1555990_at	1.29E-02	0.09477696	C22orf42	chromosome 22 open reading frame 42
228748_at	1.29E-02	0.23738096	CD59	CD59 molecule

235387_at	1.29E-02	-0.29713498	GSTCD	glutathione S-transferase C-terminal domain containing
221877_at	1.29E-02	0.18393607	IRGQ	immunity related GTPase Q
205116_at	1.29E-02	-0.31720064	LAMA2	laminin subunit alpha 2
228133_s_at	1.29E-02	0.07813685	MYH11	myosin heavy chain 11
217545_at	1.29E-02	0.14336276	MYH14	myosin, heavy chain 14, non-muscle
203920_at	1.29E-02	0.3131197	NR1H3	nuclear receptor subfamily 1 group H member 3
213421_x_at	1.29E-02	0.28209039	PRSS3	protease, serine 3
233447_at	1.29E-02	0.0839342	PSMG4	proteasome assembly chaperone 4
224950_at	1.29E-02	0.29857049	PTGFRN	prostaglandin F2 receptor inhibitor
1557521_a_at	1.29E-02	0.39054599		
215512_at	1.30E-02	0.1404618	MARCHF6	membrane associated ring-CH-type finger 6
202331_at	1.30E-02	0.17601556	BCKDHA	branched chain keto acid dehydrogenase E1, alpha polypeptide
206331_at	1.30E-02	-0.24191981	CALCRL	calcitonin receptor like receptor
202560_s_at	1.30E-02	0.18241221	CHTOP	chromatin target of PRMT1
210844_x_at	1.30E-02	0.19526625	CTNNA1	catenin alpha 1
209710_at	1.30E-02	-0.26371393	GATA2	GATA binding protein 2
209728_at	1.30E-02	-0.86962455	HLA-DRB4	major histocompatibility complex, class II, DR beta 4
1569156_at	1.30E-02	0.11725335	LOC100505555///ZNF846	uncharacterized LOC100505555///zinc finger protein 846
211068_x_at	1.30E-02	-0.16982685	LOC101930591///FAM21C	uncharacterized LOC101930591///family with sequence similarity 21 member C
210596_at	1.30E-02	-0.31841458	MAGT1	magnesium transporter 1
228171_s_at	1.30E-02	0.293951	PLEKHG4	pleckstrin homology and RhoGEF domain containing G4
229269_x_at	1.30E-02	0.21918508	SSBP4	single stranded DNA binding protein 4
223544_at	1.30E-02	0.12720989	TMEM79	transmembrane protein 79
239674_at	1.30E-02	-0.07559376		
205843_x_at	1.31E-02	0.18365924	CRAT	carnitine O-acetyltransferase
205749_at	1.31E-02	0.0961581	CYP1A1	cytochrome P450 family 1 subfamily A member 1
228665_at	1.31E-02	-0.2920257	CYYR1	cysteine and tyrosine rich 1
1554078_s_at	1.31E-02	0.20346853	DNAJA3	DnaJ heat shock protein family (Hsp40) member A3
226648_at	1.31E-02	0.25385108	HIF1AN	hypoxia inducible factor 1 alpha subunit inhibitor
211406_at	1.31E-02	-0.26375924	IER3IP1	immediate early response 3 interacting protein 1
204411_at	1.31E-02	0.27607309	KIF21B	kinesin family member 21B
222161_at	1.31E-02	-0.05758744	NAALAD2	N-acetylated alpha-linked acidic dipeptidase 2
227240_at	1.31E-02	0.30064099	NGEF	neuronal guanine nucleotide exchange factor
213030_s_at	1.31E-02	0.23985659	PLXNA2	plexin A2
210122_at	1.31E-02	0.07657506	PRM2	protamine 2
212081_x_at	1.31E-02	0.17580436	PRRC2A	proline rich coiled-coil 2A
213064_at	1.31E-02	-0.25163461	ZC3H14	zinc finger CCCH-type containing 14
202022_at	1.32E-02	0.34880607	ALDOC	aldolase, fructose-bisphosphate C
204608_at	1.32E-02	0.39008715	ASL	argininosuccinate lyase
228189_at	1.32E-02	-0.27813461	BAG4	BCL2 associated athanogene 4
216028_at	1.32E-02	0.29271015	DKFZP564C152	DKFZP564C152 protein
232787_at	1.32E-02	0.16832006	HELZ2	helicase with zinc finger 2
208713_at	1.32E-02	0.15344951	HNRNPUL1	heterogeneous nuclear ribonucleoprotein U like 1
218798_at	1.32E-02	0.12593625	KRI1	KRI1 homolog
201475_x_at	1.32E-02	0.25658651	MIR6758///MARS	microRNA 6758///methionyl-tRNA synthetase
210670_at	1.32E-02	0.10086742	PPY	pancreatic polypeptide
218784_s_at	1.32E-02	-0.22164054	SAYSD1	SAYSVFN motif domain containing 1
216591_s_at	1.32E-02	0.19432704	SDHC	succinate dehydrogenase complex subunit C
201195_s_at	1.32E-02	0.33193607	SLC7A5	solute carrier family 7 member 5
230635_at	1.32E-02	0.07051862	TUG1	taurine up-regulated 1 (non-protein coding)
233553_at	1.32E-02	-0.08157435		
242264_at	1.32E-02	-0.26472277		
229569_at	1.32E-02	-0.48351614		
206043_s_at	1.33E-02	0.38324055	ATP2C2	ATPase secretory pathway Ca2+ transporting 2
219529_at	1.33E-02	0.29175261	CLIC3	chloride intracellular channel 3
208295_x_at	1.33E-02	0.07574941	CSHL1	chorionic somatomammotropin hormone like 1
1568848_at	1.33E-02	0.08970842	LOC101927820	uncharacterized LOC101927820
229584_at	1.33E-02	-0.34331061	LRRK2	leucine rich repeat kinase 2
1568629_s_at	1.33E-02	0.19879209	PIK3R2	phosphoinositide-3-kinase regulatory subunit 2

230330_at	1.33E-02	-0.17338635	PPM1D	protein phosphatase, Mg2+/Mn2+ dependent 1D
242388_x_at	1.33E-02	-0.22218814	TAGAP	T-cell activation RhoGTPase activating protein
218753_at	1.33E-02	0.10914909	XKR8	XK related 8
219199_at	1.34E-02	0.15432761	AFF4	AF4/FMR2 family member 4
227015_at	1.34E-02	0.35513828	ASPHD2	aspartate beta-hydroxylase domain containing 2
221879_at	1.34E-02	0.24412139	CALML4	calmodulin like 4
211848_s_at	1.34E-02	-0.43293847	CEACAM7	carcinoembryonic antigen related cell adhesion molecule 7
228587_at	1.34E-02	0.13414599	FAM83G	family with sequence similarity 83 member G
222956_at	1.34E-02	0.08839121	FIGN	fidgetin, microtubule severing factor
1552912_a_at	1.34E-02	-0.11680082	IL23R	interleukin 23 receptor
239507_at	1.34E-02	-0.08152061	LINC00608	long intergenic non-protein coding RNA 608
1561233_at	1.34E-02	0.08833748	LOC283387	uncharacterized LOC283387
213126_at	1.34E-02	0.20721642	MED8	mediator complex subunit 8
225196_s_at	1.34E-02	0.30227484	MRPS26	mitochondrial ribosomal protein S26
242297_at	1.34E-02	-0.16314279	RREB1	ras responsive element binding protein 1
217807_s_at	1.34E-02	0.15929502	SNORD23///GLTSCR2	small nucleolar RNA, C/D box 23///glioma tumor suppressor candidate region gene 2
202707_at	1.34E-02	0.09963541	UMPS	uridine monophosphate synthetase
235840_at	1.34E-02	-0.24246995	ZKSCAN3	zinc finger with KRAB and SCAN domains 3
230599_at	1.34E-02	-0.28219388		
232826_at	1.34E-02	-0.12999285		
233825_s_at	1.35E-02	0.20800507	CD99L2	CD99 molecule like 2
212675_s_at	1.35E-02	-0.31342432	CEP68	centrosomal protein 68
208342_x_at	1.35E-02	0.07806559	CSH2///CSH1	chorionic somatomammotropin hormone 2///chorionic somatomammotropin hormone 1
236852_at	1.35E-02	-0.10512724	FBXO43	F-box protein 43
210164_at	1.35E-02	-0.818021	GZMB	granzyme B
213418_at	1.35E-02	0.53018846	HSPA6	heat shock protein family A (Hsp70) member 6
220574_at	1.35E-02	-0.15916348	SEMA6D	semaphorin 6D
204430_s_at	1.35E-02	-0.28957265	SLC2A5	solute carrier family 2 member 5
222801_s_at	1.35E-02	-0.23507545	STAG3L4	stromal antigen 3-like 4 (pseudogene)
229866_at	1.35E-02	0.11946744	STK32A	serine/threonine kinase 32A
235515_at	1.35E-02	0.46367832	SYNE4	spectrin repeat containing nuclear envelope family member 4
206472_s_at	1.35E-02	0.15368755	TLE3	transducin like enhancer of split 3
217135_x_at	1.35E-02	0.12883558		
237778_at	1.35E-02	-0.37202074		
244847_at	1.35E-02	-0.11097521		
233606_at	1.35E-02	-0.08124892		
243281_at	1.35E-02	-0.07947042		
226258_at	1.36E-02	-0.30211791	AMN1	antagonist of mitotic exit network 1 homolog
1555544_a_at	1.36E-02	0.07529113	CADM2	cell adhesion molecule 2
213509_x_at	1.36E-02	0.50381979	CES2	carboxylesterase 2
233474_at	1.36E-02	0.08040122	LOC284240	uncharacterized LOC284240
220954_s_at	1.36E-02	0.26601752	MIR6840///STAG3L5P-PVRIG2P-PILRB///PILRB	microRNA 6840///STAG3L5P-PVRIG2P-PILRB readthrough///paired immunoglobin-like type 2 receptor beta
201467_s_at	1.36E-02	0.51125167	NQO1	NAD(P)H quinone dehydrogenase 1
212542_s_at	1.36E-02	-0.21574068	PHIP	pleckstrin homology domain interacting protein
217854_s_at	1.36E-02	0.25329367	POLR2E	RNA polymerase II subunit E
219613_s_at	1.36E-02	0.12874106	SIRT6	sirtuin 6
239384_at	1.36E-02	-0.13633627	SRSF1	serine and arginine rich splicing factor 1
204998_s_at	1.37E-02	0.16216534	ATF5	activating transcription factor 5
222089_s_at	1.37E-02	0.07812791	C16orf71	chromosome 16 open reading frame 71
64408_s_at	1.37E-02	0.25948886	CALML4	calmodulin like 4
205003_at	1.37E-02	-0.39684763	DOCK4	dedicator of cytokinesis 4
244808_at	1.37E-02	0.20466873	GRAMD1A	GRAM domain containing 1A
1565817_at	1.37E-02	0.19084083	IKZF1	IKAROS family zinc finger 1
206750_at	1.37E-02	0.10332243	MAFK	MAF bZIP transcription factor K
218300_at	1.37E-02	0.18653306	PAGR1	PAXIP1 associated glutamate rich protein 1
203338_at	1.37E-02	-0.24630846	PPP2R5E	protein phosphatase 2 regulatory subunit B'epsilon
238572_at	1.37E-02	0.10473795	STK16	serine/threonine kinase 16

1560275_at	1.37E-02	0.15839792	TMEM44	transmembrane protein 44
244217_at	1.37E-02	0.12680104		
234592_at	1.37E-02	0.08568795		
237832_at	1.37E-02	-0.07246667		
231507_at	1.37E-02	-0.08010693		
221272_s_at	1.38E-02	0.25748436	C1orf21	chromosome 1 open reading frame 21
239049_at	1.38E-02	-0.40635458	CRIPT	CXXC repeat containing interactor of PDZ3 domain
219469_at	1.38E-02	-0.38747185	DYNC2H1	dynein cytoplasmic 2 heavy chain 1
239821_at	1.38E-02	0.09163102	FLJ30064	uncharacterized LOC644975
230298_at	1.38E-02	-0.28349207	MBLAC2	metallo-beta-lactamase domain containing 2
230477_at	1.38E-02	0.28992856	PARD6G-AS1	PARD6G antisense RNA 1
213141_at	1.38E-02	0.12567801	PSKH1	protein serine kinase H1
1562896_at	1.38E-02	0.10779118	TNRC18	trinucleotide repeat containing 18
228788_at	1.38E-02	-0.16958798	YPEL1	yippee like 1
222381_at	1.38E-02	0.10025852		
229111_at	1.38E-02	-0.38037595		
201301_s_at	1.39E-02	0.19139014	ANXA4	annexin A4
235514_at	1.39E-02	0.13428679	ASPRV1	aspartic peptidase, retroviral-like 1
1557585_at	1.39E-02	-0.1115964	ATP6V1H	ATPase H+ transporting V1 subunit H
201130_s_at	1.39E-02	0.25556855	CDH1	cadherin 1
237685_at	1.39E-02	0.07289783	LOC101929926	uncharacterized LOC101929926
229682_at	1.39E-02	0.10269216	MAPRE3	microtubule associated protein RP/EB family member 3
206538_at	1.39E-02	0.10671743	MRAS	muscle RAS oncogene homolog
202759_s_at	1.39E-02	0.35853769	PALM2-AKAP2///AKAP2	PALM2-AKAP2 readthrough///A-kinase anchoring protein 2
202771_at	1.39E-02	0.2918703	PIEZO1	piezo type mechanosensitive ion channel component 1
216638_s_at	1.39E-02	0.07597059	PRLR	prolactin receptor
211270_x_at	1.39E-02	0.15788392	PTBP1	polypyrimidine tract binding protein 1
1559582_at	1.39E-02	0.09014805	RHOQ	ras homolog family member Q
244880_at	1.39E-02	-0.18183694		
1560512_at	1.39E-02	-0.21888238		
237688_at	1.39E-02	0.23117522		
234079_at	1.39E-02	0.09636947		
223740_at	1.40E-02	0.10640731	AGPAT4-IT1	AGPAT4 intronic transcript 1
205257_s_at	1.40E-02	0.10726694	AMPH	amphiphysin
237177_at	1.40E-02	-0.086948	CNTN4	contactin 4
229731_at	1.40E-02	0.08605222	FOXS1	forkhead box S1
1565717_s_at	1.40E-02	0.21097481	FUS	FUS RNA binding protein
1568639_a_at	1.40E-02	-0.20383309	GATA2-AS1	GATA2 antisense RNA 1
238681_at	1.40E-02	-0.44432472	GDPD1	glycerophosphodiester phosphodiesterase domain containing 1
208169_s_at	1.40E-02	0.09576311	PTGER3	prostaglandin E receptor 3
202035_s_at	1.40E-02	0.0685189	SFRP1	secreted frizzled related protein 1
212287_at	1.40E-02	-0.24445568	SUZ12	SUZ12 polycomb repressive complex 2 subunit
217098_s_at	1.40E-02	-0.07784338	ZSCAN12	zinc finger and SCAN domain containing 12
240861_at	1.40E-02	-0.38473194		
87100_at	1.41E-02	0.26491396	ABHD2	abhydrolase domain containing 2
238560_at	1.41E-02	-0.18403249	CALCOCO2	calcium binding and coiled-coil domain 2
209850_s_at	1.41E-02	0.16824652	CDC42EP2	CDC42 effector protein 2
227105_at	1.41E-02	-0.27211249	CSPP1	centrosome and spindle pole associated protein 1
213766_x_at	1.41E-02	0.20516074	GNA11	G protein subunit alpha 11
211339_s_at	1.41E-02	-0.29437969	ITK	IL2 inducible T-cell kinase
218463_s_at	1.41E-02	0.17482404	MUS81	MUS81 structure-specific endonuclease subunit
236273_at	1.41E-02	-0.28346309	NBPF1	neuroblastoma breakpoint family member 1
218019_s_at	1.41E-02	0.27911088	PDXK	pyridoxal (pyridoxine, vitamin B6) kinase
209344_at	1.41E-02	0.2471774	TPM4	tropomyosin 4
218502_s_at	1.41E-02	-0.37593724	TRPS1	transcriptional repressor GATA binding 1
228350_at	1.41E-02	-0.09896267	UNC13D	unc-13 homolog D
1553550_at	1.41E-02	-0.08549525	VN1R5	vomeronasal 1 receptor 5 (gene/pseudogene)
238718_at	1.41E-02	0.14682671		
210427_x_at	1.42E-02	0.24203925	ANXA2	annexin A2
211779_x_at	1.42E-02	0.22576089	AP2A2	adaptor related protein complex 2 alpha 2 subunit
235734_at	1.42E-02	-0.10306085	ARFGAP2	ADP ribosylation factor GTPase activating protein 2

238385_at	1.42E-02	-0.07505564	C6orf58	chromosome 6 open reading frame 58
220753_s_at	1.42E-02	0.36893467	CRYL1	crystallin lambda 1
217840_at	1.42E-02	0.24295846	DDX41	DEAD-box helicase 41
212339_at	1.42E-02	0.4448625	EPB41L1	erythrocyte membrane protein band 4.1 like 1
228117_at	1.42E-02	0.07821882	LOC102723722///NDUFA6-AS1	uncharacterized LOC102723722///NDUFA6 antisense RNA 1 (head to head)
215568_x_at	1.42E-02	0.13907822	LYPLA2	lysophospholipase II
220864_s_at	1.42E-02	0.28150046	NDUFA13	NADH:ubiquinone oxidoreductase subunit A13
203415_at	1.42E-02	0.2230735	PDCD6	programmed cell death 6
224762_at	1.42E-02	0.27263613	SERINC2	serine incorporator 2
201396_s_at	1.42E-02	0.15089864	SGTA	small glutamine rich tetratricopeptide repeat containing alpha
224756_s_at	1.43E-02	0.2008384	ABHD16A	abhydrolase domain containing 16A
219514_at	1.43E-02	0.115123	ANGPTL2	angiopoietin like 2
202442_at	1.43E-02	0.19845473	AP3S1	adaptor related protein complex 3 sigma 1 subunit
235056_at	1.43E-02	-0.27168827	ETV6	ETS variant 6
226698_at	1.43E-02	0.14989882	FCHSD1	FCH and double SH3 domains 1
205505_at	1.43E-02	0.38496019	GCNT1	glucosaminyl (N-acetyl) transferase 1, core 2
235695_at	1.43E-02	-0.17327932	INPP4A	inositol polyphosphate-4-phosphatase type I A
238975_at	1.43E-02	0.15141327	MMAB	methylmalonic aciduria (cobalamin deficiency) cblB type
210276_s_at	1.43E-02	0.25098214	NOL12///TRIOBP	nucleolar protein 12///TRIO and F-actin binding protein
231101_at	1.43E-02	-0.32625885	PPP2R5E	protein phosphatase 2 regulatory subunit B'epsilon
221819_at	1.43E-02	0.11469282	RAB35	RAB35, member RAS oncogene family
239242_at	1.43E-02	-0.17681082	SLC25A5-AS1	SLC25A5 antisense RNA 1
215547_at	1.43E-02	-0.08496047	TSC22D2	TSC22 domain family member 2
1558215_s_at	1.43E-02	0.2036272	UBTF	upstream binding transcription factor, RNA polymerase I
213389_at	1.43E-02	0.17903703	ZNF592	zinc finger protein 592
239016_at	1.43E-02	-0.27437146		
237999_at	1.43E-02	-0.3362242		
236682_at	1.43E-02	-0.16518039		
220533_at	1.43E-02	0.1340899		
AFFX-HUMRGE/M1	1.43E-02	0.150984		
1563182_at	1.44E-02	-0.48946306	ACVR1C	activin A receptor type 1C
234007_at	1.44E-02	0.06803388	DEFB121	defensin beta 121
225838_at	1.44E-02	-0.17879095	EPC2	enhancer of polycomb homolog 2
1554576_a_at	1.44E-02	0.33935769	ETV4	ETS variant 4
1552721_a_at	1.44E-02	-0.05898672	FGF1	fibroblast growth factor 1
205063_at	1.44E-02	-0.39563215	GEMIN2	gem nuclear organelle associated protein 2
223278_at	1.44E-02	0.48380821	GJB2	gap junction protein beta 2
212640_at	1.44E-02	0.17004611	HACD2	3-hydroxyacyl-CoA dehydratase 2
225102_at	1.44E-02	0.32247974	MGLL	monoglyceride lipase
209723_at	1.44E-02	-0.35463577	SERPINB9	serpin family B member 9
210177_at	1.44E-02	0.250179	TRIM15	tripartite motif containing 15
218475_at	1.44E-02	0.1314977	TRMT2A	tRNA methyltransferase 2 homolog A
235663_at	1.44E-02	0.15600259		
233315_at	1.44E-02	0.12795172		
1568685_at	1.44E-02	0.06864382		
230634_x_at	1.45E-02	0.26840192	ADAT3///SCAMP4	adenosine deaminase, tRNA specific 3///secretory carrier membrane protein 4
201590_x_at	1.45E-02	0.23476216	ANXA2	annexin A2
211289_x_at	1.45E-02	0.20527151	CDK11A///CDK11B	cyclin dependent kinase 11A///cyclin dependent kinase 11B
237621_at	1.45E-02	-0.08433809	GABRG3-AS1	GABRG3 antisense RNA 1
208501_at	1.45E-02	0.08724965	GFI1B	growth factor independent 1B transcriptional repressor
205262_at	1.45E-02	0.19368909	KCNH2	potassium voltage-gated channel subfamily H member 2
204272_at	1.45E-02	0.31869663	LGALS4	galectin 4
231715_s_at	1.45E-02	0.19666485	MIR6741///PYCR2	microRNA 6741///pyrroline-5-carboxylate reductase family member 2
242719_at	1.45E-02	0.14262097	RYBP	RING1 and YY1 binding protein
205316_at	1.45E-02	-0.23257553	SLC15A2	solute carrier family 15 member 2
212679_at	1.45E-02	0.11631637	TBL2	transducin (beta)-like 2

210915_x_at	1.45E-02	-0.38298293	TRBC1	T cell receptor beta constant 1
209041_s_at	1.45E-02	0.16573479	UBE2G2	ubiquitin conjugating enzyme E2 G2
224715_at	1.45E-02	0.28100796	WDR34	WD repeat domain 34
59644_at	1.46E-02	-0.24871473	BMP2K	BMP2 inducible kinase
235992_s_at	1.46E-02	0.15142789	CYB5RL	cytochrome b5 reductase like
231297_at	1.46E-02	0.13025961	DOT1L	DOT1 like histone lysine methyltransferase
208430_s_at	1.46E-02	-0.07713375	DTNA	dystrobrevin alpha
228211_at	1.46E-02	-0.20227059	ERCC6L2	ERCC excision repair 6 like 2
217370_x_at	1.46E-02	0.11429974	FUS	FUS RNA binding protein
207972_at	1.46E-02	0.07540252	GLRA1	glycine receptor alpha 1
201554_x_at	1.46E-02	0.23725532	GYG1	glycogenin 1
220805_at	1.46E-02	0.10043315	HRH2	histamine receptor H2
231378_at	1.46E-02	0.13323098	LOC101929897	uncharacterized LOC101929897
203890_s_at	1.46E-02	0.1313548	MIR637///DAPK3	microRNA 637///death associated protein kinase 3
211960_s_at	1.46E-02	0.18942791	RAB7A	RAB7A, member RAS oncogene family
237598_at	1.46E-02	0.0867075	SEC1P	secretory blood group 1, pseudogene
233512_at	1.46E-02	-0.08542905	SH3RF3-AS1	SH3RF3 antisense RNA 1
230072_at	1.46E-02	-0.31403287	TBC1D15	TBC1 domain family member 15
211667_x_at	1.46E-02	0.08152384	TRAV12-2	T cell receptor alpha variable 12-2
209372_x_at	1.46E-02	0.18411167	TUBB2B///TUBB2A	tubulin beta 2B class IIb///tubulin beta 2A class IIa
243833_at	1.46E-02	-0.07804066	UNC5A	unc-5 netrin receptor A
228925_at	1.46E-02	-0.38246483		
222197_s_at	1.46E-02	-0.10404375		
215068_s_at	1.47E-02	0.11693919	FBXL18	F-box and leucine rich repeat protein 18
217940_s_at	1.47E-02	0.28847195	NAXD	NAD(P)HX dehydratase
233609_at	1.47E-02	-0.33289132	PTPRK	protein tyrosine phosphatase, receptor type K
224738_x_at	1.47E-02	-0.1588004	RPL7L1	ribosomal protein L7 like 1
210539_at	1.47E-02	-0.12106332	TTLL5	tubulin tyrosine ligase like 5
220667_at	1.47E-02	-0.07003877	USP49	ubiquitin specific peptidase 49
205308_at	1.47E-02	-0.36720721	ZC2HC1A	zinc finger C2HC-type containing 1A
222162_s_at	1.48E-02	-0.39946163	ADAMTS1	ADAM metallopeptidase with thrombospondin type 1 motif 1
204425_at	1.48E-02	0.25651799	ARHGAP4	Rho GTPase activating protein 4
225707_at	1.48E-02	-0.22561155	ARL6IP6	ADP ribosylation factor like GTPase 6 interacting protein 6
225823_at	1.48E-02	0.32433918	C19orf70	chromosome 19 open reading frame 70
225919_s_at	1.48E-02	-0.33541463	C9orf72	chromosome 9 open reading frame 72
217209_at	1.48E-02	0.10703668	CEACAM3	carcinoembryonic antigen related cell adhesion molecule 3
225164_s_at	1.48E-02	-0.25302001	EIF2AK4	eukaryotic translation initiation factor 2 alpha kinase 4
215660_s_at	1.48E-02	0.1156888	MAST2	microtubule associated serine/threonine kinase 2
239562_at	1.48E-02	-0.26485192	MTHFD2L	methylenetetrahydrofolate dehydrogenase (NADP+ dependent) 2-like
208690_s_at	1.48E-02	0.20687161	PDLIM1	PDZ and LIM domain 1
1553127_a_at	1.48E-02	0.06332456	RNF168	ring finger protein 168
201079_at	1.48E-02	0.30690375	SYNGR2	synaptogyrin 2
1569053_at	1.49E-02	-0.17565221	AP3M2	adaptor related protein complex 3 mu 2 subunit
226192_at	1.49E-02	0.11756741	AR	androgen receptor
225030_at	1.49E-02	-0.20738923	BOD1	biorientation of chromosomes in cell division 1
214730_s_at	1.49E-02	0.25361621	GLG1	golgi glycoprotein 1
233487_s_at	1.49E-02	0.17092898	LRRC8A	leucine rich repeat containing 8 family member A
235993_at	1.49E-02	0.24829369	PSMF1	proteasome inhibitor subunit 1
210293_s_at	1.49E-02	0.25296893	SEC23B	Sec23 homolog B, coat complex II component
209622_at	1.49E-02	0.15353087	STK16	serine/threonine kinase 16
213011_s_at	1.49E-02	0.28457276	TPI1	triosephosphate isomerase 1
232630_at	1.50E-02	-0.11932305	APIP	APAF1 interacting protein
210649_s_at	1.50E-02	0.25586164	ARID1A	AT-rich interaction domain 1A
224196_x_at	1.50E-02	-0.213054	DPH5	diphthamide biosynthesis 5
229543_at	1.50E-02	-0.17006749	FAM26F	family with sequence similarity 26 member F
230492_s_at	1.50E-02	0.42845687	GPCPD1	glycerophosphocholine phosphodiesterase 1
230194_at	1.50E-02	-0.24708411	LRPPRC	leucine rich pentatricopeptide repeat containing
202145_at	1.50E-02	0.44459036	LY6E	lymphocyte antigen 6 complex, locus E
218388_at	1.50E-02	0.26461743	PGLS	6-phosphogluconolactonase

220872_at	1.50E-02	0.0602487	PRO2964	uncharacterized protein PRO2964
212699_at	1.50E-02	0.18253341	SCAMP5	secretory carrier membrane protein 5
210613_s_at	1.50E-02	0.09703982	SYNGR1	synaptogyrin 1
1562255_at	1.50E-02	-0.10827082	SYTL3	synaptotagmin like 3
224584_at	1.50E-02	0.24674196	TMEM230	transmembrane protein 230
204728_s_at	1.50E-02	-0.33301884	WDHD1	WD repeat and HMG-box DNA binding protein 1
244743_x_at	1.50E-02	-0.2764479	ZNF138	zinc finger protein 138
216490_x_at	1.50E-02	0.10984016		
221119_at	1.51E-02	0.10112759	ARHGEF38	Rho guanine nucleotide exchange factor 38
214255_at	1.51E-02	-0.14284211	ATP10A	ATPase phospholipid transporting 10A (putative)
209056_s_at	1.51E-02	-0.17817289	CDC5L	cell division cycle 5 like
204399_s_at	1.51E-02	0.08180924	EML2	echinoderm microtubule associated protein like 2
1552445_a_at	1.51E-02	0.06769617	ESX1	ESX homeobox 1
1564112_at	1.51E-02	0.09188308	FAM71A	family with sequence similarity 71 member A
201552_at	1.51E-02	0.31211408	LAMP1	lysosomal associated membrane protein 1
212089_at	1.51E-02	0.19293808	LMNA	lamin A/C
230170_at	1.51E-02	-0.27057575	OSM	oncostatin M
243904_at	1.51E-02	-0.29121843	STXBP5	syntaxin binding protein 5
1560553_at	1.51E-02	0.08873139	TIAF1	TGFB1-induced anti-apoptotic factor 1
214008_at	1.51E-02	-0.12700045	TWF1	twinfilin actin binding protein 1
219348_at	1.51E-02	0.26950691	USE1	unconventional SNARE in the ER 1
240016_at	1.51E-02	-0.16875502		
1562612_at	1.51E-02	-0.15234669		
208848_at	1.52E-02	-0.27463768	ADH5	alcohol dehydrogenase 5 (class III), chi polypeptide
218555_at	1.52E-02	0.18255433	ANAPC2	anaphase promoting complex subunit 2
234495_at	1.52E-02	-0.10590389	KLK15	kallikrein related peptidase 15
1556511_a_at	1.52E-02	0.06385481	NFIA-AS2	NFIA antisense RNA 2
237008_at	1.52E-02	-0.09428181	POU3F3	POU class 3 homeobox 3
243401_at	1.52E-02	-0.0623568		
1570491_at	1.52E-02	0.0605904		
208637_x_at	1.53E-02	0.30522979	ACTN1	actinin alpha 1
221861_at	1.53E-02	0.1808138	ARRB1	arrestin beta 1
212943_at	1.53E-02	-0.22008288	C2CD5	C2 calcium dependent domain containing 5
213714_at	1.53E-02	-0.37065084	CACNB2	calcium voltage-gated channel auxiliary subunit beta 2
210684_s_at	1.53E-02	-0.10905005	DLG4	discs large MAGUK scaffold protein 4
208631_s_at	1.53E-02	0.2651027	HADHA	hydroxyacyl-CoA dehydrogenase/3-ketoacyl-CoA thiolase/enoyl-CoA hydratase (trifunctional protein), alpha subunit
226510_at	1.53E-02	-0.27534362	HEATR5A	HEAT repeat containing 5A
219095_at	1.53E-02	0.15131729	PLA2G4B///JMJD7-PLA2G4B	phospholipase A2 group IVB///JMJD7-PLA2G4B readthrough
201494_at	1.53E-02	-0.2965309	PRCP	prolylcarboxypeptidase
201680_x_at	1.53E-02	0.16919866	SRRT	serrate, RNA effector molecule
204496_at	1.53E-02	-0.22865627	STRN3	striatin 3
201447_at	1.53E-02	-0.2027605	TIA1	TIA1 cytotoxic granule-associated RNA binding protein
203868_s_at	1.53E-02	-0.42602071	VCAM1	vascular cell adhesion molecule 1
241569_at	1.53E-02	-0.09326937		
203608_at	1.54E-02	-0.44904716	ALDH5A1	aldehyde dehydrogenase 5 family member A1
203233_at	1.54E-02	0.19909634	IL4R	interleukin 4 receptor
241416_at	1.54E-02	-0.16735229	LOC101928806	uncharacterized LOC101928806
213178_s_at	1.54E-02	0.19381916	MAPK8IP3	mitogen-activated protein kinase 8 interacting protein 3
230352_at	1.54E-02	-0.3115287	PRPS2	phosphoribosyl pyrophosphate synthetase 2
205925_s_at	1.54E-02	0.11019936	RAB3B	RAB3B, member RAS oncogene family
208270_s_at	1.54E-02	0.23906985	RNPEP	arginyl aminopeptidase
37462_i_at	1.54E-02	0.23765531	SF3A2	splicing factor 3a subunit 2
1552281_at	1.54E-02	0.34900809	SLC39A5	solute carrier family 39 member 5
209937_at	1.54E-02	0.77514583	TM4SF4	transmembrane 4 L six family member 4
207641_at	1.54E-02	-0.08877886	TNFRSF13B	TNF receptor superfamily member 13B
241192_at	1.54E-02	-0.07090603		
1557468_at	1.54E-02	0.04919222		
221071_at	1.54E-02	0.15149332		
1563115_at	1.54E-02	-0.07505875		
210815_s_at	1.55E-02	-0.13335306	CALCRL	calcitonin receptor like receptor

231481_at	1.55E-02	0.1016354	CCNB3	cyclin B3
202799_at	1.55E-02	0.26457042	CLPP	caseinolytic mitochondrial matrix peptidase proteolytic subunit
220725_x_at	1.55E-02	-0.08983172	DNAH3	dynein axonemal heavy chain 3
218144_s_at	1.55E-02	0.21672391	INF2	inverted formin, FH2 and WH2 domain containing
227675_at	1.55E-02	0.14167768	LRSAM1	leucine rich repeat and sterile alpha motif containing 1
213490_s_at	1.55E-02	0.15715616	MAP2K2	mitogen-activated protein kinase kinase 2
225738_at	1.55E-02	0.20147008	RAPGEF1	Rap guanine nucleotide exchange factor 1
37796_at	1.55E-02	0.09890877	SAP25///LRCH4	Sin3A associated protein 25///leucine rich repeats and calponin homology domain containing 4
225649_s_at	1.55E-02	0.29176678	STK35	serine/threonine kinase 35
218521_s_at	1.55E-02	-0.27800295	UBE2W	ubiquitin conjugating enzyme E2 W (putative)
235851_s_at	1.55E-02	-0.1536166		
230925_at	1.56E-02	-0.20219578	APBB1IP	amyloid beta precursor protein binding family B member 1 interacting protein
202109_at	1.56E-02	0.20473256	ARFIP2	ADP ribosylation factor interacting protein 2
230056_at	1.56E-02	-0.30655942	BPTF	bromodomain PHD finger transcription factor
208432_s_at	1.56E-02	-0.079942	CACNA1E	calcium voltage-gated channel subunit alpha1 E
218288_s_at	1.56E-02	-0.22156505	CCDC90B	coiled-coil domain containing 90B
200698_at	1.56E-02	0.21275416	KDELR2	KDEL endoplasmic reticulum protein retention receptor 2
231011_at	1.56E-02	-0.32568628	LARP1B	La ribonucleoprotein domain family member 1B
1559765_a_at	1.56E-02	0.09229412	NALT1	NOTCH1 associated lncRNA in T-cell acute lymphoblastic leukemia 1
231387_at	1.56E-02	-0.11891203	PXDC1	PX domain containing 1
216181_at	1.56E-02	0.10116885	SYNJ2	synaptojanin 2
200055_at	1.56E-02	0.25059865	TAF10	TATA-box binding protein associated factor 10
1569502_s_at	1.56E-02	0.08507783	TP73-AS1	TP73 antisense RNA 1
214699_x_at	1.56E-02	-0.09206951	WIPI2	WD repeat domain, phosphoinositide interacting 2
232584_at	1.56E-02	0.1200884		
217401_at	1.56E-02	-0.08953052		
238169_at	1.56E-02	-0.1540573		
221815_at	1.57E-02	0.47050989	ABHD2	abhydrolase domain containing 2
240082_s_at	1.57E-02	0.07781786	C17orf74	chromosome 17 open reading frame 74
202965_s_at	1.57E-02	0.6564176	CAPN6	calpain 6
211272_s_at	1.57E-02	0.11307257	DGKA	diacylglycerol kinase alpha
207562_at	1.57E-02	0.1070362	DGKQ	diacylglycerol kinase theta
221407_at	1.57E-02	-0.08958798	GJD2	gap junction protein delta 2
34221_at	1.57E-02	0.16068186	HMGXB3	HMG-box containing 3
1560439_at	1.57E-02	0.07999526	LINGO3	leucine rich repeat and Ig domain containing 3
232129_s_at	1.57E-02	0.14943386	LZTS2	leucine zipper tumor suppressor 2
218544_s_at	1.57E-02	-0.13994017	RCL1	RNA terminal phosphate cyclase like 1
233451_at	1.57E-02	0.07836094	SLC52A3	solute carrier family 52 member 3
201247_at	1.57E-02	0.19416195	SREBF2	sterol regulatory element binding transcription factor 2
44696_at	1.57E-02	0.1235845	TBC1D13	TBC1 domain family member 13
1559667_at	1.57E-02	0.11293614		
226289_at	1.58E-02	0.17433591	CAPRIN1	cell cycle associated protein 1
1553397_at	1.58E-02	0.09391269	CCDC13	coiled-coil domain containing 13
210956_at	1.58E-02	0.08784199	CH17-360D5.1///NPY4R	neuropeptide Y receptor type 4-like///neuropeptide Y receptor Y4
213086_s_at	1.58E-02	0.18490891	CSNK1A1	casein kinase 1 alpha 1
1556048_at	1.58E-02	0.11305273	LOC100507564	uncharacterized LOC100507564
213078_x_at	1.58E-02	0.27594685	LPCAT4	lysophosphatidylcholine acyltransferase 4
214078_at	1.58E-02	-0.09322409	PAK3	p21 (RAC1) activated kinase 3
1568949_at	1.58E-02	0.10017246	PITPNC1	phosphatidylinositol transfer protein, cytoplasmic 1
224232_s_at	1.58E-02	0.27481107	PRELID1	PRELI domain containing 1
234754_at	1.58E-02	0.09293665	SLC37A1	solute carrier family 37 member 1
212198_s_at	1.58E-02	0.25432103	TM9SF4	transmembrane 9 superfamily member 4
234720_s_at	1.58E-02	0.09365084	TOB2	transducer of ERBB2, 2
225928_at	1.58E-02	-0.18984412	VTI1B	vesicle transport through interaction with t-SNAREs 1B

213908_at	1.58E-02	-0.16512276	WHAMMP2///WHAMMP3	WAS protein homolog associated with actin, golgi membranes and microtubules pseudogene 2///WAS protein homolog associated with actin, golgi membranes and microtubules pseudogene 3
1568620_at	1.59E-02	0.14411196	CSAD	cysteine sulfinic acid decarboxylase
207192_at	1.59E-02	-0.12489585	DNASE1L2	deoxyribonuclease 1 like 2
213468_at	1.59E-02	0.15169248	ERCC2	ERCC excision repair 2, TFIIH core complex helicase subunit
220615_s_at	1.59E-02	-0.27200617	FAR2	fatty acyl-CoA reductase 2
244668_at	1.59E-02	-0.0760864	LOC100505902	uncharacterized LOC100505902
214570_x_at	1.59E-02	0.09307471	LOC102725315///LOC102725072///LOC727983///POM121L10P///POM121L4P///POM121L8P///POM121L9P///POM121L1P	putative POM121-like protein 1-like///uncharacterized LOC102725072///putative POM121-like protein 1-like///POM121 transmembrane nucleoporin like 10, pseudogene///POM121 transmembrane nucleoporin like 4, pseudogene///POM121 transmembrane nucleoporin like 8, pseudogene///POM121 transmembrane nucleoporin like 9, pseudogene///POM121 transmembrane nucleoporin like 1, pseudogene
202189_x_at	1.59E-02	0.1550388	MIR4745///PTBP1	microRNA 4745///polypyrimidine tract binding protein 1
236372_at	1.59E-02	-0.11332539	PANX1	pannexin 1
209578_s_at	1.59E-02	0.15921756	POFUT2	protein O-fucosyltransferase 2
213901_x_at	1.59E-02	0.18262145	RBFOX2	RNA binding protein, fox-1 homolog 2
222138_s_at	1.59E-02	0.28778817	WDR13	WD repeat domain 13
222777_s_at	1.59E-02	-0.3362059	WHSC1	Wolf-Hirschhorn syndrome candidate 1
215012_at	1.59E-02	-0.24889666	ZNF451	zinc finger protein 451
234381_at	1.59E-02	0.09612704		
236230_at	1.59E-02	0.06622215		
1552701_a_at	1.60E-02	-0.46129452	CARD16	caspase recruitment domain family member 16
226154_at	1.60E-02	-0.20748958	DNM1L	dynamin 1 like
233261_at	1.60E-02	-0.13879443	EBF1	early B-cell factor 1
228418_at	1.60E-02	-0.35437589	EXOC5	exocyst complex component 5
218461_at	1.60E-02	-0.19998231	GPN3	GPN-loop GTPase 3
216222_s_at	1.60E-02	0.29209495	MYO10	myosin X
210016_at	1.60E-02	-0.06835084	MYT1L	myelin transcription factor 1 like
231899_at	1.60E-02	-0.83192822	ZC3H12C	zinc finger CCCH-type containing 12C
237062_at	1.60E-02	-0.3189339		
242628_at	1.60E-02	-0.19012016		
243086_at	1.60E-02	-0.12928832		
207163_s_at	1.61E-02	0.24972361	AKT1	AKT serine/threonine kinase 1
215259_s_at	1.61E-02	0.11833462	CADM4	cell adhesion molecule 4
243043_at	1.61E-02	0.06329642	EFCAB10	EF-hand calcium binding domain 10
201816_s_at	1.61E-02	-0.23478366	GBAS	glioblastoma amplified sequence
210307_s_at	1.61E-02	0.11912795	KLHL25	kelch like family member 25
201650_at	1.61E-02	0.37145041	KRT19	keratin 19
212241_at	1.61E-02	0.20672301	MYZAP///GCOM1///POLR2M	myocardial zonula adherens protein///GRINL1A complex locus 1///RNA polymerase II subunit M
227160_s_at	1.61E-02	0.33652459	NDUFAF5	NADH:ubiquinone oxidoreductase complex assembly factor 5
235971_at	1.61E-02	-0.19589012	TIFA	TRAF interacting protein with forkhead associated domain
227393_at	1.62E-02	0.32346144	ANO9	anoctamin 9
234607_at	1.62E-02	0.08980424	ARRDC1	arrestin domain containing 1
210622_x_at	1.62E-02	0.29931577	CDK10	cyclin dependent kinase 10
204203_at	1.62E-02	0.23847084	CEBPG	CCAAT/enhancer binding protein gamma
1553570_x_at	1.62E-02	0.12829391	COX2	cytochrome c oxidase subunit II
207257_at	1.62E-02	0.08497812	EPO	erythropoietin
232013_at	1.62E-02	-0.18069091	ERCC6L2	ERCC excision repair 6 like 2
202927_at	1.62E-02	0.16722163	PIN1	peptidylprolyl cis/trans isomerase, NIMA-interacting 1
203790_s_at	1.62E-02	-0.29924162	RIDA	reactive intermediate imine deaminase A homolog
242773_at	1.62E-02	0.37332122	SLC5A1	solute carrier family 5 member 1
212574_x_at	1.62E-02	0.13183875	TMEM259	transmembrane protein 259
209002_s_at	1.63E-02	0.17826927	CALCOCO1	calcium binding and coiled-coil domain 1

204236_at	1.63E-02	-0.32974953	FLI1	Fli-1 proto-oncogene, ETS transcription factor
236779_at	1.63E-02	-0.12322143	MRPS5	mitochondrial ribosomal protein S5
219891_at	1.63E-02	0.22441069	PGPEP1	pyroglutamyl-peptidase I
213721_at	1.63E-02	-0.18437646	SOX2	SRY-box 2
217658_at	1.63E-02	0.11188458	THAP3	THAP domain containing 3
201292_at	1.63E-02	-0.26763008	TOP2A	topoisomerase (DNA) II alpha
235779_at	1.63E-02	-0.09690857	ZNF790-AS1	ZNF790 antisense RNA 1
219050_s_at	1.63E-02	0.2010325	ZNHIT2	zinc finger HIT-type containing 2
234659_at	1.63E-02	0.07199167		
242409_at	1.63E-02	-0.09494592		
AFFX-BioB-3_at	1.63E-02	-0.15564675		
203462_x_at	1.64E-02	0.28350715	EIF3B	eukaryotic translation initiation factor 3 subunit B
230435_at	1.64E-02	-0.36019597	FAM228B	family with sequence similarity 228 member B
215554_at	1.64E-02	-0.13742454	GPLD1	glycosylphosphatidylinositol specific phospholipase D1
203006_at	1.64E-02	0.20609395	INPP5A	inositol polyphosphate-5-phosphatase A
220247_at	1.64E-02	-0.0568411	KNL1	kinetochore scaffold 1
210519_s_at	1.64E-02	0.49173908	NQO1	NAD(P)H quinone dehydrogenase 1
218223_s_at	1.64E-02	0.1730091	PLEKHO1	pleckstrin homology domain containing O1
231002_s_at	1.64E-02	-0.1520697	RABEP1	rabaptin, RAB GTPase binding effector protein 1
244656_at	1.64E-02	-0.09358679	RASL10B	RAS like family 10 member B
225840_at	1.64E-02	0.15230803	TEF	TEF, PAR bZIP transcription factor
240051_at	1.64E-02	-0.08264383	TPD52L3	tumor protein D52 like 3
210389_x_at	1.64E-02	-0.19780215	TUBD1	tubulin delta 1
214713_at	1.64E-02	-0.20095091	YLPM1	YLP motif containing 1
240517_at	1.65E-02	-0.09744893	CBS	cystathionine-beta-synthase
205774_at	1.65E-02	0.39089997	F12	coagulation factor XII
206678_at	1.65E-02	0.08578777	GABRA1	gamma-aminobutyric acid type A receptor alpha1 subunit
1560727_at	1.65E-02	-0.11967827	HEATR4	HEAT repeat containing 4
213281_at	1.65E-02	-0.24227906	JUN	Jun proto-oncogene, AP-1 transcription factor subunit
1553585_a_at	1.65E-02	0.0712139	MAGEA10-MAGEA5///MAGEA5	MAGEA10-MAGEA5 readthrough///MAGE family member A5
32811_at	1.65E-02	0.16009317	MYO1C	myosin IC
1558247_s_at	1.65E-02	0.0718748	NTN5	netrin 5
221027_s_at	1.65E-02	-0.26322489	PLA2G12A	phospholipase A2 group XIIA
226619_at	1.65E-02	-0.19095905	SENP1	SUMO1/sentrin specific peptidase 1
229410_at	1.65E-02	0.14286188	SLC35E1	solute carrier family 35 member E1
223399_x_at	1.65E-02	-0.07117844	SMIM4	small integral membrane protein 4
223867_at	1.65E-02	0.07178631	TEKT3	tektin 3
220117_at	1.65E-02	-0.08441213	ZNF385D	zinc finger protein 385D
213454_at	1.66E-02	-0.23872027	APITD1-CORT///APITD1	APITD1-CORT readthrough///apoptosis-inducing, TAF9-like domain 1
214953_s_at	1.66E-02	0.26414721	APP	amyloid beta precursor protein
209662_at	1.66E-02	-0.27013319	CETN3	centrin 3
201119_s_at	1.66E-02	0.26559636	COX8A	cytochrome c oxidase subunit 8A
210279_at	1.66E-02	-0.25513804	GPR18	G protein-coupled receptor 18
244490_at	1.66E-02	-0.23747456	LINC01355	long intergenic non-protein coding RNA 1355
227589_at	1.66E-02	0.07925506	PITPNC1	phosphatidylinositol transfer protein, cytoplasmic 1
221049_s_at	1.66E-02	0.12153366	POLL	polymerase (DNA) lambda
207306_at	1.66E-02	-0.14318642	TCF15	transcription factor 15 (basic helix-loop-helix)
239939_at	1.66E-02	-0.09028918		
243180_at	1.66E-02	0.08226076		
229596_at	1.67E-02	-0.3361593	AMDHD1	amidohydrolase domain containing 1
212152_x_at	1.67E-02	0.18539582	ARID1A	AT-rich interaction domain 1A
211833_s_at	1.67E-02	0.28138332	BAX	BCL2 associated X, apoptosis regulator
202315_s_at	1.67E-02	0.36148345	BCR	BCR, RhoGEF and GTPase activating protein
235026_at	1.67E-02	-0.23933334	C12orf66	chromosome 12 open reading frame 66
1556498_at	1.67E-02	-0.0948112	FAM69A	family with sequence similarity 69 member A
231976_at	1.67E-02	-0.18063939	LINS1	lines homolog 1
1560384_a_at	1.67E-02	0.08766558	LOC101928658	uncharacterized LOC101928658
227186_s_at	1.67E-02	0.25537996	MRPL41	mitochondrial ribosomal protein L41
221970_s_at	1.67E-02	-0.228188	NOL11	nucleolar protein 11
244751_at	1.67E-02	0.08159358	PCP2	Purkinje cell protein 2

1556122_at	1.67E-02	0.07154682	RAB11B-AS1	RAB11B antisense RNA 1
206146_s_at	1.67E-02	-0.11753956	RHAG	Rh-associated glycoprotein
222575_at	1.67E-02	-0.12314265	SETD5	SET domain containing 5
206487_at	1.67E-02	0.20341716	SUN1	Sad1 and UNC84 domain containing 1
207410_s_at	1.67E-02	0.07680904	TLX2	T-cell leukemia homeobox 2
1555503_a_at	1.67E-02	0.07499781	TMCC2	transmembrane and coiled-coil domain family 2
1552648_a_at	1.67E-02	0.20780654	TNFRSF10A	TNF receptor superfamily member 10a
226562_at	1.67E-02	-0.2126337	ZSCAN29	zinc finger and SCAN domain containing 29
228366_at	1.67E-02	-0.24448979		
217657_at	1.67E-02	0.09498794		
204999_s_at	1.68E-02	0.15674547	ATF5	activating transcription factor 5
1552690_a_at	1.68E-02	0.12622873	CACNA2D4	calcium voltage-gated channel auxiliary subunit alpha2delta 4
1565149_at	1.68E-02	-0.25484481	DYNC2H1	dynein cytoplasmic 2 heavy chain 1
223319_at	1.68E-02	-0.34906441	GPHN	gephyrin
201137_s_at	1.68E-02	-0.41977573	HLA-DPB1	major histocompatibility complex, class II, DP beta 1
230388_s_at	1.68E-02	-0.32904736	KANSL1-AS1	KANSL1 antisense RNA 1
219236_at	1.68E-02	0.14798127	PAQR6	progestin and adipoQ receptor family member 6
216834_at	1.68E-02	-0.41021324	RGS1	regulator of G-protein signaling 1
225134_at	1.68E-02	0.12987616	SPRYD3	SPRY domain containing 3
217567_at	1.68E-02	0.07453422	TGM4	transglutaminase 4
229076_s_at	1.68E-02	-0.26522977	TOMM22	translocase of outer mitochondrial membrane 22
46270_at	1.68E-02	0.16971581	UBAP1	ubiquitin associated protein 1
223792_at	1.68E-02	-0.13878351	ZNF2	zinc finger protein 2
215358_x_at	1.68E-02	-0.3340899	ZNF37BP	zinc finger protein 37B, pseudogene
241400_at	1.68E-02	0.16855213		
229252_at	1.69E-02	0.20828383	ATG9B	autophagy related 9B
235248_at	1.69E-02	-0.14616187	BTBD9	BTB domain containing 9
229381_at	1.69E-02	-0.09593223	C1orf64	chromosome 1 open reading frame 64
218175_at	1.69E-02	0.25356869	CCDC92	coiled-coil domain containing 92
1569685_at	1.69E-02	-0.18955534	COX10	COX10, heme A:farnesyltransferase cytochrome c oxidase assembly factor
213980_s_at	1.69E-02	-0.18578391	CTBP1	C-terminal binding protein 1
205035_at	1.69E-02	0.11599071	CTDP1	CTD phosphatase subunit 1
223976_at	1.69E-02	-0.08754813	FUT10	fucosyltransferase 10
211905_s_at	1.69E-02	0.10889255	ITGB4	integrin subunit beta 4
1557022_at	1.69E-02	0.07717775	MIR4300HG	MIR4300 host gene
222530_s_at	1.69E-02	0.32601719	MKKS	McKusick-Kaufman syndrome
209262_s_at	1.69E-02	0.16966041	NR2F6	nuclear receptor subfamily 2 group F member 6
223464_at	1.69E-02	0.24015559	OSBPL5	oxysterol binding protein like 5
201233_at	1.69E-02	0.21805354	PSMD13	proteasome 26S subunit, non-ATPase 13
219988_s_at	1.69E-02	0.1666449	RNF220	ring finger protein 220
218758_s_at	1.69E-02	0.19582679	RRP1	ribosomal RNA processing 1
236535_at	1.69E-02	-0.26322954	SMC6	structural maintenance of chromosomes 6
216228_s_at	1.69E-02	-0.34071385	WDHD1	WD repeat and HMG-box DNA binding protein 1
1558622_a_at	1.69E-02	0.14631329	ZNF548	zinc finger protein 548
231260_at	1.69E-02	-0.07482243	ZNF582-AS1	ZNF582 antisense RNA 1 (head to head)
215571_at	1.69E-02	0.08050637		
211852_s_at	1.70E-02	0.24203755	ATRN	attractin
225607_at	1.70E-02	-0.21286318	CCDC43	coiled-coil domain containing 43
210780_at	1.70E-02	-0.06987146	ESR2	estrogen receptor 2
230808_at	1.70E-02	-0.24739148	FNTA	farnesyltransferase, CAAX box, alpha
205580_s_at	1.70E-02	0.19906854	HRH1	histamine receptor H1
230166_at	1.70E-02	-0.22916455	KIAA1958	KIAA1958
204759_at	1.70E-02	-0.40751832	RCBTB2	RCC1 and BTB domain containing protein 2
212844_at	1.70E-02	0.12078952	RRP1B	ribosomal RNA processing 1B
201826_s_at	1.70E-02	-0.23420282	SCCPDH	saccharopine dehydrogenase (putative)
209611_s_at	1.70E-02	0.11900128	SLC1A4	solute carrier family 1 member 4
229513_at	1.70E-02	-0.30134732	STRBP	spermatid perinuclear RNA binding protein
216920_s_at	1.70E-02	-0.38285556	TARP///TRGV9///TRGC2	TCR gamma alternate reading frame protein///T cell receptor gamma variable 9///T cell receptor gamma constant 2
237428_at	1.70E-02	-0.07397061	TEX43	testis expressed 43

213191_at	1.70E-02	0.1670853	TICAM1	toll like receptor adaptor molecule 1
1553619_a_at	1.70E-02	-0.06290636	TRIM43B///TRIM43	tripartite motif containing 43B///tripartite motif containing 43
202009_at	1.70E-02	0.11034712	TWF2	twinfilin actin binding protein 2
215218_s_at	1.70E-02	0.10087655	WDR62	WD repeat domain 62
1558448_a_at	1.70E-02	-0.08087293		
217211_at	1.71E-02	0.15069353	ACTBP9	actin, beta pseudogene 9
218880_at	1.71E-02	0.33372637	FOSL2	FOS like 2, AP-1 transcription factor subunit
1554251_at	1.71E-02	-0.30252299	HP1BP3	heterochromatin protein 1 binding protein 3
238933_at	1.71E-02	0.21061873	IRS1	insulin receptor substrate 1
203225_s_at	1.71E-02	0.23412255	RFK	riboflavin kinase
229677_at	1.71E-02	0.08199981	SLC39A3	solute carrier family 39 member 3
214833_at	1.71E-02	0.32236702	TMEM63A	transmembrane protein 63A
221712_s_at	1.71E-02	0.26507811	WDR74	WD repeat domain 74
228216_at	1.71E-02	-0.15943996	ZBTB37	zinc finger and BTB domain containing 37
219929_s_at	1.71E-02	0.22718053	ZFYVE21	zinc finger FYVE-type containing 21
240766_at	1.71E-02	-0.05775173		
205434_s_at	1.72E-02	0.18193945	AAK1	AP2 associated kinase 1
201043_s_at	1.72E-02	0.2088042	ANP32A	acidic nuclear phosphoprotein 32 family member A
217468_at	1.72E-02	0.08367469	CYP2D6	cytochrome P450 family 2 subfamily D member 6
1568377_x_at	1.72E-02	0.13594816	DEFB124	defensin beta 124
224746_at	1.72E-02	0.22763573	KIAA1522	KIAA1522
201412_at	1.72E-02	0.27794303	LRP10	LDL receptor related protein 10
221215_s_at	1.72E-02	0.24171062	RIPK4	receptor interacting serine/threonine kinase 4
1555356_a_at	1.72E-02	-0.05998517	SCML4	sex comb on midleg-like 4 (Drosophila)
230705_at	1.72E-02	0.06458736	SLC2A5	solute carrier family 2 member 5
242408_at	1.72E-02	-0.29122285	STYX	serine/threonine/tyrosine interacting protein
237301_at	1.72E-02	0.26843434		
AFFX-BioB-5_at	1.72E-02	-0.14465549		
240623_at	1.72E-02	0.08750433		
231281_at	1.72E-02	-0.28127176		
208544_at	1.73E-02	0.096331	ADRA2B	adrenoceptor alpha 2B
206545_at	1.73E-02	-0.20278742	CD28	CD28 molecule
240757_at	1.73E-02	-0.12889842	CLASP1	cytoplasmic linker associated protein 1
213756_s_at	1.73E-02	0.09617408	HSF1	heat shock transcription factor 1
1560168_at	1.73E-02	-0.0884147	LINC01330	long intergenic non-protein coding RNA 1330
202793_at	1.73E-02	0.18957999	LPCAT3	lysophosphatidylcholine acyltransferase 3
226571_s_at	1.73E-02	-0.21690995	PTPRS	protein tyrosine phosphatase, receptor type S
226035_at	1.73E-02	-0.24287805	USP31	ubiquitin specific peptidase 31
231409_at	1.73E-02	-0.09296482		
36865_at	1.74E-02	-0.17192275	ANGEL1	angel homolog 1
224478_s_at	1.74E-02	0.28571906	C7orf50	chromosome 7 open reading frame 50
55093_at	1.74E-02	0.17273375	CHPF2	chondroitin polymerizing factor 2
234733_s_at	1.74E-02	-0.24662707	FANCM	Fanconi anemia complementation group M
215749_s_at	1.74E-02	0.18816901	GORASP1	golgi reassembly stacking protein 1
1568894_at	1.74E-02	0.08298576	LOC101929007	WAS/WASL-interacting protein family member 1
232613_at	1.74E-02	-0.16460652	PBRM1	polybromo 1
1553813_s_at	1.74E-02	0.07107834	TLE6	transducin like enhancer of split 6
1553696_s_at	1.74E-02	-0.19506287	ZNF569	zinc finger protein 569
243920_x_at	1.74E-02	0.12541821		
217352_at	1.74E-02	-0.15976163		
210098_s_at	1.74E-02	-0.30746593		
238647_at	1.75E-02	-0.29851008	C14orf28	chromosome 14 open reading frame 28
218102_at	1.75E-02	-0.22436941	DERA	deoxyribose-phosphate aldolase
213787_s_at	1.75E-02	0.30732125	EBP	emopamil binding protein (sterol isomerase)
216032_s_at	1.75E-02	0.29140195	ERGIC3	ERGIC and golgi 3
241363_at	1.75E-02	0.14454504	EXD3	exonuclease 3'-5' domain containing 3
243750_x_at	1.75E-02	0.14879987	FAM207A	family with sequence similarity 207 member A
215214_at	1.75E-02	-0.31044774	IGLC1	immunoglobulin lambda constant 1
227295_at	1.75E-02	-0.34782941	IKBIP	IKBKB interacting protein
202861_at	1.75E-02	0.15414574	MIR6883///PER1	microRNA 6883///period circadian clock 1
200654_at	1.75E-02	0.27887288	P4HB	prolyl 4-hydroxylase subunit beta

224421_x_at	1.75E-02	0.06413248	PMCHL1	pro-melanin concentrating hormone like 1 (pseudogene)
209402_s_at	1.75E-02	0.09305443	SLC12A4	solute carrier family 12 member 4
227581_at	1.75E-02	0.16607317	TECPR1	tectonin beta-propeller repeat containing 1
220528_at	1.75E-02	-0.16166019	VNN3	vanin 3
236952_at	1.75E-02	0.09120305		
204493_at	1.76E-02	-0.26704029	BID	BH3 interacting domain death agonist
206517_at	1.76E-02	0.13008137	CDH16	cadherin 16
221851_at	1.76E-02	0.10688334	DCAF15	DDB1 and CUL4 associated factor 15
1560300_a_at	1.76E-02	0.09073189	DMRTC1B///DMRTC1	DMRT like family C1B///DMRT like family C1
226521_s_at	1.76E-02	-0.3126215	FAM175A	family with sequence similarity 175 member A
219357_at	1.76E-02	0.22706282	GTPBP1	GTP binding protein 1
208652_at	1.76E-02	0.20290359	PPP2CA	protein phosphatase 2 catalytic subunit alpha
224573_at	1.76E-02	0.31873434	RNASEK-C17orf49///RNASEK///C17orf49	RNASEK-C17orf49 readthrough///ribonuclease K///chromosome 17 open reading frame 49
200716_x_at	1.76E-02	0.16219645	RPL13AP5///SNORD32A///SNORD33///SNORD34///SNORD35A///RPL13A	ribosomal protein L13a pseudogene 5///small nucleolar RNA, C/D box 32A///small nucleolar RNA, C/D box 33///small nucleolar RNA, C/D box 34///small nucleolar RNA, C/D box 35A///ribosomal protein L13a
39835_at	1.76E-02	0.16674751	SBF1	SET binding factor 1
200909_s_at	1.76E-02	0.27116035	SNORA52///RPLP2	small nucleolar RNA, H/ACA box 52///ribosomal protein lateral stalk subunit P2
1569003_at	1.76E-02	-0.3573542	VMP1	vacuole membrane protein 1
233878_s_at	1.76E-02	0.38198493	XRN2	5'-3' exoribonuclease 2
230063_at	1.76E-02	0.45696339	ZNF264	zinc finger protein 264
238607_at	1.76E-02	0.14146803	ZNF296	zinc finger protein 296
1560639_at	1.76E-02	0.07966967		
237812_at	1.76E-02	-0.0658697		
236319_at	1.76E-02	0.07901688		
232325_at	1.76E-02	-0.06175444		
220155_s_at	1.77E-02	0.17177345	BRD9	bromodomain containing 9
204600_at	1.77E-02	0.36911688	EPHB3	EPH receptor B3
227159_at	1.77E-02	0.19455848	GHDC	GH3 domain containing
238296_at	1.77E-02	-0.06848998	GLIPR1L1	GLI pathogenesis related 1 like 1
117_at	1.77E-02	0.30444027	HSPA6	heat shock protein family A (Hsp70) member 6
232418_at	1.77E-02	0.06739334	LZTFL1	leucine zipper transcription factor like 1
229327_s_at	1.77E-02	-0.32748069	MAF	MAF bZIP transcription factor
207440_at	1.77E-02	0.0970655	SLC35A2	solute carrier family 35 member A2
237386_at	1.77E-02	-0.15753205	SMIM10L1	small integral membrane protein 10 like 1
244155_x_at	1.77E-02	-0.10744628	TOR2A	torsin family 2 member A
1558976_x_at	1.77E-02	0.09651808	UBALD1	UBA like domain containing 1
208627_s_at	1.77E-02	0.28171639	YBX1	Y-box binding protein 1
216796_s_at	1.77E-02	0.0628642		
207623_at	1.78E-02	0.11826194	ABCF2	ATP binding cassette subfamily F member 2
222200_s_at	1.78E-02	0.14510052	BSDC1	BSD domain containing 1
225690_at	1.78E-02	-0.26199597	CDK12	cyclin dependent kinase 12
219036_at	1.78E-02	-0.33993771	CEP70	centrosomal protein 70
1553569_at	1.78E-02	0.13212153	COX2	cytochrome c oxidase subunit II
220342_x_at	1.78E-02	0.29367313	EDEM3	ER degradation enhancing alpha-mannosidase like protein 3
214088_s_at	1.78E-02	0.45034061	FUT3	fucosyltransferase 3 (Lewis blood group)
211034_s_at	1.78E-02	-0.18639978	HECTD4	HECT domain E3 ubiquitin protein ligase 4
204487_s_at	1.78E-02	0.24469926	KCNQ1	potassium voltage-gated channel subfamily Q member 1
232366_at	1.78E-02	-0.31736107	KIAA0232	KIAA0232
242246_x_at	1.78E-02	-0.08645134	MEG3	maternally expressed 3 (non-protein coding)
234343_s_at	1.78E-02	0.12461903	RASAL2	RAS protein activator like 2
217591_at	1.78E-02	0.27261541	SKIL	SKI-like proto-oncogene
225277_at	1.78E-02	0.1384878	SLC39A13	solute carrier family 39 member 13
208920_at	1.78E-02	-0.4368552	SRI	sorcin
201836_s_at	1.78E-02	-0.22608142	SUPT7L	SPT7-like STAGA complex gamma subunit
212408_at	1.78E-02	0.21678748	TOR1AIP1	torsin 1A interacting protein 1

234261_at	1.78E-02	0.07456382		
231562_at	1.79E-02	0.08428769	APOC2	apolipoprotein C2
201385_at	1.79E-02	-0.1099016	DHX15	DEAH-box helicase 15
239108_at	1.79E-02	-0.31011363	FAR2	fatty acyl-CoA reductase 2
206494_s_at	1.79E-02	0.09541426	ITGA2B	integrin subunit alpha 2b
1556724_at	1.79E-02	0.08417919	LOC100288490	uncharacterized LOC100288490
225222_at	1.79E-02	-0.15903685	MFSD14A	major facilitator superfamily domain containing 14A
234436_x_at	1.79E-02	0.09321627	OBP2A	odorant binding protein 2A
214485_at	1.79E-02	-0.10301032	ODF1	outer dense fiber of sperm tails 1
200971_s_at	1.79E-02	0.12295384	SERP1	stress-associated endoplasmic reticulum protein 1
206554_x_at	1.79E-02	-0.29153848	SETMAR	SET domain and mariner transposase fusion gene
204657_s_at	1.79E-02	0.18974202	SHB	SH2 domain containing adaptor protein B
206097_at	1.79E-02	0.16568896	SLC22A18AS	solute carrier family 22 member 18 antisense
209078_s_at	1.79E-02	0.16138912	TXN2	thioredoxin 2
238358_x_at	1.79E-02	0.07126459		
241419_at	1.80E-02	0.08813228	ARHGAP1	Rho GTPase activating protein 1
40850_at	1.80E-02	0.07886397	FKBP8	FK506 binding protein 8
206883_x_at	1.80E-02	0.08681408	GP9	glycoprotein IX platelet
1554453_at	1.80E-02	0.16492694	HNRNPLL	heterogeneous nuclear ribonucleoprotein L like
240661_at	1.80E-02	0.13412084	LOC100506459	uncharacterized LOC100506459
240576_at	1.80E-02	0.09283123	LOC101930296///TBC1D26///ZNF286A	uncharacterized LOC101930296///TBC1 domain family member 26///zinc finger protein 286A
235389_at	1.80E-02	-0.2540918	PHF20	PHD finger protein 20
230403_at	1.80E-02	-0.32642135	RFX3	regulatory factor X3
225931_s_at	1.80E-02	-0.24579356	RNF213	ring finger protein 213
213009_s_at	1.80E-02	-0.23734934	TRIM37	tripartite motif containing 37
217287_s_at	1.80E-02	-0.12480591	TRPC6	transient receptor potential cation channel subfamily C member 6
202452_at	1.80E-02	0.1136381	ZER1	zyg-11 related cell cycle regulator
1566965_at	1.80E-02	0.08585337		
244587_at	1.81E-02	0.13878406	ATF7	activating transcription factor 7
202946_s_at	1.81E-02	0.29982046	BTBD3	BTB domain containing 3
219309_at	1.81E-02	0.10123872	C22orf46	chromosome 22 open reading frame 46
1554411_at	1.81E-02	0.32243082	CTNNB1	catenin beta 1
214735_at	1.81E-02	-0.25010987	IPCEF1	interaction protein for cytohesin exchange factors 1
234689_at	1.81E-02	-0.10930734	PTCHD4	patched domain containing 4
225639_at	1.81E-02	0.46030513	SKAP2	src kinase associated phosphoprotein 2
46256_at	1.81E-02	0.19112911	SPSB3	splA/ryanodine receptor domain and SOCS box containing 3
234163_at	1.81E-02	0.07343432	UBE3A	ubiquitin protein ligase E3A
63825_at	1.82E-02	0.430371	ABHD2	abhydrolase domain containing 2
207607_at	1.82E-02	0.12903425	ASCL2	achaete-scute family bHLH transcription factor 2
212645_x_at	1.82E-02	0.14247172	BRE	brain and reproductive organ-expressed (TNFRSF1A modulator)
1555355_a_at	1.82E-02	-0.21996063	ETS1	ETS proto-oncogene 1, transcription factor
1561497_at	1.82E-02	0.08313364	MIR3180-2///MIR3180-1///MIR3180-3	microRNA 3180-2///microRNA 3180-1///microRNA 3180-3
228764_s_at	1.82E-02	-0.23011716	NEDD8-MDP1///MDP1	NEDD8-MDP1 readthrough///magnesium dependent phosphatase 1
215618_at	1.82E-02	-0.11717191	RSU1	Ras suppressor protein 1
233961_at	1.82E-02	0.08831312		
234862_at	1.82E-02	-0.07971474		
220084_at	1.83E-02	0.13627616	C14orf105	chromosome 14 open reading frame 105
219040_at	1.83E-02	0.12352929	CORO7	coronin 7
219349_s_at	1.83E-02	-0.25714926	EXOC2	exocyst complex component 2
211465_x_at	1.83E-02	0.26611489	FUT6	fucosyltransferase 6
1555611_s_at	1.83E-02	0.09054873	MBD1	methyl-CpG binding domain protein 1
211080_s_at	1.83E-02	0.2397533	NEK2	NIMA related kinase 2
200039_s_at	1.83E-02	0.26284905	PSMB2	proteasome subunit beta 2
219102_at	1.83E-02	0.13128359	RCN3	reticulocalbin 3
236620_at	1.83E-02	-0.23451403	RIF1	replication timing regulatory factor 1
227849_at	1.83E-02	-0.13225013	RP9	retinitis pigmentosa 9 (autosomal dominant)
1559945_at	1.83E-02	0.08173308	RUVBL2	RuvB like AAA ATPase 2

206543_at	1.83E-02	-0.13970295	SMARCA2	SWI/SNF related, matrix associated, actin dependent regulator of chromatin, subfamily a, member 2
210073_at	1.83E-02	-0.10045519	ST8SIA1	ST8 alpha-N-acetyl-neuraminide alpha-2,8-sialyltransferase 1
1554641_a_at	1.83E-02	0.06844459	TET3	tet methylcytosine dioxygenase 3
230268_at	1.83E-02	0.13103411		
233691_at	1.83E-02	0.11797363		
209641_s_at	1.84E-02	0.32273627	ABCC3	ATP binding cassette subfamily C member 3
226205_at	1.84E-02	0.10522286	ANKRD13D	ankyrin repeat domain 13D
226309_at	1.84E-02	-0.16640807	DNAL1	dynein axonemal light chain 1
218567_x_at	1.84E-02	0.18410085	DPP3	dipeptidyl peptidase 3
243270_at	1.84E-02	0.06653903	FAM205A	family with sequence similarity 205 member A
215920_s_at	1.84E-02	0.14198543	NPIPA5///NPIPB6///NPIPB15///PDXDC2P///NPIPB3///NPIPA1	nuclear pore complex interacting protein family member A5///nuclear pore complex interacting protein family member B6///nuclear pore complex interacting protein family member B15///pyridoxal dependent decarboxylase domain containing 2, pseudogene///nuclear pore complex interacting protein family member B3///nuclear pore complex interacting protein family member A1
210170_at	1.84E-02	-0.06005817	PDLIM3	PDZ and LIM domain 3
213111_at	1.84E-02	-0.1948247	PIKFYVE	phosphoinositide kinase, FYVE-type zinc finger containing
233001_at	1.84E-02	0.08381738	SAMD10	sterile alpha motif domain containing 10
203010_at	1.84E-02	0.16087896	STAT5A	signal transducer and activator of transcription 5A
227936_at	1.84E-02	-0.28377702	TMEM68	transmembrane protein 68
226445_s_at	1.84E-02	0.17298216	TRIM41	tripartite motif containing 41
232835_at	1.84E-02	-0.19419309		
1568686_at	1.85E-02	0.05685794	ATP8B5P	ATPase phospholipid transporting 8B5, pseudogene
213318_s_at	1.85E-02	0.25419568	BAG6	BCL2 associated athanogene 6
219381_at	1.85E-02	-0.22935012	C5orf42	chromosome 5 open reading frame 42
1554242_a_at	1.85E-02	-0.4680305	COCH	cochlin
223671_x_at	1.85E-02	-0.2070416	DPH5	diphthamide biosynthesis 5
203524_s_at	1.85E-02	0.23522106	MPST	mercaptopyruvate sulfurtransferase
217954_s_at	1.85E-02	-0.18532521	PHF3	PHD finger protein 3
230311_s_at	1.85E-02	0.1069962	PRDM6	PR/SET domain 6
207754_at	1.85E-02	-0.0864239	RASSF8	Ras association domain family member 8
218777_at	1.85E-02	0.18398682	REEP4	receptor accessory protein 4
201748_s_at	1.85E-02	0.19395462	SAFB	scaffold attachment factor B
213417_at	1.85E-02	0.09791332	TBX2	T-box 2
217500_at	1.85E-02	0.07163459	TIAL1	TIA1 cytotoxic granule-associated RNA binding protein-like 1
1554295_x_at	1.85E-02	0.06768891	TTBK2	tau tubulin kinase 2
1554172_a_at	1.85E-02	0.14574066	ZMYM3	zinc finger MYM-type containing 3
232137_at	1.85E-02	0.09054169	ZNF616	zinc finger protein 616
1557522_x_at	1.85E-02	0.41318304		
237693_at	1.85E-02	-0.07485383		
235842_at	1.85E-02	0.12935873		
218075_at	1.86E-02	0.11609291	AAAS	aladin WD repeat nucleoporin
215015_at	1.86E-02	0.09962259	BICDL1	BICD family like cargo adaptor 1
220151_at	1.86E-02	0.10943659	C19orf73	chromosome 19 open reading frame 73
202175_at	1.86E-02	0.14917915	CHPF	chondroitin polymerizing factor
1560404_a_at	1.86E-02	-0.15305207	DPH6	diphthamine biosynthesis 6
1569349_at	1.86E-02	-0.31033622	EMSY	EMSY, BRCA2 interacting transcriptional repressor
227500_at	1.86E-02	0.20138889	FBXL18	F-box and leucine rich repeat protein 18
208414_s_at	1.86E-02	0.08317355	HOXB3	homeobox B3
227567_at	1.86E-02	-0.29663388	LINC00674	long intergenic non-protein coding RNA 674
240427_at	1.86E-02	-0.06211843	LOC101929050	uncharacterized LOC101929050
218112_at	1.86E-02	0.20728494	MRPS34	mitochondrial ribosomal protein S34
204436_at	1.86E-02	0.16010997	PLEKHO2	pleckstrin homology domain containing O2
235349_at	1.86E-02	-0.42125965	RMDN2	regulator of microtubule dynamics 2
219809_at	1.86E-02	0.1658511	WDR55	WD repeat domain 55
215939_at	1.86E-02	-0.07123155		

237136_at	1.86E-02	0.15016931		
209080_x_at	1.87E-02	0.22613323	GLRX3	glutaredoxin 3
209192_x_at	1.87E-02	0.16177563	KAT5	lysine acetyltransferase 5
225304_s_at	1.87E-02	0.33762707	NDUFA11	NADH:ubiquinone oxidoreductase subunit A11
232992_at	1.87E-02	-0.06965269	SAYSD1	SAYSVFN motif domain containing 1
242522_at	1.87E-02	-0.07808616	TCF24	transcription factor 24
228330_at	1.87E-02	-0.22922776	ZUFSP	zinc finger with UFM1 specific peptidase domain
241526_at	1.87E-02	-0.10582322		
240061_at	1.87E-02	-0.29287208		
205875_s_at	1.88E-02	0.18235894	ATRIP///TREX1	ATR interacting protein///three prime repair exonuclease 1
225821_s_at	1.88E-02	-0.19152793	BOD1L1	biorientation of chromosomes in cell division 1 like 1
205902_at	1.88E-02	-0.0851177	KCNN3	potassium calcium-activated channel subfamily N member 3
215153_at	1.88E-02	0.08523497	NOS1AP	nitric oxide synthase 1 adaptor protein
221327_s_at	1.88E-02	0.06619804	OPN1MW3///OPN1MW2///OPN1LW///OPN1MW	opsin 1 (cone pigments), medium-wave-sensitive 3///opsin 1 (cone pigments), medium-wave-sensitive 2///opsin 1 (cone pigments), long-wave-sensitive///opsin 1 (cone pigments), medium-wave-sensitive
227289_at	1.88E-02	-0.34919333	PCDH17	protocadherin 17
207733_x_at	1.88E-02	0.09078812	PSG9	pregnancy specific beta-1-glycoprotein 9
1569775_at	1.88E-02	-0.11438695	RNF157	ring finger protein 157
1564423_a_at	1.88E-02	-0.11803463	SEC16B	SEC16 homolog B, endoplasmic reticulum export factor
38918_at	1.88E-02	0.18841709	SOX13	SRY-box 13
239870_at	1.88E-02	0.06975775	SPATS1	spermatogenesis associated serine rich 1
216454_at	1.88E-02	0.07012834	TRMT1	tRNA methyltransferase 1
242962_at	1.88E-02	-0.0835801		
1562745_at	1.88E-02	0.1087441		
235404_at	1.89E-02	0.10022187	ARID5B	AT-rich interaction domain 5B
1559439_s_at	1.89E-02	0.0674649	C21orf58	chromosome 21 open reading frame 58
35150_at	1.89E-02	0.07366075	CD40	CD40 molecule
201904_s_at	1.89E-02	0.23846286	CTDSPL	CTD small phosphatase like
213793_s_at	1.89E-02	-0.36867939	HOMER1	homer scaffolding protein 1
205266_at	1.89E-02	0.27664347	LIF	leukemia inhibitory factor
214156_at	1.89E-02	-0.7443271	MYRIP	myosin VIIA and Rab interacting protein
208455_at	1.89E-02	0.0687002	NECTIN1	nectin cell adhesion molecule 1
204854_at	1.89E-02	0.13849669	P3H3	prolyl 3-hydroxylase 3
202958_at	1.89E-02	0.16754152	PTPN9	protein tyrosine phosphatase, non-receptor type 9
200637_s_at	1.89E-02	0.25383845	PTPRF	protein tyrosine phosphatase, receptor type F
200908_s_at	1.89E-02	-0.22105367	RPLP2	ribosomal protein lateral stalk subunit P2
237741_at	1.89E-02	-0.3772734	SLC25A36	solute carrier family 25 member 36
229032_at	1.89E-02	-0.10071202	WSCD2	WSC domain containing 2
1555562_a_at	1.89E-02	0.14112339	ZCCHC7	zinc finger CCHC-type containing 7
1553096_s_at	1.90E-02	0.14285423	BCL2L11	BCL2 like 11
213949_s_at	1.90E-02	0.07488376	DOHH	deoxyhypusine hydroxylase/monooxygenase
224194_at	1.90E-02	-0.07096045	FCRL2	Fc receptor like 2
242987_x_at	1.90E-02	0.09195435	LAMA5-AS1	LAMA5 antisense RNA 1
1561559_at	1.90E-02	0.07855088	LOC101927948	uncharacterized LOC101927948
224621_at	1.90E-02	-0.16578543	MAPK1	mitogen-activated protein kinase 1
221779_at	1.90E-02	0.17800712	MICALL1	MICAL like 1
205076_s_at	1.90E-02	0.38706292	MTMR11	myotubularin related protein 11
213264_at	1.90E-02	-0.35766725	PCBP2	poly(rC) binding protein 2
205878_at	1.90E-02	-0.12601172	POU6F1	POU class 6 homeobox 1
1570061_at	1.90E-02	0.09332935	RAB7A	RAB7A, member RAS oncogene family
212707_s_at	1.90E-02	0.17500863	RASA4B///RASA4CP///RASA4	RAS p21 protein activator 4B///RAS p21 protein activator 4C, pseudogene///RAS p21 protein activator 4
206729_at	1.90E-02	0.0793171	TNFRSF8	TNF receptor superfamily member 8
225529_at	1.91E-02	0.24514736	ACAP3	ArfGAP with coiled-coil, ankyrin repeat and PH domains 3
235076_at	1.91E-02	-0.24716625	CALCOCO2	calcium binding and coiled-coil domain 2
204510_at	1.91E-02	-0.27592167	CDC7	cell division cycle 7
208817_at	1.91E-02	0.30952534	COMT	catechol-O-methyltransferase

207715_at	1.91E-02	-0.09149472	CRYGB	crystallin gamma B
1552677_a_at	1.91E-02	-0.07434709	DIP2A	disco interacting protein 2 homolog A
206065_s_at	1.91E-02	0.06497287	DPYS	dihydropyrimidinase
229972_at	1.91E-02	0.08633058	LOC101926963	uncharacterized LOC101926963
202374_s_at	1.91E-02	0.15011978	RAB3GAP2///AURKAPS1	RAB3 GTPase activating non-catalytic protein subunit 2///aurora kinase A pseudogene 1
229843_at	1.91E-02	-0.33792415	RMDN1	regulator of microtubule dynamics 1
222528_s_at	1.91E-02	0.28641121	SLC25A37	solute carrier family 25 member 37
225912_at	1.91E-02	-0.35986632	TP53INP1	tumor protein p53 inducible nuclear protein 1
224871_at	1.91E-02	0.20350196	TPRG1L	tumor protein p63 regulated 1-like
232642_at	1.91E-02	0.10349887	VWA5B2	von Willebrand factor A domain containing 5B2
226986_at	1.91E-02	0.25191831	WIPI2	WD repeat domain, phosphoinositide interacting 2
213196_at	1.91E-02	0.17077851	ZNF629	zinc finger protein 629
232947_at	1.91E-02	-0.12275789		
214808_at	1.91E-02	0.28852334		
241705_at	1.92E-02	-0.22785267	ABCA5	ATP binding cassette subfamily A member 5
229030_at	1.92E-02	0.53414498	CAPN8	calpain 8
224312_x_at	1.92E-02	0.18900271	CPSF3L	cleavage and polyadenylation specific factor 3-like
200648_s_at	1.92E-02	0.42856521	GLUL	glutamate-ammonia ligase
203828_s_at	1.92E-02	0.30946801	IL32	interleukin 32
236043_at	1.92E-02	-0.13240382	LOC100130175///BBIP1	uncharacterized LOC100130175///BBSome interacting protein 1
218710_at	1.92E-02	-0.19953485	TTC27	tetratricopeptide repeat domain 27
227441_s_at	1.93E-02	0.05246043	ANKS1B	ankyrin repeat and sterile alpha motif domain containing 1B
1552286_at	1.93E-02	-0.27259875	ATP6V1E2	ATPase H+ transporting V1 subunit E2
241733_at	1.93E-02	-0.26181309	C18orf54	chromosome 18 open reading frame 54
228865_at	1.93E-02	0.31737146	C1orf116	chromosome 1 open reading frame 116
231441_at	1.93E-02	0.08488456	C7orf62	chromosome 7 open reading frame 62
1564757_a_at	1.93E-02	0.12003092	CCDC148	coiled-coil domain containing 148
231198_at	1.93E-02	0.09461503	CDK6	cyclin dependent kinase 6
1560932_at	1.93E-02	0.05517546	FLJ31356	uncharacterized protein FLJ31356
228410_at	1.93E-02	-0.19021386	GAB3	GRB2 associated binding protein 3
209675_s_at	1.93E-02	0.12876102	HNRNPUL1	heterogeneous nuclear ribonucleoprotein U like 1
1568914_at	1.93E-02	-0.08840977	OPN5	opsin 5
204297_at	1.93E-02	-0.2019929	PIK3C3	phosphatidylinositol 3-kinase catalytic subunit type 3
210706_s_at	1.93E-02	0.1937201	RNF24	ring finger protein 24
222893_s_at	1.93E-02	-0.16790982	RPAP2	RNA polymerase II associated protein 2
233988_x_at	1.93E-02	-0.06837978	SCUBE1	signal peptide, CUB domain and EGF like domain containing 1
243818_at	1.93E-02	0.06263924	SFTA1P	surfactant associated 1, pseudogene
231154_x_at	1.93E-02	-0.53499446	TINAG	tubulointerstitial nephritis antigen
224912_at	1.93E-02	0.13658022	TTC7A	tetratricopeptide repeat domain 7A
227207_x_at	1.93E-02	0.14394516	ZNF213	zinc finger protein 213
240350_at	1.93E-02	0.06826434		
240657_at	1.93E-02	0.07378355		
1561574_at	1.93E-02	-0.09203078		
1557224_at	1.93E-02	-0.07365578		
1569759_at	1.93E-02	-0.05750958		
211004_s_at	1.94E-02	0.10866472	ALDH3B1	aldehyde dehydrogenase 3 family member B1
237827_at	1.94E-02	0.08603848	FAM205C	family with sequence similarity 205 member C
232515_at	1.94E-02	-0.16557254	GPR75-ASB3///ASB3///GPR75	GPR75-ASB3 readthrough///ankyrin repeat and SOCS box containing 3///G protein-coupled receptor 75
225571_at	1.94E-02	-0.31385221	LIFR	leukemia inhibitory factor receptor alpha
232070_at	1.94E-02	-0.07770742	LOC100506639	uncharacterized LOC100506639
1569594_a_at	1.94E-02	-0.2114349	NEMF	nuclear export mediator factor
238326_at	1.94E-02	-0.1589653	ODF3B	outer dense fiber of sperm tails 3B
223148_at	1.94E-02	0.16658118	PIGS	phosphatidylinositol glycan anchor biosynthesis class S
201602_s_at	1.94E-02	-0.29157299	PPP1R12A	protein phosphatase 1 regulatory subunit 12A
238039_at	1.94E-02	-0.22603056	SCAMP1-AS1	SCAMP1 antisense RNA 1
231362_at	1.94E-02	0.09560955		
211727_s_at	1.95E-02	-0.24302539	COX11	COX11, cytochrome c oxidase copper chaperone
237323_at	1.95E-02	0.25251848	HKDC1	hexokinase domain containing 1

219188_s_at	1.95E-02	0.43659417	MACROD1	MACRO domain containing 1
219317_at	1.95E-02	-0.30840201	POLI	DNA polymerase iota
201851_at	1.95E-02	0.13977392	SH3GL1	SH3 domain containing GRB2 like 1, endophilin A2
214566_at	1.95E-02	0.06433985	SMR3A	submaxillary gland androgen regulated protein 3A
34260_at	1.95E-02	0.17824727	TELO2	telomere maintenance 2
224087_at	1.95E-02	-0.1025955		
224346_at	1.95E-02	-0.1817139		
242900_at	1.96E-02	-0.29652538	ALG10B///ALG10	ALG10B, alpha-1,2-glucosyltransferase///ALG10, alpha-1,2-glucosyltransferase
214993_at	1.96E-02	0.18470561	ASPHD1	aspartate beta-hydroxylase domain containing 1
244542_at	1.96E-02	-0.07759443	BCDIN3D-AS1	BCDIN3D antisense RNA 1
221799_at	1.96E-02	0.15731671	CHPF2	chondroitin polymerizing factor 2
239760_at	1.96E-02	-0.18178542	COX11	COX11, cytochrome c oxidase copper chaperone
232806_s_at	1.96E-02	0.10986319	FAM131A	family with sequence similarity 131 member A
215337_at	1.96E-02	0.08081123	MED24	mediator complex subunit 24
219489_s_at	1.96E-02	0.29688992	NXN	nucleoredoxin
203317_at	1.96E-02	0.19750442	PSD4	pleckstrin and Sec7 domain containing 4
1555233_at	1.96E-02	0.09337905	RHOJ	ras homolog family member J
237280_at	1.96E-02	0.08909435	TCTE1	t-complex-associated-testis-expressed 1
214958_s_at	1.96E-02	0.17378006	TMC6	transmembrane channel like 6
224718_at	1.96E-02	0.21825482	YY1	YY1 transcription factor
244794_at	1.96E-02	-0.127607		
207583_at	1.97E-02	-0.04655073	ABCD2	ATP binding cassette subfamily D member 2
41047_at	1.97E-02	0.23484524	C9orf16	chromosome 9 open reading frame 16
220307_at	1.97E-02	-0.10979771	CD244	CD244 molecule
47773_at	1.97E-02	-0.15475937	FBXO42	F-box protein 42
1556026_at	1.97E-02	-0.19511943	LINC00893	long intergenic non-protein coding RNA 893
231739_at	1.97E-02	0.06982779	LOC100507547///PRRT1	uncharacterized LOC100507547///proline rich transmembrane protein 1
1555831_s_at	1.97E-02	0.25813123	LRRC41	leucine rich repeat containing 41
40569_at	1.97E-02	0.18169074	MZF1	myeloid zinc finger 1
228388_at	1.97E-02	0.11911552	NFKBIB	NFKB inhibitor beta
217033_x_at	1.97E-02	-0.10053215	NTRK3	neurotrophic receptor tyrosine kinase 3
235684_s_at	1.97E-02	-0.19144718	SESN3	sestrin 3
205807_s_at	1.97E-02	0.25403025	TUFT1	tuftelin 1
243185_at	1.97E-02	-0.08032002		
219072_at	1.98E-02	0.16271309	BCL7C	BCL tumor suppressor 7C
224663_s_at	1.98E-02	-0.3002191	CFL2	cofilin 2
211255_x_at	1.98E-02	0.13420536	DEDD	death effector domain containing
204102_s_at	1.98E-02	0.24465323	EEF2	eukaryotic translation elongation factor 2
208417_at	1.98E-02	0.08296759	FGF6	fibroblast growth factor 6
221837_at	1.98E-02	0.09555358	KLHL22	kelch like family member 22
225367_at	1.98E-02	-0.22078694	PGM2	phosphoglucomutase 2
58308_at	1.98E-02	-0.13891518	TRIM62	tripartite motif containing 62
233970_s_at	1.98E-02	0.28845281	TRMT6	tRNA methyltransferase 6
1562723_at	1.98E-02	0.08281053		
235435_at	1.99E-02	-0.22480736	AASDH	aminoadipate-semialdehyde dehydrogenase
218936_s_at	1.99E-02	-0.20000871	CCDC59	coiled-coil domain containing 59
201402_at	1.99E-02	0.0773983	GRK2	G protein-coupled receptor kinase 2
231929_at	1.99E-02	-0.43132641	IKZF2	IKAROS family zinc finger 2
204024_at	1.99E-02	-0.25532068	OSGIN2	oxidative stress induced growth inhibitor family member 2
211570_s_at	1.99E-02	0.08125414	RAPSN	receptor associated protein of the synapse
212783_at	1.99E-02	-0.17986547	RBBP6	RB binding protein 6, ubiquitin ligase
215450_at	1.99E-02	-0.25281669	SNRPE	small nuclear ribonucleoprotein polypeptide E
1554077_a_at	1.99E-02	0.28528133	TMEM53	transmembrane protein 53
226505_x_at	1.99E-02	-0.22238248	USP32	ubiquitin specific peptidase 32
211702_s_at	1.99E-02	-0.17077841	USP32	ubiquitin specific peptidase 32
202546_at	1.99E-02	0.19138057	VAMP8	vesicle associated membrane protein 8
236692_at	1.99E-02	-0.18887982		
1565811_at	1.99E-02	-0.23694745		
225711_at	2.00E-02	-0.25342594	ARL6IP6	ADP ribosylation factor like GTPase 6 interacting protein 6

236900_x_at	2.00E-02	-0.11564824	CCDC163	coiled-coil domain containing 163
232505_at	2.00E-02	0.07590327	DCTN2	dynactin subunit 2
240928_at	2.00E-02	-0.14098445	FCF1	FCF1 rRNA-processing protein
229857_s_at	2.00E-02	-0.06719683	KANSL1-AS1	KANSL1 antisense RNA 1
215947_s_at	2.00E-02	-0.15700651	LOC100287852///FAM136A	protein FAM136A pseudogene///family with sequence similarity 136 member A
1561368_at	2.00E-02	-0.06336878	LOC105376081	uncharacterized LOC105376081
211752_s_at	2.00E-02	0.30684905	NDUFS7	NADH:ubiquinone oxidoreductase core subunit S7
201929_s_at	2.00E-02	0.30510115	PKP4	plakophilin 4
225671_at	2.00E-02	0.32478526	SPNS2	sphingolipid transporter 2
242909_at	2.00E-02	-0.61373113		
232653_at	2.00E-02	-0.17247822		
231321_s_at	2.01E-02	-0.15453722	ACER3	alkaline ceramidase 3
218673_s_at	2.01E-02	0.1639405	ATG7	autophagy related 7
1568814_at	2.01E-02	0.08266289	DDX50	DEAD-box helicase 50
227859_at	2.01E-02	-0.17867537	DNAJC27	DnaJ heat shock protein family (Hsp40) member C27
209473_at	2.01E-02	-0.22441219	ENTPD1	ectonucleoside triphosphate diphosphohydrolase 1
200681_at	2.01E-02	-0.20442203	GLO1	glyoxalase I
1569833_at	2.01E-02	0.07582933	LINC01585	long intergenic non-protein coding RNA 1585
203652_at	2.01E-02	0.13986917	MAP3K11	mitogen-activated protein kinase kinase kinase 11
231829_at	2.01E-02	0.16696123	MAVS	mitochondrial antiviral signaling protein
236951_at	2.01E-02	0.27580374	NSFL1C	NSFL1 cofactor
207923_x_at	2.01E-02	0.07315045	PAX8	paired box 8
225881_at	2.01E-02	-0.23497807	SLC35B4	solute carrier family 35 member B4
1566208_at	2.01E-02	-0.06479348	TCEA1	transcription elongation factor A1
223656_s_at	2.01E-02	0.16263934	TMEM234	transmembrane protein 234
203122_at	2.01E-02	0.18469876	TRAPPC12	trafficking protein particle complex 12
237153_at	2.01E-02	0.07855892		
223184_s_at	2.02E-02	0.18510326	AGPAT3	1-acylglycerol-3-phosphate O-acyltransferase 3
207522_s_at	2.02E-02	0.36674522	ATP2A3	ATPase sarcoplasmic/endoplasmic reticulum Ca2+ transporting 3
1553805_at	2.02E-02	0.06056137	C3orf49	chromosome 3 open reading frame 49
206755_at	2.02E-02	0.51278059	CYP2B6	cytochrome P450 family 2 subfamily B member 6
1570156_s_at	2.02E-02	-0.158559	FMN1	formin 1
221334_s_at	2.02E-02	0.08027565	FOXP3	forkhead box P3
222240_s_at	2.02E-02	0.31138483	ISYNA1	inositol-3-phosphate synthase 1
202267_at	2.02E-02	0.33831258	LAMC2	laminin subunit gamma 2
225438_at	2.02E-02	-0.35804582	NUDCD1	NudC domain containing 1
205078_at	2.02E-02	-0.19913558	PIGF	phosphatidylinositol glycan anchor biosynthesis class F
213726_x_at	2.02E-02	0.2228872	TUBB4B	tubulin beta 4B class IVb
1560237_at	2.02E-02	-0.05272707		
236221_at	2.03E-02	0.08453458	AP4B1	adaptor related protein complex 4 beta 1 subunit
1554905_x_at	2.03E-02	0.11683665	FRMD8	FERM domain containing 8
244701_at	2.03E-02	-0.08181337	LINC00595	long intergenic non-protein coding RNA 595
234419_x_at	2.03E-02	-0.09262362	LOC100293211///IGHV4-31///IGHM///IGHG3///IGHG1///IGHA1	uncharacterized LOC100293211///immunoglobulin heavy variable 4-31///immunoglobulin heavy constant mu///immunoglobulin heavy constant gamma 3 (G3m marker)///immunoglobulin heavy constant gamma 1 (G1m marker)///immunoglobulin heavy constant alpha 1
231788_at	2.03E-02	0.14473355	LPAR5	lysophosphatidic acid receptor 5
202034_x_at	2.03E-02	-0.28789537	RB1CC1	RB1 inducible coiled-coil 1
201516_at	2.03E-02	0.26948138	SRM	spermidine synthase
202171_at	2.03E-02	-0.18651681	VEZF1	vascular endothelial zinc finger 1
242740_at	2.03E-02	-0.09753536		
225697_at	2.04E-02	-0.24582998	CDK12	cyclin dependent kinase 12
204224_s_at	2.04E-02	-0.2319896	GCH1	GTP cyclohydrolase 1
244251_at	2.04E-02	-0.11370756	LCP2	lymphocyte cytosolic protein 2
223206_s_at	2.04E-02	0.28184215	NMRAL1	NmrA like redox sensor 1
212750_at	2.04E-02	-0.21136577	PPP1R16B	protein phosphatase 1 regulatory subunit 16B
214487_s_at	2.04E-02	0.15649177	RAP2B///RAP2A	RAP2B, member of RAS oncogene family///RAP2A, member of RAS oncogene family
222982_x_at	2.04E-02	-0.15996636	SLC38A2	solute carrier family 38 member 2

200891_s_at	2.04E-02	-0.15813456	SSR1	signal sequence receptor subunit 1
217742_s_at	2.04E-02	0.16154393	WAC	WW domain containing adaptor with coiled-coil
242339_at	2.04E-02	0.06905989		
227237_x_at	2.05E-02	0.17148451	ATAD3B	ATPase family, AAA domain containing 3B
1564315_at	2.05E-02	-0.05634627	C8orf49	chromosome 8 open reading frame 49
223020_at	2.05E-02	0.27708644	CLPTM1L	CLPTM1 like
218506_x_at	2.05E-02	0.14747425	GLYR1	glyoxylate reductase 1 homolog
1562733_at	2.05E-02	0.08298958	LINC00092	long intergenic non-protein coding RNA 92
226444_at	2.05E-02	-0.39070979	LOC107985971	uncharacterized LOC107985971
224078_at	2.05E-02	-0.18429628	MFSD14C	major facilitator superfamily domain containing 14C
234850_at	2.05E-02	0.25741591	MOGAT3	monoacylglycerol O-acyltransferase 3
207576_x_at	2.05E-02	0.10435286	OXT	oxytocin/neurophysin I prepropeptide
241947_at	2.05E-02	-0.06078048	PCED1B-AS1	PCED1B antisense RNA 1
200827_at	2.05E-02	0.20041224	PLOD1	procollagen-lysine,2-oxoglutarate 5-dioxygenase 1
225033_at	2.05E-02	0.35687388	ST3GAL1	ST3 beta-galactoside alpha-2,3-sialyltransferase 1
1569076_a_at	2.05E-02	0.08278616	ZNF836	zinc finger protein 836
1557286_at	2.05E-02	0.12167808		
216198_at	2.05E-02	-0.09796447		
210080_x_at	2.06E-02	0.10067362	CELA3A	chymotrypsin like elastase family member 3A
239261_s_at	2.06E-02	0.12457166	CORIN	corin, serine peptidase
216759_at	2.06E-02	-0.07369744	HRASLS2	HRAS like suppressor 2
1561530_at	2.06E-02	-0.10360327	LOC101927164	uncharacterized LOC101927164
239241_at	2.06E-02	-0.12461462	LOC101928424	uncharacterized LOC101928424
232148_at	2.06E-02	-0.24894974	NSMAF	neutral sphingomyelinase activation associated factor
225505_s_at	2.06E-02	0.29715624	PCED1A	PC-esterase domain containing 1A
212753_at	2.06E-02	-0.2034358	PCGF3	polycomb group ring finger 3
227212_s_at	2.06E-02	0.26287569	PHF19	PHD finger protein 19
223841_s_at	2.06E-02	-0.05172086	SPATA9	spermatogenesis associated 9
219958_at	2.06E-02	0.32708516	TMEM74B	transmembrane protein 74B
223537_s_at	2.06E-02	0.0751021	WNT5B	Wnt family member 5B
228041_at	2.07E-02	-0.20577072	AASDH	aminoadipate-semialdehyde dehydrogenase
225061_at	2.07E-02	0.30852551	DNAJA4	DnaJ heat shock protein family (Hsp40) member A4
208926_at	2.07E-02	0.36706716	NEU1	neuraminidase 1 (lysosomal sialidase)
205596_s_at	2.07E-02	-0.2499444	SMURF2	SMAD specific E3 ubiquitin protein ligase 2
221700_s_at	2.07E-02	0.14898761	UBA52	ubiquitin A-52 residue ribosomal protein fusion product 1
204727_at	2.07E-02	-0.25412372	WDHD1	WD repeat and HMG-box DNA binding protein 1
1557132_at	2.07E-02	-0.2267742	WDR17	WD repeat domain 17
230786_at	2.07E-02	-0.19764753	ZCCHC8	zinc finger CCHC-type containing 8
220696_at	2.07E-02	-0.13574091		
219833_s_at	2.08E-02	-0.32706518	EFHC1	EF-hand domain containing 1
212170_at	2.08E-02	-0.22431627	RBM12	RNA binding motif protein 12
1566171_at	2.08E-02	0.08719585	RFFL	ring finger and FYVE-like domain containing E3 ubiquitin protein ligase
206321_at	2.08E-02	0.07664558	RFX1	regulatory factor X1
225779_at	2.08E-02	0.22995449	SLC27A4	solute carrier family 27 member 4
239712_at	2.09E-02	-0.08620402	CCDC171	coiled-coil domain containing 171
237097_at	2.09E-02	0.16547504	EHD4	EH domain containing 4
228638_at	2.09E-02	-0.31901587	FAM76A	family with sequence similarity 76 member A
202275_at	2.09E-02	0.20940118	G6PD	glucose-6-phosphate dehydrogenase
202923_s_at	2.09E-02	-0.19658305	GCLC	glutamate-cysteine ligase catalytic subunit
242389_at	2.09E-02	-0.28325617	LUC7L3	LUC7 like 3 pre-mRNA splicing factor
214694_at	2.09E-02	0.10161349	MPRIP	myosin phosphatase Rho interacting protein
201827_at	2.09E-02	0.22447739	SMARCD2	SWI/SNF related, matrix associated, actin dependent regulator of chromatin, subfamily d, member 2
203965_at	2.09E-02	0.16647386	USP20	ubiquitin specific peptidase 20
226650_at	2.09E-02	0.37305526	ZFAND2A	zinc finger AN1-type containing 2A
220136_s_at	2.10E-02	0.15058879	CRYBA2	crystallin beta A2
214472_at	2.10E-02	0.27106964	HIST1H3F///HIST1H3B///HIST1H3H///HIST1H3J///HIST1H3G///HIST1H3I///HIST1H3E///HIST1H3C///HIST1H3D///HIST1H3A///HIST1H2AD	histone cluster 1, H3f///histone cluster 1, H3b///histone cluster 1, H3h///histone cluster 1, H3j///histone cluster 1, H3g///histone cluster 1, H3i///histone cluster 1, H3e///histone cluster 1, H3c///histone cluster 1, H3d///histone cluster 1, H3a///histone cluster 1, H2ad

203086_at	2.10E-02	-0.22403637	KIF2A	kinesin family member 2A
217906_at	2.10E-02	-0.21416824	KLHDC2	kelch domain containing 2
1569760_at	2.10E-02	0.05926716	LRRC37A7P	leucine rich repeat containing 37 member A7, pseudogene
224448_s_at	2.10E-02	-0.26305328	MIR3934///UQCC2	microRNA 3934///ubiquinol-cytochrome c reductase complex assembly factor 2
235934_at	2.10E-02	0.07036258	NAPA-AS1	NAPA antisense RNA 1
229233_at	2.10E-02	-0.14376085	NRG3	neuregulin 3
223360_at	2.10E-02	0.27519893	SPATC1L	spermatogenesis and centriole associated 1-like
1552541_at	2.10E-02	0.1063714	TAGAP	T-cell activation RhoGTPase activating protein
211300_s_at	2.10E-02	0.13135514	TP53	tumor protein p53
231689_at	2.10E-02	-0.10773597	TRPM7	transient receptor potential cation channel subfamily M member 7
AFFX-BioB-M_at	2.10E-02	-0.15341401		
231584_s_at	2.11E-02	0.09246558	BCAS4	breast carcinoma amplified sequence 4
205538_at	2.11E-02	0.22332908	CORO2A	coronin 2A
224735_at	2.11E-02	0.15907302	CYB561A3	cytochrome b561 family member A3
208147_s_at	2.11E-02	0.16928524	CYP2C8	cytochrome P450 family 2 subfamily C member 8
223682_s_at	2.11E-02	0.17489706	EIF1AD	eukaryotic translation initiation factor 1A domain containing
230877_at	2.11E-02	0.08462247	IGHD	immunoglobulin heavy constant delta
221842_s_at	2.11E-02	-0.16654134	LOC100506639///ZNF131	uncharacterized LOC100506639///zinc finger protein 131
236295_s_at	2.11E-02	-0.23314117	NLRC3	NLR family CARD domain containing 3
223369_at	2.11E-02	0.22388427	NTMT1	N-terminal Xaa-Pro-Lys N-methyltransferase 1
208733_at	2.11E-02	-0.42575128	RAB2A	RAB2A, member RAS oncogene family
1555339_at	2.11E-02	0.07179829	RAP1A	RAP1A, member of RAS oncogene family
219143_s_at	2.11E-02	0.25183526	RPP25	ribonuclease P/MRP subunit p25
218268_at	2.11E-02	-0.18013666	TBC1D15	TBC1 domain family member 15
224849_at	2.11E-02	0.19296975	TTC17	tetratricopeptide repeat domain 17
1555982_at	2.11E-02	-0.30083441	ZFYVE16	zinc finger FYVE-type containing 16
240345_x_at	2.11E-02	0.07982452		
217748_at	2.12E-02	0.18945269	ADIPOR1	adiponectin receptor 1
223566_s_at	2.12E-02	0.28610651	BCOR	BCL6 corepressor
207776_s_at	2.12E-02	-0.1194063	CACNB2	calcium voltage-gated channel auxiliary subunit beta 2
228585_at	2.12E-02	-0.2250953	ENTPD1	ectonucleoside triphosphate diphosphohydrolase 1
1557871_at	2.12E-02	0.04932981	LOC253573	uncharacterized LOC253573
244675_at	2.12E-02	0.05729921	RGS8	regulator of G-protein signaling 8
238673_at	2.12E-02	-0.42593522	SAMD12	sterile alpha motif domain containing 12
217160_at	2.12E-02	-0.07483028	TSPY1	testis specific protein, Y-linked 1
224043_s_at	2.12E-02	0.0632583	UPB1	beta-ureidopropionase 1
226124_at	2.12E-02	-0.20456967	ZFP90	ZFP90 zinc finger protein
239371_at	2.12E-02	-0.07155305		
236007_at	2.13E-02	-0.31593276	AKAP10	A-kinase anchoring protein 10
204882_at	2.13E-02	-0.18998738	ARHGAP25	Rho GTPase activating protein 25
1554960_at	2.13E-02	-0.39727088	CCDC190	coiled-coil domain containing 190
1568636_a_at	2.13E-02	0.06396655	LINC00354	long intergenic non-protein coding RNA 354
233522_at	2.13E-02	-0.06657863	MEF2C-AS1	MEF2C antisense RNA 1
201364_s_at	2.13E-02	0.184886	OAZ2	ornithine decarboxylase antizyme 2
216609_at	2.13E-02	-0.19330405	TXN	thioredoxin
1561912_at	2.13E-02	0.04560032		
1565597_at	2.13E-02	-0.16452859		
224461_s_at	2.14E-02	0.26756293	AIFM2	apoptosis inducing factor, mitochondria associated 2
217749_at	2.14E-02	0.21190666	COPG1	coatomer protein complex subunit gamma 1
1558523_at	2.14E-02	-0.27647764	FAM184A	family with sequence similarity 184 member A
200959_at	2.14E-02	0.2372733	FUS	FUS RNA binding protein
223564_s_at	2.14E-02	0.16779666	GNB1L	G protein subunit beta 1 like
202409_at	2.14E-02	1.13186404	INS-IGF2///IGF2	INS-IGF2 readthrough///insulin like growth factor 2
229927_at	2.14E-02	0.60647488	LEMD1	LEM domain containing 1
231419_at	2.14E-02	0.13455976	LOC100505555///ZNF846	uncharacterized LOC100505555///zinc finger protein 846
202632_at	2.14E-02	0.18428887	OVCA2///DPH1	ovarian tumor suppressor candidate 2///diphthamide biosynthesis 1

200074_s_at	2.14E-02	0.21409124	RPL14	ribosomal protein L14
201225_s_at	2.14E-02	-0.17744205	SRRM1	serine and arginine repetitive matrix 1
1560017_at	2.14E-02	-0.17042663	TMTC3	transmembrane and tetratricopeptide repeat containing 3
1558088_a_at	2.14E-02	0.18820763	UBE2I	ubiquitin conjugating enzyme E2 I
228009_x_at	2.14E-02	0.25513553	ZNRD1	zinc ribbon domain containing 1
1562056_at	2.14E-02	0.19681521		
217449_at	2.14E-02	-0.08432769		
214220_s_at	2.15E-02	-0.21758163	ALMS1	ALMS1, centrosome and basal body associated protein
1557544_at	2.15E-02	0.07047675	CFAP58	cilia and flagella associated protein 58
224462_s_at	2.15E-02	0.21526632	CHCHD6	coiled-coil-helix-coiled-coil-helix domain containing 6
235545_at	2.15E-02	-0.34104908	DEPDC1	DEP domain containing 1
229391_s_at	2.15E-02	-0.43447715	FAM26F	family with sequence similarity 26 member F
235574_at	2.15E-02	-0.22561231	GBP4	guanylate binding protein 4
230796_at	2.15E-02	-0.11008401	LINC01191	long intergenic non-protein coding RNA 1191
226643_s_at	2.15E-02	-0.18653716	NUDCD2	NudC domain containing 2
209587_at	2.15E-02	0.2580103	PITX1	paired like homeodomain 1
222977_at	2.15E-02	0.19496964	SURF4	surfeit 4
213210_at	2.15E-02	0.1153562	TAF6L	TATA-box binding protein associated factor 6 like
225003_at	2.15E-02	0.25110342	TMEM205	transmembrane protein 205
225350_s_at	2.15E-02	-0.17632404	ZYG11B	zyg-11 family member B, cell cycle regulator
205584_at	2.16E-02	-0.31373055	ALG13	ALG13, UDP-N-acetylglucosaminyltransferase subunit
1554875_at	2.16E-02	-0.18730732	CAMKMT	calmodulin-lysine N-methyltransferase
209399_at	2.16E-02	0.12370384	HLCS	holocarboxylase synthetase
220294_at	2.16E-02	-0.35293045	KCNV1	potassium voltage-gated channel modifier subfamily V member 1
241792_x_at	2.16E-02	-0.22136806	LUC7L3	LUC7 like 3 pre-mRNA splicing factor
235091_at	2.16E-02	-0.07091847	PDE12	phosphodiesterase 12
206687_s_at	2.16E-02	0.18289068	PTPN6	protein tyrosine phosphatase, non-receptor type 6
1570241_at	2.16E-02	0.12342102	SPATA21	spermatogenesis associated 21
238045_at	2.16E-02	-0.19692549	TMEM65	transmembrane protein 65
222651_s_at	2.16E-02	-0.27951511	TRPS1	transcriptional repressor GATA binding 1
214964_at	2.16E-02	0.20937336		
231460_at	2.16E-02	-0.08791186		
1561127_at	2.17E-02	0.09672344	ADARB2-AS1	ADARB2 antisense RNA 1
208677_s_at	2.17E-02	0.29673384	BSG	basigin (Ok blood group)
213875_x_at	2.17E-02	-0.23732663	C6orf62	chromosome 6 open reading frame 62
225545_at	2.17E-02	0.22589641	LOC101930123///EEF2K	eukaryotic elongation factor 2 kinase///eukaryotic elongation factor 2 kinase
227747_at	2.17E-02	0.26807607	MPZL3	myelin protein zero like 3
208819_at	2.17E-02	0.15330668	RAB8A	RAB8A, member RAS oncogene family
218790_s_at	2.17E-02	-0.19341269	TMLHE	trimethyllysine hydroxylase, epsilon
212760_at	2.17E-02	-0.19781185	UBR2	ubiquitin protein ligase E3 component n-recognin 2
236236_at	2.17E-02	0.07097968	WNK3	WNK lysine deficient protein kinase 3
213542_at	2.17E-02	0.11572216	ZNF710	zinc finger protein 710
227122_at	2.17E-02	0.18843158	ZNF791	zinc finger protein 791
234038_at	2.17E-02	-0.04801193		
236942_at	2.18E-02	0.0624741	CCDC182	coiled-coil domain containing 182
203391_at	2.18E-02	0.18361842	FKBP2	FK506 binding protein 2
239522_at	2.18E-02	-0.10895202	IL12RB1	interleukin 12 receptor subunit beta 1
233557_s_at	2.18E-02	0.17992405	MON1B	MON1 homolog B, secretory trafficking associated
233829_at	2.18E-02	0.12614526	TLDC2	TBC/LysM-associated domain containing 2
231816_s_at	2.18E-02	0.10037549	UBE2Q1	ubiquitin conjugating enzyme E2 Q1
204329_s_at	2.18E-02	-0.21076137	ZNF202	zinc finger protein 202
238032_at	2.18E-02	0.1794792		
240177_at	2.18E-02	0.07327046		
236215_at	2.18E-02	-0.31085424		
236638_at	2.19E-02	0.08131634	AMER3	APC membrane recruitment protein 3
32094_at	2.19E-02	0.15169237	CHST3	carbohydrate sulfotransferase 3
203368_at	2.19E-02	0.17574842	CRELD1	cysteine rich with EGF like domains 1
227270_at	2.19E-02	-0.2392077	FAM200B	family with sequence similarity 200 member B
219249_s_at	2.19E-02	0.11295787	FKBP10	FK506 binding protein 10
224514_x_at	2.19E-02	0.10776393	IL17RC	interleukin 17 receptor C

235552_at	2.19E-02	-0.1713135	METTL14	methyltransferase like 14
235377_at	2.19E-02	0.12920686	MLIP	muscular LMNA-interacting protein
232950_s_at	2.19E-02	0.09305301	PITPNM2	phosphatidylinositol transfer protein membrane associated 2
207827_x_at	2.19E-02	0.08191011	SNCA	synuclein alpha
217596_at	2.19E-02	0.11799221	UPF3A	UPF3 regulator of nonsense transcripts homolog A (yeast)
237554_at	2.19E-02	-0.14062229		
233218_at	2.19E-02	0.08985908		
AFFX-r2-Ec-bioB-5	2.19E-02	-0.13668869		
231987_at	2.20E-02	0.11321072	CARMN	cardiac mesoderm enhancer-associated non-coding RNA
237753_at	2.20E-02	-0.18556426	IL21R	interleukin 21 receptor
203072_at	2.20E-02	0.21434525	MYO1E	myosin IE
210120_s_at	2.20E-02	0.10560789	RANBP3	RAN binding protein 3
206306_at	2.20E-02	-0.07698411	RYR3	ryanodine receptor 3
223391_at	2.20E-02	-0.31384768	SGPP1	sphingosine-1-phosphate phosphatase 1
215519_x_at	2.20E-02	0.12173607	SGSM3	small G protein signaling modulator 3
212052_s_at	2.20E-02	0.16392375	TBC1D9B	TBC1 domain family member 9B
202699_s_at	2.20E-02	0.2843973	TMEM63A	transmembrane protein 63A
220497_at	2.20E-02	-0.14896945	ZNF214	zinc finger protein 214
1570121_at	2.20E-02	-0.06766925	ZNF365	zinc finger protein 365
221048_x_at	2.21E-02	-0.08337843	C17orf80	chromosome 17 open reading frame 80
203719_at	2.21E-02	0.25164197	ERCC1	ERCC excision repair 1, endonuclease non-catalytic subunit
219709_x_at	2.21E-02	0.27530764	FAM173A	family with sequence similarity 173 member A
214005_at	2.21E-02	-0.22647634	GGCX	gamma-glutamyl carboxylase
217478_s_at	2.21E-02	-0.46416155	HLA-DMA	major histocompatibility complex, class II, DM alpha
229323_at	2.21E-02	-0.26888055	LINC00959	long intergenic non-protein coding RNA 959
229799_s_at	2.21E-02	0.08434861	NCAM1	neural cell adhesion molecule 1
207760_s_at	2.21E-02	0.19227822	NCOR2	nuclear receptor corepressor 2
231194_at	2.21E-02	0.09100107	SNX32	sorting nexin 32
227274_at	2.21E-02	-0.17583393	SYNJ2BP-COX16///SYNJ2BP	SYNJ2BP-COX16 readthrough///synaptojanin 2 binding protein
237252_at	2.21E-02	-0.13014646	THBD	thrombomodulin
201580_s_at	2.21E-02	0.33977071	TMX4	thioredoxin related transmembrane protein 4
216042_at	2.21E-02	0.09607314	TNFRSF25	TNF receptor superfamily member 25
233253_at	2.21E-02	-0.0561889		
1553515_at	2.22E-02	0.06721175	COPS9	COP9 signalosome subunit 9
202435_s_at	2.22E-02	-0.14816676	CYP1B1	cytochrome P450 family 1 subfamily B member 1
207183_at	2.22E-02	-0.24708616	GPR19	G protein-coupled receptor 19
201666_at	2.22E-02	0.39095261	TIMP1	TIMP metallopeptidase inhibitor 1
235147_at	2.22E-02	-0.64120132		
204494_s_at	2.23E-02	0.15881974	C15orf39	chromosome 15 open reading frame 39
233534_at	2.23E-02	0.05562166	KRTAP3-2	keratin associated protein 3-2
225080_at	2.23E-02	0.21366781	MYO1C	myosin IC
222908_at	2.23E-02	-0.09811626	PIEZO2	piezo type mechanosensitive ion channel component 2
224907_s_at	2.23E-02	0.15478151	SH3GLB2	SH3 domain containing GRB2 like endophilin B2
242185_at	2.23E-02	0.07616881	SREK1	splicing regulatory glutamic acid and lysine rich protein 1
215165_x_at	2.23E-02	0.16906488	UMPS	uridine monophosphate synthetase
218413_s_at	2.23E-02	-0.09348584	ZNF639	zinc finger protein 639
216797_at	2.23E-02	0.07495664		
203051_at	2.24E-02	0.17368479	BAHD1	bromo adjacent homology domain containing 1
228131_at	2.24E-02	0.22552984	ERCC1	ERCC excision repair 1, endonuclease non-catalytic subunit
218074_at	2.24E-02	0.18378709	FAM96B	family with sequence similarity 96 member B
212203_x_at	2.24E-02	0.27872668	IFITM3	interferon induced transmembrane protein 3
209341_s_at	2.24E-02	-0.2374469	IKBKB	inhibitor of kappa light polypeptide gene enhancer in B-cells, kinase beta
209841_s_at	2.24E-02	-0.18240616	LRRN3	leucine rich repeat neuronal 3
227245_at	2.24E-02	-0.22696252	NAA25	N(alpha)-acetyltransferase 25, NatB auxiliary subunit
200871_s_at	2.24E-02	0.2125603	PSAP	prosaposin

233780_at	2.24E-02	-0.17109578	RIF1	replication timing regulatory factor 1
207763_at	2.24E-02	0.10302908	S100A5	S100 calcium binding protein A5
214924_s_at	2.24E-02	0.19119227	TRAK1	trafficking kinesin protein 1
216323_x_at	2.24E-02	0.16616577	TUBA3D///TUBA3C	tubulin alpha 3d///tubulin alpha 3c
212982_at	2.24E-02	-0.21810563	ZDHHC17	zinc finger DHHC-type containing 17
1554249_a_at	2.24E-02	-0.24000289	ZNF638-IT1///ZNF638	ZNF638 intronic transcript 1///zinc finger protein 638
1557835_at	2.24E-02	0.14212075		
204306_s_at	2.25E-02	0.22657579	CD151	CD151 molecule (Raph blood group)
227177_at	2.25E-02	0.32812748	CORO2A	coronin 2A
233942_at	2.25E-02	0.05459113	CYP51A1-AS1	CYP51A1 antisense RNA 1
207977_s_at	2.25E-02	0.16880375	DPT	dermatopontin
217254_s_at	2.25E-02	-0.08923069	EPO	erythropoietin
207398_at	2.25E-02	0.09651595	HOXD13	homeobox D13
207579_at	2.25E-02	0.06644914	MAGEB3	MAGE family member B3
221572_s_at	2.25E-02	0.25281117	MIR6824///SLC26A6	microRNA 6824///solute carrier family 26 member 6
200656_s_at	2.25E-02	0.29180597	P4HB	prolyl 4-hydroxylase subunit beta
241754_at	2.25E-02	-0.20828293	SCAI	suppressor of cancer cell invasion
230564_at	2.25E-02	0.0772715	SIPA1L3	signal induced proliferation associated 1 like 3
201859_at	2.25E-02	-0.335946	SRGN	serglycin
215299_x_at	2.25E-02	0.25363661	SULT1A1	sulfotransferase family 1A member 1
244586_x_at	2.25E-02	-0.09528556		
202222_s_at	2.26E-02	0.17447248	DES	desmin
230816_at	2.26E-02	-0.08030056	FAM163B	family with sequence similarity 163 member B
238898_at	2.26E-02	-0.41435199	FOXP4-AS1	FOXP4 antisense RNA 1
227934_at	2.26E-02	-0.31482374	KPNA5	karyopherin subunit alpha 5
236798_at	2.26E-02	-0.4652532	LINC00888	long intergenic non-protein coding RNA 888
223573_s_at	2.26E-02	0.07123531	PPP2R2C	protein phosphatase 2 regulatory subunit Bgamma
204166_at	2.26E-02	0.14804654	SBNO2	strawberry notch homolog 2
209593_s_at	2.26E-02	0.23466896	TOR1B	torsin family 1 member B
222509_s_at	2.26E-02	0.09039476	ZNF672	zinc finger protein 672
220458_at	2.26E-02	-0.06382989		
240127_at	2.26E-02	-0.10039917		
240704_at	2.26E-02	-0.0781693		
237602_at	2.26E-02	-0.06513782		
1558670_at	2.26E-02	-0.07235971		
203174_s_at	2.27E-02	0.11971947	ARFRP1	ADP ribosylation factor related protein 1
227757_at	2.27E-02	0.17885064	CUL4A	cullin 4A
205670_at	2.27E-02	0.21921104	GAL3ST1	galactose-3-O-sulfotransferase 1
205227_at	2.27E-02	0.17715019	IL1RAP	interleukin 1 receptor accessory protein
1555502_at	2.27E-02	-0.0726092	NPSA	novel prostate-specific antigen
1556804_s_at	2.27E-02	-0.11157834	POLR3B	RNA polymerase III subunit B
216036_x_at	2.27E-02	0.11463153	WDTC1	WD and tetratricopeptide repeats 1
236443_at	2.27E-02	-0.06233455		
231179_at	2.28E-02	-0.08996856	IP6K3	inositol hexakisphosphate kinase 3
227764_at	2.28E-02	-0.39028653	LYPD6	LY6/PLAUR domain containing 6
235931_at	2.28E-02	-0.2416383	METTL21A	methyltransferase like 21A
218997_at	2.28E-02	0.14975546	POLR1E	RNA polymerase I subunit E
217827_s_at	2.28E-02	0.17999462	SPG21	spastic paraplegia 21 (autosomal recessive, Mast syndrome)
213352_at	2.28E-02	0.26845564	TMCC1	transmembrane and coiled-coil domain family 1
205634_x_at	2.28E-02	0.21889865	ZDHHC24	zinc finger DHHC-type containing 24
1555510_at	2.28E-02	-0.10659625	ZNF215	zinc finger protein 215
242894_at	2.28E-02	-0.23456656		
234801_s_at	2.29E-02	0.16758601	ACSS1	acyl-CoA synthetase short-chain family member 1
213101_s_at	2.29E-02	0.10681582	ACTR3	ARP3 actin related protein 3 homolog
218868_at	2.29E-02	-0.23288355	ACTR3B	ARP3 actin related protein 3 homolog B
1558740_s_at	2.29E-02	-0.17839739	LOC105369662	uncharacterized LOC105369662
231141_at	2.29E-02	0.07210528	LOC105370962	uncharacterized LOC105370962
217549_at	2.29E-02	-0.12924037	NCKAP1L	NCK associated protein 1 like
1560430_at	2.29E-02	0.08744242	NKPD1	NTPase, KAP family P-loop domain containing 1
209940_at	2.29E-02	0.21986047	PARP3	poly(ADP-ribose) polymerase family member 3
200634_at	2.29E-02	0.33753169	PFN1	profilin 1
212262_at	2.29E-02	-0.24883985	QKI	QKI, KH domain containing RNA binding

203018_s_at	2.29E-02	-0.28715965	SSX2IP	SSX family member 2 interacting protein
216565_x_at	2.29E-02	0.29678003		
234675_x_at	2.29E-02	-0.13578557		
204205_at	2.30E-02	-0.36730796	APOBEC3G	apolipoprotein B mRNA editing enzyme catalytic subunit 3G
224732_at	2.30E-02	0.1722087	CHTF8	chromosome transmission fidelity factor 8
202894_at	2.30E-02	0.19754357	EPHB4	EPH receptor B4
230371_at	2.30E-02	0.07052305	HPS6	HPS6, biogenesis of lysosomal organelles complex 2 subunit 3
1563247_at	2.30E-02	0.07964453	LOC101927526	uncharacterized LOC101927526
1565765_x_at	2.30E-02	-0.07963789	MEDAG	mesenteric estrogen dependent adipogenesis
204632_at	2.30E-02	0.10983251	RPS6KA4	ribosomal protein S6 kinase A4
222838_at	2.30E-02	-0.39509578	SLAMF7	SLAM family member 7
218485_s_at	2.30E-02	0.16121542	SLC35C1	solute carrier family 35 member C1
215601_at	2.30E-02	-0.07321059		
1558075_at	2.30E-02	-0.10568671		
217587_at	2.30E-02	0.07335795		
202229_s_at	2.31E-02	-0.06864119	CHERP	calcium homeostasis endoplasmic reticulum protein
221754_s_at	2.31E-02	0.16142188	CORO1B	coronin 1B
216734_s_at	2.31E-02	0.07290792	CXCR5	C-X-C motif chemokine receptor 5
201885_s_at	2.31E-02	0.17946253	CYB5R3	cytochrome b5 reductase 3
230806_s_at	2.31E-02	0.1032953	FAM65A	family with sequence similarity 65 member A
202104_s_at	2.31E-02	0.25753093	LOC101930112///SPG7	uncharacterized LOC101930112///SPG7, paraplegin matrix AAA peptidase subunit
238339_x_at	2.31E-02	0.07909112	LRIG1	leucine rich repeats and immunoglobulin like domains 1
1552666_a_at	2.31E-02	0.06159389	LRRC7	leucine rich repeat containing 7
243749_s_at	2.31E-02	-0.10565147	MINCR	MYC-induced long noncoding RNA
215242_at	2.31E-02	0.21425029	PIGC	phosphatidylinositol glycan anchor biosynthesis class C
216234_s_at	2.31E-02	0.09148438	PRKACA	protein kinase cAMP-activated catalytic subunit alpha
227124_at	2.31E-02	-0.20800816	SMIM13	small integral membrane protein 13
244713_at	2.31E-02	0.09557791		
220915_s_at	2.31E-02	-0.20628226		
1554951_at	2.31E-02	0.06876568		
53202_at	2.32E-02	-0.22845121	C7orf25///PSMA2	chromosome 7 open reading frame 25///proteasome subunit alpha 2
202248_at	2.32E-02	0.13970604	E2F4	E2F transcription factor 4
237298_at	2.32E-02	0.06306737	FLJ26850	FLJ26850 protein
211074_at	2.32E-02	-0.23422146	FOLR1	folate receptor 1
232936_at	2.32E-02	-0.06328236	KCNA7	potassium voltage-gated channel subfamily A member 7
225760_at	2.32E-02	-0.18284266	MYSM1	Myb like, SWIRM and MPN domains 1
229854_at	2.32E-02	0.12803433	OBSCN	obscurin, cytoskeletal calmodulin and titin-interacting RhoGEF
214305_s_at	2.32E-02	-0.26487082	SF3B1	splicing factor 3b subunit 1
210033_s_at	2.32E-02	0.06522362	SPAG6	sperm associated antigen 6
1552522_at	2.32E-02	-0.09329285	TIGD4	tigger transposable element derived 4
217285_at	2.32E-02	-0.05786155	TSSK2///DGCR14	testis specific serine kinase 2///DiGeorge syndrome critical region gene 14
207628_s_at	2.32E-02	0.22042378	WBSCR22	Williams-Beuren syndrome chromosome region 22
202144_s_at	2.33E-02	-0.2313236	ADSL	adenylosuccinate lyase
1555736_a_at	2.33E-02	0.21159039	AGTRAP	angiotensin II receptor associated protein
210981_s_at	2.33E-02	0.12830557	GRK6	G protein-coupled receptor kinase 6
1557848_at	2.33E-02	0.10613788	LOC101929084	uncharacterized LOC101929084
222334_at	2.33E-02	-0.07235633	LOC101929219///LOC100505650///C1orf186	uncharacterized LOC101929219///uncharacterized LOC100505650///chromosome 1 open reading frame 186
206503_x_at	2.33E-02	0.21050321	PML	promyelocytic leukemia
202319_at	2.33E-02	-0.26999486	SENP6	SUMO1/sentrin specific peptidase 6
229112_at	2.33E-02	-0.13919983	SIRT5	sirtuin 5
201448_at	2.33E-02	-0.21260791	TIA1	TIA1 cytotoxic granule-associated RNA binding protein
204732_s_at	2.33E-02	-0.26392422	TRIM23	tripartite motif containing 23
217100_s_at	2.33E-02	0.21560671	UBXN7	UBX domain protein 7

229743_at	2.33E-02	0.16966035	ZNF438	zinc finger protein 438
240445_at	2.33E-02	0.11718805		
229029_at	2.34E-02	-0.13112843	CAMK4	calcium/calmodulin dependent protein kinase IV
225527_at	2.34E-02	0.19625906	CEBPG	CCAAT/enhancer binding protein gamma
1556356_at	2.34E-02	0.07067034	ERICH1	glutamate rich 1
222552_at	2.34E-02	-0.21430265	GOLT1B	golgi transport 1B
234477_at	2.34E-02	0.08384045	IGHV4-31///IGHA1	immunoglobulin heavy variable 4-31///immunoglobulin heavy constant alpha 1
1560851_at	2.34E-02	-0.17877	LINC00619	long intergenic non-protein coding RNA 619
203414_at	2.34E-02	-0.21886397	MMD	monocyte to macrophage differentiation associated
216671_x_at	2.34E-02	0.10049756	MUC8	mucin 8
1567248_at	2.34E-02	0.05775657	OR9A1P	olfactory receptor family 9 subfamily A member 1 pseudogene
216913_s_at	2.34E-02	0.19785626	RRP12	ribosomal RNA processing 12 homolog
218122_s_at	2.34E-02	0.13520061	SENP2	SUMO1/sentrin/SMT3 specific peptidase 2
218848_at	2.34E-02	0.27018745	THOC6	THO complex 6
230211_at	2.34E-02	-0.25843877	TRIP11	thyroid hormone receptor interactor 11
221784_at	2.34E-02	0.1075196	WIZ	widely interspaced zinc finger motifs
1563420_at	2.34E-02	0.06556154	XGY2///XG	Xg pseudogene, Y-linked 2///Xg blood group
233204_at	2.34E-02	-0.30156681		
221929_at	2.34E-02	0.08445204		
239859_x_at	2.35E-02	-0.2201489	ATP5S	ATP synthase, H+ transporting, mitochondrial Fo complex subunit s (factor B)
204384_at	2.35E-02	0.22379599	GOLGA2	golgin A2
221214_s_at	2.35E-02	0.209298	MIR7114///NSMF	microRNA 7114///NMDA receptor synaptonuclear signaling and neuronal migration factor
218301_at	2.35E-02	0.12546367	RNPEPL1	arginyl aminopeptidase like 1
208883_at	2.35E-02	-0.26627899	UBR5	ubiquitin protein ligase E3 component n-recognin 5
244798_at	2.35E-02	0.08214106		
1561584_at	2.35E-02	-0.05502293		
230400_s_at	2.35E-02	-0.14524624		
205963_s_at	2.36E-02	0.188147	DNAJA3	DnaJ heat shock protein family (Hsp40) member A3
208557_at	2.36E-02	0.16133406	HOXA6	homeobox A6
218614_at	2.36E-02	-0.24191906	KIAA1551	KIAA1551
224483_s_at	2.36E-02	0.15912068	MFSD9	major facilitator superfamily domain containing 9
207473_at	2.36E-02	0.07316533	MLN	motilin
210574_s_at	2.36E-02	0.22977564	NUDC	nuclear distribution C, dynein complex regulator
211013_x_at	2.36E-02	0.20088432	PML	promyelocytic leukemia
1552630_a_at	2.36E-02	0.09380007	SRCAP	Snf2-related CREBBP activator protein
1569788_at	2.36E-02	-0.09891407	ST8SIA1	ST8 alpha-N-acetyl-neuraminide alpha-2,8-sialyltransferase 1
200822_x_at	2.36E-02	0.25165117	TPI1	triosephosphate isomerase 1
1569569_x_at	2.36E-02	0.07565505		
234312_s_at	2.37E-02	0.26368947	ACSS2	acyl-CoA synthetase short-chain family member 2
211453_s_at	2.37E-02	0.09331897	AKT2	AKT serine/threonine kinase 2
226290_at	2.37E-02	-0.21551935	BDP1	B double prime 1, subunit of RNA polymerase III transcription initiation factor IIIB
1552559_a_at	2.37E-02	-0.11641442	CDK15	cyclin dependent kinase 15
208485_x_at	2.37E-02	0.1331975	CFLAR	CASP8 and FADD like apoptosis regulator
218660_at	2.37E-02	-0.14053034	DYSF	dysferlin
201017_at	2.37E-02	-0.27068455	EIF1AX	eukaryotic translation initiation factor 1A, X-linked
219720_s_at	2.37E-02	-0.15596475	GPATCH2L	G-patch domain containing 2 like
223155_at	2.37E-02	-0.20684198	HDHD2	haloacid dehalogenase like hydrolase domain containing 2
1552432_at	2.37E-02	0.08207518	MFSD6L	major facilitator superfamily domain containing 6 like
200617_at	2.37E-02	0.24053383	MLEC	malectin
211846_s_at	2.37E-02	0.1023151	NECTIN1	nectin cell adhesion molecule 1
221237_s_at	2.37E-02	0.09266728	OSBP2	oxysterol binding protein 2
217747_s_at	2.37E-02	0.2047168	RPS9	ribosomal protein S9
201801_s_at	2.37E-02	0.23635238	SLC29A1	solute carrier family 29 member 1 (Augustine blood group)
201471_s_at	2.37E-02	0.24608736	SQSTM1	sequestosome 1
243009_at	2.37E-02	-0.34372148		

238655_at	2.38E-02	0.11764521	ACAD10	acyl-CoA dehydrogenase family member 10
200663_at	2.38E-02	0.26242744	CD63	CD63 molecule
213187_x_at	2.38E-02	0.25914521	FTL	ferritin light chain
219407_s_at	2.38E-02	0.10593748	LAMC3	laminin subunit gamma 3
217263_x_at	2.38E-02	0.07198874	LOC101928269///LOC100506403///RUNX1	uncharacterized LOC101928269///uncharacterized LOC100506403///runt related transcription factor 1
219643_at	2.38E-02	-0.08424742	LRP1B	LDL receptor related protein 1B
211912_at	2.38E-02	-0.09673602	MERTK	MER proto-oncogene, tyrosine kinase
238762_at	2.38E-02	-0.30691483	MTHFD2L	methylenetetrahydrofolate dehydrogenase (NADP+ dependent) 2-like
228883_at	2.38E-02	0.08159684	TUB	tubby bipartite transcription factor
202154_x_at	2.38E-02	0.25281382	TUBB3	tubulin beta 3 class III
233893_s_at	2.38E-02	-0.19661647	UVSSA	UV stimulated scaffold protein A
225349_at	2.38E-02	0.09669763	ZNF496	zinc finger protein 496
241617_x_at	2.38E-02	-0.26196478		
1561608_at	2.38E-02	-0.06500717		
241029_at	2.38E-02	-0.10038391		
209956_s_at	2.39E-02	0.06707326	CAMK2B	calcium/calmodulin dependent protein kinase II beta
230391_at	2.39E-02	-0.2716131	CD84	CD84 molecule
217973_at	2.39E-02	0.27509148	DCXR	dicarbonyl and L-xylulose reductase
201790_s_at	2.39E-02	0.35603222	DHCR7	7-dehydrocholesterol reductase
205231_s_at	2.39E-02	-0.1829186	EPM2A	epilepsy, progressive myoclonus type 2A, Lafora disease (laforin)
201350_at	2.39E-02	0.19668271	FLOT2	flotillin 2
33850_at	2.39E-02	0.13502993	MAP4	microtubule associated protein 4
215464_s_at	2.39E-02	0.28260224	P2RX5-TAX1BP3///TAX1BP3///P2RX5	P2RX5-TAX1BP3 readthrough (NMD candidate)///Tax1 binding protein 3///purinergic receptor P2X 5
200876_s_at	2.39E-02	0.21180747	PSMB1	proteasome subunit beta 1
214437_s_at	2.39E-02	0.35627515	SHMT2	serine hydroxymethyltransferase 2
211385_x_at	2.39E-02	0.22450484	SULT1A2	sulfotransferase family 1A member 2
203313_s_at	2.39E-02	0.27502488	TGIF1	TGFB induced factor homeobox 1
238454_at	2.39E-02	0.1270816	ZNF540	zinc finger protein 540
234696_at	2.39E-02	0.09042887		
239116_at	2.40E-02	-0.0743004	ANKRD10	ankyrin repeat domain 10
233895_at	2.40E-02	0.08742169	ANKRD24	ankyrin repeat domain 24
214355_x_at	2.40E-02	0.23080295	CTAGE8///CTAGE4///CTAGE9///CTAGE15	CTAGE family member 8///CTAGE family member 4///CTAGE family member 9///CTAGE family member 15
1557749_at	2.40E-02	0.13025829	EHBP1L1	EH domain binding protein 1 like 1
240871_at	2.40E-02	0.09190747	FAM221B	family with sequence similarity 221 member B
1558202_at	2.40E-02	0.06386383	LOC145783///ZNF280D	uncharacterized LOC145783///zinc finger protein 280D
232704_s_at	2.40E-02	-0.09116908	LRRFIP2	LRR binding FLII interacting protein 2
221424_s_at	2.40E-02	-0.0883567	OR51E2	olfactory receptor family 51 subfamily E member 2
234952_s_at	2.40E-02	0.15035432	PHRF1	PHD and ring finger domains 1
228084_at	2.40E-02	-0.2669546	PLA2G12A	phospholipase A2 group XIIA
218738_s_at	2.40E-02	-0.19444905	RNF138	ring finger protein 138
1562086_at	2.40E-02	-0.05402791		
242579_at	2.41E-02	-0.23769966	BMPR1B	bone morphogenetic protein receptor type 1B
213631_x_at	2.41E-02	-0.11361377	DHODH	dihydroorotate dehydrogenase (quinone)
210963_s_at	2.41E-02	0.16730771	GYG2	glycogenin 2
210515_at	2.41E-02	0.12098484	HNF1A	HNF1 homeobox A
229741_at	2.41E-02	0.29846078	MAVS	mitochondrial antiviral signaling protein
238700_at	2.41E-02	-0.24431092	PIAS2	protein inhibitor of activated STAT 2
229856_s_at	2.41E-02	-0.09102134	PITHD1	PITH domain containing 1
209730_at	2.41E-02	-0.08875872	SEMA3F	semaphorin 3F
1552295_a_at	2.41E-02	0.11663151	SLC39A13	solute carrier family 39 member 13
1555653_at	2.41E-02	0.24476908		
237459_at	2.41E-02	-0.29336834		
221636_s_at	2.42E-02	-0.33464233	MTARC2	mitochondrial amidoxime reducing component 2
89977_at	2.42E-02	0.06059252	ACSM5	acyl-CoA synthetase medium-chain family member 5
235650_at	2.42E-02	0.07687133	CDHR3	cadherin related family member 3
212611_at	2.42E-02	0.1820058	DTX4	deltex E3 ubiquitin ligase 4
210103_s_at	2.42E-02	0.27787489	FOXA2	forkhead box A2

222691_at	2.42E-02	-0.20559726	SLC35B3	solute carrier family 35 member B3
1564937_at	2.42E-02	0.05683036		
217450_at	2.43E-02	0.06582737	AGFG2	ArfGAP with FG repeats 2
210306_at	2.43E-02	-0.48217204	L3MBTL1	l(3)mbt-like 1 (Drosophila)
1554691_a_at	2.43E-02	0.14058373	PACSIN2	protein kinase C and casein kinase substrate in neurons 2
212654_at	2.43E-02	0.16498113	TPM2	tropomyosin 2 (beta)
234860_at	2.43E-02	0.07639222		
215279_at	2.43E-02	0.0894064		
237726_at	2.43E-02	0.07861486		
226109_at	2.44E-02	-0.23792007	C21orf91	chromosome 21 open reading frame 91
202878_s_at	2.44E-02	-0.30202107	CD93	CD93 molecule
209732_at	2.44E-02	-0.38560795	CLEC2B	C-type lectin domain family 2 member B
220926_s_at	2.44E-02	0.24639357	EDEM3	ER degradation enhancing alpha-mannosidase like protein 3
241898_at	2.44E-02	0.38512682	LIPH	lipase H
203007_x_at	2.44E-02	-0.25384452	LYPLA1	lysophospholipase I
212209_at	2.44E-02	-0.17895557	MED13L	mediator complex subunit 13 like
229265_at	2.44E-02	0.16417856	SKI	SKI proto-oncogene
203743_s_at	2.44E-02	-0.17139404	TDG	thymine DNA glycosylase
232117_at	2.44E-02	-0.08717671	ZNF471	zinc finger protein 471
233727_at	2.44E-02	-0.10058582		
211986_at	2.45E-02	0.29953984	AHNAK	AHNAK nucleoprotein
226383_at	2.45E-02	-0.20728117	ARL14EP	ADP ribosylation factor like GTPase 14 effector protein
244569_at	2.45E-02	-0.21694061	C8orf37	chromosome 8 open reading frame 37
201975_at	2.45E-02	-0.1657097	CLIP1	CAP-Gly domain containing linker protein 1
207261_at	2.45E-02	-0.07781161	CNGA3	cyclic nucleotide gated channel alpha 3
231944_at	2.45E-02	-0.44626122	ERO1B	endoplasmic reticulum oxidoreductase 1 beta
238790_at	2.45E-02	-0.10936556	LOC374443	C-type lectin domain family 2 member D pseudogene
201461_s_at	2.45E-02	0.1339307	MAPKAPK2	mitogen-activated protein kinase-activated protein kinase 2
43544_at	2.45E-02	0.23438626	MED16	mediator complex subunit 16
230122_at	2.45E-02	0.21362932	MLLT10	myeloid/lymphoid or mixed-lineage leukemia; translocated to, 10
208452_x_at	2.45E-02	0.09871161	MYO9B	myosin IXB
209155_s_at	2.45E-02	0.16388379	NT5C2	5'-nucleotidase, cytosolic II
225979_at	2.45E-02	0.11772984	PLEKHG2	pleckstrin homology and RhoGEF domain containing G2
219676_at	2.45E-02	-0.23306168	ZSCAN16	zinc finger and SCAN domain containing 16
1570021_at	2.45E-02	-0.30433618		
204302_s_at	2.46E-02	0.09092761	CTIF	cap binding complex dependent translation initiation factor
202596_at	2.46E-02	0.17038535	ENSA	endosulfine alpha
218778_x_at	2.46E-02	0.06282037	EPS8L1	EPS8 like 1
233498_at	2.46E-02	-0.08201838	ERBB4	erb-b2 receptor tyrosine kinase 4
1556662_at	2.46E-02	0.05986349	LOC100506142	uncharacterized LOC100506142
225719_s_at	2.46E-02	0.17483988	MRPL55	mitochondrial ribosomal protein L55
44617_at	2.46E-02	0.14820935	OGFOD2	2-oxoglutarate and iron dependent oxygenase domain containing 2
202243_s_at	2.46E-02	0.22747495	PSMB4	proteasome subunit beta 4
200677_at	2.46E-02	0.15456902	PTTG1IP	pituitary tumor-transforming 1 interacting protein
202988_s_at	2.46E-02	-0.48683319	RGS1	regulator of G-protein signaling 1
213510_x_at	2.46E-02	-0.2297814	USP32P2	ubiquitin specific peptidase 32 pseudogene 2
221998_s_at	2.46E-02	0.16784087	VRK3	vaccinia related kinase 3
1556581_at	2.46E-02	-0.11252765	ZNF778	zinc finger protein 778
239646_at	2.46E-02	-0.2147371		
1561737_at	2.46E-02	-0.14994453		
239264_at	2.46E-02	-0.26471691		
223355_at	2.47E-02	0.18295799	ALG1	ALG1, chitobiosyldiphosphodolichol beta-mannosyltransferase
210667_s_at	2.47E-02	0.19394595	C21orf33	chromosome 21 open reading frame 33
1552703_s_at	2.47E-02	-0.44205499	CARD16///CASP1	caspase recruitment domain family member 16///caspase 1

1554677_s_at	2.47E-02	0.15539104	CMTM4	CKLF like MARVEL transmembrane domain containing 4
217844_at	2.47E-02	0.13510252	CTDSP1	CTD small phosphatase 1
1552705_at	2.47E-02	-0.11952474	DUSP19	dual specificity phosphatase 19
218342_s_at	2.47E-02	0.21262832	ERMP1	endoplasmic reticulum metallopeptidase 1
203618_at	2.47E-02	0.09043104	FAIM2	Fas apoptotic inhibitory molecule 2
220312_at	2.47E-02	0.13656426	FAM83E	family with sequence similarity 83 member E
1558383_at	2.47E-02	-0.07703567	KPNA4	karyopherin subunit alpha 4
219324_at	2.47E-02	0.19630505	NOL12	nucleolar protein 12
214066_x_at	2.47E-02	0.12459744	NPR2	natriuretic peptide receptor 2
201373_at	2.47E-02	0.15427711	PLEC	plectin
206004_at	2.47E-02	0.12270082	TGM3	transglutaminase 3
205025_at	2.47E-02	0.17582756	ZBTB48	zinc finger and BTB domain containing 48
244024_at	2.47E-02	-0.24820661	ZNF182	zinc finger protein 182
1566098_at	2.47E-02	-0.07953984		
1566836_at	2.47E-02	-0.05120067		
1564139_at	2.48E-02	-0.11872212	A2M-AS1	A2M antisense RNA 1 (head to head)
1558636_s_at	2.48E-02	-0.05609231	ADAMTS5	ADAM metallopeptidase with thrombospondin type 1 motif 5
225672_at	2.48E-02	0.13175159	GOLGA2	golgin A2
207072_at	2.48E-02	-0.19441505	IL18RAP	interleukin 18 receptor accessory protein
1561728_a_at	2.48E-02	0.05587829	LINC00642	long intergenic non-protein coding RNA 642
1569315_s_at	2.48E-02	-0.23625331	LINC00894	long intergenic non-protein coding RNA 894
1563042_at	2.48E-02	0.07727467	LOC338694	uncharacterized LOC338694
219550_at	2.48E-02	-0.12606034	ROBO3	roundabout guidance receptor 3
213324_at	2.48E-02	0.19291451	SRC	SRC proto-oncogene, non-receptor tyrosine kinase
215994_x_at	2.48E-02	0.12388487	TBC1D9B	TBC1 domain family member 9B
201581_at	2.48E-02	0.38217248	TMX4	thioredoxin related transmembrane protein 4
215674_at	2.48E-02	-0.05085628		
241464_s_at	2.48E-02	0.16538531		
1552733_at	2.49E-02	-0.23640292	KLHDC1	kelch domain containing 1
210712_at	2.49E-02	-0.06428079	LDHAL6B	lactate dehydrogenase A like 6B
244138_at	2.49E-02	0.05649197	LOC101929154	uncharacterized LOC101929154
222014_x_at	2.49E-02	-0.18182265	MTO1	mitochondrial tRNA translation optimization 1
200815_s_at	2.49E-02	0.22651692	PAFAH1B1	platelet activating factor acetylhydrolase 1b regulatory subunit 1
226714_at	2.49E-02	0.09426435	SAMD4B	sterile alpha motif domain containing 4B
227426_at	2.49E-02	-0.18398279	SOS1	SOS Ras/Rac guanine nucleotide exchange factor 1
234514_at	2.49E-02	0.07443956	SRMS	src-related kinase lacking C-terminal regulatory tyrosine and N-terminal myristylation sites
202096_s_at	2.49E-02	0.28592088	TSPO	translocator protein
214900_at	2.49E-02	0.18016124	ZKSCAN1	zinc finger with KRAB and SCAN domains 1
213238_at	2.50E-02	-0.40485979	ATP10D	ATPase phospholipid transporting 10D (putative)
228851_s_at	2.50E-02	0.13624313	ENSA	endosulfine alpha
215510_at	2.50E-02	0.08868056	ETV2	ETS variant 2
216080_s_at	2.50E-02	0.18303459	FADS3	fatty acid desaturase 3
210148_at	2.50E-02	0.14290011	HIPK3	homeodomain interacting protein kinase 3
238231_at	2.50E-02	-0.1053789	NFYC	nuclear transcription factor Y subunit gamma
1554635_a_at	2.50E-02	0.06825739	NPAS3	neuronal PAS domain protein 3
211942_x_at	2.50E-02	0.22334898	RPL13AP5///RPL13AP6///SNORD32A///SNORD33///SNORD34///SNORD35A///RPL13A	ribosomal protein L13a pseudogene 5///ribosomal protein L13a pseudogene 6///small nucleolar RNA, C/D box 32A///small nucleolar RNA, C/D box 33///small nucleolar RNA, C/D box 34///small nucleolar RNA, C/D box 35A///ribosomal protein L13a
213649_at	2.50E-02	-0.25621866	SRSF7	serine and arginine rich splicing factor 7
223246_s_at	2.50E-02	-0.16872817	STRBP	spermatid perinuclear RNA binding protein
223186_at	2.50E-02	0.16214834	TMEM189-UBE2V1///TMEM189///UBE2V1	TMEM189-UBE2V1 readthrough///transmembrane protein 189///ubiquitin conjugating enzyme E2 V1
213448_at	2.50E-02	0.16542399		
228899_at	2.50E-02	-0.25563491		
236419_at	2.50E-02	-0.14821388		
212159_x_at	2.51E-02	0.21517721	AP2A2	adaptor related protein complex 2 alpha 2 subunit
224604_at	2.51E-02	-0.2516708	C4orf3	chromosome 4 open reading frame 3

233844_at	2.51E-02	0.08479976	CD99L2	CD99 molecule like 2
1559646_a_at	2.51E-02	0.07454581	LINC00184	long intergenic non-protein coding RNA 184
217335_at	2.51E-02	-0.06490473	LOC55338	uncharacterized LOC55338
233941_at	2.51E-02	-0.09721584	LRRC74A	leucine rich repeat containing 74A
200836_s_at	2.51E-02	0.09492393	MAP4	microtubule associated protein 4
215832_x_at	2.51E-02	0.15334303	PICALM	phosphatidylinositol binding clathrin assembly protein
206361_at	2.51E-02	0.08647091	PTGDR2	prostaglandin D2 receptor 2
214855_s_at	2.51E-02	-0.24448986	RALGAPA1///RALGAPA1P1	Ral GTPase activating protein catalytic alpha subunit 1///Ral GTPase activating protein catalytic alpha subunit 1 pseudogene 1
215834_x_at	2.51E-02	0.09731828	SCARB1	scavenger receptor class B member 1
224019_at	2.51E-02	0.06871225	SCD5	stearoyl-CoA desaturase 5
205622_at	2.51E-02	0.16310379	SMPD2	sphingomyelin phosphodiesterase 2
1556178_x_at	2.51E-02	-0.12877174	TAF8	TATA-box binding protein associated factor 8
1557357_at	2.51E-02	-0.15581205	THUMPD3-AS1	THUMPD3 antisense RNA 1
239120_at	2.51E-02	0.13425048	TMEM51-AS1	TMEM51 antisense RNA 1
232362_at	2.52E-02	-0.23312404	CCDC18	coiled-coil domain containing 18
214533_at	2.52E-02	0.07216011	CMA1	chymase 1
228496_s_at	2.52E-02	-0.28791645	CRIM1	cysteine rich transmembrane BMP regulator 1
200765_x_at	2.52E-02	0.15522563	CTNNA1	catenin alpha 1
1554272_at	2.52E-02	-0.06214057	ERAP2	endoplasmic reticulum aminopeptidase 2
205671_s_at	2.52E-02	0.22798016	HLA-DOB	major histocompatibility complex, class II, DO beta
211220_s_at	2.52E-02	-0.09946235	HSF2	heat shock transcription factor 2
228291_s_at	2.52E-02	0.38247438	KIZ	kizuna centrosomal protein
206316_s_at	2.52E-02	-0.20102881	KNTC1	kinetochore associated 1
208158_s_at	2.52E-02	0.51619229	OSBPL1A	oxysterol binding protein like 1A
1558785_a_at	2.52E-02	-0.11417443	RGS5	regulator of G-protein signaling 5
236690_at	2.52E-02	0.09833027	RHBDD1	rhomboid domain containing 1
229528_at	2.52E-02	-0.18575029	SBNO1	strawberry notch homolog 1
222047_s_at	2.52E-02	0.14523977	SRRT	serrate, RNA effector molecule
232626_at	2.52E-02	-0.15369991		
229143_at	2.53E-02	0.14726117	CNOT3	CCR4-NOT transcription complex subunit 3
227333_at	2.53E-02	0.17542379	DCUN1D3	defective in cullin neddylation 1 domain containing 3
229558_at	2.53E-02	0.16200041	KNOP1	lysine rich nucleolar protein 1
1568718_at	2.53E-02	0.12211094	SLC22A23	solute carrier family 22 member 23
211439_at	2.53E-02	-0.10014538	SRSF7	serine and arginine rich splicing factor 7
206308_at	2.53E-02	-0.20536734	TRDMT1	tRNA aspartic acid methyltransferase 1
238057_at	2.53E-02	-0.26682352	USP45	ubiquitin specific peptidase 45
241536_at	2.53E-02	0.09138393		
220514_at	2.53E-02	0.08222604		
205566_at	2.54E-02	0.36166926	ABHD2	abhydrolase domain containing 2
1561378_at	2.54E-02	0.06467994	C12orf42	chromosome 12 open reading frame 42
206782_s_at	2.54E-02	0.15193981	DNAJC4	DnaJ heat shock protein family (Hsp40) member C4
206871_at	2.54E-02	0.0713217	ELANE	elastase, neutrophil expressed
232297_at	2.54E-02	-0.36808303	KLHL5	kelch like family member 5
225575_at	2.54E-02	-0.34876105	LIFR	leukemia inhibitory factor receptor alpha
234816_at	2.54E-02	0.09988123	LINC00521	long intergenic non-protein coding RNA 521
212867_at	2.54E-02	-0.23944779	NCOA2	nuclear receptor coactivator 2
225758_s_at	2.54E-02	0.21167739	TUBGCP6	tubulin gamma complex associated protein 6
225982_at	2.54E-02	0.12787538	UBTF	upstream binding transcription factor, RNA polymerase I
237604_at	2.54E-02	0.0965085		
244450_at	2.54E-02	-0.21879233		
230223_at	2.55E-02	0.10365818	CINP	cyclin dependent kinase 2 interacting protein
211398_at	2.55E-02	-0.07429483	FGFR2	fibroblast growth factor receptor 2
242586_at	2.55E-02	-0.4460942	FSD1L	fibronectin type III and SPRY domain containing 1 like
200593_s_at	2.55E-02	0.19645677	HNRNPU	heterogeneous nuclear ribonucleoprotein U
215616_s_at	2.55E-02	0.07207779	KDM4B	lysine demethylase 4B
236693_at	2.55E-02	0.07748818	MIR124-2HG	MIR124-2 host gene
218931_at	2.55E-02	0.22583681	RAB17	RAB17, member RAS oncogene family
203572_s_at	2.55E-02	0.12863937	TAF6	TATA-box binding protein associated factor 6
219572_at	2.56E-02	-0.45049247	CADPS2	calcium dependent secretion activator 2
234688_x_at	2.56E-02	0.08884554	CNTROB	centrobin, centriole duplication and spindle assembly protein

201718_s_at	2.56E-02	0.39864991	EPB41L2	erythrocyte membrane protein band 4.1 like 2
201376_s_at	2.56E-02	0.23600868	HNRNPF	heterogeneous nuclear ribonucleoprotein F
1552857_a_at	2.56E-02	0.0843021	HTR6	5-hydroxytryptamine receptor 6
1557280_s_at	2.56E-02	0.07652281	ITSN1	intersectin 1
212713_at	2.56E-02	0.14997709	MFAP4	microfibrillar associated protein 4
227618_at	2.56E-02	-0.10634841	NHSL2	NHS like 2
211526_s_at	2.56E-02	0.06433596	RTEL1-TNFRSF6B///RTEL1	RTEL1-TNFRSF6B readthrough (NMD candidate)///regulator of telomere elongation helicase 1
226059_at	2.56E-02	0.23740411	TOMM40L	translocase of outer mitochondrial membrane 40 like
226566_at	2.56E-02	0.1616774	TRIM11	tripartite motif containing 11
220055_at	2.56E-02	-0.07057475	ZNF287	zinc finger protein 287
1566456_at	2.56E-02	-0.10462475		
241571_at	2.56E-02	0.07301633		
239166_at	2.56E-02	-0.19127001		
231269_at	2.57E-02	-0.27390693	ASCC3	activating signal cointegrator 1 complex subunit 3
240444_x_at	2.57E-02	0.06581791	CLIP1	CAP-Gly domain containing linker protein 1
1557843_at	2.57E-02	-0.04779756	LINC01087	long intergenic non-protein coding RNA 1087
225899_x_at	2.57E-02	0.2916209	LOC101929819///LOC100133331///LOC100133182///LINC01001///LOC100132062///LOC729737///LINC01000///LINC01002	uncharacterized LOC101929819///uncharacterized LOC100133331///putative uncharacterized protein FLJ44672///long intergenic non-protein coding RNA 1001///uncharacterized LOC100132062///uncharacterized LOC729737///long intergenic non-protein coding RNA 1000///long intergenic non-protein coding RNA 1002
204737_s_at	2.57E-02	0.06984288	MYH7///MYH6	myosin, heavy chain 7, cardiac muscle, beta///myosin heavy chain 6
1558292_s_at	2.57E-02	-0.269487	PIGW	phosphatidylinositol glycan anchor biosynthesis class W
227403_at	2.57E-02	-0.08900571	PIGX	phosphatidylinositol glycan anchor biosynthesis class X
202033_s_at	2.57E-02	-0.27449851	RB1CC1	RB1 inducible coiled-coil 1
218055_s_at	2.57E-02	-0.20110745	WDR41	WD repeat domain 41
214659_x_at	2.57E-02	-0.23115077	YLPM1	YLP motif containing 1
1567277_at	2.58E-02	-0.07183682	CTTN	cortactin
223262_s_at	2.58E-02	-0.19977266	FGFR1OP2	FGFR1 oncogene partner 2
211730_s_at	2.58E-02	0.31926356	POLR2L	RNA polymerase II subunit L
209071_s_at	2.58E-02	-0.3984567	RGS5	regulator of G-protein signaling 5
200867_at	2.58E-02	0.23329714	RNF114	ring finger protein 114
217946_s_at	2.58E-02	0.20041878	SAE1	SUMO1 activating enzyme subunit 1
243772_at	2.58E-02	0.12280519	SDCCAG8	serologically defined colon cancer antigen 8
224130_s_at	2.58E-02	0.27655705	SRA1	steroid receptor RNA activator 1
223231_at	2.58E-02	-0.24692997	TATDN1	TatD DNase domain containing 1
235618_at	2.58E-02	0.15867817	ZNF507	zinc finger protein 507
239820_at	2.58E-02	0.06775935		
237216_at	2.58E-02	-0.27108433		
234511_at	2.59E-02	-0.09272932	ANKRD60	ankyrin repeat domain 60
223715_at	2.59E-02	0.09119077	BRSK2	BR serine/threonine kinase 2
1564008_at	2.59E-02	0.07743918	COL27A1	collagen type XXVII alpha 1 chain
214411_x_at	2.59E-02	0.08837597	CTRB2	chymotrypsinogen B2
1565338_x_at	2.59E-02	0.05767402	DNAH6	dynein axonemal heavy chain 6
224404_s_at	2.59E-02	-0.29921254	FCRL5	Fc receptor like 5
209170_s_at	2.59E-02	-0.4480168	GPM6B	glycoprotein M6B
219388_at	2.59E-02	-0.19011551	GRHL2	grainyhead like transcription factor 2
230527_at	2.59E-02	-0.25619673	LOC101926907	uncharacterized LOC101926907
244429_at	2.59E-02	-0.09642331	LOC101928617	uncharacterized LOC101928617
222818_at	2.59E-02	0.09299514	OSBPL10	oxysterol binding protein like 10
1556786_at	2.59E-02	0.04692962	PDE5A	phosphodiesterase 5A
216899_s_at	2.59E-02	0.28712732	SKAP2	src kinase associated phosphoprotein 2
206641_at	2.59E-02	-0.58466257	TNFRSF17	TNF receptor superfamily member 17
212704_at	2.59E-02	-0.27356829	ZCCHC11	zinc finger CCHC-type containing 11
217313_at	2.59E-02	0.15545573		
240867_at	2.59E-02	-0.14343024		
241193_at	2.60E-02	-0.38538925	ETS2	ETS proto-oncogene 2, transcription factor

205307_s_at	2.60E-02	-0.0774224	KMO	kynurenine 3-monooxygenase (kynurenine 3-hydroxylase)
1557498_a_at	2.60E-02	-0.05770722	LINC01487	long intergenic non-protein coding RNA 1487
238824_at	2.60E-02	-0.27863816	RPS29	ribosomal protein S29
218702_at	2.60E-02	0.21091842	SARS2	seryl-tRNA synthetase 2, mitochondrial
213082_s_at	2.60E-02	0.19888305	SLC35D2	solute carrier family 35 member D2
211590_x_at	2.60E-02	0.07333447	TBXA2R	thromboxane A2 receptor
238494_at	2.60E-02	-0.18502263	TRAF3IP1	TRAF3 interacting protein 1
205917_at	2.60E-02	0.4350965	ZNF264	zinc finger protein 264
224124_at	2.60E-02	-0.07093036	ZRANB3	zinc finger RANBP2-type containing 3
1552616_a_at	2.61E-02	0.07879184	ACACB	acetyl-CoA carboxylase beta
208816_x_at	2.61E-02	0.21709663	ANXA2P2	annexin A2 pseudogene 2
1555797_a_at	2.61E-02	0.17762193	ARPC5	actin related protein 2/3 complex subunit 5
201640_x_at	2.61E-02	0.12006292	CLPTM1	CLPTM1, transmembrane protein
209059_s_at	2.61E-02	0.23767738	EDF1	endothelial differentiation related factor 1
234460_at	2.61E-02	0.09044472	Ndufaf4	NADH:ubiquinone oxidoreductase complex assembly factor 4
231509_at	2.61E-02	0.08479401	PPP1R17	protein phosphatase 1 regulatory subunit 17
209488_s_at	2.61E-02	0.37419632	RBPMS	RNA binding protein with multiple splicing
200051_at	2.61E-02	0.21100847	SART1	squamous cell carcinoma antigen recognized by T-cells 1
221259_s_at	2.61E-02	-0.09097054	TEX11	testis expressed 11
230467_at	2.61E-02	0.16025196	TMEM52	transmembrane protein 52
244571_s_at	2.61E-02	-0.24324785	TTC12	tetratricopeptide repeat domain 12
204253_s_at	2.61E-02	0.08805181	VDR	vitamin D (1,25- dihydroxyvitamin D3) receptor
214957_at	2.62E-02	-0.14452244	ACTL8	actin like 8
201170_s_at	2.62E-02	0.35981689	BHLHE40	basic helix-loop-helix family member e40
206635_at	2.62E-02	0.07568614	CHRNB2	cholinergic receptor nicotinic beta 2 subunit
235365_at	2.62E-02	-0.23446663	DFNB59	deafness, autosomal recessive 59
233934_at	2.62E-02	0.06854387	MIR490///LOC349160	microRNA 490///uncharacterized LOC349160
1562644_at	2.62E-02	0.06539101	MTHFD2L	methylenetetrahydrofolate dehydrogenase (NADP+ dependent) 2-like
204316_at	2.62E-02	-0.15547812	RGS10	regulator of G-protein signaling 10
202761_s_at	2.62E-02	-0.28885648	SYNE2	spectrin repeat containing nuclear envelope protein 2
220417_s_at	2.62E-02	0.23147589	THAP4	THAP domain containing 4
224676_at	2.62E-02	0.25001346	TMED4	transmembrane p24 trafficking protein 4
235817_at	2.62E-02	0.0826823	TMEM184A	transmembrane protein 184A
1570160_at	2.62E-02	-0.05741426		
225372_at	2.63E-02	0.17490631	C10orf54	chromosome 10 open reading frame 54
209667_at	2.63E-02	0.51363924	CES2	carboxylesterase 2
208674_x_at	2.63E-02	0.14643117	DDOST	dolichyl-diphosphooligosaccharide--protein glycosyltransferase non-catalytic subunit
229274_at	2.63E-02	-0.280643	GNAS	GNAS complex locus
238240_at	2.63E-02	0.07939257		
232660_at	2.64E-02	-0.0776282	BAD	BCL2 associated agonist of cell death
222874_s_at	2.64E-02	0.17615588	CLN8	ceroid-lipofuscinosis, neuronal 8
235097_at	2.64E-02	-0.07633657	TRMT44	tRNA methyltransferase 44 homolog (S. cerevisiae)
236507_at	2.64E-02	0.14337013	ZDHHC3	zinc finger DHHC-type containing 3
212544_at	2.64E-02	-0.24718773	ZNHIT3	zinc finger HIT-type containing 3
242971_at	2.64E-02	-0.09287176		
1561714_a_at	2.65E-02	-0.09783327	B3GALNT2	beta-1,3-N-acetylgalactosaminyltransferase 2
34726_at	2.65E-02	0.19760791	CACNB3	calcium voltage-gated channel auxiliary subunit beta 3
206198_s_at	2.65E-02	-0.39390057	CEACAM7	carcinoembryonic antigen related cell adhesion molecule 7
222234_s_at	2.65E-02	0.14458969	DBNDD1	dysbindin domain containing 1
219016_at	2.65E-02	0.24683025	FASTKD5	FAST kinase domains 5
236994_at	2.65E-02	-0.29681675	FBXL4	F-box and leucine rich repeat protein 4
1556163_a_at	2.65E-02	0.07879744	IGSF3	immunoglobulin superfamily member 3
224473_x_at	2.65E-02	0.17551524	LZTS2	leucine zipper tumor suppressor 2
225875_s_at	2.65E-02	0.18120307	NIPAL3	NIPA like domain containing 3
204766_s_at	2.65E-02	0.23309476	NUDT1	nudix hydrolase 1
213263_s_at	2.65E-02	-0.32620059	PCBP2	poly(rC) binding protein 2
223571_at	2.66E-02	0.10986825	C1QTNF6	C1q and tumor necrosis factor related protein 6

230793_at	2.66E-02	-0.31105925	CARMIL1	capping protein regulator and myosin 1 linker 1
203635_at	2.66E-02	0.18230142	DSCR3	DSCR3 arrestin fold containing
43934_at	2.66E-02	0.06918709	GPR137	G protein-coupled receptor 137
213502_x_at	2.66E-02	-0.34646349	GUSBP11	glucuronidase, beta pseudogene 11
220777_at	2.66E-02	-0.31743539	KIF13A	kinesin family member 13A
1558101_at	2.66E-02	0.11711036	NFIA	nuclear factor I A
232086_at	2.66E-02	-0.10444769	PIK3C3	phosphatidylinositol 3-kinase catalytic subunit type 3
201070_x_at	2.66E-02	-0.25433962	SF3B1	splicing factor 3b subunit 1
235366_at	2.66E-02	-0.1928526	ZNF10	zinc finger protein 10
219376_at	2.66E-02	-0.22705051	ZNF322	zinc finger protein 322
1555973_at	2.66E-02	0.24906967		
227417_at	2.67E-02	-0.45646666	MTARC2	mitochondrial amidoxime reducing component 2
1552468_a_at	2.67E-02	0.07735662	DSCR10	Down syndrome critical region 10 (non-protein coding)
204010_s_at	2.67E-02	-0.14014953	KRAS	KRAS proto-oncogene, GTPase
207517_at	2.67E-02	0.12554553	LAMC2	laminin subunit gamma 2
1560156_at	2.67E-02	-0.27781548	LOC101928054	uncharacterized LOC101928054
202004_x_at	2.67E-02	0.17224326	SDHC	succinate dehydrogenase complex subunit C
1552293_at	2.67E-02	0.07305972	TMEM196	transmembrane protein 196
208909_at	2.67E-02	0.19108494	UQCRFS1	ubiquinol-cytochrome c reductase, Rieske iron-sulfur polypeptide 1
222059_at	2.67E-02	-0.07363583	ZNF335	zinc finger protein 335
217708_x_at	2.67E-02	-0.09428789		
232210_at	2.67E-02	-0.24515163		
214259_s_at	2.68E-02	-0.21389115	AKR7A2	aldo-keto reductase family 7 member A2
220525_s_at	2.68E-02	0.17823279	AUP1	ancient ubiquitous protein 1
223050_s_at	2.68E-02	0.22232381	FBXW5	F-box and WD repeat domain containing 5
232479_at	2.68E-02	0.10051128	FERMT1	fermitin family member 1
223926_at	2.68E-02	0.08264215	KIF2B	kinesin family member 2B
1556272_a_at	2.68E-02	-0.06154604	LOC101928896	uncharacterized LOC101928896
209861_s_at	2.68E-02	-0.19686636	METAP2	methionyl aminopeptidase 2
224874_at	2.68E-02	0.29696855	POLR1D	RNA polymerase I subunit D
208132_x_at	2.68E-02	0.14677628	PRRC2A	proline rich coiled-coil 2A
239538_at	2.68E-02	-0.242451	ZRANB3	zinc finger RANBP2-type containing 3
238146_at	2.68E-02	-0.26102302		
232198_at	2.68E-02	0.23434838		
239307_at	2.68E-02	-0.27114721		
212285_s_at	2.69E-02	0.19622197	AGRN	agrin
225969_at	2.69E-02	0.20129911	ALKBH6	alkB homolog 6
213503_x_at	2.69E-02	0.20970559	ANXA2	annexin A2
236449_at	2.69E-02	0.20082529	CSTB	cystatin B
217182_at	2.69E-02	-0.07930609	MUC5AC	mucin 5AC, oligomeric mucus/gel-forming
210617_at	2.69E-02	0.09600189	PHEX	phosphate regulating endopeptidase homolog, X-linked
219215_s_at	2.69E-02	0.35465729	SLC39A4	solute carrier family 39 member 4
222410_s_at	2.69E-02	-0.20522789	SNX6	sorting nexin 6
236010_at	2.69E-02	-0.26111401		
211691_x_at	2.69E-02	0.09181343		
241110_at	2.69E-02	-0.06154855		
237036_at	2.70E-02	0.08167404	FBXO10	F-box protein 10
243649_at	2.70E-02	-0.214375	FBXO7	F-box protein 7
235472_at	2.70E-02	-0.30802074	FUT10	fucosyltransferase 10
207764_s_at	2.70E-02	0.091712	HIPK3	homeodomain interacting protein kinase 3
1556754_at	2.70E-02	-0.07219228	LINC00856	long intergenic non-protein coding RNA 856
1556696_s_at	2.70E-02	-0.10346039	NR2F1-AS1	NR2F1 antisense RNA 1
212718_at	2.70E-02	-0.15966077	PAPOLA	poly(A) polymerase alpha
204262_s_at	2.70E-02	0.18873197	PSEN2	presenilin 2
231435_at	2.71E-02	-0.06395258	C7orf34	chromosome 7 open reading frame 34
201144_s_at	2.71E-02	-0.19996815	EIF2S1	eukaryotic translation initiation factor 2 subunit alpha
235124_at	2.71E-02	-0.21056319	EIF3J-AS1	EIF3J antisense RNA 1 (head to head)
1569048_s_at	2.71E-02	-0.09711603	LMF1	lipase maturation factor 1
205204_at	2.71E-02	0.16207276	NMB	neuromedin B
201454_s_at	2.71E-02	-0.19161196	NPEPPS	aminopeptidase puromycin sensitive
228225_at	2.71E-02	-0.19851678	PEX2	peroxisomal biogenesis factor 2

226604_at	2.71E-02	-0.25874045	TMTC3	transmembrane and tetratricopeptide repeat containing 3
230220_at	2.71E-02	-0.06013229	UNC80	unc-80 homolog, NALCN activator
1566499_at	2.71E-02	0.0769893		
200710_at	2.72E-02	0.31189663	ACADVL	acyl-CoA dehydrogenase, very long chain
209145_s_at	2.72E-02	0.10756144	CBFA2T2	CBFA2/RUNX1 translocation partner 2
231600_at	2.72E-02	-0.06517846	CLEC12B	C-type lectin domain family 12 member B
202977_s_at	2.72E-02	-0.08019727	CREBZF	CREB/ATF bZIP transcription factor
230389_at	2.72E-02	-0.30156868	FNBP1	formin binding protein 1
204395_s_at	2.72E-02	0.08489218	GRK5	G protein-coupled receptor kinase 5
241453_at	2.72E-02	-0.37066973	PTK2	protein tyrosine kinase 2
218677_at	2.72E-02	0.4098258	S100A14	S100 calcium binding protein A14
231891_at	2.72E-02	0.20760017	STAMBPL1	STAM binding protein like 1
225822_at	2.72E-02	0.24536738	TMEM125	transmembrane protein 125
213575_at	2.72E-02	0.15183568	TRA2A	transformer 2 alpha homolog
231147_at	2.72E-02	0.09524293		
234065_at	2.72E-02	-0.05230204		
229417_at	2.72E-02	-0.29845785		
208077_at	2.73E-02	-0.13867339	C9orf38	chromosome 9 open reading frame 38
241696_at	2.73E-02	-0.26632396	CNTLN	centlein
219216_at	2.73E-02	-0.17466034	ETAA1	Ewing tumor associated antigen 1
225094_at	2.73E-02	-0.15084522	KMT5A	lysine methyltransferase 5A
225165_at	2.73E-02	0.38432061	PPP1R1B	protein phosphatase 1 regulatory inhibitor subunit 1B
1556413_a_at	2.73E-02	0.06995785		
233954_at	2.74E-02	0.06119726	MFSD14B	major facilitator superfamily domain containing 14B
209885_at	2.74E-02	0.24648209	RHOD	ras homolog family member D
221010_s_at	2.74E-02	-0.10023923	SIRT5	sirtuin 5
238253_at	2.74E-02	-0.28988736	WDR72	WD repeat domain 72
215461_at	2.74E-02	0.0904636	ZNRF4	zinc and ring finger 4
236503_at	2.74E-02	-0.28423006		
1560370_x_at	2.75E-02	-0.07350275	ANKH	ANKH inorganic pyrophosphate transport regulator
1562209_at	2.75E-02	-0.07396709	DCAF4L1	DDB1 and CUL4 associated factor 4 like 1
218662_s_at	2.75E-02	-0.27765994	NCAPG	non-SMC condensin I complex subunit G
1564449_at	2.75E-02	0.06936685	USP2-AS1	USP2 antisense RNA 1 (head to head)
241883_x_at	2.75E-02	-0.07712102		
1559551_at	2.75E-02	0.08992407		
1562937_at	2.75E-02	0.07495054		
1553545_at	2.76E-02	0.0640983	ILDR1	immunoglobulin like domain containing receptor 1
1562116_at	2.76E-02	0.06541573	LINC01494	long intergenic non-protein coding RNA 1494
203208_s_at	2.76E-02	-0.22676262	MTFR1	mitochondrial fission regulator 1
238066_at	2.76E-02	-0.21514385	RBP7	retinol binding protein 7
1554643_at	2.76E-02	0.08452603	RGS11	regulator of G-protein signaling 11
208855_s_at	2.76E-02	0.15507305	STK24	serine/threonine kinase 24
209820_s_at	2.76E-02	0.23290881	TBL3	transducin beta like 3
212986_s_at	2.76E-02	-0.154278	TLK2	tousled like kinase 2
238542_at	2.76E-02	0.48057194	ULBP2	UL16 binding protein 2
240019_at	2.76E-02	-0.09908305		
1566862_at	2.76E-02	-0.05661761		
206227_at	2.77E-02	0.12712228	CILP	cartilage intermediate layer protein
228518_at	2.77E-02	-0.20847082	LOC102725426///MIR8071-2///MIR8071-1///IGHM///IGHG1	uncharacterized LOC102725426///microRNA 8071-2///microRNA 8071-1///immunoglobulin heavy constant mu///immunoglobulin heavy constant gamma 1 (G1m marker)
219332_at	2.77E-02	0.28342315	MICALL2	MICAL like 2
213973_at	2.77E-02	0.20331814	RRBP1	ribosome binding protein 1
220100_at	2.77E-02	-0.13748978	SLC22A11	solute carrier family 22 member 11
235580_at	2.77E-02	-0.28865086	ZNF141	zinc finger protein 141
240057_at	2.77E-02	0.08535699		
210143_at	2.78E-02	0.75122131	ANXA10	annexin A10
225485_at	2.78E-02	-0.2568643	CEP41	centrosomal protein 41
228106_at	2.78E-02	-0.21323285	DCAF16	DDB1 and CUL4 associated factor 16
224406_s_at	2.78E-02	-0.1575827	FCRL5	Fc receptor like 5
230342_at	2.78E-02	0.10178044	FOXK1	forkhead box K1

238581_at	2.78E-02	-0.30165447	GBP5	guanylate binding protein 5
209945_s_at	2.78E-02	0.17761989	GSK3B	glycogen synthase kinase 3 beta
219770_at	2.78E-02	-0.18787794	GTDC1	glycosyltransferase like domain containing 1
218290_at	2.78E-02	0.2165461	MIR6789///PLEKHJ1	microRNA 6789///pleckstrin homology domain containing J1
201058_s_at	2.78E-02	0.26786711	MYL9	myosin light chain 9
222541_at	2.78E-02	-0.20103948	RSF1	remodeling and spacing factor 1
209813_x_at	2.78E-02	-0.26714108	TARP///TRGV9///TRGC2	TCR gamma alternate reading frame protein///T cell receptor gamma variable 9///T cell receptor gamma constant 2
219920_s_at	2.79E-02	0.20565352	AMIGO3///GMPPB	adhesion molecule with Ig like domain 3///GDP-mannose pyrophosphorylase B
201895_at	2.79E-02	0.22398196	ARAF	A-Raf proto-oncogene, serine/threonine kinase
226378_s_at	2.79E-02	0.2147451	C19orf25	chromosome 19 open reading frame 25
206375_s_at	2.79E-02	-0.32066191	HSPB3	heat shock protein family B (small) member 3
1560207_at	2.79E-02	-0.06602746	MIR3610///RAD21-AS1	microRNA 3610///RAD21 antisense RNA 1
209718_at	2.79E-02	0.1061483	NCAPH2	non-SMC condensin II complex subunit H2
1559139_at	2.79E-02	0.12298804	NOC2L	NOC2 like nucleolar associated transcriptional repressor
224186_s_at	2.79E-02	0.18537264	RNF123	ring finger protein 123
216962_at	2.79E-02	-0.10461473	RPAIN	RPA interacting protein
213665_at	2.79E-02	-0.16414673	SOX4	SRY-box 4
219312_s_at	2.79E-02	-0.52496039	ZBTB10	zinc finger and BTB domain containing 10
1566835_at	2.79E-02	0.07552584		
1566870_at	2.79E-02	0.06064876		
211473_s_at	2.80E-02	0.06305708	COL4A6	collagen type IV alpha 6 chain
220198_s_at	2.80E-02	0.26898231	EIF5A2	eukaryotic translation initiation factor 5A2
1566129_at	2.80E-02	-0.09724664	LIMS1	LIM zinc finger domain containing 1
203102_s_at	2.80E-02	-0.21551138	MGAT2	mannosyl (alpha-1,6-)-glycoprotein beta-1,2-N-acetylglucosaminyltransferase
1555099_at	2.80E-02	0.07208789	MPP4	membrane palmitoylated protein 4
218708_at	2.80E-02	0.28665242	NXT1	nuclear transport factor 2 like export factor 1
203352_at	2.80E-02	-0.19211472	ORC4	origin recognition complex subunit 4
230321_at	2.80E-02	0.1396415	TOR1AIP2	torsin 1A interacting protein 2
215309_at	2.80E-02	-0.0516834	ZNF674	zinc finger protein 674
217331_at	2.80E-02	-0.08985913		
240205_x_at	2.80E-02	-0.14319929		
235044_at	2.81E-02	-0.21700545	CYYR1	cysteine and tyrosine rich 1
202668_at	2.81E-02	0.35635725	EFNB2	ephrin B2
208372_s_at	2.81E-02	0.08790246	LIMK1	LIM domain kinase 1
227763_at	2.81E-02	-0.1852471	LYPD6	LY6/PLAUR domain containing 6
205413_at	2.81E-02	-0.31799137	MPPED2	metallophosphoesterase domain containing 2
226147_s_at	2.81E-02	-1.14032525	PIGR	polymeric immunoglobulin receptor
209482_at	2.81E-02	0.18211068	POP7	POP7 homolog, ribonuclease P/MRP subunit
223103_at	2.81E-02	0.33836515	STARD10	StAR related lipid transfer domain containing 10
210314_x_at	2.81E-02	0.33220312	TNFSF13	tumor necrosis factor superfamily member 13
214897_at	2.81E-02	0.06375355		
243739_at	2.81E-02	-0.32628898		
214293_at	2.82E-02	-0.19109664	SEPT11	septin 11
1559532_at	2.82E-02	0.08227299	ARIH2OS	ariadne homolog 2 opposite strand
219068_x_at	2.82E-02	0.13065408	ATAD3B///ATAD3A	ATPase family, AAA domain containing 3B///ATPase family, AAA domain containing 3A
1554575_a_at	2.82E-02	0.20947814	BPNT1	3'(2'), 5'-bisphosphate nucleotidase 1
230101_at	2.82E-02	-0.05673295	CXCL2	C-X-C motif chemokine ligand 2
1561618_at	2.82E-02	-0.0820614	DPP10-AS3	DPP10 antisense RNA 3
210263_at	2.82E-02	0.09099832	KCNF1	potassium voltage-gated channel modifier subfamily F member 1
241945_at	2.82E-02	0.08785321	LOC101929681	uncharacterized LOC101929681
238448_at	2.82E-02	-0.19133098	MRPL19	mitochondrial ribosomal protein L19
234818_at	2.82E-02	0.08839715	TMEM108	transmembrane protein 108
222661_at	2.83E-02	-0.19673426	AGGF1	angiogenic factor with G-patch and FHA domains 1
1555833_a_at	2.83E-02	0.19953238	IRGQ	immunity related GTPase Q
207668_x_at	2.83E-02	0.22200751	PDIA6	protein disulfide isomerase family A member 6
217875_s_at	2.83E-02	0.34544684	PMEPA1	prostate transmembrane protein, androgen induced 1

234923_at	2.83E-02	-0.1319657	RALGAPA1	Ral GTPase activating protein catalytic alpha subunit 1
205804_s_at	2.83E-02	-0.14306636	TRAF3IP3	TRAF3 interacting protein 3
1564292_at	2.83E-02	0.05584965		
219190_s_at	2.84E-02	-0.15273959	AGO4	argonaute 4, RISC catalytic component
212201_at	2.84E-02	0.15777813	ANKLE2	ankyrin repeat and LEM domain containing 2
225490_at	2.84E-02	-0.16019565	ARID2	AT-rich interaction domain 2
235240_at	2.84E-02	-0.25064051	ATXN3	ataxin 3
202140_s_at	2.84E-02	0.16913618	CLK3	CDC like kinase 3
209132_s_at	2.84E-02	0.29363013	COMMD4	COMM domain containing 4
220434_at	2.84E-02	0.08671217	COQ8B	coenzyme Q8B
1557189_at	2.84E-02	0.11048747	DNASE1	deoxyribonuclease 1
227242_s_at	2.84E-02	0.06698369	EBF3	early B-cell factor 3
235278_at	2.84E-02	0.52165253	MACROD2	MACRO domain containing 2
241572_at	2.84E-02	-0.06467674	PDZD9	PDZ domain containing 9
217806_s_at	2.84E-02	0.21197308	POLDIP2	DNA polymerase delta interacting protein 2
209545_s_at	2.84E-02	-0.24623646	RIPK2	receptor interacting serine/threonine kinase 2
221285_at	2.84E-02	-0.08105365	ST8SIA2	ST8 alpha-N-acetyl-neuraminide alpha-2,8-sialyltransferase 2
242668_x_at	2.84E-02	0.10294802	SUN5	Sad1 and UNC84 domain containing 5
226483_at	2.84E-02	-0.26210095	TMEM68	transmembrane protein 68
208862_s_at	2.84E-02	0.205026	TMX2-CTNND1///CTNND1	TMX2-CTNND1 readthrough (NMD candidate)///catenin delta 1
240520_at	2.84E-02	-0.07970856		
203639_s_at	2.85E-02	0.2000564	FGFR2	fibroblast growth factor receptor 2
218280_x_at	2.85E-02	0.29802421	HIST2H2AA4///HIST2H2AA3	histone cluster 2, H2aa4///histone cluster 2, H2aa3
218636_s_at	2.85E-02	0.14021672	MAN1B1	mannosidase alpha class 1B member 1
207041_at	2.85E-02	0.06418162	MASP2	mannan binding lectin serine peptidase 2
240098_at	2.85E-02	0.08326129	RIF1	replication timing regulatory factor 1
44673_at	2.85E-02	0.09255838	SIGLEC1	sialic acid binding Ig like lectin 1
215833_s_at	2.85E-02	0.12352564	SPPL2B	signal peptide peptidase like 2B
232993_at	2.85E-02	0.05909755	SYNJ1	synaptojanin 1
226313_at	2.86E-02	0.18068572	C10orf35	chromosome 10 open reading frame 35
203376_at	2.86E-02	-0.19911962	CDC40	cell division cycle 40
201022_s_at	2.86E-02	0.18753465	DSTN	destrin, actin depolymerizing factor
217916_s_at	2.86E-02	-0.20367297	FAM49B	family with sequence similarity 49 member B
224865_at	2.86E-02	-0.26199625	FAR1	fatty acyl-CoA reductase 1
203851_at	2.86E-02	0.2080476	IGFBP6	insulin like growth factor binding protein 6
224020_at	2.86E-02	0.06527803	LINC00626	long intergenic non-protein coding RNA 626
1558011_at	2.86E-02	-0.07579975	LOC441081	POM121 membrane glycoprotein (rat) pseudogene
211083_s_at	2.86E-02	0.06516329	MAP3K13	mitogen-activated protein kinase kinase kinase 13
206637_at	2.86E-02	-0.41905569	P2RY14	purinergic receptor P2Y14
221292_at	2.86E-02	-0.06424386	PTCH2	patched 2
203458_at	2.86E-02	0.18161717	SPR	sepiapterin reductase (7,8-dihydrobiopterin:NADP+ oxidoreductase)
201679_at	2.86E-02	0.10760383	SRRT	serrate, RNA effector molecule
205177_at	2.86E-02	-0.07514787	TNNI1	troponin I1, slow skeletal type
208609_s_at	2.86E-02	0.07823373	TNXB///TNXA	tenascin XB///tenascin XA (pseudogene)
231536_at	2.86E-02	0.05350261		
225572_at	2.87E-02	-0.15854243	CREB1	cAMP responsive element binding protein 1
206619_at	2.87E-02	0.37759727	DKK4	dickkopf WNT signaling pathway inhibitor 4
226541_at	2.87E-02	-0.18032467	FBXO30	F-box protein 30
218847_at	2.87E-02	0.2249777	IGF2BP2	insulin like growth factor 2 mRNA binding protein 2
203055_s_at	2.87E-02	0.13964188	LOC100505585///ARHGEF1	uncharacterized LOC100505585///Rho guanine nucleotide exchange factor 1
223414_s_at	2.87E-02	-0.23431199	LYAR	Ly1 antibody reactive
210946_at	2.87E-02	-0.26211276	PLPP1	phospholipid phosphatase 1
226044_at	2.87E-02	-0.13525392	TDP1	tyrosyl-DNA phosphodiesterase 1
1566042_at	2.87E-02	0.05949915		
223024_at	2.88E-02	0.21814092	AP1M1	adaptor related protein complex 1 mu 1 subunit
227014_at	2.88E-02	0.39775225	ASPHD2	aspartate beta-hydroxylase domain containing 2
222103_at	2.88E-02	-0.20304544	ATF1	activating transcription factor 1
1564381_s_at	2.88E-02	-0.23044213	CEBPZOS	CEBPZ opposite strand
205985_x_at	2.88E-02	0.0855839	CLCNKB	chloride voltage-gated channel Kb

215377_at	2.88E-02	-0.10585467	CTBP2	C-terminal binding protein 2
203385_at	2.88E-02	0.21690965	DGKA	diacylglycerol kinase alpha
204095_s_at	2.88E-02	0.09474818	ELL	elongation factor for RNA polymerase II
244378_at	2.88E-02	-0.07102499	HECTD4	HECT domain E3 ubiquitin protein ligase 4
216935_at	2.88E-02	-0.06928685	LINC00302	long intergenic non-protein coding RNA 302
239315_at	2.88E-02	0.09527025	LOC101928422///TCAF2	TRPM8 channel-associated factor 2-like///TRPM8 channel associated factor 2
207957_s_at	2.88E-02	-0.20942092	PRKCB	protein kinase C beta
231851_at	2.88E-02	-0.22215376	RAVER2	ribonucleoprotein, PTB binding 2
223603_at	2.88E-02	0.07522633	RNF112	ring finger protein 112
201248_s_at	2.88E-02	0.1020991	SREBF2	sterol regulatory element binding transcription factor 2
208845_at	2.88E-02	-0.14779058	VDAC3	voltage dependent anion channel 3
242089_at	2.88E-02	-0.06611773		
206475_x_at	2.89E-02	0.06333734	GH1///CSHL1///CSH1	growth hormone 1///chorionic somatomammotropin hormone like 1///chorionic somatomammotropin hormone 1
40562_at	2.89E-02	0.15962067	GNA11	G protein subunit alpha 11
1559543_at	2.89E-02	0.04992061	LINC00441	long intergenic non-protein coding RNA 441
221560_at	2.89E-02	0.11391233	MARK4	microtubule affinity regulating kinase 4
212064_x_at	2.89E-02	0.14521183	MAZ	MYC associated zinc finger protein
203190_at	2.89E-02	0.22424502	MIR7113///MIR4691///NDUFS8	microRNA 7113///microRNA 4691///NADH:ubiquinone oxidoreductase core subunit S8
225238_at	2.89E-02	0.18342698	MSI2	musashi RNA binding protein 2
220741_s_at	2.89E-02	-0.23535141	PPA2	pyrophosphatase (inorganic) 2
1554163_at	2.89E-02	-0.0619008	TWIST2	twist family bHLH transcription factor 2
228636_at	2.90E-02	-0.3712572	BHLHE22	basic helix-loop-helix family member e22
235562_at	2.90E-02	0.29368205	C3orf70	chromosome 3 open reading frame 70
236335_at	2.90E-02	-0.42992798	GUCY1A2	guanylate cyclase 1 soluble subunit alpha 2
242694_at	2.90E-02	-0.06122819	IQSEC3	IQ motif and Sec7 domain 3
221690_s_at	2.90E-02	0.4802893	NLRP2	NLR family pyrin domain containing 2
215355_at	2.90E-02	-0.06927074	POU2F3	POU class 2 homeobox 3
221248_s_at	2.90E-02	-0.18921317	WHSC1L1	Wolf-Hirschhorn syndrome candidate 1-like 1
236437_at	2.90E-02	-0.27528784		
232759_at	2.90E-02	-0.11284321		
1570531_at	2.90E-02	-0.09178447		
216475_at	2.90E-02	0.08745134		
224235_at	2.90E-02	-0.06387343		
203864_s_at	2.91E-02	0.077602	ACTN2	actinin alpha 2
1557380_at	2.91E-02	0.07872219	AGAP11	ArfGAP with GTPase domain, ankyrin repeat and PH domain 11
228302_x_at	2.91E-02	0.30488088	CAMK2N1	calcium/calmodulin dependent protein kinase II inhibitor 1
203098_at	2.91E-02	-0.18626556	CDYL	chromodomain Y-like
233569_at	2.91E-02	-0.06333958	COPG2	coatomer protein complex subunit gamma 2
225971_at	2.91E-02	-0.25489574	DDHD1	DDHD domain containing 1
212490_at	2.91E-02	-0.17647816	DNAJC8	DnaJ heat shock protein family (Hsp40) member C8
211726_s_at	2.91E-02	0.08914471	FMO2	flavin containing monooxygenase 2
212626_x_at	2.91E-02	0.15846539	HNRNPC	heterogeneous nuclear ribonucleoprotein C (C1/C2)
202788_at	2.91E-02	0.19228125	MAPKAPK3	mitogen-activated protein kinase-activated protein kinase 3
242289_at	2.91E-02	-0.26934218	MRPL42	mitochondrial ribosomal protein L42
240349_at	2.91E-02	-0.28086727	PRKAA2	protein kinase AMP-activated catalytic subunit alpha 2
218714_at	2.91E-02	0.13303466	PRR14	proline rich 14
221298_s_at	2.91E-02	0.08940481	SLC22A8	solute carrier family 22 member 8
223455_at	2.91E-02	-0.15237706	TCHP	trichoplein keratin filament binding
213323_s_at	2.91E-02	0.11156924	ZC3H7B	zinc finger CCCH-type containing 7B
234151_at	2.91E-02	-0.18226622		
228177_at	2.92E-02	0.18527358	CREBBP	CREB binding protein
210872_x_at	2.92E-02	-0.10376902	GAS7	growth arrest specific 7
222268_x_at	2.92E-02	0.16063432	MUC5B	mucin 5B, oligomeric mucus/gel-forming
218359_at	2.92E-02	0.09624178	NRSN2	neurensin 2
207291_at	2.92E-02	0.14761827	PRRG4	proline rich and Gla domain 4
202873_at	2.93E-02	0.06336541	ATP6V1C1	ATPase H+ transporting V1 subunit C1

225119_at	2.93E-02	0.18622735	CHMP4B	charged multivesicular body protein 4B
235429_at	2.93E-02	-0.29207907	EIF3E	eukaryotic translation initiation factor 3 subunit E
1598_g_at	2.93E-02	0.2530609	GAS6	growth arrest specific 6
220053_at	2.93E-02	0.07571868	GDF3	growth differentiation factor 3
242670_at	2.93E-02	0.08200043	LGI4	leucine rich repeat LGI family member 4
241388_at	2.93E-02	-0.28575211	LOC101927451	uncharacterized LOC101927451
1567255_at	2.93E-02	0.11222138	OR10D1P	olfactory receptor family 10 subfamily D member 1 pseudogene
205273_s_at	2.93E-02	0.20465178	PITRM1	pitrilysin metallopeptidase 1
221859_at	2.93E-02	0.13764631	SYT13	synaptotagmin 13
240158_at	2.93E-02	0.06510084		
1559331_x_at	2.93E-02	-0.08091978		
236395_at	2.93E-02	-0.1515586		
1557704_a_at	2.94E-02	0.06972089	MARCHF7	membrane associated ring-CH-type finger 7
208353_x_at	2.94E-02	0.07855812	ANK1	ankyrin 1
1553389_at	2.94E-02	-0.071607	NPHP3	nephronophthisis 3 (adolescent)
206859_s_at	2.94E-02	0.07189488	PAEP	progestagen associated endometrial protein
229996_s_at	2.94E-02	-0.09165973	PCGF5	polycomb group ring finger 5
204280_at	2.94E-02	0.10213973	RGS14	regulator of G-protein signaling 14
244103_at	2.94E-02	0.15852784	SDE2	SDE2 telomere maintenance homolog
202667_s_at	2.94E-02	0.23064161	SLC39A7	solute carrier family 39 member 7
219793_at	2.94E-02	-0.33015811	SNX16	sorting nexin 16
231143_at	2.94E-02	0.07749008	TMEM178B	transmembrane protein 178B
223132_s_at	2.94E-02	0.20827784	TRIM8	tripartite motif containing 8
217582_at	2.94E-02	0.06023281		
234492_at	2.94E-02	-0.06371631		
222056_s_at	2.95E-02	-0.21070536	FAHD2A	fumarylacetoacetate hydrolase domain containing 2A
31826_at	2.95E-02	0.10746338	FKBP15	FK506 binding protein 15
203539_s_at	2.95E-02	0.08309749	GFAP	glial fibrillary acidic protein
210948_s_at	2.95E-02	0.15648953	LEF1	lymphoid enhancer binding factor 1
1555872_a_at	2.95E-02	-0.34887853	LOC728903///GLIDR	uncharacterized LOC728903///glioblastoma down-regulated RNA
206138_s_at	2.95E-02	0.15966514	PI4KB	phosphatidylinositol 4-kinase beta
1556199_a_at	2.95E-02	-0.07341017	RGS9BP	regulator of G-protein signaling 9 binding protein
216018_at	2.95E-02	0.07917687	RNF5	ring finger protein 5
230261_at	2.95E-02	-0.29943473	ST8SIA4	ST8 alpha-N-acetyl-neuraminide alpha-2,8-sialyltransferase 4
240542_at	2.95E-02	-0.11147862		
230659_at	2.95E-02	-0.28856943		
201990_s_at	2.96E-02	-0.26687877	CREBL2	cAMP responsive element binding protein like 2
228681_x_at	2.96E-02	0.06137972	DAPK3	death associated protein kinase 3
209783_at	2.96E-02	-0.09882941	DBP	D-box binding PAR bZIP transcription factor
222875_at	2.96E-02	-0.23650413	DHX33	DEAH-box helicase 33
200878_at	2.96E-02	0.22513558	EPAS1	endothelial PAS domain protein 1
244175_at	2.96E-02	0.07643776	LIN28B-AS1	LIN28B antisense RNA 1
222317_at	2.96E-02	-0.34595419	PDE3B	phosphodiesterase 3B
212628_at	2.96E-02	-0.19913374	PKN2	protein kinase N2
206625_at	2.96E-02	0.09218476	PRPH2	peripherin 2
210768_x_at	2.96E-02	0.15064591	TMCO1	transmembrane and coiled-coil domains 1
1555135_at	2.96E-02	-0.07484971		
243616_at	2.96E-02	-0.08157529		
241483_at	2.97E-02	-0.08850148	CLNK	cytokine dependent hematopoietic cell linker
208978_at	2.97E-02	0.12561059	CRIP2	cysteine rich protein 2
207609_s_at	2.97E-02	0.08670355	CYP1A2	cytochrome P450 family 1 subfamily A member 2
216897_s_at	2.97E-02	0.09658604	FAM76A	family with sequence similarity 76 member A
1556029_s_at	2.97E-02	-0.18757721	NMNAT2	nicotinamide nucleotide adenylyltransferase 2
218849_s_at	2.97E-02	0.22082068	PPP1R13L	protein phosphatase 1 regulatory subunit 13 like
228091_at	2.97E-02	-0.18648845	STX17	syntaxin 17
227790_at	2.97E-02	-0.19530839	UBE3D	ubiquitin protein ligase E3D
218645_at	2.97E-02	-0.26861418	ZNF277	zinc finger protein 277
242693_at	2.97E-02	-0.34459879		
237526_at	2.97E-02	-0.07473952		
235609_at	2.98E-02	-0.28112261	BRIP1	BRCA1 interacting protein C-terminal helicase 1

1558094_s_at	2.98E-02	0.10171422	CCDC174	coiled-coil domain containing 174
210916_s_at	2.98E-02	0.19301269	CD44	CD44 molecule (Indian blood group)
225216_at	2.98E-02	-0.24266956	FAM199X	family with sequence similarity 199, X-linked
214702_at	2.98E-02	0.1959427	FN1	fibronectin 1
209374_s_at	2.98E-02	-0.64936866	IGHM	immunoglobulin heavy constant mu
221660_at	2.98E-02	-0.12058983	MYL10	myosin light chain 10
233134_at	2.98E-02	0.06665843	RPH3AL	rabphilin 3A-like (without C2 domains)
1553923_at	2.98E-02	0.09042402	SLC22A24	solute carrier family 22 member 24
200031_s_at	2.98E-02	0.09518721	SNORD35B///RPS11	small nucleolar RNA, C/D box 35B///ribosomal protein S11
217774_s_at	2.98E-02	0.28246171	TRMT112	tRNA methyltransferase 11-2 homolog (S. cerevisiae)
243266_at	2.98E-02	0.08504907		
206807_s_at	2.99E-02	-0.07757677	ADD2	adducin 2
220023_at	2.99E-02	0.15493386	APOBR	apolipoprotein B receptor
225347_at	2.99E-02	0.13033622	ARL8A	ADP ribosylation factor like GTPase 8A
204693_at	2.99E-02	0.1162137	CDC42EP1	CDC42 effector protein 1
206914_at	2.99E-02	-0.16975047	CRTAM	cytotoxic and regulatory T-cell molecule
222070_at	2.99E-02	-0.09388987	DND1	DND microRNA-mediated repression inhibitor 1
228272_at	2.99E-02	0.27823047	DNLZ	DNL-type zinc finger
201937_s_at	2.99E-02	0.27776828	DNPEP	aspartyl aminopeptidase
214395_x_at	2.99E-02	-0.20207918	EEF1D	eukaryotic translation elongation factor 1 delta
64488_at	2.99E-02	0.14031466	IRGQ	immunity related GTPase Q
221997_s_at	2.99E-02	-0.15824409	MRPL52	mitochondrial ribosomal protein L52
226310_at	2.99E-02	-0.29437743	RICTOR	RPTOR independent companion of MTOR complex 2
220185_at	2.99E-02	-0.07132785	SPTBN4	spectrin beta, non-erythrocytic 4
1552798_a_at	2.99E-02	0.24155389	TLR4	toll like receptor 4
234133_s_at	2.99E-02	-0.09523533	TMEM116	transmembrane protein 116
210405_x_at	2.99E-02	0.17652274	TNFRSF10B	TNF receptor superfamily member 10b
237419_at	2.99E-02	-0.32332306		
239963_at	2.99E-02	-0.13632011		
222710_at	3.00E-02	0.11873834	AMIGO3///GMPPB	adhesion molecule with Ig like domain 3///GDP-mannose pyrophosphorylase B
1555197_a_at	3.00E-02	0.0721023	C21orf58	chromosome 21 open reading frame 58
203532_x_at	3.00E-02	-0.22375352	CUL5	cullin 5
242665_at	3.00E-02	-0.29613555	FMNL2	formin like 2
207550_at	3.00E-02	0.08698184	MPL	MPL proto-oncogene, thrombopoietin receptor
218716_x_at	3.00E-02	-0.15891185	MTO1	mitochondrial tRNA translation optimization 1
1555003_at	3.00E-02	-0.09568449	RBL1	RB transcriptional corepressor like 1
219154_at	3.00E-02	0.14488682	TMEM120B	transmembrane protein 120B
227345_at	3.00E-02	0.29449908	TNFRSF10D	TNF receptor superfamily member 10d
209114_at	3.00E-02	0.43191933	TSPAN1	tetraspanin 1
216616_at	3.00E-02	-0.06806603		
219608_s_at	3.01E-02	0.11945134	FBXO38	F-box protein 38
235130_at	3.01E-02	0.21331506	PANK2	pantothenate kinase 2
212100_s_at	3.01E-02	0.17783304	POLDIP3	DNA polymerase delta interacting protein 3
208102_s_at	3.01E-02	-0.08465549	PSD	pleckstrin and Sec7 domain containing
202101_s_at	3.01E-02	0.15119894	RALB	RALB Ras like proto-oncogene B
225499_at	3.01E-02	0.31367114	RALGAPA2	Ral GTPase activating protein catalytic alpha subunit 2
1557970_s_at	3.01E-02	0.08347903	RPS6KA2	ribosomal protein S6 kinase A2
222657_s_at	3.01E-02	-0.20079656	UBE2W	ubiquitin conjugating enzyme E2 W (putative)
213759_at	3.02E-02	0.06237799	ARL4C	ADP ribosylation factor like GTPase 4C
226259_at	3.02E-02	-0.19364018	EXOC6	exocyst complex component 6
232110_at	3.02E-02	0.16902104	GALNT5	polypeptide N-acetylgalactosaminyltransferase 5
1564697_a_at	3.02E-02	0.07540863	LINC01144	long intergenic non-protein coding RNA 1144
235383_at	3.02E-02	0.13348178	MYO7B	myosin VIIB
221810_at	3.02E-02	-0.2658357	RAB15	RAB15, member RAS oncogene family
238120_at	3.02E-02	0.12221106	RPH3AL	rabphilin 3A-like (without C2 domains)
231655_x_at	3.02E-02	0.04490874	SERPINB6	serpin family B member 6
203727_at	3.02E-02	0.14638244	SKIV2L	Ski2 like RNA helicase
217778_at	3.02E-02	0.14512952	SLC39A1	solute carrier family 39 member 1
240094_at	3.02E-02	0.22521029		
243671_at	3.02E-02	-0.19161508		
243341_at	3.02E-02	0.18279858		

204903_x_at	3.03E-02	0.18804198	ATG4B	autophagy related 4B cysteine peptidase
212674_s_at	3.03E-02	0.18794013	DHX30	DEAH-box helicase 30
203116_s_at	3.03E-02	-0.25139066	FECH	ferrochelatase
202269_x_at	3.03E-02	-0.3833729	GBP1	guanylate binding protein 1
1559427_at	3.03E-02	-0.07573691	MCF2L	MCF.2 cell line derived transforming sequence like
203557_s_at	3.03E-02	0.204245	PCBD1	pterin-4 alpha-carbinolamine dehydratase 1
207785_s_at	3.03E-02	-0.19829758	RBPJ	recombination signal binding protein for immunoglobulin kappa J region
1555434_a_at	3.03E-02	0.0856924	SLC39A14	solute carrier family 39 member 14
206649_s_at	3.03E-02	0.10963118	TFE3	transcription factor binding to IGHM enhancer 3
205400_at	3.03E-02	-0.08747689	WAS	Wiskott-Aldrich syndrome
234202_at	3.03E-02	-0.06078743		
1557236_at	3.04E-02	-0.23064615	APOL6	apolipoprotein L6
218185_s_at	3.04E-02	-0.22618982	ARMC1	armadillo repeat containing 1
1559249_at	3.04E-02	-0.21856056	ATXN1	ataxin 1
205425_at	3.04E-02	0.08012286	HIP1	huntingtin interacting protein 1
209225_x_at	3.04E-02	-0.11644519	TNPO1	transportin 1
228200_at	3.04E-02	-0.18798972	ZNF252P	zinc finger protein 252, pseudogene
215239_x_at	3.04E-02	-0.25397572	ZNF273	zinc finger protein 273
211742_s_at	3.05E-02	-0.39701439	EVI2B	ecotropic viral integration site 2B
1559535_s_at	3.05E-02	-0.24960368	LOC100996419	uncharacterized LOC100996419
211916_s_at	3.05E-02	0.30085587	MYO1A	myosin IA
1570542_a_at	3.05E-02	-0.18022558	PMPCB	peptidase, mitochondrial processing beta subunit
201607_at	3.05E-02	-0.18478276	PWP1	PWP1 homolog, endonuclein
200863_s_at	3.05E-02	0.15359144	RAB11A	RAB11A, member RAS oncogene family
1554807_a_at	3.05E-02	0.32543447	SPIRE1	spire type actin nucleation factor 1
233719_s_at	3.05E-02	0.17620089	TASP1	taspase 1
243065_at	3.05E-02	0.14467141		
200602_at	3.06E-02	0.15905133	APP	amyloid beta precursor protein
203388_at	3.06E-02	0.14234723	ARRB2	arrestin beta 2
231843_at	3.06E-02	-0.19795552	DDX55	DEAD-box helicase 55
231868_at	3.06E-02	-0.10990183	HOMEZ	homeobox and leucine zipper encoding
1569840_at	3.06E-02	-0.06975041	LOC101927798	uncharacterized LOC101927798
213984_at	3.06E-02	-0.30715865	PDS5A	PDS5 cohesin associated factor A
220952_s_at	3.06E-02	-0.25283713	PLEKHA5	pleckstrin homology domain containing A5
237706_at	3.06E-02	-0.17177066	STXBP4	syntaxin binding protein 4
233272_at	3.06E-02	0.08457661		
220451_s_at	3.07E-02	0.08027346	BIRC7	baculoviral IAP repeat containing 7
215349_at	3.07E-02	0.09278247	BTBD18	BTB domain containing 18
226984_at	3.07E-02	0.08852754	FGD5	FYVE, RhoGEF and PH domain containing 5
218620_s_at	3.07E-02	0.16136208	HEMK1	HemK methyltransferase family member 1
229382_at	3.07E-02	0.10614504	LOC101928718///FAM212B	uncharacterized LOC101928718///family with sequence similarity 212 member B
1554619_at	3.07E-02	-0.0828539	MBLAC2	metallo-beta-lactamase domain containing 2
235125_x_at	3.07E-02	-0.23918095	MIGA1	mitoguardin 1
220369_at	3.07E-02	-0.13799852	PPP4R3A	protein phosphatase 4 regulatory subunit 3A
236936_at	3.07E-02	-0.0949337		
1559152_at	3.07E-02	0.05293178		
228156_at	3.07E-02	0.38114371		
1557780_at	3.07E-02	-0.14770512		
200078_s_at	3.08E-02	0.25723286	ATP6V0B	ATPase H+ transporting V0 subunit b
241198_s_at	3.08E-02	0.13894998	C11orf70	chromosome 11 open reading frame 70
225694_at	3.08E-02	-0.26321083	CDK12	cyclin dependent kinase 12
235494_at	3.08E-02	0.15444354	LSAMP	limbic system-associated membrane protein
1568933_at	3.08E-02	-0.31641413	LYPD8	LY6/PLAUR domain containing 8
202670_at	3.08E-02	0.21406449	MAP2K1	mitogen-activated protein kinase kinase 1
242370_at	3.08E-02	-0.15107859	MTHFD2L	methylenetetrahydrofolate dehydrogenase (NADP+ dependent) 2-like
202944_at	3.08E-02	0.2224116	NAGA	alpha-N-acetylgalactosaminidase
210022_at	3.08E-02	0.11110599	PCGF1	polycomb group ring finger 1
219219_at	3.08E-02	0.20330591	TMEM160	transmembrane protein 160
230789_at	3.08E-02	-0.40731591	ZNF280B	zinc finger protein 280B
230571_at	3.08E-02	0.13764899		

242564_at	3.08E-02	0.14454327		
234129_at	3.09E-02	-0.08739947	ARHGEF12	Rho guanine nucleotide exchange factor 12
204392_at	3.09E-02	0.16019114	CAMK1	calcium/calmodulin dependent protein kinase I
237086_at	3.09E-02	-0.52186076	FOXA1	forkhead box A1
227829_at	3.09E-02	0.28127601	LARGE2	LARGE xylosyl- and glucuronyltransferase 2
220254_at	3.09E-02	-0.2004884	LRP12	LDL receptor related protein 12
1558484_s_at	3.09E-02	0.11143151	LRRC27	leucine rich repeat containing 27
215731_s_at	3.09E-02	-0.29258702	MPHOSPH9	M-phase phosphoprotein 9
218256_s_at	3.09E-02	-0.18833642	NUP54	nucleoporin 54
217343_at	3.09E-02	-0.05115011		
239458_at	3.10E-02	0.06696459	ALS2CR12	amyotrophic lateral sclerosis 2 chromosome region candidate 12
202246_s_at	3.10E-02	0.23503236	CDK4	cyclin dependent kinase 4
1552496_a_at	3.10E-02	0.1749872	COBL	cordon-bleu WH2 repeat protein
227103_s_at	3.10E-02	0.23834848	ECE2	endothelin converting enzyme 2
212666_at	3.10E-02	0.23407402	SMURF1	SMAD specific E3 ubiquitin protein ligase 1
224167_at	3.10E-02	-0.06336026	SPZ1	spermatogenic leucine zipper 1
222920_s_at	3.10E-02	0.08497221	TESPA1	thymocyte expressed, positive selection associated 1
226825_s_at	3.10E-02	0.25514948	TMEM165	transmembrane protein 165
242562_at	3.10E-02	-0.21871147		
205617_at	3.11E-02	0.10791781	PRRG2	proline rich and Gla domain 2
209999_x_at	3.11E-02	0.11103237	SOCS1	suppressor of cytokine signaling 1
224314_s_at	3.12E-02	0.2078654	EGLN1	egl-9 family hypoxia inducible factor 1
231647_s_at	3.12E-02	-0.25981692	FCRL5	Fc receptor like 5
212025_s_at	3.12E-02	0.21517156	FLII	FLII, actin remodeling protein
237747_at	3.12E-02	0.1164157	LOC100507281	uncharacterized LOC100507281
227927_at	3.12E-02	0.16374154	NBPF20	neuroblastoma breakpoint family member 20
212178_s_at	3.12E-02	0.19469675	POM121C	POM121 transmembrane nucleoporin C
1555618_s_at	3.12E-02	0.24503025	SAE1	SUMO1 activating enzyme subunit 1
212054_x_at	3.12E-02	0.11750446	TBC1D9B	TBC1 domain family member 9B
237562_at	3.12E-02	-0.05954767		
220390_at	3.13E-02	-0.29514374	AGBL2	ATP/GTP binding protein like 2
227438_at	3.13E-02	-0.19355147	ALPK1	alpha kinase 1
205083_at	3.13E-02	-0.31703572	AOX1	aldehyde oxidase 1
1559534_at	3.13E-02	-0.15274135	LOC100996419	uncharacterized LOC100996419
219860_at	3.13E-02	0.16830192	LY6G5C	lymphocyte antigen 6 complex, locus G5C
227601_at	3.13E-02	-0.16788264	METTL14	methyltransferase like 14
230434_at	3.13E-02	-0.25466291	PHOSPHO2	phosphatase, orphan 2
1554608_at	3.13E-02	0.27432697	TGOLN2	trans-golgi network protein 2
203683_s_at	3.13E-02	0.15364108	VEGFB	vascular endothelial growth factor B
207189_s_at	3.13E-02	0.08996162	ZZEF1	zinc finger ZZ-type and EF-hand domain containing 1
233239_at	3.13E-02	-0.15282249		
224429_x_at	3.13E-02	0.08981959		
1552582_at	3.14E-02	-0.19090469	ABCC13	ATP binding cassette subfamily C member 13 (pseudogene)
240586_at	3.14E-02	-0.05332782	ENAM	enamelin
241874_at	3.14E-02	0.13186831	IGIP	IgA inducing protein
203042_at	3.14E-02	-0.23080772	LAMP2	lysosomal associated membrane protein 2
1564639_at	3.14E-02	-0.11113771	LOC100291323	uncharacterized LOC100291323
229422_at	3.14E-02	-0.22170212	NRDC	nardilysin convertase
221657_s_at	3.15E-02	0.14507919	ASB6	ankyrin repeat and SOCS box containing 6
240020_at	3.15E-02	-0.11173542	CYB5R4	cytochrome b5 reductase 4
224592_x_at	3.15E-02	-0.15862815	HP1BP3	heterochromatin protein 1 binding protein 3
217710_x_at	3.15E-02	-0.07136667	ITPK1	inositol-tetrakisphosphate 1-kinase
226797_at	3.15E-02	-0.2479118	MBTD1	mbt domain containing 1
1554495_s_at	3.15E-02	0.07049295	MTHFSD	methenyltetrahydrofolate synthetase domain containing
207634_at	3.15E-02	0.06741047	PDCD1	programmed cell death 1
218396_at	3.15E-02	-0.21713591	VPS13C	vacuolar protein sorting 13 homolog C
243993_at	3.15E-02	-0.30087477		
241591_at	3.15E-02	0.06295619		
243181_at	3.16E-02	-0.22197763	ANKIB1	ankyrin repeat and IBR domain containing 1
217207_s_at	3.16E-02	0.177609	BTNL3	butyrophilin like 3
223398_at	3.16E-02	0.15911223	CARD19	caspase recruitment domain family member 19

237647_at	3.16E-02	-0.07179234	GHRL	ghrelin/obestatin prepropeptide
220532_s_at	3.16E-02	0.44604521	TMEM176B	transmembrane protein 176B
230933_at	3.17E-02	0.24000076	DSTN	destrin, actin depolymerizing factor
224798_s_at	3.17E-02	0.1417741	FAM219B	family with sequence similarity 219 member B
224826_at	3.17E-02	0.32186142	GPCPD1	glycerophosphocholine phosphodiesterase 1
237324_s_at	3.17E-02	0.17330732	HKDC1	hexokinase domain containing 1
206583_at	3.17E-02	-0.2559381	KRBOX4	KRAB box domain containing 4
231040_at	3.17E-02	-0.06895117	RORB	RAR related orphan receptor B
228485_s_at	3.17E-02	0.22663188	SLC44A1	solute carrier family 44 member 1
244439_at	3.17E-02	0.13394402	SPRED1	sprouty related EVH1 domain containing 1
212382_at	3.17E-02	-0.3274652	TCF4	transcription factor 4
218478_s_at	3.17E-02	-0.14107815	ZCCHC8	zinc finger CCHC-type containing 8
241053_at	3.17E-02	0.07978524		
234789_at	3.17E-02	-0.04461544		
1554260_a_at	3.18E-02	-0.30969557	FRYL	FRY like transcription coactivator
201678_s_at	3.18E-02	0.17305927	HMCES	5-hydroxymethylcytosine (hmC) binding, ES cell-specific
211926_s_at	3.18E-02	0.20337875	MYH9	myosin, heavy chain 9, non-muscle
230219_at	3.18E-02	0.18285367	NDE1	nudE neurodevelopment protein 1
230784_at	3.18E-02	-1.11136442	PRAC1	prostate cancer susceptibility candidate 1
205087_at	3.18E-02	-0.20864835	RWDD3	RWD domain containing 3
236265_at	3.18E-02	-0.2231704	SP4	Sp4 transcription factor
220240_s_at	3.18E-02	0.30634074	TMCO3	transmembrane and coiled-coil domains 3
232440_at	3.18E-02	-0.20114797	ZDHHC13	zinc finger DHHC-type containing 13
207417_s_at	3.18E-02	-0.33316503	ZNF559-ZNF177///ZNF177	ZNF559-ZNF177 readthrough///zinc finger protein 177
212351_at	3.19E-02	0.12548438	EIF2B5	eukaryotic translation initiation factor 2B subunit epsilon
203822_s_at	3.19E-02	-0.1664198	ELF2	E74 like ETS transcription factor 2
229645_at	3.19E-02	0.1163408	FAM69C	family with sequence similarity 69 member C
1569153_at	3.19E-02	0.10140765	GRAMD4	GRAM domain containing 4
222916_s_at	3.19E-02	0.17824517	HDLBP	high density lipoprotein binding protein
218450_at	3.19E-02	-0.25897186	HEBP1	heme binding protein 1
214639_s_at	3.19E-02	0.45325318	HOXA1	homeobox A1
211532_x_at	3.19E-02	-0.08554845	KIR2DS2	killer cell immunoglobulin like receptor, two Ig domains and short cytoplasmic tail 2
201490_s_at	3.19E-02	0.29312117	PPIF	peptidylprolyl isomerase F
225203_at	3.19E-02	0.26603562	PPP1R16A	protein phosphatase 1 regulatory subunit 16A
231541_s_at	3.19E-02	0.07154577	SPAG5-AS1	SPAG5 antisense RNA 1
201314_at	3.19E-02	0.21088663	STK25	serine/threonine kinase 25
233403_x_at	3.19E-02	0.11321632	TM6SF2	transmembrane 6 superfamily member 2
218382_s_at	3.19E-02	0.14136559	U2AF2	U2 small nuclear RNA auxiliary factor 2
1562976_at	3.19E-02	-0.08093383		
240038_at	3.19E-02	-0.28880896		
1563692_at	3.19E-02	-0.40113109		
242486_at	3.19E-02	0.14564957		
225553_at	3.20E-02	-0.21037674	CNIH1	cornichon family AMPA receptor auxiliary protein 1
218854_at	3.20E-02	-0.30977826	DSE	dermatan sulfate epimerase
221980_at	3.20E-02	0.06557515	EMILIN2	elastin microfibril interfacer 2
239346_at	3.20E-02	-0.24507419	GTF2H3	general transcription factor IIH subunit 3
228227_at	3.20E-02	0.09572462	ITGB1BP1	integrin subunit beta 1 binding protein 1
206802_at	3.20E-02	-0.07284325	PAX5	paired box 5
206347_at	3.20E-02	-0.19107297	PDK3	pyruvate dehydrogenase kinase 3
202928_s_at	3.20E-02	0.09578644	PHF1	PHD finger protein 1
1564591_a_at	3.20E-02	0.07535217	TMC1	transmembrane channel like 1
217949_s_at	3.20E-02	0.25462446	VKORC1	vitamin K epoxide reductase complex subunit 1
243558_at	3.20E-02	-0.07224174		
1560492_at	3.20E-02	-0.09657493		
230602_at	3.21E-02	0.26652748	ACMSD	aminocarboxymuconate semialdehyde decarboxylase
1562836_at	3.21E-02	-0.18157797	DDX6	DEAD-box helicase 6
1554903_at	3.21E-02	0.11704162	FRMD8	FERM domain containing 8
215974_at	3.21E-02	-0.08269539	HCG4B	HLA complex group 4B (non-protein coding)
241955_at	3.21E-02	-0.31494319	HECTD1	HECT domain E3 ubiquitin protein ligase 1
205426_s_at	3.21E-02	0.0703232	HIP1	huntingtin interacting protein 1
235753_at	3.21E-02	0.49700842	HOXA7	homeobox A7

228798_x_at	3.21E-02	-0.08385007	LOC100289283	uncharacterized LOC100289283
237842_at	3.21E-02	-0.06306995	MAP7D3	MAP7 domain containing 3
225896_at	3.21E-02	0.11793703	MPRIP	myosin phosphatase Rho interacting protein
217794_at	3.21E-02	0.13462919	PRR13	proline rich 13
206872_at	3.21E-02	-0.08214573	SLC17A1	solute carrier family 17 member 1
216504_s_at	3.21E-02	-0.3218357	SLC39A8	solute carrier family 39 member 8
220650_s_at	3.21E-02	0.07079133	SLC9A5	solute carrier family 9 member A5
1553657_at	3.21E-02	-0.1702602	VWA3A	von Willebrand factor A domain containing 3A
243300_at	3.21E-02	-0.13291677		
217344_at	3.21E-02	0.13321753		
206888_s_at	3.22E-02	0.14952581	ARHGDIG	Rho GDP dissociation inhibitor gamma
242447_at	3.22E-02	0.54919562	C3orf70	chromosome 3 open reading frame 70
1557756_a_at	3.22E-02	-0.17985674	CEP128	centrosomal protein 128
227871_at	3.22E-02	-0.23054083	CHM	CHM, Rab escort protein 1
218152_at	3.22E-02	0.18272268	HMG20A	high mobility group 20A
1555697_at	3.22E-02	0.08971745	KLK4	kallikrein related peptidase 4
201527_at	3.22E-02	0.1688819	LOC101927180///ATP6V1F	V-type proton ATPase subunit F pseudogene///ATPase H+ transporting V1 subunit F
217668_at	3.22E-02	-0.08038464	LRRC75B	leucine rich repeat containing 75B
206118_at	3.22E-02	-0.22027874	STAT4	signal transducer and activator of transcription 4
231912_s_at	3.22E-02	0.13820857	TECPR1	tectonin beta-propeller repeat containing 1
230647_at	3.22E-02	0.16584148	TMEM53	transmembrane protein 53
227174_at	3.22E-02	-1.04740762	WDR72	WD repeat domain 72
212419_at	3.22E-02	0.39233268	ZCCHC24	zinc finger CCHC-type containing 24
225338_at	3.22E-02	-0.19258322	ZYG11B	zyg-11 family member B, cell cycle regulator
234378_at	3.22E-02	0.05834225		
243132_at	3.23E-02	0.06565823	APTX	aprataxin
220815_at	3.23E-02	-0.08411966	CTNNA3	catenin alpha 3
220724_at	3.23E-02	-0.42952238	CWH43	cell wall biogenesis 43 C-terminal homolog
219118_at	3.23E-02	0.3444753	FKBP11	FK506 binding protein 11
232237_at	3.23E-02	0.08297522	MDGA1	MAM domain containing glycosylphosphatidylinositol anchor 1
229894_s_at	3.23E-02	-0.12669392	RAB43	RAB43, member RAS oncogene family
214368_at	3.23E-02	-0.11237382	RASGRP2	RAS guanyl releasing protein 2
1552999_a_at	3.23E-02	0.10572304	WFDC10B	WAP four-disulfide core domain 10B
236331_at	3.24E-02	-0.20455726	CDKL2	cyclin dependent kinase like 2
236730_at	3.24E-02	0.08187809	GIPC3	GIPC PDZ domain containing family member 3
213013_at	3.24E-02	0.07778139	LOC644172///MAPK8IP1	mitogen-activated protein kinase 8 interacting protein 1 pseudogene///mitogen-activated protein kinase 8 interacting protein 1
205163_at	3.24E-02	-0.08126102	MYLPF	myosin light chain, phosphorylatable, fast skeletal muscle
229342_at	3.24E-02	-0.22612725	NCBP3	nuclear cap binding subunit 3
203879_at	3.24E-02	-0.17732123	PIK3CD	phosphatidylinositol-4,5-bisphosphate 3-kinase catalytic subunit delta
229590_at	3.24E-02	-0.21168224	SNORD68///RPL13	small nucleolar RNA, C/D box 68///ribosomal protein L13
230759_at	3.24E-02	-0.21065395	SNX14	sorting nexin 14
233225_at	3.24E-02	-0.20895385		
244679_at	3.24E-02	-0.26942769		
1559028_at	3.24E-02	0.07684001		
223874_at	3.25E-02	-0.31627849	ACTR3C	ARP3 actin-related protein 3 homolog C
235007_at	3.25E-02	-0.30224271	BBS7	Bardet-Biedl syndrome 7
219856_at	3.25E-02	0.19270187	C1orf116	chromosome 1 open reading frame 116
45633_at	3.25E-02	-0.22915257	GINS3	GINS complex subunit 3
210045_at	3.25E-02	0.233459	IDH2	isocitrate dehydrogenase (NADP(+)) 2, mitochondrial
236611_at	3.25E-02	0.07220855	LOC101928000	uncharacterized LOC101928000
1554914_at	3.25E-02	-0.05873946	PLA2G4D	phospholipase A2 group IVD
1554539_a_at	3.25E-02	0.2274062	RHOF	ras homolog family member F, filopodia associated
1562194_at	3.25E-02	-0.13460185		
235785_at	3.25E-02	-0.19608837		
1565616_at	3.25E-02	0.07553129		
202597_at	3.26E-02	0.202077	IRF6	interferon regulatory factor 6

217475_s_at	3.26E-02	0.0856606	LIMK2	LIM domain kinase 2
220744_s_at	3.26E-02	0.21513669	LOC101927266///IFT122	intraflagellar transport protein 122 homolog///intraflagellar transport 122
214667_s_at	3.26E-02	0.10047107	TP53I11	tumor protein p53 inducible protein 11
231180_at	3.26E-02	-0.66622116		
1561158_at	3.26E-02	0.07022934		
219590_x_at	3.27E-02	-0.16491581	DPH5	diphthamide biosynthesis 5
241172_at	3.27E-02	0.05229753	GRIA2	glutamate ionotropic receptor AMPA type subunit 2
220577_at	3.27E-02	-0.17984922	GVINP1	GTPase, very large interferon inducible pseudogene 1
1554819_a_at	3.27E-02	0.05842963	ITGA11	integrin subunit alpha 11
200874_s_at	3.27E-02	0.19862098	MIR1292///SNORD110///SNORD86///SNORD57///NOP56	microRNA 1292///small nucleolar RNA, C/D box 110///small nucleolar RNA, C/D box 86///small nucleolar RNA, C/D box 57///NOP56 ribonucleoprotein
242512_at	3.27E-02	-0.11010942	MYO9A	myosin IXA
59697_at	3.27E-02	-0.2662221	RAB15	RAB15, member RAS oncogene family
215918_s_at	3.27E-02	0.18713198	SPTBN1	spectrin beta, non-erythrocytic 1
222188_at	3.27E-02	-0.0547661	TRMO	tRNA methyltransferase O
234337_at	3.27E-02	-0.08268893	TTLL9	tubulin tyrosine ligase like 9
212041_at	3.28E-02	0.2515216	ATP6V0D1	ATPase H+ transporting V0 subunit d1
206621_s_at	3.28E-02	0.18612148	EIF4H	eukaryotic translation initiation factor 4H
212785_s_at	3.28E-02	-0.19968832	LARP7	La ribonucleoprotein domain family member 7
220305_at	3.28E-02	0.28818289	MAVS	mitochondrial antiviral signaling protein
223343_at	3.28E-02	-0.41384647	MS4A7	membrane spanning 4-domains A7
202440_s_at	3.28E-02	0.21654866	ST5	suppression of tumorigenicity 5
201576_s_at	3.28E-02	0.21916318	TMPPE///GLB1	transmembrane protein with metallophosphoesterase domain///galactosidase beta 1
213193_x_at	3.28E-02	-0.27020981	TRBC1	T cell receptor beta constant 1
232189_at	3.28E-02	0.06946321		
237483_at	3.28E-02	-0.29346402		
1561670_at	3.28E-02	-0.06952655		
205423_at	3.29E-02	0.12230304	AP1B1	adaptor related protein complex 1 beta 1 subunit
240982_at	3.29E-02	0.06402234	CARS	cysteinyl-tRNA synthetase
237046_x_at	3.29E-02	-0.08795448	IL34	interleukin 34
218907_s_at	3.29E-02	0.11870229	LRRC61	leucine rich repeat containing 61
226441_at	3.29E-02	-0.24514492	MAP3K2	mitogen-activated protein kinase kinase kinase 2
219024_at	3.29E-02	0.17214086	PLEKHA1	pleckstrin homology domain containing A1
207771_at	3.29E-02	0.07573242	SLC5A2	solute carrier family 5 member 2
235520_at	3.29E-02	-0.2581547	ZNF280C	zinc finger protein 280C
237951_at	3.29E-02	-0.08206623		
233300_at	3.29E-02	-0.26176567		
206993_at	3.30E-02	-0.23166803	ATP5S	ATP synthase, H+ transporting, mitochondrial Fo complex subunit s (factor B)
1561280_at	3.30E-02	-0.04299246	LEMD1-AS1	LEMD1 antisense RNA 1
209348_s_at	3.30E-02	-0.31603867	MAF	MAF bZIP transcription factor
203553_s_at	3.30E-02	-0.22193454	MAP4K5	mitogen-activated protein kinase kinase kinase kinase 5
206333_at	3.30E-02	-0.07929247	MSI1	musashi RNA binding protein 1
212879_x_at	3.30E-02	0.11407469	PIAS4	protein inhibitor of activated STAT 4
222467_s_at	3.30E-02	-0.18838479	PPP6R3	protein phosphatase 6 regulatory subunit 3
206179_s_at	3.30E-02	-0.08037759	TPPP	tubulin polymerization promoting protein
244187_at	3.30E-02	-0.43935262		
1557056_at	3.31E-02	0.09875473	C5orf47	chromosome 5 open reading frame 47
219561_at	3.31E-02	0.10202344	COPZ2	coatomer protein complex subunit zeta 2
233198_at	3.31E-02	-0.2393906	GOLGA2P5	golgin A2 pseudogene 5
208990_s_at	3.31E-02	-0.15520708	HNRNPH3	heterogeneous nuclear ribonucleoprotein H3
229714_at	3.31E-02	-0.07196192	HS6ST3	heparan sulfate 6-O-sulfotransferase 3
220413_at	3.31E-02	-0.30463905	SLC39A2	solute carrier family 39 member 2
225339_at	3.31E-02	-0.25034086	SPAG9	sperm associated antigen 9
208194_s_at	3.31E-02	0.0983388	STAM2	signal transducing adaptor molecule 2
243550_at	3.31E-02	-0.14955213	ZDHHC21	zinc finger DHHC-type containing 21
223639_s_at	3.31E-02	0.24133783	ZNRD1	zinc ribbon domain containing 1
244541_x_at	3.31E-02	-0.07165223		
240694_at	3.31E-02	-0.05752978		
217007_s_at	3.32E-02	0.11384842	ADAM15	ADAM metallopeptidase domain 15

215684_s_at	3.32E-02	0.23915573	ASCC2	activating signal cointegrator 1 complex subunit 2
218260_at	3.32E-02	0.16629752	DDA1	DET1 and DDB1 associated 1
238807_at	3.32E-02	-0.24612326	GAPDHP62///ANKRD46	glyceraldehyde 3 phosphate dehydrogenase pseudogene 62///ankyrin repeat domain 46
229367_s_at	3.32E-02	-0.20437672	GIMAP6	GTPase, IMAP family member 6
1558984_at	3.32E-02	0.10439963	MAP3K11	mitogen-activated protein kinase kinase kinase 11
202905_x_at	3.32E-02	-0.29446795	NBN	nibrin
205760_s_at	3.32E-02	0.20515921	OGG1	8-oxoguanine DNA glycosylase
214493_s_at	3.32E-02	0.12080561	PATJ	PATJ, crumbs cell polarity complex component
211811_s_at	3.32E-02	0.08988516	PCDHA5///PCDHA6	protocadherin alpha 5///protocadherin alpha 6
201267_s_at	3.32E-02	0.21622134	PSMC3	proteasome 26S subunit, ATPase 3
202914_s_at	3.33E-02	0.07380854	ARHGEF11	Rho guanine nucleotide exchange factor 11
1563559_at	3.33E-02	0.07372691	FMN1	formin 1
221688_s_at	3.33E-02	0.24758962	IMP3	IMP3, U3 small nucleolar ribonucleoprotein
217929_s_at	3.33E-02	0.12822911	KIAA0319L	KIAA0319 like
202240_at	3.33E-02	0.22313662	PLK1	polo like kinase 1
204958_at	3.33E-02	0.17697125	PLK3	polo like kinase 3
234198_at	3.33E-02	-0.08949813	PROM2	prominin 2
223567_at	3.33E-02	-0.05997055	SEMA6B	semaphorin 6B
218565_at	3.33E-02	0.17409442	SPOUT1	SPOUT domain containing methyltransferase 1
242566_at	3.33E-02	0.07594125	VASH1	vasohibin 1
224265_s_at	3.33E-02	0.08776586	ZAN	zonadhesin (gene/pseudogene)
229766_at	3.33E-02	-0.170331	ZNF445	zinc finger protein 445
1564656_at	3.33E-02	-0.11159709		
216132_at	3.33E-02	0.07441049		
215051_x_at	3.34E-02	-0.20823364	AIF1	allograft inflammatory factor 1
201949_x_at	3.34E-02	0.15572034	CAPZB	capping actin protein of muscle Z-line beta subunit
213607_x_at	3.34E-02	0.13626257	NADK	NAD kinase
223285_s_at	3.34E-02	0.11046264	ST6GALNAC4	ST6 N-acetylgalactosaminide alpha-2,6-sialyltransferase 4
237469_at	3.34E-02	-0.1778484	TOP2A	topoisomerase (DNA) II alpha
214915_at	3.34E-02	-0.06318711	ZNF362	zinc finger protein 362
241798_at	3.34E-02	-0.15133439		
232517_s_at	3.35E-02	0.1064731	HELZ2	helicase with zinc finger 2
216552_x_at	3.35E-02	0.06943279	KIR2DS4	killer cell immunoglobulin like receptor, two Ig domains and short cytoplasmic tail 4
232736_s_at	3.35E-02	0.07504545	POM121L10P///POM121L8P///POM121L9P	POM121 transmembrane nucleoporin like 10, pseudogene///POM121 transmembrane nucleoporin like 8, pseudogene///POM121 transmembrane nucleoporin like 9, pseudogene
218764_at	3.35E-02	-0.29968301	PRKCH	protein kinase C eta
202895_s_at	3.35E-02	0.08264386	SIRPA	signal regulatory protein alpha
221914_at	3.35E-02	0.1031589	SYN1	synapsin I
215411_s_at	3.35E-02	0.20099242	TRAF3IP2	TRAF3 interacting protein 2
235142_at	3.35E-02	-0.27041641	ZBTB8A	zinc finger and BTB domain containing 8A
91682_at	3.35E-02	0.08393729		
1557709_at	3.35E-02	0.07834566		
223801_s_at	3.36E-02	-0.08295835	APOL4	apolipoprotein L4
206044_s_at	3.36E-02	-0.13427901	KIAA1549///BRAF	KIAA1549///B-Raf proto-oncogene, serine/threonine kinase
240573_at	3.36E-02	-0.07683058	LOC374443	C-type lectin domain family 2 member D pseudogene
1569652_at	3.36E-02	-0.32006134	MLLT3	MLLT3, super elongation complex subunit
204073_s_at	3.36E-02	0.44978422	MYRF	myelin regulatory factor
231909_x_at	3.36E-02	-0.21005963	ODF2L	outer dense fiber of sperm tails 2 like
222711_s_at	3.36E-02	0.07733577	RHBDF1	rhomboid 5 homolog 1
222700_at	3.37E-02	-0.1536786	ATL2	atlastin GTPase 2
210208_x_at	3.37E-02	0.21072826	BAG6	BCL2 associated athanogene 6
210142_x_at	3.37E-02	0.17957818	FLOT1	flotillin 1
244111_at	3.37E-02	-0.05701698	KRT222	keratin 222
221772_s_at	3.37E-02	0.1490325	PPP2R2D	protein phosphatase 2 regulatory subunit Bdelta
212366_at	3.37E-02	-0.25138047	ZNF292	zinc finger protein 292
200793_s_at	3.38E-02	0.28776305	ACO2	aconitase 2
205197_s_at	3.38E-02	-0.33979183	ATP7A	ATPase copper transporting alpha

221585_at	3.38E-02	0.085906	CACNG4	calcium voltage-gated channel auxiliary subunit gamma 4
205545_x_at	3.38E-02	-0.1510387	DNAJC8	DnaJ heat shock protein family (Hsp40) member C8
224320_s_at	3.38E-02	0.33049735	MCM8	minichromosome maintenance 8 homologous recombination repair factor
228528_at	3.38E-02	0.38517991	MIR29C///MIR29B2	microRNA 29c///microRNA 29b-2
214997_at	3.38E-02	-0.12365453	SCAI	suppressor of cancer cell invasion
220770_s_at	3.38E-02	-0.30667384	ZBED8	zinc finger BED-type containing 8
203651_at	3.38E-02	-0.23295107	ZFYVE16	zinc finger FYVE-type containing 16
231296_at	3.38E-02	0.18957972		
1563054_at	3.38E-02	-0.05317943		
213106_at	3.39E-02	-0.4383803	ATP8A1	ATPase phospholipid transporting 8A1
1555229_a_at	3.39E-02	0.0908805	C1S	complement component 1, s subcomponent
212817_at	3.39E-02	-0.07201275	DNAJB5	DnaJ heat shock protein family (Hsp40) member B5
226967_at	3.39E-02	0.12081902	FIZ1	FLT3 interacting zinc finger 1
229377_at	3.39E-02	0.29954079	GRTP1	growth hormone regulated TBC protein 1
1554376_s_at	3.39E-02	0.09973688	HACD1	3-hydroxyacyl-CoA dehydratase 1
1569607_s_at	3.39E-02	-0.35417234	LOC102725051///LOC102723891///ANKRD20A4///ANKRD20A2///ANKRD20A3///ANKRD20A5P///ANKRD20A11P///ANKRD20A9P///ANKRD20A1	uncharacterized LOC102725051///ankyrin repeat domain-containing protein 20B-like///ankyrin repeat domain 20 family member A4///ankyrin repeat domain 20 family member A2///ankyrin repeat domain 20 family member A3///ankyrin repeat domain 20 family member A5, pseudogene///ankyrin repeat domain 20 family member A11, pseudogene///ankyrin repeat domain 20 family member A9, pseudogene///ankyrin repeat domain 20 family member A1
201383_s_at	3.39E-02	0.1716121	NBR1	NBR1, autophagy cargo receptor
216321_s_at	3.39E-02	-0.40104793	NR3C1	nuclear receptor subfamily 3 group C member 1
212636_at	3.39E-02	-0.35721853	QKI	QKI, KH domain containing RNA binding
205647_at	3.39E-02	-0.25831766	RAD52	RAD52 homolog, DNA repair protein
231068_at	3.39E-02	0.06871806	SLC47A2	solute carrier family 47 member 2
206528_at	3.39E-02	-0.13041855	TRPC6	transient receptor potential cation channel subfamily C member 6
243827_at	3.39E-02	-0.1742468		
205389_s_at	3.40E-02	-0.0690931	ANK1	ankyrin 1
238625_at	3.40E-02	-0.43800386	C1orf168	chromosome 1 open reading frame 168
217736_s_at	3.40E-02	0.23757097	EIF2AK1	eukaryotic translation initiation factor 2 alpha kinase 1
212483_at	3.40E-02	-0.205341	NIPBL	NIPBL, cohesin loading factor
242922_at	3.40E-02	0.17267126	NOMO3	NODAL modulator 3
221689_s_at	3.40E-02	-0.16332978	PIGP	phosphatidylinositol glycan anchor biosynthesis class P
1552670_a_at	3.40E-02	0.08800437	PPP1R3B	protein phosphatase 1 regulatory subunit 3B
218124_at	3.40E-02	0.22700445	RETSAT	retinol saturase
226492_at	3.40E-02	-0.55750504	SEMA6D	semaphorin 6D
218797_s_at	3.40E-02	0.16530481	SIRT7	sirtuin 7
242474_s_at	3.40E-02	-0.08506928	VMA21	VMA21 vacuolar H+-ATPase homolog (S. cerevisiae)
242255_at	3.40E-02	0.27716977	WDR37	WD repeat domain 37
241264_at	3.40E-02	-0.06163164		
1561692_at	3.40E-02	-0.0622166		
203722_at	3.41E-02	0.21905191	ALDH4A1	aldehyde dehydrogenase 4 family member A1
210930_s_at	3.41E-02	0.10273323	ERBB2	erb-b2 receptor tyrosine kinase 2
210112_at	3.41E-02	0.17188033	HPS1	HPS1, biogenesis of lysosomal organelles complex 3 subunit 1
201596_x_at	3.41E-02	0.26088742	KRT18	keratin 18
232255_at	3.41E-02	0.08005409	LINC00997	long intergenic non-protein coding RNA 997
200747_s_at	3.41E-02	0.19271834	NUMA1	nuclear mitotic apparatus protein 1
210646_x_at	3.41E-02	0.09790077	RPL13AP5///SNORD32A///SNORD33///SNORD34///SNORD35A///RPL13A	ribosomal protein L13a pseudogene 5///small nucleolar RNA, C/D box 32A///small nucleolar RNA, C/D box 33///small nucleolar RNA, C/D box 34///small nucleolar RNA, C/D box 35A///ribosomal protein L13a
235798_at	3.41E-02	-0.5169468	TMEM170B	transmembrane protein 170B
229938_at	3.41E-02	-0.14027613	TMEM238	transmembrane protein 238
243836_at	3.41E-02	-0.06145006	UNC80	unc-80 homolog, NALCN activator
233468_at	3.41E-02	-0.05557701		

239924_at	3.41E-02	-0.09617015		
1567303_at	3.41E-02	-0.04719825		
207083_s_at	3.42E-02	0.09808848	CC2D1A	coiled-coil and C2 domain containing 1A
214124_x_at	3.42E-02	-0.20550742	FGFR1OP	FGFR1 oncogene partner
1570651_at	3.42E-02	0.07202821	KYAT1	kynurenine aminotransferase 1
242304_at	3.42E-02	-0.17469285	LOC102724112///PYM1	uncharacterized LOC102724112///PYM homolog 1, exon junction complex associated factor
207480_s_at	3.42E-02	0.43616565	MEIS2	Meis homeobox 2
231079_at	3.42E-02	-0.07402316	NANOG	Nanog homeobox
1565858_at	3.42E-02	-0.07259234	SNORA71A	small nucleolar RNA, H/ACA box 71A
212242_at	3.42E-02	0.21954516	TUBA4A	tubulin alpha 4a
208628_s_at	3.42E-02	0.15769131	YBX1	Y-box binding protein 1
1563978_at	3.42E-02	0.04826591		
211277_x_at	3.43E-02	0.09063463	APP	amyloid beta precursor protein
225691_at	3.43E-02	-0.21272539	CDK12	cyclin dependent kinase 12
210272_at	3.43E-02	0.07868224	CYP2B7P	cytochrome P450 family 2 subfamily B member 7, pseudogene
239640_at	3.43E-02	-0.08003427	LOC101928623///LOC401320	uncharacterized LOC101928623///uncharacterized LOC401320
207202_s_at	3.43E-02	0.30348391	NR1I2	nuclear receptor subfamily 1 group I member 2
226670_s_at	3.43E-02	-0.41510697	PABPC1L	poly(A) binding protein cytoplasmic 1 like
202762_at	3.43E-02	-0.21393871	ROCK2	Rho associated coiled-coil containing protein kinase 2
1560618_at	3.43E-02	0.08093521		
241718_x_at	3.43E-02	-0.10008174		
235954_at	3.43E-02	0.17816178		
207369_at	3.44E-02	-0.068593	BRS3	bombesin receptor subtype 3
204724_s_at	3.44E-02	0.59252589	COL9A3	collagen type IX alpha 3 chain
214616_at	3.44E-02	0.07638094	HIST1H3E	histone cluster 1, H3e
225321_s_at	3.44E-02	0.23851201	MIR6840///STAG3L5P-PVRIG2P-PILRB///PILRB	microRNA 6840///STAG3L5P-PVRIG2P-PILRB readthrough///paired immunoglobin-like type 2 receptor beta
217738_at	3.44E-02	-0.3217097	NAMPT	nicotinamide phosphoribosyltransferase
1567256_at	3.44E-02	0.0597641	OR1J2	olfactory receptor family 1 subfamily J member 2
210363_s_at	3.44E-02	0.08858258	SCN2B	sodium voltage-gated channel beta subunit 2
235079_at	3.44E-02	-0.48243659	ZNF704	zinc finger protein 704
214102_at	3.44E-02	-0.27424655		
1559971_at	3.45E-02	0.05807323	BSDC1	BSD domain containing 1
220344_at	3.45E-02	-0.10342508	C11orf16	chromosome 11 open reading frame 16
225594_at	3.45E-02	-0.19538194	CREBZF	CREB/ATF bZIP transcription factor
238280_at	3.45E-02	0.08328136	CYB5RL	cytochrome b5 reductase like
212026_s_at	3.45E-02	-0.19290396	EXOC7	exocyst complex component 7
208749_x_at	3.45E-02	0.19870133	FLOT1	flotillin 1
233528_s_at	3.45E-02	0.07860472	GATSL3	GATS protein like 3
230272_at	3.45E-02	-0.08816968	LINC00461///MIR9-2	long intergenic non-protein coding RNA 461///microRNA 9-2
1557879_at	3.45E-02	0.07078363	LOC100129175	uncharacterized LOC100129175
230563_at	3.45E-02	-0.7613525	RASGEF1A	RasGEF domain family member 1A
222979_s_at	3.45E-02	0.17470752	SURF4	surfeit 4
214028_x_at	3.45E-02	-0.17165516	TDRD3	tudor domain containing 3
1562566_at	3.45E-02	-0.04672695	TRAF3IP2-AS1	TRAF3IP2 antisense RNA 1
220748_s_at	3.45E-02	0.13911249	ZNF580	zinc finger protein 580
1565876_x_at	3.45E-02	-0.06245556		
236659_x_at	3.45E-02	-0.2242618		
220450_at	3.45E-02	-0.11007859		
212672_at	3.46E-02	-0.19623441	ATM	ATM serine/threonine kinase
208809_s_at	3.46E-02	-0.17700883	C6orf62	chromosome 6 open reading frame 62
220739_s_at	3.46E-02	0.2208145	CNNM3	cyclin and CBS domain divalent metal cation transport mediator 3
211780_x_at	3.46E-02	0.13432961	DCTN1	dynactin subunit 1
203669_s_at	3.46E-02	0.29421902	DGAT1	diacylglycerol O-acyltransferase 1
219976_at	3.46E-02	-0.22348798	HOOK1	hook microtubule tethering protein 1
37512_at	3.46E-02	-0.26291007	HSD17B6	hydroxysteroid 17-beta dehydrogenase 6
216876_s_at	3.46E-02	-0.25197241	IL17A	interleukin 17A

1554652_s_at	3.46E-02	0.09522429	MAST4	microtubule associated serine/threonine kinase family member 4
202066_at	3.46E-02	-0.15601545	PPFIA1	PTPRF interacting protein alpha 1
224068_x_at	3.46E-02	0.16625683	RBM22	RNA binding motif protein 22
227413_at	3.46E-02	-0.17504971	UBLCP1	ubiquitin like domain containing CTD phosphatase 1
231585_at	3.46E-02	-0.10205595	VPS13A	vacuolar protein sorting 13 homolog A
222689_at	3.47E-02	-0.22522603	ACER3	alkaline ceramidase 3
1567377_at	3.47E-02	-0.0628376	DNAH1	dynein axonemal heavy chain 1
1557895_at	3.47E-02	-0.16856374	FLJ35934	FLJ35934
241355_at	3.47E-02	0.21629229	HR	hair growth associated
224111_x_at	3.47E-02	0.08598001	KLF16	Kruppel like factor 16
223322_at	3.47E-02	-0.25525512	RASSF5	Ras association domain family member 5
235656_s_at	3.47E-02	-0.14119347		
240498_at	3.47E-02	-0.24986887		
237859_at	3.47E-02	-0.05403543		
205101_at	3.48E-02	-0.18033729	CIITA	class II major histocompatibility complex transactivator
218128_at	3.48E-02	-0.25285328	NFYB	nuclear transcription factor Y subunit beta
220427_at	3.48E-02	0.0698316	OBSCN	obscurin, cytoskeletal calmodulin and titin-interacting RhoGEF
215894_at	3.48E-02	-0.49595192	PTGDR	prostaglandin D2 receptor
200773_x_at	3.48E-02	0.09785327	PTMA	prothymosin, alpha
234050_at	3.48E-02	-0.07157644	TAGAP	T-cell activation RhoGTPase activating protein
240978_at	3.48E-02	-0.06431106		
213626_at	3.49E-02	-0.27786656	CBR4	carbonyl reductase 4
223411_at	3.49E-02	-0.18343841	MIF4GD	MIF4G domain containing
220260_at	3.49E-02	-0.24715512	TBC1D19	TBC1 domain family member 19
232303_at	3.49E-02	-0.07023055	ZNF608	zinc finger protein 608
241458_at	3.49E-02	-0.21938786		
201255_x_at	3.50E-02	0.20992398	BAG6	BCL2 associated athanogene 6
242735_x_at	3.50E-02	-0.09209966	ELF2	E74 like ETS transcription factor 2
225558_at	3.50E-02	-0.17547499	GIT2	GIT ArfGAP 2
220480_at	3.50E-02	-0.08396702	HAND2	heart and neural crest derivatives expressed 2
213574_s_at	3.50E-02	-0.25215373	KPNB1	karyopherin subunit beta 1
235234_at	3.50E-02	0.13997947	PATL1	PAT1 homolog 1, processing body mRNA decay factor
230536_at	3.50E-02	0.2558148	PBX4	PBX homeobox 4
210580_x_at	3.50E-02	0.26439916	SLX1B-SULT1A4///SLX1A-SULT1A3///SULT1A4///SULT1A3	SLX1B-SULT1A4 readthrough (NMD candidate)///SLX1A-SULT1A3 readthrough (NMD candidate)///sulfotransferase family 1A member 4///sulfotransferase family 1A member 3
232188_at	3.51E-02	-0.05434859	AKAP13	A-kinase anchoring protein 13
231847_at	3.51E-02	0.13039884	ANKRD54	ankyrin repeat domain 54
235357_at	3.51E-02	0.08292929	DPF3	double PHD fingers 3
205921_s_at	3.51E-02	0.15477034	SLC6A6	solute carrier family 6 member 6
238672_at	3.51E-02	-0.23244215		
237534_at	3.51E-02	-0.0916508		
222577_at	3.52E-02	-0.08726785	CCDC90B	coiled-coil domain containing 90B
40472_at	3.52E-02	0.2722802	LPCAT4	lysophosphatidylcholine acyltransferase 4
207567_at	3.52E-02	0.10168009	SLC13A2	solute carrier family 13 member 2
243570_at	3.52E-02	-0.12720359	SPCS2	signal peptidase complex subunit 2
244161_at	3.52E-02	-0.08208686		
244078_at	3.52E-02	-0.19554926		
37549_g_at	3.53E-02	-0.19986827	BBS9	Bardet-Biedl syndrome 9
214439_x_at	3.53E-02	0.29042959	BIN1	bridging integrator 1
218142_s_at	3.53E-02	-0.20205231	CRBN	cereblon
235600_at	3.53E-02	-0.08623492	DLK1	delta like non-canonical Notch ligand 1
242561_at	3.53E-02	0.10449696	IPO9	importin 9
206791_s_at	3.53E-02	0.09244949	LOC729966///PDE4C	uncharacterized LOC729966///phosphodiesterase 4C
233802_at	3.53E-02	-0.07628564	MED28	mediator complex subunit 28
213946_s_at	3.53E-02	0.07050602	OBSL1	obscurin like 1
233168_s_at	3.53E-02	0.13426537	SELO	selenoprotein O
228696_at	3.53E-02	0.15836892	SLC45A3	solute carrier family 45 member 3
218151_x_at	3.53E-02	0.22715907	SLC52A2	solute carrier family 52 member 2
231404_at	3.53E-02	-0.11163844	SPTY2D1-AS1	SPTY2D1 antisense RNA 1

210637_at	3.53E-02	-0.10887378	TACR1	tachykinin receptor 1
224376_s_at	3.53E-02	0.2326674	TGIF2-C20orf24///C20orf24	TGIF2-C20orf24 readthrough///chromosome 20 open reading frame 24
214800_x_at	3.54E-02	-0.12344093	BTF3	basic transcription factor 3
217431_x_at	3.54E-02	0.05196711	CYBB	cytochrome b-245 beta chain
228755_at	3.54E-02	0.12729705	GIGYF1	GRB10 interacting GYF protein 1
230550_at	3.54E-02	-0.3854998	MS4A6A	membrane spanning 4-domains A6A
234524_at	3.54E-02	0.06040871	PRDM15	PR/SET domain 15
205427_at	3.54E-02	-0.18647187	ZNF354A	zinc finger protein 354A
215370_at	3.54E-02	-0.09358063		
238185_at	3.54E-02	-0.14531111		
1569748_at	3.54E-02	-0.07237226		
233053_at	3.54E-02	-0.16702564		
232356_at	3.54E-02	-0.26953275		
216773_at	3.54E-02	0.05563783		
218216_x_at	3.55E-02	0.18533337	ARL6IP4	ADP ribosylation factor like GTPase 6 interacting protein 4
201848_s_at	3.55E-02	-0.50724186	BNIP3	BCL2 interacting protein 3
238365_s_at	3.55E-02	0.06540408	C1orf228	chromosome 1 open reading frame 228
217309_s_at	3.55E-02	0.14843862	DSCR3	DSCR3 arrestin fold containing
232164_s_at	3.55E-02	-0.29129792	EPPK1	epiplakin 1
222119_s_at	3.55E-02	-0.1662201	FBXO11	F-box protein 11
205811_at	3.55E-02	-0.17082772	POLG2	DNA polymerase gamma 2, accessory subunit
217381_s_at	3.55E-02	-0.08034199	TARP	TCR gamma alternate reading frame protein
235392_at	3.55E-02	0.19856975		
212541_at	3.56E-02	0.16806885	FLAD1	flavin adenine dinucleotide synthetase 1
229893_at	3.56E-02	-0.4076593	FRMD3	FERM domain containing 3
240306_at	3.56E-02	0.07858053	LINC00312	long intergenic non-protein coding RNA 312
1559514_at	3.56E-02	0.05688991	LOC100132077	uncharacterized LOC100132077
220826_at	3.56E-02	0.08914791	TCP10L	t-complex 10-like
1570225_at	3.56E-02	0.08794952	TMEM150B	transmembrane protein 150B
238831_at	3.56E-02	-0.24192334	TMEM33	transmembrane protein 33
223956_at	3.56E-02	-0.10692402	TMPRSS13	transmembrane protease, serine 13
237059_at	3.56E-02	0.09399435	VTA1	vesicle trafficking 1
240836_at	3.56E-02	-0.06952444	ZNF19	zinc finger protein 19
1552375_at	3.56E-02	-0.1201793	ZNF333	zinc finger protein 333
233465_at	3.56E-02	0.11376819		
230141_at	3.57E-02	-0.2050763	ARID4A	AT-rich interaction domain 4A
221918_at	3.57E-02	-0.15619267	CDK17	cyclin dependent kinase 17
1563919_a_at	3.57E-02	0.08711138	CEP89	centrosomal protein 89
1554757_a_at	3.57E-02	0.07950733	INPP5A	inositol polyphosphate-5-phosphatase A
1553739_at	3.57E-02	-0.05244651	IRAK2	interleukin 1 receptor associated kinase 2
1563404_at	3.57E-02	0.07248751		
224120_at	3.57E-02	0.07590375		
235326_at	3.58E-02	-0.06620195	CPSF7	cleavage and polyadenylation specific factor 7
65635_at	3.58E-02	0.17845769	ENGASE	endo-beta-N-acetylglucosaminidase
1557915_s_at	3.58E-02	0.18140369	GSTO1	glutathione S-transferase omega 1
210450_at	3.58E-02	-0.06118816	IGHV5-78	immunoglobulin heavy variable 5-78 (pseudogene)
1552490_at	3.58E-02	-0.09663408	LACE1	lactation elevated 1
214579_at	3.58E-02	0.10123416	NIPAL3	NIPA like domain containing 3
39891_at	3.58E-02	0.12437399	ZNF710	zinc finger protein 710
1552663_a_at	3.59E-02	0.0789527	ERC1	ELKS/RAB6-interacting/CAST family member 1
211307_s_at	3.59E-02	0.0667152	FCAR	Fc fragment of IgA receptor
231745_at	3.59E-02	0.06057386	GPR55	G protein-coupled receptor 55
235449_at	3.59E-02	0.07264957	LRSAM1	leucine rich repeat and sterile alpha motif containing 1
1570585_at	3.59E-02	0.07490111	MPZL3	myelin protein zero like 3
213630_at	3.59E-02	0.05964896	NACAD	NAC alpha domain containing
229285_at	3.59E-02	0.30189706	RNASEL	ribonuclease L
220605_s_at	3.59E-02	0.08435551	SIRT2	sirtuin 2
208420_x_at	3.59E-02	0.1729423	SUPT6H	SPT6 homolog, histone chaperone
242240_at	3.59E-02	-0.216054		
232681_at	3.59E-02	-0.26701718		
1564816_at	3.60E-02	0.0893114	C14orf178	chromosome 14 open reading frame 178

212948_at	3.60E-02	0.17789374	CAMTA2	calmodulin binding transcription activator 2
207897_at	3.60E-02	0.08545623	CRHR2	corticotropin releasing hormone receptor 2
231345_s_at	3.60E-02	-0.07824521	DHRS12	dehydrogenase/reductase 12
228673_s_at	3.60E-02	-0.114665	EML4	echinoderm microtubule associated protein like 4
1556014_at	3.60E-02	0.06943723	MESP2	mesoderm posterior bHLH transcription factor 2
221142_s_at	3.60E-02	0.16032508	PECR	peroxisomal trans-2-enoyl-CoA reductase
217998_at	3.60E-02	0.16353998	PHLDA1	pleckstrin homology like domain family A member 1
235668_at	3.60E-02	-0.20392903	PRDM1	PR/SET domain 1
204223_at	3.60E-02	0.12284226	PRELP	proline and arginine rich end leucine rich repeat protein
214443_at	3.60E-02	0.09881275	PVR	poliovirus receptor
230078_at	3.60E-02	-0.18792612	RAPGEF6	Rap guanine nucleotide exchange factor 6
238007_at	3.60E-02	-0.15664966	ZNF271P	zinc finger protein 271, pseudogene
235671_at	3.60E-02	-0.07851972		
222330_at	3.60E-02	-0.17013729		
1562505_at	3.60E-02	0.13041985		
217099_s_at	3.61E-02	0.22255962	GEMIN4	gem nuclear organelle associated protein 4
232687_at	3.61E-02	-0.3803233	GPRIN3	GPRIN family member 3
216678_at	3.61E-02	0.08174707	IFT122	intraflagellar transport 122
1559392_s_at	3.61E-02	0.05721649	SYT7	synaptotagmin 7
218368_s_at	3.61E-02	0.21373206	TNFRSF12A	TNF receptor superfamily member 12A
233710_at	3.61E-02	0.07579852		
222960_at	3.62E-02	-0.0849074	CACNA1H	calcium voltage-gated channel subunit alpha1 H
202337_at	3.62E-02	0.18032929	PMF1	polyamine-modulated factor 1
221634_at	3.62E-02	-0.13521759	RPL23AP7///CENPBD1P1	ribosomal protein L23a pseudogene 7///CENPB DNA-binding domains containing 1 pseudogene 1
230624_at	3.62E-02	-0.43182928	SLC25A27	solute carrier family 25 member 27
241540_at	3.62E-02	-0.11525294		
201284_s_at	3.63E-02	0.24201303	APEH	acylaminoacyl-peptide hydrolase
229482_at	3.63E-02	-0.07796174	DDX51	DEAD-box helicase 51
219372_at	3.63E-02	-0.19750983	IFT81	intraflagellar transport 81
242838_at	3.63E-02	0.15924324	MAP6D1	MAP6 domain containing 1
1563809_a_at	3.63E-02	0.08055168	MCF2L	MCF.2 cell line derived transforming sequence like
219959_at	3.63E-02	0.42386422	MOCOS	molybdenum cofactor sulfurase
201874_at	3.63E-02	0.16818644	MPZL1	myelin protein zero like 1
227088_at	3.63E-02	-0.36857347	PDE5A	phosphodiesterase 5A
204267_x_at	3.63E-02	0.12572949	PKMYT1	protein kinase, membrane associated tyrosine/threonine 1
209267_s_at	3.63E-02	-0.49065133	SLC39A8	solute carrier family 39 member 8
226615_at	3.63E-02	-0.1876017	XPR1	xenotropic and polytropic retrovirus receptor 1
1554648_a_at	3.64E-02	0.09109579	DUOXA1	dual oxidase maturation factor 1
236835_at	3.64E-02	-0.17291765	FUT8-AS1	FUT8 antisense RNA 1
219769_at	3.64E-02	0.09578542	INCENP	inner centromere protein
1552311_a_at	3.64E-02	0.06605342	RAX2	retina and anterior neural fold homeobox 2
235425_at	3.64E-02	-0.35748421	SGO2	shugoshin 2
217899_at	3.64E-02	0.15865032	TMEM214	transmembrane protein 214
244189_at	3.64E-02	-0.27344227	TTC28-AS1	TTC28 antisense RNA 1
209088_s_at	3.64E-02	0.13988548	UBN1	ubinuclein 1
1566786_at	3.64E-02	-0.06244478		
207275_s_at	3.65E-02	0.31526104	ACSL1	acyl-CoA synthetase long-chain family member 1
210469_at	3.65E-02	0.16684503	DLG5	discs large MAGUK scaffold protein 5
219494_at	3.65E-02	-0.34388516	RAD54B	RAD54 homolog B (S. cerevisiae)
230121_at	3.65E-02	-0.10809242	SERTAD4-AS1	SERTAD4 antisense RNA 1
234716_at	3.65E-02	0.06915799	ZIC1	Zic family member 1
1563541_at	3.65E-02	-0.06542145		
235229_at	3.65E-02	-0.67155527		
238307_at	3.65E-02	0.09699025		
209005_at	3.66E-02	-0.19735113	FBXL5	F-box and leucine rich repeat protein 5
204396_s_at	3.66E-02	0.2704429	GRK5	G protein-coupled receptor kinase 5
227771_at	3.66E-02	-0.12720364	LIFR	leukemia inhibitory factor receptor alpha
218574_s_at	3.66E-02	0.25652101	LMCD1	LIM and cysteine rich domains 1
1556748_x_at	3.66E-02	0.08158405	LOC101928343	uncharacterized LOC101928343
204634_at	3.66E-02	-0.19763101	NEK4	NIMA related kinase 4

216593_s_at	3.66E-02	0.22943051	PIGC	phosphatidylinositol glycan anchor biosynthesis class C
214790_at	3.66E-02	-0.21847253	SENP6	SUMO1/sentrin specific peptidase 6
223906_s_at	3.66E-02	-0.07400621	TEX101	testis expressed 101
217149_x_at	3.66E-02	0.09086287	TNK1	tyrosine kinase non receptor 1
219540_at	3.66E-02	-0.20895501	ZNF267	zinc finger protein 267
240079_at	3.66E-02	-0.27222026	ZNF81	zinc finger protein 81
243588_at	3.66E-02	0.05868613		
242491_at	3.66E-02	-0.0638579		
1554007_at	3.66E-02	-0.37680539		
223352_s_at	3.67E-02	-0.11785658	C17orf80	chromosome 17 open reading frame 80
225722_at	3.67E-02	0.20315951	MIR3658///UCK2	microRNA 3658///uridine-cytidine kinase 2
225092_at	3.67E-02	-0.24188835	RABEP1	rabaptin, RAB GTPase binding effector protein 1
203185_at	3.67E-02	-0.21081784	RASSF2	Ras association domain family member 2
1569294_at	3.67E-02	0.05995084	RNF187	ring finger protein 187
206608_s_at	3.67E-02	0.07757364	RPGRIP1	retinitis pigmentosa GTPase regulator interacting protein 1
203330_s_at	3.67E-02	0.11860062	STX5	syntaxin 5
220832_at	3.67E-02	0.0687372	TLR8	toll like receptor 8
242242_at	3.67E-02	0.08724066	USP32///USP6	ubiquitin specific peptidase 32///ubiquitin specific peptidase 6
214547_at	3.68E-02	-0.04702257	ADCY10	adenylate cyclase 10, soluble
212114_at	3.68E-02	0.14620706	ATXN7L3B	ataxin 7 like 3B
215954_s_at	3.68E-02	0.13201043	CACTIN	cactin, spliceosome C complex subunit
217528_at	3.68E-02	-0.12755977	CLCA2	chloride channel accessory 2
211167_s_at	3.68E-02	0.06522607	GCK	glucokinase
203005_at	3.68E-02	0.17968212	LTBR	lymphotoxin beta receptor
213296_at	3.68E-02	0.17073427	RER1	retention in endoplasmic reticulum sorting receptor 1
231823_s_at	3.68E-02	0.18879992	SH3PXD2B	SH3 and PX domains 2B
1553727_at	3.69E-02	0.0857264	B4GALNT3	beta-1,4-N-acetyl-galactosaminyltransferase 3
1556299_s_at	3.69E-02	0.06053771	C12orf76	chromosome 12 open reading frame 76
214104_at	3.69E-02	0.25208018	GPR161	G protein-coupled receptor 161
1560823_at	3.69E-02	0.04621318	LOC340017	uncharacterized LOC340017
1555127_at	3.69E-02	-0.05685609	MOCS1	molybdenum cofactor synthesis 1
212140_at	3.69E-02	-0.14533707	PDS5A	PDS5 cohesin associated factor A
209441_at	3.69E-02	0.07709543	RHOBTB2	Rho related BTB domain containing 2
228502_at	3.69E-02	-0.20619633	RPS6KA6	ribosomal protein S6 kinase A6
213285_at	3.69E-02	-0.25015633	TMEM30B	transmembrane protein 30B
1566600_at	3.69E-02	-0.05084151		
215716_s_at	3.70E-02	-0.26396224	ATP2B1	ATPase plasma membrane Ca2+ transporting 1
223255_at	3.70E-02	-0.22613823	G2E3	G2/M-phase specific E3 ubiquitin protein ligase
204921_at	3.70E-02	0.11230239	GAS8	growth arrest specific 8
202455_at	3.70E-02	0.22543	HDAC5	histone deacetylase 5
218886_at	3.70E-02	-0.2390646	PAK1IP1	PAK1 interacting protein 1
1561030_at	3.70E-02	0.11773443	TMC7	transmembrane channel like 7
1568592_at	3.70E-02	-0.29219941	TRIM69	tripartite motif containing 69
206168_at	3.70E-02	0.07560975	ZC3H7B	zinc finger CCCH-type containing 7B
1562998_at	3.70E-02	-0.04102434		
223532_at	3.71E-02	0.18567274	ANKRD39	ankyrin repeat domain 39
208260_at	3.71E-02	0.07474336	AVPR1B	arginine vasopressin receptor 1B
1558739_at	3.71E-02	-0.18703019	BORCS5	BLOC-1 related complex subunit 5
240983_s_at	3.71E-02	0.13015508	CARS	cysteinyl-tRNA synthetase
228731_at	3.71E-02	-0.4059194	GUCY1A2	guanylate cyclase 1 soluble subunit alpha 2
208470_s_at	3.71E-02	-0.08292751	HPR///HP	haptoglobin-related protein///haptoglobin
233809_at	3.71E-02	-0.12799792	HYPK	huntingtin interacting protein K
237178_at	3.71E-02	-0.07963604	LOC102723932	uncharacterized LOC102723932
213653_at	3.71E-02	-0.1864747	METTL3	methyltransferase like 3
209061_at	3.71E-02	-0.22045922	NCOA3	nuclear receptor coactivator 3
210664_s_at	3.71E-02	-0.47099612	TFPI	tissue factor pathway inhibitor
206188_at	3.71E-02	-0.24873294	ZNF623	zinc finger protein 623
1569743_at	3.71E-02	-0.06529863		
229315_at	3.71E-02	-0.27935097		
228477_at	3.72E-02	-0.28437655	ARGLU1	arginine and glutamate rich 1
1565716_at	3.72E-02	0.10756311	FUS	FUS RNA binding protein

201277_s_at	3.72E-02	0.20256746	HNRNPAB	heterogeneous nuclear ribonucleoprotein A/B
215736_at	3.72E-02	-0.07940165	KCNV1	potassium voltage-gated channel modifier subfamily V member 1
242208_at	3.72E-02	0.17277953	ZNF37BP	zinc finger protein 37B, pseudogene
244085_at	3.72E-02	-0.07606903	ZNF653	zinc finger protein 653
1569601_at	3.72E-02	-0.17098008		
237822_at	3.72E-02	-0.06987785		
233706_at	3.72E-02	-0.08260363		
217755_at	3.73E-02	0.27345382	HN1	hematological and neurological expressed 1
1558392_at	3.73E-02	-0.10038986	SYNE2	spectrin repeat containing nuclear envelope protein 2
241819_at	3.73E-02	-0.0760802	TNFSF8	tumor necrosis factor superfamily member 8
215866_at	3.73E-02	-0.09816009		
240148_at	3.73E-02	-0.08527644		
238407_at	3.73E-02	-0.15848033		
239059_at	3.74E-02	0.07449807	DNAH1	dynein axonemal heavy chain 1
220600_at	3.74E-02	0.09326877	ELP6	elongator acetyltransferase complex subunit 6
1552507_at	3.74E-02	0.13374556	KCNE4	potassium voltage-gated channel subfamily E regulatory subunit 4
202527_s_at	3.74E-02	-0.32342823	SMAD4	SMAD family member 4
227572_at	3.74E-02	-0.21781553	USP30	ubiquitin specific peptidase 30
233899_x_at	3.74E-02	-0.49220935	ZBTB10	zinc finger and BTB domain containing 10
1558078_at	3.74E-02	0.26717048		
1559575_a_at	3.75E-02	0.1537538	AQP12B///AQP12A	aquaporin 12B///aquaporin 12A
1569693_at	3.75E-02	-0.05680288	BTBD8	BTB domain containing 8
205845_at	3.75E-02	0.09931993	CACNA1H	calcium voltage-gated channel subunit alpha1 H
207876_s_at	3.75E-02	0.16690291	FLNC	filamin C
215912_at	3.75E-02	-0.07059972	GNAO1	G protein subunit alpha o1
202755_s_at	3.75E-02	0.10046199	GPC1	glypican 1
1558046_x_at	3.75E-02	0.12125701	LOC389906	zinc finger protein 839 pseudogene
206218_at	3.75E-02	0.38955536	MAGEB2	MAGE family member B2
205699_at	3.75E-02	-0.15428115	MAP2K6	mitogen-activated protein kinase kinase 6
241983_at	3.75E-02	-0.09225719	PTPN2	protein tyrosine phosphatase, non-receptor type 2
225064_at	3.75E-02	-0.20988109	RABEP1	rabaptin, RAB GTPase binding effector protein 1
220859_at	3.75E-02	-0.07272404		
236404_at	3.75E-02	-0.23210989		
39248_at	3.76E-02	0.66069284	AQP3	aquaporin 3 (Gill blood group)
209964_s_at	3.76E-02	0.11966448	ATXN7	ataxin 7
1564736_a_at	3.76E-02	0.06478756	CASP12	caspase 12 (gene/pseudogene)
202470_s_at	3.76E-02	-0.21623099	CPSF6	cleavage and polyadenylation specific factor 6
230826_at	3.76E-02	0.07413785	MMD2	monocyte to macrophage differentiation associated 2
201115_at	3.76E-02	0.22310469	POLD2	DAN polymerase delta 2, accessory subunit
202126_at	3.76E-02	-0.16022978	PRPF4B	pre-mRNA processing factor 4B
224136_at	3.76E-02	-0.06739944		
228389_at	3.76E-02	-0.1999362		
217880_at	3.77E-02	-0.17475934	CDC27	cell division cycle 27
211114_x_at	3.77E-02	-0.17790147	GEMIN2	gem nuclear organelle associated protein 2
203332_s_at	3.77E-02	0.33731616	INPP5D	inositol polyphosphate-5-phosphatase D
227713_at	3.77E-02	-0.26655804	KATNAL1	katanin catalytic subunit A1 like 1
1555862_s_at	3.77E-02	0.20585356	MICALL2	MICAL like 2
209241_x_at	3.77E-02	0.10314922	MINK1	misshapen like kinase 1
234318_x_at	3.77E-02	0.08070239	NDOR1	NADPH dependent diflavin oxidoreductase 1
226522_at	3.77E-02	0.09350923	PODN	podocan
223471_at	3.77E-02	-0.19706449	RAB3IP	RAB3A interacting protein
244653_at	3.77E-02	0.12388291	SETD7	SET domain containing lysine methyltransferase 7
1566645_at	3.77E-02	-0.05860044		
218337_at	3.78E-02	0.17357889	FAM160B2	family with sequence similarity 160 member B2
211401_s_at	3.78E-02	0.09699828	FGFR2	fibroblast growth factor receptor 2
213021_at	3.78E-02	-0.15963007	GOSR1	golgi SNAP receptor complex member 1
35617_at	3.78E-02	0.11027343	MAPK7	mitogen-activated protein kinase 7
242002_at	3.78E-02	-0.25282242	NKAIN2	Na+/K+ transporting ATPase interacting 2
201011_at	3.78E-02	0.19156661	RPN1	ribophorin I
241125_at	3.78E-02	-0.06950457		
228190_at	3.79E-02	-0.21734058	ATG4C	autophagy related 4C cysteine peptidase

223257_at	3.79E-02	-0.23622423	G2E3	G2/M-phase specific E3 ubiquitin protein ligase
201698_s_at	3.79E-02	0.23487086	GATC///SRSF9	glutamyl-tRNA amidotransferase subunit C///serine and arginine rich splicing factor 9
240317_at	3.79E-02	0.14451997	PCDHB4	protocadherin beta 4
206351_s_at	3.79E-02	0.09197942	PEX10	peroxisomal biogenesis factor 10
218044_x_at	3.79E-02	0.09117481	PTMS	parathymosin
1552617_a_at	3.79E-02	0.17576578	RFWD2	ring finger and WD repeat domain 2
201357_s_at	3.79E-02	0.10609676	SF3A1	splicing factor 3a subunit 1
1566607_at	3.79E-02	-0.06599097		
229243_at	3.79E-02	-0.3059578		
205583_s_at	3.80E-02	-0.27703613	ALG13	ALG13, UDP-N-acetylglucosaminyltransferase subunit
229628_s_at	3.80E-02	-0.15475279	CCDC180///LOC100499484-C9ORF174	coiled-coil domain containing 180///LOC100499484-C9orf174 readthrough
217262_s_at	3.80E-02	0.08187598	CELSR1	cadherin EGF LAG seven-pass G-type receptor 1
208624_s_at	3.80E-02	0.2179757	EIF4G1	eukaryotic translation initiation factor 4 gamma 1
232431_at	3.80E-02	-0.29611367	NR3C1	nuclear receptor subfamily 3 group C member 1
211867_s_at	3.80E-02	0.06820667	PCDHA10	protocadherin alpha 10
209544_at	3.80E-02	-0.23361837	RIPK2	receptor interacting serine/threonine kinase 2
221216_s_at	3.80E-02	0.18853068	SCMH1	sex comb on midleg homolog 1 (Drosophila)
219205_at	3.80E-02	-0.18821307	SRR	serine racemase
1558532_at	3.80E-02	0.11082346	TPM1	tropomyosin 1 (alpha)
235810_at	3.80E-02	-0.22849224	ZNF182	zinc finger protein 182
223586_at	3.81E-02	-0.31899733	ARNTL2	aryl hydrocarbon receptor nuclear translocator like 2
212649_at	3.81E-02	-0.12878103	DHX29	DEAH-box helicase 29
224761_at	3.81E-02	-0.15369329	GNA13	G protein subunit alpha 13
239148_at	3.81E-02	-0.20190656	MARVELD3	MARVEL domain containing 3
214378_at	3.81E-02	-0.09970035	TFPI	tissue factor pathway inhibitor
212963_at	3.81E-02	-0.11858958	TM2D1	TM2 domain containing 1
228447_at	3.82E-02	0.05421018	AKAP17A	A-kinase anchoring protein 17A
210462_at	3.82E-02	0.09349165	BLZF1	basic leucine zipper nuclear factor 1
200984_s_at	3.82E-02	0.27131283	CD59	CD59 molecule
207255_at	3.82E-02	0.0740291	LEPROT///LEPR	leptin receptor overlapping transcript///leptin receptor
218515_at	3.82E-02	-0.17072679	PAXBP1	PAX3 and PAX7 binding protein 1
228001_at	3.82E-02	0.13372008	TMEM50B	transmembrane protein 50B
234308_at	3.82E-02	0.08078402	TUBGCP6	tubulin gamma complex associated protein 6
81811_at	3.82E-02	0.19176471		
205827_at	3.83E-02	0.26268439	CCK	cholecystokinin
225565_at	3.83E-02	-0.22050891	CREB1	cAMP responsive element binding protein 1
227158_at	3.83E-02	-0.26002135	DTD2	D-tyrosyl-tRNA deacylase 2 (putative)
234450_at	3.83E-02	0.08124655	PROKR2	prokineticin receptor 2
207439_s_at	3.83E-02	0.10365152	SLC35A2	solute carrier family 35 member A2
214881_s_at	3.83E-02	0.13374338	UBTF	upstream binding transcription factor, RNA polymerase I
1552430_at	3.83E-02	-0.0696878	WDR17	WD repeat domain 17
242868_at	3.83E-02	0.24342197		
243670_at	3.83E-02	0.11442544		
242010_at	3.83E-02	-0.07570252		
243154_at	3.83E-02	0.40093827		
241059_at	3.83E-02	0.07157378		
214721_x_at	3.84E-02	0.16416747	CDC42EP4	CDC42 effector protein 4
220320_at	3.84E-02	0.08259567	DOK3	docking protein 3
204773_at	3.84E-02	0.15589163	IL11RA	interleukin 11 receptor subunit alpha
1569355_at	3.84E-02	0.05947566	KCNIP3	potassium voltage-gated channel interacting protein 3
208960_s_at	3.84E-02	0.18845646	KLF6	Kruppel like factor 6
212566_at	3.84E-02	0.18435076	MAP4	microtubule associated protein 4
217465_at	3.84E-02	-0.12797818	NCKAP1	NCK associated protein 1
232593_at	3.84E-02	-0.15019404	NEURL3	neuralized E3 ubiquitin protein ligase 3
1552924_a_at	3.84E-02	0.09898176	PITPNM2	phosphatidylinositol transfer protein membrane associated 2
1567030_at	3.84E-02	0.09261089	SH3GL1P2	SH3 domain containing GRB2 like 1, endophilin A2 pseudogene 2
201506_at	3.84E-02	0.32912342	TGFBI	transforming growth factor beta induced
229348_at	3.84E-02	-0.10507157	UBIAD1	UbiA prenyltransferase domain containing 1

208542_x_at	3.84E-02	-0.05190938	ZNF718///ZNF595///ZNF208	zinc finger protein 718///zinc finger protein 595///zinc finger protein 208
230860_at	3.85E-02	-0.14722471	CEP19	centrosomal protein 19
224150_s_at	3.85E-02	-0.29005956	CEP70	centrosomal protein 70
1552390_a_at	3.85E-02	-0.44278862	ERICH5	glutamate rich 5
228452_at	3.85E-02	-0.19492104	GID4	GID complex subunit 4 homolog
1555628_a_at	3.85E-02	0.08719689	HAVCR2	hepatitis A virus cellular receptor 2
1557050_at	3.85E-02	0.09166823	HOTAIRM1	HOXA transcript antisense RNA, myeloid-specific 1
223315_at	3.85E-02	-0.45933791	NTN4	netrin 4
239222_at	3.85E-02	0.06211278	SPACA9	sperm acrosome associated 9
229965_at	3.85E-02	0.08585533		
203872_at	3.86E-02	0.06634475	ACTA1	actin, alpha 1, skeletal muscle
233543_s_at	3.86E-02	-0.19602138	FAM175A	family with sequence similarity 175 member A
232171_x_at	3.86E-02	0.20506511	KLHDC4	kelch domain containing 4
216229_x_at	3.86E-02	-0.10656824	LOC105379655	uncharacterized LOC105379655
217977_at	3.86E-02	0.21879327	MSRB1	methionine sulfoxide reductase B1
49878_at	3.86E-02	0.10714088	PEX16	peroxisomal biogenesis factor 16
219126_at	3.86E-02	-0.20362839	PHF10	PHD finger protein 10
239855_at	3.86E-02	0.0863366	PPM1L	protein phosphatase, Mg2+/Mn2+ dependent 1L
215195_at	3.86E-02	-0.22081152	PRKCA	protein kinase C alpha
220027_s_at	3.86E-02	0.13514821	RASIP1	Ras interacting protein 1
237380_at	3.86E-02	0.0647831		
1569644_at	3.86E-02	0.06880635		
240154_at	3.86E-02	-0.19383709		
243174_at	3.86E-02	-0.07053998		
231428_at	3.86E-02	-0.11951643		
203945_at	3.87E-02	-0.16146861	ARG2	arginase 2
226798_at	3.87E-02	-0.10239295	BCL2L13	BCL2 like 13
203082_at	3.87E-02	0.14416012	BMS1	BMS1, ribosome biogenesis factor
239509_at	3.87E-02	0.06505449	FLJ16779	uncharacterized LOC100192386
203697_at	3.87E-02	-0.43843317	FRZB	frizzled-related protein
216615_s_at	3.87E-02	0.08399897	HTR3A	5-hydroxytryptamine receptor 3A
214748_at	3.87E-02	-0.36120748	N4BP2L2	NEDD4 binding protein 2 like 2
238637_at	3.87E-02	-0.2059614	NUP37	nucleoporin 37
204519_s_at	3.87E-02	0.45624759	PLLP	plasmolipin
211431_s_at	3.87E-02	0.06069323	TYRO3	TYRO3 protein tyrosine kinase
1564236_at	3.87E-02	-0.25169271		
241284_at	3.87E-02	-0.05049909		
1565730_at	3.87E-02	0.0571984		
214783_s_at	3.88E-02	0.21433538	ANXA11	annexin A11
1561054_a_at	3.88E-02	-0.05718471	CCDC14	coiled-coil domain containing 14
238682_at	3.88E-02	-0.07443413	CCDC96	coiled-coil domain containing 96
206363_at	3.88E-02	-0.22016937	MAF	MAF bZIP transcription factor
228983_at	3.88E-02	-0.11729566	NCBP3	nuclear cap binding subunit 3
1553060_at	3.88E-02	0.08152031	PSKH2	protein serine kinase H2
201802_at	3.88E-02	0.23796384	SLC29A1	solute carrier family 29 member 1 (Augustine blood group)
201563_at	3.88E-02	0.2868489	SORD	sorbitol dehydrogenase
1552974_at	3.88E-02	0.04135807		
227464_at	3.89E-02	0.13585757	ACSF3	acyl-CoA synthetase family member 3
207315_at	3.89E-02	-0.07410084	CD226	CD226 molecule
233068_at	3.89E-02	-0.21455701	CRIPT	CXXC repeat containing interactor of PDZ3 domain
219411_at	3.89E-02	0.24669495	ELMO3	engulfment and cell motility 3
1557207_s_at	3.89E-02	-0.23799807	LOC283177	uncharacterized LOC283177
1563009_at	3.89E-02	0.07674434	LOC284930	uncharacterized LOC284930
204310_s_at	3.89E-02	0.14785167	NPR2	natriuretic peptide receptor 2
226836_at	3.89E-02	-0.08470461	SFT2D1	SFT2 domain containing 1
211988_at	3.89E-02	-0.23939503	SMARCE1	SWI/SNF related, matrix associated, actin dependent regulator of chromatin, subfamily e, member 1
1554618_at	3.90E-02	0.08072379	AGFG2	ArfGAP with FG repeats 2
228342_s_at	3.90E-02	0.31422586	ALPK3	alpha kinase 3
229888_at	3.90E-02	-0.26171843	C12orf60	chromosome 12 open reading frame 60
237395_at	3.90E-02	-0.10222904	CYP4Z1	cytochrome P450 family 4 subfamily Z member 1

220102_at	3.90E-02	-0.16722173	FOXL2	forkhead box L2
205582_s_at	3.90E-02	0.10502737	GGT5	gamma-glutamyltransferase 5
235031_at	3.90E-02	-0.20462995	INVS///WSB1	inversin///WD repeat and SOCS box containing 1
237343_at	3.90E-02	0.06673648	LINC01304	long intergenic non-protein coding RNA 1304
235509_at	3.90E-02	-0.2570671	LOC100506538///NDUFAF6	uncharacterized LOC100506538///NADH:ubiquinone oxidoreductase complex assembly factor 6
227394_at	3.90E-02	-0.14990758	NCAM1	neural cell adhesion molecule 1
212071_s_at	3.90E-02	0.15433668	SPTBN1	spectrin beta, non-erythrocytic 1
213685_at	3.90E-02	-0.2106408	TCEB3-AS1	TCEB3 antisense RNA 1
228997_at	3.90E-02	-0.1730931	TRNAU1AP	tRNA selenocysteine 1 associated protein 1
217593_at	3.90E-02	0.1492845	ZSCAN18	zinc finger and SCAN domain containing 18
242858_at	3.90E-02	0.09187097		
233521_at	3.90E-02	0.07536148		
207026_s_at	3.91E-02	0.08926399	ATP2B3	ATPase plasma membrane Ca2+ transporting 3
237145_at	3.91E-02	0.21996311	EIF2AK4	eukaryotic translation initiation factor 2 alpha kinase 4
215841_at	3.91E-02	0.07392417	GUCA1B	guanylate cyclase activator 1B
221097_s_at	3.91E-02	-0.06683624	KCNMB2	potassium calcium-activated channel subfamily M regulatory beta subunit 2
233665_x_at	3.91E-02	-0.15170039	MTO1	mitochondrial tRNA translation optimization 1
212044_s_at	3.91E-02	-0.23648198	RPL27A	ribosomal protein L27a
223366_at	3.91E-02	-0.42023397	ZNF704	zinc finger protein 704
234187_at	3.91E-02	0.07958108		
229307_at	3.92E-02	-0.26571721	ANKRD28	ankyrin repeat domain 28
235829_at	3.92E-02	-0.08966756	CAHM	colon adenocarcinoma hypermethylated (non-protein coding)
201989_s_at	3.92E-02	-0.21589054	CREBL2	cAMP responsive element binding protein like 2
223454_at	3.92E-02	0.3032982	CXCL16	C-X-C motif chemokine ligand 16
238356_at	3.92E-02	-0.16581162	DOCK11	dedicator of cytokinesis 11
227781_x_at	3.92E-02	-0.09283047	FAM57B	family with sequence similarity 57 member B
205600_x_at	3.92E-02	0.18772774	HOXB5	homeobox B5
215946_x_at	3.92E-02	-0.47261419	IGLL3P	immunoglobulin lambda like polypeptide 3, pseudogene
223370_at	3.92E-02	-0.14435966	PLEKHA3	pleckstrin homology domain containing A3
236454_at	3.92E-02	-0.08709487	RNF212	ring finger protein 212
200872_at	3.92E-02	0.13790444	S100A10	S100 calcium binding protein A10
1570420_at	3.92E-02	0.07958405	STXBP2	syntaxin binding protein 2
224974_at	3.92E-02	-0.18065319	SUDS3	SDS3 homolog, SIN3A corepressor complex component
231403_at	3.92E-02	-0.2117258	TRIO	trio Rho guanine nucleotide exchange factor
206978_at	3.93E-02	-0.29002776	CCR2	C-C motif chemokine receptor 2
230909_at	3.93E-02	0.06777543	COX18	COX18, cytochrome c oxidase assembly factor
226348_at	3.93E-02	0.18913285	FUT11	fucosyltransferase 11
224465_s_at	3.93E-02	0.17125666	PYM1	PYM homolog 1, exon junction complex associated factor
218262_at	3.93E-02	0.14680029	RMND5B	required for meiotic nuclear division 5 homolog B
204455_at	3.94E-02	-0.29263182	DST	dystonin
231776_at	3.94E-02	-0.13510848	EOMES	eomesodermin
209654_at	3.94E-02	-0.15191352	ICE1	interactor of little elongation complex ELL subunit 1
227029_at	3.94E-02	-0.24718126	LOC101927178///FAM177A1	uncharacterized LOC101927178///family with sequence similarity 177 member A1
1556500_a_at	3.94E-02	-0.05624178	LOC105374989	uncharacterized LOC105374989
206001_at	3.94E-02	0.0740739	NPY	neuropeptide Y
216102_at	3.94E-02	0.08075175	PHLDB1	pleckstrin homology like domain family B member 1
227945_at	3.94E-02	-0.23824967	TBC1D1	TBC1 domain family member 1
220968_s_at	3.94E-02	0.10119724	TSPAN9	tetraspanin 9
241613_at	3.94E-02	-0.15703288		
216703_at	3.94E-02	-0.0510791		
239464_at	3.95E-02	0.0794675	KCNJ15	potassium voltage-gated channel subfamily J member 15
233069_at	3.95E-02	0.07936395	PPP4R1L	protein phosphatase 4 regulatory subunit 1 like (pseudogene)
220368_s_at	3.95E-02	-0.20132532	PPP4R3A	protein phosphatase 4 regulatory subunit 3A
215458_s_at	3.95E-02	0.10834426	SMURF1	SMAD specific E3 ubiquitin protein ligase 1
244638_at	3.95E-02	0.06274678	SUCLG1	succinate-CoA ligase alpha subunit

226746_s_at	3.95E-02	-0.11324006	UBE4B	ubiquitination factor E4B
226514_at	3.95E-02	0.12705749	ZNF71	zinc finger protein 71
240505_at	3.95E-02	-0.1101602		
1561506_at	3.96E-02	0.06070572	BARX1-AS1	BARX1 antisense RNA 1 (head to head)
1552686_at	3.96E-02	0.07599337	CNBD2	cyclic nucleotide binding domain containing 2
239477_at	3.96E-02	0.09783004	EFHB	EF-hand domain family member B
229555_at	3.96E-02	0.30571292	GALNT5	polypeptide N-acetylgalactosaminyltransferase 5
221947_at	3.96E-02	0.0806092	IL17RC	interleukin 17 receptor C
226874_at	3.96E-02	-0.22244532	KLHL8	kelch like family member 8
218946_at	3.96E-02	-0.17256748	NFU1	NFU1 iron-sulfur cluster scaffold
202598_at	3.96E-02	0.32100453	S100A13	S100 calcium binding protein A13
225675_at	3.96E-02	-0.21675729	TMEM260	transmembrane protein 260
214142_at	3.96E-02	-0.34743116	ZG16	zymogen granule protein 16
1556657_at	3.96E-02	-0.11646975		
223094_s_at	3.97E-02	0.17812981	ANKH	ANKH inorganic pyrophosphate transport regulator
220941_s_at	3.97E-02	-0.20669363	C21orf91	chromosome 21 open reading frame 91
222781_s_at	3.97E-02	-0.24628142	C9orf40	chromosome 9 open reading frame 40
203784_s_at	3.97E-02	0.08123275	DDX28	DEAD-box helicase 28
230568_x_at	3.97E-02	-0.07373029	DLL3	delta like canonical Notch ligand 3
1557121_s_at	3.97E-02	-0.20089235	HEATR6	HEAT repeat containing 6
1564653_s_at	3.97E-02	-0.16225846	LEKR1	leucine, glutamate and lysine rich 1
227982_at	3.97E-02	-0.26238198	SEPSECS	Sep (O-phosphoserine) tRNA:Sec (selenocysteine) tRNA synthase
215856_at	3.97E-02	0.16782768	SIGLEC15	sialic acid binding Ig like lectin 15
210291_s_at	3.97E-02	0.10826349	ZNF174	zinc finger protein 174
237627_at	3.97E-02	-0.12449572		
213995_at	3.98E-02	-0.26667762	ATP5S	ATP synthase, H+ transporting, mitochondrial Fo complex subunit s (factor B)
205052_at	3.98E-02	-0.13798571	AUH	AU RNA binding methylglutaconyl-CoA hydratase
232611_at	3.98E-02	-0.16653426	GOLGA2P5	golgin A2 pseudogene 5
239950_at	3.98E-02	0.27546613	HOXA11-AS	HOXA11 antisense RNA
218893_at	3.98E-02	0.15089471	ISOC2	isochorismatase domain containing 2
222415_at	3.98E-02	-0.19027532	KMT2C	lysine methyltransferase 2C
231364_at	3.98E-02	0.08626493	NFIA-AS2	NFIA antisense RNA 2
214083_at	3.98E-02	-0.21242664	PPP2R5C	protein phosphatase 2 regulatory subunit B'gamma
209852_x_at	3.98E-02	0.1828503	PSME3	proteasome activator subunit 3
230842_at	3.98E-02	0.0700057		
217419_x_at	3.99E-02	0.17141313	AGRN	agrin
225486_at	3.99E-02	-0.2391326	ARID2	AT-rich interaction domain 2
208688_x_at	3.99E-02	0.21892387	EIF3B	eukaryotic translation initiation factor 3 subunit B
225991_at	3.99E-02	0.13999932	TMEM41A	transmembrane protein 41A
226376_at	3.99E-02	-0.07402367	UNK	unkempt family zinc finger
244013_at	3.99E-02	-0.09181943		
209620_s_at	4.00E-02	-0.22041577	ABCB7	ATP binding cassette subfamily B member 7
244355_at	4.00E-02	0.09147596	AVL9	AVL9 cell migration associated
211501_s_at	4.00E-02	0.25823049	EIF3B	eukaryotic translation initiation factor 3 subunit B
48612_at	4.00E-02	0.09698594	N4BP1	NEDD4 binding protein 1
1557444_at	4.00E-02	0.07728975	TREML3P	triggering receptor expressed on myeloid cells like 3, pseudogene
209196_at	4.00E-02	0.21165797	WDR46	WD repeat domain 46
241579_at	4.00E-02	-0.05792399		
242326_at	4.01E-02	0.09534545	COL22A1	collagen type XXII alpha 1 chain
1557192_at	4.01E-02	-0.18214722	COX10-AS1	COX10 antisense RNA 1
206560_s_at	4.01E-02	0.31850032	MIA	melanoma inhibitory activity
217871_s_at	4.01E-02	0.34140719	MIF	macrophage migration inhibitory factor (glycosylation-inhibiting factor)
214268_s_at	4.01E-02	-0.19878925	MTMR4	myotubularin related protein 4
203814_s_at	4.01E-02	0.31353748	NQO2	NAD(P)H quinone dehydrogenase 2
210830_s_at	4.01E-02	0.23904197	PON2	paraoxonase 2
221495_s_at	4.01E-02	0.22212123	TCF25	transcription factor 25
233450_at	4.01E-02	-0.19644948		
211560_s_at	4.02E-02	0.07366651	ALAS2	5'-aminolevulinate synthase 2
208622_s_at	4.02E-02	0.22114879	EZR	ezrin

224805_s_at	4.02E-02	0.06371093	FAM219B	family with sequence similarity 219 member B
213937_s_at	4.02E-02	0.17602659	FTSJ1	FtsJ RNA methyltransferase homolog 1 (E. coli)
1552343_s_at	4.02E-02	-0.16441541	PDE7A	phosphodiesterase 7A
1552827_s_at	4.02E-02	-0.06530468	SLC26A7	solute carrier family 26 member 7
239216_at	4.02E-02	0.06549284	TEKT1	tektin 1
233350_s_at	4.02E-02	0.202877	TEX264	testis expressed 264
225235_at	4.02E-02	0.13626113	TSPAN17	tetraspanin 17
237698_at	4.02E-02	0.06146858		
239571_at	4.02E-02	-0.21264141		
206469_x_at	4.03E-02	0.25432024	AKR7A3	aldo-keto reductase family 7 member A3
227265_at	4.03E-02	-0.45742266	FGL2	fibrinogen like 2
202061_s_at	4.03E-02	-0.18181407	SEL1L	SEL1L ERAD E3 ligase adaptor subunit
211841_s_at	4.03E-02	0.1690251	TNFRSF25	TNF receptor superfamily member 25
1554281_at	4.03E-02	0.20889098		
216295_s_at	4.04E-02	0.15660506	CLTA	clathrin light chain A
1553801_a_at	4.04E-02	-0.20581791	DTD2	D-tyrosyl-tRNA deacylase 2 (putative)
241611_s_at	4.04E-02	0.08650389	FNDC3A	fibronectin type III domain containing 3A
209626_s_at	4.04E-02	0.22205181	OSBPL3	oxysterol binding protein like 3
235004_at	4.04E-02	-0.5053485	RBM24	RNA binding motif protein 24
1553045_at	4.04E-02	0.0507322	WNT9A	Wnt family member 9A
233653_at	4.04E-02	-0.04280771		
239978_at	4.04E-02	-0.23998244		
235963_at	4.04E-02	-0.06020192		
1562380_at	4.04E-02	0.05647562		
243972_at	4.05E-02	-0.05429569	BTF3L4	basic transcription factor 3 like 4
203331_s_at	4.05E-02	0.08125929	INPP5D	inositol polyphosphate-5-phosphatase D
222468_at	4.05E-02	0.189964	KIAA0319L	KIAA0319 like
1570402_at	4.05E-02	0.09523472	KLC3	kinesin light chain 3
1558166_at	4.05E-02	-0.07685352	MGC16275	uncharacterized protein MGC16275
232321_at	4.05E-02	0.79155553	MUC17	mucin 17, cell surface associated
215273_s_at	4.05E-02	0.15580262	TADA3	transcriptional adaptor 3
233369_at	4.05E-02	-0.25668327		
240790_at	4.05E-02	0.06191232		
243420_at	4.05E-02	0.07398928		
220674_at	4.06E-02	-0.06365244	CD22	CD22 molecule
213823_at	4.06E-02	0.41266414	HOXA11	homeobox A11
211948_x_at	4.06E-02	0.15843451	PRRC2C	proline rich coiled-coil 2C
203114_at	4.06E-02	0.25296886	SSSCA1	Sjogren syndrome/scleroderma autoantigen 1
1553488_at	4.06E-02	0.08434715	TEKT5	tektin 5
202137_s_at	4.06E-02	-0.16365396	ZMYND11	zinc finger MYND-type containing 11
1556090_at	4.06E-02	0.11300359		
1555416_a_at	4.07E-02	0.06250509	ALOX15B	arachidonate 15-lipoxygenase, type B
1564251_at	4.07E-02	0.07225525	EMID1	EMI domain containing 1
230156_x_at	4.07E-02	-0.11823753	LINC01578	long intergenic non-protein coding RNA 1578
227933_at	4.07E-02	-0.09605356	LINGO1	leucine rich repeat and Ig domain containing 1
236451_at	4.07E-02	-0.38555409	LOC100996579	uncharacterized LOC100996579
217453_at	4.07E-02	-0.06128655	LOC729815	uncharacterized LOC729815
221807_s_at	4.07E-02	0.17161663	TRABD	TraB domain containing
237001_at	4.07E-02	-0.24371713		
1561777_at	4.07E-02	0.04903316		
213757_at	4.07E-02	-0.21252854		
1557325_at	4.07E-02	0.09651894		
223695_s_at	4.08E-02	-0.36881972	ARSD	arylsulfatase D
203416_at	4.08E-02	-0.28607313	CD53	CD53 molecule
1555989_at	4.08E-02	-0.38150682	DAAM1	dishevelled associated activator of morphogenesis 1
235175_at	4.08E-02	-0.25895087	GBP4	guanylate binding protein 4
1565406_a_at	4.08E-02	0.08301676	LHX9	LIM homeobox 9
236653_at	4.08E-02	0.1203449	LINC00662	long intergenic non-protein coding RNA 662
244424_at	4.08E-02	0.12692354	LOC439938	uncharacterized LOC439938
208714_at	4.08E-02	0.22967112	NDUFV1	NADH:ubiquinone oxidoreductase core subunit V1

220922_s_at	4.08E-02	0.08130118	SPANXA2///SPANXB1///SPANXC///SPANXA1	SPANX family member A2///SPANX family member B1///SPANX family member C///sperm protein associated with the nucleus, X-linked, family member A1
1559298_a_at	4.08E-02	0.06465911	SPATA41	spermatogenesis associated 41 (non-protein coding)
213200_at	4.08E-02	-0.08330446	SYP	synaptophysin
1558481_s_at	4.08E-02	-0.0671125	TMCC1-AS1	TMCC1 antisense RNA 1 (head to head)
227906_s_at	4.08E-02	-0.07304259		
235255_at	4.09E-02	-0.10625744	ATP6V0A2	ATPase H+ transporting V0 subunit a2
233366_at	4.09E-02	-0.09376012	FBXO4	F-box protein 4
212499_s_at	4.09E-02	-0.16136497	MAPK1IP1L///FCF1	mitogen-activated protein kinase 1 interacting protein 1 like///FCF1 rRNA-processing protein
203737_s_at	4.09E-02	0.15415559	PPRC1	peroxisome proliferator-activated receptor gamma, coactivator-related 1
204402_at	4.09E-02	0.13607784	RHBDD3	rhomboid domain containing 3
242814_at	4.09E-02	-0.21915872	SERPINB9	serpin family B member 9
1561923_a_at	4.09E-02	0.0858649	SF3B6	splicing factor 3b subunit 6
232180_at	4.09E-02	-0.19492089	UGP2	UDP-glucose pyrophosphorylase 2
238643_at	4.09E-02	0.09042648		
200045_at	4.10E-02	0.20234358	ABCF1	ATP binding cassette subfamily F member 1
230872_s_at	4.10E-02	0.10400249	ARPC4-TTLL3///TTLL3///ARPC4	ARPC4-TTLL3 readthrough///tubulin tyrosine ligase like 3///actin related protein 2/3 complex subunit 4
225555_x_at	4.10E-02	-0.10226353	AURKAIP1	aurora kinase A interacting protein 1
222885_at	4.10E-02	-0.24933429	EMCN	endomucin
203723_at	4.10E-02	0.17661616	ITPKB	inositol-trisphosphate 3-kinase B
243762_at	4.10E-02	-0.08185058	LINC01297	long intergenic non-protein coding RNA 1297
203713_s_at	4.10E-02	0.21885871	LLGL2	LLGL2, scribble cell polarity complex component
211037_s_at	4.10E-02	0.14435402	MBOAT7	membrane bound O-acyltransferase domain containing 7
213897_s_at	4.10E-02	0.25744706	MRPL23	mitochondrial ribosomal protein L23
212621_at	4.10E-02	-0.20216928	NEMP1	nuclear envelope integral membrane protein 1
229081_at	4.10E-02	-0.07557547	SLC25A13	solute carrier family 25 member 13
208015_at	4.10E-02	-0.19549501	SMAD1	SMAD family member 1
211106_at	4.10E-02	-0.18966502	SUPT3H	SPT3 homolog, SAGA and STAGA complex component
226322_at	4.10E-02	-0.27348324	TMTC1	transmembrane and tetratricopeptide repeat containing 1
1558938_at	4.10E-02	-0.12662465		
226014_at	4.10E-02	-0.25060402		
218220_at	4.11E-02	0.25424704	C12orf10	chromosome 12 open reading frame 10
219599_at	4.11E-02	-0.29072146	EIF4B	eukaryotic translation initiation factor 4B
204805_s_at	4.11E-02	0.27232058	H1FX	H1 histone family member X
244485_at	4.11E-02	-0.0968268	HLA-DPB1	major histocompatibility complex, class II, DP beta 1
217933_s_at	4.11E-02	-0.21178903	LAP3	leucine aminopeptidase 3
210924_at	4.11E-02	-0.0786023	OLFM1	olfactomedin 1
1557993_at	4.11E-02	-0.05059952	PLCG2	phospholipase C gamma 2
208640_at	4.11E-02	0.12079767	RAC1	ras-related C3 botulinum toxin substrate 1 (rho family, small GTP binding protein Rac1)
219864_s_at	4.11E-02	0.09003925	RCAN3	RCAN family member 3
241365_at	4.11E-02	0.69151486	SATB1	SATB homeobox 1
227606_s_at	4.11E-02	0.22400863	STAMBPL1	STAM binding protein like 1
1553011_at	4.11E-02	-0.09372744	TAF1L	TATA-box binding protein associated factor 1 like
242163_at	4.11E-02	-0.17837694	THRAP3	thyroid hormone receptor associated protein 3
1566622_at	4.11E-02	0.05743124		
240195_at	4.11E-02	0.0615717		
231190_at	4.12E-02	-0.06887437	POM121L10P	POM121 transmembrane nucleoporin like 10, pseudogene
233232_at	4.12E-02	-0.07483811	RHBDL3	rhomboid like 3
232273_at	4.12E-02	0.06480634		
233853_at	4.12E-02	-0.04945444		
233717_x_at	4.12E-02	-0.05822088		
233292_s_at	4.13E-02	0.13653059	ANKHD1-EIF4EBP3///ANKHD1	ANKHD1-EIF4EBP3 readthrough///ankyrin repeat and KH domain containing 1
219546_at	4.13E-02	-0.19196262	BMP2K	BMP2 inducible kinase

220271_x_at	4.13E-02	0.07278094	EFCAB6	EF-hand calcium binding domain 6
202421_at	4.13E-02	0.24160921	IGSF3	immunoglobulin superfamily member 3
208164_s_at	4.13E-02	-0.07745842	IL9R	interleukin 9 receptor
243066_at	4.13E-02	0.06232132	NPL	N-acetylneuraminate pyruvate lyase
214620_x_at	4.13E-02	0.26756173	PAM	peptidylglycine alpha-amidating monooxygenase
205909_at	4.13E-02	-0.27400318	POLE2	DNA polymerase epsilon 2, accessory subunit
219897_at	4.13E-02	-0.09727701	RNF122	ring finger protein 122
1555875_at	4.13E-02	0.11998128	SRGAP1	SLIT-ROBO Rho GTPase activating protein 1
226108_at	4.13E-02	0.18718948	ZC3H18	zinc finger CCCH-type containing 18
216514_at	4.13E-02	-0.08326071		
238104_at	4.13E-02	-0.10182194		
231552_at	4.13E-02	-0.13602587		
232944_at	4.13E-02	0.07629011		
231831_at	4.14E-02	0.10086441	COX19	COX19, cytochrome c oxidase assembly factor
230393_at	4.14E-02	-0.18997029	CUL5	cullin 5
207300_s_at	4.14E-02	0.17918784	F7	coagulation factor VII
1556034_s_at	4.14E-02	0.26908935	MTMR11	myotubularin related protein 11
217698_at	4.14E-02	-0.04260354		
1561392_at	4.14E-02	0.06285952		
1561179_s_at	4.15E-02	0.07153752	AMZ1	archaelysin family metallopeptidase 1
224875_at	4.15E-02	-0.32664422	C5orf24	chromosome 5 open reading frame 24
204397_at	4.15E-02	0.07267016	EML2	echinoderm microtubule associated protein like 2
240430_at	4.15E-02	0.13950334	KCMF1	potassium channel modulatory factor 1
206408_at	4.15E-02	-0.09225275	LRRTM2	leucine rich repeat transmembrane neuronal 2
233801_s_at	4.15E-02	-0.09709145	SEMA6D	semaphorin 6D
242906_at	4.15E-02	-0.10801622	SESN3	sestrin 3
220921_at	4.15E-02	0.05431255	SPANXB1	SPANX family member B1
237143_at	4.15E-02	-0.06774301		
243890_at	4.15E-02	0.09652005		
215549_x_at	4.16E-02	0.20669846	CTAGE8///CTAGE4///CTAGE9	CTAGE family member 8///CTAGE family member 4///CTAGE family member 9
1555822_at	4.16E-02	0.06538661	FAM138E///FAM138D///FAM138C///FAM138B///FAM138A///FAM138F	family with sequence similarity 138 member E///family with sequence similarity 138 member D///family with sequence similarity 138 member C///family with sequence similarity 138 member B///family with sequence similarity 138 member A///family with sequence similarity 138 member F
1560227_at	4.16E-02	-0.07370365	GDPD1	glycerophosphodiester phosphodiesterase domain containing 1
1566127_at	4.16E-02	0.06091648	LIMS1	LIM zinc finger domain containing 1
201460_at	4.16E-02	0.14407209	MAPKAPK2	mitogen-activated protein kinase-activated protein kinase 2
239754_at	4.16E-02	0.08263963	SNORD65///SNORD49B///LRRC75A-AS1///SNORD49A	small nucleolar RNA, C/D box 65///small nucleolar RNA, C/D box 49B///LRRC75A antisense RNA 1///small nucleolar RNA, C/D box 49A
228837_at	4.16E-02	-0.13679189	TCF4	transcription factor 4
242936_at	4.16E-02	-0.07036131		
241929_at	4.16E-02	-0.05420452		
227691_at	4.17E-02	0.07504894	AKAP8L	A-kinase anchoring protein 8 like
215741_x_at	4.17E-02	0.06420514	AKAP8L	A-kinase anchoring protein 8 like
1560773_at	4.17E-02	-0.0542886	DLGAP1	DLG associated protein 1
218611_at	4.17E-02	0.27751781	IER5	immediate early response 5
234891_at	4.17E-02	0.06354439	LOC81787	uncharacterized LOC81787
226630_at	4.17E-02	-0.22752752	MIS18BP1	MIS18 binding protein 1
207017_at	4.17E-02	0.13100537	RAB27B	RAB27B, member RAS oncogene family
235037_at	4.17E-02	0.14157624	TMEM41A	transmembrane protein 41A
212620_at	4.17E-02	0.08880851	ZNF609	zinc finger protein 609
1555084_at	4.17E-02	0.07455346		
1567702_at	4.17E-02	0.03990477		
212540_at	4.18E-02	0.1919757	CDC34	cell division cycle 34
232204_at	4.18E-02	-0.07917747	EBF1	early B-cell factor 1
1563674_at	4.18E-02	-0.14777282	FCRL2	Fc receptor like 2
234294_x_at	4.18E-02	0.14966594	GATAD2A	GATA zinc finger domain containing 2A

228563_at	4.18E-02	-0.09911506	GJC1	gap junction protein gamma 1
227863_at	4.18E-02	0.11039634	IFITM10	interferon induced transmembrane protein 10
223725_at	4.18E-02	0.10346136	LINC00852	long intergenic non-protein coding RNA 852
214056_at	4.18E-02	-0.25447091	MCL1	BCL2 family apoptosis regulator
227624_at	4.18E-02	-0.22996967	TET2	tet methylcytosine dioxygenase 2
233573_s_at	4.18E-02	0.11365606	WDR6	WD repeat domain 6
232175_at	4.19E-02	0.15969147	ARF1	ADP ribosylation factor 1
202966_at	4.19E-02	0.3073274	CAPN6	calpain 6
204118_at	4.19E-02	-0.27754737	CD48	CD48 molecule
1563253_s_at	4.19E-02	0.10148993	ERBB3	erb-b2 receptor tyrosine kinase 3
201655_s_at	4.19E-02	0.15410926	HSPG2	heparan sulfate proteoglycan 2
214677_x_at	4.19E-02	-0.81701048	IGLC1	immunoglobulin lambda constant 1
1561401_at	4.19E-02	0.06814158	LOC285627	uncharacterized LOC285627
239467_at	4.19E-02	-0.08021848		
205081_at	4.20E-02	0.63438237	CRIP1	cysteine rich protein 1
202676_x_at	4.20E-02	0.14796368	FASTK	Fas activated serine/threonine kinase
222671_s_at	4.20E-02	0.14095282	JMJD4	jumonji domain containing 4
209207_s_at	4.20E-02	-0.16887555	LOC102724364///LOC100996517///SEC22B	vesicle-trafficking protein SEC22b-like///vesicle-trafficking protein SEC22b///SEC22 homolog B, vesicle trafficking protein (gene/pseudogene)
47550_at	4.20E-02	-0.13272699	LZTS1	leucine zipper tumor suppressor 1
1569150_x_at	4.20E-02	0.07829876	PDLIM7	PDZ and LIM domain 7
1555943_at	4.20E-02	0.15654913	PGAM5	PGAM family member 5, mitochondrial serine/threonine protein phosphatase
206793_at	4.20E-02	0.07756931	PNMT	phenylethanolamine N-methyltransferase
218083_at	4.20E-02	0.14838013	PTGES2	prostaglandin E synthase 2
221287_at	4.20E-02	0.10240511	RNASEL	ribonuclease L
207122_x_at	4.20E-02	0.17465576	SULT1A2	sulfotransferase family 1A member 2
238226_at	4.20E-02	0.12876851	TMEM255B	transmembrane protein 255B
228054_at	4.20E-02	0.18821991	TMEM44	transmembrane protein 44
225891_at	4.20E-02	0.14764761	TPRN	taperin
236898_at	4.20E-02	0.15775059		
237959_at	4.20E-02	-0.07198685		
205722_s_at	4.21E-02	-0.07144687	GFRA2	GDNF family receptor alpha 2
211870_s_at	4.21E-02	0.07545147	PCDHA2///PCDHA3	protocadherin alpha 2///protocadherin alpha 3
227580_s_at	4.21E-02	0.22112379	TECPR1	tectonin beta-propeller repeat containing 1
212423_at	4.21E-02	0.38588268	ZCCHC24	zinc finger CCHC-type containing 24
201880_at	4.22E-02	-0.1906535	ARIH1	ariadne RBR E3 ubiquitin protein ligase 1
222210_at	4.22E-02	0.07068779	MIR6785///TMEM94	microRNA 6785///transmembrane protein 94
217054_at	4.22E-02	-0.08434165	MUC3A	mucin 3A, cell surface associated
227206_at	4.22E-02	-0.06945354	NDUFA10	NADH:ubiquinone oxidoreductase subunit A10
1553209_at	4.22E-02	-0.07797442	RNFT2	ring finger protein, transmembrane 2
226028_at	4.22E-02	-0.1641461	ROBO4	roundabout guidance receptor 4
230093_at	4.22E-02	0.36296976	RSPH1	radial spoke head 1 homolog
235034_at	4.22E-02	0.08865625	VTI1A	vesicle transport through interaction with t-SNAREs 1A
243465_at	4.22E-02	-0.19650315		
214798_at	4.23E-02	0.23055957	ATP2C2	ATPase secretory pathway Ca2+ transporting 2
229454_at	4.23E-02	-0.22771023	BCLAF1	BCL2 associated transcription factor 1
219896_at	4.23E-02	-0.07552093	CALY	calcyon neuron specific vesicular protein
1554513_s_at	4.23E-02	0.09890762	CEP89	centrosomal protein 89
213570_at	4.23E-02	0.1739008	EIF4E2	eukaryotic translation initiation factor 4E family member 2
204948_s_at	4.23E-02	0.11658445	FST	follistatin
228167_at	4.23E-02	-0.31523806	KLHL6	kelch like family member 6
1565936_a_at	4.23E-02	-0.04605333	LMO3	LIM domain only 3
1563209_a_at	4.23E-02	0.30336543	MACROD2	MACRO domain containing 2
205090_s_at	4.23E-02	0.1746641	NAGPA	N-acetylglucosamine-1-phosphodiester alpha-N-acetylglucosaminidase
230673_at	4.23E-02	-0.10847685	PKHD1L1	polycystic kidney and hepatic disease 1 (autosomal recessive)-like 1
203761_at	4.23E-02	-0.22078664	SLA	Src-like-adaptor
1566339_at	4.23E-02	0.04491689	SNORD8	small nucleolar RNA, C/D box 8
204064_at	4.23E-02	-0.18156608	THOC1	THO complex 1

209387_s_at	4.23E-02	0.44601495	TM4SF1	transmembrane 4 L six family member 1
203702_s_at	4.23E-02	0.19621692	TTLL4	tubulin tyrosine ligase like 4
1553720_a_at	4.24E-02	0.0783198	AMER2	APC membrane recruitment protein 2
229313_at	4.24E-02	-0.54721347	ANO5	anoctamin 5
203952_at	4.24E-02	0.11933188	ATF6	activating transcription factor 6
231771_at	4.24E-02	0.27037851	GJB6	gap junction protein beta 6
203297_s_at	4.24E-02	-0.18827711	JARID2	jumonji and AT-rich interaction domain containing 2
204518_s_at	4.24E-02	0.30090182	PPIC	peptidylprolyl isomerase C
1557938_s_at	4.24E-02	0.08896417	PTRF	polymerase I and transcript release factor
224711_at	4.24E-02	0.18105149	YY1	YY1 transcription factor
1555117_at	4.24E-02	0.07764894		
220208_at	4.25E-02	0.07481574	ADAMTS13	ADAM metallopeptidase with thrombospondin type 1 motif 13
232197_x_at	4.25E-02	-0.17241246	ARSB	arylsulfatase B
210137_s_at	4.25E-02	0.22327419	DCTD	dCMP deaminase
218135_at	4.25E-02	-0.20504348	ERGIC2	ERGIC and golgi 2
205380_at	4.25E-02	0.58133462	PDZK1	PDZ domain containing 1
224857_s_at	4.25E-02	0.22123569	POLR1D	RNA polymerase I subunit D
50221_at	4.25E-02	0.1852721	TFEB	transcription factor EB
229983_at	4.25E-02	-0.1995527	TIGD2	tigger transposable element derived 2
236676_at	4.25E-02	0.07621526		
218395_at	4.26E-02	-0.24421862	ACTR6	ARP6 actin-related protein 6 homolog
203566_s_at	4.26E-02	-0.20621071	AGL	amylo-alpha-1, 6-glucosidase, 4-alpha-glucanotransferase
202931_x_at	4.26E-02	0.27160534	BIN1	bridging integrator 1
1553438_at	4.26E-02	0.07489518	C11orf72	chromosome 11 open reading frame 72
234963_s_at	4.26E-02	0.08223386	FA2H	fatty acid 2-hydroxylase
202270_at	4.26E-02	-0.37071383	GBP1	guanylate binding protein 1
222997_s_at	4.26E-02	0.49338574	MRPS21	mitochondrial ribosomal protein S21
208337_s_at	4.26E-02	0.13291687	NR5A2	nuclear receptor subfamily 5 group A member 2
219658_at	4.26E-02	-0.18467075	PTCD2	pentatricopeptide repeat domain 2
236231_at	4.26E-02	-0.13138278	ZNF271P	zinc finger protein 271, pseudogene
244697_at	4.26E-02	-0.08089989		
244812_at	4.26E-02	0.06263911		
244323_at	4.27E-02	0.05642485	BHLHE22	basic helix-loop-helix family member e22
202574_s_at	4.27E-02	0.12704652	CSNK1G2	casein kinase 1 gamma 2
221509_at	4.27E-02	-0.130692	DENR	density regulated re-initiation and release factor
219424_at	4.27E-02	-0.08992882	EBI3	Epstein-Barr virus induced 3
230031_at	4.27E-02	0.19241625	HSPA5	heat shock protein family A (Hsp70) member 5
227587_at	4.27E-02	0.13366108	KRI1	KRI1 homolog
216648_s_at	4.27E-02	0.07669884	RREB1	ras responsive element binding protein 1
223179_at	4.27E-02	0.20157144	YPEL3	yippee like 3
1561268_at	4.27E-02	0.06994637		
1561989_at	4.27E-02	0.07664707		
229366_at	4.27E-02	-0.22602193		
202968_s_at	4.28E-02	0.14988534	DYRK2	dual specificity tyrosine phosphorylation regulated kinase 2
235813_at	4.28E-02	-0.08506501	LOC101927027	uncharacterized LOC101927027
227993_at	4.28E-02	-0.21062976	METAP2	methionyl aminopeptidase 2
223292_s_at	4.28E-02	-0.2940075	MRPS15	mitochondrial ribosomal protein S15
225237_s_at	4.28E-02	0.19978148	MSI2	musashi RNA binding protein 2
201455_s_at	4.28E-02	-0.18301105	NPEPPS	aminopeptidase puromycin sensitive
225592_at	4.28E-02	0.1531922	NRM	nurim (nuclear envelope membrane protein)
239512_at	4.28E-02	-0.07357663	SRSF4	serine and arginine rich splicing factor 4
223768_at	4.28E-02	0.08501212	SSR4P1	signal sequence receptor subunit 4 pseudogene 1
1563983_at	4.28E-02	0.06778406	TPT1-AS1	TPT1 antisense RNA 1
201083_s_at	4.29E-02	-0.1847337	BCLAF1	BCL2 associated transcription factor 1
225644_at	4.29E-02	-0.21754791	CCDC117	coiled-coil domain containing 117
243459_x_at	4.29E-02	0.06746116	CDK14	cyclin dependent kinase 14
209283_at	4.29E-02	0.14638644	CRYAB	crystallin alpha B
211863_x_at	4.29E-02	0.0872107	HFE	hemochromatosis
1560787_at	4.29E-02	0.0727315	LOC101929143	uncharacterized LOC101929143
1552885_a_at	4.29E-02	0.08078697	NKX6-3	NK6 homeobox 3

203401_at	4.29E-02	-0.21769756	PRPS2	phosphoribosyl pyrophosphate synthetase 2
238616_at	4.29E-02	0.08304791	QDPR	quinoid dihydropteridine reductase
1554181_at	4.29E-02	0.08089305	SNX32	sorting nexin 32
1554016_a_at	4.29E-02	0.13724819	USB1	U6 snRNA biogenesis phosphodiesterase 1
231988_x_at	4.29E-02	0.0941014	ZNF490	zinc finger protein 490
234920_at	4.29E-02	-0.0610852	ZNF7	zinc finger protein 7
217333_at	4.29E-02	0.06904728		
1558706_a_at	4.30E-02	-0.07085141	ATOH8	atonal bHLH transcription factor 8
202411_at	4.30E-02	0.4119553	IFI27	interferon alpha inducible protein 27
1559929_at	4.30E-02	-0.13229674	LINC01186	long intergenic non-protein coding RNA 1186
223012_at	4.30E-02	0.15419321	MIR4746///UBXN6	microRNA 4746///UBX domain protein 6
223358_s_at	4.30E-02	-0.25586596	PDE7A	phosphodiesterase 7A
1565604_at	4.30E-02	0.05851165	PWP2	PWP2 periodic tryptophan protein homolog (yeast)
212617_at	4.30E-02	0.12909633	ZNF609	zinc finger protein 609
236210_at	4.30E-02	-0.1569136		
238883_at	4.30E-02	-0.22923162		
236685_at	4.30E-02	-0.17701398		
235275_at	4.31E-02	-0.21730388	BMP8B	bone morphogenetic protein 8b
214832_at	4.31E-02	0.096627	HNF4A	hepatocyte nuclear factor 4 alpha
241476_at	4.31E-02	0.07085748	LOC105375115	uncharacterized LOC105375115
228594_at	4.31E-02	-0.19062378	NADK2	NAD kinase 2, mitochondrial
225510_at	4.31E-02	0.18437679	OAF	out at first homolog
227903_x_at	4.31E-02	0.30823235	TPGS1	tubulin polyglutamylase complex subunit 1
207281_x_at	4.31E-02	-0.07722503	VCX2	variable charge, X-linked 2
231251_at	4.31E-02	-0.18691812	WIPF2	WAS/WASL interacting protein family member 2
235203_at	4.31E-02	-0.29404073		
216214_at	4.31E-02	-0.06442527		
236567_at	4.31E-02	0.06325632		
217554_at	4.31E-02	-0.16705862		
242839_at	4.31E-02	-0.22063125		
238825_at	4.32E-02	-0.28093394	ACRC	acidic repeat containing
211475_s_at	4.32E-02	0.20714551	BAG1	BCL2 associated athanogene 1
213940_s_at	4.32E-02	-0.21394462	FNBP1	formin binding protein 1
235319_at	4.32E-02	-0.06155741	LINC01004	long intergenic non-protein coding RNA 1004
230433_at	4.32E-02	-0.20005309	LOC729970	hCG2028352-like
204484_at	4.32E-02	0.1690492	PIK3C2B	phosphatidylinositol-4-phosphate 3-kinase catalytic subunit type 2 beta
211546_x_at	4.32E-02	0.06923699	SNCA	synuclein alpha
201622_at	4.32E-02	0.15001579	SND1	staphylococcal nuclease and tudor domain containing 1
217126_at	4.32E-02	-0.04537213		
1565484_x_at	4.33E-02	-0.24768357	EGFR	epidermal growth factor receptor
206903_at	4.33E-02	0.05607614	EXOG	endo/exonuclease (5'-3'), endonuclease G-like
201188_s_at	4.33E-02	0.20374063	ITPR3	inositol 1,4,5-trisphosphate receptor type 3
206584_at	4.33E-02	-0.42308042	LY96	lymphocyte antigen 96
224044_at	4.33E-02	-0.11249842	RHOT1	ras homolog family member T1
207557_s_at	4.33E-02	0.09102719	RYR2	ryanodine receptor 2
204915_s_at	4.33E-02	0.10614933	SOX11	SRY-box 11
1568867_x_at	4.33E-02	-0.05543578		
243667_at	4.33E-02	-0.1524079		
1562297_at	4.33E-02	-0.05241434		
205392_s_at	4.34E-02	0.13547815	CCL15-CCL14///CCL14	CCL15-CCL14 readthrough (NMD candidate)///C-C motif chemokine ligand 14
207993_s_at	4.34E-02	0.14101917	CHP1	calcineurin like EF-hand protein 1
1555318_at	4.34E-02	0.06865559	HIF3A	hypoxia inducible factor 3 alpha subunit
209453_at	4.34E-02	0.16311652	SLC9A1	solute carrier family 9 member A1
219462_at	4.34E-02	0.17484169	TMEM53	transmembrane protein 53
202702_at	4.34E-02	0.14008836	TRIM26	tripartite motif containing 26
204704_s_at	4.35E-02	0.3121998	ALDOB	aldolase, fructose-bisphosphate B
1568954_s_at	4.35E-02	0.18869534	C16orf72	chromosome 16 open reading frame 72
220132_s_at	4.35E-02	-0.09001903	CLEC2D	C-type lectin domain family 2 member D
219767_s_at	4.35E-02	-0.18701704	CRYZL1	crystallin zeta like 1
210983_s_at	4.35E-02	0.34299539	MCM7	minichromosome maintenance complex component 7
221341_s_at	4.35E-02	0.07873821	OR1D5	olfactory receptor family 1 subfamily D member 5

226133_s_at	4.35E-02	0.12575902	TBC1D10A	TBC1 domain family member 10A
226408_at	4.35E-02	0.30553108	TEAD2	TEA domain transcription factor 2
1563085_at	4.35E-02	-0.05952851		
236776_at	4.35E-02	-0.08491676		
1562906_at	4.36E-02	0.05689344	FAM170A	family with sequence similarity 170 member A
214737_x_at	4.36E-02	0.13789958	HNRNPC	heterogeneous nuclear ribonucleoprotein C (C1/C2)
212454_x_at	4.36E-02	-0.14111831	HNRNPDL	heterogeneous nuclear ribonucleoprotein D like
1560337_at	4.36E-02	-0.05847466	LINC01289	long intergenic non-protein coding RNA 1289
214004_s_at	4.36E-02	0.169739	VGLL4	vestigial like family member 4
223850_at	4.36E-02	-0.06748443		
233091_at	4.37E-02	0.07729489	ATAD3B	ATPase family, AAA domain containing 3B
204515_at	4.37E-02	0.31376185	HSD3B1	hydroxy-delta-5-steroid dehydrogenase, 3 beta- and steroid delta-isomerase 1
228127_at	4.37E-02	0.07440946	KCNK3	potassium two pore domain channel subfamily K member 3
1570202_a_at	4.37E-02	0.05335666	MKL2	MKL1/myocardin like 2
1554428_s_at	4.37E-02	0.10769586	NLGN2	neuroligin 2
200830_at	4.37E-02	0.17331646	PSMD2	proteasome 26S subunit, non-ATPase 2
220439_at	4.37E-02	0.06593746	RIN3	Ras and Rab interactor 3
41657_at	4.37E-02	0.08747854	STK11	serine/threonine kinase 11
239389_at	4.37E-02	-0.09281012	TARBP2	TARBP2, RISC loading complex RNA binding subunit
210527_x_at	4.37E-02	0.10617746	TUBA3D///TUBA3C	tubulin alpha 3d///tubulin alpha 3c
235962_at	4.38E-02	-0.1469433	AZI2	5-azacytidine induced 2
224876_at	4.38E-02	-0.14545832	C5orf24	chromosome 5 open reading frame 24
1565657_at	4.38E-02	-0.03779049	CMTM6	CKLF like MARVEL transmembrane domain containing 6
230588_s_at	4.38E-02	-0.23275992	LOC730268///LOC285074	anaphase-promoting complex subunit 1-like///anaphase promoting complex subunit 1 pseudogene
1564897_at	4.38E-02	0.0934213	TNR	tenascin R
1563509_at	4.38E-02	-0.07753714		
234620_at	4.38E-02	0.06875303		
237199_at	4.38E-02	0.10125158		
202480_s_at	4.39E-02	0.10906567	DEDD	death effector domain containing
211426_x_at	4.39E-02	0.08942868	GNAQ	G protein subunit alpha q
213803_at	4.39E-02	-0.24995052	KPNB1	karyopherin subunit beta 1
229193_at	4.39E-02	-0.30853253	LUC7L3	LUC7 like 3 pre-mRNA splicing factor
221318_at	4.39E-02	0.0944141	NEUROD4	neuronal differentiation 4
206220_s_at	4.39E-02	0.1132103	RASA3	RAS p21 protein activator 3
233781_s_at	4.39E-02	-0.11428914	RIF1	replication timing regulatory factor 1
207735_at	4.39E-02	-0.14935892	RNF125	ring finger protein 125
224503_s_at	4.39E-02	0.15046953	ZCCHC2	zinc finger CCHC-type containing 2
210201_x_at	4.40E-02	0.2466708	BIN1	bridging integrator 1
239260_at	4.40E-02	0.10963029	CORIN	corin, serine peptidase
214333_x_at	4.40E-02	0.1522451	IDH3G	isocitrate dehydrogenase 3 (NAD(+)) gamma
201771_at	4.40E-02	0.18099085	SCAMP3	secretory carrier membrane protein 3
1554311_a_at	4.40E-02	-0.05351788	SUPT6H	SPT6 homolog, histone chaperone
1563357_at	4.40E-02	-0.20060953		
210082_at	4.41E-02	0.10596588	ABCA4	ATP binding cassette subfamily A member 4
226751_at	4.41E-02	-0.19186647	CNRIP1	cannabinoid receptor interacting protein 1
209522_s_at	4.41E-02	0.32574082	CRAT	carnitine O-acetyltransferase
209407_s_at	4.41E-02	0.10119793	DEAF1	DEAF1, transcription factor
231980_at	4.41E-02	-0.07053777	DOK6	docking protein 6
219267_at	4.41E-02	-0.1820183	GLTP	glycolipid transfer protein
234610_at	4.41E-02	0.06887521	HSPA12B	heat shock protein family A (Hsp70) member 12B
204117_at	4.41E-02	0.21145558	PREP	prolyl endopeptidase
232751_at	4.41E-02	0.11160952	RBBP9	RB binding protein 9, serine hydrolase
204468_s_at	4.41E-02	0.10004216	TIE1	tyrosine kinase with immunoglobulin like and EGF like domains 1
204523_at	4.41E-02	-0.20932853	ZNF140	zinc finger protein 140
1557240_a_at	4.41E-02	-0.24900871		
1569040_s_at	4.42E-02	-0.55055274	ANKRD36BP2	ankyrin repeat domain 36B pseudogene 2
229005_at	4.42E-02	-0.19831409	MCTP2	multiple C2 and transmembrane domain containing 2
243296_at	4.42E-02	-0.34925111	NAMPT	nicotinamide phosphoribosyltransferase

212553_at	4.42E-02	0.1488778	RPRD2	regulation of nuclear pre-mRNA domain containing 2
233167_at	4.42E-02	0.29896562	SELO	selenoprotein O
201793_x_at	4.42E-02	0.14707355	SMG7	SMG7, nonsense mediated mRNA decay factor
211705_s_at	4.42E-02	0.0635187	SORBS1	sorbin and SH3 domain containing 1
1555423_at	4.42E-02	-0.05612638	SSH2	slingshot protein phosphatase 2
232322_x_at	4.42E-02	0.27589323	STARD10	StAR related lipid transfer domain containing 10
236049_at	4.42E-02	0.05366188	WDR90	WD repeat domain 90
1560422_at	4.42E-02	-0.05916703		
241773_at	4.42E-02	-0.15158436		
225617_at	4.43E-02	0.21017656	ODF2	outer dense fiber of sperm tails 2
208361_s_at	4.43E-02	0.13396363	POLR3D	RNA polymerase III subunit D
1562447_a_at	4.43E-02	-0.07806807	PPP1R26-AS1	PPP1R26 antisense RNA 1
200763_s_at	4.43E-02	0.1247792	RPLP1	ribosomal protein lateral stalk subunit P1
1557575_at	4.43E-02	0.07817869	STBD1	starch binding domain 1
244108_at	4.43E-02	0.09829881	SYNPO2	synaptopodin 2
219620_x_at	4.43E-02	0.27531925	TOR4A	torsin family 4 member A
221229_s_at	4.43E-02	-0.15845409	TRMT61B	tRNA methyltransferase 61B
223314_at	4.43E-02	0.17834082	TSPAN14	tetraspanin 14
212320_at	4.43E-02	0.22250405	TUBB	tubulin beta class I
202706_s_at	4.43E-02	0.15228287	UMPS	uridine monophosphate synthetase
232850_at	4.43E-02	-0.24671661		
201715_s_at	4.44E-02	0.17297695	ACIN1	apoptotic chromatin condensation inducer 1
231549_at	4.44E-02	-0.08083947	C1orf158	chromosome 1 open reading frame 158
1555636_at	4.44E-02	-0.05761217	CD300LG	CD300 molecule like family member g
214447_at	4.44E-02	0.0636399	ETS1	ETS proto-oncogene 1, transcription factor
233971_at	4.44E-02	-0.13821725	FAM166A	family with sequence similarity 166 member A
206296_x_at	4.44E-02	-0.12558963	MAP4K1	mitogen-activated protein kinase kinase kinase kinase 1
223439_at	4.44E-02	-0.20767905	NKAP	NFKB activating protein
221348_at	4.44E-02	0.06866852	NPPC	natriuretic peptide C
212790_x_at	4.44E-02	0.09923753	RPL13AP5///SNORD32A///SNORD33///SNORD34///SNORD35A///RPL13A	ribosomal protein L13a pseudogene 5///small nucleolar RNA, C/D box 32A///small nucleolar RNA, C/D box 33///small nucleolar RNA, C/D box 34///small nucleolar RNA, C/D box 35A///ribosomal protein L13a
213437_at	4.44E-02	-0.16600354	RUFY3	RUN and FYVE domain containing 3
242356_at	4.44E-02	-0.21926538	VTI1A	vesicle transport through interaction with t-SNAREs 1A
1570143_at	4.44E-02	-0.25991134		
219902_at	4.45E-02	0.05372485	BHMT2	betaine--homocysteine S-methyltransferase 2
233063_s_at	4.45E-02	-0.20223504	GMDS-AS1	GMDS antisense RNA 1 (head to head)
217371_s_at	4.45E-02	-0.22274673	IL15	interleukin 15
207582_at	4.45E-02	0.07326402	PIN1P1	peptidylprolyl cis/trans isomerase, NIMA-interacting 1 pseudogene 1
223109_at	4.45E-02	0.126163	TRUB2	TruB pseudouridine synthase family member 2
237876_at	4.45E-02	-0.0534598		
241855_s_at	4.45E-02	-0.07929644		
1566703_at	4.45E-02	-0.05014847		
213709_at	4.46E-02	-0.25738302	BHLHB9	basic helix-loop-helix domain containing, class B, 9
204851_s_at	4.46E-02	0.05097403	DCX	doublecortin
228376_at	4.46E-02	-0.20817065	GGTA1P	glycoprotein, alpha-galactosyltransferase 1 pseudogene
1561355_at	4.46E-02	0.08419966	LOC692247	uncharacterized LOC692247
234875_at	4.46E-02	-0.12830475	RPL7AP10	ribosomal protein L7a pseudogene 10
1570507_at	4.46E-02	-0.12356316	SCAF11	SR-related CTD associated factor 11
200924_s_at	4.46E-02	0.20577898	SLC3A2	solute carrier family 3 member 2
1568249_at	4.46E-02	-0.20163653	SNHG17///SNORA71B	small nucleolar RNA host gene 17///small nucleolar RNA, H/ACA box 71B
1555374_at	4.46E-02	0.08322467	TTL	tubulin tyrosine ligase
237746_at	4.46E-02	-0.05599633		
205180_s_at	4.47E-02	0.18183573	ADAM8	ADAM metallopeptidase domain 8
226721_at	4.47E-02	-0.25853838	DPY19L4	dpy-19 like 4 (C. elegans)
1555908_at	4.47E-02	-0.17379741	FAM120A	family with sequence similarity 120A
239090_at	4.47E-02	-0.08400613	FAM161A	family with sequence similarity 161 member A
225988_at	4.47E-02	-0.15140727	HERC4	HECT and RLD domain containing E3 ubiquitin protein ligase 4
209575_at	4.47E-02	0.25758169	IL10RB	interleukin 10 receptor subunit beta

208931_s_at	4.47E-02	0.23998364	ILF3	interleukin enhancer binding factor 3
219245_s_at	4.47E-02	0.16696172	OGFOD2	2-oxoglutarate and iron dependent oxygenase domain containing 2
218420_s_at	4.47E-02	0.2878297	PROSER1	proline and serine rich 1
244402_at	4.47E-02	-0.07175313		
243460_at	4.47E-02	-0.05720245		
237427_at	4.47E-02	0.2711056		
237148_at	4.47E-02	0.05607403		
223183_at	4.48E-02	0.19863747	AGPAT3	1-acylglycerol-3-phosphate O-acyltransferase 3
227700_x_at	4.48E-02	0.13899374	ATAD3A	ATPase family, AAA domain containing 3A
212280_x_at	4.48E-02	0.16700871	ATG4B	autophagy related 4B cysteine peptidase
200837_at	4.48E-02	0.26514691	BCAP31	B-cell receptor-associated protein 31
217200_x_at	4.48E-02	0.089685	CYB561	cytochrome b561
230936_at	4.48E-02	0.08294742	DNAJB13	DnaJ heat shock protein family (Hsp40) member B13
1559759_at	4.48E-02	0.05935215	KIFC3	kinesin family member C3
217434_at	4.48E-02	-0.0650546	MC2R	melanocortin 2 receptor
223241_at	4.48E-02	0.27241678	MIR6836///SNX8	microRNA 6836///sorting nexin 8
223180_s_at	4.48E-02	-0.19504152	TIMM21	translocase of inner mitochondrial membrane 21
240965_at	4.48E-02	-0.06875281		
1568876_a_at	4.48E-02	-0.07658505		
242147_at	4.48E-02	0.06661001		
239070_at	4.48E-02	-0.19830831		
230102_at	4.49E-02	-0.18859969	ETV5	ETS variant 5
225258_at	4.49E-02	0.16406027	FBLIM1	filamin binding LIM protein 1
200075_s_at	4.49E-02	0.21323704	GUK1	guanylate kinase 1
219135_s_at	4.49E-02	0.13678478	LMF1	lipase maturation factor 1
1560412_at	4.49E-02	0.07281796	LOC100507506	uncharacterized LOC100507506
1555881_s_at	4.49E-02	0.14578873	LZTS2	leucine zipper tumor suppressor 2
228689_at	4.49E-02	0.07538623	NDUFA11	NADH:ubiquinone oxidoreductase subunit A11
1557126_a_at	4.49E-02	0.19248241	PLD1	phospholipase D1
242804_at	4.49E-02	0.08865577	POLN	DNA polymerase nu
220894_x_at	4.49E-02	0.09386619	PRDM12	PR/SET domain 12
212155_at	4.49E-02	0.15559043	RNF187	ring finger protein 187
223085_at	4.49E-02	-0.16940665	RNF19A	ring finger protein 19A, RBR E3 ubiquitin protein ligase
223880_x_at	4.49E-02	0.19224214	TGIF2-C20orf24///C20orf24	TGIF2-C20orf24 readthrough///chromosome 20 open reading frame 24
225765_at	4.49E-02	-0.14971956	TNPO1	transportin 1
235170_at	4.49E-02	-0.2371404	ZNF92	zinc finger protein 92
241184_x_at	4.49E-02	-0.06092503		
233263_at	4.49E-02	-0.1662979		
209717_at	4.50E-02	-0.171775	EVI5	ecotropic viral integration site 5
213826_s_at	4.50E-02	-0.16721498	H3F3A	H3 histone, family 3A
200618_at	4.50E-02	0.19791126	LASP1	LIM and SH3 protein 1
204959_at	4.50E-02	-0.32950041	MNDA	myeloid cell nuclear differentiation antigen
1568751_at	4.50E-02	-0.06515592	RGS13	regulator of G-protein signaling 13
212038_s_at	4.50E-02	0.28013549	VDAC1	voltage dependent anion channel 1
217090_at	4.51E-02	-0.05218398	ADAM3A	ADAM metallopeptidase domain 3A (pseudogene)
220890_s_at	4.51E-02	-0.14463579	APOLD1///DDX47	apolipoprotein L domain containing 1///DEAD-box helicase 47
205062_x_at	4.51E-02	-0.13910398	ARID4A	AT-rich interaction domain 4A
223243_s_at	4.51E-02	0.20919681	EDEM3	ER degradation enhancing alpha-mannosidase like protein 3
1558693_s_at	4.51E-02	0.29274566	GLMP	glycosylated lysosomal membrane protein
232813_s_at	4.51E-02	0.07406184	GOLGA6D///GOLGA6C///GOLGA6A///GOLGA6B	golgin A6 family member D///golgin A6 family member C///golgin A6 family member A///golgin A6 family member B
1568849_at	4.51E-02	0.07367617	LINC00165	long intergenic non-protein coding RNA 165
224646_x_at	4.51E-02	0.91439132	MIR675///H19	microRNA 675///H19, imprinted maternally expressed transcript (non-protein coding)
222683_at	4.51E-02	-0.1429336	RNF20	ring finger protein 20
211282_x_at	4.51E-02	0.16342259	TNFRSF25	TNF receptor superfamily member 25
237625_s_at	4.51E-02	-0.41211652		
239815_at	4.51E-02	-0.24860964		

226463_at	4.52E-02	-0.24459533	ATP6V1C1	ATPase H+ transporting V1 subunit C1
223134_at	4.52E-02	-0.21943318	BBX	BBX, HMG-box containing
213701_at	4.52E-02	-0.23953985	C12orf29	chromosome 12 open reading frame 29
226544_x_at	4.52E-02	-0.141942	EEF1E1-BLOC1S5///BLOC1S5	EEF1E1-BLOC1S5 readthrough (NMD candidate)///biogenesis of lysosomal organelles complex 1 subunit 5
216527_at	4.52E-02	-0.15648664	HCG18	HLA complex group 18 (non-protein coding)
206847_s_at	4.52E-02	0.35724226	HOXA7	homeobox A7
225133_at	4.52E-02	-0.14289803	KLF3	Kruppel like factor 3
1554185_at	4.52E-02	0.09799804	LOC554206	leucine carboxyl methyltransferase 1 pseudogene
229257_at	4.52E-02	0.14632783	TNRC18	trinucleotide repeat containing 18
212669_at	4.53E-02	0.10708488	CAMK2G	calcium/calmodulin dependent protein kinase II gamma
204726_at	4.53E-02	-0.18372411	CDH13	cadherin 13
231416_at	4.53E-02	0.12319158	DHDH	dihydrodiol dehydrogenase
219327_s_at	4.53E-02	0.20859723	GPRC5C	G protein-coupled receptor class C group 5 member C
237646_x_at	4.53E-02	-0.09190921	PLEKHG5	pleckstrin homology and RhoGEF domain containing G5
201469_s_at	4.53E-02	0.11499198	SHC1	SHC adaptor protein 1
1558673_s_at	4.53E-02	0.09072415	ZNF77	zinc finger protein 77
237079_at	4.53E-02	0.10002732		
216052_x_at	4.54E-02	0.08424609	ARTN	artemin
205457_at	4.54E-02	0.15220202	C6orf106	chromosome 6 open reading frame 106
223419_at	4.54E-02	0.14321307	FBXW9	F-box and WD repeat domain containing 9
207477_at	4.54E-02	0.05453126	PRO2958	uncharacterized LOC100128329
232790_at	4.54E-02	-0.20618196	RNASEH1-AS1	RNASEH1 antisense RNA 1
205790_at	4.54E-02	0.27559264	SKAP1	src kinase associated phosphoprotein 1
229818_at	4.54E-02	0.0729059	SVOP	SV2 related protein
1555446_s_at	4.54E-02	0.16077133	TRAPPC10	trafficking protein particle complex 10
207481_at	4.54E-02	-0.04822958		
216197_at	4.54E-02	-0.11409566		
204333_s_at	4.55E-02	-0.30627969	AGA	aspartylglucosaminidase
208764_s_at	4.55E-02	0.33014318	ATP5G2	ATP synthase, H+ transporting, mitochondrial Fo complex subunit C2 (subunit 9)
1557886_at	4.55E-02	0.0657326	CEP112	centrosomal protein 112
239871_at	4.55E-02	-0.08623806	CLTC	clathrin heavy chain
227429_at	4.55E-02	0.21705017	CRACR2B	calcium release activated channel regulator 2B
238275_at	4.55E-02	0.06910716	HAP1	huntingtin associated protein 1
213185_at	4.55E-02	0.14807665	KIAA0556	KIAA0556
224459_at	4.55E-02	-0.06036805	L2HGDH	L-2-hydroxyglutarate dehydrogenase
236598_at	4.55E-02	-0.20461706	LOC100996579	uncharacterized LOC100996579
216216_at	4.55E-02	0.05171637	SLIT3	slit guidance ligand 3
212458_at	4.55E-02	0.19142179	SPRED2	sprouty related EVH1 domain containing 2
210871_x_at	4.55E-02	-0.25329542	SSX2IP	SSX family member 2 interacting protein
222063_s_at	4.56E-02	-0.0514	CDS1	CDP-diacylglycerol synthase 1
212308_at	4.56E-02	-0.2578786	CLASP2	cytoplasmic linker associated protein 2
225401_at	4.56E-02	0.2546839	GLMP	glycosylated lysosomal membrane protein
202471_s_at	4.56E-02	0.2478639	IDH3G	isocitrate dehydrogenase 3 (NAD(+)) gamma
1569099_at	4.56E-02	0.05848573	LOC101929450	uncharacterized LOC101929450
214886_s_at	4.56E-02	-0.06082735	N4BP2L1	NEDD4 binding protein 2 like 1
235132_at	4.56E-02	-0.22588656	NIFK-AS1	NIFK antisense RNA 1
235364_at	4.56E-02	0.06786752	TSC22D3	TSC22 domain family member 3
202858_at	4.56E-02	0.17109163	U2AF1	U2 small nuclear RNA auxiliary factor 1
233756_at	4.56E-02	-0.07889622		
209811_at	4.57E-02	0.19576774	CASP2	caspase 2
1554547_at	4.57E-02	-0.33476499	FAM13C	family with sequence similarity 13 member C
234715_at	4.57E-02	-0.05376842	GOLGA2P3Y///GOLGA2P2Y	golgin A2 pseudogene 3, Y-linked///golgin A2 pseudogene 2, Y-linked
236743_at	4.57E-02	0.06511558	GPAT4	glycerol-3-phosphate acyltransferase 4
216063_at	4.57E-02	0.07244384	HBBP1	hemoglobin subunit beta pseudogene 1
225673_at	4.57E-02	0.29717376	MYADM	myeloid associated differentiation marker
216054_x_at	4.57E-02	0.07534008	MYL4	myosin light chain 4
1555426_a_at	4.57E-02	0.10421501	OTUD5	OTU deubiquitinase 5
206675_s_at	4.57E-02	0.24176921	SKIL	SKI-like proto-oncogene

206058_at	4.57E-02	0.07606137	SLC6A12	solute carrier family 6 member 12
1557986_s_at	4.57E-02	0.05462392	SMCR8	Smith-Magenis syndrome chromosome region, candidate 8
203611_at	4.57E-02	0.17387507	TERF2	telomeric repeat binding factor 2
227643_at	4.57E-02	0.05059095	TPPP	tubulin polymerization promoting protein
227854_at	4.57E-02	-0.21967758		
209837_at	4.58E-02	0.18780941	AP4M1	adaptor related protein complex 4 mu 1 subunit
229985_at	4.58E-02	0.31117365	BTNL9	butyrophilin like 9
1556318_s_at	4.58E-02	-0.07459964	CAND1	cullin associated and neddylation dissociated 1
235438_at	4.58E-02	-0.23384959	CYP7B1	cytochrome P450 family 7 subfamily B member 1
210607_at	4.58E-02	0.09318485	FLT3LG	fms related tyrosine kinase 3 ligand
223254_s_at	4.58E-02	-0.25377447	G2E3	G2/M-phase specific E3 ubiquitin protein ligase
207856_s_at	4.58E-02	0.17812162	LOC150776///SMPD4	sphingomyelin phosphodiesterase 4, neutral membrane (neutral sphingomyelinase-3) pseudogene///sphingomyelin phosphodiesterase 4
229347_at	4.58E-02	-0.38665345	MIR4458///MIR4458HG	microRNA 4458///MIR4458 host gene
235329_at	4.58E-02	0.28079014	NOXO1	NADPH oxidase organizer 1
1552482_at	4.58E-02	-0.24697447	RAPH1	Ras association (RalGDS/AF-6) and pleckstrin homology domains 1
222844_s_at	4.58E-02	-0.23309486	SRR	serine racemase
206438_x_at	4.58E-02	-0.10214145	TCTN2	tectonic family member 2
218068_s_at	4.58E-02	0.14693774	ZNF672	zinc finger protein 672
216767_at	4.58E-02	-0.06730137		
AFFX-BioC-3_at	4.58E-02	-0.33309448		
226686_at	4.59E-02	-0.20473471	CISD2	CDGSH iron sulfur domain 2
215459_at	4.59E-02	-0.07462935	CTNS	cystinosin, lysosomal cystine transporter
224822_at	4.59E-02	-0.31498197	DLC1	DLC1 Rho GTPase activating protein
215190_at	4.59E-02	-0.21087845	EIF3M	eukaryotic translation initiation factor 3 subunit M
1563475_s_at	4.59E-02	-0.10496906	ETFBKMT	electron transfer flavoprotein beta subunit lysine methyltransferase
204007_at	4.59E-02	-0.30714372	FCGR3B	Fc fragment of IgG receptor IIIb
1570013_at	4.59E-02	0.0683058	LOC101927411	uncharacterized LOC101927411
216071_x_at	4.59E-02	0.19848094	MED12	mediator complex subunit 12
206500_s_at	4.59E-02	-0.25833194	MIS18BP1	MIS18 binding protein 1
201966_at	4.59E-02	0.14944661	NDUFS2	NADH:ubiquinone oxidoreductase core subunit S2
203675_at	4.59E-02	-0.30597601	NUCB2	nucleobindin 2
218596_at	4.59E-02	0.11104575	TBC1D13	TBC1 domain family member 13
1557353_at	4.59E-02	-0.19079631		
223761_at	4.60E-02	0.17671674	FGF19	fibroblast growth factor 19
218088_s_at	4.60E-02	-0.15856921	RRAGC	Ras related GTP binding C
226022_at	4.60E-02	-0.22915168	SASH1	SAM and SH3 domain containing 1
216823_at	4.60E-02	-0.14035754	SNORD73A///RPS3A	small nucleolar RNA, C/D box 73A///ribosomal protein S3A
242232_at	4.60E-02	0.04995723		
213997_at	4.61E-02	0.13732803	FAM189A1	family with sequence similarity 189 member A1
227219_x_at	4.61E-02	0.14570904	MAP1LC3A	microtubule associated protein 1 light chain 3 alpha
212472_at	4.61E-02	0.23926911	MICAL2	microtubule associated monooxygenase, calponin and LIM domain containing 2
214087_s_at	4.61E-02	-0.44033347	MYBPC1	myosin binding protein C, slow type
214952_at	4.61E-02	-0.06231046	NCAM1	neural cell adhesion molecule 1
210841_s_at	4.61E-02	0.06948185	NRP2	neuropilin 2
244486_at	4.61E-02	0.11966061	PINK1-AS	PINK1 antisense RNA
1559502_s_at	4.62E-02	-0.15653384	LRRC25	leucine rich repeat containing 25
203353_s_at	4.62E-02	0.13004316	MBD1	methyl-CpG binding domain protein 1
203626_s_at	4.62E-02	0.07323686	SKP2	S-phase kinase-associated protein 2, E3 ubiquitin protein ligase
1566190_at	4.62E-02	-0.06590084	SUZ12	SUZ12 polycomb repressive complex 2 subunit
206702_at	4.62E-02	-0.23569833	TEK	TEK receptor tyrosine kinase
1561976_at	4.63E-02	0.06074252	C1orf167	chromosome 1 open reading frame 167
225799_at	4.63E-02	0.2154829	LOC101930489///MIR4435-2HG///LINC00152	uncharacterized LOC101930489///MIR4435-2 host gene///long intergenic non-protein coding RNA 152
1562945_at	4.63E-02	0.05967409	LOC105378130	uncharacterized LOC105378130

211989_at	4.63E-02	-0.21621887	SMARCE1	SWI/SNF related, matrix associated, actin dependent regulator of chromatin, subfamily e, member 1
210248_at	4.63E-02	0.06589591	WNT7A	Wnt family member 7A
1561361_at	4.63E-02	-0.088837	ZNF660	zinc finger protein 660
1556625_a_at	4.63E-02	-0.04989274		
202502_at	4.64E-02	-0.24264982	ACADM	acyl-CoA dehydrogenase, C-4 to C-12 straight chain
229335_at	4.64E-02	-0.07823559	CADM4	cell adhesion molecule 4
208719_s_at	4.64E-02	0.15261333	DDX17	DEAD-box helicase 17
205821_at	4.64E-02	-0.25700451	KLRC4-KLRK1///KLRK1	KLRC4-KLRK1 readthrough///killer cell lectin like receptor K1
212449_s_at	4.64E-02	-0.1695611	LYPLA1	lysophospholipase I
238679_at	4.64E-02	0.13254019	MESDC2	mesoderm development candidate 2
224598_at	4.64E-02	0.18394542	MGAT4B	mannosyl (alpha-1,3-)-glycoprotein beta-1,4-N-acetylglucosaminyltransferase, isozyme B
226397_s_at	4.64E-02	-0.15321725	PHACTR1	phosphatase and actin regulator 1
235945_at	4.64E-02	-0.05430874		
238252_at	4.64E-02	-0.16382782		
212848_s_at	4.65E-02	0.26621789	C9orf3	chromosome 9 open reading frame 3
207729_at	4.65E-02	-0.20596143	CDH9	cadherin 9
231223_at	4.65E-02	0.04711172	CSMD1	CUB and Sushi multiple domains 1
225792_at	4.65E-02	-0.17816482	HOOK1	hook microtubule tethering protein 1
1564310_a_at	4.65E-02	0.09905546	PARP15	poly(ADP-ribose) polymerase family member 15
204886_at	4.65E-02	-0.24919969	PLK4	polo like kinase 4
1560042_at	4.65E-02	-0.11549046	RMDN2	regulator of microtubule dynamics 2
226550_at	4.65E-02	-0.48469169	SLC9A7	solute carrier family 9 member A7
203721_s_at	4.65E-02	-0.18654169	UTP18	UTP18, small subunit processome component
238153_at	4.65E-02	-0.08572294		
233289_at	4.65E-02	0.07229751		
238351_x_at	4.65E-02	-0.07248647		
204719_at	4.66E-02	-0.26186975	ABCA8	ATP binding cassette subfamily A member 8
201768_s_at	4.66E-02	0.15039334	CLINT1	clathrin interactor 1
224374_s_at	4.66E-02	0.24977688	EMILIN2	elastin microfibril interfacer 2
234026_at	4.66E-02	-0.09508218	EML2-AS1	EML2 antisense RNA 1
227596_at	4.66E-02	-0.0624385	FLJ37453	uncharacterized LOC729614
239180_at	4.66E-02	0.0689067	MMP25-AS1	MMP25 antisense RNA 1
236344_at	4.66E-02	-0.13949735	PDE1C	phosphodiesterase 1C
213610_s_at	4.66E-02	-0.2036922	PHOSPHO2-KLHL23///KLHL23	PHOSPHO2-KLHL23 readthrough///kelch like family member 23
228471_at	4.67E-02	-0.29365511	ANKRD44	ankyrin repeat domain 44
202701_at	4.67E-02	0.1112393	BMP1	bone morphogenetic protein 1
1552757_s_at	4.67E-02	-0.0839998	C9orf66	chromosome 9 open reading frame 66
203654_s_at	4.67E-02	-0.19254047	COIL	coilin
200064_at	4.67E-02	0.24907245	HSP90AB1	heat shock protein 90 alpha family class B member 1
1553741_at	4.67E-02	-0.05368416	LINC00337	long intergenic non-protein coding RNA 337
224623_at	4.67E-02	0.21127291	LOC728554///THOC3	THO complex 3 pseudogene///THO complex 3
240053_x_at	4.67E-02	-0.04471495	PEX5L	peroxisomal biogenesis factor 5 like
221284_s_at	4.67E-02	0.08033819	SRC	SRC proto-oncogene, non-receptor tyrosine kinase
223771_at	4.67E-02	-0.05700911	TMEM87A	transmembrane protein 87A
229226_at	4.67E-02	0.13061501		
228362_s_at	4.68E-02	-0.08602053	FAM26F	family with sequence similarity 26 member F
213147_at	4.68E-02	0.46609356	HOXA10	homeobox A10
223828_s_at	4.68E-02	0.0612816	LGALS12	galectin 12
215133_s_at	4.68E-02	0.05544373	LOC102725213///LOC100507387///FAM153A///FAM153B	uncharacterized LOC102725213///uncharacterized LOC100507387///family with sequence similarity 153 member A///family with sequence similarity 153 member B
207091_at	4.68E-02	0.07389951	P2RX7	purinergic receptor P2X 7
210131_x_at	4.68E-02	0.16042254	SDHC	succinate dehydrogenase complex subunit C
228047_at	4.68E-02	-0.23533365	SNORA72	small nucleolar RNA, H/ACA box 72
226068_at	4.68E-02	0.2359566	SYK	spleen associated tyrosine kinase
1552519_at	4.69E-02	-0.43592459	ACVR1C	activin A receptor type 1C
1553199_at	4.69E-02	0.04497517	DCAF4L2	DDB1 and CUL4 associated factor 4 like 2
1569154_a_at	4.69E-02	0.11436302	GRAMD4	GRAM domain containing 4

210881_s_at	4.69E-02	0.17000905	INS-IGF2///IGF2	INS-IGF2 readthrough///insulin like growth factor 2
235704_at	4.69E-02	-0.20944664	LOC102723983///DAZAP2	uncharacterized LOC102723983///DAZ associated protein 2
1557483_at	4.69E-02	0.06575163	LOC284788	uncharacterized LOC284788
1568667_s_at	4.69E-02	0.07737769	PLIN5	perilipin 5
205547_s_at	4.69E-02	0.40973842	TAGLN	transgelin
222600_s_at	4.69E-02	-0.28381425	UBA6	ubiquitin like modifier activating enzyme 6
240112_at	4.69E-02	0.06142154		
208838_at	4.70E-02	-0.20095087	CAND1	cullin associated and neddylation dissociated 1
1559518_at	4.70E-02	0.06010566	HSD17B12	hydroxysteroid 17-beta dehydrogenase 12
244152_at	4.70E-02	0.06910277	IQCJ-SCHIP1-AS1	IQCJ-SCHIP1 readthrough antisense RNA 1
1555910_at	4.70E-02	-0.18813466	PTCD2	pentatricopeptide repeat domain 2
231628_s_at	4.70E-02	0.0469811	SERPINB6	serpin family B member 6
212683_at	4.70E-02	0.10448242	SLC25A44	solute carrier family 25 member 44
203272_s_at	4.70E-02	0.18603225	TUSC2	tumor suppressor candidate 2
233843_at	4.70E-02	0.09351646	ZBTB12	zinc finger and BTB domain containing 12
225634_at	4.70E-02	-0.16486436	ZC3HAV1	zinc finger CCCH-type containing, antiviral 1
240129_at	4.70E-02	0.061526		
220583_at	4.70E-02	0.0659816		
215998_at	4.70E-02	-0.06385123		
237171_at	4.70E-02	0.06614702		
226030_at	4.71E-02	-0.25285177	ACADSB	acyl-CoA dehydrogenase, short/branched chain
227878_s_at	4.71E-02	0.16257347	ALKBH7	alkB homolog 7
206991_s_at	4.71E-02	-0.17223663	CCR5	C-C motif chemokine receptor 5 (gene/pseudogene)
212528_at	4.71E-02	0.18591909	DESI1	desumoylating isopeptidase 1
1556730_at	4.71E-02	0.09074178	LOC652993	uncharacterized LOC652993
220253_s_at	4.71E-02	-0.37694342	LRP12	LDL receptor related protein 12
211019_s_at	4.71E-02	0.10567374	LSS	lanosterol synthase (2,3-oxidosqualene-lanosterol cyclase)
222836_at	4.71E-02	-0.0710203	NAA15	N(alpha)-acetyltransferase 15, NatA auxiliary subunit
230230_at	4.71E-02	-0.16660894	PTPN4	protein tyrosine phosphatase, non-receptor type 4
223724_s_at	4.71E-02	0.19187519	STAG3L2///STAG3L3///STAG3L1	stromal antigen 3-like 2 (pseudogene)///stromal antigen 3-like 3 (pseudogene)///stromal antigen 3-like 1 (pseudogene)
209108_at	4.71E-02	-0.33720508	TSPAN6	tetraspanin 6
229180_at	4.71E-02	0.0921787	WWC1	WW and C2 domain containing 1
240508_at	4.71E-02	0.08144157		
205142_x_at	4.72E-02	0.08914967	ABCD1	ATP binding cassette subfamily D member 1
216627_s_at	4.72E-02	0.08345985	B4GALT1	beta-1,4-galactosyltransferase 1
1555120_at	4.72E-02	-0.18039843	CD96	CD96 molecule
222770_s_at	4.72E-02	-0.20705351	GUF1	GUF1 homolog, GTPase
236058_at	4.72E-02	0.11526435	KDF1	keratinocyte differentiation factor 1
216520_s_at	4.72E-02	0.12174508	LOC101928826///TPT1	uncharacterized LOC101928826///tumor protein, translationally-controlled 1
205245_at	4.72E-02	0.10841789	PARD6A	par-6 family cell polarity regulator alpha
204855_at	4.72E-02	0.75581654	SERPINB5	serpin family B member 5
218994_s_at	4.72E-02	-0.15441405	STAG3L4	stromal antigen 3-like 4 (pseudogene)
243316_x_at	4.72E-02	-0.1012345	VPS26A	VPS26, retromer complex component A
239559_at	4.72E-02	-0.14171377		
1556613_s_at	4.73E-02	-0.25898088	DPY19L4	dpy-19 like 4 (C. elegans)
207231_at	4.73E-02	-0.17295999	DZIP3	DAZ interacting zinc finger protein 3
225419_at	4.73E-02	-0.16871562	MPLKIP	M-phase specific PLK1 interacting protein
1552518_s_at	4.73E-02	-0.13162641	MTBP	MDM2 binding protein
233148_at	4.73E-02	-0.06455574	PODN	podocan
243413_at	4.73E-02	-0.24021233	TTC30B	tetratricopeptide repeat domain 30B
230139_at	4.73E-02	-0.20206401		
201191_at	4.74E-02	0.19192743	PITPNA	phosphatidylinositol transfer protein alpha
241221_at	4.74E-02	-0.06841708	SEC14L3	SEC14 like lipid binding 3
238434_at	4.74E-02	-0.08009664	SMCR8	Smith-Magenis syndrome chromosome region, candidate 8
1558081_at	4.74E-02	0.06807654	SNORA65	small nucleolar RNA, H/ACA box 65
234644_x_at	4.74E-02	0.0602973	TNFRSF10C	TNF receptor superfamily member 10c
1565895_at	4.74E-02	-0.0565239		

236066_at	4.74E-02	0.12590862		
221934_s_at	4.75E-02	0.17908124	DALRD3	DALR anticodon binding domain containing 3
223811_s_at	4.75E-02	0.23609667	GET4///SUN1	golgi to ER traffic protein 4///Sad1 and UNC84 domain containing 1
202544_at	4.75E-02	-0.15957508	GMFB	glia maturation factor beta
212794_s_at	4.75E-02	-0.1511475	KIAA1033	KIAA1033
1557550_at	4.75E-02	0.0555771	LINC00906	long intergenic non-protein coding RNA 906
236997_at	4.75E-02	-0.07731607	LOC101928069	uncharacterized LOC101928069
206604_at	4.75E-02	0.13588332	OVOL1	ovo like transcriptional repressor 1
238496_at	4.75E-02	-0.30391686	WHSC1L1	Wolf-Hirschhorn syndrome candidate 1-like 1
1561526_at	4.75E-02	-0.06418195		
208427_s_at	4.76E-02	-0.12361699	ELAVL2	ELAV like neuron-specific RNA binding protein 2
223489_x_at	4.76E-02	0.14328325	EXOSC3	exosome component 3
1564463_at	4.76E-02	0.07355319	KLF13	Kruppel like factor 13
1559510_at	4.76E-02	-0.09654889	LINC00630	long intergenic non-protein coding RNA 630
1561513_at	4.76E-02	-0.09962267	LOC101927310	uncharacterized LOC101927310
216386_at	4.76E-02	0.06765767	LOC220077	dedicator of cytokinesis 1 pseudogene
214717_at	4.76E-02	0.1927483	PKI55	DKFZp434H1419
214097_at	4.76E-02	-0.32704115	RPS21	ribosomal protein S21
222084_s_at	4.76E-02	0.06385141	SBF1	SET binding factor 1
225534_at	4.76E-02	-0.23330466	SMIM19	small integral membrane protein 19
201662_s_at	4.77E-02	0.190874	ACSL3	acyl-CoA synthetase long-chain family member 3
1554327_a_at	4.77E-02	0.16058325	CANT1	calcium activated nucleotidase 1
1559590_at	4.77E-02	0.14139901	CHDH	choline dehydrogenase
212891_s_at	4.77E-02	0.25365675	GADD45GIP1	GADD45G interacting protein 1
202658_at	4.77E-02	0.18091327	PEX11B	peroxisomal biogenesis factor 11 beta
226442_at	4.78E-02	0.07700262	ABTB1	ankyrin repeat and BTB domain containing 1
234942_s_at	4.78E-02	0.296151	DNTTIP1	deoxynucleotidyltransferase terminal interacting protein 1
232599_at	4.78E-02	-0.04746021	EXOC6	exocyst complex component 6
244505_at	4.78E-02	-0.08697382	LOC102724537	uncharacterized LOC102724537
202485_s_at	4.78E-02	0.07713534	MBD2	methyl-CpG binding domain protein 2
200616_s_at	4.78E-02	0.201176	MLEC	malectin
201496_x_at	4.78E-02	0.18325317	MYH11	myosin heavy chain 11
208902_s_at	4.78E-02	-0.08658761	RPS28	ribosomal protein S28
1555819_s_at	4.78E-02	0.07544693	SAMD14	sterile alpha motif domain containing 14
202798_at	4.78E-02	-0.1648748	SEC24B	SEC24 homolog B, COPII coat complex component
218436_at	4.78E-02	0.16217463	SIL1	SIL1 nucleotide exchange factor
220992_s_at	4.78E-02	-0.1745548	TRMT1L	tRNA methyltransferase 1 like
233673_at	4.78E-02	0.07351707		
214024_s_at	4.79E-02	0.06926765	DGCR6L	DiGeorge syndrome critical region gene 6-like
231173_at	4.79E-02	-0.20448954	PYROXD1	pyridine nucleotide-disulphide oxidoreductase domain 1
1557349_at	4.79E-02	-0.10340435	RERE	arginine-glutamic acid dipeptide repeats
1558836_at	4.79E-02	0.13243761		
233302_at	4.79E-02	-0.25833051		
240105_at	4.79E-02	-0.34427037		
236856_x_at	4.79E-02	-0.23790585		
212930_at	4.80E-02	-0.23681081	ATP2B1	ATPase plasma membrane Ca2+ transporting 1
218056_at	4.80E-02	0.17151641	BFAR	bifunctional apoptosis regulator
219288_at	4.80E-02	-0.78234941	C3orf14	chromosome 3 open reading frame 14
232152_at	4.80E-02	-0.0537244	CEP57L1	centrosomal protein 57 like 1
226236_at	4.80E-02	0.27063703	LINC00493	long intergenic non-protein coding RNA 493
234288_at	4.80E-02	0.04966134	LOC101929628	uncharacterized LOC101929628
208922_s_at	4.80E-02	0.14866447	NXF1	nuclear RNA export factor 1
1555255_a_at	4.80E-02	0.15208636	PPIP5K1	diphosphoinositol pentakisphosphate kinase 1
229540_at	4.80E-02	-0.08529975	RBPJ	recombination signal binding protein for immunoglobulin kappa J region
226403_at	4.80E-02	0.28287798	TMC4	transmembrane channel like 4
244802_at	4.80E-02	-0.04600071		
225391_at	4.81E-02	-0.18635497	LOC93622	Morf4 family associated protein 1 like 1 pseudogene
1556340_at	4.81E-02	0.11846183	MAPK12	mitogen-activated protein kinase 12
204209_at	4.81E-02	0.11378604	PCYT1A	phosphate cytidylyltransferase 1, choline, alpha

223375_at	4.81E-02	0.10696769	TBC1D22B	TBC1 domain family member 22B
215590_x_at	4.82E-02	-0.07116946	ACVR2B-AS1	ACVR2B antisense RNA 1
223853_at	4.82E-02	-0.06222357	BVES	blood vessel epicardial substance
227841_at	4.82E-02	0.11446579	CEMP1///AMDHD2	cementum protein 1///amidohydrolase domain containing 2
207980_s_at	4.82E-02	0.13854759	CITED2	Cbp/p300 interacting transactivator with Glu/Asp rich carboxy-terminal domain 2
209350_s_at	4.82E-02	0.15373346	GPS2	G protein pathway suppressor 2
226098_at	4.82E-02	-0.18960798	IFT80	intraflagellar transport 80
219541_at	4.82E-02	0.12805616	LIME1	Lck interacting transmembrane adaptor 1
1553918_at	4.82E-02	0.11091216	LINC00479	long intergenic non-protein coding RNA 479
220449_at	4.82E-02	-0.14262662	LINC01260	long intergenic non-protein coding RNA 1260
211961_s_at	4.82E-02	0.12691687	RAB7A	RAB7A, member RAS oncogene family
225302_at	4.82E-02	-0.21282483	TMX3	thioredoxin related transmembrane protein 3
240237_at	4.82E-02	0.3007987		
1562294_x_at	4.83E-02	-0.1811674	ANKRD30B	ankyrin repeat domain 30B
206754_s_at	4.83E-02	0.54417093	CYP2B7P///CYP2B6	cytochrome P450 family 2 subfamily B member 7, pseudogene///cytochrome P450 family 2 subfamily B member 6
207453_s_at	4.83E-02	0.07310825	DNAJB5	DnaJ heat shock protein family (Hsp40) member B5
1563478_at	4.83E-02	0.09577487	KIAA1671	KIAA1671
226048_at	4.83E-02	-0.14961714	MAPK8	mitogen-activated protein kinase 8
216825_s_at	4.83E-02	0.07321718	MPL	MPL proto-oncogene, thrombopoietin receptor
237342_at	4.83E-02	0.07340408	TOLLIP	toll interacting protein
243563_at	4.83E-02	-0.11742441		
221146_at	4.83E-02	-0.06015991		
233578_at	4.84E-02	-0.06337171	C1orf101	chromosome 1 open reading frame 101
225747_at	4.84E-02	-0.18820014	COQ10A	coenzyme Q10A
214502_at	4.84E-02	0.07762202	HIST1H2BJ	histone cluster 1, H2bj
210036_s_at	4.84E-02	0.21386764	KCNH2	potassium voltage-gated channel subfamily H member 2
1569873_at	4.84E-02	0.04024607	LIPJ	lipase family member J
1569496_s_at	4.84E-02	0.08815429	LOC100130872	uncharacterized LOC100130872
234345_at	4.84E-02	-0.09966932	LOC101929683	uncharacterized LOC101929683
227648_at	4.84E-02	0.09679453	SMDT1	single-pass membrane protein with aspartate rich tail 1
203093_s_at	4.84E-02	0.19523537	TIMM44	translocase of inner mitochondrial membrane 44
208811_s_at	4.84E-02	0.20615849	TMEM135///DNAJB6	transmembrane protein 135///DnaJ heat shock protein family (Hsp40) member B6
220817_at	4.84E-02	-0.09132089	TRPC4	transient receptor potential cation channel subfamily C member 4
232323_s_at	4.84E-02	0.20121747	TTC17	tetratricopeptide repeat domain 17
204050_s_at	4.85E-02	0.17513878	CLTA	clathrin light chain A
1555705_a_at	4.85E-02	0.13322768	CMTM3	CKLF like MARVEL transmembrane domain containing 3
40687_at	4.85E-02	-0.08203372	GJA4	gap junction protein alpha 4
223208_at	4.85E-02	0.15660603	KCTD10	potassium channel tetramerization domain containing 10
1557465_at	4.85E-02	0.10092467	LINC00282	long intergenic non-protein coding RNA 282
225996_at	4.85E-02	0.08634089	LONRF2	LON peptidase N-terminal domain and ring finger 2
219909_at	4.85E-02	0.14957899	MMP28	matrix metallopeptidase 28
221280_s_at	4.85E-02	0.15044618	PARD3	par-3 family cell polarity regulator
214941_s_at	4.85E-02	0.12024268	PRPF40A	pre-mRNA processing factor 40 homolog A
234774_at	4.85E-02	0.07878689	R3HDML	R3H domain containing like
214113_s_at	4.85E-02	0.1582558	RBM8A	RNA binding motif protein 8A
232563_at	4.85E-02	-0.12952679	ZNF684	zinc finger protein 684
1564498_at	4.85E-02	0.09890072		
221642_at	4.85E-02	0.06413767		
223967_at	4.86E-02	0.07373379	ANGPTL6	angiopoietin like 6
222510_s_at	4.86E-02	0.15502367	MKRN2	makorin ring finger protein 2
218375_at	4.86E-02	-0.17140492	NUDT9	nudix hydrolase 9
207283_at	4.86E-02	-0.11084166	RPL23AP32	ribosomal protein L23a pseudogene 32
220371_s_at	4.86E-02	0.12008383	SLC12A9	solute carrier family 12 member 9
223917_s_at	4.86E-02	0.16190573	SLC39A3	solute carrier family 39 member 3
214441_at	4.86E-02	0.07813337	STX6	syntaxin 6

213401_s_at	4.86E-02	-0.11009094	TBL1X	transducin (beta)-like 1X-linked
213218_at	4.86E-02	-0.15424735	ZSCAN26	zinc finger and SCAN domain containing 26
237563_s_at	4.86E-02	0.25304392		
220365_at	4.87E-02	-0.07459949	ALLC	allantoicase
213041_s_at	4.87E-02	0.25557452	ATP5D	ATP synthase, H+ transporting, mitochondrial F1 complex, delta subunit
205117_at	4.87E-02	0.07519859	FGF1	fibroblast growth factor 1
206945_at	4.87E-02	0.07530886	LCT	lactase
216877_at	4.87E-02	-0.08146248	LOC100505498	uncharacterized LOC100505498
1569631_at	4.87E-02	-0.13956396	NMNAT1	nicotinamide nucleotide adenylyltransferase 1
234101_at	4.87E-02	0.05027398		
1553205_at	4.88E-02	0.06318624	C20orf166-AS1	C20orf166 antisense RNA 1
220611_at	4.88E-02	-0.07185909	DAB1	DAB1, reelin adaptor protein
238741_at	4.88E-02	-0.10208637	FAM83A	family with sequence similarity 83 member A
1569571_at	4.88E-02	-0.05688873	LIPJ	lipase family member J
229877_at	4.88E-02	-0.05937602	NEO1	neogenin 1
229624_at	4.88E-02	0.11093684	OPA3	optic atrophy 3 (autosomal recessive, with chorea and spastic paraplegia)
235245_at	4.88E-02	0.25003979	TMEM92	transmembrane protein 92
213051_at	4.88E-02	-0.16185293	ZC3HAV1	zinc finger CCCH-type containing, antiviral 1
222326_at	4.88E-02	-0.28463636		
240272_at	4.88E-02	0.05936454		
1568840_at	4.89E-02	-0.13113672	DCAF13	DDB1 and CUL4 associated factor 13
214467_at	4.89E-02	-0.30694512	GPR65	G protein-coupled receptor 65
230895_at	4.89E-02	-0.23760918	HAPLN1	hyaluronan and proteoglycan link protein 1
240807_at	4.89E-02	-0.08655405	LINC01187	long intergenic non-protein coding RNA 1187
225366_at	4.89E-02	-0.21848255	PGM2	phosphoglucomutase 2
227607_at	4.89E-02	0.19270932	STAMBPL1	STAM binding protein like 1
201229_s_at	4.90E-02	0.18473604	ARIH2	ariadne RBR E3 ubiquitin protein ligase 2
228434_at	4.90E-02	0.40340207	BTNL9	butyrophilin like 9
218664_at	4.90E-02	0.14434673	MECR	mitochondrial trans-2-enoyl-CoA reductase
235554_x_at	4.90E-02	-0.15026678	PACRGL	PARK2 coregulated like
220568_at	4.90E-02	-0.05288597	PRO1483	uncharacterized LOC55448
200730_s_at	4.90E-02	0.31922484	PTP4A1	protein tyrosine phosphatase type IVA, member 1
221037_s_at	4.90E-02	-0.04430827	SLC25A31	solute carrier family 25 member 31
241188_at	4.90E-02	-0.04059459		
202653_s_at	4.91E-02	-0.13888944	MARCHF7	membrane associated ring-CH-type finger 7
1568126_at	4.91E-02	-0.11633182	ANXA2	annexin A2
204039_at	4.91E-02	0.24262778	CEBPA	CCAAT/enhancer binding protein alpha
235469_at	4.91E-02	-0.22577121	FAM133DP///FAM133B	family with sequence similarity 133, member A pseudogene///family with sequence similarity 133 member B
237668_at	4.91E-02	-0.04508955	LOC100507201	uncharacterized LOC100507201
207496_at	4.91E-02	-0.09190699	MS4A2	membrane spanning 4-domains A2

209079_x_at	4.91E-02	0.15240322	PCDHGA1///PCDHGA2///PCDHGA3///PCDHGA4///PCDHGA5///PCDHGA6///PCDHGA7///PCDHGA9///PCDHGA10///PCDHGA11///PCDHGB1///PCDHGB2///PCDHGB3///PCDHGB5///PCDHGB6///PCDHGB7///PCDHGC4///PCDHGC5///PCDHGA12///PCDHGA8///PCDHGB4///PCDHGC3	protocadherin gamma subfamily A, 1///protocadherin gamma subfamily A, 2///protocadherin gamma subfamily A, 3///protocadherin gamma subfamily A, 4///protocadherin gamma subfamily A, 5///protocadherin gamma subfamily A, 6///protocadherin gamma subfamily A, 7///protocadherin gamma subfamily A, 9///protocadherin gamma subfamily A, 10///protocadherin gamma subfamily A, 11///protocadherin gamma subfamily B, 1///protocadherin gamma subfamily B, 2///protocadherin gamma subfamily B, 3///protocadherin gamma subfamily B, 5///protocadherin gamma subfamily B, 6///protocadherin gamma subfamily B, 7///protocadherin gamma subfamily C, 4///protocadherin gamma subfamily C, 5///protocadherin gamma subfamily A, 12///protocadherin gamma subfamily A, 8///protocadherin gamma subfamily B, 4///protocadherin gamma subfamily C, 3
226101_at	4.91E-02	0.1693629	PRKCE	protein kinase C epsilon
1569482_at	4.91E-02	-0.07724247	TNRC6C	trinucleotide repeat containing 6C
210760_x_at	4.91E-02	0.06474247	TRIP11	thyroid hormone receptor interactor 11
229812_at	4.91E-02	-0.23028228	USP48	ubiquitin specific peptidase 48
228634_s_at	4.91E-02	-0.08033704	YBX3	Y-box binding protein 3
236143_at	4.91E-02	0.11315573		
219085_s_at	4.92E-02	0.07916908	GEMIN7	gem nuclear organelle associated protein 7
227347_x_at	4.92E-02	0.21213207	HES4	hes family bHLH transcription factor 4
212671_s_at	4.92E-02	-0.5147021	LOC100509457///HLA-DQA2///HLA-DQA1	HLA class II histocompatibility antigen, DQ alpha 1 chain-like///major histocompatibility complex, class II, DQ alpha 2///major histocompatibility complex, class II, DQ alpha 1
232283_at	4.92E-02	-0.14812384	LYSMD1	LysM domain containing 1
204101_at	4.92E-02	-0.19679064	MTM1	myotubularin 1
211169_s_at	4.92E-02	0.04459274	PPP1R3A	protein phosphatase 1 regulatory subunit 3A
239528_at	4.92E-02	0.08107173	PROM2	prominin 2
238868_at	4.92E-02	-0.2097834	UACA	uveal autoantigen with coiled-coil domains and ankyrin repeats
212756_s_at	4.92E-02	-0.15219254	UBR2	ubiquitin protein ligase E3 component n-recognin 2
1553704_x_at	4.92E-02	0.15248641	ZNF791	zinc finger protein 791
216792_at	4.92E-02	-0.05501912		
204827_s_at	4.93E-02	0.13813326	CCNF	cyclin F
205661_s_at	4.93E-02	0.18465268	FLAD1	flavin adenine dinucleotide synthetase 1
208021_s_at	4.93E-02	-0.19973858	RFC1	replication factor C subunit 1
216925_s_at	4.93E-02	0.0792859	TAL1	TAL bHLH transcription factor 1, erythroid differentiation factor
227130_s_at	4.93E-02	0.07617584	TLE1	transducin like enhancer of split 1
1556744_a_at	4.93E-02	-0.20703742	ZNF654	zinc finger protein 654
216482_x_at	4.93E-02	-0.06378501	ZNF79	zinc finger protein 79
1559354_a_at	4.93E-02	-0.08302331		
223182_s_at	4.94E-02	0.19864978	AGPAT3	1-acylglycerol-3-phosphate O-acyltransferase 3
216620_s_at	4.94E-02	-0.29731532	ARHGEF10	Rho guanine nucleotide exchange factor 10
1568743_at	4.94E-02	0.08907177	ATP10A	ATPase phospholipid transporting 10A (putative)
235781_at	4.94E-02	-0.15708673	CACNA1B	calcium voltage-gated channel subunit alpha1 B
207286_at	4.94E-02	-0.04781937	CEP135	centrosomal protein 135
1570266_x_at	4.94E-02	-0.13922624	ERVH-1	endogenous retrovirus group H member 1
221333_at	4.94E-02	0.06040576	FOXP3	forkhead box P3
215632_at	4.94E-02	0.06009791	NEUROG2	neurogenin 2
208829_at	4.94E-02	0.22933794	TAPBP	TAP binding protein (tapasin)
1557632_at	4.94E-02	-0.11179525		
242816_at	4.94E-02	0.05757693		
1566157_x_at	4.94E-02	0.08091712		

215144_at	4.94E-02	0.07536022		
215488_at	4.94E-02	-0.07531051		
38340_at	4.95E-02	0.16249742	HIP1R	huntingtin interacting protein 1 related
1555222_a_at	4.95E-02	0.06363452	LOC101927122	uncharacterized LOC101927122
1553759_at	4.95E-02	-0.15669588	MCM9	minichromosome maintenance 9 homologous recombination repair factor
1555490_s_at	4.95E-02	0.07596008	PDZD3	PDZ domain containing 3
223833_at	4.95E-02	0.11979863	WDR55	WD repeat domain 55
243461_at	4.95E-02	0.18768401		
243731_at	4.95E-02	-0.08377108		
202872_at	4.96E-02	-0.22782722	ATP6V1C1	ATPase H+ transporting V1 subunit C1
216412_x_at	4.96E-02	-0.10848552	CKAP2///IGLC1	cytoskeleton associated protein 2///immunoglobulin lambda constant 1
206158_s_at	4.96E-02	0.13235925	CNBP	CCHC-type zinc finger nucleic acid binding protein
214053_at	4.96E-02	-0.08656515	ERBB4	erb-b2 receptor tyrosine kinase 4
234281_at	4.96E-02	0.06164251	ESPNP///ESPN	espin pseudogene///espin
211630_s_at	4.96E-02	0.18765114	GSS	glutathione synthetase
243815_at	4.96E-02	-0.13171902	PGBD4	piggyBac transposable element derived 4
209109_s_at	4.96E-02	-0.32581097	TSPAN6	tetraspanin 6
216811_at	4.96E-02	-0.06894396		
207321_s_at	4.97E-02	-0.0683499	ABCB9	ATP binding cassette subfamily B member 9
202046_s_at	4.97E-02	0.08446444	ARHGAP35	Rho GTPase activating protein 35
241658_at	4.97E-02	0.06798219	ARIH1	ariadne RBR E3 ubiquitin protein ligase 1
1553538_s_at	4.97E-02	0.10207949	COX1	cytochrome c oxidase subunit I
220625_s_at	4.97E-02	-0.62517859	ELF5	E74 like ETS transcription factor 5
215655_at	4.97E-02	-0.04039527	GRIK2	glutamate ionotropic receptor kainate type subunit 2
219865_at	4.97E-02	-0.14072017	LINC00339	long intergenic non-protein coding RNA 339
236768_at	4.97E-02	0.04695487	LINC01233	long intergenic non-protein coding RNA 1233
224803_s_at	4.97E-02	0.06649666	MRPL20	mitochondrial ribosomal protein L20
236910_at	4.97E-02	-0.16552491	MRPL39	mitochondrial ribosomal protein L39
1552271_at	4.97E-02	0.07102946	PRR22	proline rich 22
207188_at	4.97E-02	0.09276688	TEN1-CDK3///CDK3	TEN1-CDK3 readthrough (NMD candidate)///cyclin dependent kinase 3
202051_s_at	4.97E-02	-0.15750496	ZMYM4	zinc finger MYM-type containing 4
240277_at	4.97E-02	-0.25256278		
242544_x_at	4.97E-02	-0.04708619		
237964_at	4.97E-02	-0.09131931		
1566464_at	4.98E-02	-0.06056872	KCNK1	potassium two pore domain channel subfamily K member 1
244224_x_at	4.98E-02	0.08638973	LYNX1	Ly6/neurotoxin 1
232946_s_at	4.98E-02	0.23099502	NADSYN1	NAD synthetase 1
202400_s_at	4.98E-02	0.07618529	SRF	serum response factor
221349_at	4.98E-02	-0.04780826	VPREB1	pre-B lymphocyte 1
220107_s_at	4.98E-02	-0.17677511	ZC2HC1C	zinc finger C2HC-type containing 1C
1560087_a_at	4.98E-02	0.05217128		
235595_at	4.99E-02	0.10796565	ARHGEF2	Rho/Rac guanine nucleotide exchange factor 2
207886_s_at	4.99E-02	-0.08458741	CALCR	calcitonin receptor
224140_at	4.99E-02	-0.09639657	NPCDR1	nasopharyngeal carcinoma, down-regulated 1
241592_at	4.99E-02	-0.19609931		
1559675_at	4.99E-02	-0.09252308		

Supplementary Table 6. List of DEGs in dysadherin-knockout versus wild-type SW480 cells

#	Gene Symbol	Log2(FC)	P-value	Full descriptions of the gene	Locus on reference sequence	RefSeq mRNA	RefSeq mRNA predicte	RefSeq protein	RefSeq protein predicted	Gene type	Ensembl Gene ID	Entrez Gene ID	HGNC ID(s)
1	AASS	2.120	0.000	aminoadipate-semialdehyde synthase [Source:HGNC Symbol;Acc:HGNC:17366]	chr7:121716186-121784334	NM_005763	XM_011515726, XM_0	NP_005754	XP_011514027	protein_coding	ENSG0000000831	10157	17366
2	ABCB1	1.913	0.000	ATP binding cassette subfamily B member 1 [Source:HGNC Symbol;Acc:HGNC:40]	chr7:87132948-87461611	NM_000927	.	NP_000918	.	protein_coding	ENSG0000008556	5243	40
3	ABCG1	-2.032	0.000	ATP binding cassette subfamily G member 1 [Source:HGNC Symbol;Acc:HGNC:73]	chr21:43619798-43717354	NM_004915, NM_0	XM_011529807, XM_0	NP_004906, NP_05819	XP_011528108, XP_0115	protein_coding	ENSG0000016017	9619	73
4	ABLIM3	-1.834	0.000	actin binding LIM protein family member 3 [Source:HGNC Symbol;Acc:HGNC:29132]	chr5:148521045-148640105	NM_001301015, NM	XM_017009238, XM_0	NP_001287944, NP_00	XP_005268449, XP_0052	protein_coding	ENSG0000017321	22885	29132
5	ABTB1	-1.978	0.000	ankyrin repeat and BTB domain containing 1 [Source:HGNC Symbol;Acc:HGNC:18275]	chr3:127391782-127399763	NM_032548, NM_1	XM_017007287, XM_0	NP_115937, NP_74202	XP_006713832, XP_0067	protein_coding	ENSG0000011462	80325	18275
6	ACSL4	1.003	0.000	acyl-CoA synthetase long-chain family member 4 [Source:HGNC Symbol;Acc:HGNC:3571	chrX:108884565-108976632	NM_001318509, NM	XM_011530889, XM_0	NP_001305438, NP_00	XP_005262165, XP_0052	protein_coding	ENSG0000006836	2182	3571
7	ADAM9	1.784	0.000	ADAM metallopeptidase domain 9 [Source:HGNC Symbol;Acc:HGNC:216]	chr8:38854387-38962520	NM_003816	XM_017013942, XM_0	NP_003807	XP_011542984, XP_0168	protein_coding	ENSG0000016861	8754	216
8	ADAMTS19	2.204	0.000	ADAM metallopeptidase with thrombospondin type 1 motif 19 [Source:HGNC Symbol;A	chr5:128795957-129074376	NM_133638	XM_011543248, XM_0	NP_598377	XP_011541548, XP_0115	protein_coding	ENSG0000014580	171019	17111
9	ADAMTSL2	-2.292	0.000	ADAMTS like 2 [Source:HGNC Symbol;Acc:HGNC:14631]	chr9:136397285-136440641	NM_001145320, NM	XM_011519242, XM_0	NP_001138792, NP_05	XP_005272294, XP_0052	protein_coding	ENSG0000019785	9719	14631
10	ADGRG2	-2.903	0.000	adhesion G protein-coupled receptor G2 [Source:HGNC Symbol;Acc:HGNC:4516]	chrX:19007426-19140755	NM_001079858, NM	XM_011545436, XM_0	NP_001073327, NP_00	XP_006724518, XP_0115	protein_coding	ENSG0000017369	10149	4516
11	ADGRG6	2.298	0.000	adhesion G protein-coupled receptor G6 [Source:HGNC Symbol;Acc:HGNC:13841]	chr6:142622990-142767403	NM_001032394, NM	XM_011535964, XM_0	NP_001027566, NP_00	XP_005267118, XP_0067	protein_coding	ENSG0000011241	57211	13841
12	AGGF1	1.320	0.000	angiogenic factor with G-patch and FHA domains 1 [Source:HGNC Symbol;Acc:HGNC:24	chr5:76326209-76361059	NM_018046	.	NP_060516	.	protein_coding	ENSG0000016425	55109	24684
13	AGTPBP1	1.905	0.000	ATP/GTP binding protein 1 [Source:HGNC Symbol;Acc:HGNC:17258]	chr9:88161453-88356944	NM_001286715, NM	XM_017014546, XM_0	NP_001273644, NP_00	XP_005251904, XP_0052	protein_coding	ENSG0000013504	23287	17258
14	AHCTF1	1.671	0.000	AT-hook containing transcription factor 1 [Source:HGNC Symbol;Acc:HGNC:24618]	chr1:247002399-247094726	NM_001323342, NM	XM_011544157, XM_0	NP_001310271, NP_00	XP_006711821, XP_0115	protein_coding	ENSG0000015320	25909	24618
15	AKAP2	0.966	0.000	A-kinase anchoring protein 2 [Source:HGNC Symbol;Acc:HGNC:372]	chr9:112403067-112934792	NM_001004065, NM	.	NP_001004065, NP_00	.	protein_coding	ENSG0000024197	11217	372
16	AKAP9	1.558	0.000	A-kinase anchoring protein 9 [Source:HGNC Symbol;Acc:HGNC:379]	chr7:91570180-91739987	NM_005751, NM_1	XM_011515720, XM_0	NP_005742, NP_67171	XP_016867137, XP_0168	protein_coding	ENSG0000012791	10142	379
17	ALG1L	-1.659	0.000	ALG1, chitobiosyldiphosphodolichol beta-mannosyltransferase like [Source:HGNC Symb	chr3:125648117-125655882	NM_001015050, NM	.	NP_001015050, NP_00	XP_016861333, XP_0168	protein_coding	ENSG0000018936	200810	33721
18	ALPK1	1.749	0.000	alpha kinase 1 [Source:HGNC Symbol;Acc:HGNC:20917]	chr4:113218498-113363762	NM_001102406, NM	XM_011532283, XM_0	NP_001095876, NP_00	XP_005263302, XP_0052	protein_coding	ENSG0000007333	80216	20917
19	AMD1	1.072	0.000	adenosylmethionine decarboxylase 1 [Source:HGNC Symbol;Acc:HGNC:457]	chr6:111195972-111216916	NM_001287214, NM	.	NP_001274143, NP_00	.	protein_coding	ENSG0000012350	262	457
20	AMIGO2	2.514	0.000	adhesion molecule with Ig like domain 2 [Source:HGNC Symbol;Acc:HGNC:24073]	chr12:47469489-47630445	NM_001143668, NM	XM_005268837	NP_001137140, NP_86	XP_005268894	protein_coding	ENSG0000013921	347902	24073
21	ANGPTL4	-2.258	0.000	angiopoietin like 4 [Source:HGNC Symbol;Acc:HGNC:16039]	chr19:8429034-8439257	NM_001039667, NM	XM_005272485, XM_0	NP_001034756, NP_64	XP_005272541, XP_0052	protein_coding	ENSG0000016777	51129	16039
22	ANKIB1	1.362	0.000	ankyrin repeat and IBR domain containing 1 [Source:HGNC Symbol;Acc:HGNC:22215]	chr7:91875547-92030698	NM_019004	XM_011516335, XM_0	NP_061877	XP_005250514, XP_0052	protein_coding	ENSG0000000162	54467	22215
23	ANKMY2	1.415	0.000	ankyrin repeat and MYND domain containing 2 [Source:HGNC Symbol;Acc:HGNC:25370	chr7:16639400-16685442	NM_020319	XM_011515458	NP_064715	XP_011513760	protein_coding	ENSG0000010652	57037	25370
24	ANKRD12	1.965	0.000	ankyrin repeat domain 12 [Source:HGNC Symbol;Acc:HGNC:29135]	chr18:9136774-9285983	NM_001083625, NM	XM_017025664, XM_0	NP_001077094, NP_00	XP_005258149, XP_0052	protein_coding	ENSG0000010174	23253	29135
25	ANKRD26	2.083	0.000	ankyrin repeat domain 26 [Source:HGNC Symbol;Acc:HGNC:29186]	chr10:27293044-27389427	NM_001256053, NM	XM_011519420, XM_0	NP_001242982, NP_05	XP_006717486, XP_0067	protein_coding	ENSG0000010789	22852	29186
26	ANKRD37	-1.628	0.000	ankyrin repeat domain 37 [Source:HGNC Symbol;Acc:HGNC:29593]	chr4:186317637-186321395	NM_181726	.	NP_859077	XP_016863665	protein_coding	ENSG0000018635	353322	29593
27	ANLN	1.890	0.000	anillin actin binding protein [Source:HGNC Symbol;Acc:HGNC:14082]	chr7:36363829-36493400	NM_001284301, NM	XM_017012356, XM_0	NP_001271230, NP_00	XP_006715809, XP_0067	protein_coding	ENSG0000001142	54443	14082
28	ANO6	1.317	0.000	anoctamin 6 [Source:HGNC Symbol;Acc:HGNC:25240]	chr12:45609802-45834187	NM_001025356, NM	XM_011538024, XM_0	NP_001020527, NP_00	XP_005268763, XP_0052	protein_coding	ENSG0000017711	196527	25240
29	AP3B1	1.174	0.000	adaptor related protein complex 3 beta 1 subunit [Source:HGNC Symbol;Acc:HGNC:566	chr5:77296348-77590579	NM_001271769, NM	XM_005248618	NP_001258698, NP_00	XP_005248675, XP_0168	protein_coding	ENSG0000013284	8546	566
30	AP3M2	1.457	0.000	adaptor related protein complex 3 mu 2 subunit [Source:HGNC Symbol;Acc:HGNC:570]	chr8:42010463-42028701	NM_001134296, NM	XM_017012977	NP_001127768, NP_00	XP_016868466	protein_coding	ENSG0000007071	10947	570
31	APCDD1	-1.275	0.000	APC down-regulated 1 [Source:HGNC Symbol;Acc:HGNC:15718]	chr18:10454624-10489945	NM_153000	.	NP_694545	.	protein_coding	ENSG0000015485	147495	15718
32	APEH	-1.102	0.000	acylaminoacyl-peptide hydrolase [Source:HGNC Symbol;Acc:HGNC:586]	chr3:49711434-49726486	NM_001640	XM_011533662, XM_0	NP_001631	XP_005265154, XP_0052	protein_coding	ENSG0000016406	327	586
33	APEX2	-0.993	0.000	apurinic/apyrimidinic endodeoxyribonuclease 2 [Source:HGNC Symbol;Acc:HGNC:17889	chrX:55026789-55057497	NM_014481	.	NP_055296	.	protein_coding	ENSG0000016918	27301	17889
34	ARFGEF1	1.549	0.000	ADP ribosylation factor guanine nucleotide exchange factor 1 [Source:HGNC Symbol;Acc	chr8:68109883-68255912	NM_006421	XM_011517442, XM_0	NP_006412	XP_005251191, XP_0052	protein_coding	ENSG0000006677	10565	15772
35	ARHGAP11A	1.625	0.000	Rho GTPase activating protein 11A [Source:HGNC Symbol;Acc:HGNC:15783]	chr15:32907344-32932150	NM_001286479, NM	XM_011522262, XM_0	NP_001273408, NP_00	XP_011520562, XP_0115	protein_coding	ENSG0000019882	9824	15783
36	ARHGAP29	1.649	0.000	Rho GTPase activating protein 29 [Source:HGNC Symbol;Acc:HGNC:30207]	chr1:94637306-94703189	NM_004815	XM_011542440, XM_0	NP_001315593, NP_00	XP_011540740, XP_0115	protein_coding	ENSG0000013796	9411	30207
37	ARHGAP5	1.541	0.000	Rho GTPase activating protein 5 [Source:HGNC Symbol;Acc:HGNC:675]	chr14:32546172-32628934	NM_001030055, NM	XM_005267636, XM_0	NP_001025226, NP_00	XP_005267692, XP_0052	protein_coding	ENSG0000010085	394	675
38	ARHGEF12	1.090	0.000	Rho guanine nucleotide exchange factor 12 [Source:HGNC Symbol;Acc:HGNC:14193]	chr11:120207622-120360645	NM_001198665, NM	XM_017017421, XM_0	NP_001185594, NP_00	XP_006718868, XP_0115	protein_coding	ENSG0000019691	23365	14193
39	ASB2	-3.380	0.000	ankyrin repeat and SOCS box containing 2 [Source:HGNC Symbol;Acc:HGNC:16012]	chr14:94400498-94443137	NM_001202429, NM	XM_011536835, XM_0	NP_001189358, NP_05	XP_005267815, XP_0115	protein_coding	ENSG0000010062	51676	16012
40	ASB4	1.658	0.000	ankyrin repeat and SOCS box containing 4 [Source:HGNC Symbol;Acc:HGNC:16009]	chr7:95115212-95169544	NM_016116, NM_1	.	NP_057200, NP_66587	.	protein_coding	ENSG0000000598	51666	16009
41	ASMTL	-2.330	0.000	acetylserotonin O-methyltransferase-like [Source:HGNC Symbol;Acc:HGNC:751], mappe	chrX:1522031-1572655	NM_001173473, NM	XM_005274783, XM_0	NP_001166944, NP_00	XP_005274491, XP_0052	protein_coding	ENSG0000016909	8623	751
42	ASPM	1.651	0.000	abnormal spindle microtubule assembly [Source:HGNC Symbol;Acc:HGNC:19048]	chr1:197053257-197115824	NM_001206846, NM	.	NP_001193775, NP_06	.	protein_coding	ENSG0000006627	259266	19048
43	ASPSCR1	-1.236	0.000	ASPSCR1, UBX domain containing tether for SLC2A4 [Source:HGNC Symbol;Acc:HGNC:1	chr17:79935425-79975282	NM_001251888, NM	XM_017025043, XM_0	NP_001238817, NP_07	XP_011521903, XP_0115	protein_coding	ENSG0000016969	79058	13825
44	ASS1	-1.230	0.000	argininosuccinate synthase 1 [Source:HGNC Symbol;Acc:HGNC:758]	chr9:133320315-133376661	NM_000050, NM_0	XM_017014729, XM_0	NP_000041, NP_44646	XP_005272257, XP_0115	protein_coding	ENSG0000013070	445	758
45	ATAD2	1.711	0.000	ATPase family, AAA domain containing 2 [Source:HGNC Symbol;Acc:HGNC:30123]	chr8:124332089-124428590	NM_014109	XM_011516994	NP_054828	XP_011515296	protein_coding	ENSG0000015680	29028	30123
46	ATL2	1.280	0.000	atlastin GTPase 2 [Source:HGNC Symbol;Acc:HGNC:24047]	chr2:38522021-38604427	NM_001135673, NM	XM_011533022, XM_0	NP_001129145, NP_00	XP_006712134, XP_0115	protein_coding	ENSG0000011978	64225	24047
47	ATP11C	1.581	0.000	ATPase phospholipid transporting 11C [Source:HGNC Symbol;Acc:HGNC:13554]	chrX:138808504-139015229	NM_001010986, NM	XM_017029449, XM_0	NP_001010986, NP_77	XP_016884928, XP_0168	protein_coding	ENSG0000010197	286410	13554
48	ATP13A3	1.322	0.000	ATPase 13A3 [Source:HGNC Symbol;Acc:HGNC:24113]	chr3:194123400-194219093	NM_024524	XM_011513124, XM_0	NP_078800	XP_005269414, XP_0115	protein_coding	ENSG0000013365	79572	24113
49	ATP6V1B2	0.999	0.000	ATPase H+ transporting V1 subunit B2 [Source:HGNC Symbol;Acc:HGNC:854]	chr8:20054877-20079212	NM_001693	.	NP_001684	.	protein_coding	ENSG0000014741	526	854
50	ATR	1.573	0.000	ATR serine/threonine kinase [Source:HGNC Symbol;Acc:HGNC:882]	chr3:142168078-142297668	NM_001184	XM_011512925, XM_0	NP_001175	XP_011511226, XP_0115	protein_coding	ENSG0000017505	545	882
51	AVL9	1.211	0.000	AVL9 cell migration associated [Source:HGNC Symbol;Acc:HGNC:28994]	chr7:32535100-32628333	NM_015060	XM_011515228, XM_0	NP_055875	XP_005249725, XP_0052	protein_coding	ENSG0000010577	23080	28994
52	AZIN1	1.117	0.000	antizyme inhibitor 1 [Source:HGNC Symbol;Acc:HGNC:16432]	chr8:103838584-103876965	NM_001301668, NM	XM_011517128, XM_0	NP_001288597, NP_05	XP_005251026, XP_0115	protein_coding	ENSG0000015509	51582	16432

53	BAZ1A	1.650	0.000	bromodomain adjacent to zinc finger domain 1A [Source:HGNC Symbol;Acc:HGNC:960]	chr14:35221936-35344853	NM_013448, NM_1	XM_011536375, XM_0	NP_038476, NP_87258	XP_011534676	protein_coding	ENSG0000019860	11177	960
54	BAZ2B	1.567	0.000	bromodomain adjacent to zinc finger domain 2B [Source:HGNC Symbol;Acc:HGNC:963]	chr2:160175489-160473203	NM_001289975, NN	XM_011511055, XM_0	NP_001276904, NP_03	XP_005246545, XP_0052	protein_coding	ENSG0000012363	29994	963
55	BBC3	-1.867	0.000	BCL2 binding component 3 [Source:HGNC Symbol;Acc:HGNC:17868]	chr19:47724080-47736023	NM_001127240, NN	XM_006723141	NP_001120712, NP_00	XP_006723204, XP_0115	protein_coding	ENSG0000010532	100422832	17868
56	BCLAF1	1.417	0.000	BCL2 associated transcription factor 1 [Source:HGNC Symbol;Acc:HGNC:16863]	chr6:136578000-136610989	NM_001077440, NN	XM_005267237	NP_001070908, NP_00	XP_005267294, XP_0168	protein_coding	ENSG0000002936	9774	16863
57	BHLHE41	2.397	0.000	basic helix-loop-helix family member e41 [Source:HGNC Symbol;Acc:HGNC:16617]	chr12:26272958-26278060	NM_030762	.	NP_110389	.	protein_coding	ENSG0000012309	79365	16617
58	BIRC6	1.237	0.000	baculoviral IAP repeat containing 6 [Source:HGNC Symbol;Acc:HGNC:13516]	chr2:32582095-32843966	NM_016252	XM_011533004, XM_0	NP_057336	XP_005264506, XP_0052	protein_coding	ENSG0000011557	57448	13516
59	BMF	-1.634	0.000	Bcl2 modifying factor [Source:HGNC Symbol;Acc:HGNC:24132]	chr15:40380090-40401093	NM_001003940, NN	XM_006720734, XM_0	NP_001003940, NP_00	XP_005254817, XP_0052	protein_coding	ENSG0000010408	90427	24132
60	BRCA1	1.619	0.000	BRCA1, DNA repair associated [Source:HGNC Symbol;Acc:HGNC:1100]	chr17:41196311-41277500	NM_007294, NM_0	.	NP_009225, NP_00922	.	protein_coding	ENSG0000001204	672	1100
61	BRIP1	1.563	0.000	BRCA1 interacting protein C-terminal helicase 1 [Source:HGNC Symbol;Acc:HGNC:20473	chr17:59758626-59940882	NM_032043	XM_011525340, XM_0	NP_114432	XP_011523634, XP_0115	protein_coding	ENSG0000013649	83990	20473
62	BRSK2	-2.034	0.000	BR serine/threonine kinase 2 [Source:HGNC Symbol;Acc:HGNC:11405]	chr11:1411128-1483915	NM_001256627, NN	XM_011520465, XM_0	NP_001243556, NP_00	XP_005253275, XP_0067	protein_coding	ENSG0000017467	9024	11405
63	BST2	-2.865	0.000	bone marrow stromal cell antigen 2 [Source:HGNC Symbol;Acc:HGNC:1119]	chr19:17513747-17516457	NM_004335	.	NP_004326	.	protein_coding	ENSG0000013030	684	1119
64	BTAF1	1.529	0.000	B-TFIID TATA-box binding protein associated factor 1 [Source:HGNC Symbol;Acc:HGNC:1	chr10:93683525-93790082	NM_003972	XM_017016878, XM_0	NP_003963	XP_011538628, XP_0115	protein_coding	ENSG0000009556	9044	17307
65	BUB1	1.148	0.000	BUB1 mitotic checkpoint serine/threonine kinase [Source:HGNC Symbol;Acc:HGNC:1148	chr2:111395274-111435691	NM_001278616, NN	.	NP_001265545, NP_00	.	protein_coding	ENSG0000016967	699	1148
66	C1QTNF1	-1.585	0.000	C1q and tumor necrosis factor related protein 1 [Source:HGNC Symbol;Acc:HGNC:14324	chr17:77018895-77045870	NM_030968, NM_1	XM_006721667	NP_112230, NP_69920	XP_006721729, XP_0067	protein_coding	ENSG0000017391	114897	14324
67	C3	-1.560	0.000	complement component 3 [Source:HGNC Symbol;Acc:HGNC:1318]	chr19:6677714-6720693	NM_000064	.	NP_000055	.	protein_coding	ENSG0000012573	718	1318
68	C5orf34	1.771	0.000	chromosome 5 open reading frame 34 [Source:HGNC Symbol;Acc:HGNC:24738]	chr5:43486802-43515247	NM_198566	.	NP_940968	.	protein_coding	ENSG0000017224	375444	24738
69	CA9	-2.700	0.000	carbonic anhydrase 9 [Source:HGNC Symbol;Acc:HGNC:1383]	chr9:35673852-35681156	NM_001216	.	NP_001207	.	protein_coding	ENSG0000010715	768	1383
70	CACFD1	-1.467	0.000	calcium channel flower domain containing 1 [Source:HGNC Symbol;Acc:HGNC:1365]	chr9:136325086-136335970	NM_001135775, NN	XM_011518186, XM_0	NP_001129247, NP_00	XP_011516484, XP_0115	protein_coding	ENSG0000016032	11094	1365
71	CACNB4	1.827	0.000	calcium voltage-gated channel auxiliary subunit beta 4 [Source:HGNC Symbol;Acc:HGNC	chr2:152689281-152955989	NM_000726, NM_0	XM_011511796, XM_0	NP_000717, NP_00100	XP_006712794, XP_0115	protein_coding	ENSG0000018238	785	1404
72	CADPS2	2.412	0.000	calcium dependent secretion activator 2 [Source:HGNC Symbol;Acc:HGNC:16018]	chr7:121958478-122526813	NM_001009571, NN	XM_017012795, XM_0	NP_001009571, NP_00	XP_005250752, XP_0052	protein_coding	ENSG0000008180	93664	16018
73	CALCOCO1	-1.008	0.000	calcium binding and coiled-coil domain 1 [Source:HGNC Symbol;Acc:HGNC:29306]	chr12:54102300-54121529	NM_001143682, NN	XM_011538604, XM_0	NP_001137154, NP_06	XP_011536903, XP_0115	protein_coding	ENSG0000001282	57658	29306
74	CALD1	1.395	0.000	caldesmon 1 [Source:HGNC Symbol;Acc:HGNC:1441]	chr7:134429002-134655479	NM_004342, NM_0	XM_017012654, XM_0	NP_004333, NP_14912	XP_016868139, XP_0168	protein_coding	ENSG0000012278	800	1441
75	CALHM1	-3.187	0.000	calcium homeostasis modulator 1 [Source:HGNC Symbol;Acc:HGNC:23494]	chr10:105213143-105218645	NM_001001412	.	NP_001001412	.	protein_coding	ENSG0000018593	255022	23494
76	CALHM3	-1.450	0.000	calcium homeostasis modulator 3 [Source:HGNC Symbol;Acc:HGNC:23458]	chr10:105232560-105238997	NM_001129742	.	NP_001123214	.	protein_coding	ENSG0000018312	119395	23458
77	CALU	1.328	0.000	calumenin [Source:HGNC Symbol;Acc:HGNC:1458]	chr7:128379345-128411531	NM_001130674, NN	XM_011516588	NP_001124146, NP_00	.	protein_coding	ENSG0000012859	813	1458
78	CAMK2D	1.750	0.000	calcium/calmodulin dependent protein kinase II delta [Source:HGNC Symbol;Acc:HGNC:	chr4:114372187-114683083	NM_001221, NM_0	XM_017008678, XM_0	NP_001212, NP_00130	XP_005263309, XP_0052	protein_coding	ENSG0000014534	817	1462
79	CAMSAP2	1.307	0.000	calmodulin regulated spectrin associated protein family member 2 [Source:HGNC Symb	chr1:200708685-200829832	NM_001297707, NN	XM_005245041	NP_001284636, NP_00	XP_005245098, XP_0168	protein_coding	ENSG0000011820	23271	29188
80	CAND1	1.264	0.000	cullin associated and neddylation dissociated 1 [Source:HGNC Symbol;Acc:HGNC:30688	chr12:67663060-67713731	NM_018448	.	NP_001316603, NP_00	.	protein_coding	ENSG0000011153	55832	30688
81	CAPG	-1.101	0.000	capping actin protein, gelsolin like [Source:HGNC Symbol;Acc:HGNC:1474]	chr2:85621870-85641197	NM_001256139, NN	XM_011533123, XM_0	NP_001243068, NP_00	XP_005264638, XP_0115	protein_coding	ENSG0000004249	822	1474
82	CAPN6	2.801	0.000	calpain 6 [Source:HGNC Symbol;Acc:HGNC:1483]	chrX:110488330-110513751	NM_014289	.	NP_055104	.	protein_coding	ENSG0000007727	827	1483
83	CAPN8	-2.691	0.000	calpain 8 [Source:HGNC Symbol;Acc:HGNC:1485]	chr1:223714971-223853436	NM_001143962	.	NP_001137434	XP_016856757	protein_coding	ENSG0000020369	388743	1485
84	CAPZA1	1.558	0.000	capping actin protein of muscle Z-line alpha subunit 1 [Source:HGNC Symbol;Acc:HGNC:	chr1:113009162-113214241	NM_006135	.	NP_006126	.	protein_coding	ENSG0000011648	829	1488
85	CASC5	1.734	0.000	kinetochore scaffold 1 [Source:HGNC Symbol;Acc:HGNC:24054]	chr15:40886217-40956540	NM_144508, NM_1	XM_017022432, XM_0	NP_653091, NP_73346	XP_016877921	protein_coding	ENSG0000013781	57082	24054
86	CASP4	1.423	0.000	caspase 4 [Source:HGNC Symbol;Acc:HGNC:1505]	chr11:104813592-104840163	NM_001225, NM_0	XM_011543019	NP_001216, NP_15064	XP_011541321	protein_coding	ENSG0000019695	837	1505
87	CASP8AP2	1.678	0.000	caspase 8 associated protein 2 [Source:HGNC Symbol;Acc:HGNC:1510]	chr6:90539618-90581131	NM_001137667, NN	.	NP_001131139, NP_00	.	protein_coding	ENSG0000011841	9994	1510
88	CCDC22	-1.664	0.000	coiled-coil domain containing 22 [Source:HGNC Symbol;Acc:HGNC:28909]	chrX:49091926-49121288	NM_014008	XM_005272599	NP_054727	XP_005272656	protein_coding	ENSG0000010199	28952	28909
89	CCDC25	1.494	0.000	coiled-coil domain containing 25 [Source:HGNC Symbol;Acc:HGNC:25591]	chr8:27590827-27630151	NM_001304529, NN	XM_011544571, XM_0	NP_001291458, NP_00	XP_011542872, XP_0115	protein_coding	ENSG0000014741	55246	25591
90	CCDC28B	-1.551	0.000	coiled-coil domain containing 28B [Source:HGNC Symbol;Acc:HGNC:28163]	chr1:32665986-32670988	NM_001301011, NN	XM_011542113	NP_001287940, NP_07	XP_011540415, XP_0168	protein_coding	ENSG0000016005	79140	28163
91	CCDC69	-1.097	0.000	coiled-coil domain containing 69 [Source:HGNC Symbol;Acc:HGNC:24487]	chr5:150560612-150603706	NM_015621	.	NP_056436	.	protein_coding	ENSG0000019862	26112	24487
92	CCDC88A	1.884	0.000	coiled-coil domain containing 88A [Source:HGNC Symbol;Acc:HGNC:25523]	chr2:55514977-55647057	NM_001135597, NN	XM_011532969, XM_0	NP_001129069, NP_00	XP_005264475, XP_0052	protein_coding	ENSG0000011535	55704	25523
93	CCL2	-1.872	0.000	C-C motif chemokine ligand 2 [Source:HGNC Symbol;Acc:HGNC:10618]	chr17:32581899-32584222	NM_002982	.	NP_002973	.	protein_coding	ENSG0000010869	6347	10618
94	CCL20	-2.454	0.000	C-C motif chemokine ligand 20 [Source:HGNC Symbol;Acc:HGNC:10619]	chr2:228678557-228682280	NM_001130046, NN	.	NP_001123518, NP_00	.	protein_coding	ENSG0000011500	6364	10619
95	CCP110	1.575	0.000	centriolar coiled-coil protein 110 [Source:HGNC Symbol;Acc:HGNC:24342]	chr16:19535132-19564730	NM_001199022, NN	XM_011545993, XM_0	NP_001185951, NP_00	XP_005255776, XP_0115	protein_coding	ENSG0000010354	9738	24342
96	CCR7	-2.893	0.000	C-C motif chemokine receptor 7 [Source:HGNC Symbol;Acc:HGNC:1608]	chr17:38710020-38721724	NM_001301714, NN	.	NP_001288643, NP_00	.	protein_coding	ENSG0000012635	1236	1608
97	CCT6A	0.894	0.000	chaperonin containing TCP1 subunit 6A [Source:HGNC Symbol;Acc:HGNC:1620]	chr7:56119322-56131682	NM_001009186, NN	.	NP_001009186, NP_00	.	protein_coding	ENSG0000014673	908	1620
98	CD276	-1.145	0.000	CD276 molecule [Source:HGNC Symbol;Acc:HGNC:19137]	chr15:73976553-74006859	NM_001024736, NN	XM_017022638, XM_0	NP_001019907, NP_00	XP_005254757, XP_0115	protein_coding	ENSG0000010385	80381	19137
99	CD300A	-Inf	0.000	CD300a molecule [Source:HGNC Symbol;Acc:HGNC:19319]	chr17:72462554-72480935	NM_001256841, NN	XM_011524254, XM_0	NP_001243770, NP_00	XP_005257047, XP_0052	protein_coding	ENSG0000016785	11314	19319
100	CD46	1.244	0.000	CD46 molecule [Source:HGNC Symbol;Acc:HGNC:6953]	chr1:207925401-207968858	NM_002389, NM_1	XM_017001309, XM_0	NP_002380, NP_72254	XP_011507865, XP_0115	protein_coding	ENSG0000011733	4179	6953
101	CD99	-1.886	0.000	CD99 molecule [Source:HGNC Symbol;Acc:HGNC:7082], mapped to PAR; chrY:2559219-	chrX:2609219-2659350	NM_001122898, NN	XM_005274535, XM_0	NP_001116370, NP_00	XP_005274590, XP_0052	protein_coding	ENSG0000000258	4267	7082
102	CDC27	1.353	0.000	cell division cycle 27 [Source:HGNC Symbol;Acc:HGNC:1728]	chr17:45195068-45266632	NM_001114091, NN	XM_017025488, XM_0	NP_001107563, NP_00	XP_011523848, XP_0115	protein_coding	ENSG0000000489	996	1728
103	CDC6	1.066	0.000	cell division cycle 6 [Source:HGNC Symbol;Acc:HGNC:1744]	chr17:38443884-38460909	NM_001254	XM_011525542, XM_0	NP_001245	XP_011523843, XP_0115	protein_coding	ENSG0000009480	990	1744
104	CDC7	1.630	0.000	cell division cycle 7 [Source:HGNC Symbol;Acc:HGNC:1745]	chr1:91966449-91991321	NM_001134419, NN	XM_017002427, XM_0	NP_001127891, NP_00	XP_005271298, XP_0052	protein_coding	ENSG0000009704	8317	1745
105	CDCA2	1.591	0.000	cell division cycle associated 2 [Source:HGNC Symbol;Acc:HGNC:14623]	chr8:25316512-25365436	NM_001317906, NN	XM_011544420, XM_0	NP_001304835, NP_00	XP_006716357, XP_0115	protein_coding	ENSG0000018466	157313	14623
106	CDK12	1.087	0.000	cyclin dependent kinase 12 [Source:HGNC Symbol;Acc:HGNC:24224]	chr17:37618013-37691399	NM_015083, NM_0	XM_011524907, XM_0	NP_055898, NP_05759	XP_005257513, XP_0052	protein_coding	ENSG0000016725	51755	24224

107	CDK15	2.407	0.000	cyclin dependent kinase 15 [Source:HGNC Symbol;Acc:HGNC:14434]	chr2:202655183-202760273	NM_001261435, N	XM_011511652, XM_0	NP_001248364, NP_00	XP_005246838, XP_0052	protein_coding	ENSG0000013839	65061	14434
108	CDK16	-1.264	0.000	cyclin dependent kinase 16 [Source:HGNC Symbol;Acc:HGNC:8749]	chrX:47077631-47089396	NM_001170460, N	XM_011543928, XM_0	NP_001163931, NP_00	XP_011542222, XP_0115	protein_coding	ENSG0000010222	5127	8749
109	CEACAM21	-1.646	0.000	carcinoembryonic antigen related cell adhesion molecule 21 [Source:HGNC Symbol;Acc:	chr19:42055885-42093197	NM_001098506, N	XM_017027435, XM_0	NP_001091976, NP_00	XP_005278453, XP_0052	protein_coding	ENSG0000000712	90273	28834
110	CENPE	1.575	0.000	centromere protein E [Source:HGNC Symbol;Acc:HGNC:1856]	chr4:104026962-104119566	NM_001286734, N	XM_011531549, XM_0	NP_001273663, NP_00	XP_011529846, XP_0115	protein_coding	ENSG0000013877	1062	1856
111	CENPF	1.329	0.000	centromere protein F [Source:HGNC Symbol;Acc:HGNC:1857]	chr1:214776537-214837931	NM_016343	XM_017000086, XM_0	NP_057427	XP_011507384, XP_0168	protein_coding	ENSG0000011772	1063	1857
112	CEP152	1.850	0.000	centrosomal protein 152 [Source:HGNC Symbol;Acc:HGNC:29298]	chr15:49030131-49103338	NM_001194998, N	XM_011521379, XM_0	NP_001181927, NP_05	XP_006720500, XP_0115	protein_coding	ENSG0000010399	22995	29298
113	CEP350	1.636	0.000	centrosomal protein 350 [Source:HGNC Symbol;Acc:HGNC:24238]	chr1:179923872-180084015	NM_014810	XM_011510198, XM_0	NP_055625	XP_011508493, XP_0115	protein_coding	ENSG0000013583	9857	24238
114	CEP55	1.282	0.000	centrosomal protein 55 [Source:HGNC Symbol;Acc:HGNC:1161]	chr10:95256388-95288849	NM_001127182, N	XM_011539920, XM_0	NP_001120654, NP_06	XP_011538220, XP_0115	protein_coding	ENSG0000013818	55165	1161
115	CEP57	1.337	0.000	centrosomal protein 57 [Source:HGNC Symbol;Acc:HGNC:30794]	chr11:95523128-95565857	NM_001243776, N	XM_006718946, XM_0	NP_001230705, NP_00	XP_006719008, XP_0067	protein_coding	ENSG0000016603	9702	30794
116	CEP78	1.336	0.000	centrosomal protein 78 [Source:HGNC Symbol;Acc:HGNC:25740]	chr9:80850977-80894606	NM_001098802, N	XM_017015194, XM_0	NP_001092272, NP_11	XP_005252320, XP_0052	protein_coding	ENSG0000014801	84131	25740
117	CFAP100	-Inf	0.000	cilia and flagella associated protein 100 [Source:HGNC Symbol;Acc:HGNC:26842]	chr3:126113781-126155398	NM_182628	XM_006713623, XM_0	NP_872434	XP_005247488, XP_0067	protein_coding	ENSG0000016388	348807	26842
118	CFAP97	1.504	0.000	cilia and flagella associated protein 97 [Source:HGNC Symbol;Acc:HGNC:29276]	chr4:186080818-186125182	NM_001292033, N	XM_017008486, XM_0	NP_001278962, NP_06	XP_005263211, XP_0115	protein_coding	ENSG0000016432	57587	29276
119	CFD	-1.635	0.000	complement factor D [Source:HGNC Symbol;Acc:HGNC:2771]	chr19:859642-863630	NM_001317335, N	.	NP_001304264, NP_00	.	protein_coding	ENSG0000019776	1675	2771
120	CHCHD4	-1.150	0.000	coiled-coil-helix-coiled-coil-helix domain containing 4 [Source:HGNC Symbol;Acc:HGNC:	chr3:14153576-14166370	NM_001098502, N	.	NP_001091972, NP_65	.	protein_coding	ENSG0000016352	131474	26467
121	CHD1	1.602	0.000	chromodomain helicase DNA binding protein 1 [Source:HGNC Symbol;Acc:HGNC:1915]	chr5:98189688-98264661	NM_001270	XM_011543112, XM_0	NP_001261	XP_005271923, XP_0052	protein_coding	ENSG0000015392	1105	1915
122	CHST1	-1.425	0.000	carbohydrate sulfotransferase 1 [Source:HGNC Symbol;Acc:HGNC:1969]	chr11:45670426-45687172	NM_003654	XM_006718356	NP_003645	XP_006718419, XP_0168	protein_coding	ENSG0000017526	8534	1969
123	CHST13	-1.360	0.000	carbohydrate sulfotransferase 13 [Source:HGNC Symbol;Acc:HGNC:21755]	chr3:126243125-126262134	NM_152889	.	NP_690849	.	protein_coding	ENSG0000018076	166012	21755
124	CITED4	-1.478	0.000	Cbp/p300 interacting transactivator with Glu/Asp rich carboxy-terminal domain 4 [Sour	chr1:41326722-41328038	NM_133467	.	NP_597724	.	protein_coding	ENSG0000017986	163732	18696
125	CKAP5	1.043	0.000	cytoskeleton associated protein 5 [Source:HGNC Symbol;Acc:HGNC:28959]	chr11:46764597-46867859	NM_001008938, N	.	NP_001008938, NP_05	.	protein_coding	ENSG0000017521	9793	28959
126	CKB	-1.221	0.000	creatine kinase B [Source:HGNC Symbol;Acc:HGNC:1991]	chr14:103985995-103989448	NM_001823	.	NP_001814	XP_016876440, XP_0168	protein_coding	ENSG0000016616	1152	1991
127	CLCN3	1.362	0.000	chloride voltage-gated channel 3 [Source:HGNC Symbol;Acc:HGNC:2021]	chr4:170541671-170644824	NM_001243372, N	XM_011531586, XM_0	NP_001230301, NP_00	XP_005262783, XP_0115	protein_coding	ENSG0000010957	1182	2021
128	CLDN12	1.310	0.000	claudin 12 [Source:HGNC Symbol;Acc:HGNC:2034]	chr7:90032699-90045268	NM_001185072, N	.	NP_001172001, NP_00	.	protein_coding	ENSG0000015722	9069	2034
129	CLEC11A	-1.662	0.000	C-type lectin domain family 11 member A [Source:HGNC Symbol;Acc:HGNC:10576]	chr19:51226585-51228975	NM_002975	.	NP_002966	.	protein_coding	ENSG0000010547	6320	10576
130	CLGN	2.026	0.000	calmegin [Source:HGNC Symbol;Acc:HGNC:2060]	chr4:141309608-141349122	NM_001130675, N	.	NP_001124147, NP_00	.	protein_coding	ENSG0000015313	1047	2060
131	CLOCK	1.925	0.000	clock circadian regulator [Source:HGNC Symbol;Acc:HGNC:2082]	chr4:56294069-56413305	NM_001267843, N	XM_011534411, XM_0	NP_001254772, NP_00	XP_005265844, XP_0067	protein_coding	ENSG0000013485	9575	2082
132	CLSPN	1.674	0.000	claspin [Source:HGNC Symbol;Acc:HGNC:19715]	chr1:36185818-36235568	NM_001190481, N	XM_011541942, XM_0	NP_001177410, NP_07	XP_011540240, XP_0115	protein_coding	ENSG0000009285	63967	19715
133	CLSTN2	-2.190	0.000	calsyntenin 2 [Source:HGNC Symbol;Acc:HGNC:17448]	chr3:139654026-140296239	NM_022131	XM_017007022	NP_071414	XP_016862511	protein_coding	ENSG0000015825	64084	17448
134	CLTC	1.670	0.000	clathrin heavy chain [Source:HGNC Symbol;Acc:HGNC:2092]	chr17:57697049-57774317	NM_001288653, N	XM_011524281, XM_0	NP_001275582, NP_00	XP_005257069	protein_coding	ENSG0000014136	1213	2092
135	CMTR2	1.673	0.000	cap methyltransferase 2 [Source:HGNC Symbol;Acc:HGNC:25635]	chr16:71315291-71323512	NM_001099642, N	XM_011523229, XM_0	NP_001093112, NP_00	XP_005256103, XP_0052	protein_coding	ENSG0000018091	55783	25635
136	COL2A1	-2.393	0.000	collagen type II alpha 1 chain [Source:HGNC Symbol;Acc:HGNC:2200]	chr12:48366747-48398269	NM_001844, NM_0	XM_017018830, XM_0	NP_001835, NP_14916	XP_016874317, XP_0168	protein_coding	ENSG0000013921	1280	2200
137	COL6A2	-1.993	0.000	collagen type VI alpha 2 chain [Source:HGNC Symbol;Acc:HGNC:2212]	chr21:47518010-47552763	NM_001849, NM_0	XM_011529452, XM_0	NP_001840, NP_47805	XP_011527753, XP_0115	protein_coding	ENSG0000014217	1292	2212
138	COPB1	1.368	0.000	coatomer protein complex subunit beta 1 [Source:HGNC Symbol;Acc:HGNC:2231]	chr11:14479051-14521441	NM_001144061, N	.	NP_001137533, NP_00	.	protein_coding	ENSG0000012908	1315	2231
139	CORO1A	-1.227	0.000	coronin 1A [Source:HGNC Symbol;Acc:HGNC:2252]	chr16:30194742-30200397	NM_001193333, N	XM_011545714	NP_001180262, NP_00	XP_011544016, XP_0168	protein_coding	ENSG0000010287	11151	2252
140	COX7B	-1.161	0.000	cytochrome c oxidase subunit 7B [Source:HGNC Symbol;Acc:HGNC:2291]	chrX:77154934-77162870	NM_001866	.	NP_001857	.	protein_coding	ENSG0000013117	1349	2291
141	CPSF2	1.185	0.000	cleavage and polyadenylation specific factor 2 [Source:HGNC Symbol;Acc:HGNC:2325]	chr14:92588280-92638489	NM_017437	XM_005267767	NP_001309201, NP_05	XP_005267824	protein_coding	ENSG0000016593	53981	2325
142	CRELD1	-1.676	0.000	cysteine rich with EGF like domains 1 [Source:HGNC Symbol;Acc:HGNC:14630]	chr3:9975505-9987097	NM_001031717, N	XM_011534108	NP_001026887, NP_00	XP_011532410, XP_0168	protein_coding	ENSG0000016370	78987	14630
143	CRY1	1.422	0.000	cryptochrome circadian clock 1 [Source:HGNC Symbol;Acc:HGNC:2384]	chr12:107385141-107487607	NM_004075	XM_011537939	NP_004066	XP_016874321	protein_coding	ENSG0000000840	1407	2384
144	CSE1L	1.370	0.000	chromosome segregation 1 like [Source:HGNC Symbol;Acc:HGNC:2431]	chr20:47662782-47713497	NM_001256135, N	XM_011528599	NP_001243064, NP_00	XP_011526901	protein_coding	ENSG0000012420	1434	2431
145	CSRP1	-0.895	0.000	cysteine and glycine rich protein 1 [Source:HGNC Symbol;Acc:HGNC:2469]	chr1:201452657-201478584	NM_001193570, N	.	NP_001180499, NP_00	.	protein_coding	ENSG0000015917	1465	2469
146	CST1	-3.580	0.000	cystatin SN [Source:HGNC Symbol;Acc:HGNC:2473]	chr20:23728189-23731905	NM_001898	.	NP_001889	.	protein_coding	ENSG0000017037	1469	2473
147	CST5	-1.911	0.000	cystatin D [Source:HGNC Symbol;Acc:HGNC:2477]	chr20:23856571-23860387	NM_001900	.	NP_001891	.	protein_coding	ENSG0000017036	1473	2477
148	CTNNAL1	1.229	0.000	catenin alpha like 1 [Source:HGNC Symbol;Acc:HGNC:2512]	chr9:111696460-111775809	NM_001286974, N	XM_011519163, XM_0	NP_001273903, NP_00	XP_005252348	protein_coding	ENSG0000011932	8727	2512
149	CTSB	-1.068	0.000	cathepsin B [Source:HGNC Symbol;Acc:HGNC:2527]	chr8:11700032-11725738	NM_001908, NM_1	XM_011543812, XM_0	NP_001899, NP_68009	XP_006716307, XP_0067	protein_coding	ENSG0000016473	1508	2527
150	CTSD	-1.030	0.000	cathepsin D [Source:HGNC Symbol;Acc:HGNC:2529]	chr11:1753639-1785222	NM_001909	.	NP_001900	.	protein_coding	ENSG0000011798	1509	2529
151	CUL1	1.095	0.000	cullin 1 [Source:HGNC Symbol;Acc:HGNC:2551]	chr7:148395005-148498128	NM_003592	XM_011516632, XM_0	NP_003583	XP_005250117, XP_0115	protein_coding	ENSG0000005513	8454	2551
152	CX3CL1	-1.447	0.000	C-X3-C motif chemokine ligand 1 [Source:HGNC Symbol;Acc:HGNC:10647]	chr16:57406369-57418960	NM_001304392, N	.	NP_001291321, NP_00	.	protein_coding	ENSG0000000621	6376	10647
153	CXCL1	-1.237	0.000	C-X-C motif chemokine ligand 1 [Source:HGNC Symbol;Acc:HGNC:4602]	chr4:74735120-74736959	NM_001511	.	NP_001502	.	protein_coding	ENSG0000016373	2919	4602
154	CXCL8	-2.139	0.000	C-X-C motif chemokine ligand 8 [Source:HGNC Symbol;Acc:HGNC:6025]	chr4:74606222-74609433	NM_000584	.	NP_000575	.	protein_coding	ENSG0000016942	3576	6025
155	DBH	-3.553	0.000	dopamine beta-hydroxylase [Source:HGNC Symbol;Acc:HGNC:2689]	chr9:136501481-136524466	NM_000787	.	NP_000778	.	protein_coding	ENSG0000012345	1621	2689
156	DCLRE1A	1.684	0.000	DNA cross-link repair 1A [Source:HGNC Symbol;Acc:HGNC:17660]	chr10:115594483-115614142	NM_001271816, N	XM_011540429, XM_0	NP_001258745, NP_05	XP_006718153, XP_0115	protein_coding	ENSG0000019892	9937	17660
157	DCP2	1.305	0.000	decapping mRNA 2 [Source:HGNC Symbol;Acc:HGNC:24452]	chr5:112312398-112356667	NM_001242377, N	XM_017009163	NP_001229306, NP_68	XP_016864652	protein_coding	ENSG0000017279	167227	24452
158	DDAH1	1.453	0.000	dimethylarginine dimethylaminohydrolase 1 [Source:HGNC Symbol;Acc:HGNC:2715]	chr1:85784163-86044046	NM_001134445, N	XM_011541158, XM_0	NP_001127917, NP_03	XP_005270764, XP_0052	protein_coding	ENSG0000015390	23576	2715
159	DDIAS	1.967	0.000	DNA damage induced apoptosis suppressor [Source:HGNC Symbol;Acc:HGNC:26351]	chr11:82612739-82669319	NM_145018	XM_017017353, XM_0	NP_659455	XP_011543137, XP_0115	protein_coding	ENSG0000016549	220042	26351
160	DDR1	-0.927	0.000	discoidin domain receptor tyrosine kinase 1 [Source:HGNC Symbol;Acc:HGNC:2730]	chr6:30848625-30867933	NM_001202521, N	XM_017011269, XM_0	NP_001189450, NP_00	XP_006715248, XP_0115	protein_coding	ENSG0000020458	100616237	2730

161	DDX58	1.484	0.000	DEXD/H-box helicase 58 [Source:HGNC Symbol;Acc:HGNC:19102]	chr9:32455702-32526322	NM_014314	.	NP_055129	.	protein_coding	ENSG0000010720	23586	19102
162	DEFA5	-Inf	0.000	defensin alpha 5 [Source:HGNC Symbol;Acc:HGNC:2764]	chr8:6912825-6914256	NM_021010	.	NP_066290	.	protein_coding	ENSG0000016481	1670	2764
163	DENND4C	1.683	0.000	DENN domain containing 4C [Source:HGNC Symbol;Acc:HGNC:26079]	chr9:19230432-19372856	NM_017925	XM_006716806, XM_0	NP_060395	XP_006716867, XP_0067	protein_coding	ENSG0000013714	55667	26079
164	DEPDC1	1.521	0.000	DEP domain containing 1 [Source:HGNC Symbol;Acc:HGNC:22949]	chr1:68939834-68962904	NM_001114120, NN	.	NP_001107592, NP_06	.	protein_coding	ENSG0000002452	55635	22949
165	DEPDC7	1.726	0.000	DEP domain containing 7 [Source:HGNC Symbol;Acc:HGNC:29899]	chr11:33037409-33055128	NM_001077242, NN	.	NP_001070710, NP_63	.	protein_coding	ENSG0000012169	91614	29899
166	DES	-2.578	0.000	desmin [Source:HGNC Symbol;Acc:HGNC:2770]	chr2:220283098-220291461	NM_001927	.	NP_001918	.	protein_coding	ENSG0000017508	1674	2770
167	DHX15	1.231	0.000	DEAH-box helicase 15 [Source:HGNC Symbol;Acc:HGNC:2738]	chr4:24529097-24586173	NM_001358	.	NP_001349	.	protein_coding	ENSG0000010960	1665	2738
168	DHX30	-1.085	0.000	DEAH-box helicase 30 [Source:HGNC Symbol;Acc:HGNC:16716]	chr3:47844417-47891685	NM_014966, NM_1	XM_017005915, XM_0	NP_055781, NP_61952	XP_006713096, XP_0115	protein_coding	ENSG0000013215	22907	16716
169	DIAPH3	1.421	0.000	diaphanous related formin 3 [Source:HGNC Symbol;Acc:HGNC:15480]	chr13:60239716-60738121	NM_001042517, NN	XM_011535262, XM_0	NP_001035982, NP_00	XP_006719939, XP_0115	protein_coding	ENSG0000013973	81624	15480
170	DICER1	1.381	0.000	dicer 1, ribonuclease III [Source:HGNC Symbol;Acc:HGNC:17098]	chr14:95552564-95624347	NM_001195573, NN	XM_017021123, XM_0	NP_001182502, NP_00	XP_011534901, XP_0115	protein_coding	ENSG0000010069	23405	17098
171	DKK4	-4.661	0.000	dickkopf WNT signaling pathway inhibitor 4 [Source:HGNC Symbol;Acc:HGNC:2894]	chr8:42231585-42234750	NM_014420	XM_011544489, XM_0	NP_055235	XP_011542788, XP_0115	protein_coding	ENSG0000010437	27121	2894
172	DLGAP5	1.390	0.000	DLG associated protein 5 [Source:HGNC Symbol;Acc:HGNC:16864]	chr14:55614829-55658396	NM_001146015, NN	XM_017021840, XM_0	NP_001139487, NP_05	XP_011535716, XP_0168	protein_coding	ENSG0000012678	9787	16864
173	DLX3	-1.638	0.000	distal-less homeobox 3 [Source:HGNC Symbol;Acc:HGNC:2916]	chr17:48067368-48072588	NM_005220	.	NP_005211	.	protein_coding	ENSG0000006419	1747	2916
174	DMXL2	1.660	0.000	Dmx like 2 [Source:HGNC Symbol;Acc:HGNC:2938]	chr15:51739907-51915030	NM_001174116, NN	XM_005254256, XM_0	NP_001167587, NP_00	XP_005254312, XP_0052	protein_coding	ENSG0000010409	23312	2938
175	DNAJC10	1.536	0.000	DnaJ heat shock protein family (Hsp40) member C10 [Source:HGNC Symbol;Acc:HGNC:2	chr2:183580767-183659191	NM_001271581, NN	.	NP_001258510, NP_06	.	protein_coding	ENSG0000007723	54431	24637
176	DNTTIP1	-1.016	0.000	deoxynucleotidyltransferase terminal interacting protein 1 [Source:HGNC Symbol;Acc:H	chr20:44420575-44440066	NM_052951	.	NP_443183	.	protein_coding	ENSG0000010145	116092	16160
177	DNTTIP2	1.379	0.000	deoxynucleotidyltransferase terminal interacting protein 2 [Source:HGNC Symbol;Acc:H	chr1:94331838-94344762	NM_014597	.	NP_055412	.	protein_coding	ENSG0000006733	30836	24013
178	DPP7	-1.062	0.000	dipeptidyl peptidase 7 [Source:HGNC Symbol;Acc:HGNC:14892]	chr9:140004993-140009170	NM_013379	XM_011518601, XM_0	NP_037511	XP_005266132, XP_0067	protein_coding	ENSG0000017697	29952	14892
179	DPYSL3	1.390	0.000	dihydropyrimidinase like 3 [Source:HGNC Symbol;Acc:HGNC:3015]	chr5:146770373-146889619	NM_001197294, NN	XM_011537574	NP_001184223, NP_00	XP_011535876	protein_coding	ENSG0000011365	1809	3015
180	DSE	1.799	0.000	dermatan sulfate epimerase [Source:HGNC Symbol;Acc:HGNC:21144]	chr6:116601282-116766023	NM_001080976, NN	.	NP_001074445, NP_00	XP_016866284, XP_0168	protein_coding	ENSG0000011181	29940	21144
181	DST	1.774	0.000	dystonin [Source:HGNC Symbol;Acc:HGNC:1090]	chr6:56322784-56716714	NM_001144769, NN	XM_017011224, XM_0	NP_001138241, NP_00	XP_005249367, XP_0052	protein_coding	ENSG0000015191	667	1090
182	EBI3	-1.908	0.000	Epstein-Barr virus induced 3 [Source:HGNC Symbol;Acc:HGNC:3129]	chr19:4229494-4237528	NM_005755	XM_011527619	NP_005746	XP_011525921	protein_coding	ENSG0000010524	10148	3129
183	EBP	-1.401	0.000	emopamil binding protein (sterol isomerase) [Source:HGNC Symbol;Acc:HGNC:3133]	chrX:48379545-48387104	NM_006579	.	NP_006570	.	protein_coding	ENSG0000014715	10682	3133
184	ECEL1	-1.747	0.000	endothelin converting enzyme like 1 [Source:HGNC Symbol;Acc:HGNC:3147]	chr2:233344536-233352538	NM_001290787, NN	.	NP_001277716, NP_00	.	protein_coding	ENSG0000017155	9427	3147
185	ECT2	1.469	0.000	epithelial cell transforming 2 [Source:HGNC Symbol;Acc:HGNC:3155]	chr3:172468471-172539264	NM_001258315, NN	XM_017005836, XM_0	NP_001245244, NP_00	XP_005247233, XP_0067	protein_coding	ENSG0000011434	1894	3155
186	EDA	-2.134	0.000	ectodysplasin A [Source:HGNC Symbol;Acc:HGNC:3157]	chrX:68835910-69259319	NM_001005609, NN	XM_006724630	NP_001005609, NP_00	XP_006724693, XP_0168	protein_coding	ENSG0000015881	1896	3157
187	EDEM3	1.445	0.000	ER degradation enhancing alpha-mannosidase like protein 3 [Source:HGNC Symbol;Acc:	chr1:184659364-184724047	NM_001319960, NN	XM_011510014, XM_0	NP_001306889, NP_07	XP_005245556, XP_0115	protein_coding	ENSG0000011640	80267	16787
188	EFNB1	-1.611	0.000	ephrin B1 [Source:HGNC Symbol;Acc:HGNC:3226]	chrX:68048839-68061990	NM_004429	.	NP_004420	.	protein_coding	ENSG0000009077	1947	3226
189	EGFR	0.992	0.000	epidermal growth factor receptor [Source:HGNC Symbol;Acc:HGNC:3236]	chr7:55086713-55279321	NM_005228, NM_2	.	NP_005219, NP_95843	.	protein_coding	ENSG0000014664	1956	3236
190	EIF2S2	0.913	0.000	eukaryotic translation initiation factor 2 subunit beta [Source:HGNC Symbol;Acc:HGNC:3	chr20:32676103-32700138	NM_001316363, NN	XM_005260605	NP_001303292, NP_00	XP_016883607	protein_coding	ENSG0000012597	8894	3266
191	EIF4G2	1.119	0.000	eukaryotic translation initiation factor 4 gamma 2 [Source:HGNC Symbol;Acc:HGNC:329	chr11:10818596-10830657	NM_001042559, NN	.	NP_001036024, NP_00	.	protein_coding	ENSG0000011032	1982	3297
192	ELF3	-1.332	0.000	E74 like ETS transcription factor 3 [Source:HGNC Symbol;Acc:HGNC:3318]	chr1:201977072-201986316	NM_001114309, NN	.	NP_001107781, NP_00	.	protein_coding	ENSG0000016343	1999	3318
193	ELK1	-1.363	0.000	ELK1, ETS transcription factor [Source:HGNC Symbol;Acc:HGNC:3321]	chrX:47494919-47509990	NM_001114123, NN	XM_017029339	NP_001107595, NP_00	XP_016884828	protein_coding	ENSG0000012676	2002	3321
194	EML4	1.033	0.000	echinoderm microtubule associated protein like 4 [Source:HGNC Symbol;Acc:HGNC:131	chr2:42396489-42559688	NM_001145076, NN	XM_006711992, XM_0	NP_001138548, NP_06	XP_005264324, XP_0052	protein_coding	ENSG0000014392	27436	1316
195	ENTPD8	-1.747	0.000	ectonucleoside triphosphate diphosphohydrolase 8 [Source:HGNC Symbol;Acc:HGNC:24	chr9:140317801-140335901	NM_001033113, NN	XM_011518667, XM_0	NP_001028285, NP_94	XP_011516967, XP_0115	protein_coding	ENSG0000018883	377841	24860
196	EPRS	1.160	0.000	glutamyl-prolyl-tRNA synthetase [Source:HGNC Symbol;Acc:HGNC:3418]	chr1:220141942-220220000	NM_004446	.	NP_004437	.	protein_coding	ENSG0000013662	2058	3418
197	EPS8	1.632	0.000	epidermal growth factor receptor pathway substrate 8 [Source:HGNC Symbol;Acc:HGN(	chr12:15773091-15942510	NM_004447	XM_011520606, XM_0	NP_004438	XP_005253397, XP_0067	protein_coding	ENSG0000015149	2059	3420
198	EPT1	1.328	0.000	ethanolaminephosphotransferase 1 [Source:HGNC Symbol;Acc:HGNC:29361]	chr2:26531262-26618759	NM_033505	.	NP_277040	.	protein_coding	ENSG0000013801	85465	29361
199	ERBIN	1.374	0.000	erbb2 interacting protein [Source:HGNC Symbol;Acc:HGNC:15842]	chr5:65222302-65378377	NM_001006600, NN	XM_017009637, XM_0	NP_001006600, NP_00	XP_005248611, XP_0052	protein_coding	ENSG0000011285	55914	15842
200	ETFB	-1.140	0.000	electron transfer flavoprotein beta subunit [Source:HGNC Symbol;Acc:HGNC:3482]	chr19:51848422-51869672	NM_001014763, NN	.	NP_001014763, NP_00	.	protein_coding	ENSG0000010537	2109	3482
201	ETHE1	-1.113	0.000	ETHE1, persulfide dioxygenase [Source:HGNC Symbol;Acc:HGNC:23287]	chr19:44010870-44040282	NM_014297	XM_005258688, XM_0	NP_001307796, NP_05	XP_005258744	protein_coding	ENSG0000010575	23474	23287
202	EVI5L	-1.354	0.000	ecotropic viral integration site 5 like [Source:HGNC Symbol;Acc:HGNC:30464]	chr19:7895118-7929862	NM_001159944, NN	XM_005272458	NP_001153416, NP_66	XP_005272515, XP_0168	protein_coding	ENSG0000014245	115704	30464
203	EXOC1	1.368	0.000	exocyst complex component 1 [Source:HGNC Symbol;Acc:HGNC:30380]	chr4:56719781-56771200	NM_001024924, NN	XM_011534392, XM_0	NP_001020095, NP_06	XP_005265804, XP_0052	protein_coding	ENSG0000009098	55763	30380
204	EXPH5	1.502	0.000	exophilin 5 [Source:HGNC Symbol;Acc:HGNC:30578]	chr11:108376157-108464465	NM_001144763, NN	XM_017017403, XM_0	NP_001138235, NP_00	XP_005271518, XP_0115	protein_coding	ENSG0000011072	23086	30578
205	EZH2	1.267	0.000	enhancer of zeste 2 polycomb repressive complex 2 subunit [Source:HGNC Symbol;Acc:	chr7:148504475-148581379	NM_001203247, NN	XM_011515898, XM_0	NP_001190176, NP_00	XP_005250019, XP_0052	protein_coding	ENSG0000010646	2146	3527
206	F10	-2.211	0.000	coagulation factor X [Source:HGNC Symbol;Acc:HGNC:3528]	chr13:113777127-113803843	NM_000504, NM_0	XM_005268298	NP_000495, NP_00129	XP_005268355	protein_coding	ENSG0000012621	2159	3528
207	F13A1	-2.049	0.000	coagulation factor XIII A chain [Source:HGNC Symbol;Acc:HGNC:3531]	chr6:6144317-6321067	NM_000129	XM_011514342, XM_0	NP_000120	XP_006715073	protein_coding	ENSG0000012449	2162	3531
208	FAHD2B	-1.084	0.000	fumarylacetoacetate hydrolase domain containing 2B [Source:HGNC Symbol;Acc:HGNC:	chr2:97749319-97760619	NM_001320848, NN	XM_005263892	NP_001307777, NP_95	XP_005263949, XP_0115	protein_coding	ENSG0000014419	151313	25318
209	FAIM2	-2.288	0.000	Fas apoptotic inhibitory molecule 2 [Source:HGNC Symbol;Acc:HGNC:17067]	chr12:50260678-50297670	NM_012306	XM_017019040, XM_0	NP_036438	XP_005268787, XP_0168	protein_coding	ENSG0000013547	23017	17067
210	FAM111A	1.380	0.000	family with sequence similarity 111 member A [Source:HGNC Symbol;Acc:HGNC:24725]	chr11:58910220-58922512	NM_001142519, NN	.	NP_001135991, NP_00	.	protein_coding	ENSG0000016680	63901	24725
211	FAM111B	1.768	0.000	family with sequence similarity 111 member B [Source:HGNC Symbol;Acc:HGNC:24200]	chr11:58874657-58894883	NM_001142703, NN	.	NP_001136175, NP_00	.	protein_coding	ENSG0000018905	374393	24200
212	FAM129A	1.149	0.000	family with sequence similarity 129 member A [Source:HGNC Symbol;Acc:HGNC:16784]	chr1:184760163-184943682	NM_052966	XM_011509141, XM_0	NP_443198	XP_011507442, XP_0115	protein_coding	ENSG0000013584	116496	16784
213	FAM131B	-2.390	0.000	family with sequence similarity 131 member B [Source:HGNC Symbol;Acc:HGNC:22202]	chr7:143050492-143059845	NM_001031690, NN	XM_006716186, XM_0	NP_001026860, NP_00	XP_005250130, XP_0067	protein_coding	ENSG0000015978	9715	22202
214	FAM171B	1.402	0.000	family with sequence similarity 171 member B [Source:HGNC Symbol;Acc:HGNC:29412]	chr2:187558697-187630692	NM_177454	XM_005246362	NP_803237	.	protein_coding	ENSG0000014436	165215	29412

215	FAM178B	-3.082	0.000	family with sequence similarity 178 member B [Source:HGNC Symbol;Acc:HGNC:28036]	chr2:97541618-97652329	NM_001122646, N	XM_011511295, XM_0	NP_001116118, NP_05	XP_011509596, XP_0168	protein_coding	ENSG0000016875	51252	28036
216	FAM184A	3.341	0.000	family with sequence similarity 184 member A [Source:HGNC Symbol;Acc:HGNC:20991]	chr6:119280993-119470552	NM_001100411, N	.	NP_001093881, NP_00	.	protein_coding	ENSG0000011187	79632	20991
217	FAM208A	1.486	0.000	family with sequence similarity 208 member A [Source:HGNC Symbol;Acc:HGNC:30314]	chr3:56591200-56717265	NM_001112736, N	XM_011533553, XM_0	NP_001106207, NP_05	XP_005265056, XP_0067	protein_coding	ENSG0000016394	23272	30314
218	FAM91A1	1.390	0.000	family with sequence similarity 91 member A1 [Source:HGNC Symbol;Acc:HGNC:26306]	chr8:124780695-124827692	NM_001317917, N	XM_011516869	NP_001304846, NP_00	.	protein_coding	ENSG0000017685	157769	26306
219	FASTKD2	1.370	0.000	FAST kinase domains 2 [Source:HGNC Symbol;Acc:HGNC:29160]	chr2:207602486-207657233	NM_001136193, N	.	NP_001129665, NP_00	.	protein_coding	ENSG0000011824	22868	29160
220	FBXW5	-1.001	0.000	F-box and WD repeat domain containing 5 [Source:HGNC Symbol;Acc:HGNC:13613]	chr9:139834886-139839102	NM_018998	XM_011518789, XM_0	NP_061871	XP_005266146, XP_0115	protein_coding	ENSG0000015906	54461	13613
221	FBXW9	-1.435	0.000	F-box and WD repeat domain containing 9 [Source:HGNC Symbol;Acc:HGNC:28136]	chr19:12799730-12807457	NM_032301	XM_005260096	NP_115677	XP_005260153	protein_coding	ENSG0000013200	84261	28136
222	FERMT2	1.443	0.000	fermitin family member 2 [Source:HGNC Symbol;Acc:HGNC:15767]	chr14:53323985-53417815	NM_001134999, N	XM_006720010, XM_0	NP_001128471, NP_00	XP_005267342, XP_0067	protein_coding	ENSG0000007371	10979	15767
223	FGD4	1.577	0.000	FYVE, RhoGEF and PH domain containing 4 [Source:HGNC Symbol;Acc:HGNC:19125]	chr12:32552462-32798984	NM_001304480, N	XM_017018804, XM_0	NP_001291409, NP_00	XP_005253361, XP_0052	protein_coding	ENSG0000013913	121512	19125
224	FGD5	-2.264	0.000	FYVE, RhoGEF and PH domain containing 5 [Source:HGNC Symbol;Acc:HGNC:19117]	chr3:14860468-14976072	NM_001320276, N	XM_011533424, XM_0	NP_001307205, NP_68	XP_011531724, XP_0115	protein_coding	ENSG0000015478	152273	19117
225	FGD6	1.444	0.000	FYVE, RhoGEF and PH domain containing 6 [Source:HGNC Symbol;Acc:HGNC:21740]	chr12:95470524-95611240	NM_018351	.	NP_060821	.	protein_coding	ENSG0000018026	55785	21740
226	FGF3	-1.529	0.000	fibroblast growth factor 3 [Source:HGNC Symbol;Acc:HGNC:3681]	chr11:69624991-69633792	NM_005247	.	NP_005238	.	protein_coding	ENSG0000018689	2248	3681
227	FIGNL1	1.426	0.000	fidgetin like 1 [Source:HGNC Symbol;Acc:HGNC:13286]	chr7:50511825-50633154	NM_001042762, N	XM_011515472, XM_0	NP_001036227, NP_00	XP_011513771, XP_0115	protein_coding	ENSG0000013243	63979	13286
228	FKBP14	1.336	0.000	FK506 binding protein 14 [Source:HGNC Symbol;Acc:HGNC:18625]	chr7:30050202-30066300	NM_017946	.	NP_060416	.	protein_coding	ENSG0000010608	55033	18625
229	FLYWCH2	-1.139	0.000	FLYWCH family member 2 [Source:HGNC Symbol;Acc:HGNC:25178]	chr16:2933186-2949383	NM_001142499, N	XM_005255078	NP_001135971, NP_00	XP_005255135	protein_coding	ENSG0000016207	114984	25178
230	FNDC3A	1.227	0.000	fibronectin type III domain containing 3A [Source:HGNC Symbol;Acc:HGNC:20296]	chr13:49550047-49783915	NM_001079673, N	XM_017020442, XM_0	NP_001073141, NP_00	XP_011533299, XP_0168	protein_coding	ENSG0000010253	22862	20296
231	FOXN2	1.784	0.000	forkhead box N2 [Source:HGNC Symbol;Acc:HGNC:5281]	chr2:48541848-48606433	NM_002158	XM_017003959, XM_0	NP_002149	XP_005264339, XP_0052	protein_coding	ENSG0000017080	3344	5281
232	FOXO4	-1.967	0.000	forkhead box O4 [Source:HGNC Symbol;Acc:HGNC:7139]	chrX:70316046-70323385	NM_001170931, N	.	NP_001164402, NP_00	.	protein_coding	ENSG0000018448	4303	7139
233	FTSJ1	-1.330	0.000	FtsJ RNA methyltransferase homolog 1 (E. coli) [Source:HGNC Symbol;Acc:HGNC:13254]	chrX:48334408-48344752	NM_001282157, N	XM_011543895, XM_0	NP_001269086, NP_03	XP_005272652, XP_0115	protein_coding	ENSG0000006843	24140	13254
234	FXYD3	-1.874	0.000	FXYD domain containing ion transport regulator 3 [Source:HGNC Symbol;Acc:HGNC:402	chr19:35606782-35615228	NM_001136007, N	XM_017026878, XM_0	NP_001129479, NP_00	XP_005259053, XP_0115	protein_coding	ENSG0000008935	102466205	4027
235	FXYD5	-5.051	0.000	FXYD domain containing ion transport regulator 5 [Source:HGNC Symbol;Acc:HGNC:402	chr19:35645632-35660786	NM_001164605, N	XM_006723247, XM_0	NP_001158077, NP_00	XP_006723308, XP_0067	protein_coding	ENSG0000008932	53827	4029
236	FXYD6	-1.911	0.000	FXYD domain containing ion transport regulator 6 [Source:HGNC Symbol;Acc:HGNC:403	chr11:117672444-117748201	NM_001164831, N	.	NP_001158303, NP_00	.	protein_coding	ENSG0000013772	53826	4030
237	FZD3	1.635	0.000	frizzled class receptor 3 [Source:HGNC Symbol;Acc:HGNC:4041]	chr8:28351721-28431785	NM_017412, NM_1	XM_011544649, XM_0	NP_059108, NP_66587	.	protein_coding	ENSG0000010429	7976	4041
238	GALNT5	2.605	0.000	polypeptide N-acetylgalactosaminyltransferase 5 [Source:HGNC Symbol;Acc:HGNC:4127	chr2:158114109-158170723	NM_014568	.	NP_055383	XP_016858726	protein_coding	ENSG0000013654	11227	4127
239	GALNT7	1.321	0.000	polypeptide N-acetylgalactosaminyltransferase 7 [Source:HGNC Symbol;Acc:HGNC:4129	chr4:174089903-174245118	NM_017423	XM_011532025, XM_0	NP_059119	XP_005263119, XP_0115	protein_coding	ENSG0000010958	51809	4129
240	GAS2L3	1.726	0.000	growth arrest specific 2 like 3 [Source:HGNC Symbol;Acc:HGNC:27475]	chr12:100967460-101018697	NM_001303130, N	XM_011538221, XM_0	NP_001290059, NP_00	XP_005268861, XP_0115	protein_coding	ENSG0000013935	283431	27475
241	GAST	-2.490	0.000	gastrin [Source:HGNC Symbol;Acc:HGNC:4164]	chr17:39868577-39872221	NM_000805	.	NP_000796	.	protein_coding	ENSG0000018450	2520	4164
242	GATA6	1.881	0.000	GATA binding protein 6 [Source:HGNC Symbol;Acc:HGNC:4174]	chr18:19749403-19782491	NM_005257	.	NP_005248	.	protein_coding	ENSG0000014144	2627	4174
243	GBP3	1.671	0.000	guanylate binding protein 3 [Source:HGNC Symbol;Acc:HGNC:4184]	chr1:89472348-89488565	NM_018284	XM_011541234, XM_0	NP_060754	XP_005270800, XP_0067	protein_coding	ENSG0000011722	2635	4184
244	GDF15	-1.738	0.000	growth differentiation factor 15 [Source:HGNC Symbol;Acc:HGNC:30142]	chr19:18496967-18499986	NM_004864	.	NP_004855	.	protein_coding	ENSG0000013051	9518	30142
245	GGT5	-3.867	0.000	gamma-glutamyltransferase 5 [Source:HGNC Symbol;Acc:HGNC:4260]	chr22:24615621-24641110	NM_001099781, N	XM_017028769, XM_0	NP_001093251, NP_00	XP_005261614, XP_0052	protein_coding	ENSG0000009999	2687	4260
246	GJB1	-2.471	0.000	gap junction protein beta 1 [Source:HGNC Symbol;Acc:HGNC:4283]	chrX:70435043-70445366	NM_000166, NM_0	XM_011530907	NP_000157, NP_00109	XP_011529209, XP_0168	protein_coding	ENSG0000016956	2705	4283
247	GJB4	-1.893	0.000	gap junction protein beta 4 [Source:HGNC Symbol;Acc:HGNC:4286]	chr1:35225341-35229325	NM_153212	XM_011540679	NP_694944	XP_011538981	protein_coding	ENSG0000018943	127534	4286
248	GMFG	-1.552	0.000	glia maturation factor gamma [Source:HGNC Symbol;Acc:HGNC:4374]	chr19:39818992-39833012	NM_001301008, N	.	NP_001287937, NP_00	.	protein_coding	ENSG0000013075	9535	4374
249	GNAI2	-0.881	0.000	G protein subunit alpha i2 [Source:HGNC Symbol;Acc:HGNC:4385]	chr3:50263723-50296787	NM_001166425, N	.	NP_001159897, NP_00	.	protein_coding	ENSG0000011435	2771	4385
250	GOLGA7B	-1.215	0.000	golgin A7 family member B [Source:HGNC Symbol;Acc:HGNC:31668]	chr10:99609995-99790585	NM_001010917	.	NP_001010917	.	protein_coding	ENSG0000015526	401647	31668
251	GOLGA8A	1.773	0.000	golgin A8 family member A [Source:HGNC Symbol;Acc:HGNC:31972]	chr15:34671271-34681957	NM_181077	XM_017022020, XM_0	NP_851422	XP_006720503, XP_0168	protein_coding	ENSG0000017526	23015	31972
252	GPAM	1.396	0.000	glycerol-3-phosphate acyltransferase, mitochondrial [Source:HGNC Symbol;Acc:HGNC:2	chr10:113909621-113943521	NM_001244949, N	XM_005269998	NP_001231878, NP_06	XP_005270055	protein_coding	ENSG0000011992	57678	24865
253	GPBP1	1.548	0.000	GC-rich promoter binding protein 1 [Source:HGNC Symbol;Acc:HGNC:29520]	chr5:56469774-56560506	NM_001127235, N	XM_006714674, XM_0	NP_001120707, NP_00	XP_005248630, XP_0052	protein_coding	ENSG0000006219	65056	29520
254	GPD2	1.612	0.000	glycerol-3-phosphate dehydrogenase 2 [Source:HGNC Symbol;Acc:HGNC:4456]	chr2:157291952-157442915	NM_000408, NM_0	XM_011510979, XM_0	NP_000399, NP_00107	XP_005246526, XP_0115	protein_coding	ENSG0000011515	2820	4456
255	GPKOW	-1.533	0.000	G-patch domain and KOW motifs [Source:HGNC Symbol;Acc:HGNC:30677]	chrX:48970326-48980151	NM_015698	XM_017029415	NP_056513	XP_016884904	protein_coding	ENSG0000006839	27238	30677
256	GPR1	2.264	0.000	G protein-coupled receptor 1 [Source:HGNC Symbol;Acc:HGNC:4463]	chr2:207040039-207082771	NM_001098199, N	XM_005246471	NP_001091669, NP_00	XP_005246528, XP_0168	protein_coding	ENSG0000018367	2825	4463
257	GPRC5C	-1.370	0.000	G protein-coupled receptor class C group 5 member C [Source:HGNC Symbol;Acc:HGNC	chr17:72427051-72443568	NM_018653, NM_0	.	NP_061123, NP_07131	XP_005257571	protein_coding	ENSG0000017041	55890	13309
258	GRK6	-1.091	0.000	G protein-coupled receptor kinase 6 [Source:HGNC Symbol;Acc:HGNC:4545]	chr5:176853678-176869902	NM_001004105, N	XM_011534537, XM_0	NP_001004105, NP_00	XP_006714921, XP_0067	protein_coding	ENSG0000019805	2870	4545
259	GSAP	1.829	0.000	gamma-secretase activating protein [Source:HGNC Symbol;Acc:HGNC:28042]	chr7:76940067-77045717	NM_017439	XM_011516329, XM_0	NP_059135	XP_011514627, XP_0115	protein_coding	ENSG0000018608	54103	28042
260	GSDMA	-3.379	0.000	gasdermin A [Source:HGNC Symbol;Acc:HGNC:13311]	chr17:38109515-38134019	NM_178171	XM_006721832	NP_835465	XP_006721895, XP_0168	protein_coding	ENSG0000016791	284110	13311
261	GSN	-0.984	0.000	gelsolin [Source:HGNC Symbol;Acc:HGNC:4620]	chr9:123970077-124095121	NM_000177, NM_0	XM_011518593, XM_0	NP_000168, NP_00112	XP_005252002, XP_0115	protein_coding	ENSG0000014818	2934	4620
262	GTPBP6	-1.279	0.000	GTP binding protein 6 (putative) [Source:HGNC Symbol;Acc:HGNC:30189], mapped to P	chrX:221416-230884	NM_012227	XM_011546183, XM_0	NP_036359	XP_006724510, XP_0115	protein_coding	ENSG0000017860	8225	30189
263	GYG2	-2.739	0.000	glycogenin 2 [Source:HGNC Symbol;Acc:HGNC:4700]	chrX:2746828-2800859	NM_001079855, N	XM_017029929, XM_0	NP_001073324, NP_00	XP_011543902, XP_0168	protein_coding	ENSG0000005699	8908	4700
264	GZMB	-1.503	0.000	granzyme B [Source:HGNC Symbol;Acc:HGNC:4709]	chr14:25100159-25103473	NM_004131	XM_011536685	NP_004122	XP_011534987	protein_coding	ENSG0000010045	3002	4709
265	H1FX	-1.389	0.000	H1 histone family member X [Source:HGNC Symbol;Acc:HGNC:4722]	chr3:129033613-129035120	NM_006026	.	NP_006017	.	protein_coding	ENSG0000018489	8971	4722
266	HAUS6	1.548	0.000	HAUS augmin like complex subunit 6 [Source:HGNC Symbol;Acc:HGNC:25948]	chr9:19053140-19103117	NM_001270890, N	.	NP_001257819, NP_06	.	protein_coding	ENSG0000014787	54801	25948
267	HDAC11	-2.154	0.000	histone deacetylase 11 [Source:HGNC Symbol;Acc:HGNC:19086]	chr3:13521223-13547924	NM_001136041, N	XM_011534135, XM_0	NP_001129513, NP_07	XP_005265548, XP_0052	protein_coding	ENSG0000016351	79885	19086
268	HDAC6	-1.239	0.000	histone deacetylase 6 [Source:HGNC Symbol;Acc:HGNC:14064]	chrX:48660401-48683386	NM_001321225, N	XM_006724525, XM_0	NP_001308154, NP_00	XP_005272622, XP_0052	protein_coding	ENSG0000009463	10013	14064

269	HDAC9	2.339	0.000	histone deacetylase 9 [Source:HGNC Symbol;Acc:HGNC:14065]	chr7:18126571-19042039	NM_001204144, N	XM_017012830, XM_	NP_001191073, NP_00	XP_011513931, XP_0115	protein_coding	ENSG0000004805	9734	14065
270	HEPHL1	-3.516	0.000	hephaestin like 1 [Source:HGNC Symbol;Acc:HGNC:30477]	chr11:93754526-93846917	NM_001098672	.	NP_001092142	.	protein_coding	ENSG0000018133	341208	30477
271	HIF1A	1.536	0.000	hypoxia inducible factor 1 alpha subunit [Source:HGNC Symbol;Acc:HGNC:4910]	chr14:62162257-62214977	NM_001243084, N	.	NP_001230013, NP_00	.	protein_coding	ENSG0000010064	3091	4910
272	HJURP	0.927	0.000	Holliday junction recognition protein [Source:HGNC Symbol;Acc:HGNC:25444]	chr2:234745346-234763212	NM_001282962, N	.	NP_001269891, NP_00	.	protein_coding	ENSG0000012348	55355	25444
273	HLA-B	-1.151	0.000	major histocompatibility complex, class I, B [Source:HGNC Symbol;Acc:HGNC:4932]	chr6:31321648-31324964	NM_005514	XM_011514557, XM_	NP_005505	XP_011512859	protein_coding	ENSG0000023474	3106	4932
274	HLA-DQB1	-1.228	0.000	major histocompatibility complex, class II, DQ beta 1 [Source:HGNC Symbol;Acc:HGNC:4	chr6:32627243-32636160	NM_001243961, N	.	NP_001230890, NP_00	.	protein_coding	ENSG0000017934	3119	4944
275	HLA-DRA	-2.511	0.000	major histocompatibility complex, class II, DR alpha [Source:HGNC Symbol;Acc:HGNC:49	chr6:32407618-32412823	NM_019111	.	NP_061984	.	protein_coding	ENSG0000020428	3122	4947
276	HLA-DRB1	-3.064	0.000	major histocompatibility complex, class II, DR beta 1 [Source:HGNC Symbol;Acc:HGNC:4	chr6:32520766-32557625	NM_002124	.	NP_002115	.	protein_coding	ENSG0000019612	3123	4948
277	HMG20B	-1.112	0.000	high mobility group 20B [Source:HGNC Symbol;Acc:HGNC:5002]	chr19:3572935-3579086	NM_006339	XM_017026144	NP_006330	XP_016881633	protein_coding	ENSG0000006496	10362	5002
278	HMMR	1.104	0.000	hyaluronan mediated motility receptor [Source:HGNC Symbol;Acc:HGNC:5012]	chr5:162887371-162918951	NM_001142556, N	.	NP_001136028, NP_00	.	protein_coding	ENSG0000007257	3161	5012
279	HOOK3	1.649	0.000	hook microtubule tethering protein 3 [Source:HGNC Symbol;Acc:HGNC:23576]	chr8:42752074-42885682	NM_032410	.	NP_115786	.	protein_coding	ENSG0000016817	84376	23576
280	HPS5	1.545	0.000	HPS5, biogenesis of lysosomal organelles complex 2 subunit 2 [Source:HGNC Symbol;Ac	chr11:18300214-18343745	NM_007216, NM_1	XM_017017154, XM_	NP_009147, NP_85260	XP_011518164, XP_0115	protein_coding	ENSG0000011075	11234	17022
281	HSD17B10	-1.273	0.000	hydroxysteroid 17-beta dehydrogenase 10 [Source:HGNC Symbol;Acc:HGNC:4800]	chrX:53458205-53461320	NM_001037811, N	.	NP_001032900, NP_00	.	protein_coding	ENSG0000007250	3028	4800
282	HSP90AA1	1.401	0.000	heat shock protein 90 alpha family class A member 1 [Source:HGNC Symbol;Acc:HGNC:5	chr14:102547074-102606023	NM_001017963, N	XM_011536718	NP_001017963, NP_00	XP_011535020	protein_coding	ENSG0000008082	3320	5253
283	HSPA4L	2.242	0.000	heat shock protein family A (Hsp70) member 4 like [Source:HGNC Symbol;Acc:HGNC:17	chr4:128702975-128761888	NM_014278	XM_011531744	NP_001304310, NP_00	XP_011530046	protein_coding	ENSG0000016407	22824	17041
284	HSPH1	0.980	0.000	heat shock protein family H (Hsp110) member 1 [Source:HGNC Symbol;Acc:HGNC:1696	chr13:31709110-31736525	NM_001286503, N	XM_011534888, XM_	NP_001273432, NP_00	XP_005266293, XP_0115	protein_coding	ENSG0000012069	10808	16969
285	ICE2	1.499	0.000	interactor of little elongation complex ELL subunit 2 [Source:HGNC Symbol;Acc:HGNC:2	chr15:60711807-60771344	NM_001018089, N	XM_011522011, XM_	NP_001018099, NP_00	XP_005254718, XP_0052	protein_coding	ENSG0000012891	79664	29885
286	IFI27	-3.647	0.000	interferon alpha inducible protein 27 [Source:HGNC Symbol;Acc:HGNC:5397]	chr14:94577078-94583035	NM_001288954, N	.	NP_001275883, NP_00	.	protein_coding	ENSG0000016594	3429	5397
287	IFITM1	-2.571	0.000	interferon induced transmembrane protein 1 [Source:HGNC Symbol;Acc:HGNC:5412]	chr11:313505-315272	NM_003641	.	NP_003632	.	protein_coding	ENSG0000018588	8519	5412
288	IGFBP2	-1.155	0.000	insulin like growth factor binding protein 2 [Source:HGNC Symbol;Acc:HGNC:5471]	chr2:217498117-217529159	NM_000597	.	NP_000588, NP_00130	.	protein_coding	ENSG0000011545	3485	5471
289	IGFBP3	-2.290	0.000	insulin like growth factor binding protein 3 [Source:HGNC Symbol;Acc:HGNC:5472]	chr7:45951843-45960873	NM_000598, NM_0	.	NP_000589, NP_00101	XP_016867641	protein_coding	ENSG0000014667	3486	5472
290	IGFBP6	-1.502	0.000	insulin like growth factor binding protein 6 [Source:HGNC Symbol;Acc:HGNC:5475]	chr12:53491219-53496129	NM_002178	.	NP_002169	XP_016874753	protein_coding	ENSG0000016777	3489	5475
291	IL17RC	-1.667	0.000	interleukin 17 receptor C [Source:HGNC Symbol;Acc:HGNC:18358]	chr3:9958757-9975305	NM_001203263, N	XM_017007341, XM_	NP_001190192, NP_00	XP_016862815, XP_0168	protein_coding	ENSG0000016370	84818	18358
292	IL6ST	1.556	0.000	interleukin 6 signal transducer [Source:HGNC Symbol;Acc:HGNC:6021]	chr5:55230922-55290821	NM_001190981, N	.	NP_001177910, NP_00	.	protein_coding	ENSG0000013435	3572	6021
293	INTS10	1.143	0.000	integrator complex subunit 10 [Source:HGNC Symbol;Acc:HGNC:25548]	chr8:19674650-19709589	NM_018142	XM_011544564, XM_	NP_060612	XP_005273612, XP_0052	protein_coding	ENSG0000010461	55174	25548
294	INTS2	1.557	0.000	integrator complex subunit 2 [Source:HGNC Symbol;Acc:HGNC:29241]	chr17:59942727-60005377	NM_020748	XM_011525058, XM_	NP_065799	XP_006722050, XP_0115	protein_coding	ENSG0000010850	57508	29241
295	INTS6L	1.521	0.000	integrator complex subunit 6 like [Source:HGNC Symbol;Acc:HGNC:27334]	chrX:134654583-134716434	NM_182540	XM_011531310, XM_	NP_872346	XP_005262451, XP_0067	protein_coding	ENSG0000016535	203522	27334
296	IPO7	1.267	0.000	importin 7 [Source:HGNC Symbol;Acc:HGNC:9852]	chr11:9406168-9469673	NM_006391	.	NP_006382	.	protein_coding	ENSG0000020533	10527	9852
297	IRAK2	-2.499	0.000	interleukin 1 receptor associated kinase 2 [Source:HGNC Symbol;Acc:HGNC:6113]	chr3:10206548-10285427	NM_001570	XM_011533680	NP_001561	.	protein_coding	ENSG0000013407	3656	6113
298	ISG20	-1.715	0.000	interferon stimulated exonuclease gene 20 [Source:HGNC Symbol;Acc:HGNC:6130]	chr15:89179383-89199714	NM_001303233, N	XM_006720488, XM_	NP_001290162, NP_00	XP_005254956, XP_0067	protein_coding	ENSG0000017218	3669	6130
299	ITGA5	-1.077	0.000	integrin subunit alpha 5 [Source:HGNC Symbol;Acc:HGNC:6141]	chr12:54789044-54813244	NM_002205	.	NP_002196	.	protein_coding	ENSG0000016163	3678	6141
300	ITPR2	1.390	0.000	inositol 1,4,5-trisphosphate receptor type 2 [Source:HGNC Symbol;Acc:HGNC:6181]	chr12:26489447-26986131	NM_002223	XM_011520646, XM_	NP_002214	XP_016874755, XP_0168	protein_coding	ENSG0000012310	3709	6181
301	JMJD1C	1.720	0.000	jumonji domain containing 1C [Source:HGNC Symbol;Acc:HGNC:12313]	chr10:64926980-65225641	NM_001282948, N	XM_017015903, XM_	NP_001269877, NP_00	XP_011537805, XP_0168	protein_coding	ENSG0000017198	221037	12313
302	KANK4	-2.351	0.000	KN motif and ankyrin repeat domains 4 [Source:HGNC Symbol;Acc:HGNC:27263]	chr1:62702650-62785085	NM_181712	XM_011540846, XM_	NP_001307198, NP_85	XP_011539145, XP_0115	protein_coding	ENSG0000013285	163782	27263
303	KAT6A	1.399	0.000	lysine acetyltransferase 6A [Source:HGNC Symbol;Acc:HGNC:13013]	chr8:41786996-41909505	NM_001099412, N	XM_017013864, XM_	NP_001092882, NP_00	XP_011542958, XP_0115	protein_coding	ENSG0000008316	7994	13013
304	KCNN4	-1.444	0.000	potassium calcium-activated channel subfamily N member 4 [Source:HGNC Symbol;Acc:	chr19:44270684-44285409	NM_002250	XM_005258883, XM_	NP_002241	XP_005258939, XP_0052	protein_coding	ENSG0000010478	3783	6293
305	KCTD9	1.310	0.000	potassium channel tetramerization domain containing 9 [Source:HGNC Symbol;Acc:HGN	chr8:25285362-25315983	NM_017634	XM_005273545, XM_	NP_060104	.	protein_coding	ENSG0000010475	54793	22401
306	KDELC2	1.580	0.000	KDEL motif containing 2 [Source:HGNC Symbol;Acc:HGNC:28496]	chr11:108342831-108369159	NM_153705	XM_011542622, XM_	NP_714916	XP_011540923	protein_coding	ENSG0000017820	143888	28496
307	KDM5C	-1.397	0.000	lysine demethylase 5C [Source:HGNC Symbol;Acc:HGNC:11114]	chrX:53220502-53254604	NM_001146702, N	XM_011530830, XM_	NP_001140174, NP_00	XP_005262092, XP_0115	protein_coding	ENSG0000012601	8242	11114
308	KIAA1033	1.457	0.000	KIAA1033 [Source:HGNC Symbol;Acc:HGNC:29174]	chr12:105501491-105562912	NM_001293640, N	XM_011538074, XM_	NP_001280569, NP_05	XP_011536375, XP_0115	protein_coding	ENSG0000013605	23325	29174
309	KIAA1109	1.577	0.000	KIAA1109 [Source:HGNC Symbol;Acc:HGNC:26953]	chr4:123073487-123283913	NM_015312	XM_011532329, XM_	NP_056127	XP_005263339, XP_0052	protein_coding	ENSG0000013868	84162	26953
310	KIF12	-1.991	0.000	kinesin family member 12 [Source:HGNC Symbol;Acc:HGNC:21495]	chr9:116853917-116861337	NM_138424	XM_011518191, XM_	NP_612433	XP_006717010, XP_0115	protein_coding	ENSG0000013688	113220	21495
311	KIF14	1.927	0.000	kinesin family member 14 [Source:HGNC Symbol;Acc:HGNC:19181]	chr1:200520624-200589862	NM_014875	XM_017003007, XM_	NP_055690	XP_011508532, XP_0115	protein_coding	ENSG0000011819	9928	19181
312	KIF20B	1.722	0.000	kinesin family member 20B [Source:HGNC Symbol;Acc:HGNC:7212]	chr10:91461366-91534696	NM_001284259, N	.	NP_001271188, NP_05	.	protein_coding	ENSG0000013818	9585	7212
313	KIF21A	1.640	0.000	kinesin family member 21A [Source:HGNC Symbol;Acc:HGNC:19349]	chr12:39687030-39837192	NM_001173463, N	XM_017019607, XM_	NP_001166934, NP_00	XP_005269064, XP_0052	protein_coding	ENSG0000013911	55605	19349
314	KIF21B	-1.617	0.000	kinesin family member 21B [Source:HGNC Symbol;Acc:HGNC:29442]	chr1:200938517-200992828	NM_001252100, N	.	NP_001239029, NP_00	.	protein_coding	ENSG0000011685	23046	29442
315	KIF23	1.371	0.000	kinesin family member 23 [Source:HGNC Symbol;Acc:HGNC:6392]	chr15:69706584-69740766	NM_001281301, N	XM_011522239, XM_	NP_001268230, NP_00	XP_005254853, XP_0052	protein_coding	ENSG0000013780	9493	6392
316	KIF2A	1.446	0.000	kinesin family member 2A [Source:HGNC Symbol;Acc:HGNC:6318]	chr5:61601988-61682210	NM_001098511, N	.	NP_001091981, NP_00	.	protein_coding	ENSG0000006879	3796	6318
317	KIF5B	1.624	0.000	kinesin family member 5B [Source:HGNC Symbol;Acc:HGNC:6324]	chr10:32297937-32345359	NM_004521	XM_017016224	NP_004512	XP_016871713	protein_coding	ENSG0000017075	3799	6324
318	KISS1	-2.026	0.000	KiSS-1 metastasis-suppressor [Source:HGNC Symbol;Acc:HGNC:6341]	chr1:204159468-204165614	NM_002256	XM_011509525	NP_002247	XP_011507827	protein_coding	ENSG0000017049	3814	6341
319	KLHDC8B	-1.972	0.000	kelch domain containing 8B [Source:HGNC Symbol;Acc:HGNC:28557]	chr3:49209043-49213919	NM_173546	XM_006713016, XM_	NP_775817	XP_005264995, XP_0067	protein_coding	ENSG0000018590	200942	28557
320	KNTC1	1.942	0.000	kinetochore associated 1 [Source:HGNC Symbol;Acc:HGNC:17255]	chr12:123011794-123110943	NM_014708	XM_017020255, XM_	NP_055523	XP_006719769, XP_0115	protein_coding	ENSG0000018444	9735	17255
321	KRIT1	1.842	0.000	KRIT1, ankyrin repeat containing [Source:HGNC Symbol;Acc:HGNC:1573]	chr7:91828282-91875414	NM_001013406, N	XM_011516661, XM_	NP_001013424, NP_00	XP_005250717, XP_0052	protein_coding	ENSG0000000163	889	1573
322	KRT13	-3.269	0.000	keratin 13 [Source:HGNC Symbol;Acc:HGNC:6415]	chr17:39657232-39661865	NM_002274, NM_1	.	NP_002265, NP_70569	.	protein_coding	ENSG0000017140	3860	6415

323	KRT14	-Inf	0.000	keratin 14 [Source:HGNC Symbol;Acc:HGNC:6416]	chr17:39738530-39743173	NM_000526	.	NP_000517	.	protein_coding	ENSG0000018684	3861	6416
324	KRT6A	-2.271	0.000	keratin 6A [Source:HGNC Symbol;Acc:HGNC:6443]	chr12:52880957-52887041	NM_005554	.	NP_005545	.	protein_coding	ENSG0000020542	3853	6443
325	KRT7	-1.679	0.000	keratin 7 [Source:HGNC Symbol;Acc:HGNC:6445]	chr12:52626897-52642705	NM_005556	.	NP_005547	.	protein_coding	ENSG0000013548	3855	6445
326	KRT71	-3.723	0.000	keratin 71 [Source:HGNC Symbol;Acc:HGNC:28927]	chr12:52937692-52946931	NM_033448	XM_017018749	NP_258259	XP_016874238	protein_coding	ENSG0000013964	112802	28927
327	KRT74	1.241	0.000	keratin 74 [Source:HGNC Symbol;Acc:HGNC:28929]	chr12:52959565-52967609	NM_175053	XM_011537902	NP_778223	XP_011536204	protein_coding	ENSG0000017048	121391	28929
328	KRT81	-2.818	0.000	keratin 81 [Source:HGNC Symbol;Acc:HGNC:6458]	chr12:52668458-52702947	NM_002281	XM_011538334	NP_002272	XP_011536636	protein_coding	ENSG0000020542	3887	6458
329	LAMB1	1.352	0.000	laminin subunit beta 1 [Source:HGNC Symbol;Acc:HGNC:6486]	chr7:107564247-107643700	NM_002291	XM_017012201	NP_002282	XP_016867690	protein_coding	ENSG0000009113	3912	6486
330	LAMB2	-1.352	0.000	laminin subunit beta 2 [Source:HGNC Symbol;Acc:HGNC:6487]	chr3:49158546-49170551	NM_002292	XM_005265127	NP_002283	XP_005265184	protein_coding	ENSG0000017203	3913	6487
331	LAMB3	-1.393	0.000	laminin subunit beta 3 [Source:HGNC Symbol;Acc:HGNC:6490]	chr1:209788214-209825811	NM_000228, NM_0	XM_005273124	NP_000219, NP_00101	XP_005273181	protein_coding	ENSG0000019687	100422894	6490
332	LAPTM5	-2.752	0.000	lysosomal protein transmembrane 5 [Source:HGNC Symbol;Acc:HGNC:29612]	chr1:31205315-31230667	NM_006762	.	NP_006753	.	protein_coding	ENSG0000016251	7805	29612
333	LARP4	1.318	0.000	La ribonucleoprotein domain family member 4 [Source:HGNC Symbol;Acc:HGNC:24320]	chr12:50794591-50873787	NM_001170803, NN	XM_017018759, XM_0	NP_001164274, NP_00	XP_005268670, XP_0115	protein_coding	ENSG0000016181	113251	24320
334	LAS1L	-1.534	0.000	LAS1 like, ribosome biogenesis factor [Source:HGNC Symbol;Acc:HGNC:25726]	chrX:64732461-64754636	NM_001170649, NN	XM_017029877, XM_0	NP_001164120, NP_00	XP_005262358, XP_0115	protein_coding	ENSG0000000149	81887	25726
335	LBH	-1.126	0.000	limb bud and heart development [Source:HGNC Symbol;Acc:HGNC:29532]	chr2:30454396-30546596	NM_030915	.	NP_112177	.	protein_coding	ENSG0000021362	81606	29532
336	LCN2	-1.390	0.000	lipocalin 2 [Source:HGNC Symbol;Acc:HGNC:6526]	chr9:130911349-130915734	NM_005564	.	NP_005555	.	protein_coding	ENSG0000014834	3934	6526
337	LGR6	-1.307	0.000	leucine rich repeat containing G protein-coupled receptor 6 [Source:HGNC Symbol;Acc:I	chr1:202163028-202288909	NM_001017403, NN	XM_011509840, XM_0	NP_001017403, NP_00	XP_005245461, XP_0115	protein_coding	ENSG0000013306	59352	19719
338	LIMA1	1.290	0.000	LIM domain and actin binding 1 [Source:HGNC Symbol;Acc:HGNC:24636]	chr12:50569570-50677329	NM_001113546, NN	XM_011538455	NP_001107018, NP_00	XP_011536757, XP_0168	protein_coding	ENSG0000005040	51474	24636
339	LINC01420	-1.287	0.000	long intergenic non-protein coding RNA 1420 [Source:HGNC Symbol;Acc:HGNC:50713]	chrX:56755673-56844813	.	.	.	.	protein_coding	ENSG0000020427	.	50713
340	LRBA	1.204	0.000	LPS responsive beige-like anchor protein [Source:HGNC Symbol;Acc:HGNC:1742]	chr4:151185682-151936649	NM_001199282, NN	XM_017008872, XM_0	NP_001186211, NP_00	XP_005263429, XP_0052	protein_coding	ENSG0000019858	987	1742
341	LRG1	-1.305	0.000	leucine rich alpha-2-glycoprotein 1 [Source:HGNC Symbol;Acc:HGNC:29480]	chr19:4536420-4540486	NM_052972	.	NP_443204	.	protein_coding	ENSG0000017123	116844	29480
342	LRP2	2.593	0.000	LDL receptor related protein 2 [Source:HGNC Symbol;Acc:HGNC:6694]	chr2:169983618-170219195	NM_004525	XM_011511183	NP_004516	XP_011509485	protein_coding	ENSG0000008147	4036	6694
343	LRP3	-1.452	0.000	LDL receptor related protein 3 [Source:HGNC Symbol;Acc:HGNC:6695]	chr19:33685489-33716756	NM_002333	XM_005258945	NP_002324	XP_005259002	protein_coding	ENSG0000013088	4037	6695
344	LRRC32	-3.167	0.000	leucine rich repeat containing 32 [Source:HGNC Symbol;Acc:HGNC:4161]	chr11:76368567-76381791	NM_001128922, NN	XM_011544935, XM_0	NP_001122394, NP_00	XP_005273959, XP_0052	protein_coding	ENSG0000013750	2615	4161
345	LRRC55	-2.512	0.000	leucine rich repeat containing 55 [Source:HGNC Symbol;Acc:HGNC:32324]	chr11:56949220-56959191	NM_001005210	.	NP_001005210	.	protein_coding	ENSG0000018390	219527	32324
346	LRRC8C	1.768	0.000	leucine rich repeat containing 8 family member C [Source:HGNC Symbol;Acc:HGNC:250	chr1:90098630-90185092	NM_032270	XM_017002504, XM_0	NP_115646	XP_006711023, XP_0115	protein_coding	ENSG0000017148	84230	25075
347	LTB	-1.480	0.000	lymphotoxin beta [Source:HGNC Symbol;Acc:HGNC:6711]	chr6:31548331-31550204	NM_002341	.	NP_002332, NP_03366	.	protein_coding	ENSG0000022750	4050	6711
348	LTN1	2.005	0.000	listerin E3 ubiquitin protein ligase 1 [Source:HGNC Symbol;Acc:HGNC:13082]	chr21:30300465-30365277	NM_015565	XM_011529525	NP_001307695, NP_05	.	protein_coding	ENSG0000019886	26046	13082
349	LY6D	-Inf	0.000	lymphocyte antigen 6 complex, locus D [Source:HGNC Symbol;Acc:HGNC:13348]	chr8:143866295-143868008	NM_003695	.	NP_003686	.	protein_coding	ENSG0000016765	8581	13348
350	LYPD3	-1.519	0.000	LY6/PLAUR domain containing 3 [Source:HGNC Symbol;Acc:HGNC:24880]	chr19:43964938-43969812	NM_014400	.	NP_055215	.	protein_coding	ENSG0000012446	27076	24880
351	MACC1	2.275	0.000	MACC1, MET transcriptional regulator [Source:HGNC Symbol;Acc:HGNC:30215]	chr7:20174277-20257027	NM_182762	.	NP_877439	.	protein_coding	ENSG0000018374	346389	30215
352	MAGED2	-1.786	0.000	MAGE family member D2 [Source:HGNC Symbol;Acc:HGNC:16353]	chrX:54834031-54842445	NM_014599, NM_1	.	NP_055414, NP_80318	.	protein_coding	ENSG0000010231	10916	16353
353	MAL2	1.763	0.000	mal, T-cell differentiation protein 2 (gene/pseudogene) [Source:HGNC Symbol;Acc:HGN	chr8:120177272-120257913	NM_052886	.	NP_443118	.	protein_coding	ENSG0000014767	114569	13634
354	MAN2A1	1.779	0.000	mannosidase alpha class 2A member 1 [Source:HGNC Symbol;Acc:HGNC:6824]	chr5:109025066-109202879	NM_002372	XM_011543394	NP_002363	XP_016864961	protein_coding	ENSG0000011289	4124	6824
355	MAOA	-3.566	0.000	monoamine oxidase A [Source:HGNC Symbol;Acc:HGNC:6833]	chrX:43514154-43606071	NM_000240, NM_0	.	NP_000231, NP_00125	.	protein_coding	ENSG0000018922	4128	6833
356	MAP1B	1.756	0.000	microtubule associated protein 1B [Source:HGNC Symbol;Acc:HGNC:6836]	chr5:71403060-71505397	NM_001324255, NN	.	NP_001311184, NP_00	.	protein_coding	ENSG0000013171	4131	6836
357	MAP2K2	-0.932	0.000	mitogen-activated protein kinase kinase 2 [Source:HGNC Symbol;Acc:HGNC:6842]	chr19:4090318-4124126	NM_030662	.	NP_109587	.	protein_coding	ENSG0000012693	5605	6842
358	MAPKAPK3	-1.265	0.000	mitogen-activated protein kinase-activated protein kinase 3 [Source:HGNC Symbol;Acc:	chr3:50649338-50686728	NM_001243925, NN	.	NP_001230854, NP_00	.	protein_coding	ENSG0000011473	7867	6888
359	MBNL2	1.467	0.000	muscleblind like splicing regulator 2 [Source:HGNC Symbol;Acc:HGNC:16746]	chr13:97873687-98046374	NM_001306070, NN	XM_017020309, XM_0	NP_001292999, NP_65	XP_005254075, XP_0052	protein_coding	ENSG0000013979	10150	16746
360	MCM8	1.551	0.000	minichromosome maintenance 8 homologous recombination repair factor [Source:HGN	chr20:5931297-5979623	NM_001281520, NN	.	NP_001268449, NP_00	XP_016883594, XP_0168	protein_coding	ENSG0000012588	84515	16147
361	MED13	1.335	0.000	mediator complex subunit 13 [Source:HGNC Symbol;Acc:HGNC:22474]	chr17:60019965-60142643	NM_005121	XM_011525553, XM_0	NP_005112	XP_011523853, XP_0115	protein_coding	ENSG0000010851	9969	22474
362	MELTF	-1.213	0.000	melanotransferrin [Source:HGNC Symbol;Acc:HGNC:7037]	chr3:196728610-196756687	NM_005929, NM_0	XM_011512851, XM_0	NP_005920, NP_20157	XP_006713706, XP_0115	protein_coding	ENSG0000016397	4241	7037
363	MET	1.688	0.000	MET proto-oncogene, receptor tyrosine kinase [Source:HGNC Symbol;Acc:HGNC:7029]	chr7:116312445-116438440	NM_000245, NM_0	XM_011516223, XM_0	NP_000236, NP_00112	XP_006716053, XP_0115	protein_coding	ENSG0000010597	4233	7029
364	MFAP2	-1.750	0.000	microfibrillar associated protein 2 [Source:HGNC Symbol;Acc:HGNC:7033]	chr1:17300996-17307330	NM_001135247, NN	XM_011541476	NP_001128719, NP_00	XP_011539778	protein_coding	ENSG0000011712	4237	7033
365	MGA	1.343	0.000	MGA, MAX dimerization protein [Source:HGNC Symbol;Acc:HGNC:14010]	chr15:41952609-42062141	NM_001080541, NN	XM_011521398, XM_0	NP_001074010, NP_00	XP_005254300, XP_0052	protein_coding	ENSG0000017419	23269	14010
366	MGST1	1.494	0.000	microsomal glutathione S-transferase 1 [Source:HGNC Symbol;Acc:HGNC:7061]	chr12:16500075-16530126	NM_001260511, NN	.	NP_001247440, NP_00	.	protein_coding	ENSG0000000839	4257	7061
367	MIB2	-1.279	0.000	mindbomb E3 ubiquitin protein ligase 2 [Source:HGNC Symbol;Acc:HGNC:30577]	chr1:1550794-1565990	NM_001170686, NN	XM_011540746, XM_0	NP_001164157, NP_00	XP_006710435, XP_0115	protein_coding	ENSG0000019753	142678	30577
368	MID1IP1	-1.250	0.000	MID1 interacting protein 1 [Source:HGNC Symbol;Acc:HGNC:20715]	chrX:38660684-38665790	NM_001098790, NN	.	NP_001092260, NP_00	.	protein_coding	ENSG0000016517	58526	20715
369	MKLN1	1.597	0.000	muskelin 1 [Source:HGNC Symbol;Acc:HGNC:7109]	chr7:130794854-131181395	NM_001145354, NN	XM_006715993, XM_0	NP_001138826, NP_00	XP_006716056	protein_coding	ENSG0000012858	4289	7109
370	MLH3	1.861	0.000	mutL homolog 3 [Source:HGNC Symbol;Acc:HGNC:7128]	chr14:75480466-75518235	NM_001040108, NN	XM_017021219, XM_0	NP_001035197, NP_05	XP_005267588, XP_0052	protein_coding	ENSG0000011968	27030	7128
371	MMP11	-1.744	0.000	matrix metallopeptidase 11 [Source:HGNC Symbol;Acc:HGNC:7157]	chr22:24115005-24126503	NM_005940	.	NP_005931	.	protein_coding	ENSG0000009995	4320	7157
372	MMP9	-3.383	0.000	matrix metallopeptidase 9 [Source:HGNC Symbol;Acc:HGNC:7176]	chr20:44637546-44645200	NM_004994	.	NP_004985	.	protein_coding	ENSG0000010098	4318	7176
373	MPP5	1.510	0.000	membrane palmitoylated protein 5 [Source:HGNC Symbol;Acc:HGNC:18669]	chr14:67707825-67802536	NM_001256550, NN	XM_011537087, XM_0	NP_001243479, NP_07	XP_005268060, XP_0115	protein_coding	ENSG0000007241	64398	18669
374	MPZL2	-1.078	0.000	myelin protein zero like 2 [Source:HGNC Symbol;Acc:HGNC:3496]	chr11:118124117-118135251	NM_005797, NM_1	.	NP_005788, NP_65891	.	protein_coding	ENSG0000014957	10205	3496
375	MSN	-1.300	0.000	moesin [Source:HGNC Symbol;Acc:HGNC:7373]	chrX:64887536-64961793	NM_002444	XM_017029546, XM_0	NP_002435	XP_005262326, XP_0115	protein_coding	ENSG0000014706	4478	7373
376	MT1B	-Inf	0.000	metallothionein 1B [Source:HGNC Symbol;Acc:HGNC:7394]	chr16:56685810-56687116	NM_005947	.	NP_005938	.	protein_coding	ENSG0000016968	4490	7394

377	MTMR14	-1.371	0.000	myotubularin related protein 14 [Source:HGNC Symbol;Acc:HGNC:26190]	chr3:9691116-9744077	NM_001077525, NN	XM_011534013, XM_C	NP_001070993, NP_00	XP_005265457, XP_0115	protein_coding	ENSG0000016371	64419	26190
378	MTRNR2L8	0.923	0.000	MT-RNR2-like 8 [Source:HGNC Symbol;Acc:HGNC:37165]	chr11:10529433-10530736	NM_001190702	.	NP_001177631	.	protein_coding	ENSG0000025582	100616263	37165
379	MTUS1	2.181	0.000	microtubule associated tumor suppressor 1 [Source:HGNC Symbol;Acc:HGNC:29789]	chr8:17501303-17658426	NM_001001924, NN	XM_017013702, XM_C	NP_001001924, NP_00	XP_005273634, XP_0052	protein_coding	ENSG0000012942	57509	29789
380	MVP	-1.785	0.000	major vault protein [Source:HGNC Symbol;Acc:HGNC:7531]	chr16:29831714-29859360	NM_001293204, NN	.	NP_001280133, NP_00	.	protein_coding	ENSG0000001336	9961	7531
381	MYBL1	2.360	0.000	MYB proto-oncogene like 1 [Source:HGNC Symbol;Acc:HGNC:7547]	chr8:67474409-67525529	NM_001080416, NN	XM_017013455, XM_C	NP_001073885, NP_00	XP_011515835, XP_0115	protein_coding	ENSG0000018569	4603	7547
382	MYBL2	-1.158	0.000	MYB proto-oncogene like 2 [Source:HGNC Symbol;Acc:HGNC:7548]	chr20:42295658-42345136	NM_001278610, NN	.	NP_001265539, NP_00	.	protein_coding	ENSG0000010105	4605	7548
383	MYO1F	-1.994	0.000	myosin IF [Source:HGNC Symbol;Acc:HGNC:7600]	chr19:8585673-8642461	NM_012335	XM_011528027, XM_C	NP_036467	XP_011526326, XP_0115	protein_coding	ENSG0000014234	4542	7600
384	MYSM1	1.842	0.000	Myb like, SWIRM and MPN domains 1 [Source:HGNC Symbol;Acc:HGNC:29401]	chr1:59120410-59165764	NM_001085487	XM_017000197, XM_C	NP_001078956	XP_006710377, XP_0115	protein_coding	ENSG0000016260	114803	29401
385	NAMPT	1.291	0.000	nicotinamide phosphoribosyltransferase [Source:HGNC Symbol;Acc:HGNC:30092]	chr7:105888730-105925638	NM_005746	XM_005250100	NP_005737	XP_005250157	protein_coding	ENSG0000010583	10135	30092
386	NARS	1.181	0.000	asparaginyl-tRNA synthetase [Source:HGNC Symbol;Acc:HGNC:7643]	chr18:55267887-55289405	NM_004539	XM_005266700	NP_004530	XP_005266757	protein_coding	ENSG0000013444	4677	7643
387	NAT1	2.278	0.000	N-acetyltransferase 1 [Source:HGNC Symbol;Acc:HGNC:7645]	chr8:18067601-18081198	NM_000662, NM_0	XM_011544689, XM_C	NP_000653, NP_00115	XP_006716473, XP_0115	protein_coding	ENSG000001714 2	9	7645
388	NBN	1.643	0.000	nibrin [Source:HGNC Symbol;Acc:HGNC:7652]	chr8:90945563-90996944	NM_001024688, NN	XM_011517045, XM_C	NP_001019859, NP_00	XP_005250980, XP_0115	protein_coding	ENSG0000010432	4683	7652
389	NCAM1	-1.349	0.000	neural cell adhesion molecule 1 [Source:HGNC Symbol;Acc:HGNC:7656]	chr11:112831968-113149158	NM_000615, NM_0	.	NP_000606, NP_00107	.	protein_coding	ENSG0000014929	4684	7656
390	NCBP1	1.320	0.000	nuclear cap binding protein subunit 1 [Source:HGNC Symbol;Acc:HGNC:7658]	chr9:100395907-100436030	NM_002486	XM_017014735, XM_C	NP_002477	XP_011517011, XP_0115	protein_coding	ENSG0000013693	4686	7658
391	NCEH1	1.143	0.000	neutral cholesterol ester hydrolase 1 [Source:HGNC Symbol;Acc:HGNC:29260]	chr3:172348038-172429008	NM_001146276, NN	.	NP_001139748, NP_00	.	protein_coding	ENSG0000014495	57552	29260
392	NCR3LG1	1.343	0.000	natural killer cell cytotoxicity receptor 3 ligand 1 [Source:HGNC Symbol;Acc:HGNC:4240	chr11:17373272-17398888	NM_001202439	.	NP_001189368	XP_011518374, XP_0115	protein_coding	ENSG0000018821	374383	42400
393	NDRG1	-1.498	0.000	N-myc downstream regulated 1 [Source:HGNC Symbol;Acc:HGNC:7679]	chr8:134249413-134309829	NM_001135242, NN	XM_011516791	NP_001128714, NP_00	XP_011515093	protein_coding	ENSG0000010441	10397	7679
394	NEDD1	1.708	0.000	neural precursor cell expressed, developmentally down-regulated 1 [Source:HGNC Symb	chr12:97301000-97347555	NM_001135175, NN	XM_011537907, XM_C	NP_001128647, NP_00	XP_005268701, XP_0067	protein_coding	ENSG0000013935	121441	7723
395	NEDD4	1.410	0.000	neural precursor cell expressed, developmentally down-regulated 4, E3 ubiquitin protei	chr15:56119119-56285944	NM_001284338, NN	XM_011521627, XM_C	NP_001271267, NP_00	XP_011519926, XP_0115	protein_coding	ENSG0000006986	4734	7727
396	NEMP1	1.425	0.000	nuclear envelope integral membrane protein 1 [Source:HGNC Symbol;Acc:HGNC:29001]	chr12:57449425-57472552	NM_001130963, NN	XM_017019079, XM_C	NP_001124435, NP_05	XP_011536359, XP_0115	protein_coding	ENSG0000016688	23306	29001
397	NFE2L2	1.484	0.000	nuclear factor, erythroid 2 like 2 [Source:HGNC Symbol;Acc:HGNC:7782]	chr2:178095032-178408564	NM_001145412, NN	.	NP_001138884, NP_00	.	protein_coding	ENSG0000011604	4780	7782
398	NGFR	-2.643	0.000	nerve growth factor receptor [Source:HGNC Symbol;Acc:HGNC:7809]	chr17:47572654-47592379	NM_002507	.	NP_002498	.	protein_coding	ENSG0000006430	4804	7809
399	NKX3-1	1.991	0.000	NK3 homeobox 1 [Source:HGNC Symbol;Acc:HGNC:7838]	chr8:23536205-23540440	NM_001256339, NN	.	NP_001243268, NP_00	.	protein_coding	ENSG0000016703	4824	7838
400	NLN	1.128	0.000	neurolysin [Source:HGNC Symbol;Acc:HGNC:16058]	chr5:65018022-65125111	NM_020726	XM_006714661, XM_C	NP_065777	XP_005248616, XP_0067	protein_coding	ENSG0000012321	57486	16058
401	NONO	-1.042	0.000	non-POU domain containing, octamer-binding [Source:HGNC Symbol;Acc:HGNC:7871]	chrX:70503041-70521018	NM_001145408, NN	.	NP_001138880, NP_00	.	protein_coding	ENSG0000014714	4841	7871
402	NOP58	1.399	0.000	NOP58 ribonucleoprotein [Source:HGNC Symbol;Acc:HGNC:29926]	chr2:203130438-203168389	NM_015934	.	NP_057018	.	protein_coding	ENSG0000005504	51602	29926
403	NOS3	-3.963	0.000	nitric oxide synthase 3 [Source:HGNC Symbol;Acc:HGNC:7876]	chr7:150688082-150711676	NM_000603, NM_0	XM_017012232	NP_000594, NP_00115	XP_016867721, XP_0168	protein_coding	ENSG0000016486	4846	7876
404	NOTUM	-1.316	0.000	NOTUM, palmitoleoyl-protein carboxylesterase [Source:HGNC Symbol;Acc:HGNC:27106	chr17:79910382-79919169	NM_178493	XM_011523537	NP_848588	XP_011521839	protein_coding	ENSG0000018526	147111	27106
405	NPAT	1.635	0.000	nuclear protein, coactivator of histone transcription [Source:HGNC Symbol;Acc:HGNC:7	chr11:108027941-108093369	NM_001321307, NN	XM_011542855, XM_C	NP_001308236, NP_00	XP_011541156, XP_0168	protein_coding	ENSG0000014930	4863	7896
406	NPNT	1.729	0.000	nephronectin [Source:HGNC Symbol;Acc:HGNC:27405]	chr4:106816593-106892831	NM_001033047, NN	XM_011531825, XM_C	NP_001028219, NP_00	XP_005262945, XP_0052	protein_coding	ENSG0000016874	255743	27405
407	NPW	-1.604	0.000	neuropeptide W [Source:HGNC Symbol;Acc:HGNC:30509]	chr16:2069520-2070756	NM_001099456	.	NP_001092926	.	protein_coding	ENSG0000018397	283869	30509
408	NTNG2	-2.024	0.000	netrin G2 [Source:HGNC Symbol;Acc:HGNC:14288]	chr9:135037062-135119921	NM_032536	XM_011519107, XM_C	NP_115925	XP_006717367, XP_0115	protein_coding	ENSG0000019635	84628	14288
409	NUCB2	1.266	0.000	nucleobindin 2 [Source:HGNC Symbol;Acc:HGNC:8044]	chr11:17298287-17353758	NM_005013	XM_011520133, XM_C	NP_005004	XP_016873293, XP_0168	protein_coding	ENSG0000007008	105376575	8044
410	NUFIP2	1.213	0.000	NUFIP2, FMR1 interacting protein 2 [Source:HGNC Symbol;Acc:HGNC:17634]	chr17:27582853-27621136	NM_020772	.	NP_065823	.	protein_coding	ENSG0000010825	57532	17634
411	NUP107	1.322	0.000	nucleoporin 107 [Source:HGNC Symbol;Acc:HGNC:29914]	chr12:69080513-69139589	NM_020401	XM_011538576, XM_C	NP_065134	XP_005269094, XP_0115	protein_coding	ENSG0000011158	57122	29914
412	NUP155	1.402	0.000	nucleoporin 155 [Source:HGNC Symbol;Acc:HGNC:8063]	chr5:37288238-37371283	NM_001278312, NN	.	NP_001265241, NP_00	.	protein_coding	ENSG0000011356	9631	8063
413	NUP205	1.371	0.000	nucleoporin 205 [Source:HGNC Symbol;Acc:HGNC:18658]	chr7:135242666-135333505	NM_001329434, NN	XM_005250235	NP_001316363, NP_05	.	protein_coding	ENSG0000015556	23165	18658
414	NUP210	-1.298	0.000	nucleoporin 210 [Source:HGNC Symbol;Acc:HGNC:30052]	chr3:13357734-13461809	NM_024923	.	NP_079199	.	protein_coding	ENSG0000013218	23225	30052
415	OSBPL3	1.076	0.000	oxysterol binding protein like 3 [Source:HGNC Symbol;Acc:HGNC:16370]	chr7:24836157-25019807	NM_015550, NM_1	XM_011515259, XM_C	NP_056365, NP_66316	XP_005249755, XP_0067	protein_coding	ENSG0000007088	26031	16370
416	OSBPL5	-1.200	0.000	oxysterol binding protein like 5 [Source:HGNC Symbol;Acc:HGNC:16392]	chr11:3108345-3186584	NM_001144063, NN	XM_011519873	NP_001137535, NP_06	XP_011518175, XP_0168	protein_coding	ENSG0000002176	114879	16392
417	OTUD5	-1.446	0.000	OTU deubiquitinase 5 [Source:HGNC Symbol;Acc:HGNC:25402]	chrX:48779304-48815648	NM_001136157, NN	XM_011543933, XM_C	NP_001129629, NP_00	XP_005272679, XP_0052	protein_coding	ENSG0000006830	55593	25402
418	PADI1	-1.664	0.000	peptidyl arginine deiminase 1 [Source:HGNC Symbol;Acc:HGNC:18367]	chr1:17531620-17572500	NM_013358	XM_017001102, XM_C	NP_037490	XP_016856590, XP_0168	protein_coding	ENSG0000014262	29943	18367
419	PADI3	-3.562	0.000	peptidyl arginine deiminase 3 [Source:HGNC Symbol;Acc:HGNC:18337]	chr1:17575592-17610728	NM_016233	XM_011541571, XM_C	NP_057317	XP_011539873	protein_coding	ENSG0000014261	51702	18337
420	PAICS	0.920	0.000	phosphoribosylaminoimidazole carboxylase; phosphoribosylaminoimidazolesuccinocarb	chr4:57301906-57330745	NM_001079524, NN	XM_011534366	NP_001072992, NP_00	XP_011532668	protein_coding	ENSG0000012805	10606	8587
421	PALM	-1.409	0.000	paralemmin [Source:HGNC Symbol;Acc:HGNC:8594]	chr19:708938-748329	NM_001040134, NN	XM_017026850, XM_C	NP_001035224, NP_00	XP_005259622, XP_0052	protein_coding	ENSG0000009986	5064	8594
422	PAXBP1	1.542	0.000	PAX3 and PAX7 binding protein 1 [Source:HGNC Symbol;Acc:HGNC:13579]	chr21:34106209-34144169	NM_013329, NM_0	XM_006724067, XM_C	NP_037461, NP_05771	XP_006724129	protein_coding	ENSG0000015908	94104	13579
423	PBK	1.409	0.000	PDZ binding kinase [Source:HGNC Symbol;Acc:HGNC:18282]	chr8:27667136-27695612	NM_001278945, NN	XM_006716368	NP_001265874, NP_06	XP_006716431	protein_coding	ENSG0000016807	55872	18282
424	PCBP3	-2.209	0.000	poly(rC) binding protein 3 [Source:HGNC Symbol;Acc:HGNC:8651]	chr21:47063607-47362368	NM_001130141, NN	.	NP_001123613, NP_06	.	protein_coding	ENSG0000018357	54039	8651
425	PCM1	2.016	0.000	pericentriolar material 1 [Source:HGNC Symbol;Acc:HGNC:8727]	chr8:17780348-17887453	NM_001315508, NN	XM_011544542, XM_C	NP_001302436, NP_00	XP_005273578, XP_0067	protein_coding	ENSG0000007867	5108	8727
426	PCSK1N	-1.660	0.000	proprotein convertase subtilisin/kexin type 1 inhibitor [Source:HGNC Symbol;Acc:HGNC	chrX:48689503-48694035	NM_013271	.	NP_037403	.	protein_coding	ENSG0000010210	27344	17301
427	PDE2A	-2.578	0.000	phosphodiesterase 2A [Source:HGNC Symbol;Acc:HGNC:8777]	chr11:72287184-72385497	NM_001143839, NN	XM_011545096, XM_C	NP_001137311, NP_00	XP_005274097, XP_0115	protein_coding	ENSG0000018664	5138	8777
428	PDE3B	2.585	0.000	phosphodiesterase 3B [Source:HGNC Symbol;Acc:HGNC:8779]	chr11:14665268-14893590	NM_000922	.	NP_000913	.	protein_coding	ENSG0000015227	5140	8779
429	PDS5A	1.289	0.000	PDS5 cohesin associated factor A [Source:HGNC Symbol;Acc:HGNC:29088]	chr4:39824482-39979576	NM_001100399, NN	XM_011513674, XM_C	NP_001093869, NP_00	XP_011511974, XP_0168	protein_coding	ENSG0000012189	23244	29088
430	PEAK1	1.495	0.000	pseudopodium enriched atypical kinase 1 [Source:HGNC Symbol;Acc:HGNC:29431]	chr15:77392997-77712486	NM_024776	XM_011522039, XM_C	NP_079052	XP_005254727, XP_0052	protein_coding	ENSG0000017351	79834	29431

431	PELI3	-1.335	0.000	pellino E3 ubiquitin protein ligase family member 3 [Source:HGNC Symbol;Acc:HGNC:30	chr11:66233797-66244808	NM_001098510, NN	XM_017017465, XM_0	NP_001091980, NP_00	XP_011543186, XP_0168	protein_coding	ENSG0000017451	246330	30010
432	PEX1	1.610	0.000	peroxisomal biogenesis factor 1 [Source:HGNC Symbol;Acc:HGNC:8850]	chr7:92116333-92157845	NM_000466, NM_0	XM_005250433	NP_000457, NP_00126	.	protein_coding	ENSG0000012798	5189	8850
433	PFKFB4	-1.220	0.000	6-phosphofructo-2-kinase/fructose-2,6-biphosphatase 4 [Source:HGNC Symbol;Acc:HGN	chr3:48555116-48594323	NM_001317135, NN	XM_017006616, XM_0	NP_001304064, NP_00	XP_005265287, XP_0115	protein_coding	ENSG0000011426	5210	8875
434	PGK1	-1.290	0.000	phosphoglycerate kinase 1 [Source:HGNC Symbol;Acc:HGNC:8896]	chrX:77359670-77384793	NM_000291	.	NP_000282	.	protein_coding	ENSG0000010214	5230	8896
435	PGM2L1	1.706	0.000	phosphoglucomutase 2 like 1 [Source:HGNC Symbol;Acc:HGNC:20898]	chr11:74041362-74109518	NM_173582	XM_011544953	NP_775853	XP_011543255	protein_coding	ENSG0000016543	283209	20898
436	PHF14	1.381	0.000	PHD finger protein 14 [Source:HGNC Symbol;Acc:HGNC:22203]	chr7:11013498-11209257	NM_014660	XM_017012814, XM_0	NP_055475	XP_016868300, XP_0168	protein_coding	ENSG0000010644	9678	22203
437	PHKB	1.424	0.000	phosphorylase kinase regulatory subunit beta [Source:HGNC Symbol;Acc:HGNC:8927]	chr16:47495205-47735434	NM_000293, NM_0	XM_005255984, XM_0	NP_000284, NP_00102	XP_005256040, XP_0052	protein_coding	ENSG0000010289	5257	8927
438	PI3	-3.196	0.000	peptidase inhibitor 3 [Source:HGNC Symbol;Acc:HGNC:8947]	chr20:43803516-43805185	NM_002638	.	NP_002629	.	protein_coding	ENSG0000012410	5266	8947
439	PIGQ	-1.340	0.000	phosphatidylinositol glycan anchor biosynthesis class Q [Source:HGNC Symbol;Acc:HGN	chr16:616995-634136	NM_004204, NM_1	.	NP_004195, NP_68372	.	protein_coding	ENSG0000000754	9091	14135
440	PIK3AP1	1.376	0.000	phosphoinositide-3-kinase adaptor protein 1 [Source:HGNC Symbol;Acc:HGNC:30034]	chr10:98353068-98480271	NM_152309	XM_011539249, XM_0	NP_689522	XP_005269555, XP_0052	protein_coding	ENSG0000015562	118788	30034
441	PIK3C2A	1.548	0.000	phosphatidylinositol-4-phosphate 3-kinase catalytic subunit type 2 alpha [Source:HGNC	chr11:17108118-17191288	NM_001321378, NN	XM_017017924, XM_0	NP_001308307, NP_00	XP_005253034, XP_0052	protein_coding	ENSG0000001140	5286	8971
442	PINK1	-1.154	0.000	PTEN induced putative kinase 1 [Source:HGNC Symbol;Acc:HGNC:14581]	chr1:20959947-20978004	NM_032409	.	NP_115785	.	protein_coding	ENSG0000015882	102464833	14581
443	PITPNM1	-1.086	0.000	phosphatidylinositol transfer protein membrane associated 1 [Source:HGNC Symbol;Acc	chr11:67259238-67272843	NM_001130848, NN	XM_017018586, XM_0	NP_001124320, NP_00	XP_011543698, XP_0168	protein_coding	ENSG0000011069	9600	9003
444	PJA1	-1.384	0.000	praja ring finger ubiquitin ligase 1 [Source:HGNC Symbol;Acc:HGNC:16648]	chrX:68380696-68385636	NM_001032396, NN	XM_011531011, XM_0	NP_001027568, NP_07	XP_005262349, XP_0115	protein_coding	ENSG0000018119	64219	16648
445	PKN2	1.509	0.000	protein kinase N2 [Source:HGNC Symbol;Acc:HGNC:9406]	chr1:89149904-89301938	NM_006256	XM_011541771, XM_0	NP_001307636, NP_00	XP_016857272	protein_coding	ENSG0000006524	5586	9406
446	PKP1	-1.800	0.000	plakophilin 1 [Source:HGNC Symbol;Acc:HGNC:9023]	chr1:201252579-201302121	NM_000299, NM_0	.	NP_000290, NP_00100	.	protein_coding	ENSG0000008127	5317	9023
447	PKP2	1.324	0.000	plakophilin 2 [Source:HGNC Symbol;Acc:HGNC:9024]	chr12:32943678-33049774	NM_001005242, NN	.	NP_001005242, NP_00	.	protein_coding	ENSG0000005729	5318	9024
448	PLCD1	-1.914	0.000	phospholipase C delta 1 [Source:HGNC Symbol;Acc:HGNC:9060]	chr3:38048988-38071253	NM_001130964, NN	.	NP_001124436, NP_00	XP_016862111	protein_coding	ENSG0000018709	5333	9060
449	PLEK	-1.542	0.000	pleckstrin [Source:HGNC Symbol;Acc:HGNC:9070]	chr2:68592304-68624585	NM_002664	XM_011532916	NP_002655	.	protein_coding	ENSG0000011595	5341	9070
450	PLIN2	-1.168	0.000	perilipin 2 [Source:HGNC Symbol;Acc:HGNC:248]	chr9:19115767-19127574	NM_001122	XM_006716719	NP_001113	XP_016869748	protein_coding	ENSG0000014787	123	248
451	PLK2	1.322	0.000	polo like kinase 2 [Source:HGNC Symbol;Acc:HGNC:19699]	chr5:57749808-57756087	NM_001252226, NN	.	NP_001239155, NP_00	.	protein_coding	ENSG0000014563	10769	19699
452	PLK4	1.546	0.000	polo like kinase 4 [Source:HGNC Symbol;Acc:HGNC:11397]	chr4:128802015-128820350	NM_001190799, NN	XM_005262701	NP_001177728, NP_00	XP_005262758, XP_0168	protein_coding	ENSG0000014273	10733	11397
453	PLOD2	4.721	0.000	procollagen-lysine,2-oxoglutarate 5-dioxygenase 2 [Source:HGNC Symbol;Acc:HGNC:908	chr3:145787226-145878954	NM_000935, NM_1	XM_005247535	NP_000926, NP_89198	XP_005247592, XP_0168	protein_coding	ENSG0000015295	5352	9082
454	PLP2	-1.110	0.000	proteolipid protein 2 [Source:HGNC Symbol;Acc:HGNC:9087]	chrX:49028272-49031588	NM_002668	.	NP_002659	.	protein_coding	ENSG0000010200	5355	9087
455	PLS1	1.416	0.000	plastin 1 [Source:HGNC Symbol;Acc:HGNC:9090]	chr3:142315228-142432506	NM_001145319, NN	XM_017006627, XM_0	NP_001138791, NP_00	XP_006713723, XP_0115	protein_coding	ENSG0000012075	5357	9090
456	PNN	1.177	0.000	pinin, desmosome associated protein [Source:HGNC Symbol;Acc:HGNC:9162]	chr14:39644386-39652422	NM_002687	.	NP_002678	.	protein_coding	ENSG0000010094	5411	9162
457	PNPLA4	-1.615	0.000	patatin like phospholipase domain containing 4 [Source:HGNC Symbol;Acc:HGNC:24887	chrX:7866287-7895780	NM_001142389, NN	XM_011545588	NP_001135861, NP_00	XP_011543890	protein_coding	ENSG0000000675	8228	24887
458	PON2	1.451	0.000	paraoxonase 2 [Source:HGNC Symbol;Acc:HGNC:9205]	chr7:95034174-95064641	NM_000305, NM_0	XM_011516333, XM_0	NP_000296, NP_00101	XP_005250510	protein_coding	ENSG0000010585	5445	9205
459	PORCN	-1.942	0.000	porcupine homolog (Drosophila) [Source:HGNC Symbol;Acc:HGNC:17652]	chrX:48367349-48379202	NM_001282167, NN	XM_011543948, XM_0	NP_001269096, NP_07	XP_005272692, XP_0052	protein_coding	ENSG0000010231	64840	17652
460	PPM1M	-1.313	0.000	protein phosphatase, Mg2+/Mn2+ dependent 1M [Source:HGNC Symbol;Acc:HGNC:265	chr3:52279808-52284613	NM_001122870, NN	XM_005264881, XM_0	NP_001116342, NP_65	XP_005264936, XP_0052	protein_coding	ENSG0000016408	132160	26506
461	PPP1R12A	1.382	0.000	protein phosphatase 1 regulatory subunit 12A [Source:HGNC Symbol;Acc:HGNC:7618]	chr12:80167342-80329240	NM_001143885, NN	XM_011538385, XM_0	NP_001137357, NP_00	XP_005268944, XP_0115	protein_coding	ENSG0000005827	4659	7618
462	PPP1R14D	-2.491	0.000	protein phosphatase 1 regulatory inhibitor subunit 14D [Source:HGNC Symbol;Acc:HGN	chr15:41107642-41120907	NM_001130143, NN	.	NP_001123615, NP_06	.	protein_coding	ENSG0000016614	54866	14953
463	PPP2R2A	1.442	0.000	protein phosphatase 2 regulatory subunit Balpha [Source:HGNC Symbol;Acc:HGNC:9304	chr8:26149006-26230196	NM_001177591, NN	XM_011544567, XM_0	NP_001171062, NP_00	XP_005273616, XP_0052	protein_coding	ENSG0000022191	5520	9304
464	PPP2R3B	-1.415	0.000	protein phosphatase 2 regulatory subunit B''beta [Source:HGNC Symbol;Acc:HGNC:1341	chrX:294697-347445	NM_013239	XM_011546177	NP_037371	XP_011543932, XP_0115	protein_coding	ENSG0000016739	28227	13417
465	PRAF2	-1.950	0.000	PRA1 domain family member 2 [Source:HGNC Symbol;Acc:HGNC:28911]	chrX:48928812-48958059	NM_007213	.	NP_009144	.	protein_coding	ENSG0000024327	11230	28911
466	PRDX4	-1.676	0.000	peroxiredoxin 4 [Source:HGNC Symbol;Acc:HGNC:17169]	chrX:23685562-23704516	NM_006406	.	NP_006397	.	protein_coding	ENSG0000012313	10549	17169
467	PREPL	1.299	0.000	prolyl endopeptidase-like [Source:HGNC Symbol;Acc:HGNC:30228]	chr2:44502596-44589001	NM_001042385, NN	XM_017005385, XM_0	NP_001035844, NP_00	XP_011531500, XP_0115	protein_coding	ENSG0000013807	9581	30228
468	PRICKLE3	-1.361	0.000	prickle planar cell polarity protein 3 [Source:HGNC Symbol;Acc:HGNC:6645]	chrX:49031902-49042845	NM_001307979, NN	XM_011543913, XM_0	NP_001294908, NP_00	XP_005272662	protein_coding	ENSG0000001221	4007	6645
469	PRKCD	-1.190	0.000	protein kinase C delta [Source:HGNC Symbol;Acc:HGNC:9399]	chr3:53195135-53226733	NM_006254, NM_2	XM_006713259, XM_0	NP_001303256, NP_00	XP_006713320, XP_0067	protein_coding	ENSG0000016393	5580	9399
470	PRKD3	1.212	0.000	protein kinase D3 [Source:HGNC Symbol;Acc:HGNC:9408]	chr2:37477644-37545037	NM_005813	XM_011532732, XM_0	NP_005804	XP_005264294, XP_0052	protein_coding	ENSG0000011582	23683	9408
471	PROSC	1.353	0.000	proline synthetase cotranscribed homolog (bacterial) [Source:HGNC Symbol;Acc:HGNC:	chr8:37620110-37637285	NM_007198	.	NP_009129	.	protein_coding	ENSG0000014747	11212	9457
472	PRRT3	-1.962	0.000	proline rich transmembrane protein 3 [Source:HGNC Symbol;Acc:HGNC:26591]	chr3:9987225-9994078	NM_001318871, NN	XM_011533630, XM_0	NP_001305800, NP_99	XP_011531930, XP_0115	protein_coding	ENSG0000016370	285368	26591
473	PRRX2	-1.617	0.000	paired related homeobox 2 [Source:HGNC Symbol;Acc:HGNC:21338]	chr9:132427919-132484953	NM_016307	.	NP_057391	.	protein_coding	ENSG0000016715	51450	21338
474	PRSS3	-1.357	0.000	protease, serine 3 [Source:HGNC Symbol;Acc:HGNC:9486]	chr9:33750463-33799229	NM_001197097, NN	XM_011517965	NP_001184026, NP_00	XP_011516267	protein_coding	ENSG0000001043	5646	9486
475	PRSS56	-1.774	0.000	protease, serine 56 [Source:HGNC Symbol;Acc:HGNC:39433]	chr2:233385172-233390426	NM_001195129	.	NP_001182058	.	protein_coding	ENSG0000023741	646960	39433
476	PSD3	1.900	0.000	pleckstrin and Sec7 domain containing 3 [Source:HGNC Symbol;Acc:HGNC:19093]	chr8:18384810-18871196	NM_015310, NM_2	XM_017013261, XM_0	NP_056125, NP_99679	XP_011542772, XP_0168	protein_coding	ENSG0000015601	23362	19093
477	PSD4	-1.058	0.000	pleckstrin and Sec7 domain containing 4 [Source:HGNC Symbol;Acc:HGNC:19096]	chr2:113931547-113966973	NM_012455	XM_011510888, XM_0	NP_036587	XP_005263691, XP_0067	protein_coding	ENSG0000012563	23550	19096
478	PTBP3	1.255	0.000	polypyrimidine tract binding protein 3 [Source:HGNC Symbol;Acc:HGNC:10253]	chr9:114979995-115095947	NM_001163788, NN	XM_017015365, XM_0	NP_001157260, NP_00	XP_006717409, XP_0115	protein_coding	ENSG0000011931	9991	10253
479	PTGDS	-3.160	0.000	prostaglandin D2 synthase [Source:HGNC Symbol;Acc:HGNC:9592]	chr9:139871956-139876190	NM_000954	.	NP_000945	.	protein_coding	ENSG0000010731	5730	9592
480	PTH1R	-2.001	0.000	parathyroid hormone 1 receptor [Source:HGNC Symbol;Acc:HGNC:9608]	chr3:46919235-46945285	NM_000316, NM_0	XM_011533968, XM_0	NP_000307, NP_00117	XP_005265401, XP_0115	protein_coding	ENSG0000016080	5745	9608
481	PTOV1	-1.037	0.000	prostate tumor overexpressed 1 [Source:HGNC Symbol;Acc:HGNC:9632]	chr19:50354010-50364001	NM_001305105, NN	XM_011527035, XM_0	NP_001292034, NP_00	XP_005259055, XP_0052	protein_coding	ENSG0000010496	53635	9632
482	PTP4A3	-1.529	0.000	protein tyrosine phosphatase type IVA, member 3 [Source:HGNC Symbol;Acc:HGNC:963	chr8:142402092-142442554	NM_007079, NM_0	XM_017012998, XM_0	NP_009010, NP_11600	XP_005250820, XP_0052	protein_coding	ENSG0000018448	11156	9636
483	PTPN11	1.015	0.000	protein tyrosine phosphatase, non-receptor type 11 [Source:HGNC Symbol;Acc:HGNC:9	chr12:112856712-112947717	NM_002834, NM_0	XM_017019722, XM_0	NP_002825, NP_54216	XP_006719589, XP_0115	protein_coding	ENSG0000017929	5781	9644
484	PTPN12	1.191	0.000	protein tyrosine phosphatase, non-receptor type 12 [Source:HGNC Symbol;Acc:HGNC:9	chr7:77166591-77269388	NM_001131008, NN	XM_011516444, XM_0	NP_001124480, NP_00	XP_005250575, XP_0067	protein_coding	ENSG0000012794	5782	9645

485	PTPN7	-1.934	0.000	protein tyrosine phosphatase, non-receptor type 7 [Source:HGNC Symbol;Acc:HGNC:96	chr1:202116140-202130709	NM_001199797, NN	XM_011509819	NP_001186726, NP_00	XP_011508121, XP_0168	protein_coding	ENSG0000014385	5778	9659
486	PTPRZ1	1.810	0.000	protein tyrosine phosphatase, receptor type Z1 [Source:HGNC Symbol;Acc:HGNC:9685]	chr7:121513142-121702090	NM_001206838, NN	XM_017012477, XM_0	NP_001193767, NP_00	XP_005250576, XP_0168	protein_coding	ENSG0000010627	5803	9685
487	PUM3	1.446	0.000	pumilio RNA binding family member 3 [Source:HGNC Symbol;Acc:HGNC:29676]	chr9:2804163-2844241	NM_014878	.	NP_055693	.	protein_coding	ENSG0000008060	9933	29676
488	QARS	-1.084	0.000	glutaminyl-tRNA synthetase [Source:HGNC Symbol;Acc:HGNC:9751]	chr3:49133364-49142553	NM_001272073, NN	.	NP_001259002, NP_00	XP_016862454	protein_coding	ENSG0000017205	102465536	9751
489	QSER1	1.645	0.000	glutamine and serine rich 1 [Source:HGNC Symbol;Acc:HGNC:26154]	chr11:32914723-33014862	NM_001076786	XM_017018328, XM_0	NP_001070254	XP_016873817, XP_0168	protein_coding	ENSG0000006074	79832	26154
490	RAB26	-1.531	0.000	RAB26, member RAS oncogene family [Source:HGNC Symbol;Acc:HGNC:14259]	chr16:2198144-2204166	NM_001308053, NN	XM_011522449, XM_0	NP_001294982, NP_05	XP_011520750, XP_0115	protein_coding	ENSG0000016796	25837	14259
491	RAB3B	1.332	0.000	RAB3B, member RAS oncogene family [Source:HGNC Symbol;Acc:HGNC:9778]	chr1:52373627-52456436	NM_002867	.	NP_002858	XP_016857447	protein_coding	ENSG0000016921	5865	9778
492	RAD23B	0.946	0.000	RAD23 homolog B, nucleotide excision repair protein [Source:HGNC Symbol;Acc:HGNC:9	chr9:110045559-110094475	NM_001244713, NN	.	NP_001231642, NP_00	.	protein_coding	ENSG0000011931	5887	9813
493	RAD50	1.731	0.000	RAD50 double strand break repair protein [Source:HGNC Symbol;Acc:HGNC:9816]	chr5:131892629-131982036	NM_005732	.	NP_005723	.	protein_coding	ENSG0000011352	10111	9816
494	RAD54B	1.801	0.000	RAD54 homolog B (S. cerevisiae) [Source:HGNC Symbol;Acc:HGNC:17228]	chr8:95384187-95487337	NM_001205262, NN	.	NP_001192191, NP_03	.	protein_coding	ENSG0000019727	25788	17228
495	RAI14	1.438	0.000	retinoic acid induced 14 [Source:HGNC Symbol;Acc:HGNC:14873]	chr5:34656341-34832732	NM_001145520, NN	XM_017009334, XM_0	NP_001138992, NP_00	XP_006714532, XP_0115	protein_coding	ENSG0000003956	26064	14873
496	RAI2	-2.269	0.000	retinoic acid induced 2 [Source:HGNC Symbol;Acc:HGNC:9835]	chrX:17818168-17879457	NM_001172732, NN	XM_011545441, XM_0	NP_001166203, NP_00	XP_006724522, XP_0067	protein_coding	ENSG0000013183	10742	9835
497	RALGAPB	1.086	0.000	Ral GTPase activating protein non-catalytic beta subunit [Source:HGNC Symbol;Acc:HGN	chr20:37101458-37207504	NM_001282917, NN	XM_017027969, XM_0	NP_001269846, NP_00	XP_005260519, XP_0052	protein_coding	ENSG0000017047	57148	29221
498	RANBP2	1.447	0.000	RAN binding protein 2 [Source:HGNC Symbol;Acc:HGNC:9848]	chr2:109335936-109402267	NM_006267	XM_011511578, XM_0	NP_006258	XP_005264059, XP_0052	protein_coding	ENSG0000015320	5903	9848
499	RANBP6	1.817	0.000	RAN binding protein 6 [Source:HGNC Symbol;Acc:HGNC:9851]	chr9:6011042-6015618	NM_001243203, NN	.	NP_001230132, NP_03	.	protein_coding	ENSG0000013704	26953	9851
500	RARG	-1.437	0.000	retinoic acid receptor gamma [Source:HGNC Symbol;Acc:HGNC:9866]	chr12:53604350-53626040	NM_000966, NM_0	XM_011538628, XM_0	NP_000957, NP_00103	XP_005269113, XP_0052	protein_coding	ENSG0000017281	5916	9866
501	RARS	1.295	0.000	arginyl-tRNA synthetase [Source:HGNC Symbol;Acc:HGNC:9870]	chr5:167913449-167946304	NM_002887	.	NP_002878	.	protein_coding	ENSG0000011364	5917	9870
502	REV3L	1.733	0.000	REV3 like, DNA directed polymerase zeta catalytic subunit [Source:HGNC Symbol;Acc:HG	chr6:111620233-111804918	NM_001286431, NN	XM_011536032, XM_0	NP_001273360, NP_00	XP_006715606, XP_0067	protein_coding	ENSG0000000941	5980	9968
503	RFC1	1.277	0.000	replication factor C subunit 1 [Source:HGNC Symbol;Acc:HGNC:9969]	chr4:39289075-39367995	NM_001204747, NN	XM_011513731, XM_0	NP_001191676, NP_00	XP_011512032, XP_0115	protein_coding	ENSG0000003592	5981	9969
504	RGS9	-2.987	0.000	regulator of G-protein signaling 9 [Source:HGNC Symbol;Acc:HGNC:10004]	chr17:63097103-63223821	NM_001081955, NN	.	NP_001075424, NP_00	.	protein_coding	ENSG0000010837	8787	10004
505	RIF1	1.621	0.000	replication timing regulatory factor 1 [Source:HGNC Symbol;Acc:HGNC:23207]	chr2:152266396-152338686	NM_001177663, NN	XM_011511395, XM_0	NP_001171134, NP_00	XP_005246722, XP_0115	protein_coding	ENSG0000008034	55183	23207
506	RIMS4	-2.553	0.000	regulating synaptic membrane exocytosis 4 [Source:HGNC Symbol;Acc:HGNC:16183]	chr20:43374420-43438979	NM_001205317, NN	.	NP_001192246, NP_89	.	protein_coding	ENSG0000010109	140730	16183
507	RINT1	1.714	0.000	RAD50 interactor 1 [Source:HGNC Symbol;Acc:HGNC:21876]	chr7:105172531-105222137	NM_021930	XM_017012497	NP_068749	XP_016867986	protein_coding	ENSG0000013524	60561	21876
508	RLBP1	-1.281	0.000	retinaldehyde binding protein 1 [Source:HGNC Symbol;Acc:HGNC:10024]	chr15:89753099-89764982	NM_000326	XM_011521870	NP_000317	XP_011520172, XP_0168	protein_coding	ENSG0000014052	6017	10024
509	ROBO1	1.498	0.000	roundabout guidance receptor 1 [Source:HGNC Symbol;Acc:HGNC:10249]	chr3:78646389-79816965	NM_001145845, NN	XM_017006985, XM_0	NP_001139317, NP_00	XP_006713340, XP_0115	protein_coding	ENSG0000016985	6091	10249
510	ROBO4	-4.706	0.000	roundabout guidance receptor 4 [Source:HGNC Symbol;Acc:HGNC:17985]	chr11:124753586-124768177	NM_001301088, NN	XM_006718861	NP_001288017, NP_06	XP_006718924	protein_coding	ENSG0000015413	54538	17985
511	ROCK1	1.583	0.000	Rho associated coiled-coil containing protein kinase 1 [Source:HGNC Symbol;Acc:HGNC:	chr18:18526866-18691812	NM_005406	XM_011526136	NP_005397	.	protein_coding	ENSG0000006790	6093	10251
512	ROCK2	1.450	0.000	Rho associated coiled-coil containing protein kinase 2 [Source:HGNC Symbol;Acc:HGNC:	chr2:11319886-11484711	NM_001321643, NN	XM_011510417, XM_0	NP_001308572, NP_00	XP_005246247, XP_0115	protein_coding	ENSG0000013431	9475	10252
513	RPAP3	1.498	0.000	RNA polymerase II associated protein 3 [Source:HGNC Symbol;Acc:HGNC:26151]	chr12:48055031-48099844	NM_001146075, NN	.	NP_001139547, NP_00	.	protein_coding	ENSG0000000517	79657	26151
514	RPL15	-1.302	0.000	ribosomal protein L15 [Source:HGNC Symbol;Acc:HGNC:10306]	chr3:23933150-24022109	NM_001253379, NN	XM_011533986	NP_001240308, NP_00	XP_011532288	protein_coding	ENSG0000017474	6138	10306
515	RPL24	-1.069	0.000	ribosomal protein L24 [Source:HGNC Symbol;Acc:HGNC:10325]	chr3:101399935-101405626	NM_000986	.	NP_000977	.	protein_coding	ENSG0000011439	6152	10325
516	RPL32	-1.276	0.000	ribosomal protein L32 [Source:HGNC Symbol;Acc:HGNC:10336]	chr3:12837970-12883087	NM_000994, NM_0	.	NP_000985, NP_00100	.	protein_coding	ENSG0000014471	6161	10336
517	RPSA	-1.107	0.000	ribosomal protein SA [Source:HGNC Symbol;Acc:HGNC:6502]	chr3:39448179-39454033	NM_001304288, NN	.	NP_001291217, NP_00	.	protein_coding	ENSG0000016802	3921	6502
518	RSF1	1.312	0.000	remodeling and spacing factor 1 [Source:HGNC Symbol;Acc:HGNC:18118]	chr11:77371040-77532063	NM_016578	XM_005274051	NP_057662	XP_005274108, XP_0168	protein_coding	ENSG0000004864	51773	18118
519	S100A14	-3.032	0.000	S100 calcium binding protein A14 [Source:HGNC Symbol;Acc:HGNC:18901]	chr1:153586730-153589462	NM_020672	XM_017001875, XM_0	NP_065723	XP_005245419, XP_0168	protein_coding	ENSG0000018933	57402	18901
520	S100P	-3.781	0.000	S100 calcium binding protein P [Source:HGNC Symbol;Acc:HGNC:10504]	chr4:6694795-6698896	NM_005980	.	NP_005971	.	protein_coding	ENSG0000016399	6286	10504
521	SARDH	-2.071	0.000	sarcosine dehydrogenase [Source:HGNC Symbol;Acc:HGNC:10536]	chr9:136528681-136605077	NM_001134707, NN	XM_011518333, XM_0	NP_001128179, NP_00	XP_006717053, XP_0168	protein_coding	ENSG0000012345	1757	10536
522	SAT1	-1.327	0.000	spermidine/spermine N1-acetyltransferase 1 [Source:HGNC Symbol;Acc:HGNC:10540]	chrX:23801289-23804343	NM_002970	.	NP_002961	.	protein_coding	ENSG0000013006	6303	10540
523	SCAMP5	-1.574	0.000	secretory carrier membrane protein 5 [Source:HGNC Symbol;Acc:HGNC:30386]	chr15:75287872-75313837	NM_001178111, NN	XM_011521316, XM_0	NP_001171582, NP_00	XP_006720483, XP_0067	protein_coding	ENSG0000019879	192683	30386
524	SCAP	-1.383	0.000	SREBF chaperone [Source:HGNC Symbol;Acc:HGNC:30634]	chr3:47455183-47517603	NM_001320044, NN	XM_017005918, XM_0	NP_001306973, NP_03	XP_005265024, XP_0052	protein_coding	ENSG0000011465	22937	30634
525	SCGB2A1	-Inf	0.000	secretoglobin family 2A member 1 [Source:HGNC Symbol;Acc:HGNC:7051]	chr11:61976139-61981411	NM_002407	.	NP_002398	.	protein_coding	ENSG0000012493	4246	7051
526	SCRN1	1.157	0.000	secernin 1 [Source:HGNC Symbol;Acc:HGNC:22192]	chr7:29959718-30029905	NM_001145513, NN	XM_011515653, XM_0	NP_001138985, NP_00	XP_005249975, XP_0115	protein_coding	ENSG0000013619	9805	22192
527	SCYL2	1.437	0.000	SCY1 like pseudokinase 2 [Source:HGNC Symbol;Acc:HGNC:19286]	chr12:100661023-100735502	NM_001317784, NN	XM_017019619, XM_0	NP_001304713, NP_06	XP_005269075, XP_0168	protein_coding	ENSG0000013602	55681	19286
528	SDC4	-0.958	0.000	syndecan 4 [Source:HGNC Symbol;Acc:HGNC:10661]	chr20:43953927-43977064	NM_002999	XM_011528977	NP_002990	XP_011527279	protein_coding	ENSG0000012414	6385	10661
529	SEC13	-1.115	0.000	SEC13 homolog, nuclear pore and COPII coat complex component [Source:HGNC Symbo	chr3:10342614-10362862	NM_001136026, NN	XM_017007019, XM_0	NP_001129498, NP_00	XP_005265436, XP_0067	protein_coding	ENSG0000015702	6396	10697
530	SELPLG	-2.981	0.000	selectin P ligand [Source:HGNC Symbol;Acc:HGNC:10722]	chr12:109016052-109027735	NM_001206609, NN	XM_005269076	NP_001193538, NP_00	XP_005269133	protein_coding	ENSG0000011087	6404	10722
531	SEMA3A	2.359	0.000	semaphorin 3A [Source:HGNC Symbol;Acc:HGNC:10723]	chr7:83585092-83824761	NM_006080	XM_011515735, XM_0	NP_006071	XP_005250167, XP_0052	protein_coding	ENSG0000007521	10371	10723
532	SEMA3B	-1.385	0.000	semaphorin 3B [Source:HGNC Symbol;Acc:HGNC:10724]	chr3:50305039-50314610	NM_001005914, NN	.	NP_001005914, NP_00	.	protein_coding	ENSG0000001217	102465526	10724
533	SEMA3F	-1.585	0.000	semaphorin 3F [Source:HGNC Symbol;Acc:HGNC:10728]	chr3:50192523-50226508	NM_001318798, NN	XM_011533999, XM_0	NP_001305727, NP_00	XP_005265438, XP_0052	protein_coding	ENSG0000000161	6405	10728
534	SEMA6B	-1.284	0.000	semaphorin 6B [Source:HGNC Symbol;Acc:HGNC:10739]	chr19:4542604-4559820	NM_032108	XM_011527641, XM_0	NP_115484	XP_011525941, XP_0115	protein_coding	ENSG0000016768	10501	10739
535	SERPINA1	-1.539	0.000	serpin family A member 1 [Source:HGNC Symbol;Acc:HGNC:8941]	chr14:94843083-94857030	NM_000295, NM_0	.	NP_000286, NP_00100	XP_016876859	protein_coding	ENSG0000019724	5265	8941
536	SERPINA3	-2.859	0.000	serpin family A member 3 [Source:HGNC Symbol;Acc:HGNC:16]	chr14:95078720-95090392	.	.	.	.	protein_coding	ENSG0000019613	.	16
537	SERPINA5	-2.243	0.000	serpin family A member 5 [Source:HGNC Symbol;Acc:HGNC:8723]	chr14:95027427-95059457	NM_000624	.	NP_000615	.	protein_coding	ENSG0000018848	5104	8723
538	SETX	1.400	0.000	senataxin [Source:HGNC Symbol;Acc:HGNC:445]	chr9:135136742-135230372	NM_015046	XM_017014496, XM_0	NP_055861	XP_005272228, XP_0052	protein_coding	ENSG0000010729	23064	445

539	SF3B1	1.351	0.000	splicing factor 3b subunit 1 [Source:HGNC Symbol;Acc:HGNC:10768]	chr2:198254507-198299815	NM_001005526, NN	XM_011510868, XM_0	NP_001005526, NP_00	.	protein_coding	ENSG0000011552	23451	10768
540	SFRP5	-1.292	0.000	secreted frizzled related protein 5 [Source:HGNC Symbol;Acc:HGNC:10779]	chr10:99526507-99531709	NM_003015	.	NP_003006	.	protein_coding	ENSG0000012005	6425	10779
541	SGO2	1.526	0.000	shugoshin 2 [Source:HGNC Symbol;Acc:HGNC:30812]	chr2:201390879-201448505	NM_001160033, NN	XM_017003453, XM_0	NP_001153505, NP_00	XP_005246401, XP_0052	protein_coding	ENSG0000016353	151246	30812
542	SH2D4A	1.103	0.000	SH2 domain containing 4A [Source:HGNC Symbol;Acc:HGNC:26102]	chr8:19171127-19253729	NM_001174159, NN	XM_011544610	NP_001167630, NP_00	XP_011542912	protein_coding	ENSG0000010461	63898	26102
543	SH3BP5	-1.180	0.000	SH3 domain binding protein 5 [Source:HGNC Symbol;Acc:HGNC:10827]	chr3:15296359-15382875	NM_001018009, NN	XM_011534254, XM_0	NP_001018009, NP_00	XP_011532553, XP_0115	protein_coding	ENSG0000013137	9467	10827
544	SH3TC1	-1.372	0.000	SH3 domain and tetratricopeptide repeats 1 [Source:HGNC Symbol;Acc:HGNC:26009]	chr4:8201090-8242830	NM_001318480, NN	XM_017008300, XM_0	NP_001305409, NP_06	XP_011511790, XP_0115	protein_coding	ENSG0000012508	54436	26009
545	SH3YL1	1.389	0.000	SH3 and SYLF domain containing 1 [Source:HGNC Symbol;Acc:HGNC:29546]	chr2:218135-264810	NM_001159597, NN	.	NP_001153069, NP_00	.	protein_coding	ENSG0000003511	26751	29546
546	SHISA5	-1.022	0.000	shisa family member 5 [Source:HGNC Symbol;Acc:HGNC:30376]	chr3:48509196-48542259	NM_001272065, NN	XM_011533796, XM_0	NP_001258994, NP_00	XP_005265262, XP_0067	protein_coding	ENSG0000016405	51246	30376
547	SHOC2	1.313	0.000	SHOC2, leucine rich repeat scaffold protein [Source:HGNC Symbol;Acc:HGNC:15454]	chr10:112679304-112773425	NM_001269039, NN	XM_017016704, XM_0	NP_001255968, NP_00	XP_016872191, XP_0168	protein_coding	ENSG0000010806	8036	15454
548	SKIV2L2	1.345	0.000	Ski2 like RNA helicase 2 [Source:HGNC Symbol;Acc:HGNC:18734]	chr5:54603587-54830878	NM_015360	.	NP_056175	.	protein_coding	ENSG0000003912	23517	18734
549	SLC16A2	-1.610	0.000	solute carrier family 16 member 2 [Source:HGNC Symbol;Acc:HGNC:10923]	chrX:73641295-73753752	NM_006517	.	NP_006508	.	protein_coding	ENSG0000014710	6567	10923
550	SLC1A1	2.035	0.000	solute carrier family 1 member 1 [Source:HGNC Symbol;Acc:HGNC:10939]	chr9:4490443-4587469	NM_004170	XM_011518010, XM_0	NP_004161	XP_011516309, XP_0115	protein_coding	ENSG0000010668	6505	10939
551	SLC1A3	1.791	0.000	solute carrier family 1 member 3 [Source:HGNC Symbol;Acc:HGNC:10941]	chr5:36606456-36688436	NM_001166695, NN	XM_011514084, XM_0	NP_001160167, NP_00	XP_005248399, XP_0115	protein_coding	ENSG0000007921	6507	10941
552	SLC25A6	-1.503	0.000	solute carrier family 25 member 6 [Source:HGNC Symbol;Acc:HGNC:10992], mapped to	chrX:1505044-1511617	NM_001636	.	NP_001627	.	protein_coding	ENSG0000016910	293	10992
553	SLC26A4	2.648	0.000	solute carrier family 26 member 4 [Source:HGNC Symbol;Acc:HGNC:8818]	chr7:107301079-107358254	NM_000441	XM_005250425	NP_000432	XP_005250482	protein_coding	ENSG0000009113	5172	8818
554	SLC27A1	-1.492	0.000	solute carrier family 27 member 1 [Source:HGNC Symbol;Acc:HGNC:10995]	chr19:17581252-17616977	NM_198580	XM_011528003, XM_0	NP_940982	XP_011526302, XP_0115	protein_coding	ENSG0000013030	376497	10995
555	SLC30A2	-2.173	0.000	solute carrier family 30 member 2 [Source:HGNC Symbol;Acc:HGNC:11013]	chr1:26363742-26372624	NM_001004434, NN	.	NP_001004434, NP_11	.	protein_coding	ENSG0000015801	7780	11013
556	SLC30A6	1.476	0.000	solute carrier family 30 member 6 [Source:HGNC Symbol;Acc:HGNC:19305]	chr2:32390909-32449448	NM_001193513, NN	XM_011532963, XM_0	NP_001180442, NP_00	XP_011531261, XP_0115	protein_coding	ENSG0000015268	55676	19305
557	SLC38A5	-1.192	0.000	solute carrier family 38 member 5 [Source:HGNC Symbol;Acc:HGNC:18070]	chrX:48316919-48328644	NM_033518	XM_017029961, XM_0	NP_277053	XP_005272751, XP_0052	protein_coding	ENSG0000001748	92745	18070
558	SLC39A14	1.232	0.000	solute carrier family 39 member 14 [Source:HGNC Symbol;Acc:HGNC:20858]	chr8:22224761-22291642	NM_001128431, NN	XM_017013295, XM_0	NP_001121903, NP_00	XP_005273522, XP_0052	protein_coding	ENSG0000010463	23516	20858
559	SLC40A1	2.240	0.000	solute carrier family 40 member 1 [Source:HGNC Symbol;Acc:HGNC:10909]	chr2:190425304-190445613	NM_014585	XM_005246505	NP_055400	XP_005246562, XP_0168	protein_coding	ENSG0000013844	30061	10909
560	SLC41A3	-1.339	0.000	solute carrier family 41 member 3 [Source:HGNC Symbol;Acc:HGNC:31046]	chr3:125725197-125820400	NM_001008485, NN	XM_017006707, XM_0	NP_001008485, NP_00	XP_005247616, XP_0052	protein_coding	ENSG0000011454	54946	31046
561	SLC7A11	1.706	0.000	solute carrier family 7 member 11 [Source:HGNC Symbol;Acc:HGNC:11059]	chr4:139085250-139163503	NM_014331	XM_011531802, XM_0	NP_055146	XP_011530104	protein_coding	ENSG0000015101	23657	11059
562	SLC7A2	2.580	0.000	solute carrier family 7 member 2 [Source:HGNC Symbol;Acc:HGNC:11060]	chr8:17354596-17428082	NM_001008539, NN	XM_017013747, XM_0	NP_001008539, NP_00	XP_005273667, XP_0052	protein_coding	ENSG0000000398	6542	11060
563	SLF1	1.762	0.000	SMC5-SMC6 complex localization factor 1 [Source:HGNC Symbol;Acc:HGNC:25408]	chr5:93488670-94033326	NM_032290	XM_011543687, XM_0	NP_115666	XP_005272170, XP_0115	protein_coding	ENSG0000013330	84250	25408
564	SLPI	-1.867	0.000	secretory leukocyte peptidase inhibitor [Source:HGNC Symbol;Acc:HGNC:11092]	chr20:43880879-43883205	NM_003064	.	NP_003055	.	protein_coding	ENSG0000012410	6590	11092
565	SMARCA1	1.347	0.000	SWI/SNF related, matrix associated, actin dependent regulator of chromatin, subfamily	chrX:128580481-128657477	NM_001282874, NN	XM_006724782, XM_0	NP_001269803, NP_00	XP_005262518, XP_0052	protein_coding	ENSG0000010203	6594	11097
566	SMARCA5	1.157	0.000	SWI/SNF related, matrix associated, actin dependent regulator of chromatin, subfamily	chr4:144434615-144478639	NM_003601	.	NP_003592	.	protein_coding	ENSG0000015314	8467	11101
567	SMARCAD1	1.443	0.000	SWI/SNF-related, matrix-associated actin-dependent regulator of chromatin, subfamily	chr4:95128995-95212443	NM_001128429, NN	XM_017008463	NP_001121901, NP_00	XP_016863952	protein_coding	ENSG0000016310	56916	18398
568	SMC2	1.294	0.000	structural maintenance of chromosomes 2 [Source:HGNC Symbol;Acc:HGNC:14011]	chr9:106856540-106903698	NM_001042550, NN	XM_017014208, XM_0	NP_001036015, NP_00	XP_006716996, XP_0115	protein_coding	ENSG0000013682	10592	14011
569	SMC3	1.414	0.000	structural maintenance of chromosomes 3 [Source:HGNC Symbol;Acc:HGNC:2468]	chr10:112327448-112364394	NM_005445	.	NP_005436	.	protein_coding	ENSG0000010805	9126	2468
570	SMC5	1.516	0.000	structural maintenance of chromosomes 5 [Source:HGNC Symbol;Acc:HGNC:20465]	chr9:72873877-72969804	NM_015110	XM_005251839, XM_0	NP_055925	XP_005251894, XP_0052	protein_coding	ENSG0000019888	23137	20465
571	SMCHD1	1.479	0.000	structural maintenance of chromosomes flexible hinge domain containing 1 [Source:HG	chr18:2655736-2805015	NM_015295	XM_011525645, XM_0	NP_056110	XP_011523944, XP_0115	protein_coding	ENSG0000010159	23347	29090
572	SMCO2	-3.184	0.000	single-pass membrane protein with coiled-coil domains 2 [Source:HGNC Symbol;Acc:HG	chr12:27619742-27655118	NM_001145010	XM_011520634	NP_001138482	XP_011518936	protein_coding	ENSG0000016593	341346	34448
573	SMIM10L2A	-1.983	0.000	small integral membrane protein 10 like 2A [Source:HGNC Symbol;Acc:HGNC:34499]	chrX:134555867-134561999	.	.	.	.	protein_coding	ENSG0000017894	.	34499
574	SMS	-1.116	0.000	spermine synthase [Source:HGNC Symbol;Acc:HGNC:11123]	chrX:21958690-22012953	NM_001258423, NN	XM_011545568, XM_0	NP_001245352, NP_00	XP_005274639, XP_0115	protein_coding	ENSG0000010217	6611	11123
575	SMURF2	1.414	0.000	SMAD specific E3 ubiquitin protein ligase 2 [Source:HGNC Symbol;Acc:HGNC:16809]	chr17:62541482-62658186	NM_022739	XM_011525128, XM_0	NP_073576	XP_005257642, XP_0115	protein_coding	ENSG0000010885	64750	16809
576	SNCG	-2.438	0.000	synuclein gamma [Source:HGNC Symbol;Acc:HGNC:11141]	chr10:88718386-88723017	NM_003087	XM_011540105	NP_003078	XP_011538407	protein_coding	ENSG0000017326	6623	11141
577	SNTA1	-1.331	0.000	syntrophin alpha 1 [Source:HGNC Symbol;Acc:HGNC:11167]	chr20:31995760-32031698	NM_003098	XM_011529008, XM_0	NP_003089	XP_005260574, XP_0115	protein_coding	ENSG0000010140	6640	11167
578	SNX12	-1.166	0.000	sorting nexin 12 [Source:HGNC Symbol;Acc:HGNC:14976]	chrX:70276181-70293276	NM_001256185, NN	.	NP_001243114, NP_00	.	protein_coding	ENSG0000014716	29934	14976
579	SNX13	1.626	0.000	sorting nexin 13 [Source:HGNC Symbol;Acc:HGNC:21335]	chr7:17830384-17980117	NM_015132	XM_017011897, XM_0	NP_055947	XP_005249729, XP_0115	protein_coding	ENSG0000007118	23161	21335
580	SPAG9	1.214	0.000	sperm associated antigen 9 [Source:HGNC Symbol;Acc:HGNC:14524]	chr17:49039534-49198226	NM_001130527, NN	XM_017025284, XM_0	NP_001123999, NP_00	XP_005257825, XP_0052	protein_coding	ENSG0000000829	9043	14524
581	SPRR2D	-2.550	0.000	small proline rich protein 2D [Source:HGNC Symbol;Acc:HGNC:11264]	chr1:153012200-153014407	NM_006945	XM_017002175	NP_008876	XP_016857664	protein_coding	ENSG0000016321	6703	11264
582	SRBD1	1.509	0.000	S1 RNA binding domain 1 [Source:HGNC Symbol;Acc:HGNC:25521]	chr2:45615818-45838420	NM_018079	XM_011532946	NP_060549	XP_011531248	protein_coding	ENSG0000006878	55133	25521
583	STEAP1	1.328	0.000	STEAP family member 1 [Source:HGNC Symbol;Acc:HGNC:11378]	chr7:89783688-89794141	NM_012449	.	NP_036581	.	protein_coding	ENSG0000016464	26872	11378
584	STEAP2	1.679	0.000	STEAP2 metalloreductase [Source:HGNC Symbol;Acc:HGNC:17885]	chr7:89840999-89867451	NM_001040665, NN	XM_017011957, XM_0	NP_001035755, NP_00	XP_006715984, XP_0168	protein_coding	ENSG0000015721	261729	17885
585	STIL	1.357	0.000	SCL/TAL1 interrupting locus [Source:HGNC Symbol;Acc:HGNC:10879]	chr1:47715810-47779819	NM_001048166, NN	XM_017002123, XM_0	NP_001041631, NP_00	XP_006710897, XP_0115	protein_coding	ENSG0000012347	6491	10879
586	STK17B	1.599	0.000	serine/threonine kinase 17b [Source:HGNC Symbol;Acc:HGNC:11396]	chr2:196998289-197041227	NM_004226	XM_011512170, XM_0	NP_004217	XP_011510470, XP_0115	protein_coding	ENSG0000008132	9262	11396
587	SUCO	1.774	0.000	SUN domain containing ossification factor [Source:HGNC Symbol;Acc:HGNC:1240]	chr1:172501488-172580973	NM_001282750, NN	XM_006711376, XM_0	NP_001269679, NP_00	XP_006711437, XP_0067	protein_coding	ENSG0000009497	51430	1240
588	SULT2B1	-1.367	0.000	sulfotransferase family 2B member 1 [Source:HGNC Symbol;Acc:HGNC:11459]	chr19:49055331-49102682	NM_004605, NM_1	.	NP_004596, NP_81444	.	protein_coding	ENSG0000008800	6820	11459
589	SUPT16H	0.998	0.000	SPT16 homolog, facilitates chromatin remodeling subunit [Source:HGNC Symbol;Acc:HG	chr14:21819630-21852425	NM_007192	XM_011536381	NP_009123	XP_011534683	protein_coding	ENSG0000009220	11198	11465
590	SYTL1	-1.259	0.000	synaptotagmin like 1 [Source:HGNC Symbol;Acc:HGNC:15584]	chr1:27668510-27680423	NM_001193308, NN	XM_011542317, XM_0	NP_001180237, NP_11	XP_005246079, XP_0067	protein_coding	ENSG0000014276	84958	15584
591	SYTL2	2.811	0.000	synaptotagmin like 2 [Source:HGNC Symbol;Acc:HGNC:15585]	chr11:85405266-85522202	NM_001162951, NN	XM_011545116, XM_0	NP_001156423, NP_00	XP_005274114, XP_0052	protein_coding	ENSG0000013750	54843	15585
592	TACC1	1.688	0.000	transforming acidic coiled-coil containing protein 1 [Source:HGNC Symbol;Acc:HGNC:11	chr8:38585703-38710546	NM_001122824, NN	XM_017013774, XM_0	NP_001116296, NP_00	XP_005273682, XP_0052	protein_coding	ENSG0000014752	6867	11522

593	TAF2	1.347	0.000	TATA-box binding protein associated factor 2 [Source:HGNC Symbol;Acc:HGNC:11536]	chr8:120743014-120845103	NM_003184	XM_011517261, XM_0	NP_003175	XP_006716684, XP_0115	protein_coding	ENSG0000006431	6873	11536
594	TAOK1	1.399	0.000	TAO kinase 1 [Source:HGNC Symbol;Acc:HGNC:29259]	chr17:27717617-27878922	NM_020791, NM_0	XM_011525060	NP_065842, NP_07941	XP_011523362	protein_coding	ENSG0000016055	100616122	29259
595	TAX1BP1	1.638	0.000	Tax1 binding protein 1 [Source:HGNC Symbol;Acc:HGNC:11575]	chr7:27778991-27869384	NM_001079864, NN	XM_005249900	NP_001073333, NP_00	XP_005249957, XP_0168	protein_coding	ENSG0000010605	8887	11575
596	TBC1D10C	-3.305	0.000	TBC1 domain family member 10C [Source:HGNC Symbol;Acc:HGNC:24702]	chr11:67171385-67177560	NM_001256508, NN	XM_011545001, XM_0	NP_001243437, NP_94	XP_006718601, XP_0067	protein_coding	ENSG0000017546	374403	24702
597	TBC1D25	-1.353	0.000	TBC1 domain family member 25 [Source:HGNC Symbol;Acc:HGNC:8092]	chrX:48397844-48420997	NM_002536	.	NP_002527	.	protein_coding	ENSG0000006835	4943	8092
598	TBC1D8B	1.972	0.000	TBC1 domain family member 8B [Source:HGNC Symbol;Acc:HGNC:24715]	chrX:106045909-106119375	NM_017752, NM_1	XM_006724670, XM_0	NP_060222, NP_94258	XP_006724732, XP_0067	protein_coding	ENSG0000013313	54885	24715
599	TCF12	1.340	0.000	transcription factor 12 [Source:HGNC Symbol;Acc:HGNC:11623]	chr15:57210820-57582051	NM_001306219, NN	XM_017022520, XM_0	NP_001293148, NP_00	XP_011520261, XP_0115	protein_coding	ENSG0000014026	6938	11623
600	TCTA	-1.234	0.000	T-cell leukemia translocation altered [Source:HGNC Symbol;Acc:HGNC:11692]	chr3:49449638-49453908	NM_022171	.	NP_071503	.	protein_coding	ENSG0000014502	6988	11692
601	TENM3	1.515	0.000	teneurin transmembrane protein 3 [Source:HGNC Symbol;Acc:HGNC:29944]	chr4:183164581-183724177	NM_001080477	XM_017008393, XM_0	NP_001073946	XP_016863874, XP_0168	protein_coding	ENSG0000021833	55714	29944
602	TES	1.041	0.000	testin LIM domain protein [Source:HGNC Symbol;Acc:HGNC:14620]	chr7:115850546-115898837	NM_015641, NM_1	XM_005250258	NP_056456, NP_69004	XP_005250315	protein_coding	ENSG0000013526	26136	14620
603	TEX19	-1.544	0.000	testis expressed 19 [Source:HGNC Symbol;Acc:HGNC:33802]	chr17:80317122-80321652	NM_207459	XM_017024639, XM_0	NP_997342	XP_016880127, XP_0168	protein_coding	ENSG0000018245	400629	33802
604	TFE3	-1.263	0.000	transcription factor binding to IGHM enhancer 3 [Source:HGNC Symbol;Acc:HGNC:1175	chrX:48886241-48901012	NM_001282142, NN	XM_011543952, XM_0	NP_001269071, NP_00	.	protein_coding	ENSG0000006832	7030	11752
605	TFRC	1.077	0.000	transferrin receptor [Source:HGNC Symbol;Acc:HGNC:11763]	chr3:195776154-195809060	NM_001128148, NN	.	NP_001121620, NP_00	.	protein_coding	ENSG0000007227	7037	11763
606	THBS1	1.407	0.000	thrombospondin 1 [Source:HGNC Symbol;Acc:HGNC:11785]	chr15:39873293-39891667	NM_003246	XM_011521971, XM_0	NP_003237	XP_011520272, XP_0115	protein_coding	ENSG0000013780	7057	11785
607	THOC2	1.404	0.000	THO complex 2 [Source:HGNC Symbol;Acc:HGNC:19073]	chrX:122734412-122866906	NM_001081550	XM_017029679, XM_0	NP_001075019	XP_005262507, XP_0168	protein_coding	ENSG0000012567	57187	19073
608	TIMP1	-1.630	0.000	TIMP metallopeptidase inhibitor 1 [Source:HGNC Symbol;Acc:HGNC:11820]	chrX:47420498-47479252	NM_003254	.	NP_003245	.	protein_coding	ENSG0000010226	7076	11820
609	TJP1	1.288	0.000	tight junction protein 1 [Source:HGNC Symbol;Acc:HGNC:11827]	chr15:29991570-30114706	NM_001301025, NN	XM_017022530, XM_0	NP_001287954, NP_00	XP_005254674, XP_0052	protein_coding	ENSG0000010406	7082	11827
610	TJP3	-1.368	0.000	tight junction protein 3 [Source:HGNC Symbol;Acc:HGNC:11829]	chr19:3708106-3750811	NM_001267560, NN	.	NP_001254489, NP_00	.	protein_coding	ENSG0000010528	27134	11829
611	TKT	-1.428	0.000	transketolase [Source:HGNC Symbol;Acc:HGNC:11834]	chr3:53258722-53290047	NM_001064, NM_0	XM_011534055, XM_0	NP_001055, NP_00112	XP_011532356, XP_0115	protein_coding	ENSG0000016393	7086	11834
612	TMED8	1.240	0.000	transmembrane p24 trafficking protein family member 8 [Source:HGNC Symbol;Acc:HG	chr14:77801363-77857840	NM_213601	XM_017021224, XM_0	NP_998766	XP_016876712, XP_0168	protein_coding	ENSG0000010058	283578	18633
613	TMEM132A	-1.100	0.000	transmembrane protein 132A [Source:HGNC Symbol;Acc:HGNC:31092]	chr11:60691947-60719257	NM_017870, NM_1	.	NP_060340, NP_82117	XP_016873440, XP_0168	protein_coding	ENSG0000000611	54972	31092
614	TMEM158	-1.739	0.000	transmembrane protein 158 (gene/pseudogene) [Source:HGNC Symbol;Acc:HGNC:3029	chr3:45265957-45267770	NM_015444	.	NP_056259	.	protein_coding	ENSG0000024999	25907	30293
615	TMEM176B	-2.228	0.000	transmembrane protein 176B [Source:HGNC Symbol;Acc:HGNC:29596]	chr7:150488372-150502208	NM_001101311, NN	XM_006715933	NP_001094781, NP_00	XP_006715996	protein_coding	ENSG0000010656	28959	29596
616	TMEM2	1.261	0.000	transmembrane protein 2 [Source:HGNC Symbol;Acc:HGNC:11869]	chr9:74298281-74383800	NM_001135820, NN	XM_005251869	NP_001129292, NP_03	XP_005251926	protein_coding	ENSG0000013504	23670	11869
617	TMIE	-2.014	0.000	transmembrane inner ear [Source:HGNC Symbol;Acc:HGNC:30800]	chr3:46742822-46752376	NM_147196	.	NP_671729	.	protein_coding	ENSG0000018158	259236	30800
618	TMPRSS4	-5.396	0.000	transmembrane protease, serine 4 [Source:HGNC Symbol;Acc:HGNC:11878]	chr11:117874348-117992605	NM_001083947, NN	XM_011542902, XM_0	NP_001077416, NP_00	XP_005271670, XP_0052	protein_coding	ENSG0000013764	56649	11878
619	TMPRSS6	-3.616	0.000	transmembrane protease, serine 6 [Source:HGNC Symbol;Acc:HGNC:16517]	chr22:37461475-37505603	NM_001289000, NN	XM_011529989, XM_0	NP_001275929, NP_00	XP_006724225, XP_0067	protein_coding	ENSG0000018704	164656	16517
620	TNF	-2.034	0.000	tumor necrosis factor [Source:HGNC Symbol;Acc:HGNC:11892]	chr6:31543343-31546113	NM_000594	.	NP_000585	.	protein_coding	ENSG0000023281	7124	11892
621	TNFRSF18	-2.068	0.000	TNF receptor superfamily member 18 [Source:HGNC Symbol;Acc:HGNC:11914]	chr1:1138887-1142071	NM_004195, NM_1	.	NP_004186, NP_68369	.	protein_coding	ENSG0000018689	8784	11914
622	TNIP1	-1.020	0.000	TNFAIP3 interacting protein 1 [Source:HGNC Symbol;Acc:HGNC:16903]	chr5:150409505-150467221	NM_001252385, NN	XM_011537538, XM_0	NP_001239314, NP_00	XP_005268412, XP_0067	protein_coding	ENSG0000014590	10318	16903
623	TNK2	-1.040	0.000	tyrosine kinase non receptor 2 [Source:HGNC Symbol;Acc:HGNC:19297]	chr3:195590234-195635955	NM_001010938, NN	XM_017005510, XM_0	NP_001010938, NP_00	XP_005269325, XP_0052	protein_coding	ENSG0000006193	10188	19297
624	TNNC1	-4.308	0.000	troponin C1, slow skeletal and cardiac type [Source:HGNC Symbol;Acc:HGNC:11943]	chr3:52485117-52488086	NM_003280	.	NP_003271	.	protein_coding	ENSG0000011485	7134	11943
625	TNPO1	1.425	0.000	transportin 1 [Source:HGNC Symbol;Acc:HGNC:6401]	chr5:72112138-72212560	NM_002270, NM_1	XM_005248501, XM_0	NP_002261, NP_69485	XP_005248557, XP_0052	protein_coding	ENSG0000008331	3842	6401
626	TOP2A	1.667	0.000	topoisomerase (DNA) II alpha [Source:HGNC Symbol;Acc:HGNC:11989]	chr17:38544767-38574202	NM_001067	XM_011525165, XM_0	NP_001058	XP_005257689, XP_0115	protein_coding	ENSG0000013174	7153	11989
627	TOR1AIP1	1.412	0.000	torsin 1A interacting protein 1 [Source:HGNC Symbol;Acc:HGNC:29456]	chr1:179851176-179894135	NM_001267578, NN	XM_011509404, XM_0	NP_001254507, NP_05	XP_011507705, XP_0115	protein_coding	ENSG0000014333	26092	29456
628	TOR1AIP2	1.206	0.000	torsin 1A interacting protein 2 [Source:HGNC Symbol;Acc:HGNC:24055]	chr1:179809101-179846938	NM_001199260, NN	XM_005244939	NP_001186189, NP_07	XP_005244996, XP_0168	protein_coding	ENSG0000016990	163590	24055
629	TPR	1.306	0.000	translocated promoter region, nuclear basket protein [Source:HGNC Symbol;Acc:HGNC:	chr1:186265410-186344457	NM_003292	XM_011509955	NP_003283	.	protein_coding	ENSG0000004741	7175	12017
630	TPRA1	-1.160	0.000	transmembrane protein adipocyte associated 1 [Source:HGNC Symbol;Acc:HGNC:30413	chr3:127291911-127309931	NM_001136053, NN	XM_011512422, XM_0	NP_001129525, NP_00	XP_006713558, XP_0067	protein_coding	ENSG0000016387	131601	30413
631	TRAK2	1.390	0.000	trafficking kinesin protein 2 [Source:HGNC Symbol;Acc:HGNC:13206]	chr2:202241929-202316302	NM_015049	XM_011511691, XM_0	NP_055864	XP_011509992	protein_coding	ENSG0000011599	66008	13206
632	TREM2	-Inf	0.000	triggering receptor expressed on myeloid cells 2 [Source:HGNC Symbol;Acc:HGNC:1776	chr6:41126243-41130924	NM_001271821, NN	.	NP_001258750, NP_06	.	protein_coding	ENSG0000009597	54209	17761
633	TRPV6	-2.056	0.000	transient receptor potential cation channel subfamily V member 6 [Source:HGNC Symbo	chr7:142568955-142583507	NM_018646	.	NP_061116	.	protein_coding	ENSG0000016512	55503	14006
634	TSPAN1	-1.417	0.000	tetraspanin 1 [Source:HGNC Symbol;Acc:HGNC:20657]	chr1:46640758-46651630	NM_005727	.	NP_005718	.	protein_coding	ENSG0000011747	10103	20657
635	TSPAN12	1.735	0.000	tetraspanin 12 [Source:HGNC Symbol;Acc:HGNC:21641]	chr7:120427373-120498411	NM_012338	XM_011515994, XM_0	NP_036470	XP_005250296, XP_0115	protein_coding	ENSG0000010602	23554	21641
636	TSPAN5	1.074	0.000	tetraspanin 5 [Source:HGNC Symbol;Acc:HGNC:17753]	chr4:99391517-99579780	NM_005723	XM_005262680	NP_005714	XP_005262737	protein_coding	ENSG0000016878	10098	17753
637	TSPAN8	2.773	0.000	tetraspanin 8 [Source:HGNC Symbol;Acc:HGNC:11855]	chr12:71518864-71980090	NM_004616	XM_017019913, XM_0	NP_004607	XP_006719646, XP_0168	protein_coding	ENSG0000012732	7103	11855
638	TSPO	-1.180	0.000	translocator protein [Source:HGNC Symbol;Acc:HGNC:1158]	chr22:43547519-43559248	NM_000714, NM_0	.	NP_000705, NP_00124	.	protein_coding	ENSG0000010030	706	1158
639	TSR2	-1.251	0.000	TSR2, ribosome maturation factor [Source:HGNC Symbol;Acc:HGNC:25455]	chrX:54466833-54522599	NM_058163	.	NP_477511	.	protein_coding	ENSG0000015852	90121	25455
640	TTC3	1.265	0.000	tetratricopeptide repeat domain 3 [Source:HGNC Symbol;Acc:HGNC:12393]	chr21:38445570-38575413	NM_001001894, NN	XM_011529742, XM_0	NP_001001894, NP_00	XP_005261105, XP_0052	protein_coding	ENSG0000018267	7267	12393
641	TTC37	1.558	0.000	tetratricopeptide repeat domain 37 [Source:HGNC Symbol;Acc:HGNC:23639]	chr5:94799598-94890711	NM_014639	.	NP_055454	.	protein_coding	ENSG0000019867	9652	23639
642	TWF2	-1.416	0.000	twinfilin actin binding protein 2 [Source:HGNC Symbol;Acc:HGNC:9621]	chr3:52255095-52273276	NM_007284	.	NP_009215	.	protein_coding	ENSG0000024759	11344	9621
643	TWISTNB	1.938	0.000	TWIST neighbor [Source:HGNC Symbol;Acc:HGNC:18027]	chr7:19735084-19748710	NM_001002926	.	NP_001002926	.	protein_coding	ENSG0000010584	221830	18027
644	TXNRD1	1.183	0.000	thioredoxin reductase 1 [Source:HGNC Symbol;Acc:HGNC:12437]	chr12:104609556-104744083	NM_001093771, NN	.	NP_001087240, NP_00	.	protein_coding	ENSG0000019843	7296	12437
645	U2SURP	1.584	0.000	U2 snRNP associated SURP domain containing [Source:HGNC Symbol;Acc:HGNC:30855]	chr3:142720380-142779388	NM_001080415, NN	XM_005247249, XM_0	NP_001073884, NP_00	XP_005247305	protein_coding	ENSG0000016371	23350	30855
646	UACA	1.255	0.000	uveal autoantigen with coiled-coil domains and ankyrin repeats [Source:HGNC Symbol;A	chr15:70946892-71055932	NM_001008224, NN	XM_011521754, XM_0	NP_001008225, NP_06	XP_005254586, XP_0115	protein_coding	ENSG0000013783	55075	15947

647	UAP1	1.290	0.000	UDP-N-acetylglucosamine pyrophosphorylase 1 [Source:HGNC Symbol;Acc:HGNC:12457	chr1:162531295-162569632	NM_001324116, NN	XM_011509915, XM_0	NP_001311042, NP_00	XP_005245519, XP_0052	protein_coding	ENSG0000011714	6675	12457
648	UBA1	-1.292	0.000	ubiquitin like modifier activating enzyme 1 [Source:HGNC Symbol;Acc:HGNC:12469]	chrX:47050259-47074527	NM_003334, NM_1	XM_017029781, XM_0	NP_003325, NP_69501	XP_005272706, XP_0052	protein_coding	ENSG0000013098	7317	12469
649	UBA6	1.472	0.000	ubiquitin like modifier activating enzyme 6 [Source:HGNC Symbol;Acc:HGNC:25581]	chr4:68478369-68566897	NM_018227	.	NP_060697	XP_016863848	protein_coding	ENSG0000003317	55236	25581
650	UBE2E1	-1.216	0.000	ubiquitin conjugating enzyme E2 E1 [Source:HGNC Symbol;Acc:HGNC:12477]	chr3:23847393-23932807	NM_001202476, NN	XM_005265431	NP_001189405, NP_00	XP_005265488	protein_coding	ENSG0000017014	7324	12477
651	UGP2	1.130	0.000	UDP-glucose pyrophosphorylase 2 [Source:HGNC Symbol;Acc:HGNC:12527]	chr2:64068073-64118696	NM_001001521, NN	XM_006712088, XM_0	NP_001001521, NP_00	XP_005264594, XP_0052	protein_coding	ENSG0000016976	7360	12527
652	UHMK1	1.327	0.000	U2AF homology motif (UHM) kinase 1 [Source:HGNC Symbol;Acc:HGNC:19683]	chr1:162467040-162499419	NM_001184763, NN	.	NP_001171692, NP_65	.	protein_coding	ENSG0000015233	127933	19683
653	UNC13A	-2.030	0.000	unc-13 homolog A [Source:HGNC Symbol;Acc:HGNC:23150]	chr19:17712136-17799174	NM_001080421	XM_017026502, XM_0	NP_001073890	XP_011526112, XP_0115	protein_coding	ENSG0000013047	23025	23150
654	UNC5B	-1.742	0.000	unc-5 netrin receptor B [Source:HGNC Symbol;Acc:HGNC:12568]	chr10:72972326-73062621	NM_001244889, NN	XM_017015834, XM_0	NP_001231818, NP_73	XP_011537755, XP_0168	protein_coding	ENSG0000010773	219699	12568
655	UQCRC1	-1.122	0.000	ubiquinol-cytochrome c reductase core protein I [Source:HGNC Symbol;Acc:HGNC:1258	chr3:48636435-48647470	NM_003365	.	NP_003356	.	protein_coding	ENSG0000001025	7384	12585
656	UROC1	-1.792	0.000	urocanate hydratase 1 [Source:HGNC Symbol;Acc:HGNC:26444]	chr3:126200123-126236616	NM_001165974, NN	.	NP_001159446, NP_65	.	protein_coding	ENSG0000015965	131669	26444
657	USH1C	-2.030	0.000	USH1 protein network component harmonin [Source:HGNC Symbol;Acc:HGNC:12597]	chr11:17515441-17565963	NM_001297764, NN	XM_011519832, XM_0	NP_001284693, NP_00	XP_011518134, XP_0168	protein_coding	ENSG0000000661	10083	12597
658	USO1	1.277	0.000	USO1 vesicle transport factor [Source:HGNC Symbol;Acc:HGNC:30904]	chr4:76649776-76735439	NM_001290049, NN	XM_006714397, XM_0	NP_001276978, NP_00	XP_006714459, XP_0067	protein_coding	ENSG0000013876	8615	30904
659	USP11	-1.049	0.000	ubiquitin specific peptidase 11 [Source:HGNC Symbol;Acc:HGNC:12609]	chrX:47092088-47107727	NM_004651	XM_011543988, XM_0	NP_004642	XP_005272731, XP_0115	protein_coding	ENSG0000010222	8237	12609
660	USP47	1.487	0.000	ubiquitin specific peptidase 47 [Source:HGNC Symbol;Acc:HGNC:20076]	chr11:11862969-11980870	NM_001282659, NN	XM_005252997	NP_001269588, NP_06	XP_005253054, XP_0168	protein_coding	ENSG0000017024	55031	20076
661	USP53	1.616	0.000	ubiquitin specific peptidase 53 [Source:HGNC Symbol;Acc:HGNC:29255]	chr4:120133790-120216672	NM_019050	XM_017008315, XM_0	NP_061923	XP_005263130, XP_0052	protein_coding	ENSG0000014539	54532	29255
662	UTP20	1.272	0.000	UTP20, small subunit processome component [Source:HGNC Symbol;Acc:HGNC:17897]	chr12:101673886-101780394	NM_014503	XM_006719343	NP_055318	.	protein_coding	ENSG0000012080	27340	17897
663	UXT	-1.203	0.000	ubiquitously expressed prefoldin like chaperone [Source:HGNC Symbol;Acc:HGNC:1264	chrX:47511196-47518560	NM_004182, NM_1	.	NP_004173, NP_70558	.	protein_coding	ENSG0000012675	8409	12641
664	VASH1	-1.253	0.000	vasohibin 1 [Source:HGNC Symbol;Acc:HGNC:19964]	chr14:77228531-77249358	NM_014909	XM_011536556	NP_055724	.	protein_coding	ENSG0000007124	22846	19964
665	VAT1L	-3.725	0.000	vesicle amine transport 1 like [Source:HGNC Symbol;Acc:HGNC:29315]	chr16:77822426-78014004	NM_020927	.	NP_065978	.	protein_coding	ENSG0000017172	57687	29315
666	VAV1	-2.166	0.000	vav guanine nucleotide exchange factor 1 [Source:HGNC Symbol;Acc:HGNC:12657]	chr19:6772724-6857377	NM_001258206, NN	XM_005259642	NP_001245135, NP_00	XP_005259699	protein_coding	ENSG0000014196	7409	12657
667	VILL	-3.721	0.000	villin like [Source:HGNC Symbol;Acc:HGNC:30906]	chr3:38029549-38048679	NM_015873	XM_011533770, XM_0	NP_056957	XP_005265248, XP_0052	protein_coding	ENSG0000013605	50853	30906
668	VLDLR	1.627	0.000	very low density lipoprotein receptor [Source:HGNC Symbol;Acc:HGNC:12698]	chr9:2621833-2660053	NM_001018056, NN	XM_011518029	NP_001018066, NP_00	.	protein_coding	ENSG0000014785	7436	12698
669	VPS35	1.142	0.000	VPS35, retromer complex component [Source:HGNC Symbol;Acc:HGNC:13487]	chr16:46690043-46723104	NM_018206	XM_011523227, XM_0	NP_060676	XP_005256102, XP_0115	protein_coding	ENSG0000006932	55737	13487
670	VPS50	2.004	0.000	VPS50, EARP/GARPII complex subunit [Source:HGNC Symbol;Acc:HGNC:25956]	chr7:92861652-92990433	NM_001257998, NN	XM_011516395	NP_001244927, NP_06	XP_011514697	protein_coding	ENSG0000000476	55610	25956
671	VWA1	-1.233	0.000	von Willebrand factor A domain containing 1 [Source:HGNC Symbol;Acc:HGNC:30910]	chr1:1370902-1378262	NM_022834, NM_1	.	NP_073745, NP_95457	.	protein_coding	ENSG0000017940	64856	30910
672	WDHD1	1.671	0.000	WD repeat and HMG-box DNA binding protein 1 [Source:HGNC Symbol;Acc:HGNC:2317	chr14:55405667-55493823	NM_001008396, NN	XM_006720012	NP_001008397, NP_00	XP_006720075, XP_0168	protein_coding	ENSG0000019855	11169	23170
673	WDR13	-1.720	0.000	WD repeat domain 13 [Source:HGNC Symbol;Acc:HGNC:14352]	chrX:48455865-48467255	NM_001166426, NN	XM_011543947, XM_0	NP_001159898, NP_06	XP_006724602, XP_0067	protein_coding	ENSG0000010194	64743	14352
674	WDR35	1.338	0.000	WD repeat domain 35 [Source:HGNC Symbol;Acc:HGNC:29250]	chr2:20110020-20189892	NM_001006657, NN	.	NP_001006658, NP_06	.	protein_coding	ENSG0000011896	57539	29250
675	WDR36	1.402	0.000	WD repeat domain 36 [Source:HGNC Symbol;Acc:HGNC:30696]	chr5:110427413-110466200	NM_139281	XM_011543163	NP_644810	.	protein_coding	ENSG0000013498	134430	30696
676	WDR45	-1.995	0.000	WD repeat domain 45 [Source:HGNC Symbol;Acc:HGNC:28912]	chrX:48928812-48958059	NM_001029896, NN	.	NP_001025067, NP_00	.	protein_coding	ENSG0000019699	11152	28912
677	WDR75	1.638	0.000	WD repeat domain 75 [Source:HGNC Symbol;Acc:HGNC:25725]	chr2:190306158-190340291	NM_001303096, NN	.	NP_001290025, NP_11	.	protein_coding	ENSG0000011536	84128	25725
678	WDR89	1.623	0.000	WD repeat domain 89 [Source:HGNC Symbol;Acc:HGNC:20489]	chr14:64063756-64108579	NM_001008726, NN	XM_011536385, XM_0	NP_001008726, NP_00	XP_011534685, XP_0115	protein_coding	ENSG0000014000	112840	20489
679	WFDC3	-2.353	0.000	WAP four-disulfide core domain 3 [Source:HGNC Symbol;Acc:HGNC:15957]	chr20:44402846-44420547	NM_080614	XM_011528557, XM_0	NP_542181	XP_011526855	protein_coding	ENSG0000012411	140686	15957
680	WHSC1L1	1.328	0.000	Wolf-Hirschhorn syndrome candidate 1-like 1 [Source:HGNC Symbol;Acc:HGNC:12767]	chr8:38127214-38239790	NM_017778, NM_0	XM_011544563, XM_0	NP_060248, NP_07544	XP_005273604, XP_0052	protein_coding	ENSG0000014754	54904	12767
681	WNT10A	-1.049	0.000	Wnt family member 10A [Source:HGNC Symbol;Acc:HGNC:13829]	chr2:219745084-219758653	NM_025216	.	NP_079492	XP_011510231	protein_coding	ENSG0000013592	80326	13829
682	WNT7A	-1.443	0.000	Wnt family member 7A [Source:HGNC Symbol;Acc:HGNC:12786]	chr3:13857754-13921618	NM_004625	.	NP_004616	.	protein_coding	ENSG0000015476	7476	12786
683	WRN	1.828	0.000	Werner syndrome RecQ like helicase [Source:HGNC Symbol;Acc:HGNC:12791]	chr8:30891316-31031285	NM_000553	XM_011544639	NP_000544	XP_011542941	protein_coding	ENSG0000016539	7486	12791
684	WWC2	1.376	0.000	WW and C2 domain containing 2 [Source:HGNC Symbol;Acc:HGNC:24148]	chr4:184020445-184241930	NM_024949	XM_011532269	NP_079225	XP_011530571	protein_coding	ENSG0000015171	80014	24148
685	WWP1	1.300	0.000	WW domain containing E3 ubiquitin protein ligase 1 [Source:HGNC Symbol;Acc:HGNC:1	chr8:87354966-87480732	NM_007013	XM_011516802, XM_0	NP_008944	XP_005250817, XP_0052	protein_coding	ENSG0000012312	11059	17004
686	XPO1	1.159	0.000	exportin 1 [Source:HGNC Symbol;Acc:HGNC:12825]	chr2:61704983-61765418	NM_003400	XM_011533099, XM_0	NP_003391	XP_005264601, XP_0052	protein_coding	ENSG0000008289	7514	12825
687	XPO7	1.270	0.000	exportin 7 [Source:HGNC Symbol;Acc:HGNC:14108]	chr8:21777181-21864096	NM_015024	XM_017013246	NP_055839	XP_016868733, XP_0168	protein_coding	ENSG0000013022	23039	14108
688	XPOT	1.384	0.000	exportin for tRNA [Source:HGNC Symbol;Acc:HGNC:12826]	chr12:64798129-64844907	NM_007235	XM_017018748	NP_009166	XP_016874237	protein_coding	ENSG0000018457	11260	12826
689	YOD1	1.570	0.000	YOD1 deubiquitinase [Source:HGNC Symbol;Acc:HGNC:25035]	chr1:207217193-207226325	NM_001276320, NN	XM_011509725	NP_001263249, NP_06	XP_011508027	protein_coding	ENSG0000018066	55432	25035
690	YPEL3	-1.248	0.000	yippee like 3 [Source:HGNC Symbol;Acc:HGNC:18327]	chr16:30103634-30108236	NM_001145524, NN	.	NP_001138996, NP_11	.	protein_coding	ENSG0000009023	83719	18327
691	ZAP70	-6.464	0.000	zeta chain of T cell receptor associated protein kinase 70 [Source:HGNC Symbol;Acc:HG	chr2:98330022-98356323	NM_001079, NM_2	XM_011511783, XM_0	NP_001070, NP_99740	.	protein_coding	ENSG0000011508	7535	12858
692	ZC3H11A	1.183	0.000	zinc finger CCCH-type containing 11A [Source:HGNC Symbol;Acc:HGNC:29093]	chr1:203764798-203823252	NM_001319239, NN	XM_011510204, XM_0	NP_001306167, NP_00	XP_005245700, XP_0052	protein_coding	ENSG0000005867	9877	29093
693	ZC3H6	1.735	0.000	zinc finger CCCH-type containing 6 [Source:HGNC Symbol;Acc:HGNC:24762]	chr2:113033170-113097640	NM_198581	XM_011511156, XM_0	NP_940983	XP_006712582	protein_coding	ENSG0000018817	376940	24762
694	ZDBF2	1.657	0.000	zinc finger DBF-type containing 2 [Source:HGNC Symbol;Acc:HGNC:29313]	chr2:207139386-207179152	NM_001285549, NN	XM_011511537, XM_0	NP_001272478, NP_06	XP_005246768, XP_0052	protein_coding	ENSG0000020418	57683	29313
695	ZDHHC2	1.503	0.000	zinc finger DHHC-type containing 2 [Source:HGNC Symbol;Acc:HGNC:18469]	chr8:17013537-17082308	NM_016353	XM_011544548, XM_0	NP_057437	XP_011542846, XP_0115	protein_coding	ENSG0000010421	51201	18469
696	ZMYM2	1.365	0.000	zinc finger MYM-type containing 2 [Source:HGNC Symbol;Acc:HGNC:12989]	chr13:20532809-20665968	NM_001190964, NN	XM_011535221, XM_0	NP_001177893, NP_00	XP_005266574, XP_0052	protein_coding	ENSG0000012174	7750	12989
697	ZMYM3	-1.197	0.000	zinc finger MYM-type containing 3 [Source:HGNC Symbol;Acc:HGNC:13054]	chrX:70459473-70474996	NM_001171162, NN	XM_011531062, XM_0	NP_001164633, NP_00	XP_005262366, XP_0052	protein_coding	ENSG0000014713	9203	13054
698	ZNF107	2.484	0.000	zinc finger protein 107 [Source:HGNC Symbol;Acc:HGNC:12887]	chr7:64126460-64171960	NM_001013746, NN	XM_017012286, XM_0	NP_001013768, NP_00	XP_016867772, XP_0168	protein_coding	ENSG0000019624	51427	12887
699	ZNF12	1.583	0.000	zinc finger protein 12 [Source:HGNC Symbol;Acc:HGNC:12902]	chr7:6728063-6746554	NM_006956, NM_0	.	NP_008887, NP_05734	.	protein_coding	ENSG0000016463	7559	12902
700	ZNF146	1.730	0.000	zinc finger protein 146 [Source:HGNC Symbol;Acc:HGNC:12931]	chr19:36672961-36737159	NM_001099638, NN	XM_017027245, XM_0	NP_001093108, NP_00	XP_005259271, XP_0168	protein_coding	ENSG0000016763	7705	12931

701	ZNF280C	1.793	0.000	zinc finger protein 280C [Source:HGNC Symbol;Acc:HGNC:25955]	chrX:129336684-129402873	NM_017666	XM_011531362, XM_0	NP_060136	XP_006724828, XP_0115	protein_coding	ENSG0000005627	55609	25955
702	ZNF358	-1.268	0.000	zinc finger protein 358 [Source:HGNC Symbol;Acc:HGNC:16838]	chr19:7562444-7585912	NM_018083	XM_017026296, XM_0	NP_060553	XP_005272517, XP_0115	protein_coding	ENSG0000019881	140467	16838
703	ZNF449	2.017	0.000	zinc finger protein 449 [Source:HGNC Symbol;Acc:HGNC:21039]	chrX:134478720-134497077	NM_152695	XM_011531312	NP_689908	XP_011529614	protein_coding	ENSG0000017327	203523	21039
704	ZNF451	1.511	0.000	zinc finger protein 451 [Source:HGNC Symbol;Acc:HGNC:21091]	chr6:56954807-57035105	NM_001031623, NM	XM_011514460, XM_0	NP_001026794, NP_00	XP_005249051, XP_0115	protein_coding	ENSG0000011220	26036	21091
705	ZNF518A	1.770	0.000	zinc finger protein 518A [Source:HGNC Symbol;Acc:HGNC:29009]	chr10:97889471-97923508	NM_001278524, NM	XM_011540421, XM_0	NP_001265453, NP_00	XP_011538707, XP_0115	protein_coding	ENSG0000017785	9849	29009
706	ZNF581	-1.221	0.000	zinc finger protein 581 [Source:HGNC Symbol;Acc:HGNC:25017]	chr19:56146824-56156988	NM_016535	XM_006723240	NP_057619	XP_006723303, XP_0168	protein_coding	ENSG0000017142	51545	25017
707	ZNF800	1.737	0.000	zinc finger protein 800 [Source:HGNC Symbol;Acc:HGNC:27267]	chr7:127010096-127032748	NM_176814	XM_005250181, XM_0	NP_789784	XP_005250238, XP_0067	protein_coding	ENSG0000004840	168850	27267
708	ABL2	1.390	0.000	ABL proto-oncogene 2, non-receptor tyrosine kinase [Source:HGNC Symbol;Acc:HGNC:7	chr1:179068461-179198819	NM_001136000, NM	XM_005245088	NP_001129472, NP_00	XP_005245145, XP_0168	protein_coding	ENSG0000014332	27	77
709	ACAD9	-1.078	0.000	acyl-CoA dehydrogenase family member 9 [Source:HGNC Symbol;Acc:HGNC:21497]	chr3:128598453-128712958	NM_014049	XM_011512742	NP_054768	.	protein_coding	ENSG0000017764	28976	21497
710	ADD3	1.293	0.000	adducin 3 [Source:HGNC Symbol;Acc:HGNC:245]	chr10:111765561-111895323	NM_001121, NM_0	XM_011539289, XM_0	NP_001112, NP_00130	XP_005269586, XP_0052	protein_coding	ENSG0000014870	120	245
711	ALPP	-2.035	0.000	alkaline phosphatase, placental [Source:HGNC Symbol;Acc:HGNC:439]	chr2:233243243-233247599	NM_001632	.	NP_001623	.	protein_coding	ENSG0000016328	250	439
712	AMER1	-1.232	0.000	APC membrane recruitment protein 1 [Source:HGNC Symbol;Acc:HGNC:26837]	chrX:63404996-63425624	NM_152424	XM_011530858	NP_689637	XP_011529160	protein_coding	ENSG0000018467	139285	26837
713	AMMECR1	1.262	0.000	Alport syndrome, mental retardation, midface hypoplasia and elliptocytosis chromosom	chrX:109437413-109699562	NM_001025580, NM	.	NP_001020751, NP_00	.	protein_coding	ENSG0000010193	9949	467
714	ANKRD50	1.709	0.000	ankyrin repeat domain 50 [Source:HGNC Symbol;Acc:HGNC:29223]	chr4:125585206-125633887	NM_001167882, NM	XM_011532146	NP_001161354, NP_06	XP_011530448, XP_0168	protein_coding	ENSG0000015145	57182	29223
715	ARHGAP18	1.596	0.000	Rho GTPase activating protein 18 [Source:HGNC Symbol;Acc:HGNC:21035]	chr6:129897276-130031370	NM_033515	XM_005267213, XM_0	NP_277050	XP_005267269, XP_0052	protein_coding	ENSG0000014637	93663	21035
716	ARHGAP42	1.745	0.000	Rho GTPase activating protein 42 [Source:HGNC Symbol;Acc:HGNC:26545]	chr11:100558383-100862668	NM_152432	XM_011542617, XM_0	NP_689645	XP_011540917, XP_0115	protein_coding	ENSG0000016589	143872	26545
717	ARID4A	1.665	0.000	AT-rich interaction domain 4A [Source:HGNC Symbol;Acc:HGNC:9885]	chr14:58765102-58840605	NM_002892, NM_0	XM_017021562, XM_0	NP_002883, NP_07537	XP_005268021, XP_0052	protein_coding	ENSG0000003221	5926	9885
718	ARL5B	1.432	0.000	ADP ribosylation factor like GTPase 5B [Source:HGNC Symbol;Acc:HGNC:23052]	chr10:18948333-18970568	NM_178815	XM_005252401, XM_0	NP_848930	XP_005252457, XP_0052	protein_coding	ENSG0000016599	221079	23052
719	ASPH	1.087	0.000	aspartate beta-hydroxylase [Source:HGNC Symbol;Acc:HGNC:757]	chr8:62200513-62627199	NM_001164751, NM	XM_017013439, XM_0	NP_001158223, NP_00	XP_005251292, XP_0052	protein_coding	ENSG0000019836	444	757
720	B4GALNT4	-1.078	0.000	beta-1,4-N-acetyl-galactosaminyltransferase 4 [Source:HGNC Symbol;Acc:HGNC:26315]	chr11:369803-382116	NM_178537	XM_005252892	NP_848632	XP_016873143	protein_coding	ENSG0000018227	338707	26315
721	BACH1	1.373	0.000	BTB domain and CNC homolog 1 [Source:HGNC Symbol;Acc:HGNC:935]	chr21:30671191-30718469	NM_001186, NM_2	.	NP_001177, NP_99674	.	protein_coding	ENSG0000015627	100379661	935
722	BIN1	-1.205	0.000	bridging integrator 1 [Source:HGNC Symbol;Acc:HGNC:1052]	chr2:127805602-127864931	NM_004305, NM_1	XM_011510975, XM_0	NP_001307561, NP_00	XP_005263699, XP_0052	protein_coding	ENSG0000013671	274	1052
723	BZW1	1.214	0.000	basic leucine zipper and W2 domains 1 [Source:HGNC Symbol;Acc:HGNC:18380]	chr2:201676268-201692061	NM_001207067, NM	XM_011512216, XM_0	NP_001193996, NP_00	XP_011510517, XP_0115	protein_coding	ENSG0000008215	9689	18380
724	C16orf54	-3.041	0.000	chromosome 16 open reading frame 54 [Source:HGNC Symbol;Acc:HGNC:26649]	chr16:29753783-29757327	NM_175900	XM_011508594	NP_787096	XP_011506896	protein_coding	ENSG0000018590	105369277	26649
725	C9orf72	1.904	0.000	chromosome 9 open reading frame 72 [Source:HGNC Symbol;Acc:HGNC:28337]	chr9:27546542-27573864	NM_001256054, NM	.	NP_001242983, NP_06	.	protein_coding	ENSG0000014789	203228	28337
726	CD4	-2.633	0.000	CD4 molecule [Source:HGNC Symbol;Acc:HGNC:1678]	chr12:6898637-6929974	NM_000616	.	NP_000607	.	protein_coding	ENSG0000001061	920	1678
727	CD6	-1.794	0.000	CD6 molecule [Source:HGNC Symbol;Acc:HGNC:1691]	chr11:60739151-60787849	NM_001254750, NM	XM_011545362, XM_0	NP_001241679, NP_00	XP_006718801, XP_0067	protein_coding	ENSG0000001372	923	1691
728	CD74	-0.994	0.000	CD74 molecule [Source:HGNC Symbol;Acc:HGNC:1697]	chr5:149781199-149792492	NM_001025158, NM	.	NP_001020329, NP_00	XP_016865578	protein_coding	ENSG0000001958	972	1697
729	CDC42BPA	1.215	0.000	CDC42 binding protein kinase alpha [Source:HGNC Symbol;Acc:HGNC:1737]	chr1:227177565-227506175	NM_003607, NM_0	XM_011544309, XM_0	NP_003598, NP_05564	XP_005273374, XP_0052	protein_coding	ENSG0000014377	8476	1737
730	CDKN1A	-0.953	0.000	cyclin dependent kinase inhibitor 1A [Source:HGNC Symbol;Acc:HGNC:1784]	chr6:36644236-36655116	NM_000389, NM_0	.	NP_000380, NP_00120	.	protein_coding	ENSG0000012476	1026	1784
731	CHST7	-1.626	0.000	carbohydrate sulfotransferase 7 [Source:HGNC Symbol;Acc:HGNC:13817]	chrX:46433218-46457843	NM_019886	.	NP_063939	.	protein_coding	ENSG0000014711	56548	13817
732	CIPC	1.289	0.000	CLOCK interacting pacemaker [Source:HGNC Symbol;Acc:HGNC:20365]	chr14:77564439-77583630	NM_033426	.	NP_219494	.	protein_coding	ENSG0000019889	85457	20365
733	CLMN	1.187	0.000	calmin [Source:HGNC Symbol;Acc:HGNC:19972]	chr14:95648276-95786243	NM_024734	XM_011537158	NP_079010	XP_011535460, XP_0115	protein_coding	ENSG0000016595	79789	19972
734	COL16A1	-1.218	0.000	collagen type XVI alpha 1 chain [Source:HGNC Symbol;Acc:HGNC:2193]	chr1:32117847-32169920	NM_001856	XM_011540730, XM_0	NP_001847	XP_005270538, XP_0115	protein_coding	ENSG0000008463	1307	2193
735	CPD	1.242	0.000	carboxypeptidase D [Source:HGNC Symbol;Acc:HGNC:2301]	chr17:28705922-28797007	NM_001199775, NM	.	NP_001186704, NP_00	.	protein_coding	ENSG0000010858	1362	2301
736	CYBRD1	0.948	0.000	cytochrome b reductase 1 [Source:HGNC Symbol;Acc:HGNC:20797]	chr2:172378756-172414643	NM_001127383, NM	.	NP_001120855, NP_00	.	protein_coding	ENSG0000007196	79901	20797
737	DDX18	1.156	0.000	DEAD-box helicase 18 [Source:HGNC Symbol;Acc:HGNC:2741]	chr2:118572225-118589955	NM_006773	.	NP_006764	.	protein_coding	ENSG0000008820	8886	2741
738	DHX36	1.296	0.000	DEAH-box helicase 36 [Source:HGNC Symbol;Acc:HGNC:14410]	chr3:153990334-154042286	NM_001114397, NM	.	NP_001107869, NP_06	.	protein_coding	ENSG0000017495	170506	14410
739	EFNA5	1.791	0.000	ephrin A5 [Source:HGNC Symbol;Acc:HGNC:3225]	chr5:106712589-107006596	NM_001962	XM_011543251, XM_0	NP_001953	XP_006714628, XP_0115	protein_coding	ENSG0000018434	1946	3225
740	EHF	-1.662	0.000	ETS homologous factor [Source:HGNC Symbol;Acc:HGNC:3246]	chr11:34642657-34682604	NM_001206615, NM	XM_011519985, XM_0	NP_001193544, NP_00	XP_005252914, XP_0052	protein_coding	ENSG0000013537	26298	3246
741	EIF3A	0.960	0.000	eukaryotic translation initiation factor 3 subunit A [Source:HGNC Symbol;Acc:HGNC:327	chr10:120789227-120840335	NM_003750	.	NP_003741	.	protein_coding	ENSG0000010758	8661	3271
742	ELL2	1.458	0.000	elongation factor for RNA polymerase II 2 [Source:HGNC Symbol;Acc:HGNC:17064]	chr5:95220801-95297775	NM_012081	XM_017009242, XM_0	NP_036213	XP_006714638, XP_0168	protein_coding	ENSG0000011898	22936	17064
743	EXO1	1.141	0.000	exonuclease 1 [Source:HGNC Symbol;Acc:HGNC:3511]	chr1:242011481-242053241	NM_001319224, NM	XM_011544324, XM_0	NP_001306153, NP_00	XP_006711903, XP_0115	protein_coding	ENSG0000017437	9156	3511
744	EXTL2	1.577	0.000	exostosin like glycosyltransferase 2 [Source:HGNC Symbol;Acc:HGNC:3516]	chr1:101337927-101361554	NM_001033025, NM	XM_017000651, XM_0	NP_001028197, NP_00	XP_005270678, XP_0115	protein_coding	ENSG0000016269	2135	3516
745	FAM208B	1.277	0.000	family with sequence similarity 208 member B [Source:HGNC Symbol;Acc:HGNC:23484]	chr10:5726800-5805703	NM_001321783, NM	XM_017016365, XM_0	NP_001308712, NP_00	XP_005252531, XP_0052	protein_coding	ENSG0000010802	54906	23484
746	FAS	1.415	0.000	Fas cell surface death receptor [Source:HGNC Symbol;Acc:HGNC:11920]	chr10:90750413-90775542	NM_000043, NM_1	XM_011539767, XM_0	NP_000034, NP_69061	XP_011538068, XP_0115	protein_coding	ENSG0000002610	355	11920
747	FRAT2	-1.326	0.000	frequently rearranged in advanced T-cell lymphomas 2 [Source:HGNC Symbol;Acc:HGN	chr10:99092253-99094466	NM_012083	.	NP_036215	.	protein_coding	ENSG0000018127	23401	16048
748	FUT10	1.543	0.000	fucosyltransferase 10 [Source:HGNC Symbol;Acc:HGNC:19234]	chr8:33228341-33330940	NM_032664	.	NP_116053	.	protein_coding	ENSG0000017272	84750	19234
749	GLB1	-1.093	0.000	galactosidase beta 1 [Source:HGNC Symbol;Acc:HGNC:4298]	chr3:33038099-33138722	NM_000404, NM_0	.	NP_000395, NP_00107	.	protein_coding	ENSG0000017026	2720	4298
750	GLCCI1	1.736	0.000	glucocorticoid induced 1 [Source:HGNC Symbol;Acc:HGNC:18713]	chr7:8008424-8128710	NM_138426	.	NP_612435	.	protein_coding	ENSG0000010641	113263	18713
751	GNAI1	1.639	0.000	G protein subunit alpha i1 [Source:HGNC Symbol;Acc:HGNC:4384]	chr7:79764103-79848718	NM_001256414, NM	.	NP_001243343, NP_00	.	protein_coding	ENSG0000012795	2770	4384
752	GPAA1	-1.113	0.000	glycosylphosphatidylinositol anchor attachment 1 [Source:HGNC Symbol;Acc:HGNC:444	chr8:145137512-145141119	NM_003801	.	NP_003792	.	protein_coding	ENSG0000019785	8733	4446
753	HIST3H2A	-1.166	0.000	histone cluster 3, H2a [Source:HGNC Symbol;Acc:HGNC:20507]	chr1:228644679-228645574	NM_033445	.	NP_254280	.	protein_coding	ENSG0000018121	92815	20507
754	HLA-A	-0.906	0.000	major histocompatibility complex, class I, A [Source:HGNC Symbol;Acc:HGNC:4931]	chr6:29909036-29913661	NM_002116	.	NP_002107	.	protein_coding	ENSG0000020650	3105	4931

755	HLA-C	-0.882	0.000	major histocompatibility complex, class I, C [Source:HGNC Symbol;Acc:HGNC:4933]	chr6:31236525-31239863	NM_002117	.	NP_002108	.	protein_coding	ENSG0000020452	3107		4933
756	HOXD10	1.679	0.000	homeobox D10 [Source:HGNC Symbol;Acc:HGNC:5133]	chr2:176981306-176984670	NM_002148	.	NP_002139	.	protein_coding	ENSG0000012871	3236		5133
757	HSP90B1	0.984	0.000	heat shock protein 90 beta family member 1 [Source:HGNC Symbol;Acc:HGNC:12028]	chr12:104324111-104341704	NM_003299	.	NP_003290	.	protein_coding	ENSG0000016659	100500842		12028
758	IFRD2	-1.053	0.000	interferon related developmental regulator 2 [Source:HGNC Symbol;Acc:HGNC:5457]	chr3:50316457-50336899	NM_006764	.	NP_006755	.	protein_coding	ENSG0000021470	7866		5457
759	IL17RE	-1.552	0.000	interleukin 17 receptor E [Source:HGNC Symbol;Acc:HGNC:18439]	chr3:9944302-9958086	NM_001193380, NN	XM_011533362, XM_0	NP_001180309, NP_70	XP_006713038, XP_0067	protein_coding	ENSG0000016370	132014		18439
760	IL23A	-1.115	0.000	interleukin 23 subunit alpha [Source:HGNC Symbol;Acc:HGNC:15488]	chr12:56732658-56734194	NM_016584	XM_011538477	NP_057668	XP_011536779	protein_coding	ENSG0000011094	51561		15488
761	ITFG1	1.327	0.000	integrin alpha FG-GAP repeat containing 1 [Source:HGNC Symbol;Acc:HGNC:30697]	chr16:47188297-47495185	NM_001305002, NN	.	NP_001291931, NP_11	.	protein_coding	ENSG0000012963	81533		30697
762	KPNB1	0.961	0.000	karyopherin subunit beta 1 [Source:HGNC Symbol;Acc:HGNC:6400]	chr17:45726841-45762871	NM_001276453, NN	.	NP_001263382, NP_00	.	protein_coding	ENSG0000010842	3837		6400
763	LAD1	-0.932	0.000	ladinin 1 [Source:HGNC Symbol;Acc:HGNC:6472]	chr1:201349965-201368736	NM_005558	.	NP_005549	.	protein_coding	ENSG0000015916	3898		6472
764	LAMP3	1.075	0.000	lysosomal associated membrane protein 3 [Source:HGNC Symbol;Acc:HGNC:14582]	chr3:182840000-182880699	NM_014398	.	NP_055213	XP_005247417	protein_coding	ENSG0000007808	27074		14582
765	LRRCC1	1.834	0.000	leucine rich repeat and coiled-coil centrosomal protein 1 [Source:HGNC Symbol;Acc:HGI	chr8:86019381-86058311	NM_033402	XM_005251320, XM_0	NP_208325	XP_005251375, XP_0052	protein_coding	ENSG0000013373	85444		29373
766	MAGED1	-1.048	0.000	MAGE family member D1 [Source:HGNC Symbol;Acc:HGNC:6813]	chrX:51546102-51645453	NM_001005332, NN	XM_017029978, XM_0	NP_001005332, NP_00	XP_011529137, XP_0168	protein_coding	ENSG0000017922	9500		6813
767	MAGI3	1.346	0.000	membrane associated guanylate kinase, WW and PDZ domain containing 3 [Source:HGN	chr1:113933370-114228545	NM_001142782, NN	XM_011541208, XM_0	NP_001136254, NP_69	XP_005270794, XP_0115	protein_coding	ENSG0000008102	260425		29647
768	MCM2	-0.965	0.000	minichromosome maintenance complex component 2 [Source:HGNC Symbol;Acc:HGNC	chr3:127317065-127341279	NM_004526	.	NP_004517	.	protein_coding	ENSG0000007311	4171		6944
769	MEFV	-1.425	0.000	Mediterranean fever [Source:HGNC Symbol;Acc:HGNC:6998]	chr16:3292027-3306627	NM_000243, NM_0	.	NP_000234, NP_00118	XP_016878725	protein_coding	ENSG0000010331	4210		6998
770	MGAT4B	-1.071	0.000	mannosyl (alpha-1,3-)-glycoprotein beta-1,4-N-acetylglucosaminyltransferase, isozyme	chr5:179224596-179233934	NM_014275, NM_0	.	NP_055090, NP_46345	XP_016864487	protein_coding	ENSG0000016101	11282		7048
771	MMP15	-1.098	0.000	matrix metallopeptidase 15 [Source:HGNC Symbol;Acc:HGNC:7161]	chr16:58059469-58080805	NM_002428	.	NP_002419	.	protein_coding	ENSG0000010299	4324		7161
772	MSH2	1.088	0.000	mutS homolog 2 [Source:HGNC Symbol;Acc:HGNC:7325]	chr2:47630107-47740373	NM_000251, NM_0	XM_011532867, XM_0	NP_000242, NP_00124	XP_005264389, XP_0115	protein_coding	ENSG0000009500	4436		7325
773	MSLN	-1.315	0.000	mesothelin [Source:HGNC Symbol;Acc:HGNC:7371]	chr16:810761-818865	NM_001177355, NN	XM_017022858, XM_0	NP_001170826, NP_00	XP_005255091, XP_0067	protein_coding	ENSG0000010285	10232		7371
774	MT3	-2.166	0.000	metallothionein 3 [Source:HGNC Symbol;Acc:HGNC:7408]	chr16:56622985-56625000	NM_005954	.	NP_005945	.	protein_coding	ENSG0000008725	4504		7408
775	NDC80	1.097	0.000	NDC80, kinetochore complex component [Source:HGNC Symbol;Acc:HGNC:16909]	chr18:2571509-2616634	NM_006101	.	NP_006092	.	protein_coding	ENSG0000008098	10403		16909
776	NRIP1	1.514	0.000	nuclear receptor interacting protein 1 [Source:HGNC Symbol;Acc:HGNC:8001]	chr21:16333555-16437321	NM_003489	XM_017028476, XM_0	NP_003480	XP_005261120, XP_0052	protein_coding	ENSG0000018053	8204		8001
777	OSBPL8	1.228	0.000	oxysterol binding protein like 8 [Source:HGNC Symbol;Acc:HGNC:16396]	chr12:76745576-76953589	NM_001003712, NN	XM_017018769, XM_0	NP_001003712, NP_00	XP_005268678, XP_0052	protein_coding	ENSG0000009103	114882		16396
778	OSBPL9	1.167	0.000	oxysterol binding protein like 9 [Source:HGNC Symbol;Acc:HGNC:16386]	chr1:52042850-52254141	NM_024586, NM_1	XM_017000220, XM_0	NP_078862, NP_68370	XP_006710381, XP_0067	protein_coding	ENSG0000011785	114883		16386
779	PHF3	1.215	0.000	PHD finger protein 3 [Source:HGNC Symbol;Acc:HGNC:8921]	chr6:64345740-64425377	NM_001290259, NN	XM_011535648, XM_0	NP_001277188, NP_00	XP_005248758, XP_0052	protein_coding	ENSG0000011848	23469		8921
780	PLEKHA2	1.183	0.000	pleckstrin homology domain containing A2 [Source:HGNC Symbol;Acc:HGNC:14336]	chr8:38758752-38831428	NM_021623	XM_017013720, XM_0	NP_067636	XP_011542907, XP_0115	protein_coding	ENSG0000016949	59339		14336
781	POT1	1.493	0.000	protection of telomeres 1 [Source:HGNC Symbol;Acc:HGNC:17284]	chr7:124462439-124570037	NM_001042594, NN	XM_017011942, XM_0	NP_001036059, NP_05	XP_006715980, XP_0115	protein_coding	ENSG0000012851	25913		17284
782	PPIG	1.290	0.000	peptidylprolyl isomerase G [Source:HGNC Symbol;Acc:HGNC:14650]	chr2:170440849-170497916	NM_004792	XM_005246967, XM_0	NP_004783	XP_005247023, XP_0052	protein_coding	ENSG0000013839	9360		14650
783	PTGES	-1.308	0.000	prostaglandin E synthase [Source:HGNC Symbol;Acc:HGNC:9599]	chr9:132500609-132515326	NM_004878	.	NP_004869	.	protein_coding	ENSG0000014834	9536		9599
784	PURB	1.074	0.000	purine rich element binding protein B [Source:HGNC Symbol;Acc:HGNC:9702]	chr7:44915891-44924960	NM_033224	.	NP_150093	.	protein_coding	ENSG0000014667	100616393		9702
785	RAB6B	-1.802	0.000	RAB6B, member RAS oncogene family [Source:HGNC Symbol;Acc:HGNC:14902]	chr3:133543082-133614660	NM_016577	XM_011512893	NP_057661	XP_011511195	protein_coding	ENSG0000015491	51560		14902
786	RASGRF1	-2.941	0.000	Ras protein specific guanine nucleotide releasing factor 1 [Source:HGNC Symbol;Acc:HG	chr15:79252288-79383115	NM_001145648, NN	XM_017022456, XM_0	NP_001139120, NP_00	XP_011520168, XP_0115	protein_coding	ENSG0000005833	5923		9875
787	RFWD3	1.065	0.000	ring finger and WD repeat domain 3 [Source:HGNC Symbol;Acc:HGNC:25539]	chr16:74655291-74700779	NM_018124	XM_017023391, XM_0	NP_060594	XP_005256078, XP_0052	protein_coding	ENSG0000016841	55159		25539
788	RHOA	-0.895	0.000	ras homolog family member A [Source:HGNC Symbol;Acc:HGNC:667]	chr3:49396577-49449635	NM_001664	XM_011533695	NP_001300870, NP_00	XP_011531997	protein_coding	ENSG0000006756	387		667
789	RHOB	-0.891	0.000	ras homolog family member B [Source:HGNC Symbol;Acc:HGNC:668]	chr2:20646834-20649206	NM_004040	.	NP_004031	.	protein_coding	ENSG0000014387	388		668
790	RSBN1L	1.154	0.000	round spermatid basic protein 1 like [Source:HGNC Symbol;Acc:HGNC:24765]	chr7:77325759-77412339	NM_198467	.	NP_940869	.	protein_coding	ENSG0000018725	222194		24765
791	SBDS	0.997	0.000	SBDS ribosome assembly guanine nucleotide exchange factor [Source:HGNC Symbol;Acc	chr7:66452663-66460588	NM_016038	.	NP_057122	.	protein_coding	ENSG0000012652	51119		19440
792	SEPT11	1.094	0.000	septin 11 [Source:HGNC Symbol;Acc:HGNC:25589]	chr4:77870855-77961537	NM_001306147, NN	XM_017008400, XM_0	NP_001293076, NP_06	XP_005263177, XP_0067	protein_coding	ENSG0000013875	55752		25589
793	SERTAD4	2.186	0.000	SERTA domain containing 4 [Source:HGNC Symbol;Acc:HGNC:25236]	chr1:210406143-210419600	NM_019605	.	NP_062551	.	protein_coding	ENSG0000008249	56256		25236
794	SLC38A9	1.379	0.000	solute carrier family 38 member 9 [Source:HGNC Symbol;Acc:HGNC:26907]	chr5:54921672-55008554	NM_001258286, NN	XM_011543183, XM_0	NP_001245215, NP_00	XP_006714600, XP_0067	protein_coding	ENSG0000017705	153129		26907
795	SLC6A6	-1.160	0.000	solute carrier family 6 member 6 [Source:HGNC Symbol;Acc:HGNC:11052]	chr3:14444075-14597582	NM_001134367, NN	XM_011534031, XM_0	NP_001127839, NP_00	XP_006713370, XP_0115	protein_coding	ENSG0000013138	6533		11052
796	SP3	1.086	0.000	Sp3 transcription factor [Source:HGNC Symbol;Acc:HGNC:11208]	chr2:174771186-174830430	NM_001017371, NN	.	NP_001017371, NP_00	.	protein_coding	ENSG0000017284	6670		11208
797	SUMF1	-1.231	0.000	sulfatase modifying factor 1 [Source:HGNC Symbol;Acc:HGNC:20376]	chr3:4402829-4508965	NM_001164674, NN	.	NP_001158146, NP_00	XP_016861743, XP_0168	protein_coding	ENSG0000014445	285362		20376
798	SYP	-1.873	0.000	synaptophysin [Source:HGNC Symbol;Acc:HGNC:11506]	chrX:49044256-49056718	NM_003179	XM_011543950	NP_003170	.	protein_coding	ENSG0000010200	6855		11506
799	THEM6	-1.001	0.000	thioesterase superfamily member 6 [Source:HGNC Symbol;Acc:HGNC:29656]	chr8:143808620-143818345	NM_016647	XM_005250955	NP_057731	XP_005251012	protein_coding	ENSG0000013019	51337		29656
800	THNSL1	1.785	0.000	threonine synthase like 1 [Source:HGNC Symbol;Acc:HGNC:26160]	chr10:25270907-25351208	NM_024838	XM_017016665, XM_0	NP_079114	XP_005252654, XP_0168	protein_coding	ENSG0000018587	79896		26160
801	THUMPD1	1.283	0.000	THUMP domain containing 1 [Source:HGNC Symbol;Acc:HGNC:23807]	chr16:20744985-20753406	NM_001304550, NN	.	NP_001291479, NP_06	XP_016878922	protein_coding	ENSG0000006665	55623		23807
802	TLE4	1.270	0.000	transducin like enhancer of split 4 [Source:HGNC Symbol;Acc:HGNC:11840]	chr9:82186687-82341797	NM_001282748, NN	XM_011518969, XM_0	NP_001269677, NP_00	XP_005252234, XP_0067	protein_coding	ENSG0000010682	7091		11840
803	TMEM209	1.357	0.000	transmembrane protein 209 [Source:HGNC Symbol;Acc:HGNC:21898]	chr7:129804554-129845338	NM_001301163, NN	XM_011516649, XM_0	NP_001288092, NP_11	XP_005250712, XP_0115	protein_coding	ENSG0000014684	84928		21898
804	TMSB4X	-0.899	0.000	thymosin beta 4, X-linked [Source:HGNC Symbol;Acc:HGNC:11881]	chrX:12993226-12995346	NM_021109	.	NP_066932	.	protein_coding	ENSG0000020554	7114		11881
805	TPGS1	-1.506	0.000	tubulin polyglutamylase complex subunit 1 [Source:HGNC Symbol;Acc:HGNC:25058]	chr19:507496-519654	NM_033513	.	NP_277048	.	protein_coding	ENSG0000014193	91978		25058
806	TRIM5	1.602	0.000	tripartite motif containing 5 [Source:HGNC Symbol;Acc:HGNC:16276]	chr11:5684786-5893588	NM_033034, NM_0	XM_011520426, XM_0	NP_149023, NP_14908	XP_005253240, XP_0052	protein_coding	ENSG0000013225	85363		16276
807	TRIP12	1.034	0.000	thyroid hormone receptor interactor 12 [Source:HGNC Symbol;Acc:HGNC:12306]	chr2:230628553-230786725	NM_001284214, NN	XM_017005293, XM_0	NP_001271143, NP_00	XP_005247011, XP_0052	protein_coding	ENSG0000015382	9320		12306
808	TSTA3	-1.062	0.000	tissue specific transplantation antigen P35B [Source:HGNC Symbol;Acc:HGNC:12390]	chr8:144694787-144699736	NM_001317783, NN	XM_011517269, XM_0	NP_001304712, NP_00	XP_005251108, XP_0115	protein_coding	ENSG0000010452	7264		12390

809	UFL1	1.461	0.000	UFM1 specific ligase 1 [Source:HGNC Symbol;Acc:HGNC:23039]	chr6:96969683-97003152	NM_015323	.	NP_056138	.	protein_coding	ENSG0000001412	23376		23039
810	UPF2	1.301	0.000	UPF2 regulator of nonsense transcripts homolog (yeast) [Source:HGNC Symbol;Acc:HGN	chr10:11962020-12085169	NM_015542, NM_0	XM_011519448, XM_0	NP_056357, NP_54216	.	protein_coding	ENSG0000015146	26019		17854
811	USP25	1.625	0.000	ubiquitin specific peptidase 25 [Source:HGNC Symbol;Acc:HGNC:12624]	chr21:17102343-17252388	NM_001283041, NN	XM_017028321, XM_0	NP_001269970, NP_00	XP_011527833, XP_0168	protein_coding	ENSG0000015531	29761		12624
812	VEZT	1.260	0.000	vezatin, adherens junctions transmembrane protein [Source:HGNC Symbol;Acc:HGNC:1	chr12:95611521-95696566	NM_017599	XM_017019601, XM_0	NP_060069	XP_006719540, XP_0067	protein_coding	ENSG0000002820	55591		18258
813	VMP1	1.068	0.000	vacuole membrane protein 1 [Source:HGNC Symbol;Acc:HGNC:29559]	chr17:57774666-57918453	NM_030938	.	NP_001316323, NP_00	.	protein_coding	ENSG0000006271	406991		29559
814	VPS13C	1.475	0.000	vacuolar protein sorting 13 homolog C [Source:HGNC Symbol;Acc:HGNC:23594]	chr15:62144587-62352672	NM_001018088, NN	.	NP_001018098, NP_06	.	protein_coding	ENSG0000012900	54832		23594
815	WDCP	1.259	0.000	WD repeat and coiled coil containing [Source:HGNC Symbol;Acc:HGNC:26157]	chr2:24252209-24270231	NM_001142319, NN	.	NP_001135791, NP_07	XP_016860518	protein_coding	ENSG0000016302	80304		26157
816	WDR47	1.434	0.000	WD repeat domain 47 [Source:HGNC Symbol;Acc:HGNC:29141]	chr1:109512835-109584735	NM_001142550, NN	XM_017000697, XM_0	NP_001136022, NP_00	XP_006710523, XP_0115	protein_coding	ENSG0000008543	22911		29141
817	YTHDC2	1.293	0.000	YTH domain containing 2 [Source:HGNC Symbol;Acc:HGNC:24721]	chr5:112849379-112930981	NM_022828	XM_011543585, XM_0	NP_073739	XP_011541883, XP_0115	protein_coding	ENSG0000004718	64848		24721
818	YTHDF3	1.232	0.000	YTH N6-methyladenosine RNA binding protein 3 [Source:HGNC Symbol;Acc:HGNC:2646	chr8:64081111-64125344	NM_001277813, NN	XM_011517510, XM_0	NP_001264742, NP_00	XP_011515811, XP_0115	protein_coding	ENSG0000018572	253943		26465
819	ZBTB47	-1.874	0.000	zinc finger and BTB domain containing 47 [Source:HGNC Symbol;Acc:HGNC:26955]	chr3:42695175-42707346	NM_145166	.	NP_660149	.	protein_coding	ENSG0000011485	92999		26955
820	ZNF638	1.374	0.000	zinc finger protein 638 [Source:HGNC Symbol;Acc:HGNC:17894]	chr2:71503722-71662191	NM_001014972, NN	XM_017003809, XM_0	NP_001014972, NP_00	XP_005264320, XP_0067	protein_coding	ENSG0000007529	27332		17894
821	ACOX2	-2.883	0.000	acyl-CoA oxidase 2 [Source:HGNC Symbol;Acc:HGNC:120]	chr3:58490862-58523046	NM_003500	XM_011534147, XM_0	NP_003491	XP_005265562, XP_0067	protein_coding	ENSG0000016830	8309		120
822	ALDH1L1	-1.534	0.000	aldehyde dehydrogenase 1 family member L1 [Source:HGNC Symbol;Acc:HGNC:3978]	chr3:125822404-125900029	NM_001270364, NN	XM_011512356, XM_0	NP_001257293, NP_00	XP_006713544, XP_0115	protein_coding	ENSG0000014490	10840		3978
823	ALS2CL	-1.193	0.000	ALS2 C-terminal like [Source:HGNC Symbol;Acc:HGNC:20605]	chr3:46710678-46735191	NM_001190707, NN	.	NP_001177636, NP_66	XP_006713156	protein_coding	ENSG0000017803	259173		20605
824	ANKEF1	1.301	0.000	ankyrin repeat and EF-hand domain containing 1 [Source:HGNC Symbol;Acc:HGNC:1580	chr20:10015688-10038951	NM_001303472, NN	XM_005260792	NP_001290401, NP_07	XP_005260849	protein_coding	ENSG0000013262	63926		15803
825	ARHGAP21	1.156	0.000	Rho GTPase activating protein 21 [Source:HGNC Symbol;Acc:HGNC:23725]	chr10:24872537-25012597	NM_020824	XM_017016459, XM_0	NP_065875	XP_005252599, XP_0052	protein_coding	ENSG0000010786	57584		23725
826	ASCC3	1.563	0.000	activating signal cointegrator 1 complex subunit 3 [Source:HGNC Symbol;Acc:HGNC:186	chr6:100956069-101329240	NM_001284271, NN	XM_011535396, XM_0	NP_001271200, NP_00	XP_011533696, XP_0115	protein_coding	ENSG0000011224	10973		18697
827	BAZ1B	0.940	0.000	bromodomain adjacent to zinc finger domain 1B [Source:HGNC Symbol;Acc:HGNC:961]	chr7:72854727-72936608	NM_032408	.	NP_115784	XP_016868262	protein_coding	ENSG0000000995	9031		961
828	BCAM	-1.011	0.000	basal cell adhesion molecule (Lutheran blood group) [Source:HGNC Symbol;Acc:HGNC:6	chr19:45312315-45324677	NM_001013257, NN	.	NP_001013275, NP_00	.	protein_coding	ENSG0000018724	4059		6722
829	BDP1	1.301	0.000	B double prime 1, subunit of RNA polymerase III transcription initiation factor IIIB [Sourc	chr5:70751441-70863647	NM_018429	XM_011543512, XM_0	NP_060899	XP_011541813, XP_0115	protein_coding	ENSG0000014573	55814		13652
830	CEP192	1.602	0.000	centrosomal protein 192 [Source:HGNC Symbol;Acc:HGNC:25515]	chr18:12991360-13125049	NM_032142	XM_011525676, XM_0	NP_115518	XP_005258164, XP_0052	protein_coding	ENSG0000010163	55125		25515
831	CERS6	1.075	0.000	ceramide synthase 6 [Source:HGNC Symbol;Acc:HGNC:23826]	chr2:169312371-169631647	NM_001256126, NN	.	NP_001243055, NP_98	.	protein_coding	ENSG0000017229	253782		23826
832	CFAP69	1.734	0.000	cilia and flagella associated protein 69 [Source:HGNC Symbol;Acc:HGNC:26107]	chr7:89874487-89940377	NM_001039706, NN	XM_017012624, XM_0	NP_001034795, NP_00	XP_011514879, XP_0115	protein_coding	ENSG0000010579	79846		26107
833	CNTRL	1.518	0.000	centriolin [Source:HGNC Symbol;Acc:HGNC:1858]	chr9:123837140-123939888	NM_007018	XM_017014226, XM_0	NP_008949	XP_005251736, XP_0067	protein_coding	ENSG0000011939	11064		1858
834	CYS1	-1.284	0.000	cystin 1 [Source:HGNC Symbol;Acc:HGNC:18525]	chr2:10196906-10220538	NM_001037160	.	NP_001032237	.	protein_coding	ENSG0000020579	192668		18525
835	DCDC2	1.310	0.000	doublecortin domain containing 2 [Source:HGNC Symbol;Acc:HGNC:18141]	chr6:24171983-24358512	NM_001195610, NN	.	NP_001182539, NP_05	.	protein_coding	ENSG0000014603	51473		18141
836	DCTN6	1.507	0.000	dynactin subunit 6 [Source:HGNC Symbol;Acc:HGNC:16964]	chr8:30013812-30041156	NM_006571	.	NP_006562	.	protein_coding	ENSG0000010467	10671		16964
837	DDX21	0.883	0.000	DEAD-box helicase 21 [Source:HGNC Symbol;Acc:HGNC:2744]	chr10:70715883-70744829	NM_001256910, NN	XM_011540336	NP_001243839, NP_00	XP_011538638	protein_coding	ENSG0000016573	9188		2744
838	DOCK5	1.523	0.000	dedicator of cytokinesis 5 [Source:HGNC Symbol;Acc:HGNC:23476]	chr8:25042237-25273232	NM_001322810, NN	XM_017013868, XM_0	NP_001309739, NP_07	XP_011542963, XP_0115	protein_coding	ENSG0000014745	80005		23476
839	ECE2	-1.567	0.000	endothelin converting enzyme 2 [Source:HGNC Symbol;Acc:HGNC:13275]	chr3:183967437-184010819	NM_001037324, NN	.	NP_001032401, NP_00	.	protein_coding	ENSG0000014519	9718		13275
840	EGFL7	-1.031	0.000	EGF like domain multiple 7 [Source:HGNC Symbol;Acc:HGNC:20594]	chr9:139553307-139567130	NM_016215, NM_2	XM_011518766, XM_0	NP_057299, NP_95885	XP_006717204, XP_0115	protein_coding	ENSG0000017288	51162		20594
841	FAR1	1.069	0.000	fatty acyl-CoA reductase 1 [Source:HGNC Symbol;Acc:HGNC:26222]	chr11:13690216-13753893	NM_032228	XM_011520401, XM_0	NP_115604	XP_011518702	protein_coding	ENSG0000019760	84188		26222
842	FRMD6	1.358	0.000	FERM domain containing 6 [Source:HGNC Symbol;Acc:HGNC:19839]	chr14:51955854-52197445	NM_001042481, NN	XM_011536424, XM_0	NP_001035946, NP_00	XP_006720092, XP_0067	protein_coding	ENSG0000013992	122786		19839
843	GLTSCR2	-0.951	0.000	glioma tumor suppressor candidate region gene 2 [Source:HGNC Symbol;Acc:HGNC:433	chr19:48248778-48260315	NM_015710	.	NP_056525	.	protein_coding	ENSG0000010537	29997		4333
844	GNPTAB	1.413	0.000	N-acetylglucosamine-1-phosphate transferase alpha and beta subunits [Source:HGNC Sy	chr12:102139274-102224716	NM_024312	XM_017019961, XM_0	NP_077288	XP_006719656, XP_0115	protein_coding	ENSG0000011167	79158		29670
845	GPSM3	-1.393	0.000	G-protein signaling modulator 3 [Source:HGNC Symbol;Acc:HGNC:13945]	chr6:32158542-32191844	NM_001276501, NN	.	NP_001263430, NP_07	.	protein_coding	ENSG0000021365	63940		13945
846	HCST	-1.891	0.000	hematopoietic cell signal transducer [Source:HGNC Symbol;Acc:HGNC:16977]	chr19:36393381-36395279	NM_001007469, NN	.	NP_001007470, NP_05	.	protein_coding	ENSG0000012626	10870		16977
847	HEATR5B	1.337	0.000	HEAT repeat containing 5B [Source:HGNC Symbol;Acc:HGNC:29273]	chr2:37208039-37311485	NM_019024	XM_017004380, XM_0	NP_061897	XP_006712097, XP_0067	protein_coding	ENSG0000000886	54497		29273
848	HNMT	3.017	0.000	histamine N-methyltransferase [Source:HGNC Symbol;Acc:HGNC:5028]	chr2:138721589-138773934	NM_001024074, NN	.	NP_001019245, NP_00	.	protein_coding	ENSG0000015054	3176		5028
849	HOOK1	1.324	0.000	hook microtubule tethering protein 1 [Source:HGNC Symbol;Acc:HGNC:19884]	chr1:60280532-60342050	NM_015888	XM_011541562	NP_056972	XP_011539864	protein_coding	ENSG0000013470	51361		19884
850	HSPA1A	-0.993	0.000	heat shock protein family A (Hsp70) member 1A [Source:HGNC Symbol;Acc:HGNC:5232]	chr6:31783240-31785723	NM_005345	.	NP_005336	.	protein_coding	ENSG0000020438	3303		5232
851	KMT2E	1.152	0.000	lysine methyltransferase 2E [Source:HGNC Symbol;Acc:HGNC:18541]	chr7:104654625-104754532	NM_018682, NM_1	XM_011516400, XM_0	NP_061152, NP_89184	XP_005250550, XP_0115	protein_coding	ENSG0000000548	55904		18541
852	KTN1	1.338	0.000	kinectin 1 [Source:HGNC Symbol;Acc:HGNC:6467]	chr14:56047032-56151301	NM_001079521, NN	XM_017021283, XM_0	NP_001072989, NP_00	XP_006720201, XP_0067	protein_coding	ENSG0000012677	3895		6467
853	LDAH	1.311	0.000	lipid droplet associated hydrolase [Source:HGNC Symbol;Acc:HGNC:26145]	chr2:20883773-21022890	NM_001282719, NN	XM_011533014	NP_001269648, NP_00	XP_011531316	protein_coding	ENSG0000011896	60526		26145
854	LMO7	1.223	0.000	LIM domain 7 [Source:HGNC Symbol;Acc:HGNC:6646]	chr13:76194569-76434006	NM_005358, NM_0	XM_017020598, XM_0	NP_001293009, NP_00	XP_011533377, XP_0115	protein_coding	ENSG0000013615	4008		6646
855	LNPEP	1.195	0.000	leucyl and cystinyl aminopeptidase [Source:HGNC Symbol;Acc:HGNC:6656]	chr5:96271167-96373219	NM_005575, NM_1	.	NP_005566, NP_78711	.	protein_coding	ENSG0000011344	4012		6656
856	LRRC8B	1.788	0.000	leucine rich repeat containing 8 family member B [Source:HGNC Symbol;Acc:HGNC:3069	chr1:89990461-90063423	NM_001134476, NN	XM_011541146, XM_0	NP_001127948, NP_05	XP_005270758, XP_0052	protein_coding	ENSG0000019714	23507		30692
857	MARCH7	1.380	0.000	membrane associated ring-CH-type finger 7 [Source:HGNC Symbol;Acc:HGNC:17393]	chr2:160568967-160761221	NM_001282805, NN	XM_017004721, XM_0	NP_001269734, NP_00	XP_005246830, XP_0052	protein_coding	ENSG0000013653	64844		17393
858	MATR3	0.938	0.000	matrin 3 [Source:HGNC Symbol;Acc:HGNC:6912]	chr5:138629336-138667048	NM_001194956, NN	.	NP_001181885, NP_06	.	protein_coding	ENSG0000001547	9782		6912
859	MCOLN2	1.404	0.000	mucolipin 2 [Source:HGNC Symbol;Acc:HGNC:13357]	chr1:85391265-85462795	NM_153259	XM_011541188, XM_0	NP_694991	XP_005270776, XP_0067	protein_coding	ENSG0000015389	255231		13357
860	MFN1	1.198	0.000	mitofusin 1 [Source:HGNC Symbol;Acc:HGNC:18262]	chr3:179065479-179112719	NM_033540	XM_011512963, XM_0	NP_284941	XP_005247653, XP_0115	protein_coding	ENSG0000017110	55669		18262
861	MIOS	1.361	0.000	meiosis regulator for oocyte development [Source:HGNC Symbol;Acc:HGNC:21905]	chr7:7606502-7647127	NM_019005	XM_005249783, XM_0	NP_061878	XP_005249837, XP_0052	protein_coding	ENSG0000016465	54468		21905
862	MMP2	-1.992	0.000	matrix metallopeptidase 2 [Source:HGNC Symbol;Acc:HGNC:7166]	chr16:55423611-55540603	NM_001127891, NN	.	NP_001121363, NP_00	.	protein_coding	ENSG0000008724	4313		7166

863	MT1X	-0.955	0.000	metallothionein 1X [Source:HGNC Symbol;Acc:HGNC:7405]	chr16:56716335-56718108	NM_005952	.	NP_005943	.	protein_coding	ENSG0000018719	4501	7405
864	MYO9A	1.450	0.000	myosin IXA [Source:HGNC Symbol;Acc:HGNC:7608]	chr15:72114629-72436029	NM_006901	XM_011521622, XM_0	NP_008832	XP_006720602, XP_0115	protein_coding	ENSG0000006693	4649	7608
865	NEURL3	-2.078	0.000	neuralized E3 ubiquitin protein ligase 3 [Source:HGNC Symbol;Acc:HGNC:25162]	chr2:97163379-97173846	NM_001285485, NM	XM_011512179, XM_0	NP_001272414, NP_00	XP_011510480, XP_0115	protein_coding	ENSG0000016312	93082	25162
866	NF1	1.185	0.000	neurofibromin 1 [Source:HGNC Symbol;Acc:HGNC:7765]	chr17:29421944-29704695	NM_000267, NM_0	XM_011524857, XM_0	NP_000258, NP_00103	XP_005258040, XP_0052	protein_coding	ENSG0000019671	4763	7765
867	OBSCN	-1.055	0.000	obscurin, cytoskeletal calmodulin and titin-interacting RhoGEF [Source:HGNC Symbol;Ac	chr1:228391206-228566575	NM_001098623, NM	XM_011544299, XM_0	NP_001092093, NP_00	XP_005273344, XP_0052	protein_coding	ENSG0000015435	84033	15719
868	OSGIN2	1.293	0.000	oxidative stress induced growth inhibitor family member 2 [Source:HGNC Symbol;Acc:H	chr8:90914086-90940116	NM_001126111, NM	XM_011517287	NP_001119583, NP_00	XP_011515589	protein_coding	ENSG0000016482	734	1355
869	PBXIP1	-0.989	0.000	PBX homeobox interacting protein 1 [Source:HGNC Symbol;Acc:HGNC:21199]	chr1:154916551-154928597	NM_001317734, NM	.	NP_001304663, NP_06	.	protein_coding	ENSG0000016334	57326	21199
870	PDZD11	-0.919	0.000	PDZ domain containing 11 [Source:HGNC Symbol;Acc:HGNC:28034]	chrX:69506444-69509829	NM_016484	XM_011530973	NP_057568	XP_011529275	protein_coding	ENSG0000012050	51248	28034
871	PPIP5K2	1.431	0.000	diphosphoinositol pentakisphosphate kinase 2 [Source:HGNC Symbol;Acc:HGNC:29035]	chr5:102455903-102548500	NM_001276277, NM	XM_017009269, XM_0	NP_001263206, NP_00	XP_005271992, XP_0052	protein_coding	ENSG0000014572	23262	29035
872	PPP2R5E	1.400	0.000	protein phosphatase 2 regulatory subunit B'epsilon [Source:HGNC Symbol;Acc:HGNC:93	chr14:63838074-64010092	NM_001282179, NM	XM_011536922	NP_001269108, NP_00	XP_011535224	protein_coding	ENSG0000015400	5529	9313
873	PPP4R3B	1.254	0.000	protein phosphatase 4 regulatory subunit 3B [Source:HGNC Symbol;Acc:HGNC:29267]	chr2:55774427-55844749	NM_001122964, NM	XM_017004531, XM_0	NP_001116436, NP_00	XP_005264499, XP_0052	protein_coding	ENSG0000027505	57223	29267
874	RAB11FIP1	0.937	0.000	RAB11 family interacting protein 1 [Source:HGNC Symbol;Acc:HGNC:30265]	chr8:37716135-37756985	NM_001002814, NM	.	NP_001002814, NP_07	XP_016869358	protein_coding	ENSG0000015667	80223	30265
875	RABGEF1	1.124	0.000	RAB guanine nucleotide exchange factor 1 [Source:HGNC Symbol;Acc:HGNC:17676]	chr7:66204961-66276451	NM_001287060, NM	.	NP_001273989, NP_00	.	protein_coding	ENSG0000015471	27342	17676
876	RASAL2	1.596	0.000	RAS protein activator like 2 [Source:HGNC Symbol;Acc:HGNC:9874]	chr1:178063275-178466074	NM_004841, NM_1	XM_011510169, XM_0	NP_004832, NP_73379	XP_005245679, XP_0115	protein_coding	ENSG0000007539	9462	9874
877	RASGRP3	1.502	0.000	RAS guanyl releasing protein 3 [Source:HGNC Symbol;Acc:HGNC:14545]	chr2:33661390-33789817	NM_001139488, NM	XM_017003763, XM_0	NP_001132960, NP_05	XP_005264303, XP_0115	protein_coding	ENSG0000015268	25780	14545
878	REEP2	-1.177	0.000	receptor accessory protein 2 [Source:HGNC Symbol;Acc:HGNC:17975]	chr5:137774705-137782658	NM_001271803, NM	.	NP_001258732, NP_05	.	protein_coding	ENSG0000013256	51308	17975
879	REST	1.185	0.000	RE1 silencing transcription factor [Source:HGNC Symbol;Acc:HGNC:9966]	chr4:57774041-57802010	NM_001193508, NM	XM_011534401	NP_001180437, NP_00	XP_011532703	protein_coding	ENSG0000008409	5978	9966
880	S100A16	-0.971	0.000	S100 calcium binding protein A16 [Source:HGNC Symbol;Acc:HGNC:20441]	chr1:153579361-153585559	NM_001317008, NM	XM_005244906, XM_0	NP_001303936, NP_00	XP_005244962	protein_coding	ENSG0000018864	140576	20441
881	SHPRH	1.459	0.000	SNF2 histone linker PHD RING helicase [Source:HGNC Symbol;Acc:HGNC:19336]	chr6:146205942-146285559	NM_001042683, NM	XM_011535719, XM_0	NP_001036148, NP_77	XP_006715502, XP_0067	protein_coding	ENSG0000014641	257218	19336
882	SLAIN1	1.431	0.000	SLAIN motif family member 1 [Source:HGNC Symbol;Acc:HGNC:26387]	chr13:78271988-78338377	NM_001040153, NM	XM_011534930, XM_0	NP_001035243, NP_00	XP_011533231, XP_0115	protein_coding	ENSG0000013973	122060	26387
883	SLC20A1	0.897	0.000	solute carrier family 20 member 1 [Source:HGNC Symbol;Acc:HGNC:10946]	chr2:113403433-113421404	NM_005415	XM_011511686	NP_005406	.	protein_coding	ENSG0000014413	6574	10946
884	SLC38A1	0.897	0.000	solute carrier family 38 member 1 [Source:HGNC Symbol;Acc:HGNC:13447]	chr12:46576845-46662902	NM_001077484, NM	XM_011538787, XM_0	NP_001070952, NP_00	XP_011537086, XP_0115	protein_coding	ENSG0000011137	81539	13447
885	SNAP25	1.637	0.000	synaptosome associated protein 25 [Source:HGNC Symbol;Acc:HGNC:11132]	chr20:10199477-10288066	NM_003081, NM_1	XM_017028022, XM_0	NP_001309831, NP_00	XP_005260865, XP_0052	protein_coding	ENSG0000013263	6616	11132
886	SNX10	1.193	0.000	sorting nexin 10 [Source:HGNC Symbol;Acc:HGNC:14974]	chr7:26331514-26413949	NM_001199835, NM	XM_006715712, XM_0	NP_001186764, NP_00	XP_006715773, XP_0067	protein_coding	ENSG0000008630	29887	14974
887	STK17A	1.125	0.000	serine/threonine kinase 17a [Source:HGNC Symbol;Acc:HGNC:11395]	chr7:43622663-43666385	NM_004760	.	NP_004751	.	protein_coding	ENSG0000016454	9263	11395
888	STK32C	-1.429	0.000	serine/threonine kinase 32C [Source:HGNC Symbol;Acc:HGNC:21332]	chr10:134020995-134121444	NM_001318879, NM	XM_011539696, XM_0	NP_001305808, NP_00	XP_011537990, XP_0115	protein_coding	ENSG0000016575	282974	21332
889	STX10	-1.021	0.000	syntaxin 10 [Source:HGNC Symbol;Acc:HGNC:11428]	chr19:13254871-13261160	NM_001271609, NM	.	NP_001258538, NP_00	.	protein_coding	ENSG0000010491	8677	11428
890	STX17	1.410	0.000	syntaxin 17 [Source:HGNC Symbol;Acc:HGNC:11432]	chr9:102668914-102732623	NM_017919	XM_011518822, XM_0	NP_060389	XP_011517122, XP_0115	protein_coding	ENSG0000013687	55014	11432
891	TEAD1	1.259	0.000	TEA domain transcription factor 1 [Source:HGNC Symbol;Acc:HGNC:11714]	chr11:12696137-12966030	NM_021961	.	NP_068780	.	protein_coding	ENSG0000018707	7003	11714
892	TMA7	-0.988	0.000	translation machinery associated 7 homolog [Source:HGNC Symbol;Acc:HGNC:26932]	chr3:48481666-48485616	NM_001329417, NM	.	NP_001316346, NP_05	.	protein_coding	ENSG0000023211	51372	26932
893	TRIM37	1.284	0.000	tripartite motif containing 37 [Source:HGNC Symbol;Acc:HGNC:7523]	chr17:56833229-57184282	NM_001005207, NM	XM_011524837, XM_0	NP_001005207, NP_00	XP_005257442, XP_0052	protein_coding	ENSG0000010839	4591	7523
894	TTPAL	1.050	0.000	alpha tocopherol transfer protein like [Source:HGNC Symbol;Acc:HGNC:16114]	chr20:43104525-43123244	NM_001039199, NM	XM_011529044, XM_0	NP_001034288, NP_00	XP_011527345, XP_0115	protein_coding	ENSG0000012412	79183	16114
895	UBXN4	1.051	0.000	UBX domain protein 4 [Source:HGNC Symbol;Acc:HGNC:14860]	chr2:136499188-136542625	NM_014607	.	NP_055422	.	protein_coding	ENSG0000014422	23190	14860
896	UBXN6	-0.967	0.000	UBX domain protein 6 [Source:HGNC Symbol;Acc:HGNC:14928]	chr19:4445002-4457819	NM_001171091, NM	.	NP_001164562, NP_07	.	protein_coding	ENSG0000016767	80700	14928
897	UPRT	-1.314	0.000	uracil phosphoribosyltransferase homolog [Source:HGNC Symbol;Acc:HGNC:28334]	chrX:74493922-74524435	NM_001307944, NM	XM_011530866	NP_001294873, NP_65	XP_011529168, XP_0115	protein_coding	ENSG0000009484	139596	28334
898	USP24	1.152	0.000	ubiquitin specific peptidase 24 [Source:HGNC Symbol;Acc:HGNC:12623]	chr1:55532031-55680786	NM_015306	XM_017000834, XM_0	NP_056121	XP_016856321, XP_0168	protein_coding	ENSG0000016240	23358	12623
899	VPS36	1.120	0.000	vacuolar protein sorting 36 homolog [Source:HGNC Symbol;Acc:HGNC:20312]	chr13:52986736-53024757	NM_001282168, NM	.	NP_001269097, NP_00	.	protein_coding	ENSG0000013610	51028	20312
900	VPS54	1.264	0.000	VPS54, GARP complex subunit [Source:HGNC Symbol;Acc:HGNC:18652]	chr2:64119279-64246206	NM_001005739, NM	XM_006712029	NP_001005739, NP_05	XP_006712092	protein_coding	ENSG0000014395	51542	18652
901	WDR18	-1.090	0.000	WD repeat domain 18 [Source:HGNC Symbol;Acc:HGNC:17956]	chr19:984270-994569	NM_024100	.	NP_077005	XP_016882516	protein_coding	ENSG0000006526	57418	17956
902	ZFP91	1.022	0.000	ZFP91 zinc finger protein [Source:HGNC Symbol;Acc:HGNC:14983]	chr11:58346583-58388515	NM_001197051, NM	.	NP_001183980, NP_44	.	protein_coding	ENSG0000018666	80829	14983
903	ZZZ3	1.323	0.000	zinc finger ZZ-type containing 3 [Source:HGNC Symbol;Acc:HGNC:24523]	chr1:78030184-78149104	NM_001308237, NM	XM_005270729, XM_0	NP_001295166, NP_05	XP_005270782, XP_0052	protein_coding	ENSG0000003654	26009	24523
904	AC013461.1	1.072	0.000	Mitogen-activated protein kinase kinase kinase MLT [Source:UniProtKB/Swiss-Prot;Acc:	chr2:173940162-174132738	NM_016653, NM_1	XM_005246640	NP_057737, NP_59840	XP_005246697, XP_0168	protein_coding	ENSG0000009143	51776	.
905	AGL	1.368	0.000	amylo-alpha-1, 6-glucosidase, 4-alpha-glucanotransferase [Source:HGNC Symbol;Acc:HG	chr1:100315639-100389579	NM_000028, NM_0	XM_005270557	NP_000019, NP_00063	XP_005270614	protein_coding	ENSG0000016268	178	321
906	APOBEC3G	-1.040	0.000	apolipoprotein B mRNA editing enzyme catalytic subunit 3G [Source:HGNC Symbol;Acc:	chr22:39436608-39483748	NM_021822	XM_006724290	NP_068594	XP_006724353	protein_coding	ENSG0000023971	60489	17357
907	ARHGAP12	1.210	0.000	Rho GTPase activating protein 12 [Source:HGNC Symbol;Acc:HGNC:16348]	chr10:32094331-32217804	NM_001270695, NM	XM_011519761, XM_0	NP_001257624, NP_00	XP_005252701, XP_0115	protein_coding	ENSG0000016532	94134	16348
908	ASB9	-1.567	0.000	ankyrin repeat and SOCS box containing 9 [Source:HGNC Symbol;Acc:HGNC:17184]	chrX:15262108-15288589	NM_001031739, NM	XM_011545460, XM_0	NP_001026909, NP_00	XP_005274503, XP_0115	protein_coding	ENSG0000010204	140462	17184
909	BLM	1.258	0.000	Bloom syndrome RecQ like helicase [Source:HGNC Symbol;Acc:HGNC:1058]	chr15:91260557-91359395	NM_000057, NM_0	.	NP_000048, NP_00127	.	protein_coding	ENSG0000019729	641	1058
910	BRCA2	1.324	0.000	BRCA2, DNA repair associated [Source:HGNC Symbol;Acc:HGNC:1101]	chr13:32889610-32974403	NM_000059	XM_011535204, XM_0	NP_000050	XP_011533505	protein_coding	ENSG0000013961	675	1101
911	CADM1	1.262	0.000	cell adhesion molecule 1 [Source:HGNC Symbol;Acc:HGNC:5951]	chr11:115039955-115375283	NM_001098517, NM	XM_017017461, XM_0	NP_001091987, NP_00	XP_005271551, XP_0168	protein_coding	ENSG0000018298	23705	5951
912	CDC25B	-0.896	0.000	cell division cycle 25B [Source:HGNC Symbol;Acc:HGNC:1726]	chr20:3767418-3786768	NM_001287516, NM	.	NP_001274445, NP_00	.	protein_coding	ENSG0000010122	994	1726
913	CWF19L2	1.526	0.000	CWF19-like 2, cell cycle control (S. pombe) [Source:HGNC Symbol;Acc:HGNC:26508]	chr11:107197070-107328570	NM_152434	.	NP_689647	.	protein_coding	ENSG0000015240	143884	26508
914	CXorf57	2.429	0.000	chromosome X open reading frame 57 [Source:HGNC Symbol;Acc:HGNC:25486]	chrX:105855159-105922672	NM_001184782, NM	.	NP_001171711, NP_06	.	protein_coding	ENSG0000014723	55086	25486
915	CYBA	-0.947	0.000	cytochrome b-245 alpha chain [Source:HGNC Symbol;Acc:HGNC:2577]	chr16:88709690-88717560	NM_000101	XM_011522905	NP_000092	XP_011521207	protein_coding	ENSG0000005152	1535	2577
916	DHX40	1.064	0.000	DEAH-box helicase 40 [Source:HGNC Symbol;Acc:HGNC:18018]	chr17:57642885-57685706	NM_001166301, NM	XM_011525254, XM_0	NP_001159773, NP_07	XP_006722146, XP_0115	protein_coding	ENSG0000010840	79665	18018

917	DIO3	-2.278	0.000	deiodinase, iodothyronine, type III [Source:HGNC Symbol;Acc:HGNC:2885]	chr14:102027687-102029789	NM_001362	.	NP_001353	.	protein_coding	ENSG0000019740	1735		2885
918	DUSP13	-2.119	0.000	dual specificity phosphatase 13 [Source:HGNC Symbol;Acc:HGNC:19681]	chr10:76854189-76868979	NM_001007271, NN	XM_017016313, XM_0	NP_001007272, NP_00	XP_005269940, XP_0052	protein_coding	ENSG0000007939	51207		19681
919	EHBP1	1.250	0.000	EH domain binding protein 1 [Source:HGNC Symbol;Acc:HGNC:29144]	chr2:62900985-63273622	NM_001142614, NN	XM_017003653, XM_0	NP_001136086, NP_00	XP_005264282, XP_0052	protein_coding	ENSG0000011550	23301		29144
920	EIF5	0.950	0.000	eukaryotic translation initiation factor 5 [Source:HGNC Symbol;Acc:HGNC:3299]	chr14:103800338-103811362	NM_001969, NM_1	.	NP_001960, NP_89211	.	protein_coding	ENSG0000010066	1983		3299
921	ESF1	1.236	0.000	ESF1 nucleolar pre-rRNA processing protein homolog [Source:HGNC Symbol;Acc:HGNC:	chr20:13694968-13765532	NM_001276380, NN	.	NP_001263309, NP_05	XP_016883363	protein_coding	ENSG0000008904	51575		15898
922	FAAH2	-1.790	0.000	fatty acid amide hydrolase 2 [Source:HGNC Symbol;Acc:HGNC:26440]	chrX:57313138-57515629	NM_174912	XM_011530767, XM_0	NP_777572	XP_005262039, XP_0052	protein_coding	ENSG0000016559	158584		26440
923	FAM133B	1.357	0.000	family with sequence similarity 133 member B [Source:HGNC Symbol;Acc:HGNC:28629]	chr7:92190106-92219708	NM_001040057, NN	XM_011516005, XM_0	NP_001035146, NP_00	XP_011514306, XP_0115	protein_coding	ENSG0000023454	257415		28629
924	FAM57B	-1.701	0.000	family with sequence similarity 57 member B [Source:HGNC Symbol;Acc:HGNC:25295]	chr16:30034654-30042580	NM_001318504, NN	XM_017023754, XM_0	NP_001305433, NP_11	XP_005255673, XP_0168	protein_coding	ENSG0000014992	83723		25295
925	FIBCD1	-1.058	0.000	fibrinogen C domain containing 1 [Source:HGNC Symbol;Acc:HGNC:25922]	chr9:133777824-133814455	NM_001145106, NN	.	NP_001138578, NP_11	.	protein_coding	ENSG0000013072	84929		25922
926	FNTA	1.002	0.000	farnesyltransferase, CAAX box, alpha [Source:HGNC Symbol;Acc:HGNC:3782]	chr8:42911295-42940931	NM_002027	.	NP_002018	.	protein_coding	ENSG0000016852	2339		3782
927	GPX1	-0.885	0.000	glutathione peroxidase 1 [Source:HGNC Symbol;Acc:HGNC:4553]	chr3:49394608-49396033	NM_000581, NM_0	.	NP_000572, NP_00131	.	protein_coding	ENSG0000023327	2876		4553
928	GRB7	-1.060	0.000	growth factor receptor bound protein 7 [Source:HGNC Symbol;Acc:HGNC:4567]	chr17:37894179-37903544	NM_001030002, NN	XM_011524683	NP_001025173, NP_00	XP_016880026	protein_coding	ENSG0000014173	2886		4567
929	HLA-F	-1.107	0.000	major histocompatibility complex, class I, F [Source:HGNC Symbol;Acc:HGNC:4963]	chr6:29690551-29695073	NM_001098478, NN	XM_005249054	NP_001091948, NP_00	XP_011512866, XP_0168	protein_coding	ENSG0000020464	3134		4963
930	IFI6	-1.079	0.000	interferon alpha inducible protein 6 [Source:HGNC Symbol;Acc:HGNC:4054]	chr1:27992571-27998729	NM_002038, NM_0	.	NP_002029, NP_07501	.	protein_coding	ENSG0000012670	2537		4054
931	IMPDH2	-0.909	0.000	inosine monophosphate dehydrogenase 2 [Source:HGNC Symbol;Acc:HGNC:6053]	chr3:49061757-49066823	NM_000884	XM_006713128	NP_000875	XP_006713191	protein_coding	ENSG0000017803	3615		6053
932	JAGN1	-1.181	0.000	jagunal homolog 1 [Source:HGNC Symbol;Acc:HGNC:26926]	chr3:9932237-9936033	NM_032492	.	NP_115881	.	protein_coding	ENSG0000017113	84522		26926
933	KIAA2026	1.498	0.000	KIAA2026 [Source:HGNC Symbol;Acc:HGNC:23378]	chr9:5919007-6007787	NM_001017969	XM_011517760, XM_0	NP_001017969	XP_005251442, XP_0115	protein_coding	ENSG0000018335	158358		23378
934	LRRC10B	-1.529	0.000	leucine rich repeat containing 10B [Source:HGNC Symbol;Acc:HGNC:37215]	chr11:61276271-61278490	NM_001145077	.	NP_001138549	.	protein_coding	ENSG0000020495	390205		37215
935	MAOB	-1.233	0.000	monoamine oxidase B [Source:HGNC Symbol;Acc:HGNC:6834]	chrX:43625857-43741693	NM_000898	XM_017029524, XM_0	NP_000889	XP_005272665, XP_0168	protein_coding	ENSG0000006953	4129		6834
936	MED1	1.133	0.000	mediator complex subunit 1 [Source:HGNC Symbol;Acc:HGNC:9234]	chr17:37560537-37607539	NM_004774	XM_006721957, XM_0	NP_004765	XP_005257522, XP_0067	protein_coding	ENSG0000012568	5469		9234
937	MMP14	-0.917	0.000	matrix metallopeptidase 14 [Source:HGNC Symbol;Acc:HGNC:7160]	chr14:23305765-23316970	NM_004995	.	NP_004986	.	protein_coding	ENSG0000015722	4323		7160
938	MS4A15	-2.037	0.000	membrane spanning 4-domains A15 [Source:HGNC Symbol;Acc:HGNC:28573]	chr11:60524425-60544204	NM_001098835, NN	XM_011544814, XM_0	NP_001092305, NP_00	XP_006718522, XP_0115	protein_coding	ENSG0000016696	219995		28573
939	MSANTD2	1.441	0.000	Myb/SANT DNA binding domain containing 2 [Source:HGNC Symbol;Acc:HGNC:26266]	chr11:124636393-124670569	NM_001301087, NN	XM_011542983, XM_0	NP_001288016, NP_00	XP_005271730, XP_0067	protein_coding	ENSG0000012045	79684		26266
940	MTM1	1.250	0.000	myotubularin 1 [Source:HGNC Symbol;Acc:HGNC:7448]	chrX:149737068-149841795	NM_000252	XM_017029549, XM_0	NP_000243	XP_005274744, XP_0115	protein_coding	ENSG0000017110	4534		7448
941	MYO6	1.285	0.000	myosin VI [Source:HGNC Symbol;Acc:HGNC:7605]	chr6:76458908-76629254	NM_001300899, NN	XM_005248726, XM_0	NP_001287828, NP_00	XP_005248776, XP_0052	protein_coding	ENSG0000019658	4646		7605
942	NOC3L	1.201	0.000	NOC3 like DNA replication regulator [Source:HGNC Symbol;Acc:HGNC:24034]	chr10:96092982-96122696	NM_022451	.	NP_071896	.	protein_coding	ENSG0000017314	64318		24034
943	ORC2	1.089	0.000	origin recognition complex subunit 2 [Source:HGNC Symbol;Acc:HGNC:8488]	chr2:201773695-201828403	NM_006190	XM_011511254, XM_0	NP_006181	XP_006712618, XP_0115	protein_coding	ENSG0000011594	4999		8488
944	PANX2	-1.467	0.000	pannexin 2 [Source:HGNC Symbol;Acc:HGNC:8600]	chr22:50609159-50618723	NM_001160300, NN	.	NP_001153772, NP_44	.	protein_coding	ENSG0000007315	56666		8600
945	PHIP	1.328	0.000	pleckstrin homology domain interacting protein [Source:HGNC Symbol;Acc:HGNC:1567	chr6:79645583-79787953	NM_017934	XM_011535918, XM_0	NP_060404	XP_005248786, XP_0115	protein_coding	ENSG0000014624	55023		15673
946	PHKA2	-1.089	0.000	phosphorylase kinase regulatory subunit alpha 2 [Source:HGNC Symbol;Acc:HGNC:8926	chrX:18910417-19002716	NM_000292	XM_011545537, XM_0	NP_000283	XP_005274605, XP_0052	protein_coding	ENSG0000004444	5256		8926
947	PLS3	0.954	0.000	plastin 3 [Source:HGNC Symbol;Acc:HGNC:9091]	chrX:114795508-114885181	NM_001136025, NN	XM_011537534	NP_001129497, NP_00	XP_011535836	protein_coding	ENSG0000010202	5358		9091
948	POMGNT2	-1.098	0.000	protein O-linked mannose N-acetylglucosaminyltransferase 2 (beta 1,4-) [Source:HGNC	chr3:43120723-43147568	NM_032806	XM_011534164, XM_0	NP_116195	XP_005265572, XP_0115	protein_coding	ENSG0000014464	84892		25902
949	PQBP1	-1.398	0.000	polyglutamine binding protein 1 [Source:HGNC Symbol;Acc:HGNC:9330]	chrX:48750729-48760419	NM_001032381, NN	XM_017029207, XM_0	NP_001027553, NP_00	XP_005272628, XP_0052	protein_coding	ENSG0000010210	10084		9330
950	PYCARD	-0.985	0.000	PYD and CARD domain containing [Source:HGNC Symbol;Acc:HGNC:16608]	chr16:31212805-31214313	NM_013258, NM_1	.	NP_037390, NP_66018	.	protein_coding	ENSG0000010349	29108		16608
951	RAB3GAP2	1.334	0.000	RAB3 GTPase activating non-catalytic protein subunit 2 [Source:HGNC Symbol;Acc:HGN	chr1:220321634-220445796	NM_012414	.	NP_036546	.	protein_coding	ENSG0000011887	25782		17168
952	RASA1	1.356	0.000	RAS p21 protein activator 1 [Source:HGNC Symbol;Acc:HGNC:9871]	chr5:86563704-86687748	NM_002890, NM_0	XM_011543526, XM_0	NP_002881, NP_07217	XP_011541827, XP_0115	protein_coding	ENSG0000014571	5921		9871
953	RRP9	-0.922	0.000	ribosomal RNA processing 9, small subunit (SSU) processome component, homolog (yea	chr3:51967445-51975957	NM_004704	.	NP_004695	.	protein_coding	ENSG0000011476	9136		16829
954	SEL1L	1.116	0.000	SEL1L ERAD E3 ligase adaptor subunit [Source:HGNC Symbol;Acc:HGNC:10717]	chr14:81937892-82000205	NM_001244984, NN	XM_005267989, XM_0	NP_001231913, NP_00	XP_005268045, XP_0052	protein_coding	ENSG0000007153	6400		10717
955	SNX2	1.158	0.000	sorting nexin 2 [Source:HGNC Symbol;Acc:HGNC:11173]	chr5:122110690-122165803	NM_001278199, NN	.	NP_001265128, NP_00	.	protein_coding	ENSG0000020530	6643		11173
956	SSX2IP	1.473	0.000	SSX family member 2 interacting protein [Source:HGNC Symbol;Acc:HGNC:16509]	chr1:85109389-85156228	NM_001166293, NN	XM_017000237, XM_0	NP_001159765, NP_00	XP_005270484, XP_0052	protein_coding	ENSG0000011715	117178		16509
957	ST3GAL4	-1.053	0.000	ST3 beta-galactoside alpha-2,3-sialyltransferase 4 [Source:HGNC Symbol;Acc:HGNC:108	chr11:126225554-126284536	NM_001254757, NN	XM_017018134	NP_001241686, NP_00	XP_016873623	protein_coding	ENSG0000011008	6484		10864
958	STK38L	1.151	0.000	serine/threonine kinase 38 like [Source:HGNC Symbol;Acc:HGNC:17848]	chr12:27397107-27478892	NM_015000	XM_006719059, XM_0	NP_055815	XP_005253399, XP_0067	protein_coding	ENSG0000021145	23012		17848
959	TMEM245	1.031	0.000	transmembrane protein 245 [Source:HGNC Symbol;Acc:HGNC:1363]	chr9:111777431-111882225	NM_032012	XM_011518452, XM_0	NP_114401	XP_011516748, XP_0115	protein_coding	ENSG0000010677	23731		1363
960	TMX3	1.397	0.000	thioredoxin related transmembrane protein 3 [Source:HGNC Symbol;Acc:HGNC:24718]	chr18:66340924-66382535	NM_019022	XM_011526033, XM_0	NP_061895	XP_005266771, XP_0115	protein_coding	ENSG0000016647	54495		24718
961	TNKS2	1.201	0.000	tankyrase 2 [Source:HGNC Symbol;Acc:HGNC:15677]	chr10:93558068-93625033	NM_025235	XM_017016699, XM_0	NP_079511	XP_005270242, XP_0115	protein_coding	ENSG0000010785	80351		15677
962	TOPBP1	1.263	0.000	topoisomerase (DNA) II binding protein 1 [Source:HGNC Symbol;Acc:HGNC:17008]	chr3:133319416-133380737	NM_007027	XM_017005636, XM_0	NP_008958	XP_005247133, XP_0168	protein_coding	ENSG0000016378	11073		17008
963	TWSG1	1.098	0.000	twisted gastrulation BMP signaling modulator 1 [Source:HGNC Symbol;Acc:HGNC:12429	chr18:9334764-9402418	NM_020648	.	NP_065699	.	protein_coding	ENSG0000012879	57045		12429
964	UBE2L6	-1.167	0.000	ubiquitin conjugating enzyme E2 L6 [Source:HGNC Symbol;Acc:HGNC:12490]	chr11:57319128-57335757	NM_004223, NM_1	.	NP_004214, NP_93782	.	protein_coding	ENSG0000015658	9246		12490
965	VHL	-1.185	0.000	von Hippel-Lindau tumor suppressor [Source:HGNC Symbol;Acc:HGNC:12687]	chr3:10182691-10193904	NM_000551, NM_1	.	NP_000542, NP_93779	.	protein_coding	ENSG0000013408	7428		12687
966	ZBED5	1.196	0.000	zinc finger BED-type containing 5 [Source:HGNC Symbol;Acc:HGNC:30803]	chr11:10874250-10879620	NM_001143667, NN	.	NP_001137139, NP_06	.	protein_coding	ENSG0000023628	58486		30803
967	ZC3HAV1L	1.480	0.000	zinc finger CCCH-type containing, antiviral 1 like [Source:HGNC Symbol;Acc:HGNC:2242	chr7:138710451-138720775	NM_080660	.	NP_542391	XP_011514990	protein_coding	ENSG0000014685	92092		22423
968	ACVRL1	-2.286	0.000	activin A receptor like type 1 [Source:HGNC Symbol;Acc:HGNC:175]	chr12:52301201-52317143	NM_000020, NM_0	XM_011539008, XM_0	NP_000011, NP_00107	XP_005269292, XP_0115	protein_coding	ENSG0000013956	94		175
969	BHLHE40	-0.916	0.000	basic helix-loop-helix family member e40 [Source:HGNC Symbol;Acc:HGNC:1046]	chr3:5020800-5027008	NM_003670	.	NP_003661	.	protein_coding	ENSG0000013410	8553		1046
970	BRPF1	-1.147	0.000	bromodomain and PHD finger containing 1 [Source:HGNC Symbol;Acc:HGNC:14255]	chr3:9773412-9789702	NM_001003694, NN	XM_011534102, XM_0	NP_001003694, NP_00	XP_005265506, XP_0052	protein_coding	ENSG0000015698	7862		14255

971	C12orf4	1.505	0.000	chromosome 12 open reading frame 4 [Source:HGNC Symbol;Acc:HGNC:1184]	chr12:4596893-4647674	NM_001304811, NM	XM_011520986, XM_0	NP_001291740, NP_06	XP_005253773	protein_coding	ENSG0000004762	57102		1184	
972	CAPRIN2	1.306	0.000	caprin family member 2 [Source:HGNC Symbol;Acc:HGNC:21259]	chr12:30862486-30907448	NM_001002259, NM	XM_011520829, XM_0	NP_001002259, NP_00	XP_006719201, XP_0067	protein_coding	ENSG0000011088	65981		21259	
973	CCL7	-1.882	0.000	C-C motif chemokine ligand 7 [Source:HGNC Symbol;Acc:HGNC:10634]	chr17:32597239-32599261	NM_006273	.	NP_006264	.	protein_coding	ENSG0000010868	6354		10634	
974	CCT8	0.939	0.000	chaperonin containing TCP1 subunit 8 [Source:HGNC Symbol;Acc:HGNC:1623]	chr21:30428642-30446118	NM_001282907, NM	.	NP_001269836, NP_00	.	protein_coding	ENSG0000015626	10694		1623	
975	CEP120	1.398	0.000	centrosomal protein 120 [Source:HGNC Symbol;Acc:HGNC:26690]	chr5:122680578-122759286	NM_001166226, NM	XM_011543185, XM_0	NP_001159698, NP_69	XP_005271958, XP_0115	protein_coding	ENSG0000016894	153241		26690	
976	CXCL2	-1.585	0.000	C-X-C motif chemokine ligand 2 [Source:HGNC Symbol;Acc:HGNC:4603]	chr4:74962751-74965010	NM_002089	.	NP_002080	.	protein_coding	ENSG0000008104	2920		4603	
977	CYP2U1	1.278	0.000	cytochrome P450 family 2 subfamily U member 1 [Source:HGNC Symbol;Acc:HGNC:205	chr4:108852524-108874613	NM_183075	XM_005262717	NP_898898	XP_005262774	protein_coding	ENSG0000015501	113612		20582	
978	DYRK1B	-1.339	0.000	dual specificity tyrosine phosphorylation regulated kinase 1B [Source:HGNC Symbol;Acc	chr19:40315989-40324841	NM_004714, NM_0	XM_011527470, XM_0	NP_004705, NP_00647	XP_005259455	protein_coding	ENSG0000010520	9149		3092	
979	ETF1	0.841	0.000	eukaryotic translation termination factor 1 [Source:HGNC Symbol;Acc:HGNC:3477]	chr5:137841783-137878989	NM_001256302, NM	XM_005271921	NP_001243231, NP_00	XP_005271978	protein_coding	ENSG0000012070	2107		3477	
980	FAM13B	1.424	0.000	family with sequence similarity 13 member B [Source:HGNC Symbol;Acc:HGNC:1335]	chr5:137225125-137368775	NM_001101800, NM	XM_017009550, XM_0	NP_001095270, NP_00	XP_005272064, XP_0067	protein_coding	ENSG0000003100	51306		1335	
981	FAM35A	1.235	0.000	family with sequence similarity 35 member A [Source:HGNC Symbol;Acc:HGNC:28773]	chr10:88854952-88951225	NM_019054	XM_017016351, XM_0	NP_061927	XP_005269977, XP_0067	protein_coding	ENSG0000012237	54537		28773	
982	FEZ1	-1.454	0.000	fasciculation and elongation protein zeta 1 [Source:HGNC Symbol;Acc:HGNC:3659]	chr11:125315656-125366206	NM_005103, NM_0	XM_005271735, XM_0	NP_005094, NP_07204	XP_005271791, XP_0052	protein_coding	ENSG0000014955	9638		3659	
983	FSTL4	-1.685	0.000	follistatin like 4 [Source:HGNC Symbol;Acc:HGNC:21389]	chr5:132532146-132948255	NM_015082	XM_011543284, XM_0	NP_055897	XP_011541585, XP_0115	protein_coding	ENSG0000005310	23105		21389	
984	FXR1	1.060	0.000	FMR1 autosomal homolog 1 [Source:HGNC Symbol;Acc:HGNC:4023]	chr3:180630089-180700541	NM_001013438, NM	XM_011513217, XM_0	NP_001013456, NP_00	XP_005247870, XP_0052	protein_coding	ENSG0000011441	8087		4023	
985	GALNT3	1.151	0.000	polypeptide N-acetylgalactosaminyltransferase 3 [Source:HGNC Symbol;Acc:HGNC:4125	chr2:166604100-166651192	NM_004482	XM_011510929, XM_0	NP_004473	XP_005246506, XP_0115	protein_coding	ENSG0000011533	2591		4125	
986	GFPT1	0.930	0.000	glutamine--fructose-6-phosphate transaminase 1 [Source:HGNC Symbol;Acc:HGNC:4241	chr2:69546900-69614386	NM_001244710, NM	.	NP_001231639, NP_00	XP_016859290, XP_0168	protein_coding	ENSG0000019838	2673		4241	
987	GOLGA8B	1.684	0.000	golgin A8 family member B [Source:HGNC Symbol;Acc:HGNC:31973]	chr15:34817407-34828211	NM_001023567	XM_011521598, XM_0	NP_001018861	XP_011519896, XP_0115	protein_coding	ENSG0000021525	440270		31973	
988	GPR180	1.295	0.000	G protein-coupled receptor 180 [Source:HGNC Symbol;Acc:HGNC:28899]	chr13:95254156-95286899	NM_180989	.	NP_851320	.	protein_coding	ENSG0000015274	160897		28899	
989	KCTD17	-1.130	0.000	potassium channel tetramerization domain containing 17 [Source:HGNC Symbol;Acc:HG	chr22:37447775-37459430	NM_001282684, NM	XM_005261744, XM_0	NP_001269613, NP_00	XP_005261798, XP_0052	protein_coding	ENSG0000010037	79734		25705	
990	KIAA1147	1.105	0.000	KIAA1147 [Source:HGNC Symbol;Acc:HGNC:29472]	chr7:141356527-141401953	NM_001080392	.	NP_001073861	.	protein_coding	ENSG0000025709	57189		29472	
991	MCRIP1	-1.098	0.000	MAPK regulated corepressor interacting protein 1 [Source:HGNC Symbol;Acc:HGNC:280	chr17:79780236-79791178	NM_001093767, NM	XM_011523572, XM_0	NP_001087236, NP_00	XP_011521873, XP_0115	protein_coding	ENSG0000022566	348262		28007	
992	MOB3A	-1.223	0.000	MOB kinase activator 3A [Source:HGNC Symbol;Acc:HGNC:29802]	chr19:2071036-2096331	NM_130807	XM_011527683	NP_570719	XP_011525985	protein_coding	ENSG0000017208	126308		29802	
993	MPND	-1.300	0.000	MPN domain containing [Source:HGNC Symbol;Acc:HGNC:25934]	chr19:4343523-4360083	NM_001159846, NM	XM_006722926	NP_001153318, NP_00	XP_006722989	protein_coding	ENSG0000000838	84954		25934	
994	MVB12A	-1.094	0.000	multivesicular body subunit 12A [Source:HGNC Symbol;Acc:HGNC:25153]	chr19:17529889-17544533	NM_138401	.	NP_612410	.	protein_coding	ENSG0000014197	93343		25153	
995	NDUFB11	-1.275	0.000	NADH:ubiquinone oxidoreductase subunit B11 [Source:HGNC Symbol;Acc:HGNC:20372]	chrX:47001614-47046212	NM_001135998, NM	.	NP_001129470, NP_06	.	protein_coding	ENSG0000014712	54539		20372	
996	NLRC5	-1.307	0.000	NLR family CARD domain containing 5 [Source:HGNC Symbol;Acc:HGNC:29933]	chr16:57023409-57117436	NM_032206	XM_017023767, XM_0	NP_115582	XP_005256250, XP_0052	protein_coding	ENSG0000014085	84166		29933	
997	NPDC1	-1.027	0.000	neural proliferation, differentiation and control 1 [Source:HGNC Symbol;Acc:HGNC:789	chr9:139933921-139940655	NM_015392	.	NP_056207	.	protein_coding	ENSG0000010728	56654		7899	
998	NUP153	1.100	0.000	nucleoporin 153 [Source:HGNC Symbol;Acc:HGNC:8062]	chr6:17615265-17707065	NM_001278209, NM	XM_011515028, XM_0	NP_001265138, NP_00	XP_005249564, XP_0067	protein_coding	ENSG0000012478	9972		8062	
999	PDDC1	-1.017	0.000	Parkinson disease 7 domain containing 1 [Source:HGNC Symbol;Acc:HGNC:26616]	chr11:767221-777487	NM_001318818, NM	XM_005252898	NP_001305747, NP_00	XP_005252955, XP_0168	protein_coding	ENSG0000017722	347862		26616	
1000	PIKFYVE	1.227	0.000	phosphoinositide kinase, FYVE-type zinc finger containing [Source:HGNC Symbol;Acc:HG	chr2:209130990-209223475	NM_001178000, NM	XM_017003574, XM_0	NP_001171471, NP_05	XP_011509080, XP_0115	protein_coding	ENSG0000011502	200576		23785	
1001	PLAT	-1.173	0.000	plasminogen activator, tissue type [Source:HGNC Symbol;Acc:HGNC:9051]	chr8:42032750-42065242	NM_000930, NM_0	.	NP_000921, NP_00130	.	protein_coding	ENSG0000010436	5327		9051	
1002	PLCB4	1.321	0.000	phospholipase C beta 4 [Source:HGNC Symbol;Acc:HGNC:9059]	chr20:9049700-9461889	NM_000933, NM_0	XM_017027881, XM_0	NP_000924, NP_00116	XP_005260781, XP_0067	protein_coding	ENSG0000010133	5332		9059	
1003	PODXL2	-1.042	0.000	podocalyxin like 2 [Source:HGNC Symbol;Acc:HGNC:17936]	chr3:127348023-127391652	NM_015720	.	NP_056535	.	protein_coding	ENSG0000011463	50512		17936	
1004	PTPRU	-0.945	0.000	protein tyrosine phosphatase, receptor type U [Source:HGNC Symbol;Acc:HGNC:9683]	chr1:29563027-29653325	NM_001195001, NM	XM_006710272, XM_0	NP_001181930, NP_00	XP_006710332, XP_0168	protein_coding	ENSG0000006065	10076		9683	
1005	RASSF8	1.224	0.000	Ras association domain family member 8 [Source:HGNC Symbol;Acc:HGNC:13232]	chr12:26111961-26232825	NM_001164746, NM	XM_017018746, XM_0	NP_001158218, NP_00	XP_006719090, XP_0067	protein_coding	ENSG0000012309	11228		13232	
1006	RECQL	1.210	0.000	RecQ like helicase [Source:HGNC Symbol;Acc:HGNC:9948]	chr12:21590610-21654527	NM_002907, NM_0	XM_011520811, XM_0	NP_002898, NP_11655	XP_005253518, XP_0052	protein_coding	ENSG0000000470	5965		9948	
1007	SCN4B	-1.296	0.000	sodium voltage-gated channel beta subunit 4 [Source:HGNC Symbol;Acc:HGNC:10592]	chr11:118004091-118023603	NM_001142348, NM	.	NP_001135820, NP_00	.	protein_coding	ENSG0000017709	6330		10592	
1008	SDC3	-1.048	0.000	syndecan 3 [Source:HGNC Symbol;Acc:HGNC:10660]	chr1:31342313-31381608	NM_014654	.	NP_055469	XP_011540765, XP_0115	protein_coding	ENSG0000016251	9672		10660	
1009	SNAPC1	1.182	0.000	small nuclear RNA activating complex polypeptide 1 [Source:HGNC Symbol;Acc:HGNC:1	chr14:62229074-62263146	NM_003082	.	NP_003073	.	protein_coding	ENSG0000002360	6617		11134	
1010	TNRC6A	1.109	0.000	trinucleotide repeat containing 6A [Source:HGNC Symbol;Acc:HGNC:11969]	chr16:24741015-24837548	NM_014494	XM_017023143, XM_0	NP_055309	XP_005255311, XP_0052	protein_coding	ENSG0000009090	27327		11969	
1011	TRIM24	1.313	0.000	tripartite motif containing 24 [Source:HGNC Symbol;Acc:HGNC:11812]	chr7:138145078-138274738	NM_003852, NM_0	.	NP_003843, NP_05698	.	protein_coding	ENSG0000012277	8805		11812	
1012	UGT1A5	1.657	0.000	UDP glucuronosyltransferase family 1 member A5 [Source:HGNC Symbol;Acc:HGNC:125	chr2:234526290-234681956	NM_019078	.	NP_061951	.	protein_coding	ENSG0000024022	54579		12537	
1013	USP15	1.383	0.000	ubiquitin specific peptidase 15 [Source:HGNC Symbol;Acc:HGNC:12613]	chr12:62654118-62810169	NM_001252078, NM	XM_005269259	NP_001239007, NP_00	.	protein_coding	ENSG0000013565	102465133		12613	
1014	USP32	1.034	0.000	ubiquitin specific peptidase 32 [Source:HGNC Symbol;Acc:HGNC:19143]	chr17:58256454-58469495	NM_032582	XM_011525379, XM_0	NP_115971	XP_011523673, XP_0115	protein_coding	ENSG0000017083	84669		19143	
1015	USP8	1.204	0.000	ubiquitin specific peptidase 8 [Source:HGNC Symbol;Acc:HGNC:12631]	chr15:50716578-50838902	NM_001128610, NM	XM_011522194, XM_0	NP_001122082, NP_00	XP_006720824, XP_0067	protein_coding	ENSG0000013859	9101		12631	
1016	ZKSCAN5	1.109	0.000	zinc finger with KRAB and SCAN domains 5 [Source:HGNC Symbol;Acc:HGNC:12867]	chr7:99102273-99131445	NM_001318082, NM	XM_017011922, XM_0	NP_001305011, NP_05	XP_006715976, XP_0168	protein_coding	ENSG0000019665	23660		12867	
1017	AFF4	0.978	0.000	AF4/FMR2 family member 4 [Source:HGNC Symbol;Acc:HGNC:17869]	chr5:132211070-132299326	NM_014423	XM_006714587, XM_0	NP_055238	XP_005272020, XP_0067	protein_coding	ENSG0000007236	27125		17869	
1018	AGRN	-0.841	0.000	agrin [Source:HGNC Symbol;Acc:HGNC:329]	chr1:955502-991498	NM_001305275, NM	XM_011541430, XM_0	NP_001292204, NP_94	XP_005244806, XP_0115	protein_coding	ENSG0000018815	375790		329	
1019	ALG8	1.300	0.000	ALG8, alpha-1,3-glucosyltransferase [Source:HGNC Symbol;Acc:HGNC:23161]	chr11:77811987-77850706	NM_001007027, NM	XM_011545252, XM_0	NP_001007028, NP_07	XP_005274304, XP_0115	protein_coding	ENSG0000015906	79053		23161	
1020	ARL4A	1.097	0.000	ADP ribosylation factor like GTPase 4A [Source:HGNC Symbol;Acc:HGNC:695]	chr7:12726480-12730559	NM_001037164, NM	.	NP_001032241, NP_00	.	protein_coding	ENSG0000012264	10124		695	
1021	ATG4A	1.247	0.000	autophagy related 4A cysteine peptidase [Source:HGNC Symbol;Acc:HGNC:16489]	chrX:107334897-107397901	NM_001321288, NM	XM_011530845, XM_0	NP_001308217, NP_00	XP_011529143, XP_0115	protein_coding	ENSG0000010184	115201		16489	
1022	C12orf66	1.351	0.000	chromosome 12 open reading frame 66 [Source:HGNC Symbol;Acc:HGNC:26517]	chr12:64580095-64616076	NM_001300940, NM	.	NP_001287869, NP_00	.	protein_coding	ENSG0000017420	144577		26517	
1023	CLIP1	1.241	0.000	CAP-Gly domain containing linker protein 1 [Source:HGNC Symbol;Acc:HGNC:10461]	chr12:122755978-122907179	NM_001247997, NM	XM_017019786, XM_0	NP_001234926, NP_00	XP_005253650, XP_0067	protein_coding	ENSG0000013077	6249		10461	
1024	COBL	0.871	0.000	cordon-bleu WH2 repeat protein [Source:HGNC Symbol;Acc:HGNC:22199]	chr7:51083908-51384515	NM_001287436, NM	XM_017011901, XM_0	NP_001274365, NP_00	XP_005271807, XP_0052	protein_coding	ENSG0000010607	23242		22199	

1025	COQ10B	1.197	0.000	coenzyme Q10B [Source:HGNC Symbol;Acc:HGNC:25819]	chr2:198318146-198340032	NM_025147	XM_006712776	NP_001307747, NP_00	XP_006712839	protein_coding	ENSG0000011552	80219	25819
1026	CUL2	1.317	0.000	cullin 2 [Source:HGNC Symbol;Acc:HGNC:2552]	chr10:35297478-35379570	NM_001198777, N	XM_011519747, XM_0	NP_001185706, NP_00	XP_011518045, XP_0115	protein_coding	ENSG0000010809	8453	2552
1027	CUL4B	1.054	0.000	cullin 4B [Source:HGNC Symbol;Acc:HGNC:2555]	chrX:119658463-119709649	NM_001079872, N	XM_011531400, XM_0	NP_001073341, NP_00	XP_005262538, XP_0067	protein_coding	ENSG0000015829	8450	2555
1028	DBF4	1.162	0.000	DBF4 zinc finger [Source:HGNC Symbol;Acc:HGNC:17364]	chr7:87462882-87538856	NM_006716	XM_011515764, XM_0	NP_006707	.	protein_coding	ENSG0000000663	10926	17364
1029	DDX46	1.009	0.000	DEAD-box helicase 46 [Source:HGNC Symbol;Acc:HGNC:18681]	chr5:134094460-134166828	NM_001300860, N	.	NP_001287789, NP_05	.	protein_coding	ENSG0000014583	9879	18681
1030	DGKE	1.439	0.000	diacylglycerol kinase epsilon [Source:HGNC Symbol;Acc:HGNC:2852]	chr17:54911459-54946928	NM_003647	XM_011525398, XM_0	NP_003638	XP_011523696	protein_coding	ENSG0000015393	8526	2852
1031	DOCK4	1.363	0.000	dedicator of cytokinesis 4 [Source:HGNC Symbol;Acc:HGNC:19192]	chr7:111366533-111846466	NM_014705	XM_017012822, XM_0	NP_055520	XP_006716251, XP_0067	protein_coding	ENSG0000012851	9732	19192
1032	DVL1	-0.876	0.000	dishevelled segment polarity protein 1 [Source:HGNC Symbol;Acc:HGNC:3084]	chr1:1266693-1284730	NM_004421	XM_005244733, XM_0	NP_004412	XP_005244788, XP_0052	protein_coding	ENSG0000010740	102466740	3084
1033	EIF5B	0.943	0.000	eukaryotic translation initiation factor 5B [Source:HGNC Symbol;Acc:HGNC:30793]	chr2:99953815-100106497	NM_015904	.	NP_056988	.	protein_coding	ENSG0000015841	9669	30793
1034	G2E3	1.564	0.000	G2/M-phase specific E3 ubiquitin protein ligase [Source:HGNC Symbol;Acc:HGNC:20338	chr14:31028328-31089269	NM_001308097, N	XM_011536927, XM_0	NP_001295026, NP_06	XP_011535227, XP_0115	protein_coding	ENSG0000009214	55632	20338
1035	HIPK3	1.314	0.000	homeodomain interacting protein kinase 3 [Source:HGNC Symbol;Acc:HGNC:4915]	chr11:33278217-33378569	NM_001048200, N	XM_017017077, XM_0	NP_001041665, NP_00	XP_005252786, XP_0115	protein_coding	ENSG0000011042	10114	4915
1036	HIST1H4B	-Inf	0.000	histone cluster 1, H4b [Source:HGNC Symbol;Acc:HGNC:4789]	chr6:26027123-26027480	NM_003544	.	NP_003535	.	protein_coding	ENSG0000012452	8366	4789
1037	HSDL2	1.077	0.000	hydroxysteroid dehydrogenase like 2 [Source:HGNC Symbol;Acc:HGNC:18572]	chr9:115142216-115234690	NM_001195822, N	.	NP_001182751, NP_11	.	protein_coding	ENSG0000011947	84263	18572
1038	JRKL	1.296	0.000	JRK-like [Source:HGNC Symbol;Acc:HGNC:6200]	chr11:96123157-96126421	NM_001261833, N	.	NP_001248762, NP_00	.	protein_coding	ENSG0000018334	8690	6200
1039	MAP3K7	1.154	0.000	mitogen-activated protein kinase kinase kinase 7 [Source:HGNC Symbol;Acc:HGNC:6859	chr6:91223291-91296764	NM_003188, NM_1	XM_017011226, XM_0	NP_003179, NP_66330	XP_006715616, XP_0168	protein_coding	ENSG0000013534	6885	6859
1040	MFSD3	-1.198	0.000	major facilitator superfamily domain containing 3 [Source:HGNC Symbol;Acc:HGNC:251	chr8:145734456-145736596	NM_138431	XM_011516806	NP_612440	XP_011515108	protein_coding	ENSG0000016770	113655	25157
1041	MOB2	-1.095	0.000	MOB kinase activator 2 [Source:HGNC Symbol;Acc:HGNC:24904]	chr11:1490687-1507976	NM_001172223, N	.	NP_001165694, NP_44	.	protein_coding	ENSG0000018220	81532	24904
1042	NFKBIE	-1.004	0.000	NFKB inhibitor epsilon [Source:HGNC Symbol;Acc:HGNC:7799]	chr6:44225902-44233525	NM_004556	.	NP_004547	.	protein_coding	ENSG0000014623	4794	7799
1043	NGEF	-1.344	0.000	neuronal guanine nucleotide exchange factor [Source:HGNC Symbol;Acc:HGNC:7807]	chr2:233743395-233877982	NM_001114090, N	XM_011510923	NP_001107562, NP_06	XP_011509225	protein_coding	ENSG0000006624	25791	7807
1044	NKX2-8	-1.493	0.000	NK2 homeobox 8 [Source:HGNC Symbol;Acc:HGNC:16364]	chr14:37049783-37051812	NM_014360	.	NP_055175	.	protein_coding	ENSG0000013632	26257	16364
1045	PCBP4	-1.050	0.000	poly(rC) binding protein 4 [Source:HGNC Symbol;Acc:HGNC:8652]	chr3:51989329-52002426	NM_001174100, N	XM_017006919, XM_0	NP_001167571, NP_06	XP_005265387, XP_0052	protein_coding	ENSG0000009009	57060	8652
1046	PDLIM4	-0.912	0.000	PDZ and LIM domain 4 [Source:HGNC Symbol;Acc:HGNC:16501]	chr5:131593363-131609147	NM_001131027, N	.	NP_001124499, NP_00	.	protein_coding	ENSG0000013143	8572	16501
1047	PGAP1	1.499	0.000	post-GPI attachment to proteins 1 [Source:HGNC Symbol;Acc:HGNC:25712]	chr2:197697727-197792519	NM_001321099, N	XM_017004993, XM_0	NP_001308028, NP_07	XP_011510182, XP_0168	protein_coding	ENSG0000019712	80055	25712
1048	PIGW	1.221	0.000	phosphatidylinositol glycan anchor biosynthesis class W [Source:HGNC Symbol;Acc:HGN	chr17:34890868-34895159	NM_178517	XM_011524646, XM_0	NP_848612	XP_005257295, XP_0115	protein_coding	ENSG0000027716	284098	23213
1049	PKN1	-0.871	0.000	protein kinase N1 [Source:HGNC Symbol;Acc:HGNC:9405]	chr19:14544168-14582678	NM_002741, NM_2	XM_011528129, XM_0	NP_002732, NP_99872	XP_011526430	protein_coding	ENSG0000012314	5585	9405
1050	PNISR	1.297	0.000	PNN interacting serine and arginine rich protein [Source:HGNC Symbol;Acc:HGNC:21222	chr6:99845926-99873207	NM_001322405, N	XM_005266917, XM_0	NP_001309334, NP_00	XP_005266969, XP_0052	protein_coding	ENSG0000013242	25957	21222
1051	PRR36	-1.376	0.000	proline rich 36 [Source:HGNC Symbol;Acc:HGNC:26172]	chr19:7933604-7939264	NM_001190467	.	NP_001177396	.	protein_coding	ENSG0000018324	80164	26172
1052	PUM2	1.039	0.000	pumilio RNA binding family member 2 [Source:HGNC Symbol;Acc:HGNC:14958]	chr2:20448451-20550611	NM_001282752, N	XM_017003714, XM_0	NP_001269681, NP_00	XP_005262664, XP_0052	protein_coding	ENSG0000005591	23369	14958
1053	RCC2	-0.894	0.000	regulator of chromosome condensation 2 [Source:HGNC Symbol;Acc:HGNC:30297]	chr1:17733255-17766220	NM_001136204, N	.	NP_001129676, NP_06	.	protein_coding	ENSG0000017905	55920	30297
1054	SEPT10	1.036	0.000	septin 10 [Source:HGNC Symbol;Acc:HGNC:14349]	chr2:110300558-110371783	NM_001321498, N	XM_011510704, XM_0	NP_001308427, NP_00	XP_006712380, XP_0115	protein_coding	ENSG0000018652	151011	14349
1055	SGK3	1.724	0.000	serum/glucocorticoid regulated kinase family member 3 [Source:HGNC Symbol;Acc:HGN	chr8:67579850-67774257	NM_001033578, N	.	NP_001028750, NP_03	.	protein_coding	ENSG0000010420	23678	10812
1056	SHF	-1.181	0.000	Src homology 2 domain containing F [Source:HGNC Symbol;Acc:HGNC:25116]	chr15:45459411-45493373	NM_001301168, N	XM_011522180, XM_0	NP_001288097, NP_00	XP_006720805, XP_0115	protein_coding	ENSG0000013860	90525	25116
1057	SMAD5	1.079	0.000	SMAD family member 5 [Source:HGNC Symbol;Acc:HGNC:6771]	chr5:135468533-135518422	NM_001001419, N	XM_017009470	NP_001001419, NP_00	XP_016864959	protein_coding	ENSG0000011365	4090	6771
1058	THAP12	0.965	0.000	THAP domain containing 12 [Source:HGNC Symbol;Acc:HGNC:9440]	chr11:76060999-76091986	NM_004705	.	NP_004696	.	protein_coding	ENSG0000013749	5612	9440
1059	TIMM17B	-1.484	0.000	translocase of inner mitochondrial membrane 17 homolog B (yeast) [Source:HGNC Sym	chrX:48750729-48760419	NM_001167947, N	XM_011543886	NP_001161419, NP_00	XP_011542188	protein_coding	ENSG0000012676	10245	17310
1060	TXNIP	1.022	0.000	thioredoxin interacting protein [Source:HGNC Symbol;Acc:HGNC:16952]	chr1:145438468-145442635	NM_006472	.	NP_001300901, NP_00	XP_016855574	protein_coding	ENSG0000026597	10628	16952
1061	UBLCP1	1.243	0.000	ubiquitin like domain containing CTD phosphatase 1 [Source:HGNC Symbol;Acc:HGNC:2	chr5:158690088-158713048	NM_145049	.	NP_659486	.	protein_coding	ENSG0000016433	134510	28110
1062	ZFC3H1	1.446	0.000	zinc finger C3H1-type containing [Source:HGNC Symbol;Acc:HGNC:28328]	chr12:72003251-72074419	NM_144982	.	NP_659419	.	protein_coding	ENSG0000013385	196441	28328
1063	ZFR	1.201	0.000	zinc finger RNA binding protein [Source:HGNC Symbol;Acc:HGNC:17277]	chr5:32354455-32444867	NM_016107	.	NP_057191	.	protein_coding	ENSG0000005609	51663	17277
1064	ZFYVE16	1.388	0.000	zinc finger FYVE-type containing 16 [Source:HGNC Symbol;Acc:HGNC:20756]	chr5:79703831-79775169	NM_001105251, N	XM_017010092, XM_0	NP_001098721, NP_00	XP_005248689, XP_0115	protein_coding	ENSG0000003931	9765	20756
1065	ZNF26	1.503	0.000	zinc finger protein 26 [Source:HGNC Symbol;Acc:HGNC:13053]	chr12:133563049-133603689	NM_001256279, N	XM_011534830, XM_0	NP_001243208, NP_06	XP_005266239, XP_0052	protein_coding	ENSG0000019839	7574	13053
1066	ASUN	0.984	0.000	asunder, spermatogenesis regulator [Source:HGNC Symbol;Acc:HGNC:20174]	chr12:27058113-27091254	NM_018164	XM_011520752, XM_0	NP_060634	XP_005253479, XP_0115	protein_coding	ENSG0000006410	55726	20174
1067	CDS1	1.258	0.000	CDP-diacylglycerol synthase 1 [Source:HGNC Symbol;Acc:HGNC:1800]	chr4:85504131-85572491	NM_001263	XM_005262687	NP_001254	XP_005262744, XP_0168	protein_coding	ENSG0000016362	1040	1800
1068	CHI3L1	-1.383	0.000	chitinase 3 like 1 [Source:HGNC Symbol;Acc:HGNC:1932]	chr1:203148058-203155877	NM_001276	XM_011509108, XM_0	NP_001267	.	protein_coding	ENSG0000013304	1116	1932
1069	COL18A1	-0.907	0.000	collagen type XVIII alpha 1 chain [Source:HGNC Symbol;Acc:HGNC:2195]	chr21:46825051-46964325	NM_030582, NM_1	.	NP_085059, NP_56971	.	protein_coding	ENSG0000018287	80781	2195
1070	DNA2	1.409	0.000	DNA replication helicase/nuclease 2 [Source:HGNC Symbol;Acc:HGNC:2939]	chr10:70173820-70231878	NM_001080449	XM_006717680	NP_001073918	XP_006717743	protein_coding	ENSG0000013834	1763	2939
1071	EFCAB14	0.974	0.000	EF-hand calcium binding domain 14 [Source:HGNC Symbol;Acc:HGNC:29051]	chr1:47140830-47184736	NM_014774	.	NP_055589	.	protein_coding	ENSG0000015965	9813	29051
1072	FAM109A	-1.218	0.000	family with sequence similarity 109 member A [Source:HGNC Symbol;Acc:HGNC:26509]	chr12:111798454-111806925	NM_001177996, N	XM_011537976, XM_0	NP_001171467, NP_00	XP_006719320, XP_0115	protein_coding	ENSG0000019832	144717	26509
1073	FAM46A	1.488	0.000	family with sequence similarity 46 member A [Source:HGNC Symbol;Acc:HGNC:18345]	chr6:82455446-82462491	NM_017633	.	NP_060103	.	protein_coding	ENSG0000011277	55603	18345
1074	FANCM	1.891	0.000	Fanconi anemia complementation group M [Source:HGNC Symbol;Acc:HGNC:23168]	chr14:45605142-45670090	NM_001308133, N	XM_011537036, XM_0	NP_001295062, NP_00	XP_005267986, XP_0115	protein_coding	ENSG0000018779	57697	23168
1075	FASTKD3	1.293	0.000	FAST kinase domains 3 [Source:HGNC Symbol;Acc:HGNC:28758]	chr5:7859271-7869115	NM_024091	XM_006714498	NP_076996	XP_006714561	protein_coding	ENSG0000012427	79072	28758
1076	G0S2	-1.316	0.000	G0/G1 switch 2 [Source:HGNC Symbol;Acc:HGNC:30229]	chr1:209848764-209849733	NM_015714	.	NP_056529	.	protein_coding	ENSG0000012368	50486	30229
1077	GRIPAP1	-1.076	0.000	GRIP1 associated protein 1 [Source:HGNC Symbol;Acc:HGNC:18706]	chrX:48830133-48858675	NM_020137	XM_017029659, XM_0	NP_064522	XP_011542237, XP_0115	protein_coding	ENSG0000006840	56850	18706
1078	HERPUD2	1.163	0.000	HERPUD family member 2 [Source:HGNC Symbol;Acc:HGNC:21915]	chr7:35672268-35734745	NM_022373	XM_017012506	NP_071768	XP_016867995	protein_coding	ENSG0000012255	64224	21915

1079	HSPA5	0.868	0.000	heat shock protein family A (Hsp70) member 5 [Source:HGNC Symbol;Acc:HGNC:5238]	chr9:127997131-128003609	NM_005347	.	NP_005338	.	protein_coding	ENSG0000004457	3309	5238
1080	IL17C	-2.742	0.000	interleukin 17C [Source:HGNC Symbol;Acc:HGNC:5983]	chr16:88705000-88706881	NM_013278	.	NP_037410	.	protein_coding	ENSG0000012439	27189	5983
1081	INIP	1.161	0.000	INTS3 and NABP interacting protein [Source:HGNC Symbol;Acc:HGNC:24994]	chr9:115446205-115480516	NM_001329585, NM	.	NP_001316514, NP_00	.	protein_coding	ENSG0000014815	58493	24994
1082	IP6K1	-1.000	0.000	inositol hexakisphosphate kinase 1 [Source:HGNC Symbol;Acc:HGNC:18360]	chr3:49761726-49823975	NM_001006115, NM	.	NP_001006115, NP_00	.	protein_coding	ENSG0000017609	9807	18360
1083	KIAA1429	1.138	0.000	KIAA1429 [Source:HGNC Symbol;Acc:HGNC:24500]	chr8:95499920-95565757	NM_015496, NM_1	.	NP_056311, NP_89212	.	protein_coding	ENSG0000016494	25962	24500
1084	MACF1	1.076	0.000	microtubule-actin crosslinking factor 1 [Source:HGNC Symbol;Acc:HGNC:13664]	chr1:39546987-39952849	NM_012090, NM_0	XM_017000878, XM_0	NP_036222, NP_05585	XP_005270751, XP_0052	protein_coding	ENSG0000012760	643314	13664
1085	MAP7D3	1.167	0.000	MAP7 domain containing 3 [Source:HGNC Symbol;Acc:HGNC:25742]	chrX:135295378-135338641	NM_001173516, NM	XM_005262472	NP_001166987, NP_00	XP_005262529, XP_0168	protein_coding	ENSG0000012968	79649	25742
1086	MMS22L	1.518	0.000	MMS22 like, DNA repair protein [Source:HGNC Symbol;Acc:HGNC:21475]	chr6:97590036-97731052	NM_198468	XM_011535675, XM_0	NP_940870	XP_006715493, XP_0115	protein_coding	ENSG0000014626	253714	21475
1087	NCKIPSD	-1.064	0.000	NCK interacting protein with SH3 domain [Source:HGNC Symbol;Acc:HGNC:15486]	chr3:48711276-48723360	NM_016453, NM_1	XM_017006600	NP_057537, NP_90911	XP_016862089	protein_coding	ENSG0000021367	51517	15486
1088	OPA1	1.050	0.000	OPA1, mitochondrial dynamin like GTPase [Source:HGNC Symbol;Acc:HGNC:8140]	chr3:193310932-193415612	NM_015560, NM_1	XM_017006500, XM_0	NP_056375, NP_57084	XP_016861989	protein_coding	ENSG0000019883	4976	8140
1089	PCF11	1.104	0.000	PCF11 cleavage and polyadenylation factor subunit [Source:HGNC Symbol;Acc:HGNC:30	chr11:82868029-82898493	NM_015885	XM_005274049, XM_0	NP_056969	XP_005274105, XP_0052	protein_coding	ENSG0000016549	51585	30097
1090	PHLDA1	0.884	0.000	pleckstrin homology like domain family A member 1 [Source:HGNC Symbol;Acc:HGNC:8	chr12:76419226-76427712	NM_007350	.	NP_031376	.	protein_coding	ENSG0000013928	22822	8933
1091	PLXNB1	-0.999	0.000	plexin B1 [Source:HGNC Symbol;Acc:HGNC:9103]	chr3:48445260-48471460	NM_001130082, NM	XM_017006630, XM_0	NP_001123554, NP_00	XP_011532135, XP_0115	protein_coding	ENSG0000016405	5364	9103
1092	RAB39A	2.292	0.000	RAB39A, member RAS oncogene family [Source:HGNC Symbol;Acc:HGNC:16521]	chr11:107799228-107834208	NM_017516	.	NP_059986	.	protein_coding	ENSG0000017933	54734	16521
1093	RASL10A	-1.655	0.000	RAS like family 10 member A [Source:HGNC Symbol;Acc:HGNC:16954]	chr22:29708921-29711745	NM_006477	.	NP_006468	.	protein_coding	ENSG0000010027	10633	16954
1094	RPS4X	-0.883	0.000	ribosomal protein S4, X-linked [Source:HGNC Symbol;Acc:HGNC:10424]	chrX:71491891-71497150	NM_001007	.	NP_000998	.	protein_coding	ENSG0000019803	6191	10424
1095	SBNO1	1.228	0.000	strawberry notch homolog 1 [Source:HGNC Symbol;Acc:HGNC:22973]	chr12:123773655-123849390	NM_001167856, NM	XM_017019559, XM_0	NP_001161328, NP_06	XP_005253629, XP_0052	protein_coding	ENSG0000013969	102466877	22973
1096	SERAC1	1.295	0.000	serine active site containing 1 [Source:HGNC Symbol;Acc:HGNC:21061]	chr6:158530546-158589302	NM_032861	XM_011536198, XM_0	NP_116250	XP_006715649, XP_0115	protein_coding	ENSG0000012233	84947	21061
1097	SLC25A20	-1.262	0.000	solute carrier family 25 member 20 [Source:HGNC Symbol;Acc:HGNC:1421]	chr3:48894363-48936426	NM_000387	.	NP_000378	.	protein_coding	ENSG0000017853	788	1421
1098	SLC30A3	-1.272	0.000	solute carrier family 30 member 3 [Source:HGNC Symbol;Acc:HGNC:11014]	chr2:27477439-27486127	NM_003459	XM_005264547	NP_001305878, NP_00	XP_005264604	protein_coding	ENSG0000011519	7781	11014
1099	TAGLN3	-1.801	0.000	transgelin 3 [Source:HGNC Symbol;Acc:HGNC:29868]	chr3:111717510-111732734	NM_001008272, NM	.	NP_001008273, NP_00	.	protein_coding	ENSG0000014483	29114	29868
1100	TARS	0.903	0.000	threonyl-tRNA synthetase [Source:HGNC Symbol;Acc:HGNC:11572]	chr5:33440801-33469644	NM_001258437, NM	.	NP_001245366, NP_00	.	protein_coding	ENSG0000011340	6897	11572
1101	TEX264	-1.030	0.000	testis expressed 264 [Source:HGNC Symbol;Acc:HGNC:30247]	chr3:51705190-51738339	NM_001129884, NM	XM_017006573, XM_0	NP_001123356, NP_00	XP_006713258, XP_0067	protein_coding	ENSG0000016408	51368	30247
1102	USPL1	1.304	0.000	ubiquitin specific peptidase like 1 [Source:HGNC Symbol;Acc:HGNC:20294]	chr13:31191919-31234907	NM_001321533, NM	XM_006719751, XM_0	NP_001308462, NP_00	XP_005266270, XP_0067	protein_coding	ENSG0000013295	10208	20294
1103	ZNF318	1.050	0.000	zinc finger protein 318 [Source:HGNC Symbol;Acc:HGNC:13578]	chr6:43303807-43337181	NM_014345	.	NP_055160	XP_011512753, XP_0115	protein_coding	ENSG0000017146	24149	13578
1104	ADGRB1	-1.641	0.000	adhesion G protein-coupled receptor B1 [Source:HGNC Symbol;Acc:HGNC:943]	chr8:143530790-143626370	NM_001702	XM_011517201, XM_0	NP_001693	XP_011515501	protein_coding	ENSG0000018179	575	943
1105	APC	1.371	0.000	APC, WNT signaling pathway regulator [Source:HGNC Symbol;Acc:HGNC:583]	chr5:112073584-112181936	NM_000038, NM_0	.	NP_000029, NP_00112	.	protein_coding	ENSG0000013498	324	583
1106	ATAD5	1.445	0.000	ATPase family, AAA domain containing 5 [Source:HGNC Symbol;Acc:HGNC:25752]	chr17:29158987-29222887	NM_024857	XM_011525270, XM_0	NP_079133	XP_011523571, XP_0115	protein_coding	ENSG0000017620	79915	25752
1107	C1orf27	1.365	0.000	chromosome 1 open reading frame 27 [Source:HGNC Symbol;Acc:HGNC:24299]	chr1:186344969-186390510	NM_001164245, NM	XM_011509661, XM_0	NP_001157717, NP_00	XP_011507962, XP_0115	protein_coding	ENSG0000015718	54953	24299
1108	CAPRIN1	0.890	0.000	cell cycle associated protein 1 [Source:HGNC Symbol;Acc:HGNC:6743]	chr11:34073229-34122703	NM_005898, NM_2	XM_017017749	NP_005889, NP_97624	XP_016873238	protein_coding	ENSG0000013538	4076	6743
1109	CENPJ	1.156	0.000	centromere protein J [Source:HGNC Symbol;Acc:HGNC:17272]	chr13:25456421-25497027	NM_018451	XM_011535149	NP_060921	XP_011533451, XP_0115	protein_coding	ENSG0000015184	55835	17272
1110	CHCHD6	-1.125	0.000	coiled-coil-helix-coiled-coil-helix domain containing 6 [Source:HGNC Symbol;Acc:HGNC:	chr3:126423062-126679249	NM_001320610, NM	XM_011513234, XM_0	NP_001307539, NP_11	XP_011511536	protein_coding	ENSG0000015968	84303	28184
1111	CYTH4	-1.358	0.000	cytohesin 4 [Source:HGNC Symbol;Acc:HGNC:9505]	chr22:37678423-37711386	NM_001318024, NM	XM_011530148, XM_0	NP_001304953, NP_03	XP_011528449, XP_0115	protein_coding	ENSG0000010005	27128	9505
1112	DAG1	-0.860	0.000	dystroglycan 1 [Source:HGNC Symbol;Acc:HGNC:2666]	chr3:49506135-49573051	NM_001165928, NM	.	NP_001159400, NP_00	.	protein_coding	ENSG0000017340	1605	2666
1113	DMXL1	1.259	0.000	Dmx like 1 [Source:HGNC Symbol;Acc:HGNC:2937]	chr5:118407083-118584822	NM_001290321, NM	XM_017009142, XM_0	NP_001277250, NP_00	XP_005271966, XP_0052	protein_coding	ENSG0000017286	1657	2937
1114	EFNA1	-1.034	0.000	ephrin A1 [Source:HGNC Symbol;Acc:HGNC:3221]	chr1:155099935-155107333	NM_004428, NM_1	.	NP_004419, NP_87262	.	protein_coding	ENSG0000016924	1942	3221
1115	ESCO2	1.678	0.000	establishment of sister chromatid cohesion N-acetyltransferase 2 [Source:HGNC Symbol	chr8:27631902-27665745	NM_001017420	XM_011544422, XM_0	NP_001017420	XP_011542723, XP_0115	protein_coding	ENSG0000017132	157570	27230
1116	FKTN	1.301	0.000	fukutin [Source:HGNC Symbol;Acc:HGNC:3622]	chr9:108320410-108403399	NM_001079802, NM	XM_017014479, XM_0	NP_001073270, NP_00	XP_016869953, XP_0168	protein_coding	ENSG0000010669	2218	3622
1117	GGNBP2	0.990	0.000	gametogenetin binding protein 2 [Source:HGNC Symbol;Acc:HGNC:19357]	chr17:34900736-34946278	NM_024835	XM_011525266, XM_0	NP_079111	XP_005257746, XP_0115	protein_coding	ENSG0000027831	79893	19357
1118	GSDMD	-0.973	0.000	gasdermin D [Source:HGNC Symbol;Acc:HGNC:25697]	chr8:144635376-144645232	NM_001166237, NM	XM_011517301	NP_001159709, NP_07	XP_011515603	protein_coding	ENSG0000010451	79792	25697
1119	IGBP1	-0.915	0.000	immunoglobulin (CD79A) binding protein 1 [Source:HGNC Symbol;Acc:HGNC:5461]	chrX:69353298-69386174	NM_001551	XM_017029489	NP_001542	XP_016884978	protein_coding	ENSG0000008928	3476	5461
1120	IL34	-2.038	0.000	interleukin 34 [Source:HGNC Symbol;Acc:HGNC:28529]	chr16:70613797-70694585	NM_001172771, NM	XM_011522903, XM_0	NP_001166242, NP_00	XP_011521204, XP_0115	protein_coding	ENSG0000015736	146433	28529
1121	ING4	-1.099	0.000	inhibitor of growth family member 4 [Source:HGNC Symbol;Acc:HGNC:19423]	chr12:6759445-6772314	NM_001127582, NM	XM_011520965, XM_0	NP_001121054, NP_00	XP_005253755, XP_0115	protein_coding	ENSG0000011165	51147	19423
1122	IRAK4	1.215	0.000	interleukin 1 receptor associated kinase 4 [Source:HGNC Symbol;Acc:HGNC:17967]	chr12:44152746-44183346	NM_001114182, NM	XM_017019391, XM_0	NP_001107654, NP_00	XP_005269000, XP_0052	protein_coding	ENSG0000019800	51135	17967
1123	LACE1	1.622	0.000	lactation elevated 1 [Source:HGNC Symbol;Acc:HGNC:16411]	chr6:108616097-108847204	NM_145315	XM_011535658, XM_0	NP_660358	XP_005266942, XP_0115	protein_coding	ENSG0000013553	246269	16411
1124	LHPP	-1.274	0.000	phospholysine phosphohistidine inorganic pyrophosphate phosphatase [Source:HGNC S	chr10:126150402-126302710	NM_001167880, NM	XM_006717943, XM_0	NP_001161352, NP_00	XP_005270084, XP_0067	protein_coding	ENSG0000010790	64077	30042
1125	MED12	-1.073	0.000	mediator complex subunit 12 [Source:HGNC Symbol;Acc:HGNC:11957]	chrX:70338405-70362304	NM_005120	XM_005262319, XM_0	NP_005111	.	protein_coding	ENSG0000018463	9968	11957
1126	MGAT3	-1.167	0.000	mannosyl (beta-1,4-)-glycoprotein beta-1,4-N-acetylglucosaminyltransferase [Source:H	chr22:39853348-39888199	NM_001098270, NM	XM_011530194, XM_0	NP_001091740, NP_00	XP_005261665, XP_0052	protein_coding	ENSG0000012826	4248	7046
1127	NEBL	1.148	0.000	nebulette [Source:HGNC Symbol;Acc:HGNC:16932]	chr10:21068901-21463116	NM_001173484, NM	XM_017015468, XM_0	NP_001166955, NP_00	XP_005252399, XP_0052	protein_coding	ENSG0000007811	10529	16932
1128	NFXL1	1.322	0.000	nuclear transcription factor, X-box binding like 1 [Source:HGNC Symbol;Acc:HGNC:1872	chr4:47849249-47916684	NM_001278623, NM	.	NP_001265552, NP_00	.	protein_coding	ENSG0000017044	152518	18726
1129	NIPAL1	1.480	0.000	NIPA like domain containing 1 [Source:HGNC Symbol;Acc:HGNC:27194]	chr4:48018790-48042190	NM_207330	XM_017007784	NP_997213	XP_016863273	protein_coding	ENSG0000016329	152519	27194
1130	NUP58	0.980	0.000	nucleoporin 58 [Source:HGNC Symbol;Acc:HGNC:20261]	chr13:25875661-25916554	NM_001008564, NM	XM_011535330, XM_0	NP_001008564, NP_05	XP_005266657, XP_0115	protein_coding	ENSG0000013949	9818	20261
1131	NXPH3	-1.448	0.000	neurexophilin 3 [Source:HGNC Symbol;Acc:HGNC:8077]	chr17:47653219-47659652	NM_007225	.	NP_009156	.	protein_coding	ENSG0000018257	11248	8077
1132	PGC	-2.004	0.000	progastricsin [Source:HGNC Symbol;Acc:HGNC:8890]	chr6:41704448-41715139	NM_001166424, NM	.	NP_001159896, NP_00	.	protein_coding	ENSG0000009608	5225	8890

1133	PIM2	-1.029	0.000	Pim-2 proto-oncogene, serine/threonine kinase [Source:HGNC Symbol;Acc:HGNC:8987]	chrX:48770458-48776301	NM_006875	XM_011543887	NP_006866	.	protein_coding	ENSG0000010209	11040	8987
1134	PLD3	-0.909	0.000	phospholipase D family member 3 [Source:HGNC Symbol;Acc:HGNC:17158]	chr19:40854584-40884390	NM_001031696, NM	XM_017026547, XM_0	NP_001026866, NP_00	XP_005258761, XP_0052	protein_coding	ENSG0000010522	23646	17158
1135	PNPLA7	-1.071	0.000	patatin like phospholipase domain containing 7 [Source:HGNC Symbol;Acc:HGNC:24768	chr9:140354404-140444986	NM_001098537, NM	XM_017014709, XM_0	NP_001092007, NP_68	XP_006717165, XP_0067	protein_coding	ENSG0000013065	375775	24768
1136	PSMD12	0.921	0.000	proteasome 26S subunit, non-ATPase 12 [Source:HGNC Symbol;Acc:HGNC:9557]	chr17:65334031-65362743	NM_002816, NM_1	XM_011525049, XM_0	NP_001303270, NP_00	.	protein_coding	ENSG0000019717	5718	9557
1137	PTAR1	1.626	0.000	protein prenyltransferase alpha subunit repeat containing 1 [Source:HGNC Symbol;Acc:	chr9:72324437-72374875	NM_001099666	XM_011518640, XM_0	NP_001093136	XP_005252033, XP_0052	protein_coding	ENSG0000018864	375743	30449
1138	RBM3	-1.012	0.000	RNA binding motif (RNP1, RRM) protein 3 [Source:HGNC Symbol;Acc:HGNC:9900]	chrX:48432911-48437454	NM_006743	XM_011543938	NP_006734	.	protein_coding	ENSG0000010231	5935	9900
1139	RND3	1.151	0.000	Rho family GTPase 3 [Source:HGNC Symbol;Acc:HGNC:671]	chr2:151324708-151344208	NM_001254738, NM	.	NP_001241667, NP_00	.	protein_coding	ENSG0000011596	390	671
1140	RPL29	-0.818	0.000	ribosomal protein L29 [Source:HGNC Symbol;Acc:HGNC:10331]	chr3:52027615-52029958	NM_000992	.	NP_000983	.	protein_coding	ENSG0000016224	6159	10331
1141	RTP3	-2.294	0.000	receptor transporter protein 3 [Source:HGNC Symbol;Acc:HGNC:15572]	chr3:46538980-46542439	NM_031440	.	NP_113628	.	protein_coding	ENSG0000016382	83597	15572
1142	SH3PXD2A	-1.260	0.000	SH3 and PX domains 2A [Source:HGNC Symbol;Acc:HGNC:23664]	chr10:105353783-105615301	NM_014631	XM_017016970, XM_0	NP_055446	XP_005270351, XP_0052	protein_coding	ENSG0000010795	9644	23664
1143	SLC35B3	1.456	0.000	solute carrier family 35 member B3 [Source:HGNC Symbol;Acc:HGNC:21601]	chr6:8413300-8435716	NM_001142541, NM	XM_011514648, XM_0	NP_001136013, NP_05	XP_005249213, XP_0052	protein_coding	ENSG0000012478	51000	21601
1144	SLC44A3	1.511	0.000	solute carrier family 44 member 3 [Source:HGNC Symbol;Acc:HGNC:28689]	chr1:95285897-95360802	NM_001114106, NM	XM_017000262, XM_0	NP_001107578, NP_00	XP_005270497, XP_0052	protein_coding	ENSG0000014303	126969	28689
1145	SOCS4	1.283	0.000	suppressor of cytokine signaling 4 [Source:HGNC Symbol;Acc:HGNC:19392]	chr14:55493947-55516206	NM_080867, NM_1	XM_011536426, XM_0	NP_543143, NP_95545	XP_011534727, XP_0115	protein_coding	ENSG0000018000	122809	19392
1146	SPNS1	-1.129	0.000	sphingolipid transporter 1 (putative) [Source:HGNC Symbol;Acc:HGNC:30621]	chr16:28986092-28995869	NM_001142448, NM	XM_017023759, XM_0	NP_001135920, NP_00	XP_006721159, XP_0168	protein_coding	ENSG0000016968	83985	30621
1147	STAG2	1.070	0.000	stromal antigen 2 [Source:HGNC Symbol;Acc:HGNC:11355]	chrX:123094368-123236506	NM_001042749, NM	XM_011531254, XM_0	NP_001036214, NP_00	XP_005262414, XP_0052	protein_coding	ENSG0000010197	10735	11355
1148	TRA2A	1.041	0.000	transformer 2 alpha homolog [Source:HGNC Symbol;Acc:HGNC:16645]	chr7:23544398-23571660	NM_001282757, NM	XM_017012090, XM_0	NP_001269686, NP_00	XP_005249782, XP_0067	protein_coding	ENSG0000016454	29896	16645
1149	WDR37	1.220	0.000	WD repeat domain 37 [Source:HGNC Symbol;Acc:HGNC:31406]	chr10:1095477-1178237	NM_014023	.	NP_054742	.	protein_coding	ENSG0000004705	22884	31406
1150	XRCC2	1.199	0.000	X-ray repair cross complementing 2 [Source:HGNC Symbol;Acc:HGNC:12829]	chr7:152343588-152373250	NM_005431	.	NP_005422	.	protein_coding	ENSG0000019658	7516	12829
1151	ZC3H11B	1.572	0.000	zinc finger CCCH-type containing 11B pseudogene [Source:HGNC Symbol;Acc:HGNC:256	chr1:219781688-219786487	.	.	NP_001258604	XP_016866378	protein_coding	ENSG0000021581	441155	25659
1152	AKAP10	1.142	0.000	A-kinase anchoring protein 10 [Source:HGNC Symbol;Acc:HGNC:368]	chr17:19807614-19881150	NM_007202	XM_006721432, XM_0	NP_009133	XP_006721495, XP_0168	protein_coding	ENSG0000010859	11216	368
1153	APLNR	-4.129	0.000	apelin receptor [Source:HGNC Symbol;Acc:HGNC:339]	chr11:57001066-57004788	NM_005161	.	NP_005152	.	protein_coding	ENSG0000013481	187	339
1154	ATP2B1	1.361	0.000	ATPase plasma membrane Ca2+ transporting 1 [Source:HGNC Symbol;Acc:HGNC:814]	chr12:89981825-90102608	NM_001001323, NM	XM_011538411, XM_0	NP_001001323, NP_00	XP_011536709, XP_0115	protein_coding	ENSG0000007096	490	814
1155	CDC42EP5	-1.218	0.000	CDC42 effector protein 5 [Source:HGNC Symbol;Acc:HGNC:17408]	chr19:54976209-54984411	NM_145057	.	NP_659494	.	protein_coding	ENSG0000016761	148170	17408
1156	CXCR1	-1.970	0.000	C-X-C motif chemokine receptor 1 [Source:HGNC Symbol;Acc:HGNC:6026]	chr2:219027567-219031718	NM_000634	.	NP_000625	.	protein_coding	ENSG0000016346	3577	6026
1157	DESI2	1.016	0.000	desumoylating isopeptidase 2 [Source:HGNC Symbol;Acc:HGNC:24264]	chr1:244816236-244872335	NM_001297746, NM	XM_011544203	NP_001284675, NP_05	XP_011542505, XP_0115	protein_coding	ENSG0000012164	51029	24264
1158	DHRS1	-1.080	0.000	dehydrogenase/reductase 1 [Source:HGNC Symbol;Acc:HGNC:16445]	chr14:24759803-24769039	NM_001136050, NM	.	NP_001129522, NP_61	.	protein_coding	ENSG0000015737	115817	16445
1159	ENTPD2	-1.118	0.000	ectonucleoside triphosphate diphosphohydrolase 2 [Source:HGNC Symbol;Acc:HGNC:33	chr9:139942549-139948497	NM_001246, NM_2	XM_011519212, XM_0	NP_001237, NP_98229	XP_011517514	protein_coding	ENSG0000005417	954	3364
1160	FAM126B	1.437	0.000	family with sequence similarity 126 member B [Source:HGNC Symbol;Acc:HGNC:28593]	chr2:201838440-201936392	NM_001321618, NM	XM_011511003, XM_0	NP_001308547, NP_00	XP_006712506, XP_0115	protein_coding	ENSG0000015574	285172	28593
1161	FKBP8	-0.818	0.000	FK506 binding protein 8 [Source:HGNC Symbol;Acc:HGNC:3724]	chr19:18642560-18654470	NM_001308373, NM	XM_011527864, XM_0	NP_001295302, NP_03	XP_011526164, XP_0115	protein_coding	ENSG0000010570	23770	3724
1162	FLNB	-1.204	0.000	filamin B [Source:HGNC Symbol;Acc:HGNC:3755]	chr3:57994126-58157978	NM_001164317, NM	XM_005264981, XM_0	NP_001157789, NP_00	XP_005265035	protein_coding	ENSG0000013606	2317	3755
1163	HERC6	1.567	0.000	HECT and RLD domain containing E3 ubiquitin protein ligase family member 6 [Source:H	chr4:89299890-89364263	NM_001165136, NM	XM_011532053, XM_0	NP_001158608, NP_06	XP_005263140, XP_0115	protein_coding	ENSG0000013864	55008	26072
1164	KCNH2	-1.167	0.000	potassium voltage-gated channel subfamily H member 2 [Source:HGNC Symbol;Acc:HGN	chr7:150642048-150675403	NM_000238, NM_1	.	NP_000229, NP_74205	XP_011514487, XP_0168	protein_coding	ENSG0000005511	3757	6251
1165	LA16c-380H5	-1.557	0.000	.	chr16:3038256-3052017	.	.	.	.	protein_coding	ENSG0000027016	.	.
1166	LIG4	1.530	0.000	DNA ligase 4 [Source:HGNC Symbol;Acc:HGNC:6601]	chr13:108859786-108870716	NM_001098268, NM	XM_017020573, XM_0	NP_001091738, NP_00	XP_005254113, XP_0052	protein_coding	ENSG0000017440	3981	6601
1167	NFKBIA	-0.982	0.000	NFKB inhibitor alpha [Source:HGNC Symbol;Acc:HGNC:7797]	chr14:35870716-35873952	NM_020529	.	NP_065390	.	protein_coding	ENSG0000010090	4792	7797
1168	NTN1	-1.065	0.000	netrin 1 [Source:HGNC Symbol;Acc:HGNC:8029]	chr17:8924858-9147317	NM_004822	XM_006721595	NP_004813	XP_006721658	protein_coding	ENSG0000006532	9423	8029
1169	PDZD8	1.143	0.000	PDZ domain containing 8 [Source:HGNC Symbol;Acc:HGNC:26974]	chr10:119000603-119134978	NM_173791	.	NP_776152	.	protein_coding	ENSG0000016565	118987	26974
1170	POC1A	-0.932	0.000	POC1 centriolar protein A [Source:HGNC Symbol;Acc:HGNC:24488]	chr3:52109268-52188706	NM_001161580, NM	XM_011533561, XM_0	NP_001155052, NP_00	XP_011531862, XP_0115	protein_coding	ENSG0000016408	25886	24488
1171	PUS7L	1.562	0.000	pseudouridylate synthase 7 like [Source:HGNC Symbol;Acc:HGNC:25276]	chr12:44112795-44152620	NM_001098614, NM	XM_011538790, XM_0	NP_001092084, NP_00	XP_005269225, XP_0115	protein_coding	ENSG0000012931	83448	25276
1172	R3HCC1L	1.386	0.000	R3H domain and coiled-coil containing 1 like [Source:HGNC Symbol;Acc:HGNC:23512]	chr10:99894380-100004654	NM_001256619, NM	XM_017016083, XM_0	NP_001243548, NP_00	XP_011537941, XP_0115	protein_coding	ENSG0000016602	27291	23512
1173	RASAL1	-1.174	0.000	RAS protein activator like 1 [Source:HGNC Symbol;Acc:HGNC:9873]	chr12:113536623-113574044	NM_001193520, NM	XM_011538853, XM_0	NP_001180449, NP_00	XP_005254007, XP_0067	protein_coding	ENSG0000011134	8437	9873
1174	RBP1	-0.903	0.000	retinol binding protein 1 [Source:HGNC Symbol;Acc:HGNC:9919]	chr3:139236275-139258671	NM_001130992, NM	.	NP_001124464, NP_00	.	protein_coding	ENSG0000011411	5947	9919
1175	RDX	0.987	0.000	radixin [Source:HGNC Symbol;Acc:HGNC:9944]	chr11:110045604-110167447	NM_001260492, NM	.	NP_001247421, NP_00	.	protein_coding	ENSG0000013771	5962	9944
1176	RNF6	1.018	0.000	ring finger protein 6 [Source:HGNC Symbol;Acc:HGNC:10069]	chr13:26786898-26796630	NM_005977, NM_1	XM_011535177, XM_0	NP_005968, NP_89886	XP_005266542, XP_0052	protein_coding	ENSG0000012787	6049	10069
1177	RRM1	0.862	0.000	ribonucleotide reductase catalytic subunit M1 [Source:HGNC Symbol;Acc:HGNC:10451]	chr11:4116038-4160106	NM_001033, NM_0	XM_011520279, XM_0	NP_001024, NP_00130	XP_011518580	protein_coding	ENSG0000016732	6240	10451
1178	SATB2	1.263	0.000	SATB homeobox 2 [Source:HGNC Symbol;Acc:HGNC:21637]	chr2:200134222-200335989	NM_001172509, NM	XM_011510840, XM_0	NP_001165980, NP_00	XP_005246453, XP_0067	protein_coding	ENSG0000011904	23314	21637
1179	SDCBP	0.979	0.000	syndecan binding protein [Source:HGNC Symbol;Acc:HGNC:10662]	chr8:59465727-59495419	NM_001007067, NM	XM_017013728, XM_0	NP_001007068, NP_00	XP_011515873, XP_0168	protein_coding	ENSG0000013757	6386	10662
1180	SHCBP1	1.106	0.000	SHC binding and spindle associated 1 [Source:HGNC Symbol;Acc:HGNC:29547]	chr16:46614465-46655538	NM_024745	.	NP_079021	.	protein_coding	ENSG0000017124	79801	29547
1181	SLC35A2	-1.146	0.000	solute carrier family 35 member A2 [Source:HGNC Symbol;Acc:HGNC:11022]	chrX:48760456-48769235	NM_001032289, NM	.	NP_001027460, NP_00	.	protein_coding	ENSG0000010210	7355	11022
1182	SSFA2	0.987	0.000	sperm specific antigen 2 [Source:HGNC Symbol;Acc:HGNC:11319]	chr2:182756614-182795465	NM_001130445, NM	XM_011511703, XM_0	NP_001123917, NP_00	XP_005246869, XP_0052	protein_coding	ENSG0000013843	6744	11319
1183	TCEA2	-1.106	0.000	transcription elongation factor A2 [Source:HGNC Symbol;Acc:HGNC:11614]	chr20:62688645-62703699	NM_003195, NM_1	XM_011529027, XM_0	NP_003186, NP_94201	XP_005260286, XP_0115	protein_coding	ENSG0000017170	6919	11614
1184	TGFB2	1.619	0.000	transforming growth factor beta 2 [Source:HGNC Symbol;Acc:HGNC:11768]	chr1:218519576-218617961	NM_001135599, NM	.	NP_001129071, NP_00	.	protein_coding	ENSG0000009296	7042	11768
1185	TRAPPC8	1.440	0.000	trafficking protein particle complex 8 [Source:HGNC Symbol;Acc:HGNC:29169]	chr18:29409135-29523069	NM_014939	XM_011525884, XM_0	NP_055754	XP_005258290, XP_0052	protein_coding	ENSG0000015333	22878	29169
1186	ZUFSP	1.381	0.000	zinc finger with UFM1 specific peptidase domain [Source:HGNC Symbol;Acc:HGNC:2122	chr6:116956780-116989957	NM_145062	XM_011535567, XM_0	NP_659499	XP_011533867, XP_0115	protein_coding	ENSG0000015397	221302	21224

1187	BIRC3	1.229	0.001	baculoviral IAP repeat containing 3 [Source:HGNC Symbol;Acc:HGNC:591]	chr11:102188180-102210134	NM_001165, NM_1	XM_017017643	NP_001156, NP_89200	XP_016873132	protein_coding	ENSG0000002344	330	591
1188	BRK1	-1.011	0.001	BRICK1, SCAR/WAVE actin nucleating complex subunit [Source:HGNC Symbol;Acc:HGNC	chr3:10157275-10168874	NM_018462	.	NP_060932	.	protein_coding	ENSG0000025499	55845	23057
1189	CCNT1	1.042	0.001	cyclin T1 [Source:HGNC Symbol;Acc:HGNC:1599]	chr12:49082240-49110781	NM_001240, NM_0	XM_017020197	NP_001231, NP_00126	XP_016875686	protein_coding	ENSG0000012931	904	1599
1190	CEP295	1.500	0.001	centrosomal protein 295 [Source:HGNC Symbol;Acc:HGNC:29366]	chr11:93394804-93463524	NM_033395	XM_017018475, XM_0	NP_203753	XP_005274423, XP_0052	protein_coding	ENSG0000016600	85459	29366
1191	CERS4	-1.222	0.001	ceramide synthase 4 [Source:HGNC Symbol;Acc:HGNC:23747]	chr19:8274196-8327305	NM_024552	XM_011528296, XM_0	NP_078828	XP_006722959, XP_0115	protein_coding	ENSG0000009066	79603	23747
1192	CPXM1	-1.427	0.001	carboxypeptidase X, M14 family member 1 [Source:HGNC Symbol;Acc:HGNC:15771]	chr20:2774714-2781283	NM_001184699, NN	.	NP_001171628, NP_06	.	protein_coding	ENSG0000008888	56265	15771
1193	CTDSPL2	1.250	0.001	CTD small phosphatase like 2 [Source:HGNC Symbol;Acc:HGNC:26936]	chr15:44719431-44821236	NM_016396	XM_011521665, XM_0	NP_057480	XP_005254498, XP_0067	protein_coding	ENSG0000013777	51496	26936
1194	DGKH	1.286	0.001	diacylglycerol kinase eta [Source:HGNC Symbol;Acc:HGNC:2854]	chr13:42614171-42817032	NM_001204504, NN	.	NP_001191433, NP_00	.	protein_coding	ENSG0000010278	160851	2854
1195	DHX9	0.836	0.001	DEAH-box helicase 9 [Source:HGNC Symbol;Acc:HGNC:2750]	chr1:182808503-182856886	NM_001357	.	NP_001348	.	protein_coding	ENSG0000013582	1660	2750
1196	EMC10	-0.942	0.001	ER membrane protein complex subunit 10 [Source:HGNC Symbol;Acc:HGNC:27609]	chr19:50970043-50994127	NM_175063, NM_2	.	NP_778233, NP_99626	.	protein_coding	ENSG0000016167	284361	27609
1197	ENTPD4	1.460	0.001	ectonucleoside triphosphate diphosphohydrolase 4 [Source:HGNC Symbol;Acc:HGNC:14	chr8:23154701-23315208	NM_001128930, NN	.	NP_001122402, NP_00	.	protein_coding	ENSG0000019721	9583	14573
1198	FAM69B	-1.072	0.001	family with sequence similarity 69 member B [Source:HGNC Symbol;Acc:HGNC:28290]	chr9:139607021-139618502	NM_152421	.	NP_689634	XP_006717017, XP_0168	protein_coding	ENSG0000016571	138311	28290
1199	FNIP2	1.379	0.001	folliculin interacting protein 2 [Source:HGNC Symbol;Acc:HGNC:29280]	chr4:159690289-159956333	NM_001323916, NN	XM_011532154, XM_0	NP_001310845, NP_06	XP_005263213, XP_0052	protein_coding	ENSG0000005279	57600	29280
1200	GOLGA5	1.060	0.001	golgin A5 [Source:HGNC Symbol;Acc:HGNC:4428]	chr14:93260575-93306308	NM_005113	XM_011537420	NP_005104	XP_011535722	protein_coding	ENSG0000006645	9950	4428
1201	GTF3C3	1.001	0.001	general transcription factor IIIC subunit 3 [Source:HGNC Symbol;Acc:HGNC:4666]	chr2:197627755-197664425	NM_001206774, NN	.	NP_001193703, NP_03	.	protein_coding	ENSG0000011904	9330	4666
1202	HCCS	-0.938	0.001	holocytochrome c synthase [Source:HGNC Symbol;Acc:HGNC:4837]	chrX:11129420-11141198	NM_001122608, NN	.	NP_001116080, NP_00	.	protein_coding	ENSG0000000496	3052	4837
1203	HECTD1	1.089	0.001	HECT domain E3 ubiquitin protein ligase 1 [Source:HGNC Symbol;Acc:HGNC:20157]	chr14:31569323-31677010	NM_015382	XM_011536621, XM_0	NP_056197	XP_005267559, XP_0115	protein_coding	ENSG0000009214	25831	20157
1204	IARS	0.824	0.001	isoleucyl-tRNA synthetase [Source:HGNC Symbol;Acc:HGNC:5330]	chr9:94972488-95056038	NM_002161, NM_0	.	NP_002152, NP_03820	.	protein_coding	ENSG0000019630	3376	5330
1205	IPO5	0.871	0.001	importin 5 [Source:HGNC Symbol;Acc:HGNC:6402]	chr13:98605928-98676551	NM_002271	XM_017020562, XM_0	NP_002262	XP_005254109, XP_0052	protein_coding	ENSG0000006515	3843	6402
1206	JUND	-0.980	0.001	JunD proto-oncogene, AP-1 transcription factor subunit [Source:HGNC Symbol;Acc:HGN	chr19:18390569-18392432	NM_001286968, NN	.	NP_001273897, NP_00	.	protein_coding	ENSG0000013052	3727	6206
1207	MAPK6	1.176	0.001	mitogen-activated protein kinase 6 [Source:HGNC Symbol;Acc:HGNC:6879]	chr15:52311397-52359569	NM_002748	XM_011521782, XM_0	NP_002739	XP_005254593, XP_0052	protein_coding	ENSG0000006995	5597	6879
1208	MAZ	-0.910	0.001	MYC associated zinc finger protein [Source:HGNC Symbol;Acc:HGNC:6914]	chr16:29817426-29822485	NM_001042539, NN	XM_006721047	NP_001036004, NP_00	XP_006721110	protein_coding	ENSG0000010349	4150	6914
1209	MTIF2	1.052	0.001	mitochondrial translational initiation factor 2 [Source:HGNC Symbol;Acc:HGNC:7441]	chr2:55463730-55496384	NM_001005369, NN	XM_011532873, XM_0	NP_001005369, NP_00	XP_005264392, XP_0115	protein_coding	ENSG0000008576	4528	7441
1210	MTRNR2L12	1.052	0.001	MT-RNR2-like 12 [Source:HGNC Symbol;Acc:HGNC:37169]	chr3:96336031-96337080	NM_001190470	.	NP_001177399	.	protein_coding	ENSG0000026902	100462981	37169
1211	NAA25	1.155	0.001	N(alpha)-acetyltransferase 25, NatB auxiliary subunit [Source:HGNC Symbol;Acc:HGNC:2	chr12:112464492-112546826	NM_024953	XM_006719606, XM_0	NP_079229	XP_005253995, XP_0067	protein_coding	ENSG0000011130	80018	25783
1212	OAF	-1.054	0.001	out at first homolog [Source:HGNC Symbol;Acc:HGNC:28752]	chr11:120081474-120101041	NM_178507	.	NP_848602	.	protein_coding	ENSG0000018423	220323	28752
1213	PDE8A	1.018	0.001	phosphodiesterase 8A [Source:HGNC Symbol;Acc:HGNC:8793]	chr15:85523670-85682376	NM_001243137, NN	.	NP_001230066, NP_00	.	protein_coding	ENSG0000007341	5151	8793
1214	PPP2CB	0.967	0.001	protein phosphatase 2 catalytic subunit beta [Source:HGNC Symbol;Acc:HGNC:9300]	chr8:30643125-30670388	NM_001009552	.	NP_001009552	.	protein_coding	ENSG0000010469	5516	9300
1215	PRRX1	1.663	0.001	paired related homeobox 1 [Source:HGNC Symbol;Acc:HGNC:9142]	chr1:170632274-170708560	NM_006902, NM_0	XM_006711388	NP_008833, NP_07320	XP_006711451	protein_coding	ENSG0000011613	5396	9142
1216	SOAT1	1.167	0.001	sterol O-acyltransferase 1 [Source:HGNC Symbol;Acc:HGNC:11177]	chr1:179262848-179327815	NM_001252511, NN	XM_011509911	NP_001239440, NP_00	XP_011508213	protein_coding	ENSG0000005725	6646	11177
1217	SPATS2L	0.996	0.001	spermatogenesis associated serine rich 2 like [Source:HGNC Symbol;Acc:HGNC:24574]	chr2:201170615-201346986	NM_001100422, NN	XM_017003785, XM_0	NP_001093892, NP_00	XP_005246513, XP_0052	protein_coding	ENSG0000019614	26010	24574
1218	STRIP2	1.301	0.001	striatin interacting protein 2 [Source:HGNC Symbol;Acc:HGNC:22209]	chr7:129074273-129128240	NM_001134336, NN	XM_011516432, XM_0	NP_001127808, NP_06	XP_006716132, XP_0115	protein_coding	ENSG0000012857	57464	22209
1219	SUV39H1	-1.008	0.001	suppressor of variegation 3-9 homolog 1 [Source:HGNC Symbol;Acc:HGNC:11479]	chrX:48553944-48567403	NM_001282166, NN	.	NP_001269095, NP_00	.	protein_coding	ENSG0000010194	6839	11479
1220	SYDE2	1.693	0.001	synapse defective Rho GTPase homolog 2 [Source:HGNC Symbol;Acc:HGNC:25841]	chr1:85622555-85666729	NM_032184	XM_011542257, XM_0	NP_115560	.	protein_coding	ENSG0000009709	84144	25841
1221	TRIM29	-0.901	0.001	tripartite motif containing 29 [Source:HGNC Symbol;Acc:HGNC:17274]	chr11:119981982-120009161	NM_012101	XM_011542732, XM_0	NP_036233	XP_005271547, XP_0115	protein_coding	ENSG0000013769	23650	17274
1222	TRPM7	1.261	0.001	transient receptor potential cation channel subfamily M member 7 [Source:HGNC Symb	chr15:50849354-50979012	NM_001301212, NN	XM_017022353, XM_0	NP_001288141, NP_06	XP_005254543, XP_0168	protein_coding	ENSG0000009243	54822	17994
1223	TTLL7	1.718	0.001	tubulin tyrosine ligase like 7 [Source:HGNC Symbol;Acc:HGNC:26242]	chr1:84330710-84464833	NM_024686	XM_017002347, XM_0	NP_078962	XP_005271265, XP_0115	protein_coding	ENSG0000013794	79739	26242
1224	UBN2	1.270	0.001	ubinuclein 2 [Source:HGNC Symbol;Acc:HGNC:21931]	chr7:138916230-138992982	NM_173569	XM_011516002, XM_0	NP_775840	XP_005250306, XP_0067	protein_coding	ENSG0000015774	254048	21931
1225	UGGT1	0.909	0.001	UDP-glucose glycoprotein glucosyltransferase 1 [Source:HGNC Symbol;Acc:HGNC:15663	chr2:128848884-128953251	NM_020120	XM_017004508, XM_0	NP_064505	XP_006712697, XP_0067	protein_coding	ENSG0000013673	56886	15663
1226	WAC	0.999	0.001	WW domain containing adaptor with coiled-coil [Source:HGNC Symbol;Acc:HGNC:17327	chr10:28821421-28912041	NM_016628, NM_1	XM_011519491, XM_0	NP_057712, NP_56782	XP_011517793, XP_0168	protein_coding	ENSG0000009578	51322	17327
1227	ZC4H2	-1.404	0.001	zinc finger C4H2-type containing [Source:HGNC Symbol;Acc:HGNC:24931]	chrX:64135681-64254593	NM_001178032, NN	.	NP_001171503, NP_00	.	protein_coding	ENSG0000012697	55906	24931
1228	ZNF121	1.513	0.001	zinc finger protein 121 [Source:HGNC Symbol;Acc:HGNC:12904]	chr19:9671028-9695209	NM_001008727, NN	XM_011528268, XM_0	NP_001008727, NP_00	XP_005260119, XP_0115	protein_coding	ENSG0000019796	7675	12904
1229	ZNF680	1.566	0.001	zinc finger protein 680 [Source:HGNC Symbol;Acc:HGNC:26897]	chr7:63980261-64023484	NM_001130022, NN	.	NP_001123494, NP_84	XP_006716022	protein_coding	ENSG0000017304	340252	26897
1230	ZNF766	1.322	0.001	zinc finger protein 766 [Source:HGNC Symbol;Acc:HGNC:28063]	chr19:52772823-52799299	NM_001010851	.	NP_001010851	XP_011525762, XP_0168	protein_coding	ENSG0000019621	90321	28063
1231	ABHD14B	-1.329	0.001	abhydrolase domain containing 14B [Source:HGNC Symbol;Acc:HGNC:28235]	chr3:52002525-52023218	NM_001146314, NN	XM_011534162	NP_001139786, NP_11	XP_011532464	protein_coding	ENSG0000011477	84836	28235
1232	ADIRF	-0.954	0.001	adipogenesis regulatory factor [Source:HGNC Symbol;Acc:HGNC:24043]	chr10:88727948-88730672	NM_006829	.	NP_006820	.	protein_coding	ENSG0000014867	10974	24043
1233	BOD1L1	1.112	0.001	biorientation of chromosomes in cell division 1 like 1 [Source:HGNC Symbol;Acc:HGNC:3	chr4:13570361-13629347	NM_148894	XM_017008010, XM_0	NP_683692	XP_005248207, XP_0052	protein_coding	ENSG0000003821	259282	31792
1234	C10orf54	-1.531	0.001	chromosome 10 open reading frame 54 [Source:HGNC Symbol;Acc:HGNC:30085]	chr10:73156690-73575704	NM_022153	.	NP_071436	.	protein_coding	ENSG0000010773	64115	30085
1235	C5orf22	1.225	0.001	chromosome 5 open reading frame 22 [Source:HGNC Symbol;Acc:HGNC:25639]	chr5:31532372-31555165	NM_018356	XM_017009613, XM_0	NP_060826	XP_006714542, XP_0067	protein_coding	ENSG0000008221	55322	25639
1236	CCND2	0.934	0.001	cyclin D2 [Source:HGNC Symbol;Acc:HGNC:1583]	chr12:4382937-4414516	NM_001759	.	NP_001750	.	protein_coding	ENSG0000011897	894	1583
1237	CEBPG	0.960	0.001	CCAAT/enhancer binding protein gamma [Source:HGNC Symbol;Acc:HGNC:1837]	chr19:33864235-33873592	NM_001252296, NN	.	NP_001239225, NP_00	.	protein_coding	ENSG0000015387	1054	1837
1238	EPHA8	-1.985	0.001	EPH receptor A8 [Source:HGNC Symbol;Acc:HGNC:3391]	chr1:22890056-22930087	NM_001006943, NN	XM_011540974, XM_0	NP_001006944, NP_06	XP_011539271, XP_0115	protein_coding	ENSG0000007088	2046	3391
1239	FAM220A	1.109	0.001	family with sequence similarity 220 member A [Source:HGNC Symbol;Acc:HGNC:22422]	chr7:6369039-6388590	NM_001037163	.	NP_001032240	.	protein_coding	ENSG0000017839	84792	22422
1240	FSD1	-1.063	0.001	fibronectin type III and SPRY domain containing 1 [Source:HGNC Symbol;Acc:HGNC:137	chr19:4304596-4323833	NM_024333	XM_005259644	NP_077309	XP_005259701	protein_coding	ENSG0000010525	79187	13745

1241	FUT8	1.218	0.001	fucosyltransferase 8 [Source:HGNC Symbol;Acc:HGNC:4019]	chr14:65877309-66210839	NM_004480, NM_1	XM_011536613	NP_004471, NP_83536	XP_011534915	protein_coding	ENSG0000003317	2530	4019
1242	GNL2	0.926	0.001	G protein nucleolar 2 [Source:HGNC Symbol;Acc:HGNC:29925]	chr1:38022519-38061522	NM_013285	XM_011541300	NP_037417	.	protein_coding	ENSG0000013469	29889	29925
1243	GUF1	1.188	0.001	GUF1 homolog, GTPase [Source:HGNC Symbol;Acc:HGNC:25799]	chr4:44680445-44702943	NM_021927	XM_011513733, XM_0	NP_068746	XP_005248179, XP_0115	protein_coding	ENSG0000015180	60558	25799
1244	IFITM2	-0.841	0.001	interferon induced transmembrane protein 2 [Source:HGNC Symbol;Acc:HGNC:5413]	chr11:307630-309395	NM_006435	.	NP_006426	.	protein_coding	ENSG0000018520	10581	5413
1245	KIAA1324L	1.241	0.001	KIAA1324 like [Source:HGNC Symbol;Acc:HGNC:21945]	chr7:86506221-86689015	NM_001142749, NN	XM_011515921, XM_0	NP_001136221, NP_00	XP_006715957, XP_0115	protein_coding	ENSG0000016465	222223	21945
1246	LMAN1	1.027	0.001	lectin, mannose binding 1 [Source:HGNC Symbol;Acc:HGNC:6631]	chr18:56995054-57027194	NM_005570	.	NP_005561	.	protein_coding	ENSG0000007469	3998	6631
1247	MYH14	-0.964	0.001	myosin heavy chain 14 [Source:HGNC Symbol;Acc:HGNC:23212]	chr19:50691442-50813802	NM_001077186, NN	XM_011527323, XM_0	NP_001070654, NP_00	XP_006723449, XP_0115	protein_coding	ENSG0000010535	79784	23212
1248	NCOR1	0.883	0.001	nuclear receptor corepressor 1 [Source:HGNC Symbol;Acc:HGNC:7672]	chr17:15932470-16118874	NM_001190438, NN	XM_011524086, XM_0	NP_001177367, NP_00	XP_005256923, XP_0052	protein_coding	ENSG0000014102	9611	7672
1249	OCLN	1.423	0.001	occludin [Source:HGNC Symbol;Acc:HGNC:8104]	chr5:68788118-68853931	NM_001205254, NN	.	NP_001192183, NP_00	.	protein_coding	ENSG0000019782	100506658	8104
1250	PALB2	1.188	0.001	partner and localizer of BRCA2 [Source:HGNC Symbol;Acc:HGNC:26144]	chr16:23614488-23652631	NM_024675	XM_011545948, XM_0	NP_078951	XP_011544248, XP_0115	protein_coding	ENSG0000008309	79728	26144
1251	PHLPP2	1.258	0.001	PH domain and leucine rich repeat protein phosphatase 2 [Source:HGNC Symbol;Acc:HG	chr16:71678826-71758604	NM_001289003, NN	.	NP_001275932, NP_05	.	protein_coding	ENSG0000004019	23035	29149
1252	PLXND1	-0.901	0.001	plexin D1 [Source:HGNC Symbol;Acc:HGNC:9107]	chr3:129274017-129325661	NM_015103	XM_011512589, XM_0	NP_055918	XP_011510890, XP_0115	protein_coding	ENSG0000000439	23129	9107
1253	PPP2R5A	1.118	0.001	protein phosphatase 2 regulatory subunit B'alpha [Source:HGNC Symbol;Acc:HGNC:930	chr1:212458878-212535205	NM_001199756, NN	.	NP_001186685, NP_00	.	protein_coding	ENSG0000006602	5525	9309
1254	PRKAA2	1.217	0.001	protein kinase AMP-activated catalytic subunit alpha 2 [Source:HGNC Symbol;Acc:HGNC	chr1:57110994-57181008	NM_006252	XM_017001694, XM_0	NP_006243	XP_016857182, XP_0168	protein_coding	ENSG0000016240	5563	9377
1255	RALGPS2	1.220	0.001	Ral GEF with PH domain and SH3 binding motif 2 [Source:HGNC Symbol;Acc:HGNC:3027	chr1:178694281-178890976	NM_001286247, NN	XM_011509688, XM_0	NP_001273176, NP_68	XP_006711473, XP_0115	protein_coding	ENSG0000011619	55103	30279
1256	RAPGEF6	1.736	0.001	Rap guanine nucleotide exchange factor 6 [Source:HGNC Symbol;Acc:HGNC:20655]	chr5:130759613-131132756	NM_001164386, NN	.	NP_001157858, NP_00	.	protein_coding	ENSG0000015898	51735	20655
1257	RNF208	-1.177	0.001	ring finger protein 208 [Source:HGNC Symbol;Acc:HGNC:25420]	chr9:140114698-140116033	NM_031297	.	NP_112587	.	protein_coding	ENSG0000021286	727800	25420
1258	S100A13	-0.849	0.001	S100 calcium binding protein A13 [Source:HGNC Symbol;Acc:HGNC:10490]	chr1:153591274-153604513	NM_001024210, NN	XM_011509864, XM_0	NP_001019381, NP_00	XP_005245491, XP_0115	protein_coding	ENSG0000018917	6284	10490
1259	SECISBP2	0.991	0.001	SECIS binding protein 2 [Source:HGNC Symbol;Acc:HGNC:30972]	chr9:91933420-91974577	NM_001282688, NN	XM_011519003, XM_0	NP_001269617, NP_00	XP_005252253, XP_0067	protein_coding	ENSG0000018774	79048	30972
1260	SERPINF1	-0.984	0.001	serpin family F member 1 [Source:HGNC Symbol;Acc:HGNC:8824]	chr17:1665252-1680868	NM_002615	.	NP_002606	XP_016880243	protein_coding	ENSG0000013238	5176	8824
1261	SLFN5	1.255	0.001	schlafen family member 5 [Source:HGNC Symbol;Acc:HGNC:28286]	chr17:33570054-33600674	NM_144975	XM_005257934	NP_659412	XP_005257991	protein_coding	ENSG0000016675	162394	28286
1262	SLK	1.105	0.001	STE20 like kinase [Source:HGNC Symbol;Acc:HGNC:11088]	chr10:105726958-105788991	NM_001304743, NN	XM_011540401	NP_001291672, NP_05	XP_011538703	protein_coding	ENSG0000006561	9748	11088
1263	SPARC	-4.712	0.001	secreted protein acidic and cysteine rich [Source:HGNC Symbol;Acc:HGNC:11219]	chr5:151040656-151066726	NM_001309443, NN	.	NP_001296372, NP_00	.	protein_coding	ENSG0000011314	6678	11219
1264	SUZ12	1.064	0.001	SUZ12 polycomb repressive complex 2 subunit [Source:HGNC Symbol;Acc:HGNC:17101]	chr17:30264036-30328064	NM_015355	XM_006721794, XM_0	NP_001308136, NP_05	XP_005258011, XP_0067	protein_coding	ENSG0000017869	23512	17101
1265	TRPV2	-0.907	0.001	transient receptor potential cation channel subfamily V member 2 [Source:HGNC Symbo	chr17:16318855-16340317	NM_016113	XM_011523922, XM_0	NP_057197	XP_005256733, XP_0052	protein_coding	ENSG0000018768	51393	18082
1266	USP19	-0.898	0.001	ubiquitin specific peptidase 19 [Source:HGNC Symbol;Acc:HGNC:12617]	chr3:49145478-49158371	NM_001199160, NN	XM_017005626, XM_0	NP_001186089, NP_00	XP_005264880, XP_0052	protein_coding	ENSG0000017204	10869	12617
1267	ZNF100	1.833	0.001	zinc finger protein 100 [Source:HGNC Symbol;Acc:HGNC:12880]	chr19:21905567-21950430	NM_173531	XM_011527744, XM_0	NP_775802	XP_011526045, XP_0115	protein_coding	ENSG0000019702	163227	12880
1268	ALG3	-0.905	0.001	ALG3, alpha-1,3- mannosyltransferase [Source:HGNC Symbol;Acc:HGNC:23056]	chr3:183960121-183967336	NM_001006941, NN	XM_011512323	NP_001006942, NP_00	XP_011510624, XP_0115	protein_coding	ENSG0000021416	10195	23056
1269	ARHGDIG	-1.794	0.001	Rho GDP dissociation inhibitor gamma [Source:HGNC Symbol;Acc:HGNC:680]	chr16:330605-333010	NM_001176	.	NP_001167	.	protein_coding	ENSG0000024217	398	680
1270	BIRC2	1.213	0.001	baculoviral IAP repeat containing 2 [Source:HGNC Symbol;Acc:HGNC:590]	chr11:102217944-102249401	NM_001166, NM_0	.	NP_001157, NP_00124	.	protein_coding	ENSG0000011033	329	590
1271	CAMK1	-1.685	0.001	calcium/calmodulin dependent protein kinase I [Source:HGNC Symbol;Acc:HGNC:1459]	chr3:9791627-9811661	NM_003656	XM_005265517, XM_0	NP_003647	XP_005265573, XP_0052	protein_coding	ENSG0000013407	8536	1459
1272	COL6A1	-0.954	0.001	collagen type VI alpha 1 chain [Source:HGNC Symbol;Acc:HGNC:2211]	chr21:47401650-47424964	NM_001848	.	NP_001839	.	protein_coding	ENSG0000014215	1291	2211
1273	CPZ	-1.337	0.001	carboxypeptidase Z [Source:HGNC Symbol;Acc:HGNC:2333]	chr4:8594386-8621486	NM_001014447, NN	.	NP_001014447, NP_00	.	protein_coding	ENSG0000010962	8532	2333
1274	CTXN1	-1.232	0.001	cortexin 1 [Source:HGNC Symbol;Acc:HGNC:31108]	chr19:7989369-7991051	NM_206833	.	NP_996664	.	protein_coding	ENSG0000017853	404217	31108
1275	DENND5B	1.545	0.001	DENN domain containing 5B [Source:HGNC Symbol;Acc:HGNC:28338]	chr12:31535156-31744031	NM_001308339, NN	XM_011520589, XM_0	NP_001295268, NP_65	XP_005253383, XP_0052	protein_coding	ENSG0000017045	160518	28338
1276	DNPH1	-0.968	0.001	2'-deoxynucleoside 5'-phosphate N-hydrolase 1 [Source:HGNC Symbol;Acc:HGNC:21218	chr6:43193366-43197222	NM_006443, NM_1	.	NP_006434, NP_95465	.	protein_coding	ENSG0000011266	10591	21218
1277	EFNB3	-1.056	0.001	ephrin B3 [Source:HGNC Symbol;Acc:HGNC:3228]	chr17:7608519-7614696	NM_001406	.	NP_001397	.	protein_coding	ENSG0000010894	1949	3228
1278	EXOC4	0.842	0.001	exocyst complex component 4 [Source:HGNC Symbol;Acc:HGNC:30389]	chr7:132937828-133750512	NM_001037126, NN	.	NP_001032203, NP_06	XP_005250580	protein_coding	ENSG0000013155	60412	30389
1279	GABRA3	-1.313	0.001	gamma-aminobutyric acid type A receptor alpha3 subunit [Source:HGNC Symbol;Acc:HG	chrX:151334705-151619830	NM_000808	XM_011531134, XM_0	NP_000799	XP_006724874	protein_coding	ENSG0000001167	2556	4077
1280	HMCES	-0.870	0.001	5-hydroxymethylcytosine binding, ES cell specific [Source:HGNC Symbol;Acc:HGNC:2444	chr3:128997682-129025029	NM_001006109, NN	XM_011512994, XM_0	NP_001006109, NP_06	XP_005247693, XP_0052	protein_coding	ENSG0000018362	56941	24446
1281	ITCH	0.904	0.001	itchy E3 ubiquitin protein ligase [Source:HGNC Symbol;Acc:HGNC:13890]	chr20:32951040-33099198	NM_001257137, NN	XM_017028091, XM_0	NP_001244066, NP_00	XP_011527379, XP_0115	protein_coding	ENSG0000007874	83737	13890
1282	KLHDC3	-0.920	0.001	kelch domain containing 3 [Source:HGNC Symbol;Acc:HGNC:20704]	chr6:42981840-42989036	NM_057161	.	NP_476502	.	protein_coding	ENSG0000012470	116138	20704
1283	KMT2C	1.043	0.001	lysine methyltransferase 2C [Source:HGNC Symbol;Acc:HGNC:13726]	chr7:151832009-152133090	NM_170606	XM_011516456, XM_0	NP_733751	XP_005250082, XP_0052	protein_coding	ENSG0000005560	58508	13726
1284	MCM10	1.069	0.001	minichromosome maintenance 10 replication initiation factor [Source:HGNC Symbol;Ac	chr10:13203553-13253104	NM_018518, NM_1	XM_011519538	NP_060988, NP_87742	XP_011517840	protein_coding	ENSG0000006532	55388	18043
1285	NT5DC3	1.166	0.001	5'-nucleotidase domain containing 3 [Source:HGNC Symbol;Acc:HGNC:30826]	chr12:104166087-104234975	NM_001031701	.	NP_001026871	.	protein_coding	ENSG0000011169	51559	30826
1286	PDE7A	1.404	0.001	phosphodiesterase 7A [Source:HGNC Symbol;Acc:HGNC:8791]	chr8:66629744-66754187	NM_001242318, NN	XM_011517540	NP_001229247, NP_00	XP_011515842, XP_0168	protein_coding	ENSG0000020526	5150	8791
1287	PPP1R9A	1.296	0.001	protein phosphatase 1 regulatory subunit 9A [Source:HGNC Symbol;Acc:HGNC:14946]	chr7:94536948-94925727	NM_001166160, NN	XM_017012406, XM_0	NP_001159632, NP_00	XP_011514682, XP_0115	protein_coding	ENSG0000015852	55607	14946
1288	PRKAA1	1.145	0.001	protein kinase AMP-activated catalytic subunit alpha 1 [Source:HGNC Symbol;Acc:HGNC	chr5:40759480-40798476	NM_006251, NM_2	XM_006714482	NP_006242, NP_99679	XP_006714545, XP_0168	protein_coding	ENSG0000013235	5562	9376
1289	RPS9	-0.918	0.001	ribosomal protein S9 [Source:HGNC Symbol;Acc:HGNC:10442]	chr19:54704609-54711515	NM_001013, NM_0	XM_005278288, XM_0	NP_001004, NP_00130	XP_005278344, XP_0052	protein_coding	ENSG0000017088	6203	10442
1290	SLC44A1	1.175	0.001	solute carrier family 44 member 1 [Source:HGNC Symbol;Acc:HGNC:18798]	chr9:108006902-108201452	NM_001286730, NN	XM_017014560, XM_0	NP_001273659, NP_53	XP_005251912, XP_0067	protein_coding	ENSG0000007021	23446	18798
1291	TRAF5	1.238	0.001	TNF receptor associated factor 5 [Source:HGNC Symbol;Acc:HGNC:12035]	chr1:211499956-211548288	NM_001033910, NN	XM_011509960, XM_0	NP_001029082, NP_00	XP_011508259, XP_0115	protein_coding	ENSG0000008251	7188	12035
1292	UBR1	1.571	0.001	ubiquitin protein ligase E3 component n-recognin 1 [Source:HGNC Symbol;Acc:HGNC:16	chr15:43235094-43398311	NM_174916	.	NP_777576	.	protein_coding	ENSG0000015945	197131	16808
1293	YES1	0.907	0.001	YES proto-oncogene 1, Src family tyrosine kinase [Source:HGNC Symbol;Acc:HGNC:1284	chr18:721587-812547	NM_005433	XM_017025960, XM_0	NP_005424	XP_005258196, XP_0115	protein_coding	ENSG0000017610	7525	12841
1294	AHR	1.205	0.001	aryl hydrocarbon receptor [Source:HGNC Symbol;Acc:HGNC:348]	chr7:17338245-17506858	NM_001621	.	NP_001612	.	protein_coding	ENSG0000010654	196	348

1295	API5	0.962	0.001	apoptosis inhibitor 5 [Source:HGNC Symbol;Acc:HGNC:594]	chr11:43333512-43366079	NM_001142930, NM	XM_017018465, XM_0	NP_001136402, NP_00	XP_006718422, XP_0168	protein_coding	ENSG0000016618	8539	594
1296	ATP5D	-0.858	0.001	ATP synthase, H+ transporting, mitochondrial F1 complex, delta subunit [Source:HGNC S	chr19:1241744-1244825	NM_001001975, NM	.	NP_001001975, NP_00	.	protein_coding	ENSG0000009962	513	837
1297	BCAP29	1.315	0.001	B-cell receptor associated protein 29 [Source:HGNC Symbol;Acc:HGNC:24131]	chr7:107220421-107263762	NM_001008405, NM	XM_011516408, XM_0	NP_001008405, NP_06	XP_006716114, XP_0115	protein_coding	ENSG0000007579	55973	24131
1298	BEX4	-1.490	0.001	brain expressed X-linked 4 [Source:HGNC Symbol;Acc:HGNC:25475]	chrX:102470019-102472174	NM_001080425, NM	.	NP_001073894, NP_00	.	protein_coding	ENSG0000010240	56271	25475
1299	CEBPZ	1.630	0.001	CCAAT/enhancer binding protein zeta [Source:HGNC Symbol;Acc:HGNC:24218]	chr2:37394962-37476303	NM_005760	.	NP_005751	.	protein_coding	ENSG0000011581	10153	24218
1300	CKAP2L	1.299	0.001	cytoskeleton associated protein 2 like [Source:HGNC Symbol;Acc:HGNC:26877]	chr2:113479062-113522254	NM_001304361, NM	.	NP_001291290, NP_68	.	protein_coding	ENSG0000016960	150468	26877
1301	DLD	1.034	0.001	dihydrolipoamide dehydrogenase [Source:HGNC Symbol;Acc:HGNC:2898]	chr7:107531414-107561648	NM_000108, NM_0	.	NP_000099, NP_00127	.	protein_coding	ENSG0000009114	1738	2898
1302	HEMK1	-1.492	0.001	HemK methyltransferase family member 1 [Source:HGNC Symbol;Acc:HGNC:24923]	chr3:50595455-50633599	NM_016173	XM_011533814, XM_0	NP_001304780, NP_05	XP_011532108, XP_0115	protein_coding	ENSG0000011473	51409	24923
1303	HPS3	1.133	0.001	HPS3, biogenesis of lysosomal organelles complex 2 subunit 1 [Source:HGNC Symbol;Ac	chr3:148847370-148939842	NM_001308258, NM	XM_011513236, XM_0	NP_001295187, NP_11	XP_011511538, XP_0168	protein_coding	ENSG0000016375	84343	15597
1304	HYAL2	-0.882	0.001	hyaluronoglucosaminidase 2 [Source:HGNC Symbol;Acc:HGNC:5321]	chr3:50355220-50360337	NM_003773, NM_0	XM_005265525, XM_0	NP_003764, NP_14934	XP_005265581, XP_0052	protein_coding	ENSG0000006800	8692	5321
1305	IQSEC1	-1.072	0.001	IQ motif and Sec7 domain 1 [Source:HGNC Symbol;Acc:HGNC:29112]	chr3:12938718-13114617	NM_001134382, NM	XM_011534315, XM_0	NP_001127854, NP_05	XP_011532606, XP_0115	protein_coding	ENSG0000014471	9922	29112
1306	KPNA2	0.788	0.001	karyopherin subunit alpha 2 [Source:HGNC Symbol;Acc:HGNC:6395]	chr17:66031634-66042958	NM_001320611, NM	XM_011524783	NP_001307540, NP_00	XP_011523085	protein_coding	ENSG0000018248	3838	6395
1307	METAP2	1.058	0.001	methionyl aminopeptidase 2 [Source:HGNC Symbol;Acc:HGNC:16672]	chr12:95867726-95909615	NM_006838	XM_005268583	NP_001304111, NP_00	XP_005268640	protein_coding	ENSG0000011114	10988	16672
1308	MPST	-1.431	0.001	mercaptopyruvate sulfurtransferase [Source:HGNC Symbol;Acc:HGNC:7223]	chr22:37406899-37425863	NM_001013436, NM	XM_011530196, XM_0	NP_001013454, NP_00	XP_005261667, XP_0115	protein_coding	ENSG0000012830	4357	7223
1309	NCKAP1	0.962	0.001	NCK associated protein 1 [Source:HGNC Symbol;Acc:HGNC:7666]	chr2:183773842-183903586	NM_013436, NM_2	XM_006712201, XM_0	NP_038464, NP_99531	XP_006712263, XP_0067	protein_coding	ENSG0000006167	10787	7666
1310	NEIL3	1.309	0.001	nei like DNA glycosylase 3 [Source:HGNC Symbol;Acc:HGNC:24573]	chr4:178230989-178284097	NM_018248	.	NP_060718	.	protein_coding	ENSG0000010967	55247	24573
1311	NETO2	1.087	0.001	neuropilin and tolloid like 2 [Source:HGNC Symbol;Acc:HGNC:14644]	chr16:47111613-47177908	NM_001201477, NM	XM_006721291, XM_0	NP_001188406, NP_06	XP_006721352, XP_0067	protein_coding	ENSG0000017120	81831	14644
1312	NUP35	1.169	0.001	nucleoporin 35 [Source:HGNC Symbol;Acc:HGNC:29797]	chr2:183982241-184026408	NM_001287584, NM	XM_011510578, XM_0	NP_001274513, NP_00	XP_006712317, XP_0115	protein_coding	ENSG0000016300	129401	29797
1313	PANK3	1.025	0.001	pantothenate kinase 3 [Source:HGNC Symbol;Acc:HGNC:19365]	chr5:167975499-168006605	NM_024594	.	NP_078870	.	protein_coding	ENSG0000012013	79646	19365
1314	PRDX5	-0.805	0.001	peroxiredoxin 5 [Source:HGNC Symbol;Acc:HGNC:9355]	chr11:64085559-64089283	NM_012094, NM_1	XM_005273882	NP_036226, NP_85763	XP_005273939	protein_coding	ENSG0000012643	25824	9355
1315	SCAPER	1.350	0.001	S-phase cyclin A associated protein in the ER [Source:HGNC Symbol;Acc:HGNC:13081]	chr15:76640244-77197676	NM_001145923, NM	XM_017022273, XM_0	NP_001139395, NP_06	XP_005254474, XP_0052	protein_coding	ENSG0000014038	49855	13081
1316	SPSB3	-0.959	0.001	splA/ryanodine receptor domain and SOCS box containing 3 [Source:HGNC Symbol;Acc:	chr16:1826712-1832821	NM_080861	XM_011522733, XM_0	NP_001311010, NP_54	XP_005255730, XP_0115	protein_coding	ENSG0000016203	90864	30629
1317	TMEM161B	1.386	0.001	transmembrane protein 161B [Source:HGNC Symbol;Acc:HGNC:28483]	chr5:87485449-87564674	NM_001289007, NM	XM_011543206, XM_0	NP_001275936, NP_00	XP_006714616, XP_0067	protein_coding	ENSG0000016418	153396	28483
1318	TMEM40	-1.977	0.001	transmembrane protein 40 [Source:HGNC Symbol;Acc:HGNC:25620]	chr3:12775023-12810956	NM_001284406, NM	XM_011533937, XM_0	NP_001271335, NP_00	XP_006713312, XP_0115	protein_coding	ENSG0000008872	55287	25620
1319	UBALD2	-0.886	0.001	UBA like domain containing 2 [Source:HGNC Symbol;Acc:HGNC:28438]	chr17:74261282-74267380	NM_182565	.	NP_872371	.	protein_coding	ENSG0000018526	283991	28438
1320	XPO4	1.001	0.001	exportin 4 [Source:HGNC Symbol;Acc:HGNC:17796]	chr13:21351467-21476949	NM_022459	XM_011535200, XM_0	NP_071904	XP_005266559, XP_0052	protein_coding	ENSG0000013295	64328	17796
1321	ASH1L	1.118	0.001	ASH1 like histone lysine methyltransferase [Source:HGNC Symbol;Acc:HGNC:19088]	chr1:155305058-155532598	NM_018489	XM_017001785, XM_0	NP_060959	XP_006711513, XP_0067	protein_coding	ENSG0000011653	693140	19088
1322	CBX3	0.796	0.001	chromobox 3 [Source:HGNC Symbol;Acc:HGNC:1553]	chr7:26240781-26252976	NM_007276, NM_0	XM_005249612, XM_0	NP_009207, NP_05767	XP_005249668, XP_0052	protein_coding	ENSG0000012256	11335	1553
1323	CCDC15	1.645	0.001	coiled-coil domain containing 15 [Source:HGNC Symbol;Acc:HGNC:25798]	chr11:124824016-124911385	NM_025004	.	NP_079280	.	protein_coding	ENSG0000014954	80071	25798
1324	DAAM1	1.192	0.001	dishevelled associated activator of morphogenesis 1 [Source:HGNC Symbol;Acc:HGNC:1	chr14:59655397-59838123	NM_001270520, NM	XM_005267431, XM_0	NP_001257449, NP_05	XP_005267487, XP_0052	protein_coding	ENSG0000010059	23002	18142
1325	DNAJC3	1.059	0.001	DnaJ heat shock protein family (Hsp40) member C3 [Source:HGNC Symbol;Acc:HGNC:94	chr13:96329392-96447243	NM_006260	XM_017020674, XM_0	NP_006251	XP_011519406, XP_0115	protein_coding	ENSG0000010258	5611	9439
1326	EEF1D	-0.819	0.001	eukaryotic translation elongation factor 1 delta [Source:HGNC Symbol;Acc:HGNC:3211]	chr8:144661866-144679914	NM_001130053, NM	XM_011516906, XM_0	NP_001123525, NP_00	XP_005250877, XP_0052	protein_coding	ENSG0000010452	1936	3211
1327	GPCPD1	1.279	0.001	glycerophosphocholine phosphodiesterase 1 [Source:HGNC Symbol;Acc:HGNC:26957]	chr20:5525049-5591672	NM_019593	XM_006723596, XM_0	NP_062539	XP_005260815, XP_0052	protein_coding	ENSG0000012577	56261	26957
1328	INO80B	-1.015	0.001	INO80 complex subunit B [Source:HGNC Symbol;Acc:HGNC:13324]	chr2:74682149-74685087	NM_031288	.	NP_112578	.	protein_coding	ENSG0000011527	83444	13324
1329	ITGB2	-1.494	0.001	integrin subunit beta 2 [Source:HGNC Symbol;Acc:HGNC:6155]	chr21:46305867-46348788	NM_000211, NM_0	XM_017028341, XM_0	NP_000202, NP_00112	XP_006724064, XP_0168	protein_coding	ENSG0000016025	3689	6155
1330	KBTBD2	0.901	0.001	kelch repeat and BTB domain containing 2 [Source:HGNC Symbol;Acc:HGNC:21751]	chr7:32907783-32931509	NM_015483	XM_011515256, XM_0	NP_056298	XP_005249753, XP_0052	protein_coding	ENSG0000017085	25948	21751
1331	KCNJ8	1.640	0.001	potassium voltage-gated channel subfamily J member 8 [Source:HGNC Symbol;Acc:HGN	chr12:21917888-21927755	NM_004982	XM_017019284, XM_0	NP_004973	XP_005253415, XP_0168	protein_coding	ENSG0000012136	3764	6269
1332	MYO15B	-0.928	0.001	myosin XVB [Source:HGNC Symbol;Acc:HGNC:14083]	chr17:73584138-73622564	.	.	.	.	protein_coding	ENSG0000026671	.	14083
1333	NFKB2	-0.824	0.001	nuclear factor kappa B subunit 2 [Source:HGNC Symbol;Acc:HGNC:7795]	chr10:104153866-104162286	NM_001077494, NM	XM_005269861, XM_0	NP_001070962, NP_00	XP_005269917, XP_0052	protein_coding	ENSG0000007715	4791	7795
1334	NR2F1	1.272	0.001	nuclear receptor subfamily 2 group F member 1 [Source:HGNC Symbol;Acc:HGNC:7975]	chr5:92919042-92930321	NM_005654	.	NP_005645	.	protein_coding	ENSG0000017574	7025	7975
1335	POC5	1.302	0.001	POC5 centriolar protein [Source:HGNC Symbol;Acc:HGNC:26658]	chr5:74969948-75013306	NM_001099271, NM	XM_011543160, XM_0	NP_001092741, NP_68	XP_005248493, XP_0115	protein_coding	ENSG0000015235	134359	26658
1336	RALGAPA1	1.320	0.001	Ral GTPase activating protein catalytic alpha subunit 1 [Source:HGNC Symbol;Acc:HGNC	chr14:36007557-36278510	NM_001283043, NM	XM_017021145, XM_0	NP_001269972, NP_00	XP_005267548, XP_0052	protein_coding	ENSG0000017437	253959	17770
1337	RANBP17	1.322	0.001	RAN binding protein 17 [Source:HGNC Symbol;Acc:HGNC:14428]	chr5:170288873-170727019	NM_022897	XM_011534635, XM_0	NP_075048	XP_011532929, XP_0115	protein_coding	ENSG0000020476	64901	14428
1338	SULT1A1	-1.074	0.001	sulfotransferase family 1A member 1 [Source:HGNC Symbol;Acc:HGNC:11453]	chr16:28616516-28634907	NM_001055, NM_1	XM_017023613, XM_0	NP_001046, NP_80356	XP_016879093, XP_0168	protein_coding	ENSG0000019650	6817	11453
1339	SYNJ1	1.227	0.001	synaptojanin 1 [Source:HGNC Symbol;Acc:HGNC:11503]	chr21:34001068-34100359	NM_001160302, NM	XM_017028505, XM_0	NP_001153774, NP_00	XP_016883983, XP_0168	protein_coding	ENSG0000015908	8867	11503
1340	TBX20	1.803	0.001	T-box 20 [Source:HGNC Symbol;Acc:HGNC:11598]	chr7:35242041-35293758	NM_001077653	.	NP_001071121	.	protein_coding	ENSG0000016453	57057	11598
1341	TTC21B	1.332	0.001	tetratricopeptide repeat domain 21B [Source:HGNC Symbol;Acc:HGNC:25660]	chr2:166729871-166810353	NM_024753	XM_017004969, XM_0	NP_079029	XP_016860456, XP_0168	protein_coding	ENSG0000012360	79809	25660
1342	UGT8	1.099	0.001	UDP glycosyltransferase 8 [Source:HGNC Symbol;Acc:HGNC:12555]	chr4:115519610-115599380	NM_001128174, NM	XM_011532232, XM_0	NP_001121646, NP_00	XP_006714365, XP_0067	protein_coding	ENSG0000017460	7368	12555
1343	ZBTB41	1.434	0.001	zinc finger and BTB domain containing 41 [Source:HGNC Symbol;Acc:HGNC:24819]	chr1:197122809-197169672	NM_194314	.	NP_919290	.	protein_coding	ENSG0000017788	360023	24819
1344	ZDHHC13	1.109	0.001	zinc finger DHHC-type containing 13 [Source:HGNC Symbol;Acc:HGNC:18413]	chr11:19138675-19197962	NM_001001483, NM	XM_011520195, XM_0	NP_001001483, NP_06	XP_005253053, XP_0115	protein_coding	ENSG0000017705	54503	18413
1345	CACNG6	1.230	0.001	calcium voltage-gated channel auxiliary subunit gamma 6 [Source:HGNC Symbol;Acc:HG	chr19:54495541-54515923	NM_031897, NM_1	.	NP_114103, NP_66581	.	protein_coding	ENSG0000013043	59285	13625
1346	CDK13	1.089	0.001	cyclin dependent kinase 13 [Source:HGNC Symbol;Acc:HGNC:1733]	chr7:39989635-40136733	NM_003718, NM_0	.	NP_003709, NP_11255	.	protein_coding	ENSG0000006588	8621	1733
1347	CENPC	1.420	0.001	centromere protein C [Source:HGNC Symbol;Acc:HGNC:1854]	chr4:68334465-68411324	NM_001812	XM_011531541, XM_0	NP_001803	XP_006714127	protein_coding	ENSG0000014524	1060	1854
1348	CPNE8	1.664	0.001	copine 8 [Source:HGNC Symbol;Acc:HGNC:23498]	chr12:39045986-39301232	NM_153634	XM_017018852	NP_705898	XP_016874340, XP_0168	protein_coding	ENSG0000013911	144402	23498

1349	CTSH	-1.486	0.001	cathepsin H [Source:HGNC Symbol;Acc:HGNC:2535]	chr15:79213399-79237433	NM_001319137, NN	XM_005254181	NP_001306066, NP_00	XP_005254238, XP_0168	protein_coding	ENSG0000010381	1512	2535
1350	DCLK1	0.879	0.001	doublecortin like kinase 1 [Source:HGNC Symbol;Acc:HGNC:2700]	chr13:36342788-36705443	NM_001195415, NN	XM_017020847, XM_0	NP_001182344, NP_00	XP_005266649, XP_0067	protein_coding	ENSG0000013308	9201	2700
1351	DNAJB14	1.336	0.001	DnaJ heat shock protein family (Hsp40) member B14 [Source:HGNC Symbol;Acc:HGNC:2	chr4:100817404-100867883	NM_001031723, NN	XM_011532261	NP_001026893, NP_00	.	protein_coding	ENSG0000016403	79982	25881
1352	ECSIT	-1.040	0.001	ECSIT signalling integrator [Source:HGNC Symbol;Acc:HGNC:29548]	chr19:11594241-11639989	NM_001142464, NN	.	NP_001135936, NP_00	.	protein_coding	ENSG0000013015	51295	29548
1353	EFHD2	-0.798	0.001	EF-hand domain family member D2 [Source:HGNC Symbol;Acc:HGNC:28670]	chr1:15736390-15756839	NM_024329	.	NP_077305	.	protein_coding	ENSG0000014263	79180	28670
1354	FZD6	1.184	0.001	frizzled class receptor 6 [Source:HGNC Symbol;Acc:HGNC:4044]	chr8:104310660-104345087	NM_001164615, NN	.	NP_001158087, NP_00	.	protein_coding	ENSG0000016493	8323	4044
1355	HIBADH	0.942	0.001	3-hydroxyisobutyrate dehydrogenase [Source:HGNC Symbol;Acc:HGNC:4907]	chr7:27565060-27702614	NM_152740	.	NP_689953	.	protein_coding	ENSG0000010604	11112	4907
1356	HIPK2	0.996	0.001	homeodomain interacting protein kinase 2 [Source:HGNC Symbol;Acc:HGNC:14402]	chr7:139246315-139477577	NM_001113239, NN	XM_017012069, XM_0	NP_001106710, NP_07	XP_006715998, XP_0115	protein_coding	ENSG0000006439	28996	14402
1357	KLK3	-2.518	0.001	kallikrein related peptidase 3 [Source:HGNC Symbol;Acc:HGNC:6364]	chr19:51358170-51364020	NM_001030047, NN	XM_011526924, XM_0	NP_001025218, NP_00	.	protein_coding	ENSG0000014251	354	6364
1358	LRPPRC	1.113	0.001	leucine rich pentatricopeptide repeat containing [Source:HGNC Symbol;Acc:HGNC:1571	chr2:44113646-44223144	NM_133259	XM_011532474, XM_0	NP_573566	XP_006711978, XP_0115	protein_coding	ENSG0000013809	10128	15714
1359	LRRC8D	1.061	0.001	leucine rich repeat containing 8 family member D [Source:HGNC Symbol;Acc:HGNC:169	chr1:90286590-90402170	NM_001134479, NN	XM_011541690, XM_0	NP_001127951, NP_06	XP_011539991, XP_0115	protein_coding	ENSG0000017149	55144	16992
1360	MTF1	1.025	0.001	metal regulatory transcription factor 1 [Source:HGNC Symbol;Acc:HGNC:7428]	chr1:38275238-38325292	NM_005955	XM_011541494, XM_0	NP_005946	XP_011539793, XP_0115	protein_coding	ENSG0000018878	4520	7428
1361	MYO1B	1.049	0.001	myosin IB [Source:HGNC Symbol;Acc:HGNC:7596]	chr2:192110001-192290115	NM_001130158, NN	XM_017004158, XM_0	NP_001123630, NP_00	XP_005246629, XP_0067	protein_coding	ENSG0000012864	4430	7596
1362	OXGR1	1.680	0.001	oxoglutarate receptor 1 [Source:HGNC Symbol;Acc:HGNC:4531]	chr13:97637972-97646623	NM_080818	XM_011521077, XM_0	NP_543008	XP_005254100, XP_0052	protein_coding	ENSG0000016562	27199	4531
1363	PARP8	1.278	0.001	poly(ADP-ribose) polymerase family member 8 [Source:HGNC Symbol;Acc:HGNC:26124	chr5:49961732-50142356	NM_001178055, NN	XM_017009851, XM_0	NP_001171526, NP_00	XP_005248653, XP_0115	protein_coding	ENSG0000015188	79668	26124
1364	PCNX1	1.040	0.001	pecanex homolog 1 (Drosophila) [Source:HGNC Symbol;Acc:HGNC:19740]	chr14:71374121-71582099	NM_001308160, NN	XM_006720082, XM_0	NP_001295089, NP_05	XP_005267477, XP_0052	protein_coding	ENSG0000010073	22990	19740
1365	PTPN3	0.901	0.001	protein tyrosine phosphatase, non-receptor type 3 [Source:HGNC Symbol;Acc:HGNC:96	chr9:112137973-112260593	NM_001145368, NN	XM_017014955, XM_0	NP_001138840, NP_00	XP_006717260, XP_0067	protein_coding	ENSG0000007015	5774	9655
1366	RABGAP1L	1.327	0.001	RAB GTPase activating protein 1 like [Source:HGNC Symbol;Acc:HGNC:24663]	chr1:174128547-174964445	NM_001035230, NN	XM_017002997, XM_0	NP_001030307, NP_00	XP_016858483, XP_0168	protein_coding	ENSG0000015206	9910	24663
1367	RHOT1	1.191	0.001	ras homolog family member T1 [Source:HGNC Symbol;Acc:HGNC:21168]	chr17:30469472-30552789	NM_001033566, NN	XM_017024821, XM_0	NP_001028738, NP_00	XP_011523271, XP_0115	protein_coding	ENSG0000012685	55288	21168
1368	TRAF6	1.172	0.001	TNF receptor associated factor 6 [Source:HGNC Symbol;Acc:HGNC:12036]	chr11:36508576-36531822	NM_004620, NM_1	.	NP_004611, NP_66580	.	protein_coding	ENSG0000017510	7189	12036
1369	TRIP11	1.529	0.001	thyroid hormone receptor interactor 11 [Source:HGNC Symbol;Acc:HGNC:12305]	chr14:92432334-92572965	NM_001321851, NN	XM_006720321, XM_0	NP_001308780, NP_00	XP_016877277	protein_coding	ENSG0000010081	9321	12305
1370	ARCN1	0.933	0.001	archain 1 [Source:HGNC Symbol;Acc:HGNC:649]	chr11:118443104-118473748	NM_001142281, NN	XM_005271542	NP_001135753, NP_00	XP_005271599	protein_coding	ENSG0000009513	372	649
1371	BPTF	1.018	0.001	bromodomain PHD finger transcription factor [Source:HGNC Symbol;Acc:HGNC:3581]	chr17:65821779-65980494	NM_004459, NM_1	XM_011524526, XM_0	NP_004450, NP_87257	XP_005257207, XP_0052	protein_coding	ENSG0000017163	2186	3581
1372	BTBD10	1.093	0.001	BTB domain containing 10 [Source:HGNC Symbol;Acc:HGNC:21445]	chr11:13409547-13484844	NM_001297741, NN	XM_017018408, XM_0	NP_001284670, NP_00	XP_011518706, XP_0168	protein_coding	ENSG0000014892	84280	21445
1373	DDX1	0.864	0.001	DEAD/H-box helicase 1 [Source:HGNC Symbol;Acc:HGNC:2734]	chr2:15731301-15771235	NM_004939	.	NP_004930	.	protein_coding	ENSG0000007978	1653	2734
1374	DHX29	1.172	0.001	DEAH-box helicase 29 [Source:HGNC Symbol;Acc:HGNC:15815]	chr5:54552072-54603550	NM_019030	XM_011543494, XM_0	NP_061903	XP_006714716	protein_coding	ENSG0000006724	54505	15815
1375	DPY19L1	1.006	0.001	dpy-19 like 1 [Source:HGNC Symbol;Acc:HGNC:22205]	chr7:34968487-35077653	NM_015283	XM_011515246, XM_0	NP_056098	XP_011513547, XP_0115	protein_coding	ENSG0000017385	23333	22205
1376	EDRF1	1.242	0.001	erythroid differentiation regulatory factor 1 [Source:HGNC Symbol;Acc:HGNC:24640]	chr10:127408144-127452708	NM_001202438, NN	XM_011539621	NP_001189367, NP_05	.	protein_coding	ENSG0000010793	26098	24640
1377	EXOC2	1.070	0.001	exocyst complex component 2 [Source:HGNC Symbol;Acc:HGNC:24968]	chr6:485132-693111	NM_018303	XM_017011021, XM_0	NP_060773	XP_016866507, XP_0168	protein_coding	ENSG0000011268	55770	24968
1378	FOS	-0.950	0.001	Fos proto-oncogene, AP-1 transcription factor subunit [Source:HGNC Symbol;Acc:HGNC	chr14:75745476-75748933	NM_005252	.	NP_005243	.	protein_coding	ENSG0000017034	2353	3796
1379	GEMIN8	-1.188	0.001	gem nuclear organelle associated protein 8 [Source:HGNC Symbol;Acc:HGNC:26044]	chrX:14026397-14048012	NM_001042479, NN	.	NP_001035944, NP_00	XP_016885106, XP_0168	protein_coding	ENSG0000004664	54960	26044
1380	HOXC13	-1.625	0.001	homeobox C13 [Source:HGNC Symbol;Acc:HGNC:5125]	chr12:54332548-54340328	NM_017410	.	NP_059106	.	protein_coding	ENSG0000012336	3229	5125
1381	HSD17B8	-1.048	0.001	hydroxysteroid 17-beta dehydrogenase 8 [Source:HGNC Symbol;Acc:HGNC:3554]	chr6:33172418-33174608	NM_014234	.	NP_055049	.	protein_coding	ENSG0000020422	7923	3554
1382	ICE1	1.030	0.001	interactor of little elongation complex ELL subunit 1 [Source:HGNC Symbol;Acc:HGNC:29	chr5:5422806-5490347	NM_015325	.	NP_056140	XP_011512301	protein_coding	ENSG0000016415	23379	29154
1383	ITGB5	-0.791	0.001	integrin subunit beta 5 [Source:HGNC Symbol;Acc:HGNC:6160]	chr3:124480794-124606144	NM_002213	XM_006713630, XM_0	NP_002204	XP_005247493, XP_0067	protein_coding	ENSG0000008278	3693	6160
1384	NRIP3	0.896	0.001	nuclear receptor interacting protein 3 [Source:HGNC Symbol;Acc:HGNC:1167]	chr11:9002122-9025596	NM_020645	XM_011520221	NP_065696	XP_011518523	protein_coding	ENSG0000017535	56675	1167
1385	PCSK5	1.085	0.001	proprotein convertase subtilisin/kexin type 5 [Source:HGNC Symbol;Acc:HGNC:8747]	chr9:78505559-78977255	NM_001190482, NN	XM_011518770, XM_0	NP_001177411, NP_00	XP_005252096, XP_0115	protein_coding	ENSG0000009913	5125	8747
1386	PIGK	1.094	0.001	phosphatidylinositol glycan anchor biosynthesis class K [Source:HGNC Symbol;Acc:HGN(	chr1:77554674-77685115	NM_005482	.	NP_005473	.	protein_coding	ENSG0000014289	10026	8965
1387	PRKAG2	1.083	0.001	protein kinase AMP-activated non-catalytic subunit gamma 2 [Source:HGNC Symbol;Acc	chr7:151253196-151574210	NM_001040633, NN	XM_017012273, XM_0	NP_001035723, NP_00	XP_005250059, XP_0052	protein_coding	ENSG0000010661	51422	9386
1388	SDAD1	1.019	0.001	SDA1 domain containing 1 [Source:HGNC Symbol;Acc:HGNC:25537]	chr4:76871068-76912113	NM_001288983, NN	XM_005263105, XM_0	NP_001275912, NP_00	XP_005263159, XP_0052	protein_coding	ENSG0000019830	55153	25537
1389	SMC6	1.467	0.001	structural maintenance of chromosomes 6 [Source:HGNC Symbol;Acc:HGNC:20466]	chr2:17845078-17981462	NM_001142286, NN	XM_011533108, XM_0	NP_001135758, NP_07	XP_011531409, XP_0115	protein_coding	ENSG0000016302	79677	20466
1390	TMEM60	1.127	0.001	transmembrane protein 60 [Source:HGNC Symbol;Acc:HGNC:21754]	chr7:77423044-77427897	NM_032936	.	NP_116325	.	protein_coding	ENSG0000013521	85025	21754
1391	TRIM44	0.850	0.001	tripartite motif containing 44 [Source:HGNC Symbol;Acc:HGNC:19016]	chr11:35684352-35839557	NM_017583	.	NP_060053	.	protein_coding	ENSG0000016632	54765	19016
1392	ZNF160	1.139	0.001	zinc finger protein 160 [Source:HGNC Symbol;Acc:HGNC:12948]	chr19:53569858-53606687	NM_001102603, NN	XM_011527466, XM_0	NP_001096073, NP_00	XP_005259435, XP_0052	protein_coding	ENSG0000017094	90338	12948
1393	ZNF165	1.329	0.001	zinc finger protein 165 [Source:HGNC Symbol;Acc:HGNC:12953]	chr6:28048752-28057341	NM_003447	.	NP_003438	XP_016866747, XP_0168	protein_coding	ENSG0000019727	7718	12953
1394	AKR1A1	-0.866	0.001	aldo-keto reductase family 1 member A1 [Source:HGNC Symbol;Acc:HGNC:380]	chr1:46016214-46035721	NM_001202413, NN	XM_011540492	NP_001189342, NP_00	XP_011538794	protein_coding	ENSG0000011744	10327	380
1395	ANKRD65	-1.975	0.001	ankyrin repeat domain 65 [Source:HGNC Symbol;Acc:HGNC:42950]	chr1:1353799-1357149	NM_001145210, NN	XM_011541490, XM_0	NP_001138682, NP_00	XP_005244809, XP_0067	protein_coding	ENSG0000023509	441869	42950
1396	CCR3	-2.213	0.001	C-C motif chemokine receptor 3 [Source:HGNC Symbol;Acc:HGNC:1604]	chr3:46205095-46308197	NM_001164680, NN	XM_011533335, XM_0	NP_001158152, NP_00	XP_006713023, XP_0115	protein_coding	ENSG0000018362	1232	1604
1397	DOCK7	1.189	0.001	dedicator of cytokinesis 7 [Source:HGNC Symbol;Acc:HGNC:19190]	chr1:62920396-63154039	NM_001271999, NN	XM_017002639, XM_0	NP_001258928, NP_00	XP_005271349, XP_0115	protein_coding	ENSG0000011664	85440	19190
1398	FAM213B	-0.885	0.001	family with sequence similarity 213 member B [Source:HGNC Symbol;Acc:HGNC:28390]	chr1:2518188-2564429	NM_001195736, NN	XM_011540666, XM_0	NP_001182665, NP_00	XP_006710417, XP_0115	protein_coding	ENSG0000015787	127281	28390
1399	FBXO2	-0.950	0.001	F-box protein 2 [Source:HGNC Symbol;Acc:HGNC:13581]	chr1:11708423-11723384	NM_012168	.	NP_036300	.	protein_coding	ENSG0000011666	26232	13581
1400	GTDC1	1.390	0.001	glycosyltransferase like domain containing 1 [Source:HGNC Symbol;Acc:HGNC:20887]	chr2:144695634-145090101	NM_001006636, NN	XM_017004933, XM_0	NP_001006637, NP_00	XP_005263831, XP_0052	protein_coding	ENSG0000012196	79712	20887
1401	HPCAL1	-0.854	0.001	hippocalcin like 1 [Source:HGNC Symbol;Acc:HGNC:5145]	chr2:10443032-10567743	NM_001258357, NN	XM_011510349, XM_0	NP_001245286, NP_00	XP_005246217, XP_0052	protein_coding	ENSG0000011575	3241	5145
1402	INPP4B	2.108	0.001	inositol polyphosphate-4-phosphatase type II B [Source:HGNC Symbol;Acc:HGNC:6075]	chr4:142944312-143767453	NM_001101669, NN	XM_011532393, XM_0	NP_001095139, NP_00	XP_005263380, XP_0052	protein_coding	ENSG0000010945	8821	6075

1403	ITSN2	1.098	0.001	intersectin 2 [Source:HGNC Symbol;Acc:HGNC:6184]	chr2:24425732-24583583	NM_006277, NM_0	.	NP_006268, NP_06254	.	protein_coding	ENSG0000019839	50618	6184
1404	KIAA1551	1.371	0.001	KIAA1551 [Source:HGNC Symbol;Acc:HGNC:25559]	chr12:32112352-32146041	NM_018169	XM_011520722, XM_0	NP_060639	XP_005253462, XP_0067	protein_coding	ENSG0000017471	55196	25559
1405	KLK6	-0.820	0.001	kallikrein related peptidase 6 [Source:HGNC Symbol;Acc:HGNC:6367]	chr19:51461886-51472929	NM_001012964, NM	.	NP_001012982, NP_00	.	protein_coding	ENSG0000016775	5653	6367
1406	LRRFIP1	0.928	0.001	LRR binding FLII interacting protein 1 [Source:HGNC Symbol;Acc:HGNC:6702]	chr2:238536218-238688958	NM_001137550, NM	XM_011512166, XM_0	NP_001131022, NP_00	XP_016860742, XP_0168	protein_coding	ENSG0000012483	9208	6702
1407	PRRG2	-1.130	0.001	proline rich and Gla domain 2 [Source:HGNC Symbol;Acc:HGNC:9470]	chr19:50084582-50094272	NM_000951, NM_0	XM_011527124, XM_0	NP_000942, NP_00130	XP_006723349, XP_0115	protein_coding	ENSG0000012646	5639	9470
1408	RAPGEF2	1.135	0.001	Rap guanine nucleotide exchange factor 2 [Source:HGNC Symbol;Acc:HGNC:16854]	chr4:160188888-160281321	NM_014247	XM_011532427, XM_0	NP_055062	XP_006714485, XP_0115	protein_coding	ENSG0000010975	9693	16854
1409	RBM24	1.613	0.001	RNA binding motif protein 24 [Source:HGNC Symbol;Acc:HGNC:21539]	chr6:17281576-17294106	NM_001143941, NM	XM_011514387, XM_0	NP_001137413, NP_00	XP_005248989, XP_0115	protein_coding	ENSG0000011218	221662	21539
1410	SERPINH1	-0.792	0.001	serpin family H member 1 [Source:HGNC Symbol;Acc:HGNC:1546]	chr11:75273100-75283828	NM_001207014, NM	XM_011545327, XM_0	NP_001193943, NP_00	XP_006718792, XP_0115	protein_coding	ENSG0000014925	871	1546
1411	TANC2	1.057	0.001	tetratricopeptide repeat, ankyrin repeat and coiled-coil containing 2 [Source:HGNC Sym	chr17:61086916-61505060	NM_025185	XM_017024429, XM_0	NP_079461	XP_005257260, XP_0067	protein_coding	ENSG0000017092	26115	30212
1412	TMEM141	-0.880	0.001	transmembrane protein 141 [Source:HGNC Symbol;Acc:HGNC:28211]	chr9:139685806-139687709	NM_032928	.	NP_116317	.	protein_coding	ENSG0000024418	85014	28211
1413	TMEM86A	-1.593	0.001	transmembrane protein 86A [Source:HGNC Symbol;Acc:HGNC:26890]	chr11:18720337-18747727	NM_153347	.	NP_699178	.	protein_coding	ENSG0000015111	144110	26890
1414	ZNF600	1.163	0.001	zinc finger protein 600 [Source:HGNC Symbol;Acc:HGNC:30951]	chr19:53267447-53290016	NM_198457	XM_011526566	NP_001308795, NP_00	XP_011524868, XP_0168	protein_coding	ENSG0000018919	162966	30951
1415	ACTL8	-2.428	0.001	actin like 8 [Source:HGNC Symbol;Acc:HGNC:24018]	chr1:18081807-18153558	NM_030812	XM_011542214, XM_0	NP_110439	XP_011540514, XP_0115	protein_coding	ENSG0000011714	81569	24018
1416	ALOX12B	-1.231	0.001	arachidonate 12-lipoxygenase, 12R type [Source:HGNC Symbol;Acc:HGNC:430]	chr17:7975953-7991021	NM_001139	.	NP_001130	.	protein_coding	ENSG0000017947	242	430
1417	ARL6IP4	-0.916	0.001	ADP ribosylation factor like GTPase 6 interacting protein 4 [Source:HGNC Symbol;Acc:H	chr12:123405497-123467460	NM_001002251, NM	.	NP_001002251, NP_00	.	protein_coding	ENSG0000018219	51329	18076
1418	CALM3	-0.760	0.001	calmodulin 3 [Source:HGNC Symbol;Acc:HGNC:1449]	chr19:47021935-47114050	NM_005184	.	NP_001292554, NP_00	.	protein_coding	ENSG0000016001	808	1449
1419	CITED1	-1.389	0.001	Cbp/p300 interacting transactivator with Glu/Asp rich carboxy-terminal domain 1 [Sour	chrX:71521487-71527037	NM_001144885, NM	XM_011530958	NP_001138357, NP_00	XP_011529260	protein_coding	ENSG0000012593	4435	1986
1420	COIL	0.999	0.001	coilin [Source:HGNC Symbol;Acc:HGNC:2184]	chr17:55015547-55038415	NM_004645	.	NP_004636	.	protein_coding	ENSG0000012105	8161	2184
1421	CRYL1	-1.008	0.001	crystallin lambda 1 [Source:HGNC Symbol;Acc:HGNC:18246]	chr13:20977805-21099996	NM_015974	XM_005266415	NP_057058	XP_005266472	protein_coding	ENSG0000016547	51084	18246
1422	DDHD2	1.264	0.001	DDHD domain containing 2 [Source:HGNC Symbol;Acc:HGNC:29106]	chr8:38088860-38120351	NM_001164232, NM	XM_017013252, XM_0	NP_001157704, NP_05	XP_005273511, XP_0052	protein_coding	ENSG0000008578	23259	29106
1423	DIP2B	0.895	0.001	disco interacting protein 2 homolog B [Source:HGNC Symbol;Acc:HGNC:29284]	chr12:50898889-51142450	NM_173602	XM_017019705, XM_0	NP_775873	XP_005269101, XP_0067	protein_coding	ENSG0000006608	57609	29284
1424	INPP5K	-0.857	0.001	inositol polyphosphate-5-phosphatase K [Source:HGNC Symbol;Acc:HGNC:33882]	chr17:1397864-1420182	NM_001135642, NM	XM_017024758, XM_0	NP_001129114, NP_05	XP_005256740, XP_0052	protein_coding	ENSG0000013237	51763	33882
1425	KRT80	-0.804	0.001	keratin 80 [Source:HGNC Symbol;Acc:HGNC:27056]	chr12:52562779-52585784	NM_001081492, NM	.	NP_001074961, NP_87	.	protein_coding	ENSG0000016776	144501	27056
1426	MAP7D2	-0.930	0.001	MAP7 domain containing 2 [Source:HGNC Symbol;Acc:HGNC:25899]	chrX:20024830-20135035	NM_001168465, NM	XM_011545485, XM_0	NP_001161937, NP_00	XP_005274535, XP_0052	protein_coding	ENSG0000018436	256714	25899
1427	POLD1	-0.810	0.001	DNA polymerase delta 1, catalytic subunit [Source:HGNC Symbol;Acc:HGNC:9175]	chr19:50887579-50934570	NM_001256849, NM	XM_017026881, XM_0	NP_001243778, NP_00	XP_005259065, XP_0115	protein_coding	ENSG0000006282	5424	9175
1428	RAPGEF5	1.081	0.001	Rap guanine nucleotide exchange factor 5 [Source:HGNC Symbol;Acc:HGNC:16862]	chr7:22157855-22396763	NM_012294	XM_017012837, XM_0	NP_036426	XP_005249971, XP_0052	protein_coding	ENSG0000013623	9771	16862
1429	SIK2	1.009	0.001	salt inducible kinase 2 [Source:HGNC Symbol;Acc:HGNC:21680]	chr11:111473114-111637151	NM_015191	XM_011542716	NP_056006	XP_016872906	protein_coding	ENSG0000017014	23235	21680
1430	SLC16A1	0.842	0.001	solute carrier family 16 member 1 [Source:HGNC Symbol;Acc:HGNC:10922]	chr1:113454468-113498975	NM_001166496, NM	XM_011542027, XM_0	NP_001159968, NP_00	XP_011540328, XP_0115	protein_coding	ENSG0000015538	6566	10922
1431	SLIT1	-1.073	0.001	slit guidance ligand 1 [Source:HGNC Symbol;Acc:HGNC:11085]	chr10:98757794-98945677	NM_003061	.	NP_003052	.	protein_coding	ENSG0000018712	6585	11085
1432	SYT17	-1.730	0.001	synaptotagmin 17 [Source:HGNC Symbol;Acc:HGNC:24119]	chr16:19179570-19279656	NM_001308157, NM	XM_011545872, XM_0	NP_001295086, NP_05	XP_005255419, XP_0115	protein_coding	ENSG0000010352	51760	24119
1433	TMEM256	-0.870	0.001	transmembrane protein 256 [Source:HGNC Symbol;Acc:HGNC:28618]	chr17:7306293-7307456	NM_152766	.	NP_689979	.	protein_coding	ENSG0000020554	254863	28618
1434	ZNF106	0.935	0.001	zinc finger protein 106 [Source:HGNC Symbol;Acc:HGNC:12886]	chr15:42705020-42783303	NM_001284306, NM	XM_005254592, XM_0	NP_001271235, NP_00	XP_005254648, XP_0052	protein_coding	ENSG0000010399	64397	12886
1435	ZNF322	1.327	0.001	zinc finger protein 322 [Source:HGNC Symbol;Acc:HGNC:23640]	chr6:26634610-26659980	NM_001242797, NM	.	NP_001229726, NP_00	.	protein_coding	ENSG0000018131	79692	23640
1436	ZNF519	1.560	0.001	zinc finger protein 519 [Source:HGNC Symbol;Acc:HGNC:30574]	chr18:14099936-14132429	NM_145287	XM_011525604	NP_660330	XP_011523906, XP_0168	protein_coding	ENSG0000017532	162655	30574
1437	ZNF524	-1.044	0.001	zinc finger protein 524 [Source:HGNC Symbol;Acc:HGNC:28322]	chr19:56111734-56114504	NM_153219	.	NP_694951	.	protein_coding	ENSG0000017144	147807	28322
1438	ZNF664	0.842	0.001	zinc finger protein 664 [Source:HGNC Symbol;Acc:HGNC:25406]	chr12:124457669-124573140	NM_001204298, NM	.	NP_001191227, NP_68	.	protein_coding	ENSG0000017919	144348	25406
1439	ANAPC1	0.798	0.001	anaphase promoting complex subunit 1 [Source:HGNC Symbol;Acc:HGNC:19988]	chr2:112525213-112642267	NM_022662	XM_011511635, XM_0	NP_073153	XP_011509935, XP_0115	protein_coding	ENSG0000015310	64682	19988
1440	ATG2A	-1.079	0.001	autophagy related 2A [Source:HGNC Symbol;Acc:HGNC:29028]	chr11:64662017-64684720	NM_015104	XM_011544866, XM_0	NP_055919	XP_005273906, XP_0052	protein_coding	ENSG0000011004	23130	29028
1441	CDK19	1.211	0.001	cyclin dependent kinase 19 [Source:HGNC Symbol;Acc:HGNC:19338]	chr6:110931180-111136840	NM_001300960, NM	XM_017010588, XM_0	NP_001287889, NP_00	XP_005266928, XP_0168	protein_coding	ENSG0000015511	23097	19338
1442	COL7A1	-1.233	0.001	collagen type VII alpha 1 chain [Source:HGNC Symbol;Acc:HGNC:2214]	chr3:48601505-48632700	NM_000094	XM_011533339, XM_0	NP_000085	XP_011531639, XP_0168	protein_coding	ENSG0000011427	100313843	2214
1443	CTDSP1	-0.891	0.001	CTD small phosphatase 1 [Source:HGNC Symbol;Acc:HGNC:21614]	chr2:219263060-219270664	NM_001206878, NM	.	NP_001193807, NP_06	.	protein_coding	ENSG0000014457	58190	21614
1444	DDX6	0.892	0.001	DEAD-box helicase 6 [Source:HGNC Symbol;Acc:HGNC:2747]	chr11:118618474-118661856	NM_001257191, NM	XM_011542645, XM_0	NP_001244120, NP_00	XP_005271474, XP_0115	protein_coding	ENSG0000011036	1656	2747
1445	DLG3	-0.856	0.001	discs large MAGUK scaffold protein 3 [Source:HGNC Symbol;Acc:HGNC:2902]	chrX:69664710-69725340	NM_001166278, NM	XM_011530883, XM_0	NP_001159750, NP_06	XP_005262305, XP_0067	protein_coding	ENSG0000008245	1741	2902
1446	EPS8L3	-0.880	0.001	EPS8 like 3 [Source:HGNC Symbol;Acc:HGNC:21297]	chr1:110292701-110306649	NM_001319952, NM	XM_017002329, XM_0	NP_001306881, NP_07	XP_011540434, XP_0115	protein_coding	ENSG0000019875	79574	21297
1447	GJB2	0.897	0.001	gap junction protein beta 2 [Source:HGNC Symbol;Acc:HGNC:4284]	chr13:20761608-20767037	NM_004004	XM_011535049	NP_003995	XP_011533351	protein_coding	ENSG0000016547	2706	4284
1448	GPSM2	1.005	0.001	G-protein signaling modulator 2 [Source:HGNC Symbol;Acc:HGNC:29501]	chr1:109358519-109506106	NM_013296	XM_011541302, XM_0	NP_001307967, NP_00	XP_005270844, XP_0067	protein_coding	ENSG0000012195	29899	29501
1449	HDAC8	-1.228	0.001	histone deacetylase 8 [Source:HGNC Symbol;Acc:HGNC:13315]	chrX:71549365-71792953	NM_001166418, NM	XM_011530986	NP_001159890, NP_00	XP_011529288	protein_coding	ENSG0000014709	55869	13315
1450	IL32	-1.091	0.001	interleukin 32 [Source:HGNC Symbol;Acc:HGNC:16830]	chr16:3115297-3119820	NM_001012631, NM	XM_005255686	NP_001012649, NP_00	XP_005255743	protein_coding	ENSG0000000851	9235	16830
1451	ITGAV	0.978	0.001	integrin subunit alpha V [Source:HGNC Symbol;Acc:HGNC:6150]	chr2:187454791-187545628	NM_001144999, NM	XM_006712513	NP_001138471, NP_00	.	protein_coding	ENSG0000013844	3685	6150
1452	KIF11	0.983	0.001	kinesin family member 11 [Source:HGNC Symbol;Acc:HGNC:6388]	chr10:94353042-94415152	NM_004523	.	NP_004514	.	protein_coding	ENSG0000013816	3832	6388
1453	KLHL9	1.152	0.001	kelch like family member 9 [Source:HGNC Symbol;Acc:HGNC:18732]	chr9:21329669-21335379	NM_018847	.	NP_061335	.	protein_coding	ENSG0000019864	55958	18732
1454	NOL11	0.918	0.001	nucleolar protein 11 [Source:HGNC Symbol;Acc:HGNC:24557]	chr17:65713948-65740647	NM_001303272, NM	.	NP_001290201, NP_05	.	protein_coding	ENSG0000013093	25926	24557
1455	RNF19A	1.213	0.001	ring finger protein 19A, RBR E3 ubiquitin protein ligase [Source:HGNC Symbol;Acc:HGN	chr8:101269287-101322555	NM_001280539, NM	XM_011516956, XM_0	NP_001267468, NP_05	XP_005250910, XP_0115	protein_coding	ENSG0000003467	25897	13432
1456	RPL22L1	-1.178	0.001	ribosomal protein L22 like 1 [Source:HGNC Symbol;Acc:HGNC:27610]	chr3:170582663-170588272	NM_001099645	XM_005247205	NP_001093115, NP_00	XP_005247262	protein_coding	ENSG0000016358	200916	27610

1457	SEC63	1.026	0.001	SEC63 homolog, protein translocation regulator [Source:HGNC Symbol;Acc:HGNC:21082	chr6:108188959-108279393	NM_007214	XM_011535399	NP_009145	XP_011533701	protein_coding	ENSG0000002579	11231	21082
1458	TDRD7	1.042	0.001	tudor domain containing 7 [Source:HGNC Symbol;Acc:HGNC:30831]	chr9:100174231-100258407	NM_001302884, NN	XM_006717026	NP_001289813, NP_05	XP_006717089	protein_coding	ENSG0000019611	23424	30831
1459	UGDH	0.990	0.001	UDP-glucose 6-dehydrogenase [Source:HGNC Symbol;Acc:HGNC:12525]	chr4:39500374-39529238	NM_001184700, NN	XM_005262667	NP_001171629, NP_00	XP_005262724	protein_coding	ENSG0000010981	7358	12525
1460	WDR76	1.152	0.001	WD repeat domain 76 [Source:HGNC Symbol;Acc:HGNC:25773]	chr15:44119160-44160617	NM_001167941, NN	XM_005254680	NP_001161413, NP_07	XP_005254737	protein_coding	ENSG0000009247	79968	25773
1461	ZBTB6	1.289	0.001	zinc finger and BTB domain containing 6 [Source:HGNC Symbol;Acc:HGNC:16764]	chr9:125670334-125675609	NM_006626	.	NP_006617	.	protein_coding	ENSG0000018613	10773	16764
1462	ZSCAN12	1.584	0.001	zinc finger and SCAN domain containing 12 [Source:HGNC Symbol;Acc:HGNC:13172]	chr6:28346731-28367511	.	.	NP_001156863	XP_011513316, XP_0115	protein_coding	ENSG0000015869	9753	13172
1463	ABCD1	-0.964	0.001	ATP binding cassette subfamily D member 1 [Source:HGNC Symbol;Acc:HGNC:61]	chrX:152990322-153010216	NM_000033	.	NP_000024	.	protein_coding	ENSG0000010198	215	61
1464	ACOT11	-1.090	0.001	acyl-CoA thioesterase 11 [Source:HGNC Symbol;Acc:HGNC:18156]	chr1:55013900-55104865	NM_015547, NM_1	.	NP_056362, NP_67151	.	protein_coding	ENSG0000016239	26027	18156
1465	BAP1	-0.814	0.001	BRCA1 associated protein 1 [Source:HGNC Symbol;Acc:HGNC:950]	chr3:52435028-52444366	NM_004656	XM_017007303, XM_0	NP_004647	XP_011532451, XP_0115	protein_coding	ENSG0000016393	8314	950
1466	CCDC167	-1.159	0.001	coiled-coil domain containing 167 [Source:HGNC Symbol;Acc:HGNC:21239]	chr6:37450695-37467698	NM_138493	.	NP_612502	.	protein_coding	ENSG0000019893	154467	21239
1467	CCDC51	-0.929	0.001	coiled-coil domain containing 51 [Source:HGNC Symbol;Acc:HGNC:25714]	chr3:48473573-48481529	NM_001256964, NN	.	NP_001243893, NP_00	.	protein_coding	ENSG0000016405	79714	25714
1468	CTR9	0.857	0.001	CTR9 homolog, Paf1/RNA polymerase II complex component [Source:HGNC Symbol;Acc	chr11:10772533-10801290	NM_014633	.	NP_055448	.	protein_coding	ENSG0000019873	9646	16850
1469	FAM73A	1.226	0.001	mitoguardin 1 [Source:HGNC Symbol;Acc:HGNC:24741]	chr1:78245308-78345224	NM_001270384, NN	XM_017001230, XM_0	NP_001257313, NP_94	XP_005270903, XP_0052	protein_coding	ENSG0000018048	374986	24741
1470	GNAZ	-1.337	0.001	G protein subunit alpha z [Source:HGNC Symbol;Acc:HGNC:4395]	chr22:23401592-23487208	NM_002073	.	NP_002064	.	protein_coding	ENSG0000012826	2781	4395
1471	LZTS1	1.198	0.001	leucine zipper tumor suppressor 1 [Source:HGNC Symbol;Acc:HGNC:13861]	chr8:20103675-20161474	NM_021020	XM_011544387, XM_0	NP_066300	XP_005273451, XP_0115	protein_coding	ENSG0000006133	11178	13861
1472	MCOLN3	1.541	0.001	mucolipin 3 [Source:HGNC Symbol;Acc:HGNC:13358]	chr1:85464829-85598821	NM_001253693, NN	XM_011541743, XM_0	NP_001240622, NP_06	XP_005271060, XP_0067	protein_coding	ENSG0000005573	55283	13358
1473	NEK1	1.406	0.001	NIMA related kinase 1 [Source:HGNC Symbol;Acc:HGNC:7744]	chr4:170314425-170533780	NM_001199397, NN	XM_011532004, XM_0	NP_001186326, NP_00	XP_006714291, XP_0115	protein_coding	ENSG0000013760	4750	7744
1474	NT5DC2	-0.829	0.001	5'-nucleotidase domain containing 2 [Source:HGNC Symbol;Acc:HGNC:25717]	chr3:52529353-52569070	NM_001134231, NN	XM_006713305, XM_0	NP_001127703, NP_07	XP_005265463, XP_0067	protein_coding	ENSG0000016826	64943	25717
1475	PDE4D	0.930	0.001	phosphodiesterase 4D [Source:HGNC Symbol;Acc:HGNC:8783]	chr5:58264864-59783903	NM_001104631, NN	XM_017009569, XM_0	NP_001098101, NP_00	XP_005248594, XP_0052	protein_coding	ENSG0000011344	5144	8783
1476	POLQ	1.211	0.001	DNA polymerase theta [Source:HGNC Symbol;Acc:HGNC:9186]	chr3:121150273-121265488	NM_199420	XM_011512350, XM_0	NP_955452	XP_011510645, XP_0115	protein_coding	ENSG0000005134	10721	9186
1477	PRMT3	0.945	0.001	protein arginine methyltransferase 3 [Source:HGNC Symbol;Acc:HGNC:30163]	chr11:20409075-20530799	NM_001145166, NN	XM_011519837, XM_0	NP_001138638, NP_00	XP_011518138, XP_0168	protein_coding	ENSG0000018523	10196	30163
1478	RARRES3	-2.019	0.001	retinoic acid receptor responder 3 [Source:HGNC Symbol;Acc:HGNC:9869]	chr11:63304287-63313934	NM_004585	.	NP_004576	.	protein_coding	ENSG0000013332	5920	9869
1479	RMI1	1.184	0.001	RecQ mediated genome instability 1 [Source:HGNC Symbol;Acc:HGNC:25764]	chr9:86595712-86618989	NM_024945	XM_017015140, XM_0	NP_079221	XP_005252268, XP_0052	protein_coding	ENSG0000017896	80010	25764
1480	TFF2	-2.691	0.001	trefoil factor 2 [Source:HGNC Symbol;Acc:HGNC:11756]	chr21:43766465-43771237	NM_005423	.	NP_005414	.	protein_coding	ENSG0000016018	7032	11756
1481	TIGAR	1.047	0.001	TP53 induced glycolysis regulatory phosphatase [Source:HGNC Symbol;Acc:HGNC:1185]	chr12:4416928-4463759	NM_020375	.	NP_065108	.	protein_coding	ENSG0000007823	57103	1185
1482	TMEM5	1.128	0.001	transmembrane protein 5 [Source:HGNC Symbol;Acc:HGNC:13530]	chr12:64173582-64203338	NM_001278237, NN	XM_005268563, XM_0	NP_001265166, NP_05	XP_005268619, XP_0052	protein_coding	ENSG0000011860	10329	13530
1483	TPP2	1.039	0.001	tripeptidyl peptidase 2 [Source:HGNC Symbol;Acc:HGNC:12016]	chr13:103249352-103332308	NM_003291	XM_005254073, XM_0	NP_003282	XP_005254127, XP_0052	protein_coding	ENSG0000013490	7174	12016
1484	TTC14	1.456	0.001	tetratricopeptide repeat domain 14 [Source:HGNC Symbol;Acc:HGNC:24697]	chr3:180319917-180397288	NM_001042601, NN	.	NP_001036066, NP_00	.	protein_coding	ENSG0000016372	151613	24697
1485	UAP1L1	-1.002	0.001	UDP-N-acetylglucosamine pyrophosphorylase 1 like 1 [Source:HGNC Symbol;Acc:HGNC:	chr9:139971952-139978991	NM_207309	XM_006717317	NP_997192	XP_006717380	protein_coding	ENSG0000019735	91373	28082
1486	USP34	1.287	0.001	ubiquitin specific peptidase 34 [Source:HGNC Symbol;Acc:HGNC:20066]	chr2:61404552-61697904	NM_014709	.	NP_055524	.	protein_coding	ENSG0000011546	9736	20066
1487	WAPL	1.096	0.001	WAPL cohesin release factor [Source:HGNC Symbol;Acc:HGNC:23293]	chr10:88195012-88281572	NM_001318328, NN	XM_011539548, XM_0	NP_001305257, NP_05	XP_011537850	protein_coding	ENSG0000006265	23063	23293
1488	ZNF292	1.270	0.001	zinc finger protein 292 [Source:HGNC Symbol;Acc:HGNC:18410]	chr6:87862550-87973914	NM_015021	XM_017010583, XM_0	NP_055836	XP_005248754, XP_0115	protein_coding	ENSG0000018899	23036	18410
1489	ZNF354A	1.363	0.001	zinc finger protein 354A [Source:HGNC Symbol;Acc:HGNC:11628]	chr5:178138592-178157703	NM_001324339, NN	XM_017009791	NP_001311268, NP_00	XP_011532947, XP_0168	protein_coding	ENSG0000016913	6940	11628
1490	ADCK3	-0.838	0.001	coenzyme Q8A [Source:HGNC Symbol;Acc:HGNC:16812]	chr1:227085236-227175246	NM_020247	XM_011544241, XM_0	NP_064632	XP_005273258, XP_0115	protein_coding	ENSG0000016305	56997	16812
1491	AQR	1.068	0.001	aquarius intron-binding spliceosomal factor [Source:HGNC Symbol;Acc:HGNC:29513]	chr15:35143982-35262040	NM_014691	.	NP_055506	.	protein_coding	ENSG0000002177	9716	29513
1492	CCDC14	1.232	0.001	coiled-coil domain containing 14 [Source:HGNC Symbol;Acc:HGNC:25766]	chr3:123632299-123680255	NM_022757	XM_017007056, XM_0	NP_001295246, NP_07	XP_005247767, XP_0052	protein_coding	ENSG0000017545	64770	25766
1493	CNOT1	0.882	0.001	CCR4-NOT transcription complex subunit 1 [Source:HGNC Symbol;Acc:HGNC:7877]	chr16:58549382-58663790	NM_001265612, NN	.	NP_001252541, NP_05	.	protein_coding	ENSG0000012510	23019	7877
1494	COL9A2	-1.751	0.001	collagen type IX alpha 2 chain [Source:HGNC Symbol;Acc:HGNC:2218]	chr1:40766158-40782966	NM_001852	XM_017000333, XM_0	NP_001843	XP_006710428, XP_0115	protein_coding	ENSG0000004908	1298	2218
1495	DDX49	-0.959	0.001	DEAD-box helicase 49 [Source:HGNC Symbol;Acc:HGNC:18684]	chr19:19030483-19039442	NM_019070	XM_011528084, XM_0	NP_061943	XP_011526385, XP_0115	protein_coding	ENSG0000010567	54555	18684
1496	HDAC5	-0.865	0.001	histone deacetylase 5 [Source:HGNC Symbol;Acc:HGNC:14068]	chr17:42154113-42201006	NM_001015053, NN	XM_017023995, XM_0	NP_001015053, NP_00	XP_005256961, XP_0052	protein_coding	ENSG0000010884	10014	14068
1497	INTS7	1.097	0.001	integrator complex subunit 7 [Source:HGNC Symbol;Acc:HGNC:24484]	chr1:212113742-212208884	NM_001199809, NN	XM_011509397, XM_0	NP_001186738, NP_00	XP_011507698	protein_coding	ENSG0000014349	25896	24484
1498	LY6E	-0.795	0.001	lymphocyte antigen 6 complex, locus E [Source:HGNC Symbol;Acc:HGNC:6727]	chr8:144099398-144105249	NM_001127213, NN	.	NP_001120685, NP_00	.	protein_coding	ENSG0000016093	4061	6727
1499	RASGEF1A	-1.337	0.001	RasGEF domain family member 1A [Source:HGNC Symbol;Acc:HGNC:24246]	chr10:43689982-43762367	NM_001282862, NN	XM_011539500, XM_0	NP_001269791, NP_66	XP_005271866, XP_0115	protein_coding	ENSG0000019891	221002	24246
1500	STK26	0.947	0.001	serine/threonine protein kinase 26 [Source:HGNC Symbol;Acc:HGNC:18174]	chrX:131157292-131209971	NM_001042452, NN	XM_011531350, XM_0	NP_001035917, NP_00	XP_011529651, XP_0115	protein_coding	ENSG0000013460	51765	18174
1501	TADA3	-1.216	0.001	transcriptional adaptor 3 [Source:HGNC Symbol;Acc:HGNC:19422]	chr3:9821543-9896822	NM_001278270, NN	XM_006712930	NP_001265199, NP_00	.	protein_coding	ENSG0000017114	10474	19422
1502	TMEM167B	1.009	0.001	transmembrane protein 167B [Source:HGNC Symbol;Acc:HGNC:30187]	chr1:109632424-109639556	NM_020141	.	NP_064526	.	protein_coding	ENSG0000021571	56900	30187
1503	TTC29	1.303	0.001	tetratricopeptide repeat domain 29 [Source:HGNC Symbol;Acc:HGNC:29936]	chr4:147627789-147867034	NM_001300761, NN	XM_006714339, XM_0	NP_001287690, NP_00	XP_005263327, XP_0067	protein_coding	ENSG0000013747	83894	29936
1504	ZCCHC8	1.140	0.001	zinc finger CCHC-type containing 8 [Source:HGNC Symbol;Acc:HGNC:25265]	chr12:122956146-122985620	NM_017612	XM_011538555	NP_060082	XP_011536857	protein_coding	ENSG0000003303	55596	25265
1505	ALDH1A3	-0.747	0.001	aldehyde dehydrogenase 1 family member A3 [Source:HGNC Symbol;Acc:HGNC:409]	chr15:101419580-101456831	NM_000693, NM_0	.	NP_000684, NP_00128	.	protein_coding	ENSG0000018425	220	409
1506	ANKRD13C	1.129	0.001	ankyrin repeat domain 13C [Source:HGNC Symbol;Acc:HGNC:25374]	chr1:70726270-70820417	NM_030816	XM_006710929, XM_0	NP_110443	XP_005271291, XP_0052	protein_coding	ENSG0000011845	81573	25374
1507	CD82	-0.816	0.001	CD82 molecule [Source:HGNC Symbol;Acc:HGNC:6210]	chr11:44587140-44641913	NM_001024844, NN	XM_006718223	NP_001020015, NP_00	XP_006718286	protein_coding	ENSG0000008511	3732	6210
1508	CDC14A	1.166	0.001	cell division cycle 14A [Source:HGNC Symbol;Acc:HGNC:1718]	chr1:100810580-100985833	NM_001319211, NN	XM_011542344, XM_0	NP_001306139, NP_00	XP_005271351, XP_0052	protein_coding	ENSG0000007933	8556	1718
1509	CLDN11	-0.972	0.001	claudin 11 [Source:HGNC Symbol;Acc:HGNC:8514]	chr3:170136652-170578169	NM_005602	.	NP_005593	.	protein_coding	ENSG0000001329	101928583	8514
1510	FAM43A	-1.041	0.001	family with sequence similarity 43 member A [Source:HGNC Symbol;Acc:HGNC:26888]	chr3:194407272-194409766	NM_153690	.	NP_710157	.	protein_coding	ENSG0000018511	131583	26888

1511	FSCN2	-1.547	0.001	fascin actin-bundling protein 2, retinal [Source:HGNC Symbol;Acc:HGNC:3960]	chr17:79495421-79504156	NM_001077182, NM	XM_011524593, XM_0	NP_001070650, NP_03	XP_005257252, XP_0115	protein_coding	ENSG0000018676	25794	3960
1512	GBAS	0.966	0.001	glioblastoma amplified sequence [Source:HGNC Symbol;Acc:HGNC:4179]	chr7:56032273-56067874	NM_001202469, NM	.	NP_001189398, NP_00	.	protein_coding	ENSG0000014672	2631	4179
1513	RSPRY1	1.144	0.001	ring finger and SPRY domain containing 1 [Source:HGNC Symbol;Acc:HGNC:29420]	chr16:57220202-57274387	NM_001305164, NM	XM_011523431, XM_0	NP_001292092, NP_00	XP_005256277, XP_0052	protein_coding	ENSG0000015957	89970	29420
1514	SIRT6	-1.035	0.001	sirtuin 6 [Source:HGNC Symbol;Acc:HGNC:14934]	chr19:4174105-4182601	NM_001193285, NM	XM_005259577, XM_0	NP_001180214, NP_00	.	protein_coding	ENSG0000007746	51548	14934
1515	TLE1	1.200	0.001	transducin like enhancer of split 1 [Source:HGNC Symbol;Acc:HGNC:11837]	chr9:84198597-84304220	NM_001303103, NM	XM_011518951, XM_0	NP_001290032, NP_00	XP_005252208, XP_0052	protein_coding	ENSG0000019678	7088	11837
1516	TNKS1BP1	-0.751	0.001	tankyrase 1 binding protein 1 [Source:HGNC Symbol;Acc:HGNC:19081]	chr11:57067115-57092401	NM_033396	XM_006718725	NP_203754	XP_006718788	protein_coding	ENSG0000014911	85456	19081
1517	ZGLP1	-1.649	0.001	zinc finger, GATA-like protein 1 [Source:HGNC Symbol;Acc:HGNC:37245]	chr19:10415478-10420556	NM_001103167	.	NP_001096637	.	protein_coding	ENSG0000022020	100125288	37245
1518	ACSF2	-0.986	0.001	acyl-CoA synthetase family member 2 [Source:HGNC Symbol;Acc:HGNC:26101]	chr17:48503518-48552198	NM_001288968, NM	XM_011525294	NP_001275897, NP_00	XP_011523596	protein_coding	ENSG0000016710	80221	26101
1519	ARHGAP4	-0.809	0.001	Rho GTPase activating protein 4 [Source:HGNC Symbol;Acc:HGNC:674]	chrX:153172820-153191714	NM_001164741, NM	.	NP_001158213, NP_00	.	protein_coding	ENSG0000008982	393	674
1520	ASPRV1	-1.931	0.001	aspartic peptidase, retroviral-like 1 [Source:HGNC Symbol;Acc:HGNC:26321]	chr2:70187220-70189397	NM_152792	.	NP_690005	.	protein_coding	ENSG0000024461	151516	26321
1521	C19orf60	-0.951	0.001	chromosome 19 open reading frame 60 [Source:HGNC Symbol;Acc:HGNC:26098]	chr19:18699534-18703146	NM_001100418, NM	.	NP_001093888, NP_00	.	protein_coding	ENSG0000000601	55049	26098
1522	CSDE1	0.800	0.001	cold shock domain containing E1 [Source:HGNC Symbol;Acc:HGNC:29905]	chr1:115259533-115300671	NM_001007553, NM	.	NP_001007554, NP_00	.	protein_coding	ENSG0000000930	7812	29905
1523	EMC3	-0.957	0.001	ER membrane protein complex subunit 3 [Source:HGNC Symbol;Acc:HGNC:23999]	chr3:10004220-10028804	NM_018447	XM_011533956	NP_060917	XP_011532258	protein_coding	ENSG0000012503	55831	23999
1524	IKBKE	-0.912	0.001	inhibitor of kappa light polypeptide gene enhancer in B-cells, kinase epsilon [Source:HG	chr1:206643790-206670223	NM_001193321, NM	XM_005273356	NP_001180250, NP_00	XP_005273413, XP_0168	protein_coding	ENSG0000014346	9641	14552
1525	MBOAT2	1.067	0.001	membrane bound O-acyltransferase domain containing 2 [Source:HGNC Symbol;Acc:HG	chr2:8992819-9143942	NM_001321265, NM	XM_011510309	NP_001308194, NP_00	.	protein_coding	ENSG0000014379	129642	25193
1526	MYCBP2	1.001	0.001	MYC binding protein 2, E3 ubiquitin protein ligase [Source:HGNC Symbol;Acc:HGNC:233	chr13:77618791-77901179	NM_015057	XM_011535004, XM_0	NP_055872	XP_005266356, XP_0067	protein_coding	ENSG0000000581	23077	23386
1527	NUSAP1	0.903	0.001	nucleolar and spindle associated protein 1 [Source:HGNC Symbol;Acc:HGNC:18538]	chr15:41624891-41673248	NM_001243142, NM	XM_006720563, XM_0	NP_001230071, NP_00	XP_005254485, XP_0052	protein_coding	ENSG0000013780	51203	18538
1528	PIGN	1.346	0.001	phosphatidylinositol glycan anchor biosynthesis class N [Source:HGNC Symbol;Acc:HGN	chr18:59710799-59854271	NM_012327, NM_1	XM_011525898, XM_0	NP_036459, NP_78974	XP_011524191, XP_0115	protein_coding	ENSG0000019756	23556	8967
1529	PSME4	1.152	0.001	proteasome activator subunit 4 [Source:HGNC Symbol;Acc:HGNC:20635]	chr2:54091203-54532437	NM_014614	XM_011532708, XM_0	NP_055429	XP_006712032, XP_0115	protein_coding	ENSG0000006887	23198	20635
1530	PUS7	0.902	0.001	pseudouridylate synthase 7 (putative) [Source:HGNC Symbol;Acc:HGNC:26033]	chr7:105096957-105162705	NM_001318163, NM	XM_017012367, XM_0	NP_001305092, NP_00	XP_005250519, XP_0052	protein_coding	ENSG0000009112	54517	26033
1531	RGS14	-0.936	0.001	regulator of G-protein signaling 14 [Source:HGNC Symbol;Acc:HGNC:9996]	chr5:176784837-176799602	NM_006480	XM_005265795, XM_0	NP_006471	XP_005265851, XP_0052	protein_coding	ENSG0000016922	10636	9996
1532	RTEL1	-0.943	0.001	regulator of telomere elongation helicase 1 [Source:HGNC Symbol;Acc:HGNC:15888]	chr20:62289162-62327606	NM_001283009, NM	.	NP_001269938, NP_00	.	protein_coding	ENSG0000025836	51750	15888
1533	SP4	1.343	0.001	Sp4 transcription factor [Source:HGNC Symbol;Acc:HGNC:11209]	chr7:21467651-21554440	NM_001326542, NM	XM_005249828	NP_001313471, NP_00	XP_005249885, XP_0115	protein_coding	ENSG0000010586	6671	11209
1534	TMX4	0.967	0.001	thioredoxin related transmembrane protein 4 [Source:HGNC Symbol;Acc:HGNC:25237]	chr20:7957994-8000476	NM_021156	.	NP_066979	.	protein_coding	ENSG0000012582	56255	25237
1535	TRUB1	1.203	0.001	TruB pseudouridine synthase family member 1 [Source:HGNC Symbol;Acc:HGNC:16060]	chr10:116697951-116737435	NM_139169	XM_011539321	NP_631908	.	protein_coding	ENSG0000016583	142940	16060
1536	XPR1	1.118	0.001	xenotropic and polytropic retrovirus receptor 1 [Source:HGNC Symbol;Acc:HGNC:12827	chr1:180601139-180859387	NM_001135669, NM	.	NP_001129141, NP_00	.	protein_coding	ENSG0000014332	9213	12827
1537	ZNF92	1.257	0.001	zinc finger protein 92 [Source:HGNC Symbol;Acc:HGNC:13168]	chr7:64838711-64866048	NM_001287532, NM	.	NP_001274461, NP_00	.	protein_coding	ENSG0000014675	168374	13168
1538	ARRDC4	1.328	0.001	arrestin domain containing 4 [Source:HGNC Symbol;Acc:HGNC:28087]	chr15:98503927-98517068	NM_183376	.	NP_899232	.	protein_coding	ENSG0000014045	91947	28087
1539	ATAD2B	1.260	0.001	ATPase family, AAA domain containing 2B [Source:HGNC Symbol;Acc:HGNC:29230]	chr2:23971533-24149984	NM_001242338, NM	XM_011532923, XM_0	NP_001229267, NP_06	XP_005264429, XP_0067	protein_coding	ENSG0000011977	54454	29230
1540	CCAR1	0.963	0.001	cell division cycle and apoptosis regulator 1 [Source:HGNC Symbol;Acc:HGNC:24236]	chr10:70480900-70552134	NM_001282959, NM	.	NP_001269888, NP_00	.	protein_coding	ENSG0000006033	55749	24236
1541	CEP162	1.403	0.001	centrosomal protein 162 [Source:HGNC Symbol;Acc:HGNC:21107]	chr6:84833959-84937353	NM_001286206, NM	XM_017010484, XM_0	NP_001273135, NP_05	XP_005248731, XP_0067	protein_coding	ENSG0000013531	22832	21107
1542	DTX4	-1.190	0.001	deltex E3 ubiquitin ligase 4 [Source:HGNC Symbol;Acc:HGNC:29151]	chr11:58938902-58980424	NM_001300727, NM	XM_006718483	NP_001287656, NP_05	XP_006718546	protein_coding	ENSG0000011004	23220	29151
1543	EXOSC7	-0.837	0.001	exosome component 7 [Source:HGNC Symbol;Acc:HGNC:28112]	chr3:45017721-45052962	NM_015004	.	NP_055819	.	protein_coding	ENSG0000007591	23016	28112
1544	FBXO38	0.988	0.001	F-box protein 38 [Source:HGNC Symbol;Acc:HGNC:28844]	chr5:147763497-147822399	NM_001271723, NM	XM_006714797, XM_0	NP_001258652, NP_11	XP_005268570, XP_0067	protein_coding	ENSG0000014586	81545	28844
1545	GLS	0.974	0.001	glutaminase [Source:HGNC Symbol;Acc:HGNC:4331]	chr2:191745552-191830278	NM_001256310, NM	XM_006712435, XM_0	NP_001243239, NP_05	XP_005246524, XP_0067	protein_coding	ENSG0000011541	2744	4331
1546	GRHPR	-0.809	0.001	glyoxylate and hydroxypyruvate reductase [Source:HGNC Symbol;Acc:HGNC:4570]	chr9:37422662-37436987	NM_012203	XM_011518073	NP_036335	XP_011516375	protein_coding	ENSG0000013710	9380	4570
1547	GTF2H1	1.033	0.001	general transcription factor IIH subunit 1 [Source:HGNC Symbol;Acc:HGNC:4655]	chr11:18343841-18388591	NM_001142307, NM	XM_006718208	NP_001135779, NP_00	XP_006718271	protein_coding	ENSG0000011076	2965	4655
1548	HLA-DPA1	-2.328	0.001	major histocompatibility complex, class II, DP alpha 1 [Source:HGNC Symbol;Acc:HGNC:	chr6:33032345-33054978	NM_001242524, NM	.	NP_001229453, NP_00	.	protein_coding	ENSG0000023138	3113	4938
1549	HOXA7	-1.094	0.001	homeobox A7 [Source:HGNC Symbol;Acc:HGNC:5108]	chr7:27193334-27196296	NM_006896	.	NP_008827	.	protein_coding	ENSG0000012259	3204	5108
1550	MDH1	0.847	0.001	malate dehydrogenase 1 [Source:HGNC Symbol;Acc:HGNC:6970]	chr2:63348517-63834331	NM_001199111, NM	.	NP_001186040, NP_00	.	protein_coding	ENSG0000001464	4190	6970
1551	MTPN	1.055	0.001	myotrophin [Source:HGNC Symbol;Acc:HGNC:15667]	chr7:135611508-135662101	NM_145808	.	NP_665807	.	protein_coding	ENSG0000010588	136319	15667
1552	MTX3	1.687	0.001	metaxin 3 [Source:HGNC Symbol;Acc:HGNC:24812]	chr5:79272553-79287085	NM_001010891, NM	XM_005248495	NP_001010891, NP_00	XP_005248552	protein_coding	ENSG0000017703	345778	24812
1553	MXD4	-1.036	0.001	MAX dimerization protein 4 [Source:HGNC Symbol;Acc:HGNC:13906]	chr4:2249158-2264021	NM_006454	.	NP_006445	XP_016863145	protein_coding	ENSG0000012393	100616358	13906
1554	PHF6	0.989	0.001	PHD finger protein 6 [Source:HGNC Symbol;Acc:HGNC:18145]	chrX:133507282-133562821	NM_001015877, NM	.	NP_001015877, NP_11	.	protein_coding	ENSG0000015653	84295	18145
1555	RBM27	1.162	0.001	RNA binding motif protein 27 [Source:HGNC Symbol;Acc:HGNC:29243]	chr5:145583112-145720083	NM_018989	XM_011537662, XM_0	NP_061862	XP_005268523, XP_0052	protein_coding	ENSG0000009100	54439	29243
1556	SEPSECS	1.364	0.001	Sep (O-phosphoserine) tRNA:Sec (selenocysteine) tRNA synthase [Source:HGNC Symbol	chr4:25121635-25162064	NM_016955	XM_017008278, XM_0	NP_058651	XP_011512148, XP_0115	protein_coding	ENSG0000010961	51091	30605
1557	ZNF22	1.084	0.001	zinc finger protein 22 [Source:HGNC Symbol;Acc:HGNC:13012]	chr10:45495922-45500774	NM_006963	.	NP_008894	.	protein_coding	ENSG0000016551	7570	13012
1558	CYP51A1	0.826	0.001	cytochrome P450 family 51 subfamily A member 1 [Source:HGNC Symbol;Acc:HGNC:26	chr7:91741464-91764117	NM_000786, NM_0	.	NP_000777, NP_00113	.	protein_coding	ENSG0000000163	1595	2649
1559	DARS	0.857	0.001	aspartyl-tRNA synthetase [Source:HGNC Symbol;Acc:HGNC:2678]	chr2:136664246-136743254	NM_001293312, NM	.	NP_001280241, NP_00	XP_016858978	protein_coding	ENSG0000011586	1615	2678
1560	GM2A	-0.897	0.001	GM2 ganglioside activator [Source:HGNC Symbol;Acc:HGNC:4367]	chr5:150632452-150650001	NM_000405, NM_0	.	NP_000396, NP_00116	.	protein_coding	ENSG0000019674	2760	4367
1561	IZUMO4	-3.107	0.001	IZUMO family member 4 [Source:HGNC Symbol;Acc:HGNC:26950]	chr19:2096867-2099589	NM_001031735, NM	XM_005259480	NP_001026905, NP_00	XP_005259537	protein_coding	ENSG0000009984	113177	26950
1562	LY6G6C	-1.488	0.001	lymphocyte antigen 6 complex, locus G6C [Source:HGNC Symbol;Acc:HGNC:13936]	chr6:31686424-31689622	NM_025261	.	NP_079537	.	protein_coding	ENSG0000020442	80740	13936
1563	NHLRC2	1.155	0.001	NHL repeat containing 2 [Source:HGNC Symbol;Acc:HGNC:24731]	chr10:115614419-115676953	NM_198514	XM_011539769	NP_940916	XP_011538071	protein_coding	ENSG0000019686	374354	24731
1564	OXCT1	0.974	0.001	3-oxoacid CoA-transferase 1 [Source:HGNC Symbol;Acc:HGNC:8527]	chr5:41730166-41870621	NM_000436	.	NP_000427	.	protein_coding	ENSG0000008372	5019	8527

1565	PHF20	0.925	0.001	PHD finger protein 20 [Source:HGNC Symbol;Acc:HGNC:16098]	chr20:34359904-34538303	NM_016436	XM_017027871, XM_0	NP_057520	XP_005260478, XP_0115	protein_coding	ENSG0000002529	51230			16098
1566	PLRG1	0.933	0.001	pleiotropic regulator 1 [Source:HGNC Symbol;Acc:HGNC:9089]	chr4:155456157-155471587	NM_001201564, NM	.	NP_001188493, NP_00	.	protein_coding	ENSG0000017156	5356			9089
1567	PRTFDC1	1.355	0.001	phosphoribosyl transferase domain containing 1 [Source:HGNC Symbol;Acc:HGNC:2333	chr10:25137535-25241533	NM_001282786, NM	XM_011519590, XM_0	NP_001269715, NP_06	XP_011517891	protein_coding	ENSG0000009925	56952			23333
1568	RBBP8	1.100	0.001	RB binding protein 8, endonuclease [Source:HGNC Symbol;Acc:HGNC:9891]	chr18:20513277-20606451	NM_002894, NM_2	XM_011526132, XM_0	NP_002885, NP_97603	XP_005258382, XP_0067	protein_coding	ENSG0000010177	100616139			9891
1569	RNF219	1.208	0.001	ring finger protein 219 [Source:HGNC Symbol;Acc:HGNC:20308]	chr13:79188425-79233314	NM_024546	XM_011535225	NP_078822	XP_011533527	protein_coding	ENSG0000015219	79596			20308
1570	SLC39A6	1.179	0.001	solute carrier family 39 member 6 [Source:HGNC Symbol;Acc:HGNC:18607]	chr18:33688494-33709348	NM_001099406, NM	XM_011525901, XM_0	NP_001092876, NP_03	XP_011524202, XP_0115	protein_coding	ENSG0000014142	25800			18607
1571	SREK1	1.188	0.001	splicing regulatory glutamic acid and lysine rich protein 1 [Source:HGNC Symbol;Acc:HG	chr5:65440045-65479443	NM_001077199, NM	XM_011543171, XM_0	NP_001070667, NP_00	XP_005248496, XP_0115	protein_coding	ENSG0000015391	140890			17882
1572	STAP2	-0.963	0.001	signal transducing adaptor family member 2 [Source:HGNC Symbol;Acc:HGNC:30430]	chr19:4324039-4338874	NM_001013841, NM	XM_011528123	NP_001013863, NP_06	XP_011526425	protein_coding	ENSG0000017807	55620			30430
1573	TLK1	1.155	0.001	tousled like kinase 1 [Source:HGNC Symbol;Acc:HGNC:11841]	chr2:171847332-172087824	NM_001136554, NM	XM_011512238, XM_0	NP_001130026, NP_00	XP_011510540	protein_coding	ENSG0000019858	9874			11841
1574	TUBB4A	-1.061	0.001	tubulin beta 4A class IVa [Source:HGNC Symbol;Acc:HGNC:20774]	chr19:6494329-6502595	NM_001289123, NM	.	NP_001276052, NP_00	.	protein_coding	ENSG0000010483	10382			20774
1575	UBQLN1	0.815	0.001	ubiquilin 1 [Source:HGNC Symbol;Acc:HGNC:12508]	chr9:86274877-86323118	NM_013438, NM_0	.	NP_038466, NP_44429	.	protein_coding	ENSG0000013501	29979			12508
1576	WDR44	1.083	0.001	WD repeat domain 44 [Source:HGNC Symbol;Acc:HGNC:30512]	chrX:117480035-117583924	NM_001184965, NM	XM_011531353	NP_001171894, NP_00	XP_011529655	protein_coding	ENSG0000013172	54521			30512
1577	ZBTB2	1.039	0.001	zinc finger and BTB domain containing 2 [Source:HGNC Symbol;Acc:HGNC:20868]	chr6:151685251-151712683	NM_020861	XM_011536004, XM_0	NP_065912	XP_005267133, XP_0115	protein_coding	ENSG0000018147	57621			20868
1578	ZBTB33	1.023	0.001	zinc finger and BTB domain containing 33 [Source:HGNC Symbol;Acc:HGNC:16682]	chrX:119384606-119392253	NM_001184742, NM	.	NP_001171671, NP_00	.	protein_coding	ENSG0000017748	10009			16682
1579	AAGAB	0.870	0.001	alpha- and gamma-adaptin binding protein [Source:HGNC Symbol;Acc:HGNC:25662]	chr15:67493370-67794142	NM_001271885, NM	XM_011522020	NP_001258814, NP_00	XP_011520322	protein_coding	ENSG0000010359	79719			25662
1580	ABCE1	0.959	0.001	ATP binding cassette subfamily E member 1 [Source:HGNC Symbol;Acc:HGNC:69]	chr4:145915726-146050331	NM_001040876, NM	.	NP_001035809, NP_00	.	protein_coding	ENSG0000016416	6059			69
1581	CNOT2	1.014	0.001	CCR4-NOT transcription complex subunit 2 [Source:HGNC Symbol;Acc:HGNC:7878]	chr12:70636773-70748773	NM_001199302, NM	XM_011538400, XM_0	NP_001186231, NP_00	XP_006719492, XP_0067	protein_coding	ENSG0000011159	4848			7878
1582	CPEB4	1.393	0.001	cytoplasmic polyadenylation element binding protein 4 [Source:HGNC Symbol;Acc:HGN	chr5:173315282-173388979	NM_001308189, NM	XM_005265996, XM_0	NP_001295118, NP_00	XP_005266051, XP_0052	protein_coding	ENSG0000011374	80315			21747
1583	CSNK1G3	1.182	0.001	casein kinase 1 gamma 3 [Source:HGNC Symbol;Acc:HGNC:2456]	chr5:122847792-122952739	NM_001031812, NM	XM_005271896, XM_0	NP_001026982, NP_00	XP_005271948, XP_0052	protein_coding	ENSG0000015129	1456			2456
1584	EEA1	1.327	0.001	early endosome antigen 1 [Source:HGNC Symbol;Acc:HGNC:3185]	chr12:93115280-93323107	NM_003566	XM_011538815, XM_0	NP_003557	XP_011537116, XP_0115	protein_coding	ENSG0000010218	8411			3185
1585	FEM1C	1.161	0.001	fem-1 homolog C [Source:HGNC Symbol;Acc:HGNC:16933]	chr5:114856604-114880591	NM_020177	XM_005272035	NP_064562	XP_005272092	protein_coding	ENSG0000014578	56929			16933
1586	FLOT1	-0.855	0.001	flotillin 1 [Source:HGNC Symbol;Acc:HGNC:3757]	chr6:30695485-30710510	NM_005803	XM_006714947, XM_0	NP_005794	XP_005248837, XP_0067	protein_coding	ENSG0000013731	10211			3757
1587	HSD17B1	-1.511	0.001	hydroxysteroid 17-beta dehydrogenase 1 [Source:HGNC Symbol;Acc:HGNC:5210]	chr17:40701231-40707231	NM_000413	XM_011524731, XM_0	NP_000404	XP_005257349, XP_0067	protein_coding	ENSG0000010878	3292			5210
1588	IER5L	-1.005	0.001	immediate early response 5 like [Source:HGNC Symbol;Acc:HGNC:23679]	chr9:131937830-131940541	NM_203434	.	NP_982258	.	protein_coding	ENSG0000018848	389792			23679
1589	IL10RA	-1.466	0.001	interleukin 10 receptor subunit alpha [Source:HGNC Symbol;Acc:HGNC:5964]	chr11:117857062-117872194	NM_001558	.	NP_001549	.	protein_coding	ENSG0000011032	3587			5964
1590	ITGA2B	-1.503	0.001	integrin subunit alpha 2b [Source:HGNC Symbol;Acc:HGNC:6138]	chr17:42449550-42466873	NM_000419	XM_011524750, XM_0	NP_000410	XP_011523051, XP_0115	protein_coding	ENSG0000000596	3674			6138
1591	LDHD	-1.802	0.001	lactate dehydrogenase D [Source:HGNC Symbol;Acc:HGNC:19708]	chr16:75145757-75150669	NM_153486, NM_1	.	NP_705690, NP_91941	.	protein_coding	ENSG0000016681	197257			19708
1592	PAPOLG	1.150	0.001	poly(A) polymerase gamma [Source:HGNC Symbol;Acc:HGNC:14982]	chr2:60983364-61026098	NM_022894	XM_011533041, XM_0	NP_075045	XP_005264557, XP_0052	protein_coding	ENSG0000011542	64895			14982
1593	PRRC2C	0.835	0.001	proline rich coiled-coil 2C [Source:HGNC Symbol;Acc:HGNC:24903]	chr1:171454689-171562650	NM_015172	XM_011509343, XM_0	NP_055987	XP_005245072, XP_0052	protein_coding	ENSG0000011752	23215			24903
1594	RWDD2B	0.965	0.001	RWD domain containing 2B [Source:HGNC Symbol;Acc:HGNC:1302]	chr21:30376704-30391699	NM_016940	.	NP_001307653, NP_05	.	protein_coding	ENSG0000015625	10069			1302
1595	SCN5A	-1.141	0.001	sodium voltage-gated channel alpha subunit 5 [Source:HGNC Symbol;Acc:HGNC:10593]	chr3:38589547-38691164	NM_000335, NM_0	XM_017007017, XM_0	NP_000326, NP_00109	XP_006713345, XP_0115	protein_coding	ENSG0000018387	6331			10593
1596	TMC4	-1.010	0.001	transmembrane channel like 4 [Source:HGNC Symbol;Acc:HGNC:22998]	chr19:54663845-54676944	NM_001145303, NM	.	NP_001138775, NP_65	.	protein_coding	ENSG0000016760	147798			22998
1597	TMEM87B	0.978	0.001	transmembrane protein 87B [Source:HGNC Symbol;Acc:HGNC:25913]	chr2:112812799-112876895	NM_032824	XM_006712807, XM_0	NP_116213	XP_005263884, XP_0067	protein_coding	ENSG0000015321	84910			25913
1598	VGLL4	-1.299	0.001	vestigial like family member 4 [Source:HGNC Symbol;Acc:HGNC:28966]	chr3:11314101-11762220	NM_001128219, NM	XM_011534269, XM_0	NP_001121691, NP_00	XP_011532569, XP_0115	protein_coding	ENSG0000014456	9686			28966
1599	WDR43	0.850	0.001	WD repeat domain 43 [Source:HGNC Symbol;Acc:HGNC:28945]	chr2:29117508-29171088	NM_015131	.	NP_055946	.	protein_coding	ENSG0000016381	23160			28945
1600	ZNF281	1.096	0.001	zinc finger protein 281 [Source:HGNC Symbol;Acc:HGNC:13075]	chr1:200374067-200379184	NM_001281293, NM	XM_017000888	NP_001268222, NP_00	XP_016856376, XP_0168	protein_coding	ENSG0000016270	23528			13075
1601	ZNF507	1.265	0.001	zinc finger protein 507 [Source:HGNC Symbol;Acc:HGNC:23783]	chr19:32836499-32878573	NM_001136156, NM	.	NP_001129628, NP_05	.	protein_coding	ENSG0000016881	22847			23783
1602	AASDH	1.345	0.001	aminoadipate-semialdehyde dehydrogenase [Source:HGNC Symbol;Acc:HGNC:23993]	chr4:57204452-57253674	NM_001286668, NM	XM_011534373, XM_0	NP_001273597, NP_00	XP_005265778, XP_0115	protein_coding	ENSG0000015742	132949			23993
1603	CHID1	-0.823	0.001	chitinase domain containing 1 [Source:HGNC Symbol;Acc:HGNC:28474]	chr11:867859-915058	NM_001142674, NM	XM_011520300, XM_0	NP_001136146, NP_00	XP_005253129, XP_0052	protein_coding	ENSG0000017783	66005			28474
1604	DBT	1.771	0.001	dihydrolipoamide branched chain transacylase E2 [Source:HGNC Symbol;Acc:HGNC:269	chr1:100652474-100715390	NM_001918	XM_005270545	NP_001909	XP_005270602	protein_coding	ENSG0000013799	1629			2698
1605	EFR3A	1.027	0.001	EFR3 homolog A [Source:HGNC Symbol;Acc:HGNC:28970]	chr8:132916334-133025889	NM_001323553, NM	XM_011516945, XM_0	NP_001310482, NP_00	XP_005250906	protein_coding	ENSG0000013229	23167			28970
1606	FBXW2	0.847	0.001	F-box and WD repeat domain containing 2 [Source:HGNC Symbol;Acc:HGNC:13608]	chr9:123519251-123555690	NM_012164	XM_011518518, XM_0	NP_036296	XP_005251967, XP_0052	protein_coding	ENSG0000011940	26190			13608
1607	FGF19	-0.775	0.001	fibroblast growth factor 19 [Source:HGNC Symbol;Acc:HGNC:3675]	chr11:69512999-69519410	NM_005117	.	NP_005108	.	protein_coding	ENSG0000016234	9965			3675
1608	FRYL	1.226	0.001	FRY like transcription coactivator [Source:HGNC Symbol;Acc:HGNC:29127]	chr4:48499377-48782316	NM_015030	XM_017008046, XM_0	NP_055845	XP_005248147, XP_0115	protein_coding	ENSG0000007553	285527			29127
1609	HPCAL4	-1.137	0.001	hippocalcin like 4 [Source:HGNC Symbol;Acc:HGNC:18212]	chr1:40144319-40157361	NM_001282396, NM	XM_017001447	NP_001269325, NP_00	XP_016856936	protein_coding	ENSG0000011698	51440			18212
1610	MAP3K5	1.191	0.001	mitogen-activated protein kinase kinase kinase 5 [Source:HGNC Symbol;Acc:HGNC:6857	chr6:136878184-137113656	NM_005923	XM_017010873, XM_0	NP_005914	XP_016866359, XP_0168	protein_coding	ENSG0000019744	4217			6857
1611	METTL4	1.131	0.001	methyltransferase like 4 [Source:HGNC Symbol;Acc:HGNC:24726]	chr18:2537523-2571508	NM_001308401, NM	XM_006722339	NP_001295330, NP_07	XP_006722402	protein_coding	ENSG0000010157	64863			24726
1612	MFGE8	-1.050	0.001	milk fat globule-EGF factor 8 protein [Source:HGNC Symbol;Acc:HGNC:7036]	chr15:89441943-89456642	NM_001114614, NM	.	NP_001108086, NP_00	.	protein_coding	ENSG0000014054	4240			7036
1613	RABAC1	-0.795	0.001	Rab acceptor 1 [Source:HGNC Symbol;Acc:HGNC:9794]	chr19:42460832-42463542	NM_006423	.	NP_006414	.	protein_coding	ENSG0000010540	10567			9794
1614	SOCS5	1.085	0.001	suppressor of cytokine signaling 5 [Source:HGNC Symbol;Acc:HGNC:16852]	chr2:46926090-46990268	NM_014011, NM_1	.	NP_054730, NP_65919	.	protein_coding	ENSG0000017115	9655			16852
1615	USP16	0.984	0.001	ubiquitin specific peptidase 16 [Source:HGNC Symbol;Acc:HGNC:12614]	chr21:30396949-30426809	NM_001001992, NM	XM_017028261, XM_0	NP_001001992, NP_00	XP_016883746, XP_0168	protein_coding	ENSG0000015625	10600			12614
1616	WSCD1	-1.844	0.001	WSC domain containing 1 [Source:HGNC Symbol;Acc:HGNC:29060]	chr17:5972425-6027747	NM_015253	XM_011523776, XM_0	NP_056068	XP_005256629, XP_0052	protein_coding	ENSG0000017931	339166			29060
1617	XRCC5	0.757	0.001	X-ray repair cross complementing 5 [Source:HGNC Symbol;Acc:HGNC:12833]	chr2:216972186-217071026	NM_021141	.	NP_066964	.	protein_coding	ENSG0000007924	7520			12833
1618	ZW10	0.987	0.001	zw10 kinetochore protein [Source:HGNC Symbol;Acc:HGNC:13194]	chr11:113603908-113644533	NM_004724	XM_017018559	NP_004715	XP_016874048	protein_coding	ENSG0000008682	9183			13194

1619	ACY1	-1.115	0.001	aminoacylase 1 [Source:HGNC Symbol;Acc:HGNC:177]	chr3:52002525-52023218	NM_000666, NM_0	.	NP_000657, NP_00118	.	protein_coding	ENSG0000024398	95	177
1620	BEGAIN	-1.150	0.001	brain enriched guanylate kinase associated [Source:HGNC Symbol;Acc:HGNC:24163]	chr14:101003483-101044289	NM_001159531, NN	XM_011537033, XM_0	NP_001153003, NP_06	XP_005267977, XP_0052	protein_coding	ENSG0000018309	57596	24163
1621	C10orf88	1.314	0.001	chromosome 10 open reading frame 88 [Source:HGNC Symbol;Acc:HGNC:25822]	chr10:124691023-124713919	NM_024942	.	NP_079218	.	protein_coding	ENSG0000011996	80007	25822
1622	CDC40	1.026	0.001	cell division cycle 40 [Source:HGNC Symbol;Acc:HGNC:17350]	chr6:110501343-110679475	NM_015891	XM_011535880	NP_056975	.	protein_coding	ENSG0000016843	51362	17350
1623	DNAJC13	1.042	0.001	DnaJ heat shock protein family (Hsp40) member C13 [Source:HGNC Symbol;Acc:HGNC:3	chr3:132136369-132257876	NM_001329126, NN	XM_005247245	NP_001316055, NP_05	.	protein_coding	ENSG0000013824	23317	30343
1624	DPM3	-1.019	0.001	dolichyl-phosphate mannosyltransferase subunit 3 [Source:HGNC Symbol;Acc:HGNC:30	chr1:155112366-155113071	NM_018973, NM_1	XM_017001498	NP_061846, NP_71496	XP_016856987	protein_coding	ENSG0000017908	54344	3007
1625	DYRK1A	1.043	0.001	dual specificity tyrosine phosphorylation regulated kinase 1A [Source:HGNC Symbol;Acc	chr21:38739878-38889753	NM_001396, NM_1	XM_011529485, XM_0	NP_001387, NP_56782	XP_006724039, XP_0067	protein_coding	ENSG0000015754	1859	3091
1626	ELK4	1.117	0.001	ELK4, ETS transcription factor [Source:HGNC Symbol;Acc:HGNC:3326]	chr1:205577070-205601090	NM_001973, NM_0	XM_005244951, XM_0	NP_001964, NP_06856	XP_005245007, XP_0052	protein_coding	ENSG0000015871	2005	3326
1627	FAM72A	1.349	0.001	family with sequence similarity 72 member A [Source:HGNC Symbol;Acc:HGNC:24044]	chr1:206137236-206155151	NM_001123168	XM_011509967, XM_0	NP_001116640, NP_00	XP_006711589, XP_0115	protein_coding	ENSG0000019655	729533	24044
1628	FNDC11	-1.189	0.001	fibronectin type III domain containing 11 [Source:HGNC Symbol;Acc:HGNC:28764]	chr20:62184372-62188061	NM_001319152, NN	XM_011529041, XM_0	NP_001306081, NP_00	XP_005260288, XP_0052	protein_coding	ENSG0000012553	79025	28764
1629	ILVBL	-1.012	0.001	ilvB acetolactate synthase like [Source:HGNC Symbol;Acc:HGNC:6041]	chr19:15218213-15236596	NM_006844	XM_011527651, XM_0	NP_006835	XP_005259774, XP_0115	protein_coding	ENSG0000010513	10994	6041
1630	MEIS3	-0.936	0.001	Meis homeobox 3 [Source:HGNC Symbol;Acc:HGNC:29537]	chr19:47906380-47922780	NM_001009813, NN	XM_011527140, XM_0	NP_001009813, NP_00	XP_011525438, XP_0115	protein_coding	ENSG0000010541	56917	29537
1631	NDUFS7	-0.880	0.001	NADH:ubiquinone oxidoreductase core subunit S7 [Source:HGNC Symbol;Acc:HGNC:771	chr19:1383525-1395588	NM_024407	XM_005259556	NP_077718	XP_005259613	protein_coding	ENSG0000011528	374291	7714
1632	RAP1GAP	-1.043	0.001	RAP1 GTPase activating protein [Source:HGNC Symbol;Acc:HGNC:9858]	chr1:21922707-21995856	NM_001145657, NN	XM_017001988, XM_0	NP_001139129, NP_00	XP_005246012, XP_0067	protein_coding	ENSG0000007686	5909	9858
1633	RXRB	-0.883	0.001	retinoid X receptor beta [Source:HGNC Symbol;Acc:HGNC:10478]	chr6:33161364-33168465	NM_001270401, NN	XM_011514796, XM_0	NP_001257330, NP_06	XP_005249335, XP_0115	protein_coding	ENSG0000020423	6257	10478
1634	SRPK1	0.854	0.001	SRSF protein kinase 1 [Source:HGNC Symbol;Acc:HGNC:11305]	chr6:35800742-35889119	NM_003137	.	NP_003128	.	protein_coding	ENSG0000009606	6732	11305
1635	TST	-1.110	0.001	thiosulfate sulfurtransferase [Source:HGNC Symbol;Acc:HGNC:12388]	chr22:37406899-37425863	NM_001270483, NN	.	NP_001257412, NP_00	.	protein_coding	ENSG0000012831	7263	12388
1636	YWHAG	0.767	0.001	tyrosine 3-monooxygenase/tryptophan 5-monooxygenase activation protein gamma [Sc	chr7:75956110-75988348	NM_012479	.	NP_036611	.	protein_coding	ENSG0000017002	7532	12852
1637	ASCL5	-1.574	0.001	achaete-scute family bHLH transcription factor 5 [Source:HGNC Symbol;Acc:HGNC:3316	chr1:201083080-201096312	NM_001270601	.	NP_001257530	.	protein_coding	ENSG0000023223	647219	33169
1638	C5orf51	1.131	0.001	chromosome 5 open reading frame 51 [Source:HGNC Symbol;Acc:HGNC:27750]	chr5:41904450-41921738	NM_175921	XM_011514032	NP_787117	XP_011512334	protein_coding	ENSG0000020576	285636	27750
1639	CCDC124	-0.811	0.001	coiled-coil domain containing 124 [Source:HGNC Symbol;Acc:HGNC:25171]	chr19:18043824-18054800	NM_001136203, NN	.	NP_001129675, NP_61	.	protein_coding	ENSG0000000708	115098	25171
1640	CKAP2	0.891	0.001	cytoskeleton associated protein 2 [Source:HGNC Symbol;Acc:HGNC:1990]	chr13:53029573-53050763	NM_001098525, NN	XM_011535043, XM_0	NP_001091995, NP_00	XP_005266401, XP_0115	protein_coding	ENSG0000013610	26586	1990
1641	DUSP5	-0.768	0.001	dual specificity phosphatase 5 [Source:HGNC Symbol;Acc:HGNC:3071]	chr10:112257595-112271302	NM_004419	.	NP_004410	.	protein_coding	ENSG0000013816	1847	3071
1642	GAP43	1.285	0.001	growth associated protein 43 [Source:HGNC Symbol;Acc:HGNC:4140]	chr3:115342170-115440337	NM_001130064, NN	.	NP_001123536, NP_00	XP_016861617	protein_coding	ENSG0000017202	2596	4140
1643	GRHL1	1.057	0.001	grainyhead like transcription factor 1 [Source:HGNC Symbol;Acc:HGNC:17923]	chr2:10091826-10142405	NM_198182	XM_011510343, XM_0	NP_937825	XP_005246216, XP_0067	protein_coding	ENSG0000013431	29841	17923
1644	H2AFJ	-0.794	0.001	H2A histone family member J [Source:HGNC Symbol;Acc:HGNC:14456]	chr12:14927316-14927960	NM_177925	.	NP_808760	.	protein_coding	ENSG0000024670	55766	14456
1645	IBTK	1.067	0.001	inhibitor of Bruton tyrosine kinase [Source:HGNC Symbol;Acc:HGNC:17853]	chr6:82879699-82957471	NM_001300906, NN	XM_006715454, XM_0	NP_001287835, NP_05	XP_006715516, XP_0067	protein_coding	ENSG0000000570	25998	17853
1646	IFITM3	-0.763	0.001	interferon induced transmembrane protein 3 [Source:HGNC Symbol;Acc:HGNC:5414]	chr11:319668-327537	NM_021034	.	NP_066362	.	protein_coding	ENSG0000014208	10410	5414
1647	IP6K2	-0.936	0.001	inositol hexakisphosphate kinase 2 [Source:HGNC Symbol;Acc:HGNC:17313]	chr3:48725435-48754711	NM_001005909, NN	XM_017006584, XM_0	NP_001005909, NP_00	XP_006713262, XP_0115	protein_coding	ENSG0000006874	51447	17313
1648	LPP	1.030	0.001	LIM domain containing preferred translocation partner in lipoma [Source:HGNC Symbol	chr3:187871071-188608460	NM_001167671, NN	XM_017006379, XM_0	NP_001161143, NP_00	XP_005247503, XP_0052	protein_coding	ENSG0000014501	4026	6679
1649	MSX2	-1.055	0.001	msh homeobox 2 [Source:HGNC Symbol;Acc:HGNC:7392]	chr5:174151535-174157896	NM_002449	.	NP_002440	XP_016864978	protein_coding	ENSG0000012014	4488	7392
1650	PDHA1	-1.104	0.001	pyruvate dehydrogenase (lipoamide) alpha 1 [Source:HGNC Symbol;Acc:HGNC:8806]	chrX:19362010-19533379	NM_000284, NM_0	XM_011545532, XM_0	NP_000275, NP_00116	.	protein_coding	ENSG0000013182	5160	8806
1651	RBM38	-0.775	0.001	RNA binding motif protein 38 [Source:HGNC Symbol;Acc:HGNC:15818]	chr20:55966462-55984389	NM_017495, NM_1	.	NP_059965, NP_90627	.	protein_coding	ENSG0000013281	55544	15818
1652	SARAF	0.827	0.001	store-operated calcium entry associated regulatory factor [Source:HGNC Symbol;Acc:HG	chr8:29920527-29940724	NM_001284239, NN	.	NP_001271168, NP_05	XP_016869056	protein_coding	ENSG0000013387	51669	28789
1653	SORBS2	1.249	0.001	sorbin and SH3 domain containing 2 [Source:HGNC Symbol;Acc:HGNC:24098]	chr4:186506597-186877806	NM_001145670, NN	XM_011532380, XM_0	NP_001139142, NP_00	XP_005263364, XP_0052	protein_coding	ENSG0000015455	8470	24098
1654	TMEM67	1.312	0.001	transmembrane protein 67 [Source:HGNC Symbol;Acc:HGNC:28396]	chr8:94767084-94830285	NM_001142301, NN	XM_006716686	NP_001135773, NP_71	XP_006716749	protein_coding	ENSG0000016495	91147	28396
1655	TP53BP2	0.998	0.001	tumor protein p53 binding protein 2 [Source:HGNC Symbol;Acc:HGNC:12000]	chr1:223967600-224033674	NM_001031685, NN	XM_017002220, XM_0	NP_001026855, NP_00	XP_011542570, XP_0115	protein_coding	ENSG0000014351	7159	12000
1656	UBR5	0.944	0.001	ubiquitin protein ligase E3 component n-recognin 5 [Source:HGNC Symbol;Acc:HGNC:16	chr8:103265239-103425069	NM_001282873, NN	XM_011517106, XM_0	NP_001269802, NP_05	XP_011515406, XP_0115	protein_coding	ENSG0000010451	51366	16806
1657	AADAT	1.207	0.002	aminoadipate aminotransferase [Source:HGNC Symbol;Acc:HGNC:17929]	chr4:170981372-171011538	NM_001286682, NN	XM_006714231	NP_001273611, NP_00	XP_006714294	protein_coding	ENSG0000010957	51166	17929
1658	AKAP11	1.046	0.002	A-kinase anchoring protein 11 [Source:HGNC Symbol;Acc:HGNC:369]	chr13:42846288-42897403	NM_016248	XM_017020383, XM_0	NP_057332	XP_005266304, XP_0052	protein_coding	ENSG0000002351	11215	369
1659	ARID5A	-0.920	0.002	AT-rich interaction domain 5A [Source:HGNC Symbol;Acc:HGNC:17361]	chr2:97202485-97218375	NM_001319093, NN	XM_011510501, XM_0	NP_001306021, NP_00	XP_011508801, XP_0115	protein_coding	ENSG0000019684	10865	17361
1660	DUSP9	-1.096	0.002	dual specificity phosphatase 9 [Source:HGNC Symbol;Acc:HGNC:3076]	chrX:152907987-152916781	NM_001318503, NN	XM_011531124, XM_0	NP_001305432, NP_00	XP_005274712, XP_0115	protein_coding	ENSG0000013082	1852	3076
1661	EPB41L1	-0.891	0.002	erythrocyte membrane protein band 4.1 like 1 [Source:HGNC Symbol;Acc:HGNC:3378]	chr20:34679425-34820721	NM_001258329, NN	XM_017027717, XM_0	NP_001245258, NP_00	XP_011526973, XP_0115	protein_coding	ENSG0000008836	2036	3378
1662	ERVMER34-1	0.883	0.002	endogenous retrovirus group MER34 member 1 [Source:HGNC Symbol;Acc:HGNC:4297	chr4:53608718-53617807	NM_001242690, NN	.	NP_001229619, NP_07	.	protein_coding	ENSG0000022688	100288413	42970
1663	FBL	-0.743	0.002	fibrillarin [Source:HGNC Symbol;Acc:HGNC:3599]	chr19:40325097-40337054	NM_001436	XM_011526623, XM_0	NP_001427	XP_005258708, XP_0115	protein_coding	ENSG0000010520	2091	3599
1664	PAWR	0.886	0.002	pro-apoptotic WT1 regulator [Source:HGNC Symbol;Acc:HGNC:8614]	chr12:79978653-80084877	NM_002583	XM_006719436, XM_0	NP_002574	XP_006719498, XP_0067	protein_coding	ENSG0000017742	5074	8614
1665	PHTF2	1.081	0.002	putative homeodomain transcription factor 2 [Source:HGNC Symbol;Acc:HGNC:13411]	chr7:77428108-77586820	NM_001127357, NN	XM_011516423, XM_0	NP_001120829, NP_00	XP_005250565, XP_0052	protein_coding	ENSG0000000657	57157	13411
1666	RFNG	-0.921	0.002	RFNG O-fucosylpeptide 3-beta-N-acetylglucosaminyltransferase [Source:HGNC Symbol;A	chr17:80005777-80009650	NM_002917	XM_011523587	NP_002908	XP_011521889	protein_coding	ENSG0000016973	5986	9974
1667	RIN1	-0.946	0.002	Ras and Rab interactor 1 [Source:HGNC Symbol;Acc:HGNC:18749]	chr11:66099532-66104311	NM_004292	XM_011545401, XM_0	NP_004283	XP_011543701, XP_0115	protein_coding	ENSG0000017479	9610	18749
1668	SIPA1L1	0.892	0.002	signal induced proliferation associated 1 like 1 [Source:HGNC Symbol;Acc:HGNC:20284]	chr14:71996041-72207946	NM_001284245, NN	XM_017021185, XM_0	NP_001271174, NP_00	XP_005267573, XP_0052	protein_coding	ENSG0000019755	26037	20284
1669	SLC52A3	-0.927	0.002	solute carrier family 52 member 3 [Source:HGNC Symbol;Acc:HGNC:16187]	chr20:740723-756658	NM_033409	XM_011529148, XM_0	NP_212134	XP_005260712, XP_0115	protein_coding	ENSG0000010127	113278	16187
1670	VPS28	-0.990	0.002	VPS28, ESCRT-I subunit [Source:HGNC Symbol;Acc:HGNC:18178]	chr8:145648983-145653946	NM_016208, NM_1	XM_011517100, XM_0	NP_057292, NP_89888	XP_005272380, XP_0052	protein_coding	ENSG0000016094	51160	18178
1671	ANKRD18A	1.399	0.002	ankyrin repeat domain 18A [Source:HGNC Symbol;Acc:HGNC:23643]	chr9:38571354-38620657	NM_147195	XM_011517839, XM_0	NP_671728	XP_006716812, XP_0115	protein_coding	ENSG0000018007	253650	23643
1672	CD81	-0.736	0.002	CD81 molecule [Source:HGNC Symbol;Acc:HGNC:1701]	chr11:2398523-2418649	NM_001297649, NN	XM_011520492	NP_001284578, NP_00	XP_011518794, XP_0168	protein_coding	ENSG0000011065	975	1701

1673	DMBX1	-1.273	0.002	diencephalon/mesencephalon homeobox 1 [Source:HGNC Symbol;Acc:HGNC:19026]	chr1:46972667-46979898	NM_147192, NM_1	XM_017000289, XM_0	NP_671725, NP_75737	XP_011538969, XP_0115	protein_coding	ENSG0000019758	127343	19026
1674	GPR108	-0.815	0.002	G protein-coupled receptor 108 [Source:HGNC Symbol;Acc:HGNC:17829]	chr19:6729924-6737614	NM_001080452	XM_011528137, XM_0	NP_001073921	XP_006722864, XP_0115	protein_coding	ENSG0000012573	102465474	17829
1675	MBNL1	0.995	0.002	muscleblind like splicing regulator 1 [Source:HGNC Symbol;Acc:HGNC:6923]	chr3:151985828-152183569	NM_021038, NM_2	XM_017006427, XM_0	NP_001300986, NP_06	XP_005247514, XP_0052	protein_coding	ENSG0000015260	4154	6923
1676	MIS12	0.974	0.002	MIS12, kinetochore complex component [Source:HGNC Symbol;Acc:HGNC:24967]	chr17:5389693-5394134	NM_001258217, NN	XM_005256798, XM_0	NP_001245146, NP_00	XP_005256854, XP_0052	protein_coding	ENSG0000016784	79003	24967
1677	MORC3	1.100	0.002	MORC family CW-type zinc finger 3 [Source:HGNC Symbol;Acc:HGNC:23572]	chr21:37692486-37748944	NM_001320445, NN	XM_017028311, XM_0	NP_001307374, NP_00	XP_011527820, XP_0168	protein_coding	ENSG0000015925	23515	23572
1678	NRDC	0.830	0.002	nardilysin convertase [Source:HGNC Symbol;Acc:HGNC:7995]	chr1:52254862-52344609	NM_001101662, NN	XM_011541525, XM_0	NP_001095132, NP_00	XP_005270960, XP_0115	protein_coding	ENSG0000007861	100313892	7995
1679	NT5C3A	1.005	0.002	5'-nucleotidase, cytosolic IIIA [Source:HGNC Symbol;Acc:HGNC:17820]	chr7:33053725-33102409	NM_001002009, NN	XM_011515409	NP_001002009, NP_00	XP_011513711	protein_coding	ENSG0000012264	51251	17820
1680	PLEK2	-0.790	0.002	pleckstrin 2 [Source:HGNC Symbol;Acc:HGNC:19238]	chr14:67853699-67878917	NM_016445	XM_011536644	NP_057529	XP_011534946	protein_coding	ENSG0000010055	26499	19238
1681	USP28	0.927	0.002	ubiquitin specific peptidase 28 [Source:HGNC Symbol;Acc:HGNC:12625]	chr11:113668595-113746292	NM_001301029, NN	XM_017018062, XM_0	NP_001287958, NP_06	XP_005271687, XP_0052	protein_coding	ENSG0000004802	57646	12625
1682	ZRANB2	0.975	0.002	zinc finger RANBP2-type containing 2 [Source:HGNC Symbol;Acc:HGNC:13058]	chr1:71528973-71546980	NM_005455, NM_2	.	NP_005446, NP_97622	.	protein_coding	ENSG0000013248	9406	13058
1683	ARHGEF40	-1.191	0.002	Rho guanine nucleotide exchange factor 40 [Source:HGNC Symbol;Acc:HGNC:25516]	chr14:21484921-21572881	NM_001278529, NN	XM_011536937, XM_0	NP_001265458, NP_00	XP_005267901, XP_0115	protein_coding	ENSG0000016580	55701	25516
1684	C15orf39	-0.850	0.002	chromosome 15 open reading frame 39 [Source:HGNC Symbol;Acc:HGNC:24497]	chr15:75491232-75504510	NM_015492	XM_011521793, XM_0	NP_056307	XP_005254608, XP_0115	protein_coding	ENSG0000016717	56905	24497
1685	CSF2	-0.946	0.002	colony stimulating factor 2 [Source:HGNC Symbol;Acc:HGNC:2434]	chr5:131409482-131411863	NM_000758	.	NP_000749	.	protein_coding	ENSG0000016440	1437	2434
1686	NUP50	0.896	0.002	nucleoporin 50 [Source:HGNC Symbol;Acc:HGNC:8065]	chr22:45559721-45583896	NM_007172, NM_1	XM_011529833, XM_0	NP_009103, NP_70593	XP_005261369, XP_0052	protein_coding	ENSG0000009300	10762	8065
1687	RNF146	1.279	0.002	ring finger protein 146 [Source:HGNC Symbol;Acc:HGNC:21336]	chr6:127587754-127609712	NM_001242844, NN	XM_011536164, XM_0	NP_001229773, NP_00	XP_006715634, XP_0115	protein_coding	ENSG0000011851	81847	21336
1688	SLC26A6	-0.999	0.002	solute carrier family 26 member 6 [Source:HGNC Symbol;Acc:HGNC:14472]	chr3:48663155-48672926	NM_001040454, NN	.	NP_001035544, NP_00	.	protein_coding	ENSG0000022569	102465495	14472
1689	SMIM1	-1.399	0.002	small integral membrane protein 1 (Vel blood group) [Source:HGNC Symbol;Acc:HGNC:4	chr1:3689351-3692546	NM_001163724, NN	.	NP_001157196, NP_00	.	protein_coding	ENSG0000023516	388588	44204
1690	SMURF1	0.828	0.002	SMAD specific E3 ubiquitin protein ligase 1 [Source:HGNC Symbol;Acc:HGNC:16807]	chr7:98625060-98741723	NM_001199847, NN	XM_017012457, XM_0	NP_001186776, NP_06	XP_016867946	protein_coding	ENSG0000019874	57154	16807
1691	SNX14	0.965	0.002	sorting nexin 14 [Source:HGNC Symbol;Acc:HGNC:14977]	chr6:86215213-86303874	NM_001297614, NN	XM_017011101, XM_0	NP_001284543, NP_00	XP_005248795, XP_0052	protein_coding	ENSG0000013531	57231	14977
1692	TCAF1	0.943	0.002	TRPM8 channel associated factor 1 [Source:HGNC Symbol;Acc:HGNC:22201]	chr7:143548467-143599248	NM_001206938, NN	XM_006716192, XM_0	NP_001193867, NP_05	XP_005250131, XP_0052	protein_coding	ENSG0000019842	9747	22201
1693	WDR92	1.029	0.002	WD repeat domain 92 [Source:HGNC Symbol;Acc:HGNC:25176]	chr2:68356934-68384692	NM_001256476, NN	.	NP_001243405, NP_61	.	protein_coding	ENSG0000024366	116143	25176
1694	APLP1	-0.968	0.002	amyloid beta precursor like protein 1 [Source:HGNC Symbol;Acc:HGNC:597]	chr19:36358800-36370690	NM_001024807, NN	.	NP_001019978, NP_00	.	protein_coding	ENSG0000010529	333	597
1695	BIK	-0.914	0.002	BCL2 interacting killer [Source:HGNC Symbol;Acc:HGNC:1051]	chr22:43506753-43525718	NM_001197	.	NP_001188	.	protein_coding	ENSG0000010029	638	1051
1696	C9orf16	-0.808	0.002	chromosome 9 open reading frame 16 [Source:HGNC Symbol;Acc:HGNC:17823]	chr9:130922538-130926207	NM_024112	.	NP_077017	.	protein_coding	ENSG0000017115	79095	17823
1697	GALT	-1.014	0.002	galactose-1-phosphate uridylyltransferase [Source:HGNC Symbol;Acc:HGNC:4135]	chr9:34646585-34661889	NM_000155, NM_0	.	NP_000146, NP_00124	.	protein_coding	ENSG0000021393	2592	4135
1698	GDAP1	1.394	0.002	ganglioside induced differentiation associated protein 1 [Source:HGNC Symbol;Acc:HGN	chr8:75262616-75279111	NM_001040875, NN	.	NP_001035808, NP_06	.	protein_coding	ENSG0000010438	54332	15968
1699	GRIK4	1.294	0.002	glutamate ionotropic receptor kainate type subunit 4 [Source:HGNC Symbol;Acc:HGNC:	chr11:120382467-120859613	NM_001282470, NN	XM_011542784, XM_0	NP_001269399, NP_00	XP_011541086	protein_coding	ENSG0000014940	2900	4582
1700	MERTK	1.267	0.002	MER proto-oncogene, tyrosine kinase [Source:HGNC Symbol;Acc:HGNC:7027]	chr2:112656055-112787138	NM_006343	XM_011510490, XM_0	NP_006334	XP_005263622, XP_0115	protein_coding	ENSG0000015320	10461	7027
1701	NCR3	-3.411	0.002	natural cytotoxicity triggering receptor 3 [Source:HGNC Symbol;Acc:HGNC:19077]	chr6:31553900-31560762	NM_001145466, NN	XM_011514459, XM_0	NP_001138938, NP_00	XP_006715112, XP_0115	protein_coding	ENSG0000020447	259197	19077
1702	NPHS1	-1.447	0.002	NPHS1, nephrin [Source:HGNC Symbol;Acc:HGNC:7908]	chr19:36316865-36342740	NM_004646	.	NP_004637	.	protein_coding	ENSG0000016127	4868	7908
1703	NUCB1	-0.804	0.002	nucleobindin 1 [Source:HGNC Symbol;Acc:HGNC:8043]	chr19:49403306-49426629	NM_006184	.	NP_006175	XP_016882334	protein_coding	ENSG0000010480	4924	8043
1704	PCP4L1	-1.856	0.002	Purkinje cell protein 4 like 1 [Source:HGNC Symbol;Acc:HGNC:20448]	chr1:161228516-161255240	NM_001102566	.	NP_001096036	XP_016857643	protein_coding	ENSG0000024848	654790	20448
1705	RLF	1.208	0.002	rearranged L-myc fusion [Source:HGNC Symbol;Acc:HGNC:10025]	chr1:40627044-40706593	NM_012421	.	NP_036553	.	protein_coding	ENSG0000011700	6018	10025
1706	RNF5	-0.876	0.002	ring finger protein 5 [Source:HGNC Symbol;Acc:HGNC:10068]	chr6:32146130-32148570	NM_006913	.	NP_008844	.	protein_coding	ENSG0000020430	6048	10068
1707	RPS6KA5	1.290	0.002	ribosomal protein S6 kinase A5 [Source:HGNC Symbol;Acc:HGNC:10434]	chr14:91314205-91526952	NM_004755, NM_1	.	NP_001309158, NP_00	.	protein_coding	ENSG0000010078	9252	10434
1708	RTN4	0.748	0.002	reticulon 4 [Source:HGNC Symbol;Acc:HGNC:14085]	chr2:55199322-55277964	NM_001321859, NN	XM_017004519, XM_0	NP_001308788, NP_00	XP_005264491, XP_0115	protein_coding	ENSG0000011531	57142	14085
1709	SLC35F5	1.026	0.002	solute carrier family 35 member F5 [Source:HGNC Symbol;Acc:HGNC:23617]	chr2:114470368-114514400	NM_025181	XM_011511923, XM_0	NP_079457	XP_005263856, XP_0115	protein_coding	ENSG0000011508	80255	23617
1710	TMEM151A	-1.172	0.002	transmembrane protein 151A [Source:HGNC Symbol;Acc:HGNC:28497]	chr11:66059340-66064135	NM_153266	.	NP_694998	.	protein_coding	ENSG0000017929	256472	28497
1711	TRMT1L	1.123	0.002	tRNA methyltransferase 1 like [Source:HGNC Symbol;Acc:HGNC:16782]	chr1:185087229-185126113	NM_001202423, NN	XM_011510028, XM_0	NP_001189352, NP_11	XP_011508330	protein_coding	ENSG0000012148	81627	16782
1712	ZBTB21	1.140	0.002	zinc finger and BTB domain containing 21 [Source:HGNC Symbol;Acc:HGNC:13083]	chr21:43406939-43430496	NM_001098402, NN	XM_017028361, XM_0	NP_001091872, NP_00	XP_005261178, XP_0052	protein_coding	ENSG0000017327	49854	13083
1713	ZCWPW1	1.202	0.002	zinc finger CW-type and PWWP domain containing 1 [Source:HGNC Symbol;Acc:HGNC:2	chr7:99998475-100026302	NM_001258008, NN	XM_011516366, XM_0	NP_001244937, NP_06	XP_005250536, XP_0052	protein_coding	ENSG0000007848	55063	23486
1714	ARHGEF10	1.043	0.002	Rho guanine nucleotide exchange factor 10 [Source:HGNC Symbol;Acc:HGNC:14103]	chr8:1772141-1906807	NM_001308152, NN	XM_017014003, XM_0	NP_001295081, NP_00	XP_005266097, XP_0052	protein_coding	ENSG0000010472	9639	14103
1715	BAIAP2L2	-1.689	0.002	BAI1 associated protein 2 like 2 [Source:HGNC Symbol;Acc:HGNC:26203]	chr22:38480895-38506677	NM_025045	XM_011530385, XM_0	NP_079321	XP_005261808, XP_0115	protein_coding	ENSG0000012829	80115	26203
1716	DCTN3	-0.855	0.002	dynactin subunit 3 [Source:HGNC Symbol;Acc:HGNC:2713]	chr9:34613541-34620520	NM_001281425, NN	.	NP_001268354, NP_00	.	protein_coding	ENSG0000013710	11258	2713
1717	GPX4	-0.738	0.002	glutathione peroxidase 4 [Source:HGNC Symbol;Acc:HGNC:4556]	chr19:1103924-1106789	NM_001039847, NN	.	NP_001034936, NP_00	.	protein_coding	ENSG0000016746	2879	4556
1718	KRT75	-5.913	0.002	keratin 75 [Source:HGNC Symbol;Acc:HGNC:24431]	chr12:52817853-52828309	NM_004693	.	NP_004684	.	protein_coding	ENSG0000017045	9119	24431
1719	LPGAT1	0.967	0.002	lysophosphatidylglycerol acyltransferase 1 [Source:HGNC Symbol;Acc:HGNC:28985]	chr1:211916798-212004114	NM_001320808, NN	XM_011510229, XM_0	NP_001307737, NP_05	XP_005273419, XP_0052	protein_coding	ENSG0000012368	9926	28985
1720	NBEAL2	-0.981	0.002	neurobeachin like 2 [Source:HGNC Symbol;Acc:HGNC:31928]	chr3:46963215-47051193	NM_015175	XM_017006011, XM_0	NP_055990	XP_006713135, XP_0115	protein_coding	ENSG0000016079	23218	31928
1721	PPP1R15B	0.934	0.002	protein phosphatase 1 regulatory subunit 15B [Source:HGNC Symbol;Acc:HGNC:14951]	chr1:204372514-204380919	NM_032833	.	NP_116222	.	protein_coding	ENSG0000015861	84919	14951
1722	RBAK	1.184	0.002	RB associated KRAB zinc finger [Source:HGNC Symbol;Acc:HGNC:17680]	chr7:5023348-5112852	NM_001204456, NN	.	NP_001191385, NP_06	.	protein_coding	ENSG0000014658	57786	17680
1723	RICTOR	1.189	0.002	RPTOR independent companion of MTOR complex 2 [Source:HGNC Symbol;Acc:HGNC:2	chr5:38938020-39074510	NM_001285439, NN	XM_017009315, XM_0	NP_001272368, NP_00	XP_006714526, XP_0115	protein_coding	ENSG0000016432	253260	28611
1724	SLC39A8	0.953	0.002	solute carrier family 39 member 8 [Source:HGNC Symbol;Acc:HGNC:20862]	chr4:103172197-103266631	NM_001135146, NN	XM_017008540, XM_0	NP_001128618, NP_00	XP_005263234, XP_0168	protein_coding	ENSG0000013882	64116	20862
1725	TMEM115	-1.092	0.002	transmembrane protein 115 [Source:HGNC Symbol;Acc:HGNC:30055]	chr3:50384918-50541675	NM_007024	.	NP_008955	.	protein_coding	ENSG0000012606	11070	30055
1726	UTP15	1.154	0.002	UTP15, small subunit processome component [Source:HGNC Symbol;Acc:HGNC:25758]	chr5:72861565-72879202	NM_001284430, NN	XM_011543680	NP_001271359, NP_00	XP_011541982	protein_coding	ENSG0000016433	84135	25758

1727	ACADM	1.054	0.002	acyl-CoA dehydrogenase, C-4 to C-12 straight chain [Source:HGNC Symbol;Acc:HGNC:89	chr1:76190031-76229364	NM_000016, NM_0	.	NP_000007, NP_00112	.	protein_coding	ENSG0000011705	34	89
1728	AP2M1	-0.730	0.002	adaptor related protein complex 2 mu 1 subunit [Source:HGNC Symbol;Acc:HGNC:564]	chr3:183892476-183901879	NM_001025205, NN	.	NP_001020376, NP_00	.	protein_coding	ENSG0000016120	1173	564
1729	ARHGEF3	0.896	0.002	Rho guanine nucleotide exchange factor 3 [Source:HGNC Symbol;Acc:HGNC:683]	chr3:56761445-57113336	NM_001128615, NN	XM_011533767, XM_0	NP_001122087, NP_00	XP_005265243, XP_0052	protein_coding	ENSG0000016394	50650	683
1730	ATF6	0.909	0.002	activating transcription factor 6 [Source:HGNC Symbol;Acc:HGNC:791]	chr1:161736083-161933860	NM_007348	XM_011509309, XM_0	NP_031374	XP_006711287, XP_0115	protein_coding	ENSG0000011821	22926	791
1731	CARNS1	-1.202	0.002	carnosine synthase 1 [Source:HGNC Symbol;Acc:HGNC:29268]	chr11:67183148-67193078	NM_001166222, NN	XM_011545190, XM_0	NP_001159694, NP_06	XP_011543491, XP_0115	protein_coding	ENSG0000017250	57571	29268
1732	CD320	-0.856	0.002	CD320 molecule [Source:HGNC Symbol;Acc:HGNC:16692]	chr19:8367010-8373239	NM_001165895, NN	.	NP_001159367, NP_05	.	protein_coding	ENSG0000016777	51293	16692
1733	DOCK11	1.259	0.002	dedicator of cytokinesis 11 [Source:HGNC Symbol;Acc:HGNC:23483]	chrX:117629860-117820126	NM_144658	XM_011531277, XM_0	NP_653259	XP_005262425, XP_0052	protein_coding	ENSG0000014725	139818	23483
1734	ENKD1	-0.961	0.002	enkurin domain containing 1 [Source:HGNC Symbol;Acc:HGNC:25246]	chr16:67696847-67700667	NM_032140	.	NP_115516	.	protein_coding	ENSG0000012407	84080	25246
1735	FAM20B	0.916	0.002	family with sequence similarity 20 member B [Source:HGNC Symbol;Acc:HGNC:23017]	chr1:178995030-179045697	NM_001324310, NN	XM_011510225, XM_0	NP_001311239, NP_00	XP_011508526, XP_0115	protein_coding	ENSG0000011619	9917	23017
1736	JUNB	-0.829	0.002	JunB proto-oncogene, AP-1 transcription factor subunit [Source:HGNC Symbol;Acc:HGN	chr19:12902309-12904129	NM_002229	.	NP_002220	.	protein_coding	ENSG0000017122	3726	6205
1737	KLHL12	0.897	0.002	kelch like family member 12 [Source:HGNC Symbol;Acc:HGNC:19360]	chr1:202860227-202896390	NM_001303051, NN	XM_011509835	NP_001289980, NP_00	XP_011508137	protein_coding	ENSG0000011715	59349	19360
1738	KLHL23	1.022	0.002	kelch like family member 23 [Source:HGNC Symbol;Acc:HGNC:27506]	chr2:170550963-170608398	NM_001199290, NN	.	NP_001186219, NP_65	.	protein_coding	ENSG0000021316	100526832	27506
1739	LRPAP1	-0.804	0.002	LDL receptor related protein associated protein 1 [Source:HGNC Symbol;Acc:HGNC:670	chr4:3508102-3534286	NM_002337	.	NP_002328	.	protein_coding	ENSG0000016395	4043	6701
1740	NAB1	0.964	0.002	NGFI-A binding protein 1 [Source:HGNC Symbol;Acc:HGNC:7626]	chr2:191513795-191557492	NM_005966	XM_011511223, XM_0	NP_001308241, NP_00	XP_005246636, XP_0052	protein_coding	ENSG0000013838	4664	7626
1741	PRKCSH	-0.761	0.002	protein kinase C substrate 80K-H [Source:HGNC Symbol;Acc:HGNC:9411]	chr19:11531271-11561783	NM_001001329, NN	XM_017026977, XM_0	NP_001001329, NP_00	XP_011526432, XP_0115	protein_coding	ENSG0000013017	5589	9411
1742	PTER	1.237	0.002	phosphotriesterase related [Source:HGNC Symbol;Acc:HGNC:9590]	chr10:16478941-16564004	NM_001001484, NN	XM_017016930, XM_0	NP_001001484, NP_00	XP_016872416, XP_0168	protein_coding	ENSG0000016598	9317	9590
1743	SYNM	1.026	0.002	synemin [Source:HGNC Symbol;Acc:HGNC:24466]	chr15:99638419-99675798	NM_015286, NM_1	.	NP_056101, NP_66378	.	protein_coding	ENSG0000018225	23336	24466
1744	TINCR	-1.446	0.002	tissue differentiation-inducing non-protein coding RNA [Source:HGNC Symbol;Acc:HGN	chr19:5558177-5568045	.	.	.	.	protein_coding	ENSG0000022357	.	14607
1745	TMEM43	-0.835	0.002	transmembrane protein 43 [Source:HGNC Symbol;Acc:HGNC:28472]	chr3:14166439-14185179	NM_024334	XM_011534109	NP_077310	XP_016862665	protein_coding	ENSG0000017087	79188	28472
1746	ACTR2	0.729	0.002	ARP2 actin related protein 2 homolog [Source:HGNC Symbol;Acc:HGNC:169]	chr2:65454886-65498387	NM_001005386, NN	.	NP_001005386, NP_00	.	protein_coding	ENSG0000013807	10097	169
1747	ADAT1	1.071	0.002	adenosine deaminase, tRNA specific 1 [Source:HGNC Symbol;Acc:HGNC:228]	chr16:75633899-75657198	NM_012091	.	NP_001311374, NP_03	.	protein_coding	ENSG0000006545	23536	228
1748	DYNC1I2	0.877	0.002	dynein cytoplasmic 1 intermediate chain 2 [Source:HGNC Symbol;Acc:HGNC:2964]	chr2:172543918-172604930	NM_001271785, NN	XM_017003525, XM_0	NP_001258714, NP_00	XP_005246423, XP_0052	protein_coding	ENSG0000007738	1781	2964
1749	EPB41L4A	0.897	0.002	erythrocyte membrane protein band 4.1 like 4A [Source:HGNC Symbol;Acc:HGNC:13278	chr5:111498314-111755013	NM_022140	XM_011543533, XM_0	NP_071423	XP_011541832, XP_0115	protein_coding	ENSG0000012959	64097	13278
1750	JPH1	1.082	0.002	junctophilin 1 [Source:HGNC Symbol;Acc:HGNC:14201]	chr8:75146934-75233563	NM_020647	XM_005251275, XM_0	NP_001304759, NP_06	XP_005251330, XP_0052	protein_coding	ENSG0000010436	56704	14201
1751	N4BP3	-0.934	0.002	NEDD4 binding protein 3 [Source:HGNC Symbol;Acc:HGNC:29852]	chr5:177540443-177553088	NM_015111	XM_011534474, XM_0	NP_055926	XP_006714897, XP_0115	protein_coding	ENSG0000014591	23138	29852
1752	NAE1	1.009	0.002	NEDD8 activating enzyme E1 subunit 1 [Source:HGNC Symbol;Acc:HGNC:621]	chr16:66836777-66864900	NM_001018159, NN	XM_011523423, XM_0	NP_001018169, NP_00	XP_005256272, XP_0115	protein_coding	ENSG0000015959	8883	621
1753	NEMP2	1.174	0.002	nuclear envelope integral membrane protein 2 [Source:HGNC Symbol;Acc:HGNC:33700	chr2:191369067-191399448	NM_001142645	XM_011510456, XM_0	NP_001136117	XP_005246270, XP_0115	protein_coding	ENSG0000018936	100131211	33700
1754	SERPINB2	-1.590	0.002	serpin family B member 2 [Source:HGNC Symbol;Acc:HGNC:8584]	chr18:61554933-61571124	NM_001143818, NN	.	NP_001137290, NP_00	.	protein_coding	ENSG0000019763	5055	8584
1755	SPIN1	0.874	0.002	spindlin 1 [Source:HGNC Symbol;Acc:HGNC:11243]	chr9:91003333-91093609	NM_006717	.	NP_006708	.	protein_coding	ENSG0000010672	10927	11243
1756	TRMT5	1.120	0.002	tRNA methyltransferase 5 [Source:HGNC Symbol;Acc:HGNC:23141]	chr14:61438168-61550430	NM_020810	XM_005267916	NP_065861	XP_005267973, XP_0115	protein_coding	ENSG0000012681	57570	23141
1757	ACOT9	-0.851	0.002	acyl-CoA thioesterase 9 [Source:HGNC Symbol;Acc:HGNC:17152]	chrX:23720369-23784592	NM_001033583, NN	XM_005274472, XM_0	NP_001028755, NP_00	XP_005274529	protein_coding	ENSG0000012313	23597	17152
1758	ARRB1	-0.949	0.002	arrestin beta 1 [Source:HGNC Symbol;Acc:HGNC:711]	chr11:74975225-75062749	NM_004041, NM_0	XM_011545036, XM_0	NP_004032, NP_06464	XP_011543336, XP_0115	protein_coding	ENSG0000013748	408	711
1759	BEX2	-0.990	0.002	brain expressed X-linked 2 [Source:HGNC Symbol;Acc:HGNC:30933]	chrX:102564273-102565974	NM_001168399, NN	.	NP_001161871, NP_00	.	protein_coding	ENSG0000013313	84707	30933
1760	CEBPB	-0.862	0.002	CCAAT/enhancer binding protein beta [Source:HGNC Symbol;Acc:HGNC:1834]	chr20:48807270-48809226	NM_001285878, NN	.	NP_001272807, NP_00	.	protein_coding	ENSG0000017221	1051	1834
1761	HMGCR	0.803	0.002	3-hydroxy-3-methylglutaryl-CoA reductase [Source:HGNC Symbol;Acc:HGNC:5006]	chr5:74632153-74657929	NM_000859, NM_0	XM_011543359, XM_0	NP_000850, NP_00112	XP_011541659, XP_0115	protein_coding	ENSG0000011316	3156	5006
1762	JKAMP	1.071	0.002	JNK1/MAPK8-associated membrane protein [Source:HGNC Symbol;Acc:HGNC:20184]	chr14:59951160-60043549	NM_001098625, NN	.	NP_001092095, NP_00	.	protein_coding	ENSG0000005013	51528	20184
1763	MON2	1.222	0.002	MON2 homolog, regulator of endosome-to-Golgi trafficking [Source:HGNC Symbol;Acc:I	chr12:62860596-62994259	NM_001278469, NN	XM_011538052, XM_0	NP_001265398, NP_00	XP_016874530, XP_0168	protein_coding	ENSG0000006198	23041	29177
1764	PTGIR	-2.339	0.002	prostaglandin I2 (prostacyclin) receptor (IP) [Source:HGNC Symbol;Acc:HGNC:9602]	chr19:47123724-47128375	NM_000960	XM_005259093	NP_000951	XP_005259150	protein_coding	ENSG0000016001	5739	9602
1765	RHEB	0.829	0.002	Ras homolog enriched in brain [Source:HGNC Symbol;Acc:HGNC:10011]	chr7:151163097-151217206	NM_005614	XM_011516457	NP_005605	XP_011514759	protein_coding	ENSG0000010661	6009	10011
1766	STAT1	0.843	0.002	signal transducer and activator of transcription 1 [Source:HGNC Symbol;Acc:HGNC:1136	chr2:191833759-191878976	NM_007315, NM_1	XM_006712718	NP_009330, NP_64467	XP_006712781, XP_0168	protein_coding	ENSG0000011541	6772	11362
1767	TMEM61	-1.557	0.002	transmembrane protein 61 [Source:HGNC Symbol;Acc:HGNC:27296]	chr1:55446464-55457966	NM_182532	XM_011540911	NP_872338	XP_005270643, XP_0115	protein_coding	ENSG0000014300	199964	27296
1768	UBXN10	-0.948	0.002	UBX domain protein 10 [Source:HGNC Symbol;Acc:HGNC:26354]	chr1:20512577-20522541	NM_152376	XM_011540699, XM_0	NP_689589	XP_005245799, XP_0115	protein_coding	ENSG0000016254	127733	26354
1769	ALAS1	-0.767	0.002	5'-aminolevulinate synthase 1 [Source:HGNC Symbol;Acc:HGNC:396]	chr3:52232101-52248343	NM_000688, NM_0	XM_011533478, XM_0	NP_000679, NP_00129	XP_011531779, XP_0115	protein_coding	ENSG0000002333	211	396
1770	ANXA6	-0.726	0.002	annexin A6 [Source:HGNC Symbol;Acc:HGNC:544]	chr5:150480665-150537443	NM_001155, NM_0	XM_005268432	NP_001146, NP_00118	XP_005268489	protein_coding	ENSG0000019704	309	544
1771	CCDC71	-0.985	0.002	coiled-coil domain containing 71 [Source:HGNC Symbol;Acc:HGNC:25760]	chr3:49199967-49203754	NM_022903	.	NP_075054	.	protein_coding	ENSG0000017735	64925	25760
1772	EXD3	-1.200	0.002	exonuclease 3'-5' domain containing 3 [Source:HGNC Symbol;Acc:HGNC:26023]	chr9:140201356-140317714	NM_001286823, NN	XM_011518810, XM_0	NP_001273752, NP_06	XP_011517112	protein_coding	ENSG0000018760	54932	26023
1773	FNBP1L	1.028	0.002	formin binding protein 1 like [Source:HGNC Symbol;Acc:HGNC:20851]	chr1:93913687-94020218	NM_001024948, NN	XM_011541625	NP_001020119, NP_00	XP_011539927	protein_coding	ENSG0000013794	54874	20851
1774	LANCL1	1.149	0.002	LanC like 1 [Source:HGNC Symbol;Acc:HGNC:6508]	chr2:211295972-211341499	NM_001136574, NN	XM_005246243	NP_001130046, NP_00	XP_005246300	protein_coding	ENSG0000011536	10314	6508
1775	MGAT4A	1.671	0.002	mannosyl (alpha-1,3-)-glycoprotein beta-1,4-N-acetylglucosaminyltransferase, isozyme	chr2:99235568-99347589	NM_001160154, NN	.	NP_001153626, NP_03	.	protein_coding	ENSG0000007107	11320	7047
1776	NSRP1	1.152	0.002	nuclear speckle splicing regulatory protein 1 [Source:HGNC Symbol;Acc:HGNC:25305]	chr17:28443798-28513493	NM_001261467, NN	XM_017025212	NP_001248396, NP_11	XP_011523647, XP_0168	protein_coding	ENSG0000012665	494335	25305
1777	RYR1	-1.000	0.002	ryanodine receptor 1 [Source:HGNC Symbol;Acc:HGNC:10483]	chr19:38924338-39078204	NM_000540, NM_0	XM_011527205, XM_0	NP_000531, NP_00103	XP_006723380, XP_0067	protein_coding	ENSG0000019621	6261	10483
1778	SLC12A2	1.040	0.002	solute carrier family 12 member 2 [Source:HGNC Symbol;Acc:HGNC:10911]	chr5:127419457-127525380	NM_001046, NM_0	.	NP_001037, NP_00124	.	protein_coding	ENSG0000006465	6558	10911
1779	TLX2	-2.562	0.002	T-cell leukemia homeobox 2 [Source:HGNC Symbol;Acc:HGNC:5057]	chr2:74740685-74744274	NM_016170	.	NP_057254	.	protein_coding	ENSG0000011529	3196	5057
1780	YPEL4	-1.300	0.002	yippee like 4 [Source:HGNC Symbol;Acc:HGNC:18328]	chr11:57412560-57417417	NM_145008	XM_011544801	NP_659445	XP_011543103, XP_0168	protein_coding	ENSG0000016679	219539	18328

1781	ZKSCAN1	0.886	0.002	zinc finger with KRAB and SCAN domains 1 [Source:HGNC Symbol;Acc:HGNC:13101]	chr7:99613194-99639312	NM_001287054, N	XM_011516559	NP_001273983, NP_00	XP_011514861	protein_coding	ENSG0000010626	7586	13101
1782	ZNF24	1.120	0.002	zinc finger protein 24 [Source:HGNC Symbol;Acc:HGNC:13032]	chr18:32912175-32924420	NM_001308123, N	XM_005258342, XM_0	NP_001295052, NP_00	XP_005258398, XP_0052	protein_coding	ENSG0000017246	7572	13032
1783	AFF1	0.924	0.002	AF4/FMR2 family member 1 [Source:HGNC Symbol;Acc:HGNC:7135]	chr4:87856153-88062206	NM_001166693, N	XM_011531974, XM_0	NP_001160165, NP_00	XP_005263064, XP_0052	protein_coding	ENSG0000017249	4299	7135
1784	FCGRT	-0.839	0.002	Fc fragment of IgG receptor and transporter [Source:HGNC Symbol;Acc:HGNC:3621]	chr19:50015535-50029590	NM_001136019, N	XM_011526627	NP_001129491, NP_00	XP_011524929	protein_coding	ENSG0000010487	2217	3621
1785	IARS2	0.816	0.002	isoleucyl-tRNA synthetase 2, mitochondrial [Source:HGNC Symbol;Acc:HGNC:29685]	chr1:220267443-220321383	NM_018060	.	NP_060530	.	protein_coding	ENSG0000006770	55699	29685
1786	MAK16	1.371	0.002	MAK16 homolog [Source:HGNC Symbol;Acc:HGNC:13703]	chr8:33342267-33371119	NM_032509	.	NP_115898	.	protein_coding	ENSG0000019804	84549	13703
1787	PNMT	-1.625	0.002	phenylethanolamine N-methyltransferase [Source:HGNC Symbol;Acc:HGNC:9160]	chr17:37824233-37826728	NM_002686	XM_011524909	NP_002677	XP_011523211	protein_coding	ENSG0000014174	5409	9160
1788	PRDX2	-0.726	0.002	peroxiredoxin 2 [Source:HGNC Symbol;Acc:HGNC:9353]	chr19:12907633-12912673	NM_005809	.	NP_005800	.	protein_coding	ENSG0000016781	7001	9353
1789	PSMC2	0.762	0.002	proteasome 26S subunit, ATPase 2 [Source:HGNC Symbol;Acc:HGNC:9548]	chr7:102985360-103086624	NM_001204453, N	XM_005250505	NP_001191382, NP_00	XP_005250562	protein_coding	ENSG0000016105	5701	9548
1790	SLC25A37	0.885	0.002	solute carrier family 25 member 37 [Source:HGNC Symbol;Acc:HGNC:29786]	chr8:23386317-23432976	NM_016612	.	NP_057696	.	protein_coding	ENSG0000014745	51312	29786
1791	SRSF6	0.756	0.002	serine and arginine rich splicing factor 6 [Source:HGNC Symbol;Acc:HGNC:10788]	chr20:42086567-42092887	NM_006275	.	NP_006266	.	protein_coding	ENSG0000012419	6431	10788
1792	TTF2	0.823	0.002	transcription termination factor 2 [Source:HGNC Symbol;Acc:HGNC:12398]	chr1:117602924-117650075	NM_003594	XM_011542305, XM_0	NP_003585	XP_005271334, XP_0115	protein_coding	ENSG0000011683	8458	12398
1793	ZNF365	1.186	0.002	zinc finger protein 365 [Source:HGNC Symbol;Acc:HGNC:18194]	chr10:64133950-64431771	NM_014951, NM_1	XM_011539532	NP_055766, NP_95552	XP_016871426	protein_coding	ENSG0000013831	22891	18194
1794	AZIN2	-1.172	0.002	antizyme inhibitor 2 [Source:HGNC Symbol;Acc:HGNC:29957]	chr1:33546713-33586130	NM_001293562, N	XM_011540557, XM_0	NP_001280491, NP_00	XP_005270461, XP_0052	protein_coding	ENSG0000014292	113451	29957
1795	BCL3	-0.999	0.002	B-cell CLL/lymphoma 3 [Source:HGNC Symbol;Acc:HGNC:998]	chr19:45251803-45263301	NM_005178	XM_011527200, XM_0	NP_005169	XP_011525499, XP_0115	protein_coding	ENSG0000006939	102465879	998
1796	CUEDC2	-0.765	0.002	CUE domain containing 2 [Source:HGNC Symbol;Acc:HGNC:28352]	chr10:104183001-104192418	NM_024040	.	NP_076945	.	protein_coding	ENSG0000010787	79004	28352
1797	DTL	0.920	0.002	denticleless E3 ubiquitin protein ligase homolog [Source:HGNC Symbol;Acc:HGNC:3028	chr1:212208894-212278348	NM_001286229, N	XM_011509614	NP_001273158, NP_00	XP_011507916	protein_coding	ENSG0000014347	51514	30288
1798	FAM72B	1.283	0.002	family with sequence similarity 72 member B [Source:HGNC Symbol;Acc:HGNC:24805]	chr1:120839004-120855681	NM_001100910, N	XM_011542020, XM_0	NP_001094380, NP_00	XP_011540320, XP_0115	protein_coding	ENSG0000018861	653820	24805
1799	KLHDC10	0.892	0.002	kelch domain containing 10 [Source:HGNC Symbol;Acc:HGNC:22194]	chr7:129710349-129775560	NM_014997	XM_005250233	NP_055812	XP_005250290	protein_coding	ENSG0000012860	23008	22194
1800	LARS	0.882	0.002	leucyl-tRNA synthetase [Source:HGNC Symbol;Acc:HGNC:6512]	chr5:145492600-145562223	NM_001317965, N	XM_011537657, XM_0	NP_001304893, NP_00	XP_011535958	protein_coding	ENSG0000013370	51520	6512
1801	MAP4K5	1.093	0.002	mitogen-activated protein kinase kinase kinase kinase 5 [Source:HGNC Symbol;Acc:HGN	chr14:50885242-50999327	NM_006575, NM_1	XM_011536378, XM_0	NP_006566, NP_94208	XP_006720076, XP_0115	protein_coding	ENSG0000001298	11183	6867
1802	NAT14	-0.800	0.002	N-acetyltransferase 14 (putative) [Source:HGNC Symbol;Acc:HGNC:28918]	chr19:55996370-55998935	NM_020378	.	NP_065111	.	protein_coding	ENSG0000009097	57106	28918
1803	UHRF1BP1L	1.217	0.002	UHRF1 binding protein 1 like [Source:HGNC Symbol;Acc:HGNC:29102]	chr12:100430853-100536626	NM_001006947, N	XM_005268739, XM_0	NP_001006948, NP_05	XP_005268795, XP_0052	protein_coding	ENSG0000011164	23074	29102
1804	ZBTB18	1.089	0.002	zinc finger and BTB domain containing 18 [Source:HGNC Symbol;Acc:HGNC:13030]	chr1:244212240-244220778	NM_001278196, N	XM_005273006	NP_001265125, NP_00	XP_005273063, XP_0168	protein_coding	ENSG0000017945	10472	13030
1805	ZNF217	0.889	0.002	zinc finger protein 217 [Source:HGNC Symbol;Acc:HGNC:13009]	chr20:52183603-52210378	NM_006526	XM_017028059	NP_006517	XP_005260602, XP_0067	protein_coding	ENSG0000017194	7764	13009
1806	BCAS2	1.029	0.002	breast carcinoma amplified sequence 2 [Source:HGNC Symbol;Acc:HGNC:975]	chr1:115110177-115124260	NM_005872	.	NP_005863	.	protein_coding	ENSG0000011675	10286	975
1807	C3orf18	-1.494	0.002	chromosome 3 open reading frame 18 [Source:HGNC Symbol;Acc:HGNC:24837]	chr3:50595455-50633599	NM_001171740, N	XM_011533790, XM_0	NP_001165211, NP_00	XP_011532084, XP_0115	protein_coding	ENSG0000008854	51161	24837
1808	CCDC82	1.115	0.002	coiled-coil domain containing 82 [Source:HGNC Symbol;Acc:HGNC:26282]	chr11:96085932-96121068	NM_001318736, N	XM_011542989, XM_0	NP_001305665, NP_07	XP_005274349, XP_0067	protein_coding	ENSG0000014923	79780	26282
1809	DLG1	0.877	0.002	discs large MAGUK scaffold protein 1 [Source:HGNC Symbol;Acc:HGNC:2900]	chr3:196769430-197026171	NM_001098424, N	XM_017005810, XM_0	NP_001091894, NP_00	XP_005269346, XP_0115	protein_coding	ENSG0000007571	1739	2900
1810	EHMT2	-0.823	0.002	euchromatic histone lysine methyltransferase 2 [Source:HGNC Symbol;Acc:HGNC:1412	chr6:31847535-31865461	NM_001289413, N	XM_017010202, XM_0	.	XP_005248881, XP_0067	protein_coding	ENSG0000020437	10919	14129
1811	GALNT16	-1.809	0.002	polypeptide N-acetylgalactosaminyltransferase 16 [Source:HGNC Symbol;Acc:HGNC:232	chr14:69726680-69823750	NM_001168368, N	XM_011537007, XM_0	NP_001161840, NP_06	XP_011535306, XP_0115	protein_coding	ENSG0000010062	57452	23233
1812	PREX1	-0.993	0.002	phosphatidylinositol-3,4,5-trisphosphate dependent Rac exchange factor 1 [Source:HGN	chr20:47240789-47444420	NM_020820	.	NP_065871	.	protein_coding	ENSG0000012412	57580	32594
1813	TM2D2	0.961	0.002	TM2 domain containing 2 [Source:HGNC Symbol;Acc:HGNC:24127]	chr8:38846326-38854052	NM_001024380, N	XM_011544667, XM_0	NP_001019551, NP_00	XP_005273714, XP_0067	protein_coding	ENSG0000016949	83877	24127
1814	TMEM38A	-1.064	0.002	transmembrane protein 38A [Source:HGNC Symbol;Acc:HGNC:28462]	chr19:16771937-16800840	NM_024074	.	NP_076979	.	protein_coding	ENSG0000007295	79041	28462
1815	ADNP	0.777	0.002	activity dependent neuroprotector homeobox [Source:HGNC Symbol;Acc:HGNC:15766]	chr20:49505455-49547777	NM_001282531, N	XM_017027759, XM_0	NP_001269460, NP_00	XP_011527049, XP_0115	protein_coding	ENSG0000010112	23394	15766
1816	AZGP1	-1.136	0.002	alpha-2-glycoprotein 1, zinc-binding [Source:HGNC Symbol;Acc:HGNC:910]	chr7:99564352-99573780	NM_001185	.	NP_001176	.	protein_coding	ENSG0000016086	563	910
1817	B3GALNT1	-3.429	0.002	beta-1,3-N-acetylgalactosaminyltransferase 1 (globoside blood group) [Source:HGNC Sy	chr3:160801670-160823158	NM_001038628, N	XM_017007412, XM_0	NP_001033717, NP_00	XP_005247916, XP_0052	protein_coding	ENSG0000016925	8706	918
1818	CDC42SE2	0.946	0.002	CDC42 small effector 2 [Source:HGNC Symbol;Acc:HGNC:18547]	chr5:130599701-130730383	NM_001038702, N	.	NP_001033791, NP_06	XP_016865137, XP_0168	protein_coding	ENSG0000015898	56990	18547
1819	COG5	1.452	0.002	component of oligomeric golgi complex 5 [Source:HGNC Symbol;Acc:HGNC:14857]	chr7:106809405-107218909	NM_001161520, N	.	NP_001154992, NP_00	.	protein_coding	ENSG0000016459	10466	14857
1820	DLC1	1.166	0.002	DLC1 Rho GTPase activating protein [Source:HGNC Symbol;Acc:HGNC:2897]	chr8:12940869-13372396	NM_001164271, N	XM_017012951, XM_0	NP_001157743, NP_00	XP_005273431, XP_0052	protein_coding	ENSG0000016474	10395	2897
1821	ESRP1	0.852	0.002	epithelial splicing regulatory protein 1 [Source:HGNC Symbol;Acc:HGNC:25966]	chr8:95653301-95719694	NM_001034915, N	XM_005250992, XM_0	NP_001030087, NP_00	XP_005251048, XP_0052	protein_coding	ENSG0000010441	54845	25966
1822	GOLGA4	1.023	0.002	golgin A4 [Source:HGNC Symbol;Acc:HGNC:4427]	chr3:37284667-37408242	NM_001172713, N	XM_017006186, XM_0	NP_001166184, NP_00	XP_005265126, XP_0052	protein_coding	ENSG0000014467	2803	4427
1823	LRP12	1.139	0.002	LDL receptor related protein 12 [Source:HGNC Symbol;Acc:HGNC:31708]	chr8:105501458-105601252	NM_001135703, N	.	NP_001129175, NP_03	.	protein_coding	ENSG0000014765	29967	31708
1824	MED23	1.086	0.002	mediator complex subunit 23 [Source:HGNC Symbol;Acc:HGNC:2372]	chr6:131894283-131949369	NM_001270521, N	XM_011536257, XM_0	NP_001257450, NP_00	XP_005267280, XP_0067	protein_coding	ENSG0000011228	9439	2372
1825	MPHOSPH10	0.935	0.002	M-phase phosphoprotein 10 [Source:HGNC Symbol;Acc:HGNC:7213]	chr2:71357443-71377231	NM_005791	.	NP_005782	.	protein_coding	ENSG0000012438	10199	7213
1826	PIM1	-0.814	0.002	Pim-1 proto-oncogene, serine/threonine kinase [Source:HGNC Symbol;Acc:HGNC:8986]	chr6:37137978-37143202	NM_001243186, N	.	NP_001230115, NP_00	.	protein_coding	ENSG0000013719	5292	8986
1827	POLR1D	-0.767	0.002	RNA polymerase I subunit D [Source:HGNC Symbol;Acc:HGNC:20422]	chr13:28195055-28318374	NM_001206559, N	XM_005266414, XM_0	NP_001193488, NP_05	XP_005266469, XP_0052	protein_coding	ENSG0000018618	51082	20422
1828	SECISBP2L	1.021	0.002	SECIS binding protein 2 like [Source:HGNC Symbol;Acc:HGNC:28997]	chr15:49280834-49338760	NM_001193489, N	.	NP_001180418, NP_05	.	protein_coding	ENSG0000013859	9728	28997
1829	TMEM9	-0.797	0.002	transmembrane protein 9 [Source:HGNC Symbol;Acc:HGNC:18823]	chr1:201103899-201140702	NM_001288564, N	XM_011509387, XM_0	NP_001275493, NP_00	XP_011507687, XP_0115	protein_coding	ENSG0000011685	252839	18823
1830	YME1L1	1.001	0.002	YME1 like 1 ATPase [Source:HGNC Symbol;Acc:HGNC:12843]	chr10:27399040-27475853	NM_001253866, N	XM_011519300	NP_001240795, NP_05	XP_011517602	protein_coding	ENSG0000013675	10730	12843
1831	ZCCHC6	1.252	0.002	zinc finger CCHC-type containing 6 [Source:HGNC Symbol;Acc:HGNC:25817]	chr9:88902647-88969369	NM_001185059, N	XM_011519017, XM_0	NP_001171988, NP_00	XP_005252264, XP_0052	protein_coding	ENSG0000008322	79670	25817
1832	CILP2	-1.040	0.002	cartilage intermediate layer protein 2 [Source:HGNC Symbol;Acc:HGNC:24213]	chr19:19649056-19657468	NM_153221	.	NP_694953	.	protein_coding	ENSG0000016016	148113	24213
1833	DCTN4	0.850	0.002	dynactin subunit 4 [Source:HGNC Symbol;Acc:HGNC:15518]	chr5:150088001-150138671	NM_001135643, N	XM_011537646, XM_0	NP_001129115, NP_00	XP_005268514, XP_0115	protein_coding	ENSG0000013291	51164	15518
1834	EPHB3	-1.046	0.002	EPH receptor B3 [Source:HGNC Symbol;Acc:HGNC:3394]	chr3:184279571-184300197	NM_004443	.	NP_004434	.	protein_coding	ENSG0000018258	2049	3394

1835	RPL18A	-0.731	0.002	ribosomal protein L18a [Source:HGNC Symbol;Acc:HGNC:10311]	chr19:17970684-17974962	NM_000980	.	NP_000971	.	protein_coding	ENSG0000010564	6142	10311
1836	SHANK1	-1.173	0.002	SH3 and multiple ankyrin repeat domains 1 [Source:HGNC Symbol;Acc:HGNC:15474]	chr19:51165083-51222707	NM_016148	XM_011527014, XM_0	NP_057232	XP_006723296, XP_0115	protein_coding	ENSG0000016168	50944	15474
1837	SLC16A9	1.251	0.002	solute carrier family 16 member 9 [Source:HGNC Symbol;Acc:HGNC:23520]	chr10:61410518-61495760	NM_194298	XM_011539488	NP_001310910, NP_91	XP_011537790, XP_0168	protein_coding	ENSG0000016544	220963	23520
1838	SMC4	0.879	0.002	structural maintenance of chromosomes 4 [Source:HGNC Symbol;Acc:HGNC:14013]	chr3:159974773-160167617	NM_001002800, NM	XM_011512313, XM_0	NP_001002800, NP_00	XP_006713522, XP_0115	protein_coding	ENSG0000011381	10051	14013
1839	TNPO3	0.797	0.002	transportin 3 [Source:HGNC Symbol;Acc:HGNC:17103]	chr7:128594233-128695227	NM_001191028, NM	XM_011515989	NP_001177957, NP_03	XP_011514291	protein_coding	ENSG0000006441	23534	17103
1840	TTYH3	-0.761	0.002	tweety family member 3 [Source:HGNC Symbol;Acc:HGNC:22222]	chr7:2671584-2704436	NM_025250	XM_017012656, XM_0	NP_079526	XP_011513837, XP_0168	protein_coding	ENSG0000013629	80727	22222
1841	C9orf3	-0.985	0.002	chromosome 9 open reading frame 3 [Source:HGNC Symbol;Acc:HGNC:1361]	chr9:97488982-97849441	NM_001193329, NM	XM_011519128, XM_0	NP_001180258, NP_00	XP_005252341, XP_0067	protein_coding	ENSG0000014812	407012	1361
1842	CCDC88B	-1.792	0.002	coiled-coil domain containing 88B [Source:HGNC Symbol;Acc:HGNC:26757]	chr11:64107694-64125006	NM_032251	XM_011544962, XM_0	NP_115627	XP_006718582, XP_0115	protein_coding	ENSG0000016807	283234	26757
1843	KIDINS220	0.955	0.002	kinase D-interacting substrate 220kDa [Source:HGNC Symbol;Acc:HGNC:29508]	chr2:8868986-8977760	NM_020738	.	NP_065789	.	protein_coding	ENSG0000013431	57498	29508
1844	MBTD1	1.199	0.002	mbt domain containing 1 [Source:HGNC Symbol;Acc:HGNC:19866]	chr17:49254785-49337427	NM_017643	XM_011524933, XM_0	NP_060113	XP_005257524, XP_0052	protein_coding	ENSG0000001125	54799	19866
1845	MYO1D	0.688	0.002	myosin ID [Source:HGNC Symbol;Acc:HGNC:7598]	chr17:30819539-31204195	NM_001303279, NM	.	NP_001290208, NP_00	XP_016880174	protein_coding	ENSG0000017665	4642	7598
1846	SELM	-1.047	0.002	selenoprotein M [Source:EntrezGene;Acc:140606]	chr22:31500757-31516055	NM_080430	.	NP_536355	.	protein_coding	ENSG0000019883	140606	.
1847	UNC13D	-0.751	0.002	unc-13 homolog D [Source:HGNC Symbol;Acc:HGNC:23147]	chr17:73823305-73840798	NM_199242	XM_011524507, XM_0	NP_954712	XP_011522806, XP_0115	protein_coding	ENSG0000009292	201294	23147
1848	ZNF75D	1.097	0.002	zinc finger protein 75D [Source:HGNC Symbol;Acc:HGNC:13145]	chrX:134419719-134478012	NM_001185063, NM	XM_011531396, XM_0	NP_001171992, NP_00	XP_005262526, XP_0115	protein_coding	ENSG0000018637	7626	13145
1849	CEP170	1.585	0.002	centrosomal protein 170 [Source:HGNC Symbol;Acc:HGNC:28920]	chr1:243287729-243418623	NM_001042404, NM	XM_017002936, XM_0	NP_001035863, NP_00	XP_006711906, XP_0115	protein_coding	ENSG0000014370	645455	28920
1850	DIS3	1.637	0.002	DIS3 homolog, exosome endoribonuclease and 3'-5' exoribonuclease [Source:HGNC Sym	chr13:73301886-73590599	NM_001128226, NM	XM_006719778, XM_0	NP_001121698, NP_00	XP_005266351	protein_coding	ENSG0000008352	22894	20604
1851	KIAA0232	1.083	0.002	KIAA0232 [Source:HGNC Symbol;Acc:HGNC:28992]	chr4:6784458-6885897	NM_001100590, NM	XM_011513597	NP_001094060, NP_05	XP_005248098, XP_0115	protein_coding	ENSG0000017087	9778	28992
1852	KIF16B	1.018	0.002	kinesin family member 16B [Source:HGNC Symbol;Acc:HGNC:15869]	chr20:16252748-16554079	NM_001199865, NM	XM_011529288, XM_0	NP_001186794, NP_00	XP_005260807, XP_0052	protein_coding	ENSG0000008917	55614	15869
1853	METRN	-1.233	0.002	meteorin, glial cell differentiation regulator [Source:HGNC Symbol;Acc:HGNC:14151]	chr16:765114-769655	NM_024042	.	NP_076947	.	protein_coding	ENSG0000010326	79006	14151
1854	RELB	-0.821	0.002	RELB proto-oncogene, NF-kB subunit [Source:HGNC Symbol;Acc:HGNC:9956]	chr19:45504687-45541456	NM_006509	XM_005259128, XM_0	NP_006500	XP_005259184, XP_0052	protein_coding	ENSG0000010485	5971	9956
1855	SLC26A2	1.049	0.002	solute carrier family 26 member 2 [Source:HGNC Symbol;Acc:HGNC:10994]	chr5:149340299-149366968	NM_000112	.	NP_000103	XP_016864680	protein_coding	ENSG0000015585	1836	10994
1856	TMEM30A	0.899	0.002	transmembrane protein 30A [Source:HGNC Symbol;Acc:HGNC:16667]	chr6:75962639-75994684	NM_001143958, NM	.	NP_001137430, NP_06	.	protein_coding	ENSG0000011269	55754	16667
1857	ANK2	1.376	0.002	ankyrin 2, neuronal [Source:HGNC Symbol;Acc:HGNC:493]	chr4:113739264-114304896	NM_001127493, NM	XM_017008096, XM_0	NP_001120965, NP_00	XP_005263002, XP_0168	protein_coding	ENSG0000014536	287	493
1858	ARHGEF26	1.350	0.002	Rho guanine nucleotide exchange factor 26 [Source:HGNC Symbol;Acc:HGNC:24490]	chr3:153838791-153975616	NM_001251962, NM	XM_011512672	NP_001238891, NP_00	XP_011510974	protein_coding	ENSG0000011479	26084	24490
1859	GEM	1.447	0.002	GTP binding protein overexpressed in skeletal muscle [Source:HGNC Symbol;Acc:HGNC:	chr8:95261480-95274578	NM_005261, NM_1	.	NP_005252, NP_85905	.	protein_coding	ENSG0000016494	2669	4234
1860	GPX8	1.063	0.002	glutathione peroxidase 8 (putative) [Source:HGNC Symbol;Acc:HGNC:33100]	chr5:54408822-54469003	NM_001008397, NM	XM_011543416, XM_0	NP_001008398, NP_00	XP_006714694, XP_0115	protein_coding	ENSG0000016429	493869	33100
1861	INHBE	-1.131	0.002	inhibin beta E subunit [Source:HGNC Symbol;Acc:HGNC:24029]	chr12:57849103-57851791	NM_031479	.	NP_113667	.	protein_coding	ENSG0000013926	83729	24029
1862	MYO5A	0.943	0.002	myosin VA [Source:HGNC Symbol;Acc:HGNC:7602]	chr15:52599479-52821247	NM_000259, NM_0	XM_011521612, XM_0	NP_000250, NP_00113	XP_005254454, XP_0115	protein_coding	ENSG0000019753	4644	7602
1863	NINJ1	-0.760	0.002	ninjurin 1 [Source:HGNC Symbol;Acc:HGNC:7824]	chr9:95883770-95896570	NM_004148	.	NP_004139	.	protein_coding	ENSG0000013166	4814	7824
1864	SACS	0.997	0.002	sacsin molecular chaperone [Source:HGNC Symbol;Acc:HGNC:10519]	chr13:23902964-23985652	NM_001278055, NM	XM_017020539, XM_0	NP_001264984, NP_05	XP_005266395, XP_0115	protein_coding	ENSG0000015183	26278	10519
1865	UBE3A	0.929	0.002	ubiquitin protein ligase E3A [Source:HGNC Symbol;Acc:HGNC:12496]	chr15:25578874-25684203	NM_000462, NM_1	XM_017022552, XM_0	NP_000453, NP_57085	XP_005268324, XP_0052	protein_coding	ENSG0000011406	7337	12496
1866	UBE4A	0.854	0.002	ubiquitination factor E4A [Source:HGNC Symbol;Acc:HGNC:12499]	chr11:118230299-118269926	NM_001204077, NM	.	NP_001191006, NP_00	.	protein_coding	ENSG0000011034	9354	12499
1867	ZNF485	1.452	0.002	zinc finger protein 485 [Source:HGNC Symbol;Acc:HGNC:23440]	chr10:44101854-44113351	NM_001318140, NM	XM_006717706	NP_001305069, NP_00	XP_006717769	protein_coding	ENSG0000019829	220992	23440
1868	B3GNT3	-0.912	0.002	UDP-GlcNAc:betaGal beta-1,3-N-acetylglucosaminyltransferase 3 [Source:HGNC Symbol	chr19:17905918-17923891	NM_014256	XM_011527627, XM_0	NP_055071	XP_011525928, XP_0115	protein_coding	ENSG0000017991	10331	13528
1869	DDX42	0.751	0.002	DEAD-box helicase 42 [Source:HGNC Symbol;Acc:HGNC:18676]	chr17:61851222-61896677	NM_007372, NM_2	XM_017024111, XM_0	NP_031398, NP_98709	XP_006721720, XP_0168	protein_coding	ENSG0000019823	11325	18676
1870	ETV4	-0.723	0.002	ETS variant 4 [Source:HGNC Symbol;Acc:HGNC:3493]	chr17:41605211-41623800	NM_001079675, NM	XM_011524514, XM_0	NP_001073143, NP_00	XP_011522816, XP_0168	protein_coding	ENSG0000017583	2118	3493
1871	HAPLN3	-1.306	0.002	hyaluronan and proteoglycan link protein 3 [Source:HGNC Symbol;Acc:HGNC:21446]	chr15:89420518-89438857	NM_001307952, NM	XM_011521261, XM_0	NP_001294881, NP_83	XP_011519562, XP_0115	protein_coding	ENSG0000014051	145864	21446
1872	HMGA1	-0.722	0.002	high mobility group AT-hook 1 [Source:HGNC Symbol;Acc:HGNC:5010]	chr6:34204649-34214008	NM_001319077, NM	XM_011514565, XM_0	NP_001306006, NP_00	XP_005249118, XP_0115	protein_coding	ENSG0000013730	3159	5010
1873	LTBP3	-0.776	0.002	latent transforming growth factor beta binding protein 3 [Source:HGNC Symbol;Acc:HG	chr11:65306275-65325699	NM_001130144, NM	XM_011545033, XM_0	NP_001123616, NP_00	XP_011543334, XP_0115	protein_coding	ENSG0000016805	4054	6716
1874	MIB1	0.901	0.002	mindbomb E3 ubiquitin protein ligase 1 [Source:HGNC Symbol;Acc:HGNC:21086]	chr18:19321280-19450918	NM_020774	.	NP_065825	.	protein_coding	ENSG0000010175	57534	21086
1875	PLA2G4D	-1.166	0.002	phospholipase A2 group IVD [Source:HGNC Symbol;Acc:HGNC:30038]	chr15:42359880-42386752	NM_178034	XM_011521467	NP_828848	XP_011519769	protein_coding	ENSG0000015933	283748	30038
1876	USP33	0.953	0.002	ubiquitin specific peptidase 33 [Source:HGNC Symbol;Acc:HGNC:20059]	chr1:78161671-78225537	NM_015017, NM_2	XM_011541055, XM_0	NP_055832, NP_96391	XP_005270705, XP_0052	protein_coding	ENSG0000007725	23032	20059
1877	WFDC2	-0.787	0.002	WAP four-disulfide core domain 2 [Source:HGNC Symbol;Acc:HGNC:15939]	chr20:44095909-44110172	NM_006103	XM_011528495	NP_006094	XP_011526797	protein_coding	ENSG0000010144	10406	15939
1878	CBX1	0.775	0.002	chromobox 1 [Source:HGNC Symbol;Acc:HGNC:1551]	chr17:46147413-46178883	NM_001127228, NM	.	NP_001120700, NP_00	.	protein_coding	ENSG0000010846	10951	1551
1879	CNOT7	1.205	0.002	CCR4-NOT transcription complex subunit 7 [Source:HGNC Symbol;Acc:HGNC:14101]	chr8:17082472-17155533	NM_013354, NM_0	XM_011544511, XM_0	NP_001309016, NP_00	XP_005273538, XP_0115	protein_coding	ENSG0000019879	29883	14101
1880	DDX59	1.051	0.002	DEAD-box helicase 59 [Source:HGNC Symbol;Acc:HGNC:25360]	chr1:200593023-200639097	NM_001031725	XM_011510035, XM_0	NP_001026895, NP_00	XP_005245576, XP_0052	protein_coding	ENSG0000011819	83479	25360
1881	PJA2	1.000	0.002	praja ring finger ubiquitin ligase 2 [Source:HGNC Symbol;Acc:HGNC:17481]	chr5:108670409-108745695	NM_014819	XM_017010099	NP_055634	XP_016865588	protein_coding	ENSG0000019896	9867	17481
1882	SCARA3	0.875	0.002	scavenger receptor class A member 3 [Source:HGNC Symbol;Acc:HGNC:19000]	chr8:27491384-27534293	NM_016240, NM_1	XM_017013535	NP_057324, NP_87818	XP_016869024, XP_0168	protein_coding	ENSG0000016807	51435	19000
1883	SEC24B	1.029	0.002	SEC24 homolog B, COPII coat complex component [Source:HGNC Symbol;Acc:HGNC:107	chr4:110354927-110462052	NM_001042734, NM	XM_011531539, XM_0	NP_001036199, NP_00	XP_005262745, XP_0052	protein_coding	ENSG0000013880	10427	10704
1884	SYNE4	-1.149	0.002	spectrin repeat containing nuclear envelope family member 4 [Source:HGNC Symbol;Ac	chr19:36494211-36499695	NM_001039876, NM	XM_005258598	NP_001034965, NP_00	.	protein_coding	ENSG0000018139	163183	26703
1885	ZNF280B	1.357	0.002	zinc finger protein 280B [Source:HGNC Symbol;Acc:HGNC:23022]	chr22:22838766-22863472	NM_080764	XM_011529897, XM_0	NP_542942	XP_011528195, XP_0115	protein_coding	ENSG0000027500	140883	23022
1886	AHCYL2	0.922	0.002	adenosylhomocysteinase like 2 [Source:HGNC Symbol;Acc:HGNC:22204]	chr7:128864863-129070052	NM_001130720, NM	XM_017011907, XM_0	NP_001124192, NP_00	XP_005250294, XP_0115	protein_coding	ENSG0000015846	23382	22204
1887	AHNAK2	-1.106	0.002	AHNAK nucleoprotein 2 [Source:HGNC Symbol;Acc:HGNC:20125]	chr14:105391152-105444694	NM_138420	XM_005267299	NP_612429	XP_005267356	protein_coding	ENSG0000018556	113146	20125
1888	C1GALT1	1.330	0.002	core 1 synthase, glycoprotein-N-acetylgalactosamine 3-beta-galactosyltransferase 1 [So	chr7:7222245-7288282	NM_020156	XM_017012448, XM_0	NP_064541	XP_005249869, XP_0115	protein_coding	ENSG0000010639	56913	24337

1889	CLIC3	-1.211	0.002	chloride intracellular channel 3 [Source:HGNC Symbol;Acc:HGNC:2064]	chr9:139889086-139891255	NM_004669	.	NP_004660	XP_016870770	protein_coding	ENSG0000016958	9022	2064
1890	CNFN	-1.108	0.002	cornifelin [Source:HGNC Symbol;Acc:HGNC:30183]	chr19:42891170-42894439	NM_032488	XM_011527396, XM_0	NP_115877	XP_005259389, XP_0115	protein_coding	ENSG0000010542	84518	30183
1891	ERCC6L2	1.128	0.002	ERCC excision repair 6 like 2 [Source:HGNC Symbol;Acc:HGNC:26922]	chr9:98637982-98731122	NM_001010895	XM_011518651	NP_001010895	.	protein_coding	ENSG0000018215	375748	26922
1892	ETV7	-1.081	0.002	ETS variant 7 [Source:HGNC Symbol;Acc:HGNC:18160]	chr6:36321867-36355577	NM_001207035, NM	XM_011514659	NP_001193964, NP_00	XP_011512961	protein_coding	ENSG0000001003	51513	18160
1893	GCC2	1.003	0.002	GRIP and coiled-coil domain containing 2 [Source:HGNC Symbol;Acc:HGNC:23218]	chr2:109065016-109125854	NM_181453	XM_006712871, XM_0	NP_852118	XP_006712933, XP_0067	protein_coding	ENSG0000013596	9648	23218
1894	GPATCH4	0.873	0.002	G-patch domain containing 4 [Source:HGNC Symbol;Acc:HGNC:25982]	chr1:156564278-156571275	NM_015590, NM_1	XM_005245287	NP_056405, NP_87262	XP_005245344	protein_coding	ENSG0000016081	54865	25982
1895	HELZ	1.085	0.002	helicase with zinc finger [Source:HGNC Symbol;Acc:HGNC:16878]	chr17:65066553-65241299	NM_014877	XM_017025477, XM_0	NP_055692	XP_005257945, XP_0052	protein_coding	ENSG0000019826	9931	16878
1896	HOXD13	1.141	0.002	homeobox D13 [Source:HGNC Symbol;Acc:HGNC:5136]	chr2:176957618-176960666	NM_000523	.	NP_000514	.	protein_coding	ENSG0000012871	3239	5136
1897	ISG15	-0.738	0.002	ISG15 ubiquitin-like modifier [Source:HGNC Symbol;Acc:HGNC:4053]	chr1:936517-949921	NM_005101	.	NP_005092	.	protein_coding	ENSG0000018760	9636	4053
1898	ITPA	-0.784	0.002	inosine triphosphatase [Source:HGNC Symbol;Acc:HGNC:6176]	chr20:3190005-3204516	NM_001267623, NM	XM_006723564	NP_001254552, NP_25	XP_006723627	protein_coding	ENSG0000012587	3704	6176
1899	NSF	0.878	0.002	N-ethylmaleimide sensitive factor, vesicle fusing ATPase [Source:HGNC Symbol;Acc:HGN	chr17:44668034-44834830	NM_006178	XM_011524863	NP_006169	.	protein_coding	ENSG0000007396	4905	8016
1900	PBX3	1.075	0.002	PBX homeobox 3 [Source:HGNC Symbol;Acc:HGNC:8634]	chr9:128509623-128729656	NM_001134778, NM	XM_011518755, XM_0	NP_001128250, NP_00	XP_006717193, XP_0067	protein_coding	ENSG0000016708	5090	8634
1901	PRKDC	0.714	0.002	protein kinase, DNA-activated, catalytic polypeptide [Source:HGNC Symbol;Acc:HGNC:9	chr8:48685668-48872743	NM_001081640, NM	XM_011517568, XM_0	NP_001075109, NP_00	.	protein_coding	ENSG0000025372	5591	9413
1902	SULT1A2	-1.232	0.002	sulfotransferase family 1A member 2 [Source:HGNC Symbol;Acc:HGNC:11454]	chr16:28603263-28608430	NM_001054, NM_1	XM_017023600, XM_0	NP_001045, NP_80356	XP_006721139, XP_0067	protein_coding	ENSG0000019716	6799	11454
1903	TH	-1.160	0.002	tyrosine hydroxylase [Source:HGNC Symbol;Acc:HGNC:11782]	chr11:2185158-2193107	NM_000360, NM_1	XM_011520335	NP_000351, NP_95498	XP_011518637	protein_coding	ENSG0000018017	7054	11782
1904	BMS1	0.808	0.003	BMS1, ribosome biogenesis factor [Source:HGNC Symbol;Acc:HGNC:23505]	chr10:43278248-43330385	NM_014753	XM_011540402, XM_0	NP_055568	XP_005271903, XP_0052	protein_coding	ENSG0000016573	9790	23505
1905	DOCK3	-1.133	0.003	dedicator of cytokinesis 3 [Source:HGNC Symbol;Acc:HGNC:2989]	chr3:50712671-51421629	NM_004947	XM_017005825, XM_0	NP_004938	XP_005264971, XP_0052	protein_coding	ENSG0000008853	1795	2989
1906	ENPP5	1.371	0.003	ectonucleotide pyrophosphatase/phosphodiesterase 5 (putative) [Source:HGNC Symbol	chr6:46126923-46138708	NM_001290072, NM	XM_011514786, XM_0	NP_001277001, NP_00	XP_005249317, XP_0115	protein_coding	ENSG0000011279	59084	13717
1907	FAF2	0.746	0.003	Fas associated factor family member 2 [Source:HGNC Symbol;Acc:HGNC:24666]	chr5:175875355-175937075	NM_014613	XM_011534475	NP_055428	XP_011532777	protein_coding	ENSG0000011319	23197	24666
1908	GDI2	0.726	0.003	GDP dissociation inhibitor 2 [Source:HGNC Symbol;Acc:HGNC:4227]	chr10:5807185-5855512	NM_001115156, NM	.	NP_001108628, NP_00	XP_016871560	protein_coding	ENSG0000005760	2665	4227
1909	GSTO2	-1.074	0.003	glutathione S-transferase omega 2 [Source:HGNC Symbol;Acc:HGNC:23064]	chr10:106028630-106064703	NM_001191013, NM	XM_011539270	NP_001177942, NP_00	XP_011537572	protein_coding	ENSG0000006562	119391	23064
1910	OAZ2	-0.774	0.003	ornithine decarboxylase antizyme 2 [Source:HGNC Symbol;Acc:HGNC:8096]	chr15:64979771-64995480	NM_001301302, NM	.	NP_001288231, NP_00	.	protein_coding	ENSG0000018030	4947	8096
1911	RABEP1	0.868	0.003	rabaptin, RAB GTPase binding effector protein 1 [Source:HGNC Symbol;Acc:HGNC:1767	chr17:5185594-5289132	NM_001083585, NM	XM_011524061, XM_0	NP_001077054, NP_00	XP_011522362	protein_coding	ENSG0000002972	9135	17677
1912	ZNF260	1.082	0.003	zinc finger protein 260 [Source:HGNC Symbol;Acc:HGNC:13499]	chr19:37001596-37019562	NM_001012756, NM	XM_011526897	NP_001012774, NP_00	XP_011525199, XP_0168	protein_coding	ENSG0000025400	339324	13499
1913	ACTR1B	-0.737	0.003	ARP1 actin-related protein 1 homolog B, centractin beta [Source:HGNC Symbol;Acc:HGN	chr2:98272425-98280570	NM_005735	XM_005263854	NP_005726	XP_005263911	protein_coding	ENSG0000011507	10120	168
1914	CBX6	-0.706	0.003	chromobox 6 [Source:HGNC Symbol;Acc:HGNC:1556]	chr22:39257454-39268319	NM_001303494, NM	XM_011530024	NP_001290423, NP_05	.	protein_coding	ENSG0000018374	23466	1556
1915	CLIC4	0.774	0.003	chloride intracellular channel 4 [Source:HGNC Symbol;Acc:HGNC:13518]	chr1:25071847-25170815	NM_013943	.	NP_039234	.	protein_coding	ENSG0000016950	25932	13518
1916	DDHD1	1.189	0.003	DDHD domain containing 1 [Source:HGNC Symbol;Acc:HGNC:19714]	chr14:53503462-53620000	NM_001160147, NM	XM_011537189, XM_0	NP_001153619, NP_00	XP_005268159, XP_0052	protein_coding	ENSG0000010052	80821	19714
1917	ERMP1	0.850	0.003	endoplasmic reticulum metallopeptidase 1 [Source:HGNC Symbol;Acc:HGNC:23703]	chr9:5784572-5833117	NM_024896	XM_017015139, XM_0	NP_079172	XP_005251644, XP_0168	protein_coding	ENSG0000009921	79956	23703
1918	NDC1	0.899	0.003	NDC1 transmembrane nucleoporin [Source:HGNC Symbol;Acc:HGNC:25525]	chr1:54231132-54304533	NM_001168551, NM	XM_011541766, XM_0	NP_001162023, NP_06	XP_011540068	protein_coding	ENSG0000005880	55706	25525
1919	PRKACB	1.079	0.003	protein kinase cAMP-activated catalytic subunit beta [Source:HGNC Symbol;Acc:HGNC:9	chr1:84543744-84704181	NM_001242857, NM	XM_017001713, XM_0	NP_001229786, NP_00	XP_005271072, XP_0052	protein_coding	ENSG0000014287	5567	9381
1920	RAET1L	-1.420	0.003	retinoic acid early transcript 1L [Source:HGNC Symbol;Acc:HGNC:16798]	chr6:150339469-150346668	NM_130900	XM_011535486	NP_570970	XP_011533788	protein_coding	ENSG0000015591	154064	16798
1921	TATDN2	-0.844	0.003	TatD DNase domain containing 2 [Source:HGNC Symbol;Acc:HGNC:28988]	chr3:10289706-10322902	NM_014760	.	NP_055575	.	protein_coding	ENSG0000015701	9797	28988
1922	WDFY3	1.010	0.003	WD repeat and FYVE domain containing 3 [Source:HGNC Symbol;Acc:HGNC:20751]	chr4:85590762-85887544	NM_014991	XM_017007908, XM_0	NP_055806	XP_005262915, XP_0115	protein_coding	ENSG0000016362	23001	20751
1923	CLU	0.708	0.003	clusterin [Source:HGNC Symbol;Acc:HGNC:2095]	chr8:27454433-27472548	NM_001831	XM_006716284	NP_001822	XP_006716347	protein_coding	ENSG0000012088	102465508	2095
1924	DEK	0.760	0.003	DEK proto-oncogene [Source:HGNC Symbol;Acc:HGNC:2768]	chr6:18224098-18265054	NM_001134709, NM	.	NP_001128181, NP_00	XP_016866762	protein_coding	ENSG0000012479	7913	2768
1925	NUP54	0.868	0.003	nucleoporin 54 [Source:HGNC Symbol;Acc:HGNC:17359]	chr4:77035816-77069668	NM_001278603, NM	XM_011532033, XM_0	NP_001265532, NP_05	XP_006714300, XP_0115	protein_coding	ENSG0000013875	53371	17359
1926	ODF3B	-1.401	0.003	outer dense fiber of sperm tails 3B [Source:HGNC Symbol;Acc:HGNC:34388]	chr22:50968837-50971008	NM_001014440	XM_005261918	NP_001014440	XP_005261975	protein_coding	ENSG0000017798	440836	34388
1927	PTPN14	0.891	0.003	protein tyrosine phosphatase, non-receptor type 14 [Source:HGNC Symbol;Acc:HGNC:9	chr1:214522038-214724566	NM_005401	.	NP_005392	XP_016857430	protein_coding	ENSG0000015210	5784	9647
1928	RGS16	-1.057	0.003	regulator of G-protein signaling 16 [Source:HGNC Symbol;Acc:HGNC:9997]	chr1:182567757-182573543	NM_002928	.	NP_002919	.	protein_coding	ENSG0000014333	6004	9997
1929	RNGTT	0.914	0.003	RNA guanylyltransferase and 5'-phosphatase [Source:HGNC Symbol;Acc:HGNC:10073]	chr6:89319990-89673440	NM_001286426, NM	XM_017011401	NP_001273355, NP_00	XP_016866890	protein_coding	ENSG0000011188	8732	10073
1930	RNPEPL1	-0.790	0.003	arginyl aminopeptidase like 1 [Source:HGNC Symbol;Acc:HGNC:10079]	chr2:241508103-241520789	NM_018226	XM_005247036	NP_060696	XP_005247093	protein_coding	ENSG0000014232	.	10079
1931	TLR4	1.133	0.003	toll like receptor 4 [Source:HGNC Symbol;Acc:HGNC:11850]	chr9:120466609-120479149	NM_003266, NM_1	.	NP_003257, NP_61256	.	protein_coding	ENSG0000013686	7099	11850
1932	WDR11	0.918	0.003	WD repeat domain 11 [Source:HGNC Symbol;Acc:HGNC:13831]	chr10:122610686-122669036	NM_018117	XM_005269963, XM_0	NP_060587	XP_005270019, XP_0052	protein_coding	ENSG0000012000	55717	13831
1933	WDR54	-0.833	0.003	WD repeat domain 54 [Source:HGNC Symbol;Acc:HGNC:25770]	chr2:74648855-74652882	NM_001320823, NM	XM_006712111, XM_0	NP_001307752, NP_00	XP_005264643	protein_coding	ENSG0000000544	84058	25770
1934	WNK1	0.768	0.003	WNK lysine deficient protein kinase 1 [Source:HGNC Symbol;Acc:HGNC:14540]	chr12:861758-1020618	NM_001184985, NM	XM_017019838, XM_0	NP_001171914, NP_05	XP_006719066, XP_0115	protein_coding	ENSG0000006023	65125	14540
1935	ANAPC4	1.128	0.003	anaphase promoting complex subunit 4 [Source:HGNC Symbol;Acc:HGNC:19990]	chr4:25378834-25420120	NM_001286756, NM	XM_017008138, XM_0	NP_001273685, NP_03	XP_011512140, XP_0115	protein_coding	ENSG0000005390	29945	19990
1936	DDIT4	-0.746	0.003	DNA damage inducible transcript 4 [Source:HGNC Symbol;Acc:HGNC:24944]	chr10:74033677-74035794	NM_019058	.	NP_061931	.	protein_coding	ENSG0000016820	54541	24944
1937	FAM156B	-1.354	0.003	family with sequence similarity 156 member B [Source:HGNC Symbol;Acc:HGNC:31962]	chrX:52926338-52937587	NM_001099684, NM	XM_011530821, XM_0	NP_001093154, NP_00	XP_005262081, XP_0052	protein_coding	ENSG0000017930	727866	31962
1938	IL13RA1	0.817	0.003	interleukin 13 receptor subunit alpha 1 [Source:HGNC Symbol;Acc:HGNC:5974]	chrX:117861534-117928502	NM_001560	XM_011531336	NP_001551	XP_011529638	protein_coding	ENSG0000013172	3597	5974
1939	IL21R	-1.458	0.003	interleukin 21 receptor [Source:HGNC Symbol;Acc:HGNC:6006]	chr16:27413482-27463363	NM_021798, NM_1	XM_011545858, XM_0	NP_068570, NP_85156	XP_011544159, XP_0115	protein_coding	ENSG0000010352	50615	6006
1940	LRCOL1	-1.658	0.003	leucine rich colipase like 1 [Source:HGNC Symbol;Acc:HGNC:44160]	chr12:133179754-133186960	NM_001195520	.	NP_001182449	.	protein_coding	ENSG0000020458	100507055	44160
1941	SMG7	0.770	0.003	SMG7, nonsense mediated mRNA decay factor [Source:HGNC Symbol;Acc:HGNC:16792]	chr1:183441612-183523328	NM_001174061, NM	XM_017002971, XM_0	NP_001167532, NP_77	XP_005245705, XP_0052	protein_coding	ENSG0000011669	9887	16792
1942	ACAA1	-0.786	0.003	acetyl-CoA acyltransferase 1 [Source:HGNC Symbol;Acc:HGNC:82]	chr3:38080695-38178733	NM_001130410, NM	XM_006713123, XM_0	NP_001123882, NP_00	XP_006713185, XP_0067	protein_coding	ENSG0000006097	30	82

1943	ARPC4	-1.250	0.003	actin related protein 2/3 complex subunit 4 [Source:HGNC Symbol;Acc:HGNC:707]	chr3:9821543-9896822	NM_001024959, NM	.	NP_001020130, NP_00	.	protein_coding	ENSG0000024155	10093	707
1944	FADS3	-0.807	0.003	fatty acid desaturase 3 [Source:HGNC Symbol;Acc:HGNC:3576]	chr11:61640990-61659523	NM_021727	XM_017017723, XM_0	NP_068373	XP_011543325, XP_0168	protein_coding	ENSG0000022196	3995	3576
1945	GRAMD1A	-0.943	0.003	GRAM domain containing 1A [Source:HGNC Symbol;Acc:HGNC:29305]	chr19:35485687-35517375	NM_001136199, NM	XM_017027035, XM_0	NP_001129671, NP_00	XP_011525451, XP_0115	protein_coding	ENSG0000008935	57655	29305
1946	GXYLT1	1.124	0.003	glucoside xylosyltransferase 1 [Source:HGNC Symbol;Acc:HGNC:27482]	chr12:42475646-42538681	NM_001099650, NM	XM_017019211	NP_001093120, NP_77	XP_016874700	protein_coding	ENSG0000015123	283464	27482
1947	KLF16	-0.922	0.003	Kruppel like factor 16 [Source:HGNC Symbol;Acc:HGNC:16857]	chr19:1852397-1863567	NM_031918	.	NP_114124	.	protein_coding	ENSG0000012991	83855	16857
1948	REL	1.189	0.003	REL proto-oncogene, NF-kB subunit [Source:HGNC Symbol;Acc:HGNC:9954]	chr2:61108655-61158745	NM_001291746, NM	.	NP_001278675, NP_00	XP_016860116	protein_coding	ENSG0000016292	5966	9954
1949	CCDC74B	-1.205	0.003	coiled-coil domain containing 74B [Source:HGNC Symbol;Acc:HGNC:25267]	chr2:130896859-130902707	NM_001258307, NM	XM_006712837, XM_0	NP_001245236, NP_99	XP_005263897, XP_0052	protein_coding	ENSG0000015207	91409	25267
1950	ECI1	-0.835	0.003	enoyl-CoA delta isomerase 1 [Source:HGNC Symbol;Acc:HGNC:2703]	chr16:2289395-2302301	NM_001178029, NM	.	NP_001171500, NP_00	.	protein_coding	ENSG0000016796	1632	2703
1951	ENAH	1.047	0.003	enabled homolog (Drosophila) [Source:HGNC Symbol;Acc:HGNC:18271]	chr1:225674536-225840844	NM_001008493, NM	XM_017001757, XM_0	NP_001008493, NP_06	XP_005273244, XP_0115	protein_coding	ENSG0000015438	55740	18271
1952	FKBP5	0.849	0.003	FK506 binding protein 5 [Source:HGNC Symbol;Acc:HGNC:3721]	chr6:35541361-35696360	NM_001145775, NM	.	NP_001139247, NP_00	.	protein_coding	ENSG0000009606	2289	3721
1953	GAN	1.101	0.003	gigaxonin [Source:HGNC Symbol;Acc:HGNC:4137]	chr16:81348556-81424489	NM_022041	.	NP_071324	.	protein_coding	ENSG0000026160	100616150	4137
1954	GOLGB1	0.960	0.003	golgin B1 [Source:HGNC Symbol;Acc:HGNC:4429]	chr3:121382045-121468614	NM_001256486, NM	XM_017006195, XM_0	NP_001243415, NP_00	XP_005247428, XP_0052	protein_coding	ENSG0000017323	2804	4429
1955	ITGA2	0.832	0.003	integrin subunit alpha 2 [Source:HGNC Symbol;Acc:HGNC:6137]	chr5:52285155-52390609	NM_002203	.	NP_002194	.	protein_coding	ENSG0000016417	3673	6137
1956	KCNJ5	1.048	0.003	potassium voltage-gated channel subfamily J member 5 [Source:HGNC Symbol;Acc:HGN	chr11:128761250-128790930	NM_000890	XM_011542810, XM_0	NP_000881	XP_011541111, XP_0115	protein_coding	ENSG0000012045	3762	6266
1957	LRRC45	-0.813	0.003	leucine rich repeat containing 45 [Source:HGNC Symbol;Acc:HGNC:28302]	chr17:79981177-79989027	NM_144999	.	NP_659436	.	protein_coding	ENSG0000016968	201255	28302
1958	NDUFS8	-0.749	0.003	NADH:ubiquinone oxidoreductase core subunit S8 [Source:HGNC Symbol;Acc:HGNC:771	chr11:67798093-67804111	NM_002496	XM_011545053, XM_0	NP_002487	XP_005274070, XP_0052	protein_coding	ENSG0000011071	102465669	7715
1959	TMEM263	1.182	0.003	transmembrane protein 263 [Source:HGNC Symbol;Acc:HGNC:28281]	chr12:107349496-107367814	NM_001319661, NM	XM_017020198, XM_0	NP_001306590, NP_00	XP_011537267, XP_0168	protein_coding	ENSG0000015113	90488	28281
1960	TNFAIP8L1	-0.801	0.003	TNF alpha induced protein 8 like 1 [Source:HGNC Symbol;Acc:HGNC:28279]	chr19:4639529-4655580	NM_001167942, NM	XM_011527680, XM_0	NP_001161414, NP_68	XP_005259544, XP_0115	protein_coding	ENSG0000018536	126282	28279
1961	ZBTB26	1.187	0.003	zinc finger and BTB domain containing 26 [Source:HGNC Symbol;Acc:HGNC:23383]	chr9:125677844-125693779	NM_001304363, NM	.	NP_001291292, NP_00	.	protein_coding	ENSG0000017144	57684	23383
1962	ZNF652	1.152	0.003	zinc finger protein 652 [Source:HGNC Symbol;Acc:HGNC:29147]	chr17:47371926-47439835	NM_001145365, NM	XM_005257166	NP_001138837, NP_05	XP_005257223	protein_coding	ENSG0000019874	22834	29147
1963	CBL	0.778	0.003	Cbl proto-oncogene [Source:HGNC Symbol;Acc:HGNC:1541]	chr11:119076985-119178859	NM_005188	XM_017018511	NP_005179	XP_016874000	protein_coding	ENSG0000011039	867	1541
1964	FMNL2	1.048	0.003	formin like 2 [Source:HGNC Symbol;Acc:HGNC:18267]	chr2:153191750-153506348	NM_052905	XM_011510535, XM_0	NP_443137	XP_005246320, XP_0052	protein_coding	ENSG0000015782	114793	18267
1965	GPR155	1.045	0.003	G protein-coupled receptor 155 [Source:HGNC Symbol;Acc:HGNC:22951]	chr2:175296298-175351822	NM_001033045, NM	XM_017003487, XM_0	NP_001028217, NP_00	XP_016858974, XP_0168	protein_coding	ENSG0000016332	151556	22951
1966	SENP6	1.024	0.003	SUMO1/sentrin specific peptidase 6 [Source:HGNC Symbol;Acc:HGNC:20944]	chr6:76311224-76427994	NM_001100409, NM	XM_011535747, XM_0	NP_001093879, NP_00	XP_006715520, XP_0115	protein_coding	ENSG0000011270	26054	20944
1967	SLC37A3	0.906	0.003	solute carrier family 37 member 3 [Source:HGNC Symbol;Acc:HGNC:20651]	chr7:140033551-140098349	NM_001287498, NM	XM_011516627, XM_0	NP_001274427, NP_11	XP_011514928, XP_0115	protein_coding	ENSG0000015780	84255	20651
1968	SPIRE1	0.854	0.003	spire type actin nucleation factor 1 [Source:HGNC Symbol;Acc:HGNC:30622]	chr18:12446510-12658133	NM_001128626, NM	XM_011525702, XM_0	NP_001122098, NP_00	XP_005258179, XP_0115	protein_coding	ENSG0000013427	56907	30622
1969	STARD8	-1.251	0.003	StAR related lipid transfer domain containing 8 [Source:HGNC Symbol;Acc:HGNC:19161	chrX:67867510-67945682	NM_001142503, NM	XM_011531070, XM_0	NP_001135975, NP_00	XP_005262371, XP_0052	protein_coding	ENSG0000013005	9754	19161
1970	THOP1	-0.794	0.003	thimet oligopeptidase 1 [Source:HGNC Symbol;Acc:HGNC:11793]	chr19:2785457-2815805	NM_003249	.	NP_003240	.	protein_coding	ENSG0000017200	7064	11793
1971	TNFRSF10D	0.816	0.003	TNF receptor superfamily member 10d [Source:HGNC Symbol;Acc:HGNC:11907]	chr8:22993100-23021543	NM_003840	.	NP_003831	.	protein_coding	ENSG0000017353	8793	11907
1972	TRAPPC11	1.021	0.003	trafficking protein particle complex 11 [Source:HGNC Symbol;Acc:HGNC:25751]	chr4:184580419-184634747	NM_021942, NM_1	XM_011532180	NP_068761, NP_95100	XP_016864026, XP_0168	protein_coding	ENSG0000016853	60684	25751
1973	TRIM33	1.036	0.003	tripartite motif containing 33 [Source:HGNC Symbol;Acc:HGNC:16290]	chr1:114935398-115053781	NM_015906, NM_0	XM_011541568, XM_0	NP_056990, NP_14898	XP_005270993, XP_0052	protein_coding	ENSG0000019732	51592	16290
1974	TRPV4	-0.977	0.003	transient receptor potential cation channel subfamily V member 4 [Source:HGNC Symbo	chr12:110220889-110271206	NM_001177428, NM	XM_011538633, XM_0	NP_001170899, NP_00	XP_005253975, XP_0115	protein_coding	ENSG0000011119	59341	18083
1975	VCPIP1	1.036	0.003	valosin containing protein interacting protein 1 [Source:HGNC Symbol;Acc:HGNC:30897	chr8:67540721-67579452	NM_025054	.	NP_079330	.	protein_coding	ENSG0000017507	80124	30897
1976	ZFP1	1.299	0.003	ZFP1 zinc finger protein [Source:HGNC Symbol;Acc:HGNC:23328]	chr16:75182389-75206134	NM_001318469, NM	XM_017022987, XM_0	NP_001305398, NP_00	XP_005255874, XP_0115	protein_coding	ENSG0000018451	162239	23328
1977	CCDC6	0.916	0.003	coiled-coil domain containing 6 [Source:HGNC Symbol;Acc:HGNC:18782]	chr10:61548520-61666414	NM_005436	.	NP_005427	.	protein_coding	ENSG0000010809	8030	18782
1978	INF2	-0.817	0.003	inverted formin, FH2 and WH2 domain containing [Source:HGNC Symbol;Acc:HGNC:237	chr14:105155942-105188872	NM_001031714, NM	XM_005268005, XM_0	NP_001026884, NP_07	XP_005268061, XP_0052	protein_coding	ENSG0000020348	64423	23791
1979	KIF18A	1.456	0.003	kinesin family member 18A [Source:HGNC Symbol;Acc:HGNC:29441]	chr11:28042166-28355054	NM_031217	.	NP_112494	XP_016873868	protein_coding	ENSG0000012162	81930	29441
1980	SYT1	0.981	0.003	synaptotagmin 1 [Source:HGNC Symbol;Acc:HGNC:11509]	chr12:79257772-79845788	NM_001135805, NM	XM_017019909, XM_0	NP_001129277, NP_00	XP_005269170, XP_0067	protein_coding	ENSG0000006771	6857	11509
1981	TRIM16	-0.738	0.003	tripartite motif containing 16 [Source:HGNC Symbol;Acc:HGNC:17241]	chr17:15474804-15587614	NM_006470	.	NP_006461	.	protein_coding	ENSG0000022192	10626	17241
1982	TRMT6	0.901	0.003	tRNA methyltransferase 6 [Source:HGNC Symbol;Acc:HGNC:20900]	chr20:5918478-5931204	NM_001281467, NM	.	NP_001268396, NP_05	XP_016883364	protein_coding	ENSG0000008919	51605	20900
1983	ZFPM2	1.307	0.003	zinc finger protein, FOG family member 2 [Source:HGNC Symbol;Acc:HGNC:16700]	chr8:106330919-106816760	NM_012082	XM_011516948	NP_036214	XP_011515249, XP_0115	protein_coding	ENSG0000016994	23414	16700
1984	ATP12A	-1.282	0.003	ATPase H+/K+ transporting non-gastric alpha2 subunit [Source:HGNC Symbol;Acc:HGNC	chr13:25254548-25286631	NM_001185085, NM	.	NP_001172014, NP_00	.	protein_coding	ENSG0000007567	479	13816
1985	AVPR2	-2.209	0.003	arginine vasopressin receptor 2 [Source:HGNC Symbol;Acc:HGNC:897]	chrX:153170188-153172620	NM_000054, NM_0	XM_006724828	NP_000045, NP_00113	XP_006724891	protein_coding	ENSG0000012689	554	897
1986	BUB1B	0.928	0.003	BUB1 mitotic checkpoint serine/threonine kinase B [Source:HGNC Symbol;Acc:HGNC:11	chr15:40453226-40569688	NM_001211	.	NP_001202	.	protein_coding	ENSG0000015697	701	1149
1987	C6orf15	-1.408	0.003	chromosome 6 open reading frame 15 [Source:HGNC Symbol;Acc:HGNC:13927]	chr6:31078999-31080336	NM_014070	.	NP_054789	.	protein_coding	ENSG0000020454	29113	13927
1988	CANX	0.674	0.003	calnexin [Source:HGNC Symbol;Acc:HGNC:1473]	chr5:179125417-179157926	NM_001024649, NM	XM_011534665, XM_0	NP_001019820, NP_00	XP_011532966, XP_0115	protein_coding	ENSG0000012702	821	1473
1989	CNN3	1.525	0.003	calponin 3 [Source:HGNC Symbol;Acc:HGNC:2157]	chr1:95362504-95392834	NM_001286055, NM	.	NP_001272984, NP_00	XP_016855734	protein_coding	ENSG0000011751	1266	2157
1990	CSRNP1	-0.911	0.003	cysteine and serine rich nuclear protein 1 [Source:HGNC Symbol;Acc:HGNC:14300]	chr3:39183345-39196053	NM_001320559, NM	XM_011534019, XM_0	NP_001307488, NP_00	XP_005265460, XP_0115	protein_coding	ENSG0000014465	64651	14300
1991	EIF2AK2	1.049	0.003	eukaryotic translation initiation factor 2 alpha kinase 2 [Source:HGNC Symbol;Acc:HGNC	chr2:37311593-37384190	NM_001135651, NM	XM_011532987	NP_001129123, NP_00	XP_011531289	protein_coding	ENSG0000005533	5610	9437
1992	GNA13	0.987	0.003	G protein subunit alpha 13 [Source:HGNC Symbol;Acc:HGNC:4381]	chr17:63006832-63052957	NM_001282425, NM	.	NP_001269354, NP_00	.	protein_coding	ENSG0000012006	10672	4381
1993	HMBOX1	1.207	0.003	homeobox containing 1 [Source:HGNC Symbol;Acc:HGNC:26137]	chr8:28747910-28910787	NM_001135726, NM	XM_006716396, XM_0	NP_001129198, NP_00	XP_005273692, XP_0052	protein_coding	ENSG0000014742	79618	26137
1994	KMT2A	0.795	0.003	lysine methyltransferase 2A [Source:HGNC Symbol;Acc:HGNC:7132]	chr11:118307204-118397547	NM_001197104, NM	XM_011542833, XM_0	NP_001184033, NP_00	XP_006718902, XP_0115	protein_coding	ENSG0000011805	4297	7132
1995	LTV1	0.891	0.003	LTV1 ribosome biogenesis factor [Source:HGNC Symbol;Acc:HGNC:21173]	chr6:144164480-144184949	NM_032860	.	NP_116249	.	protein_coding	ENSG0000013552	84946	21173
1996	MECR	-0.860	0.003	mitochondrial trans-2-enoyl-CoA reductase [Source:HGNC Symbol;Acc:HGNC:19691]	chr1:29519384-29557444	NM_001024732, NM	XM_011541554, XM_0	NP_001019903, NP_05	XP_005245942, XP_0052	protein_coding	ENSG0000011635	51102	19691

1997	TBC1D4	0.877	0.003	TBC1 domain family member 4 [Source:HGNC Symbol;Acc:HGNC:19165]	chr13:75858800-76056250	NM_001286658, NN	XM_011535331, XM_0	NP_001273587, NP_00	XP_005266660, XP_0052	protein_coding	ENSG0000013611	9882	19165
1998	TXNDC16	1.287	0.003	thioredoxin domain containing 16 [Source:HGNC Symbol;Acc:HGNC:19965]	chr14:52897307-53019224	NM_001160047, NN	.	NP_001153519, NP_06	.	protein_coding	ENSG0000008730	57544	19965
1999	B3GNT7	-1.391	0.003	UDP-GlcNAc:betaGal beta-1,3-N-acetylglucosaminyltransferase 7 [Source:HGNC Symbol	chr2:232260253-232265875	NM_145236	XM_005246953	NP_660279	XP_005247010	protein_coding	ENSG0000015696	93010	18811
2000	CROT	1.394	0.003	carnitine O-octanoyltransferase [Source:HGNC Symbol;Acc:HGNC:2366]	chr7:86974996-87029111	NM_001143935, NN	XM_011516337	NP_001137407, NP_00	XP_011514639	protein_coding	ENSG0000000546	54677	2366
2001	DSG2	0.883	0.003	desmoglein 2 [Source:HGNC Symbol;Acc:HGNC:3049]	chr18:29078005-29128971	NM_001943	.	NP_001934	.	protein_coding	ENSG0000004660	1829	3049
2002	FAM135A	1.397	0.003	family with sequence similarity 135 member A [Source:HGNC Symbol;Acc:HGNC:21084]	chr6:71123010-71270877	NM_001105531, NN	XM_017011134, XM_0	NP_001099001, NP_00	XP_005248800, XP_0052	protein_coding	ENSG0000008226	57579	21084
2003	IFI27L1	-1.005	0.003	interferon alpha inducible protein 27 like 1 [Source:HGNC Symbol;Acc:HGNC:19754]	chr14:94547627-94569060	NM_145249, NM_2	XM_011536407, XM_0	NP_660292, NP_99683	XP_011534707, XP_0115	protein_coding	ENSG0000016594	122509	19754
2004	IL3RA	-2.694	0.003	interleukin 3 receptor subunit alpha [Source:HGNC Symbol;Acc:HGNC:6012], mapped to	chrX:1455508-1501582	NM_001267713, NN	XM_017030043, XM_0	NP_001254642, NP_00	XP_005274488, XP_0052	protein_coding	ENSG0000018529	3563	6012
2005	KIAA1468	1.180	0.003	KIAA1468 [Source:HGNC Symbol;Acc:HGNC:29289]	chr18:59854490-59975062	NM_020854	XM_011526112, XM_0	NP_065905	XP_005266787, XP_0115	protein_coding	ENSG0000013444	57614	29289
2006	NMD3	0.835	0.003	NMD3 ribosome export adaptor [Source:HGNC Symbol;Acc:HGNC:24250]	chr3:160939077-160971320	NM_001320227, NN	XM_005247512, XM_0	NP_001307156, NP_05	XP_005247568, XP_0052	protein_coding	ENSG0000016925	51068	24250
2007	PDIK1L	1.302	0.003	PDLIM1 interacting kinase 1 like [Source:HGNC Symbol;Acc:HGNC:18981]	chr1:26437655-26452034	NM_001243532, NN	XM_005245743	NP_001230461, NP_00	XP_005245800	protein_coding	ENSG0000017508	149420	18981
2008	S100A5	-1.468	0.003	S100 calcium binding protein A5 [Source:HGNC Symbol;Acc:HGNC:10495]	chr1:153509622-153514241	NM_002962	.	NP_002953	XP_016857520, XP_0168	protein_coding	ENSG0000019642	6276	10495
2009	SPTY2D1	0.904	0.003	SPT2 chromatin protein domain containing 1 [Source:HGNC Symbol;Acc:HGNC:26818]	chr11:18621333-18656040	NM_194285	XM_011519919	NP_919261	XP_011518221	protein_coding	ENSG0000017911	144108	26818
2010	AIFM3	-1.103	0.003	apoptosis inducing factor, mitochondria associated 3 [Source:HGNC Symbol;Acc:HGNC:2	chr22:21319395-21335649	NM_001018060, NN	.	NP_001018070, NP_00	.	protein_coding	ENSG0000018377	150209	26398
2011	ITGB6	1.199	0.003	integrin subunit beta 6 [Source:HGNC Symbol;Acc:HGNC:6161]	chr2:160956176-161056824	NM_000888, NM_0	.	NP_000879, NP_00126	.	protein_coding	ENSG0000011522	100505984	6161
2012	MAP2K4	0.944	0.003	mitogen-activated protein kinase kinase 4 [Source:HGNC Symbol;Acc:HGNC:6844]	chr17:11924140-12047147	NM_001281435, NN	XM_005256755, XM_0	NP_001268364, NP_00	XP_005256810, XP_0052	protein_coding	ENSG0000006555	6416	6844
2013	RAP2A	1.015	0.003	RAP2A, member of RAS oncogene family [Source:HGNC Symbol;Acc:HGNC:9861]	chr13:98086475-98121382	NM_021033	.	NP_066361	.	protein_coding	ENSG0000012524	5911	9861
2014	TBC1D31	1.172	0.003	TBC1 domain family member 31 [Source:HGNC Symbol;Acc:HGNC:30888]	chr8:124084919-124164393	NM_001145088, NN	XM_017013988, XM_0	NP_001138560, NP_66	XP_005251159, XP_0052	protein_coding	ENSG0000015678	93594	30888
2015	TMEM102	-0.869	0.003	transmembrane protein 102 [Source:HGNC Symbol;Acc:HGNC:26722]	chr17:7338761-7340998	NM_001320444, NN	XM_011523798, XM_0	NP_001307373, NP_84	XP_005256671, XP_0115	protein_coding	ENSG0000018128	284114	26722
2016	UBQLN2	-0.908	0.003	ubiquilin 2 [Source:HGNC Symbol;Acc:HGNC:12509]	chrX:56590071-56594301	NM_013444	.	NP_038472	.	protein_coding	ENSG0000018802	29978	12509
2017	CCDC120	-0.987	0.003	coiled-coil domain containing 120 [Source:HGNC Symbol;Acc:HGNC:28910]	chrX:48911100-48927509	NM_001163321, NN	.	NP_001156793, NP_00	.	protein_coding	ENSG0000014714	90060	28910
2018	EIF3H	0.742	0.003	eukaryotic translation initiation factor 3 subunit H [Source:HGNC Symbol;Acc:HGNC:327	chr8:117654368-117778494	NM_003756	.	NP_003747	.	protein_coding	ENSG0000014767	8667	3273
2019	RBM25	0.937	0.003	RNA binding motif protein 25 [Source:HGNC Symbol;Acc:HGNC:23244]	chr14:73525143-73590606	NM_021239	XM_011537044	NP_067062	XP_011535346	protein_coding	ENSG0000011970	58517	23244
2020	SIDT2	-0.967	0.003	SID1 transmembrane family member 2 [Source:HGNC Symbol;Acc:HGNC:24272]	chr11:117049448-117068161	NM_001040455	.	NP_001035545	.	protein_coding	ENSG0000014957	51092	24272
2021	TM4SF19	-1.609	0.003	transmembrane 4 L six family member 19 [Source:HGNC Symbol;Acc:HGNC:25167]	chr3:196046212-196065374	NM_001204897, NN	.	NP_001191826, NP_00	.	protein_coding	ENSG0000014510	116211	25167
2022	TNFAIP2	-0.696	0.003	TNF alpha induced protein 2 [Source:HGNC Symbol;Acc:HGNC:11895]	chr14:103589797-103603776	NM_006291	XM_011537114, XM_0	NP_006282	XP_006720306, XP_0115	protein_coding	ENSG0000018521	7127	11895
2023	ACE	-0.974	0.003	angiotensin I converting enzyme [Source:HGNC Symbol;Acc:HGNC:2707]	chr17:61554421-61575741	NM_000789, NM_0	XM_006721737, XM_0	NP_000780, NP_00117	XP_006721800	protein_coding	ENSG0000015964	1636	2707
2024	AIM1L	-1.034	0.003	absent in melanoma 1-like [Source:HGNC Symbol;Acc:HGNC:17295]	chr1:26648349-26680621	NM_001039775	XM_011541674, XM_0	NP_001034864	XP_005245975, XP_0115	protein_coding	ENSG0000017609	55057	17295
2025	ALMS1	1.006	0.003	ALMS1, centrosome and basal body associated protein [Source:HGNC Symbol;Acc:HGN(	chr2:73612885-73837046	NM_015120	.	NP_055935	.	protein_coding	ENSG0000011612	7840	428
2026	GSTP1	-0.712	0.003	glutathione S-transferase pi 1 [Source:HGNC Symbol;Acc:HGNC:4638]	chr11:67351065-67354131	NM_000852	.	NP_000843	.	protein_coding	ENSG0000008420	2950	4638
2027	IDE	0.891	0.003	insulin degrading enzyme [Source:HGNC Symbol;Acc:HGNC:5381]	chr10:94211440-94333833	NM_001165946, NN	XM_005269766	NP_001159418, NP_00	XP_016871676, XP_0168	protein_coding	ENSG0000011991	3416	5381
2028	KRT16	-3.254	0.003	keratin 16 [Source:HGNC Symbol;Acc:HGNC:6423]	chr17:39766029-39769005	NM_005557	.	NP_005548	.	protein_coding	ENSG0000018683	3868	6423
2029	MEIS2	1.262	0.003	Meis homeobox 2 [Source:HGNC Symbol;Acc:HGNC:7001]	chr15:37181404-37393500	NM_001220482, NN	XM_006720529, XM_0	NP_001207411, NP_00	XP_006720585, XP_0067	protein_coding	ENSG0000013413	4212	7001
2030	SLAIN2	0.912	0.003	SLAIN motif family member 2 [Source:HGNC Symbol;Acc:HGNC:29282]	chr4:48343338-48428229	NM_020846	XM_005248121	NP_065897	XP_005248178	protein_coding	ENSG0000010917	57606	29282
2031	TARDBP	0.864	0.003	TAR DNA binding protein [Source:HGNC Symbol;Acc:HGNC:11571]	chr1:11072456-11086477	NM_007375	.	NP_031401	XP_016856352, XP_0168	protein_coding	ENSG0000012094	23435	11571
2032	ZNF841	1.157	0.003	zinc finger protein 841 [Source:HGNC Symbol;Acc:HGNC:27611]	chr19:52567718-52599018	NM_001136499, NN	XM_017026657, XM_0	NP_001129971, NP_00	XP_011525135, XP_0115	protein_coding	ENSG0000019760	284371	27611
2033	C16orf13	-0.856	0.003	methyltransferase like 26 [Source:HGNC Symbol;Acc:HGNC:14141]	chr16:684426-686366	NM_001040160, NN	XM_011522714, XM_0	NP_001035250, NP_00	XP_011521015, XP_0115	protein_coding	ENSG0000013073	84326	14141
2034	EXOC5	1.509	0.003	exocyst complex component 5 [Source:HGNC Symbol;Acc:HGNC:10696]	chr14:57667224-57765460	NM_006544	XM_005267272	NP_006535	XP_005267329	protein_coding	ENSG0000007036	10640	10696
2035	IREB2	1.032	0.003	iron responsive element binding protein 2 [Source:HGNC Symbol;Acc:HGNC:6115]	chr15:78730530-78793798	NM_001320942, NN	.	NP_001307871, NP_00	.	protein_coding	ENSG0000013638	3658	6115
2036	MALL	-0.932	0.003	mal, T-cell differentiation protein like [Source:HGNC Symbol;Acc:HGNC:6818]	chr2:110841446-110874143	NM_005434	.	NP_005425	.	protein_coding	ENSG0000014406	7851	6818
2037	NFIB	1.446	0.003	nuclear factor I B [Source:HGNC Symbol;Acc:HGNC:7785]	chr9:14081841-14398982	NM_001190737, NN	XM_017014741, XM_0	NP_001177666, NP_00	XP_005251524, XP_0052	protein_coding	ENSG0000014786	4781	7785
2038	PNPLA8	1.027	0.003	patatin like phospholipase domain containing 8 [Source:HGNC Symbol;Acc:HGNC:28900	chr7:108110865-108166762	NM_001256007, NN	XM_011516275, XM_0	NP_001242936, NP_00	XP_005250453, XP_0115	protein_coding	ENSG0000013524	50640	28900
2039	SELK	-0.809	0.003	selenoprotein K [Source:EntrezGene;Acc:58515]	chr3:53918436-53926015	NM_021237	.	NP_067060	.	protein_coding	ENSG0000011381	58515	.
2040	SKAP2	0.893	0.003	src kinase associated phosphoprotein 2 [Source:HGNC Symbol;Acc:HGNC:15687]	chr7:26706680-26904362	NM_001303468, NN	.	NP_001290397, NP_00	XP_016868260	protein_coding	ENSG0000005502	8935	15687
2041	STK11	-0.821	0.003	serine/threonine kinase 11 [Source:HGNC Symbol;Acc:HGNC:11389]	chr19:1205803-1238026	NM_000455	XM_011528209, XM_0	NP_000446	XP_005259674, XP_0115	protein_coding	ENSG0000011804	6794	11389
2042	SUV39H2	1.370	0.003	suppressor of variegation 3-9 homolog 2 [Source:HGNC Symbol;Acc:HGNC:17287]	chr10:14920861-14996431	NM_001193424, NN	XM_011519662, XM_0	NP_001180353, NP_00	XP_006717566, XP_0115	protein_coding	ENSG0000015245	79723	17287
2043	TLE3	-0.722	0.003	transducin like enhancer of split 3 [Source:HGNC Symbol;Acc:HGNC:11839]	chr15:70340128-70390515	NM_001105192, NN	XM_011521983, XM_0	NP_001098662, NP_00	XP_005254679, XP_0052	protein_coding	ENSG0000014033	7090	11839
2044	ZNF700	1.189	0.003	zinc finger protein 700 [Source:HGNC Symbol;Acc:HGNC:25292]	chr19:12035882-12091196	NM_001271848, NN	.	NP_001258777, NP_65	.	protein_coding	ENSG0000019675	90592	25292
2045	CDKN2D	-1.008	0.003	cyclin dependent kinase inhibitor 2D [Source:HGNC Symbol;Acc:HGNC:1790]	chr19:10677137-10679735	NM_001800, NM_0	.	NP_001791, NP_52414	.	protein_coding	ENSG0000012935	1032	1790
2046	EXOC6B	0.940	0.003	exocyst complex component 6B [Source:HGNC Symbol;Acc:HGNC:17085]	chr2:72403112-73053170	NM_001321729, NN	XM_017003641, XM_0	NP_001308658, NP_00	XP_005264280, XP_0115	protein_coding	ENSG0000014403	23233	17085
2047	FAM171A2	-1.070	0.003	family with sequence similarity 171 member A2 [Source:HGNC Symbol;Acc:HGNC:30480	chr17:42430582-42441243	NM_198475	.	NP_940877	.	protein_coding	ENSG0000016168	284069	30480
2048	HIP1	0.725	0.003	huntingtin interacting protein 1 [Source:HGNC Symbol;Acc:HGNC:4913]	chr7:75162620-75368265	NM_001243198, NN	XM_017012099, XM_0	NP_001230127, NP_00	XP_005250361, XP_0052	protein_coding	ENSG0000012794	3092	4913
2049	IL15RA	-0.984	0.003	interleukin 15 receptor subunit alpha [Source:HGNC Symbol;Acc:HGNC:5978]	chr10:5985601-6020150	NM_001243539, NN	XM_011519477, XM_0	NP_001230468, NP_00	XP_011517778, XP_0115	protein_coding	ENSG0000013447	3601	5978
2050	MMP25	-1.289	0.003	matrix metallopeptidase 25 [Source:HGNC Symbol;Acc:HGNC:14246]	chr16:3096681-3110727	NM_022468	XM_011522603	NP_071913	.	protein_coding	ENSG0000000851	64386	14246

2051	PKIG	-0.840	0.003	protein kinase (cAMP-dependent, catalytic) inhibitor gamma [Source:HGNC Symbol;Acc	chr20:43160425-43280383	NM_001281444, NN	.	NP_001268373, NP_00	XP_016883103, XP_0168	protein_coding	ENSG0000016873	11142	9019
2052	SEPT7	0.891	0.003	septin 7 [Source:HGNC Symbol;Acc:HGNC:1717]	chr7:35840541-35946715	NM_001011553, NN	XM_011515662, XM_0	NP_001011553, NP_00	XP_006715869, XP_0168	protein_coding	ENSG0000012254	989	1717
2053	SYNRG	0.944	0.003	synergin, gamma [Source:HGNC Symbol;Acc:HGNC:557]	chr17:35874899-35969544	NM_001163544, NN	XM_011524252, XM_0	NP_001157016, NP_00	XP_005257037, XP_0168	protein_coding	ENSG0000027506	11276	557
2054	TGM2	-0.781	0.003	transglutaminase 2 [Source:HGNC Symbol;Acc:HGNC:11778]	chr20:36755788-36793774	NM_001323316, NN	XM_011529029, XM_0	NP_001310245, NP_00	XP_011527330, XP_0115	protein_coding	ENSG0000019895	7052	11778
2055	TM9SF3	0.802	0.003	transmembrane 9 superfamily member 3 [Source:HGNC Symbol;Acc:HGNC:21529]	chr10:98277865-98346809	NM_020123	XM_011539977, XM_0	NP_064508	XP_011538278, XP_0115	protein_coding	ENSG0000007714	56889	21529
2056	VPS41	0.961	0.003	VPS41, HOPS complex subunit [Source:HGNC Symbol;Acc:HGNC:12713]	chr7:38762562-38948829	NM_014396, NM_0	.	NP_055211, NP_54219	.	protein_coding	ENSG0000000671	27072	12713
2057	ANXA3	0.712	0.003	annexin A3 [Source:HGNC Symbol;Acc:HGNC:541]	chr4:79472672-79531575	NM_005139	XM_011531906	NP_005130	.	protein_coding	ENSG0000013877	306	541
2058	DDX50	0.875	0.003	DEAD-box helicase 50 [Source:HGNC Symbol;Acc:HGNC:17906]	chr10:70661033-70706603	NM_024045	XM_011540144, XM_0	NP_076950	XP_005270205, XP_0115	protein_coding	ENSG0000010762	79009	17906
2059	DNM1L	0.963	0.003	dynamin 1 like [Source:HGNC Symbol;Acc:HGNC:2973]	chr12:32832133-32898584	NM_001278463, NN	XM_011520544, XM_0	NP_001265392, NP_00	XP_005253339, XP_0115	protein_coding	ENSG0000008747	10059	2973
2060	FAM167A	-1.285	0.003	family with sequence similarity 167 member A [Source:HGNC Symbol;Acc:HGNC:15549]	chr8:11278971-11324276	NM_053279	XM_011543840, XM_0	NP_444509	XP_005272455, XP_0115	protein_coding	ENSG0000015431	83648	15549
2061	FOPNL	0.808	0.003	FGFR1OP N-terminal like [Source:HGNC Symbol;Acc:HGNC:26435]	chr16:15959576-15982482	NM_001304497, NN	.	NP_001291426, NP_00	.	protein_coding	ENSG0000013339	123811	26435
2062	SLC25A36	1.038	0.003	solute carrier family 25 member 36 [Source:HGNC Symbol;Acc:HGNC:25554]	chr3:140660671-140698775	NM_001104647, NN	.	NP_001098117, NP_06	.	protein_coding	ENSG0000011412	55186	25554
2063	TAB1	-0.832	0.003	TGF-beta activated kinase 1 (MAP3K7) binding protein 1 [Source:HGNC Symbol;Acc:HGN	chr22:39795745-39832828	NM_006116, NM_1	.	NP_006107, NP_70571	.	protein_coding	ENSG0000010032	10454	18157
2064	TMEM87A	0.939	0.003	transmembrane protein 87A [Source:HGNC Symbol;Acc:HGNC:24522]	chr15:42502649-42649133	NM_001110503, NN	XM_011521443, XM_0	NP_001103973, NP_00	XP_005254344, XP_0115	protein_coding	ENSG0000010397	25963	24522
2065	TULP4	1.019	0.003	tubby like protein 4 [Source:HGNC Symbol;Acc:HGNC:15530]	chr6:158733519-158932860	NM_001007466, NN	XM_017011070, XM_0	NP_001007467, NP_06	XP_011534248, XP_0168	protein_coding	ENSG0000013033	56995	15530
2066	APBB1	-0.997	0.003	amyloid beta precursor protein binding family B member 1 [Source:HGNC Symbol;Acc:H	chr11:6416353-6440644	NM_001164, NM_0	XM_017017641, XM_0	NP_001155, NP_00124	XP_006718272, XP_0067	protein_coding	ENSG0000016631	322	581
2067	PAM	0.709	0.003	peptidylglycine alpha-amidating monooxygenase [Source:HGNC Symbol;Acc:HGNC:859	chr5:102201429-102366809	NM_000919, NM_0	XM_017009509, XM_0	NP_000910, NP_00117	XP_006714695, XP_0067	protein_coding	ENSG0000014573	5066	8596
2068	TMED2	0.706	0.003	transmembrane p24 trafficking protein 2 [Source:HGNC Symbol;Acc:HGNC:16996]	chr12:124069077-124083124	NM_006815	.	NP_001308374, NP_00	.	protein_coding	ENSG0000008659	10959	16996
2069	ZNF367	0.880	0.003	zinc finger protein 367 [Source:HGNC Symbol;Acc:HGNC:18320]	chr9:99148222-99180611	NM_153695	.	NP_710162	.	protein_coding	ENSG0000016524	195828	18320
2070	ARHGEF9	-1.100	0.003	Cdc42 guanine nucleotide exchange factor 9 [Source:HGNC Symbol;Acc:HGNC:14561]	chrX:62854846-63005438	NM_001173479, NN	XM_017029367, XM_0	NP_001166950, NP_00	XP_005262306, XP_0052	protein_coding	ENSG0000013108	23229	14561
2071	FIG4	1.055	0.003	FIG4 phosphoinositide 5-phosphatase [Source:HGNC Symbol;Acc:HGNC:16873]	chr6:110012514-110146631	NM_014845	XM_011536281	NP_055660	XP_011534583	protein_coding	ENSG0000011236	9896	16873
2072	GATSL3	-1.402	0.003	GATS protein like 3 [Source:HGNC Symbol;Acc:HGNC:34423]	chr22:30681105-30685616	NM_001037666	.	NP_001032755	.	protein_coding	ENSG0000023928	652968	34423
2073	GNAQ	0.914	0.003	G protein subunit alpha q [Source:HGNC Symbol;Acc:HGNC:4390]	chr9:80331002-80646374	NM_002072	.	NP_002063	.	protein_coding	ENSG0000015605	2776	4390
2074	GOLGA1	0.935	0.003	golgin A1 [Source:HGNC Symbol;Acc:HGNC:4424]	chr9:127640635-127703378	NM_002077	XM_006717063, XM_0	NP_002068	XP_005251986, XP_0067	protein_coding	ENSG0000013693	2800	4424
2075	PPP4R3A	0.856	0.003	protein phosphatase 4 regulatory subunit 3A [Source:HGNC Symbol;Acc:HGNC:20219]	chr14:91923954-91976822	NM_001284280, NN	XM_006720194, XM_0	NP_001271209, NP_00	XP_005267899, XP_0067	protein_coding	ENSG0000010079	55671	20219
2076	PTGER2	1.746	0.003	prostaglandin E receptor 2 [Source:HGNC Symbol;Acc:HGNC:9594]	chr14:52781022-52795324	NM_000956	.	NP_000947	.	protein_coding	ENSG0000012538	5732	9594
2077	RRP7A	-0.716	0.003	ribosomal RNA processing 7 homolog A [Source:HGNC Symbol;Acc:HGNC:24286]	chr22:42905973-42915808	NM_015703	.	NP_056518	.	protein_coding	ENSG0000018930	27341	24286
2078	TAF1D	1.158	0.003	TATA-box binding protein associated factor, RNA polymerase I subunit D [Source:HGNC	chr11:93468732-93497915	NM_024116	.	NP_077021	.	protein_coding	ENSG0000016601	100302240	28759
2079	VPS45	0.976	0.003	vacuolar protein sorting 45 homolog [Source:HGNC Symbol;Acc:HGNC:14579]	chr1:150039349-150117505	NM_001279353, NN	.	NP_001266282, NP_00	.	protein_coding	ENSG0000013663	11311	14579
2080	C21orf2	-1.136	0.003	chromosome 21 open reading frame 2 [Source:HGNC Symbol;Acc:HGNC:1260]	chr21:45748826-45759285	NM_001271440, NN	XM_017028470	NP_001258369, NP_00	XP_016883959	protein_coding	ENSG0000016022	755	1260
2081	GJC2	-1.779	0.003	gap junction protein gamma 2 [Source:HGNC Symbol;Acc:HGNC:17494]	chr1:228337552-228347527	NM_020435	.	NP_065168	.	protein_coding	ENSG0000019883	57165	17494
2082	GUK1	-0.736	0.003	guanylate kinase 1 [Source:HGNC Symbol;Acc:HGNC:4693]	chr1:228327662-228336685	NM_000858, NM_0	XM_017001096, XM_0	NP_000849, NP_00115	XP_005273160, XP_0052	protein_coding	ENSG0000014377	2987	4693
2083	LYNX1	-1.445	0.003	Ly6/neurotoxin 1 [Source:HGNC Symbol;Acc:HGNC:29604]	chr8:143845751-143859640	NM_023946, NM_1	XM_011517238	NP_076435, NP_80325	XP_011515540	protein_coding	ENSG0000018015	66004	29604
2084	SOS1	1.074	0.003	SOS Ras/Rac guanine nucleotide exchange factor 1 [Source:HGNC Symbol;Acc:HGNC:11	chr2:39208536-39348192	NM_005633	XM_011533065, XM_0	NP_005624	XP_005264572, XP_0115	protein_coding	ENSG0000011590	6654	11187
2085	ARFGEF2	0.821	0.003	ADP ribosylation factor guanine nucleotide exchange factor 2 [Source:HGNC Symbol;Acc	chr20:47538426-47653230	NM_006420	XM_005260252	NP_006411	XP_005260309	protein_coding	ENSG0000012419	10564	15853
2086	CDR2	0.903	0.003	cerebellar degeneration related protein 2 [Source:HGNC Symbol;Acc:HGNC:1799]	chr16:22357256-22385938	NM_001802	XM_005276502	NP_001793	XP_005276559	protein_coding	ENSG0000014074	101060399	1799
2087	CLDN9	-1.189	0.003	claudin 9 [Source:HGNC Symbol;Acc:HGNC:2051]	chr16:3062456-3064506	NM_020982	.	NP_066192	.	protein_coding	ENSG0000021393	9080	2051
2088	GLO1	0.765	0.003	glyoxalase I [Source:HGNC Symbol;Acc:HGNC:4323]	chr6:38643700-38670917	NM_006708	.	NP_006699	.	protein_coding	ENSG0000012476	2739	4323
2089	NR2C1	1.116	0.003	nuclear receptor subfamily 2 group C member 1 [Source:HGNC Symbol;Acc:HGNC:7971]	chr12:95414004-95467479	NM_001032287, NN	XM_011538717, XM_0	NP_001027458, NP_00	XP_005269190, XP_0067	protein_coding	ENSG0000012079	7181	7971
2090	PGLS	-0.812	0.003	6-phosphogluconolactonase [Source:HGNC Symbol;Acc:HGNC:8903]	chr19:17622437-17632097	NM_012088	.	NP_036220	.	protein_coding	ENSG0000013031	25796	8903
2091	SLC38A2	0.770	0.003	solute carrier family 38 member 2 [Source:HGNC Symbol;Acc:HGNC:13448]	chr12:46751971-46766650	NM_001307936, NN	XM_005268983	NP_001294865, NP_06	XP_005269040	protein_coding	ENSG0000013429	54407	13448
2092	STAU2	1.064	0.003	staufen double-stranded RNA binding protein 2 [Source:HGNC Symbol;Acc:HGNC:11371	chr8:74332603-74659943	NM_001164380, NN	.	NP_001157852, NP_00	.	protein_coding	ENSG0000004034	27067	11371
2093	ARRDC2	-0.844	0.004	arrestin domain containing 2 [Source:HGNC Symbol;Acc:HGNC:25225]	chr19:18111940-18124911	NM_001025604, NN	.	NP_001020775, NP_00	.	protein_coding	ENSG0000010564	27106	25225
2094	CRABP1	-1.068	0.004	cellular retinoic acid binding protein 1 [Source:HGNC Symbol;Acc:HGNC:2338]	chr15:78632665-78640572	NM_004378	.	NP_004369	.	protein_coding	ENSG0000016642	1381	2338
2095	CTH	1.015	0.004	cystathionine gamma-lyase [Source:HGNC Symbol;Acc:HGNC:2501]	chr1:70876900-70905534	NM_001190463, NN	XM_005270509	NP_001177392, NP_00	XP_005270566	protein_coding	ENSG0000011676	1491	2501
2096	DAB2IP	-0.814	0.004	DAB2 interacting protein [Source:HGNC Symbol;Acc:HGNC:17294]	chr9:124329380-124547809	NM_032552, NM_1	XM_017014300, XM_0	NP_115941, NP_61972	XP_005251776, XP_0052	protein_coding	ENSG0000013684	153090	17294
2097	DHX57	0.948	0.004	DEAH-box helicase 57 [Source:HGNC Symbol;Acc:HGNC:20086]	chr2:39024870-39103049	NM_198963	XM_011533156, XM_0	NP_945314	XP_011531456, XP_0115	protein_coding	ENSG0000016321	90957	20086
2098	MRPS26	-0.731	0.004	mitochondrial ribosomal protein S26 [Source:HGNC Symbol;Acc:HGNC:14045]	chr20:3026590-3028900	NM_030811	.	NP_110438	.	protein_coding	ENSG0000012590	64949	14045
2099	RAP2C	0.956	0.004	RAP2C, member of RAS oncogene family [Source:HGNC Symbol;Acc:HGNC:21165]	chrX:131337051-131353508	NM_001271186, NN	XM_011531376, XM_0	NP_001258115, NP_00	XP_006724838, XP_0115	protein_coding	ENSG0000012372	57826	21165
2100	CDC42EP1	-0.788	0.004	CDC42 effector protein 1 [Source:HGNC Symbol;Acc:HGNC:17014]	chr22:37956453-37965412	NM_152243	XM_017028541, XM_0	NP_689449	XP_006724170, XP_0067	protein_coding	ENSG0000012828	11135	17014
2101	FAM200A	1.097	0.004	family with sequence similarity 200 member A [Source:HGNC Symbol;Acc:HGNC:25401]	chr7:99143930-99149757	NM_145111	XM_011515913, XM_0	NP_659802	XP_005250256, XP_0115	protein_coding	ENSG0000022190	221786	25401
2102	GALM	-0.837	0.004	galactose mutarotase [Source:HGNC Symbol;Acc:HGNC:24063]	chr2:38893051-38961910	NM_138801	.	NP_620156	.	protein_coding	ENSG0000014389	130589	24063
2103	PPP1CC	0.741	0.004	protein phosphatase 1 catalytic subunit gamma [Source:HGNC Symbol;Acc:HGNC:9283]	chr12:111157484-111180744	NM_001244974, NN	XM_011538505, XM_0	NP_001231903, NP_00	XP_011536806, XP_0115	protein_coding	ENSG0000018629	5501	9283
2104	RRAS	-0.738	0.004	related RAS viral (r-ras) oncogene homolog [Source:HGNC Symbol;Acc:HGNC:10447]	chr19:50138548-50143458	NM_006270	.	NP_006261	.	protein_coding	ENSG0000012645	6237	10447

2105	EPC1	1.140	0.004	enhancer of polycomb homolog 1 [Source:HGNC Symbol;Acc:HGNC:19876]	chr10:32556678-32667726	NM_001272004, NN	XM_006717510	NP_001258933, NP_00	XP_006717573	protein_coding	ENSG0000012061	80314	19876
2106	FOXO3	0.872	0.004	forkhead box O3 [Source:HGNC Symbol;Acc:HGNC:3821]	chr6:108881037-109005977	NM_001455, NM_2	XM_011535629, XM_0	NP_001446, NP_96385	XP_005266925, XP_0115	protein_coding	ENSG0000011868	2309	3821
2107	PPAT	0.855	0.004	phosphoribosyl pyrophosphate amidotransferase [Source:HGNC Symbol;Acc:HGNC:923	chr4:57259527-57301781	NM_002703	XM_011534387	NP_002694	XP_011532689	protein_coding	ENSG0000012805	5471	9238
2108	SLC16A4	1.132	0.004	solute carrier family 16 member 4 [Source:HGNC Symbol;Acc:HGNC:10925]	chr1:110905469-110933704	NM_001201546, NN	XM_006711033, XM_0	NP_001188475, NP_00	XP_005271374, XP_0067	protein_coding	ENSG0000016867	9122	10925
2109	TMEM133	1.742	0.004	transmembrane protein 133 [Source:HGNC Symbol;Acc:HGNC:24033]	chr11:100862719-100864672	NM_032021	.	NP_114410	.	protein_coding	ENSG0000017064	83935	24033
2110	ATG4C	1.149	0.004	autophagy related 4C cysteine peptidase [Source:HGNC Symbol;Acc:HGNC:16040]	chr1:63249805-63331184	NM_032852, NM_1	XM_011542313, XM_0	NP_116241, NP_83573	XP_005271345, XP_0115	protein_coding	ENSG0000012570	84938	16040
2111	ATP8B2	-1.080	0.004	ATPase phospholipid transporting 8B2 [Source:HGNC Symbol;Acc:HGNC:13534]	chr1:154298028-154323783	NM_001005855, NN	XM_017001872, XM_0	NP_001005855, NP_06	XP_005245412, XP_0052	protein_coding	ENSG0000014351	57198	13534
2112	C2CD5	0.935	0.004	C2 calcium dependent domain containing 5 [Source:HGNC Symbol;Acc:HGNC:29062]	chr12:22601516-22697480	NM_001286173, NN	XM_011520886, XM_0	NP_001273102, NP_00	XP_005253595, XP_0067	protein_coding	ENSG0000011173	9847	29062
2113	NUF2	0.948	0.004	NUF2, NDC80 kinetochore complex component [Source:HGNC Symbol;Acc:HGNC:14621	chr1:163291689-163325554	NM_031423, NM_1	XM_011510036	NP_113611, NP_66373	XP_011508338	protein_coding	ENSG0000014322	83540	14621
2114	VDAC3	0.745	0.004	voltage dependent anion channel 3 [Source:HGNC Symbol;Acc:HGNC:12674]	chr8:42249381-42263415	NM_001135694, NN	.	NP_001129166, NP_00	.	protein_coding	ENSG0000007866	7419	12674
2115	CENPI	1.075	0.004	centromere protein I [Source:HGNC Symbol;Acc:HGNC:3968]	chrX:100355425-100418670	NM_006733	XM_011530902, XM_0	NP_001305450, NP_00	XP_005262168, XP_0115	protein_coding	ENSG0000010238	2491	3968
2116	CST4	-4.249	0.004	cystatin S [Source:HGNC Symbol;Acc:HGNC:2476]	chr20:23666276-23669677	NM_001899	.	NP_001890	.	protein_coding	ENSG0000010144	1472	2476
2117	GYS1	-0.759	0.004	glycogen synthase 1 [Source:HGNC Symbol;Acc:HGNC:4706]	chr19:49471381-49496567	NM_001161587, NN	.	NP_001155059, NP_00	.	protein_coding	ENSG0000010481	2997	4706
2118	TNFAIP8	1.167	0.004	TNF alpha induced protein 8 [Source:HGNC Symbol;Acc:HGNC:17260]	chr5:118604386-118735383	NM_001077654, NN	.	NP_001071122, NP_00	XP_016864816	protein_coding	ENSG0000014577	25816	17260
2119	ATMIN	0.823	0.004	ATM interactor [Source:HGNC Symbol;Acc:HGNC:29034]	chr16:81069451-81080963	NM_001300728, NN	.	NP_001287657, NP_05	.	protein_coding	ENSG0000016645	23300	29034
2120	IVNS1ABP	0.765	0.004	influenza virus NS1A binding protein [Source:HGNC Symbol;Acc:HGNC:16951]	chr1:185265519-185286461	NM_006469	XM_011509080, XM_0	NP_006460	XP_005244900, XP_0115	protein_coding	ENSG0000011667	10625	16951
2121	MOB1B	1.064	0.004	MOB kinase activator 1B [Source:HGNC Symbol;Acc:HGNC:29801]	chr4:71768052-71853885	NM_001244766, NN	XM_017008837, XM_0	NP_001231695, NP_77	XP_005265766, XP_0115	protein_coding	ENSG0000017354	92597	29801
2122	NR2F6	-0.761	0.004	nuclear receptor subfamily 2 group F member 6 [Source:HGNC Symbol;Acc:HGNC:7977]	chr19:17342691-17375605	NM_005234	.	NP_005225	.	protein_coding	ENSG0000016011	2063	7977
2123	PPM1H	0.866	0.004	protein phosphatase, Mg2+/Mn2+ dependent 1H [Source:HGNC Symbol;Acc:HGNC:185	chr12:63037761-63328817	NM_020700	XM_011538579, XM_0	NP_065751	XP_011536880, XP_0115	protein_coding	ENSG0000011111	57460	18583
2124	TREX1	-1.014	0.004	three prime repair exonuclease 1 [Source:HGNC Symbol;Acc:HGNC:12269]	chr3:48507209-48509044	NM_007248, NM_0	.	NP_009179, NP_05746	.	protein_coding	ENSG0000021368	11277	12269
2125	USP40	0.885	0.004	ubiquitin specific peptidase 40 [Source:HGNC Symbol;Acc:HGNC:20069]	chr2:234384165-234475428	NM_018218	XM_017004426, XM_0	NP_060688	XP_006712675, XP_0115	protein_coding	ENSG0000008598	55230	20069
2126	CAMK4	1.541	0.004	calcium/calmodulin dependent protein kinase IV [Source:HGNC Symbol;Acc:HGNC:1464	chr5:110559647-110830584	NM_001323374, NN	.	NP_001310303, NP_00	.	protein_coding	ENSG0000015249	814	1464
2127	FNDC4	-0.943	0.004	fibronectin type III domain containing 4 [Source:HGNC Symbol;Acc:HGNC:20239]	chr2:27714749-27718112	NM_022823	XM_005264499	NP_073734	XP_005264556	protein_coding	ENSG0000011522	64838	20239
2128	HIST1H3E	2.229	0.004	histone cluster 1, H3e [Source:HGNC Symbol;Acc:HGNC:4769]	chr6:26224426-26227701	NM_003532	.	NP_003523	.	protein_coding	ENSG0000027475	8353	4769
2129	PIM3	-0.717	0.004	Pim-3 proto-oncogene, serine/threonine kinase [Source:HGNC Symbol;Acc:HGNC:1931	chr22:50354160-50357728	NM_001001852	.	NP_001001852	.	protein_coding	ENSG0000019839	415116	19310
2130	SIX4	0.899	0.004	SIX homeobox 4 [Source:HGNC Symbol;Acc:HGNC:10890]	chr14:61176245-61190852	NM_017420	.	NP_059116	XP_005267816	protein_coding	ENSG0000010062	51804	10890
2131	SPECC1L	0.766	0.004	sperm antigen with calponin homology and coiled-coil domains 1 like [Source:HGNC Syr	chr22:24666784-24813708	NM_001145468, NN	.	NP_001138940, NP_00	.	protein_coding	ENSG0000010001	23384	29022
2132	ZFP62	0.944	0.004	ZFP62 zinc finger protein [Source:HGNC Symbol;Acc:HGNC:23241]	chr5:180274614-180288277	NM_001172638, NN	XM_017009717, XM_0	NP_001166109, NP_68	XP_016865199, XP_0168	protein_coding	ENSG0000019667	643836	23241
2133	EHBP1L1	-0.672	0.004	EH domain binding protein 1 like 1 [Source:HGNC Symbol;Acc:HGNC:30682]	chr11:65343508-65360121	NM_001099409	XM_011544885, XM_0	NP_001092879	XP_005273924, XP_0052	protein_coding	ENSG0000017344	254102	30682
2134	GJB3	-0.723	0.004	gap junction protein beta 3 [Source:HGNC Symbol;Acc:HGNC:4285]	chr1:35246789-35251970	NM_001005752, NN	.	NP_001005752, NP_07	.	protein_coding	ENSG0000018891	2707	4285
2135	METTL14	1.053	0.004	methyltransferase like 14 [Source:HGNC Symbol;Acc:HGNC:29330]	chr4:119606522-119636588	NM_020961	XM_005263166	NP_066012	XP_005263223	protein_coding	ENSG0000014538	57721	29330
2136	NFAT5	0.855	0.004	nuclear factor of activated T-cells 5 [Source:HGNC Symbol;Acc:HGNC:7774]	chr16:69599868-69738569	NM_001113178, NN	XM_011522822, XM_0	NP_001106649, NP_00	XP_006721188, XP_0115	protein_coding	ENSG0000010290	10725	7774
2137	PSTPIP2	0.856	0.004	proline-serine-threonine phosphatase interacting protein 2 [Source:HGNC Symbol;Acc:H	chr18:43563501-43652226	NM_024430	XM_011526256, XM_0	NP_077748	XP_011524555, XP_0115	protein_coding	ENSG0000015222	9050	9581
2138	UBR3	0.948	0.004	ubiquitin protein ligase E3 component n-recognin 3 (putative) [Source:HGNC Symbol;Ac	chr2:170683967-170940641	NM_172070	XM_006712271, XM_0	NP_742067	XP_005246356, XP_0052	protein_coding	ENSG0000014435	130507	30467
2139	AHI1	1.713	0.004	Abelson helper integration site 1 [Source:HGNC Symbol;Acc:HGNC:21575]	chr6:135604669-135818914	NM_001134830, NN	XM_017010981, XM_0	NP_001128302, NP_00	XP_011534212, XP_0115	protein_coding	ENSG0000013554	54806	21575
2140	CHCHD10	-0.823	0.004	coiled-coil-helix-coiled-coil-helix domain containing 10 [Source:HGNC Symbol;Acc:HGNC	chr22:24105240-24110630	NM_001301339, NN	.	NP_001288268, NP_99	.	protein_coding	ENSG0000025047	400916	15559
2141	KIAA1841	1.120	0.004	KIAA1841 [Source:HGNC Symbol;Acc:HGNC:29387]	chr2:61293067-61365169	NM_001129993, NN	XM_017005108, XM_0	NP_001123465, NP_11	XP_005264662, XP_0052	protein_coding	ENSG0000016292	84542	29387
2142	LSM3	-1.059	0.004	LSM3 homolog, U6 small nuclear RNA and mRNA degradation associated [Source:HGNC	chr3:14186646-14242619	NM_014463	.	NP_055278	.	protein_coding	ENSG0000017086	27258	17874
2143	MKL2	1.000	0.004	MKL1/myocardin like 2 [Source:HGNC Symbol;Acc:HGNC:29819]	chr16:14165178-14360629	NM_001308142, NN	XM_017023504, XM_0	NP_001295071, NP_05	XP_005255508, XP_0052	protein_coding	ENSG0000018626	57496	29819
2144	NCAPG2	0.755	0.004	non-SMC condensin II complex subunit G2 [Source:HGNC Symbol;Acc:HGNC:21904]	chr7:158424004-158497520	NM_001281932, NN	XM_011516357, XM_0	NP_001268861, NP_00	XP_005249604, XP_0115	protein_coding	ENSG0000014691	54892	21904
2145	PARN	0.914	0.004	poly(A)-specific ribonuclease [Source:HGNC Symbol;Acc:HGNC:8609]	chr16:14529557-14724133	NM_001134477, NN	XM_011522513, XM_0	NP_001127949, NP_00	XP_011520812, XP_0115	protein_coding	ENSG0000014069	5073	8609
2146	PPP1R18	-0.825	0.004	protein phosphatase 1 regulatory subunit 18 [Source:HGNC Symbol;Acc:HGNC:29413]	chr6:30644165-30655672	NM_001134870, NN	.	NP_001128342, NP_59	.	protein_coding	ENSG0000014611	170954	29413
2147	PSMD1	0.717	0.004	proteasome 26S subunit, non-ATPase 1 [Source:HGNC Symbol;Acc:HGNC:9554]	chr2:231921577-232037541	NM_001191037, NN	.	NP_001177966, NP_00	.	protein_coding	ENSG0000017369	5707	9554
2148	SLU7	0.783	0.004	SLU7 homolog, splicing factor [Source:HGNC Symbol;Acc:HGNC:16939]	chr5:159828647-159846401	NM_006425	XM_011534418, XM_0	NP_006416	XP_005265850, XP_0115	protein_coding	ENSG0000016460	10569	16939
2149	ARMT1	0.966	0.004	acidic residue methyltransferase 1 [Source:HGNC Symbol;Acc:HGNC:17872]	chr6:151773392-151791236	NM_001286562, NN	.	NP_001273491, NP_07	.	protein_coding	ENSG0000014647	79624	17872
2150	HIVEP2	0.925	0.004	human immunodeficiency virus type I enhancer binding protein 2 [Source:HGNC Symbo	chr6:143072603-143266338	NM_006734	XM_017010805	NP_006725	XP_016866294	protein_coding	ENSG0000001081	3097	4921
2151	LSM7	-0.797	0.004	LSM7 homolog, U6 small nuclear RNA and mRNA degradation associated [Source:HGNC	chr19:2321515-2328619	NM_016199	.	NP_057283	.	protein_coding	ENSG0000013033	51690	20470
2152	ULK1	-0.907	0.004	unc-51 like autophagy activating kinase 1 [Source:HGNC Symbol;Acc:HGNC:12558]	chr12:132379195-132407712	NM_003565	XM_011538799, XM_0	NP_003556	XP_011537100, XP_0115	protein_coding	ENSG0000017716	8408	12558
2153	ZNF277	0.916	0.004	zinc finger protein 277 [Source:HGNC Symbol;Acc:HGNC:13070]	chr7:111846642-111983151	NM_021994	XM_011515768, XM_0	NP_068834	XP_005250186, XP_0115	protein_coding	ENSG0000019883	11179	13070
2154	CDCP1	-0.688	0.004	CUB domain containing protein 1 [Source:HGNC Symbol;Acc:HGNC:24357]	chr3:45123769-45187914	NM_022842, NM_1	XM_011534024	NP_073753, NP_83548	XP_011532326	protein_coding	ENSG0000016381	64866	24357
2155	CDK8	0.693	0.004	cyclin dependent kinase 8 [Source:HGNC Symbol;Acc:HGNC:1779]	chr13:26828275-26979375	NM_001260, NM_0	XM_011534864, XM_0	NP_001251, NP_00130	XP_005266273, XP_0115	protein_coding	ENSG0000013296	1024	1779
2156	GATA2	-0.878	0.004	GATA binding protein 2 [Source:HGNC Symbol;Acc:HGNC:4171]	chr3:128198269-128212028	NM_001145661, NN	.	NP_001139133, NP_00	.	protein_coding	ENSG0000017934	2624	4171
2157	NUBP2	-0.744	0.004	nucleotide binding protein 2 [Source:HGNC Symbol;Acc:HGNC:8042]	chr16:1832901-1839192	NM_001284501, NN	XM_005255030, XM_0	NP_001271430, NP_00	XP_005255084, XP_0052	protein_coding	ENSG0000009590	10101	8042
2158	NUCKS1	0.692	0.004	nuclear casein kinase and cyclin dependent kinase substrate 1 [Source:HGNC Symbol;Ac	chr1:205681946-205719404	NM_022731	XM_005245453	NP_073568	XP_005245510	protein_coding	ENSG0000006927	64710	29923

2159	SERINC1	0.903	0.004	serine incorporator 1 [Source:HGNC Symbol;Acc:HGNC:13464]	chr6:122764498-122792967	NM_020755	.	NP_065806	.	protein_coding	ENSG0000011189	57515	13464
2160	SP5	-0.783	0.004	Sp5 transcription factor [Source:HGNC Symbol;Acc:HGNC:14529]	chr2:171571860-171574588	NM_001003845	.	NP_001003845	.	protein_coding	ENSG0000020433	389058	14529
2161	CCDC94	-0.885	0.004	coiled-coil domain containing 94 [Source:HGNC Symbol;Acc:HGNC:25518]	chr19:4247075-4269087	NM_018074	.	NP_060544	.	protein_coding	ENSG0000010524	55702	25518
2162	CDC37	-0.681	0.004	cell division cycle 37 [Source:HGNC Symbol;Acc:HGNC:1735]	chr19:10501809-10514209	NM_007065	XM_011527652	NP_008996	XP_011525954	protein_coding	ENSG0000010540	100302213	1735
2163	DDX5	0.686	0.004	DEAD-box helicase 5 [Source:HGNC Symbol;Acc:HGNC:2746]	chr17:62495733-62502639	NM_004396	XM_011524457, XM_0	NP_001307524, NP_00	XP_005257168, XP_0067	protein_coding	ENSG0000010865	100616408	2746
2164	FOLR1	-1.006	0.004	folate receptor 1 [Source:HGNC Symbol;Acc:HGNC:3791]	chr11:71900601-71907345	NM_000802, NM_0	.	NP_000793, NP_05793	.	protein_coding	ENSG0000011019	2348	3791
2165	LUZP1	0.791	0.004	leucine zipper protein 1 [Source:HGNC Symbol;Acc:HGNC:14985]	chr1:23410515-23504301	NM_001142546, NN	XM_017002252, XM_0	NP_001136018, NP_36	XP_011540392, XP_0115	protein_coding	ENSG0000016964	7798	14985
2166	RNF145	0.756	0.004	ring finger protein 145 [Source:HGNC Symbol;Acc:HGNC:20853]	chr5:158584416-158637061	NM_001199380, NN	XM_005265826	NP_001186309, NP_00	XP_016864627	protein_coding	ENSG0000014586	153830	20853
2167	ZNF462	1.052	0.004	zinc finger protein 462 [Source:HGNC Symbol;Acc:HGNC:21684]	chr9:109625377-109775915	NM_021224	XM_011518892, XM_0	NP_067047	XP_006717272, XP_0067	protein_coding	ENSG0000014814	58499	21684
2168	ERO1B	0.945	0.004	endoplasmic reticulum oxidoreductase 1 beta [Source:HGNC Symbol;Acc:HGNC:14355]	chr1:236378854-236445285	NM_019891	.	NP_063944	.	protein_coding	ENSG0000008661	56605	14355
2169	MRPL4	-0.722	0.004	mitochondrial ribosomal protein L4 [Source:HGNC Symbol;Acc:HGNC:14276]	chr19:10362639-10370721	NM_015956, NM_1	XM_011528045	NP_057040, NP_66649	XP_011526347	protein_coding	ENSG0000010536	51073	14276
2170	NIPSNAP3A	-0.989	0.004	nipsnap homolog 3A [Source:HGNC Symbol;Acc:HGNC:23619]	chr9:107509968-107522403	NM_015469	.	NP_056284	.	protein_coding	ENSG0000013678	25934	23619
2171	NOSIP	-0.723	0.004	nitric oxide synthase interacting protein [Source:HGNC Symbol;Acc:HGNC:17946]	chr19:50058967-50083829	NM_001270960, NN	XM_011527017, XM_0	NP_001257889, NP_05	XP_005259021, XP_0067	protein_coding	ENSG0000014254	51070	17946
2172	YTHDC1	0.841	0.004	YTH domain containing 1 [Source:HGNC Symbol;Acc:HGNC:30626]	chr4:69176104-69215807	NM_001031732, NN	XM_005265708, XM_0	NP_001026902, NP_58	XP_005265763, XP_0052	protein_coding	ENSG0000008389	91746	30626
2173	CYP2R1	1.001	0.004	cytochrome P450 family 2 subfamily R member 1 [Source:HGNC Symbol;Acc:HGNC:2058	chr11:14898985-14913798	NM_024514	XM_017017194, XM_0	NP_078790	XP_005252845, XP_0052	protein_coding	ENSG0000018610	120227	20580
2174	KDM7A	1.022	0.004	lysine demethylase 7A [Source:HGNC Symbol;Acc:HGNC:22224]	chr7:139784545-139876835	NM_030647	XM_011516586	NP_085150	.	protein_coding	ENSG0000000645	80853	22224
2175	MYC	-0.648	0.004	v-myc avian myelocytomatosis viral oncogene homolog [Source:HGNC Symbol;Acc:HGN	chr8:128747679-128753680	NM_002467	.	NP_002458	.	protein_coding	ENSG0000013699	4609	7553
2176	PEX3	1.010	0.004	peroxisomal biogenesis factor 3 [Source:HGNC Symbol;Acc:HGNC:8858]	chr6:143772116-143811147	NM_003630	.	NP_003621	.	protein_coding	ENSG0000003469	8504	8858
2177	PRPF40A	1.073	0.004	pre-mRNA processing factor 40 homolog A [Source:HGNC Symbol;Acc:HGNC:16463]	chr2:153508106-153617767	NM_017892	XM_011511449, XM_0	NP_060362	XP_005246733, XP_0052	protein_coding	ENSG0000019650	55660	16463
2178	RHOBTB3	1.151	0.004	Rho related BTB domain containing 3 [Source:HGNC Symbol;Acc:HGNC:18757]	chr5:95066849-95158709	NM_014899	XM_011543279	NP_055714	XP_011541581	protein_coding	ENSG0000016429	22836	18757
2179	TEX2	0.771	0.004	testis expressed 2 [Source:HGNC Symbol;Acc:HGNC:30884]	chr17:62224586-62340661	NM_001288732, NN	XM_017024846, XM_0	NP_001275661, NP_00	XP_011523300, XP_0115	protein_coding	ENSG0000013647	55852	30884
2180	PNPLA6	-0.787	0.004	patatin like phospholipase domain containing 6 [Source:HGNC Symbol;Acc:HGNC:16268	chr19:7587511-7626650	NM_001166111, NN	.	NP_001159583, NP_00	.	protein_coding	ENSG0000003244	10908	16268
2181	RAB6A	0.698	0.004	RAB6A, member RAS oncogene family [Source:HGNC Symbol;Acc:HGNC:9786]	chr11:73386682-73472182	NM_001243718, NN	.	NP_001230647, NP_00	.	protein_coding	ENSG0000017558	5870	9786
2182	TBC1D10A	-0.861	0.004	TBC1 domain family member 10A [Source:HGNC Symbol;Acc:HGNC:23609]	chr22:30687978-30723035	NM_001204240, NN	.	NP_001191169, NP_11	.	protein_coding	ENSG0000009999	83874	23609
2183	UFC1	0.774	0.004	ubiquitin-fold modifier conjugating enzyme 1 [Source:HGNC Symbol;Acc:HGNC:26941]	chr1:161123541-161128646	NM_016406	.	NP_057490	XP_016856939, XP_0168	protein_coding	ENSG0000014322	51506	26941
2184	PHC2	-0.714	0.004	polyhomeotic homolog 2 [Source:HGNC Symbol;Acc:HGNC:3183]	chr1:33789223-33841194	NM_004427, NM_1	XM_017000520, XM_0	NP_004418, NP_93215	XP_005270627, XP_0052	protein_coding	ENSG0000013468	100500853	3183
2185	SRP72	0.990	0.004	signal recognition particle 72 [Source:HGNC Symbol;Acc:HGNC:11303]	chr4:57333080-57369273	NM_001267722, NN	.	NP_001254651, NP_00	.	protein_coding	ENSG0000017478	6731	11303
2186	GPR19	1.559	0.004	G protein-coupled receptor 19 [Source:HGNC Symbol;Acc:HGNC:4473]	chr12:12813824-12849121	NM_006143	XM_011520625	NP_006134	XP_011518925, XP_0115	protein_coding	ENSG0000018315	2842	4473
2187	HIPK1	0.819	0.004	homeodomain interacting protein kinase 1 [Source:HGNC Symbol;Acc:HGNC:19006]	chr1:114471813-114520491	NM_152696, NM_1	XM_017000605, XM_0	NP_689909, NP_85200	XP_005270666, XP_0052	protein_coding	ENSG0000016334	204851	19006
2188	RABGAP1	0.824	0.004	RAB GTPase activating protein 1 [Source:HGNC Symbol;Acc:HGNC:17155]	chr9:125703287-125867145	NM_005294, NM_0	XM_017014569, XM_0	NP_005285, NP_03632	XP_005251990, XP_0115	protein_coding	ENSG0000001145	23637	17155
2189	STRN	0.956	0.004	striatin [Source:HGNC Symbol;Acc:HGNC:11424]	chr2:37070782-37193615	NM_003162	XM_011533073, XM_0	NP_003153	XP_005264576, XP_0115	protein_coding	ENSG0000011580	6801	11424
2190	TSNARE1	-0.971	0.004	t-SNARE domain containing 1 [Source:HGNC Symbol;Acc:HGNC:26437]	chr8:143293440-143484601	NM_001291931, NN	XM_017013178, XM_0	NP_001278860, NP_65	XP_011515223, XP_0168	protein_coding	ENSG0000017104	203062	26437
2191	ZC3H12A	-0.816	0.004	zinc finger CCCH-type containing 12A [Source:HGNC Symbol;Acc:HGNC:26259]	chr1:37940152-37949980	NM_001323550, NN	XM_011542196, XM_0	NP_001310479, NP_07	.	protein_coding	ENSG0000016387	102465438	26259
2192	C19orf81	-1.399	0.004	chromosome 19 open reading frame 81 [Source:HGNC Symbol;Acc:HGNC:40041]	chr19:51152701-51162567	NM_001195076	XM_011526911, XM_0	NP_001182005	.	protein_coding	ENSG0000023503	342918	40041
2193	MNT	-0.845	0.004	MAX network transcriptional repressor [Source:HGNC Symbol;Acc:HGNC:7188]	chr17:2287353-2304412	NM_020310	XM_011523868	NP_064706	XP_011522170	protein_coding	ENSG0000007044	4335	7188
2194	MTF2	1.080	0.004	metal response element binding transcription factor 2 [Source:HGNC Symbol;Acc:HGNC	chr1:93544791-93604638	NM_001164391, NN	XM_017000676, XM_0	NP_001157863, NP_00	XP_011539318, XP_0115	protein_coding	ENSG0000014303	22823	29535
2195	NTRK2	1.017	0.004	neurotrophic receptor tyrosine kinase 2 [Source:HGNC Symbol;Acc:HGNC:8032]	chr9:87283465-87641985	NM_001007097, NN	XM_017014761, XM_0	NP_001007098, NP_00	XP_005252058, XP_0052	protein_coding	ENSG0000014805	4915	8032
2196	SLTM	0.869	0.004	SAFB like transcription modulator [Source:HGNC Symbol;Acc:HGNC:20709]	chr15:59171243-59225852	NM_001013843, NN	XM_017022579, XM_0	NP_001013865, NP_07	XP_006720749, XP_0067	protein_coding	ENSG0000013777	79811	20709
2197	TSPAN6	0.886	0.004	tetraspanin 6 [Source:HGNC Symbol;Acc:HGNC:11858]	chrX:99882105-99892101	NM_001278740, NN	XM_011531018, XM_0	NP_001265669, NP_00	XP_011529320	protein_coding	ENSG0000000000	7105	11858
2198	VPS8	1.016	0.004	VPS8, CORVET complex subunit [Source:HGNC Symbol;Acc:HGNC:29122]	chr3:184529930-184770402	NM_001009921, NN	XM_005247251	NP_001009921, NP_05	XP_005247308	protein_coding	ENSG0000015693	23355	29122
2199	HIP1R	-0.805	0.004	huntingtin interacting protein 1 related [Source:HGNC Symbol;Acc:HGNC:18415]	chr12:123319972-123347507	NM_003959	XM_011538963	NP_003950	XP_011537265	protein_coding	ENSG0000013078	9026	18415
2200	IPP	1.048	0.004	intracisternal A particle-promoted polypeptide [Source:HGNC Symbol;Acc:HGNC:6108]	chr1:46164382-46216321	NM_001145349, NN	XM_006710622	NP_001138821, NP_00	.	protein_coding	ENSG0000019742	3652	6108
2201	PAPD4	1.076	0.004	poly(A) RNA polymerase D4, non-canonical [Source:HGNC Symbol;Acc:HGNC:26776]	chr5:78908242-78982471	NM_001114393, NN	XM_017009157, XM_0	NP_001107865, NP_00	XP_011541521, XP_0115	protein_coding	ENSG0000016432	167153	26776
2202	SRC	-0.811	0.004	SRC proto-oncogene, non-receptor tyrosine kinase [Source:HGNC Symbol;Acc:HGNC:11	chr20:35974556-36034453	NM_005417, NM_1	XM_011529014, XM_0	NP_005408, NP_93803	XP_011527315, XP_0115	protein_coding	ENSG0000019712	6714	11283
2203	SSR1	0.929	0.004	signal sequence receptor subunit 1 [Source:HGNC Symbol;Acc:HGNC:11323]	chr6:7268538-7313440	NM_001292008, NN	.	NP_001278937, NP_00	.	protein_coding	ENSG0000012478	6745	11323
2204	UBXN1	-0.673	0.004	UBX domain protein 1 [Source:HGNC Symbol;Acc:HGNC:18402]	chr11:62443969-62446589	NM_001286077, NN	XM_005274033	NP_001273006, NP_00	XP_005274090, XP_0168	protein_coding	ENSG0000016219	51035	18402
2205	ZNF275	0.853	0.004	zinc finger protein 275 [Source:HGNC Symbol;Acc:HGNC:13069]	chrX:152599612-152618384	NM_001080485	XM_005274645, XM_0	NP_001073954	XP_005274702	protein_coding	ENSG0000006358	105373378	13069
2206	BDH1	-0.779	0.004	3-hydroxybutyrate dehydrogenase, type 1 [Source:HGNC Symbol;Acc:HGNC:1027]	chr3:197236653-197300194	NM_004051, NM_2	XM_011513068, XM_0	NP_004042, NP_97605	XP_005269409, XP_0115	protein_coding	ENSG0000016126	622	1027
2207	CCS	-0.844	0.004	copper chaperone for superoxide dismutase [Source:HGNC Symbol;Acc:HGNC:1613]	chr11:66357646-66373490	NM_005125	.	NP_005116	.	protein_coding	ENSG0000017399	9973	1613
2208	EIF2AK3	0.926	0.004	eukaryotic translation initiation factor 2 alpha kinase 3 [Source:HGNC Symbol;Acc:HGNC	chr2:88856258-88927094	NM_001313915, NN	XM_005264649	NP_001300844, NP_00	XP_016860865	protein_coding	ENSG0000017207	9451	3255
2209	FAM102B	1.086	0.004	family with sequence similarity 102 member B [Source:HGNC Symbol;Acc:HGNC:27637]	chr1:109102710-109187522	NM_001010883	.	NP_001010883	.	protein_coding	ENSG0000016263	284611	27637
2210	FAM104B	-1.059	0.004	family with sequence similarity 104 member B [Source:HGNC Symbol;Acc:HGNC:25085]	chrX:55169534-55187743	NM_001166699, NN	XM_006724612	NP_001160171, NP_00	XP_016885442	protein_coding	ENSG0000018251	90736	25085
2211	GABRD	-1.594	0.004	gamma-aminobutyric acid type A receptor delta subunit [Source:HGNC Symbol;Acc:HGN	chr1:1950762-1962190	NM_000815	.	NP_000806	XP_011539496	protein_coding	ENSG0000018773	2563	4084
2212	HKDC1	-1.081	0.004	hexokinase domain containing 1 [Source:HGNC Symbol;Acc:HGNC:23302]	chr10:70980058-71027315	NM_025130	.	NP_079406	.	protein_coding	ENSG0000015651	80201	23302

2213	PRR9	-5.607	0.004	proline rich 9 [Source:HGNC Symbol;Acc:HGNC:32057]	chr1:153190059-153191793	NM_001195571	XM_011509807	NP_001182500	XP_011508109	protein_coding	ENSG0000020378	574414	32057
2214	ZNF385A	-0.699	0.004	zinc finger protein 385A [Source:HGNC Symbol;Acc:HGNC:17521]	chr12:54762916-54785082	NM_001130967, NM	XM_011538171, XM_0	NP_001124439, NP_00	XP_005268840, XP_0067	protein_coding	ENSG0000016164	25946	17521
2215	KLHL11	1.179	0.005	kelch like family member 11 [Source:HGNC Symbol;Acc:HGNC:19008]	chr17:40009796-40021684	NM_018143	.	NP_060613	.	protein_coding	ENSG0000017850	55175	19008
2216	PGM3	1.210	0.005	phosphoglucomutase 3 [Source:HGNC Symbol;Acc:HGNC:8907]	chr6:83777384-83908651	NM_001199917, NM	XM_011535903, XM_0	NP_001186846, NP_00	XP_011534205, XP_0168	protein_coding	ENSG0000001337	5238	8907
2217	TCIRG1	-0.768	0.005	T-cell immune regulator 1, ATPase H+ transporting V0 subunit a3 [Source:HGNC Symbol	chr11:67806482-67818362	NM_006019, NM_0	XM_011544727, XM_0	NP_006010, NP_00604	XP_005273766, XP_0115	protein_coding	ENSG0000011071	10312	11647
2218	ERLIN2	0.874	0.005	ER lipid raft associated 2 [Source:HGNC Symbol;Acc:HGNC:1356]	chr8:37594116-37616619	NM_001003790, NM	XM_006716280, XM_0	NP_001003790, NP_00	XP_005273449, XP_0067	protein_coding	ENSG0000014747	11160	1356
2219	KCNQ1	-1.043	0.005	potassium voltage-gated channel subfamily Q member 1 [Source:HGNC Symbol;Acc:HGI	chr11:2466220-2870339	NM_000218, NM_1	.	NP_000209, NP_86146	.	protein_coding	ENSG0000005391	3784	6294
2220	PSMD10	0.862	0.005	proteasome 26S subunit, non-ATPase 10 [Source:HGNC Symbol;Acc:HGNC:9555]	chrX:107327436-107334848	NM_002814, NM_1	.	NP_002805, NP_73660	.	protein_coding	ENSG0000010184	5716	9555
2221	RUFY2	1.105	0.005	RUN and FYVE domain containing 2 [Source:HGNC Symbol;Acc:HGNC:19761]	chr10:70103275-70167051	NM_001042417, NM	XM_011539943, XM_0	NP_001035882, NP_00	XP_005270010, XP_0052	protein_coding	ENSG0000020413	55680	19761
2222	KCNMA1	-1.966	0.005	potassium calcium-activated channel subfamily M alpha 1 [Source:HGNC Symbol;Acc:HC	chr10:78637354-79398353	NM_001014797, NM	XM_017016218, XM_0	NP_001014797, NP_00	XP_005269833, XP_0052	protein_coding	ENSG0000015611	3778	6284
2223	TMEM176A	-2.148	0.005	transmembrane protein 176A [Source:HGNC Symbol;Acc:HGNC:24930]	chr7:150488372-150502208	NM_018487	XM_017012393, XM_0	NP_060957	XP_011514678, XP_0115	protein_coding	ENSG0000000293	55365	24930
2224	ABCB4	1.106	0.005	ATP binding cassette subfamily B member 4 [Source:HGNC Symbol;Acc:HGNC:45]	chr7:87031360-87105055	NM_000443, NM_0	XM_011516314, XM_0	NP_000434, NP_06133	XP_011514610, XP_0115	protein_coding	ENSG0000000547	5244	45
2225	OGFR	-0.688	0.005	opioid growth factor receptor [Source:HGNC Symbol;Acc:HGNC:15768]	chr20:61436186-61445352	NM_007346	.	NP_031372	.	protein_coding	ENSG0000006049	11054	15768
2226	PFKL	-0.673	0.005	phosphofructokinase, liver type [Source:HGNC Symbol;Acc:HGNC:8876]	chr21:45719933-45747259	NM_001002021, NM	XM_017028371, XM_0	NP_001002021, NP_00	XP_005261192, XP_0052	protein_coding	ENSG0000014195	5211	8876
2227	PLA2G4C	-1.389	0.005	phospholipase A2 group IVC [Source:HGNC Symbol;Acc:HGNC:9037]	chr19:48551099-48614074	NM_001159322, NM	XM_011527431	NP_001152794, NP_00	XP_011525733, XP_0168	protein_coding	ENSG0000010549	8605	9037
2228	SLC25A38	-0.781	0.005	solute carrier family 25 member 38 [Source:HGNC Symbol;Acc:HGNC:26054]	chr3:39424838-39438842	NM_017875	XM_011533871, XM_0	NP_060345	XP_006713277, XP_0115	protein_coding	ENSG0000014465	54977	26054
2229	SLC30A9	0.891	0.005	solute carrier family 30 member 9 [Source:HGNC Symbol;Acc:HGNC:1329]	chr4:41992488-42092474	NM_006345	.	NP_006336	XP_016863143	protein_coding	ENSG0000001482	10463	1329
2230	SRD5A3	0.972	0.005	steroid 5 alpha-reductase 3 [Source:HGNC Symbol;Acc:HGNC:25812]	chr4:56212275-56239263	NM_024592	XM_005265767	NP_078868	XP_005265824	protein_coding	ENSG0000012803	79644	25812
2231	APOD	-1.911	0.005	apolipoprotein D [Source:HGNC Symbol;Acc:HGNC:612]	chr3:195295572-195311076	NM_001647	.	NP_001638	.	protein_coding	ENSG0000018905	347	612
2232	BAD	-0.870	0.005	BCL2 associated agonist of cell death [Source:HGNC Symbol;Acc:HGNC:936]	chr11:64037301-64056972	NM_004322, NM_0	.	NP_004313, NP_11678	.	protein_coding	ENSG0000000233	572	936
2233	ESM1	-1.416	0.005	endothelial cell specific molecule 1 [Source:HGNC Symbol;Acc:HGNC:3466]	chr5:54273691-54281491	NM_001135604, NM	.	NP_001129076, NP_00	.	protein_coding	ENSG0000016428	11082	3466
2234	GTF2A1	1.063	0.005	general transcription factor IIA subunit 1 [Source:HGNC Symbol;Acc:HGNC:4646]	chr14:81641795-81687575	NM_001278940, NM	.	NP_001265869, NP_05	.	protein_coding	ENSG0000016541	2957	4646
2235	MST1R	-1.004	0.005	macrophage stimulating 1 receptor [Source:HGNC Symbol;Acc:HGNC:7381]	chr3:49924434-49941306	NM_001244937, NM	XM_011533745, XM_0	NP_001231866, NP_00	XP_005265227, XP_0115	protein_coding	ENSG0000016407	4486	7381
2236	SPCS3	0.786	0.005	signal peptidase complex subunit 3 [Source:HGNC Symbol;Acc:HGNC:26212]	chr4:177241114-177253396	NM_021928	.	NP_068747	.	protein_coding	ENSG0000012912	60559	26212
2237	TMED10	0.669	0.005	transmembrane p24 trafficking protein 10 [Source:HGNC Symbol;Acc:HGNC:16998]	chr14:75598172-75643334	NM_006827	.	NP_006818	.	protein_coding	ENSG0000017034	10972	16998
2238	CCDC110	1.545	0.005	coiled-coil domain containing 110 [Source:HGNC Symbol;Acc:HGNC:28504]	chr4:186350543-186392913	NM_001145411, NM	XM_017007988, XM_0	NP_001138883, NP_68	XP_005262948, XP_0067	protein_coding	ENSG0000016849	256309	28504
2239	CDC34	-0.698	0.005	cell division cycle 34 [Source:HGNC Symbol;Acc:HGNC:1734]	chr19:531711-542092	NM_004359	XM_006722952	NP_004350	XP_006723015	protein_coding	ENSG0000009980	997	1734
2240	FAT1	0.714	0.005	FAT atypical cadherin 1 [Source:HGNC Symbol;Acc:HGNC:3595]	chr4:187508936-187645009	NM_005245	XM_006714139, XM_0	NP_005236	XP_005262891, XP_0052	protein_coding	ENSG0000008385	2195	3595
2241	GPR173	-1.093	0.005	G protein-coupled receptor 173 [Source:HGNC Symbol;Acc:HGNC:18186]	chrX:53078272-53109797	NM_018969	XM_011530798, XM_0	NP_061842	XP_011529099, XP_0115	protein_coding	ENSG0000018419	54328	18186
2242	PHLDA3	-0.872	0.005	pleckstrin homology like domain family A member 3 [Source:HGNC Symbol;Acc:HGNC:8	chr1:201433510-201438365	NM_012396	.	NP_036528	.	protein_coding	ENSG0000017430	23612	8934
2243	POU4F1	1.236	0.005	POU class 4 homeobox 1 [Source:HGNC Symbol;Acc:HGNC:9218]	chr13:79172496-79177695	NM_006237	.	NP_006228	.	protein_coding	ENSG0000015219	5457	9218
2244	VASH2	1.756	0.005	vasohibin 2 [Source:HGNC Symbol;Acc:HGNC:25723]	chr1:213123861-213164927	NM_001136474, NM	XM_011509993, XM_0	NP_001129946, NP_00	XP_011508289, XP_0115	protein_coding	ENSG0000014349	79805	25723
2245	ZNF569	1.367	0.005	zinc finger protein 569 [Source:HGNC Symbol;Acc:HGNC:24737]	chr19:37902056-37958339	NM_152484	XM_017026377, XM_0	NP_689697	XP_005258621, XP_0067	protein_coding	ENSG0000019643	148266	24737
2246	RNMT	0.869	0.005	RNA guanine-7 methyltransferase [Source:HGNC Symbol;Acc:HGNC:10075]	chr18:13726658-13764557	NM_001308263, NM	XM_017026061, XM_0	NP_001295192, NP_00	XP_005258219, XP_0067	protein_coding	ENSG0000010165	8731	10075
2247	SFN	-0.712	0.005	stratifin [Source:HGNC Symbol;Acc:HGNC:10773]	chr1:27189628-27190948	NM_006142	.	NP_006133	.	protein_coding	ENSG0000017579	2810	10773
2248	UROD	-0.685	0.005	uroporphyrinogen decarboxylase [Source:HGNC Symbol;Acc:HGNC:12591]	chr1:45477818-45481247	NM_000374	XM_011542080, XM_0	NP_000365	XP_005271226, XP_0052	protein_coding	ENSG0000012608	7389	12591
2249	USP14	0.899	0.005	ubiquitin specific peptidase 14 [Source:HGNC Symbol;Acc:HGNC:12612]	chr18:158382-268027	NM_001037334, NM	.	NP_001032411, NP_00	.	protein_coding	ENSG0000010155	9097	12612
2250	ATP2A3	-0.886	0.005	ATPase sarcoplasmic/endoplasmic reticulum Ca2+ transporting 3 [Source:HGNC Symbol	chr17:3827163-3867758	NM_005173, NM_1	XM_011523891, XM_0	NP_005164, NP_77761	XP_011522183, XP_0115	protein_coding	ENSG0000007437	489	813
2251	COMTD1	-0.857	0.005	catechol-O-methyltransferase domain containing 1 [Source:HGNC Symbol;Acc:HGNC:26	chr10:76993726-76995769	NM_144589	.	NP_653190	.	protein_coding	ENSG0000016564	118881	26309
2252	INTS6	0.968	0.005	integrator complex subunit 6 [Source:HGNC Symbol;Acc:HGNC:14879]	chr13:51909908-52027343	NM_001039937, NM	XM_011535041, XM_0	NP_001035026, NP_00	XP_005266397, XP_0115	protein_coding	ENSG0000010278	26512	14879
2253	PIK3IP1	-1.151	0.005	phosphoinositide-3-kinase interacting protein 1 [Source:HGNC Symbol;Acc:HGNC:24942	chr22:31677578-31688520	NM_001135911, NM	.	NP_001129383, NP_44	.	protein_coding	ENSG0000010010	113791	24942
2254	PPP2R1A	-0.670	0.005	protein phosphatase 2 scaffold subunit Aalpha [Source:HGNC Symbol;Acc:HGNC:9302]	chr19:52693291-52732786	NM_014225	XM_017026929	NP_055040	XP_016882418	protein_coding	ENSG0000010556	5518	9302
2255	RPLP1	-0.870	0.005	ribosomal protein lateral stalk subunit P1 [Source:HGNC Symbol;Acc:HGNC:10372]	chr15:69745122-69748533	NM_001003, NM_2	.	NP_000994, NP_99889	.	protein_coding	ENSG0000013781	6176	10372
2256	ZNF184	1.122	0.005	zinc finger protein 184 [Source:HGNC Symbol;Acc:HGNC:12975]	chr6:27418521-27440897	NM_001318892, NM	XM_011514861, XM_0	NP_001305820, NP_00	XP_005249436, XP_0052	protein_coding	ENSG0000009665	7738	12975
2257	ACAP1	-1.658	0.005	ArfGAP with coiled-coil, ankyrin repeat and PH domains 1 [Source:HGNC Symbol;Acc:HC	chr17:7239847-7254797	NM_014716	XM_005256878	NP_055531	.	protein_coding	ENSG0000007281	9744	16467
2258	CHRNB4	-1.382	0.005	cholinergic receptor nicotinic beta 4 subunit [Source:HGNC Symbol;Acc:HGNC:1964]	chr15:78916460-78933587	NM_000750, NM_0	.	NP_000741, NP_00124	XP_011519488, XP_0115	protein_coding	ENSG0000011797	1143	1964
2259	ECD	0.876	0.005	ecdysoneless cell cycle regulator [Source:HGNC Symbol;Acc:HGNC:17029]	chr10:74893413-74927829	NM_001135752, NM	.	NP_001129224, NP_00	.	protein_coding	ENSG0000012288	11319	17029
2260	GGT1	-1.871	0.005	gamma-glutamyltransferase 1 [Source:HGNC Symbol;Acc:HGNC:4250]	chr22:24979717-25024972	NM_001288833, NM	XM_005261815	NP_001275762, NP_03	XP_005261872, XP_0067	protein_coding	ENSG0000010003	102724197	4250
2261	GPR3	-1.036	0.005	G protein-coupled receptor 3 [Source:HGNC Symbol;Acc:HGNC:4484]	chr1:27719147-27722318	NM_005281	.	NP_005272	.	protein_coding	ENSG0000018177	2827	4484
2262	HMG20A	0.829	0.005	high mobility group 20A [Source:HGNC Symbol;Acc:HGNC:5001]	chr15:77712992-77777949	NM_001304504, NM	XM_011521158	NP_001291433, NP_00	XP_011519460	protein_coding	ENSG0000014038	10363	5001
2263	PDE5A	1.290	0.005	phosphodiesterase 5A [Source:HGNC Symbol;Acc:HGNC:8784]	chr4:120415549-120550146	NM_001083, NM_0	.	NP_001074, NP_23691	.	protein_coding	ENSG0000013873	8654	8784
2264	PIK3CA	0.981	0.005	phosphatidylinositol-4,5-bisphosphate 3-kinase catalytic subunit alpha [Source:HGNC Sy	chr3:178866310-178984790	NM_006218	XM_011512894, XM_0	NP_006209	XP_006713721, XP_0115	protein_coding	ENSG0000012187	5290	8975
2265	PSG1	-0.997	0.005	pregnancy specific beta-1-glycoprotein 1 [Source:HGNC Symbol;Acc:HGNC:9514]	chr19:43370615-43383871	NM_001184825, NM	XM_017026994	NP_001171754, NP_00	XP_005259122, XP_0168	protein_coding	ENSG0000023192	5669	9514
2266	TGFBR3	1.224	0.005	transforming growth factor beta receptor 3 [Source:HGNC Symbol;Acc:HGNC:11774]	chr1:92145901-92371892	NM_001195683, NM	XM_006710868, XM_0	NP_001182612, NP_00	XP_006710930, XP_0067	protein_coding	ENSG0000006970	7049	11774

2267	AGPAT5	0.901	0.005	1-acylglycerol-3-phosphate O-acyltransferase 5 [Source:HGNC Symbol;Acc:HGNC:20886	chr8:6565877-6617187	NM_018361	.	NP_060831	.	protein_coding	ENSG0000015518	55326	20886
2268	ASXL2	0.798	0.005	additional sex combs like 2, transcriptional regulator [Source:HGNC Symbol;Acc:HGNC:2	chr2:25956621-26101385	NM_018263	XM_017004429, XM_0	NP_060733	XP_006712102, XP_0067	protein_coding	ENSG0000014397	55252	23805
2269	PLEKHH2	1.925	0.005	pleckstrin homology, MyTH4 and FERM domain containing H2 [Source:HGNC Symbol;Ac	chr2:43864411-43995126	NM_172069	XM_017003351	NP_742066	XP_016858840	protein_coding	ENSG0000015252	130271	30506
2270	SHKBP1	-0.664	0.005	SH3KBP1 binding protein 1 [Source:HGNC Symbol;Acc:HGNC:19214]	chr19:41082756-41097303	NM_138392	XM_017027473, XM_0	NP_612401	XP_006723537, XP_0067	protein_coding	ENSG0000016041	92799	19214
2271	DEPDC1B	0.959	0.005	DEP domain containing 1B [Source:HGNC Symbol;Acc:HGNC:24902]	chr5:59892738-59995988	NM_001145208, NM	XM_011543509	NP_001138680, NP_06	XP_011541811	protein_coding	ENSG0000003549	55789	24902
2272	EPS15	0.908	0.005	epidermal growth factor receptor pathway substrate 15 [Source:HGNC Symbol;Acc:HGN	chr1:51819934-51985000	NM_001981	XM_005270618	NP_001972	XP_005270675	protein_coding	ENSG0000008583	2060	3419
2273	NAA35	0.900	0.005	N(alpha)-acetyltransferase 35, NatC auxiliary subunit [Source:HGNC Symbol;Acc:HGNC:2	chr9:88556060-88637213	NM_001321881, NM	XM_011518902, XM_0	NP_001308810, NP_00	XP_005252183, XP_0052	protein_coding	ENSG0000013504	60560	24340
2274	PRR14L	0.914	0.005	proline rich 14 like [Source:HGNC Symbol;Acc:HGNC:28738]	chr22:32077332-32146117	NM_173566	XM_011530081, XM_0	NP_775837	XP_006724282, XP_0067	protein_coding	ENSG0000018353	253143	28738
2275	CD40	-1.294	0.005	CD40 molecule [Source:HGNC Symbol;Acc:HGNC:11919]	chr20:44746910-44758502	NM_001250, NM_0	XM_005260619, XM_0	NP_001241, NP_00128	XP_005260676, XP_0115	protein_coding	ENSG0000010101	958	11919
2276	MRPL28	-0.686	0.005	mitochondrial ribosomal protein L28 [Source:HGNC Symbol;Acc:HGNC:14484]	chr16:417383-420527	NM_006428	XM_011522351, XM_0	NP_006419	XP_005255098, XP_0115	protein_coding	ENSG0000008650	10573	14484
2277	RBM39	0.771	0.005	RNA binding motif protein 39 [Source:HGNC Symbol;Acc:HGNC:15923]	chr20:34291531-34330234	NM_001242599, NM	XM_011529111, XM_0	NP_001229528, NP_00	XP_006723953, XP_0067	protein_coding	ENSG0000013105	9584	15923
2278	VEZF1	0.822	0.005	vascular endothelial zinc finger 1 [Source:HGNC Symbol;Acc:HGNC:12949]	chr17:56051119-56065513	NM_007146	XM_005257644, XM_0	NP_009077	XP_005257700, XP_0052	protein_coding	ENSG0000013645	7716	12949
2279	FAM49B	0.917	0.005	family with sequence similarity 49 member B [Source:HGNC Symbol;Acc:HGNC:25216]	chr8:130851838-131028918	NM_001256763, NM	XM_017013551, XM_0	NP_001243692, NP_05	XP_005251023, XP_0115	protein_coding	ENSG0000015331	51571	25216
2280	GTF2E2	0.736	0.005	general transcription factor IIE subunit 2 [Source:HGNC Symbol;Acc:HGNC:4651]	chr8:30435834-30515768	NM_002095	.	NP_002086	XP_016868852, XP_0168	protein_coding	ENSG0000019726	2961	4651
2281	SLC39A10	1.018	0.005	solute carrier family 39 member 10 [Source:HGNC Symbol;Acc:HGNC:20861]	chr2:196521470-196602426	NM_001127257, NM	XM_011511506, XM_0	NP_001120729, NP_06	XP_005246746, XP_0115	protein_coding	ENSG0000019695	57181	20861
2282	TIMM21	0.983	0.005	translocase of inner mitochondrial membrane 21 [Source:HGNC Symbol;Acc:HGNC:2501	chr18:71815745-71827765	NM_014177	XM_005266689	NP_054896	XP_005266746	protein_coding	ENSG0000007533	29090	25010
2283	C2	-3.488	0.005	complement component 2 [Source:HGNC Symbol;Acc:HGNC:1248]	chr6:31865561-31919861	NM_000063, NM_0	.	NP_000054, NP_00113	.	protein_coding	ENSG0000016627	717	1248
2284	FCHO2	1.167	0.005	FCH domain only 2 [Source:HGNC Symbol;Acc:HGNC:25180]	chr5:72251807-72386349	NM_001146032, NM	.	NP_001139504, NP_62	.	protein_coding	ENSG0000015710	115548	25180
2285	MTHFR	-0.908	0.005	methylenetetrahydrofolate reductase (NAD(P)H) [Source:HGNC Symbol;Acc:HGNC:7436	chr1:11821843-11866115	NM_005957	XM_011541496, XM_0	NP_005948	XP_005263515, XP_0052	protein_coding	ENSG0000017700	4524	7436
2286	MVD	-0.723	0.005	mevalonate diphosphate decarboxylase [Source:HGNC Symbol;Acc:HGNC:7529]	chr16:88718342-88729518	NM_002461	XM_011523088, XM_0	NP_002452	XP_011521388, XP_0115	protein_coding	ENSG0000016750	4597	7529
2287	NIN	0.886	0.005	ninein [Source:HGNC Symbol;Acc:HGNC:14906]	chr14:51186480-51297839	NM_016350, NM_0	XM_011536824, XM_0	NP_057434, NP_06597	XP_011535119, XP_0115	protein_coding	ENSG0000010050	51199	14906
2288	PBX4	-0.975	0.005	PBX homeobox 4 [Source:HGNC Symbol;Acc:HGNC:13403]	chr19:19672521-19729725	NM_025245	XM_011528322, XM_0	NP_079521	XP_011526622, XP_0115	protein_coding	ENSG0000010571	80714	13403
2289	PTPN4	0.993	0.005	protein tyrosine phosphatase, non-receptor type 4 [Source:HGNC Symbol;Acc:HGNC:96	chr2:120517206-120741394	NM_002830	XM_017004600, XM_0	NP_002821	XP_011509862, XP_0115	protein_coding	ENSG0000008817	5775	9656
2290	WDR26	0.786	0.005	WD repeat domain 26 [Source:HGNC Symbol;Acc:HGNC:21208]	chr1:224572844-224622001	NM_001115113, NM	XM_011544281, XM_0	NP_001108585, NP_07	XP_005273330, XP_0067	protein_coding	ENSG0000016292	80232	21208
2291	MAT2A	0.719	0.005	methionine adenosyltransferase 2A [Source:HGNC Symbol;Acc:HGNC:6904]	chr2:85766287-85788655	NM_005911	.	NP_005902	.	protein_coding	ENSG0000016890	4144	6904
2292	OGFRL1	0.995	0.005	opioid growth factor receptor like 1 [Source:HGNC Symbol;Acc:HGNC:21378]	chr6:71998505-72018653	NM_001324266, NM	XM_017011305, XM_0	NP_001311195, NP_07	XP_011534419, XP_0115	protein_coding	ENSG0000011990	79627	21378
2293	OMA1	1.195	0.005	OMA1 zinc metallopeptidase [Source:HGNC Symbol;Acc:HGNC:29661]	chr1:58946390-59012472	NM_145243	.	NP_660286	.	protein_coding	ENSG0000016260	115209	29661
2294	PRKCH	0.973	0.005	protein kinase C eta [Source:HGNC Symbol;Acc:HGNC:9403]	chr14:61788434-62124682	NM_006255	XM_011536955, XM_0	NP_006246	XP_011535256, XP_0115	protein_coding	ENSG0000002707	5583	9403
2295	AP1G1	0.992	0.005	adaptor related protein complex 1 gamma 1 subunit [Source:HGNC Symbol;Acc:HGNC:5	chr16:71762902-71843104	NM_001030007, NM	.	NP_001025178, NP_00	.	protein_coding	ENSG0000016674	164	555
2296	GDPD5	-0.883	0.005	glycerophosphodiester phosphodiesterase domain containing 5 [Source:HGNC Symbol;/	chr11:75145684-75236948	NM_030792	XM_011545287, XM_0	NP_110419	XP_006718760, XP_0115	protein_coding	ENSG0000015855	81544	28804
2297	INAFM1	-0.928	0.005	InaF motif containing 1 [Source:HGNC Symbol;Acc:HGNC:27406]	chr19:47777709-47778964	NM_178511	.	NP_848606	.	protein_coding	ENSG0000025770	255783	27406
2298	LGALS7B	-3.089	0.005	galectin 7B [Source:HGNC Symbol;Acc:HGNC:34447]	chr19:39279850-39282389	NM_001042507	.	NP_001035972	.	protein_coding	ENSG0000017893	653499	34447
2299	NDUFA11	-0.725	0.005	NADH:ubiquinone oxidoreductase subunit A11 [Source:HGNC Symbol;Acc:HGNC:20371]	chr19:5865836-5903797	NM_001193375, NM	.	NP_001180304, NP_78	.	protein_coding	ENSG0000017488	126328	20371
2300	PRRG4	0.931	0.005	proline rich and Gla domain 4 [Source:HGNC Symbol;Acc:HGNC:30799]	chr11:32851488-32879669	NM_024081	XM_017018275, XM_0	NP_076986	XP_006718377, XP_0168	protein_coding	ENSG0000013537	79056	30799
2301	RBL1	0.928	0.005	RB transcriptional corepressor like 1 [Source:HGNC Symbol;Acc:HGNC:9893]	chr20:35624751-35724398	NM_002895, NM_1	XM_011528959, XM_0	NP_002886, NP_89966	XP_006723905, XP_0115	protein_coding	ENSG0000008083	5933	9893
2302	SLC27A4	-0.838	0.005	solute carrier family 27 member 4 [Source:HGNC Symbol;Acc:HGNC:10998]	chr9:131102924-131123749	NM_005094	XM_017014222	NP_005085	XP_016869711	protein_coding	ENSG0000016711	10999	10998
2303	ACADS	-0.923	0.005	acyl-CoA dehydrogenase, C-2 to C-3 short chain [Source:HGNC Symbol;Acc:HGNC:90]	chr12:121163537-121177811	NM_000017, NM_0	.	NP_000008, NP_00128	.	protein_coding	ENSG0000012297	35	90
2304	NDRG4	-0.964	0.005	NDRG family member 4 [Source:HGNC Symbol;Acc:HGNC:14466]	chr16:58497568-58547532	NM_001130487, NM	XM_017023589, XM_0	NP_001123959, NP_00	XP_006721315, XP_0067	protein_coding	ENSG0000010303	65009	14466
2305	PPFIA1	0.738	0.005	PTPRF interacting protein alpha 1 [Source:HGNC Symbol;Acc:HGNC:9245]	chr11:70116805-70230506	NM_003626, NM_1	XM_017018449, XM_0	NP_003617, NP_80317	XP_006718779, XP_0115	protein_coding	ENSG0000013162	8500	9245
2306	SPANXB1	-1.169	0.005	SPANX family member B1 [Source:HGNC Symbol;Acc:HGNC:14329]	chrX:140084755-140085871	NM_032461	.	NP_115850	XP_016855435	protein_coding	ENSG0000022723	107984060	14329
2307	STAM2	0.973	0.005	signal transducing adaptor molecule 2 [Source:HGNC Symbol;Acc:HGNC:11358]	chr2:152973314-153032506	NM_005843	.	NP_005834	.	protein_coding	ENSG0000011514	10254	11358
2308	TSHZ3	-1.685	0.005	teashirt zinc finger homeobox 3 [Source:HGNC Symbol;Acc:HGNC:30700]	chr19:31765850-31840453	NM_020856	.	NP_065907	.	protein_coding	ENSG0000012129	57616	30700
2309	CAP2	1.148	0.005	CAP, adenylate cyclase-associated protein, 2 (yeast) [Source:HGNC Symbol;Acc:HGNC:2	chr6:17393446-17558023	NM_006366	XM_011514233	NP_006357	XP_011512535	protein_coding	ENSG0000011218	10486	20039
2310	ZNF30	1.132	0.005	zinc finger protein 30 [Source:HGNC Symbol;Acc:HGNC:13090]	chr19:35417806-35436074	NM_001099437, NM	XM_017027427, XM_0	NP_001092907, NP_00	XP_011525745, XP_0115	protein_coding	ENSG0000016866	90075	13090
2311	FAM98A	0.757	0.005	family with sequence similarity 98 member A [Source:HGNC Symbol;Acc:HGNC:24520]	chr2:33808724-33824429	NM_001304538, NM	.	NP_001291467, NP_05	.	protein_coding	ENSG0000011981	25940	24520
2312	MTRR	0.884	0.005	5-methyltetrahydrofolate-homocysteine methyltransferase reductase [Source:HGNC Sy	chr5:7869216-7901237	NM_002454, NM_0	XM_011514044, XM_0	NP_002445, NP_07691	XP_011512346	protein_coding	ENSG0000012427	107986404	7473
2313	RRM2B	1.081	0.005	ribonucleotide reductase regulatory TP53 inducible subunit M2B [Source:HGNC Symbol	chr8:103216729-103251346	NM_001172477, NM	.	NP_001165948, NP_00	.	protein_coding	ENSG0000004839	50484	17296
2314	SPNS3	-1.047	0.005	sphingolipid transporter 3 (putative) [Source:HGNC Symbol;Acc:HGNC:28433]	chr17:4336982-4391503	NM_001320449, NM	XM_011523727, XM_0	NP_001307378, NP_87	XP_011522025, XP_0115	protein_coding	ENSG0000018255	201305	28433
2315	STXBP3	0.964	0.005	syntaxin binding protein 3 [Source:HGNC Symbol;Acc:HGNC:11446]	chr1:109289309-109352148	NM_007269	.	NP_009200	.	protein_coding	ENSG0000011626	6814	11446
2316	TRAPPC5	-0.854	0.005	trafficking protein particle complex 5 [Source:HGNC Symbol;Acc:HGNC:23067]	chr19:7741513-7752589	NM_001042461, NM	.	NP_001035926, NP_00	.	protein_coding	ENSG0000018102	126003	23067
2317	CMTM6	0.695	0.005	CKLF like MARVEL transmembrane domain containing 6 [Source:HGNC Symbol;Acc:HGN	chr3:32522803-32544900	NM_017801	.	NP_060271	.	protein_coding	ENSG0000009131	54918	19177
2318	EGR4	-1.164	0.005	early growth response 4 [Source:HGNC Symbol;Acc:HGNC:3241]	chr2:73518056-73520833	NM_001965	.	NP_001956	.	protein_coding	ENSG0000013562	1961	3241
2319	PBDC1	-0.888	0.005	polysaccharide biosynthesis domain containing 1 [Source:HGNC Symbol;Acc:HGNC:2879	chrX:75392770-75398039	NM_001300888, NM	.	NP_001287817, NP_05	.	protein_coding	ENSG0000010239	51260	28790
2320	PIK3C2B	-0.894	0.005	phosphatidylinositol-4-phosphate 3-kinase catalytic subunit type 2 beta [Source:HGNC S	chr1:204391755-204459552	NM_002646	XM_017001474, XM_0	NP_002637	XP_005245314, XP_0052	protein_coding	ENSG0000013305	5287	8972

2321	SAC3D1	-0.780	0.005	SAC3 domain containing 1 [Source:HGNC Symbol;Acc:HGNC:30179]	chr11:64808372-64826021	NM_013299	.	NP_037431	.	protein_coding	ENSG0000016806	29901		30179
2322	STRN4	-0.721	0.005	striatin 4 [Source:HGNC Symbol;Acc:HGNC:15721]	chr19:47222763-47261742	NM_001039877, NN	XM_011526878, XM_0	NP_001034966, NP_03	XP_006723234, XP_0067	protein_coding	ENSG0000009037	29888		15721
2323	WDR19	1.042	0.005	WD repeat domain 19 [Source:HGNC Symbol;Acc:HGNC:18340]	chr4:39184023-39287430	NM_001317924, NN	XM_017008501, XM_0	NP_001304853, NP_07	XP_011512026, XP_0115	protein_coding	ENSG0000015779	57728		18340
2324	ENPP4	0.994	0.006	ectonucleotide pyrophosphatase/phosphodiesterase 4 (putative) [Source:HGNC Symbol	chr6:46097729-46114436	NM_014936	.	NP_055751	.	protein_coding	ENSG0000000156	22875		3359
2325	MTO1	0.944	0.006	mitochondrial tRNA translation optimization 1 [Source:HGNC Symbol;Acc:HGNC:19261]	chr6:74171300-74218959	NM_001123226, NN	XM_017010700, XM_0	NP_001116698, NP_03	XP_006715507, XP_0067	protein_coding	ENSG0000013529	25821		19261
2326	MYEF2	1.024	0.006	myelin expression factor 2 [Source:HGNC Symbol;Acc:HGNC:17940]	chr15:48413168-48470714	NM_001301210, NN	XM_006720553, XM_0	NP_001288139, NP_05	XP_005254479, XP_0052	protein_coding	ENSG0000010417	50804		17940
2327	RANGAP1	-0.638	0.006	Ran GTPase activating protein 1 [Source:HGNC Symbol;Acc:HGNC:9854]	chr22:41641614-41682255	NM_001278651, NN	XM_011530296, XM_0	NP_001265580, NP_00	XP_005261752, XP_0052	protein_coding	ENSG0000010040	5905		9854
2328	ZMYM4	0.924	0.006	zinc finger MYM-type containing 4 [Source:HGNC Symbol;Acc:HGNC:13055]	chr1:35734567-35887545	NM_005095	XM_017002803, XM_0	NP_005086	XP_005271385, XP_0052	protein_coding	ENSG0000014646	9202		13055
2329	BLVRB	-0.714	0.006	biliverdin reductase B [Source:HGNC Symbol;Acc:HGNC:1063]	chr19:40953695-40971747	NM_000713	.	NP_000704	.	protein_coding	ENSG0000009001	645		1063
2330	CHRD	-0.975	0.006	chordin [Source:HGNC Symbol;Acc:HGNC:1949]	chr3:184097860-184107617	NM_001304472, NN	XM_011513256, XM_0	NP_001291401, NP_00	XP_011511556, XP_0115	protein_coding	ENSG0000009053	8646		1949
2331	CTBS	1.057	0.006	chitobiase [Source:HGNC Symbol;Acc:HGNC:2496]	chr1:85015288-85040147	NM_004388	.	NP_004379	.	protein_coding	ENSG0000011715	1486		2496
2332	LRIG2	1.028	0.006	leucine rich repeats and immunoglobulin like domains 2 [Source:HGNC Symbol;Acc:HGN	chr1:113615830-113674882	NM_014813	XM_005271369	NP_001299615, NP_05	XP_005271426	protein_coding	ENSG0000019879	9860		20889
2333	PER3	1.201	0.006	period circadian clock 3 [Source:HGNC Symbol;Acc:HGNC:8847]	chr1:7844379-7913572	NM_001289861, NN	XM_017002731, XM_0	NP_001276790, NP_00	XP_005263581, XP_0115	protein_coding	ENSG0000004924	8863		8847
2334	RHBDD3	-0.921	0.006	rhomboid domain containing 3 [Source:HGNC Symbol;Acc:HGNC:1308]	chr22:29655840-29663967	NM_012265	XM_011530107, XM_0	NP_001316465, NP_03	XP_006724287, XP_0115	protein_coding	ENSG0000010026	25807		1308
2335	ABCA2	-0.703	0.006	ATP binding cassette subfamily A member 2 [Source:HGNC Symbol;Acc:HGNC:32]	chr9:139901678-139923374	NM_001606, NM_2	XM_011518346, XM_0	NP_001597, NP_99769	XP_006717059	protein_coding	ENSG0000010733	20		32
2336	EEF1A2	-0.654	0.006	eukaryotic translation elongation factor 1 alpha 2 [Source:HGNC Symbol;Acc:HGNC:319	chr20:62119365-62130668	NM_001958	.	NP_001949	.	protein_coding	ENSG0000010121	1917		3192
2337	HSPD1	0.850	0.006	heat shock protein family D (Hsp60) member 1 [Source:HGNC Symbol;Acc:HGNC:5261]	chr2:198351304-198418423	NM_002156, NM_1	XM_005246518	NP_002147, NP_95547	XP_005246575	protein_coding	ENSG0000014438	3329		5261
2338	MCRIP2	-0.758	0.006	MAPK regulated corepressor interacting protein 2 [Source:HGNC Symbol;Acc:HGNC:141	chr16:691812-698474	NM_138418	.	NP_612427	.	protein_coding	ENSG0000017236	100287175		14142
2339	OASL	-1.134	0.006	2'-5'-oligoadenylate synthetase like [Source:HGNC Symbol;Acc:HGNC:8090]	chr12:121456913-121477045	NM_001261825, NN	.	NP_001248754, NP_00	XP_016875630	protein_coding	ENSG0000013511	8638		8090
2340	MFAP3	1.047	0.006	microfibrillar associated protein 3 [Source:HGNC Symbol;Acc:HGNC:7034]	chr5:153418518-153434593	NM_001135037, NN	.	NP_001128509, NP_00	.	protein_coding	ENSG0000003774	4238		7034
2341	OTUD4	0.964	0.006	OTU deubiquitinase 4 [Source:HGNC Symbol;Acc:HGNC:24949]	chr4:146054800-146101313	NM_001102653, NN	XM_011532042, XM_0	NP_001096123, NP_05	XP_005263136, XP_0052	protein_coding	ENSG0000016416	54726		24949
2342	PAFAH1B3	-0.749	0.006	platelet activating factor acetylhydrolase 1b catalytic subunit 3 [Source:HGNC Symbol;A	chr19:42801184-42814973	NM_001145939, NN	.	NP_001139411, NP_00	XP_016882335, XP_0168	protein_coding	ENSG0000007946	5050		8576
2343	SENP5	0.844	0.006	SUMO1/sentrin specific peptidase 5 [Source:HGNC Symbol;Acc:HGNC:28407]	chr3:196594726-196661585	NM_001308045, NN	XM_011512544, XM_0	NP_001294974, NP_68	XP_005269367, XP_0052	protein_coding	ENSG0000011923	205564		28407
2344	SLC20A2	0.991	0.006	solute carrier family 20 member 2 [Source:HGNC Symbol;Acc:HGNC:10947]	chr8:42273992-42410336	NM_001257180, NN	XM_017013748, XM_0	NP_001244109, NP_00	XP_005273670, XP_0052	protein_coding	ENSG0000016857	6575		10947
2345	FAM43B	-1.337	0.006	family with sequence similarity 43 member B [Source:HGNC Symbol;Acc:HGNC:31791]	chr1:20878931-20881503	NM_207334	.	NP_997217	.	protein_coding	ENSG0000018311	163933		31791
2346	TGFBR1	0.817	0.006	transforming growth factor beta receptor 1 [Source:HGNC Symbol;Acc:HGNC:11772]	chr9:101867370-101916474	NM_001130916, NN	XM_011518950, XM_0	NP_001124388, NP_00	XP_005252207, XP_0115	protein_coding	ENSG0000010679	7046		11772
2347	TMEM63C	-0.969	0.006	transmembrane protein 63C [Source:HGNC Symbol;Acc:HGNC:23787]	chr14:77648169-77725838	NM_020431	.	NP_065164	.	protein_coding	ENSG0000016554	57156		23787
2348	TUBGCP5	0.961	0.006	tubulin gamma complex associated protein 5 [Source:HGNC Symbol;Acc:HGNC:18600]	chr15:22833394-22873892	NM_001102610, NN	XM_011543870, XM_0	NP_001096080, NP_44	XP_005272597, XP_0115	protein_coding	ENSG0000027583	114791		18600
2349	UPF3B	0.880	0.006	UPF3 regulator of nonsense transcripts homolog B (yeast) [Source:HGNC Symbol;Acc:HG	chrX:118967984-118986961	NM_023010, NM_0	.	NP_075386, NP_54219	.	protein_coding	ENSG0000012535	65109		20439
2350	URI1	0.775	0.006	URI1, prefoldin like chaperone [Source:HGNC Symbol;Acc:HGNC:13236]	chr19:30414550-30507515	NM_001252641, NN	XM_005259363, XM_0	NP_001239570, NP_00	XP_005259419, XP_0052	protein_coding	ENSG0000010517	8725		13236
2351	C2orf78	1.255	0.006	chromosome 2 open reading frame 78 [Source:HGNC Symbol;Acc:HGNC:34349]	chr2:74011315-74044274	NM_001080474	XM_005264302	NP_001073943	XP_005264359	protein_coding	ENSG0000018783	388960		34349
2352	ME2	0.932	0.006	malic enzyme 2 [Source:HGNC Symbol;Acc:HGNC:6984]	chr18:48405418-48480627	NM_001168335, NN	.	NP_001161807, NP_00	.	protein_coding	ENSG0000008221	4200		6984
2353	SLC4A7	1.004	0.006	solute carrier family 4 member 7 [Source:HGNC Symbol;Acc:HGNC:11033]	chr3:27414211-27525911	NM_001258379, NN	XM_017007529, XM_0	NP_001245308, NP_00	XP_005265655, XP_0052	protein_coding	ENSG0000003386	9497		11033
2354	STRADB	0.882	0.006	STE20-related kinase adaptor beta [Source:HGNC Symbol;Acc:HGNC:13205]	chr2:202316391-202345569	NM_001206864, NN	XM_005246669	NP_001193793, NP_06	XP_005246726	protein_coding	ENSG0000008214	55437		13205
2355	TMEM256-PL	-1.009	0.006	TMEM256-PLSCR3 readthrough (NMD candidate) [Source:HGNC Symbol;Acc:HGNC:491	chr17:7283852-7298162	NM_001201576, NN	.	NP_001188505, NP_06	.	protein_coding	ENSG0000018783	57048		49186
2356	APPBP2	0.910	0.006	amyloid beta precursor protein binding protein 2 [Source:HGNC Symbol;Acc:HGNC:622]	chr17:58520509-58603580	NM_001282476, NN	XM_011524198	NP_001269405, NP_00	XP_011522500	protein_coding	ENSG0000006272	10513		622
2357	CCNT2	0.973	0.006	cyclin T2 [Source:HGNC Symbol;Acc:HGNC:1600]	chr2:135676394-135716912	NM_001241, NM_0	.	NP_001232, NP_00130	.	protein_coding	ENSG0000008225	905		1600
2358	GCLC	0.844	0.006	glutamate-cysteine ligase catalytic subunit [Source:HGNC Symbol;Acc:HGNC:4311]	chr6:53362138-53409927	NM_001197115, NN	.	NP_001184044, NP_00	.	protein_coding	ENSG0000000108	2729		4311
2359	HSPBP1	-0.687	0.006	HSPA (Hsp70) binding protein 1 [Source:HGNC Symbol;Acc:HGNC:24989]	chr19:55773598-55791728	NM_001130106, NN	XM_017026545, XM_0	NP_001123578, NP_00	XP_005258757, XP_0052	protein_coding	ENSG0000013326	23640		24989
2360	ITGA6	0.677	0.006	integrin subunit alpha 6 [Source:HGNC Symbol;Acc:HGNC:6142]	chr2:173292145-173371010	NM_000210, NM_0	XM_017004007, XM_0	NP_000201, NP_00107	XP_006712573, XP_0067	protein_coding	ENSG0000009140	3655		6142
2361	OSBPL6	0.957	0.006	oxysterol binding protein like 6 [Source:HGNC Symbol;Acc:HGNC:16388]	chr2:179059207-179267618	NM_001201480, NN	XM_017003271, XM_0	NP_001188409, NP_00	XP_005246325, XP_0115	protein_coding	ENSG0000007915	114880		16388
2362	SLCO4A1	-0.653	0.006	solute carrier organic anion transporter family member 4A1 [Source:HGNC Symbol;Acc:	chr20:61273796-61303647	NM_016354	XM_011528793, XM_0	NP_057438	XP_005260260, XP_0115	protein_coding	ENSG0000010118	28231		10953
2363	TRABD	-0.733	0.006	TraB domain containing [Source:HGNC Symbol;Acc:HGNC:28805]	chr22:50624343-50638027	NM_001320484, NN	XM_017028944, XM_0	NP_001307413, NP_00	XP_006724485, XP_0067	protein_coding	ENSG0000017063	80305		28805
2364	CXCL6	-1.746	0.006	C-X-C motif chemokine ligand 6 [Source:HGNC Symbol;Acc:HGNC:10643]	chr4:74702213-74704477	NM_002993	.	NP_002984	.	protein_coding	ENSG0000012487	6372		10643
2365	HNRNPH1	0.693	0.006	heterogeneous nuclear ribonucleoprotein H1 (H) [Source:HGNC Symbol;Acc:HGNC:5041	chr5:179041178-179051670	NM_001257293, NN	XM_017009423, XM_0	NP_001244222, NP_00	XP_005265952, XP_0052	protein_coding	ENSG0000016904	3187		5041
2366	HTRA3	-1.130	0.006	HtrA serine peptidase 3 [Source:HGNC Symbol;Acc:HGNC:30406]	chr4:8271491-8308838	NM_001297559, NN	XM_011513596	NP_001284488, NP_44	XP_011511898	protein_coding	ENSG0000017080	94031		30406
2367	INPP5J	-1.018	0.006	inositol polyphosphate-5-phosphatase J [Source:HGNC Symbol;Acc:HGNC:8956]	chr22:31518908-31530682	NM_001002837, NN	XM_011530143	NP_001002837, NP_00	XP_011528445, XP_0168	protein_coding	ENSG0000018513	27124		8956
2368	SEC62	0.945	0.006	SEC62 homolog, preprotein translocation factor [Source:HGNC Symbol;Acc:HGNC:1184	chr3:169684552-169716161	NM_003262	XM_011513114	NP_003253	XP_011511416	protein_coding	ENSG0000000895	7095		11846
2369	TYW1	0.842	0.006	tRNA-yW synthesizing protein 1 homolog [Source:HGNC Symbol;Acc:HGNC:25598]	chr7:66461801-66704506	NM_018264	XM_011516374, XM_0	NP_060734	XP_011514674	protein_coding	ENSG0000019887	55253		25598
2370	ARNTL2	0.881	0.006	aryl hydrocarbon receptor nuclear translocator like 2 [Source:HGNC Symbol;Acc:HGNC:	chr12:27485786-27578222	NM_001248002, NN	XM_017019669, XM_0	NP_001234931, NP_00	XP_006719175, XP_0067	protein_coding	ENSG0000002915	56938		18984
2371	B3GNT5	0.990	0.006	UDP-GlcNAc:betaGal beta-1,3-N-acetylglucosaminyltransferase 5 [Source:HGNC Symbol	chr3:182895830-183146063	NM_032047	XM_011513229, XM_0	NP_114436	XP_005247880, XP_0052	protein_coding	ENSG0000017659	84002		15684
2372	CARD19	-0.728	0.006	caspase recruitment domain family member 19 [Source:HGNC Symbol;Acc:HGNC:28148	chr9:95858499-95875564	NM_032310	XM_005252273	NP_115686	.	protein_coding	ENSG0000016523	84270		28148
2373	MDN1	0.796	0.006	midasin AAA ATPase 1 [Source:HGNC Symbol;Acc:HGNC:18302]	chr6:90352217-90529442	NM_014611	XM_011535636, XM_0	NP_055426	XP_011533937, XP_0115	protein_coding	ENSG0000011215	23195		18302
2374	NDUFB7	-0.702	0.006	NADH:ubiquinone oxidoreductase subunit B7 [Source:HGNC Symbol;Acc:HGNC:7702]	chr19:14676889-14682874	NM_004146	.	NP_004137	.	protein_coding	ENSG0000009979	4713		7702

2375	PHLDB3	-0.861	0.006	pleckstrin homology like domain family B member 3 [Source:HGNC Symbol;Acc:HGNC:3	chr19:43979254-44009087	NM_198850	XM_011527236, XM_0	NP_942147	XP_005259226, XP_0052	protein_coding	ENSG0000017653	653583	30499
2376	SPAG5	0.677	0.006	sperm associated antigen 5 [Source:HGNC Symbol;Acc:HGNC:13452]	chr17:26904591-26926297	NM_006461	.	NP_006452	.	protein_coding	ENSG0000007638	10615	13452
2377	SRFBP1	1.036	0.006	serum response factor binding protein 1 [Source:HGNC Symbol;Acc:HGNC:26333]	chr5:121297655-121364314	NM_152546	.	NP_689759	.	protein_coding	ENSG0000015130	153443	26333
2378	STARD4	1.028	0.006	StAR related lipid transfer domain containing 4 [Source:HGNC Symbol;Acc:HGNC:18058	chr5:110831730-110848288	NM_001308056, NM	XM_017009044, XM_0	NP_001294985, NP_00	XP_005271938, XP_0115	protein_coding	ENSG0000016421	134429	18058
2379	TNNT1	-0.715	0.006	troponin T1, slow skeletal type [Source:HGNC Symbol;Acc:HGNC:11948]	chr19:55644161-55660722	NM_001126132, NM	XM_011527246, XM_0	NP_001119604, NP_00	XP_011525547, XP_0115	protein_coding	ENSG0000010504	7138	11948
2380	ACSL3	0.806	0.006	acyl-CoA synthetase long-chain family member 3 [Source:HGNC Symbol;Acc:HGNC:3570	chr2:223725651-223809357	NM_004457, NM_2	.	NP_004448, NP_97625	XP_016859073	protein_coding	ENSG0000012398	2181	3570
2381	ATP6V1C1	1.053	0.006	ATPase H+ transporting V1 subunit C1 [Source:HGNC Symbol;Acc:HGNC:856]	chr8:104033290-104085279	NM_001695	.	NP_001686	.	protein_coding	ENSG0000015509	528	856
2382	BEX5	-1.024	0.006	brain expressed X-linked 5 [Source:HGNC Symbol;Acc:HGNC:27990]	chrX:101408679-101411029	NM_001012978, NM	.	NP_001012996, NP_00	.	protein_coding	ENSG0000018451	340542	27990
2383	DLX6	1.078	0.006	distal-less homeobox 6 [Source:HGNC Symbol;Acc:HGNC:2919]	chr7:96634859-96640351	NM_005222	.	NP_005213	.	protein_coding	ENSG0000000637	1750	2919
2384	EPB41L5	0.939	0.006	erythrocyte membrane protein band 4.1 like 5 [Source:HGNC Symbol;Acc:HGNC:19819]	chr2:120770580-120936695	NM_001184937, NM	XM_017004567, XM_0	NP_001171866, NP_00	XP_006712714, XP_0067	protein_coding	ENSG0000011510	57669	19819
2385	GNG7	-1.173	0.006	G protein subunit gamma 7 [Source:HGNC Symbol;Acc:HGNC:4410]	chr19:2511216-2702707	NM_052847	XM_017026606	NP_443079	XP_016882095	protein_coding	ENSG0000017653	2788	4410
2386	LCA5	1.162	0.006	LCA5, lebercilin [Source:HGNC Symbol;Acc:HGNC:31923]	chr6:80194707-80247175	NM_001122769, NM	XM_011535504, XM_0	NP_001116241, NP_85	XP_005248722, XP_0115	protein_coding	ENSG0000013533	167691	31923
2387	PLPP5	1.176	0.006	phospholipid phosphatase 5 [Source:HGNC Symbol;Acc:HGNC:25026]	chr8:38120647-38126761	NM_001102559, NM	XM_011544673, XM_0	NP_001096029, NP_00	XP_005273720, XP_0115	protein_coding	ENSG0000014753	84513	25026
2388	SLC25A42	-0.973	0.006	solute carrier family 25 member 42 [Source:HGNC Symbol;Acc:HGNC:28380]	chr19:19174807-19223697	NM_001321544, NM	XM_011527937, XM_0	NP_001308473, NP_84	XP_005259918, XP_0115	protein_coding	ENSG0000018103	284439	28380
2389	ABHD17A	-0.764	0.006	abhydrolase domain containing 17A [Source:HGNC Symbol;Acc:HGNC:28756]	chr19:1876808-1885546	NM_001130111, NM	XM_011528338	NP_001123583, NP_11	XP_011526640, XP_0168	protein_coding	ENSG0000012996	81926	28756
2390	ACADVL	-0.657	0.006	acyl-CoA dehydrogenase, very long chain [Source:HGNC Symbol;Acc:HGNC:92]	chr17:7093208-7128592	NM_000018, NM_0	XM_011523830, XM_0	NP_000009, NP_00102	XP_006721579, XP_0115	protein_coding	ENSG0000007277	37	92
2391	CNOT6L	0.927	0.006	CCR4-NOT transcription complex subunit 6 like [Source:HGNC Symbol;Acc:HGNC:18042]	chr4:78634540-78740522	NM_001286790, NM	XM_017007957, XM_0	NP_001273719, NP_65	XP_011530108, XP_0115	protein_coding	ENSG0000013876	246175	18042
2392	KLRC2	-2.534	0.006	killer cell lectin like receptor C2 [Source:HGNC Symbol;Acc:HGNC:6375]	chr12:10564910-10607284	NM_002260	.	NP_002251	.	protein_coding	ENSG0000020580	3822	6375
2393	TTBK2	0.916	0.006	tau tubulin kinase 2 [Source:HGNC Symbol;Acc:HGNC:19141]	chr15:43030931-43213007	NM_173500	XM_017021950, XM_0	NP_775771	XP_005254228, XP_0052	protein_coding	ENSG0000012888	146057	19141
2394	ZC3H8	1.007	0.006	zinc finger CCCH-type containing 8 [Source:HGNC Symbol;Acc:HGNC:30941]	chr2:112969101-113012688	NM_032494	XM_005263826	NP_115883	.	protein_coding	ENSG0000014416	84524	30941
2395	CDT1	-0.698	0.006	chromatin licensing and DNA replication factor 1 [Source:HGNC Symbol;Acc:HGNC:2457	chr16:88869620-88875666	NM_030928	.	NP_112190	.	protein_coding	ENSG0000016751	81620	24576
2396	CEBPA	-0.897	0.006	CCAAT/enhancer binding protein alpha [Source:HGNC Symbol;Acc:HGNC:1833]	chr19:33790839-33793470	NM_001285829, NM	.	NP_001272758, NP_00	.	protein_coding	ENSG0000024584	1050	1833
2397	COMMD5	-0.846	0.006	COMM domain containing 5 [Source:HGNC Symbol;Acc:HGNC:17902]	chr8:146075550-146079121	NM_001081003, NM	.	NP_001074472, NP_00	.	protein_coding	ENSG0000017061	28991	17902
2398	FA2H	-0.947	0.006	fatty acid 2-hydroxylase [Source:HGNC Symbol;Acc:HGNC:21197]	chr16:74746852-74808723	NM_024306	.	NP_077282	.	protein_coding	ENSG0000010308	79152	21197
2399	FAM161A	1.131	0.006	family with sequence similarity 161 member A [Source:HGNC Symbol;Acc:HGNC:25808]	chr2:62051990-62081278	NM_001201543, NM	XM_017005074, XM_0	NP_001188472, NP_11	XP_016860561, XP_0168	protein_coding	ENSG0000017026	84140	25808
2400	LRRC29	-1.819	0.006	leucine rich repeat containing 29 [Source:HGNC Symbol;Acc:HGNC:13605]	chr16:67241042-67260930	NM_001004055, NM	XM_011522998, XM_0	NP_001004055, NP_03	XP_011521300, XP_0168	protein_coding	ENSG0000012512	26231	13605
2401	MZT2A	-0.703	0.006	mitotic spindle organizing protein 2A [Source:HGNC Symbol;Acc:HGNC:33187]	chr2:132241532-132249995	NM_001085365	.	NP_001078834	.	protein_coding	ENSG0000017327	100616378	33187
2402	NDUFB4	-0.674	0.006	NADH:ubiquinone oxidoreductase subunit B4 [Source:HGNC Symbol;Acc:HGNC:7699]	chr3:120315155-120321347	NM_001168331, NM	.	NP_001161803, NP_00	.	protein_coding	ENSG0000006551	4710	7699
2403	SCAND1	-0.724	0.006	SCAN domain containing 1 [Source:HGNC Symbol;Acc:HGNC:10566]	chr20:34541538-34547394	NM_016558, NM_0	.	NP_057642, NP_36101	.	protein_coding	ENSG0000017122	51282	10566
2404	CLUAP1	0.949	0.006	clusterin associated protein 1 [Source:HGNC Symbol;Acc:HGNC:19009]	chr16:3550923-3627392	NM_015041, NM_0	XM_006720867	NP_055856, NP_07906	XP_006720930, XP_0168	protein_coding	ENSG0000010335	23059	19009
2405	HS1BP3	-0.757	0.006	HCLS1 binding protein 3 [Source:HGNC Symbol;Acc:HGNC:24979]	chr2:20817581-20850849	NM_022460	XM_017004700	NP_071905	XP_016860189	protein_coding	ENSG0000011896	64342	24979
2406	KRT5	-0.748	0.006	keratin 5 [Source:HGNC Symbol;Acc:HGNC:6442]	chr12:52908358-52914471	NM_000424	.	NP_000415	.	protein_coding	ENSG0000018608	3852	6442
2407	PKD2	1.039	0.006	polycystin 2, transient receptor potential cation channel [Source:HGNC Symbol;Acc:HGN	chr4:88928819-88998929	NM_000297	XM_011532029, XM_0	NP_000288	XP_011530330, XP_0115	protein_coding	ENSG0000011876	5311	9009
2408	S100A6	-0.674	0.006	S100 calcium binding protein A6 [Source:HGNC Symbol;Acc:HGNC:10496]	chr1:153507074-153508720	NM_014624	.	NP_055439	XP_016857522	protein_coding	ENSG0000019795	6277	10496
2409	SAPCD2	-0.648	0.006	suppressor APC domain containing 2 [Source:HGNC Symbol;Acc:HGNC:28055]	chr9:139956575-139965040	NM_178448	XM_011519180	NP_848543	XP_011517482	protein_coding	ENSG0000018619	89958	28055
2410	ZNF787	-0.735	0.006	zinc finger protein 787 [Source:HGNC Symbol;Acc:HGNC:26998]	chr19:56598731-56632649	NM_001002836	XM_011526445	NP_001002836	XP_011524747	protein_coding	ENSG0000014240	126208	26998
2411	APOBEC3C	-0.634	0.006	apolipoprotein B mRNA editing enzyme catalytic subunit 3C [Source:HGNC Symbol;Acc:H	chr22:39410087-39429281	NM_014508	.	NP_055323	.	protein_coding	ENSG0000024450	27350	17353
2412	FIBP	-0.697	0.006	FGF1 intracellular binding protein [Source:HGNC Symbol;Acc:HGNC:3705]	chr11:65651391-65656010	NM_004214, NM_1	.	NP_004205, NP_94260	.	protein_coding	ENSG0000017250	9158	3705
2413	KIZ	0.794	0.006	kizuna centrosomal protein [Source:HGNC Symbol;Acc:HGNC:15865]	chr20:21106623-21227260	NM_001163022, NM	XM_011529299, XM_0	NP_001156494, NP_00	XP_011527598, XP_0115	protein_coding	ENSG0000008897	55857	15865
2414	CLASP1	0.810	0.006	cytoplasmic linker associated protein 1 [Source:HGNC Symbol;Acc:HGNC:17088]	chr2:122095351-122407163	NM_001142273, NM	XM_017003673, XM_0	NP_001135745, NP_00	XP_006712440, XP_0067	protein_coding	ENSG0000007405	23332	17088
2415	KIAA0930	-0.722	0.006	KIAA0930 [Source:HGNC Symbol;Acc:HGNC:1314]	chr22:45586218-45636650	NM_001009880, NM	XM_011530017	NP_001009880, NP_05	XP_011528319, XP_0168	protein_coding	ENSG0000010036	23313	1314
2416	PTP4A2	0.749	0.006	protein tyrosine phosphatase type IVA, member 2 [Source:HGNC Symbol;Acc:HGNC:963	chr1:32372021-32385084	NM_001195100, NM	XM_006710928, XM_0	NP_001182029, NP_00	XP_005271286, XP_0052	protein_coding	ENSG0000018400	8073	9635
2417	RIMKLB	0.988	0.006	ribosomal modification protein rimK like family member B [Source:HGNC Symbol;Acc:HG	chr12:8850495-8935681	NM_001297776, NM	XM_017019697, XM_0	NP_001284705, NP_06	XP_006719179, XP_0067	protein_coding	ENSG0000016653	57494	29228
2418	RUNDC3A	-0.878	0.006	RUN domain containing 3A [Source:HGNC Symbol;Acc:HGNC:16984]	chr17:42385926-42396039	NM_001144825, NM	XM_005256964, XM_0	NP_001138297, NP_00	XP_005257021, XP_0168	protein_coding	ENSG0000010830	10900	16984
2419	ZBTB44	0.974	0.006	zinc finger and BTB domain containing 44 [Source:HGNC Symbol;Acc:HGNC:25001]	chr11:130096572-130184581	NM_001301098, NM	XM_006718826, XM_0	NP_001288027, NP_00	XP_005271578, XP_0052	protein_coding	ENSG0000019632	29068	25001
2420	CBY1	-0.852	0.006	chibby family member 1, beta catenin antagonist [Source:HGNC Symbol;Acc:HGNC:1307	chr22:39052659-39069859	NM_001002880, NM	.	NP_001002880, NP_05	.	protein_coding	ENSG0000010021	25776	1307
2421	FPGS	-0.684	0.006	folylpolyglutamate synthase [Source:HGNC Symbol;Acc:HGNC:3824]	chr9:130565136-130576360	NM_001018078, NM	XM_011518438, XM_0	NP_001018088, NP_00	XP_005251921, XP_0115	protein_coding	ENSG0000013687	2356	3824
2422	RAB3GAP1	0.852	0.006	RAB3 GTPase activating protein catalytic subunit 1 [Source:HGNC Symbol;Acc:HGNC:17	chr2:135809834-136288806	NM_001172435, NM	XM_011510825, XM_0	NP_001165906, NP_03	XP_011509125, XP_0115	protein_coding	ENSG0000011583	22930	17063
2423	SMIM22	-0.814	0.006	small integral membrane protein 22 [Source:HGNC Symbol;Acc:HGNC:48329]	chr16:4827669-4846492	NM_001253790, NM	XM_011522501, XM_0	NP_001240719, NP_00	XP_011520801, XP_0115	protein_coding	ENSG0000026779	440335	48329
2424	ZBTB7C	-0.971	0.006	zinc finger and BTB domain containing 7C [Source:HGNC Symbol;Acc:HGNC:31700]	chr18:45553043-45935793	NM_001039360	XM_017025609, XM_0	NP_001034449, NP_00	XP_005258284, XP_0052	protein_coding	ENSG0000018482	201501	31700
2425	ALOX5AP	-1.638	0.006	arachidonate 5-lipoxygenase activating protein [Source:HGNC Symbol;Acc:HGNC:436]	chr13:31287614-31338563	NM_001204406, NM	.	NP_001191335, NP_00	.	protein_coding	ENSG0000013296	241	436
2426	BAG5	0.748	0.006	BCL2 associated athanogene 5 [Source:HGNC Symbol;Acc:HGNC:941]	chr14:104022880-104029151	NM_001015048, NM	.	NP_001015048, NP_00	.	protein_coding	ENSG0000016617	9529	941
2427	C9orf69	-0.678	0.006	chromosome 9 open reading frame 69 [Source:HGNC Symbol;Acc:HGNC:31009]	chr9:139006426-139010709	NM_001256526, NM	.	NP_001243455, NP_69	.	protein_coding	ENSG0000023822	90120	31009
2428	CSTF1	0.786	0.006	cleavage stimulation factor subunit 1 [Source:HGNC Symbol;Acc:HGNC:2483]	chr20:54967449-54981425	NM_001033521, NM	XM_011528600	NP_001028693, NP_00	XP_011526902	protein_coding	ENSG0000010113	1477	2483

2429	MBP	0.792	0.006	myelin basic protein [Source:HGNC Symbol;Acc:HGNC:6925]	chr18:74690782-74844800	NM_001025081, NN	XM_017025780, XM_(	NP_001020252, NP_00	XP_016881267, XP_0168	protein_coding	ENSG0000019797	4155	6925
2430	MTCL1	0.884	0.006	microtubule crosslinking factor 1 [Source:HGNC Symbol;Acc:HGNC:29121]	chr18:8705658-8832776	NM_015210	XM_011525641, XM_(	NP_056025	XP_005258155, XP_0052	protein_coding	ENSG0000016850	23255	29121
2431	OBSL1	-0.730	0.006	obscurin like 1 [Source:HGNC Symbol;Acc:HGNC:29092]	chr2:220415463-220436186	NM_001173408, NN	XM_017003698, XM_(	NP_001166879, NP_00	XP_005246481, XP_0052	protein_coding	ENSG0000012400	23363	29092
2432	ACSF3	-0.854	0.006	acyl-CoA synthetase family member 3 [Source:HGNC Symbol;Acc:HGNC:27288]	chr16:89160216-89222254	NM_001127214, NN	XM_005256293	NP_001120686, NP_00	XP_005256350, XP_0168	protein_coding	ENSG0000017671	197322	27288
2433	LATS2	0.810	0.006	large tumor suppressor kinase 2 [Source:HGNC Symbol;Acc:HGNC:6515]	chr13:21547170-21635686	NM_014572	XM_005266342	NP_055387	XP_005266399	protein_coding	ENSG0000015045	26524	6515
2434	SSBP2	-1.330	0.006	single stranded DNA binding protein 2 [Source:HGNC Symbol;Acc:HGNC:15831]	chr5:80708839-81047072	NM_001256732, NN	XM_017009309, XM_(	NP_001243661, NP_00	XP_011541599, XP_0168	protein_coding	ENSG0000014568	23635	15831
2435	ASCL2	-0.816	0.006	achaete-scute family bHLH transcription factor 2 [Source:HGNC Symbol;Acc:HGNC:739]	chr11:2289724-2292182	NM_005170	.	NP_005161	.	protein_coding	ENSG0000018373	430	739
2436	CUL5	0.865	0.006	cullin 5 [Source:HGNC Symbol;Acc:HGNC:2556]	chr11:107879458-107978503	NM_003478	XM_005271682	NP_003469	XP_005271739, XP_0168	protein_coding	ENSG0000016626	8065	2556
2437	SH3BP1	-0.694	0.006	SH3 domain binding protein 1 [Source:HGNC Symbol;Acc:HGNC:10824]	chr22:38035481-38052124	NM_018957	.	NP_061830	.	protein_coding	ENSG0000010009	23616	10824
2438	SOX13	-0.710	0.006	SRY-box 13 [Source:HGNC Symbol;Acc:HGNC:11192]	chr1:204042731-204096871	NM_005686	XM_005245623	NP_005677	XP_005245680	protein_coding	ENSG0000014384	9580	11192
2439	CCDC3	1.343	0.006	coiled-coil domain containing 3 [Source:HGNC Symbol;Acc:HGNC:23813]	chr10:12938624-13180308	NM_001282658, NN	.	NP_001269587, NP_11	.	protein_coding	ENSG0000015146	83643	23813
2440	HES1	-0.835	0.006	hes family bHLH transcription factor 1 [Source:HGNC Symbol;Acc:HGNC:5192]	chr3:193853933-193856521	NM_005524	.	NP_005515	.	protein_coding	ENSG0000011431	3280	5192
2441	KLF14	1.217	0.006	Kruppel like factor 14 [Source:HGNC Symbol;Acc:HGNC:23025]	chr7:130416062-130418883	NM_138693	.	NP_619638	.	protein_coding	ENSG0000026626	136259	23025
2442	PPP2R5C	0.792	0.006	protein phosphatase 2 regulatory subunit B'gamma [Source:HGNC Symbol;Acc:HGNC:93	chr14:102228134-102394326	NM_001161725, NN	XM_011536921, XM_(	NP_001155197, NP_00	XP_005267876, XP_0052	protein_coding	ENSG0000007830	5527	9311
2443	TUBD1	1.152	0.006	tubulin delta 1 [Source:HGNC Symbol;Acc:HGNC:16811]	chr17:57936842-57970306	NM_001193609, NN	XM_011524871, XM_(	NP_001180538, NP_00	XP_005257482, XP_0115	protein_coding	ENSG0000010842	51174	16811
2444	ELOVL7	0.949	0.007	ELOVL fatty acid elongase 7 [Source:HGNC Symbol;Acc:HGNC:26292]	chr5:60047617-60140216	NM_001104558, NN	XM_017009885, XM_(	NP_001098028, NP_00	XP_005248663, XP_0052	protein_coding	ENSG0000016418	79993	26292
2445	OTUD3	0.985	0.007	OTU deubiquitinase 3 [Source:HGNC Symbol;Acc:HGNC:29038]	chr1:20209005-20239438	NM_015207	XM_011541072, XM_(	NP_056022	XP_005245849, XP_0052	protein_coding	ENSG0000016991	23252	29038
2446	SSNA1	-0.698	0.007	SS nuclear autoantigen 1 [Source:HGNC Symbol;Acc:HGNC:11321]	chr9:140083098-140084822	NM_003731	.	NP_003722	.	protein_coding	ENSG0000017610	8636	11321
2447	CDK17	0.956	0.007	cyclin dependent kinase 17 [Source:HGNC Symbol;Acc:HGNC:8750]	chr12:96672038-96794338	NM_001170464, NN	XM_011538436, XM_(	NP_001163935, NP_00	XP_006719507, XP_0115	protein_coding	ENSG0000005975	5128	8750
2448	G3BP2	0.691	0.007	G3BP stress granule assembly factor 2 [Source:HGNC Symbol;Acc:HGNC:30291]	chr4:76567965-76599153	NM_012297, NM_2	XM_017008877, XM_(	NP_036429, NP_98710	XP_005263439, XP_0052	protein_coding	ENSG0000013875	9908	30291
2449	GEMIN5	0.706	0.007	gem nuclear organelle associated protein 5 [Source:HGNC Symbol;Acc:HGNC:20043]	chr5:154266975-154317769	NM_001252156, NN	.	NP_001239085, NP_05	.	protein_coding	ENSG0000008251	25929	20043
2450	GMIP	-0.830	0.007	GEM interacting protein [Source:HGNC Symbol;Acc:HGNC:24852]	chr19:19740284-19754476	NM_001288998, NN	XM_011528048, XM_(	NP_001275927, NP_00	XP_005259984, XP_0115	protein_coding	ENSG0000008963	51291	24852
2451	KCNK13	-0.965	0.007	potassium two pore domain channel subfamily K member 13 [Source:HGNC Symbol;Acc	chr14:90528108-90652201	NM_022054	.	NP_071337	.	protein_coding	ENSG0000015231	56659	6275
2452	NSUN6	1.011	0.007	NOP2/Sun RNA methyltransferase family member 6 [Source:HGNC Symbol;Acc:HGNC:2	chr10:18834489-18940551	NM_182543	XM_017015906, XM_(	NP_872349	XP_011517685, XP_0115	protein_coding	ENSG0000024105	221078	23529
2453	PLXNA1	-0.631	0.007	plexin A1 [Source:HGNC Symbol;Acc:HGNC:9099]	chr3:126707436-126756235	NM_032242	XM_011512909, XM_(	NP_115618	XP_011511210, XP_0115	protein_coding	ENSG0000011455	5361	9099
2454	TTC39B	1.123	0.007	tetratricopeptide repeat domain 39B [Source:HGNC Symbol;Acc:HGNC:23704]	chr9:15163619-15307358	NM_001168339, NN	XM_011517732	NP_001161811, NP_00	XP_011516034	protein_coding	ENSG0000015515	158219	23704
2455	GNG12	0.801	0.007	G protein subunit gamma 12 [Source:HGNC Symbol;Acc:HGNC:19663]	chr1:68167148-68299150	NM_018841	XM_017001809	NP_061329	XP_016857298, XP_0168	protein_coding	ENSG0000017238	55970	19663
2456	IPO8	0.794	0.007	importin 8 [Source:HGNC Symbol;Acc:HGNC:9853]	chr12:30781921-30848920	NM_001190995, NN	XM_017018692, XM_(	NP_001177924, NP_00	XP_016874180, XP_0168	protein_coding	ENSG0000013370	10526	9853
2457	PIN4	-1.092	0.007	peptidylprolyl cis/trans isomerase, NIMA-interacting 4 [Source:HGNC Symbol;Acc:HGNC	chrX:71401525-71483814	NM_001170747, NN	.	NP_001164218, NP_00	.	protein_coding	ENSG0000010230	5303	8992
2458	ZNF112	1.249	0.007	zinc finger protein 112 [Source:HGNC Symbol;Acc:HGNC:12892]	chr19:44830707-44905774	NM_001083335, NN	.	NP_001076804, NP_03	.	protein_coding	ENSG0000006237	7771	12892
2459	CNOT6	0.741	0.007	CCR4-NOT transcription complex subunit 6 [Source:HGNC Symbol;Acc:HGNC:14099]	chr5:179921416-180005405	NM_001303241	XM_017009671, XM_(	NP_001290170	XP_005266010, XP_0115	protein_coding	ENSG0000011330	57472	14099
2460	COX4I1	-0.641	0.007	cytochrome c oxidase subunit 4I1 [Source:HGNC Symbol;Acc:HGNC:2265]	chr16:85833288-85840650	NM_001318797, NN	XM_011522871, XM_(	NP_001305715, NP_00	XP_005255855	protein_coding	ENSG0000013114	1327	2265
2461	DRG2	-0.696	0.007	developmentally regulated GTP binding protein 2 [Source:HGNC Symbol;Acc:HGNC:303	chr17:17991199-18011285	NM_001388	XM_017024297, XM_(	NP_001379	XP_005256556, XP_0052	protein_coding	ENSG0000010859	1819	3030
2462	GLCE	0.873	0.007	glucuronic acid epimerase [Source:HGNC Symbol;Acc:HGNC:17855]	chr15:69452972-69564556	NM_001324093, NN	XM_017022073, XM_(	NP_001311022, NP_00	XP_005254355, XP_0168	protein_coding	ENSG0000013860	26035	17855
2463	USF2	-0.688	0.007	upstream transcription factor 2, c-fos interacting [Source:HGNC Symbol;Acc:HGNC:1259	chr19:35759880-35770718	NM_003367, NM_2	XM_005259197	NP_001308079, NP_00	XP_005259254	protein_coding	ENSG0000010569	7392	12594
2464	CTB-50L17.1	-0.701	0.007	Hepatoma-derived growth factor-related protein 2 [Source:UniProtKB/Swiss-Prot;Acc:C	chr19:4472283-4518477	NM_001001520, NN	XM_011528377, XM_(	NP_001001520, NP_11	XP_011526677, XP_0115	protein_coding	ENSG0000016767	84717	.
2465	PPTC7	0.891	0.007	PTC7 protein phosphatase homolog [Source:HGNC Symbol;Acc:HGNC:30695]	chr12:110971049-111021125	NM_139283	.	NP_644812	.	protein_coding	ENSG0000019685	160760	30695
2466	RNF170	1.117	0.007	ring finger protein 170 [Source:HGNC Symbol;Acc:HGNC:25358]	chr8:42704779-42751866	NM_001160223, NN	XM_011544666, XM_(	NP_001153695, NP_00	XP_006716467, XP_0067	protein_coding	ENSG0000012092	100616115	25358
2467	TSN	0.805	0.007	translin [Source:HGNC Symbol;Acc:HGNC:12379]	chr2:122513108-122525429	NM_001261401, NN	.	NP_001248330, NP_00	.	protein_coding	ENSG0000021146	7247	12379
2468	UQCRC2	0.753	0.007	ubiquinol-cytochrome c reductase core protein II [Source:HGNC Symbol;Acc:HGNC:1258	chr16:21963980-21994981	NM_003366	.	NP_003357	.	protein_coding	ENSG0000014074	7385	12586
2469	ZNF131	0.898	0.007	zinc finger protein 131 [Source:HGNC Symbol;Acc:HGNC:12915]	chr5:43121609-43176426	NM_001297548, NN	XM_017009835, XM_(	NP_001284477, NP_00	XP_005248416, XP_0052	protein_coding	ENSG0000017226	7690	12915
2470	ZNF185	-0.977	0.007	zinc finger protein 185 (LIM domain) [Source:HGNC Symbol;Acc:HGNC:12976]	chrX:152082985-152142024	NM_001178106, NN	XM_011531196, XM_(	NP_001171577, NP_00	XP_005274787, XP_0052	protein_coding	ENSG0000014739	7739	12976
2471	ZNF823	1.197	0.007	zinc finger protein 823 [Source:HGNC Symbol;Acc:HGNC:30936]	chr19:11832079-11849824	NM_001080493, NN	.	NP_001073962, NP_05	.	protein_coding	ENSG0000019793	55552	30936
2472	CDC5L	0.765	0.007	cell division cycle 5 like [Source:HGNC Symbol;Acc:HGNC:1743]	chr6:44355261-44418163	NM_001253	XM_006715289	NP_001244	.	protein_coding	ENSG0000009640	988	1743
2473	FAM188A	0.932	0.007	family with sequence similarity 188 member A [Source:HGNC Symbol;Acc:HGNC:23578]	chr10:15820168-15902519	NM_024948	XM_011519694, XM_(	NP_079224	XP_005252657, XP_0052	protein_coding	ENSG0000014848	80013	23578
2474	GATAD1	0.741	0.007	GATA zinc finger domain containing 1 [Source:HGNC Symbol;Acc:HGNC:29941]	chr7:92076766-92088150	NM_021167	.	NP_066990	.	protein_coding	ENSG0000015725	57798	29941
2475	NDUFV1	-0.635	0.007	NADH:ubiquinone oxidoreductase core subunit V1 [Source:HGNC Symbol;Acc:HGNC:77	chr11:67374322-67380006	NM_001166102, NN	.	NP_001159574, NP_00	.	protein_coding	ENSG0000016779	4723	7716
2476	PALD1	-0.654	0.007	phosphatase domain containing, paladin 1 [Source:HGNC Symbol;Acc:HGNC:23530]	chr10:72238576-72328205	NM_014431	XM_017016072, XM_(	NP_055246	XP_006717828, XP_0115	protein_coding	ENSG0000010771	27143	23530
2477	ACOT7	-0.652	0.007	acyl-CoA thioesterase 7 [Source:HGNC Symbol;Acc:HGNC:24157]	chr1:6324328-6454451	NM_007274, NM_1	.	NP_009205, NP_86365	.	protein_coding	ENSG0000009702	11332	24157
2478	ADAM22	1.077	0.007	ADAM metallopeptidase domain 22 [Source:HGNC Symbol;Acc:HGNC:201]	chr7:87563457-87832204	NM_001324417, NN	XM_017012339, XM_(	NP_001311346, NP_00	XP_005250502, XP_0067	protein_coding	ENSG0000000827	53616	201
2479	ARID2	0.979	0.007	AT-rich interaction domain 2 [Source:HGNC Symbol;Acc:HGNC:18037]	chr12:46123447-46301823	NM_152641	XM_006719272	NP_689854	XP_006719335	protein_coding	ENSG0000018907	196528	18037
2480	CLINT1	0.708	0.007	clathrin interactor 1 [Source:HGNC Symbol;Acc:HGNC:23186]	chr5:157212750-157286183	NM_001195555, NN	XM_011534701	NP_001182484, NP_00	XP_011533003	protein_coding	ENSG0000011328	9685	23186
2481	FGF21	-1.781	0.007	fibroblast growth factor 21 [Source:HGNC Symbol;Acc:HGNC:3678]	chr19:49258815-49261587	NM_019113	XM_005258731	NP_061986	XP_005258788	protein_coding	ENSG0000010555	26291	3678
2482	KLHL22	-0.858	0.007	kelch like family member 22 [Source:HGNC Symbol;Acc:HGNC:25888]	chr22:20795805-20850170	NM_032775	XM_017029021	NP_116164	XP_016884508, XP_0168	protein_coding	ENSG0000009991	84861	25888

2483	PGM2	1.055	0.007	phosphoglucomutase 2 [Source:HGNC Symbol;Acc:HGNC:8906]	chr4:37828254-37864558	NM_018290	XM_011513711	NP_060760	XP_011512013	protein_coding	ENSG0000016929	55276	8906
2484	PIR	-0.858	0.007	pirin [Source:HGNC Symbol;Acc:HGNC:30048]	chrX:15402920-15574652	NM_001018109, NN	.	NP_001018119, NP_00	.	protein_coding	ENSG0000008784	8544	30048
2485	SCRN2	-0.714	0.007	secernin 2 [Source:HGNC Symbol;Acc:HGNC:30381]	chr17:45908992-45918699	NM_001145023, NN	XM_005257776	NP_001138495, NP_61	XP_005257833, XP_0168	protein_coding	ENSG0000014129	90507	30381
2486	SS18	0.942	0.007	SS18, nBAF chromatin remodeling complex subunit [Source:HGNC Symbol;Acc:HGNC:11	chr18:23596216-23671181	NM_001007559, NN	XM_011526152, XM_0	NP_001007560, NP_00	XP_005258391, XP_0067	protein_coding	ENSG0000014138	6760	11340
2487	STRA13	-0.689	0.007	stimulated by retinoic acid 13 [Source:HGNC Symbol;Acc:HGNC:11422]	chr17:79976578-79980794	NM_001271006, NN	XM_005256339	NP_001257935, NP_00	XP_005256396	protein_coding	ENSG0000016968	201254	11422
2488	WASL	0.835	0.007	Wiskott-Aldrich syndrome like [Source:HGNC Symbol;Acc:HGNC:12735]	chr7:123321988-123389121	NM_003941	.	NP_003932	.	protein_coding	ENSG0000010629	8976	12735
2489	CALHM2	-0.854	0.007	calcium homeostasis modulator 2 [Source:HGNC Symbol;Acc:HGNC:23493]	chr10:105206542-105212159	NM_015916	XM_017016308, XM_0	NP_057000	XP_006717943, XP_0067	protein_coding	ENSG0000013817	51063	23493
2490	COX5B	-0.633	0.007	cytochrome c oxidase subunit 5B [Source:HGNC Symbol;Acc:HGNC:2269]	chr2:98262502-98264846	NM_001862	.	NP_001853	.	protein_coding	ENSG0000013594	1329	2269
2491	FBXO5	0.837	0.007	F-box protein 5 [Source:HGNC Symbol;Acc:HGNC:13584]	chr6:153291663-153304714	NM_001142522, NN	.	NP_001135994, NP_03	.	protein_coding	ENSG0000011202	26271	13584
2492	HNRNPH3	0.687	0.007	heterogeneous nuclear ribonucleoprotein H3 [Source:HGNC Symbol;Acc:HGNC:5043]	chr10:70042416-70102944	NM_001322434, NN	XM_006717817, XM_0	NP_001309363, NP_00	XP_005269805, XP_0052	protein_coding	ENSG0000009674	3189	5043
2493	MTMR2	0.858	0.007	myotubularin related protein 2 [Source:HGNC Symbol;Acc:HGNC:7450]	chr11:95566045-95657459	NM_001243571, NN	XM_011543059, XM_0	NP_001230500, NP_05	XP_005274431, XP_0052	protein_coding	ENSG0000008705	8898	7450
2494	TRIT1	0.810	0.007	tRNA isopentenyltransferase 1 [Source:HGNC Symbol;Acc:HGNC:20286]	chr1:40306693-40349178	NM_001312691, NN	XM_006710706, XM_0	NP_001299620, NP_00	XP_005271011, XP_0067	protein_coding	ENSG0000004351	54802	20286
2495	AASDHPPT	0.892	0.007	aminoadipate-semialdehyde dehydrogenase-phosphopantetheinyl transferase [Source:	chr11:105921824-105969437	NM_015423	.	NP_056238	.	protein_coding	ENSG0000014931	60496	14235
2496	CDC73	0.895	0.007	cell division cycle 73 [Source:HGNC Symbol;Acc:HGNC:16783]	chr1:193091146-193223031	NM_024529	.	NP_078805	.	protein_coding	ENSG0000013437	79577	16783
2497	FAM102A	-0.770	0.007	family with sequence similarity 102 member A [Source:HGNC Symbol;Acc:HGNC:31419]	chr9:130702857-130742792	NM_001035254, NN	XM_011518678, XM_0	NP_001030331, NP_97	XP_005252041, XP_0115	protein_coding	ENSG0000016710	399665	31419
2498	GSPT1	0.707	0.007	G1 to S phase transition 1 [Source:HGNC Symbol;Acc:HGNC:4621]	chr16:11961984-12009939	NM_001130006, NN	.	NP_001123478, NP_00	.	protein_coding	ENSG0000010334	2935	4621
2499	IQSEC2	-0.902	0.007	IQ motif and Sec7 domain 2 [Source:HGNC Symbol;Acc:HGNC:29059]	chrX:53262057-53350522	NM_001111125, NN	XM_011530774, XM_0	NP_001104595, NP_05	XP_006724642, XP_0067	protein_coding	ENSG0000012431	23096	29059
2500	JAK2	1.236	0.007	Janus kinase 2 [Source:HGNC Symbol;Acc:HGNC:6192]	chr9:4985244-5128183	NM_001322194, NN	.	NP_001309123, NP_00	.	protein_coding	ENSG0000009696	3717	6192
2501	KIF26A	-0.781	0.007	kinesin family member 26A [Source:HGNC Symbol;Acc:HGNC:20226]	chr14:104605059-104647231	NM_015656	XM_011536641	NP_056471	XP_011534943	protein_coding	ENSG0000006673	26153	20226
2502	LIG1	-0.698	0.007	DNA ligase 1 [Source:HGNC Symbol;Acc:HGNC:6598]	chr19:48618701-48673860	NM_000234, NM_0	XM_006723216, XM_0	NP_000225, NP_00127	XP_006723279	protein_coding	ENSG0000010548	3978	6598
2503	STOX2	1.004	0.007	storkhead box 2 [Source:HGNC Symbol;Acc:HGNC:25450]	chr4:184826508-184944679	NM_020225	.	NP_064610	XP_011530433, XP_0168	protein_coding	ENSG0000017332	56977	25450
2504	SYPL1	0.684	0.007	synaptophysin like 1 [Source:HGNC Symbol;Acc:HGNC:11507]	chr7:105730948-105753022	NM_006754, NM_1	XM_006716099	NP_006745, NP_87438	.	protein_coding	ENSG0000000828	6856	11507
2505	WDR81	-0.785	0.007	WD repeat domain 81 [Source:HGNC Symbol;Acc:HGNC:26600]	chr17:1619816-1641893	NM_001163673, NN	XM_011523651	NP_001157145, NP_00	XP_011521953, XP_0168	protein_coding	ENSG0000016771	124997	26600
2506	MAP6D1	-0.907	0.007	MAP6 domain containing 1 [Source:HGNC Symbol;Acc:HGNC:25753]	chr3:183533663-183543382	NM_024871	.	NP_079147	.	protein_coding	ENSG0000018083	79929	25753
2507	OAS2	0.968	0.007	2'-5'-oligoadenylate synthetase 2 [Source:HGNC Symbol;Acc:HGNC:8087]	chr12:113416199-113449528	NM_001032731, NN	.	NP_001027903, NP_00	.	protein_coding	ENSG0000011133	4939	8087
2508	AIDA	0.817	0.007	axin interactor, dorsalization associated [Source:HGNC Symbol;Acc:HGNC:25761]	chr1:222841354-222885866	NM_022831	.	NP_073742	.	protein_coding	ENSG0000018606	64853	25761
2509	CABYR	0.961	0.007	calcium binding tyrosine phosphorylation regulated [Source:HGNC Symbol;Acc:HGNC:15	chr18:21718919-21741567	NM_001308231, NN	XM_017025706, XM_0	NP_001295160, NP_03	XP_005258302, XP_0052	protein_coding	ENSG0000015404	26256	15569
2510	EIF4A2	0.721	0.007	eukaryotic translation initiation factor 4A2 [Source:HGNC Symbol;Acc:HGNC:3284]	chr3:186501335-186524484	NM_001967	.	NP_001958	.	protein_coding	ENSG0000015697	100302143	3284
2511	PLA2G16	-0.840	0.007	phospholipase A2 group XVI [Source:HGNC Symbol;Acc:HGNC:17825]	chr11:63340666-63381941	NM_001128203, NN	XM_006718426	NP_001121675, NP_00	XP_006718489	protein_coding	ENSG0000017648	11145	17825
2512	TCP11L1	0.911	0.007	t-complex 11 like 1 [Source:HGNC Symbol;Acc:HGNC:25655]	chr11:33060962-33095109	NM_001145541, NN	XM_011520207, XM_0	NP_001139013, NP_06	XP_011518506, XP_0115	protein_coding	ENSG0000017614	55346	25655
2513	UBALD1	-0.864	0.007	UBA like domain containing 1 [Source:HGNC Symbol;Acc:HGNC:29576]	chr16:4658883-4665028	NM_145253	.	NP_660296	XP_016878426	protein_coding	ENSG0000015344	124402	29576
2514	VIPR1	-0.870	0.007	vasoactive intestinal peptide receptor 1 [Source:HGNC Symbol;Acc:HGNC:12694]	chr3:42530790-42579065	NM_001251882, NN	XM_011534079, XM_0	NP_001238811, NP_00	XP_005265494, XP_0052	protein_coding	ENSG0000011481	7433	12694
2515	ARIH2	-0.670	0.007	ariadne RBR E3 ubiquitin protein ligase 2 [Source:HGNC Symbol;Acc:HGNC:690]	chr3:48955220-49023815	NM_006321	XM_011533275, XM_0	NP_001304262, NP_00	XP_005264855, XP_0115	protein_coding	ENSG0000017747	10425	690
2516	FHL3	-0.785	0.007	four and a half LIM domains 3 [Source:HGNC Symbol;Acc:HGNC:3704]	chr1:38462441-38471177	NM_001243878, NN	.	NP_001230807, NP_00	.	protein_coding	ENSG0000018338	2275	3704
2517	KCTD11	-0.669	0.007	potassium channel tetramerization domain containing 11 [Source:HGNC Symbol;Acc:HG	chr17:7255480-7258263	NM_001002914	.	NP_001002914	.	protein_coding	ENSG0000021385	147040	21302
2518	PSMD5	0.740	0.007	proteasome 26S subunit, non-ATPase 5 [Source:HGNC Symbol;Acc:HGNC:9563]	chr9:123578000-123605262	NM_001270427, NN	.	NP_001257356, NP_00	.	protein_coding	ENSG0000009526	5711	9563
2519	SFXN3	-0.670	0.007	sideroflexin 3 [Source:HGNC Symbol;Acc:HGNC:16087]	chr10:102790990-102801001	NM_030971	.	NP_112233	.	protein_coding	ENSG0000010781	81855	16087
2520	ZDHHC17	1.259	0.007	zinc finger DHHC-type containing 17 [Source:HGNC Symbol;Acc:HGNC:18412]	chr12:77157367-77247476	NM_015336	XM_011538083, XM_0	NP_056151	XP_005268807, XP_0115	protein_coding	ENSG0000018690	23390	18412
2521	ZYG11B	0.944	0.007	zyg-11 family member B, cell cycle regulator [Source:HGNC Symbol;Acc:HGNC:25820]	chr1:53192138-53293014	NM_024646	XM_006710898	NP_078922	XP_006710961, XP_0168	protein_coding	ENSG0000016237	79699	25820
2522	AFTPH	0.970	0.007	aftiphilin [Source:HGNC Symbol;Acc:HGNC:25951]	chr2:64751438-64820139	NM_001002243, NN	XM_005264380	NP_001002243, NP_06	XP_005264437	protein_coding	ENSG0000011984	54812	25951
2523	CHD9	1.312	0.007	chromodomain helicase DNA binding protein 9 [Source:HGNC Symbol;Acc:HGNC:25701	chr16:53088944-53363062	NM_001308319, NN	XM_017023723, XM_0	NP_001295248, NP_07	XP_005256225, XP_0052	protein_coding	ENSG0000017720	80205	25701
2524	GCNT1	0.861	0.007	glucosaminyl (N-acetyl) transferase 1, core 2 [Source:HGNC Symbol;Acc:HGNC:4203]	chr9:79056581-79122332	NM_001097633, NN	XM_017014621, XM_0	NP_001091102, NP_00	XP_006717115, XP_0168	protein_coding	ENSG0000018721	2650	4203
2525	GTPBP10	0.998	0.007	GTP binding protein 10 [Source:HGNC Symbol;Acc:HGNC:25106]	chr7:89975978-90020769	NM_001042717, NN	.	NP_001036182, NP_14	.	protein_coding	ENSG0000010579	101927446	25106
2526	KLC3	-1.143	0.007	kinesin light chain 3 [Source:HGNC Symbol;Acc:HGNC:20717]	chr19:45844010-45873876	NM_177417	XM_005258536	NP_803136	.	protein_coding	ENSG0000010489	147700	20717
2527	MOSPD1	0.872	0.007	motile sperm domain containing 1 [Source:HGNC Symbol;Acc:HGNC:25235]	chrX:134021655-134049297	NM_001306188, NN	XM_011531366, XM_0	NP_001293117, NP_06	XP_005262503, XP_0115	protein_coding	ENSG0000010192	56180	25235
2528	SP6	-0.774	0.007	Sp6 transcription factor [Source:HGNC Symbol;Acc:HGNC:14530]	chr17:45922273-45933240	NM_001258248, NN	XM_006722115	NP_001245177, NP_95	XP_006722178	protein_coding	ENSG0000018912	80320	14530
2529	CBX5	1.019	0.007	chromobox 5 [Source:HGNC Symbol;Acc:HGNC:1555]	chr12:54624723-54673886	NM_001127321, NN	.	NP_001120793, NP_00	.	protein_coding	ENSG0000009491	100616400	1555
2530	KCNJ12	-0.820	0.007	potassium voltage-gated channel subfamily J member 12 [Source:HGNC Symbol;Acc:HG	chr17:21279508-21323184	NM_021012	XM_011523831, XM_0	NP_066292	XP_005256682, XP_0115	protein_coding	ENSG0000018418	3768	6258
2531	RNF123	-1.215	0.007	ring finger protein 123 [Source:HGNC Symbol;Acc:HGNC:21148]	chr3:49726931-49761384	NM_022064	XM_017007018, XM_0	NP_071347	XP_006713348, XP_0115	protein_coding	ENSG0000016406	63891	21148
2532	SH3BP4	0.679	0.007	SH3 domain binding protein 4 [Source:HGNC Symbol;Acc:HGNC:10826]	chr2:235860616-235964358	NM_014521	XM_011510894, XM_0	NP_055336	XP_011509193, XP_0115	protein_coding	ENSG0000013014	23677	10826
2533	TMF1	0.890	0.007	TATA element modulatory factor 1 [Source:HGNC Symbol;Acc:HGNC:11870]	chr3:69068977-69101454	NM_007114	XM_011534056	NP_009045	XP_011532358	protein_coding	ENSG0000014474	7110	11870
2534	ZNF510	0.999	0.007	zinc finger protein 510 [Source:HGNC Symbol;Acc:HGNC:29161]	chr9:99518146-99540338	NM_014930	XM_005251808, XM_0	NP_001300988, NP_00	XP_005251864, XP_0052	protein_coding	ENSG0000008138	22869	29161
2535	AIP	-0.669	0.007	aryl hydrocarbon receptor interacting protein [Source:HGNC Symbol;Acc:HGNC:358]	chr11:67250511-67258574	NM_001302960, NN	.	NP_001289889, NP_00	.	protein_coding	ENSG0000011071	9049	358
2536	METTL8	1.042	0.007	methyltransferase like 8 [Source:HGNC Symbol;Acc:HGNC:25856]	chr2:172173914-172341562	NM_024770	XM_011511874, XM_0	NP_001308083, NP_00	XP_006712826, XP_0115	protein_coding	ENSG0000012360	79828	25856

2537	RBMS1	0.884	0.007	RNA binding motif single stranded interacting protein 1 [Source:HGNC Symbol;Acc:HGN	chr2:161128661-161350305	NM_002897, NM_0	XM_017004626, XM_(	NP_002888, NP_05852	XP_005246794, XP_0052	protein_coding	ENSG0000015325	100616364	9907
2538	S100A4	-0.663	0.007	S100 calcium binding protein A4 [Source:HGNC Symbol;Acc:HGNC:10494]	chr1:153516088-153522612	NM_002961, NM_0	.	NP_002952, NP_06242	.	protein_coding	ENSG0000019615	6275	10494
2539	SMTNL2	-1.319	0.007	smoothelin like 2 [Source:HGNC Symbol;Acc:HGNC:24764]	chr17:4487293-4511614	NM_001114974, NN	XM_006721515, XM_(	NP_001108446, NP_94	XP_005256679, XP_0067	protein_coding	ENSG0000018817	342527	24764
2540	ZNF440	1.009	0.007	zinc finger protein 440 [Source:HGNC Symbol;Acc:HGNC:20874]	chr19:11925098-11946016	NM_152357	XM_005259731	NP_689570	XP_005259788, XP_0168	protein_coding	ENSG0000017129	126070	20874
2541	FBXO28	1.027	0.007	F-box protein 28 [Source:HGNC Symbol;Acc:HGNC:29046]	chr1:224301788-224349749	NM_001136115, NN	.	NP_001129587, NP_05	.	protein_coding	ENSG0000014375	23219	29046
2542	MCTP1	1.200	0.007	multiple C2 and transmembrane domain containing 1 [Source:HGNC Symbol;Acc:HGNC:	chr5:94040804-94620279	NM_001002796, NN	XM_011543650, XM_(	NP_001002796, NP_00	XP_005272139, XP_0052	protein_coding	ENSG0000017547	79772	26183
2543	P3H4	-0.790	0.007	prolyl 3-hydroxylase family member 4 (non-enzymatic) [Source:HGNC Symbol;Acc:HGN(	chr17:39958198-39968855	NM_006455	XM_006721640, XM_(	NP_006446	XP_006721703	protein_coding	ENSG0000014169	10609	16946
2544	PICALM	0.747	0.007	phosphatidylinositol binding clathrin assembly protein [Source:HGNC Symbol;Acc:HGN(	chr11:85668726-85780924	NM_001008660, NN	XM_011545293, XM_(	NP_001008660, NP_00	XP_005274379, XP_0052	protein_coding	ENSG0000007392	8301	15514
2545	SLC29A2	-0.770	0.007	solute carrier family 29 member 2 [Source:HGNC Symbol;Acc:HGNC:11004]	chr11:66129991-66139685	NM_001300868, NN	XM_017017632, XM_(	NP_001287797, NP_00	XP_011543276, XP_0115	protein_coding	ENSG0000017466	3177	11004
2546	SPAG1	0.902	0.007	sperm associated antigen 1 [Source:HGNC Symbol;Acc:HGNC:11212]	chr8:101170133-101254131	NM_003114, NM_1	XM_017013755, XM_(	NP_003105, NP_75736	XP_011515542, XP_0115	protein_coding	ENSG0000010445	6674	11212
2547	CD22	-1.103	0.007	CD22 molecule [Source:HGNC Symbol;Acc:HGNC:1643]	chr19:35820071-35838258	NM_001185099, NN	.	NP_001172028, NP_00	.	protein_coding	ENSG0000001212	100847070	1643
2548	E2F8	0.891	0.007	E2F transcription factor 8 [Source:HGNC Symbol;Acc:HGNC:24727]	chr11:19245609-19263167	NM_001256371, NN	XM_011520367	NP_001243300, NP_00	XP_011518669	protein_coding	ENSG0000012917	79733	24727
2549	ELP6	-0.765	0.007	elongator acetyltransferase complex subunit 6 [Source:HGNC Symbol;Acc:HGNC:25976]	chr3:47537129-47555251	NM_001031703	XM_011533853, XM_(	NP_001026873	XP_005265298, XP_0115	protein_coding	ENSG0000016383	54859	25976
2550	YIPF3	-0.708	0.007	Yip1 domain family member 3 [Source:HGNC Symbol;Acc:HGNC:21023]	chr6:43479564-43484728	NM_015388	XM_011514458, XM_(	NP_056203	XP_005249046, XP_0052	protein_coding	ENSG0000013720	25844	21023
2551	C6orf1	-0.867	0.007	chromosome 6 open reading frame 1 [Source:HGNC Symbol;Acc:HGNC:1340]	chr6:34214156-34217247	NM_001008703, NN	.	NP_001008703, NP_00	.	protein_coding	ENSG0000018657	221491	1340
2552	NDUFA13	-0.662	0.007	NADH:ubiquinone oxidoreductase subunit A13 [Source:HGNC Symbol;Acc:HGNC:17194]	chr19:19626563-19648390	NM_015965	.	NP_057049	.	protein_coding	ENSG0000018601	51079	17194
2553	PTDSS2	-0.735	0.007	phosphatidylserine synthase 2 [Source:HGNC Symbol;Acc:HGNC:15463]	chr11:450279-491387	NM_030783	XM_011520392, XM_(	NP_001316473, NP_11	XP_011518693, XP_0115	protein_coding	ENSG0000017491	81490	15463
2554	B2M	-0.650	0.007	beta-2-microglobulin [Source:HGNC Symbol;Acc:HGNC:914]	chr15:45003674-45011075	NM_004048	.	NP_004039	XP_005254606	protein_coding	ENSG0000016671	567	914
2555	E2F3	0.867	0.007	E2F transcription factor 3 [Source:HGNC Symbol;Acc:HGNC:3115]	chr6:20402136-20493946	NM_001243076, NN	XM_011514326, XM_(	NP_001230005, NP_00	XP_005248922, XP_0115	protein_coding	ENSG0000011224	1871	3115
2556	TRAM1	0.788	0.007	translocation associated membrane protein 1 [Source:HGNC Symbol;Acc:HGNC:20568]	chr8:71485676-71520604	NM_001317805, NN	XM_011517496	NP_001304733, NP_00	XP_011515798, XP_0168	protein_coding	ENSG0000006716	23471	20568
2557	ZNF326	0.917	0.007	zinc finger protein 326 [Source:HGNC Symbol;Acc:HGNC:14104]	chr1:90460673-90501090	NM_001320185, NN	XM_011541290, XM_(	NP_001307114, NP_86	XP_005270836, XP_0115	protein_coding	ENSG0000016266	284695	14104
2558	KLHDC9	-1.046	0.008	kelch domain containing 9 [Source:HGNC Symbol;Acc:HGNC:28489]	chr1:161068183-161070136	NM_001007255, NN	.	NP_001007256, NP_68	.	protein_coding	ENSG0000016275	126823	28489
2559	NBL1	-0.736	0.008	neuroblastoma 1, DAN family BMP antagonist [Source:HGNC Symbol;Acc:HGNC:7650]	chr1:19923470-19984949	NM_001204084, NN	.	NP_001191013, NP_00	.	protein_coding	ENSG0000015874	4681	7650
2560	NEK3	1.401	0.008	NIMA related kinase 3 [Source:HGNC Symbol;Acc:HGNC:7746]	chr13:52706774-52733996	NM_001146099, NN	.	NP_001139571, NP_00	.	protein_coding	ENSG0000013609	4752	7746
2561	VPS37A	1.703	0.008	VPS37A, ESCRT-I subunit [Source:HGNC Symbol;Acc:HGNC:24928]	chr8:17082472-17155533	NM_001145152, NN	XM_017013024, XM_(	NP_001138624, NP_68	XP_005273457, XP_0052	protein_coding	ENSG0000015597	137492	24928
2562	ACAP3	-0.767	0.008	ArfGAP with coiled-coil, ankyrin repeat and PH domains 3 [Source:HGNC Symbol;Acc:HG	chr1:1227755-1243398	NM_030649	XM_011540609, XM_(	NP_085152	XP_005244772, XP_0115	protein_coding	ENSG0000013158	116983	16754
2563	ANKRD1	0.684	0.008	ankyrin repeat domain 1 [Source:HGNC Symbol;Acc:HGNC:15819]	chr10:92671852-92681033	NM_014391	.	NP_055206	.	protein_coding	ENSG0000014867	27063	15819
2564	CWC22	0.887	0.008	CWC22 homolog, spliceosome-associated protein [Source:HGNC Symbol;Acc:HGNC:293	chr2:180809602-180871780	NM_020943	XM_005246726	NP_065994	XP_005246783	protein_coding	ENSG0000016351	57703	29322
2565	TERT	1.177	0.008	telomerase reverse transcriptase [Source:HGNC Symbol;Acc:HGNC:11730]	chr5:1253281-1295162	NM_001193376, NN	XM_011514106, XM_(	NP_001180305, NP_93	.	protein_coding	ENSG0000016436	7015	11730
2566	ARSD	-0.936	0.008	arylsulfatase D [Source:HGNC Symbol;Acc:HGNC:717]	chrX:2822010-2847392	NM_001669	XM_005274515, XM_(	NP_001660	XP_005274571, XP_0052	protein_coding	ENSG0000000675	414	717
2567	DNAAF3	-0.924	0.008	dynein axonemal assembly factor 3 [Source:HGNC Symbol;Acc:HGNC:30492]	chr19:55670028-55678090	NM_001256714, NN	.	NP_001243643, NP_00	.	protein_coding	ENSG0000016764	352909	30492
2568	PLEKHA5	0.988	0.008	pleckstrin homology domain containing A5 [Source:HGNC Symbol;Acc:HGNC:30036]	chr12:19282685-19529334	NM_001143821, NN	XM_017019497, XM_(	NP_001137293, NP_00	XP_005253457, XP_0067	protein_coding	ENSG0000005212	54477	30036
2569	PPP6R3	0.723	0.008	protein phosphatase 6 regulatory subunit 3 [Source:HGNC Symbol;Acc:HGNC:1173]	chr11:68228198-68382802	NM_001164160, NN	XM_017017982, XM_(	NP_001157632, NP_00	XP_006718671, XP_0067	protein_coding	ENSG0000011007	55291	1173
2570	USE1	-0.841	0.008	unconventional SNARE in the ER 1 [Source:HGNC Symbol;Acc:HGNC:30882]	chr19:17326167-17330638	NM_018467	XM_017026976	NP_060937	XP_016882464, XP_0168	protein_coding	ENSG0000005350	55850	30882
2571	APOLD1	0.967	0.008	apolipoprotein L domain containing 1 [Source:HGNC Symbol;Acc:HGNC:25268]	chr12:12878864-12944400	NM_001130415, NN	.	NP_001123887, NP_11	.	protein_coding	ENSG0000017887	81575	25268
2572	APOO	-0.866	0.008	apolipoprotein O [Source:HGNC Symbol;Acc:HGNC:28727]	chrX:23851469-23957624	NM_024122	XM_011545587, XM_(	NP_077027	XP_011543886, XP_0115	protein_coding	ENSG0000018483	79135	28727
2573	BMPR2	0.792	0.008	bone morphogenetic protein receptor type 2 [Source:HGNC Symbol;Acc:HGNC:1078]	chr2:203241658-203432474	NM_001204	XM_011511688, XM_(	NP_001195	XP_011509989, XP_0115	protein_coding	ENSG0000020421	659	1078
2574	KIF3A	0.987	0.008	kinesin family member 3A [Source:HGNC Symbol;Acc:HGNC:6319]	chr5:132028319-132073330	NM_001300791, NN	XM_006714526	NP_001287720, NP_00	XP_006714589, XP_0168	protein_coding	ENSG0000013143	11127	6319
2575	MAN1A2	0.882	0.008	mannosidase alpha class 1A member 2 [Source:HGNC Symbol;Acc:HGNC:6822]	chr1:117910070-118071494	NM_006699	XM_011540536, XM_(	NP_006690	XP_006710365, XP_0115	protein_coding	ENSG0000019816	10905	6822
2576	PIH1D1	-0.843	0.008	PIH1 domain containing 1 [Source:HGNC Symbol;Acc:HGNC:26075]	chr19:49949555-49974305	NM_017916	XM_006723254	NP_060386	XP_006723317	protein_coding	ENSG0000010487	55011	26075
2577	RSBN1	1.150	0.008	round spermatid basic protein 1 [Source:HGNC Symbol;Acc:HGNC:25642]	chr1:114304453-114355098	NM_018364	.	NP_060834	.	protein_coding	ENSG0000008101	54665	25642
2578	TNFRSF10B	0.758	0.008	TNF receptor superfamily member 10b [Source:HGNC Symbol;Acc:HGNC:11905]	chr8:22844929-22926692	NM_003842, NM_1	.	NP_003833, NP_67171	.	protein_coding	ENSG0000012088	8795	11905
2579	BCOR	-0.671	0.008	BCL6 corepressor [Source:HGNC Symbol;Acc:HGNC:20893]	chrX:39909067-40036582	NM_001123383, NN	XM_011543931, XM_(	NP_001116855, NP_00	XP_005272673, XP_0052	protein_coding	ENSG0000018333	54880	20893
2580	CCT2	0.624	0.008	chaperonin containing TCP1 subunit 2 [Source:HGNC Symbol;Acc:HGNC:1615]	chr12:69979113-69995350	NM_001198842, NN	.	NP_001185771, NP_00	.	protein_coding	ENSG0000016622	10576	1615
2581	CRYBB2	-1.023	0.008	crystallin beta B2 [Source:HGNC Symbol;Acc:HGNC:2398]	chr22:25615488-25627836	NM_000496	XM_011529901, XM_(	NP_000487	XP_006724204, XP_0115	protein_coding	ENSG0000024475	1415	2398
2582	LTBP4	-0.658	0.008	latent transforming growth factor beta binding protein 4 [Source:HGNC Symbol;Acc:HG	chr19:41099071-41135723	NM_001042544, NN	XM_011527387, XM_(	NP_001036009, NP_00	XP_011525678, XP_0115	protein_coding	ENSG0000009000	8425	6717
2583	ZNHIT6	0.875	0.008	zinc finger HIT-type containing 6 [Source:HGNC Symbol;Acc:HGNC:26089]	chr1:86115105-86174116	NM_001170670, NN	.	NP_001164141, NP_06	.	protein_coding	ENSG0000011717	54680	26089
2584	CA11	-0.800	0.008	carbonic anhydrase 11 [Source:HGNC Symbol;Acc:HGNC:1370]	chr19:49141198-49149569	NM_001217	.	NP_001208	.	protein_coding	ENSG0000006318	770	1370
2585	CDH13	0.842	0.008	cadherin 13 [Source:HGNC Symbol;Acc:HGNC:1753]	chr16:82660407-83834245	NM_001220488, NN	.	NP_001207417, NP_00	.	protein_coding	ENSG0000014094	1012	1753
2586	COG3	0.857	0.008	component of oligomeric golgi complex 3 [Source:HGNC Symbol;Acc:HGNC:18619]	chr13:46039059-46110765	NM_031431	XM_011535268, XM_(	NP_113619	XP_011533568	protein_coding	ENSG0000013615	83548	18619
2587	CTSZ	-0.623	0.008	cathepsin Z [Source:HGNC Symbol;Acc:HGNC:2547]	chr20:57570239-57582302	NM_001336	.	NP_001327	.	protein_coding	ENSG0000010116	1522	2547
2588	MTDH	0.780	0.008	metadherin [Source:HGNC Symbol;Acc:HGNC:29608]	chr8:98656406-98740998	NM_178812	XM_011517370, XM_(	NP_848927	XP_005251156, XP_0115	protein_coding	ENSG0000014764	92140	29608
2589	OSTM1	0.879	0.008	osteopetrosis associated transmembrane protein 1 [Source:HGNC Symbol;Acc:HGNC:21	chr6:108362612-108395941	NM_014028	XM_011535775	NP_054747	.	protein_coding	ENSG0000008108	28962	21652
2590	QPRT	-0.877	0.008	quinolinate phosphoribosyltransferase [Source:HGNC Symbol;Acc:HGNC:9755]	chr16:29674299-29710020	NM_014298	XM_017023101, XM_(	NP_001305179, NP_05	XP_005255280, XP_0168	protein_coding	ENSG0000010348	105369247	9755

2591	TUBE1	1.103	0.008	tubulin epsilon 1 [Source:HGNC Symbol;Acc:HGNC:20775]	chr6:112375274-112408716	NM_016262	XM_011535875	NP_057346	XP_011534177	protein_coding	ENSG0000007493	51175	20775
2592	KLF15	-1.120	0.008	Kruppel like factor 15 [Source:HGNC Symbol;Acc:HGNC:14536]	chr3:126061477-126076285	NM_014079	XM_005247400	NP_054798	XP_005247457, XP_0115	protein_coding	ENSG0000016388	28999	14536
2593	RPL3	-0.682	0.008	ribosomal protein L3 [Source:HGNC Symbol;Acc:HGNC:10332]	chr22:39708886-39715776	NM_000967, NM_0	.	NP_000958, NP_00102	.	protein_coding	ENSG0000010031	6122	10332
2594	SOS2	1.041	0.008	SOS Ras/Rho guanine nucleotide exchange factor 2 [Source:HGNC Symbol;Acc:HGNC:11	chr14:50583837-50698276	NM_006939	XM_011537103, XM_0	NP_008870	.	protein_coding	ENSG0000010048	6655	11188
2595	ZKSCAN8	0.977	0.008	zinc finger with KRAB and SCAN domains 8 [Source:HGNC Symbol;Acc:HGNC:12983]	chr6:28109715-28127250	NM_001278119, NN	XM_017011266, XM_0	NP_001265048, NP_00	XP_011513172, XP_0115	protein_coding	ENSG0000019831	7745	12983
2596	ACVR1B	0.630	0.008	activin A receptor type 1B [Source:HGNC Symbol;Acc:HGNC:172]	chr12:52345450-52390862	NM_004302, NM_0	.	NP_004293, NP_06473	XP_016875688, XP_0168	protein_coding	ENSG0000013550	91	172
2597	ALG6	1.187	0.008	ALG6, alpha-1,3-glucosyltransferase [Source:HGNC Symbol;Acc:HGNC:23157]	chr1:63833260-63904233	NM_013339	.	NP_037471	.	protein_coding	ENSG0000008803	29929	23157
2598	DHX58	-1.317	0.008	DEXH-box helicase 58 [Source:HGNC Symbol;Acc:HGNC:29517]	chr17:40253421-40264751	NM_024119	XM_017025060, XM_0	NP_077024	XP_016880548, XP_0168	protein_coding	ENSG0000010877	79132	29517
2599	DYNC1LI2	0.688	0.008	dynein cytoplasmic 1 light intermediate chain 2 [Source:HGNC Symbol;Acc:HGNC:2966]	chr16:66754795-66785701	NM_001286157, NN	.	NP_001273086, NP_00	XP_016878497	protein_coding	ENSG0000013572	1783	2966
2600	EVI5	1.020	0.008	ecotropic viral integration site 5 [Source:HGNC Symbol;Acc:HGNC:3501]	chr1:92974252-93257961	NM_001308248, NN	XM_011542107, XM_0	NP_001295177, NP_00	XP_011540408, XP_0115	protein_coding	ENSG0000006720	7813	3501
2601	FAH	-0.787	0.008	fumarylacetoacetate hydrolase [Source:HGNC Symbol;Acc:HGNC:3579]	chr15:80445121-80479288	NM_000137	.	NP_000128	.	protein_coding	ENSG0000010387	2184	3579
2602	MTERF3	0.893	0.008	mitochondrial transcription termination factor 3 [Source:HGNC Symbol;Acc:HGNC:2425	chr8:97251625-97273838	NM_001286643, NN	XM_011517055, XM_0	NP_001273572, NP_05	XP_005250983, XP_0067	protein_coding	ENSG0000015646	51001	24258
2603	SRPK2	0.721	0.008	SRSF protein kinase 2 [Source:HGNC Symbol;Acc:HGNC:11306]	chr7:104756822-105029341	NM_001278273, NN	XM_011516540, XM_0	NP_001265202, NP_87	XP_005250607, XP_0052	protein_coding	ENSG0000013525	6733	11306
2604	BCAT2	-0.680	0.008	branched chain amino acid transaminase 2 [Source:HGNC Symbol;Acc:HGNC:977]	chr19:49298318-49314286	NM_001164773, NN	.	NP_001158245, NP_00	.	protein_coding	ENSG0000010555	587	977
2605	ERP29	-0.651	0.008	endoplasmic reticulum protein 29 [Source:HGNC Symbol;Acc:HGNC:13799]	chr12:112451119-112461255	NM_001034025, NN	.	NP_001029197, NP_00	XP_016874209	protein_coding	ENSG0000008924	10961	13799
2606	NUDT2	-0.834	0.008	nudix hydrolase 2 [Source:HGNC Symbol;Acc:HGNC:8049]	chr9:34329503-34343711	NM_001161, NM_0	.	NP_001152, NP_00123	.	protein_coding	ENSG0000016497	318	8049
2607	NUP43	0.824	0.008	nucleoporin 43 [Source:HGNC Symbol;Acc:HGNC:21182]	chr6:150045450-150067708	NM_198887	XM_005266960	NP_942590	XP_005267017	protein_coding	ENSG0000012025	348995	21182
2608	ZNF283	1.247	0.008	zinc finger protein 283 [Source:HGNC Symbol;Acc:HGNC:13077]	chr19:44331443-44356169	NM_001297752, NN	.	NP_001284681, NP_86	XP_005258841, XP_0052	protein_coding	ENSG0000016763	284349	13077
2609	ARRDC1	-0.671	0.008	arrestin domain containing 1 [Source:HGNC Symbol;Acc:HGNC:28633]	chr9:140500105-140509810	NM_152285	XM_005266121, XM_0	NP_001304897, NP_68	XP_005266176, XP_0168	protein_coding	ENSG0000019707	92714	28633
2610	MELK	0.705	0.008	maternal embryonic leucine zipper kinase [Source:HGNC Symbol;Acc:HGNC:16870]	chr9:36572858-36677680	NM_001256685, NN	XM_011518085, XM_0	NP_001243614, NP_00	XP_011516378, XP_0115	protein_coding	ENSG0000016530	9833	16870
2611	ORC6	0.754	0.008	origin recognition complex subunit 6 [Source:HGNC Symbol;Acc:HGNC:17151]	chr16:46723557-46732306	NM_014321	.	NP_055136	.	protein_coding	ENSG0000009165	23594	17151
2612	LMF1	-0.986	0.008	lipase maturation factor 1 [Source:HGNC Symbol;Acc:HGNC:14154]	chr16:903633-1020999	NM_022773	XM_011522617, XM_0	NP_073610	XP_006720991, XP_0067	protein_coding	ENSG0000010322	64788	14154
2613	MAF1	-0.659	0.008	MAF1 homolog, negative regulator of RNA polymerase III [Source:HGNC Symbol;Acc:HG	chr8:145159401-145162514	NM_032272	.	NP_115648	XP_016869393	protein_coding	ENSG0000017963	84232	24966
2614	SERPING1	-1.065	0.008	serpin family G member 1 [Source:HGNC Symbol;Acc:HGNC:1228]	chr11:57364990-57382326	NM_000062, NM_0	.	NP_000053, NP_00102	.	protein_coding	ENSG0000014913	710	1228
2615	YWHAZ	0.627	0.008	tyrosine 3-monooxygenase/tryptophan 5-monooxygenase activation protein zeta [Sour	chr8:101928752-101965569	NM_001135699, NN	XM_017013810, XM_0	NP_001129171, NP_00	XP_005251118, XP_0052	protein_coding	ENSG0000016492	7534	12855
2616	ACBD3	0.889	0.008	acyl-CoA binding domain containing 3 [Source:HGNC Symbol;Acc:HGNC:15453]	chr1:226332379-226374431	NM_022735	.	NP_073572	.	protein_coding	ENSG0000018282	64746	15453
2617	MARCH6	0.856	0.008	membrane associated ring-CH-type finger 6 [Source:HGNC Symbol;Acc:HGNC:30550]	chr5:10353814-10440500	NM_001270660, NN	XM_011513937, XM_0	NP_001257589, NP_00	XP_011512234, XP_0115	protein_coding	ENSG0000014549	10299	30550
2618	PPP1R37	-0.717	0.008	protein phosphatase 1 regulatory subunit 37 [Source:HGNC Symbol;Acc:HGNC:27607]	chr19:45596219-45650543	NM_019121	XM_006723159	NP_061994	XP_006723222	protein_coding	ENSG0000010486	284352	27607
2619	ERP44	0.712	0.008	endoplasmic reticulum protein 44 [Source:HGNC Symbol;Acc:HGNC:18311]	chr9:102741460-102861322	NM_015051	.	NP_055866	.	protein_coding	ENSG0000002331	23071	18311
2620	FAM126A	1.312	0.008	family with sequence similarity 126 member A [Source:HGNC Symbol;Acc:HGNC:24587]	chr7:22973829-23053748	NM_032581	XM_011515589, XM_0	NP_115970	XP_005249951, XP_0052	protein_coding	ENSG0000012259	84668	24587
2621	KRT23	-0.610	0.008	keratin 23 [Source:HGNC Symbol;Acc:HGNC:6438]	chr17:39078947-39093702	NM_001282433, NN	XM_011524595, XM_0	NP_001269362, NP_05	XP_005257257, XP_0115	protein_coding	ENSG0000010824	25984	6438
2622	TSPAN4	-0.816	0.008	tetraspanin 4 [Source:HGNC Symbol;Acc:HGNC:11859]	chr11:842807-867116	NM_001025234, NN	XM_011520341, XM_0	NP_001020405, NP_00	XP_005253159, XP_0052	protein_coding	ENSG0000021406	7106	11859
2623	UCKL1	-0.701	0.008	uridine-cytidine kinase 1 like 1 [Source:HGNC Symbol;Acc:HGNC:15938]	chr20:62571181-62587768	NM_001193379, NN	XM_017027903, XM_0	NP_001180308, NP_06	XP_005260273, XP_0067	protein_coding	ENSG0000019827	100302137	15938
2624	UNC93B1	-0.687	0.008	unc-93 homolog B1 (C. elegans) [Source:HGNC Symbol;Acc:HGNC:13481]	chr11:67758574-67771593	NM_030930	.	NP_112192	.	protein_coding	ENSG0000011005	81622	13481
2625	ZCCHC2	1.030	0.008	zinc finger CCHC-type containing 2 [Source:HGNC Symbol;Acc:HGNC:22916]	chr18:60190239-60245816	NM_017742	XM_011526044, XM_0	NP_060212	XP_006722556, XP_0115	protein_coding	ENSG0000014166	54877	22916
2626	GLI3	0.682	0.008	GLI family zinc finger 3 [Source:HGNC Symbol;Acc:HGNC:4319]	chr7:42000547-42276658	NM_000168	XM_011515274, XM_0	NP_000159	XP_005249760, XP_0052	protein_coding	ENSG0000010657	2737	4319
2627	NUS1	0.818	0.008	NUS1 dehydrodolichyl diphosphate synthase subunit [Source:HGNC Symbol;Acc:HGNC:2	chr6:117996664-118031803	NM_138459	.	NP_612468	.	protein_coding	ENSG0000015398	116150	21042
2628	TMEM160	-0.785	0.008	transmembrane protein 160 [Source:HGNC Symbol;Acc:HGNC:26042]	chr19:47549164-47551888	NM_017854	.	NP_060324	.	protein_coding	ENSG0000013074	54958	26042
2629	MRPL23	-0.670	0.008	mitochondrial ribosomal protein L23 [Source:HGNC Symbol;Acc:HGNC:10322]	chr11:1968507-2005752	NM_021134	XM_011520276	NP_066957	XP_006718334, XP_0115	protein_coding	ENSG0000021402	107987373	10322
2630	NT5E	1.245	0.008	5'-nucleotidase ecto [Source:HGNC Symbol;Acc:HGNC:8021]	chr6:86159301-86205509	NM_001204813, NN	.	NP_001191742, NP_00	.	protein_coding	ENSG0000013531	4907	8021
2631	PAK1IP1	0.827	0.008	PAK1 interacting protein 1 [Source:HGNC Symbol;Acc:HGNC:20882]	chr6:10671650-10710015	NM_017906	XM_005249204	NP_060376	XP_005249261	protein_coding	ENSG0000011184	55003	20882
2632	STK39	0.775	0.008	serine/threonine kinase 39 [Source:HGNC Symbol;Acc:HGNC:17717]	chr2:168810529-169104651	NM_013233	XM_011510967, XM_0	NP_037365	XP_005246522, XP_0168	protein_coding	ENSG0000019864	27347	17717
2633	CDKN2A	-0.728	0.008	cyclin dependent kinase inhibitor 2A [Source:HGNC Symbol;Acc:HGNC:1787]	chr9:21967751-21995300	NM_000077, NM_0	XM_011517679, XM_0	NP_000068, NP_00118	XP_005251400, XP_0115	protein_coding	ENSG0000014788	1029	1787
2634	FAM76B	1.066	0.008	family with sequence similarity 76 member B [Source:HGNC Symbol;Acc:HGNC:28492]	chr11:95502105-95522955	NM_144664	XM_011542613, XM_0	NP_653265	XP_005273832, XP_0052	protein_coding	ENSG0000007745	143684	28492
2635	LACC1	1.304	0.008	laccase domain containing 1 [Source:HGNC Symbol;Acc:HGNC:26789]	chr13:44410487-44468068	NM_001128303, NN	XM_017020394, XM_0	NP_001121775, NP_69	XP_005266318, XP_0067	protein_coding	ENSG0000017963	144811	26789
2636	LSM1	0.897	0.008	LSM1 homolog, mRNA degradation associated [Source:HGNC Symbol;Acc:HGNC:20472]	chr8:38020838-38070819	NM_014462	.	NP_055277	.	protein_coding	ENSG0000017532	27257	20472
2637	NECTIN1	-0.689	0.008	nectin cell adhesion molecule 1 [Source:HGNC Symbol;Acc:HGNC:9706]	chr11:119508807-119599794	NM_002855, NM_2	.	NP_002846, NP_97603	.	protein_coding	ENSG0000011040	5818	9706
2638	PIK3R2	-0.699	0.008	phosphoinositide-3-kinase regulatory subunit 2 [Source:HGNC Symbol;Acc:HGNC:8980]	chr19:18263927-18281350	NM_005027	.	NP_005018	.	protein_coding	ENSG0000010564	5296	8980
2639	UROS	-0.842	0.008	uroporphyrinogen III synthase [Source:HGNC Symbol;Acc:HGNC:12592]	chr10:127477146-127511796	NM_000375, NM_0	XM_006717960, XM_0	NP_000366, NP_00131	XP_005270198	protein_coding	ENSG0000018869	7390	12592
2640	ZNF273	1.202	0.008	zinc finger protein 273 [Source:HGNC Symbol;Acc:HGNC:13067]	chr7:64363624-64389635	NM_021148	XM_017011688	NP_066971	XP_016867177	protein_coding	ENSG0000019803	10793	13067
2641	AHCY	-0.620	0.008	adenosylhomocysteinase [Source:HGNC Symbol;Acc:HGNC:343]	chr20:32868073-32899608	NM_000687, NM_0	XM_011528660, XM_0	NP_000678, NP_00115	XP_005260373, XP_0052	protein_coding	ENSG0000010144	191	343
2642	MUC1	-0.999	0.008	mucin 1, cell surface associated [Source:HGNC Symbol;Acc:HGNC:7508]	chr1:155158299-155162706	NM_001018016, NN	.	NP_001018016, NP_00	.	protein_coding	ENSG0000018549	4582	7508
2643	PRSS8	-1.996	0.008	protease, serine 8 [Source:HGNC Symbol;Acc:HGNC:9491]	chr16:31142753-31147083	NM_002773	.	NP_002764	.	protein_coding	ENSG0000005234	5652	9491
2644	VPS51	-0.740	0.008	VPS51, GARP complex subunit [Source:HGNC Symbol;Acc:HGNC:1172]	chr11:64863630-64883828	NM_013265	.	NP_037397	.	protein_coding	ENSG0000014982	738	1172

2645	ZMAT3	1.060	0.008	zinc finger matrin-type 3 [Source:HGNC Symbol;Acc:HGNC:29983]	chr3:178735010-178790067	NM_022470, NM_1	XM_011513073, XM_0	NP_071915, NP_68942	XP_005247763, XP_0115	protein_coding	ENSG0000017266	64393	29983
2646	ZNF845	1.313	0.008	zinc finger protein 845 [Source:HGNC Symbol;Acc:HGNC:25112]	chr19:53833927-53895470	NM_138374	XM_006723470, XM_0	NP_001308451, NP_00	XP_005259458	protein_coding	ENSG0000021379	91664	25112
2647	ALS2	0.933	0.008	ALS2, alsin Rho guanine nucleotide exchange factor [Source:HGNC Symbol;Acc:HGNC:44	chr2:202565276-202645912	NM_001135745, NM	XM_017004569, XM_0	NP_001129217, NP_06	XP_006712717, XP_0115	protein_coding	ENSG0000000339	57679	443
2648	LACTB2	0.846	0.008	lactamase beta 2 [Source:HGNC Symbol;Acc:HGNC:18512]	chr8:71547552-71581409	NM_016027	.	NP_057111	.	protein_coding	ENSG0000014759	51110	18512
2649	MAGEC2	-7.306	0.008	MAGE family member C2 [Source:HGNC Symbol;Acc:HGNC:13574]	chrX:141290130-141293076	NM_016249	.	NP_057333	.	protein_coding	ENSG0000004677	51438	13574
2650	NDUFS6	-0.711	0.008	NADH:ubiquinone oxidoreductase subunit S6 [Source:HGNC Symbol;Acc:HGNC:7713]	chr5:1801513-1816719	NM_004553	.	NP_004544	.	protein_coding	ENSG0000014549	4726	7713
2651	RPL35A	-0.683	0.008	ribosomal protein L35a [Source:HGNC Symbol;Acc:HGNC:10345]	chr3:197615945-197686890	NM_000996, NM_0	XM_005269351	NP_000987, NP_00130	XP_005269408	protein_coding	ENSG0000018289	6165	10345
2652	SERPINF2	-0.795	0.008	serpin family F member 2 [Source:HGNC Symbol;Acc:HGNC:9075]	chr17:1646129-1658562	NM_000934, NM_0	XM_017024765, XM_0	NP_000925, NP_00115	XP_005256758, XP_0168	protein_coding	ENSG0000016771	5345	9075
2653	ZNF740	-0.670	0.008	zinc finger protein 740 [Source:HGNC Symbol;Acc:HGNC:27465]	chr12:53551446-53601091	NM_001004304	XM_006719344	NP_001004304	XP_006719407, XP_0168	protein_coding	ENSG0000013965	283337	27465
2654	CNPY3	-0.720	0.009	canopy FGF signaling regulator 3 [Source:HGNC Symbol;Acc:HGNC:11968]	chr6:42896929-42907025	NM_006586	XM_005248818, XM_0	NP_006577	.	protein_coding	ENSG0000013716	10695	11968
2655	KDM3A	0.797	0.009	lysine demethylase 3A [Source:HGNC Symbol;Acc:HGNC:20815]	chr2:86667923-86719839	NM_001146688, NM	XM_017004493, XM_0	NP_001140160, NP_06	XP_006712114, XP_0168	protein_coding	ENSG0000011554	55818	20815
2656	TC2N	0.939	0.009	tandem C2 domains, nuclear [Source:HGNC Symbol;Acc:HGNC:19859]	chr14:92246094-92333873	NM_001128595, NM	.	NP_001122067, NP_00	.	protein_coding	ENSG0000016592	123036	19859
2657	TMEM134	-0.919	0.009	transmembrane protein 134 [Source:HGNC Symbol;Acc:HGNC:26142]	chr11:67229180-67236721	NM_001078650, NM	XM_006718693	NP_001072118, NP_00	XP_006718756	protein_coding	ENSG0000017266	80194	26142
2658	ZNF33B	0.987	0.009	zinc finger protein 33B [Source:HGNC Symbol;Acc:HGNC:13097]	chr10:43084554-43133992	NM_001305033, NM	XM_006717966	NP_001291962, NP_00	.	protein_coding	ENSG0000019669	7582	13097
2659	ANAPC2	-0.750	0.009	anaphase promoting complex subunit 2 [Source:HGNC Symbol;Acc:HGNC:19989]	chr9:140069235-140082989	NM_013366	.	NP_037498	.	protein_coding	ENSG0000017624	29882	19989
2660	HNRNPA2B1	0.631	0.009	heterogeneous nuclear ribonucleoprotein A2/B1 [Source:HGNC Symbol;Acc:HGNC:5033	chr7:26229546-26240413	NM_002137, NM_0	XM_006715714, XM_0	NP_002128, NP_11253	XP_005249786, XP_0168	protein_coding	ENSG0000012256	3181	5033
2661	INTS12	1.194	0.009	integrator complex subunit 12 [Source:HGNC Symbol;Acc:HGNC:25067]	chr4:106603783-106768885	NM_001142471, NM	XM_011532145, XM_0	NP_001135943, NP_06	XP_005263205, XP_0115	protein_coding	ENSG0000013878	57117	25067
2662	RIOK3	0.774	0.009	RIO kinase 3 [Source:HGNC Symbol;Acc:HGNC:11451]	chr18:21032786-21063104	NM_003831	XM_011526243, XM_0	NP_003822	XP_011524544, XP_0115	protein_coding	ENSG0000010178	8780	11451
2663	VSNL1	0.835	0.009	visinin like 1 [Source:HGNC Symbol;Acc:HGNC:12722]	chr2:17720392-17838285	NM_003385	.	NP_003376	.	protein_coding	ENSG0000016303	7447	12722
2664	NIPSNAP1	-0.633	0.009	nipsnap homolog 1 (C. elegans) [Source:HGNC Symbol;Acc:HGNC:7827]	chr22:29950796-29977326	NM_001202502, NM	.	NP_001189431, NP_00	.	protein_coding	ENSG0000018411	8508	7827
2665	AP2B1	0.615	0.009	adaptor related protein complex 2 beta 1 subunit [Source:HGNC Symbol;Acc:HGNC:563	chr17:33914292-34053428	NM_001030006, NM	XM_011524455, XM_0	NP_001025177, NP_00	XP_005257994, XP_0052	protein_coding	ENSG0000000612	163	563
2666	ARHGEF10L	-0.912	0.009	Rho guanine nucleotide exchange factor 10 like [Source:HGNC Symbol;Acc:HGNC:25540	chr1:17866329-18024364	NM_001011722, NM	XM_017001622, XM_0	NP_001011722, NP_00	XP_005245980, XP_0052	protein_coding	ENSG0000007496	55160	25540
2667	CROCC	-0.856	0.009	ciliary rootlet coiled-coil, rootletin [Source:HGNC Symbol;Acc:HGNC:21299]	chr1:17248444-17299474	NM_014675	XM_017002913, XM_0	NP_055490	XP_006711121, XP_0115	protein_coding	ENSG0000005845	9696	21299
2668	ILDR2	1.404	0.009	immunoglobulin like domain containing receptor 2 [Source:HGNC Symbol;Acc:HGNC:18	chr1:166877427-166944719	NM_199351	.	NP_955383	XP_016856741, XP_0168	protein_coding	ENSG0000014319	387597	18131
2669	MAPT	-0.854	0.009	microtubule associated protein tau [Source:HGNC Symbol;Acc:HGNC:6893]	chr17:43971747-44105700	NM_001123066, NM	XM_005257371, XM_0	NP_001116539, NP_00	XP_005257419, XP_0052	protein_coding	ENSG0000018686	4137	6893
2670	DHRS13	-0.881	0.009	dehydrogenase/reductase 13 [Source:HGNC Symbol;Acc:HGNC:28326]	chr17:27224798-27230089	NM_144683	.	NP_653284	.	protein_coding	ENSG0000016753	147015	28326
2671	GPAT3	0.913	0.009	glycerol-3-phosphate acyltransferase 3 [Source:HGNC Symbol;Acc:HGNC:28157]	chr4:84457066-84527028	NM_001256421, NM	XM_017008781, XM_0	NP_001243350, NP_00	XP_011530685, XP_0115	protein_coding	ENSG0000013867	84803	28157
2672	HSPA4	0.851	0.009	heat shock protein family A (Hsp70) member 4 [Source:HGNC Symbol;Acc:HGNC:5237]	chr5:132387653-132442141	NM_002154	.	NP_002145	.	protein_coding	ENSG0000017060	3308	5237
2673	NDUFAF3	-1.442	0.009	NADH:ubiquinone oxidoreductase complex assembly factor 3 [Source:HGNC Symbol;Acc	chr3:49027318-49060928	NM_199069, NM_1	.	NP_951032, NP_95103	.	protein_coding	ENSG0000017805	25915	29918
2674	RPS28	-0.622	0.009	ribosomal protein S28 [Source:HGNC Symbol;Acc:HGNC:10418]	chr19:8386383-8408146	NM_001031	.	NP_001022	.	protein_coding	ENSG0000023392	6234	10418
2675	TMEM238	-0.984	0.009	transmembrane protein 238 [Source:HGNC Symbol;Acc:HGNC:40042]	chr19:55890612-55895966	NM_001190764	.	NP_001177693	.	protein_coding	ENSG0000023349	388564	40042
2676	CCDC93	0.738	0.009	coiled-coil domain containing 93 [Source:HGNC Symbol;Acc:HGNC:25611]	chr2:118673053-118771709	NM_019044	XM_011511362, XM_0	NP_061917	XP_011509661, XP_0115	protein_coding	ENSG0000012563	54520	25611
2677	CHD6	0.703	0.009	chromodomain helicase DNA binding protein 6 [Source:HGNC Symbol;Acc:HGNC:19057]	chr20:40030740-40247133	NM_032221	XM_017028100, XM_0	NP_115597	XP_005260630, XP_0115	protein_coding	ENSG0000012417	84181	19057
2678	FBXL3	0.874	0.009	F-box and leucine rich repeat protein 3 [Source:HGNC Symbol;Acc:HGNC:13599]	chr13:77579388-77601285	NM_012158	XM_005266336	NP_036290	XP_005266393	protein_coding	ENSG0000000581	26224	13599
2679	MKNK2	-0.615	0.009	MAP kinase interacting serine/threonine kinase 2 [Source:HGNC Symbol;Acc:HGNC:711	chr19:2037463-2051243	NM_017572, NM_1	.	NP_060042, NP_95100	.	protein_coding	ENSG0000009987	2872	7111
2680	BCL7C	-0.751	0.009	BCL tumor suppressor 7C [Source:HGNC Symbol;Acc:HGNC:1006]	chr16:30844946-30906281	NM_001286526, NM	XM_011545980	NP_001273455, NP_00	XP_011544282	protein_coding	ENSG0000009938	9274	1006
2681	CCDC130	-0.885	0.009	coiled-coil domain containing 130 [Source:HGNC Symbol;Acc:HGNC:28118]	chr19:13842573-13874110	NM_001320565, NM	XM_005260088, XM_0	NP_001307490, NP_00	XP_005260142, XP_0052	protein_coding	ENSG0000010495	81576	28118
2682	PFKFB2	0.846	0.009	6-phosphofructo-2-kinase/fructose-2,6-biphosphatase 2 [Source:HGNC Symbol;Acc:HGN	chr1:207226604-207254369	NM_001018053, NM	XM_011509628, XM_0	NP_001018063, NP_00	XP_011507927, XP_0115	protein_coding	ENSG0000012383	5208	8873
2683	POLG2	0.924	0.009	DNA polymerase gamma 2, accessory subunit [Source:HGNC Symbol;Acc:HGNC:9180]	chr17:62473901-62493154	NM_007215	.	NP_009146	.	protein_coding	ENSG0000025652	11232	9180
2684	SLC35D1	0.903	0.009	solute carrier family 35 member D1 [Source:HGNC Symbol;Acc:HGNC:20800]	chr1:67465014-67519782	NM_015139	XM_011541070, XM_0	NP_055954	XP_006710541, XP_0115	protein_coding	ENSG0000011670	23169	20800
2685	TRAP1	-0.628	0.009	TNF receptor associated protein 1 [Source:HGNC Symbol;Acc:HGNC:16264]	chr16:3692938-3767598	NM_001272049, NM	XM_011522345	NP_001258978, NP_05	XP_011520647	protein_coding	ENSG0000012660	10131	16264
2686	CLTB	-0.637	0.009	clathrin light chain B [Source:HGNC Symbol;Acc:HGNC:2091]	chr5:175819455-175843570	NM_001834, NM_0	.	NP_001825, NP_00902	XP_016864516	protein_coding	ENSG0000017541	1212	2091
2687	FBXL4	1.051	0.009	F-box and leucine rich repeat protein 4 [Source:HGNC Symbol;Acc:HGNC:13601]	chr6:99316413-99395882	NM_001278716, NM	XM_005266930	NP_001265645, NP_03	XP_005266987, XP_0168	protein_coding	ENSG0000011223	26235	13601
2688	HUWE1	-0.610	0.009	HECT, UBA and WWE domain containing 1, E3 ubiquitin protein ligase [Source:HGNC Sy	chrX:53559056-53713674	NM_031407	XM_011530758, XM_0	NP_113584	XP_005262022, XP_0115	protein_coding	ENSG0000008675	10075	30892
2689	PPP1R9B	-0.653	0.009	protein phosphatase 1 regulatory subunit 9B [Source:HGNC Symbol;Acc:HGNC:9298]	chr17:48211098-48227991	NM_032595	XM_011525380	NP_115984	.	protein_coding	ENSG0000010881	84687	9298
2690	PTPRH	-0.876	0.009	protein tyrosine phosphatase, receptor type H [Source:HGNC Symbol;Acc:HGNC:9672]	chr19:55692615-55720824	NM_001161440, NM	XM_017027063, XM_0	NP_001154912, NP_00	XP_011525485, XP_0115	protein_coding	ENSG0000008003	5794	9672
2691	FLOT2	-0.626	0.009	flotillin 2 [Source:HGNC Symbol;Acc:HGNC:3758]	chr17:27206352-27224697	NM_004475	XM_017024396, XM_0	NP_004466	XP_005258007, XP_0052	protein_coding	ENSG0000013258	2319	3758
2692	NYNRIN	-0.898	0.009	NYN domain and retroviral integrase containing [Source:HGNC Symbol;Acc:HGNC:20165	chr14:24867991-24888494	NM_025081	.	NP_079357	.	protein_coding	ENSG0000020597	57523	20165
2693	TIGD2	1.240	0.009	tigger transposable element derived 2 [Source:HGNC Symbol;Acc:HGNC:18333]	chr4:90032650-90036050	NM_145715	.	NP_663761	.	protein_coding	ENSG0000018034	166815	18333
2694	DUSP2	-0.825	0.009	dual specificity phosphatase 2 [Source:HGNC Symbol;Acc:HGNC:3068]	chr2:96808904-96811179	NM_004418	.	NP_004409	.	protein_coding	ENSG0000015805	1844	3068
2695	NCAPG	0.867	0.009	non-SMC condensin I complex subunit G [Source:HGNC Symbol;Acc:HGNC:24304]	chr4:17812524-18023499	NM_022346	XM_011513876	NP_071741	XP_011512178, XP_0168	protein_coding	ENSG0000010980	64151	24304
2696	SLC25A17	0.773	0.009	solute carrier family 25 member 17 [Source:HGNC Symbol;Acc:HGNC:10987]	chr22:41165633-41215403	NM_001282726, NM	.	NP_001269655, NP_00	.	protein_coding	ENSG0000010037	10478	10987
2697	AGFG1	0.931	0.009	ArfGAP with FG repeats 1 [Source:HGNC Symbol;Acc:HGNC:5175]	chr2:228336867-228425930	NM_001135187, NM	XM_011511072, XM_0	NP_001128659, NP_00	XP_005246573, XP_0067	protein_coding	ENSG0000017374	3267	5175
2698	NBR1	0.658	0.009	NBR1, autophagy cargo receptor [Source:HGNC Symbol;Acc:HGNC:6746]	chr17:41322497-41363708	NM_001291571, NM	XM_011524813, XM_0	NP_001278500, NP_00	XP_006721966, XP_0115	protein_coding	ENSG0000018855	4077	6746

2699	SMIM4	-1.473	0.009	small integral membrane protein 4 [Source:HGNC Symbol;Acc:HGNC:37257]	chr3:52570617-52719852	NM_001124767	.	NP_001118239	.	protein_coding	ENSG0000016827	440957	37257
2700	LPIN2	0.781	0.009	lipin 2 [Source:HGNC Symbol;Acc:HGNC:14450]	chr18:2916991-3011945	NM_014646	XM_017026099, XM_0	NP_055461	XP_005258234, XP_0052	protein_coding	ENSG0000010157	9663	14450
2701	PHF20L1	1.138	0.009	PHD finger protein 20-like 1 [Source:HGNC Symbol;Acc:HGNC:24280]	chr8:133787603-133861052	NM_001277196, N	XM_017013517, XM_0	NP_001264125, NP_05	XP_005250988, XP_0115	protein_coding	ENSG0000012929	51105	24280
2702	QRICH1	-0.678	0.009	glutamine rich 1 [Source:HGNC Symbol;Acc:HGNC:24713]	chr3:49067139-49131796	NM_017730, NM_1	XM_011533863, XM_0	NP_001307509, NP_00	XP_005265305, XP_0067	protein_coding	ENSG0000019821	54870	24713
2703	TRIM66	0.910	0.009	tripartite motif containing 66 [Source:HGNC Symbol;Acc:HGNC:29005]	chr11:8633583-8693413	NM_014818	XM_017018631, XM_0	NP_055633	XP_006718460, XP_0067	protein_coding	ENSG0000016643	9866	29005
2704	ATP6V1E1	0.712	0.009	ATPase H+ transporting V1 subunit E1 [Source:HGNC Symbol;Acc:HGNC:857]	chr22:18074901-18111584	NM_001039366, N	.	NP_001034455, NP_00	.	protein_coding	ENSG0000013110	529	857
2705	NBPF14	0.905	0.009	neuroblastoma breakpoint family member 14 [Source:HGNC Symbol;Acc:HGNC:25232]	chr1:148003659-148025848	NM_015383	XM_011509395, XM_0	NP_056198	XP_006711766, XP_0067	protein_coding	ENSG0000012249	100996763	25232
2706	SLC27A2	0.783	0.009	solute carrier family 27 member 2 [Source:HGNC Symbol;Acc:HGNC:10996]	chr15:50474392-50528592	NM_001159629, N	.	NP_001153101, NP_00	.	protein_coding	ENSG0000014028	11001	10996
2707	CLEC3B	-2.400	0.009	C-type lectin domain family 3 member B [Source:HGNC Symbol;Acc:HGNC:11891]	chr3:45067674-45077563	NM_001308394, N	XM_005265426	NP_001295323, NP_00	XP_005265483	protein_coding	ENSG0000016381	7123	11891
2708	INO80D	1.014	0.009	INO80 complex subunit D [Source:HGNC Symbol;Acc:HGNC:25997]	chr2:206858444-206950906	NM_017759	XM_011511377, XM_0	NP_060229	XP_011509671, XP_0115	protein_coding	ENSG0000011493	54891	25997
2709	MEMO1	0.796	0.009	mediator of cell motility 1 [Source:HGNC Symbol;Acc:HGNC:14014]	chr2:32090128-32236043	NM_001137602, N	XM_017004255, XM_0	NP_001131074, NP_00	XP_011531194, XP_0168	protein_coding	ENSG0000016295	51072	14014
2710	MGEA5	0.696	0.009	meningioma expressed antigen 5 (hyaluronidase) [Source:HGNC Symbol;Acc:HGNC:705	chr10:103544199-103578222	NM_001142434, N	XM_011539182, XM_0	NP_001135906, NP_03	.	protein_coding	ENSG0000019840	10724	7056
2711	PLEKHA1	0.920	0.009	pleckstrin homology domain containing A1 [Source:HGNC Symbol;Acc:HGNC:14335]	chr10:124134211-124202118	NM_001001974, N	XM_017016491, XM_0	NP_001001974, NP_00	XP_005270073, XP_0052	protein_coding	ENSG0000010767	59338	14335
2712	SERTAD2	0.791	0.009	SERTA domain containing 2 [Source:HGNC Symbol;Acc:HGNC:30784]	chr2:64858754-64881047	NM_014755	XM_011533205, XM_0	NP_055570	XP_005264726, XP_0115	protein_coding	ENSG0000017983	9792	30784
2713	CENPU	1.304	0.009	centromere protein U [Source:HGNC Symbol;Acc:HGNC:21348]	chr4:185570766-185655287	NM_024629	XM_005263218	NP_078905	XP_005263275	protein_coding	ENSG0000015172	79682	21348
2714	IFI30	-0.750	0.009	IFI30, lysosomal thiol reductase [Source:HGNC Symbol;Acc:HGNC:5398]	chr19:18284476-18288927	NM_006332	.	NP_006323	.	protein_coding	ENSG0000021649	10437	5398
2715	KIAA1958	1.052	0.009	KIAA1958 [Source:HGNC Symbol;Acc:HGNC:23427]	chr9:115249126-115431677	NM_001287036, N	XM_011518311, XM_0	NP_001273965, NP_59	XP_006717041, XP_0115	protein_coding	ENSG0000016518	158405	23427
2716	NCOA4	0.698	0.009	nuclear receptor coactivator 4 [Source:HGNC Symbol;Acc:HGNC:7671]	chr10:51565107-51590734	NM_001145260, N	.	NP_001138732, NP_00	.	protein_coding	ENSG0000026641	8031	7671
2717	TCEB2	-0.682	0.009	transcription elongation factor B subunit 2 [Source:HGNC Symbol;Acc:HGNC:11619]	chr16:2821414-2827298	NM_007108, NM_2	.	NP_009039, NP_99689	.	protein_coding	ENSG0000010336	6923	11619
2718	ZNF791	0.886	0.009	zinc finger protein 791 [Source:HGNC Symbol;Acc:HGNC:26895]	chr19:12721731-12744654	NM_153358	XM_017026412	NP_699189	XP_016881901	protein_coding	ENSG0000017387	163049	26895
2719	C1orf35	-0.763	0.009	chromosome 1 open reading frame 35 [Source:HGNC Symbol;Acc:HGNC:19032]	chr1:228288431-228291163	NM_024319	XM_011544274	NP_077295	.	protein_coding	ENSG0000014379	79169	19032
2720	CHMP6	-0.870	0.009	charged multivesicular body protein 6 [Source:HGNC Symbol;Acc:HGNC:25675]	chr17:78965640-78973933	NM_024591	XM_005257668	NP_078867	XP_005257725	protein_coding	ENSG0000017610	79643	25675
2721	FZR1	-0.829	0.009	fizzy/cell division cycle 20 related 1 [Source:HGNC Symbol;Acc:HGNC:24824]	chr19:3506270-3538328	NM_001136197, N	XM_005259573	NP_001129669, NP_00	XP_005259630, XP_0168	protein_coding	ENSG0000010532	51343	24824
2722	ZNF224	1.036	0.009	zinc finger protein 224 [Source:HGNC Symbol;Acc:HGNC:13017]	chr19:44598544-44614039	NM_001321645, N	XM_017027261, XM_0	NP_001308574, NP_03	XP_005259278, XP_0168	protein_coding	ENSG0000026768	7767	13017
2723	DNAJC28	1.352	0.009	DnaJ heat shock protein family (Hsp40) member C28 [Source:HGNC Symbol;Acc:HGNC:1	chr21:34857836-34864027	NM_001040192, N	XM_006724027	NP_001035282, NP_00	XP_006724090	protein_coding	ENSG0000017769	54943	1297
2724	FAAP100	-0.761	0.009	Fanconi anemia core complex associated protein 100 [Source:HGNC Symbol;Acc:HGNC:	chr17:79506910-79519403	NM_025161	XM_017025163, XM_0	NP_079437	XP_006722174, XP_0067	protein_coding	ENSG0000018550	80233	26171
2725	FMR1	0.806	0.009	fragile X mental retardation 1 [Source:HGNC Symbol;Acc:HGNC:3775]	chrX:146993468-147032645	NM_001185075, N	.	NP_001172004, NP_00	.	protein_coding	ENSG0000010208	2332	3775
2726	GAS6	-0.630	0.009	growth arrest specific 6 [Source:HGNC Symbol;Acc:HGNC:4168]	chr13:114523530-114567040	NM_000820	.	NP_000811	.	protein_coding	ENSG0000018308	2621	4168
2727	MLST8	-0.738	0.009	MTOR associated protein, LST8 homolog [Source:HGNC Symbol;Acc:HGNC:24825]	chr16:2255177-2261951	NM_001199173, N	XM_017023548, XM_0	NP_001186102, NP_00	XP_005255532, XP_0052	protein_coding	ENSG0000016796	64223	24825
2728	SLC22A17	-1.017	0.009	solute carrier family 22 member 17 [Source:HGNC Symbol;Acc:HGNC:23095]	chr14:23815514-23822121	NM_020372	XM_005267747	NP_065105	XP_005267804, XP_0168	protein_coding	ENSG0000009209	51310	23095
2729	STXBP2	-0.645	0.009	syntaxin binding protein 2 [Source:HGNC Symbol;Acc:HGNC:11445]	chr19:7701998-7712759	NM_001127396, N	.	NP_001120868, NP_00	.	protein_coding	ENSG0000007694	6813	11445
2730	TMSB15A	-2.675	0.009	thymosin beta 15a [Source:HGNC Symbol;Acc:HGNC:30744]	chrX:101768603-101771712	NM_021992	.	NP_068832	.	protein_coding	ENSG0000015816	11013	30744
2731	ADAM15	-0.639	0.009	ADAM metallopeptidase domain 15 [Source:HGNC Symbol;Acc:HGNC:193]	chr1:155023761-155035251	NM_001261464, N	.	NP_001248393, NP_00	.	protein_coding	ENSG0000014353	8751	193
2732	CLCN7	-0.697	0.009	chloride voltage-gated channel 7 [Source:HGNC Symbol;Acc:HGNC:2025]	chr16:1494935-1525581	NM_001114331, N	XM_011522354	NP_001107803, NP_00	XP_011520656	protein_coding	ENSG0000010324	1186	2025
2733	FBLIM1	-0.834	0.009	filamin binding LIM protein 1 [Source:HGNC Symbol;Acc:HGNC:24686]	chr1:16083153-16113089	NM_001024215, N	XM_017001525, XM_0	NP_001019386, NP_00	XP_005245957, XP_0052	protein_coding	ENSG0000016245	54751	24686
2734	GPAT4	0.635	0.009	glycerol-3-phosphate acyltransferase 4 [Source:HGNC Symbol;Acc:HGNC:20880]	chr8:41435718-41482520	NM_178819	XM_011544396, XM_0	NP_848934	XP_011542694, XP_0115	protein_coding	ENSG0000015866	137964	20880
2735	MTBP	1.014	0.009	MDM2 binding protein [Source:HGNC Symbol;Acc:HGNC:7417]	chr8:121457665-121535875	NM_022045	.	NP_071328	.	protein_coding	ENSG0000017216	27085	7417
2736	SCFD1	0.900	0.009	sec1 family domain containing 1 [Source:HGNC Symbol;Acc:HGNC:20726]	chr14:31091317-31205018	NM_001257376, N	XM_005267469	NP_001244305, NP_00	XP_005267526	protein_coding	ENSG0000009210	23256	20726
2737	VIM	0.595	0.009	vimentin [Source:HGNC Symbol;Acc:HGNC:12692]	chr10:17270257-17279592	NM_003380	XM_011519649, XM_0	NP_003371	XP_006717563, XP_0115	protein_coding	ENSG0000002602	7431	12692
2738	BLZF1	1.011	0.009	basic leucine zipper nuclear factor 1 [Source:HGNC Symbol;Acc:HGNC:1065]	chr1:169337207-169429907	NM_001320972, N	XM_011510092	NP_001307901, NP_00	XP_011508394	protein_coding	ENSG0000011747	8548	1065
2739	LETM2	1.029	0.009	leucine zipper and EF-hand containing transmembrane protein 2 [Source:HGNC Symbol;	chr8:38243820-38266062	NM_001199659, N	XM_011544410, XM_0	NP_001186588, NP_00	XP_006716354, XP_0115	protein_coding	ENSG0000016504	137994	14648
2740	TFG	-0.729	0.009	TRK-fused gene [Source:HGNC Symbol;Acc:HGNC:11758]	chr3:100428174-100467810	NM_001007565, N	XM_017005529, XM_0	NP_001007566, NP_00	XP_005247123, XP_0067	protein_coding	ENSG0000011435	10342	11758
2741	TTC30A	1.097	0.009	tetratricopeptide repeat domain 30A [Source:HGNC Symbol;Acc:HGNC:25853]	chr2:178477719-178483694	NM_152275	.	NP_689488	.	protein_coding	ENSG0000019755	92104	25853
2742	C2orf82	-1.763	0.009	chromosome 2 open reading frame 82 [Source:HGNC Symbol;Acc:HGNC:33763]	chr2:233733723-233741116	NM_206895	.	NP_996778	XP_016859570, XP_0168	protein_coding	ENSG0000018260	389084	33763
2743	FABP3	-0.977	0.009	fatty acid binding protein 3 [Source:HGNC Symbol;Acc:HGNC:3557]	chr1:31838471-31845923	NM_004102	.	NP_001307925, NP_00	.	protein_coding	ENSG0000012176	2170	3557
2744	KRAS	0.738	0.009	KRAS proto-oncogene, GTPase [Source:HGNC Symbol;Acc:HGNC:6407]	chr12:25348149-25403870	NM_004985, NM_0	XM_011520653, XM_0	NP_004976, NP_20352	XP_006719132, XP_0115	protein_coding	ENSG0000013370	3845	6407
2745	NSMAF	0.738	0.009	neutral sphingomyelinase activation associated factor [Source:HGNC Symbol;Acc:HGNC	chr8:59496062-59572403	NM_001144772, N	.	NP_001138244, NP_00	.	protein_coding	ENSG0000003568	8439	8017
2746	VWA5B2	-0.979	0.009	von Willebrand factor A domain containing 5B2 [Source:HGNC Symbol;Acc:HGNC:25144	chr3:183948216-183960117	NM_138345	XM_011513292, XM_0	NP_001307302, NP_61	XP_006713881, XP_0115	protein_coding	ENSG0000014519	90113	25144
2747	ZSCAN32	0.804	0.009	zinc finger and SCAN domain containing 32 [Source:HGNC Symbol;Acc:HGNC:20812]	chr16:3432080-3451065	NM_001284527, N	XM_017023371, XM_0	NP_001271456, NP_00	XP_005255463, XP_0115	protein_coding	ENSG0000014098	54925	20812
2748	FBXW7	1.114	0.010	F-box and WD repeat domain containing 7 [Source:HGNC Symbol;Acc:HGNC:16712]	chr4:153242410-153457253	NM_001013415, N	XM_011532088, XM_0	NP_001013433, NP_00	XP_011530385, XP_0115	protein_coding	ENSG0000010967	55294	16712
2749	PTPDC1	0.896	0.010	protein tyrosine phosphatase domain containing 1 [Source:HGNC Symbol;Acc:HGNC:30	chr9:96793075-96872138	NM_001253829, N	XM_017014281, XM_0	NP_001240758, NP_00	XP_005251768, XP_0067	protein_coding	ENSG0000015807	138639	30184
2750	RAP1GDS1	0.827	0.010	Rap1 GTPase-GDP dissociation stimulator 1 [Source:HGNC Symbol;Acc:HGNC:9859]	chr4:99182567-99365012	NM_001100426, N	XM_011532173, XM_0	NP_001093896, NP_00	.	protein_coding	ENSG0000013869	5910	9859
2751	REPS2	-0.903	0.010	RALBP1 associated Eps domain containing 2 [Source:HGNC Symbol;Acc:HGNC:9963]	chrX:16964813-17171403	NM_001080975, N	XM_011545605, XM_0	NP_001074444, NP_00	XP_005274682, XP_0052	protein_coding	ENSG0000016989	9185	9963
2752	SLC35E4	-0.856	0.010	solute carrier family 35 member E4 [Source:HGNC Symbol;Acc:HGNC:17058]	chr22:31031638-31063877	NM_001001479	.	NP_001001479, NP_00	XP_016884283, XP_0168	protein_coding	ENSG0000010003	339665	17058

2753	ZC3H12C	1.051	0.010	zinc finger CCCH-type containing 12C [Source:HGNC Symbol;Acc:HGNC:29362]	chr11:109964086-110042566	NM_033390	XM_011543056, XM_0	NP_203748	XP_005271772, XP_0115	protein_coding	ENSG0000014928	85463	29362
2754	EIF1B	-0.771	0.010	eukaryotic translation initiation factor 1B [Source:HGNC Symbol;Acc:HGNC:30792]	chr3:40351174-40353915	NM_005875	.	NP_005866	.	protein_coding	ENSG0000011478	10289	30792
2755	GABARAP	-0.646	0.010	GABA type A receptor-associated protein [Source:HGNC Symbol;Acc:HGNC:4067]	chr17:7143332-7146089	NM_007278	.	NP_009209	.	protein_coding	ENSG0000017029	11337	4067
2756	MRPL54	-0.757	0.010	mitochondrial ribosomal protein L54 [Source:HGNC Symbol;Acc:HGNC:16685]	chr19:3762661-3767563	NM_172251	.	NP_758455	.	protein_coding	ENSG0000018361	116541	16685
2757	PRKAR1A	0.647	0.010	protein kinase cAMP-dependent type I regulatory subunit alpha [Source:HGNC Symbol;A	chr17:66508172-66597530	NM_001276289, NM	XM_011524985, XM_0	NP_001263218, NP_00	XP_011523285, XP_0115	protein_coding	ENSG0000010894	5573	9388
2758	ZCCHC11	0.874	0.010	zinc finger CCHC-type containing 11 [Source:HGNC Symbol;Acc:HGNC:28981]	chr1:52888946-53018772	NM_001009881, NM	XM_011541100, XM_0	NP_001009881, NP_05	XP_005270733, XP_0052	protein_coding	ENSG0000013474	23318	28981
2759	FAM3C	0.773	0.010	family with sequence similarity 3 member C [Source:HGNC Symbol;Acc:HGNC:18664]	chr7:120988904-121036388	NM_001040020, NM	XM_011515737, XM_0	NP_001035109, NP_05	XP_011514038, XP_0115	protein_coding	ENSG0000019693	10447	18664
2760	FURIN	-0.612	0.010	furin, paired basic amino acid cleaving enzyme [Source:HGNC Symbol;Acc:HGNC:8568]	chr15:91411821-91426688	NM_001289823, NM	.	NP_001276752, NP_00	.	protein_coding	ENSG0000014056	5045	8568
2761	ID3	-0.931	0.010	inhibitor of DNA binding 3, HLH protein [Source:HGNC Symbol;Acc:HGNC:5362]	chr1:23884408-23886285	NM_002167	.	NP_002158	.	protein_coding	ENSG0000011731	3399	5362
2762	OLFM2	-1.215	0.010	olfactomedin 2 [Source:HGNC Symbol;Acc:HGNC:17189]	chr19:9964393-10047228	NM_001304347, NM	.	NP_001291276, NP_00	.	protein_coding	ENSG0000010508	93145	17189
2763	SLC35C2	-0.663	0.010	solute carrier family 35 member C2 [Source:HGNC Symbol;Acc:HGNC:17117]	chr20:44974618-44993097	NM_001281457, NM	XM_017027858, XM_0	NP_001268386, NP_00	XP_011527133, XP_0115	protein_coding	ENSG0000008018	51006	17117
2764	ANKRD17	0.756	0.010	ankyrin repeat domain 17 [Source:HGNC Symbol;Acc:HGNC:23575]	chr4:73939092-74124515	NM_001286771, NM	XM_005265671, XM_0	NP_001273700, NP_05	XP_005265728, XP_0168	protein_coding	ENSG0000013246	26057	23575
2765	EIF4G3	0.746	0.010	eukaryotic translation initiation factor 4 gamma 3 [Source:HGNC Symbol;Acc:HGNC:329	chr1:21132784-21503381	NM_001198801, NM	XM_017002708, XM_0	NP_001185730, NP_00	XP_011540658, XP_0115	protein_coding	ENSG0000007515	8672	3298
2766	MED21	0.761	0.010	mediator complex subunit 21 [Source:HGNC Symbol;Acc:HGNC:11473]	chr12:27175478-27183689	NM_001271811, NM	.	NP_001258740, NP_00	.	protein_coding	ENSG0000015294	9412	11473
2767	GAPVD1	0.747	0.010	GTPase activating protein and VPS9 domains 1 [Source:HGNC Symbol;Acc:HGNC:23375]	chr9:128024107-128129486	NM_001282679, NM	XM_011518508, XM_0	NP_001269608, NP_00	XP_005251958, XP_0052	protein_coding	ENSG0000016521	26130	23375
2768	THAP1	0.903	0.010	THAP domain containing 1 [Source:HGNC Symbol;Acc:HGNC:20856]	chr8:42691816-42698468	NM_018105, NM_1	.	NP_060575, NP_94535	.	protein_coding	ENSG0000013193	55145	20856
2769	UHRF1BP1	0.877	0.010	UHRF1 binding protein 1 [Source:HGNC Symbol;Acc:HGNC:21216]	chr6:34759791-34855866	NM_017754	XM_011514714, XM_0	NP_060224	.	protein_coding	ENSG0000006506	54887	21216
2770	HLA-E	-0.606	0.010	major histocompatibility complex, class I, E [Source:HGNC Symbol;Acc:HGNC:4962]	chr6:30457243-30461982	NM_005516	XM_017010807	NP_005507	XP_016866296, XP_0168	protein_coding	ENSG0000020459	3133	4962
2771	LRRC40	0.978	0.010	leucine rich repeat containing 40 [Source:HGNC Symbol;Acc:HGNC:26004]	chr1:70610487-70671303	NM_017768	XM_011541763, XM_0	NP_060238	XP_005271070, XP_0115	protein_coding	ENSG0000006655	55631	26004
2772	OSBP	0.604	0.010	oxysterol binding protein [Source:HGNC Symbol;Acc:HGNC:8503]	chr11:59341870-59383617	NM_002556	.	NP_002547	.	protein_coding	ENSG0000011004	5007	8503
2773	PRSS27	-1.511	0.010	protease, serine 27 [Source:HGNC Symbol;Acc:HGNC:15475]	chr16:2762418-2770216	NM_001318395, NM	.	NP_001305324, NP_11	.	protein_coding	ENSG0000017238	83886	15475
2774	STK11IP	-0.778	0.010	serine/threonine kinase 11 interacting protein [Source:HGNC Symbol;Acc:HGNC:19184]	chr2:220462581-220481173	NM_052902	XM_011510527, XM_0	NP_443134	XP_011508827, XP_0115	protein_coding	ENSG0000014458	114790	19184
2775	ARHGEF19	-0.856	0.010	Rho guanine nucleotide exchange factor 19 [Source:HGNC Symbol;Acc:HGNC:26604]	chr1:16524598-16539104	NM_153213	XM_011540706	NP_694945	XP_011539008	protein_coding	ENSG0000014263	128272	26604
2776	CTHRC1	0.891	0.010	collagen triple helix repeat containing 1 [Source:HGNC Symbol;Acc:HGNC:18831]	chr8:104383742-104395225	NM_001256099, NM	.	NP_001243028, NP_61	.	protein_coding	ENSG0000016493	115908	18831
2777	DGAT1	-0.820	0.010	diacylglycerol O-acyltransferase 1 [Source:HGNC Symbol;Acc:HGNC:2843]	chr8:145539953-145550573	NM_012079	XM_011517356	NP_036211	XP_011515658	protein_coding	ENSG0000018500	102465511	2843
2778	FUK	-0.851	0.010	fucokinase [Source:HGNC Symbol;Acc:HGNC:29500]	chr16:70488323-70514177	NM_145059	XM_017023012, XM_0	NP_659496	XP_006721224, XP_0067	protein_coding	ENSG0000015735	197258	29500
2779	MAP3K2	1.008	0.010	mitogen-activated protein kinase kinase kinase 2 [Source:HGNC Symbol;Acc:HGNC:6854	chr2:128056305-128146041	NM_006609	.	NP_006600	.	protein_coding	ENSG0000016996	10746	6854
2780	PRKCI	0.766	0.010	protein kinase C iota [Source:HGNC Symbol;Acc:HGNC:9404]	chr3:169940152-170023769	NM_002740	.	NP_002731	.	protein_coding	ENSG0000016355	5584	9404
2781	TANK	0.948	0.010	TRAF family member associated NFKB activator [Source:HGNC Symbol;Acc:HGNC:11562	chr2:161993418-162092732	NM_001199135, NM	XM_017003096, XM_0	NP_001186064, NP_00	XP_005246264, XP_0168	protein_coding	ENSG0000013656	10010	11562
2782	ZBTB11	0.983	0.010	zinc finger and BTB domain containing 11 [Source:HGNC Symbol;Acc:HGNC:16740]	chr3:101367732-101396339	NM_014415	XM_011512689	NP_055230	XP_011510991	protein_coding	ENSG0000006642	27107	16740
2783	ATP10D	1.182	0.010	ATPase phospholipid transporting 10D (putative) [Source:HGNC Symbol;Acc:HGNC:1354	chr4:47487304-47595503	NM_020453	XM_011513722, XM_0	NP_065186	XP_005248176, XP_0052	protein_coding	ENSG0000014524	57205	13549
2784	SLC7A7	-0.894	0.010	solute carrier family 7 member 7 [Source:HGNC Symbol;Acc:HGNC:11065]	chr14:23242430-23291750	NM_001126105, NM	XM_011537299, XM_0	NP_001119577, NP_00	XP_006720365, XP_0115	protein_coding	ENSG0000015546	9056	11065
2785	SMIM10L2B	-1.060	0.010	small integral membrane protein 10 like 2B [Source:HGNC Symbol;Acc:HGNC:34500]	chrX:134229016-134232664	.	.	.	.	protein_coding	ENSG0000019697	.	34500
2786	TMEM116	1.082	0.010	transmembrane protein 116 [Source:HGNC Symbol;Acc:HGNC:25084]	chr12:112369085-112450943	NM_001193453, NM	XM_011538958, XM_0	NP_001180382, NP_00	XP_006719741, XP_0115	protein_coding	ENSG0000019827	89894	25084
2787	ZBTB49	1.021	0.010	zinc finger and BTB domain containing 49 [Source:HGNC Symbol;Acc:HGNC:19883]	chr4:4291977-4323513	NM_145291	XM_006713864	NP_660334	XP_006713927, XP_0168	protein_coding	ENSG0000016882	166793	19883
2788	MPG	-0.856	0.010	N-methylpurine DNA glycosylase [Source:HGNC Symbol;Acc:HGNC:7211]	chr16:127017-188859	NM_001015052, NM	.	NP_001015052, NP_00	.	protein_coding	ENSG0000010315	4350	7211
2789	RASGRP2	-1.337	0.010	RAS guanyl releasing protein 2 [Source:HGNC Symbol;Acc:HGNC:9879]	chr11:64494382-64512928	NM_001098670, NM	XM_011544724, XM_0	NP_001092140, NP_00	XP_005273764, XP_0115	protein_coding	ENSG0000006883	10235	9879
2790	RTCA	0.828	0.010	RNA 3'-terminal phosphate cyclase [Source:HGNC Symbol;Acc:HGNC:17981]	chr1:100731762-100758325	NM_001130841, NM	XM_005271299, XM_0	NP_001124313, NP_00	XP_005271355, XP_0052	protein_coding	ENSG0000013799	8634	17981
2791	SESN1	1.054	0.010	sestrin 1 [Source:HGNC Symbol;Acc:HGNC:21595]	chr6:109307639-109416022	NM_001199933, NM	.	NP_001186862, NP_00	.	protein_coding	ENSG0000008054	27244	21595
2792	RFX7	0.916	0.010	regulatory factor X7 [Source:HGNC Symbol;Acc:HGNC:25777]	chr15:56379480-56535464	NM_022841	XM_011521926, XM_0	NP_073752	XP_005254660, XP_0115	protein_coding	ENSG0000018182	64864	25777
2793	TRIM38	0.791	0.010	tripartite motif containing 38 [Source:HGNC Symbol;Acc:HGNC:10059]	chr6:25963029-25991454	NM_006355	XM_005248800, XM_0	NP_006346	XP_005248856, XP_0052	protein_coding	ENSG0000011234	10475	10059
2794	BSG	-0.608	0.010	basigin (Ok blood group) [Source:HGNC Symbol;Acc:HGNC:1116]	chr19:571276-583493	NM_001322243, NM	XM_017027173, XM_0	NP_001309172, NP_00	XP_005259676, XP_0168	protein_coding	ENSG0000017227	682	1116
2795	PHLDB1	-0.699	0.010	pleckstrin homology like domain family B member 1 [Source:HGNC Symbol;Acc:HGNC:2	chr11:118477154-118550382	NM_001144758, NM	XM_011542710, XM_0	NP_001138230, NP_00	XP_005271525, XP_0052	protein_coding	ENSG0000001914	102466719	23697
2796	QPCTL	-0.765	0.010	glutaminyl-peptide cyclotransferase like [Source:HGNC Symbol;Acc:HGNC:25952]	chr19:46195740-46207247	NM_001163377, NM	.	NP_001156849, NP_06	.	protein_coding	ENSG0000001147	54814	25952
2797	FAM131C	-0.908	0.010	family with sequence similarity 131 member C [Source:HGNC Symbol;Acc:HGNC:26717]	chr1:16384263-16400121	NM_182623	.	NP_872429	.	protein_coding	ENSG0000018551	348487	26717
2798	KISS1R	-1.075	0.010	KISS1 receptor [Source:HGNC Symbol;Acc:HGNC:4510]	chr19:917341-921015	NM_032551	.	NP_115940	XP_016882871	protein_coding	ENSG0000011601	84634	4510
2799	RAB40C	-0.822	0.010	RAB40C, member RAS oncogene family [Source:HGNC Symbol;Acc:HGNC:18285]	chr16:639356-679272	NM_001172663, NM	.	NP_001166134, NP_00	.	protein_coding	ENSG0000019756	57799	18285
2800	SLC6A8	-0.660	0.010	solute carrier family 6 member 8 [Source:HGNC Symbol;Acc:HGNC:11055]	chrX:152953553-152962048	NM_001142805, NM	.	NP_001136277, NP_00	.	protein_coding	ENSG0000013082	6535	11055
2801	UQCR11	-0.795	0.010	ubiquinol-cytochrome c reductase, complex III subunit XI [Source:HGNC Symbol;Acc:HG	chr19:1597170-1605489	NM_006830	.	NP_006821	.	protein_coding	ENSG0000012754	10975	30862
2802	ZNF699	1.180	0.010	zinc finger protein 699 [Source:HGNC Symbol;Acc:HGNC:24750]	chr19:9404950-9420249	NM_198535	XM_005259897	NP_940937	XP_005259954	protein_coding	ENSG0000019611	374879	24750
2803	NEK2	0.811	0.010	NIMA related kinase 2 [Source:HGNC Symbol;Acc:HGNC:7745]	chr1:211831998-211848972	NM_001204182, NM	XM_005273147	NP_001191111, NP_00	XP_005273204	protein_coding	ENSG0000011765	4751	7745
2804	CCDC47	0.781	0.010	coiled-coil domain containing 47 [Source:HGNC Symbol;Acc:HGNC:24856]	chr17:61822609-61851135	NM_020198	XM_005257527	NP_064583	XP_005257584	protein_coding	ENSG0000010858	57003	24856
2805	DSP	0.599	0.010	desmoplakin [Source:HGNC Symbol;Acc:HGNC:3052]	chr6:7541807-7586950	NM_001008844, NM	XM_011514323	NP_001008844, NP_00	.	protein_coding	ENSG0000009669	1832	3052
2806	PQLC1	-0.812	0.010	PQ loop repeat containing 1 [Source:HGNC Symbol;Acc:HGNC:26188]	chr18:77662419-77711664	NM_001146343, NM	XM_011526184, XM_0	NP_001139815, NP_00	XP_005266827	protein_coding	ENSG0000012249	80148	26188

2807	SRSF11	0.725	0.010	serine and arginine rich splicing factor 11 [Source:HGNC Symbol;Acc:HGNC:10782]	chr1:70671364-70718735	NM_001190987, N	XM_011542431, XM_0	NP_001177916, NP_00	XP_005271395, XP_0052	protein_coding	ENSG0000011675	9295	10782
2808	TTK	0.750	0.010	TTK protein kinase [Source:HGNC Symbol;Acc:HGNC:12401]	chr6:80713603-80752244	NM_001166691, N	XM_011536100, XM_0	NP_001160163, NP_00	XP_011534401, XP_0115	protein_coding	ENSG0000011274	7272	12401
2809	ERI3	-0.692	0.010	ERI1 exoribonuclease family member 3 [Source:HGNC Symbol;Acc:HGNC:17276]	chr1:44686741-44820880	NM_001301698, N	XM_017002301, XM_0	NP_001288627, NP_07	XP_005271243, XP_0067	protein_coding	ENSG0000011741	79033	17276
2810	PPP1R1B	-1.270	0.010	protein phosphatase 1 regulatory inhibitor subunit 1B [Source:HGNC Symbol;Acc:HGNC	chr17:37782992-37792879	NM_001242464, N	XM_017025217, XM_0	NP_001229393, NP_11	XP_006722200, XP_0168	protein_coding	ENSG0000013177	84152	9287
2811	RAB42	-0.718	0.010	RAB42, member RAS oncogene family [Source:HGNC Symbol;Acc:HGNC:28702]	chr1:28918711-28921955	NM_152304	.	NP_689517	.	protein_coding	ENSG0000018806	115273	28702
2812	XAB2	-0.677	0.010	XPA binding protein 2 [Source:HGNC Symbol;Acc:HGNC:14089]	chr19:7684410-7694451	NM_020196	.	NP_064581	.	protein_coding	ENSG0000007692	56949	14089
2813	DENND4A	1.350	0.010	DENN domain containing 4A [Source:HGNC Symbol;Acc:HGNC:24321]	chr15:65903742-66084462	NM_001144823, N	XM_017021862, XM_0	NP_001138295, NP_00	XP_005254177, XP_0052	protein_coding	ENSG0000017448	10260	24321
2814	PCDH1	-0.644	0.010	protocadherin 1 [Source:HGNC Symbol;Acc:HGNC:8655]	chr5:141232675-141257975	NM_001278613, N	.	NP_001265542, NP_00	XP_005268509	protein_coding	ENSG0000015645	5097	8655
2815	RAB40B	-1.023	0.010	RAB40B, member RAS oncogene family [Source:HGNC Symbol;Acc:HGNC:18284]	chr17:80612848-80656604	NM_006822	.	NP_006813	.	protein_coding	ENSG0000014154	10966	18284
2816	SASS6	1.148	0.010	SAS-6 centriolar assembly protein [Source:HGNC Symbol;Acc:HGNC:25403]	chr1:100549118-100598511	NM_001304829, N	.	NP_001291758, NP_91	XP_016855975	protein_coding	ENSG0000015687	163786	25403
2817	BCAS4	-0.853	0.010	breast carcinoma amplified sequence 4 [Source:HGNC Symbol;Acc:HGNC:14367]	chr20:49411430-49499213	NM_001010974, N	XM_011528887	NP_001010974, NP_06	XP_011527189	protein_coding	ENSG0000012424	55653	14367
2818	CPEB2	1.241	0.010	cytoplasmic polyadenylation element binding protein 2 [Source:HGNC Symbol;Acc:HGN	chr4:15004297-15071777	NM_001177381, N	XM_011513777, XM_0	NP_001170852, NP_00	XP_005248192, XP_0115	protein_coding	ENSG0000013744	132864	21745
2819	EFL1	0.795	0.010	elongation factor like GTPase 1 [Source:HGNC Symbol;Acc:HGNC:25789]	chr15:82422570-82555104	NM_001040610, N	XM_011522000, XM_0	NP_001035700, NP_00	XP_011520300, XP_0115	protein_coding	ENSG0000014059	79631	25789
2820	EPC2	0.948	0.010	enhancer of polycomb homolog 2 [Source:HGNC Symbol;Acc:HGNC:24543]	chr2:149402552-149545137	NM_015630	XM_011510941	NP_056445	XP_011509243	protein_coding	ENSG0000013599	26122	24543
2821	LRRC73	-1.356	0.010	leucine rich repeat containing 73 [Source:HGNC Symbol;Acc:HGNC:21375]	chr6:43474706-43478424	NM_001012974, N	.	NP_001012992, NP_00	.	protein_coding	ENSG0000020405	221424	21375
2822	MORC4	0.708	0.010	MORC family CW-type zinc finger 4 [Source:HGNC Symbol;Acc:HGNC:23485]	chrX:106183963-106243474	NM_001085354, N	XM_006724691, XM_0	NP_001078823, NP_07	XP_005262247, XP_0067	protein_coding	ENSG0000013313	79710	23485
2823	OCRL	0.685	0.010	OCRL, inositol polyphosphate-5-phosphatase [Source:HGNC Symbol;Acc:HGNC:8108]	chrX:128674251-128726538	NM_000276, NM_0	XM_011531346, XM_0	NP_000267, NP_00130	XP_005262479, XP_0115	protein_coding	ENSG0000012212	4952	8108
2824	SPDL1	0.905	0.010	spindle apparatus coiled-coil protein 1 [Source:HGNC Symbol;Acc:HGNC:26010]	chr5:169010637-169031782	NM_001329641, N	XM_011534589, XM_0	NP_001316570, NP_06	XP_011532889, XP_0115	protein_coding	ENSG0000004027	54908	26010
2825	ZNF143	0.917	0.010	zinc finger protein 143 [Source:HGNC Symbol;Acc:HGNC:12928]	chr11:9482068-9550071	NM_001282656, N	XM_017018255, XM_0	NP_001269585, NP_00	XP_005253178, XP_0052	protein_coding	ENSG0000016647	7702	12928
2826	P2RX5	-0.720	0.010	purinergic receptor P2X 5 [Source:HGNC Symbol;Acc:HGNC:8536]	chr17:3575492-3599698	NM_001204519, N	.	NP_001191448, NP_00	.	protein_coding	ENSG0000008345	5026	8536
2827	ANTXR2	1.624	0.010	anthrax toxin receptor 2 [Source:HGNC Symbol;Acc:HGNC:21732]	chr4:80822302-80994626	NM_001145794, N	XM_011531587	NP_001139266, NP_00	XP_011529889	protein_coding	ENSG0000016329	118429	21732
2828	RBM26	0.841	0.010	RNA binding motif protein 26 [Source:HGNC Symbol;Acc:HGNC:20327]	chr13:79893002-79980356	NM_001286631, N	XM_011535195, XM_0	NP_001273560, NP_00	XP_005266548, XP_0052	protein_coding	ENSG0000013974	64062	20327
2829	YIF1B	-0.722	0.010	Yip1 interacting factor homolog B, membrane trafficking protein [Source:HGNC Symbol;	chr19:38795743-38806603	NM_001039671, N	XM_017027450, XM_0	NP_001034760, NP_00	XP_005259442, XP_0115	protein_coding	ENSG0000016764	90522	30511
2830	ARMC8	0.980	0.011	armadillo repeat containing 8 [Source:HGNC Symbol;Acc:HGNC:24999]	chr3:137906089-138048728	NM_001267041, N	XM_011512633, XM_0	NP_001253970, NP_00	XP_006713627, XP_0067	protein_coding	ENSG0000011409	25852	24999
2831	C12orf10	-0.629	0.011	chromosome 12 open reading frame 10 [Source:HGNC Symbol;Acc:HGNC:17590]	chr12:53693469-53700961	NM_021640	.	NP_067653	.	protein_coding	ENSG0000013963	60314	17590
2832	CD164L2	-1.116	0.011	CD164 molecule like 2 [Source:HGNC Symbol;Acc:HGNC:32043]	chr1:27705665-27709870	NM_207397	XM_011541441, XM_0	NP_997280	XP_005245925, XP_0115	protein_coding	ENSG0000017495	388611	32043
2833	IFRD1	0.638	0.011	interferon related developmental regulator 1 [Source:HGNC Symbol;Acc:HGNC:5456]	chr7:112063198-112117255	NM_001007245, N	XM_011516143, XM_0	NP_001007246, NP_00	XP_011514444, XP_0115	protein_coding	ENSG0000000665	3475	5456
2834	PIK3R1	1.008	0.011	phosphoinositide-3-kinase regulatory subunit 1 [Source:HGNC Symbol;Acc:HGNC:8979]	chr5:67511547-67597649	NM_001242466, N	XM_011543493, XM_0	NP_001229395, NP_85	XP_005248599, XP_0115	protein_coding	ENSG0000014567	5295	8979
2835	FAM21A	1.026	0.011	family with sequence similarity 21 member A [Source:HGNC Symbol;Acc:HGNC:23416]	chr10:51827647-51893266	NM_001005751, N	XM_017016233, XM_0	NP_001005751, NP_00	XP_005269863, XP_0052	protein_coding	ENSG0000009929	387680	23416
2836	IGSF8	-0.715	0.011	immunoglobulin superfamily member 8 [Source:HGNC Symbol;Acc:HGNC:17813]	chr1:160061129-160068618	NM_001206665, N	XM_017002837, XM_0	NP_001193594, NP_00	XP_005245671, XP_0067	protein_coding	ENSG0000016272	93185	17813
2837	SURF1	-0.715	0.011	surfeit 1 [Source:HGNC Symbol;Acc:HGNC:11474]	chr9:136218609-136223361	NM_001280787, N	XM_011518942	NP_001267716, NP_00	XP_011517244	protein_coding	ENSG0000014829	6834	11474
2838	AP4E1	0.968	0.011	adaptor related protein complex 4 epsilon 1 subunit [Source:HGNC Symbol;Acc:HGNC:5	chr15:51200869-51298097	NM_001252127, N	XM_011521409, XM_0	NP_001239056, NP_03	XP_005254321, XP_0067	protein_coding	ENSG0000008101	23431	573
2839	CRNKL1	0.724	0.011	crooked neck pre-mRNA splicing factor 1 [Source:HGNC Symbol;Acc:HGNC:15762]	chr20:20015011-20341346	NM_001278625, N	.	NP_001265554, NP_00	.	protein_coding	ENSG0000010134	51340	15762
2840	FAM199X	0.708	0.011	family with sequence similarity 199, X-linked [Source:HGNC Symbol;Acc:HGNC:25195]	chrX:103411300-103440583	NM_207318	XM_017029270, XM_0	NP_997201	XP_005262136, XP_0168	protein_coding	ENSG0000012357	139231	25195
2841	GSC	-3.222	0.011	goosecoid homeobox [Source:HGNC Symbol;Acc:HGNC:4612]	chr14:95234552-95236567	NM_173849	.	NP_776248	.	protein_coding	ENSG0000013393	145258	4612
2842	SLC35A5	1.004	0.011	solute carrier family 35 member A5 [Source:HGNC Symbol;Acc:HGNC:20792]	chr3:112280856-112304424	NM_017945	XM_011512948	NP_060415	XP_011511250	protein_coding	ENSG0000013845	55032	20792
2843	LRRC1	0.793	0.011	leucine rich repeat containing 1 [Source:HGNC Symbol;Acc:HGNC:14307]	chr6:53659777-53788919	NM_018214	XM_011514726	NP_060684	XP_011513028	protein_coding	ENSG0000013726	55227	14307
2844	SGSM3	-0.939	0.011	small G protein signaling modulator 3 [Source:HGNC Symbol;Acc:HGNC:25228]	chr22:40766614-41032695	NM_001301849, N	XM_011530162, XM_0	NP_001288778, NP_05	XP_005261629, XP_0052	protein_coding	ENSG0000010035	27352	25228
2845	VPS13A	1.064	0.011	vacuolar protein sorting 13 homolog A [Source:HGNC Symbol;Acc:HGNC:1908]	chr9:79792268-80036457	NM_001018037, N	.	NP_001018047, NP_00	.	protein_coding	ENSG0000019796	23230	1908
2846	ADAL	1.050	0.011	adenosine deaminase like [Source:HGNC Symbol;Acc:HGNC:31853]	chr15:43622871-43646753	NM_001012969, N	XM_011521297, XM_0	NP_001012987, NP_00	XP_005254261, XP_0115	protein_coding	ENSG0000016880	161823	31853
2847	HELQ	1.011	0.011	helicase, POLQ-like [Source:HGNC Symbol;Acc:HGNC:18536]	chr4:84328495-84377009	NM_001297755, N	XM_011531579, XM_0	NP_001284684, NP_00	XP_005262768, XP_0052	protein_coding	ENSG0000016331	113510	18536
2848	ZBTB45	-0.866	0.011	zinc finger and BTB domain containing 45 [Source:HGNC Symbol;Acc:HGNC:23715]	chr19:59024896-59031589	NM_001316979, N	XM_006723445, XM_0	NP_001303907, NP_00	XP_005259398, XP_0052	protein_coding	ENSG0000011957	84878	23715
2849	ASAH1	0.810	0.011	N-acylsphingosine amidohydrolase 1 [Source:HGNC Symbol;Acc:HGNC:735]	chr8:17913500-17942507	NM_001127505, N	XM_005273504	NP_001120977, NP_00	XP_005273561	protein_coding	ENSG0000010476	427	735
2850	BAG4	1.562	0.011	BCL2 associated athanogene 4 [Source:HGNC Symbol;Acc:HGNC:940]	chr8:38020838-38070819	NM_001204878, N	.	NP_001191807, NP_00	.	protein_coding	ENSG0000015673	9530	940
2851	IWS1	0.693	0.011	IWS1, SUPT6H interacting protein [Source:HGNC Symbol;Acc:HGNC:25467]	chr2:128238381-128284044	NM_017969	XM_017004468, XM_0	NP_060439	XP_005263764, XP_0067	protein_coding	ENSG0000016316	55677	25467
2852	KPNA3	0.705	0.011	karyopherin subunit alpha 3 [Source:HGNC Symbol;Acc:HGNC:6396]	chr13:50273442-50367057	NM_002267	.	NP_002258	XP_016876050	protein_coding	ENSG0000010275	3839	6396
2853	SQSTM1	-0.882	0.011	sequestosome 1 [Source:HGNC Symbol;Acc:HGNC:11280]	chr5:179247758-179285887	NM_001142298, N	.	NP_001135770, NP_00	XP_016865499	protein_coding	ENSG0000016101	8878	11280
2854	TBL3	-0.689	0.011	transducin beta like 3 [Source:HGNC Symbol;Acc:HGNC:11587]	chr16:2022037-2032934	NM_006453	.	NP_006444	.	protein_coding	ENSG0000018375	10607	11587
2855	KDM4B	-0.761	0.011	lysine demethylase 4B [Source:HGNC Symbol;Acc:HGNC:29136]	chr19:4969123-5153606	NM_015015	XM_011527818, XM_0	NP_055830	XP_005259578, XP_0115	protein_coding	ENSG0000012766	23030	29136
2856	MRPS25	-1.208	0.011	mitochondrial ribosomal protein S25 [Source:HGNC Symbol;Acc:HGNC:14511]	chr3:14989090-15106842	NM_022497	XM_011534014	NP_071942	XP_011532316	protein_coding	ENSG0000013136	64432	14511
2857	RNF38	0.876	0.011	ring finger protein 38 [Source:HGNC Symbol;Acc:HGNC:18052]	chr9:36336392-36401195	NM_022781, NM_1	XM_017014297, XM_0	NP_073618, NP_91930	XP_005251423, XP_0052	protein_coding	ENSG0000013707	152006	18052
2858	PTPN23	-0.655	0.011	protein tyrosine phosphatase, non-receptor type 23 [Source:HGNC Symbol;Acc:HGNC:1	chr3:47422509-47454931	NM_001304482, N	XM_005265031	NP_001291411, NP_05	XP_005265088	protein_coding	ENSG0000007620	25930	14406
2859	SRSF10	0.757	0.011	serine and arginine rich splicing factor 10 [Source:HGNC Symbol;Acc:HGNC:16713]	chr1:24291293-24306846	NM_001191005, N	.	NP_001177934, NP_00	.	protein_coding	ENSG0000018852	10772	16713
2860	ABCB8	-0.771	0.011	ATP binding cassette subfamily B member 8 [Source:HGNC Symbol;Acc:HGNC:49]	chr7:150725508-150744869	NM_001282291, N	.	NP_001269220, NP_00	.	protein_coding	ENSG0000019715	11194	49

2861	RHBDD1	0.894	0.011	rhomboid domain containing 1 [Source:HGNC Symbol;Acc:HGNC:23081]	chr2:227700651-227863931	NM_001167608, N	XM_017005091, XM_	NP_001161080, NP_11	XP_016860572, XP_0168	protein_coding	ENSG0000014446	84236	23081
2862	SH3RF1	0.720	0.011	SH3 domain containing ring finger 1 [Source:HGNC Symbol;Acc:HGNC:17650]	chr4:170015406-170192256	NM_020870	.	NP_065921	.	protein_coding	ENSG0000015444	57630	17650
2863	TRAIP	-0.781	0.011	TRAF interacting protein [Source:HGNC Symbol;Acc:HGNC:30764]	chr3:49866031-49894007	NM_005879	XM_011533264	NP_005870	XP_011531566	protein_coding	ENSG0000018376	10293	30764
2864	LAT2	-0.806	0.011	linker for activation of T-cells family member 2 [Source:HGNC Symbol;Acc:HGNC:12749]	chr7:73623725-73644164	NM_014146, NM_0	XM_011516558	NP_054865, NP_11585	XP_011514860	protein_coding	ENSG0000008673	7462	12749
2865	RBPJ	0.955	0.011	recombination signal binding protein for immunoglobulin kappa J region [Source:HGNC	chr4:26321331-26436753	NM_005349, NM_0	XM_017008170, XM_	NP_005340, NP_05695	XP_005248218, XP_0067	protein_coding	ENSG0000016821	3516	5724
2866	ASH2L	0.760	0.011	ASH2 like histone lysine methyltransferase complex subunit [Source:HGNC Symbol;Acc:	chr8:37962759-37997598	NM_001105214, N	XM_006716413, XM_	NP_001098684, NP_00	XP_005273739, XP_0052	protein_coding	ENSG0000012969	9070	744
2867	NOLC1	0.602	0.011	nucleolar and coiled-body phosphoprotein 1 [Source:HGNC Symbol;Acc:HGNC:15608]	chr10:103911932-103923628	NM_001284388, N	XM_005270273	NP_001271317, NP_00	XP_005270330	protein_coding	ENSG0000016619	9221	15608
2868	AURKA	0.653	0.011	aurora kinase A [Source:HGNC Symbol;Acc:HGNC:11393]	chr20:54944444-54967393	NM_001323304, N	XM_017028035, XM_	NP_001310232, NP_00	XP_005260591, XP_0067	protein_coding	ENSG0000008758	6790	11393
2869	CDA	-0.709	0.011	cytidine deaminase [Source:HGNC Symbol;Acc:HGNC:1712]	chr1:20915440-20945401	NM_001785	.	NP_001776	.	protein_coding	ENSG0000015882	978	1712
2870	MCC	0.800	0.011	mutated in colorectal cancers [Source:HGNC Symbol;Acc:HGNC:6935]	chr5:112357795-112824527	NM_001085377, N	XM_005271991	NP_001078846, NP_00	XP_005272048, XP_0168	protein_coding	ENSG0000017144	4163	6935
2871	FGF20	0.929	0.011	fibroblast growth factor 20 [Source:HGNC Symbol;Acc:HGNC:3677]	chr8:16849677-16859690	NM_019851	.	NP_062825	.	protein_coding	ENSG0000007857	26281	3677
2872	PIP5K1A	0.655	0.011	phosphatidylinositol-4-phosphate 5-kinase type 1 alpha [Source:HGNC Symbol;Acc:HGN	chr1:151171027-151222007	NM_001135636, N	XM_011510050, XM_	NP_001129108, NP_00	XP_005245582, XP_0052	protein_coding	ENSG0000014339	8394	8994
2873	POC1B	0.814	0.011	POC1 centriolar protein B [Source:HGNC Symbol;Acc:HGNC:30836]	chr12:89813494-89920039	NM_001199777, N	.	NP_001186706, NP_75	.	protein_coding	ENSG0000013932	282809	30836
2874	RIC1	0.937	0.011	RIC1 homolog, RAB6A GEF complex partner 1 [Source:HGNC Symbol;Acc:HGNC:17686]	chr9:5629024-5776557	NM_001135920, N	XM_005251523	NP_001129392, NP_00	XP_005251580, XP_0168	protein_coding	ENSG0000010703	57589	17686
2875	STK33	1.087	0.011	serine/threonine kinase 33 [Source:HGNC Symbol;Acc:HGNC:14568]	chr11:8413417-8615775	NM_001289058, N	XM_011520298, XM_	NP_001275987, NP_00	XP_011518590, XP_0115	protein_coding	ENSG0000013041	65975	14568
2876	USP46	0.949	0.011	ubiquitin specific peptidase 46 [Source:HGNC Symbol;Acc:HGNC:20075]	chr4:53457137-53525502	NM_001134223, N	.	NP_001127695, NP_00	XP_016864046	protein_coding	ENSG0000010918	64854	20075
2877	ZNF189	0.914	0.011	zinc finger protein 189 [Source:HGNC Symbol;Acc:HGNC:12980]	chr9:104161154-104172942	NM_001278231, N	XM_017015121, XM_	NP_001265160, NP_00	XP_006717343, XP_0067	protein_coding	ENSG0000013687	7743	12980
2878	PLEKHA8	1.013	0.011	pleckstrin homology domain containing A8 [Source:HGNC Symbol;Acc:HGNC:30037]	chr7:30067976-30170099	NM_001197026, N	XM_011515596, XM_	NP_001183955, NP_00	XP_005249954, XP_0115	protein_coding	ENSG0000010608	84725	30037
2879	SPOPL	0.856	0.011	speckle type BTB/POZ protein like [Source:HGNC Symbol;Acc:HGNC:27934]	chr2:139259370-139331117	NM_001001664	XM_005263657, XM_	NP_001001664	XP_005263712, XP_0052	protein_coding	ENSG0000014422	339745	27934
2880	ZBTB38	0.750	0.011	zinc finger and BTB domain containing 38 [Source:HGNC Symbol;Acc:HGNC:26636]	chr3:141106653-141168634	NM_001080412	XM_011512611, XM_	NP_001073881	XP_005247313, XP_0052	protein_coding	ENSG0000017731	253461	26636
2881	CTSL	-0.712	0.011	cathepsin L [Source:HGNC Symbol;Acc:HGNC:2537]	chr9:90340433-90346307	NM_001257971, N	XM_011518263, XM_	NP_001244900, NP_00	XP_005251773, XP_0115	protein_coding	ENSG0000013504	1514	2537
2882	CTTNBP2NL	0.861	0.011	CTTNBP2 N-terminal like [Source:HGNC Symbol;Acc:HGNC:25330]	chr1:112938802-113003786	NM_018704	XM_011541781	NP_061174	XP_011540083, XP_0168	protein_coding	ENSG0000014307	55917	25330
2883	LEMD3	0.824	0.011	LEM domain containing 3 [Source:HGNC Symbol;Acc:HGNC:28887]	chr12:65563350-65642107	NM_001167614, N	.	NP_001161086, NP_05	.	protein_coding	ENSG0000017410	23592	28887
2884	C1orf106	-0.790	0.011	chromosome 1 open reading frame 106 [Source:HGNC Symbol;Acc:HGNC:25599]	chr1:200860468-200884863	NM_001142569, N	XM_011509755, XM_	NP_001136041, NP_06	XP_011508056, XP_0115	protein_coding	ENSG0000016336	55765	25599
2885	HSPA8	0.666	0.011	heat shock protein family A (Hsp70) member 8 [Source:HGNC Symbol;Acc:HGNC:5241]	chr11:122928196-122933043	NM_006597, NM_1	XM_011542798	NP_006588, NP_69488	XP_011541100	protein_coding	ENSG0000010997	3312	5241
2886	MCAT	-0.788	0.011	malonyl-CoA-acyl carrier protein transacylase [Source:HGNC Symbol;Acc:HGNC:29622]	chr22:43528211-43539400	NM_014507, NM_1	.	NP_055322, NP_77573	.	protein_coding	ENSG0000010029	27349	29622
2887	PLK1	-0.605	0.011	polo like kinase 1 [Source:HGNC Symbol;Acc:HGNC:9077]	chr16:23690142-23724821	NM_005030	.	NP_005021	.	protein_coding	ENSG0000016685	5347	9077
2888	STRA6	-0.648	0.011	stimulated by retinoic acid 6 [Source:HGNC Symbol;Acc:HGNC:30650]	chr15:74471806-74504608	NM_001142617, N	XM_017022479, XM_	NP_001136089, NP_00	XP_011520185, XP_0115	protein_coding	ENSG0000013786	64220	30650
2889	TAB2	0.776	0.011	TGF-beta activated kinase 1/MAP3K7 binding protein 2 [Source:HGNC Symbol;Acc:HGN	chr6:149638669-149732749	NM_001292034, N	XM_017010591, XM_	NP_001278963, NP_00	XP_006715466, XP_0115	protein_coding	ENSG0000005520	23118	17075
2890	TBX18	1.187	0.011	T-box 18 [Source:HGNC Symbol;Acc:HGNC:11595]	chr6:85397090-85474237	NM_001080508	.	NP_001073977	.	protein_coding	ENSG0000011283	9096	11595
2891	ABHD6	-0.719	0.011	abhydrolase domain containing 6 [Source:HGNC Symbol;Acc:HGNC:21398]	chr3:58223232-58280461	NM_001320126, N	XM_005265336, XM_	NP_001307055, NP_06	XP_005265391, XP_0052	protein_coding	ENSG0000016368	57406	21398
2892	COX7A2L	-0.625	0.011	cytochrome c oxidase subunit 7A2 like [Source:HGNC Symbol;Acc:HGNC:2289]	chr2:42576477-42596150	NM_004718	XM_011533158	NP_001305965, NP_00	XP_011531460	protein_coding	ENSG0000011594	9167	2289
2893	GJB5	-2.741	0.011	gap junction protein beta 5 [Source:HGNC Symbol;Acc:HGNC:4287]	chr1:35220647-35224113	NM_005268	XM_005270751	NP_005259	XP_005270808	protein_coding	ENSG0000018928	2709	4287
2894	NPTX2	-0.731	0.011	neuronal pentraxin 2 [Source:HGNC Symbol;Acc:HGNC:7953]	chr7:98246608-98259180	NM_002523	.	NP_002514	.	protein_coding	ENSG0000010623	4885	7953
2895	RNASET2	-1.124	0.011	ribonuclease T2 [Source:HGNC Symbol;Acc:HGNC:21686]	chr6:166822851-167370672	NM_003730	.	NP_003721	XP_016866886	protein_coding	ENSG0000002629	8635	21686
2896	SHC2	-1.403	0.011	SHC adaptor protein 2 [Source:HGNC Symbol;Acc:HGNC:29869]	chr19:416582-460996	NM_012435	XM_011527895, XM_	NP_036567	XP_011526195, XP_0115	protein_coding	ENSG0000012994	25759	29869
2897	APRT	-0.613	0.011	adenine phosphoribosyltransferase [Source:HGNC Symbol;Acc:HGNC:626]	chr16:88875746-88878352	NM_000485, NM_0	.	NP_000476, NP_00102	.	protein_coding	ENSG0000019893	353	626
2898	ATF7IP	0.817	0.011	activating transcription factor 7 interacting protein [Source:HGNC Symbol;Acc:HGNC:20	chr12:14518565-14655869	NM_001286514, N	XM_017019639, XM_	NP_001273443, NP_00	XP_005253481, XP_0067	protein_coding	ENSG0000017168	55729	20092
2899	FAM45A	0.949	0.011	family with sequence similarity 45 member A [Source:HGNC Symbol;Acc:HGNC:31793]	chr10:120863628-120896707	NM_001303112, N	XM_011539807, XM_	NP_001290040, NP_00	XP_006717912, XP_0067	protein_coding	ENSG0000011997	404636	31793
2900	LRRC75A	-0.807	0.011	leucine rich repeat containing 75A [Source:HGNC Symbol;Acc:HGNC:32403]	chr17:16345327-16395467	NM_001113567, N	.	NP_001107039, NP_99	.	protein_coding	ENSG0000018135	388341	32403
2901	SF3B5	-0.596	0.011	splicing factor 3b subunit 5 [Source:HGNC Symbol;Acc:HGNC:21083]	chr6:144416017-144416710	NM_031287	.	NP_112577	.	protein_coding	ENSG0000016997	83443	21083
2902	CACUL1	0.719	0.012	CDK2 associated cullin domain 1 [Source:HGNC Symbol;Acc:HGNC:23727]	chr10:120435922-120514758	NM_153810	.	NP_722517	.	protein_coding	ENSG0000015189	143384	23727
2903	SH3GL1	-0.631	0.012	SH3 domain containing GRB2 like 1, endophilin A2 [Source:HGNC Symbol;Acc:HGNC:108	chr19:4360366-4400544	NM_001199943, N	XM_017027146, XM_	NP_001186872, NP_00	XP_011526485, XP_0168	protein_coding	ENSG0000014198	6455	10830
2904	EPG5	0.845	0.012	ectopic P-granules autophagy protein 5 homolog [Source:HGNC Symbol;Acc:HGNC:2933	chr18:43427573-43547240	NM_020964	XM_011526126, XM_	NP_066015	XP_011524424, XP_0168	protein_coding	ENSG0000015222	57724	29331
2905	ABCD4	-0.733	0.012	ATP binding cassette subfamily D member 4 [Source:HGNC Symbol;Acc:HGNC:68]	chr14:74752125-74769759	NM_005050	XM_011537042, XM_	NP_005041	XP_005267996, XP_0052	protein_coding	ENSG0000011968	5826	68
2906	APBB2	0.779	0.012	amyloid beta precursor protein binding family B member 2 [Source:HGNC Symbol;Acc:H	chr4:40812043-41216635	NM_001166050, N	XM_017008149, XM_	NP_001159522, NP_00	XP_005248158, XP_0067	protein_coding	ENSG0000016369	323	582
2907	ARAF	-1.118	0.012	A-Raf proto-oncogene, serine/threonine kinase [Source:HGNC Symbol;Acc:HGNC:646]	chrX:47420498-47479252	NM_001256196, N	XM_011543908, XM_	NP_001243125, NP_00	XP_006724592, XP_0115	protein_coding	ENSG0000007806	369	646
2908	PRDX3	0.652	0.012	peroxiredoxin 3 [Source:HGNC Symbol;Acc:HGNC:9354]	chr10:120927214-120938345	NM_001302272, N	.	NP_001289201, NP_00	.	protein_coding	ENSG0000016567	10935	9354
2909	MOSPD3	-0.728	0.012	motile sperm domain containing 3 [Source:HGNC Symbol;Acc:HGNC:25078]	chr7:100209724-100213007	NM_001040097, N	XM_005250531, XM_	NP_001035186, NP_00	XP_005250586, XP_0052	protein_coding	ENSG0000010633	64598	25078
2910	PIN1	-0.635	0.012	peptidylprolyl cis/trans isomerase, NIMA-interacting 1 [Source:HGNC Symbol;Acc:HGNC	chr19:9945998-9960358	NM_006221	.	NP_006212	.	protein_coding	ENSG0000012744	5300	8988
2911	ZGRF1	1.498	0.012	zinc finger GRF-type containing 1 [Source:HGNC Symbol;Acc:HGNC:25654]	chr4:113460488-113558066	NM_018392	XM_017008369, XM_	NP_060862	XP_005263172, XP_0115	protein_coding	ENSG0000013865	55345	25654
2912	ZNF704	0.713	0.012	zinc finger protein 704 [Source:HGNC Symbol;Acc:HGNC:32291]	chr8:81540685-81787016	NM_001033723	XM_017013725, XM_	NP_001028895	XP_005251336, XP_0168	protein_coding	ENSG0000016468	619279	32291
2913	AGK	0.830	0.012	acylglycerol kinase [Source:HGNC Symbol;Acc:HGNC:21869]	chr7:141250988-141355044	NM_018238	XM_011516397	NP_060708	XP_011514699	protein_coding	ENSG0000000653	55750	21869
2914	HACL1	-0.940	0.012	2-hydroxyacyl-CoA lyase 1 [Source:HGNC Symbol;Acc:HGNC:17856]	chr3:15602210-15687329	NM_001284413, N	.	NP_001271342, NP_00	.	protein_coding	ENSG0000013137	26061	17856

2915	PPWD1	0.855	0.012	peptidylprolyl isomerase domain and WD repeat containing 1 [Source:HGNC Symbol;Ac	chr5:64859065-64883376	NM_001278926, NN	XM_006714583	NP_001265855, NP_00	XP_006714646	protein_coding	ENSG0000011359	23398		28954
2916	SLC25A24	0.766	0.012	solute carrier family 25 member 24 [Source:HGNC Symbol;Acc:HGNC:20662]	chr1:108676657-108743471	NM_013386, NM_2	.	NP_037518, NP_99881	.	protein_coding	ENSG0000008549	29957		20662
2917	ABCC4	0.917	0.012	ATP binding cassette subfamily C member 4 [Source:HGNC Symbol;Acc:HGNC:55]	chr13:95672082-95953700	NM_001105515, NN	XM_017020319, XM_0	NP_001098985, NP_00	XP_005254082, XP_0168	protein_coding	ENSG0000012525	10257		55
2918	MUT	0.861	0.012	methylmalonyl-CoA mutase [Source:HGNC Symbol;Acc:HGNC:7526]	chr6:49398072-49430904	NM_000255	XM_005249143	NP_000246	XP_005249200	protein_coding	ENSG0000014608	4594		7526
2919	PIP4K2A	0.794	0.012	phosphatidylinositol-5-phosphate 4-kinase type 2 alpha [Source:HGNC Symbol;Acc:HGN	chr10:22823777-23003484	NM_005028	XM_017016331, XM_0	NP_005019	XP_006717513, XP_0115	protein_coding	ENSG0000015086	5305		8997
2920	TOM1	-0.781	0.012	target of myb1 membrane trafficking protein [Source:HGNC Symbol;Acc:HGNC:11982]	chr22:35695267-35743987	NM_001135729, NN	XM_017028530, XM_0	NP_001129201, NP_00	XP_011528120, XP_0115	protein_coding	ENSG0000010028	10043		11982
2921	VKORC1L1	0.865	0.012	vitamin K epoxide reductase complex subunit 1 like 1 [Source:HGNC Symbol;Acc:HGNC:	chr7:65338253-65424550	NM_001284342, NN	XM_011515831	NP_001271271, NP_77	XP_011514133	protein_coding	ENSG0000019671	154807		21492
2922	ARRDC3	0.957	0.012	arrestin domain containing 3 [Source:HGNC Symbol;Acc:HGNC:29263]	chr5:90664540-90679176	NM_020801	.	NP_065852	.	protein_coding	ENSG0000011336	57561		29263
2923	MAPK3	-0.658	0.012	mitogen-activated protein kinase 3 [Source:HGNC Symbol;Acc:HGNC:6877]	chr16:30125425-30134630	NM_001040056, NN	.	NP_001035145, NP_00	.	protein_coding	ENSG0000010288	5595		6877
2924	RDH11	0.617	0.012	retinol dehydrogenase 11 (all-trans/9-cis/11-cis) [Source:HGNC Symbol;Acc:HGNC:1796	chr14:68143517-68162531	NM_001252650, NN	.	NP_001239579, NP_05	.	protein_coding	ENSG0000007204	51109		17964
2925	ATAD1	0.828	0.012	ATPase family, AAA domain containing 1 [Source:HGNC Symbol;Acc:HGNC:25903]	chr10:89511268-89577917	NM_001321967, NN	XM_011540302, XM_0	NP_001308896, NP_11	XP_005270308, XP_0052	protein_coding	ENSG0000013813	84896		25903
2926	PEBP1	-0.601	0.012	phosphatidylethanolamine binding protein 1 [Source:HGNC Symbol;Acc:HGNC:8630]	chr12:118573662-118583389	NM_002567	.	NP_002558	.	protein_coding	ENSG0000008922	5037		8630
2927	PRPF4B	0.798	0.012	pre-mRNA processing factor 4B [Source:HGNC Symbol;Acc:HGNC:17346]	chr6:4021559-4062141	NM_003913	.	NP_003904	XP_016866899, XP_0168	protein_coding	ENSG0000011273	8899		17346
2928	RFPL4AL1	2.835	0.012	ret finger protein like 4A like 1 [Source:HGNC Symbol;Acc:HGNC:45147]	chr19:56280506-56284545	NM_001277397	XM_011527259	NP_001264326	XP_011525561	protein_coding	ENSG0000022929	729974		45147
2929	WDR6	-0.865	0.012	WD repeat domain 6 [Source:HGNC Symbol;Acc:HGNC:12758]	chr3:49027318-49060928	NM_001320547, NN	XM_011533314, XM_0	NP_001307475, NP_00	XP_005264894	protein_coding	ENSG0000017825	11180		12758
2930	ARHGEF1	-0.616	0.012	Rho guanine nucleotide exchange factor 1 [Source:HGNC Symbol;Acc:HGNC:681]	chr19:42387227-42411606	NM_004706, NM_1	XM_011527468, XM_0	NP_004697, NP_94532	XP_005259443, XP_0052	protein_coding	ENSG0000007692	100505585		681
2931	SNX25	0.719	0.012	sorting nexin 25 [Source:HGNC Symbol;Acc:HGNC:21883]	chr4:186125390-186317053	NM_001317781, NN	XM_011532309, XM_0	NP_001304710, NP_11	XP_005263324, XP_0115	protein_coding	ENSG0000010976	83891		21883
2932	RAB7A	-0.583	0.012	RAB7A, member RAS oncogene family [Source:HGNC Symbol;Acc:HGNC:9788]	chr3:128444964-128533639	NM_004637	.	NP_004628	.	protein_coding	ENSG0000007578	7879		9788
2933	EBF4	-0.867	0.012	early B-cell factor 4 [Source:HGNC Symbol;Acc:HGNC:29278]	chr20:2673523-2740753	NM_001110514	XM_006723600	NP_001103984	XP_006723663, XP_0168	protein_coding	ENSG0000008888	57593		29278
2934	HOXD1	2.235	0.012	homeobox D1 [Source:HGNC Symbol;Acc:HGNC:5132]	chr2:177053306-177055635	NM_024501	.	NP_078777	.	protein_coding	ENSG0000012864	102465802		5132
2935	YAP1	0.687	0.012	Yes associated protein 1 [Source:HGNC Symbol;Acc:HGNC:16262]	chr11:101981191-102104154	NM_001130145, NN	XM_011542556, XM_0	NP_001123617, NP_00	XP_005271435, XP_0052	protein_coding	ENSG0000013769	10413		16262
2936	AACS	-0.656	0.012	acetoacetyl-CoA synthetase [Source:HGNC Symbol;Acc:HGNC:21298]	chr12:125549924-125627866	NM_023928	XM_005253610, XM_0	NP_076417	.	protein_coding	ENSG0000008176	65985		21298
2937	C20orf27	-0.645	0.012	chromosome 20 open reading frame 27 [Source:HGNC Symbol;Acc:HGNC:15873]	chr20:3734154-3749034	NM_001039140, NN	.	NP_001034229, NP_00	.	protein_coding	ENSG0000010122	54976		15873
2938	CD151	-0.594	0.012	CD151 molecule (Raph blood group) [Source:HGNC Symbol;Acc:HGNC:1630]	chr11:832842-838834	NM_001039490, NN	.	NP_001034579, NP_00	.	protein_coding	ENSG0000017769	977		1630
2939	DARS2	0.667	0.012	aspartyl-tRNA synthetase 2, mitochondrial [Source:HGNC Symbol;Acc:HGNC:25538]	chr1:173793640-173827684	NM_018122	XM_006711427	NP_060592	XP_006711490	protein_coding	ENSG0000011759	55157		25538
2940	FAM217B	1.200	0.012	family with sequence similarity 217 member B [Source:HGNC Symbol;Acc:HGNC:16170]	chr20:58508818-58523735	NM_001190826, NN	XM_011528986, XM_0	NP_001177755, NP_00	XP_011527287, XP_0115	protein_coding	ENSG0000019622	63939		16170
2941	FBXO30	0.868	0.012	F-box protein 30 [Source:HGNC Symbol;Acc:HGNC:15600]	chr6:146114637-146135889	NM_032145	XM_011536177, XM_0	NP_115521	XP_005267216, XP_0115	protein_coding	ENSG0000011849	84085		15600
2942	KATNB1	-0.619	0.012	katanin regulatory subunit B1 [Source:HGNC Symbol;Acc:HGNC:6217]	chr16:57769641-57791162	NM_005886	XM_017022863, XM_0	NP_005877	XP_005255829, XP_0067	protein_coding	ENSG0000014085	10300		6217
2943	OPLAH	-0.814	0.012	5-oxoprolinase (ATP-hydrolysing) [Source:HGNC Symbol;Acc:HGNC:8149]	chr8:145106166-145115606	NM_017570	XM_011516960	NP_060040	XP_011515262	protein_coding	ENSG0000017881	26873		8149
2944	RB1	0.729	0.012	RB transcriptional corepressor 1 [Source:HGNC Symbol;Acc:HGNC:9884]	chr13:48877910-49056122	NM_000321	XM_011535171	NP_000312	XP_011533473	protein_coding	ENSG0000013968	5925		9884
2945	ELOVL6	0.861	0.012	ELOVL fatty acid elongase 6 [Source:HGNC Symbol;Acc:HGNC:15829]	chr4:110967001-111119832	NM_001130721, NN	XM_011532234, XM_0	NP_001124193, NP_07	XP_011530535, XP_0115	protein_coding	ENSG0000017052	79071		15829
2946	S100A3	-3.378	0.012	S100 calcium binding protein A3 [Source:HGNC Symbol;Acc:HGNC:10493]	chr1:153516088-153522612	NM_002960	.	NP_002951	.	protein_coding	ENSG0000018801	6274		10493
2947	SEC14L4	-0.783	0.012	SEC14 like lipid binding 4 [Source:HGNC Symbol;Acc:HGNC:20627]	chr22:30884899-30901682	NM_001161368, NN	XM_006724235, XM_0	NP_001154840, NP_77	XP_006724298	protein_coding	ENSG0000013348	284904		20627
2948	C11orf68	-0.766	0.012	chromosome 11 open reading frame 68 [Source:HGNC Symbol;Acc:HGNC:28801]	chr11:65684278-65686588	NM_001135635, NN	.	NP_001129107, NP_11	.	protein_coding	ENSG0000017557	83638		28801
2949	TRIM15	-0.692	0.012	tripartite motif containing 15 [Source:HGNC Symbol;Acc:HGNC:16284]	chr6:30130982-30140473	NM_033229	XM_011514988, XM_0	NP_150232	XP_011513289, XP_0115	protein_coding	ENSG0000020461	89870		16284
2950	UBR2	0.782	0.012	ubiquitin protein ligase E3 component n-recognin 2 [Source:HGNC Symbol;Acc:HGNC:21	chr6:42531799-42661242	NM_001184801, NN	XM_017010596, XM_0	NP_001171730, NP_05	XP_005249022, XP_0052	protein_coding	ENSG0000002404	23304		21289
2951	VPS37D	-0.970	0.012	VPS37D, ESCRT-I subunit [Source:HGNC Symbol;Acc:HGNC:18287]	chr7:73082154-73086442	NM_001077621	.	NP_001071089	.	protein_coding	ENSG0000017642	155382		18287
2952	CPSF3	0.648	0.012	cleavage and polyadenylation specific factor 3 [Source:HGNC Symbol;Acc:HGNC:2326]	chr2:9563696-9613230	NM_016207	XM_011510362, XM_0	NP_001308762, NP_00	XP_005246225, XP_0115	protein_coding	ENSG0000011920	51692		2326
2953	EVA1B	-0.808	0.012	eva-1 homolog B [Source:HGNC Symbol;Acc:HGNC:25558]	chr1:36787631-36789755	NM_001304762, NN	.	NP_001291691, NP_06	XP_016857116	protein_coding	ENSG0000014269	55194		25558
2954	LOXL1	-0.769	0.012	lysyl oxidase like 1 [Source:HGNC Symbol;Acc:HGNC:6665]	chr15:74218798-74244478	NM_005576	.	NP_005567	XP_011519857	protein_coding	ENSG0000012903	4016		6665
2955	PRKCE	0.899	0.012	protein kinase C epsilon [Source:HGNC Symbol;Acc:HGNC:9401]	chr2:45878912-46415129	NM_005400	XM_011532981, XM_0	NP_005391	XP_005264485, XP_0115	protein_coding	ENSG0000017113	5581		9401
2956	FAM129B	-0.568	0.013	family with sequence similarity 129 member B [Source:HGNC Symbol;Acc:HGNC:25282]	chr9:130267617-130341268	NM_001035534, NN	XM_011518925, XM_0	NP_001030611, NP_07	XP_005252192, XP_0115	protein_coding	ENSG0000013683	64855		25282
2957	GOPC	1.090	0.013	golgi associated PDZ and coiled-coil motif containing [Source:HGNC Symbol;Acc:HGNC:1	chr6:117609462-117923705	NM_001017408, NN	.	NP_001017408, NP_06	.	protein_coding	ENSG0000004793	57120		17643
2958	KIF15	0.841	0.013	kinesin family member 15 [Source:HGNC Symbol;Acc:HGNC:17273]	chr3:44803208-44894753	NM_020242	XM_011533964, XM_0	NP_064627	XP_006713327, XP_0115	protein_coding	ENSG0000016380	56992		17273
2959	LYSMD3	0.950	0.013	LysM domain containing 3 [Source:HGNC Symbol;Acc:HGNC:26969]	chr5:89811427-89825401	NM_001286812, NN	XM_005248421	NP_001273741, NP_93	XP_005248478	protein_coding	ENSG0000017601	116068		26969
2960	MIF	-0.597	0.013	macrophage migration inhibitory factor (glycosylation-inhibiting factor) [Source:HGNC S	chr22:24198889-24237413	NM_002415	.	NP_002406	.	protein_coding	ENSG0000024097	4282		7097
2961	CEACAM1	-0.841	0.013	carcinoembryonic antigen related cell adhesion molecule 1 [Source:HGNC Symbol;Acc:H	chr19:43011455-43032661	NM_001024912, NN	.	NP_001020083, NP_00	.	protein_coding	ENSG0000007938	634		1814
2962	CENPM	-0.753	0.013	centromere protein M [Source:HGNC Symbol;Acc:HGNC:18352]	chr22:42334724-42343168	NM_001002876, NN	.	NP_001002876, NP_00	.	protein_coding	ENSG0000010016	79019		18352
2963	HOXB5	-0.669	0.013	homeobox B5 [Source:HGNC Symbol;Acc:HGNC:5116]	chr17:46668618-46671323	NM_002147	.	NP_002138	.	protein_coding	ENSG0000012007	3215		5116
2964	STAM	0.814	0.013	signal transducing adaptor molecule [Source:HGNC Symbol;Acc:HGNC:11357]	chr10:17686123-17757913	NM_003473	XM_011519695, XM_0	NP_001311212, NP_00	XP_011517997	protein_coding	ENSG0000013673	8027		11357
2965	AGAP6	0.998	0.013	ArfGAP with GTPase domain, ankyrin repeat and PH domain 6 [Source:HGNC Symbol;Ac	chr10:51741949-51770259	NM_001077665, NN	XM_017016270, XM_0	NP_001071133, NP_00	XP_011538117, XP_0168	protein_coding	ENSG0000020414	414189		23466
2966	MYOF	0.687	0.013	myoferlin [Source:HGNC Symbol;Acc:HGNC:3656]	chr10:95066186-95242074	NM_013451, NM_1	XM_011539633, XM_0	NP_038479, NP_57989	XP_005269750, XP_0052	protein_coding	ENSG0000013811	26509		3656
2967	NRTN	-1.028	0.013	neurturin [Source:HGNC Symbol;Acc:HGNC:8007]	chr19:5823812-5828335	NM_004558	XM_011528041	NP_004549	XP_011526343	protein_coding	ENSG0000017111	4902		8007
2968	SLC25A1	-0.603	0.013	solute carrier family 25 member 1 [Source:HGNC Symbol;Acc:HGNC:10979]	chr22:19163087-19166343	NM_001256534, NN	.	NP_001243463, NP_00	.	protein_coding	ENSG0000010007	6576		10979

2969	KPNA4	0.641	0.013	karyopherin subunit alpha 4 [Source:HGNC Symbol;Acc:HGNC:6397]	chr3:160212782-160283376	NM_002268	.	NP_002259	.	protein_coding	ENSG0000018643	3840	6397
2970	NFX1	0.737	0.013	nuclear transcription factor, X-box binding 1 [Source:HGNC Symbol;Acc:HGNC:7803]	chr9:33290508-33371155	NM_001318758, N	XM_005251475, XM_0	NP_001305687, NP_00	.	protein_coding	ENSG0000008610	4799	7803
2971	RAC2	-0.682	0.013	ras-related C3 botulinum toxin substrate 2 (rho family, small GTP binding protein Rac2)	chr22:37621300-37640488	NM_002872	.	NP_002863	.	protein_coding	ENSG0000012834	5880	9802
2972	SIX5	-0.886	0.013	SIX homeobox 5 [Source:HGNC Symbol;Acc:HGNC:10891]	chr19:46268042-46272484	NM_175875	.	NP_787071	.	protein_coding	ENSG0000017704	147912	10891
2973	C6orf62	0.905	0.013	chromosome 6 open reading frame 62 [Source:HGNC Symbol;Acc:HGNC:20998]	chr6:24667262-24721064	NM_030939	XM_011514928	NP_112201	XP_011513230	protein_coding	ENSG0000011230	81688	20998
2974	DSN1	0.715	0.013	DSN1 homolog, MIS12 kinetochore complex component [Source:HGNC Symbol;Acc:HGN	chr20:35380193-35402221	NM_001145315, N	XM_006723876	NP_001138787, NP_00	XP_006723939, XP_0168	protein_coding	ENSG0000014963	79980	16165
2975	FAM72D	1.102	0.013	family with sequence similarity 72 member D [Source:HGNC Symbol;Acc:HGNC:33593]	chr1:143896447-143913143	NM_207418	.	NP_997301	.	protein_coding	ENSG0000021578	728833	33593
2976	HIST1H2BN	1.749	0.013	histone cluster 1, H2bn [Source:HGNC Symbol;Acc:HGNC:4749]	chr6:27805537-27833576	NM_003520	.	NP_003511	.	protein_coding	ENSG0000023382	8341	4749
2977	ME1	0.746	0.013	malic enzyme 1 [Source:HGNC Symbol;Acc:HGNC:6983]	chr6:83920107-84140790	NM_002395	XM_011535836	NP_002386	XP_011534138	protein_coding	ENSG0000006583	4199	6983
2978	NIPAL2	1.078	0.013	NIPA like domain containing 2 [Source:HGNC Symbol;Acc:HGNC:25854]	chr8:99202060-99306621	NM_001321635, N	XM_005251069	NP_001308564, NP_07	.	protein_coding	ENSG0000010436	79815	25854
2979	SMOX	-0.681	0.013	spermine oxidase [Source:HGNC Symbol;Acc:HGNC:15862]	chr20:4129425-4168394	NM_001270691, N	XM_011529261	NP_001257620, NP_78	XP_011527563	protein_coding	ENSG0000008882	54498	15862
2980	TUSC2	-0.621	0.013	tumor suppressor candidate 2 [Source:HGNC Symbol;Acc:HGNC:17034]	chr3:50362337-50365674	NM_007275	.	NP_009206	.	protein_coding	ENSG0000011438	11334	17034
2981	ZSCAN2	-1.143	0.013	zinc finger and SCAN domain containing 2 [Source:HGNC Symbol;Acc:HGNC:20994]	chr15:85144235-85166948	NM_001007072, N	XM_006720596, XM_0	NP_001007073, NP_06	XP_005254992, XP_0067	protein_coding	ENSG0000017637	54993	20994
2982	MKI67	0.587	0.013	marker of proliferation Ki-67 [Source:HGNC Symbol;Acc:HGNC:7107]	chr10:129894922-129924649	NM_001145966, N	XM_011539818	NP_001139438, NP_00	XP_011538120	protein_coding	ENSG0000014877	4288	7107
2983	RNF126	-0.695	0.013	ring finger protein 126 [Source:HGNC Symbol;Acc:HGNC:21151]	chr19:647525-663277	NM_194460	XM_005259594	NP_919442	XP_005259651	protein_coding	ENSG0000007042	55658	21151
2984	USP38	0.931	0.013	ubiquitin specific peptidase 38 [Source:HGNC Symbol;Acc:HGNC:20067]	chr4:144106069-144144983	NM_001290325, N	XM_011532360	NP_001277254, NP_00	XP_011530662	protein_coding	ENSG0000017018	84640	20067
2985	ARIH1	0.749	0.013	ariadne RBR E3 ubiquitin protein ligase 1 [Source:HGNC Symbol;Acc:HGNC:689]	chr15:72766666-72895326	NM_005744	XM_011521439, XM_0	NP_005735	XP_011519740, XP_0115	protein_coding	ENSG0000016623	693215	689
2986	CYR61	0.634	0.013	cysteine rich angiogenic inducer 61 [Source:HGNC Symbol;Acc:HGNC:2654]	chr1:86046443-86049645	NM_001554	.	NP_001545	.	protein_coding	ENSG0000014287	3491	2654
2987	PC	-1.359	0.013	pyruvate carboxylase [Source:HGNC Symbol;Acc:HGNC:8636]	chr11:66615703-66725847	NM_000920, NM_0	XM_017017871, XM_0	NP_000911, NP_00103	XP_005274088, XP_0052	protein_coding	ENSG0000017359	5091	8636
2988	TIMM13	-0.751	0.013	translocase of inner mitochondrial membrane 13 [Source:HGNC Symbol;Acc:HGNC:1181	chr19:2389783-2427892	NM_012458	.	NP_036590	.	protein_coding	ENSG0000009980	26517	11816
2989	CLDN7	-0.701	0.013	claudin 7 [Source:HGNC Symbol;Acc:HGNC:2049]	chr17:7146909-7166532	NM_001185022, N	.	NP_001171951, NP_00	.	protein_coding	ENSG0000018188	1366	2049
2990	VAMP3	0.649	0.013	vesicle associated membrane protein 3 [Source:HGNC Symbol;Acc:HGNC:12644]	chr1:7831328-7841492	NM_004781	.	NP_004772	.	protein_coding	ENSG0000004924	9341	12644
2991	DPP8	0.946	0.013	dipeptidyl peptidase 8 [Source:HGNC Symbol;Acc:HGNC:16490]	chr15:65734800-65810042	NM_017743, NM_1	XM_017022373, XM_0	NP_001307804, NP_00	XP_005254557, XP_0115	protein_coding	ENSG0000007460	54878	16490
2992	EPM2AIP1	0.865	0.013	EPM2A interacting protein 1 [Source:HGNC Symbol;Acc:HGNC:19735]	chr3:37026533-37034659	NM_014805	.	NP_055620	.	protein_coding	ENSG0000017856	9852	19735
2993	JPH3	-0.960	0.013	junctophilin 3 [Source:HGNC Symbol;Acc:HGNC:14203]	chr16:87636510-87736197	NM_020655	.	NP_065706	.	protein_coding	ENSG0000015411	57338	14203
2994	KANSL1	0.741	0.013	KAT8 regulatory NSL complex subunit 1 [Source:HGNC Symbol;Acc:HGNC:24565]	chr17:44107281-44302740	NM_001193465, N	XM_011524632, XM_0	NP_001180394, NP_00	XP_006721886, XP_0067	protein_coding	ENSG0000012007	284058	24565
2995	PPP1R32	-1.390	0.013	protein phosphatase 1 regulatory subunit 32 [Source:HGNC Symbol;Acc:HGNC:28869]	chr11:61248591-61258403	NM_001170753, N	.	NP_001164224, NP_65	.	protein_coding	ENSG0000016214	220004	28869
2996	GTPBP4	0.657	0.013	GTP binding protein 4 [Source:HGNC Symbol;Acc:HGNC:21535]	chr10:1033958-1071799	NM_012341	.	NP_036473	.	protein_coding	ENSG0000010793	23560	21535
2997	NUP160	0.737	0.013	nucleoporin 160 [Source:HGNC Symbol;Acc:HGNC:18017]	chr11:47799638-47870107	NM_001318399, N	XM_006718170	NP_001305328, NP_05	.	protein_coding	ENSG0000003006	23279	18017
2998	STARD7	0.564	0.013	StAR related lipid transfer domain containing 7 [Source:HGNC Symbol;Acc:HGNC:18063	chr2:96850596-96874556	NM_020151	XM_011511499	NP_064536	XP_011509801	protein_coding	ENSG0000008409	56910	18063
2999	HAT1	0.662	0.013	histone acetyltransferase 1 [Source:HGNC Symbol;Acc:HGNC:4821]	chr2:172778974-172848596	NM_003642	XM_011512027, XM_0	NP_003633	XP_006712871, XP_0115	protein_coding	ENSG0000012870	8520	4821
3000	SBF2	0.809	0.013	SET binding factor 2 [Source:HGNC Symbol;Acc:HGNC:2135]	chr11:9800213-10315754	NM_030962	XM_011520394, XM_0	NP_112224	XP_005253211, XP_0052	protein_coding	ENSG0000013381	81846	2135
3001	MSH6	0.669	0.013	mutS homolog 6 [Source:HGNC Symbol;Acc:HGNC:7329]	chr2:48010220-48132932	NM_000179, NM_0	XM_011532800, XM_0	NP_000170, NP_00126	XP_011531100, XP_0115	protein_coding	ENSG0000011606	2956	7329
3002	PTGS1	-1.197	0.013	prostaglandin-endoperoxide synthase 1 [Source:HGNC Symbol;Acc:HGNC:9604]	chr9:125132808-125157982	NM_000962, NM_0	XM_011518876, XM_0	NP_000953, NP_00125	XP_005252162, XP_0115	protein_coding	ENSG0000009530	5742	9604
3003	ZNF282	-0.690	0.013	zinc finger protein 282 [Source:HGNC Symbol;Acc:HGNC:13076]	chr7:148892576-148923339	NM_001303481, N	XM_006716151	NP_001290410, NP_00	XP_006716214	protein_coding	ENSG0000017026	8427	13076
3004	NECTIN3	1.150	0.013	nectin cell adhesion molecule 3 [Source:HGNC Symbol;Acc:HGNC:17664]	chr3:110790589-110912386	NM_001243286, N	XM_011512663	NP_001230215, NP_00	XP_011510965, XP_0168	protein_coding	ENSG0000017770	25945	17664
3005	TMEM42	-0.794	0.013	transmembrane protein 42 [Source:HGNC Symbol;Acc:HGNC:28444]	chr3:44903360-44907162	NM_144638	.	NP_653239	.	protein_coding	ENSG0000016996	693149	28444
3006	EIF2AK4	0.656	0.013	eukaryotic translation initiation factor 2 alpha kinase 4 [Source:HGNC Symbol;Acc:HGN	chr15:40226346-40327791	NM_001013703	XM_011521600, XM_0	NP_001013725	XP_005254449, XP_0115	protein_coding	ENSG0000012882	440275	19687
3007	FAM175B	0.835	0.013	family with sequence similarity 175 member B [Source:HGNC Symbol;Acc:HGNC:28975]	chr10:126490353-126525239	NM_032182	XM_011539554	NP_115558	XP_011537856	protein_coding	ENSG0000016566	23172	28975
3008	DOCK8	1.086	0.013	dedicator of cytokinesis 8 [Source:HGNC Symbol;Acc:HGNC:19191]	chr9:212823-465259	NM_001190458, N	XM_017015174, XM_0	NP_001177387, NP_00	XP_011516347, XP_0115	protein_coding	ENSG0000010709	81704	19191
3009	OCIAD1	0.719	0.013	OCIA domain containing 1 [Source:HGNC Symbol;Acc:HGNC:16074]	chr4:48833014-48863834	NM_001079839, N	.	NP_001073308, NP_00	XP_016863818	protein_coding	ENSG0000010918	54940	16074
3010	SOX12	-0.718	0.013	SRY-box 12 [Source:HGNC Symbol;Acc:HGNC:11198]	chr20:306238-310868	NM_006943	.	NP_008874	.	protein_coding	ENSG0000017773	6666	11198
3011	ZMPSTE24	0.779	0.013	zinc metallopeptidase STE24 [Source:HGNC Symbol;Acc:HGNC:12877]	chr1:40723778-40759856	NM_005857	XM_011540486	NP_005848	.	protein_coding	ENSG0000008407	10269	12877
3012	FGFRL1	-0.633	0.013	fibroblast growth factor receptor-like 1 [Source:HGNC Symbol;Acc:HGNC:3693]	chr4:1003766-1020685	NM_001004356, N	XM_011513487, XM_0	NP_001004356, NP_00	XP_011511788, XP_0115	protein_coding	ENSG0000012741	53834	3693
3013	RPS6KB2	-0.641	0.013	ribosomal protein S6 kinase B2 [Source:HGNC Symbol;Acc:HGNC:10437]	chr11:67195971-67202879	NM_003952	XM_017018108, XM_0	NP_003943	XP_005274221, XP_0052	protein_coding	ENSG0000017563	6199	10437
3014	TNFSF9	-0.810	0.013	tumor necrosis factor superfamily member 9 [Source:HGNC Symbol;Acc:HGNC:11939]	chr19:6531009-6535939	NM_003811	XM_006722931	NP_003802	XP_006722994	protein_coding	ENSG0000012565	8744	11939
3015	CCNF	-0.661	0.013	cyclin F [Source:HGNC Symbol;Acc:HGNC:1591]	chr16:2479394-2508855	NM_001323538, N	XM_017023822	NP_001310467, NP_00	XP_016879311	protein_coding	ENSG0000016206	899	1591
3016	DGKZ	-0.636	0.013	diacylglycerol kinase zeta [Source:HGNC Symbol;Acc:HGNC:2857]	chr11:46354454-46402104	NM_001105540, N	XM_017018456, XM_0	NP_001099010, NP_00	XP_005253238, XP_0052	protein_coding	ENSG0000014909	8525	2857
3017	POLRMT	-0.627	0.013	RNA polymerase mitochondrial [Source:HGNC Symbol;Acc:HGNC:9200]	chr19:617223-633604	NM_005035	XM_005259580	NP_005026	XP_005259637	protein_coding	ENSG0000009982	5442	9200
3018	RNASEH2A	-0.621	0.013	ribonuclease H2 subunit A [Source:HGNC Symbol;Acc:HGNC:18518]	chr19:12917393-12924451	NM_006397	XM_006722619	NP_006388	XP_006722682	protein_coding	ENSG0000010488	10535	18518
3019	ZER1	-0.633	0.013	zyg-11 related cell cycle regulator [Source:HGNC Symbol;Acc:HGNC:30960]	chr9:131492064-131534172	NM_006336	XM_017014188, XM_0	NP_006327	XP_005251702, XP_0052	protein_coding	ENSG0000016044	10444	30960
3020	AP1M2	-0.667	0.013	adaptor related protein complex 1 mu 2 subunit [Source:HGNC Symbol;Acc:HGNC:558]	chr19:10683346-10697991	NM_001300887, N	XM_011527615	NP_001287816, NP_00	.	protein_coding	ENSG0000012935	10053	558
3021	UEVLD	1.179	0.013	UEV and lactate/malate dehyrogenase domains [Source:HGNC Symbol;Acc:HGNC:3086	chr11:18551155-18610293	NM_001040697, N	XM_017017985, XM_0	NP_001035787, NP_00	XP_016873474	protein_coding	ENSG0000015111	55293	30866
3022	AAR2	-0.638	0.014	AAR2 splicing factor homolog [Source:HGNC Symbol;Acc:HGNC:15886]	chr20:34824380-34858840	NM_001271874, N	XM_011528763, XM_0	NP_001258803, NP_05	XP_006723833, XP_0115	protein_coding	ENSG0000013104	25980	15886

3023	CDKL3	1.540	0.014	cyclin dependent kinase like 3 [Source:HGNC Symbol;Acc:HGNC:15483]	chr5:133634109-133706732	NM_001113575, NN	XM_011543433, XM_0	NP_001107047, NP_00	XP_011541731, XP_0115	protein_coding	ENSG0000000683	51265	15483
3024	ERLEC1	0.942	0.014	endoplasmic reticulum lectin 1 [Source:HGNC Symbol;Acc:HGNC:25222]	chr2:53897117-54087297	NM_001127397, NN	XM_011532766	NP_001120869, NP_00	XP_011531068	protein_coding	ENSG0000006891	27248	25222
3025	KIAA1755	-0.924	0.014	KIAA1755 [Source:HGNC Symbol;Acc:HGNC:29372]	chr20:36838889-36922294	NM_001029864	.	NP_001025035	.	protein_coding	ENSG0000014963	85449	29372
3026	UTRN	1.535	0.014	utrophin [Source:HGNC Symbol;Acc:HGNC:12635]	chr6:144612964-145174170	NM_007124	XM_017011243, XM_0	NP_009055	XP_005267184, XP_0052	protein_coding	ENSG0000015281	7402	12635
3027	C7orf25	0.793	0.014	chromosome 7 open reading frame 25 [Source:HGNC Symbol;Acc:HGNC:21703]	chr7:42948324-42951768	NM_001099858, NN	XM_005249844	NP_001093328, NP_07	XP_005249901, XP_0168	protein_coding	ENSG0000013619	79020	21703
3028	EEF2	-0.648	0.014	eukaryotic translation elongation factor 2 [Source:HGNC Symbol;Acc:HGNC:3214]	chr19:3976053-3985467	NM_001961	.	NP_001952	.	protein_coding	ENSG0000016765	1938	3214
3029	PCYOX1	0.682	0.014	prenylcysteine oxidase 1 [Source:HGNC Symbol;Acc:HGNC:20588]	chr2:70485268-70508323	NM_016297	.	NP_057381	.	protein_coding	ENSG0000011600	51449	20588
3030	PTPA	-0.621	0.014	protein phosphatase 2 phosphatase activator [Source:HGNC Symbol;Acc:HGNC:9308]	chr9:131873228-131911225	NM_001193397, NN	XM_011518838, XM_0	NP_001180326, NP_00	XP_011517136, XP_0115	protein_coding	ENSG0000011938	5524	9308
3031	SHARPIN	-0.744	0.014	SHANK associated RH domain interactor [Source:HGNC Symbol;Acc:HGNC:25321]	chr8:145153539-145159093	NM_030974	XM_017013887	NP_112236	XP_016869376, XP_0168	protein_coding	ENSG0000017952	81858	25321
3032	SLC45A1	-1.063	0.014	solute carrier family 45 member 1 [Source:HGNC Symbol;Acc:HGNC:17939]	chr1:8377885-8404227	NM_001080397	XM_011541531, XM_0	NP_001073866	XP_011539832, XP_0115	protein_coding	ENSG0000016242	50651	17939
3033	TM4SF5	-1.023	0.014	transmembrane 4 L six family member 5 [Source:HGNC Symbol;Acc:HGNC:11857]	chr17:4675186-4686506	NM_003963	.	NP_003954	.	protein_coding	ENSG0000014248	9032	11857
3034	ZNF215	1.010	0.014	zinc finger protein 215 [Source:HGNC Symbol;Acc:HGNC:13007]	chr11:6947653-7005863	NM_013250	XM_011520360, XM_0	NP_037382	XP_006718374, XP_0115	protein_coding	ENSG0000014905	7762	13007
3035	TMCC1	0.825	0.014	transmembrane and coiled-coil domain family 1 [Source:HGNC Symbol;Acc:HGNC:2911	chr3:129366634-129612419	NM_001017395, NN	XM_017005937, XM_0	NP_001017395, NP_00	XP_006713605, XP_0067	protein_coding	ENSG0000017276	23023	29116
3036	ZNF148	1.003	0.014	zinc finger protein 148 [Source:HGNC Symbol;Acc:HGNC:12933]	chr3:124944404-125094198	NM_021964	.	NP_068799	.	protein_coding	ENSG0000016384	7707	12933
3037	ARFIP1	0.866	0.014	ADP ribosylation factor interacting protein 1 [Source:HGNC Symbol;Acc:HGNC:21496]	chr4:153701088-153833509	NM_001025593, NN	XM_011531870, XM_0	NP_001020764, NP_00	XP_011530170, XP_0115	protein_coding	ENSG0000016414	27236	21496
3038	STK25	-0.658	0.014	serine/threonine kinase 25 [Source:HGNC Symbol;Acc:HGNC:11404]	chr2:242295657-242448987	NM_001271977, NN	XM_011510495, XM_0	NP_001258906, NP_00	XP_011508796, XP_0115	protein_coding	ENSG0000011569	10494	11404
3039	ABCF3	-0.630	0.014	ATP binding cassette subfamily F member 3 [Source:HGNC Symbol;Acc:HGNC:72]	chr3:183903810-183911795	NM_018358	XM_011512959	NP_060828	XP_011511261	protein_coding	ENSG0000016120	55324	72
3040	MFSD6	0.697	0.014	major facilitator superfamily domain containing 6 [Source:HGNC Symbol;Acc:HGNC:247	chr2:191273080-191367041	NM_017694	XM_011511368, XM_0	NP_060164	XP_005246711, XP_0052	protein_coding	ENSG0000015169	54842	24711
3041	CIB2	-0.893	0.014	calcium and integrin binding family member 2 [Source:HGNC Symbol;Acc:HGNC:24579]	chr15:78370149-78423886	NM_001271888, NN	XM_011521161, XM_0	NP_001258817, NP_00	XP_006720437, XP_0115	protein_coding	ENSG0000013642	10518	24579
3042	NUP133	0.709	0.014	nucleoporin 133 [Source:HGNC Symbol;Acc:HGNC:18016]	chr1:229576006-229644088	NM_018230	.	NP_060700	.	protein_coding	ENSG0000006924	55746	18016
3043	TMEM187	-0.783	0.014	transmembrane protein 187 [Source:HGNC Symbol;Acc:HGNC:13705]	chrX:153237777-153248646	NM_003492	XM_011531199	NP_003483	XP_011529501	protein_coding	ENSG0000017785	8269	13705
3044	NAXE	-0.597	0.014	NAD(P)HX epimerase [Source:HGNC Symbol;Acc:HGNC:18453]	chr1:156561553-156564091	NM_144772	.	NP_658985	.	protein_coding	ENSG0000016338	128240	18453
3045	STK4	0.642	0.014	serine/threonine kinase 4 [Source:HGNC Symbol;Acc:HGNC:11408]	chr20:43595114-43708600	NM_006282	XM_005260533, XM_0	NP_006273	XP_005260587, XP_0052	protein_coding	ENSG0000010110	6789	11408
3046	GLYCTK	-0.887	0.014	glycerate kinase [Source:HGNC Symbol;Acc:HGNC:24247]	chr3:52321104-52327492	NM_001144951, NN	XM_005264878	NP_001138423, NP_66	XP_005264935	protein_coding	ENSG0000016823	132158	24247
3047	MCM6	0.574	0.014	minichromosome maintenance complex component 6 [Source:HGNC Symbol;Acc:HGNC	chr2:136597195-136633996	NM_005915	.	NP_005906	.	protein_coding	ENSG0000007600	4175	6949
3048	NFKBIL1	-0.768	0.014	NFKB inhibitor like 1 [Source:HGNC Symbol;Acc:HGNC:7800]	chr6:31515357-31526606	NM_001144961, NN	.	NP_001138433, NP_00	.	protein_coding	ENSG0000020449	4795	7800
3049	PSMD2	-0.568	0.014	proteasome 26S subunit, non-ATPase 2 [Source:HGNC Symbol;Acc:HGNC:9559]	chr3:184016496-184026842	NM_001278708, NN	.	NP_001265637, NP_00	.	protein_coding	ENSG0000017516	5708	9559
3050	SYMPK	-0.589	0.014	symplekin [Source:HGNC Symbol;Acc:HGNC:22935]	chr19:46318692-46366548	NM_004819	XM_011527354, XM_0	NP_004810	XP_005259343, XP_0115	protein_coding	ENSG0000012575	8189	22935
3051	CD2AP	0.835	0.014	CD2 associated protein [Source:HGNC Symbol;Acc:HGNC:14258]	chr6:47445524-47594999	NM_012120	XM_011514449, XM_0	NP_036252	XP_005249033, XP_0115	protein_coding	ENSG0000019808	23607	14258
3052	PSMA3	0.666	0.014	proteasome subunit alpha 3 [Source:HGNC Symbol;Acc:HGNC:9532]	chr14:58711548-58738730	NM_002788, NM_1	.	NP_002779, NP_68703	.	protein_coding	ENSG0000010056	5684	9532
3053	RACGAP1	0.597	0.014	Rac GTPase activating protein 1 [Source:HGNC Symbol;Acc:HGNC:9804]	chr12:50361021-50419277	NM_001126103, NN	XM_017019220, XM_0	NP_001119575, NP_00	XP_005268869, XP_0052	protein_coding	ENSG0000016180	29127	9804
3054	ZC3H7A	0.762	0.014	zinc finger CCCH-type containing 7A [Source:HGNC Symbol;Acc:HGNC:30959]	chr16:11844441-11891123	NM_014153	XM_017023174, XM_0	NP_054872	XP_006720940, XP_0115	protein_coding	ENSG0000012229	29066	30959
3055	HLCS	0.704	0.014	holocarboxylase synthetase [Source:HGNC Symbol;Acc:HGNC:4976]	chr21:38123188-38362536	NM_000411, NM_0	XM_011529541, XM_0	NP_000402, NP_00122	XP_005261010, XP_0052	protein_coding	ENSG0000015926	3141	4976
3056	TPPP3	-1.041	0.014	tubulin polymerization promoting protein family member 3 [Source:HGNC Symbol;Acc:H	chr16:67423711-67427438	NM_015964, NM_0	.	NP_057048, NP_05722	.	protein_coding	ENSG0000015971	51673	24162
3057	ADPRHL2	-0.660	0.014	ADP-ribosylhydrolase like 2 [Source:HGNC Symbol;Acc:HGNC:21304]	chr1:36554475-36559533	NM_017825	.	NP_060295	.	protein_coding	ENSG0000011686	54936	21304
3058	CERCAM	-0.727	0.014	cerebral endothelial cell adhesion molecule [Source:HGNC Symbol;Acc:HGNC:23723]	chr9:131174044-131199630	NM_001286760, NN	XM_011518763, XM_0	NP_001273689, NP_05	XP_005252092, XP_0052	protein_coding	ENSG0000016712	51148	23723
3059	KLF12	0.955	0.014	Kruppel like factor 12 [Source:HGNC Symbol;Acc:HGNC:6346]	chr13:74260225-74569186	NM_007249	XM_017020385, XM_0	NP_009180	XP_005266308, XP_0052	protein_coding	ENSG0000011892	11278	6346
3060	MYRF	-0.672	0.014	myelin regulatory factor [Source:HGNC Symbol;Acc:HGNC:1181]	chr11:61520120-61555990	NM_001127392, NN	XM_011545234, XM_0	NP_001120864, NP_03	XP_005274279, XP_0052	protein_coding	ENSG0000012492	745	1181
3061	TRPC1	0.932	0.014	transient receptor potential cation channel subfamily C member 1 [Source:HGNC Symbo	chr3:142442915-142526730	NM_001251845, NN	XM_005247739, XM_0	NP_001238774, NP_00	XP_005247795, XP_0052	protein_coding	ENSG0000014493	7220	12333
3062	ACTR3C	1.148	0.014	ARP3 actin-related protein 3 homolog C [Source:HGNC Symbol;Acc:HGNC:37282]	chr7:149941007-150000069	NM_001164458, NN	XM_011516514, XM_0	NP_001157930, NP_00	XP_005250100, XP_0115	protein_coding	ENSG0000010652	653857	37282
3063	C16orf72	0.796	0.014	chromosome 16 open reading frame 72 [Source:HGNC Symbol;Acc:HGNC:30103]	chr16:9185504-9215497	NM_014117	.	NP_054836	.	protein_coding	ENSG0000018283	29035	30103
3064	HYAL1	-0.828	0.014	hyaluronoglucosaminidase 1 [Source:HGNC Symbol;Acc:HGNC:5320]	chr3:50337319-50349812	NM_033159, NM_1	XM_011533669, XM_0	NP_149349, NP_69501	XP_011531969, XP_0115	protein_coding	ENSG0000011437	3373	5320
3065	PHF8	-0.710	0.014	PHD finger protein 8 [Source:HGNC Symbol;Acc:HGNC:20672]	chrX:53963108-54071682	NM_001184896, NN	XM_011530778, XM_0	NP_001171825, NP_00	XP_005262053, XP_0052	protein_coding	ENSG0000017294	23133	20672
3066	C4orf46	0.843	0.014	chromosome 4 open reading frame 46 [Source:HGNC Symbol;Acc:HGNC:27320]	chr4:159587830-159593198	NM_001008393	.	NP_001008394	.	protein_coding	ENSG0000020520	201725	27320
3067	OTUD1	-0.914	0.014	OTU deubiquitinase 1 [Source:HGNC Symbol;Acc:HGNC:27346]	chr10:23728386-23731319	NM_001145373	.	NP_001138845	.	protein_coding	ENSG0000016531	220213	27346
3068	RFX8	1.241	0.014	RFX family member 8, lacking RFX DNA binding domain [Source:HGNC Symbol;Acc:HGN	chr2:102013865-102091165	NM_001145664	XM_017004852, XM_0	NP_001139136	XP_011510073, XP_0115	protein_coding	ENSG0000019646	731220	37253
3069	ATP1A3	-0.777	0.014	ATPase Na+/K+ transporting subunit alpha 3 [Source:HGNC Symbol;Acc:HGNC:801]	chr19:42470733-42498428	NM_001256213, NN	XM_011526991	NP_001243142, NP_00	XP_011525293	protein_coding	ENSG0000010540	478	801
3070	COLGALT1	-0.597	0.014	collagen beta(1-O)galactosyltransferase 1 [Source:HGNC Symbol;Acc:HGNC:26182]	chr19:17666402-17693968	NM_024656	XM_011528298, XM_0	NP_078932	XP_005260137, XP_0115	protein_coding	ENSG0000013030	79709	26182
3071	MGLL	-0.737	0.014	monoglyceride lipase [Source:HGNC Symbol;Acc:HGNC:17038]	chr3:127407904-127542093	NM_001003794, NN	XM_011512383, XM_0	NP_001003794, NP_00	XP_011510679, XP_0115	protein_coding	ENSG0000007441	11343	17038
3072	PMS2	0.783	0.014	PMS1 homolog 2, mismatch repair system component [Source:HGNC Symbol;Acc:HGNC	chr7:6012869-6048756	NM_000535	XM_011515430, XM_0	NP_000526, NP_00130	XP_011513731, XP_0115	protein_coding	ENSG0000012251	107984056	9122
3073	TTF1	0.809	0.014	transcription termination factor 1 [Source:HGNC Symbol;Acc:HGNC:12397]	chr9:135250934-135282238	NM_001205296, NN	XM_006717273	NP_001192225, NP_03	XP_006717336, XP_0168	protein_coding	ENSG0000012548	7270	12397
3074	PLOD1	-0.602	0.014	procollagen-lysine,2-oxoglutarate 5-dioxygenase 1 [Source:HGNC Symbol;Acc:HGNC:90	chr1:11994809-12035594	NM_000302	XM_011541594, XM_0	NP_000293, NP_00130	.	protein_coding	ENSG0000008344	5351	9081
3075	ZNF444	-0.733	0.014	zinc finger protein 444 [Source:HGNC Symbol;Acc:HGNC:16052]	chr19:56652555-56672262	NM_001253792, NN	XM_011527068, XM_0	NP_001240721, NP_06	XP_005259091, XP_0052	protein_coding	ENSG0000016768	55311	16052
3076	RAB37	-0.891	0.014	RAB37, member RAS oncogene family [Source:HGNC Symbol;Acc:HGNC:30268]	chr17:72666716-72743474	NM_001006638, NN	XM_005257289, XM_0	NP_001006639, NP_00	XP_005257345	protein_coding	ENSG0000017279	326624	30268

3077	TRAPPC6B	0.829	0.014	trafficking protein particle complex 6B [Source:HGNC Symbol;Acc:HGNC:23066]	chr14:39617015-39639597	NM_001079537, NN	XM_011536409	NP_001073005, NP_80	XP_011534711, XP_0168	protein_coding	ENSG0000018240	122553		23066
3078	E2F7	0.875	0.014	E2F transcription factor 7 [Source:HGNC Symbol;Acc:HGNC:23820]	chr12:77415026-77459360	NM_203394	XM_011537968, XM_0	NP_976328	XP_011536268	protein_coding	ENSG0000016589	144455		23820
3079	LEPROT	0.806	0.014	leptin receptor overlapping transcript [Source:HGNC Symbol;Acc:HGNC:29477]	chr1:65886269-66107242	NM_001198681, NN	.	NP_001185610, NP_05	.	protein_coding	ENSG0000021362	54741		29477
3080	SWAP70	0.720	0.014	SWAP switching B-cell complex 70kDa subunit [Source:HGNC Symbol;Acc:HGNC:17070]	chr11:9685623-9774538	NM_001297714, NN	.	NP_001284643, NP_05	.	protein_coding	ENSG0000013378	23075		17070
3081	TMEM150A	-0.874	0.014	transmembrane protein 150A [Source:HGNC Symbol;Acc:HGNC:24677]	chr2:85825670-85829821	NM_001031738	XM_011532508, XM_0	NP_001026908	XP_006711993, XP_0067	protein_coding	ENSG0000016889	129303		24677
3082	GSTCD	1.129	0.014	glutathione S-transferase C-terminal domain containing [Source:HGNC Symbol;Acc:HGN	chr4:106603783-106768885	NM_001031720, NN	XM_011532252, XM_0	NP_001026890, NP_07	XP_005263279, XP_0115	protein_coding	ENSG0000013878	79807		25806
3083	NELL2	0.584	0.014	neural EGFL like 2 [Source:HGNC Symbol;Acc:HGNC:7751]	chr12:44902057-45307711	NM_001145107, NN	XM_017019342, XM_0	NP_001138579, NP_00	XP_005268962, XP_0115	protein_coding	ENSG0000018461	4753		7751
3084	SNRPB	-0.575	0.014	small nuclear ribonucleoprotein polypeptides B and B1 [Source:HGNC Symbol;Acc:HGN(	chr20:2442279-2451499	NM_003091, NM_1	.	NP_003082, NP_93785	.	protein_coding	ENSG0000012583	6628		11153
3085	CD63	-0.589	0.015	CD63 molecule [Source:HGNC Symbol;Acc:HGNC:1692]	chr12:56119106-56123491	NM_001257389, NN	.	NP_001244318, NP_00	.	protein_coding	ENSG0000013540	967		1692
3086	NECAB3	-0.799	0.015	N-terminal EF-hand calcium binding protein 3 [Source:HGNC Symbol;Acc:HGNC:15851]	chr20:32244892-32262269	NM_031231, NM_0	XM_011528988, XM_0	NP_112508, NP_11250	XP_005260567, XP_0115	protein_coding	ENSG0000012596	63941		15851
3087	PNKP	-0.696	0.015	polynucleotide kinase 3'-phosphatase [Source:HGNC Symbol;Acc:HGNC:9154]	chr19:50364460-50371143	NM_007254	.	NP_009185	.	protein_coding	ENSG0000003965	11284		9154
3088	CPNE3	0.714	0.015	copine 3 [Source:HGNC Symbol;Acc:HGNC:2316]	chr8:87526655-87573726	NM_003909	XM_017013945, XM_0	NP_003900	XP_005251150, XP_0168	protein_coding	ENSG0000008571	8895		2316
3089	POLB	0.731	0.015	DNA polymerase beta [Source:HGNC Symbol;Acc:HGNC:9174]	chr8:42195972-42229326	NM_002690	XM_005273539, XM_0	NP_002681	XP_005273592, XP_0052	protein_coding	ENSG0000007050	5423		9174
3090	THADA	0.783	0.015	THADA, armadillo repeat containing [Source:HGNC Symbol;Acc:HGNC:19217]	chr2:43457974-43823185	NM_001083953, NN	XM_006712069, XM_0	NP_001077422, NP_00	XP_006712124, XP_0067	protein_coding	ENSG0000011597	63892		19217
3091	KCNQ2	-0.881	0.015	potassium voltage-gated channel subfamily Q member 2 [Source:HGNC Symbol;Acc:HGI	chr20:62031562-62104030	NM_004518, NM_1	XM_017027844, XM_0	NP_004509, NP_74210	XP_011527112, XP_0115	protein_coding	ENSG0000007504	3785		6296
3092	LBR	0.653	0.015	lamin B receptor [Source:HGNC Symbol;Acc:HGNC:6518]	chr1:225589203-225616557	NM_002296, NM_1	XM_011544186, XM_0	NP_002287, NP_91942	XP_005273182, XP_0115	protein_coding	ENSG0000014381	3930		6518
3093	NCOA2	0.941	0.015	nuclear receptor coactivator 2 [Source:HGNC Symbol;Acc:HGNC:7669]	chr8:71021996-71316040	NM_001321703, NN	XM_011517440, XM_0	NP_001308632, NP_00	XP_005251185, XP_0052	protein_coding	ENSG0000014039	10499		7669
3094	HACE1	0.948	0.015	HECT domain and ankyrin repeat containing E3 ubiquitin protein ligase 1 [Source:HGNC	chr6:105175967-105307794	NM_001321083, NN	XM_011535989, XM_0	NP_001308012, NP_06	.	protein_coding	ENSG0000008538	57531		21033
3095	SEC24D	0.825	0.015	SEC24 homolog D, COPII coat complex component [Source:HGNC Symbol;Acc:HGNC:107	chr4:119643977-119757326	NM_001318066, NN	XM_011532437, XM_0	NP_001304995, NP_05	XP_005263436	protein_coding	ENSG0000015096	9871		10706
3096	TBC1D30	0.884	0.015	TBC1 domain family member 30 [Source:HGNC Symbol;Acc:HGNC:29164]	chr12:65179790-65274812	NM_015279	XM_017019087, XM_0	NP_056094	XP_011536380, XP_0168	protein_coding	ENSG0000011149	23329		29164
3097	VTI1A	0.789	0.015	vesicle transport through interaction with t-SNAREs 1A [Source:HGNC Symbol;Acc:HGN(	chr10:114207015-114578503	NM_001318203, NN	XM_011539330, XM_0	NP_001305132, NP_00	XP_005269601, XP_0052	protein_coding	ENSG0000015153	143187		17792
3098	ACBD6	-0.657	0.015	acyl-CoA binding domain containing 6 [Source:HGNC Symbol;Acc:HGNC:23339]	chr1:180257364-180472089	NM_032360	.	NP_115736	.	protein_coding	ENSG0000023012	100527964		23339
3099	CREBZF	0.717	0.015	CREB/ATF bZIP transcription factor [Source:HGNC Symbol;Acc:HGNC:24905]	chr11:85371295-85393951	NM_001039618	XM_011545195	NP_001034707	XP_006718705, XP_0115	protein_coding	ENSG0000013750	58487		24905
3100	PCNX4	1.502	0.015	pecanex homolog 4 (Drosophila) [Source:HGNC Symbol;Acc:HGNC:20349]	chr14:60558628-60636574	NM_022495	XM_005268009	NP_071940	XP_005268066	protein_coding	ENSG0000012677	64430		20349
3101	RB1CC1	0.803	0.015	RB1 inducible coiled-coil 1 [Source:HGNC Symbol;Acc:HGNC:15574]	chr8:53535015-53626992	NM_001083617, NN	XM_017014109, XM_0	NP_001077086, NP_05	XP_011515945, XP_0115	protein_coding	ENSG0000002328	9821		15574
3102	CNNM3	-0.693	0.015	cyclin and CBS domain divalent metal cation transport mediator 3 [Source:HGNC Symbo	chr2:97481981-97499648	NM_017623, NM_1	.	NP_060093, NP_95106	XP_011509259, XP_0168	protein_coding	ENSG0000016876	26505		104
3103	FAM234B	0.758	0.015	family with sequence similarity 234 member B [Source:HGNC Symbol;Acc:HGNC:29288]	chr12:13197217-13236383	NM_020853	.	NP_065904	XP_016875195	protein_coding	ENSG0000008444	57613		29288
3104	ZNF816	1.498	0.015	zinc finger protein 816 [Source:HGNC Symbol;Acc:HGNC:26995]	chr19:53379424-53466135	NM_001031665, NN	.	NP_001026835, NP_00	.	protein_coding	ENSG0000018025	125893		26995
3105	GK5	0.854	0.015	glycerol kinase 5 (putative) [Source:HGNC Symbol;Acc:HGNC:28635]	chr3:141876368-141944449	NM_001039547	XM_017006090, XM_0	NP_001034636	XP_011510926, XP_0168	protein_coding	ENSG0000017506	256356		28635
3106	MIER1	0.911	0.015	MIER1 transcriptional regulator [Source:HGNC Symbol;Acc:HGNC:29657]	chr1:67390577-67454302	NM_001077700, NN	XM_017001934, XM_0	NP_001071168, NP_00	XP_005271133, XP_0052	protein_coding	ENSG0000019816	57708		29657
3107	SIVA1	-0.583	0.015	SIVA1 apoptosis inducing factor [Source:HGNC Symbol;Acc:HGNC:17712]	chr14:105219436-105225996	NM_006427, NM_0	.	NP_006418, NP_06835	.	protein_coding	ENSG0000018499	10572		17712
3108	TMTC2	1.011	0.015	transmembrane and tetratricopeptide repeat containing 2 [Source:HGNC Symbol;Acc:H(	chr12:83080658-83528631	NM_001320321, NN	XM_011537979, XM_0	NP_001307250, NP_00	.	protein_coding	ENSG0000017910	160335		25440
3109	CDKN2AIP	0.783	0.015	CDKN2A interacting protein [Source:HGNC Symbol;Acc:HGNC:24325]	chr4:184365743-184369351	NM_017632	XM_005263118	NP_001304272, NP_06	.	protein_coding	ENSG0000016856	55602		24325
3110	DDX10	0.683	0.015	DEAD-box helicase 10 [Source:HGNC Symbol;Acc:HGNC:2735]	chr11:108535751-108811657	NM_004398	.	NP_004389	.	protein_coding	ENSG0000017810	1662		2735
3111	NNT	0.742	0.015	nicotinamide nucleotide transhydrogenase [Source:HGNC Symbol;Acc:HGNC:7863]	chr5:43602824-43707507	NM_012343, NM_1	XM_011514002, XM_0	NP_036475, NP_89202	XP_005248331, XP_0067	protein_coding	ENSG0000011299	23530		7863
3112	ARAP1	-0.729	0.015	ArfGAP with RhoGAP domain, ankyrin repeat and PH domain 1 [Source:HGNC Symbol;A(	chr11:72396113-72504726	NM_001040118, NN	.	NP_001035207, NP_00	.	protein_coding	ENSG0000018663	116985		16925
3113	CHCHD3	0.594	0.015	coiled-coil-helix-coiled-coil-helix domain containing 3 [Source:HGNC Symbol;Acc:HGNC:	chr7:132469628-132766842	NM_001317177, NN	XM_011516363	NP_001304106, NP_06	.	protein_coding	ENSG0000010655	54927		21906
3114	ELP3	0.720	0.015	elongator acetyltransferase complex subunit 3 [Source:HGNC Symbol;Acc:HGNC:20696]	chr8:27947747-28048673	NM_001284220, NN	XM_006716354	NP_001271149, NP_00	XP_006716417, XP_0168	protein_coding	ENSG0000013401	55140		20696
3115	FAM78A	-0.927	0.015	family with sequence similarity 78 member A [Source:HGNC Symbol;Acc:HGNC:25465]	chr9:134133462-134151934	NM_033387	.	NP_203745	.	protein_coding	ENSG0000012688	286336		25465
3116	IFNAR1	0.713	0.015	interferon alpha and beta receptor subunit 1 [Source:HGNC Symbol;Acc:HGNC:5432]	chr21:34697208-34732168	NM_000629	XM_005260964	NP_000620	XP_005261021	protein_coding	ENSG0000014216	3454		5432
3117	STRN3	1.312	0.015	striatin 3 [Source:HGNC Symbol;Acc:HGNC:15720]	chr14:31343748-31565656	NM_001083893, NN	XM_011536681, XM_0	NP_001077362, NP_05	XP_005267626, XP_0052	protein_coding	ENSG0000019679	29966		15720
3118	TCF25	-0.618	0.015	transcription factor 25 [Source:HGNC Symbol;Acc:HGNC:29181]	chr16:89939993-89977792	NM_014972	XM_011522961, XM_0	NP_055787	XP_005256354, XP_0052	protein_coding	ENSG0000014100	22980		29181
3119	TSSC4	-0.651	0.015	tumor suppressing subtransferable candidate 4 [Source:HGNC Symbol;Acc:HGNC:12386	chr11:2421717-2425108	NM_001297658, NN	XM_011519830, XM_0	NP_001284587, NP_00	XP_006718181, XP_0115	protein_coding	ENSG0000018428	10078		12386
3120	CXCL3	-0.929	0.015	C-X-C motif chemokine ligand 3 [Source:HGNC Symbol;Acc:HGNC:4604]	chr4:74902305-74904406	NM_002090	.	NP_002081	.	protein_coding	ENSG0000016373	2921		4604
3121	C1R	-1.156	0.015	complement C1r subcomponent [Source:HGNC Symbol;Acc:HGNC:1246]	chr12:7187835-7245131	NM_001733	XM_017019915	NP_001724	XP_016875404	protein_coding	ENSG0000015940	715		1246
3122	SLC25A30	0.820	0.015	solute carrier family 25 member 30 [Source:HGNC Symbol;Acc:HGNC:27371]	chr13:45967450-45992590	NM_001010875, NN	XM_017020524, XM_0	NP_001010875, NP_00	XP_011533329, XP_0168	protein_coding	ENSG0000017403	253512		27371
3123	FAM8A1	0.797	0.015	family with sequence similarity 8 member A1 [Source:HGNC Symbol;Acc:HGNC:16372]	chr6:17600585-17611950	NM_016255	.	NP_057339	.	protein_coding	ENSG0000013741	51439		16372
3124	SLC39A9	0.733	0.015	solute carrier family 39 member 9 [Source:HGNC Symbol;Acc:HGNC:20182]	chr14:69865406-69929105	NM_001252148, NN	XM_005267834	NP_001239077, NP_00	XP_005267891	protein_coding	ENSG0000002936	55334		20182
3125	ELP4	0.723	0.015	elongator acetyltransferase complex subunit 4 [Source:HGNC Symbol;Acc:HGNC:1171]	chr11:31531296-31805546	NM_001288725, NN	XM_005252865	NP_001275654, NP_00	.	protein_coding	ENSG0000010991	26610		1171
3126	RNF128	0.878	0.015	ring finger protein 128, E3 ubiquitin protein ligase [Source:HGNC Symbol;Acc:HGNC:211	chrX:105937067-106040223	NM_024539, NM_1	.	NP_078815, NP_91944	.	protein_coding	ENSG0000013313	79589		21153
3127	RNPC3	1.248	0.015	RNA binding region (RNP1, RRM) containing 3 [Source:HGNC Symbol;Acc:HGNC:18666]	chr1:104068312-104122152	NM_017619	.	NP_060089	.	protein_coding	ENSG0000018594	55599		18666
3128	SYT12	-1.013	0.015	synaptotagmin 12 [Source:HGNC Symbol;Acc:HGNC:18381]	chr11:66774248-66818334	NM_001177880, NN	XM_011545347, XM_0	NP_001171351, NP_80	XP_006718800, XP_0115	protein_coding	ENSG0000017322	91683		18381
3129	EXOC6	0.839	0.015	exocyst complex component 6 [Source:HGNC Symbol;Acc:HGNC:23196]	chr10:94594469-94819250	NM_001013848, NN	XM_017016346, XM_0	NP_001013870, NP_00	XP_016871833, XP_0168	protein_coding	ENSG0000013819	54536		23196
3130	TUFM	-0.554	0.015	Tu translation elongation factor, mitochondrial [Source:HGNC Symbol;Acc:HGNC:12420]	chr16:28853731-28857729	NM_003321	XM_011545928	NP_003312	XP_011544230, XP_0168	protein_coding	ENSG0000017895	100616256		12420

3131	ADRA2C	-0.608	0.015	adrenoceptor alpha 2C [Source:HGNC Symbol;Acc:HGNC:283]	chr4:3768074-3770253	NM_000683	.	NP_000674	.	protein_coding	ENSG0000018416	152	283
3132	AKR7A2	-0.620	0.015	aldo-keto reductase family 7 member A2 [Source:HGNC Symbol;Acc:HGNC:389]	chr1:19630458-19638640	NM_003689	.	NP_001307908, NP_00	.	protein_coding	ENSG0000005337	8574	389
3133	ARL8A	-0.729	0.015	ADP ribosylation factor like GTPase 8A [Source:HGNC Symbol;Acc:HGNC:25192]	chr1:202091985-202113871	NM_001256129, NM	.	NP_001243058, NP_62	.	protein_coding	ENSG0000014386	127829	25192
3134	MFSD10	-0.616	0.015	major facilitator superfamily domain containing 10 [Source:HGNC Symbol;Acc:HGNC:16	chr4:2932287-2936586	NM_001120, NM_0	XM_005247928, XM_0	NP_001111, NP_00113	XP_005247984, XP_0052	protein_coding	ENSG0000010973	10227	16894
3135	LIN52	0.738	0.016	lin-52 DREAM MuvB core complex component [Source:HGNC Symbol;Acc:HGNC:19856]	chr14:74551498-74667938	NM_001024674	XM_017021763	NP_001019845	XP_016877252	protein_coding	ENSG0000020565	91750	19856
3136	LMBR1	0.671	0.016	limb development membrane protein 1 [Source:HGNC Symbol;Acc:HGNC:13243]	chr7:156470484-156685924	NM_022458	XM_011516462, XM_0	NP_071903	XP_005249612, XP_0168	protein_coding	ENSG0000010598	64327	13243
3137	TCEA1	0.686	0.016	transcription elongation factor A1 [Source:HGNC Symbol;Acc:HGNC:11612]	chr8:54879111-54935089	NM_006756, NM_2	XM_011517579, XM_0	NP_006747, NP_95884	XP_006716530, XP_0115	protein_coding	ENSG0000018773	6917	11612
3138	ZMAT5	-0.803	0.016	zinc finger matrin-type 5 [Source:HGNC Symbol;Acc:HGNC:28046]	chr22:30116072-30162950	NM_001003692, NM	XM_005261683	NP_001003692, NP_00	XP_005261740	protein_coding	ENSG0000010031	55954	28046
3139	NPRL2	-1.065	0.016	NPR2-like, GATOR1 complex subunit [Source:HGNC Symbol;Acc:HGNC:24969]	chr3:50384918-50541675	NM_006545	XM_011533288, XM_0	NP_006536	XP_005264865, XP_0115	protein_coding	ENSG0000011438	10641	24969
3140	FXYD1	-2.235	0.016	FXYD domain containing ion transport regulator 1 [Source:HGNC Symbol;Acc:HGNC:402	chr19:35629711-35634013	NM_001278717, NM	.	NP_001265646, NP_00	XP_016882363	protein_coding	ENSG0000026696	5348	4025
3141	ARHGAP26	0.681	0.016	Rho GTPase activating protein 26 [Source:HGNC Symbol;Acc:HGNC:17073]	chr5:142149948-142608576	NM_001135608, NM	XM_005268402, XM_0	NP_001129080, NP_05	XP_005268455, XP_0052	protein_coding	ENSG0000014581	23092	17073
3142	BORCS6	-0.751	0.016	BLOC-1 related complex subunit 6 [Source:HGNC Symbol;Acc:HGNC:25939]	chr17:8091650-8094225	NM_017622	.	NP_060092	.	protein_coding	ENSG0000019654	54785	25939
3143	GAS2L1	-0.791	0.016	growth arrest specific 2 like 1 [Source:HGNC Symbol;Acc:HGNC:16955]	chr22:29702571-29708774	NM_001278730, NM	XM_017028533, XM_0	NP_001265659, NP_00	XP_011528126, XP_0115	protein_coding	ENSG0000018534	10634	16955
3144	MRPS6	-0.873	0.016	mitochondrial ribosomal protein S6 [Source:HGNC Symbol;Acc:HGNC:14051]	chr21:35445822-35515334	NM_032476	.	NP_115865	.	protein_coding	ENSG0000024392	64968	14051
3145	ST3GAL6	1.365	0.016	ST3 beta-galactoside alpha-2,3-sialyltransferase 6 [Source:HGNC Symbol;Acc:HGNC:180	chr3:98451079-98514689	NM_001271142, NM	XM_006713476, XM_0	NP_001258071, NP_00	XP_005247126, XP_0052	protein_coding	ENSG0000006422	10402	18080
3146	ZCCHC7	0.734	0.016	zinc finger CCHC-type containing 7 [Source:HGNC Symbol;Acc:HGNC:26209]	chr9:37120535-37358146	NM_001289119, NM	XM_011518050, XM_0	NP_001276048, NP_00	XP_005251665, XP_0052	protein_coding	ENSG0000014790	84186	26209
3147	DDX39A	-0.599	0.016	DEAD-box helicase 39A [Source:HGNC Symbol;Acc:HGNC:17821]	chr19:14519662-14530195	NM_005804	XM_011527620	NP_005795	XP_011525922	protein_coding	ENSG0000012313	10212	17821
3148	MMGT1	0.861	0.016	membrane magnesium transporter 1 [Source:HGNC Symbol;Acc:HGNC:28100]	chrX:135044228-135129428	NM_173470	.	NP_775741	.	protein_coding	ENSG0000016944	93380	28100
3149	NAA15	1.431	0.016	N(alpha)-acetyltransferase 15, NatA auxiliary subunit [Source:HGNC Symbol;Acc:HGNC:3	chr4:140211070-140341187	NM_057175	XM_005263236	NP_476516	XP_005263293	protein_coding	ENSG0000016413	80155	30782
3150	POLR3B	0.945	0.016	RNA polymerase III subunit B [Source:HGNC Symbol;Acc:HGNC:30348]	chr12:106751435-106903976	NM_001160708, NM	.	NP_001154180, NP_06	.	protein_coding	ENSG0000001350	55703	30348
3151	LONRF3	0.875	0.016	LON peptidase N-terminal domain and ring finger 3 [Source:HGNC Symbol;Acc:HGNC:21	chrX:118108576-118152318	NM_001031855, NM	XM_005262476	NP_001027026, NP_00	XP_005262533	protein_coding	ENSG0000017555	79836	21152
3152	MARK4	-0.741	0.016	microtubule affinity regulating kinase 4 [Source:HGNC Symbol;Acc:HGNC:13538]	chr19:45754515-45808541	NM_001199867, NM	XM_006723307	NP_001186796, NP_11	XP_006723370	protein_coding	ENSG0000000704	57787	13538
3153	SCN8A	0.898	0.016	sodium voltage-gated channel alpha subunit 8 [Source:HGNC Symbol;Acc:HGNC:10596]	chr12:51984709-52206648	NM_001177984, NM	XM_017019795, XM_0	NP_001171455, NP_05	XP_006719619, XP_0115	protein_coding	ENSG0000019687	6334	10596
3154	AGPAT2	-0.590	0.016	1-acylglycerol-3-phosphate O-acyltransferase 2 [Source:HGNC Symbol;Acc:HGNC:325]	chr9:139567594-139581875	NM_001012727, NM	.	NP_001012745, NP_00	.	protein_coding	ENSG0000016969	10555	325
3155	NRG2	-0.842	0.016	neuregulin 2 [Source:HGNC Symbol;Acc:HGNC:7998]	chr5:139226363-139422884	NM_001184935, NM	XM_011537711, XM_0	NP_001171864, NP_00	XP_006714871, XP_0067	protein_coding	ENSG0000015845	9542	7998
3156	AMFR	-0.605	0.016	autocrine motility factor receptor [Source:HGNC Symbol;Acc:HGNC:463]	chr16:56395363-56459450	NM_001144	XM_005255890, XM_0	NP_001135	XP_005255947	protein_coding	ENSG0000015946	267	463
3157	CELF5	-1.413	0.016	CUGBP, Elav-like family member 5 [Source:HGNC Symbol;Acc:HGNC:14058]	chr19:3224700-3297074	NM_001172673, NM	XM_011528175, XM_0	NP_001166144, NP_06	XP_006722895, XP_0067	protein_coding	ENSG0000016108	60680	14058
3158	GRM8	1.165	0.016	glutamate metabotropic receptor 8 [Source:HGNC Symbol;Acc:HGNC:4600]	chr7:126078651-126893348	NM_000845, NM_0	XM_011516102, XM_0	NP_000836, NP_00112	XP_006716001, XP_0115	protein_coding	ENSG0000017960	2918	4600
3159	IGFBP4	-0.577	0.016	insulin like growth factor binding protein 4 [Source:HGNC Symbol;Acc:HGNC:5473]	chr17:38599712-38613983	NM_001552	.	NP_001543	.	protein_coding	ENSG0000014175	3487	5473
3160	OCEL1	-0.777	0.016	occludin/ELL domain containing 1 [Source:HGNC Symbol;Acc:HGNC:26221]	chr19:17337012-17340028	NM_024578	XM_017027306, XM_0	NP_078854	XP_005260136, XP_0067	protein_coding	ENSG0000009933	79629	26221
3161	PSAP	-0.567	0.016	prosaposin [Source:HGNC Symbol;Acc:HGNC:9498]	chr10:73576054-73611132	NM_001042465, NM	.	NP_001035930, NP_00	.	protein_coding	ENSG0000019774	5660	9498
3162	SH3D19	0.787	0.016	SH3 domain containing 19 [Source:HGNC Symbol;Acc:HGNC:30418]	chr4:152041430-152246784	NM_001009555, NM	XM_017007783, XM_0	NP_001009555, NP_00	XP_005262824, XP_0115	protein_coding	ENSG0000010968	152503	30418
3163	ZBTB14	0.845	0.016	zinc finger and BTB domain containing 14 [Source:HGNC Symbol;Acc:HGNC:12860]	chr18:5289017-5297052	NM_001143823, NM	.	NP_001137295, NP_00	.	protein_coding	ENSG0000019808	7541	12860
3164	CD99L2	-0.715	0.016	CD99 molecule like 2 [Source:HGNC Symbol;Acc:HGNC:18237]	chrX:149934809-150067289	NM_001184808, NM	XM_006724849, XM_0	NP_001171737, NP_00	XP_011529504	protein_coding	ENSG0000010218	83692	18237
3165	BTG3	0.667	0.016	BTG anti-proliferation factor 3 [Source:HGNC Symbol;Acc:HGNC:1132]	chr21:18965970-18985265	NM_001130914, NM	XM_011529441	NP_001124386, NP_00	XP_011527743, XP_0168	protein_coding	ENSG0000015464	10950	1132
3166	MCTS1	0.651	0.016	MCTS1, re-initiation and release factor [Source:HGNC Symbol;Acc:HGNC:23357]	chrX:119737834-119746334	NM_001137554, NM	.	NP_001131026, NP_05	.	protein_coding	ENSG0000023211	28985	23357
3167	AREL1	0.620	0.016	apoptosis resistant E3 ubiquitin protein ligase 1 [Source:HGNC Symbol;Acc:HGNC:20363	chr14:75127954-75179818	NM_001039479	XM_011537417, XM_0	NP_001034568	XP_005268301, XP_0067	protein_coding	ENSG0000011968	9870	20363
3168	PARP2	0.705	0.016	poly(ADP-ribose) polymerase 2 [Source:HGNC Symbol;Acc:HGNC:272]	chr14:20811740-20826064	NM_001042618, NM	XM_011536312, XM_0	NP_001036083, NP_00	XP_005267304	protein_coding	ENSG0000012948	10038	272
3169	STYX	0.851	0.016	serine/threonine/tyrosine interacting protein [Source:HGNC Symbol;Acc:HGNC:11447]	chr14:53196897-53241716	NM_001130701, NM	XM_011537108, XM_0	NP_001124173, NP_66	XP_006720305, XP_0115	protein_coding	ENSG0000019825	6815	11447
3170	HS3ST3B1	0.971	0.016	heparan sulfate-glucosamine 3-sulfotransferase 3B1 [Source:HGNC Symbol;Acc:HGNC:5	chr17:14204399-14252721	NM_006041	.	NP_006032	.	protein_coding	ENSG0000012543	9953	5198
3171	MTRNR2L1	0.565	0.016	MT-RNR2-like 1 [Source:HGNC Symbol;Acc:HGNC:37155]	chr17:22022436-22023989	NM_001190452	.	NP_001177381	.	protein_coding	ENSG0000025661	100462977	37155
3172	TOP1	0.597	0.016	topoisomerase (DNA) I [Source:HGNC Symbol;Acc:HGNC:11986]	chr20:39657457-39753127	NM_003286	.	NP_003277	.	protein_coding	ENSG0000019890	7150	11986
3173	STAMBPL1	0.984	0.016	STAM binding protein like 1 [Source:HGNC Symbol;Acc:HGNC:24105]	chr10:90639490-90734910	NM_020799	XM_011539985, XM_0	NP_065850	XP_006717991, XP_0067	protein_coding	ENSG0000013813	57559	24105
3174	UCHL1	-0.852	0.016	ubiquitin C-terminal hydrolase L1 [Source:HGNC Symbol;Acc:HGNC:12513]	chr4:41258785-41270446	NM_004181	.	NP_004172	.	protein_coding	ENSG0000015427	7345	12513
3175	ZNF570	1.170	0.016	zinc finger protein 570 [Source:HGNC Symbol;Acc:HGNC:26416]	chr19:37958673-37979554	NM_001300993, NM	XM_011526544, XM_0	NP_001287922, NP_00	XP_011524843, XP_0115	protein_coding	ENSG0000017182	148268	26416
3176	ABHD3	0.751	0.016	abhydrolase domain containing 3 [Source:HGNC Symbol;Acc:HGNC:18718]	chr18:19230857-19284766	NM_001308256, NM	XM_011525849, XM_0	NP_001295185, NP_00	XP_011524150, XP_0115	protein_coding	ENSG0000015820	171586	18718
3177	NAV2	0.693	0.016	neuron navigator 2 [Source:HGNC Symbol;Acc:HGNC:15997]	chr11:19372270-20143144	NM_001111018, NM	XM_017018524, XM_0	NP_001104488, NP_00	XP_005253271, XP_0067	protein_coding	ENSG0000016683	89797	15997
3178	FGFR4	-0.635	0.016	fibroblast growth factor receptor 4 [Source:HGNC Symbol;Acc:HGNC:3691]	chr5:176513886-176525145	NM_001291980, NM	XM_011534465, XM_0	NP_001278909, NP_00	XP_005265895, XP_0115	protein_coding	ENSG0000016086	2264	3691
3179	PNKD	-0.975	0.017	paroxysmal nonkinesigenic dyskinesia [Source:HGNC Symbol;Acc:HGNC:9153]	chr2:219135114-219211516	NM_001077399, NM	.	NP_001070867, NP_05	.	protein_coding	ENSG0000012783	25953	9153
3180	PSMB1	-0.554	0.017	proteasome subunit beta 1 [Source:HGNC Symbol;Acc:HGNC:9537]	chr6:170844204-170862429	NM_002793	.	NP_002784	.	protein_coding	ENSG0000000801	5689	9537
3181	SPTLC2	0.615	0.017	serine palmitoyltransferase long chain base subunit 2 [Source:HGNC Symbol;Acc:HGNC:	chr14:77972339-78083116	NM_004863	XM_011537384	NP_004854	XP_011535686	protein_coding	ENSG0000010059	9517	11278
3182	PARG	0.854	0.017	poly(ADP-ribose) glycohydrolase [Source:HGNC Symbol;Acc:HGNC:8605]	chr10:51026324-51130715	NM_001303486, NM	XM_011540306, XM_0	NP_001290415, NP_00	XP_011538607	protein_coding	ENSG0000022734	8505	8605
3183	PLA2G7	1.021	0.017	phospholipase A2 group VII [Source:HGNC Symbol;Acc:HGNC:9040]	chr6:46655611-46703430	NM_001168357, NM	XM_005249408	NP_001161829, NP_00	XP_005249465	protein_coding	ENSG0000014607	7941	9040
3184	ZNF736	1.001	0.017	zinc finger protein 736 [Source:HGNC Symbol;Acc:HGNC:32467]	chr7:63773185-63815238	NM_001170905	.	NP_001164376	.	protein_coding	ENSG0000023444	728927	32467

3185	LMBR1L	-0.735	0.017	limb development membrane protein 1 like [Source:HGNC Symbol;Acc:HGNC:18268]	chr12:49490333-49504681	NM_001300750, NN	XM_011538567, XM_C	NP_001287679, NP_00	XP_011536865, XP_0115	protein_coding	ENSG0000013963	55716	18268
3186	PPP1R1C	1.101	0.017	protein phosphatase 1 regulatory inhibitor subunit 1C [Source:HGNC Symbol;Acc:HGNC	chr2:182850594-182996125	NM_001080545, NN	.	NP_001074014, NP_00	.	protein_coding	ENSG0000015072	151242	14940
3187	DGCR2	-0.621	0.017	DiGeorge syndrome critical region gene 2 [Source:HGNC Symbol;Acc:HGNC:2845]	chr22:19023794-19109967	NM_001173533, NN	.	NP_001167004, NP_00	.	protein_coding	ENSG0000007041	9993	2845
3188	POLD2	-0.551	0.017	DNA polymerase delta 2, accessory subunit [Source:HGNC Symbol;Acc:HGNC:9176]	chr7:44154279-44163158	NM_001127218, NN	XM_011515431	NP_001120690, NP_00	XP_011513733	protein_coding	ENSG0000010662	5425	9176
3189	RAB36	-0.832	0.017	RAB36, member RAS oncogene family [Source:HGNC Symbol;Acc:HGNC:9775]	chr22:23487512-23506537	NM_004914	XM_011530547, XM_C	NP_004905	XP_005261916, XP_0067	protein_coding	ENSG0000010022	9609	9775
3190	THUMPD2	0.897	0.017	THUMP domain containing 2 [Source:HGNC Symbol;Acc:HGNC:14890]	chr2:39963444-40006407	NM_025264	XM_011533121, XM_C	NP_079540	XP_016860539	protein_coding	ENSG0000013805	80745	14890
3191	PFAS	-0.661	0.017	phosphoribosylformylglycinamidine synthase [Source:HGNC Symbol;Acc:HGNC:8863]	chr17:8152603-8173803	NM_012393	XM_011523938, XM_C	NP_036525	.	protein_coding	ENSG0000017892	5198	8863
3192	CDK5RAP2	0.663	0.017	CDK5 regulatory subunit associated protein 2 [Source:HGNC Symbol;Acc:HGNC:18672]	chr9:123151146-123342448	NM_001011649, NN	XM_011518861, XM_C	NP_001011649, NP_00	XP_006717245, XP_0067	protein_coding	ENSG0000013686	55755	18672
3193	PRMT7	-0.752	0.017	protein arginine methyltransferase 7 [Source:HGNC Symbol;Acc:HGNC:25557]	chr16:68344958-68482591	NM_001184824, NN	XM_011523119, XM_C	NP_001171753, NP_00	XP_011521414, XP_0115	protein_coding	ENSG0000013260	54496	25557
3194	RPH3AL	-0.729	0.017	rabphilin 3A-like (without C2 domains) [Source:HGNC Symbol;Acc:HGNC:10296]	chr17:62179-202888	NM_001190411, NN	XM_011535368, XM_C	NP_001177340, NP_00	XP_011533666, XP_0115	protein_coding	ENSG0000018103	9501	10296
3195	BCL10	0.820	0.017	B-cell CLL/lymphoma 10 [Source:HGNC Symbol;Acc:HGNC:989]	chr1:85731930-85742773	NM_003921	XM_005271311	NP_001307644, NP_00	XP_011540701	protein_coding	ENSG0000014286	8915	989
3196	CARHSP1	-0.895	0.017	calcium regulated heat stable protein 1 [Source:HGNC Symbol;Acc:HGNC:17150]	chr16:8946798-8962869	NM_001042476, NN	XM_011522444, XM_C	NP_001035941, NP_00	XP_005255285, XP_0052	protein_coding	ENSG0000015304	23589	17150
3197	FLT3LG	-0.878	0.017	fms related tyrosine kinase 3 ligand [Source:HGNC Symbol;Acc:HGNC:3766]	chr19:49977484-49989488	NM_001204502, NN	XM_017026535, XM_C	NP_001191431, NP_00	XP_005258737, XP_0052	protein_coding	ENSG0000009055	2323	3766
3198	GAMT	-0.700	0.017	guanidinoacetate N-methyltransferase [Source:HGNC Symbol;Acc:HGNC:4136]	chr19:1397082-1401569	NM_000156, NM_1	.	NP_000147, NP_62027	.	protein_coding	ENSG0000013000	2593	4136
3199	LCLAT1	0.759	0.017	lysocardiolipin acyltransferase 1 [Source:HGNC Symbol;Acc:HGNC:26756]	chr2:30670101-30867091	NM_001002257, NN	XM_011532744, XM_C	NP_001002257, NP_00	XP_005264301, XP_0052	protein_coding	ENSG0000017295	253558	26756
3200	WDR27	0.988	0.017	WD repeat domain 27 [Source:HGNC Symbol;Acc:HGNC:21248]	chr6:169857306-170101749	NM_001202550, NN	XM_011535692, XM_C	NP_001189479, NP_87	XP_011533984, XP_0115	protein_coding	ENSG0000018446	253769	21248
3201	AP1S2	-0.815	0.017	adaptor related protein complex 1 sigma 2 subunit [Source:HGNC Symbol;Acc:HGNC:56	chrX:15843928-15873137	NM_001272071, NN	XM_017029925, XM_C	NP_001259000, NP_00	XP_011543901, XP_0168	protein_coding	ENSG0000018228	8905	560
3202	DGCR6L	-0.659	0.017	DiGeorge syndrome critical region gene 6-like [Source:HGNC Symbol;Acc:HGNC:18551]	chr22:20301798-20307603	NM_033257	XM_011530470	NP_150282	.	protein_coding	ENSG0000012818	85359	18551
3203	SRP54	0.669	0.017	signal recognition particle 54 [Source:HGNC Symbol;Acc:HGNC:11301]	chr14:35451162-35498773	NM_001146282, NN	XM_011537107, XM_C	NP_001139754, NP_00	XP_005268081, XP_0115	protein_coding	ENSG0000010088	6729	11301
3204	SUSD3	-0.776	0.017	sushi domain containing 3 [Source:HGNC Symbol;Acc:HGNC:28391]	chr9:95820969-95847420	NM_001287005, NN	XM_011518361, XM_C	NP_001273934, NP_00	XP_011516660, XP_0115	protein_coding	ENSG0000015730	203328	28391
3205	WEE1	0.684	0.017	WEE1 G2 checkpoint kinase [Source:HGNC Symbol;Acc:HGNC:12761]	chr11:9595227-9611531	NM_001143976, NN	XM_005253119, XM_C	NP_001137448, NP_00	.	protein_coding	ENSG0000016648	7465	12761
3206	AIMP1	0.708	0.017	aminoacyl tRNA synthetase complex interacting multifunctional protein 1 [Source:HGN(	chr4:106962755-107270383	NM_001142415, NN	.	NP_001135887, NP_00	XP_016864324, XP_0168	protein_coding	ENSG0000016402	9255	10648
3207	RBBP7	-0.621	0.017	RB binding protein 7, chromatin remodeling factor [Source:HGNC Symbol;Acc:HGNC:989	chrX:16804549-16888537	NM_001198719, NN	XM_011545554, XM_C	NP_001185648, NP_00	.	protein_coding	ENSG0000010205	5931	9890
3208	FDPS	-0.555	0.017	farnesyl diphosphate synthase [Source:HGNC Symbol;Acc:HGNC:3631]	chr1:155278538-155290457	NM_001135821, NN	XM_005244963, XM_C	NP_001129293, NP_00	XP_005245019, XP_0052	protein_coding	ENSG0000016075	2224	3631
3209	EPHX1	-0.737	0.017	epoxide hydrolase 1 [Source:HGNC Symbol;Acc:HGNC:3401]	chr1:225997796-226070069	NM_000120, NM_0	.	NP_000111, NP_00112	.	protein_coding	ENSG0000014381	2052	3401
3210	NAA10	-0.600	0.017	N(alpha)-acetyltransferase 10, NatA catalytic subunit [Source:HGNC Symbol;Acc:HGNC:	chrX:153194694-153200676	NM_001256119, NN	.	NP_001243048, NP_00	.	protein_coding	ENSG0000010203	8260	18704
3211	A4GALT	-0.824	0.017	alpha 1,4-galactosyltransferase [Source:HGNC Symbol;Acc:HGNC:18149]	chr22:43088126-43091639	NM_001318038, NN	XM_011530234, XM_C	NP_001304967, NP_05	XP_005261700, XP_0052	protein_coding	ENSG0000012827	53947	18149
3212	TMEM167A	0.706	0.017	transmembrane protein 167A [Source:HGNC Symbol;Acc:HGNC:28330]	chr5:82348664-82373315	NM_174909	.	NP_777569	.	protein_coding	ENSG0000017469	153339	28330
3213	COLEC11	-0.963	0.017	collectin subfamily member 11 [Source:HGNC Symbol;Acc:HGNC:17213]	chr2:3642421-3692234	NM_001255982, NN	XM_006711897, XM_C	NP_001242911, NP_00	XP_005263910, XP_0067	protein_coding	ENSG0000011800	78989	17213
3214	FRMD5	0.962	0.017	FERM domain containing 5 [Source:HGNC Symbol;Acc:HGNC:28214]	chr15:44162958-44487429	NM_001286490, NN	XM_011522123, XM_C	NP_001273419, NP_00	XP_005254787	protein_coding	ENSG0000017187	84978	28214
3215	ICK	0.824	0.017	intestinal cell kinase [Source:HGNC Symbol;Acc:HGNC:21219]	chr6:52866076-52926600	NM_014920, NM_0	XM_017010492, XM_C	NP_055735, NP_05759	XP_011512721, XP_0115	protein_coding	ENSG0000011214	22858	21219
3216	MDM4	1.088	0.017	MDM4, p53 regulator [Source:HGNC Symbol;Acc:HGNC:6974]	chr1:204485506-204527248	NM_001204171, NN	XM_011509568, XM_C	NP_001191100, NP_00	XP_011507868, XP_0115	protein_coding	ENSG0000019862	4194	6974
3217	RPL35	-0.602	0.017	ribosomal protein L35 [Source:HGNC Symbol;Acc:HGNC:10344]	chr9:127615695-127624240	NM_007209	.	NP_009140	.	protein_coding	ENSG0000013694	11224	10344
3218	CREB3L2	0.818	0.017	cAMP responsive element binding protein 3 like 2 [Source:HGNC Symbol;Acc:HGNC:237	chr7:137559724-137686813	NM_001253775, NN	XM_017012541, XM_C	NP_001240704, NP_00	XP_005250598, XP_0168	protein_coding	ENSG0000018215	64764	23720
3219	GRINA	-0.580	0.017	glutamate ionotropic receptor NMDA type subunit associated protein 1 [Source:HGNC S	chr8:145064225-145067583	NM_000837, NM_0	XM_005250899	NP_000828, NP_00100	XP_005250956	protein_coding	ENSG0000017871	2907	4589
3220	LSMEM1	1.278	0.017	leucine rich single-pass membrane protein 1 [Source:HGNC Symbol;Acc:HGNC:22036]	chr7:112120907-112131117	NM_001134468, NN	XM_011516076, XM_C	NP_001127940, NP_87	XP_006715994, XP_0067	protein_coding	ENSG0000018101	286006	22036
3221	ZNF207	0.596	0.017	zinc finger protein 207 [Source:HGNC Symbol;Acc:HGNC:12998]	chr17:30677135-30708905	NM_001032293, NN	XM_011525185, XM_C	NP_001027464, NP_00	XP_011523487, XP_0168	protein_coding	ENSG0000001024	693217	12998
3222	AP1S1	-0.571	0.017	adaptor related protein complex 1 sigma 1 subunit [Source:HGNC Symbol;Acc:HGNC:55	chr7:100797677-100804558	NM_001283	.	NP_001274	.	protein_coding	ENSG0000010636	1174	559
3223	MICALCL	0.972	0.017	MICAL C-terminal like [Source:HGNC Symbol;Acc:HGNC:25933]	chr11:12308446-12380691	NM_032867	.	NP_116256	.	protein_coding	ENSG0000013380	84953	25933
3224	LINC00116	-0.639	0.017	long intergenic non-protein coding RNA 116 [Source:HGNC Symbol;Acc:HGNC:27339]	chr2:110969105-111002997	.	.	.	.	protein_coding	ENSG0000017570	.	27339
3225	KLHL35	-0.869	0.017	kelch like family member 35 [Source:HGNC Symbol;Acc:HGNC:26597]	chr11:75133437-75141674	NM_001039548	XM_011544954	NP_001034637	XP_011543256	protein_coding	ENSG0000014924	283212	26597
3226	SPRY4	-0.616	0.017	sprouty RTK signaling antagonist 4 [Source:HGNC Symbol;Acc:HGNC:15533]	chr5:141689991-141704605	NM_001127496, NN	XM_011537685, XM_C	NP_001120968, NP_00	XP_005268576, XP_0115	protein_coding	ENSG0000018767	81848	15533
3227	FAM207A	-0.637	0.017	family with sequence similarity 207 member A [Source:HGNC Symbol;Acc:HGNC:15811]	chr21:46359924-46396904	NM_001316983, NN	XM_005261193, XM_C	NP_001303912, NP_00	XP_005261247	protein_coding	ENSG0000016025	85395	15811
3228	NBAS	0.676	0.017	neuroblastoma amplified sequence [Source:HGNC Symbol;Acc:HGNC:15625]	chr2:15307032-15701454	NM_015909	XM_017004317, XM_C	NP_056993	XP_011508659, XP_0115	protein_coding	ENSG0000015177	51594	15625
3229	HOXD11	0.788	0.018	homeobox D11 [Source:HGNC Symbol;Acc:HGNC:5134]	chr2:176972013-176974316	NM_021192	.	NP_067015	.	protein_coding	ENSG0000012871	3237	5134
3230	TSPAN15	-0.671	0.018	tetraspanin 15 [Source:HGNC Symbol;Acc:HGNC:23298]	chr10:71211228-71267425	NM_012339	.	NP_036471	.	protein_coding	ENSG0000009928	23555	23298
3231	CHST2	-0.809	0.018	carbohydrate sulfotransferase 2 [Source:HGNC Symbol;Acc:HGNC:1970]	chr3:142838172-142842856	NM_004267	.	NP_004258	.	protein_coding	ENSG0000017504	9435	1970
3232	MYBBP1A	-0.620	0.018	MYB binding protein 1a [Source:HGNC Symbol;Acc:HGNC:7546]	chr17:4402132-4458926	NM_001105538, NN	XM_011523616	NP_001099008, NP_05	XP_011521918	protein_coding	ENSG0000013238	10514	7546
3233	SNAI3	-0.940	0.018	snail family transcriptional repressor 3 [Source:HGNC Symbol;Acc:HGNC:18411]	chr16:88744089-88752901	NM_178310	.	NP_840101	.	protein_coding	ENSG0000018566	333929	18411
3234	DNPEP	-0.597	0.018	aspartyl aminopeptidase [Source:HGNC Symbol;Acc:HGNC:2981]	chr2:220238267-220264729	NM_001319121, NN	XM_011510887, XM_C	NP_001306045, NP_00	XP_011509185	protein_coding	ENSG0000012399	23549	2981
3235	GORASP1	-0.678	0.018	golgi reassembly stacking protein 1 [Source:HGNC Symbol;Acc:HGNC:16769]	chr3:39138149-39180391	NM_001278789, NN	XM_011534023, XM_C	NP_001265718, NP_00	XP_006713364	protein_coding	ENSG0000011474	64689	16769
3236	NR3C1	0.926	0.018	nuclear receptor subfamily 3 group C member 1 [Source:HGNC Symbol;Acc:HGNC:7978]	chr5:142657495-142815077	NM_000176, NM_0	XM_011537637, XM_C	NP_000167, NP_00101	XP_005268476, XP_0052	protein_coding	ENSG0000011358	2908	7978
3237	RANGRF	-0.651	0.018	RAN guanine nucleotide release factor [Source:HGNC Symbol;Acc:HGNC:17679]	chr17:8191080-8198661	NM_001177801, NN	XM_005256618	NP_001171272, NP_00	XP_005256675	protein_coding	ENSG0000010896	29098	17679
3238	UBASH3B	0.664	0.018	ubiquitin associated and SH3 domain containing B [Source:HGNC Symbol;Acc:HGNC:298	chr11:122526382-122685181	NM_032873	XM_011543042, XM_C	NP_116262	XP_005271769, XP_0115	protein_coding	ENSG0000015412	84959	29884

3239	DEDD2	-0.653	0.018	death effector domain containing 2 [Source:HGNC Symbol;Acc:HGNC:24450]	chr19:42702749-42724275	NM_001270614, NN	XM_011526572	NP_001257543, NP_00	XP_011524874	protein_coding	ENSG0000016057	162989	24450
3240	PTK6	-0.610	0.018	protein tyrosine kinase 6 [Source:HGNC Symbol;Acc:HGNC:9617]	chr20:62159353-62168723	NM_001256358, NN	.	NP_001243287, NP_00	.	protein_coding	ENSG0000010121	5753	9617
3241	ZBTB7B	-0.671	0.018	zinc finger and BTB domain containing 7B [Source:HGNC Symbol;Acc:HGNC:18668]	chr1:154975105-154990998	NM_001252406, NN	XM_011509599, XM_0	NP_001239335, NP_00	XP_006711412, XP_0067	protein_coding	ENSG0000016068	51043	18668
3242	ZNF714	1.363	0.018	zinc finger protein 714 [Source:HGNC Symbol;Acc:HGNC:27124]	chr19:21264979-21308073	NM_182515	.	NP_872321	.	protein_coding	ENSG0000016035	148206	27124
3243	PLIN3	-0.576	0.018	perilipin 3 [Source:HGNC Symbol;Acc:HGNC:16893]	chr19:4838352-4867780	NM_001164189, NN	.	NP_001157661, NP_00	.	protein_coding	ENSG0000010535	10226	16893
3244	TMEM97	0.773	0.018	transmembrane protein 97 [Source:HGNC Symbol;Acc:HGNC:28106]	chr17:26646120-26662515	NM_014573	XM_005257965	NP_055388	XP_005258022	protein_coding	ENSG0000010908	27346	28106
3245	XRN2	0.570	0.018	5'-3' exoribonuclease 2 [Source:HGNC Symbol;Acc:HGNC:12836]	chr20:21283941-21370465	NM_001317960, NN	XM_011529184	NP_001304889, NP_03	.	protein_coding	ENSG0000008889	22803	12836
3246	EIF6	-0.571	0.018	eukaryotic translation initiation factor 6 [Source:HGNC Symbol;Acc:HGNC:6159]	chr20:33866709-33872788	NM_001267810, NN	.	NP_001254739, NP_00	.	protein_coding	ENSG0000024237	3692	6159
3247	EML2	-0.590	0.018	echinoderm microtubule associated protein like 2 [Source:HGNC Symbol;Acc:HGNC:180	chr19:46110251-46148887	NM_001193268, NN	XM_011526700, XM_0	NP_001180197, NP_00	XP_006723188, XP_0067	protein_coding	ENSG0000012574	442902	18035
3248	POU2F1	0.778	0.018	POU class 2 homeobox 1 [Source:HGNC Symbol;Acc:HGNC:9212]	chr1:167190065-167396582	NM_001198783, NN	XM_011509655, XM_0	NP_001185712, NP_00	XP_011507955, XP_0115	protein_coding	ENSG0000014319	5451	9212
3249	ZNF180	1.149	0.018	zinc finger protein 180 [Source:HGNC Symbol;Acc:HGNC:12970]	chr19:44978644-45004575	NM_001278508, NN	XM_017027254, XM_0	NP_001265437, NP_00	XP_011525582, XP_0115	protein_coding	ENSG0000016738	7733	12970
3250	ATM	1.444	0.018	ATM serine/threonine kinase [Source:HGNC Symbol;Acc:HGNC:795]	chr11:108093558-108338258	NM_000051	XM_017017790, XM_0	NP_000042	XP_005271618, XP_0052	protein_coding	ENSG0000014931	472	795
3251	DHX33	0.624	0.018	DEAH-box helicase 33 [Source:HGNC Symbol;Acc:HGNC:16718]	chr17:5344231-5372380	NM_001199699, NN	.	NP_001186628, NP_06	.	protein_coding	ENSG0000000510	56919	16718
3252	HMGA2	0.711	0.018	high mobility group AT-hook 2 [Source:HGNC Symbol;Acc:HGNC:5009]	chr12:66217910-66360075	NM_001300918, NN	.	NP_001287847, NP_00	XP_016875478	protein_coding	ENSG0000014994	8091	5009
3253	BTBD2	-0.609	0.018	BTB domain containing 2 [Source:HGNC Symbol;Acc:HGNC:15504]	chr19:1985436-2015723	NM_017797	XM_005259593	NP_060267	XP_005259650	protein_coding	ENSG0000013324	55643	15504
3254	CCDC24	-1.118	0.018	coiled-coil domain containing 24 [Source:HGNC Symbol;Acc:HGNC:28688]	chr1:44457171-44497058	NM_152499	XM_017000434, XM_0	NP_689712	XP_016855920, XP_0168	protein_coding	ENSG0000015921	149473	28688
3255	CRBN	-0.841	0.018	cereblon [Source:HGNC Symbol;Acc:HGNC:30185]	chr3:3191694-3221394	NM_001173482, NN	XM_011533793, XM_0	NP_001166953, NP_05	XP_005265259, XP_0115	protein_coding	ENSG0000011385	51185	30185
3256	DISC1	0.917	0.018	disrupted in schizophrenia 1 [Source:HGNC Symbol;Acc:HGNC:2888]	chr1:231762560-232177018	NM_001012957, NN	.	NP_001012975, NP_00	.	protein_coding	ENSG0000016294	27185	2888
3257	PYCRL	-0.750	0.018	pyrroline-5-carboxylate reductase-like [Source:HGNC Symbol;Acc:HGNC:25846]	chr8:144686082-144691943	NM_023078	.	NP_075566	.	protein_coding	ENSG0000010452	65263	25846
3258	RORA	1.247	0.018	RAR related orphan receptor A [Source:HGNC Symbol;Acc:HGNC:10258]	chr15:60780482-61521518	NM_002943, NM_1	XM_011521879, XM_0	NP_002934, NP_59902	XP_005254641, XP_0115	protein_coding	ENSG0000006966	6095	10258
3259	ZDHHC14	0.848	0.018	zinc finger DHHC-type containing 14 [Source:HGNC Symbol;Acc:HGNC:20341]	chr6:157802164-158099178	NM_024630, NM_1	XM_011536123, XM_0	NP_078906, NP_71496	XP_011534425	protein_coding	ENSG0000017504	79683	20341
3260	GMNN	0.691	0.018	geminin, DNA replication inhibitor [Source:HGNC Symbol;Acc:HGNC:17493]	chr6:24775158-24786327	NM_001251989, NN	XM_005249159	NP_001238918, NP_00	XP_005249216, XP_0115	protein_coding	ENSG0000011231	51053	17493
3261	NAT8L	-0.688	0.018	N-acetyltransferase 8 like [Source:HGNC Symbol;Acc:HGNC:26742]	chr4:2061238-2070816	NM_178557	.	NP_848652	.	protein_coding	ENSG0000018581	339983	26742
3262	CCDC71L	0.784	0.018	coiled-coil domain containing 71-like [Source:HGNC Symbol;Acc:HGNC:26685]	chr7:106297210-106301442	NM_175884	.	NP_787080	.	protein_coding	ENSG0000025327	168455	26685
3263	NFE2L3	0.544	0.018	nuclear factor, erythroid 2 like 3 [Source:HGNC Symbol;Acc:HGNC:7783]	chr7:26191859-26226745	NM_004289	.	NP_004280	.	protein_coding	ENSG0000005034	9603	7783
3264	SMDT1	-0.695	0.018	single-pass membrane protein with aspartate rich tail 1 [Source:HGNC Symbol;Acc:HGN	chr22:42475698-42480288	NM_033318	XM_011530509	NP_201575	XP_011528811	protein_coding	ENSG0000018317	91689	25055
3265	AL592284.1	-1.152	0.018	Protein LOC642441 [Source:UniProtKB/TrEMBL;Acc:F5H305]	chr1:144339737-144521058	.	.	.	XP_005273044, XP_0011	protein_coding	ENSG0000023136	730256	.
3266	CXADR	0.998	0.018	coxsackie virus and adenovirus receptor [Source:HGNC Symbol;Acc:HGNC:2559]	chr21:18884699-18965897	NM_001207063, NN	XM_011529479, XM_0	NP_001193992, NP_00	XP_011527778, XP_0115	protein_coding	ENSG0000015463	1525	2559
3267	IL15	1.287	0.018	interleukin 15 [Source:HGNC Symbol;Acc:HGNC:5977]	chr4:142557751-142655140	NM_000585, NM_1	.	NP_000576, NP_75191	.	protein_coding	ENSG0000016413	3600	5977
3268	RSL1D1	0.578	0.018	ribosomal L1 domain containing 1 [Source:HGNC Symbol;Acc:HGNC:24534]	chr16:11927706-11945442	NM_015659	.	NP_056474	.	protein_coding	ENSG0000017149	26156	24534
3269	FKBP15	0.659	0.018	FK506 binding protein 15 [Source:HGNC Symbol;Acc:HGNC:23397]	chr9:115927799-115983641	NM_015258	XM_006717019, XM_0	NP_056073	XP_006717081, XP_0067	protein_coding	ENSG0000011932	23307	23397
3270	NUDT12	0.863	0.018	nudix hydrolase 12 [Source:HGNC Symbol;Acc:HGNC:18826]	chr5:102884555-102898494	NM_001300741, NN	XM_005272097, XM_0	NP_001287670, NP_11	XP_005272152, XP_0052	protein_coding	ENSG0000011287	83594	18826
3271	PIK3R3	0.932	0.018	phosphoinositide-3-kinase regulatory subunit 3 [Source:HGNC Symbol;Acc:HGNC:8981]	chr1:46505811-46598708	NM_001114172, NN	XM_017002614	NP_001107644, NP_00	XP_016858103	protein_coding	ENSG0000011746	8503	8981
3272	SPATA20	-0.605	0.018	spermatogenesis associated 20 [Source:HGNC Symbol;Acc:HGNC:26125]	chr17:48624449-48633213	NM_001258372, NN	.	NP_001245301, NP_00	.	protein_coding	ENSG0000000628	64847	26125
3273	FGF13	0.905	0.018	fibroblast growth factor 13 [Source:HGNC Symbol;Acc:HGNC:3670]	chrX:137696891-138287185	NM_001139498, NN	XM_011531315, XM_0	NP_001132970, NP_00	XP_005262456	protein_coding	ENSG0000012968	2258	3670
3274	GOSR1	0.851	0.018	golgi SNAP receptor complex member 1 [Source:HGNC Symbol;Acc:HGNC:4430]	chr17:28804379-28854610	NM_001007024, NN	XM_005258072	NP_001007025, NP_00	XP_005258129, XP_0168	protein_coding	ENSG0000010858	9527	4430
3275	NADK	-0.644	0.018	NAD kinase [Source:HGNC Symbol;Acc:HGNC:29831]	chr1:1682670-1711508	NM_001198993, NN	XM_011542011, XM_0	NP_001185922, NP_00	XP_005244835, XP_0067	protein_coding	ENSG0000000813	65220	29831
3276	RAB9A	-0.860	0.018	RAB9A, member RAS oncogene family [Source:HGNC Symbol;Acc:HGNC:9792]	chrX:13707243-13728625	NM_001195328, NN	.	NP_001182257, NP_00	XP_016885453	protein_coding	ENSG0000012359	9367	9792
3277	EIF2S3	-0.562	0.018	eukaryotic translation initiation factor 2 subunit gamma [Source:HGNC Symbol;Acc:HGN	chrX:24072832-24096088	NM_001415	.	NP_001406	.	protein_coding	ENSG0000013074	1968	3267
3278	RPRD1A	0.816	0.018	regulation of nuclear pre-mRNA domain containing 1A [Source:HGNC Symbol;Acc:HGNC	chr18:33569786-33647539	NM_001303411, NN	XM_011526050, XM_0	NP_001290340, NP_00	XP_011524350, XP_0115	protein_coding	ENSG0000014142	55197	25560
3279	CDK10	-0.966	0.018	cyclin dependent kinase 10 [Source:HGNC Symbol;Acc:HGNC:1770]	chr16:89747144-89768113	NM_001098533, NN	XM_011523416, XM_0	NP_001092003, NP_00	XP_006721371, XP_0067	protein_coding	ENSG0000018532	8558	1770
3280	PHGDH	-0.556	0.018	phosphoglycerate dehydrogenase [Source:HGNC Symbol;Acc:HGNC:8923]	chr1:120254509-120286838	NM_006623	XM_011541231, XM_0	NP_006614	XP_011539528, XP_0115	protein_coding	ENSG0000009262	26227	8923
3281	MIA3	0.873	0.018	MIA family member 3, ER export factor [Source:HGNC Symbol;Acc:HGNC:24008]	chr1:222791427-222841354	NM_001300867, NN	XM_006711304, XM_0	NP_001287796, NP_00	XP_006711367, XP_0168	protein_coding	ENSG0000015430	375056	24008
3282	PAPD5	0.888	0.018	poly(A) RNA polymerase D5, non-canonical [Source:HGNC Symbol;Acc:HGNC:30758]	chr16:50186828-50265742	NM_001040284, NN	XM_017023555, XM_0	NP_001035374, NP_00	XP_005256149, XP_0052	protein_coding	ENSG0000012127	64282	30758
3283	TMEM192	1.018	0.018	transmembrane protein 192 [Source:HGNC Symbol;Acc:HGNC:26775]	chr4:165991759-166244308	NM_001100389	XM_011531718, XM_0	NP_001093859	XP_011530019, XP_0115	protein_coding	ENSG0000017008	201931	26775
3284	TREML2	-0.688	0.018	triggering receptor expressed on myeloid cells like 2 [Source:HGNC Symbol;Acc:HGNC:2	chr6:41158014-41168932	NM_024807	.	NP_079083	.	protein_coding	ENSG0000011219	79865	21092
3285	CPT1B	-0.870	0.018	carnitine palmitoyltransferase 1B [Source:HGNC Symbol;Acc:HGNC:2329]	chr22:51007289-51016884	NM_001145134, NN	.	NP_001138606, NP_00	.	protein_coding	ENSG0000020556	1375	2329
3286	SYNCRIP	0.578	0.018	synaptotagmin binding cytoplasmic RNA interacting protein [Source:HGNC Symbol;Acc:	chr6:86317502-86353003	NM_001159673, NN	XM_017010179, XM_0	NP_001153145, NP_00	XP_005248692, XP_0052	protein_coding	ENSG0000013531	10492	16918
3287	PAFAH2	0.776	0.018	platelet activating factor acetylhydrolase 2 [Source:HGNC Symbol;Acc:HGNC:8579]	chr1:26286257-26324648	NM_000437	XM_011541528, XM_0	NP_000428	XP_006710733, XP_0115	protein_coding	ENSG0000015800	5051	8579
3288	PLEKHB2	0.594	0.018	pleckstrin homology domain containing B2 [Source:HGNC Symbol;Acc:HGNC:19236]	chr2:131862419-132111282	NM_001100623, NN	XM_011511386, XM_0	NP_001094093, NP_00	XP_011509687, XP_0115	protein_coding	ENSG0000011576	55041	19236
3289	PPP1R1A	-3.485	0.018	protein phosphatase 1 regulatory inhibitor subunit 1A [Source:HGNC Symbol;Acc:HGNC	chr12:54943133-54982443	NM_006741	XM_005268995	NP_006732	XP_005269052	protein_coding	ENSG0000013544	5502	9286
3290	PTPRG	0.848	0.018	protein tyrosine phosphatase, receptor type G [Source:HGNC Symbol;Acc:HGNC:9671]	chr3:61547584-62283288	NM_002841	XM_017006962, XM_0	NP_002832	XP_005265410, XP_0168	protein_coding	ENSG0000014472	5793	9671
3291	RPUSD3	-1.425	0.018	RNA pseudouridylate synthase domain containing 3 [Source:HGNC Symbol;Acc:HGNC:28	chr3:9821543-9896822	NM_001142547, NN	XM_011533627	NP_001136019, NP_77	XP_011531929	protein_coding	ENSG0000015699	285367	28437
3292	CYFIP2	-0.699	0.018	cytoplasmic FMR1 interacting protein 2 [Source:HGNC Symbol;Acc:HGNC:13760]	chr5:156693088-156822603	NM_001037333, NN	XM_017009341, XM_0	NP_001032410, NP_00	XP_011532818, XP_0168	protein_coding	ENSG0000005516	26999	13760

3293	URAD	-1.717	0.018	ureidoimidazoline (2-oxo-4-hydroxy-4-carboxy-5-) decarboxylase [Source:HGNC Symbol	chr13:28551850-28562791	NM_001105577	.	NP_001099047	.	protein_coding	ENSG0000018346	646625	17785
3294	BTBD7	0.772	0.019	BTB domain containing 7 [Source:HGNC Symbol;Acc:HGNC:18269]	chr14:93703895-93799438	NM_001002860, N	XM_011536939	NP_001002860, NP_00	XP_011535241	protein_coding	ENSG0000001111	55727	18269
3295	TMEM184C	1.103	0.019	transmembrane protein 184C [Source:HGNC Symbol;Acc:HGNC:25587]	chr4:148538533-148605381	NM_018241	.	NP_060711	.	protein_coding	ENSG0000016416	55751	25587
3296	KIAA0196	0.728	0.019	KIAA0196 [Source:HGNC Symbol;Acc:HGNC:28984]	chr8:126036501-126104082	NM_014846	XM_017014113, XM_	NP_055661	XP_005251177, XP_0115	protein_coding	ENSG0000016496	9897	28984
3297	KLHL25	-0.745	0.019	kelch like family member 25 [Source:HGNC Symbol;Acc:HGNC:25732]	chr15:86302553-86338261	NM_022480	.	NP_071925	.	protein_coding	ENSG0000018365	64410	25732
3298	SGMS2	1.057	0.019	sphingomyelin synthase 2 [Source:HGNC Symbol;Acc:HGNC:28395]	chr4:108745718-108836203	NM_001136257, N	XM_017007840, XM_	NP_001129729, NP_00	XP_006714184, XP_0115	protein_coding	ENSG0000016402	166929	28395
3299	AKR1B1	-1.320	0.019	aldo-keto reductase family 1 member B [Source:HGNC Symbol;Acc:HGNC:381]	chr7:134127101-134143894	NM_001628	XM_005250234	NP_001619	XP_005250291	protein_coding	ENSG0000008566	231	381
3300	TMEM219	-0.619	0.019	transmembrane protein 219 [Source:HGNC Symbol;Acc:HGNC:25201]	chr16:29952205-29984373	NM_001083613, N	XM_005255123	NP_001077082, NP_91	XP_005255180	protein_coding	ENSG0000014993	124446	25201
3301	ELMO3	-0.649	0.019	engulfment and cell motility 3 [Source:HGNC Symbol;Acc:HGNC:17289]	chr16:67233013-67237932	NM_024712	.	NP_078988	.	protein_coding	ENSG0000010289	79767	17289
3302	JUP	-0.546	0.019	junction plakoglobin [Source:HGNC Symbol;Acc:HGNC:6207]	chr17:39910855-39942950	NM_002230, NM_0	XM_017024590, XM_	NP_002221, NP_06883	XP_006721936, XP_0067	protein_coding	ENSG0000017380	3728	6207
3303	SIM2	-0.825	0.019	single-minded family bHLH transcription factor 2 [Source:HGNC Symbol;Acc:HGNC:1088	chr21:38071433-38122218	NM_005069, NM_0	.	NP_005060, NP_03366	.	protein_coding	ENSG0000015926	6493	10883
3304	TNFRSF6B	-1.174	0.019	TNF receptor superfamily member 6b [Source:HGNC Symbol;Acc:HGNC:11921]	chr20:62328020-62367494	NM_003823	.	NP_003814	.	protein_coding	ENSG0000024350	8771	11921
3305	ALKBH8	0.903	0.019	alkB homolog 8, tRNA methyltransferase [Source:HGNC Symbol;Acc:HGNC:25189]	chr11:107373451-107436472	NM_001301010, N	XM_005271729, XM_	NP_001287939, NP_62	XP_005271785, XP_0052	protein_coding	ENSG0000013776	91801	25189
3306	RWDD4	0.874	0.019	RWD domain containing 4 [Source:HGNC Symbol;Acc:HGNC:23750]	chr4:184560787-184580378	NM_001307922, N	XM_006714134	NP_001294851, NP_68	XP_006714197	protein_coding	ENSG0000018255	201965	23750
3307	HSF2	0.836	0.019	heat shock transcription factor 2 [Source:HGNC Symbol;Acc:HGNC:5225]	chr6:122720690-122754264	NM_001135564, N	.	NP_001129036, NP_00	.	protein_coding	ENSG0000002515	3298	5225
3308	PRTN3	-1.087	0.019	proteinase 3 [Source:HGNC Symbol;Acc:HGNC:9495]	chr19:840959-848175	NM_002777	.	NP_002768	.	protein_coding	ENSG0000019641	5657	9495
3309	RASD2	-0.855	0.019	RASD family member 2 [Source:HGNC Symbol;Acc:HGNC:18229]	chr22:35936914-35950048	NM_014310	XM_011530040, XM_	NP_055125	XP_005261499, XP_0115	protein_coding	ENSG0000010030	23551	18229
3310	RIOK1	0.755	0.019	RIO kinase 1 [Source:HGNC Symbol;Acc:HGNC:18656]	chr6:7326891-7418270	NM_031480	XM_011514933, XM_	NP_113668	XP_005249503, XP_0115	protein_coding	ENSG0000012478	83732	18656
3311	YWHAQ	0.541	0.019	tyrosine 3-monooxygenase/tryptophan 5-monooxygenase activation protein theta [Sou	chr2:9724100-9771143	NM_006826	.	NP_006817	.	protein_coding	ENSG0000013430	10971	12854
3312	ZNF627	0.823	0.019	zinc finger protein 627 [Source:HGNC Symbol;Acc:HGNC:30570]	chr19:11708234-11729950	NM_001290084, N	XM_011527780	NP_001277012, NP_00	XP_011526082	protein_coding	ENSG0000019855	199692	30570
3313	KLHL20	0.836	0.019	kelch like family member 20 [Source:HGNC Symbol;Acc:HGNC:25056]	chr1:173684122-173755840	NM_014458	XM_011509420, XM_	NP_055273	XP_005245150, XP_0115	protein_coding	ENSG0000007632	27252	25056
3314	POLR1B	0.598	0.019	RNA polymerase I subunit B [Source:HGNC Symbol;Acc:HGNC:20454]	chr2:113299491-113334727	NM_001137604, N	.	NP_001131076, NP_00	.	protein_coding	ENSG0000012563	84172	20454
3315	RNFT1	1.105	0.019	ring finger protein, transmembrane 1 [Source:HGNC Symbol;Acc:HGNC:30206]	chr17:57970406-58050462	NM_016125	.	NP_057209	.	protein_coding	ENSG0000018905	51136	30206
3316	PMVK	-0.680	0.019	phosphomevalonate kinase [Source:HGNC Symbol;Acc:HGNC:9141]	chr1:154897209-154909467	NM_006556	.	NP_006547	.	protein_coding	ENSG0000016334	10654	9141
3317	TNNI3	-0.681	0.019	troponin I3, cardiac type [Source:HGNC Symbol;Acc:HGNC:11947]	chr19:55663137-55669100	NM_000363	.	NP_000354	.	protein_coding	ENSG0000012999	7137	11947
3318	CKMT2	-0.753	0.019	creatine kinase, mitochondrial 2 [Source:HGNC Symbol;Acc:HGNC:1996]	chr5:80529103-80562216	NM_001099735, N	.	NP_001093205, NP_00	.	protein_coding	ENSG0000013173	1160	1996
3319	CYP1A1	-0.953	0.019	cytochrome P450 family 1 subfamily A member 1 [Source:HGNC Symbol;Acc:HGNC:259	chr15:75011882-75017951	NM_000499, NM_0	XM_017021953, XM_	NP_000490, NP_00130	XP_005254242, XP_0168	protein_coding	ENSG0000014046	1543	2595
3320	GNL3L	-0.744	0.019	G protein nucleolar 3 like [Source:HGNC Symbol;Acc:HGNC:25553]	chrX:54556643-54587504	NM_001184819, N	XM_011530799	NP_001171748, NP_06	XP_011529101	protein_coding	ENSG0000013011	54552	25553
3321	MZT2B	-0.865	0.019	mitotic spindle organizing protein 2B [Source:HGNC Symbol;Acc:HGNC:25886]	chr2:130908964-130948300	NM_025029	XM_011511888, XM_	NP_079305	XP_005263852, XP_0115	protein_coding	ENSG0000015208	80097	25886
3322	NR1H2	-0.572	0.019	nuclear receptor subfamily 1 group H member 2 [Source:HGNC Symbol;Acc:HGNC:7965	chr19:50879714-50886239	NM_001256647, N	.	NP_001243576, NP_00	.	protein_coding	ENSG0000013140	7376	7965
3323	SCAI	1.367	0.019	suppressor of cancer cell invasion [Source:HGNC Symbol;Acc:HGNC:26709]	chr9:127704886-127905775	NM_001144877, N	.	NP_001138349, NP_77	.	protein_coding	ENSG0000017361	286205	26709
3324	TMEM168	1.062	0.019	transmembrane protein 168 [Source:HGNC Symbol;Acc:HGNC:25826]	chr7:112402436-112430647	NM_001287497, N	XM_011516471, XM_	NP_001274426, NP_07	XP_011514772, XP_0115	protein_coding	ENSG0000014680	64418	25826
3325	ZNF688	-0.997	0.019	zinc finger protein 688 [Source:HGNC Symbol;Acc:HGNC:30489]	chr16:30580666-30584055	NM_001024683, N	.	NP_001019854, NP_66	.	protein_coding	ENSG0000022980	146542	30489
3326	MRPL57	-0.589	0.019	mitochondrial ribosomal protein L57 [Source:HGNC Symbol;Acc:HGNC:14514]	chr13:21750783-21753223	NM_024026	.	NP_076931	XP_016876229	protein_coding	ENSG0000017314	78988	14514
3327	FBXL15	-0.827	0.019	F-box and leucine rich repeat protein 15 [Source:HGNC Symbol;Acc:HGNC:28155]	chr10:104162373-104182893	NM_024326	XM_005270151, XM_	NP_077302	XP_005270207, XP_0052	protein_coding	ENSG0000010787	79176	28155
3328	RPP25L	-0.676	0.019	ribonuclease P/MRP subunit p25 like [Source:HGNC Symbol;Acc:HGNC:19909]	chr9:34610482-34612101	NM_148178, NM_1	.	NP_680544, NP_68054	.	protein_coding	ENSG0000016496	138716	19909
3329	ZNF484	0.937	0.019	zinc finger protein 484 [Source:HGNC Symbol;Acc:HGNC:23385]	chr9:95607312-95640320	NM_001007101, N	.	NP_001007102, NP_00	XP_006717362, XP_0115	protein_coding	ENSG0000012708	83744	23385
3330	SLC39A3	-0.794	0.019	solute carrier family 39 member 3 [Source:HGNC Symbol;Acc:HGNC:17128]	chr19:2714564-2740150	NM_144564, NM_2	.	NP_653165, NP_99873	.	protein_coding	ENSG0000014187	29985	17128
3331	B9D1	-0.845	0.019	B9 domain containing 1 [Source:HGNC Symbol;Acc:HGNC:24123]	chr17:19240866-19290553	NM_001243473, N	XM_011523793, XM_	NP_001230402, NP_00	XP_005256662, XP_0052	protein_coding	ENSG0000010864	27077	24123
3332	TMEM121	-0.895	0.019	transmembrane protein 121 [Source:HGNC Symbol;Acc:HGNC:20511]	chr14:105992939-105996539	NM_025268	XM_011537186, XM_	NP_079544	XP_005268158, XP_0067	protein_coding	ENSG0000018498	80757	20511
3333	NRAS	0.703	0.019	neuroblastoma RAS viral oncogene homolog [Source:HGNC Symbol;Acc:HGNC:7989]	chr1:115247089-115259515	NM_002524	.	NP_002515	.	protein_coding	ENSG0000021328	4893	7989
3334	PLXNB2	-0.573	0.019	plexin B2 [Source:HGNC Symbol;Acc:HGNC:9104]	chr22:50713407-50746056	NM_012401	XM_017028704, XM_	NP_036533	XP_005261966, XP_0052	protein_coding	ENSG0000019657	23654	9104
3335	TAGLN2	-0.529	0.019	transgelin 2 [Source:HGNC Symbol;Acc:HGNC:11554]	chr1:159887896-159895522	NM_001277223, N	.	NP_001264152, NP_00	.	protein_coding	ENSG0000015871	8407	11554
3336	TRAM2	0.863	0.019	translocation associated membrane protein 2 [Source:HGNC Symbol;Acc:HGNC:16855]	chr6:52226920-52441713	NM_012288	XM_011515005	NP_036420	XP_011513307	protein_coding	ENSG0000006530	9697	16855
3337	WSB1	0.880	0.019	WD repeat and SOCS box containing 1 [Source:HGNC Symbol;Acc:HGNC:19221]	chr17:25621105-25642952	NM_015626, NM_1	XM_011524605, XM_	NP_056441, NP_59902	XP_005258020	protein_coding	ENSG0000010904	26118	19221
3338	MRPS18A	-0.622	0.019	mitochondrial ribosomal protein S18A [Source:HGNC Symbol;Acc:HGNC:14515]	chr6:43612782-43655528	NM_001193343, N	XM_006715134	NP_001180272, NP_06	XP_006715197	protein_coding	ENSG0000009608	55168	14515
3339	RHBDL1	-1.070	0.019	rhomboid like 1 [Source:HGNC Symbol;Acc:HGNC:10007]	chr16:725665-728268	NM_001278720, N	XM_011522732, XM_	NP_001265649, NP_00	XP_005255723	protein_coding	ENSG0000010326	9028	10007
3340	THAP5	1.369	0.019	THAP domain containing 5 [Source:HGNC Symbol;Acc:HGNC:23188]	chr7:108202670-108215294	NM_001130475, N	.	NP_001123947, NP_87	.	protein_coding	ENSG0000017768	168451	23188
3341	FAM234A	-0.615	0.019	family with sequence similarity 234 member A [Source:HGNC Symbol;Acc:HGNC:14163]	chr16:284544-325929	NM_001284497, N	XM_017023762, XM_	NP_001271426, NP_11	XP_005255679, XP_0052	protein_coding	ENSG0000016793	83986	14163
3342	IMPA1	0.921	0.019	inositol monophosphatase 1 [Source:HGNC Symbol;Acc:HGNC:6050]	chr8:82570195-82598928	NM_001144878, N	.	NP_001138350, NP_00	.	protein_coding	ENSG0000013373	3612	6050
3343	RHOT2	-0.599	0.019	ras homolog family member T2 [Source:HGNC Symbol;Acc:HGNC:21169]	chr16:718085-724174	NM_138769	XM_017023839, XM_	NP_620124	XP_005255717, XP_0052	protein_coding	ENSG0000014098	89941	21169
3344	SPIN2B	-0.932	0.019	spindlin family member 2B [Source:HGNC Symbol;Acc:HGNC:33147]	chrX:57146114-57147979	NM_001006681, N	XM_011530790, XM_	NP_001006682, NP_00	XP_005262066, XP_0052	protein_coding	ENSG0000018678	474343	33147
3345	CHMP3	0.702	0.019	charged multivesicular body protein 3 [Source:HGNC Symbol;Acc:HGNC:29865]	chr2:86730553-87005164	NM_001005753, N	.	NP_001005753, NP_00	.	protein_coding	ENSG0000011556	51652	29865
3346	UBD	-1.376	0.019	ubiquitin D [Source:HGNC Symbol;Acc:HGNC:18795]	chr6:29523291-29600962	NM_006398	.	.	.	protein_coding	ENSG0000021388	10537	18795

3347	ALKBH7	-0.813	0.019	alkB homolog 7 [Source:HGNC Symbol;Acc:HGNC:21306]	chr19:6372443-6379069	NM_032306	.	NP_115682	.	protein_coding	ENSG0000012565	84266	21306
3348	BMP2K	1.285	0.019	BMP2 inducible kinase [Source:HGNC Symbol;Acc:HGNC:18041]	chr4:79697531-79860592	NM_017593, NM_1	XM_017008382, XM_0	NP_060063, NP_94259	XP_005263174, XP_0115	protein_coding	ENSG0000013875	55589	18041
3349	MAGIX	-1.500	0.019	MAGI family member, X-linked [Source:HGNC Symbol;Acc:HGNC:30006]	chrX:49020120-49024820	NM_001099681, NM	XM_011543987, XM_0	NP_001093151, NP_00	XP_005278119, XP_0052	protein_coding	ENSG0000026931	79917	30006
3350	ARHGAP35	0.567	0.019	Rho GTPase activating protein 35 [Source:HGNC Symbol;Acc:HGNC:4591]	chr19:47421932-47508334	NM_004491	XM_017026714	NP_004482	XP_011525175, XP_0168	protein_coding	ENSG0000016000	2909	4591
3351	CYHR1	-0.736	0.019	cysteine and histidine rich 1 [Source:HGNC Symbol;Acc:HGNC:17806]	chr8:145674964-145691060	NM_001129888, NM	XM_006716562, XM_0	NP_001123360, NP_11	XP_006716623, XP_0067	protein_coding	ENSG0000018795	50626	17806
3352	EHHADH	0.775	0.019	enoyl-CoA, hydratase/3-hydroxyacyl CoA dehydrogenase [Source:HGNC Symbol;Acc:HG	chr3:184908411-184971886	NM_001166415, NM	XM_011512517, XM_0	NP_001159887, NP_00	XP_006713588, XP_0115	protein_coding	ENSG0000011379	1962	3247
3353	GALNT1	0.813	0.019	polypeptide N-acetylgalactosaminyltransferase 1 [Source:HGNC Symbol;Acc:HGNC:4123	chr18:33234523-33291798	NM_020474	XM_017025692, XM_0	NP_065207	XP_005258296, XP_0168	protein_coding	ENSG0000014142	2589	4123
3354	MSX1	-0.586	0.019	msh homeobox 1 [Source:HGNC Symbol;Acc:HGNC:7391]	chr4:4861392-4865663	NM_002448	.	NP_002439	.	protein_coding	ENSG0000016313	4487	7391
3355	PARD6B	0.877	0.019	par-6 family cell polarity regulator beta [Source:HGNC Symbol;Acc:HGNC:16245]	chr20:49348080-49373332	NM_032521	.	NP_115910	.	protein_coding	ENSG0000012417	84612	16245
3356	RPS5	-0.587	0.019	ribosomal protein S5 [Source:HGNC Symbol;Acc:HGNC:10426]	chr19:58897766-58906173	NM_001009	.	NP_001000	.	protein_coding	ENSG0000008384	6193	10426
3357	BOK	-0.617	0.020	BOK, BCL2 family apoptosis regulator [Source:HGNC Symbol;Acc:HGNC:1087]	chr2:242498135-242513553	NM_032515	XM_011511696, XM_0	NP_115904	XP_005247094, XP_0115	protein_coding	ENSG0000017672	666	1087
3358	EDF1	-0.551	0.020	endothelial differentiation related factor 1 [Source:HGNC Symbol;Acc:HGNC:3164]	chr9:139756570-139760738	NM_001281297, NM	.	NP_001268226, NP_00	.	protein_coding	ENSG0000010722	8721	3164
3359	GPSM1	-0.883	0.020	G-protein signaling modulator 1 [Source:HGNC Symbol;Acc:HGNC:17858]	chr9:139221931-139258241	NM_001145638, NM	XM_011518498	NP_001139110, NP_00	XP_011516800, XP_0168	protein_coding	ENSG0000016036	26086	17858
3360	MBLAC2	0.935	0.020	metallo-beta-lactamase domain containing 2 [Source:HGNC Symbol;Acc:HGNC:33711]	chr5:89754019-89770585	NM_203406	.	NP_981951	.	protein_coding	ENSG0000017605	153364	33711
3361	MRC2	-0.575	0.020	mannose receptor C type 2 [Source:HGNC Symbol;Acc:HGNC:16875]	chr17:60704761-60770958	NM_006039	.	NP_006030	.	protein_coding	ENSG0000001102	9902	16875
3362	DGCR6	-0.808	0.020	DiGeorge syndrome critical region gene 6 [Source:HGNC Symbol;Acc:HGNC:2846]	chr22:18893540-18899601	NM_005675	XM_011530394	NP_005666	XP_011528696	protein_coding	ENSG0000018362	8214	2846
3363	RAD21	0.609	0.020	RAD21 cohesin complex component [Source:HGNC Symbol;Acc:HGNC:9811]	chr8:117778741-117887105	NM_006265	.	NP_006256	.	protein_coding	ENSG0000016475	100500914	9811
3364	GCC1	0.653	0.020	GRIP and coiled-coil domain containing 1 [Source:HGNC Symbol;Acc:HGNC:19095]	chr7:127220671-127225661	NM_024523	.	NP_078799	.	protein_coding	ENSG0000017956	79571	19095
3365	KDM5A	0.720	0.020	lysine demethylase 5A [Source:HGNC Symbol;Acc:HGNC:9886]	chr12:389294-551811	NM_001042603	.	NP_001036068	.	protein_coding	ENSG0000007361	5927	9886
3366	PLCXD1	-0.915	0.020	phosphatidylinositol specific phospholipase C X domain containing 1 [Source:HGNC Sym	chrX:192988-220023	NM_018390	XM_011546180, XM_0	NP_060860	XP_006724507, XP_0067	protein_coding	ENSG0000018237	55344	23148
3367	QTRT1	-0.699	0.020	queuine tRNA-ribosyltransferase catalytic subunit 1 [Source:HGNC Symbol;Acc:HGNC:23	chr19:10812126-10824041	NM_031209	.	NP_112486	.	protein_coding	ENSG0000021333	81890	23797
3368	RPL18	-0.612	0.020	ribosomal protein L18 [Source:HGNC Symbol;Acc:HGNC:10310]	chr19:49118584-49140695	NM_000979, NM_0	.	NP_000970, NP_00125	.	protein_coding	ENSG0000006317	6141	10310
3369	ATXN1	0.641	0.020	ataxin 1 [Source:HGNC Symbol;Acc:HGNC:10548]	chr6:16299342-16761721	NM_000332, NM_0	.	NP_000323, NP_00112	.	protein_coding	ENSG0000012478	6310	10548
3370	BID	-0.722	0.020	BH3 interacting domain death agonist [Source:HGNC Symbol;Acc:HGNC:1050]	chr22:18216903-18257431	NM_001196, NM_0	.	NP_001187, NP_00123	.	protein_coding	ENSG0000001547	637	1050
3371	CARS2	-0.660	0.020	cysteinyl-tRNA synthetase 2, mitochondrial (putative) [Source:HGNC Symbol;Acc:HGNC:	chr13:111293763-111358504	NM_024537	XM_011521116, XM_0	NP_078813	XP_006720016, XP_0115	protein_coding	ENSG0000013490	79587	25695
3372	NPTXR	-0.672	0.020	neuronal pentraxin receptor [Source:HGNC Symbol;Acc:HGNC:7954]	chr22:39214456-39239987	NM_014293	.	NP_055108	.	protein_coding	ENSG0000022189	23467	7954
3373	RBM15	0.783	0.020	RNA binding motif protein 15 [Source:HGNC Symbol;Acc:HGNC:14959]	chr1:110881127-110889303	NM_001201545, NM	XM_011541969, XM_0	NP_001188474, NP_07	XP_011540266, XP_0115	protein_coding	ENSG0000016277	64783	14959
3374	SLMAP	0.779	0.020	sarcolemma associated protein [Source:HGNC Symbol;Acc:HGNC:16643]	chr3:57741956-57914895	NM_001304420, NM	XM_011534107, XM_0	NP_001291349, NP_00	XP_005265513, XP_0052	protein_coding	ENSG0000016368	7871	16643
3375	C21orf33	-0.990	0.020	chromosome 21 open reading frame 33 [Source:HGNC Symbol;Acc:HGNC:1273]	chr21:45527170-45565605	NM_001320384, NM	XM_011508907, XM_0	NP_001307313, NP_00	XP_005261242, XP_0067	protein_coding	ENSG0000016022	102724023	1273
3376	HENMT1	0.861	0.020	HEN1 methyltransferase homolog 1 [Source:HGNC Symbol;Acc:HGNC:26400]	chr1:109190911-109204148	NM_001102592, NM	XM_005270411	NP_001096062, NP_65	XP_005270468	protein_coding	ENSG0000016263	113802	26400
3377	P3H1	-0.706	0.020	prolyl 3-hydroxylase 1 [Source:HGNC Symbol;Acc:HGNC:19316]	chr1:43212005-43232755	NM_001146289, NM	XM_011541949, XM_0	NP_001139761, NP_00	XP_005271167, XP_0115	protein_coding	ENSG0000011738	64175	19316
3378	PTRH1	-0.864	0.020	peptidyl-tRNA hydrolase 1 homolog [Source:HGNC Symbol;Acc:HGNC:27039]	chr9:130469270-130497596	NM_001002913	XM_006716955	NP_001002913	XP_006717018, XP_0168	protein_coding	ENSG0000018702	138428	27039
3379	TPM1	0.667	0.020	tropomyosin 1 (alpha) [Source:HGNC Symbol;Acc:HGNC:12010]	chr15:63334830-63364114	NM_000366, NM_0	XM_006720667, XM_0	NP_000357, NP_00101	XP_005254694, XP_0052	protein_coding	ENSG0000014041	7168	12010
3380	MTMR12	0.735	0.020	myotubularin related protein 12 [Source:HGNC Symbol;Acc:HGNC:18191]	chr5:32227099-32313115	NM_001040446, NM	XM_011514060, XM_0	NP_001035536, NP_00	XP_005248370, XP_0115	protein_coding	ENSG0000015071	54545	18191
3381	TSTD1	-0.688	0.020	thiosulfate sulfurtransferase like domain containing 1 [Source:HGNC Symbol;Acc:HGNC:	chr1:161007420-161008780	NM_001113205, NM	.	NP_001106676, NP_00	.	protein_coding	ENSG0000021584	100131187	35410
3382	SGTA	-0.573	0.020	small glutamine rich tetratricopeptide repeat containing alpha [Source:HGNC Symbol;Ac	chr19:2754711-2783369	NM_003021	XM_011528178	NP_003012	XP_011526480	protein_coding	ENSG0000010496	6449	10819
3383	AEBP2	0.911	0.020	AE binding protein 2 [Source:HGNC Symbol;Acc:HGNC:24051]	chr12:19556999-19675173	NM_001114176, NM	XM_006719031	NP_001107648, NP_00	XP_006719094, XP_0168	protein_coding	ENSG0000013915	121536	24051
3384	NAPEPLD	0.942	0.020	N-acyl phosphatidylethanolamine phospholipase D [Source:HGNC Symbol;Acc:HGNC:21	chr7:102740222-102790007	NM_001122838, NM	XM_017011855, XM_0	NP_001116310, NP_94	XP_005250271, XP_0052	protein_coding	ENSG0000016104	222236	21683
3385	XXYLT1	-0.723	0.020	xyloside xylosyltransferase 1 [Source:HGNC Symbol;Acc:HGNC:26639]	chr3:194789007-194991896	NM_001308069, NM	XM_005269282	NP_001294998, NP_68	XP_005269339, XP_0168	protein_coding	ENSG0000017395	152002	26639
3386	ZNF497	-1.125	0.020	zinc finger protein 497 [Source:HGNC Symbol;Acc:HGNC:23714]	chr19:58865722-58874214	NM_001207009, NM	.	NP_001193938, NP_94	.	protein_coding	ENSG0000017458	162968	23714
3387	ZNF607	0.785	0.020	zinc finger protein 607 [Source:HGNC Symbol;Acc:HGNC:28192]	chr19:38187263-38210691	NM_001172677, NM	XM_006723436, XM_0	NP_001166148, NP_11	XP_006723498, XP_0067	protein_coding	ENSG0000019818	84775	28192
3388	LIN9	0.822	0.020	lin-9 DREAM MuvB core complex component [Source:HGNC Symbol;Acc:HGNC:30830]	chr1:226418857-226497434	NM_001270409, NM	XM_006711767, XM_0	NP_001257338, NP_00	XP_005273159, XP_0067	protein_coding	ENSG0000018381	286826	30830
3389	RADIL	-1.070	0.020	Rap associating with DIL domain [Source:HGNC Symbol;Acc:HGNC:22226]	chr7:4838735-4923350	NM_018059	.	NP_060529	.	protein_coding	ENSG0000015792	55698	22226
3390	AVPI1	-0.665	0.020	arginine vasopressin induced 1 [Source:HGNC Symbol;Acc:HGNC:30898]	chr10:99437180-99447080	NM_021732	XM_017016494	NP_068378	XP_016871983	protein_coding	ENSG0000011998	60370	30898
3391	EIF4E	0.813	0.020	eukaryotic translation initiation factor 4E [Source:HGNC Symbol;Acc:HGNC:3287]	chr4:99792834-99851788	NM_001130678, NM	XM_006714126	NP_001124150, NP_00	XP_006714189	protein_coding	ENSG0000015124	1977	3287
3392	ARMCX5	0.837	0.020	armadillo repeat containing, X-linked 5 [Source:HGNC Symbol;Acc:HGNC:25772]	chrX:101854095-101859087	NM_001168478, NM	.	NP_001161950, NP_00	.	protein_coding	ENSG0000012596	64860	25772
3393	CST2	-2.615	0.020	cystatin SA [Source:HGNC Symbol;Acc:HGNC:2474]	chr20:23804405-23807368	NM_001322	.	NP_001313	.	protein_coding	ENSG0000017036	1470	2474
3394	ESAM	0.909	0.020	endothelial cell adhesion molecule [Source:HGNC Symbol;Acc:HGNC:17474]	chr11:124623018-124632180	NM_138961	XM_011543060	NP_620411	XP_011541362	protein_coding	ENSG0000014956	90952	17474
3395	LRIF1	0.804	0.020	ligand dependent nuclear receptor interacting factor 1 [Source:HGNC Symbol;Acc:HGNC	chr1:111489806-111506701	NM_001006945, NM	XM_017001768, XM_0	NP_001006946, NP_06	XP_005271086, XP_0115	protein_coding	ENSG0000012193	55791	30299
3396	PTMS	-0.786	0.020	parathymosin [Source:HGNC Symbol;Acc:HGNC:9629]	chr12:6857161-6880118	NM_002824	XM_011520987	NP_002815	XP_011519289	protein_coding	ENSG0000015933	5763	9629
3397	SLC19A2	0.840	0.020	solute carrier family 19 member 2 [Source:HGNC Symbol;Acc:HGNC:10938]	chr1:169433146-169455241	NM_001319667, NM	XM_011509077	NP_001306596, NP_00	XP_011507379	protein_coding	ENSG0000011747	10560	10938
3398	C19orf66	-0.748	0.020	chromosome 19 open reading frame 66 [Source:HGNC Symbol;Acc:HGNC:25649]	chr19:10196805-10213472	NM_001308277, NM	XM_017026934, XM_0	NP_001295206, NP_06	XP_011526422, XP_0115	protein_coding	ENSG0000013081	55337	25649
3399	EDAR	0.546	0.020	ectodysplasin A receptor [Source:HGNC Symbol;Acc:HGNC:2895]	chr2:109510926-109605828	NM_022336	XM_011510503, XM_0	NP_071731	XP_006712267, XP_0115	protein_coding	ENSG0000013596	10913	2895
3400	HAND1	-1.299	0.020	heart and neural crest derivatives expressed 1 [Source:HGNC Symbol;Acc:HGNC:4807]	chr5:153854531-153857824	NM_004821	XM_005268531	NP_004812	XP_005268588	protein_coding	ENSG0000011319	9421	4807

3401	PRIM1	0.677	0.020	primase (DNA) subunit 1 [Source:HGNC Symbol;Acc:HGNC:9369]	chr12:57125405-57181574	NM_000946	.	NP_000937	.	protein_coding	ENSG0000019805	5557	9369
3402	RBBP5	0.730	0.020	RB binding protein 5, histone lysine methyltransferase complex subunit [Source:HGNC S	chr1:205055269-205091143	NM_001193272, NN	XM_011509834, XM_0	NP_001180201, NP_00	XP_011508135	protein_coding	ENSG0000011722	5929	9888
3403	YIF1A	-0.600	0.020	Yip1 interacting factor homolog A, membrane trafficking protein [Source:HGNC Symbol;	chr11:66052050-66056641	NM_001300861, NN	XM_005273720	NP_001287790, NP_06	XP_005273777, XP_0168	protein_coding	ENSG0000017485	10897	16688
3404	HPSE	1.027	0.020	heparanase [Source:HGNC Symbol;Acc:HGNC:5164]	chr4:84213613-84256306	NM_001098540, NN	.	NP_001092010, NP_00	.	protein_coding	ENSG0000017308	10855	5164
3405	ZFAND2A	0.755	0.020	zinc finger AN1-type containing 2A [Source:HGNC Symbol;Acc:HGNC:28073]	chr7:1191706-1199814	NM_182491	.	NP_872297	XP_016868263	protein_coding	ENSG0000017838	90637	28073
3406	CEP131	-0.674	0.020	centrosomal protein 131 [Source:HGNC Symbol;Acc:HGNC:29511]	chr17:79163392-79196799	NM_001009811, NN	XM_017024369, XM_0	NP_001009811, NP_00	XP_011522849, XP_0115	protein_coding	ENSG0000014157	22994	29511
3407	CLCN2	-1.057	0.020	chloride voltage-gated channel 2 [Source:HGNC Symbol;Acc:HGNC:2020]	chr3:184053713-184079439	NM_001171087, NN	XM_011512402, XM_0	NP_001164558, NP_00	XP_006713552, XP_0115	protein_coding	ENSG0000011485	1181	2020
3408	GPR35	-1.117	0.020	G protein-coupled receptor 35 [Source:HGNC Symbol;Acc:HGNC:4492]	chr2:241526132-241570676	NM_001195381, NN	.	NP_001182310, NP_00	.	protein_coding	ENSG0000017862	2859	4492
3409	RAD52	0.844	0.020	RAD52 homolog, DNA repair protein [Source:HGNC Symbol;Acc:HGNC:9824]	chr12:1021242-1099219	NM_001297419, NN	XM_011520996, XM_0	NP_001284348, NP_00	XP_005253777, XP_0052	protein_coding	ENSG0000000201	5893	9824
3410	ARHGAP19	0.757	0.020	Rho GTPase activating protein 19 [Source:HGNC Symbol;Acc:HGNC:23724]	chr10:98981929-99052430	NM_001204300, NN	.	NP_001191229, NP_00	.	protein_coding	ENSG0000021339	84986	23724
3411	CYC1	-0.545	0.020	cytochrome c1 [Source:HGNC Symbol;Acc:HGNC:2579]	chr8:145149929-145152428	NM_001916	.	NP_001907	.	protein_coding	ENSG0000017909	1537	2579
3412	RETSAT	-0.609	0.020	retinol saturase [Source:HGNC Symbol;Acc:HGNC:25991]	chr2:85569210-85581743	NM_017750	.	NP_060220	.	protein_coding	ENSG0000004244	54884	25991
3413	COL9A3	-0.586	0.021	collagen type IX alpha 3 chain [Source:HGNC Symbol;Acc:HGNC:2219]	chr20:61448413-61493115	NM_001853	XM_011528546, XM_0	NP_001844	XP_011526847	protein_coding	ENSG0000009275	1299	2219
3414	RPS10	-0.590	0.021	ribosomal protein S10 [Source:HGNC Symbol;Acc:HGNC:10383]	chr6:34247455-34393902	NM_001014, NM_0	.	NP_001005, NP_00119	.	protein_coding	ENSG0000012461	6204	10383
3415	DBNDD1	-0.598	0.021	dysbindin domain containing 1 [Source:HGNC Symbol;Acc:HGNC:28455]	chr16:90071272-90086536	NM_001042610, NN	.	NP_001036075, NP_00	.	protein_coding	ENSG0000000324	79007	28455
3416	SSBP4	-0.986	0.021	single stranded DNA binding protein 4 [Source:HGNC Symbol;Acc:HGNC:15676]	chr19:18530145-18549111	NM_001009998, NN	XM_011527748, XM_0	NP_001009998, NP_11	XP_005259847, XP_0067	protein_coding	ENSG0000013051	170463	15676
3417	ADSS	0.715	0.021	adenylosuccinate synthase [Source:HGNC Symbol;Acc:HGNC:292]	chr1:244571795-244615436	NM_001126	XM_011544109, XM_0	NP_001117	XP_011542410	protein_coding	ENSG0000003568	159	292
3418	MARCKSL1	-0.596	0.021	MARCKS like 1 [Source:HGNC Symbol;Acc:HGNC:7142]	chr1:32799432-32801980	NM_023009	.	NP_075385	.	protein_coding	ENSG0000017513	65108	7142
3419	SIX2	-1.098	0.021	SIX homeobox 2 [Source:HGNC Symbol;Acc:HGNC:10888]	chr2:45232299-45236569	NM_016932	XM_005264100	NP_058628	XP_005264157	protein_coding	ENSG0000017057	10736	10888
3420	SERBP1	0.569	0.021	SERPINE1 mRNA binding protein 1 [Source:HGNC Symbol;Acc:HGNC:17860]	chr1:67873492-67896098	NM_001018067, NN	.	NP_001018077, NP_00	.	protein_coding	ENSG0000014286	26135	17860
3421	NEU3	0.684	0.021	neuraminidase 3 [Source:HGNC Symbol;Acc:HGNC:7760]	chr11:74699178-74729938	NM_006656	.	NP_006647	.	protein_coding	ENSG0000016213	10825	7760
3422	SBK3	-1.088	0.021	SH3 domain binding kinase family member 3 [Source:HGNC Symbol;Acc:HGNC:44121]	chr19:56052022-56056909	NM_001199824	.	NP_001186753	.	protein_coding	ENSG0000023127	100130827	44121
3423	SETD3	0.589	0.021	SET domain containing 3 [Source:HGNC Symbol;Acc:HGNC:20493]	chr14:99864082-99947226	NM_032233, NM_1	XM_017021700, XM_0	NP_115609, NP_95457	XP_005268184, XP_0115	protein_coding	ENSG0000018357	84193	20493
3424	TMEM8A	-0.608	0.021	transmembrane protein 8A [Source:HGNC Symbol;Acc:HGNC:17205]	chr16:420772-431982	NM_021259	.	NP_067082	.	protein_coding	ENSG0000012992	58986	17205
3425	GRAP2	-1.510	0.021	GRB2-related adaptor protein 2 [Source:HGNC Symbol;Acc:HGNC:4563]	chr22:40297085-40369725	NM_001291824, NN	XM_011530516, XM_0	NP_001278753, NP_00	XP_006724439, XP_0115	protein_coding	ENSG0000010035	9402	4563
3426	BBS7	0.797	0.021	Bardet-Biedl syndrome 7 [Source:HGNC Symbol;Acc:HGNC:18758]	chr4:122745594-122791652	NM_018190, NM_1	XM_011532081, XM_0	NP_060660, NP_78979	XP_005263163, XP_0115	protein_coding	ENSG0000013868	55212	18758
3427	TBK1	0.760	0.021	TANK binding kinase 1 [Source:HGNC Symbol;Acc:HGNC:11584]	chr12:64845659-64895888	NM_013254	XM_005268810, XM_0	NP_037386	XP_005268866, XP_0052	protein_coding	ENSG0000018373	29110	11584
3428	TMEM123	0.579	0.021	transmembrane protein 123 [Source:HGNC Symbol;Acc:HGNC:30138]	chr11:102267062-102323775	NM_052932	.	NP_443164	.	protein_coding	ENSG0000015255	114908	30138
3429	ZBTB34	0.853	0.021	zinc finger and BTB domain containing 34 [Source:HGNC Symbol;Acc:HGNC:31446]	chr9:129622943-129648157	NM_001099270	XM_011518699, XM_0	NP_001092740	XP_005252046, XP_0115	protein_coding	ENSG0000017712	403341	31446
3430	DNMBP	0.625	0.021	dynamin binding protein [Source:HGNC Symbol;Acc:HGNC:30373]	chr10:101635333-101769676	NM_001318326, NN	XM_011539560, XM_0	NP_001305255, NP_00	XP_006717798, XP_0115	protein_coding	ENSG0000010755	23268	30373
3431	LEPROTL1	1.003	0.021	leptin receptor overlapping transcript-like 1 [Source:HGNC Symbol;Acc:HGNC:6555]	chr8:29952913-30002202	NM_001128208, NN	.	NP_001121680, NP_05	.	protein_coding	ENSG0000010466	23484	6555
3432	ORC3	0.717	0.021	origin recognition complex subunit 3 [Source:HGNC Symbol;Acc:HGNC:8489]	chr6:88299784-88377171	NM_001197259, NN	XM_017010639, XM_0	NP_001184188, NP_03	XP_005248761, XP_0052	protein_coding	ENSG0000013533	23595	8489
3433	SGMS1	0.799	0.021	sphingomyelin synthase 1 [Source:HGNC Symbol;Acc:HGNC:29799]	chr10:52065345-52383743	NM_147156	XM_011539585, XM_0	NP_671512	XP_005269732, XP_0115	protein_coding	ENSG0000019896	259230	29799
3434	CCDC43	0.702	0.021	coiled-coil domain containing 43 [Source:HGNC Symbol;Acc:HGNC:26472]	chr17:42754807-42767147	NM_001099225, NN	.	NP_001092695, NP_65	.	protein_coding	ENSG0000018032	124808	26472
3435	EXOSC4	-0.653	0.021	exosome component 4 [Source:HGNC Symbol;Acc:HGNC:18189]	chr8:145133528-145135550	NM_019037	XM_011517133	NP_061910	.	protein_coding	ENSG0000017889	102465510	18189
3436	BMPR1A	0.814	0.021	bone morphogenetic protein receptor type 1A [Source:HGNC Symbol;Acc:HGNC:1076]	chr10:88516406-88692595	NM_004329	XM_011540104, XM_0	NP_004320	XP_011538405, XP_0115	protein_coding	ENSG0000010777	657	1076
3437	FYN	0.576	0.021	FYN proto-oncogene, Src family tyrosine kinase [Source:HGNC Symbol;Acc:HGNC:4037]	chr6:111981534-112194655	NM_002037, NM_1	XM_011535668, XM_0	NP_002028, NP_69459	XP_005266947, XP_0052	protein_coding	ENSG0000001081	2534	4037
3438	LATS1	0.999	0.021	large tumor suppressor kinase 1 [Source:HGNC Symbol;Acc:HGNC:6514]	chr6:149979288-150039392	NM_001270519, NN	XM_017011482, XM_0	NP_001257448, NP_00	XP_011534552, XP_0168	protein_coding	ENSG0000013102	9113	6514
3439	RNF187	-0.567	0.021	ring finger protein 187 [Source:HGNC Symbol;Acc:HGNC:27146]	chr1:228674761-228683467	NM_001010858	.	NP_001010858	.	protein_coding	ENSG0000016815	149603	27146
3440	LBX2	-0.950	0.021	ladybird homeobox 2 [Source:HGNC Symbol;Acc:HGNC:15525]	chr2:74724643-74730443	NM_001009812, NN	.	NP_001009812, NP_00	.	protein_coding	ENSG0000017952	85474	15525
3441	RPL13A	-0.605	0.021	ribosomal protein L13a [Source:HGNC Symbol;Acc:HGNC:10304]	chr19:49990810-49995565	NM_001270491, NN	.	NP_001257420, NP_03	.	protein_coding	ENSG0000014254	23521	10304
3442	CRIP2	-0.772	0.021	cysteine rich protein 2 [Source:HGNC Symbol;Acc:HGNC:2361]	chr14:105939298-105946507	NM_001270837, NN	.	NP_001257766, NP_00	.	protein_coding	ENSG0000018280	1397	2361
3443	DUSP14	0.575	0.021	dual specificity phosphatase 14 [Source:HGNC Symbol;Acc:HGNC:17007]	chr17:35849936-35873603	NM_007026	XM_011524234, XM_0	NP_008957	XP_005257034, XP_0115	protein_coding	ENSG0000027602	11072	17007
3444	C1QL1	-0.892	0.021	complement C1q like 1 [Source:HGNC Symbol;Acc:HGNC:24182]	chr17:43037060-43045439	NM_006688	.	NP_006679	.	protein_coding	ENSG0000013109	10882	24182
3445	EPB41L4B	0.780	0.021	erythrocyte membrane protein band 4.1 like 4B [Source:HGNC Symbol;Acc:HGNC:19818	chr9:111934254-112083244	NM_018424, NM_0	XM_017014813, XM_0	NP_060894, NP_06198	XP_011517092, XP_0115	protein_coding	ENSG0000009520	54566	19818
3446	SUSD4	1.031	0.021	sushi domain containing 4 [Source:HGNC Symbol;Acc:HGNC:25470]	chr1:223394160-223537544	NM_001037175, NN	XM_017001588, XM_0	NP_001032252, NP_06	XP_005273226, XP_0052	protein_coding	ENSG0000014350	55061	25470
3447	B3GNT2	0.636	0.021	UDP-GlcNAc:betaGal beta-1,3-N-acetylglucosaminyltransferase 2 [Source:HGNC Symbol	chr2:62423247-62451866	NM_001319075, NN	XM_011532486	NP_001306004, NP_00	XP_011530788	protein_coding	ENSG0000017034	10678	15629
3448	THAP9	1.044	0.021	THAP domain containing 9 [Source:HGNC Symbol;Acc:HGNC:23192]	chr4:83821904-83841122	NM_024672	XM_005263220	NP_078948	XP_005263277, XP_0168	protein_coding	ENSG0000016815	79725	23192
3449	CDK1	0.600	0.021	cyclin dependent kinase 1 [Source:HGNC Symbol;Acc:HGNC:1722]	chr10:62538211-62554610	NM_001320918, NN	XM_006718082, XM_0	NP_001307847, NP_00	XP_005270360, XP_0067	protein_coding	ENSG0000017031	983	1722
3450	HDHD2	0.859	0.021	haloacid dehalogenase like hydrolase domain containing 2 [Source:HGNC Symbol;Acc:H	chr18:44633773-44676891	NM_001318765, NN	XM_011526228, XM_0	NP_001305694, NP_11	XP_006722620, XP_0115	protein_coding	ENSG0000016722	84064	25364
3451	LRRC37B	0.880	0.021	leucine rich repeat containing 37B [Source:HGNC Symbol;Acc:HGNC:29070]	chr17:30335006-30380523	NM_001321350, NN	XM_011524268, XM_0	NP_001308279, NP_44	XP_011522570	protein_coding	ENSG0000018515	114659	29070
3452	MIER3	1.746	0.021	MIER family member 3 [Source:HGNC Symbol;Acc:HGNC:26678]	chr5:56205086-56247952	NM_001297598, NN	XM_017009149, XM_0	NP_001284527, NP_00	XP_011541518, XP_0115	protein_coding	ENSG0000015554	166968	26678
3453	LMLN	0.868	0.021	leishmanolysin like peptidase [Source:HGNC Symbol;Acc:HGNC:15991]	chr3:197687070-197770591	NM_001136049, NN	XM_017007428, XM_0	NP_001129521, NP_14	XP_011511572, XP_0115	protein_coding	ENSG0000018562	89782	15991
3454	SLC2A4RG	-0.651	0.021	SLC2A4 regulator [Source:HGNC Symbol;Acc:HGNC:15930]	chr20:62367994-62374858	NM_020062	.	NP_064446	.	protein_coding	ENSG0000012552	56731	15930

3455	STXBP5	1.002	0.021	syntaxin binding protein 5 [Source:HGNC Symbol;Acc:HGNC:19665]	chr6:147525560-147711612	NM_001127715, NM	XM_011535453, XM_0	NP_001121187, NP_64	XP_006715402, XP_0115	protein_coding	ENSG0000016450	134957	19665
3456	IER3	-0.542	0.021	immediate early response 3 [Source:HGNC Symbol;Acc:HGNC:5392]	chr6:30710975-30712331	NM_003897	.	NP_003888	.	protein_coding	ENSG0000013733	8870	5392
3457	KRT19	-0.562	0.021	keratin 19 [Source:HGNC Symbol;Acc:HGNC:6436]	chr17:39679868-39684560	NM_002276	.	NP_002267	.	protein_coding	ENSG0000017134	3880	6436
3458	LEMD1	-0.726	0.022	LEM domain containing 1 [Source:HGNC Symbol;Acc:HGNC:18725]	chr1:205350505-205425082	NM_001001552, NM	XM_011510162, XM_0	NP_001001552, NP_00	XP_011508462, XP_0115	protein_coding	ENSG0000018600	93273	18725
3459	RGL1	0.769	0.022	ral guanine nucleotide dissociation stimulator like 1 [Source:HGNC Symbol;Acc:HGNC:30	chr1:183605219-183897665	NM_001297669, NM	XM_011509342, XM_0	NP_001284598, NP_00	XP_011507641, XP_0115	protein_coding	ENSG0000014334	23179	30281
3460	CIT	0.594	0.022	citron rho-interacting serine/threonine kinase [Source:HGNC Symbol;Acc:HGNC:1985]	chr12:120123594-120315095	NM_001206999, NM	XM_017018735, XM_0	NP_001193928, NP_00	XP_006719269, XP_0115	protein_coding	ENSG0000012296	100302274	1985
3461	CLIC1	-0.581	0.022	chloride intracellular channel 1 [Source:HGNC Symbol;Acc:HGNC:2062]	chr6:31694814-31707540	NM_001287593, NM	.	NP_001274522, NP_00	.	protein_coding	ENSG0000021371	1192	2062
3462	FOXO6	-0.927	0.022	forkhead box O6 [Source:HGNC Symbol;Acc:HGNC:24814]	chr1:41827593-41849262	NM_001291281	.	NP_001278210	.	protein_coding	ENSG0000020406	100132074	24814
3463	CBR4	0.799	0.022	carbonyl reductase 4 [Source:HGNC Symbol;Acc:HGNC:25891]	chr4:169908740-169931409	NM_032783	XM_006714392, XM_0	NP_116172	XP_005263373, XP_0067	protein_coding	ENSG0000014543	84869	25891
3464	NPEPPS	0.547	0.022	aminopeptidase puromycin sensitive [Source:HGNC Symbol;Acc:HGNC:7900]	chr17:45600322-45700642	NM_006310	XM_011525496	NP_006301	XP_011523798, XP_0168	protein_coding	ENSG0000014127	9520	7900
3465	ZBED8	0.890	0.022	zinc finger BED-type containing 8 [Source:HGNC Symbol;Acc:HGNC:30804]	chr5:159820154-159827104	NM_001303251, NM	.	NP_001290180, NP_07	.	protein_coding	ENSG0000022188	63920	30804
3466	FBXL6	-0.692	0.022	F-box and leucine rich repeat protein 6 [Source:HGNC Symbol;Acc:HGNC:13603]	chr8:145579090-145582172	NM_012162, NM_0	.	NP_036294, NP_07883	.	protein_coding	ENSG0000018232	26233	13603
3467	ROGDI	-0.741	0.022	rogdi homolog [Source:HGNC Symbol;Acc:HGNC:29478]	chr16:4846968-4852951	NM_024589	XM_006720948, XM_0	NP_078865	XP_006721010, XP_0067	protein_coding	ENSG0000006783	79641	29478
3468	THOC6	-0.819	0.022	THO complex 6 [Source:HGNC Symbol;Acc:HGNC:28369]	chr16:3072620-3086927	NM_001142350, NM	.	NP_001135822, NP_07	.	protein_coding	ENSG0000013165	79228	28369
3469	HRAS	-0.621	0.022	HRas proto-oncogene, GTPase [Source:HGNC Symbol;Acc:HGNC:5173]	chr11:532241-535550	NM_001130442, NM	.	NP_001123914, NP_00	.	protein_coding	ENSG0000017477	3265	5173
3470	MYL12A	0.525	0.022	myosin light chain 12A [Source:HGNC Symbol;Acc:HGNC:16701]	chr18:3247478-3256234	NM_001303047, NM	XM_005258078	NP_001289976, NP_00	XP_005258135	protein_coding	ENSG0000010160	10627	16701
3471	SPATS2	0.655	0.022	spermatogenesis associated serine rich 2 [Source:HGNC Symbol;Acc:HGNC:18650]	chr12:49761247-49921207	NM_001293285, NM	XM_011538691, XM_0	NP_001280214, NP_00	XP_011536992, XP_0115	protein_coding	ENSG0000012335	65244	18650
3472	STOM	1.076	0.022	stomatin [Source:HGNC Symbol;Acc:HGNC:3383]	chr9:124101265-124132582	NM_001270526, NM	.	NP_001257455, NP_00	.	protein_coding	ENSG0000014817	2040	3383
3473	CDK5R2	-1.010	0.022	cyclin dependent kinase 5 regulatory subunit 2 [Source:HGNC Symbol;Acc:HGNC:1776]	chr2:219824376-219826884	NM_003936	.	NP_003927	.	protein_coding	ENSG0000017145	8941	1776
3474	TSC22D1	-0.529	0.022	TSC22 domain family member 1 [Source:HGNC Symbol;Acc:HGNC:16826]	chr13:45006278-45150701	NM_001243797, NM	.	NP_001230726, NP_00	XP_005266640, XP_0168	protein_coding	ENSG0000010280	8848	16826
3475	ARL4C	-0.560	0.022	ADP ribosylation factor like GTPase 4C [Source:HGNC Symbol;Acc:HGNC:698]	chr2:235401684-235405697	NM_001282431, NM	.	NP_001269360, NP_00	.	protein_coding	ENSG0000018804	10123	698
3476	LRP11	0.682	0.022	LDL receptor related protein 11 [Source:HGNC Symbol;Acc:HGNC:16936]	chr6:150139933-150185162	NM_032832	XM_011536194, XM_0	NP_116221	XP_011534496, XP_0168	protein_coding	ENSG0000012025	84918	16936
3477	DUSP23	-0.709	0.022	dual specificity phosphatase 23 [Source:HGNC Symbol;Acc:HGNC:21480]	chr1:159750721-159752333	NM_001319658, NM	XM_005245289	NP_001306587, NP_00	XP_005245346	protein_coding	ENSG0000015871	54935	21480
3478	FBXL5	0.765	0.022	F-box and leucine rich repeat protein 5 [Source:HGNC Symbol;Acc:HGNC:13602]	chr4:15606161-15657035	NM_001193534, NM	XM_011513833, XM_0	NP_001180463, NP_00	XP_006714022, XP_0115	protein_coding	ENSG0000011856	26234	13602
3479	HOXB8	-0.608	0.022	homeobox B8 [Source:HGNC Symbol;Acc:HGNC:5119]	chr17:46689707-46692301	NM_024016	XM_005257286	NP_076921	XP_005257343, XP_0168	protein_coding	ENSG0000012006	3218	5119
3480	PYGB	-0.624	0.022	phosphorylase, glycogen; brain [Source:HGNC Symbol;Acc:HGNC:9723]	chr20:25228704-25371619	NM_002862	.	NP_002853	.	protein_coding	ENSG0000010099	5834	9723
3481	SNX27	0.691	0.022	sorting nexin family member 27 [Source:HGNC Symbol;Acc:HGNC:20073]	chr1:151584557-151671567	NM_030918	XM_017002417, XM_0	NP_112180	XP_005245566, XP_0052	protein_coding	ENSG0000014337	81609	20073
3482	TGFBR3L	-0.881	0.022	transforming growth factor beta receptor 3 like [Source:HGNC Symbol;Acc:HGNC:44152	chr19:7981029-7983982	NM_001195259	XM_011527613, XM_0	NP_001182188	XP_011525912, XP_0115	protein_coding	ENSG0000026000	100507588	44152
3483	MTFP1	-0.710	0.022	mitochondrial fission process 1 [Source:HGNC Symbol;Acc:HGNC:26945]	chr22:30792845-30825040	NM_001003704, NM	.	NP_001003704, NP_05	.	protein_coding	ENSG0000024211	51537	26945
3484	CD274	1.019	0.022	CD274 molecule [Source:HGNC Symbol;Acc:HGNC:17635]	chr9:5450502-5470566	NM_001267706, NM	.	NP_001254635, NP_05	.	protein_coding	ENSG0000012021	29126	17635
3485	DMPK	-0.711	0.022	dystrophia myotonica protein kinase [Source:HGNC Symbol;Acc:HGNC:2933]	chr19:46272974-46285747	NM_001081560, NM	.	NP_001075029, NP_00	.	protein_coding	ENSG0000010493	1760	2933
3486	MMP24-AS1	-0.722	0.022	MMP24 antisense RNA 1 [Source:HGNC Symbol;Acc:HGNC:44421]	chr20:33864952-33865932	.	.	.	.	protein_coding	ENSG0000012600	.	44421
3487	PSMB10	-0.635	0.022	proteasome subunit beta 10 [Source:HGNC Symbol;Acc:HGNC:9538]	chr16:67968404-67970990	NM_002801	.	NP_002792	.	protein_coding	ENSG0000020522	5699	9538
3488	TMEM56	1.171	0.022	transmembrane protein 56 [Source:HGNC Symbol;Acc:HGNC:26477]	chr1:95582893-95712781	NM_001199679, NM	.	NP_001186608, NP_68	.	protein_coding	ENSG0000015207	148534	26477
3489	ZNF770	0.730	0.022	zinc finger protein 770 [Source:HGNC Symbol;Acc:HGNC:26061]	chr15:35270541-35280488	NM_014106	XM_011521744	NP_054825	XP_011520046	protein_coding	ENSG0000019814	54989	26061
3490	FAM63B	0.875	0.022	family with sequence similarity 63 member B [Source:HGNC Symbol;Acc:HGNC:26954]	chr15:59063512-59154099	NM_001040450, NM	XM_011521690, XM_0	NP_001035540, NP_00	XP_011519989, XP_0115	protein_coding	ENSG0000012892	54629	26954
3491	KLK8	-1.044	0.022	kallikrein related peptidase 8 [Source:HGNC Symbol;Acc:HGNC:6369]	chr19:51499262-51505967	NM_001281431, NM	.	NP_001268360, NP_00	.	protein_coding	ENSG0000012945	11202	6369
3492	LRCH1	0.635	0.022	leucine rich repeats and calponin homology domain containing 1 [Source:HGNC Symbol	chr13:47127302-47327175	NM_001164211, NM	.	NP_001157683, NP_00	XP_016875972	protein_coding	ENSG0000013614	23143	20309
3493	FAM172A	0.936	0.022	family with sequence similarity 172 member A [Source:HGNC Symbol;Acc:HGNC:25365]	chr5:92953774-93447381	NM_001163417, NM	XM_011543673, XM_0	NP_001156889, NP_00	XP_005272160, XP_0052	protein_coding	ENSG0000011339	100313887	25365
3494	TTC30B	0.945	0.022	tetratricopeptide repeat domain 30B [Source:HGNC Symbol;Acc:HGNC:26425]	chr2:178413725-178417742	NM_152517	.	NP_689730	.	protein_coding	ENSG0000019665	150737	26425
3495	STAMBP	0.708	0.022	STAM binding protein [Source:HGNC Symbol;Acc:HGNC:16950]	chr2:74056042-74100760	NM_006463, NM_2	XM_017003180, XM_0	NP_006454, NP_96401	XP_005264145, XP_0115	protein_coding	ENSG0000012435	10617	16950
3496	TATDN1	0.764	0.022	TatD DNase domain containing 1 [Source:HGNC Symbol;Acc:HGNC:24220]	chr8:125500741-125551329	NM_001146160, NM	XM_017013895, XM_0	NP_001139632, NP_00	XP_006716729, XP_0067	protein_coding	ENSG0000014768	102466200	24220
3497	CAV2	0.956	0.022	caveolin 2 [Source:HGNC Symbol;Acc:HGNC:1528]	chr7:116139443-116148595	NM_001206747, NM	.	NP_001193676, NP_00	.	protein_coding	ENSG0000010597	858	1528
3498	SH3BGRL2	0.721	0.022	SH3 domain binding glutamate rich protein like 2 [Source:HGNC Symbol;Acc:HGNC:1556	chr6:80340999-80413376	NM_031469	XM_011536165	NP_113657	XP_011534467, XP_0168	protein_coding	ENSG0000019847	83699	15567
3499	TMEM221	-0.933	0.022	transmembrane protein 221 [Source:HGNC Symbol;Acc:HGNC:21943]	chr19:17546317-17571763	NM_001190844	XM_011527603	NP_001177773	XP_011525905	protein_coding	ENSG0000018805	100130519	21943
3500	ANK3	1.054	0.022	ankyrin 3, node of Ranvier (ankyrin G) [Source:HGNC Symbol;Acc:HGNC:494]	chr10:61786055-62493248	NM_001149, NM_0	XM_017016133, XM_0	NP_001140, NP_00119	XP_005269772, XP_0067	protein_coding	ENSG0000015115	288	494
3501	ARHGAP28	1.179	0.022	Rho GTPase activating protein 28 [Source:HGNC Symbol;Acc:HGNC:25509]	chr18:6729716-6915715	NM_001010000	XM_005258146, XM_0	NP_001010000	XP_005258201, XP_0052	protein_coding	ENSG0000008875	79822	25509
3502	DPY19L3	0.832	0.022	dpy-19 like 3 (C. elegans) [Source:HGNC Symbol;Acc:HGNC:27120]	chr19:32895914-32976801	NM_001172774, NM	XM_011526526, XM_0	NP_001166245, NP_99	XP_006723108, XP_0115	protein_coding	ENSG0000017890	147991	27120
3503	TMEM44	-0.747	0.022	transmembrane protein 44 [Source:HGNC Symbol;Acc:HGNC:25120]	chr3:194308401-194354418	NM_001011655, NM	XM_011513322, XM_0	NP_001011655, NP_00	XP_005269428, XP_0115	protein_coding	ENSG0000014501	93109	25120
3504	CCDC12	-1.080	0.022	coiled-coil domain containing 12 [Source:HGNC Symbol;Acc:HGNC:28332]	chr3:46963215-47051193	NM_001277074, NM	XM_011533395	NP_001264003, NP_65	XP_011531697	protein_coding	ENSG0000016079	151903	28332
3505	LZTS2	-0.579	0.022	leucine zipper tumor suppressor 2 [Source:HGNC Symbol;Acc:HGNC:29381]	chr10:102756828-102790914	NM_001318099, NM	XM_017016780, XM_0	NP_001305028, NP_00	XP_005270279, XP_0052	protein_coding	ENSG0000010781	84445	29381
3506	ZNF420	0.986	0.022	zinc finger protein 420 [Source:HGNC Symbol;Acc:HGNC:20649]	chr19:37569336-37621213	NM_144689	XM_017026338, XM_0	NP_001316444, NP_00	XP_006723101, XP_0115	protein_coding	ENSG0000019705	147923	20649
3507	DENND2C	1.013	0.023	DENN domain containing 2C [Source:HGNC Symbol;Acc:HGNC:24748]	chr1:115125468-115212732	NM_001256404, NM	.	NP_001243333, NP_94	.	protein_coding	ENSG0000017598	163259	24748
3508	IRF2BP1	-0.652	0.023	interferon regulatory factor 2 binding protein 1 [Source:HGNC Symbol;Acc:HGNC:21728	chr19:46386864-46389428	NM_015649	.	NP_056464	.	protein_coding	ENSG0000017060	26145	21728

3509	ISYNA1	-0.732	0.023	inositol-3-phosphate synthase 1 [Source:HGNC Symbol;Acc:HGNC:29821]	chr19:18530145-18549111	NM_001170938, NN	XM_011528059	NP_001164409, NP_00	XP_011526361	protein_coding	ENSG0000010565	51477	29821
3510	FAM114A2	0.912	0.023	family with sequence similarity 114 member A2 [Source:HGNC Symbol;Acc:HGNC:1333]	chr5:153369687-153418481	NM_001317993, NN	XM_011537544, XM_0	NP_001304922, NP_00	XP_005268417, XP_0052	protein_coding	ENSG0000005514	10827	1333
3511	SMIM10	0.786	0.023	small integral membrane protein 10 [Source:HGNC Symbol;Acc:HGNC:41913]	chrX:134124967-134126503	NM_001163438	.	NP_001156910	.	protein_coding	ENSG0000018478	644538	41913
3512	F3	0.726	0.023	coagulation factor III, tissue factor [Source:HGNC Symbol;Acc:HGNC:3541]	chr1:94994780-95007356	NM_001178096, NN	.	NP_001171567, NP_00	.	protein_coding	ENSG0000011752	2152	3541
3513	GPC1	-0.545	0.023	glypican 1 [Source:HGNC Symbol;Acc:HGNC:4449]	chr2:241375087-241407495	NM_002081	XM_011510976	NP_002072	XP_011509278	protein_coding	ENSG0000006366	2817	4449
3514	RUVBL2	-0.551	0.023	RuvB like AAA ATPase 2 [Source:HGNC Symbol;Acc:HGNC:10475]	chr19:49496704-49520338	NM_001321191, NN	XM_011526330, XM_0	NP_001308119, NP_00	XP_011524632	protein_coding	ENSG0000018320	10856	10475
3515	UBA7	-0.861	0.023	ubiquitin like modifier activating enzyme 7 [Source:HGNC Symbol;Acc:HGNC:12471]	chr3:49842639-49851379	NM_003335	XM_011534072, XM_0	NP_003326	XP_005265487, XP_0067	protein_coding	ENSG0000018217	100847079	12471
3516	HCN2	-0.667	0.023	hyperpolarization activated cyclic nucleotide gated potassium channel 2 [Source:HGNC	chr19:589892-617159	NM_001194	.	NP_001185	.	protein_coding	ENSG0000009982	610	4846
3517	TINAGL1	-0.540	0.023	tubulointerstitial nephritis antigen like 1 [Source:HGNC Symbol;Acc:HGNC:19168]	chr1:32042085-32053287	NM_001204414, NN	XM_005271107, XM_0	NP_001191343, NP_00	XP_005271163, XP_0052	protein_coding	ENSG0000014291	64129	19168
3518	HACD2	0.678	0.023	3-hydroxyacyl-CoA dehydratase 2 [Source:HGNC Symbol;Acc:HGNC:9640]	chr3:123210400-123304032	NM_198402	.	NP_940684	.	protein_coding	ENSG0000020652	201562	9640
3519	NCOA1	0.697	0.023	nuclear receptor coactivator 1 [Source:HGNC Symbol;Acc:HGNC:7668]	chr2:24714800-24993571	NM_003743, NM_1	XM_017005168, XM_0	NP_003734, NP_67175	XP_005264682, XP_0052	protein_coding	ENSG0000008467	8648	7668
3520	RIOK2	0.799	0.023	RIO kinase 2 [Source:HGNC Symbol;Acc:HGNC:18999]	chr5:96496570-96518964	NM_001159749, NN	.	NP_001153221, NP_06	.	protein_coding	ENSG0000005872	55781	18999
3521	SH3BP5L	-0.653	0.023	SH3 binding domain protein 5 like [Source:HGNC Symbol;Acc:HGNC:29360]	chr1:249104649-249120832	NM_030645	.	NP_085148	.	protein_coding	ENSG0000017513	80851	29360
3522	ZNF718	0.973	0.023	zinc finger protein 718 [Source:HGNC Symbol;Acc:HGNC:26889]	chr4:124361-157779	NM_001039127, NN	XM_011513424	NP_001034216, NP_00	XP_011511726, XP_0168	protein_coding	ENSG0000025031	255403	26889
3523	CHEK1	0.661	0.023	checkpoint kinase 1 [Source:HGNC Symbol;Acc:HGNC:1925]	chr11:125495030-125551018	NM_001114121, NN	XM_011542563, XM_0	NP_001107593, NP_00	XP_011540862, XP_0115	protein_coding	ENSG0000014955	1111	1925
3524	ST6GALNAC6	-0.609	0.023	ST6 N-acetylgalactosaminide alpha-2,6-sialyltransferase 6 [Source:HGNC Symbol;Acc:HG	chr9:130647599-130667627	NM_001286999, NN	XM_017014665, XM_0	NP_001273928, NP_00	XP_011516912, XP_0115	protein_coding	ENSG0000016040	30815	23364
3525	ZNF670	0.995	0.023	zinc finger protein 670 [Source:HGNC Symbol;Acc:HGNC:28167]	chr1:247200085-247242057	NM_001204220, NN	.	NP_001191149, NP_14	.	protein_coding	ENSG0000027746	93474	28167
3526	MAP3K7CL	0.980	0.023	MAP3K7 C-terminal like [Source:HGNC Symbol;Acc:HGNC:16457]	chr21:30449953-30548210	NM_001286617, NN	.	NP_001273546, NP_00	.	protein_coding	ENSG0000015626	56911	16457
3527	PLAG1	1.096	0.023	PLAG1 zinc finger [Source:HGNC Symbol;Acc:HGNC:9045]	chr8:57073462-57123859	NM_001114634, NN	XM_017013576, XM_0	NP_001108106, NP_00	XP_005251317, XP_0115	protein_coding	ENSG0000018169	5324	9045
3528	B3GALNT2	0.803	0.023	beta-1,3-N-acetylgalactosaminyltransferase 2 [Source:HGNC Symbol;Acc:HGNC:28596]	chr1:235613237-235667781	NM_001277155, NN	XM_006711749	NP_001264084, NP_68	XP_006711812	protein_coding	ENSG0000016288	148789	28596
3529	MINPP1	0.769	0.023	multiple inositol-polyphosphate phosphatase 1 [Source:HGNC Symbol;Acc:HGNC:7102]	chr10:89264631-89313217	NM_001178117, NN	XM_011540379	NP_001171588, NP_00	XP_011538681, XP_0168	protein_coding	ENSG0000010778	9562	7102
3530	RPL34	-0.552	0.023	ribosomal protein L34 [Source:HGNC Symbol;Acc:HGNC:10340]	chr4:109541721-109551568	NM_000995, NM_0	XM_006714287, XM_0	NP_000986, NP_00130	XP_005263229, XP_0067	protein_coding	ENSG0000010947	6164	10340
3531	ATP11B	0.672	0.023	ATPase phospholipid transporting 11B (putative) [Source:HGNC Symbol;Acc:HGNC:1355	chr3:182511287-182639423	NM_014616	XM_017006008, XM_0	NP_055431	XP_005247298, XP_0052	protein_coding	ENSG0000005806	23200	13553
3532	GPATCH3	-0.706	0.023	G-patch domain containing 3 [Source:HGNC Symbol;Acc:HGNC:25720]	chr1:27216979-27226957	NM_022078	.	NP_071361	.	protein_coding	ENSG0000019874	63906	25720
3533	CHN1	0.755	0.023	chimerin 1 [Source:HGNC Symbol;Acc:HGNC:1943]	chr2:175664090-175870097	NM_001025201, NN	.	NP_001020372, NP_00	.	protein_coding	ENSG0000012865	1123	1943
3534	LGALSL	0.711	0.023	galectin like [Source:HGNC Symbol;Acc:HGNC:25012]	chr2:64681225-64688515	NM_014181	.	NP_054900	.	protein_coding	ENSG0000011986	29094	25012
3535	SPATA5	0.841	0.023	spermatogenesis associated 5 [Source:HGNC Symbol;Acc:HGNC:18119]	chr4:123844228-124240605	NM_145207	XM_011531679, XM_0	NP_660208	XP_005262840, XP_0115	protein_coding	ENSG0000014537	166378	18119
3536	TSPAN13	0.614	0.023	tetraspanin 13 [Source:HGNC Symbol;Acc:HGNC:21643]	chr7:16793159-16824161	NM_014399	.	NP_055214	.	protein_coding	ENSG0000010653	27075	21643
3537	ROBO3	-0.917	0.023	roundabout guidance receptor 3 [Source:HGNC Symbol;Acc:HGNC:13433]	chr11:124735281-124751362	NM_022370	XM_017018122, XM_0	NP_071765	XP_016873611	protein_coding	ENSG0000015413	64221	13433
3538	EML3	-0.626	0.023	echinoderm microtubule associated protein like 3 [Source:HGNC Symbol;Acc:HGNC:266	chr11:62369689-62382592	NM_001300793, NN	XM_011544898, XM_0	NP_001287722, NP_00	XP_005273935, XP_0067	protein_coding	ENSG0000014949	256364	26666
3539	HIST1H3H	-1.189	0.023	histone cluster 1, H3h [Source:HGNC Symbol;Acc:HGNC:4775]	chr6:27777841-27779078	NM_003536	.	NP_003527	.	protein_coding	ENSG0000027882	8357	4775
3540	SUN2	-0.594	0.023	Sad1 and UNC84 domain containing 2 [Source:HGNC Symbol;Acc:HGNC:14210]	chr22:39130729-39190203	NM_001199579, NN	XM_011530105, XM_0	NP_001186508, NP_00	XP_011528406, XP_0115	protein_coding	ENSG0000010024	25777	14210
3541	ERCC4	0.817	0.023	ERCC excision repair 4, endonuclease catalytic subunit [Source:HGNC Symbol;Acc:HGNC	chr16:14014013-14046202	NM_005236	XM_011522424	NP_005227	XP_011520726	protein_coding	ENSG0000017559	2072	3436
3542	NES	-0.532	0.023	nestin [Source:HGNC Symbol;Acc:HGNC:7756]	chr1:156638554-156647189	NM_006617	.	NP_006608	.	protein_coding	ENSG0000013268	10763	7756
3543	SEH1L	0.581	0.023	SEH1 like nucleoporin [Source:HGNC Symbol;Acc:HGNC:30379]	chr18:12947993-12987535	NM_001013437, NN	XM_011525744, XM_0	NP_001013455, NP_11	XP_005258209, XP_0115	protein_coding	ENSG0000008541	81929	30379
3544	ULK2	0.880	0.023	unc-51 like autophagy activating kinase 2 [Source:HGNC Symbol;Acc:HGNC:13480]	chr17:19674142-19771249	NM_001142610, NN	.	NP_001136082, NP_05	XP_016880913, XP_0168	protein_coding	ENSG0000008329	9706	13480
3545	FBXL17	0.786	0.023	F-box and leucine rich repeat protein 17 [Source:HGNC Symbol;Acc:HGNC:13615]	chr5:107194735-107717799	NM_001163315	XM_005272048	NP_001156787	XP_005272105	protein_coding	ENSG0000014574	64839	13615
3546	GCLM	0.696	0.023	glutamate-cysteine ligase modifier subunit [Source:HGNC Symbol;Acc:HGNC:4312]	chr1:94350760-94375012	NM_002061	XM_011541261, XM_0	NP_001295182, NP_00	XP_005270811, XP_0115	protein_coding	ENSG0000002390	2730	4312
3547	LGALS9	-1.030	0.023	galectin 9 [Source:HGNC Symbol;Acc:HGNC:6570]	chr17:25956823-25976586	NM_002308, NM_0	XM_006721895, XM_0	NP_002299, NP_03366	XP_006721955, XP_0067	protein_coding	ENSG0000016896	3965	6570
3548	UBE2H	0.592	0.023	ubiquitin conjugating enzyme E2 H [Source:HGNC Symbol;Acc:HGNC:12484]	chr7:129470571-129592789	NM_001202498, NN	.	NP_001189427, NP_00	.	protein_coding	ENSG0000018659	7328	12484
3549	CASC4	0.712	0.024	cancer susceptibility candidate 4 [Source:HGNC Symbol;Acc:HGNC:24892]	chr15:44580928-44707956	NM_138423, NM_1	.	NP_612432, NP_81692	.	protein_coding	ENSG0000016673	113201	24892
3550	CIB1	-0.576	0.024	calcium and integrin binding 1 [Source:HGNC Symbol;Acc:HGNC:16920]	chr15:90773206-90789043	NM_001277764, NN	.	NP_001264693, NP_00	.	protein_coding	ENSG0000018504	10519	16920
3551	NAT16	-0.723	0.024	N-acetyltransferase 16 (putative) [Source:HGNC Symbol;Acc:HGNC:22030]	chr7:100813773-100823557	NM_198571	XM_011516169, XM_0	NP_940973	XP_005250382, XP_0115	protein_coding	ENSG0000016701	375607	22030
3552	ACTN1	-0.540	0.024	actinin alpha 1 [Source:HGNC Symbol;Acc:HGNC:163]	chr14:69340859-69446157	NM_001102, NM_0	XM_017021727, XM_0	NP_001093, NP_00112	XP_011535567, XP_0115	protein_coding	ENSG0000007211	87	163
3553	EIF2AK1	0.663	0.024	eukaryotic translation initiation factor 2 alpha kinase 1 [Source:HGNC Symbol;Acc:HGNC	chr7:6048875-6098861	NM_001134335, NN	.	NP_001127807, NP_05	.	protein_coding	ENSG0000008623	27102	24921
3554	MCFD2	0.640	0.024	multiple coagulation factor deficiency 2 [Source:HGNC Symbol;Acc:HGNC:18451]	chr2:47129008-47303274	NM_001171506, NN	.	NP_001164977, NP_00	.	protein_coding	ENSG0000018039	90411	18451
3555	UBA2	0.678	0.024	ubiquitin like modifier activating enzyme 2 [Source:HGNC Symbol;Acc:HGNC:30661]	chr19:34919267-34962156	NM_005499	XM_011526304, XM_0	NP_005490	XP_005258461, XP_0067	protein_coding	ENSG0000012626	10054	30661
3556	CBLC	-0.875	0.024	Cbl proto-oncogene C [Source:HGNC Symbol;Acc:HGNC:15961]	chr19:45281125-45303891	NM_001130852, NN	.	NP_001124324, NP_03	XP_005258753, XP_0115	protein_coding	ENSG0000014227	23624	15961
3557	POLR3D	0.680	0.024	RNA polymerase III subunit D [Source:HGNC Symbol;Acc:HGNC:1080]	chr8:22102616-22112113	NM_001722	.	NP_001713	.	protein_coding	ENSG0000016849	661	1080
3558	STRAP	0.548	0.024	serine/threonine kinase receptor associated protein [Source:HGNC Symbol;Acc:HGNC:3	chr12:16035327-16056412	NM_007178	XM_005253294	NP_009109	XP_005253351	protein_coding	ENSG0000002373	11171	30796
3559	DDX17	0.637	0.024	DEAD-box helicase 17 [Source:HGNC Symbol;Acc:HGNC:2740]	chr22:38864066-38902342	NM_001098504, NN	.	NP_001091974, NP_00	.	protein_coding	ENSG0000010020	10521	2740
3560	RPS6KC1	0.828	0.024	ribosomal protein S6 kinase C1 [Source:HGNC Symbol;Acc:HGNC:10439]	chr1:213224574-213448116	NM_001136138, NN	XM_017001033, XM_0	NP_001129610, NP_00	XP_005273152, XP_0052	protein_coding	ENSG0000013664	26750	10439
3561	SCARB2	0.646	0.024	scavenger receptor class B member 2 [Source:HGNC Symbol;Acc:HGNC:1665]	chr4:77079889-77135046	NM_001204255, NN	.	NP_001191184, NP_00	.	protein_coding	ENSG0000013876	950	1665
3562	TMEM163	-0.687	0.024	transmembrane protein 163 [Source:HGNC Symbol;Acc:HGNC:25380]	chr2:135213329-135476570	NM_030923	.	NP_112185	.	protein_coding	ENSG0000015212	81615	25380

3563	AAAS	-0.574	0.024	aladin WD repeat nucleoporin [Source:HGNC Symbol;Acc:HGNC:13666]	chr12:53701239-53718542	NM_001173466, N	XM_011538780, XM_0	NP_001166937, NP_05	XP_011537080, XP_0115	protein_coding	ENSG0000009491	8086	13666
3564	CNPPD1	-0.562	0.024	cyclin Pas1/PHO80 domain containing 1 [Source:HGNC Symbol;Acc:HGNC:25220]	chr2:220036618-220042828	NM_001321390, N	XM_006712419, XM_0	NP_001308318, NP_00	XP_005246519, XP_0052	protein_coding	ENSG0000011564	27013	25220
3565	RNF4	0.557	0.024	ring finger protein 4 [Source:HGNC Symbol;Acc:HGNC:10067]	chr4:2470663-2517582	NM_001185009, N	.	NP_001171938, NP_00	.	protein_coding	ENSG0000006397	6047	10067
3566	NUDCD1	1.333	0.024	NudC domain containing 1 [Source:HGNC Symbol;Acc:HGNC:24306]	chr8:110253147-110358181	NM_001128211, N	.	NP_001121683, NP_11	.	protein_coding	ENSG0000012052	84955	24306
3567	BRIX1	0.827	0.024	BRX1, biogenesis of ribosomes [Source:HGNC Symbol;Acc:HGNC:24170]	chr5:34905368-34926101	NM_018321	.	NP_060791	.	protein_coding	ENSG0000011346	55299	24170
3568	MFSD14C	-0.793	0.024	major facilitator superfamily domain containing 14C [Source:HGNC Symbol;Acc:HGNC:2	chr9:99705194-99775882	.	.	.	.	protein_coding	ENSG0000019631	.	23672
3569	RNF13	0.709	0.024	ring finger protein 13 [Source:HGNC Symbol;Acc:HGNC:10057]	chr3:149530494-149679926	NM_007282, NM_1	XM_017005656, XM_0	NP_009213, NP_89923	XP_005247149, XP_0115	protein_coding	ENSG0000008299	11342	10057
3570	RTKN	-0.545	0.024	rhotekin [Source:HGNC Symbol;Acc:HGNC:10466]	chr2:74652962-74669026	NM_001015055, N	XM_005264479, XM_0	NP_001015055, NP_00	XP_005264535, XP_0052	protein_coding	ENSG0000011499	6242	10466
3571	ITGAX	-1.348	0.024	integrin subunit alpha X [Source:HGNC Symbol;Acc:HGNC:6152]	chr16:31366454-31394318	NM_000887, NM_0	.	NP_000878, NP_00127	.	protein_coding	ENSG0000014067	3687	6152
3572	TSPAN10	-1.476	0.024	tetraspanin 10 [Source:HGNC Symbol;Acc:HGNC:29942]	chr17:79604223-79615778	NM_001290212, N	.	NP_001277141, NP_11	.	protein_coding	ENSG0000018261	83882	29942
3573	MAFF	-0.581	0.024	MAF bZIP transcription factor F [Source:HGNC Symbol;Acc:HGNC:6780]	chr22:38597888-38612518	NM_001161572, N	.	NP_001155044, NP_00	.	protein_coding	ENSG0000018502	23764	6780
3574	SPICE1	0.797	0.024	spindle and centriole associated protein 1 [Source:HGNC Symbol;Acc:HGNC:25083]	chr3:113161564-113234003	NM_144718	XM_011512461	NP_653319	XP_011510763, XP_0168	protein_coding	ENSG0000016361	152185	25083
3575	UCHL3	0.656	0.024	ubiquitin C-terminal hydrolase L3 [Source:HGNC Symbol;Acc:HGNC:12515]	chr13:76123615-76180156	NM_001270952, N	XM_017020727, XM_0	NP_001257881, NP_00	XP_011533514, XP_0115	protein_coding	ENSG0000011893	7347	12515
3576	LZTR1	-0.627	0.024	leucine zipper like transcription regulator 1 [Source:HGNC Symbol;Acc:HGNC:6742]	chr22:21336301-21353327	NM_006767	.	NP_006758	.	protein_coding	ENSG0000009994	8216	6742
3577	RUVBL1	-0.874	0.024	RuvB like AAA ATPase 1 [Source:HGNC Symbol;Acc:HGNC:10474]	chr3:127770483-128127489	NM_001319086, N	XM_011513250, XM_0	NP_001306015, NP_00	XP_005247898, XP_0115	protein_coding	ENSG0000017579	8607	10474
3578	FADD	-0.562	0.024	Fas associated via death domain [Source:HGNC Symbol;Acc:HGNC:3573]	chr11:70049268-70053496	NM_003824	.	NP_003815	.	protein_coding	ENSG0000016804	8772	3573
3579	KCTD3	0.700	0.024	potassium channel tetramerization domain containing 3 [Source:HGNC Symbol;Acc:HGN	chr1:215740734-215795149	NM_001319294, N	XM_005273158, XM_0	NP_001306223, NP_05	XP_005273215	protein_coding	ENSG0000013663	51133	21305
3580	KIF27	1.225	0.024	kinesin family member 27 [Source:HGNC Symbol;Acc:HGNC:18632]	chr9:86451612-86536380	NM_001271927, N	XM_011518855, XM_0	NP_001258856, NP_00	XP_011517150, XP_0115	protein_coding	ENSG0000016511	55582	18632
3581	MFSD8	1.303	0.024	major facilitator superfamily domain containing 8 [Source:HGNC Symbol;Acc:HGNC:284	chr4:128838959-128960866	NM_152778	XM_011531830, XM_0	NP_689991	XP_005262950, XP_0052	protein_coding	ENSG0000016407	256471	28486
3582	NT5C2	0.710	0.024	5'-nucleotidase, cytosolic II [Source:HGNC Symbol;Acc:HGNC:8022]	chr10:104678049-104953063	NM_001134373, N	XM_017015959, XM_0	NP_001127845, NP_03	XP_005269689, XP_0052	protein_coding	ENSG0000007668	22978	8022
3583	ZNF561	0.808	0.024	zinc finger protein 561 [Source:HGNC Symbol;Acc:HGNC:28684]	chr19:9718001-9732075	NM_152289	XM_017027482, XM_0	NP_689502	XP_005260207, XP_0052	protein_coding	ENSG0000017146	93134	28684
3584	ATF6B	-0.606	0.024	activating transcription factor 6 beta [Source:HGNC Symbol;Acc:HGNC:2349]	chr6:32083040-32096030	NM_001136153, N	.	NP_001129625, NP_00	.	protein_coding	ENSG0000021367	1388	2349
3585	GIPC1	-0.568	0.024	GIPC PDZ domain containing family member 1 [Source:HGNC Symbol;Acc:HGNC:1226]	chr19:14588571-14606961	NM_005716, NM_2	XM_017026147, XM_0	NP_005707, NP_97419	XP_011525945, XP_0168	protein_coding	ENSG0000012315	10755	1226
3586	FLYWCH1	-0.632	0.024	FLYWCH-type zinc finger 1 [Source:HGNC Symbol;Acc:HGNC:25404]	chr16:2961937-3001209	NM_001308068, N	XM_011522704, XM_0	NP_001294997, NP_06	XP_005255685, XP_0115	protein_coding	ENSG0000005912	84256	25404
3587	NHP2	-0.588	0.024	NHP2 ribonucleoprotein [Source:HGNC Symbol;Acc:HGNC:14377]	chr5:177557996-177580968	NM_001034833, N	.	NP_001030005, NP_06	.	protein_coding	ENSG0000014591	55651	14377
3588	UBXN8	0.996	0.024	UBX domain protein 8 [Source:HGNC Symbol;Acc:HGNC:30307]	chr8:30589763-30624522	NM_001282189, N	XM_011544655	NP_001269118, NP_00	XP_011542957	protein_coding	ENSG0000010469	7993	30307
3589	KIAA1715	0.803	0.024	lunapark, ER junction formation factor [Source:HGNC Symbol;Acc:HGNC:21610]	chr2:176788619-176867073	NM_001305008, N	XM_017005055, XM_0	NP_001291937, NP_00	XP_005246935, XP_0052	protein_coding	ENSG0000014432	80856	21610
3590	MYCN	-1.259	0.024	v-myc avian myelocytomatosis viral oncogene neuroblastoma derived homolog [Source	chr2:16080685-16087129	NM_001293228, N	XM_017004168	NP_001280157, NP_00	XP_016859657	protein_coding	ENSG0000013432	4613	7559
3591	RABGGTB	0.644	0.024	Rab geranylgeranyltransferase beta subunit [Source:HGNC Symbol;Acc:HGNC:9796]	chr1:76251885-76260764	NM_004582	.	NP_004573	.	protein_coding	ENSG0000013795	5876	9796
3592	TOR4A	-0.551	0.024	torsin family 4 member A [Source:HGNC Symbol;Acc:HGNC:25981]	chr9:140172200-140177093	NM_017723	.	NP_060193	.	protein_coding	ENSG0000019811	54863	25981
3593	F2R	1.021	0.025	coagulation factor II thrombin receptor [Source:HGNC Symbol;Acc:HGNC:3537]	chr5:76011867-76031606	NM_001992	.	NP_001983	.	protein_coding	ENSG0000018110	2149	3537
3594	HDAC2	0.590	0.025	histone deacetylase 2 [Source:HGNC Symbol;Acc:HGNC:4853]	chr6:114254191-114292449	NM_001527	XM_011535788, XM_0	NP_001518	XP_011534090, XP_0168	protein_coding	ENSG0000019659	3066	4853
3595	MXD1	0.768	0.025	MAX dimerization protein 1 [Source:HGNC Symbol;Acc:HGNC:6761]	chr2:70142172-70170077	NM_001202513, N	.	NP_001189442, NP_00	.	protein_coding	ENSG0000005972	4084	6761
3596	NOL10	0.671	0.025	nucleolar protein 10 [Source:HGNC Symbol;Acc:HGNC:25862]	chr2:10710891-10830113	NM_001261392, N	XM_011510399, XM_0	NP_001248321, NP_00	XP_011508700, XP_0115	protein_coding	ENSG0000011576	79954	25862
3597	PEMT	-0.679	0.025	phosphatidylethanolamine N-methyltransferase [Source:HGNC Symbol;Acc:HGNC:8830	chr17:17408876-17495022	NM_001267551, N	XM_006721418	NP_001254480, NP_00	XP_006721481	protein_coding	ENSG0000013302	10400	8830
3598	HOMER1	0.793	0.025	homer scaffolding protein 1 [Source:HGNC Symbol;Acc:HGNC:17512]	chr5:78668458-78810040	NM_001277077, N	.	NP_001264006, NP_00	.	protein_coding	ENSG0000015241	9456	17512
3599	APBA3	-0.678	0.025	amyloid beta precursor protein binding family A member 3 [Source:HGNC Symbol;Acc:H	chr19:3750877-3761697	NM_004886	XM_006722950	NP_004877	XP_006723013	protein_coding	ENSG0000001113	9546	580
3600	ATL3	0.683	0.025	atlastin GTPase 3 [Source:HGNC Symbol;Acc:HGNC:24526]	chr11:63391558-63439381	NM_001290048, N	XM_006718493	NP_001276977, NP_05	XP_006718556	protein_coding	ENSG0000018474	25923	24526
3601	PRKAB2	0.730	0.025	protein kinase AMP-activated non-catalytic subunit beta 2 [Source:HGNC Symbol;Acc:H	chr1:146626684-146644123	NM_005399	XM_011509729	NP_005390	XP_011508031	protein_coding	ENSG0000013179	5565	9379
3602	WNK2	-0.646	0.025	WNK lysine deficient protein kinase 2 [Source:HGNC Symbol;Acc:HGNC:14542]	chr9:95947211-96108696	NM_001282394, N	XM_011518940, XM_0	NP_001269323, NP_00	XP_005252194, XP_0052	protein_coding	ENSG0000016523	65268	14542
3603	APOL3	-0.806	0.025	apolipoprotein L3 [Source:HGNC Symbol;Acc:HGNC:14868]	chr22:36536377-36562225	NM_014349, NM_0	XM_017028952, XM_0	NP_055164, NP_08514	XP_006724386, XP_0067	protein_coding	ENSG0000012828	80833	14868
3604	PEX12	0.850	0.025	peroxisomal biogenesis factor 12 [Source:HGNC Symbol;Acc:HGNC:8854]	chr17:33901813-33905656	NM_000286	.	NP_000277	.	protein_coding	ENSG0000010873	5193	8854
3605	CDH1	0.616	0.025	cadherin 1 [Source:HGNC Symbol;Acc:HGNC:1748]	chr16:68771127-68869451	NM_001317185, N	XM_011523489, XM_0	NP_001304113, NP_00	XP_011521790, XP_0115	protein_coding	ENSG0000003906	999	1748
3606	HSPA9	0.514	0.025	heat shock protein family A (Hsp70) member 9 [Source:HGNC Symbol;Acc:HGNC:5244]	chr5:137890570-137911133	NM_004134	.	NP_004125	.	protein_coding	ENSG0000011301	3313	5244
3607	SPAG4	-0.752	0.025	sperm associated antigen 4 [Source:HGNC Symbol;Acc:HGNC:11214]	chr20:34203813-34208971	NM_001317931, N	XM_011529010, XM_0	NP_001304860, NP_00	XP_005260577, XP_0115	protein_coding	ENSG0000006165	6676	11214
3608	BBX	0.742	0.025	BBX, HMG-box containing [Source:HGNC Symbol;Acc:HGNC:14422]	chr3:107241782-107530171	NM_001142568, N	XM_011513010, XM_0	NP_001136040, NP_00	XP_005247699, XP_0052	protein_coding	ENSG0000011443	56987	14422
3609	HMGCS1	0.619	0.025	3-hydroxy-3-methylglutaryl-CoA synthase 1 [Source:HGNC Symbol;Acc:HGNC:5007]	chr5:43289496-43313614	NM_001098272, N	XM_017009412, XM_0	NP_001091742, NP_00	XP_011512336, XP_0168	protein_coding	ENSG0000011297	3157	5007
3610	OGG1	-1.290	0.025	8-oxoguanine DNA glycosylase [Source:HGNC Symbol;Acc:HGNC:8125]	chr3:9791627-9811661	NM_002542, NM_0	.	NP_002533, NP_05821	XP_011532062, XP_0168	protein_coding	ENSG0000011402	4968	8125
3611	RBCK1	-0.593	0.025	RANBP2-type and C3HC4-type zinc finger containing 1 [Source:HGNC Symbol;Acc:HGNC	chr20:388696-411610	NM_001323960, N	XM_011529138, XM_0	NP_001310885, NP_00	XP_005260702, XP_0115	protein_coding	ENSG0000012582	10616	15864
3612	ZNF248	1.087	0.025	zinc finger protein 248 [Source:HGNC Symbol;Acc:HGNC:13041]	chr10:38117692-38147012	NM_001267597, N	XM_017016446, XM_0	NP_001254526, NP_00	XP_006717548, XP_0115	protein_coding	ENSG0000019810	57209	13041
3613	EDEM2	-0.612	0.025	ER degradation enhancing alpha-mannosidase like protein 2 [Source:HGNC Symbol;Acc:	chr20:33703159-33735167	NM_001145025, N	.	NP_001138497, NP_06	.	protein_coding	ENSG0000008829	55741	15877
3614	PLEKHF2	0.843	0.025	pleckstrin homology and FYVE domain containing 2 [Source:HGNC Symbol;Acc:HGNC:20	chr8:96146031-96168912	NM_024613	.	NP_078889	.	protein_coding	ENSG0000017589	79666	20757
3615	RPLP0	-0.575	0.025	ribosomal protein lateral stalk subunit P0 [Source:HGNC Symbol;Acc:HGNC:10371]	chr12:120634488-120639014	NM_001002, NM_0	.	NP_000993, NP_44450	.	protein_coding	ENSG0000008915	6175	10371
3616	HS2ST1	0.950	0.025	heparan sulfate 2-O-sulfotransferase 1 [Source:HGNC Symbol;Acc:HGNC:5193]	chr1:87380330-87634887	NM_001134492, N	.	NP_001127964, NP_03	.	protein_coding	ENSG0000015393	9653	5193

3617	LINGO1	-0.934	0.025	leucine rich repeat and Ig domain containing 1 [Source:HGNC Symbol;Acc:HGNC:21205]	chr15:77905368-77988490	NM_001301186, NN	XM_011522118, XM_0	NP_001288115, NP_00	XP_011520419, XP_0115	protein_coding	ENSG0000016978	84894	21205
3618	SDHA	-0.531	0.025	succinate dehydrogenase complex flavoprotein subunit A [Source:HGNC Symbol;Acc:HG	chr5:218355-256815	NM_001294332, NN	XM_011514073, XM_0	NP_001281261, NP_00	XP_005248388, XP_0115	protein_coding	ENSG0000007357	6389	10680
3619	BTD	-1.190	0.025	biotinidase [Source:HGNC Symbol;Acc:HGNC:1122]	chr3:15602210-15687329	NM_000060, NM_0	XM_011534041, XM_0	NP_000051, NP_00126	XP_006713377, XP_0115	protein_coding	ENSG0000016981	686	1122
3620	CCDC174	-0.729	0.025	coiled-coil domain containing 174 [Source:HGNC Symbol;Acc:HGNC:28033]	chr3:14693252-14714166	NM_016474	.	NP_057558	XP_016862043	protein_coding	ENSG0000015478	51244	28033
3621	DMTF1	1.776	0.025	cyclin D binding myb like transcription factor 1 [Source:HGNC Symbol;Acc:HGNC:14603]	chr7:86781676-86849903	NM_001142326, NN	XM_017012874, XM_0	NP_001135798, NP_00	XP_005250791, XP_0067	protein_coding	ENSG0000013516	9988	14603
3622	PHACTR4	0.672	0.025	phosphatase and actin regulator 4 [Source:HGNC Symbol;Acc:HGNC:25793]	chr1:28696113-28826881	NM_001048183, NN	XM_011542030, XM_0	NP_001041648, NP_07	XP_005246027, XP_0168	protein_coding	ENSG0000020413	65979	25793
3623	ETAA1	0.835	0.025	Ewing tumor associated antigen 1 [Source:HGNC Symbol;Acc:HGNC:24648]	chr2:67624450-67637677	NM_019002	XM_005264378, XM_0	NP_061875	.	protein_coding	ENSG0000014397	54465	24648
3624	ENDOD1	0.567	0.025	endonuclease domain containing 1 [Source:HGNC Symbol;Acc:HGNC:29129]	chr11:94822973-94865809	NM_015036	.	NP_055851	.	protein_coding	ENSG0000014921	23052	29129
3625	C6orf226	-0.931	0.025	chromosome 6 open reading frame 226 [Source:HGNC Symbol;Acc:HGNC:34431]	chr6:42858002-42858554	NM_001008739	.	NP_001008739	.	protein_coding	ENSG0000022182	441150	34431
3626	NOL8	1.181	0.025	nucleolar protein 8 [Source:HGNC Symbol;Acc:HGNC:23387]	chr9:95059639-95432547	NM_001256394, NN	XM_011518827, XM_0	NP_001243323, NP_06	XP_006717229, XP_0067	protein_coding	ENSG0000019800	55035	23387
3627	SNX17	-0.545	0.025	sorting nexin 17 [Source:HGNC Symbol;Acc:HGNC:14979]	chr2:27593362-27603592	NM_001267059, NN	XM_011533203	NP_001253988, NP_00	XP_011531505	protein_coding	ENSG0000011523	9784	14979
3628	UCN2	-1.266	0.025	urocortin 2 [Source:HGNC Symbol;Acc:HGNC:18414]	chr3:48599159-48601206	NM_033199	.	NP_149976	.	protein_coding	ENSG0000014504	90226	18414
3629	BZW2	0.542	0.025	basic leucine zipper and W2 domains 2 [Source:HGNC Symbol;Acc:HGNC:18808]	chr7:16685755-16746148	NM_001159767, NN	XM_006715708, XM_0	NP_001153239, NP_05	XP_006715769, XP_0067	protein_coding	ENSG0000013626	28969	18808
3630	PARP10	-0.653	0.025	poly(ADP-ribose) polymerase family member 10 [Source:HGNC Symbol;Acc:HGNC:2589	chr8:145051320-145060628	NM_001317895, NN	XM_011517336, XM_0	NP_001304824, NP_11	XP_005251138, XP_0115	protein_coding	ENSG0000017868	84875	25895
3631	SELO	-0.627	0.025	selenoprotein O [Source:EntrezGene;Acc:83642]	chr22:50639407-50656045	NM_031454	.	NP_113642	.	protein_coding	ENSG0000007316	83642	.
3632	SLC35B4	0.931	0.025	solute carrier family 35 member B4 [Source:HGNC Symbol;Acc:HGNC:20584]	chr7:133974083-134001803	NM_032826	.	NP_116215	XP_011514947	protein_coding	ENSG0000020506	84912	20584
3633	IMPA2	-0.625	0.025	inositol monophosphatase 2 [Source:HGNC Symbol;Acc:HGNC:6051]	chr18:11981035-12030882	NM_014214	XM_011525661	NP_055029	XP_011523963	protein_coding	ENSG0000014140	3613	6051
3634	MAN2C1	-0.767	0.025	mannosidase alpha class 2C member 1 [Source:HGNC Symbol;Acc:HGNC:6827]	chr15:75639330-75660971	NM_001256494, NN	XM_011521569, XM_0	NP_001243423, NP_00	XP_005254441, XP_0168	protein_coding	ENSG0000014040	693216	6827
3635	CALR	-0.545	0.025	calreticulin [Source:HGNC Symbol;Acc:HGNC:1455]	chr19:13049420-13055303	NM_004343	.	NP_004334	.	protein_coding	ENSG0000017921	811	1455
3636	CDIPT	-0.603	0.025	CDP-diacylglycerol--inositol 3-phosphatidyltransferase [Source:HGNC Symbol;Acc:HGNC	chr16:29869677-29875057	NM_001286585, NN	XM_005255038	NP_001273514, NP_00	XP_005255095	protein_coding	ENSG0000010350	10423	1769
3637	ANG	-1.514	0.026	angiogenin [Source:HGNC Symbol;Acc:HGNC:483]	chr14:21152258-21169374	NM_001097577, NN	.	NP_001091046, NP_00	.	protein_coding	ENSG0000021427	283	483
3638	COPG2	0.739	0.026	coatomer protein complex subunit gamma 2 [Source:HGNC Symbol;Acc:HGNC:2237]	chr7:130295819-130372268	NM_001290033, NN	.	NP_001276962, NP_03	.	protein_coding	ENSG0000015862	26958	2237
3639	MDM1	0.979	0.026	Mdm1 nuclear protein [Source:HGNC Symbol;Acc:HGNC:29917]	chr12:68688347-68726161	NM_001205028, NN	XM_005269027, XM_0	NP_001191957, NP_00	XP_005269083	protein_coding	ENSG0000011155	56890	29917
3640	ZXDC	-0.646	0.026	ZXD family zinc finger C [Source:HGNC Symbol;Acc:HGNC:28160]	chr3:126156443-126194762	NM_001040653, NN	XM_011513119	NP_001035743, NP_07	XP_011511421	protein_coding	ENSG0000007047	79364	28160
3641	MFSD12	-0.560	0.026	major facilitator superfamily domain containing 12 [Source:HGNC Symbol;Acc:HGNC:28	chr19:3539151-3544028	NM_001287529, NN	.	NP_001274458, NP_77	XP_011525986	protein_coding	ENSG0000016109	126321	28299
3642	STX4	-0.613	0.026	syntaxin 4 [Source:HGNC Symbol;Acc:HGNC:11439]	chr16:31044415-31051489	NM_001272095, NN	XM_005255521	NP_001259024, NP_00	XP_005255578	protein_coding	ENSG0000010349	6810	11439
3643	USP48	0.746	0.026	ubiquitin specific peptidase 48 [Source:HGNC Symbol;Acc:HGNC:18533]	chr1:22004790-22110099	NM_001032730, NN	XM_011542269, XM_0	NP_001027902, NP_11	XP_005246066, XP_0067	protein_coding	ENSG0000009068	84196	18533
3644	CYB5B	-0.561	0.026	cytochrome b5 type B [Source:HGNC Symbol;Acc:HGNC:24374]	chr16:69458427-69500169	NM_030579	.	NP_085056	.	protein_coding	ENSG0000010301	80777	24374
3645	NRBF2	0.725	0.026	nuclear receptor binding factor 2 [Source:HGNC Symbol;Acc:HGNC:19692]	chr10:64893006-64914791	NM_001282405, NN	.	NP_001269334, NP_11	.	protein_coding	ENSG0000014857	29982	19692
3646	RHOD	-0.570	0.026	ras homolog family member D [Source:HGNC Symbol;Acc:HGNC:670]	chr11:66824288-66839484	NM_001300886, NN	.	NP_001287815, NP_05	.	protein_coding	ENSG0000017315	29984	670
3647	MSMO1	0.639	0.026	methylsterol monooxygenase 1 [Source:HGNC Symbol;Acc:HGNC:10545]	chr4:166248774-166264312	NM_001017369, NN	XM_005263176	NP_001017369, NP_00	XP_005263233	protein_coding	ENSG0000005280	6307	10545
3648	ARHGEF4	-0.734	0.026	Rho guanine nucleotide exchange factor 4 [Source:HGNC Symbol;Acc:HGNC:684]	chr2:131594488-131804836	NM_015320, NM_0	XM_011511275, XM_0	NP_056135, NP_12746	XP_005263738, XP_0052	protein_coding	ENSG0000013600	50649	684
3649	FAN1	0.808	0.026	FANCD2/FANCI-associated nuclease 1 [Source:HGNC Symbol;Acc:HGNC:29170]	chr15:31196054-31283807	NM_001146094, NN	XM_011521371, XM_0	NP_001139566, NP_00	XP_005254289, XP_0052	protein_coding	ENSG0000019869	22909	29170
3650	FUOM	-0.774	0.026	fucose mutarotase [Source:HGNC Symbol;Acc:HGNC:24733]	chr10:135168657-135171539	NM_001098483, NN	XM_011539674, XM_0	NP_001091953, NP_00	XP_011537972, XP_0115	protein_coding	ENSG0000014880	282969	24733
3651	MED25	-0.609	0.026	mediator complex subunit 25 [Source:HGNC Symbol;Acc:HGNC:28845]	chr19:50321535-50340237	NM_030973	XM_011527353	NP_112235	XP_011525655	protein_coding	ENSG0000010497	102465480	28845
3652	PPP6C	0.612	0.026	protein phosphatase 6 catalytic subunit [Source:HGNC Symbol;Acc:HGNC:9323]	chr9:127908851-127952218	NM_001123355, NN	XM_011518847	NP_001116827, NP_00	XP_011517149	protein_coding	ENSG0000011941	5537	9323
3653	EFNB2	0.769	0.026	ephrin B2 [Source:HGNC Symbol;Acc:HGNC:3227]	chr13:107142078-107187462	NM_004093	.	NP_004084	.	protein_coding	ENSG0000012526	1948	3227
3654	FBXO46	-0.631	0.026	F-box protein 46 [Source:HGNC Symbol;Acc:HGNC:25069]	chr19:46213886-46234162	NM_001080469, NN	.	NP_001073938, NP_00	.	protein_coding	ENSG0000017705	23403	25069
3655	MSRB3	0.957	0.026	methionine sulfoxide reductase B3 [Source:HGNC Symbol;Acc:HGNC:27375]	chr12:65672422-65860687	NM_001031679, NN	.	NP_001026849, NP_00	.	protein_coding	ENSG0000017409	253827	27375
3656	NMT2	0.661	0.026	N-myristoyltransferase 2 [Source:HGNC Symbol;Acc:HGNC:7858]	chr10:15149864-15210692	NM_001308295, NN	XM_006717539, XM_0	NP_001295224, NP_00	XP_005252699, XP_0052	protein_coding	ENSG0000015246	9397	7858
3657	PLEKHO1	-0.704	0.026	pleckstrin homology domain containing O1 [Source:HGNC Symbol;Acc:HGNC:24310]	chr1:150121372-150132260	NM_001304724, NN	.	NP_001291653, NP_05	.	protein_coding	ENSG0000002390	51177	24310
3658	DNAJC2	0.988	0.026	DnaJ heat shock protein family (Hsp40) member C2 [Source:HGNC Symbol;Acc:HGNC:1	chr7:102937868-102985320	NM_001129887, NN	XM_011516029, XM_0	NP_001123359, NP_05	XP_005250326, XP_0115	protein_coding	ENSG0000010582	27000	13192
3659	PDIA4	0.537	0.026	protein disulfide isomerase family A member 4 [Source:HGNC Symbol;Acc:HGNC:30167	chr7:148700153-148725733	NM_004911	XM_006716185	NP_004902	XP_006716248	protein_coding	ENSG0000015566	9601	30167
3660	ZKSCAN2	0.810	0.026	zinc finger with KRAB and SCAN domains 2 [Source:HGNC Symbol;Acc:HGNC:25677]	chr16:25247321-25269252	NM_001012981	XM_017023200	NP_001012999	XP_016878689	protein_coding	ENSG0000015559	342357	25677
3661	RXFP4	-1.310	0.026	relaxin/insulin like family peptide receptor 4 [Source:HGNC Symbol;Acc:HGNC:14666]	chr1:155911500-155912740	NM_181885	.	NP_871001	.	protein_coding	ENSG0000017308	339403	14666
3662	ABLIM1	0.530	0.026	actin binding LIM protein 1 [Source:HGNC Symbol;Acc:HGNC:78]	chr10:116190871-116444762	NM_001003407, NN	XM_017016255, XM_0	NP_001003407, NP_00	XP_005269875, XP_0052	protein_coding	ENSG0000009920	3983	78
3663	SCRIB	-0.547	0.026	scribbled planar cell polarity protein [Source:HGNC Symbol;Acc:HGNC:30377]	chr8:144873089-144897549	NM_015356, NM_1	.	NP_056171, NP_87436	.	protein_coding	ENSG0000018090	100126338	30377
3664	BABAM1	-0.564	0.026	BRISC and BRCA1 A complex member 1 [Source:HGNC Symbol;Acc:HGNC:25008]	chr19:17378158-17390146	NM_001033549, NN	.	NP_001028721, NP_00	.	protein_coding	ENSG0000010539	29086	25008
3665	DVL3	-0.631	0.026	dishevelled segment polarity protein 3 [Source:HGNC Symbol;Acc:HGNC:3087]	chr3:183873175-183891398	NM_004423	XM_005247172	NP_004414	XP_005247229	protein_coding	ENSG0000016120	1857	3087
3666	ERAP2	1.365	0.026	endoplasmic reticulum aminopeptidase 2 [Source:HGNC Symbol;Acc:HGNC:29499]	chr5:96211642-96255420	NM_001130140, NN	XM_011543544	NP_001123612, NP_00	XP_011541846	protein_coding	ENSG0000016430	64167	29499
3667	ICA1	0.668	0.026	islet cell autoantigen 1 [Source:HGNC Symbol;Acc:HGNC:5343]	chr7:8152813-8302242	NM_001136020, NN	XM_017012125, XM_0	NP_001129492, NP_00	XP_005249792, XP_0115	protein_coding	ENSG0000000314	3382	5343
3668	RPL8	-0.570	0.026	ribosomal protein L8 [Source:HGNC Symbol;Acc:HGNC:10368]	chr8:146015153-146017972	NM_000973, NM_0	XM_011517210	NP_000964, NP_00130	XP_011515512	protein_coding	ENSG0000016101	102465978	10368
3669	NPAS1	-0.724	0.026	neuronal PAS domain protein 1 [Source:HGNC Symbol;Acc:HGNC:7894]	chr19:47523076-47549033	NM_001321086, NN	XM_011527007, XM_0	NP_001308015, NP_00	XP_006723294, XP_0115	protein_coding	ENSG0000013075	4861	7894
3670	TIMM50	-0.549	0.026	translocase of inner mitochondrial membrane 50 [Source:HGNC Symbol;Acc:HGNC:2365	chr19:39971051-39981528	NM_001001563	XM_011527491	NP_001001563	XP_011525793	protein_coding	ENSG0000010519	92609	23656

3671	CAB39	0.610	0.026	calcium binding protein 39 [Source:HGNC Symbol;Acc:HGNC:20292]	chr2:231577559-231685790	NM_001130849, NN	.	NP_001124321, NP_00	.	protein_coding	ENSG0000013593	51719	20292
3672	FCF1	0.803	0.026	FCF1 rRNA-processing protein [Source:HGNC Symbol;Acc:HGNC:20220]	chr14:75179846-75205323	NM_015962	XM_011536816, XM_0	NP_001305437, NP_05	XP_005267787, XP_0115	protein_coding	ENSG0000011961	51077	20220
3673	PDPK1	0.644	0.026	3-phosphoinositide dependent protein kinase 1 [Source:HGNC Symbol;Acc:HGNC:8816]	chr16:2587964-2653189	NM_001261816, NN	XM_011522523, XM_0	NP_001248745, NP_00	XP_005255413, XP_0115	protein_coding	ENSG0000014099	5170	8816
3674	PIAS4	-0.688	0.026	protein inhibitor of activated STAT 4 [Source:HGNC Symbol;Acc:HGNC:17002]	chr19:4007643-4039384	NM_015897	XM_017026868, XM_0	NP_056981	XP_011526362, XP_0168	protein_coding	ENSG0000010522	51588	17002
3675	SLC25A48	-1.375	0.026	solute carrier family 25 member 48 [Source:HGNC Symbol;Acc:HGNC:30451]	chr5:135170337-135224326	NM_145282	XM_006714546	NP_660325	XP_006714609, XP_0168	protein_coding	ENSG0000014583	153328	30451
3676	ZC3H7B	-0.522	0.026	zinc finger CCCH-type containing 7B [Source:HGNC Symbol;Acc:HGNC:30869]	chr22:41697525-41756151	NM_017590	.	NP_060060	.	protein_coding	ENSG0000010040	23264	30869
3677	SEC31A	0.664	0.026	SEC31 homolog A, COPII coat complex component [Source:HGNC Symbol;Acc:HGNC:170	chr4:83739813-83821691	NM_001077206, NN	XM_011531749, XM_0	NP_001070674, NP_00	XP_005262907	protein_coding	ENSG0000013867	22872	17052
3678	CCL24	-1.052	0.026	C-C motif chemokine ligand 24 [Source:HGNC Symbol;Acc:HGNC:10623]	chr7:75440982-75452674	NM_002991	XM_011516460, XM_0	NP_002982	XP_011514761, XP_0115	protein_coding	ENSG0000010617	6369	10623
3679	HBS1L	0.816	0.026	HBS1 like translational GTPase [Source:HGNC Symbol;Acc:HGNC:4834]	chr6:135281515-135376036	NM_001145158, NN	.	NP_001138630, NP_00	.	protein_coding	ENSG0000011233	10767	4834
3680	MRAS	-0.970	0.026	muscle RAS oncogene homolog [Source:HGNC Symbol;Acc:HGNC:7227]	chr3:138066489-138124376	NM_001085049, NN	XM_005247229, XM_0	NP_001078518, NP_00	XP_005247285, XP_0052	protein_coding	ENSG0000015818	22808	7227
3681	MTR	0.666	0.026	5-methyltetrahydrofolate-homocysteine methyltransferase [Source:HGNC Symbol;Acc:H	chr1:236958580-237067281	NM_000254, NM_0	XM_011544194, XM_0	NP_000245, NP_00127	XP_005273198, XP_0067	protein_coding	ENSG0000011698	4548	7468
3682	ETV1	0.850	0.026	ETS variant 1 [Source:HGNC Symbol;Acc:HGNC:3490]	chr7:13930852-14031050	NM_001163147, NN	XM_011515172, XM_0	NP_001156619, NP_00	XP_005249692, XP_0052	protein_coding	ENSG0000000646	2115	3490
3683	GEN1	1.332	0.026	GEN1, Holliday junction 5' flap endonuclease [Source:HGNC Symbol;Acc:HGNC:26881]	chr2:17845078-17981462	NM_001130009, NN	XM_011532822, XM_0	NP_001123481, NP_87	XP_005262670, XP_0067	protein_coding	ENSG0000017829	348654	26881
3684	SMU1	0.595	0.026	DNA replication regulator and spliceosomal factor [Source:HGNC Symbol;Acc:HGNC:182	chr9:33041761-33076657	NM_018225	XM_005251503	NP_060695	XP_005251560	protein_coding	ENSG0000012269	55234	18247
3685	ACTR3	0.722	0.027	ARP3 actin related protein 3 homolog [Source:HGNC Symbol;Acc:HGNC:170]	chr2:114647536-114720173	NM_001277140, NN	.	NP_001264069, NP_00	.	protein_coding	ENSG0000011509	10096	170
3686	TIGD5	-0.721	0.027	tigger transposable element derived 5 [Source:HGNC Symbol;Acc:HGNC:18336]	chr8:144680004-144685394	NM_032862	.	NP_116251	.	protein_coding	ENSG0000017988	84948	18336
3687	STX2	0.739	0.027	syntaxin 2 [Source:HGNC Symbol;Acc:HGNC:3403]	chr12:131274144-131323811	NM_001980, NM_1	XM_017018980, XM_0	NP_001971, NP_91933	XP_005253613, XP_0052	protein_coding	ENSG0000011145	2054	3403
3688	KIF22	-0.523	0.027	kinesin family member 22 [Source:HGNC Symbol;Acc:HGNC:6391]	chr16:29802039-29816706	NM_001256269, NN	.	NP_001243198, NP_00	.	protein_coding	ENSG0000007961	3835	6391
3689	LRRC8E	-0.682	0.027	leucine rich repeat containing 8 family member E [Source:HGNC Symbol;Acc:HGNC:2627	chr19:7953389-7966906	NM_001268284, NN	XM_011528319	NP_001255213, NP_07	XP_011526621	protein_coding	ENSG0000017101	80131	26272
3690	PDK2	-0.664	0.027	pyruvate dehydrogenase kinase 2 [Source:HGNC Symbol;Acc:HGNC:8810]	chr17:48172100-48207246	NM_001199898, NN	.	NP_001186827, NP_00	.	protein_coding	ENSG0000000588	5164	8810
3691	AKAP12	-0.554	0.027	A-kinase anchoring protein 12 [Source:HGNC Symbol;Acc:HGNC:370]	chr6:151561133-151679692	NM_005100, NM_1	XM_017011517, XM_0	NP_005091, NP_65308	XP_005267290, XP_0052	protein_coding	ENSG0000013101	9590	370
3692	FOXQ1	-0.571	0.027	forkhead box Q1 [Source:HGNC Symbol;Acc:HGNC:20951]	chr6:1312674-1314983	NM_033260	.	NP_150285	.	protein_coding	ENSG0000016437	94234	20951
3693	LIAS	0.771	0.027	lipoic acid synthetase [Source:HGNC Symbol;Acc:HGNC:16429]	chr4:39460619-39479273	NM_001278590, NN	.	NP_001265519, NP_00	.	protein_coding	ENSG0000012189	11019	16429
3694	PALLD	0.702	0.027	palladin, cytoskeletal associated protein [Source:HGNC Symbol;Acc:HGNC:17068]	chr4:169418216-169849608	NM_001166108, NN	XM_011531776, XM_0	NP_001159580, NP_00	XP_005262918, XP_0052	protein_coding	ENSG0000012911	23022	17068
3695	PARP14	0.699	0.027	poly(ADP-ribose) polymerase family member 14 [Source:HGNC Symbol;Acc:HGNC:2923	chr3:122399464-122449687	NM_017554	XM_011512928	NP_060024	XP_011511230	protein_coding	ENSG0000017319	54625	29232
3696	RCN1	0.520	0.027	reticulocalbin 1 [Source:HGNC Symbol;Acc:HGNC:9934]	chr11:31806339-32127301	NM_002901	.	NP_002892	.	protein_coding	ENSG0000004944	440034	9934
3697	REEP6	-0.691	0.027	receptor accessory protein 6 [Source:HGNC Symbol;Acc:HGNC:30078]	chr19:1490745-1497926	NM_138393	.	NP_612402	.	protein_coding	ENSG0000011525	92840	30078
3698	WDR12	0.583	0.027	WD repeat domain 12 [Source:HGNC Symbol;Acc:HGNC:14098]	chr2:203739504-203851786	NM_018256	XM_011511469	NP_060726	XP_011509771	protein_coding	ENSG0000013844	55759	14098
3699	ADK	0.690	0.027	adenosine kinase [Source:HGNC Symbol;Acc:HGNC:257]	chr10:75910942-76469061	NM_001123, NM_0	XM_011539297	NP_001114, NP_00118	XP_016871190	protein_coding	ENSG0000015611	132	257
3700	LRRC3	-0.711	0.027	leucine rich repeat containing 3 [Source:HGNC Symbol;Acc:HGNC:14965]	chr21:45875368-45882079	NM_030891	.	NP_112153	.	protein_coding	ENSG0000016023	81543	14965
3701	ATG7	-1.003	0.027	autophagy related 7 [Source:HGNC Symbol;Acc:HGNC:16935]	chr3:11314101-11762220	NM_001136031, NN	XM_011533286, XM_0	NP_001129503, NP_00	XP_006712994, XP_0067	protein_coding	ENSG0000019754	10533	16935
3702	EPS8L2	-0.559	0.027	EPS8 like 2 [Source:HGNC Symbol;Acc:HGNC:21296]	chr11:706112-727727	NM_022772	XM_017018132, XM_0	NP_073609	XP_016873619, XP_0168	protein_coding	ENSG0000017710	64787	21296
3703	FAM189A1	0.882	0.027	family with sequence similarity 189 member A1 [Source:HGNC Symbol;Acc:HGNC:29075	chr15:29412456-29862927	NM_015307	XM_011521407	NP_056122	XP_011519709	protein_coding	ENSG0000010405	23359	29075
3704	LARS2	-0.592	0.027	leucyl-tRNA synthetase 2, mitochondrial [Source:HGNC Symbol;Acc:HGNC:17095]	chr3:45429997-45590913	NM_015340	XM_011533554, XM_0	NP_056155	XP_005265063, XP_0115	protein_coding	ENSG0000001137	23395	17095
3705	MIER2	-0.624	0.027	MIER family member 2 [Source:HGNC Symbol;Acc:HGNC:29210]	chr19:301443-344782	NM_017550	XM_011528080, XM_0	NP_060020	XP_005259639, XP_0067	protein_coding	ENSG0000010555	54531	29210
3706	ZNF532	0.830	0.027	zinc finger protein 532 [Source:HGNC Symbol;Acc:HGNC:30940]	chr18:56529831-56653712	NM_001318726, NN	XM_011526061, XM_0	NP_001305655, NP_00	XP_005266780, XP_0052	protein_coding	ENSG0000007465	55205	30940
3707	C18orf25	0.746	0.027	chromosome 18 open reading frame 25 [Source:HGNC Symbol;Acc:HGNC:28172]	chr18:43753992-43846954	NM_001008239, NN	XM_017025555, XM_0	NP_001008240, NP_65	XP_011524124, XP_0168	protein_coding	ENSG0000015224	147339	28172
3708	DOC2B	-5.464	0.027	double C2 domain beta [Source:HGNC Symbol;Acc:HGNC:2986]	chr17:6010-31427	NM_003585	XM_011535363	NP_003576	XP_011533665	protein_coding	ENSG0000027263	8447	2986
3709	ETV2	-0.973	0.027	ETS variant 2 [Source:HGNC Symbol;Acc:HGNC:3491]	chr19:36132646-36135773	NM_001300974, NN	XM_011526624, XM_0	NP_001287903, NP_00	XP_005258709, XP_0115	protein_coding	ENSG0000010567	2116	3491
3710	GPX3	0.684	0.027	glutathione peroxidase 3 [Source:HGNC Symbol;Acc:HGNC:4555]	chr5:150399998-150408554	NM_002084	.	NP_002075	.	protein_coding	ENSG0000021144	2878	4555
3711	PTH2	-2.826	0.027	parathyroid hormone 2 [Source:HGNC Symbol;Acc:HGNC:30828]	chr19:49925670-49926698	NM_178449	.	NP_848544	.	protein_coding	ENSG0000014253	113091	30828
3712	ROR1	0.991	0.027	receptor tyrosine kinase like orphan receptor 1 [Source:HGNC Symbol;Acc:HGNC:10256	chr1:64239692-64647181	NM_001083592, NN	XM_017001376, XM_0	NP_001077061, NP_00	XP_011539828, XP_0168	protein_coding	ENSG0000018548	4919	10256
3713	SREK1IP1	0.757	0.027	SREK1 interacting protein 1 [Source:HGNC Symbol;Acc:HGNC:26716]	chr5:64013970-64064512	NM_173829	XM_011543344	NP_776190	XP_011541646	protein_coding	ENSG0000015300	285672	26716
3714	HK1	-0.515	0.027	hexokinase 1 [Source:HGNC Symbol;Acc:HGNC:4922]	chr10:71029755-71161638	NM_000188, NM_0	XM_011539734, XM_0	NP_000179, NP_00130	XP_005269793, XP_0052	protein_coding	ENSG0000015651	3098	4922
3715	ECE1	-0.511	0.027	endothelin converting enzyme 1 [Source:HGNC Symbol;Acc:HGNC:3146]	chr1:21543739-21671997	NM_001113347, NN	XM_017000511, XM_0	NP_001106818, NP_00	XP_006710461, XP_0115	protein_coding	ENSG0000011729	1889	3146
3716	SLC7A6	0.766	0.027	solute carrier family 7 member 6 [Source:HGNC Symbol;Acc:HGNC:11064]	chr16:68298418-68344848	NM_001076785, NN	XM_011523439, XM_0	NP_001070253, NP_00	XP_011521735, XP_0115	protein_coding	ENSG0000010306	9057	11064
3717	TIMM17A	0.596	0.027	translocase of inner mitochondrial membrane 17 homolog A (yeast) [Source:HGNC Sym	chr1:201924618-201939789	NM_006335	.	NP_006326	.	protein_coding	ENSG0000013437	10440	17315
3718	TMEM129	-0.566	0.027	transmembrane protein 129 [Source:HGNC Symbol;Acc:HGNC:25137]	chr4:1717678-1723085	NM_001127266, NN	.	NP_001120738, NP_61	.	protein_coding	ENSG0000016893	92305	25137
3719	CECR5	-0.576	0.027	cat eye syndrome chromosome region, candidate 5 [Source:HGNC Symbol;Acc:HGNC:18	chr22:17618400-17646177	NM_017829, NM_0	XM_011546126, XM_0	NP_060299, NP_14906	XP_005261293, XP_0115	protein_coding	ENSG0000006999	27440	1843
3720	GON4L	0.685	0.027	gon-4 like [Source:HGNC Symbol;Acc:HGNC:25973]	chr1:155719452-155829191	NM_001282856, NN	XM_011509659, XM_0	NP_001269785, NP_00	XP_005245341, XP_0067	protein_coding	ENSG0000011658	54856	25973
3721	RNLS	1.144	0.027	renalase, FAD dependent amine oxidase [Source:HGNC Symbol;Acc:HGNC:25641]	chr10:90033620-90344287	NM_001031709, NN	XM_011539925, XM_0	NP_001026879, NP_06	XP_005270003, XP_0052	protein_coding	ENSG0000018471	55328	25641
3722	TECR	-0.534	0.027	trans-2,3-enoyl-CoA reductase [Source:HGNC Symbol;Acc:HGNC:4551]	chr19:14640371-14676792	NM_001321170, NN	XM_011528442, XM_0	NP_001308099, NP_61	XP_006723008, XP_0115	protein_coding	ENSG0000009979	693224	4551
3723	ERCC6	0.883	0.027	ERCC excision repair 6, chromatin remodeling factor [Source:HGNC Symbol;Acc:HGNC:3	chr10:50663413-50747584	NM_000124, NM_0	.	NP_000115, NP_00126	.	protein_coding	ENSG0000022583	2074	3438
3724	KPTN	-0.776	0.027	kaptin, actin binding protein [Source:HGNC Symbol;Acc:HGNC:6404]	chr19:47978400-47987525	NM_001291296, NN	XM_011526399, XM_0	NP_001278225, NP_00	XP_011524701	protein_coding	ENSG0000011816	11133	6404

3725	DERA	0.605	0.027	deoxyribose-phosphate aldolase [Source:HGNC Symbol;Acc:HGNC:24269]	chr12:16064105-16190216	NM_001300779, NN	.	NP_001287708, NP_05	.	protein_coding	ENSG0000002369	51071	24269
3726	FAM98B	0.664	0.027	family with sequence similarity 98 member B [Source:HGNC Symbol;Acc:HGNC:26773]	chr15:38746327-38779911	NM_173611	.	NP_775882	.	protein_coding	ENSG0000017126	283742	26773
3727	ROMO1	-0.645	0.027	reactive oxygen species modulator 1 [Source:HGNC Symbol;Acc:HGNC:16185]	chr20:34255976-34288906	NM_080748	XM_017027678	NP_542786	XP_016883167	protein_coding	ENSG0000012599	140823	16185
3728	UPK3A	-1.771	0.027	uroplakin 3A [Source:HGNC Symbol;Acc:HGNC:12580]	chr22:45680862-45691755	NM_001167574, NN	.	NP_001161046, NP_00	.	protein_coding	ENSG0000010037	7380	12580
3729	WDR41	0.803	0.027	WD repeat domain 41 [Source:HGNC Symbol;Acc:HGNC:25601]	chr5:76726757-76788421	NM_018268	XM_011543505, XM_0	NP_060738	XP_005248608, XP_0052	protein_coding	ENSG0000016425	55255	25601
3730	SEMA4C	-0.577	0.027	semaphorin 4C [Source:HGNC Symbol;Acc:HGNC:10731]	chr2:97525452-97535708	NM_017789	XM_011511383, XM_0	NP_060259	XP_011509680, XP_0115	protein_coding	ENSG0000016875	54910	10731
3731	C1orf159	-0.662	0.028	chromosome 1 open reading frame 159 [Source:HGNC Symbol;Acc:HGNC:26062]	chr1:1017197-1051478	NM_017891	XM_011541667, XM_0	NP_060361	XP_005244822, XP_0067	protein_coding	ENSG0000013159	54991	26062
3732	TMEM74B	-0.824	0.028	transmembrane protein 74B [Source:HGNC Symbol;Acc:HGNC:15893]	chr20:1161204-1165117	NM_001304748, NN	XM_011529284, XM_0	NP_001291677, NP_00	XP_011527584, XP_0115	protein_coding	ENSG0000012589	55321	15893
3733	APLN	0.683	0.028	apelin [Source:HGNC Symbol;Acc:HGNC:16665]	chrX:128779235-128788914	NM_017413	.	NP_059109	.	protein_coding	ENSG0000017138	8862	16665
3734	FANCF	0.701	0.028	Fanconi anemia complementation group F [Source:HGNC Symbol;Acc:HGNC:3587]	chr11:22644064-22648333	NM_022725	.	NP_073562	.	protein_coding	ENSG0000018316	2188	3587
3735	MEGF9	0.819	0.028	multiple EGF like domains 9 [Source:HGNC Symbol;Acc:HGNC:3234]	chr9:123363090-123476748	NM_001080497	.	NP_001073966	.	protein_coding	ENSG0000010678	1955	3234
3736	FAM110C	0.780	0.028	family with sequence similarity 110 member C [Source:HGNC Symbol;Acc:HGNC:33340]	chr2:38813-46385	NM_001077710	XM_011510372	NP_001071178	XP_011508674, XP_0168	protein_coding	ENSG0000018473	642273	33340
3737	RIN2	0.838	0.028	Ras and Rab interactor 2 [Source:HGNC Symbol;Acc:HGNC:18750]	chr20:19867164-19983101	NM_001242581, NN	XM_017027890, XM_0	NP_001229510, NP_06	XP_005260788, XP_0067	protein_coding	ENSG0000013266	54453	18750
3738	TWF1	0.595	0.028	twinfilin actin binding protein 1 [Source:HGNC Symbol;Acc:HGNC:9620]	chr12:44187525-44200178	NM_001242397, NN	.	NP_001229326, NP_00	.	protein_coding	ENSG0000015123	5756	9620
3739	ATP6V0B	-0.538	0.028	ATPase H+ transporting V0 subunit b [Source:HGNC Symbol;Acc:HGNC:861]	chr1:44440319-44443967	NM_001039457, NN	.	NP_001034546, NP_00	.	protein_coding	ENSG0000011741	533	861
3740	BCL2L15	0.609	0.028	BCL2 like 15 [Source:HGNC Symbol;Acc:HGNC:33624]	chr1:114423324-114430169	NM_001010922	.	NP_001010922	.	protein_coding	ENSG0000018876	440603	33624
3741	MYB	0.951	0.028	MYB proto-oncogene, transcription factor [Source:HGNC Symbol;Acc:HGNC:7545]	chr6:135502452-135540311	NM_001130172, NN	.	NP_001123644, NP_00	.	protein_coding	ENSG0000011851	4602	7545
3742	TMEM181	0.672	0.028	transmembrane protein 181 [Source:HGNC Symbol;Acc:HGNC:20958]	chr6:158957467-159056460	NM_020823	XM_011536002, XM_0	NP_065874	XP_005267131, XP_0115	protein_coding	ENSG0000014643	57583	20958
3743	WT1	0.776	0.028	Wilms tumor 1 [Source:HGNC Symbol;Acc:HGNC:12796]	chr11:32409320-32457176	NM_000378, NM_0	.	NP_000369, NP_00118	.	protein_coding	ENSG0000018493	7490	12796
3744	MLLT3	0.905	0.028	MLLT3, super elongation complex subunit [Source:HGNC Symbol;Acc:HGNC:7136]	chr9:20341662-20622542	NM_001286691, NN	.	NP_001273620, NP_00	.	protein_coding	ENSG0000017184	4300	7136
3745	RIMS2	0.968	0.028	regulating synaptic membrane exocytosis 2 [Source:HGNC Symbol;Acc:HGNC:17283]	chr8:104512975-105268322	NM_001100117, NN	XM_006716694	NP_001093587, NP_00	XP_016869516, XP_0168	protein_coding	ENSG0000017640	9699	17283
3746	SERGEF	-0.641	0.028	secretion regulating guanine nucleotide exchange factor [Source:HGNC Symbol;Acc:HGN	chr11:17809594-18034709	NM_012139	.	NP_036271	.	protein_coding	ENSG0000012915	26297	17499
3747	CCSER2	0.940	0.028	coiled-coil serine rich protein 2 [Source:HGNC Symbol;Acc:HGNC:29197]	chr10:86088341-86278277	NM_001284240, NN	XM_017016340, XM_0	NP_001271169, NP_00	XP_005269962, XP_0052	protein_coding	ENSG0000010777	54462	29197
3748	MTA1	-0.535	0.028	metastasis associated 1 [Source:HGNC Symbol;Acc:HGNC:7410]	chr14:105886156-105937066	NM_001203258, NN	XM_011537313, XM_0	NP_001190187, NP_00	XP_011535607, XP_0115	protein_coding	ENSG0000018297	9112	7410
3749	SCAMP1	0.780	0.028	secretory carrier membrane protein 1 [Source:HGNC Symbol;Acc:HGNC:10563]	chr5:77656406-77774894	NM_001290229, NN	XM_011543727	NP_001277158, NP_00	XP_011542029	protein_coding	ENSG0000008536	9522	10563
3750	XYLB	-0.887	0.028	xylulokinase [Source:HGNC Symbol;Acc:HGNC:12839]	chr3:38388250-38456467	NM_005108	XM_011534330, XM_0	NP_005099	XP_011532627, XP_0115	protein_coding	ENSG0000009321	9942	12839
3751	BLOC1S6	1.165	0.028	biogenesis of lysosomal organelles complex 1 subunit 6 [Source:HGNC Symbol;Acc:HGN	chr15:45879320-45983492	NM_001311255, NN	.	NP_001298184, NP_03	.	protein_coding	ENSG0000010416	26258	8549
3752	NOCT	0.713	0.028	nocturnin [Source:HGNC Symbol;Acc:HGNC:14254]	chr4:139936942-139967091	NM_012118	.	NP_036250	.	protein_coding	ENSG0000015101	25819	14254
3753	PTPN1	0.512	0.028	protein tyrosine phosphatase, non-receptor type 1 [Source:HGNC Symbol;Acc:HGNC:96	chr20:49126857-49201778	NM_001278618, NN	.	NP_001265547, NP_00	.	protein_coding	ENSG0000019639	5770	9642
3754	SLC12A7	-0.540	0.028	solute carrier family 12 member 7 [Source:HGNC Symbol;Acc:HGNC:10915]	chr5:1050490-1112150	NM_006598	XM_017008959, XM_0	NP_006589	XP_005248288, XP_0115	protein_coding	ENSG0000011350	10723	10915
3755	TDRD3	0.794	0.028	tudor domain containing 3 [Source:HGNC Symbol;Acc:HGNC:20612]	chr13:60970590-61148012	NM_001146070, NN	XM_011535248, XM_0	NP_001139542, NP_00	XP_005266613, XP_0052	protein_coding	ENSG0000008354	81550	20612
3756	ZNF765	0.804	0.028	zinc finger protein 765 [Source:HGNC Symbol;Acc:HGNC:25092]	chr19:53898402-53933666	NM_001040185	XM_006723466	NP_001035275	XP_006723529	protein_coding	ENSG0000019641	91661	25092
3757	MAMDC4	-1.026	0.028	MAM domain containing 4 [Source:HGNC Symbol;Acc:HGNC:24083]	chr9:139746818-139755249	NM_206920	.	NP_996803	.	protein_coding	ENSG0000017794	158056	24083
3758	BICC1	1.022	0.028	BicC family RNA binding protein 1 [Source:HGNC Symbol;Acc:HGNC:19351]	chr10:60272899-60591197	NM_001080512	XM_017016678, XM_0	NP_001073981	XP_005270226, XP_0115	protein_coding	ENSG0000012287	80114	19351
3759	DNAJC24	0.783	0.028	DnaJ heat shock protein family (Hsp40) member C24 [Source:HGNC Symbol;Acc:HGNC:2	chr11:31391386-31453396	NM_181706	.	NP_859057	.	protein_coding	ENSG0000017094	120526	26979
3760	SFXN1	0.758	0.028	sideroflexin 1 [Source:HGNC Symbol;Acc:HGNC:16085]	chr5:174905397-174956745	NM_001322977, NN	XM_005266012	NP_001309906, NP_07	.	protein_coding	ENSG0000016446	94081	16085
3761	CASP9	-0.721	0.028	caspase 9 [Source:HGNC Symbol;Acc:HGNC:1511]	chr1:15802595-15851221	NM_001229, NM_0	XM_011542273, XM_0	NP_001220, NP_00126	XP_005246071, XP_0115	protein_coding	ENSG0000013290	842	1511
3762	NRN1	-0.753	0.028	neuritin 1 [Source:HGNC Symbol;Acc:HGNC:17972]	chr6:5998231-6007838	NM_001278710, NN	.	NP_001265639, NP_00	.	protein_coding	ENSG0000012478	51299	17972
3763	ZNF644	1.054	0.028	zinc finger protein 644 [Source:HGNC Symbol;Acc:HGNC:29222]	chr1:91380854-91487829	NM_016620, NM_0	XM_017002491, XM_0	NP_057704, NP_11556	XP_005271314, XP_0052	protein_coding	ENSG0000012248	84146	29222
3764	C19orf24	-0.596	0.028	chromosome 19 open reading frame 24 [Source:HGNC Symbol;Acc:HGNC:26073]	chr19:1275436-1279248	NM_017914	.	NP_060384	.	protein_coding	ENSG0000022830	55009	26073
3765	GTF3A	-0.548	0.028	general transcription factor IIIA [Source:HGNC Symbol;Acc:HGNC:4662]	chr13:27998680-28024711	NM_002097	.	NP_002088	.	protein_coding	ENSG0000012203	2971	4662
3766	PITX1	-0.555	0.028	paired like homeodomain 1 [Source:HGNC Symbol;Acc:HGNC:9004]	chr5:134363424-134370324	NM_002653	.	NP_002644	.	protein_coding	ENSG0000006901	5307	9004
3767	POLR3F	0.870	0.028	RNA polymerase III subunit F [Source:HGNC Symbol;Acc:HGNC:15763]	chr20:18364012-18465287	NM_001282526, NN	XM_011529141	NP_001269455, NP_00	XP_011527443	protein_coding	ENSG0000013266	10621	15763
3768	RBP7	-0.652	0.028	retinol binding protein 7 [Source:HGNC Symbol;Acc:HGNC:30316]	chr1:10057273-10076078	NM_052960	.	NP_443192	.	protein_coding	ENSG0000016244	116362	30316
3769	SH3BGR	0.958	0.028	SH3 domain binding glutamate rich protein [Source:HGNC Symbol;Acc:HGNC:10822]	chr21:40817780-40887433	NM_001001713, NN	XM_006724042, XM_0	NP_001001713, NP_00	XP_005261081, XP_0052	protein_coding	ENSG0000018543	6450	10822
3770	CSGALNACT1	-0.962	0.029	chondroitin sulfate N-acetylgalactosaminyltransferase 1 [Source:HGNC Symbol;Acc:HGN	chr8:19261671-19540094	NM_001130518, NN	XM_017013637, XM_0	NP_001123990, NP_06	XP_006716421, XP_0067	protein_coding	ENSG0000014740	55790	24290
3771	FECH	-0.748	0.029	ferrochelatase [Source:HGNC Symbol;Acc:HGNC:3647]	chr18:55215514-55254004	NM_000140, NM_0	XM_011525882, XM_0	NP_000131, NP_00101	XP_011524183, XP_0115	protein_coding	ENSG0000006692	2235	3647
3772	OTUD6B	0.860	0.029	OTU domain containing 6B [Source:HGNC Symbol;Acc:HGNC:24281]	chr8:92082423-92099323	NM_001286745, NN	XM_011517129	NP_001273674, NP_05	XP_011515431	protein_coding	ENSG0000015510	51633	24281
3773	PMM1	-0.644	0.029	phosphomannomutase 1 [Source:HGNC Symbol;Acc:HGNC:9114]	chr22:41972897-41985894	NM_002676	XM_011530230, XM_0	NP_002667	XP_005261695, XP_0115	protein_coding	ENSG0000010041	5372	9114
3774	NCBP2-AS2	-0.597	0.029	NCBP2 antisense RNA 2 (head to head) [Source:HGNC Symbol;Acc:HGNC:25121]	chr3:196669493-196670411	.	.	.	.	protein_coding	ENSG0000027017	.	25121
3775	ACBD5	0.776	0.029	acyl-CoA binding domain containing 5 [Source:HGNC Symbol;Acc:HGNC:23338]	chr10:27484142-27531059	NM_001042473, NN	XM_017016908, XM_0	NP_001035938, NP_00	XP_006717591, XP_0067	protein_coding	ENSG0000010789	91452	23338
3776	PNRC1	-0.845	0.029	proline rich nuclear receptor coactivator 1 [Source:HGNC Symbol;Acc:HGNC:17278]	chr6:89790469-89794879	NM_006813	.	NP_006804	.	protein_coding	ENSG0000014627	10957	17278
3777	ZNF138	0.961	0.029	zinc finger protein 138 [Source:HGNC Symbol;Acc:HGNC:12922]	chr7:64254765-64294059	NM_001160183, NN	.	NP_001153655, NP_00	.	protein_coding	ENSG0000019700	7697	12922
3778	DYNLT1	-0.582	0.029	dynein light chain Tctex-type 1 [Source:HGNC Symbol;Acc:HGNC:11697]	chr6:159057505-159065771	NM_001291603, NN	.	NP_001278532, NP_00	.	protein_coding	ENSG0000014642	6993	11697

3779	PCOLCE	-0.814	0.029	procollagen C-endopeptidase enhancer [Source:HGNC Symbol;Acc:HGNC:8738]	chr7:100199799-100205798	NM_002593	.	NP_002584	.	protein_coding	ENSG0000010633	5118	8738
3780	CD14	-0.971	0.029	CD14 molecule [Source:HGNC Symbol;Acc:HGNC:1628]	chr5:140011312-140013286	NM_000591, NM_0	.	NP_000582, NP_00103	.	protein_coding	ENSG0000017045	929	1628
3781	RCOR2	-0.819	0.029	REST corepressor 2 [Source:HGNC Symbol;Acc:HGNC:27455]	chr11:63678692-63684316	NM_173587	XM_005273932	NP_775858	XP_005273989	protein_coding	ENSG0000016777	283248	27455
3782	COL26A1	-0.855	0.029	collagen type XXVI alpha 1 chain [Source:HGNC Symbol;Acc:HGNC:18038]	chr7:101006100-101202304	NM_001278563, NN	.	NP_001265492, NP_59	XP_016867232	protein_coding	ENSG0000016096	136227	18038
3783	CSNK1A1L	0.944	0.029	casein kinase 1 alpha 1 like [Source:HGNC Symbol;Acc:HGNC:20289]	chr13:37677395-37679801	NM_145203	.	NP_660204	.	protein_coding	ENSG0000018013	122011	20289
3784	RASSF1	-0.584	0.029	Ras association domain family member 1 [Source:HGNC Symbol;Acc:HGNC:9882]	chr3:50367212-50378367	NM_001206957, NN	XM_011533316, XM_0	NP_001193886, NP_00	XP_011531617, XP_0115	protein_coding	ENSG0000006802	11186	9882
3785	VASP	-0.506	0.029	vasodilator-stimulated phosphoprotein [Source:HGNC Symbol;Acc:HGNC:12652]	chr19:46010673-46030241	NM_003370	XM_017027200, XM_0	NP_003361	XP_005259256, XP_0052	protein_coding	ENSG0000012575	7408	12652
3786	BLOC1S1	-0.577	0.029	biogenesis of lysosomal organelles complex 1 subunit 1 [Source:HGNC Symbol;Acc:HGN	chr12:56109819-56118489	NM_001487	.	NP_001478	.	protein_coding	ENSG0000013544	2647	4200
3787	MGST3	-0.562	0.029	microsomal glutathione S-transferase 3 [Source:HGNC Symbol;Acc:HGNC:7064]	chr1:165600097-165631033	NM_004528	XM_005245174	NP_004519	XP_005245231	protein_coding	ENSG0000014319	4259	7064
3788	VWA9	0.645	0.029	von Willebrand factor A domain containing 9 [Source:HGNC Symbol;Acc:HGNC:25372]	chr15:65871119-65903627	NM_001136043, NN	XM_011522101, XM_0	NP_001129515, NP_00	XP_006720771, XP_0067	protein_coding	ENSG0000013861	81556	25372
3789	ZNF397	1.091	0.029	zinc finger protein 397 [Source:HGNC Symbol;Acc:HGNC:18818]	chr18:32821000-32870209	NM_001135178, NN	XM_011526231, XM_0	NP_001128650, NP_11	XP_006722621, XP_0115	protein_coding	ENSG0000018681	84307	18818
3790	BPNT1	-0.649	0.029	3'(2'), 5'-bisphosphate nucleotidase 1 [Source:HGNC Symbol;Acc:HGNC:1096]	chr1:220230823-220263804	NM_001286149, NN	XM_017000043, XM_0	NP_001273078, NP_00	XP_005273055, XP_0052	protein_coding	ENSG0000016281	10380	1096
3791	LRR1	0.745	0.029	leucine rich repeat protein 1 [Source:HGNC Symbol;Acc:HGNC:19742]	chr14:50065414-50081390	NM_152329, NM_2	.	NP_689542, NP_98229	.	protein_coding	ENSG0000016550	122769	19742
3792	NCOA7	0.774	0.029	nuclear receptor coactivator 7 [Source:HGNC Symbol;Acc:HGNC:21081]	chr6:126102306-126253176	NM_001122842, NN	XM_011535455, XM_0	NP_001116314, NP_00	XP_005266879, XP_0067	protein_coding	ENSG0000011191	135112	21081
3793	MAP1S	-0.593	0.029	microtubule associated protein 1S [Source:HGNC Symbol;Acc:HGNC:15715]	chr19:17830160-17845325	NM_001308363, NN	XM_017026930, XM_0	NP_001295292, NP_06	XP_011526420, XP_0168	protein_coding	ENSG0000013047	55201	15715
3794	ZNF564	1.078	0.029	zinc finger protein 564 [Source:HGNC Symbol;Acc:HGNC:31106]	chr19:12571997-12662343	NM_144976	.	NP_659413	.	protein_coding	ENSG0000024970	163050	31106
3795	C2orf69	0.694	0.029	chromosome 2 open reading frame 69 [Source:HGNC Symbol;Acc:HGNC:26799]	chr2:200775978-200792996	NM_153689	.	NP_710156	.	protein_coding	ENSG0000017807	205327	26799
3796	DAPP1	1.287	0.029	dual adaptor of phosphotyrosine and 3-phosphoinositides 1 [Source:HGNC Symbol;Acc:	chr4:100737989-100791311	NM_001306151, NN	XM_011531842, XM_0	NP_001293080, NP_05	XP_011530142, XP_0115	protein_coding	ENSG0000007019	27071	16500
3797	ECHS1	-0.523	0.029	enoyl-CoA hydratase, short chain 1 [Source:HGNC Symbol;Acc:HGNC:3151]	chr10:135175983-135187193	NM_004092	.	NP_004083	.	protein_coding	ENSG0000012788	1892	3151
3798	PCGF5	0.717	0.029	polycomb group ring finger 5 [Source:HGNC Symbol;Acc:HGNC:28264]	chr10:92922768-93044088	NM_001256549, NN	XM_017016776, XM_0	NP_001243478, NP_00	XP_006718093, XP_0115	protein_coding	ENSG0000018062	84333	28264
3799	ZMYM1	0.832	0.029	zinc finger MYM-type containing 1 [Source:HGNC Symbol;Acc:HGNC:26253]	chr1:35525386-35581460	NM_001289088, NN	XM_011542166, XM_0	NP_001276017, NP_00	XP_011540465, XP_0115	protein_coding	ENSG0000019709	79830	26253
3800	CHMP7	0.545	0.029	charged multivesicular body protein 7 [Source:HGNC Symbol;Acc:HGNC:28439]	chr8:23101149-23119512	NM_001317899, NN	XM_011544695, XM_0	NP_001304828, NP_68	XP_006716478	protein_coding	ENSG0000014745	91782	28439
3801	NDUFB10	-0.541	0.029	NADH:ubiquinone oxidoreductase subunit B10 [Source:HGNC Symbol;Acc:HGNC:7696]	chr16:2009508-2011976	NM_004548	.	NP_004539	.	protein_coding	ENSG0000014099	4716	7696
3802	N4BP1	0.678	0.029	NEDD4 binding protein 1 [Source:HGNC Symbol;Acc:HGNC:29850]	chr16:48572636-48644120	NM_153029	XM_011523482	NP_694574	XP_011521784	protein_coding	ENSG0000010292	9683	29850
3803	MON1A	-0.686	0.029	MON1 homolog A, secretory trafficking associated [Source:HGNC Symbol;Acc:HGNC:282	chr3:49946294-49967606	NM_001142501, NN	XM_011534160, XM_0	NP_001135973, NP_11	XP_006713408, XP_0115	protein_coding	ENSG0000016407	84315	28207
3804	PARVB	-0.592	0.029	parvin beta [Source:HGNC Symbol;Acc:HGNC:14653]	chr22:44395090-44568829	NM_001003828, NN	XM_005261596	NP_001003828, NP_00	XP_005261653, XP_0168	protein_coding	ENSG0000018867	29780	14653
3805	NF2	0.604	0.029	neurofibromin 2 [Source:HGNC Symbol;Acc:HGNC:7773]	chr22:29999544-30094587	NM_000268, NM_0	XM_017028809	NP_000259, NP_05750	XP_016884298, XP_0168	protein_coding	ENSG0000018657	4771	7773
3806	PKP4	1.021	0.029	plakophilin 4 [Source:HGNC Symbol;Acc:HGNC:9026]	chr2:159313608-159539391	NM_001005476, NN	XM_011512024, XM_0	NP_001005476, NP_00	XP_011510318, XP_0115	protein_coding	ENSG0000014428	8502	9026
3807	SEPP1	-1.377	0.029	selenoprotein P, plasma, 1 [Source:HGNC Symbol;Acc:HGNC:10751]	chr5:42756902-42825998	NM_001085486, NN	.	NP_001078955, NP_00	.	protein_coding	ENSG0000025072	6414	10751
3808	ZNF44	0.962	0.029	zinc finger protein 44 [Source:HGNC Symbol;Acc:HGNC:13110]	chr19:12382626-12405702	NM_001164276, NN	XM_017026871, XM_0	NP_001157748, NP_05	XP_005259991, XP_0115	protein_coding	ENSG0000019785	51710	13110
3809	NUP98	0.545	0.029	nucleoporin 98 [Source:HGNC Symbol;Acc:HGNC:8068]	chr11:3696239-3819022	NM_005387, NM_0	XM_011520138, XM_0	NP_005378, NP_05740	XP_005253007, XP_0115	protein_coding	ENSG0000011071	4928	8068
3810	VRK2	1.181	0.029	vaccinia related kinase 2 [Source:HGNC Symbol;Acc:HGNC:12719]	chr2:58134785-58468485	NM_001130480, NN	XM_011533094, XM_0	NP_001123952, NP_00	XP_005264597, XP_0052	protein_coding	ENSG0000002811	7444	12719
3811	IFI27L2	-0.581	0.029	interferon alpha inducible protein 27 like 2 [Source:HGNC Symbol;Acc:HGNC:19753]	chr14:94594117-94596590	NM_032036	.	NP_114425	.	protein_coding	ENSG0000011963	83982	19753
3812	MRPS33	0.673	0.030	mitochondrial ribosomal protein S33 [Source:HGNC Symbol;Acc:HGNC:16634]	chr7:140702409-140715028	NM_016071, NM_0	.	NP_057155, NP_44426	.	protein_coding	ENSG0000009026	51650	16634
3813	NLE1	-0.615	0.030	notchless homolog 1 [Source:HGNC Symbol;Acc:HGNC:19889]	chr17:33448597-33469334	NM_001014445, NN	XM_017024777, XM_0	NP_001014445, NP_06	XP_016880266	protein_coding	ENSG0000007353	54475	19889
3814	LSM14A	0.570	0.030	LSM14A, mRNA processing body assembly factor [Source:HGNC Symbol;Acc:HGNC:2448	chr19:34663429-34720420	NM_001114093, NN	XM_005258720, XM_0	NP_001107565, NP_05	XP_005258776, XP_0052	protein_coding	ENSG0000025710	26065	24489
3815	POLK	1.794	0.030	DNA polymerase kappa [Source:HGNC Symbol;Acc:HGNC:9183]	chr5:74666927-74896969	NM_016218	XM_011543468, XM_0	NP_057302	XP_005248591, XP_0115	protein_coding	ENSG0000012200	51426	9183
3816	SLC35B1	0.602	0.030	solute carrier family 35 member B1 [Source:HGNC Symbol;Acc:HGNC:20798]	chr17:47778304-47785561	NM_001278784, NN	XM_011524180, XM_0	NP_001265713, NP_00	XP_005256986, XP_0067	protein_coding	ENSG0000012107	10237	20798
3817	ASNSD1	0.681	0.030	asparagine synthetase domain containing 1 [Source:HGNC Symbol;Acc:HGNC:24910]	chr2:190526110-190535557	NM_019048	XM_005246653	NP_061921	XP_005246710, XP_0168	protein_coding	ENSG0000013838	54529	24910
3818	ZC3HAV1	0.686	0.030	zinc finger CCCH-type containing, antiviral 1 [Source:HGNC Symbol;Acc:HGNC:23721]	chr7:138728265-138794465	NM_020119, NM_0	XM_005250501	NP_064504, NP_07890	XP_005250558	protein_coding	ENSG0000010593	56829	23721
3819	ZC3H13	0.586	0.030	zinc finger CCCH-type containing 13 [Source:HGNC Symbol;Acc:HGNC:20368]	chr13:46528599-46626894	NM_015070	XM_017020482, XM_0	NP_055885	XP_005266358, XP_0052	protein_coding	ENSG0000012320	23091	20368
3820	PSG11	-1.062	0.030	pregnancy specific beta-1-glycoprotein 11 [Source:HGNC Symbol;Acc:HGNC:9516]	chr19:43511807-43530664	NM_001113410, NN	.	NP_001106881, NP_00	.	protein_coding	ENSG0000024313	5680	9516
3821	MED13L	0.590	0.030	mediator complex subunit 13 like [Source:HGNC Symbol;Acc:HGNC:22962]	chr12:116391676-116715143	NM_015335	XM_011538082, XM_0	NP_056150	XP_011536382, XP_0115	protein_coding	ENSG0000012306	23389	22962
3822	SPATA2L	-0.970	0.030	spermatogenesis associated 2 like [Source:HGNC Symbol;Acc:HGNC:28393]	chr16:89747144-89768113	NM_152339	XM_005256279	NP_689552	XP_005256336	protein_coding	ENSG0000015879	124044	28393
3823	TMEM110	-0.650	0.030	transmembrane protein 110 [Source:HGNC Symbol;Acc:HGNC:30526]	chr3:52867129-52931612	NM_198563	.	NP_940965	.	protein_coding	ENSG0000021353	375346	30526
3824	ZNRD1	-0.605	0.030	zinc ribbon domain containing 1 [Source:HGNC Symbol;Acc:HGNC:13182]	chr6:30029030-30032686	NM_001278785, NN	.	NP_001265714, NP_00	.	protein_coding	ENSG0000006637	30834	13182
3825	FANCI	1.143	0.030	Fanconi anemia complementation group I [Source:HGNC Symbol;Acc:HGNC:25568]	chr15:89787179-89878092	NM_001113378, NN	XM_011521769, XM_0	NP_001106849, NP_06	XP_011520058, XP_0115	protein_coding	ENSG0000014052	55215	25568
3826	FKBP4	-0.498	0.030	FK506 binding protein 4 [Source:HGNC Symbol;Acc:HGNC:3720]	chr12:2904118-2914589	NM_002014	XM_011520929	NP_002005	XP_011519231	protein_coding	ENSG0000000447	2288	3720
3827	RNASE1	-0.935	0.030	ribonuclease A family member 1, pancreatic [Source:HGNC Symbol;Acc:HGNC:10044]	chr14:21269386-21271437	NM_002933, NM_1	.	NP_002924, NP_93787	.	protein_coding	ENSG0000012953	6035	10044
3828	CRK	0.543	0.030	CRK proto-oncogene, adaptor protein [Source:HGNC Symbol;Acc:HGNC:2362]	chr17:1323982-1359552	NM_005206, NM_0	.	NP_005197, NP_05843	.	protein_coding	ENSG0000016719	1398	2362
3829	IRS1	0.736	0.030	insulin receptor substrate 1 [Source:HGNC Symbol;Acc:HGNC:6125]	chr2:227596032-227664475	NM_005544	.	NP_005535	.	protein_coding	ENSG0000016904	3667	6125
3830	LCOR	0.826	0.030	ligand dependent nuclear receptor corepressor [Source:HGNC Symbol;Acc:HGNC:29503	chr10:98592016-98724198	NM_001170765, NN	XM_017016788, XM_0	NP_001164236, NP_00	XP_006718096, XP_0067	protein_coding	ENSG0000019623	84458	29503
3831	ZNF23	0.901	0.030	zinc finger protein 23 [Source:HGNC Symbol;Acc:HGNC:13023]	chr16:71481499-71496662	NM_001304492, NN	.	NP_001291421, NP_00	.	protein_coding	ENSG0000016737	7571	13023
3832	BTRC	0.676	0.030	beta-transducin repeat containing E3 ubiquitin protein ligase [Source:HGNC Symbol;Acc	chr10:103113789-103317078	NM_001256856, NN	XM_006718054	NP_001243785, NP_00	XP_006718117, XP_0168	protein_coding	ENSG0000016616	8945	1144

3833	VARS2	-0.673	0.030	valyl-tRNA synthetase 2, mitochondrial [Source:HGNC Symbol;Acc:HGNC:21642]	chr6:30881981-30894236	NM_001167733, NN	.	NP_001161205, NP_00	.	protein_coding	ENSG0000013741	57176	21642
3834	ZSWIM4	-0.689	0.030	zinc finger SWIM-type containing 4 [Source:HGNC Symbol;Acc:HGNC:25704]	chr19:13906273-13943044	NM_023072	XM_005260023	NP_075560	XP_005260080, XP_0168	protein_coding	ENSG0000013200	65249	25704
3835	AP1S3	0.904	0.030	adaptor related protein complex 1 sigma 3 subunit [Source:HGNC Symbol;Acc:HGNC:18	chr2:224616402-224702319	NM_001039569	.	NP_001034658	XP_011508902	protein_coding	ENSG0000015205	130340	18971
3836	ESYT2	0.550	0.030	extended synaptotagmin 2 [Source:HGNC Symbol;Acc:HGNC:22211]	chr7:158523685-158622729	NM_020728	.	NP_065779	.	protein_coding	ENSG0000011786	57488	22211
3837	GINS4	0.619	0.030	GINS complex subunit 4 [Source:HGNC Symbol;Acc:HGNC:28226]	chr8:41386724-41402565	NM_032336	XM_005273659	NP_115712	XP_005273716	protein_coding	ENSG0000014753	84296	28226
3838	HIST1H2AG	0.883	0.030	histone cluster 1, H2ag [Source:HGNC Symbol;Acc:HGNC:4737]	chr6:27100820-27103070	NM_021064	.	NP_066408	.	protein_coding	ENSG0000019678	8969	4737
3839	FRS2	1.042	0.030	fibroblast growth factor receptor substrate 2 [Source:HGNC Symbol;Acc:HGNC:16971]	chr12:69864154-69973569	NM_001042555, NN	XM_017018719, XM_0	NP_001036020, NP_00	XP_016874206, XP_0168	protein_coding	ENSG0000016622	10818	16971
3840	ANTXR1	0.786	0.030	anthrax toxin receptor 1 [Source:HGNC Symbol;Acc:HGNC:21014]	chr2:69240309-69476459	NM_018153, NM_0	XM_011533124	NP_060623, NP_11558	XP_016860564, XP_0168	protein_coding	ENSG0000016960	84168	21014
3841	ZNF821	-0.821	0.030	zinc finger protein 821 [Source:HGNC Symbol;Acc:HGNC:28043]	chr16:71893582-71918107	NM_001201552, NN	XM_017023423, XM_0	NP_001188481, NP_00	XP_005256090, XP_0067	protein_coding	ENSG0000010298	55565	28043
3842	EGR2	-0.920	0.030	early growth response 2 [Source:HGNC Symbol;Acc:HGNC:3239]	chr10:64571755-64578927	NM_000399, NM_0	XM_011539429, XM_0	NP_000390, NP_00112	XP_011537729, XP_0115	protein_coding	ENSG0000012287	1959	3239
3843	ZNF354B	0.915	0.030	zinc finger protein 354B [Source:HGNC Symbol;Acc:HGNC:17197]	chr5:178286953-178312029	NM_058230	.	NP_478137	.	protein_coding	ENSG0000017833	117608	17197
3844	B4GAT1	-0.641	0.030	beta-1,4-glucuronyltransferase 1 [Source:HGNC Symbol;Acc:HGNC:15685]	chr11:66112842-66115163	NM_006876	.	NP_006867	.	protein_coding	ENSG0000017468	11041	15685
3845	ADCY6	-0.593	0.031	adenylate cyclase 6 [Source:HGNC Symbol;Acc:HGNC:237]	chr12:49159980-49182820	NM_015270, NM_0	XM_011537811, XM_0	NP_056085, NP_06619	XP_005268662, XP_0067	protein_coding	ENSG0000017423	112	237
3846	MIPEP	-0.609	0.031	mitochondrial intermediate peptidase [Source:HGNC Symbol;Acc:HGNC:7104]	chr13:24304327-24463558	NM_005932	XM_011535097	NP_005923	XP_011533399	protein_coding	ENSG0000002700	4285	7104
3847	SAAL1	0.619	0.031	serum amyloid A like 1 [Source:HGNC Symbol;Acc:HGNC:25158]	chr11:18101838-18127638	NM_138421	.	NP_612430	.	protein_coding	ENSG0000016678	113174	25158
3848	ASNS	0.503	0.031	asparagine synthetase (glutamine-hydrolyzing) [Source:HGNC Symbol;Acc:HGNC:753]	chr7:97481429-97501854	NM_001178075, NN	.	NP_001171546, NP_00	.	protein_coding	ENSG0000007066	440	753
3849	HIVEP1	0.822	0.031	human immunodeficiency virus type I enhancer binding protein 1 [Source:HGNC Symbo	chr6:12012570-12165232	NM_002114	XM_011514553, XM_0	NP_002105	XP_011512848, XP_0115	protein_coding	ENSG0000009595	3096	4920
3850	ITPR3	-0.495	0.031	inositol 1,4,5-trisphosphate receptor type 3 [Source:HGNC Symbol;Acc:HGNC:6182]	chr6:33588141-33664351	NM_002224	XM_011514577, XM_0	NP_002215	XP_011512879	protein_coding	ENSG0000009643	3710	6182
3851	RC3H2	1.069	0.031	ring finger and CCCH-type domains 2 [Source:HGNC Symbol;Acc:HGNC:21461]	chr9:125606834-125667620	NM_001100588, NN	.	NP_001094058, NP_06	.	protein_coding	ENSG0000005658	54542	21461
3852	WDTC1	-0.533	0.031	WD and tetratricopeptide repeats 1 [Source:HGNC Symbol;Acc:HGNC:29175]	chr1:27561006-27635124	NM_001276252, NN	XM_011541057	NP_001263181, NP_05	XP_011539359	protein_coding	ENSG0000014278	23038	29175
3853	ABHD11	-0.567	0.031	abhydrolase domain containing 11 [Source:HGNC Symbol;Acc:HGNC:16407]	chr7:73150423-73153197	NM_001145364, NN	XM_011516590, XM_0	NP_001138836, NP_00	XP_011514892	protein_coding	ENSG0000010607	83451	16407
3854	CLYBL	-0.887	0.031	citrate lyase beta like [Source:HGNC Symbol;Acc:HGNC:18355]	chr13:100258922-100549388	NM_206808	XM_011521052, XM_0	NP_996531	XP_005254087, XP_0052	protein_coding	ENSG0000012524	171425	18355
3855	SDK1	-0.655	0.031	sidekick cell adhesion molecule 1 [Source:HGNC Symbol;Acc:HGNC:19307]	chr7:3341079-4308632	NM_152744	XM_017011837, XM_0	NP_689957	XP_011513490, XP_0115	protein_coding	ENSG0000014655	221935	19307
3856	CENPB	-0.527	0.031	centromere protein B [Source:HGNC Symbol;Acc:HGNC:1852]	chr20:3764497-3767337	NM_001810	.	NP_001801	.	protein_coding	ENSG0000012581	1059	1852
3857	CLSTN3	-0.625	0.031	calsyntenin 3 [Source:HGNC Symbol;Acc:HGNC:18371]	chr12:7282794-7311541	NM_014718	XM_006719163	NP_055533	XP_006719226	protein_coding	ENSG0000013918	9746	18371
3858	DUSP7	-0.641	0.031	dual specificity phosphatase 7 [Source:HGNC Symbol;Acc:HGNC:3073]	chr3:52082934-52090566	NM_001947	.	NP_001938	.	protein_coding	ENSG0000016408	1849	3073
3859	LIN54	0.905	0.031	lin-54 DREAM MuvB core complex component [Source:HGNC Symbol;Acc:HGNC:25397]	chr4:83845755-83934079	NM_001115007, NN	XM_006714081, XM_0	NP_001108479, NP_00	XP_005262807, XP_0067	protein_coding	ENSG0000018930	132660	25397
3860	TM2D3	0.740	0.031	TM2 domain containing 3 [Source:HGNC Symbol;Acc:HGNC:24128]	chr15:102173179-102192594	NM_001307960, NN	XM_005254981, XM_0	NP_001294889, NP_00	XP_005255037, XP_0052	protein_coding	ENSG0000018427	80213	24128
3861	ATP2C2	-0.669	0.031	ATPase secretory pathway Ca2+ transporting 2 [Source:HGNC Symbol;Acc:HGNC:29103	chr16:84402132-84497793	NM_001286527, NN	XM_011523486	NP_001273456, NP_00	XP_011521788	protein_coding	ENSG0000006427	9914	29103
3862	RPS6KB1	0.974	0.031	ribosomal protein S6 kinase B1 [Source:HGNC Symbol;Acc:HGNC:10436]	chr17:57970406-58050462	NM_001272042, NN	XM_011525103, XM_0	NP_001258971, NP_00	XP_011523403, XP_0115	protein_coding	ENSG0000010844	6198	10436
3863	C15orf41	0.900	0.031	chromosome 15 open reading frame 41 [Source:HGNC Symbol;Acc:HGNC:26929]	chr15:36871811-37102447	NM_001130010, NN	XM_011522112, XM_0	NP_001123482, NP_00	XP_011520412, XP_0115	protein_coding	ENSG0000018607	84529	26929
3864	TYK2	-0.599	0.031	tyrosine kinase 2 [Source:HGNC Symbol;Acc:HGNC:12440]	chr19:10461204-10491352	NM_003331	XM_011528248, XM_0	NP_003322	XP_011526547, XP_0115	protein_coding	ENSG0000010539	7297	12440
3865	ZMYND11	0.754	0.031	zinc finger MYND-type containing 11 [Source:HGNC Symbol;Acc:HGNC:16966]	chr10:180404-300577	NM_001202464, NN	XM_017015592, XM_0	NP_001189393, NP_00	XP_005252416, XP_0052	protein_coding	ENSG0000001517	10771	16966
3866	TMEM30B	0.731	0.031	transmembrane protein 30B [Source:HGNC Symbol;Acc:HGNC:27254]	chr14:61744087-61748558	NM_001017970	.	NP_001017970	.	protein_coding	ENSG0000018210	161291	27254
3867	MALT1	0.785	0.031	MALT1 paracaspase [Source:HGNC Symbol;Acc:HGNC:6819]	chr18:56338617-56421709	NM_006785, NM_1	.	NP_006776, NP_77621	.	protein_coding	ENSG0000017217	10892	6819
3868	PRC1	0.540	0.031	protein regulator of cytokinesis 1 [Source:HGNC Symbol;Acc:HGNC:9341]	chr15:91509267-91538859	NM_001267580, NN	XM_017022715, XM_0	NP_001254509, NP_00	XP_005255044, XP_0067	protein_coding	ENSG0000019890	9055	9341
3869	ASNA1	-0.546	0.031	arsA arsenite transporter, ATP-binding, homolog 1 (bacterial) [Source:HGNC Symbol;Acc	chr19:12847952-12859137	NM_004317	.	NP_004308	.	protein_coding	ENSG0000019835	439	752
3870	DHFR	0.738	0.031	dihydrofolate reductase [Source:HGNC Symbol;Acc:HGNC:2861]	chr5:79922046-80172279	NM_000791, NM_0	.	NP_000782, NP_00127	.	protein_coding	ENSG0000022871	1719	2861
3871	TALDO1	-0.498	0.031	transaldolase 1 [Source:HGNC Symbol;Acc:HGNC:11559]	chr11:747328-765024	NM_006755	.	NP_006746	.	protein_coding	ENSG0000017715	6888	11559
3872	ANXA9	-0.793	0.031	annexin A9 [Source:HGNC Symbol;Acc:HGNC:547]	chr1:150954492-150968110	NM_003568	XM_011510058, XM_0	NP_003559	XP_011508360	protein_coding	ENSG0000014341	8416	547
3873	GSR	0.533	0.031	glutathione-disulfide reductase [Source:HGNC Symbol;Acc:HGNC:4623]	chr8:30535577-30585443	NM_000637, NM_0	.	NP_000628, NP_00118	.	protein_coding	ENSG0000010468	2936	4623
3874	MTHFD2	0.549	0.031	methylenetetrahydrofolate dehydrogenase (NADP+ dependent) 2, methenyltetrahydrof	chr2:74425688-74570536	NM_006636	.	NP_006627	.	protein_coding	ENSG0000006591	10797	7434
3875	PSMD8	-0.504	0.031	proteasome 26S subunit, non-ATPase 8 [Source:HGNC Symbol;Acc:HGNC:9566]	chr19:38865175-38874464	NM_002812	.	NP_002803	.	protein_coding	ENSG0000009934	5714	9566
3876	SEL1L3	0.542	0.031	SEL1L family member 3 [Source:HGNC Symbol;Acc:HGNC:29108]	chr4:25749048-25865217	NM_001297592, NN	XM_011513820, XM_0	NP_001284521, NP_00	XP_011512121	protein_coding	ENSG0000009149	23231	29108
3877	LINC00493	-0.616	0.031	long intergenic non-protein coding RNA 493 [Source:HGNC Symbol;Acc:HGNC:43430]	chr20:18547990-18550207	.	.	.	.	protein_coding	ENSG0000023238	.	43430
3878	RHPN2	0.580	0.031	rhophilin Rho GTPase binding protein 2 [Source:HGNC Symbol;Acc:HGNC:19974]	chr19:33469498-33555794	NM_033103	.	NP_149094	.	protein_coding	ENSG0000013194	85415	19974
3879	WBP11	0.528	0.031	WW domain binding protein 11 [Source:HGNC Symbol;Acc:HGNC:16461]	chr12:14937512-14956474	NM_016312	.	NP_057396	.	protein_coding	ENSG0000008446	51729	16461
3880	CALCA	-2.269	0.031	calcitonin related polypeptide alpha [Source:HGNC Symbol;Acc:HGNC:1437]	chr11:14926542-15103888	NM_001033952, NN	.	NP_001029124, NP_00	XP_016873772, XP_0168	protein_coding	ENSG0000011068	796	1437
3881	DDX54	-0.527	0.031	DEAD-box helicase 54 [Source:HGNC Symbol;Acc:HGNC:20084]	chr12:113587662-113623284	NM_001111322, NN	.	NP_001104792, NP_07	.	protein_coding	ENSG0000012306	79039	20084
3882	FKBPL	-0.690	0.031	FK506 binding protein like [Source:HGNC Symbol;Acc:HGNC:13949]	chr6:32096483-32098068	NM_022110	XM_011514803	NP_071393	XP_011513105	protein_coding	ENSG0000020431	63943	13949
3883	KLHL8	0.781	0.031	kelch like family member 8 [Source:HGNC Symbol;Acc:HGNC:18644]	chr4:88081254-88141760	NM_001292003, NN	.	NP_001278932, NP_00	.	protein_coding	ENSG0000014533	57563	18644
3884	KLK1	-0.664	0.031	kallikrein 1 [Source:HGNC Symbol;Acc:HGNC:6357]	chr19:51322408-51327040	NM_002257	XM_011526942	NP_002248	XP_011525244	protein_coding	ENSG0000016774	3816	6357
3885	ARMC1	0.653	0.032	armadillo repeat containing 1 [Source:HGNC Symbol;Acc:HGNC:17684]	chr8:66514690-66546452	NM_001286702, NN	XM_017013605, XM_0	NP_001273631, NP_06	XP_005251321, XP_0067	protein_coding	ENSG0000010444	55156	17684
3886	TSPAN17	-0.538	0.032	tetraspanin 17 [Source:HGNC Symbol;Acc:HGNC:13594]	chr5:176074387-176086055	NM_001006616, NN	XM_005265876	NP_001006617, NP_03	XP_005265933	protein_coding	ENSG0000004814	26262	13594

3887	ATF2	0.840	0.032	activating transcription factor 2 [Source:HGNC Symbol;Acc:HGNC:784]	chr2:175936977-176032934	NM_001256090, N	.	NP_001243019, NP_00	.	protein_coding	ENSG0000011596	1386		784
3888	ATP6V1A	0.594	0.032	ATPase H+ transporting V1 subunit A [Source:HGNC Symbol;Acc:HGNC:851]	chr3:113465865-113530903	NM_001690	.	NP_001681	.	protein_coding	ENSG0000011457	523		851
3889	PTTG1IP	-0.494	0.032	pituitary tumor-transforming 1 interacting protein [Source:HGNC Symbol;Acc:HGNC:135	chr21:46269499-46293818	NM_001286822, N	.	NP_001273751, NP_00	.	protein_coding	ENSG0000018325	754		13524
3890	INSL3	-0.933	0.032	insulin like 3 [Source:HGNC Symbol;Acc:HGNC:6086]	chr19:17927320-17932383	NM_001265587, N	.	NP_001252516, NP_00	.	protein_coding	ENSG0000024809	3640		6086
3891	PHLPP1	0.740	0.032	PH domain and leucine rich repeat protein phosphatase 1 [Source:HGNC Symbol;Acc:H	chr18:60382682-60647666	NM_194449	.	NP_919431	.	protein_coding	ENSG0000008191	23239		20610
3892	TLX1	-0.881	0.032	T-cell leukemia homeobox 1 [Source:HGNC Symbol;Acc:HGNC:5056]	chr10:102890261-102897546	NM_001195517, N	XM_011539745, XM_	NP_001182446, NP_00	XP_011538046, XP_0115	protein_coding	ENSG0000010780	3195		5056
3893	NUDT8	-0.698	0.032	nudix hydrolase 8 [Source:HGNC Symbol;Acc:HGNC:8055]	chr11:67395408-67397401	NM_001243750, N	.	NP_001230679, NP_86	.	protein_coding	ENSG0000016779	254552		8055
3894	ZBED3	0.816	0.032	zinc finger BED-type containing 3 [Source:HGNC Symbol;Acc:HGNC:20711]	chr5:76367896-76383148	NM_032367	.	NP_001316493, NP_11	.	protein_coding	ENSG0000013284	84327		20711
3895	HK2	0.556	0.032	hexokinase 2 [Source:HGNC Symbol;Acc:HGNC:4923]	chr2:75061107-75120486	NM_000189	XM_011532807, XM_	NP_000180	XP_005264337, XP_0115	protein_coding	ENSG0000015939	3099		4923
3896	KRT15	-0.729	0.032	keratin 15 [Source:HGNC Symbol;Acc:HGNC:6421]	chr17:39669994-39678665	NM_002275	XM_011524785, XM_	NP_002266	XP_005257402, XP_0115	protein_coding	ENSG0000017134	102466658		6421
3897	SAP18	-0.501	0.032	Sin3A associated protein 18 [Source:HGNC Symbol;Acc:HGNC:10530]	chr13:21714652-21723223	NM_005870	.	NP_005861	.	protein_coding	ENSG0000015045	10284		10530
3898	NOL9	0.703	0.032	nucleolar protein 9 [Source:HGNC Symbol;Acc:HGNC:26265]	chr1:6581406-6614595	NM_024654	.	NP_078930	.	protein_coding	ENSG0000016240	79707		26265
3899	SNX19	0.610	0.032	sorting nexin 19 [Source:HGNC Symbol;Acc:HGNC:21532]	chr11:130745330-130786404	NM_001301089, N	XM_011542820, XM_	NP_001288018, NP_05	XP_005271603, XP_0115	protein_coding	ENSG0000012045	399979		21532
3900	ZFPM1	-0.744	0.032	zinc finger protein, FOG family member 1 [Source:HGNC Symbol;Acc:HGNC:19762]	chr16:88519724-88603424	NM_153813	XM_011522918	NP_722520	XP_011521214, XP_0115	protein_coding	ENSG0000017958	161882		19762
3901	LRP6	0.710	0.032	LDL receptor related protein 6 [Source:HGNC Symbol;Acc:HGNC:6698]	chr12:12268958-12419746	NM_002336	XM_017019304, XM_	NP_002327	XP_006719141, XP_0115	protein_coding	ENSG0000007001	4040		6698
3902	SENP3	-0.510	0.032	SUMO1/sentrin/SMT3 specific peptidase 3 [Source:HGNC Symbol;Acc:HGNC:17862]	chr17:7465191-7475287	NM_015670	.	NP_056485	.	protein_coding	ENSG0000016195	26168		17862
3903	GPATCH1	0.685	0.032	G-patch domain containing 1 [Source:HGNC Symbol;Acc:HGNC:24658]	chr19:33571785-33621448	NM_018025	.	NP_060495	.	protein_coding	ENSG0000007665	55094		24658
3904	NUMB	0.576	0.032	NUMB, endocytic adaptor protein [Source:HGNC Symbol;Acc:HGNC:8060]	chr14:73741814-73925323	NM_001005743, N	XM_011537264, XM_	NP_001005743, NP_00	XP_005268199, XP_0052	protein_coding	ENSG0000013396	101928143		8060
3905	LRRC41	-0.708	0.032	leucine rich repeat containing 41 [Source:HGNC Symbol;Acc:HGNC:16917]	chr1:46713366-46769280	NM_006369	.	NP_006360	.	protein_coding	ENSG0000013212	10489		16917
3906	XRN1	0.789	0.032	5'-3' exoribonuclease 1 [Source:HGNC Symbol;Acc:HGNC:30654]	chr3:142025448-142166904	NM_001282857, N	XM_011512923, XM_	NP_001269786, NP_00	XP_011511221, XP_0115	protein_coding	ENSG0000011412	54464		30654
3907	ANO1	-0.520	0.032	anoctamin 1 [Source:HGNC Symbol;Acc:HGNC:21625]	chr11:69924407-70035634	NM_018043	XM_017017957, XM_	NP_060513	XP_006718665, XP_0067	protein_coding	ENSG0000013162	55107		21625
3908	DNAJC6	0.725	0.032	DnaJ heat shock protein family (Hsp40) member C6 [Source:HGNC Symbol;Acc:HGNC:1	chr1:65730376-65881552	NM_001256864, N	XM_011542488, XM_	NP_001243793, NP_00	XP_011540788, XP_0115	protein_coding	ENSG0000011667	9829		15469
3909	RC3H1	0.891	0.032	ring finger and CCCH-type domains 1 [Source:HGNC Symbol;Acc:HGNC:29434]	chr1:173900351-173991435	NM_001300850, N	XM_011509231, XM_	NP_001287779, NP_00	XP_005244978, XP_0115	protein_coding	ENSG0000013587	149041		29434
3910	ZNF559	0.864	0.032	zinc finger protein 559 [Source:HGNC Symbol;Acc:HGNC:28197]	chr19:9434447-9493293	NM_001202406, N	.	NP_001189335, NP_00	.	protein_coding	ENSG0000018832	84527		28197
3911	PAXIP1	0.644	0.032	PAX interacting protein 1 [Source:HGNC Symbol;Acc:HGNC:8624]	chr7:154735396-154794794	NM_007349	XM_011515983, XM_	NP_031375	XP_005249596, XP_0115	protein_coding	ENSG0000015721	22976		8624
3912	MBD1	0.694	0.033	methyl-CpG binding domain protein 1 [Source:HGNC Symbol;Acc:HGNC:6916]	chr18:47793251-47808144	NM_001204136, N	XM_017025771, XM_	NP_001191065, NP_00	XP_005258328, XP_0067	protein_coding	ENSG0000014164	4152		6916
3913	MCF2L	-0.746	0.033	MCF.2 cell line derived transforming sequence like [Source:HGNC Symbol;Acc:HGNC:14	chr13:113548691-113754053	NM_001112732, N	XM_011537493, XM_	NP_001106203, NP_00	XP_005268371, XP_0052	protein_coding	ENSG0000012621	23263		14576
3914	NREP	0.761	0.033	neuronal regeneration related protein [Source:HGNC Symbol;Acc:HGNC:16834]	chr5:111004887-111333161	NM_001142474, N	XM_006714733, XM_	NP_001135946, NP_00	XP_006714795, XP_0067	protein_coding	ENSG0000013498	9315		16834
3915	RPL28	-0.671	0.033	ribosomal protein L28 [Source:HGNC Symbol;Acc:HGNC:10330]	chr19:55896712-55919145	NM_000991, NM_0	XM_005259132	NP_000982, NP_00112	XP_005259189	protein_coding	ENSG0000010810	102465483		10330
3916	STAT6	-0.842	0.033	signal transducer and activator of transcription 6 [Source:HGNC Symbol;Acc:HGNC:113	chr12:57482676-57607134	NM_001178078, N	XM_011538709, XM_	NP_001171549, NP_00	XP_006719637, XP_0067	protein_coding	ENSG0000016688	6778		11368
3917	CSNK1G2	-0.509	0.033	casein kinase 1 gamma 2 [Source:HGNC Symbol;Acc:HGNC:2455]	chr19:1941187-1981337	NM_001319	XM_005259500, XM_	NP_001310	XP_005259555, XP_0052	protein_coding	ENSG0000013327	1455		2455
3918	ENDOV	-0.803	0.033	endonuclease V [Source:HGNC Symbol;Acc:HGNC:26640]	chr17:78388966-78411886	NM_001164637, N	XM_017024516, XM_	NP_001158109, NP_00	XP_005257301, XP_0052	protein_coding	ENSG0000017381	284131		26640
3919	PATJ	0.957	0.033	PATJ, crumbs cell polarity complex component [Source:HGNC Symbol;Acc:HGNC:28881]	chr1:62208148-62644347	NM_176877	XM_011540469, XM_	NP_795352	XP_005270398, XP_0052	protein_coding	ENSG0000013284	10207		28881
3920	TMEM261	-0.539	0.033	transmembrane protein 261 [Source:HGNC Symbol;Acc:HGNC:30536]	chr9:7796489-7800067	NM_001318058, N	XM_011518062	NP_001304987, NP_21	.	protein_coding	ENSG0000013703	90871		30536
3921	DDB1	-0.576	0.033	damage specific DNA binding protein 1 [Source:HGNC Symbol;Acc:HGNC:2717]	chr11:61066922-61136981	NM_001923	.	NP_001914	.	protein_coding	ENSG0000016798	1642		2717
3922	TCERG1	0.585	0.033	transcription elongation regulator 1 [Source:HGNC Symbol;Acc:HGNC:15630]	chr5:145826874-145891524	NM_001040006, N	XM_011537548, XM_	NP_001035095, NP_00	XP_005268422, XP_0168	protein_coding	ENSG0000011364	10915		15630
3923	ZNF302	0.775	0.033	zinc finger protein 302 [Source:HGNC Symbol;Acc:HGNC:13848]	chr19:35168543-35177302	NM_001012320, N	XM_017026986, XM_	NP_001012320, NP_00	XP_011525413, XP_0115	protein_coding	ENSG0000008933	55900		13848
3924	PHACTR3	-0.877	0.033	phosphatase and actin regulator 3 [Source:HGNC Symbol;Acc:HGNC:15833]	chr20:58152563-58422766	NM_001199505, N	XM_011528526, XM_	NP_001186434, NP_00	XP_011526827, XP_0115	protein_coding	ENSG0000008749	116154		15833
3925	PURA	0.683	0.033	purine rich element binding protein A [Source:HGNC Symbol;Acc:HGNC:9701]	chr5:139493707-139505204	NM_005859	.	NP_005850	.	protein_coding	ENSG0000018512	5813		9701
3926	RAB1B	-0.493	0.033	RAB1B, member RAS oncogene family [Source:HGNC Symbol;Acc:HGNC:18370]	chr11:66036003-66044963	NM_030981	.	NP_112243	.	protein_coding	ENSG0000017490	81876		18370
3927	STX16	0.529	0.033	syntaxin 16 [Source:HGNC Symbol;Acc:HGNC:11431]	chr20:57226308-57254582	NM_001001433, N	.	NP_001001433, NP_00	.	protein_coding	ENSG0000012422	8675		11431
3928	IKBKAP	0.600	0.033	inhibitor of kappa light polypeptide gene enhancer in B-cells, kinase complex-associated	chr9:111629796-111696396	NM_001318360, N	XM_011519137, XM_	NP_001305289, NP_00	XP_011517438, XP_0115	protein_coding	ENSG0000007006	8518		5959
3929	PRPF4	0.572	0.033	pre-mRNA processing factor 4 [Source:HGNC Symbol;Acc:HGNC:17349]	chr9:116029287-116055185	NM_001244926, N	XM_011519181	NP_001231855, NP_00	.	protein_coding	ENSG0000013687	9128		17349
3930	CWC25	0.658	0.033	CWC25 spliceosome associated protein homolog [Source:HGNC Symbol;Acc:HGNC:2598	chr17:36956686-36981734	NM_017748	.	NP_060218	.	protein_coding	ENSG0000027355	54883		25989
3931	STOML1	-0.794	0.033	stomatin like 1 [Source:HGNC Symbol;Acc:HGNC:14560]	chr15:74271263-74286963	NM_001256672, N	XM_006720773, XM_	NP_001243601, NP_00	XP_005254849, XP_0067	protein_coding	ENSG0000006722	9399		14560
3932	KREMEN1	-0.531	0.033	kringle containing transmembrane protein 1 [Source:HGNC Symbol;Acc:HGNC:17550]	chr22:29469065-29564321	NM_001039570, N	XM_011530429	NP_001034659, NP_11	XP_011528731	protein_coding	ENSG0000018376	83999		17550
3933	TBC1D23	0.807	0.033	TBC1 domain family member 23 [Source:HGNC Symbol;Acc:HGNC:25622]	chr3:99979844-100044095	NM_001199198, N	XM_017006841, XM_	NP_001186127, NP_06	XP_011511276, XP_0168	protein_coding	ENSG0000003605	55773		25622
3934	R3HDM4	-0.540	0.033	R3H domain containing 4 [Source:HGNC Symbol;Acc:HGNC:28270]	chr19:896502-913245	NM_138774	XM_011528416	NP_620129	XP_011526718	protein_coding	ENSG0000019885	91300		28270
3935	TMCC2	-0.698	0.033	transmembrane and coiled-coil domain family 2 [Source:HGNC Symbol;Acc:HGNC:2423	chr1:205197303-205242471	NM_001242925, N	XM_017003000, XM_	NP_001229854, NP_00	XP_005245741, XP_0052	protein_coding	ENSG0000013306	9911		24239
3936	CYLD	0.701	0.033	CYLD lysine 63 deubiquitinase [Source:HGNC Symbol;Acc:HGNC:2584]	chr16:50775960-50835846	NM_001042355, N	XM_017022980, XM_	NP_001035814, NP_00	XP_005255869, XP_0067	protein_coding	ENSG0000008379	1540		2584
3937	KPNA6	0.545	0.033	karyopherin subunit alpha 6 [Source:HGNC Symbol;Acc:HGNC:6399]	chr1:32573638-32642169	NM_012316	XM_011541160, XM_	NP_036448	XP_005270768	protein_coding	ENSG0000002580	23633		6399
3938	TMEM79	-0.802	0.033	transmembrane protein 79 [Source:HGNC Symbol;Acc:HGNC:28196]	chr1:156252738-156262234	NM_032323	.	NP_115699	.	protein_coding	ENSG0000016347	84283		28196
3939	TRIQK	1.174	0.033	triple QxxK/R motif containing [Source:HGNC Symbol;Acc:HGNC:27828]	chr8:93895757-94029901	NM_001171795, N	XM_017013350, XM_	NP_001165266, NP_00	XP_011515284, XP_0168	protein_coding	ENSG0000020513	286144		27828
3940	GSTT1	-0.646	0.033	glutathione S-transferase theta 1 [Source:HGNC Symbol;Acc:HGNC:4641]	chr22:24376132-24384284	NM_000853, NM_0	XM_005261587	NP_000844, NP_00128	.	protein_coding	ENSG0000018467	2952		4641

3941	PRPF3	0.596	0.033	pre-mRNA processing factor 3 [Source:HGNC Symbol;Acc:HGNC:17348]	chr1:150293924-150325671	NM_004698	XM_011510129, XM_0	NP_004689	XP_011508431	protein_coding	ENSG0000011736	9129	17348
3942	LTA4H	0.525	0.033	leukotriene A4 hydrolase [Source:HGNC Symbol;Acc:HGNC:6710]	chr12:96394605-96437298	NM_000895, NM_0	XM_011538348, XM_0	NP_000886, NP_00124	XP_005268928, XP_0115	protein_coding	ENSG0000011114	4048	6710
3943	SLC41A2	0.803	0.033	solute carrier family 41 member 2 [Source:HGNC Symbol;Acc:HGNC:31045]	chr12:105196330-105322433	NM_032148	XM_017020013, XM_0	NP_115524	XP_005269233, XP_0052	protein_coding	ENSG0000013605	84102	31045
3944	MGRN1	-0.623	0.033	mahogunin ring finger 1 [Source:HGNC Symbol;Acc:HGNC:20254]	chr16:4674790-4740975	NM_001142289, NN	XM_011522441, XM_0	NP_001135761, NP_00	XP_005255274, XP_0052	protein_coding	ENSG0000010285	23295	20254
3945	CAPN5	-0.644	0.033	calpain 5 [Source:HGNC Symbol;Acc:HGNC:1482]	chr11:76777978-76837201	NM_004055	XM_017018223, XM_0	NP_004046	XP_011543527, XP_0168	protein_coding	ENSG0000014926	726	1482
3946	GCH1	0.686	0.033	GTP cyclohydrolase 1 [Source:HGNC Symbol;Acc:HGNC:4193]	chr14:55308725-55369570	NM_000161, NM_0	XM_005267530	NP_000152, NP_00101	XP_005267587	protein_coding	ENSG0000013197	2643	4193
3947	MAP3K9	0.608	0.033	mitogen-activated protein kinase kinase kinase 9 [Source:HGNC Symbol;Acc:HGNC:6861	chr14:71189242-71276251	NM_001284230, NN	XM_011536793, XM_0	NP_001271159, NP_00	XP_005267740, XP_0115	protein_coding	ENSG0000000643	4293	6861
3948	RNF7	-0.604	0.033	ring finger protein 7 [Source:HGNC Symbol;Acc:HGNC:10070]	chr3:141457045-141466402	NM_001201370, NN	.	NP_001188299, NP_05	.	protein_coding	ENSG0000011412	9616	10070
3949	SH3KBP1	-0.556	0.033	SH3 domain containing kinase binding protein 1 [Source:HGNC Symbol;Acc:HGNC:13867	chrX:19552092-19905719	NM_001024666, NN	XM_011545503, XM_0	NP_001019837, NP_00	XP_005274551, XP_0115	protein_coding	ENSG0000014701	30011	13867
3950	TYW3	1.091	0.033	tRNA-yW synthesizing protein 3 homolog [Source:HGNC Symbol;Acc:HGNC:24757]	chr1:75171165-75232358	NM_001162916, NN	XM_006710347	NP_001156388, NP_61	XP_006710410	protein_coding	ENSG0000016262	127253	24757
3951	DALRD3	-1.534	0.033	DALR anticodon binding domain containing 3 [Source:HGNC Symbol;Acc:HGNC:25536]	chr3:49027318-49060928	NM_001009996, NN	XM_011533893, XM_0	NP_001009996, NP_00	XP_005265326, XP_0067	protein_coding	ENSG0000017814	55152	25536
3952	MAP7	0.656	0.033	microtubule associated protein 7 [Source:HGNC Symbol;Acc:HGNC:6869]	chr6:136663418-136871957	NM_001198608, NN	XM_011536246, XM_0	NP_001185537, NP_00	XP_006715661, XP_0067	protein_coding	ENSG0000013552	9053	6869
3953	AAMP	-0.490	0.034	angio associated migratory cell protein [Source:HGNC Symbol;Acc:HGNC:18]	chr2:219128845-219134980	NM_001087, NM_0	.	NP_001078, NP_00128	.	protein_coding	ENSG0000012783	14	18
3954	ITGB8	0.988	0.034	integrin subunit beta 8 [Source:HGNC Symbol;Acc:HGNC:6163]	chr7:20370324-20455377	NM_002214	XM_017012183, XM_0	NP_002205	XP_011513695, XP_0115	protein_coding	ENSG0000010585	3696	6163
3955	PDGFB	-0.510	0.034	platelet derived growth factor subunit B [Source:HGNC Symbol;Acc:HGNC:8800]	chr22:39619363-39640756	NM_002608, NM_0	.	NP_002599, NP_14893	.	protein_coding	ENSG0000010031	5155	8800
3956	OSBPL1A	0.638	0.034	oxysterol binding protein like 1A [Source:HGNC Symbol;Acc:HGNC:16398]	chr18:21742007-21977823	NM_001242508, NN	XM_006722383, XM_0	NP_001229437, NP_06	XP_006722443, XP_0067	protein_coding	ENSG0000014144	114876	16398
3957	ERICH2	0.928	0.034	glutamate rich 2 [Source:HGNC Symbol;Acc:HGNC:44395]	chr2:171640295-171655481	NM_001289947	.	NP_001276876	.	protein_coding	ENSG0000020433	285141	44395
3958	INTS8	1.040	0.034	integrator complex subunit 8 [Source:HGNC Symbol;Acc:HGNC:26048]	chr8:95835526-96070938	NM_017864	XM_011517157, XM_0	NP_060334	XP_016869105	protein_coding	ENSG0000016494	55656	26048
3959	RPS3	-0.605	0.034	ribosomal protein S3 [Source:HGNC Symbol;Acc:HGNC:10420]	chr11:75110529-75133324	NM_001005, NM_0	.	NP_000996, NP_00124	.	protein_coding	ENSG0000014927	6188	10420
3960	RNF212	0.927	0.034	ring finger protein 212 [Source:HGNC Symbol;Acc:HGNC:27729]	chr4:1065265-1107350	NM_001131034, NN	XM_011513443, XM_0	NP_001124506, NP_00	XP_011511741, XP_0115	protein_coding	ENSG0000017822	285498	27729
3961	SRCIN1	-0.819	0.034	SRC kinase signaling inhibitor 1 [Source:HGNC Symbol;Acc:HGNC:29506]	chr17:36686250-36762183	NM_025248	XM_017025172, XM_0	NP_079524	XP_016880660, XP_0168	protein_coding	ENSG0000027736	80725	29506
3962	KLHL2	1.192	0.034	kelch like family member 2 [Source:HGNC Symbol;Acc:HGNC:6353]	chr4:165991759-166244308	NM_001161521, NN	XM_017007676, XM_0	NP_001154993, NP_00	XP_005262767, XP_0115	protein_coding	ENSG0000010946	11275	6353
3963	MEN1	-0.540	0.034	menin 1 [Source:HGNC Symbol;Acc:HGNC:7010]	chr11:64570981-64578766	NM_000244, NM_1	XM_017017769, XM_0	NP_000235, NP_57071	XP_005274058, XP_0115	protein_coding	ENSG0000013389	4221	7010
3964	PRR5	-1.219	0.034	proline rich 5 [Source:HGNC Symbol;Acc:HGNC:31682]	chr22:45064426-45258668	NM_001017528, NN	.	NP_001017528, NP_00	.	protein_coding	ENSG0000018665	55615	31682
3965	TNFRSF1A	-0.557	0.034	TNF receptor superfamily member 1A [Source:HGNC Symbol;Acc:HGNC:11916]	chr12:6437922-6451280	NM_001065	.	NP_001056	.	protein_coding	ENSG0000006718	7132	11916
3966	VAMP2	-0.800	0.034	vesicle associated membrane protein 2 [Source:HGNC Symbol;Acc:HGNC:12643]	chr17:8043789-8066864	NM_014232	XM_005256775	NP_055047	XP_005256832	protein_coding	ENSG0000022020	6844	12643
3967	C17orf80	1.307	0.034	chromosome 17 open reading frame 80 [Source:HGNC Symbol;Acc:HGNC:29601]	chr17:71188770-71258491	NM_001100621, NN	XM_017024806, XM_0	NP_001094091, NP_00	XP_005257544, XP_0067	protein_coding	ENSG0000014121	55028	29601
3968	C21orf91	1.086	0.034	chromosome 21 open reading frame 91 [Source:HGNC Symbol;Acc:HGNC:16459]	chr21:19161283-19191703	NM_001100420, NN	.	NP_001093890, NP_00	.	protein_coding	ENSG0000015464	54149	16459
3969	CCPG1	1.085	0.034	cell cycle progression 1 [Source:HGNC Symbol;Acc:HGNC:24227]	chr15:55609381-55700708	NM_001204450, NN	.	NP_001191379, NP_00	.	protein_coding	ENSG0000026091	693213	24227
3970	YDJC	-0.550	0.034	YdjC homolog (bacterial) [Source:HGNC Symbol;Acc:HGNC:27158]	chr22:21982377-21984353	NM_001017964	XM_011529906	NP_001017964	XP_011528208	protein_coding	ENSG0000016117	150223	27158
3971	KIF1B	0.599	0.034	kinesin family member 1B [Source:HGNC Symbol;Acc:HGNC:16636]	chr1:10270862-10441661	NM_015074, NM_1	.	NP_055889, NP_90432	.	protein_coding	ENSG0000005452	23095	16636
3972	LAMC1	0.525	0.034	laminin subunit gamma 1 [Source:HGNC Symbol;Acc:HGNC:6492]	chr1:182992594-183114727	NM_002293	.	NP_002284	.	protein_coding	ENSG0000013586	3915	6492
3973	RASL11A	-0.858	0.034	RAS like family 11 member A [Source:HGNC Symbol;Acc:HGNC:23802]	chr13:27844463-27847827	NM_206827	.	NP_996563	.	protein_coding	ENSG0000012203	387496	23802
3974	ANKRD10	-0.650	0.034	ankyrin repeat domain 10 [Source:HGNC Symbol;Acc:HGNC:20265]	chr13:111530886-111567416	NM_001286721, NN	.	NP_001273650, NP_06	.	protein_coding	ENSG0000008844	55608	20265
3975	ACBD4	-0.705	0.034	acyl-CoA binding domain containing 4 [Source:HGNC Symbol;Acc:HGNC:23337]	chr17:43209966-43221548	NM_001135704, NN	XM_017025085, XM_0	NP_001129176, NP_00	XP_005257734, XP_0067	protein_coding	ENSG0000018151	79777	23337
3976	CDKAL1	0.717	0.034	CDK5 regulatory subunit associated protein 1 like 1 [Source:HGNC Symbol;Acc:HGNC:21	chr6:20534687-21232635	NM_017774	XM_011514718	NP_060244	XP_011513020	protein_coding	ENSG0000014599	54901	21050
3977	EBPL	-0.637	0.034	emopamil binding protein like [Source:HGNC Symbol;Acc:HGNC:18061]	chr13:50234858-50265611	NM_001278636, NN	.	NP_001265565, NP_11	.	protein_coding	ENSG0000012317	84650	18061
3978	PML	-0.629	0.034	promyelocytic leukemia [Source:HGNC Symbol;Acc:HGNC:9113]	chr15:74287013-74340153	NM_002675, NM_0	.	NP_002666, NP_15024	.	protein_coding	ENSG0000014046	5371	9113
3979	GFOD1	0.753	0.034	glucose-fructose oxidoreductase domain containing 1 [Source:HGNC Symbol;Acc:HGNC:	chr6:13358061-13487894	NM_001242628, NN	XM_011514699	NP_001229557, NP_00	XP_011513001	protein_coding	ENSG0000014599	54438	21096
3980	SSH3	-0.550	0.034	slingshot protein phosphatase 3 [Source:HGNC Symbol;Acc:HGNC:30581]	chr11:67070918-67080076	NM_017857	XM_017017942, XM_0	NP_060327	XP_016873431	protein_coding	ENSG0000017283	54961	30581
3981	DCPS	-0.580	0.034	decapping enzyme, scavenger [Source:HGNC Symbol;Acc:HGNC:29812]	chr11:126173646-126215644	NM_014026	XM_011542778	NP_054745	XP_011541080	protein_coding	ENSG0000011006	28960	29812
3982	DNAJB4	0.769	0.034	DnaJ heat shock protein family (Hsp40) member B4 [Source:HGNC Symbol;Acc:HGNC:14	chr1:78470537-78483648	NM_001317099, NN	.	NP_001304028, NP_00	.	protein_coding	ENSG0000016261	11080	14886
3983	PDS5B	0.685	0.034	PDS5 cohesin associated factor B [Source:HGNC Symbol;Acc:HGNC:20418]	chr13:33160563-33352157	NM_015032	XM_017020451, XM_0	NP_055847	XP_005266355, XP_0115	protein_coding	ENSG0000008364	23047	20418
3984	TSC22D2	0.730	0.034	TSC22 domain family member 2 [Source:HGNC Symbol;Acc:HGNC:29095]	chr3:150126121-150184218	NM_014779	.	NP_001290193, NP_05	.	protein_coding	ENSG0000019642	9819	29095
3985	FAM132A	-1.588	0.034	family with sequence similarity 132 member A [Source:HGNC Symbol;Acc:HGNC:32308]	chr1:1177832-1182102	NM_001014980	XM_011541436, XM_0	NP_001014980	XP_011539737, XP_0115	protein_coding	ENSG0000018416	388581	32308
3986	LUC7L3	0.960	0.034	LUC7 like 3 pre-mRNA splicing factor [Source:HGNC Symbol;Acc:HGNC:24309]	chr17:48796904-48833574	NM_006107, NM_0	XM_005257454, XM_0	NP_006098, NP_05750	XP_005257505, XP_0052	protein_coding	ENSG0000010884	51747	24309
3987	NKX2-1	-1.046	0.034	NK2 homeobox 1 [Source:HGNC Symbol;Acc:HGNC:11825]	chr14:36985596-36990354	NM_001079668, NN	.	NP_001073136, NP_00	.	protein_coding	ENSG0000013635	7080	11825
3988	PPP1R12C	-0.539	0.034	protein phosphatase 1 regulatory subunit 12C [Source:HGNC Symbol;Acc:HGNC:14947]	chr19:55602280-55628927	NM_001271618, NN	XM_011527045, XM_0	NP_001258547, NP_06	XP_005259070, XP_0115	protein_coding	ENSG0000012550	54776	14947
3989	ETV5	-0.491	0.034	ETS variant 5 [Source:HGNC Symbol;Acc:HGNC:3494]	chr3:185764096-185826901	NM_004454	.	NP_004445	.	protein_coding	ENSG0000024440	2119	3494
3990	DDN	-0.670	0.034	dendrin [Source:HGNC Symbol;Acc:HGNC:24458]	chr12:49388931-49393092	NM_015086	.	NP_055901	XP_011536357	protein_coding	ENSG0000018141	23109	24458
3991	PPP2R3A	0.907	0.034	protein phosphatase 2 regulatory subunit B''alpha [Source:HGNC Symbol;Acc:HGNC:930	chr3:135684514-135866733	NM_001190447, NN	XM_011512956, XM_0	NP_001177376, NP_00	XP_006713749, XP_0115	protein_coding	ENSG0000007371	5523	9307
3992	UVRAG	0.680	0.034	UV radiation resistance associated [Source:HGNC Symbol;Acc:HGNC:12640]	chr11:75526211-75854239	NM_003369	XM_011545226	NP_003360	XP_011543528	protein_coding	ENSG0000019838	7405	12640
3993	AGTRAP	-0.573	0.035	angiotensin II receptor associated protein [Source:HGNC Symbol;Acc:HGNC:13539]	chr1:11796140-11810828	NM_001040194, NN	XM_011541802, XM_0	NP_001035284, NP_00	XP_011540102, XP_0115	protein_coding	ENSG0000017767	57085	13539
3994	DPF1	-0.769	0.035	double PHD fingers 1 [Source:HGNC Symbol;Acc:HGNC:20225]	chr19:38701645-38720354	NM_001135155, NN	XM_011527358, XM_0	NP_001128627, NP_00	XP_005259346, XP_0052	protein_coding	ENSG0000001133	8193	20225

3995	FBXO34	0.657	0.035	F-box protein 34 [Source:HGNC Symbol;Acc:HGNC:20201]	chr14:55738020-55820327	NM_017943, NM_1	XM_017021391, XM_0	NP_060413, NP_68941	XP_005267848, XP_0067	protein_coding	ENSG0000017897	55030	20201
3996	HAUS3	0.917	0.035	HAUS augmin like complex subunit 3 [Source:HGNC Symbol;Acc:HGNC:28719]	chr4:2073644-2243891	NM_001303143, NN	.	NP_001290072, NP_07	.	protein_coding	ENSG0000021436	79441	28719
3997	KIAA0226L	0.755	0.035	RUN and cysteine rich domain containing beclin 1 interacting protein like [Source:HGNC	chr13:46916134-47012325	NM_001286761, NN	.	NP_001273690, NP_00	.	protein_coding	ENSG0000010244	80183	20420
3998	STK3	0.730	0.035	serine/threonine kinase 3 [Source:HGNC Symbol;Acc:HGNC:11406]	chr8:99466858-99954799	NM_001256312, NN	XM_011517250, XM_0	NP_001243241, NP_00	XP_011515550	protein_coding	ENSG0000010437	6788	11406
3999	LONRF1	0.799	0.035	LON peptidase N-terminal domain and ring finger 1 [Source:HGNC Symbol;Acc:HGNC:26	chr8:12579402-12612999	NM_152271	XM_011544694, XM_0	NP_689484	XP_005273742, XP_0115	protein_coding	ENSG0000015435	91694	26302
4000	MAGEF1	-0.516	0.035	MAGE family member F1 [Source:HGNC Symbol;Acc:HGNC:29639]	chr3:184428154-184429790	NM_022149	.	NP_071432	.	protein_coding	ENSG0000017738	64110	29639
4001	UBL4A	-0.499	0.035	ubiquitin like 4A [Source:HGNC Symbol;Acc:HGNC:12505]	chrX:153712055-153715009	NM_014235	.	NP_055050	.	protein_coding	ENSG0000010217	8266	12505
4002	CA8	0.776	0.035	carbonic anhydrase 8 [Source:HGNC Symbol;Acc:HGNC:1382]	chr8:61099905-61193971	NM_004056	XM_011517586	NP_001308766, NP_00	.	protein_coding	ENSG0000017853	767	1382
4003	LMBRD2	0.881	0.035	LMBR1 domain containing 2 [Source:HGNC Symbol;Acc:HGNC:25287]	chr5:36098513-36152063	NM_001007527	XM_011514162	NP_001007528	XP_011512464	protein_coding	ENSG0000016418	92255	25287
4004	AP2S1	-0.526	0.035	adaptor related protein complex 2 sigma 1 subunit [Source:HGNC Symbol;Acc:HGNC:56	chr19:47341392-47354249	NM_001301076, NN	XM_011526424, XM_0	NP_001288005, NP_00	XP_011524725, XP_0115	protein_coding	ENSG0000004275	1175	565
4005	GRK3	0.858	0.035	G protein-coupled receptor kinase 3 [Source:HGNC Symbol;Acc:HGNC:290]	chr22:25960815-26125261	NM_005160	XM_011529975, XM_0	NP_005151	XP_011528277	protein_coding	ENSG0000010007	157	290
4006	DPH1	-0.775	0.035	diphthamide biosynthesis 1 [Source:HGNC Symbol;Acc:HGNC:3003]	chr17:1933403-1946724	NM_001383	.	NP_001374	.	protein_coding	ENSG0000010896	1801	3003
4007	TIPARP	0.577	0.035	TCDD inducible poly(ADP-ribose) polymerase [Source:HGNC Symbol;Acc:HGNC:23696]	chr3:156391023-156424559	NM_001184717, NN	XM_011512668	NP_001171646, NP_00	XP_011510970	protein_coding	ENSG0000016365	25976	23696
4008	BRMS1	-0.519	0.035	breast cancer metastasis suppressor 1 [Source:HGNC Symbol;Acc:HGNC:17262]	chr11:66104803-66112596	NM_001024957, NN	.	NP_001020128, NP_05	.	protein_coding	ENSG0000017474	25855	17262
4009	DRAP1	-0.490	0.035	DR1 associated protein 1 [Source:HGNC Symbol;Acc:HGNC:3019]	chr11:65686727-65689032	NM_006442	.	NP_006433	.	protein_coding	ENSG0000017555	10589	3019
4010	IFT81	0.868	0.035	intraflagellar transport 81 [Source:HGNC Symbol;Acc:HGNC:14313]	chr12:110562139-110656602	NM_001143779, NN	XM_011538233, XM_0	NP_001137251, NP_05	XP_011536534, XP_0168	protein_coding	ENSG0000012297	28981	14313
4011	POLR2B	1.185	0.035	RNA polymerase II subunit B [Source:HGNC Symbol;Acc:HGNC:9188]	chr4:57829535-57976551	NM_000938, NM_0	.	NP_000929, NP_00129	.	protein_coding	ENSG0000004731	5431	9188
4012	BCAP31	-0.491	0.035	B-cell receptor-associated protein 31 [Source:HGNC Symbol;Acc:HGNC:16695]	chrX:152965951-152990152	NM_001139441, NN	.	NP_001132913, NP_00	.	protein_coding	ENSG0000018582	10134	16695
4013	DDX47	0.518	0.035	DEAD-box helicase 47 [Source:HGNC Symbol;Acc:HGNC:18682]	chr12:12966249-12982915	NM_016355, NM_2	.	NP_057439, NP_95751	.	protein_coding	ENSG0000021378	51202	18682
4014	TFB2M	0.679	0.035	transcription factor B2, mitochondrial [Source:HGNC Symbol;Acc:HGNC:18559]	chr1:246703861-246729626	NM_022366	.	NP_071761	.	protein_coding	ENSG0000016285	64216	18559
4015	BARD1	0.908	0.035	BRCA1 associated RING domain 1 [Source:HGNC Symbol;Acc:HGNC:952]	chr2:215590369-215674435	NM_000465, NM_0	XM_017004613, XM_0	NP_000456, NP_00126	XP_016860102	protein_coding	ENSG0000013837	580	952
4016	MARS2	0.622	0.035	methionyl-tRNA synthetase 2, mitochondrial [Source:HGNC Symbol;Acc:HGNC:25133]	chr2:198570092-198573111	NM_138395	.	NP_612404	.	protein_coding	ENSG0000024762	92935	25133
4017	MIR1199	-0.684	0.035	MISP family member 3 [Source:HGNC Symbol;Acc:HGNC:26963]	chr19:14183347-14185874	NM_001291291	.	NP_001278220	.	protein_coding	ENSG0000014185	102466515	26963
4018	MINK1	-0.496	0.035	misshapen like kinase 1 [Source:HGNC Symbol;Acc:HGNC:17565]	chr17:4736682-4806369	NM_001024937, NN	XM_011523908, XM_0	NP_001020108, NP_00	XP_006721594, XP_0067	protein_coding	ENSG0000014150	50488	17565
4019	MYOM3	-0.703	0.035	myomesin 3 [Source:HGNC Symbol;Acc:HGNC:26679]	chr1:24382524-24438665	NM_152372	.	NP_689585	.	protein_coding	ENSG0000014266	127294	26679
4020	HPDL	-0.604	0.035	4-hydroxyphenylpyruvate dioxygenase like [Source:HGNC Symbol;Acc:HGNC:28242]	chr1:45792576-45794205	NM_032756	.	NP_116145	.	protein_coding	ENSG0000018660	84842	28242
4021	NKD2	-0.569	0.035	naked cuticle homolog 2 [Source:HGNC Symbol;Acc:HGNC:17046]	chr5:1008943-1039058	NM_001271082, NN	XM_011514150, XM_0	NP_001258011, NP_14	XP_005248439, XP_0052	protein_coding	ENSG0000014550	85409	17046
4022	ZNF71	-0.684	0.035	zinc finger protein 71 [Source:HGNC Symbol;Acc:HGNC:13141]	chr19:57106667-57137849	NM_021216	.	NP_067039	XP_016882569	protein_coding	ENSG0000019795	58491	13141
4023	ZBTB22	-0.674	0.035	zinc finger and BTB domain containing 22 [Source:HGNC Symbol;Acc:HGNC:13085]	chr6:33282182-33285719	NM_001145338, NN	.	NP_001138810, NP_00	.	protein_coding	ENSG0000023610	9278	13085
4024	CPSF3L	-0.575	0.035	cleavage and polyadenylation specific factor 3-like [Source:HGNC Symbol;Acc:HGNC:26	chr1:1243946-1260071	NM_001256456, NN	XM_011541650, XM_0	NP_001243385, NP_00	XP_011539949, XP_0115	protein_coding	ENSG0000012705	102465435	26052
4025	FASTK	-0.603	0.035	Fas activated serine/threonine kinase [Source:HGNC Symbol;Acc:HGNC:24676]	chr7:150773679-150777949	NM_001258461, NN	XM_011515763, XM_0	NP_001245390, NP_00	XP_005249989, XP_0052	protein_coding	ENSG0000016489	10922	24676
4026	MSANTD4	0.880	0.035	Myb/SANT DNA binding domain containing 4 with coiled-coils [Source:HGNC Symbol;Ac	chr11:105878628-105892954	NM_032424	XM_011543022, XM_0	NP_001305676, NP_00	XP_005271754, XP_0052	protein_coding	ENSG0000017090	84437	29383
4027	RBMX	0.487	0.035	RNA binding motif protein, X-linked [Source:HGNC Symbol;Acc:HGNC:9910]	chrX:135951345-135962923	NM_001164803, NN	.	NP_001158275, NP_00	.	protein_coding	ENSG0000014727	27316	9910
4028	PPP1R14B	-0.507	0.035	protein phosphatase 1 regulatory inhibitor subunit 14B [Source:HGNC Symbol;Acc:HGN	chr11:64011951-64014413	NM_138689	.	NP_619634	.	protein_coding	ENSG0000017345	26472	9057
4029	NPHP3	1.123	0.035	nephrocystin 3 [Source:HGNC Symbol;Acc:HGNC:7907]	chr3:132400471-132441286	NM_153240	.	NP_694972	.	protein_coding	ENSG0000011397	27031	7907
4030	FANCG	-0.522	0.035	Fanconi anemia complementation group G [Source:HGNC Symbol;Acc:HGNC:3588]	chr9:35073834-35080013	NM_004629	.	NP_004620	.	protein_coding	ENSG0000022182	2189	3588
4031	ASPHD1	-0.643	0.036	aspartate beta-hydroxylase domain containing 1 [Source:HGNC Symbol;Acc:HGNC:2738	chr16:29911699-29917377	NM_181718	.	NP_859069	.	protein_coding	ENSG0000017493	253982	27380
4032	CSNK1G1	0.784	0.036	casein kinase 1 gamma 1 [Source:HGNC Symbol;Acc:HGNC:2454]	chr15:64457715-64648458	NM_001329606, NN	.	NP_001316534, NP_00	.	protein_coding	ENSG0000016911	53944	2454
4033	RPS11	-0.503	0.036	ribosomal protein S11 [Source:HGNC Symbol;Acc:HGNC:10384]	chr19:49999630-50002946	NM_001015	.	NP_001006	.	protein_coding	ENSG0000014253	6205	10384
4034	SH3BGRL	-0.980	0.036	SH3 domain binding glutamate rich protein like [Source:HGNC Symbol;Acc:HGNC:10823	chrX:80457441-80554046	NM_003022	.	NP_003013	.	protein_coding	ENSG0000013117	6451	10823
4035	ZNF566	0.861	0.036	zinc finger protein 566 [Source:HGNC Symbol;Acc:HGNC:25919]	chr19:36936020-36980763	NM_001145343, NN	XM_017027400, XM_0	NP_001138815, NP_00	XP_005259413, XP_0067	protein_coding	ENSG0000018601	84924	25919
4036	ZNF468	1.018	0.036	zinc finger protein 468 [Source:HGNC Symbol;Acc:HGNC:33105]	chr19:53300661-53360902	NM_001008801, NN	XM_017027444	NP_001008801, NP_00	XP_016882932, XP_0168	protein_coding	ENSG0000020460	90333	33105
4037	ATP13A1	-0.597	0.036	ATPase 13A1 [Source:HGNC Symbol;Acc:HGNC:24215]	chr19:19756008-19774502	NM_020410	XM_011528141, XM_0	NP_065143	.	protein_coding	ENSG0000010572	57130	24215
4038	EPB41	0.608	0.036	erythrocyte membrane protein band 4.1 [Source:HGNC Symbol;Acc:HGNC:3377]	chr1:29213602-29450447	NM_001166005, NN	XM_011540966, XM_0	NP_001159477, NP_00	XP_005245810, XP_0052	protein_coding	ENSG0000015902	2035	3377
4039	RAB11FIP3	-0.582	0.036	RAB11 family interacting protein 3 [Source:HGNC Symbol;Acc:HGNC:17224]	chr16:475618-573011	NM_001142272, NN	XM_011522765, XM_0	NP_001135744, NP_05	XP_005255770, XP_0052	protein_coding	ENSG0000009056	9727	17224
4040	RCN3	-0.751	0.036	reticulocalbin 3 [Source:HGNC Symbol;Acc:HGNC:21145]	chr19:50030874-50046895	NM_020650	XM_011527143	NP_065701	XP_011525445, XP_0168	protein_coding	ENSG0000014255	57333	21145
4041	ZC3H14	0.666	0.036	zinc finger CCCH-type containing 14 [Source:HGNC Symbol;Acc:HGNC:20509]	chr14:89029252-89259096	NM_001160103, NN	XM_011537161, XM_0	NP_001153575, NP_00	XP_005268124, XP_0052	protein_coding	ENSG0000010072	79882	20509
4042	AKT1S1	-0.685	0.036	AKT1 substrate 1 [Source:HGNC Symbol;Acc:HGNC:28426]	chr19:50372289-50392006	NM_001098632, NN	.	NP_001092102, NP_00	.	protein_coding	ENSG0000020467	84335	28426
4043	TONSL	-0.600	0.036	tonsoku-like, DNA repair protein [Source:HGNC Symbol;Acc:HGNC:7801]	chr8:145654190-145669827	NM_013432	XM_011517047	NP_038460	.	protein_coding	ENSG0000016094	4796	7801
4044	MANEA	0.826	0.036	mannosidase endo-alpha [Source:HGNC Symbol;Acc:HGNC:21072]	chr6:96025418-96057333	NM_024641	XM_005267147	NP_078917	XP_005267204	protein_coding	ENSG0000017246	79694	21072
4045	RING1	-0.605	0.036	ring finger protein 1 [Source:HGNC Symbol;Acc:HGNC:10018]	chr6:33176271-33180499	NM_002931	.	NP_002922	.	protein_coding	ENSG0000020422	6015	10018
4046	RNF217	1.038	0.036	ring finger protein 217 [Source:HGNC Symbol;Acc:HGNC:21487]	chr6:125283690-125413779	NM_001286398, NN	XM_011535500, XM_0	NP_001273327, NP_68	XP_011533796, XP_0115	protein_coding	ENSG0000014637	154214	21487
4047	TRIB3	-0.506	0.036	tribbles pseudokinase 3 [Source:HGNC Symbol;Acc:HGNC:16228]	chr20:361260-378203	NM_001301188, NN	.	NP_001288117, NP_00	XP_016883478	protein_coding	ENSG0000010125	57761	16228
4048	ETNK2	-0.658	0.036	ethanolamine kinase 2 [Source:HGNC Symbol;Acc:HGNC:25575]	chr1:204100189-204121131	NM_001297760, NN	XM_011509715, XM_0	NP_001284689, NP_00	XP_011508017	protein_coding	ENSG0000014384	55224	25575

4049	STRBP	0.604	0.036	spermatid perinuclear RNA binding protein [Source:HGNC Symbol;Acc:HGNC:16462]	chr9:125883909-126030855	NM_001171137, N	XM_011518842, XM_0	NP_001164608, NP_06	XP_011517142, XP_0115	protein_coding	ENSG0000016520	55342	16462
4050	ZNF558	0.705	0.036	zinc finger protein 558 [Source:HGNC Symbol;Acc:HGNC:26422]	chr19:8916845-8943004	NM_144693	.	NP_653294	.	protein_coding	ENSG0000016778	148156	26422
4051	CRB2	-0.844	0.036	crumbs 2, cell polarity complex component [Source:HGNC Symbol;Acc:HGNC:18688]	chr9:126118448-126141032	NM_173689	XM_011518558, XM_0	NP_775960	XP_005251991, XP_0115	protein_coding	ENSG0000014820	286204	18688
4052	ETV3	0.507	0.036	ETS variant 3 [Source:HGNC Symbol;Acc:HGNC:3492]	chr1:157090982-157108266	NM_001145312, N	XM_006711210	NP_001138784, NP_00	XP_006711273	protein_coding	ENSG0000011703	2117	3492
4053	VMO1	-1.003	0.036	vitelline membrane outer layer 1 homolog [Source:HGNC Symbol;Acc:HGNC:30387]	chr17:4688579-4689728	NM_001144939, N	.	NP_001138411, NP_00	.	protein_coding	ENSG0000018285	284013	30387
4054	C11orf54	1.067	0.036	chromosome 11 open reading frame 54 [Source:HGNC Symbol;Acc:HGNC:30204]	chr11:93468732-93497915	NM_001286067, N	XM_011542782, XM_0	NP_001272996, NP_00	XP_005273992, XP_0067	protein_coding	ENSG0000018291	28970	30204
4055	FAM173A	-0.693	0.036	family with sequence similarity 173 member A [Source:HGNC Symbol;Acc:HGNC:14152]	chr16:770974-776473	NM_001271285, N	.	NP_001258214, NP_07	.	protein_coding	ENSG0000010325	65990	14152
4056	POPDC3	0.780	0.036	popeye domain containing 3 [Source:HGNC Symbol;Acc:HGNC:17649]	chr6:105606154-105627735	NM_022361	XM_011536070, XM_0	NP_071756	XP_011534369, XP_0115	protein_coding	ENSG0000013242	64208	17649
4057	RTTN	0.831	0.036	rotatin [Source:HGNC Symbol;Acc:HGNC:18654]	chr18:67671028-67873181	NM_001318520, N	XM_017025694, XM_0	NP_001305449, NP_77	XP_006722497, XP_0067	protein_coding	ENSG0000017622	25914	18654
4058	LEF1	0.653	0.036	lymphoid enhancer binding factor 1 [Source:HGNC Symbol;Acc:HGNC:6551]	chr4:108968700-109090112	NM_001130713, N	XM_005263046	NP_001124185, NP_00	XP_005263103	protein_coding	ENSG0000013879	51176	6551
4059	EWSR1	-0.502	0.036	EWS RNA binding protein 1 [Source:HGNC Symbol;Acc:HGNC:3508]	chr22:29663997-29696515	NM_001163285, N	XM_011530002, XM_0	NP_001156757, NP_00	XP_005261446, XP_0052	protein_coding	ENSG0000018294	2130	3508
4060	USP7	0.533	0.037	ubiquitin specific peptidase 7 [Source:HGNC Symbol;Acc:HGNC:12630]	chr16:8985950-9057341	NM_001286457, N	.	NP_001273386, NP_00	XP_016879141, XP_0168	protein_coding	ENSG0000018755	7874	12630
4061	ZNF417	0.869	0.037	zinc finger protein 417 [Source:HGNC Symbol;Acc:HGNC:20646]	chr19:58418213-58427959	NM_001297734, N	.	NP_001284663, NP_68	XP_016881804	protein_coding	ENSG0000017348	147687	20646
4062	ENC1	1.170	0.037	ectodermal-neural cortex 1 [Source:HGNC Symbol;Acc:HGNC:3345]	chr5:73923230-74162663	NM_001256574, N	XM_011543697, XM_0	NP_001243503, NP_00	XP_011541998, XP_0115	protein_coding	ENSG0000017161	8507	3345
4063	GNPTG	-0.759	0.037	N-acetylglucosamine-1-phosphate transferase gamma subunit [Source:HGNC Symbol;Ac	chr16:1401923-1464752	NM_032520	.	NP_115909	XP_016879271	protein_coding	ENSG0000009058	84572	23026
4064	PLEKHF1	-0.694	0.037	pleckstrin homology and FYVE domain containing 1 [Source:HGNC Symbol;Acc:HGNC:20	chr19:30155962-30166364	NM_024310	XM_011527309, XM_0	NP_077286	XP_006723441, XP_0115	protein_coding	ENSG0000016628	79156	20764
4065	USP27X	-0.775	0.037	ubiquitin specific peptidase 27, X-linked [Source:HGNC Symbol;Acc:HGNC:13486]	chrX:49644550-49647169	NM_001145073	.	NP_001138545	.	protein_coding	ENSG0000027382	389856	13486
4066	SMPD2	-0.631	0.037	sphingomyelin phosphodiesterase 2 [Source:HGNC Symbol;Acc:HGNC:11121]	chr6:109711417-109765122	NM_003080	XM_005267109	NP_003071	XP_005267166	protein_coding	ENSG0000013558	6610	11121
4067	AKAP7	0.957	0.037	A-kinase anchoring protein 7 [Source:HGNC Symbol;Acc:HGNC:377]	chr6:131456805-131604675	NM_004842, NM_0	XM_017011510, XM_0	NP_004833, NP_05746	XP_005267285, XP_0052	protein_coding	ENSG0000011850	9465	377
4068	SLC11A2	0.606	0.037	solute carrier family 11 member 2 [Source:HGNC Symbol;Acc:HGNC:10908]	chr12:51373317-51422349	NM_000617, NM_0	XM_017019355, XM_0	NP_000608, NP_00116	XP_005268968, XP_0052	protein_coding	ENSG0000011091	4891	10908
4069	APAF1	0.737	0.037	apoptotic peptidase activating factor 1 [Source:HGNC Symbol;Acc:HGNC:576]	chr12:99038918-100378049	NM_001160, NM_0	XM_011538252	NP_001151, NP_03736	XP_016874739	protein_coding	ENSG0000012086	317	576
4070	APH1A	-0.507	0.037	aph-1 homolog A, gamma-secretase subunit [Source:HGNC Symbol;Acc:HGNC:29509]	chr1:150237798-150253327	NM_001077628, N	XM_006711362	NP_001071096, NP_00	.	protein_coding	ENSG0000011736	51107	29509
4071	CPNE1	-0.646	0.037	copine 1 [Source:HGNC Symbol;Acc:HGNC:2314]	chr20:34213952-34252838	NM_001198863, N	.	NP_001185792, NP_00	.	protein_coding	ENSG0000021407	8904	2314
4072	PSMB8	-0.730	0.037	proteasome subunit beta 8 [Source:HGNC Symbol;Acc:HGNC:9545]	chr6:32808493-32827362	NM_004159, NM_1	.	NP_004150, NP_68372	.	protein_coding	ENSG0000020426	5696	9545
4073	ACER3	0.889	0.037	alkaline ceramidase 3 [Source:HGNC Symbol;Acc:HGNC:16066]	chr11:76571916-76737841	NM_001300953, N	XM_011545152, XM_0	NP_001287882, NP_00	XP_011543453, XP_0115	protein_coding	ENSG0000007812	55331	16066
4074	FNIP1	0.956	0.037	folliculin interacting protein 1 [Source:HGNC Symbol;Acc:HGNC:29418]	chr5:130759613-131132756	NM_001008738, N	.	NP_001008738, NP_58	.	protein_coding	ENSG0000021712	96459	29418
4075	ANGEL2	0.730	0.037	angel homolog 2 [Source:HGNC Symbol;Acc:HGNC:30534]	chr1:213165523-213189168	NM_001300753, N	XM_017002774, XM_0	NP_001287682, NP_00	XP_005273401, XP_0052	protein_coding	ENSG0000017460	90806	30534
4076	ARL2	-0.549	0.037	ADP ribosylation factor like GTPase 2 [Source:HGNC Symbol;Acc:HGNC:693]	chr11:64781584-64789656	NM_001199745, N	.	NP_001186674, NP_00	.	protein_coding	ENSG0000021346	402	693
4077	TPM4	0.479	0.037	tropomyosin 4 [Source:HGNC Symbol;Acc:HGNC:12013]	chr19:16178316-16213815	NM_001145160, N	.	NP_001138632, NP_00	XP_005260099, XP_0067	protein_coding	ENSG0000016746	7171	12013
4078	LINC00998	0.571	0.037	long intergenic non-protein coding RNA 998 [Source:HGNC Symbol;Acc:HGNC:48953]	chr7:112756772-112758668	.	.	.	.	protein_coding	ENSG0000021419	.	48953
4079	PGF	-0.745	0.037	placental growth factor [Source:HGNC Symbol;Acc:HGNC:8893]	chr14:75408536-75422487	NM_001207012, N	.	NP_001193941, NP_00	.	protein_coding	ENSG0000011963	5228	8893
4080	RBL2	0.640	0.037	RB transcriptional corepressor like 2 [Source:HGNC Symbol;Acc:HGNC:9894]	chr16:53468331-53537163	NM_001323611, N	XM_011523253, XM_0	NP_001310537, NP_00	XP_011521554, XP_0115	protein_coding	ENSG0000010347	5934	9894
4081	ASAP1	0.603	0.037	ArfGAP with SH3 domain, ankyrin repeat and PH domain 1 [Source:HGNC Symbol;Acc:H	chr8:131064352-131455906	NM_001247996, N	XM_017013468, XM_0	NP_001234925, NP_06	XP_005250982, XP_0067	protein_coding	ENSG0000015331	50807	2720
4082	TOMM40L	-0.660	0.037	translocase of outer mitochondrial membrane 40 like [Source:HGNC Symbol;Acc:HGNC:	chr1:161195728-161208092	NM_001286373, N	XM_017002481, XM_0	NP_001273302, NP_00	XP_006711635, XP_0115	protein_coding	ENSG0000015888	100847090	25756
4083	HOXB13	-0.662	0.037	homeobox B13 [Source:HGNC Symbol;Acc:HGNC:5112]	chr17:46802124-46806540	NM_006361	.	NP_006352	.	protein_coding	ENSG0000015918	10481	5112
4084	WIPF2	0.555	0.037	WAS/WASL interacting protein family member 2 [Source:HGNC Symbol;Acc:HGNC:3092	chr17:38375573-38440388	NM_133264	XM_011524413, XM_0	NP_573571	XP_005257140, XP_0052	protein_coding	ENSG0000017147	147179	30923
4085	VAMP8	-0.493	0.037	vesicle associated membrane protein 8 [Source:HGNC Symbol;Acc:HGNC:12647]	chr2:85788684-85809154	NM_003761	XM_017005170	NP_003752	XP_016860659	protein_coding	ENSG0000011864	8673	12647
4086	CBX2	-0.539	0.037	chromobox 2 [Source:HGNC Symbol;Acc:HGNC:1552]	chr17:77751930-77761782	NM_005189, NM_0	XM_011525382	NP_005180, NP_11603	XP_011523684	protein_coding	ENSG0000017389	84733	1552
4087	SLC2A6	-0.596	0.037	solute carrier family 2 member 6 [Source:HGNC Symbol;Acc:HGNC:11011]	chr9:136336216-136344259	NM_001145099, N	XM_017014238, XM_0	NP_001138571, NP_06	XP_011516491, XP_0168	protein_coding	ENSG0000016032	11182	11011
4088	CRTAC1	-3.575	0.037	cartilage acidic protein 1 [Source:HGNC Symbol;Acc:HGNC:14882]	chr10:99609995-99790585	NM_001206528, N	XM_011539917, XM_0	NP_001193457, NP_06	XP_005269995, XP_0115	protein_coding	ENSG0000009571	55118	14882
4089	PEX6	-0.580	0.037	peroxisomal biogenesis factor 6 [Source:HGNC Symbol;Acc:HGNC:8859]	chr6:42928495-42946958	NM_000287	XM_011514661, XM_0	NP_000278	XP_011512963	protein_coding	ENSG0000012458	5190	8859
4090	ZBTB10	0.923	0.037	zinc finger and BTB domain containing 10 [Source:HGNC Symbol;Acc:HGNC:30953]	chr8:81397853-81438500	NM_001105539, N	XM_005251287	NP_001099009, NP_00	.	protein_coding	ENSG0000020518	65986	30953
4091	KLF6	0.554	0.037	Kruppel like factor 6 [Source:HGNC Symbol;Acc:HGNC:2235]	chr10:3818187-3827473	NM_001160124, N	.	NP_001153596, NP_00	.	protein_coding	ENSG0000006708	1316	2235
4092	NSUN2	0.510	0.038	NOP2/Sun RNA methyltransferase family member 2 [Source:HGNC Symbol;Acc:HGNC:2	chr5:6599351-6633404	NM_001193455, N	.	NP_001180384, NP_06	.	protein_coding	ENSG0000003747	54888	25994
4093	GAA	-0.534	0.038	glucosidase alpha, acid [Source:HGNC Symbol;Acc:HGNC:4065]	chr17:78075392-78093678	NM_000152, NM_0	XM_005257194, XM_0	NP_000143, NP_00107	XP_005257250, XP_0052	protein_coding	ENSG0000017129	2548	4065
4094	SH3GLB2	-1.009	0.038	SH3 domain containing GRB2 like endophilin B2 [Source:HGNC Symbol;Acc:HGNC:10834	chr9:131683173-131790579	NM_001287045, N	XM_006717191, XM_0	NP_001273974, NP_00	XP_005252155, XP_0052	protein_coding	ENSG0000014834	56904	10834
4095	BUB3	0.590	0.038	BUB3, mitotic checkpoint protein [Source:HGNC Symbol;Acc:HGNC:1151]	chr10:124913792-124929983	NM_001007793, N	.	NP_001007794, NP_00	.	protein_coding	ENSG0000015447	9184	1151
4096	CDKN1C	-0.688	0.038	cyclin dependent kinase inhibitor 1C [Source:HGNC Symbol;Acc:HGNC:1786]	chr11:2904442-2907111	NM_000076, NM_0	XM_005252732	NP_000067, NP_00111	XP_005252789, XP_0168	protein_coding	ENSG0000012975	1028	1786
4097	RHPN1	-0.601	0.038	rhophilin Rho GTPase binding protein 1 [Source:HGNC Symbol;Acc:HGNC:19973]	chr8:144451056-144466390	NM_052924	XM_011516823, XM_0	NP_443156	XP_005250829, XP_0052	protein_coding	ENSG0000015810	114822	19973
4098	BSDC1	-0.533	0.038	BSD domain containing 1 [Source:HGNC Symbol;Acc:HGNC:25501]	chr1:32830703-32860049	NM_001143888, N	XM_011541678, XM_0	NP_001137360, NP_00	XP_005271043, XP_0115	protein_coding	ENSG0000016005	55108	25501
4099	PAQR3	0.947	0.038	progestin and adipoQ receptor family member 3 [Source:HGNC Symbol;Acc:HGNC:3013	chr4:79697531-79860592	NM_001040202	XM_006714106, XM_0	NP_001035292	XP_005262826, XP_0168	protein_coding	ENSG0000016329	152559	30130
4100	RFXANK	-0.808	0.038	regulatory factor X associated ankyrin containing protein [Source:HGNC Symbol;Acc:HG	chr19:19256375-19314233	NM_001278727, N	XM_017027415, XM_0	NP_001265656, NP_00	XP_005260191, XP_0052	protein_coding	ENSG0000006449	8625	9987
4101	C19orf48	-0.492	0.038	chromosome 19 open reading frame 48 [Source:HGNC Symbol;Acc:HGNC:29667]	chr19:51300960-51308186	NM_001290149, N	.	NP_001277078, NP_00	.	protein_coding	ENSG0000016774	84798	29667
4102	DOCK6	-0.631	0.038	dedicator of cytokinesis 6 [Source:HGNC Symbol;Acc:HGNC:19189]	chr19:11309970-11373128	NM_020812	XM_011528152, XM_0	NP_065863	XP_005260057, XP_0052	protein_coding	ENSG0000013015	57572	19189

4103	PPDPF	-0.496	0.038	pancreatic progenitor cell differentiation and proliferation factor [Source:HGNC Symbol	chr20:62152076-62153559	NM_024299	.	NP_077275	.	protein_coding	ENSG0000012553	79144	16142
4104	SUSD1	0.682	0.038	sushi domain containing 1 [Source:HGNC Symbol;Acc:HGNC:25413]	chr9:114803064-114937688	NM_001282640, NN	XM_011518919, XM_0	NP_001269569, NP_00	XP_011517217, XP_0115	protein_coding	ENSG0000010686	64420	25413
4105	HM13	-0.512	0.038	histocompatibility minor 13 [Source:HGNC Symbol;Acc:HGNC:16435]	chr20:30102230-30159409	NM_030789, NM_1	XM_011529072, XM_0	NP_110416, NP_84869	.	protein_coding	ENSG0000010129	81502	16435
4106	RPS2	-0.539	0.038	ribosomal protein S2 [Source:HGNC Symbol;Acc:HGNC:10404]	chr16:2012052-2014861	NM_002952	.	NP_002943	.	protein_coding	ENSG0000014098	6187	10404
4107	DSCC1	0.676	0.038	DNA replication and sister chromatid cohesion 1 [Source:HGNC Symbol;Acc:HGNC:2445	chr8:120846215-120868250	NM_024094	XM_005251065	NP_076999	XP_005251122	protein_coding	ENSG0000013698	79075	24453
4108	USP5	-0.488	0.038	ubiquitin specific peptidase 5 [Source:HGNC Symbol;Acc:HGNC:12628]	chr12:6961291-6975793	NM_001098536, NN	.	NP_001092006, NP_00	.	protein_coding	ENSG0000011166	8078	12628
4109	PEX11G	-0.844	0.038	peroxisomal biogenesis factor 11 gamma [Source:HGNC Symbol;Acc:HGNC:20208]	chr19:7541760-7562335	NM_001270539, NN	XM_011528431, XM_0	NP_001257468, NP_00	XP_005272563, XP_0052	protein_coding	ENSG0000010488	92960	20208
4110	VKORC1	-0.762	0.038	vitamin K epoxide reductase complex subunit 1 [Source:HGNC Symbol;Acc:HGNC:23663	chr16:31085742-31107301	NM_001311311, NN	XM_011545945, XM_0	NP_001298240, NP_07	XP_011544246, XP_0115	protein_coding	ENSG0000016739	79001	23663
4111	TTI1	0.529	0.038	TELO2 interacting protein 1 [Source:HGNC Symbol;Acc:HGNC:29029]	chr20:36611408-36661870	NM_001303457, NN	.	NP_001290386, NP_05	XP_016883637	protein_coding	ENSG0000010140	9675	29029
4112	SNX24	0.871	0.038	sorting nexin 24 [Source:HGNC Symbol;Acc:HGNC:21533]	chr5:122181143-122344907	NM_014035	XM_011543351, XM_0	NP_054754	XP_005272029, XP_0115	protein_coding	ENSG0000006465	28966	21533
4113	ECHDC2	-0.757	0.038	enoyl-CoA hydratase domain containing 2 [Source:HGNC Symbol;Acc:HGNC:23408]	chr1:53361581-53387446	NM_001198961, NN	XM_011541725, XM_0	NP_001185890, NP_00	XP_011540012, XP_0115	protein_coding	ENSG0000012131	55268	23408
4114	CCDC74A	-0.794	0.038	coiled-coil domain containing 74A [Source:HGNC Symbol;Acc:HGNC:25197]	chr2:132285405-132291239	NM_001258304, NN	XM_017005235, XM_0	NP_001245233, NP_00	XP_005263892, XP_0052	protein_coding	ENSG0000016304	90557	25197
4115	SDHAF1	-0.588	0.038	succinate dehydrogenase complex assembly factor 1 [Source:HGNC Symbol;Acc:HGNC:3	chr19:36486100-36487217	NM_001042631	.	NP_001036096	.	protein_coding	ENSG0000020513	644096	33867
4116	TPMT	0.625	0.038	thiopurine S-methyltransferase [Source:HGNC Symbol;Acc:HGNC:12014]	chr6:18128541-18155305	NM_000367	XM_011514840, XM_0	NP_000358	XP_011513141, XP_0168	protein_coding	ENSG0000013736	7172	12014
4117	TEAD2	-0.573	0.038	TEA domain transcription factor 2 [Source:HGNC Symbol;Acc:HGNC:11715]	chr19:49838652-49878373	NM_001256658, NN	XM_011527404, XM_0	NP_001243587, NP_00	XP_005259391, XP_0067	protein_coding	ENSG0000007421	8463	11715
4118	USP1	0.581	0.038	ubiquitin specific peptidase 1 [Source:HGNC Symbol;Acc:HGNC:12607]	chr1:62901974-62917475	NM_001017415, NN	.	NP_001017415, NP_00	.	protein_coding	ENSG0000016260	7398	12607
4119	GRIN1	-1.533	0.038	glutamate ionotropic receptor NMDA type subunit 1 [Source:HGNC Symbol;Acc:HGNC:4	chr9:140032841-140066861	NM_000832, NM_0	XM_011518583, XM_0	NP_000823, NP_00117	XP_005266128, XP_0052	protein_coding	ENSG0000017688	2902	4584
4120	HARBI1	0.786	0.038	harbinger transposase derived 1 [Source:HGNC Symbol;Acc:HGNC:26522]	chr11:46624410-46638777	NM_173811	.	NP_776172	XP_016873093, XP_0168	protein_coding	ENSG0000018042	283254	26522
4121	RPL36	-0.594	0.038	ribosomal protein L36 [Source:HGNC Symbol;Acc:HGNC:13631]	chr19:5674957-5720583	NM_015414, NM_0	.	NP_056229, NP_37866	.	protein_coding	ENSG0000013025	25873	13631
4122	FAAP20	-0.763	0.039	Fanconi anemia core complex associated protein 20 [Source:HGNC Symbol;Acc:HGNC:2	chr1:2004900-2126214	NM_001256945, NN	XM_011540922, XM_0	NP_001243874, NP_00	XP_006710484, XP_0115	protein_coding	ENSG0000016258	199990	26428
4123	GRAMD3	0.817	0.039	GRAM domain containing 3 [Source:HGNC Symbol;Acc:HGNC:24911]	chr5:125695823-125830063	NM_001146319, NN	XM_011543595, XM_0	NP_001139791, NP_00	XP_005272114, XP_0052	protein_coding	ENSG0000015532	65983	24911
4124	AKT3	0.871	0.039	AKT serine/threonine kinase 3 [Source:HGNC Symbol;Acc:HGNC:393]	chr1:243419357-244013430	NM_001206729, NN	XM_011544011, XM_0	NP_001193658, NP_00	XP_005273051, XP_0052	protein_coding	ENSG0000011702	10000	393
4125	FBXL19	-0.693	0.039	F-box and leucine rich repeat protein 19 [Source:HGNC Symbol;Acc:HGNC:25300]	chr16:30934391-30960104	NM_001099784, NN	.	NP_001093254, NP_00	.	protein_coding	ENSG0000009936	54620	25300
4126	LRRC4B	-0.947	0.039	leucine rich repeat containing 4B [Source:HGNC Symbol;Acc:HGNC:25042]	chr19:51020148-51071302	NM_001080457	XM_011527520, XM_0	NP_001073926	XP_005259486, XP_0067	protein_coding	ENSG0000013140	94030	25042
4127	SLC2A12	1.009	0.039	solute carrier family 2 member 12 [Source:HGNC Symbol;Acc:HGNC:18067]	chr6:134273307-134373774	NM_145176	.	NP_660159	.	protein_coding	ENSG0000014641	154091	18067
4128	ZBTB12	-1.037	0.039	zinc finger and BTB domain containing 12 [Source:HGNC Symbol;Acc:HGNC:19066]	chr6:31865561-31919861	NM_181842	.	NP_862825	XP_011512685	protein_coding	ENSG0000020436	221527	19066
4129	CCNL2	0.581	0.039	cyclin L2 [Source:HGNC Symbol;Acc:HGNC:20570]	chr1:1321874-1334697	NM_001039577, NN	XM_011542221, XM_0	NP_001034666, NP_11	XP_011540518, XP_0115	protein_coding	ENSG0000022197	81669	20570
4130	WBP1L	0.539	0.039	WW domain binding protein 1-like [Source:HGNC Symbol;Acc:HGNC:23510]	chr10:104503726-104576024	NM_001083913, NN	XM_011539913	NP_001077382, NP_06	XP_011538215	protein_coding	ENSG0000016627	54838	23510
4131	FAM83F	-0.731	0.039	family with sequence similarity 83 member F [Source:HGNC Symbol;Acc:HGNC:25148]	chr22:40390952-40439533	NM_138435	.	NP_612444	.	protein_coding	ENSG0000013347	113828	25148
4132	CES3	-0.903	0.039	carboxylesterase 3 [Source:HGNC Symbol;Acc:HGNC:1865]	chr16:66995139-67009051	NM_001185176, NN	XM_011522975	NP_001172105, NP_00	XP_011521277	protein_coding	ENSG0000017282	23491	1865
4133	CMTM7	-0.572	0.039	CKLF like MARVEL transmembrane domain containing 7 [Source:HGNC Symbol;Acc:HGN	chr3:32433162-32497020	NM_138410, NM_1	.	NP_612419, NP_85213	XP_011531621	protein_coding	ENSG0000015355	112616	19178
4134	CORO1C	0.477	0.039	coronin 1C [Source:HGNC Symbol;Acc:HGNC:2254]	chr12:109038884-109125372	NM_001105237, NN	.	NP_001098707, NP_00	XP_016874610, XP_0168	protein_coding	ENSG0000011088	23603	2254
4135	NECAP1	0.588	0.039	NECAP endocytosis associated 1 [Source:HGNC Symbol;Acc:HGNC:24539]	chr12:8234806-8250367	NM_015509	.	NP_056324	.	protein_coding	ENSG0000008981	25977	24539
4136	SPRYD3	-0.685	0.039	SPRY domain containing 3 [Source:HGNC Symbol;Acc:HGNC:25920]	chr12:53440752-53473204	NM_032840	.	NP_116229	.	protein_coding	ENSG0000016777	84926	25920
4137	PEX16	-0.571	0.039	peroxisomal biogenesis factor 16 [Source:HGNC Symbol;Acc:HGNC:8857]	chr11:45931219-45940363	NM_004813, NM_0	XM_011520474	NP_004804, NP_47651	XP_011518776	protein_coding	ENSG0000012168	9409	8857
4138	ATE1	0.718	0.039	arginyltransferase 1 [Source:HGNC Symbol;Acc:HGNC:782]	chr10:123499935-123688316	NM_001001976, NN	XM_005269459, XM_0	NP_001001976, NP_00	XP_005269515, XP_0052	protein_coding	ENSG0000010766	11101	782
4139	PDIA2	-1.161	0.039	protein disulfide isomerase family A member 2 [Source:HGNC Symbol;Acc:HGNC:14180]	chr16:333151-337215	NM_006849	.	NP_006840	.	protein_coding	ENSG0000018561	64714	14180
4140	RP11-382A20	0.621	0.039	Hepatoma-derived growth factor-related protein 3 [Source:UniProtKB/Swiss-Prot;Acc:C	chr15:83776158-83876770	NM_016073	XM_006720554	NP_057157	XP_006720617	protein_coding	ENSG0000016650	50810	.
4141	TAF15	0.492	0.039	TATA-box binding protein associated factor 15 [Source:HGNC Symbol;Acc:HGNC:11547]	chr17:34136458-34174246	NM_003487, NM_1	XM_011525315, XM_0	NP_003478, NP_63196	XP_011523617	protein_coding	ENSG0000027064	8148	11547
4142	ATRNL1	0.801	0.039	attractin like 1 [Source:HGNC Symbol;Acc:HGNC:29063]	chr10:116853123-117708503	NM_001276282, NN	XM_011539591, XM_0	NP_001263211, NP_99	XP_011537889, XP_0115	protein_coding	ENSG0000010751	26033	29063
4143	BAK1	-0.612	0.039	BCL2 antagonist/killer 1 [Source:HGNC Symbol;Acc:HGNC:949]	chr6:33540323-33548070	NM_001188	XM_011514780, XM_0	NP_001179	XP_011513081, XP_0115	protein_coding	ENSG0000003011	578	949
4144	IMMP1L	-0.758	0.039	inner mitochondrial membrane peptidase subunit 1 [Source:HGNC Symbol;Acc:HGNC:2	chr11:31453947-31531192	NM_001304274, NN	XM_011519948, XM_0	NP_001291203, NP_65	XP_005252869, XP_0115	protein_coding	ENSG0000014895	196294	26317
4145	CCNH	0.607	0.039	cyclin H [Source:HGNC Symbol;Acc:HGNC:1594]	chr5:86689233-86708836	NM_001199189, NN	XM_005248629, XM_0	NP_001186118, NP_00	XP_005248684, XP_0052	protein_coding	ENSG0000013448	902	1594
4146	PLAGL1	0.793	0.039	PLAG1 like zinc finger 1 [Source:HGNC Symbol;Acc:HGNC:9046]	chr6:144261436-144385736	NM_001080951, NN	.	NP_001074420, NP_00	.	protein_coding	ENSG0000011849	5325	9046
4147	KDM6B	-0.610	0.039	lysine demethylase 6B [Source:HGNC Symbol;Acc:HGNC:29012]	chr17:7743221-7758114	NM_001080424	XM_011523753, XM_0	NP_001073893	XP_005256606, XP_0052	protein_coding	ENSG0000013251	23135	29012
4148	PDLIM5	0.791	0.039	PDZ and LIM domain 5 [Source:HGNC Symbol;Acc:HGNC:17468]	chr4:95373007-95589378	NM_001011513, NN	XM_017007658, XM_0	NP_001011513, NP_00	XP_005262750, XP_0052	protein_coding	ENSG0000016311	10611	17468
4149	CHD8	0.553	0.040	chromodomain helicase DNA binding protein 8 [Source:HGNC Symbol;Acc:HGNC:20153]	chr14:21853352-21924239	NM_001170629, NN	.	NP_001164100, NP_06	.	protein_coding	ENSG0000010088	57680	20153
4150	SLX4IP	0.778	0.040	SLX4 interacting protein [Source:HGNC Symbol;Acc:HGNC:16225]	chr20:10415950-10608682	NM_001009608	XM_017027651, XM_0	NP_001009608	XP_011527462, XP_0168	protein_coding	ENSG0000014934	128710	16225
4151	ADGRL1	-0.551	0.040	adhesion G protein-coupled receptor L1 [Source:HGNC Symbol;Acc:HGNC:20973]	chr19:14258554-14316999	NM_001008701, NN	XM_017026479, XM_0	NP_001008701, NP_05	XP_005259875, XP_0115	protein_coding	ENSG0000007207	22859	20973
4152	DGKQ	-0.614	0.040	diacylglycerol kinase theta [Source:HGNC Symbol;Acc:HGNC:2856]	chr4:952674-967344	NM_001347	XM_011513411	NP_001338	XP_011511713	protein_coding	ENSG0000014521	1609	2856
4153	FOXG1	0.893	0.040	forkhead box G1 [Source:HGNC Symbol;Acc:HGNC:3811]	chr14:29234593-29239483	NM_005249	.	NP_005240	.	protein_coding	ENSG0000017616	2290	3811
4154	MGMT	-0.682	0.040	O-6-methylguanine-DNA methyltransferase [Source:HGNC Symbol;Acc:HGNC:7059]	chr10:131265447-131566271	NM_002412	.	NP_002403	.	protein_coding	ENSG0000017043	4255	7059
4155	SECTM1	-0.674	0.040	secreted and transmembrane 1 [Source:HGNC Symbol;Acc:HGNC:10707]	chr17:80278899-80291874	NM_003004	XM_011523588, XM_0	NP_002995	XP_005256449, XP_0115	protein_coding	ENSG0000014157	6398	10707
4156	STX3	0.518	0.040	syntaxin 3 [Source:HGNC Symbol;Acc:HGNC:11438]	chr11:59522531-59573355	NM_001178040, NN	XM_011545221, XM_0	NP_001171511, NP_00	XP_005274252, XP_0052	protein_coding	ENSG0000016690	6809	11438

4157	IL1RAPL1	1.050	0.040	interleukin 1 receptor accessory protein like 1 [Source:HGNC Symbol;Acc:HGNC:5996]	chrX:28605515-29974840	NM_014271	XM_011545445, XM_0	NP_055086	XP_005274498, XP_0115	protein_coding	ENSG0000016930	11141	5996
4158	POMK	0.897	0.040	protein-O-mannose kinase [Source:HGNC Symbol;Acc:HGNC:26267]	chr8:42948648-42978577	NM_001277971, NN	XM_011544669, XM_0	NP_001264900, NP_11	XP_011542970, XP_0115	protein_coding	ENSG0000018590	84197	26267
4159	BLOC1S5	0.687	0.040	biogenesis of lysosomal organelles complex 1 subunit 5 [Source:HGNC Symbol;Acc:HGN	chr6:8013799-8064647	NM_001199322, NN	.	NP_001186251, NP_00	.	protein_coding	ENSG0000018842	63915	18561
4160	DCTPP1	-0.541	0.040	dCTP pyrophosphatase 1 [Source:HGNC Symbol;Acc:HGNC:28777]	chr16:30418617-30442429	NM_024096	.	NP_077001	.	protein_coding	ENSG0000017995	79077	28777
4161	GNAI3	0.550	0.040	G protein subunit alpha i3 [Source:HGNC Symbol;Acc:HGNC:4387]	chr1:110091232-110160943	NM_006496	.	NP_006487	.	protein_coding	ENSG0000006513	406974	4387
4162	MPHOSPH9	1.530	0.040	M-phase phosphoprotein 9 [Source:HGNC Symbol;Acc:HGNC:7215]	chr12:123638761-123742506	NM_022782	XM_011537748, XM_0	NP_073619	XP_011536038, XP_0115	protein_coding	ENSG0000005182	10198	7215
4163	RAPGEF3	-1.064	0.040	Rap guanine nucleotide exchange factor 3 [Source:HGNC Symbol;Acc:HGNC:16629]	chr12:48128149-48214395	NM_001098531, NN	XM_011537758, XM_0	NP_001092001, NP_00	XP_011536054, XP_0115	protein_coding	ENSG0000007933	10411	16629
4164	TIPIN	0.674	0.040	TIMELESS interacting protein [Source:HGNC Symbol;Acc:HGNC:30750]	chr15:66628543-66649054	NM_001289986, NN	XM_017022390, XM_0	NP_001276915, NP_06	XP_005254578, XP_0052	protein_coding	ENSG0000007513	54962	30750
4165	ATP1A1	-0.488	0.040	ATPase Na+/K+ transporting subunit alpha 1 [Source:HGNC Symbol;Acc:HGNC:799]	chr1:116915881-116947396	NM_000701, NM_0	XM_006710655	NP_000692, NP_00115	XP_006710718, XP_0168	protein_coding	ENSG0000016339	476	799
4166	PPARG	-0.711	0.040	peroxisome proliferator activated receptor gamma [Source:HGNC Symbol;Acc:HGNC:92	chr3:12328866-12475855	NM_005037, NM_0	XM_011533844, XM_0	NP_005028, NP_05695	XP_011532142, XP_0115	protein_coding	ENSG0000013217	5468	9236
4167	TRAK1	-0.534	0.040	trafficking kinesin protein 1 [Source:HGNC Symbol;Acc:HGNC:29947]	chr3:42055293-42267381	NM_001042646, NN	XM_011533489, XM_0	NP_001036111, NP_00	XP_005265013, XP_0052	protein_coding	ENSG0000018260	22906	29947
4168	VAT1	-0.474	0.040	vesicle amine transport 1 [Source:HGNC Symbol;Acc:HGNC:16919]	chr17:41166621-41174435	NM_006373	.	NP_006364	.	protein_coding	ENSG0000010882	10493	16919
4169	NFIA	0.941	0.040	nuclear factor I A [Source:HGNC Symbol;Acc:HGNC:7784]	chr1:61330930-61928465	NM_001134673, NN	XM_011541515, XM_0	NP_001128145, NP_00	XP_011539814, XP_0115	protein_coding	ENSG0000016259	4774	7784
4170	PTGES2	-0.535	0.040	prostaglandin E synthase 2 [Source:HGNC Symbol;Acc:HGNC:17822]	chr9:130882971-130890741	NM_001256335, NN	XM_011519051, XM_0	NP_001243264, NP_07	XP_005252296, XP_0115	protein_coding	ENSG0000014833	80142	17822
4171	NAA38	-1.032	0.040	N(alpha)-acetyltransferase 38, NatC auxiliary subunit [Source:HGNC Symbol;Acc:HGNC:2	chr17:7760002-7816078	NM_032356	XM_011524037, XM_0	NP_001307853, NP_00	XP_005256879, XP_0052	protein_coding	ENSG0000018301	84316	28212
4172	NAP1L1	0.519	0.040	nucleosome assembly protein 1 like 1 [Source:HGNC Symbol;Acc:HGNC:7637]	chr12:76430366-76478813	NM_001307924, NN	XM_011538395, XM_0	NP_001294853, NP_00	XP_006719488, XP_0115	protein_coding	ENSG0000018710	4673	7637
4173	USP54	0.670	0.040	ubiquitin specific peptidase 54 [Source:HGNC Symbol;Acc:HGNC:23513]	chr10:75257295-75385408	NM_152586	XM_011539368, XM_0	NP_689799	XP_005269639, XP_0115	protein_coding	ENSG0000016634	159195	23513
4174	FARSB	0.558	0.040	phenylalanyl-tRNA synthetase beta subunit [Source:HGNC Symbol;Acc:HGNC:17800]	chr2:223435254-223521056	NM_005687	XM_011510466, XM_0	NP_005678	XP_006712232, XP_0115	protein_coding	ENSG0000011612	10056	17800
4175	GAS8	-0.606	0.040	growth arrest specific 8 [Source:HGNC Symbol;Acc:HGNC:4166]	chr16:90089007-90111379	NM_001286205, NN	XM_017023125, XM_0	NP_001273134, NP_00	XP_006721238, XP_0115	protein_coding	ENSG0000014101	2622	4166
4176	ITPKC	-0.613	0.040	inositol-trisphosphate 3-kinase C [Source:HGNC Symbol;Acc:HGNC:14897]	chr19:41223007-41256397	NM_025194	XM_006723404	NP_079470	XP_006723467	protein_coding	ENSG0000008654	80271	14897
4177	ZNF701	0.878	0.040	zinc finger protein 701 [Source:HGNC Symbol;Acc:HGNC:25597]	chr19:53073527-53090427	NM_001172655, NN	XM_011527092	NP_001166126, NP_06	XP_011525394	protein_coding	ENSG0000016756	55762	25597
4178	ADM	-0.715	0.040	adrenomedullin [Source:HGNC Symbol;Acc:HGNC:259]	chr11:10326226-10328944	NM_001124	.	NP_001115	.	protein_coding	ENSG0000014892	133	259
4179	MSL2	0.660	0.040	male-specific lethal 2 homolog (Drosophila) [Source:HGNC Symbol;Acc:HGNC:25544]	chr3:135867763-135914688	NM_001145417, NN	XM_011512949, XM_0	NP_001138889, NP_06	XP_005247628, XP_0052	protein_coding	ENSG0000017457	55167	25544
4180	PELI1	0.702	0.040	pellino E3 ubiquitin protein ligase 1 [Source:HGNC Symbol;Acc:HGNC:8827]	chr2:64319785-64371588	NM_020651	XM_011532996, XM_0	NP_065702	XP_005264494, XP_0115	protein_coding	ENSG0000019732	57162	8827
4181	UTP11	0.550	0.040	UTP11, small subunit processome component homolog (S. cerevisiae) [Source:HGNC Sy	chr1:38478413-38490496	NM_016037	XM_011541555	NP_057121	XP_011539857	protein_coding	ENSG0000018352	51118	24329
4182	CHMP1A	-0.506	0.040	charged multivesicular body protein 1A [Source:HGNC Symbol;Acc:HGNC:8740]	chr16:89710838-89737675	NM_001083314, NN	.	NP_001076783, NP_00	.	protein_coding	ENSG0000013116	5119	8740
4183	TTC17	0.593	0.040	tetratricopeptide repeat domain 17 [Source:HGNC Symbol;Acc:HGNC:25596]	chr11:43380481-43516483	NM_001307943, NN	XM_006718263, XM_0	NP_001294872, NP_06	XP_005253073, XP_0052	protein_coding	ENSG0000005284	55761	25596
4184	TLR2	0.907	0.041	toll like receptor 2 [Source:HGNC Symbol;Acc:HGNC:11848]	chr4:154622651-154626851	NM_003264	XM_011532216, XM_0	NP_001305716, NP_00	XP_005263250, XP_0052	protein_coding	ENSG0000013746	7097	11848
4185	CEP128	0.936	0.041	centrosomal protein 128 [Source:HGNC Symbol;Acc:HGNC:20359]	chr14:80962816-81408129	NM_152446	XM_011536496, XM_0	NP_689659	XP_006720119, XP_0115	protein_coding	ENSG0000010062	145508	20359
4186	MYH10	0.568	0.041	myosin heavy chain 10 [Source:HGNC Symbol;Acc:HGNC:7568]	chr17:8377522-8534079	NM_001256012, NN	XM_017024682, XM_0	NP_001242941, NP_00	XP_005256708, XP_0115	protein_coding	ENSG0000013302	4628	7568
4187	RPS17	-0.486	0.041	ribosomal protein S17 [Source:HGNC Symbol;Acc:HGNC:10397]	chr15:83205503-83316728	NM_001021	.	NP_001012	.	protein_coding	ENSG0000018277	6218	10397
4188	ABTB2	-0.554	0.041	ankyrin repeat and BTB domain containing 2 [Source:HGNC Symbol;Acc:HGNC:23842]	chr11:34172534-34379555	NM_145804	.	NP_665803	.	protein_coding	ENSG0000016601	25841	23842
4189	CENPL	0.689	0.041	centromere protein L [Source:HGNC Symbol;Acc:HGNC:17879]	chr1:173768687-173793460	NM_001127181, NN	.	NP_001120653, NP_00	.	protein_coding	ENSG0000012033	91687	17879
4190	MBOAT7	-0.505	0.041	membrane bound O-acyltransferase domain containing 7 [Source:HGNC Symbol;Acc:HG	chr19:54677106-54693733	NM_001146056, NN	XM_011527300, XM_0	NP_001139528, NP_00	XP_011525601, XP_0115	protein_coding	ENSG0000012550	79143	15505
4191	BNIP3L	0.703	0.041	BCL2 interacting protein 3 like [Source:HGNC Symbol;Acc:HGNC:1085]	chr8:26240413-26270645	NM_004331	XM_011544630, XM_0	NP_004322	XP_005273674, XP_0115	protein_coding	ENSG0000010476	665	1085
4192	CAV1	0.840	0.041	caveolin 1 [Source:HGNC Symbol;Acc:HGNC:1527]	chr7:116164838-116201238	NM_001172895, NN	.	NP_001166366, NP_00	.	protein_coding	ENSG0000010597	857	1527
4193	CHPF	-0.502	0.041	chondroitin polymerizing factor [Source:HGNC Symbol;Acc:HGNC:24291]	chr2:220403668-220415317	NM_001195731, NN	.	NP_001182660, NP_07	.	protein_coding	ENSG0000012398	79586	24291
4194	SKA2	0.777	0.041	spindle and kinetochore associated complex subunit 2 [Source:HGNC Symbol;Acc:HGNC	chr17:57187311-57232630	NM_001100595, NN	XM_011524745	NP_001094065, NP_87	XP_011523047	protein_coding	ENSG0000018262	348235	28006
4195	CCNE1	-0.668	0.041	cyclin E1 [Source:HGNC Symbol;Acc:HGNC:1589]	chr19:30302804-30315215	NM_001238	XM_011527440, XM_0	NP_001229, NP_00130	XP_006723520, XP_0115	protein_coding	ENSG0000010517	898	1589
4196	METAP1	0.555	0.041	methionyl aminopeptidase 1 [Source:HGNC Symbol;Acc:HGNC:15789]	chr4:99916770-99983964	NM_015143	XM_017007917, XM_0	NP_055958	XP_011530079, XP_0115	protein_coding	ENSG0000016402	23173	15789
4197	NEMF	1.648	0.041	nuclear export mediator factor [Source:HGNC Symbol;Acc:HGNC:10663]	chr14:50234325-50319921	NM_001301732, NN	XM_005268201, XM_0	NP_001288661, NP_00	.	protein_coding	ENSG0000016552	9147	10663
4198	NPEPL1	-0.570	0.041	aminopeptidase-like 1 [Source:HGNC Symbol;Acc:HGNC:16244]	chr20:57264186-57290900	NM_001204872, NN	.	NP_001191801, NP_00	.	protein_coding	ENSG0000021544	79716	16244
4199	CCNJ	0.765	0.041	cyclin J [Source:HGNC Symbol;Acc:HGNC:23434]	chr10:97803150-97820627	NM_001134375, NN	XM_017016356, XM_0	NP_001127847, NP_00	XP_011538187, XP_0168	protein_coding	ENSG0000010744	54619	23434
4200	NAPB	0.687	0.041	NSF attachment protein beta [Source:HGNC Symbol;Acc:HGNC:15751]	chr20:23355155-23402156	NM_001283018, NN	XM_011529314, XM_0	NP_001269947, NP_00	XP_011527615, XP_0115	protein_coding	ENSG0000012581	63908	15751
4201	TRAPPC13	1.164	0.041	trafficking protein particle complex 13 [Source:HGNC Symbol;Acc:HGNC:25828]	chr5:64920542-65017957	NM_001093755, NN	XM_011543652	NP_001087224, NP_00	XP_011541954	protein_coding	ENSG0000011359	80006	25828
4202	UBAC1	-0.512	0.041	UBA domain containing 1 [Source:HGNC Symbol;Acc:HGNC:30221]	chr9:138824814-138853226	NM_016172	.	NP_057256	.	protein_coding	ENSG0000013056	10422	30221
4203	COPE	-0.508	0.041	coatomer protein complex subunit epsilon [Source:HGNC Symbol;Acc:HGNC:2234]	chr19:19010322-19030206	NM_007263, NM_1	XM_011527656, XM_0	NP_009194, NP_95547	XP_011525957, XP_0115	protein_coding	ENSG0000010566	11316	2234
4204	GMPPB	-0.950	0.041	GDP-mannose pyrophosphorylase B [Source:HGNC Symbol;Acc:HGNC:22932]	chr3:49726931-49761384	NM_013334, NM_0	.	NP_037466, NP_06880	.	protein_coding	ENSG0000017354	29925	22932
4205	PEPD	-0.539	0.041	peptidase D [Source:HGNC Symbol;Acc:HGNC:8840]	chr19:33877855-34012700	NM_000285, NM_0	.	NP_000276, NP_00115	.	protein_coding	ENSG0000012429	5184	8840
4206	RNF20	0.565	0.041	ring finger protein 20 [Source:HGNC Symbol;Acc:HGNC:10062]	chr9:104296132-104325626	NM_019592	XM_011518862	NP_062538	XP_011517164	protein_coding	ENSG0000015582	56254	10062
4207	BBS4	0.677	0.041	Bardet-Biedl syndrome 4 [Source:HGNC Symbol;Acc:HGNC:969]	chr15:72978519-73030817	NM_001252678, NN	XM_017022451, XM_0	NP_001239607, NP_00	XP_011520150, XP_0115	protein_coding	ENSG0000014046	585	969
4208	BRD3	-0.715	0.041	bromodomain containing 3 [Source:HGNC Symbol;Acc:HGNC:1104]	chr9:136890560-136933657	NM_007371	XM_017015165, XM_0	NP_031397	XP_006717354, XP_0115	protein_coding	ENSG0000016992	8019	1104
4209	RNF168	0.668	0.041	ring finger protein 168 [Source:HGNC Symbol;Acc:HGNC:26661]	chr3:196195653-196230639	NM_152617	.	NP_689830	.	protein_coding	ENSG0000016396	165918	26661
4210	CARNMT1	0.731	0.041	carnosine N-methyltransferase 1 [Source:HGNC Symbol;Acc:HGNC:23435]	chr9:77595935-77643339	NM_001320497, NN	XM_017014269, XM_0	NP_001307426, NP_68	XP_005251758, XP_0168	protein_coding	ENSG0000015601	138199	23435

4211	COX6B1	-0.469	0.041	cytochrome c oxidase subunit 6B1 [Source:HGNC Symbol;Acc:HGNC:2280]	chr19:36139124-36149763	NM_001863	.	NP_001854	.	protein_coding	ENSG0000012626	1340	2280
4212	CYP27A1	-0.538	0.041	cytochrome P450 family 27 subfamily A member 1 [Source:HGNC Symbol;Acc:HGNC:26	chr2:219646478-219680016	NM_000784	.	NP_000775	.	protein_coding	ENSG0000013592	1593	2605
4213	PRCP	0.505	0.041	prolylcarboxypeptidase [Source:HGNC Symbol;Acc:HGNC:9344]	chr11:82534543-82611639	NM_005040, NM_1	.	NP_005031, NP_95545	.	protein_coding	ENSG0000013750	5547	9344
4214	ZSCAN29	0.645	0.041	zinc finger and SCAN domain containing 29 [Source:HGNC Symbol;Acc:HGNC:26673]	chr15:43650369-43662260	NM_152455	XM_011521266, XM_0	NP_689668	XP_006720464, XP_0115	protein_coding	ENSG0000014026	146050	26673
4215	DMWD	-0.594	0.041	dystrophia myotonica, WD repeat containing [Source:HGNC Symbol;Acc:HGNC:2936]	chr19:46286204-46296060	NM_004943	.	NP_004934	.	protein_coding	ENSG0000018580	1762	2936
4216	MTERF1	0.724	0.041	mitochondrial transcription termination factor 1 [Source:HGNC Symbol;Acc:HGNC:2146	chr7:91500242-91510034	NM_001301134, NM	XM_006716126, XM_0	NP_001288063, NP_00	XP_005250650, XP_0067	protein_coding	ENSG0000012798	7978	21463
4217	METTL10	0.699	0.041	methyltransferase like 10 [Source:HGNC Symbol;Acc:HGNC:33787]	chr10:126448522-126480439	NM_001304467, NM	.	NP_001291396, NP_99	.	protein_coding	ENSG0000020379	399818	33787
4218	ITGA3	-0.467	0.041	integrin subunit alpha 3 [Source:HGNC Symbol;Acc:HGNC:6139]	chr17:48133473-48167844	NM_002204, NM_0	XM_005257308	NP_002195, NP_00549	XP_005257365	protein_coding	ENSG0000000588	3675	6139
4219	ZNF613	0.842	0.041	zinc finger protein 613 [Source:HGNC Symbol;Acc:HGNC:25827]	chr19:52430472-52449876	NM_001031721, NM	XM_011527333, XM_0	NP_001026891, NP_07	XP_005259326, XP_0115	protein_coding	ENSG0000017602	79898	25827
4220	MCM5	-0.505	0.041	minichromosome maintenance complex component 5 [Source:HGNC Symbol;Acc:HGNC	chr22:35796055-35821423	NM_006739	XM_006724242	NP_006730	XP_006724305	protein_coding	ENSG0000010029	4174	6948
4221	C10orf95	-0.987	0.042	chromosome 10 open reading frame 95 [Source:HGNC Symbol;Acc:HGNC:25880]	chr10:104209593-104211300	NM_024886	.	NP_079162	.	protein_coding	ENSG0000012005	79946	25880
4222	PAK2	0.512	0.042	p21 (RAC1) activated kinase 2 [Source:HGNC Symbol;Acc:HGNC:8591]	chr3:196466727-196559518	NM_002577	XM_017006501, XM_0	NP_002568	XP_011511172, XP_0168	protein_coding	ENSG0000018037	5062	8591
4223	CAST	0.577	0.042	calpastatin [Source:HGNC Symbol;Acc:HGNC:1515]	chr5:95997776-96143803	NM_001042440, NM	XM_017009912, XM_0	NP_001035905, NP_00	XP_006714759, XP_0067	protein_coding	ENSG0000015311	831	1515
4224	COLGALT2	0.697	0.042	collagen beta(1-O)galactosyltransferase 2 [Source:HGNC Symbol;Acc:HGNC:16790]	chr1:183904970-184006863	NM_001303420, NM	XM_011509338, XM_0	NP_001290349, NP_00	XP_011507634, XP_0115	protein_coding	ENSG0000019875	23127	16790
4225	NANOS3	-0.998	0.042	nanos C2HC-type zinc finger 3 [Source:HGNC Symbol;Acc:HGNC:22048]	chr19:13988060-13991564	NM_001098622	XM_011527964	NP_001092092	XP_011526266	protein_coding	ENSG0000018755	342977	22048
4226	RPS15	-0.493	0.042	ribosomal protein S15 [Source:HGNC Symbol;Acc:HGNC:10388]	chr19:1438356-1440493	NM_001018, NM_0	XM_011528176	NP_001009, NP_00129	XP_011526478	protein_coding	ENSG0000011526	6209	10388
4227	BLOC1S4	-0.613	0.042	biogenesis of lysosomal organelles complex 1 subunit 4 [Source:HGNC Symbol;Acc:HGN	chr4:6717781-6719398	NM_018366	.	NP_060836	.	protein_coding	ENSG0000018622	55330	24206
4228	LDLRAD1	-1.487	0.042	low density lipoprotein receptor class A domain containing 1 [Source:HGNC Symbol;Acc	chr1:54472971-54483859	NM_001010978, NM	XM_011541446	NP_001010978, NP_00	XP_011539748	protein_coding	ENSG0000020398	388633	32069
4229	CPT1A	-0.488	0.042	carnitine palmitoyltransferase 1A [Source:HGNC Symbol;Acc:HGNC:2328]	chr11:68522087-68609399	NM_001031847, NM	XM_005273763, XM_0	NP_001027017, NP_00	XP_005273819, XP_0052	protein_coding	ENSG0000011009	1374	2328
4230	ID1	-0.719	0.042	inhibitor of DNA binding 1, HLH protein [Source:HGNC Symbol;Acc:HGNC:5360]	chr20:30193085-30194318	NM_002165, NM_1	.	NP_002156, NP_85199	.	protein_coding	ENSG0000012596	3397	5360
4231	KCTD20	0.568	0.042	potassium channel tetramerization domain containing 20 [Source:HGNC Symbol;Acc:HG	chr6:36358327-36458920	NM_001286579, NM	XM_011514398, XM_0	NP_001273508, NP_00	XP_005248997, XP_0067	protein_coding	ENSG0000011207	222658	21052
4232	GGN	-1.124	0.042	gametogenetin [Source:HGNC Symbol;Acc:HGNC:18869]	chr19:38874904-38878666	NM_152657	XM_011526603, XM_0	NP_689870	XP_005258676, XP_0115	protein_coding	ENSG0000017916	199720	18869
4233	MTMR6	0.632	0.042	myotubularin related protein 6 [Source:HGNC Symbol;Acc:HGNC:7453]	chr13:25820338-25862147	NM_004685	XM_011535307, XM_0	NP_004676	XP_011533608, XP_0115	protein_coding	ENSG0000013950	9107	7453
4234	SEC24A	1.087	0.042	SEC24 homolog A, COPII coat complex component [Source:HGNC Symbol;Acc:HGNC:107	chr5:133936833-134063513	NM_001252231, NM	XM_017008961, XM_0	NP_001239160, NP_06	XP_006714586, XP_0168	protein_coding	ENSG0000011361	10802	10703
4235	CREBBP	0.605	0.042	CREB binding protein [Source:HGNC Symbol;Acc:HGNC:2348]	chr16:3775054-3930727	NM_001079846, NM	XM_017022944, XM_0	NP_001073315, NP_00	XP_005255181, XP_0052	protein_coding	ENSG0000000533	1387	2348
4236	FBXO31	-0.570	0.042	F-box protein 31 [Source:HGNC Symbol;Acc:HGNC:16510]	chr16:87336406-87417363	NM_024735	.	NP_079011	.	protein_coding	ENSG0000010326	79791	16510
4237	MAP4K2	-0.614	0.042	mitogen-activated protein kinase kinase kinase kinase 2 [Source:HGNC Symbol;Acc:HGN	chr11:64552385-64570713	NM_001307990, NM	XM_011545204, XM_0	NP_001294919, NP_00	XP_011543504, XP_0115	protein_coding	ENSG0000016806	5871	6864
4238	FASN	-0.475	0.042	fatty acid synthase [Source:HGNC Symbol;Acc:HGNC:3594]	chr17:80036213-80056208	NM_004104	XM_011523538	NP_004095	XP_011521840	protein_coding	ENSG0000016971	2194	3594
4239	LGALS3BP	-0.484	0.042	galectin 3 binding protein [Source:HGNC Symbol;Acc:HGNC:6564]	chr17:76967319-76976191	NM_005567	.	NP_005558	.	protein_coding	ENSG0000010867	3959	6564
4240	RRP12	-0.534	0.042	ribosomal RNA processing 12 homolog [Source:HGNC Symbol;Acc:HGNC:29100]	chr10:99116457-99185831	NM_001145114, NM	XM_011539555	NP_001138586, NP_00	XP_011537857	protein_coding	ENSG0000005274	23223	29100
4241	TMTC3	0.807	0.042	transmembrane and tetratricopeptide repeat containing 3 [Source:HGNC Symbol;Acc:H	chr12:88536072-88593664	NM_181783	XM_011537980	NP_861448	XP_011536282	protein_coding	ENSG0000013932	160418	26899
4242	CNIH3	-0.937	0.042	cornichon family AMPA receptor auxiliary protein 3 [Source:HGNC Symbol;Acc:HGNC:26	chr1:224803994-224928251	NM_152495	.	NP_001309234, NP_68	.	protein_coding	ENSG0000014378	149111	26802
4243	PARM1	0.785	0.042	prostate androgen-regulated mucin-like protein 1 [Source:HGNC Symbol;Acc:HGNC:245	chr4:75858304-75975325	NM_015393	XM_011531833	NP_056208	XP_011530135	protein_coding	ENSG0000016911	25849	24536
4244	REXO4	-0.520	0.042	REX4 homolog, 3'-5' exonuclease [Source:HGNC Symbol;Acc:HGNC:12820]	chr9:136243237-136283216	NM_001279349, NM	.	NP_001266278, NP_00	.	protein_coding	ENSG0000014830	57109	12820
4245	CCL5	-1.178	0.042	C-C motif chemokine ligand 5 [Source:HGNC Symbol;Acc:HGNC:10632]	chr17:34198494-34207797	NM_001278736, NM	.	NP_001265665, NP_00	.	protein_coding	ENSG0000027150	6352	10632
4246	ATAD3A	-0.506	0.042	ATPase family, AAA domain containing 3A [Source:HGNC Symbol;Acc:HGNC:25567]	chr1:1447530-1470067	NM_001170535, NM	.	NP_001164006, NP_00	.	protein_coding	ENSG0000019778	55210	25567
4247	CHD7	0.751	0.042	chromodomain helicase DNA binding protein 7 [Source:HGNC Symbol;Acc:HGNC:20626]	chr8:61591336-61780587	NM_017780	XM_017013613, XM_0	NP_001303619, NP_06	XP_011515855, XP_0115	protein_coding	ENSG0000017131	55636	20626
4248	ARSB	0.840	0.042	arylsulfatase B [Source:HGNC Symbol;Acc:HGNC:714]	chr5:78073031-78281910	NM_000046, NM_1	XM_011543390	NP_000037, NP_94200	XP_011541692, XP_0115	protein_coding	ENSG0000011327	411	714
4249	BATF2	-0.871	0.042	basic leucine zipper ATF-like transcription factor 2 [Source:HGNC Symbol;Acc:HGNC:251	chr11:64755414-64764517	NM_001300807, NM	.	NP_001287736, NP_00	.	protein_coding	ENSG0000016806	116071	25163
4250	CEP83	0.839	0.042	centrosomal protein 83 [Source:HGNC Symbol;Acc:HGNC:17966]	chr12:94701471-94853728	NM_001042399, NM	XM_011538428, XM_0	NP_001035858, NP_05	XP_006719500, XP_0115	protein_coding	ENSG0000017358	51134	17966
4251	SLC41A1	0.532	0.042	solute carrier family 41 member 1 [Source:HGNC Symbol;Acc:HGNC:19429]	chr1:205758220-205782304	NM_173854	XM_005245069	NP_776253	XP_005245126	protein_coding	ENSG0000013306	254428	19429
4252	H2AFY	-0.476	0.042	H2A histone family member Y [Source:HGNC Symbol;Acc:HGNC:4740]	chr5:134669589-134735577	NM_001040158, NM	XM_011543732, XM_0	NP_001035248, NP_00	XP_005272189, XP_0115	protein_coding	ENSG0000011364	9555	4740
4253	ABHD18	1.349	0.042	abhydrolase domain containing 18 [Source:HGNC Symbol;Acc:HGNC:26111]	chr4:128838959-128960866	NM_001039717, NM	XM_011532278, XM_0	NP_001034806, NP_00	XP_005263295, XP_0067	protein_coding	ENSG0000016407	80167	26111
4254	WNT9A	-0.643	0.042	Wnt family member 9A [Source:HGNC Symbol;Acc:HGNC:12778]	chr1:228106356-228135599	NM_003395	.	NP_003386	.	protein_coding	ENSG0000014381	7483	12778
4255	TBKBP1	-0.608	0.043	TBK1 binding protein 1 [Source:HGNC Symbol;Acc:HGNC:30140]	chr17:45772629-45789416	NM_014726	XM_011525513, XM_0	NP_055541	XP_005257916, XP_0052	protein_coding	ENSG0000019893	9755	30140
4256	IGIP	0.923	0.043	IgA inducing protein [Source:HGNC Symbol;Acc:HGNC:33847]	chr5:139505519-139508977	NM_001007189	.	NP_001007190	.	protein_coding	ENSG0000018270	492311	33847
4257	MPI	-0.696	0.043	mannose phosphate isomerase [Source:HGNC Symbol;Acc:HGNC:7216]	chr15:75182347-75199462	NM_001289155, NM	XM_011521593, XM_0	NP_001276084, NP_00	XP_011519894, XP_0115	protein_coding	ENSG0000017880	4351	7216
4258	ATP11A	0.568	0.043	ATPase phospholipid transporting 11A [Source:HGNC Symbol;Acc:HGNC:13552]	chr13:113344642-113541482	NM_015205, NM_0	XM_011537480, XM_0	NP_056020, NP_11556	XP_005268356, XP_0052	protein_coding	ENSG0000006865	23250	13552
4259	MEGF8	-0.521	0.043	multiple EGF like domains 8 [Source:HGNC Symbol;Acc:HGNC:3233]	chr19:42829760-42882921	NM_001271938, NM	.	NP_001258867, NP_00	.	protein_coding	ENSG0000010542	1954	3233
4260	MED7	0.732	0.043	mediator complex subunit 7 [Source:HGNC Symbol;Acc:HGNC:2378]	chr5:156564422-156570195	NM_001100816, NM	.	NP_001094286, NP_00	.	protein_coding	ENSG0000015586	9443	2378
4261	RAB31	0.630	0.043	RAB31, member RAS oncogene family [Source:HGNC Symbol;Acc:HGNC:9771]	chr18:9708161-9862548	NM_006868	.	NP_006859	.	protein_coding	ENSG0000016846	11031	9771
4262	LAT	-0.600	0.043	linker for activation of T-cells [Source:HGNC Symbol;Acc:HGNC:18874]	chr16:28996146-29002104	NM_001014987, NM	.	NP_001014987, NP_00	.	protein_coding	ENSG0000021365	27040	18874
4263	DNM2	-0.488	0.043	dynamin 2 [Source:HGNC Symbol;Acc:HGNC:2974]	chr19:10828754-10942579	NM_001005360, NM	.	NP_001005360, NP_00	.	protein_coding	ENSG0000007980	1785	2974
4264	KCND1	-0.822	0.043	potassium voltage-gated channel subfamily D member 1 [Source:HGNC Symbol;Acc:HG	chrX:48818638-48827976	NM_004979	XM_011543910	NP_004970	XP_011542212	protein_coding	ENSG0000010205	3750	6237

4265	TCF20	0.502	0.043	transcription factor 20 [Source:HGNC Symbol;Acc:HGNC:11631]	chr22:42556018-42611448	NM_005650, NM_1	XM_011530354, XM_0	NP_005641, NP_85246	XP_005261779, XP_0067	protein_coding	ENSG0000010020	6942	11631
4266	E4F1	-0.579	0.043	E4F transcription factor 1 [Source:HGNC Symbol;Acc:HGNC:3121]	chr16:2273566-2285743	NM_001288776, NN	XM_011522403, XM_0	NP_001275705, NP_00	XP_005255212, XP_0052	protein_coding	ENSG0000016796	1877	3121
4267	KIF3B	0.522	0.043	kinesin family member 3B [Source:HGNC Symbol;Acc:HGNC:6320]	chr20:30865466-30922814	NM_004798	.	NP_004789	.	protein_coding	ENSG0000010135	9371	6320
4268	NKAIN1	-0.777	0.043	Na+/K+ transporting ATPase interacting 1 [Source:HGNC Symbol;Acc:HGNC:25743]	chr1:31652591-31712401	NM_024522	.	NP_078798	.	protein_coding	ENSG0000008462	79570	25743
4269	ZNF567	0.843	0.043	zinc finger protein 567 [Source:HGNC Symbol;Acc:HGNC:28696]	chr19:37178529-37263727	NM_001300979, NN	XM_017026421, XM_0	NP_001287908, NP_00	XP_011524878, XP_0115	protein_coding	ENSG0000018904	163081	28696
4270	HSD17B12	0.511	0.043	hydroxysteroid 17-beta dehydrogenase 12 [Source:HGNC Symbol;Acc:HGNC:18646]	chr11:43702107-43878160	NM_016142	XM_011520156	NP_057226	XP_011518458	protein_coding	ENSG0000014908	51144	18646
4271	MID2	0.708	0.043	midline 2 [Source:HGNC Symbol;Acc:HGNC:7096]	chrX:107069108-107170423	NM_012216, NM_0	XM_005262062	NP_036348, NP_43811	XP_005262119, XP_0168	protein_coding	ENSG0000008056	11043	7096
4272	SC5D	0.627	0.043	sterol-C5-desaturase [Source:HGNC Symbol;Acc:HGNC:10547]	chr11:121163161-121179403	NM_001024956, NN	.	NP_001020127, NP_00	.	protein_coding	ENSG0000010992	6309	10547
4273	C2CD4D	-1.583	0.043	C2 calcium dependent domain containing 4D [Source:HGNC Symbol;Acc:HGNC:37210]	chr1:151810294-151813033	NM_001136003	XM_011509056, XM_0	NP_001129475	XP_011507357, XP_0115	protein_coding	ENSG0000022555	100191040	37210
4274	NRP2	0.604	0.043	neuropilin 2 [Source:HGNC Symbol;Acc:HGNC:8005]	chr2:206547223-206662857	NM_003872, NM_0	XM_017005185, XM_0	NP_003863, NP_06100	XP_005246990, XP_0052	protein_coding	ENSG0000011825	8828	8005
4275	RBM7	0.914	0.043	RNA binding motif protein 7 [Source:HGNC Symbol;Acc:HGNC:9904]	chr11:114262169-114284925	NM_001286045, NN	.	NP_001272974, NP_00	.	protein_coding	ENSG0000007605	10179	9904
4276	SHROOM4	0.722	0.043	shroom family member 4 [Source:HGNC Symbol;Acc:HGNC:29215]	chrX:50334646-50557302	NM_020717	XM_011530801, XM_0	NP_065768	XP_006724653	protein_coding	ENSG0000015835	57477	29215
4277	SLC35G1	0.777	0.044	solute carrier family 35 member G1 [Source:HGNC Symbol;Acc:HGNC:26607]	chr10:95653729-95663581	NM_001134658, NN	XM_011539371	NP_001128130, NP_69	XP_011537673	protein_coding	ENSG0000017627	159371	26607
4278	SOD1	-0.453	0.044	superoxide dismutase 1, soluble [Source:HGNC Symbol;Acc:HGNC:11179]	chr21:33031934-33041244	NM_000454	.	NP_000445	.	protein_coding	ENSG0000014216	6647	11179
4279	ALDH2	-0.659	0.044	aldehyde dehydrogenase 2 family (mitochondrial) [Source:HGNC Symbol;Acc:HGNC:404	chr12:112123874-112255333	NM_000690, NM_0	.	NP_000681, NP_00119	.	protein_coding	ENSG0000011127	217	404
4280	SMC1A	-0.480	0.044	structural maintenance of chromosomes 1A [Source:HGNC Symbol;Acc:HGNC:11111]	chrX:53401069-53449677	NM_001281463, NN	.	NP_001268392, NP_00	.	protein_coding	ENSG0000007250	8243	11111
4281	TRMT13	0.842	0.044	tRNA methyltransferase 13 homolog [Source:HGNC Symbol;Acc:HGNC:25502]	chr1:100598705-100643829	NM_019083	XM_005270946, XM_0	NP_061956	XP_005271002, XP_0052	protein_coding	ENSG0000012243	54482	25502
4282	UVSSA	0.737	0.044	UV stimulated scaffold protein A [Source:HGNC Symbol;Acc:HGNC:29304]	chr4:1341053-1381732	NM_001317935, NN	XM_017008494, XM_0	NP_001304863, NP_00	XP_005272347, XP_0067	protein_coding	ENSG0000016394	57654	29304
4283	TFAP4	-0.574	0.044	transcription factor AP-4 [Source:HGNC Symbol;Acc:HGNC:11745]	chr16:4307186-4323076	NM_003223	.	NP_003214	XP_011520935	protein_coding	ENSG0000009044	7023	11745
4284	CPOX	-0.600	0.044	coproporphyrinogen oxidase [Source:HGNC Symbol;Acc:HGNC:2321]	chr3:98298285-98312567	NM_000097	.	NP_000088	.	protein_coding	ENSG0000008081	1371	2321
4285	B4GALT6	0.969	0.044	beta-1,4-galactosyltransferase 6 [Source:HGNC Symbol;Acc:HGNC:929]	chr18:29203131-29264686	NM_004775	XM_006722579, XM_0	NP_004766	XP_005258444, XP_0067	protein_coding	ENSG0000011827	9331	929
4286	KRBOX4	-0.798	0.044	KRAB box domain containing 4 [Source:HGNC Symbol;Acc:HGNC:26007]	chrX:46306291-46356857	NM_001129898, NN	.	NP_001123370, NP_00	.	protein_coding	ENSG0000014712	55634	26007
4287	RPS14	-0.533	0.044	ribosomal protein S14 [Source:HGNC Symbol;Acc:HGNC:10387]	chr5:149822752-149829319	NM_001025070, NN	.	NP_001020241, NP_00	.	protein_coding	ENSG0000016458	6208	10387
4288	ZNF557	0.846	0.044	zinc finger protein 557 [Source:HGNC Symbol;Acc:HGNC:28632]	chr19:7069454-7087979	NM_001044387, NN	.	NP_001037852, NP_00	.	protein_coding	ENSG0000013054	79230	28632
4289	PKIB	1.073	0.044	protein kinase (cAMP-dependent, catalytic) inhibitor beta [Source:HGNC Symbol;Acc:HG	chr6:122793075-123047518	NM_001270393, NN	XM_011535937, XM_0	NP_001257322, NP_00	XP_011534232, XP_0115	protein_coding	ENSG0000013554	5570	9018
4290	SBF1	-0.551	0.044	SET binding factor 1 [Source:HGNC Symbol;Acc:HGNC:10542]	chr22:50781732-50913453	NM_002972	XM_017028905, XM_0	NP_002963	XP_005261992, XP_0115	protein_coding	ENSG0000010024	6305	10542
4291	ATP6V0D1	-0.516	0.044	ATPase H+ transporting V0 subunit d1 [Source:HGNC Symbol;Acc:HGNC:13724]	chr16:67471921-67515140	NM_004691	XM_011523447, XM_0	NP_004682	XP_011521746, XP_0115	protein_coding	ENSG0000015972	9114	13724
4292	ENOX2	0.659	0.044	ecto-NOX disulfide-thiol exchanger 2 [Source:HGNC Symbol;Acc:HGNC:2259]	chrX:129757342-130037291	NM_001281736, NN	XM_011531252, XM_0	NP_001268665, NP_00	XP_005262411, XP_0115	protein_coding	ENSG0000016567	10495	2259
4293	SERINC3	0.554	0.044	serine incorporator 3 [Source:HGNC Symbol;Acc:HGNC:11699]	chr20:43124864-43150750	NM_006811, NM_1	XM_017027601	NP_006802, NP_94517	XP_016883090	protein_coding	ENSG0000013282	10955	11699
4294	SOCS6	0.760	0.044	suppressor of cytokine signaling 6 [Source:HGNC Symbol;Acc:HGNC:16833]	chr18:67956136-67997436	NM_004232	XM_017026087, XM_0	NP_004223	XP_005266840, XP_0168	protein_coding	ENSG0000017067	9306	16833
4295	ENO1	-0.512	0.044	enolase 1 [Source:HGNC Symbol;Acc:HGNC:3350]	chr1:8921060-8938749	NM_001428	XM_006710433	NP_001419	XP_006710496	protein_coding	ENSG0000007480	2023	3350
4296	PRSS33	-0.806	0.044	protease, serine 33 [Source:HGNC Symbol;Acc:HGNC:30405]	chr16:2833953-2837949	NM_152891	XM_011522452, XM_0	NP_690851	XP_011520753, XP_0115	protein_coding	ENSG0000010335	260429	30405
4297	CRIM1	0.556	0.044	cysteine rich transmembrane BMP regulator 1 [Source:HGNC Symbol;Acc:HGNC:2359]	chr2:36583068-36778278	NM_016441	XM_011532901, XM_0	NP_057525	XP_005264414, XP_0115	protein_coding	ENSG0000015093	51232	2359
4298	SDCBP2	-0.786	0.044	syndecan binding protein 2 [Source:HGNC Symbol;Acc:HGNC:15756]	chr20:1290552-1309883	NM_001199784, NN	.	NP_001186713, NP_05	.	protein_coding	ENSG0000012577	27111	15756
4299	LYPD6B	0.636	0.044	LY6/PLAUR domain containing 6B [Source:HGNC Symbol;Acc:HGNC:27018]	chr2:149894980-150071776	NM_001317004, NN	XM_011510624, XM_0	NP_001303931, NP_00	XP_005246361, XP_0052	protein_coding	ENSG0000015055	130576	27018
4300	MFSD4B	0.749	0.044	major facilitator superfamily domain containing 4B [Source:HGNC Symbol;Acc:HGNC:21	chr6:111580550-111592370	NM_153369	.	NP_699200	.	protein_coding	ENSG0000017321	91749	21053
4301	CSRNP2	0.594	0.044	cysteine and serine rich nuclear protein 2 [Source:HGNC Symbol;Acc:HGNC:16006]	chr12:51454987-51477333	NM_030809	XM_006719621	NP_110436	XP_006719684	protein_coding	ENSG0000011092	81566	16006
4302	DACT3	-0.970	0.044	dishevelled binding antagonist of beta catenin 3 [Source:HGNC Symbol;Acc:HGNC:3074	chr19:47150868-47164395	NM_001301046, NN	XM_011526500, XM_0	NP_001287975, NP_65	XP_011524800, XP_0115	protein_coding	ENSG0000019738	147906	30745
4303	SLC16A13	-0.699	0.044	solute carrier family 16 member 13 [Source:HGNC Symbol;Acc:HGNC:31037]	chr17:6939393-6943440	NM_201566	.	NP_963860	.	protein_coding	ENSG0000017432	201232	31037
4304	GPRIN2	-0.985	0.044	G protein regulated inducer of neurite outgrowth 2 [Source:HGNC Symbol;Acc:HGNC:23	chr10:46994086-47000573	NM_014696	XM_011540400, XM_0	NP_055511	XP_005277723, XP_0115	protein_coding	ENSG0000020417	9721	23730
4305	GFM1	0.540	0.044	G elongation factor mitochondrial 1 [Source:HGNC Symbol;Acc:HGNC:13780]	chr3:158362066-158410364	NM_001308164, NN	XM_011513247, XM_0	NP_001295093, NP_00	XP_006713858, XP_0115	protein_coding	ENSG0000016882	85476	13780
4306	MTCH1	-0.475	0.044	mitochondrial carrier 1 [Source:HGNC Symbol;Acc:HGNC:17586]	chr6:36935916-36954074	NM_001271641, NN	XM_011514450, XM_0	NP_001258570, NP_05	XP_005249035, XP_0052	protein_coding	ENSG0000013740	23787	17586
4307	ALDH3B1	-0.611	0.045	aldehyde dehydrogenase 3 family member B1 [Source:HGNC Symbol;Acc:HGNC:410]	chr11:67776047-67796743	NM_000694, NM_0	.	NP_000685, NP_00102	.	protein_coding	ENSG0000000653	221	410
4308	CACNA1S	-5.865	0.045	calcium voltage-gated channel subunit alpha1 S [Source:HGNC Symbol;Acc:HGNC:1397]	chr1:201008639-201081694	NM_000069	XM_005245478	NP_000060	XP_005245535	protein_coding	ENSG0000008124	779	1397
4309	CLPP	-0.518	0.045	caseinolytic mitochondrial matrix peptidase proteolytic subunit [Source:HGNC Symbol;A	chr19:6361462-6368919	NM_006012	.	NP_006003	.	protein_coding	ENSG0000012565	8192	2084
4310	DDX52	0.545	0.045	DEAD-box helicase 52 [Source:HGNC Symbol;Acc:HGNC:20038]	chr17:35969825-36003493	NM_001291476, NN	XM_011524232	NP_001278405, NP_00	XP_011522534	protein_coding	ENSG0000027805	11056	20038
4311	PKP3	-0.485	0.045	plakophilin 3 [Source:HGNC Symbol;Acc:HGNC:9025]	chr11:394216-404908	NM_001303029, NN	.	NP_001289958, NP_00	.	protein_coding	ENSG0000018436	11187	9025
4312	RAB11FIP5	-0.520	0.045	RAB11 family interacting protein 5 [Source:HGNC Symbol;Acc:HGNC:24845]	chr2:73300509-73340146	NM_015470	XM_006711985, XM_0	NP_056285	XP_005264308, XP_0052	protein_coding	ENSG0000013563	26056	24845
4313	SMKR1	0.707	0.045	small lysine rich protein 1 [Source:HGNC Symbol;Acc:HGNC:43561]	chr7:129142319-129152773	NM_001195243	.	NP_001182172	.	protein_coding	ENSG0000024020	100287482	43561
4314	ZNF48	-0.595	0.045	zinc finger protein 48 [Source:HGNC Symbol;Acc:HGNC:13114]	chr16:30406432-30411429	NM_001214906, NN	.	NP_001201835, NP_00	.	protein_coding	ENSG0000018003	197407	13114
4315	ZSCAN16	0.737	0.045	zinc finger and SCAN domain containing 16 [Source:HGNC Symbol;Acc:HGNC:20813]	chr6:28092337-28097860	NM_025231	XM_011514919	NP_001307484, NP_07	XP_011513221, XP_0168	protein_coding	ENSG0000019681	80345	20813
4316	TRADD	-0.618	0.045	TNFRSF1A associated via death domain [Source:HGNC Symbol;Acc:HGNC:12030]	chr16:67188082-67198079	NM_003789	XM_011523420, XM_0	NP_001310481, NP_00	XP_005256270, XP_0115	protein_coding	ENSG0000010287	8717	12030
4317	COPS8	0.636	0.045	COP9 signalosome subunit 8 [Source:HGNC Symbol;Acc:HGNC:24335]	chr2:237993954-238009109	NM_006710, NM_1	.	NP_006701, NP_93783	.	protein_coding	ENSG0000019861	10920	24335
4318	GTF2I	0.476	0.045	general transcription factor IIi [Source:HGNC Symbol;Acc:HGNC:4659]	chr7:74072010-74175022	NM_001163636, NN	XM_011516110	NP_001157108, NP_00	.	protein_coding	ENSG0000026300	2969	4659

4319	P4HA1	0.635	0.045	prolyl 4-hydroxylase subunit alpha 1 [Source:HGNC Symbol;Acc:HGNC:8546]	chr10:74766974-74856732	NM_000917, NM_0	.	NP_000908, NP_00101	.	protein_coding	ENSG0000012288	5033	8546
4320	SP1	0.523	0.045	Sp1 transcription factor [Source:HGNC Symbol;Acc:HGNC:11205]	chr12:53773977-53810230	NM_001251825, NN	XM_011538696	NP_001238754, NP_00	XP_011536998	protein_coding	ENSG0000018559	6667	11205
4321	CDX2	-0.551	0.045	caudal type homeobox 2 [Source:HGNC Symbol;Acc:HGNC:1806]	chr13:28536273-28545276	NM_001265	XM_011534877, XM_0	NP_001256	XP_011533177, XP_0115	protein_coding	ENSG0000016555	1045	1806
4322	ZNF443	1.149	0.045	zinc finger protein 443 [Source:HGNC Symbol;Acc:HGNC:20878]	chr19:12500829-12551926	NM_005815	.	NP_005806	XP_011525924, XP_0115	protein_coding	ENSG0000018085	10224	20878
4323	TSR3	-0.603	0.045	TSR3, acp transferase ribosome maturation factor [Source:HGNC Symbol;Acc:HGNC:141	chr16:1383601-1401912	NM_001001410	.	NP_001001410	.	protein_coding	ENSG0000000752	115939	14175
4324	DIAPH2	0.722	0.045	diaphanous related formin 2 [Source:HGNC Symbol;Acc:HGNC:2877]	chrX:95939661-96859996	NM_006729, NM_0	.	NP_006720, NP_00929	.	protein_coding	ENSG0000014720	1730	2877
4325	SGF29	-0.681	0.045	SAGA complex associated factor 29 [Source:HGNC Symbol;Acc:HGNC:25156]	chr16:28565246-28603111	NM_138414	.	NP_612423	.	protein_coding	ENSG0000017647	112869	25156
4326	WBSCR27	-0.606	0.045	Williams Beuren syndrome chromosome region 27 [Source:HGNC Symbol;Acc:HGNC:19	chr7:73248919-73256865	NM_152559	XM_011515839	NP_689772	XP_011514141	protein_coding	ENSG0000016517	155368	19068
4327	DDI2	0.644	0.045	DNA damage inducible 1 homolog 2 [Source:HGNC Symbol;Acc:HGNC:24578]	chr1:15943994-15995539	NM_032341	.	NP_115717	.	protein_coding	ENSG0000019731	84301	24578
4328	NAPRT	-0.516	0.045	nicotinate phosphoribosyltransferase [Source:HGNC Symbol;Acc:HGNC:30450]	chr8:144656954-144660783	NM_001286829, NN	XM_011517377	NP_001273758, NP_66	.	protein_coding	ENSG0000014781	93100	30450
4329	GDPD3	-1.192	0.045	glycerophosphodiester phosphodiesterase domain containing 3 [Source:HGNC Symbol;A	chr16:30116130-30125177	NM_024307	.	NP_077283	.	protein_coding	ENSG0000010288	79153	28638
4330	C1orf210	-0.674	0.045	chromosome 1 open reading frame 210 [Source:HGNC Symbol;Acc:HGNC:28755]	chr1:43747553-43751288	NM_001164829, NN	XM_011540802	NP_001158301, NP_87	XP_011539104	protein_coding	ENSG0000025331	149466	28755
4331	DCAF17	1.334	0.045	DDB1 and CUL4 associated factor 17 [Source:HGNC Symbol;Acc:HGNC:25784]	chr2:172173914-172341562	NM_001164821, NN	XM_011511882, XM_0	NP_001158293, NP_07	XP_006712830, XP_0067	protein_coding	ENSG0000011582	80067	25784
4332	PRX	-0.638	0.045	periaxin [Source:HGNC Symbol;Acc:HGNC:13797]	chr19:40899674-40919273	NM_020956, NM_1	XM_017027047, XM_0	NP_066007, NP_87099	XP_011525473, XP_0168	protein_coding	ENSG0000010522	57716	13797
4333	KIAA0368	0.515	0.045	KIAA0368 [Source:HGNC Symbol;Acc:HGNC:29020]	chr9:114122971-114247025	NM_001080398	XM_011518422, XM_0	NP_001073867	XP_005251907, XP_0052	protein_coding	ENSG0000013681	23392	29020
4334	RPN1	-0.455	0.045	ribophorin I [Source:HGNC Symbol;Acc:HGNC:10381]	chr3:128338812-128399918	NM_002950	.	NP_002941	.	protein_coding	ENSG0000016390	6184	10381
4335	RRP1B	0.480	0.045	ribosomal RNA processing 1B [Source:HGNC Symbol;Acc:HGNC:23818]	chr21:45079428-45115960	NM_015056	.	NP_055871	.	protein_coding	ENSG0000016020	23076	23818
4336	CTPS2	-0.634	0.045	CTP synthase 2 [Source:HGNC Symbol;Acc:HGNC:2520]	chrX:16606125-16731059	NM_001144002, NN	XM_011545546, XM_0	NP_001137474, NP_06	XP_005274619, XP_0067	protein_coding	ENSG0000004723	56474	2520
4337	EED	0.594	0.045	embryonic ectoderm development [Source:HGNC Symbol;Acc:HGNC:3188]	chr11:85955585-85989855	NM_001308007, NN	XM_011545330, XM_0	NP_001294936, NP_00	XP_005274430, XP_0115	protein_coding	ENSG0000007426	102465452	3188
4338	POLR2M	0.730	0.045	RNA polymerase II subunit M [Source:HGNC Symbol;Acc:HGNC:14862]	chr15:57884105-58009755	NM_001018102, NN	.	NP_001018112, NP_05	.	protein_coding	ENSG0000025552	81488	14862
4339	SMAD1	0.700	0.045	SMAD family member 1 [Source:HGNC Symbol;Acc:HGNC:6767]	chr4:146402943-146479231	NM_001003688, NN	XM_011531964, XM_0	NP_001003688, NP_00	XP_005263048, XP_0052	protein_coding	ENSG0000017036	4086	6767
4340	CDK6	0.965	0.045	cyclin dependent kinase 6 [Source:HGNC Symbol;Acc:HGNC:1777]	chr7:92234234-92465908	NM_001145306, NN	XM_011515731, XM_0	NP_001138778, NP_00	XP_006715898, XP_0115	protein_coding	ENSG0000010581	1021	1777
4341	ITGB1	0.515	0.046	integrin subunit beta 1 [Source:HGNC Symbol;Acc:HGNC:6153]	chr10:33189246-33247137	NM_002211, NM_0	XM_005252448	NP_002202, NP_39198	XP_005252505	protein_coding	ENSG0000015009	3688	6153
4342	GRN	-0.465	0.046	granulin [Source:HGNC Symbol;Acc:HGNC:4601]	chr17:42422453-42430470	NM_002087	XM_005257253	NP_002078	XP_005257310	protein_coding	ENSG0000003058	2896	4601
4343	TEC	0.791	0.046	tec protein tyrosine kinase [Source:HGNC Symbol;Acc:HGNC:11719]	chr4:48137799-48271881	NM_003215	XM_011513739, XM_0	NP_003206	XP_011512037, XP_0115	protein_coding	ENSG0000013560	7006	11719
4344	ASGR1	-0.790	0.046	asialoglycoprotein receptor 1 [Source:HGNC Symbol;Acc:HGNC:742]	chr17:7076749-7082883	NM_001197216, NN	XM_011523861, XM_0	NP_001184145, NP_00	XP_011522163	protein_coding	ENSG0000014150	432	742
4345	ANKRD16	-0.723	0.046	ankyrin repeat domain 16 [Source:HGNC Symbol;Acc:HGNC:23471]	chr10:5903579-5931869	NM_001009941, NN	.	NP_001009941, NP_00	.	protein_coding	ENSG0000013446	54522	23471
4346	TRAPPC10	0.650	0.046	trafficking protein particle complex 10 [Source:HGNC Symbol;Acc:HGNC:11868]	chr21:45432199-45526433	NM_003274	XM_016999834, XM_0	NP_003265	XP_005261225, XP_0115	protein_coding	ENSG0000016021	7109	11868
4347	ATP6AP2	-0.575	0.046	ATPase H+ transporting accessory protein 2 [Source:HGNC Symbol;Acc:HGNC:18305]	chrX:40440126-40466100	NM_005765	.	NP_005756	.	protein_coding	ENSG0000018222	10159	18305
4348	CDK5R1	0.656	0.046	cyclin dependent kinase 5 regulatory subunit 1 [Source:HGNC Symbol;Acc:HGNC:1775]	chr17:30813636-30818274	NM_003885	XM_011525438	NP_003876	XP_011523740, XP_0168	protein_coding	ENSG0000017674	8851	1775
4349	POLR2H	-0.486	0.046	RNA polymerase II subunit H [Source:HGNC Symbol;Acc:HGNC:9195]	chr3:184079505-184086384	NM_001278698, NN	XM_006713670, XM_0	NP_001265627, NP_00	XP_005247598, XP_0067	protein_coding	ENSG0000016388	5437	9195
4350	RITA1	-0.541	0.046	RBPJ interacting and tubulin associated 1 [Source:HGNC Symbol;Acc:HGNC:25925]	chr12:113623330-113630173	NM_001286215, NN	XM_005253972	NP_001273144, NP_11	XP_005254029	protein_coding	ENSG0000013940	84934	25925
4351	GPS1	-0.484	0.046	G protein pathway suppressor 1 [Source:HGNC Symbol;Acc:HGNC:4549]	chr17:80009740-80015346	NM_001321092, NN	XM_017024535, XM_0	NP_001308018, NP_00	XP_005256407, XP_0052	protein_coding	ENSG0000016972	2873	4549
4352	KRT86	-1.879	0.046	keratin 86 [Source:HGNC Symbol;Acc:HGNC:6463]	chr12:52668458-52702947	NM_001320198, NN	XM_011538338, XM_0	NP_001307127, NP_00	XP_005268923, XP_0115	protein_coding	ENSG0000017044	3892	6463
4353	POGZ	0.591	0.046	pogo transposable element with ZNF domain [Source:HGNC Symbol;Acc:HGNC:18801]	chr1:151375199-151431941	NM_001194937, NN	XM_017000749, XM_0	NP_001181866, NP_00	XP_005245056, XP_0052	protein_coding	ENSG0000014344	23126	18801
4354	SMG1	0.918	0.046	SMG1, nonsense mediated mRNA decay associated PI3K related kinase [Source:HGNC S	chr16:18816174-18937776	NM_015092	XM_011545769, XM_0	NP_055907	XP_005255239, XP_0052	protein_coding	ENSG0000015710	23049	30045
4355	CSGALNACT2	0.719	0.046	chondroitin sulfate N-acetylgalactosaminyltransferase 2 [Source:HGNC Symbol;Acc:HGN	chr10:43633933-43680756	NM_018590	.	NP_061060	.	protein_coding	ENSG0000016982	55454	24292
4356	CDK2AP2	-0.512	0.046	cyclin dependent kinase 2 associated protein 2 [Source:HGNC Symbol;Acc:HGNC:30833	chr11:67273967-67276102	NM_005851	.	NP_005842	.	protein_coding	ENSG0000016779	10263	30833
4357	HAGHL	-0.625	0.046	hydroxyacylglutathione hydrolase-like [Source:HGNC Symbol;Acc:HGNC:14177]	chr16:776935-779733	NM_001290137, NN	XM_011522712, XM_0	NP_001277066, NP_00	XP_005255688, XP_0052	protein_coding	ENSG0000010325	84264	14177
4358	SPRED1	0.617	0.046	sprouty related EVH1 domain containing 1 [Source:HGNC Symbol;Acc:HGNC:20249]	chr15:38544526-38649450	NM_152594	XM_011521290, XM_0	NP_689807	XP_005254259, XP_0115	protein_coding	ENSG0000016606	161742	20249
4359	OPN3	1.106	0.046	opsin 3 [Source:HGNC Symbol;Acc:HGNC:14007]	chr1:241695433-241803663	NM_014322	.	NP_055137	.	protein_coding	ENSG0000005427	23596	14007
4360	DCBLD1	0.900	0.047	discoidin, CUB and LCCL domain containing 1 [Source:HGNC Symbol;Acc:HGNC:21479]	chr6:117609462-117923705	NM_173674	XM_017010775, XM_0	NP_775945	XP_005266998, XP_0067	protein_coding	ENSG0000016446	285761	21479
4361	DSTYK	0.586	0.047	dual serine/threonine and tyrosine protein kinase [Source:HGNC Symbol;Acc:HGNC:290	chr1:205111632-205180694	NM_015375, NM_1	XM_011509394, XM_0	NP_056190, NP_95574	XP_011507694, XP_0115	protein_coding	ENSG0000013305	25778	29043
4362	SRRM1	0.535	0.047	serine and arginine repetitive matrix 1 [Source:HGNC Symbol;Acc:HGNC:16638]	chr1:24969502-24999758	NM_001303448, NN	XM_017000019, XM_0	NP_001290377, NP_00	XP_005245774, XP_0052	protein_coding	ENSG0000013322	10250	16638
4363	ZNF805	0.863	0.047	zinc finger protein 805 [Source:HGNC Symbol;Acc:HGNC:23272]	chr19:57752052-57766503	NM_001023563, NN	.	NP_001018857, NP_00	.	protein_coding	ENSG0000020452	390980	23272
4364	FOXP4	-0.574	0.047	forkhead box P4 [Source:HGNC Symbol;Acc:HGNC:20842]	chr6:41514163-41570122	NM_001012426, NN	XM_011514293, XM_0	NP_001012426, NP_00	XP_006715054, XP_0115	protein_coding	ENSG0000013716	116113	20842
4365	MBD3	-0.488	0.047	methyl-CpG binding domain protein 3 [Source:HGNC Symbol;Acc:HGNC:6918]	chr19:1573594-1592800	NM_001281453, NN	.	NP_001268382, NP_00	.	protein_coding	ENSG0000007165	53615	6918
4366	ACVR1	0.630	0.047	activin A receptor type 1 [Source:HGNC Symbol;Acc:HGNC:171]	chr2:158592955-158732374	NM_001105, NM_0	XM_011512108, XM_0	NP_001096, NP_00110	XP_005246996, XP_0052	protein_coding	ENSG0000011517	90	171
4367	EXOSC5	-0.866	0.047	exosome component 5 [Source:HGNC Symbol;Acc:HGNC:24662]	chr19:41836227-41930910	NM_020158	.	NP_064543	.	protein_coding	ENSG0000007734	56915	24662
4368	RPS16	-0.479	0.047	ribosomal protein S16 [Source:HGNC Symbol;Acc:HGNC:10396]	chr19:39923846-39926588	NM_001020	XM_005259137	NP_001011, NP_00130	XP_005259194	protein_coding	ENSG0000010519	6217	10396
4369	SLC8B1	-0.608	0.047	solute carrier family 8 member B1 [Source:HGNC Symbol;Acc:HGNC:26175]	chr12:113736563-113772925	NM_024959	XM_011538753, XM_0	NP_079235	XP_006719670, XP_0115	protein_coding	ENSG0000008906	80024	26175
4370	LLGL2	-0.539	0.047	LLGL2, scribble cell polarity complex component [Source:HGNC Symbol;Acc:HGNC:6629	chr17:73521806-73571289	NM_001015002, NN	XM_017024631, XM_0	NP_001015002, NP_00	XP_006721960, XP_0115	protein_coding	ENSG0000007335	3993	6629
4371	NR6A1	0.729	0.047	nuclear receptor subfamily 6 group A member 1 [Source:HGNC Symbol;Acc:HGNC:7985	chr9:127279553-127533589	NM_001278546, NN	XM_005251917	NP_001265475, NP_00	XP_005251974, XP_0052	protein_coding	ENSG0000014820	2649	7985
4372	EPHA4	0.554	0.047	EPH receptor A4 [Source:HGNC Symbol;Acc:HGNC:3388]	chr2:222282748-222438922	NM_001304536, NN	XM_005246374	NP_001291465, NP_00	XP_005246431	protein_coding	ENSG0000011610	2043	3388

4373	ZNF79	0.749	0.047	zinc finger protein 79 [Source:HGNC Symbol;Acc:HGNC:13153]	chr9:130186652-130207651	NM_001286696, NM	XM_011518989, XM_0	NP_001273625, NP_00	XP_006717342, XP_0115	protein_coding	ENSG00000196152	7633	13153
4374	ARHGAP44	-0.724	0.047	Rho GTPase activating protein 44 [Source:HGNC Symbol;Acc:HGNC:29096]	chr17:12692855-12894960	NM_001321166, NM	XM_006721615, XM_0	NP_001308093, NP_00	XP_005256945, XP_0052	protein_coding	ENSG00000006740	9912	29096
4375	CHML	1.072	0.047	CHM like, Rab escort protein 2 [Source:HGNC Symbol;Acc:HGNC:1941]	chr1:241695433-241803663	NM_001821	.	NP_001812	.	protein_coding	ENSG00000203668	1122	1941
4376	C19orf57	-0.765	0.047	chromosome 19 open reading frame 57 [Source:HGNC Symbol;Acc:HGNC:28153]	chr19:13993167-14016978	NM_024323	XM_006722885	NP_077299	XP_006722948	protein_coding	ENSG00000132016	79173	28153
4377	KYAT1	-0.567	0.047	kynurenine aminotransferase 1 [Source:HGNC Symbol;Acc:HGNC:1564]	chr9:131595220-131644354	NM_001122671, NM	XM_017015261, XM_0	NP_001116143, NP_00	XP_006717374, XP_0115	protein_coding	ENSG00000171097	883	1564
4378	SLC25A43	0.589	0.047	solute carrier family 25 member 43 [Source:HGNC Symbol;Acc:HGNC:30557]	chrX:118533022-118588441	NM_145305	.	NP_660348	.	protein_coding	ENSG00000077713	203427	30557
4379	PRMT9	1.154	0.047	protein arginine methyltransferase 9 [Source:HGNC Symbol;Acc:HGNC:25099]	chr4:148538533-148605381	NM_001304458, NM	XM_005263338	NP_001291387, NP_61	.	protein_coding	ENSG00000164169	90826	25099
4380	RP5-862P8.2	0.701	0.047	Mitogen-activated protein kinase kinase kinase MLK4 [Source:UniProtKB/Swiss-Prot;Ac	chr1:233463513-233520894	NM_032435	XM_011544305	NP_115811	XP_011542607	protein_coding	ENSG00000143674	84451	.
4381	RPLP2	-0.466	0.047	ribosomal protein lateral stalk subunit P2 [Source:HGNC Symbol;Acc:HGNC:10377]	chr11:809646-812875	NM_001004	.	NP_000995	.	protein_coding	ENSG00000177600	6181	10377
4382	CCL11	-2.312	0.047	C-C motif chemokine ligand 11 [Source:HGNC Symbol;Acc:HGNC:10610]	chr17:32612686-32615353	NM_002986	.	NP_002977	.	protein_coding	ENSG00000172156	6356	10610
4383	CEP95	0.859	0.047	centrosomal protein 95 [Source:HGNC Symbol;Acc:HGNC:25141]	chr17:62502705-62534060	NM_138363	XM_006722167, XM_0	NP_612372	XP_005257836, XP_0052	protein_coding	ENSG00000258890	90799	25141
4384	BAG6	-0.473	0.047	BCL2 associated athanogene 6 [Source:HGNC Symbol;Acc:HGNC:13919]	chr6:31606804-31625987	NM_001098534, NM	XM_011514908, XM_0	NP_001092004, NP_00	XP_011513194, XP_0115	protein_coding	ENSG00000204463	7917	13919
4385	NTMT1	-0.586	0.047	N-terminal Xaa-Pro-Lys N-methyltransferase 1 [Source:HGNC Symbol;Acc:HGNC:23373]	chr9:132371162-132404448	NM_001286796, NM	XM_005251939	NP_001273725, NP_00	XP_005251996, XP_0168	protein_coding	ENSG00000148335	28989	23373
4386	PRR14	-0.586	0.047	proline rich 14 [Source:HGNC Symbol;Acc:HGNC:28458]	chr16:30662187-30667761	NM_024031	XM_005255566	NP_001307393, NP_07	XP_005255623	protein_coding	ENSG00000156858	78994	28458
4387	UBE2W	0.832	0.047	ubiquitin conjugating enzyme E2 W (putative) [Source:HGNC Symbol;Acc:HGNC:25616]	chr8:74698454-74791145	NM_001001481, NM	.	NP_001001481, NP_00	.	protein_coding	ENSG00000104343	55284	25616
4388	HSPG2	-0.501	0.047	heparan sulfate proteoglycan 2 [Source:HGNC Symbol;Acc:HGNC:5273]	chr1:22138757-22263790	NM_001291860, NM	XM_017001122, XM_0	NP_001278789, NP_00	XP_011539620, XP_0168	protein_coding	ENSG00000142798	3339	5273
4389	CTU1	-0.676	0.047	cytosolic thiouridylase subunit 1 [Source:HGNC Symbol;Acc:HGNC:29590]	chr19:51600862-51611627	NM_145232	.	NP_660275	.	protein_coding	ENSG00000142544	90353	29590
4390	SGPL1	0.494	0.047	sphingosine-1-phosphate lyase 1 [Source:HGNC Symbol;Acc:HGNC:10817]	chr10:72575725-72640930	NM_003901	XM_011540319, XM_0	NP_003892	XP_005270320, XP_0067	protein_coding	ENSG00000166224	8879	10817
4391	SOWAHC	0.522	0.047	sosondowah ankyrin repeat domain family member C [Source:HGNC Symbol;Acc:HGNC:	chr2:110371910-110376567	NM_023016	.	NP_075392	.	protein_coding	ENSG00000198142	65124	26149
4392	RRN3	1.055	0.048	RRN3 homolog, RNA polymerase I transcription factor [Source:HGNC Symbol;Acc:HGNC	chr16:15068447-15233196	NM_001301064, NM	XM_011522542, XM_0	NP_001287993, NP_06	XP_005255433, XP_0052	protein_coding	ENSG00000085721	54700	30346
4393	ZNF222	0.884	0.048	zinc finger protein 222 [Source:HGNC Symbol;Acc:HGNC:13015]	chr19:44529493-44537265	NM_001129996, NM	.	NP_001123468, NP_03	XP_005259265, XP_0052	protein_coding	ENSG00000159885	7673	13015
4394	ZNF514	0.671	0.048	zinc finger protein 514 [Source:HGNC Symbol;Acc:HGNC:25894]	chr2:95813681-95825352	NM_032788	XM_006712806, XM_0	NP_116177	XP_006712869	protein_coding	ENSG00000144026	84874	25894
4395	LRFN1	-0.655	0.048	leucine rich repeat and fibronectin type III domain containing 1 [Source:HGNC Symbol;A	chr19:39797207-39805976	NM_020862	XM_017027033, XM_0	NP_065913	XP_005259159, XP_0168	protein_coding	ENSG00000128011	57622	29290
4396	GPN1	0.778	0.048	GPN-loop GTPase 1 [Source:HGNC Symbol;Acc:HGNC:17030]	chr2:27848505-27917843	NM_001145047, NM	.	NP_001138519, NP_00	.	protein_coding	ENSG00000198522	11321	17030
4397	MROH1	-0.731	0.048	maestro heat like repeat family member 1 [Source:HGNC Symbol;Acc:HGNC:26958]	chr8:145202938-145316843	NM_001099280, NM	XM_011517283, XM_0	NP_001092750, NP_00	XP_011515572, XP_0115	protein_coding	ENSG00000179832	727957	26958
4398	ZC3H15	0.507	0.048	zinc finger CCCH-type containing 15 [Source:HGNC Symbol;Acc:HGNC:29528]	chr2:187350882-187374090	NM_018471	.	NP_060941	.	protein_coding	ENSG00000065548	55854	29528
4399	TRUB2	-0.645	0.048	TruB pseudouridine synthase family member 2 [Source:HGNC Symbol;Acc:HGNC:17170]	chr9:131067439-131096351	NM_015679	.	NP_056494	.	protein_coding	ENSG00000167112	26995	17170
4400	ERI1	0.770	0.048	exoribonuclease 1 [Source:HGNC Symbol;Acc:HGNC:23994]	chr8:8859656-8890851	NM_153332	XM_011543851, XM_0	NP_699163	XP_005272458, XP_0115	protein_coding	ENSG00000104626	90459	23994
4401	AMPD2	-0.526	0.048	adenosine monophosphate deaminase 2 [Source:HGNC Symbol;Acc:HGNC:469]	chr1:110162458-110174673	NM_001257360, NM	XM_011541247, XM_0	NP_001244289, NP_00	XP_005270809	protein_coding	ENSG00000116337	271	469
4402	CREBL2	0.569	0.048	cAMP responsive element binding protein like 2 [Source:HGNC Symbol;Acc:HGNC:2350]	chr12:12764825-12798042	NM_001310	.	NP_001301	.	protein_coding	ENSG00000111269	1389	2350
4403	SLC43A2	-0.676	0.048	solute carrier family 43 member 2 [Source:HGNC Symbol;Acc:HGNC:23087]	chr17:1472560-1532130	NM_001284498, NM	XM_017024178, XM_0	NP_001271427, NP_00	XP_011521947, XP_0115	protein_coding	ENSG00000167703	124935	23087
4404	C7orf31	0.806	0.048	chromosome 7 open reading frame 31 [Source:HGNC Symbol;Acc:HGNC:21722]	chr7:25174315-25219973	NM_138811	XM_005249620, XM_0	NP_620166	XP_005249676, XP_0052	protein_coding	ENSG00000153790	136895	21722
4405	PLEKHM2	-0.501	0.048	pleckstrin homology and RUN domain containing M2 [Source:HGNC Symbol;Acc:HGNC:2	chr1:16010826-16061264	NM_015164	XM_017000758, XM_0	NP_055979	XP_005245847, XP_0168	protein_coding	ENSG00000116786	23207	29131
4406	ZNF554	-0.750	0.048	zinc finger protein 554 [Source:HGNC Symbol;Acc:HGNC:26629]	chr19:2819871-2835771	NM_001102651	.	NP_001096121	XP_011525962, XP_0115	protein_coding	ENSG00000172006	115196	26629
4407	FANCL	0.770	0.048	Fanconi anemia complementation group L [Source:HGNC Symbol;Acc:HGNC:20748]	chr2:58134785-58468485	NM_001114636, NM	XM_011532945, XM_0	NP_001108108, NP_06	XP_005264452, XP_0052	protein_coding	ENSG00000115392	55120	20748
4408	HS3ST6	-2.482	0.048	heparan sulfate-glucosamine 3-sulfotransferase 6 [Source:HGNC Symbol;Acc:HGNC:141	chr16:1961463-1968441	NM_001009606	.	NP_001009606	.	protein_coding	ENSG00000162040	64711	14178
4409	SIRT5	0.668	0.048	sirtuin 5 [Source:HGNC Symbol;Acc:HGNC:14933]	chr6:13574760-13615390	NM_001193267, NM	XM_011514446, XM_0	NP_001180196, NP_00	XP_005249024, XP_0052	protein_coding	ENSG00000124523	23408	14933
4410	DNAJB6	0.495	0.048	DnaJ heat shock protein family (Hsp40) member B6 [Source:HGNC Symbol;Acc:HGNC:14	chr7:157129659-157210133	NM_005494, NM_0	XM_011515704, XM_0	NP_005485, NP_49064	XP_005249572, XP_0052	protein_coding	ENSG00000105993	10049	14888
4411	ELOF1	-0.516	0.048	elongation factor 1 homolog [Source:HGNC Symbol;Acc:HGNC:28691]	chr19:11661961-11670051	NM_032377	XM_017027357, XM_0	NP_115753	XP_016882845, XP_0168	protein_coding	ENSG00000130165	84337	28691
4412	TRIM56	0.462	0.048	tripartite motif containing 56 [Source:HGNC Symbol;Acc:HGNC:19028]	chr7:100728719-100741248	NM_030961	XM_011516589	NP_112223	XP_011514891	protein_coding	ENSG00000169871	81844	19028
4413	YIPF5	0.590	0.048	Yip1 domain family member 5 [Source:HGNC Symbol;Acc:HGNC:24877]	chr5:143537722-143550223	NM_001024947, NM	.	NP_001020118, NP_00	.	protein_coding	ENSG00000145817	81555	24877
4414	GZF1	0.589	0.049	GDNF inducible zinc finger protein 1 [Source:HGNC Symbol;Acc:HGNC:15808]	chr20:23342818-23353700	NM_001317012, NM	XM_011529322, XM_0	NP_001303941, NP_07	XP_005260854, XP_0052	protein_coding	ENSG00000125812	64412	15808
4415	ZNF428	-0.707	0.049	zinc finger protein 428 [Source:HGNC Symbol;Acc:HGNC:20804]	chr19:44100543-44124026	NM_182498	XM_005258517	NP_872304	XP_005258574	protein_coding	ENSG00000131116	126299	20804
4416	MSI2	0.618	0.049	musashi RNA binding protein 2 [Source:HGNC Symbol;Acc:HGNC:18585]	chr17:55333211-55762046	NM_138962, NM_1	XM_011524286, XM_0	NP_001309179, NP_62	XP_005257071, XP_0052	protein_coding	ENSG00000153944	124540	18585
4417	MEGF11	0.770	0.049	multiple EGF like domains 11 [Source:HGNC Symbol;Acc:HGNC:29635]	chr15:66187416-66546085	NM_032445	XM_017022672, XM_0	NP_115821	XP_016878160, XP_0168	protein_coding	ENSG00000157890	84465	29635
4418	MOV10	-0.527	0.049	Mov10 RISC complex RNA helicase [Source:HGNC Symbol;Acc:HGNC:7200]	chr1:113217042-113243368	NM_001130079, NM	XM_005270870, XM_0	NP_001123551, NP_00	XP_005270925, XP_0052	protein_coding	ENSG00000155363	4343	7200
4419	MIIP	-0.542	0.049	migration and invasion inhibitory protein [Source:HGNC Symbol;Acc:HGNC:25715]	chr1:12079522-12092102	NM_021933	XM_011541896, XM_0	NP_068752	XP_005263544, XP_0115	protein_coding	ENSG00000116691	60672	25715
4420	EBF3	0.820	0.049	early B-cell factor 3 [Source:HGNC Symbol;Acc:HGNC:19087]	chr10:131633546-131762105	NM_001005463	XM_006717744, XM_0	NP_001005463	XP_005252724, XP_0052	protein_coding	ENSG00000108001	253738	19087
4421	TEX30	0.654	0.049	testis expressed 30 [Source:HGNC Symbol;Acc:HGNC:25188]	chr13:103418339-103426161	NM_001286775, NM	XM_011521135, XM_0	NP_001273704, NP_00	XP_005254154, XP_0115	protein_coding	ENSG00000151287	93081	25188
4422	HOXA6	-0.987	0.049	homeobox A6 [Source:HGNC Symbol;Acc:HGNC:5107]	chr7:27185014-27187393	NM_024014	.	NP_076919	.	protein_coding	ENSG00000106006	3203	5107
4423	NUDT14	-0.534	0.049	nudix hydrolase 14 [Source:HGNC Symbol;Acc:HGNC:20141]	chr14:105639275-105647640	NM_177533	.	NP_803877	.	protein_coding	ENSG00000183828	256281	20141
4424	C19orf43	-0.486	0.049	chromosome 19 open reading frame 43 [Source:HGNC Symbol;Acc:HGNC:28424]	chr19:12841453-12845589	NM_024038	.	NP_076943	.	protein_coding	ENSG00000123144	79002	28424
4425	MTFR1	0.559	0.049	mitochondrial fission regulator 1 [Source:HGNC Symbol;Acc:HGNC:29510]	chr8:66556973-66622796	NM_001145838, NM	.	NP_001139310, NP_05	.	protein_coding	ENSG00000066855	9650	29510
4426	CRMP1	-0.533	0.049	collapsin response mediator protein 1 [Source:HGNC Symbol;Acc:HGNC:2365]	chr4:5822490-5894785	NM_001014809, NM	.	NP_001014809, NP_00	.	protein_coding	ENSG00000072832	1400	2365

4427	MNX1	-0.608	0.049	motor neuron and pancreas homeobox 1 [Source:HGNC Symbol;Acc:HGNC:4979]	chr7:156786744-156803345	NM_001165255, NN	.	NP_001158727, NP_00	.	protein_coding	ENSG0000013067	3110	4979
4428	ZFAND2B	-0.550	0.049	zinc finger AN1-type containing 2B [Source:HGNC Symbol;Acc:HGNC:25206]	chr2:220071505-220074373	NM_001270998, NN	XM_017003368, XM_0	NP_001257927, NP_00	XP_006712348, XP_0067	protein_coding	ENSG0000015855	130617	25206
4429	CCDC90B	0.719	0.049	coiled-coil domain containing 90B [Source:HGNC Symbol;Acc:HGNC:28108]	chr11:82970138-82997450	NM_001286116, NN	XM_005274159, XM_0	NP_001273045, NP_00	XP_005274211, XP_0052	protein_coding	ENSG0000013750	60492	28108
4430	PTK2	0.867	0.049	protein tyrosine kinase 2 [Source:HGNC Symbol;Acc:HGNC:9611]	chr8:141667998-142011412	NM_001199649, NN	XM_017013686, XM_0	NP_001186578, NP_00	XP_005251060, XP_0052	protein_coding	ENSG0000016939	5747	9611
4431	CYSRT1	-0.734	0.049	cysteine rich tail 1 [Source:HGNC Symbol;Acc:HGNC:30529]	chr9:140119086-140120763	NM_199001	.	NP_945352	.	protein_coding	ENSG0000019719	375791	30529
4432	HS6ST1	-0.547	0.049	heparan sulfate 6-O-sulfotransferase 1 [Source:HGNC Symbol;Acc:HGNC:5201]	chr2:129023053-129076151	NM_004807	.	NP_004798	.	protein_coding	ENSG0000013672	9394	5201
4433	MEX3C	0.583	0.049	mex-3 RNA binding family member C [Source:HGNC Symbol;Acc:HGNC:28040]	chr18:48700921-48723690	NM_016626	.	NP_057710	.	protein_coding	ENSG0000017662	51320	28040
4434	WDR3	0.940	0.049	WD repeat domain 3 [Source:HGNC Symbol;Acc:HGNC:12755]	chr1:118472342-118727846	NM_006784	.	NP_006775	.	protein_coding	ENSG0000006518	10885	12755
4435	GGA1	-0.520	0.049	golgi associated, gamma adaptin ear containing, ARF binding protein 1 [Source:HGNC Sy	chr22:38004481-38029571	NM_001001560, NN	XM_017028760, XM_0	NP_001001560, NP_00	XP_005261574, XP_0052	protein_coding	ENSG0000010008	26088	17842
4436	MTX2	0.523	0.049	metaxin 2 [Source:HGNC Symbol;Acc:HGNC:7506]	chr2:177134146-177202753	NM_001006635, NN	XM_011510498, XM_0	NP_001006636, NP_00	XP_011508799	protein_coding	ENSG0000012865	10651	7506
4437	PRF1	-0.461	0.049	perforin 1 [Source:HGNC Symbol;Acc:HGNC:9360]	chr10:72357103-72362531	NM_001083116, NN	.	NP_001076585, NP_00	.	protein_coding	ENSG0000018064	5551	9360

Supplementary Table 7. List of dysadherin-binding proteins identified by co-IP and subsequent LC-MS analysis.

				Probe identification probability (Spectral counts)	
	Identified proteins	Accession number	Molecular weight	IP M53	IP IgG
1	Microfibrillar protein 2 (Fragment) OS=Homo sapiens PE=2	tr|Q9NP29|Q9NP29_HUMAN	14 kDa	100% (190)	100% (16)
2	Actin, cytoplasmic 2 OS=Homo sapiens GN=ACTG1 PE=1	sp|P63261|ACTG_HUMAN	42 kDa	100% (98)	100% (46)
3	Serum albumin OS=Homo sapiens GN=ALB PE=1 SV=2	sp|P02768|ALBU_HUMAN (+1)	69 kDa	100% (50)	100% (14)
4	Isoform 2 of General transcription factor II-I OS=Homo	sp|P78347-2|GTF2I_HUMAN (+4)	108 kDa	100% (29)	100% (35)
5	Uncharacterized protein DKFZp686C15213 OS=Homo	tr|Q6MZU6|Q6MZU6_HUMAN	51 kDa	100% (35)	100% (9)
6	Tubulin alpha-1B chain OS=Homo sapiens GN=TUBA1B	sp|P68363|TBA1B_HUMAN	50 kDa	100% (44)	100% (15)
7	Tubulin beta chain OS=Homo sapiens GN=TUBB PE=1	sp|P07437|TBB5_HUMAN	50 kDa	100% (49)	100% (16)
8	Isoform 2 of Clathrin heavy chain 1 OS=Homo sapiens	sp|Q00610-2|CLH1_HUMAN (+2)	188 kDa	100% (61)	
9	Elongation factor 1-alpha 1 OS=Homo sapiens GN=EEF1A1	sp|P68104|EF1A1_HUMAN (+2)	50 kDa	100% (44)	100% (13)
10	Desmoplakin OS=Homo sapiens GN=DSP PE=1 SV=3	sp|P15924|DESP_HUMAN	332 kDa	100% (36)	91% (1)
11	Cytochrome c OS=Homo sapiens GN=CYCS PE=1 SV=2	sp|P99999|CYC_HUMAN (+2)	12 kDa	100% (1)	100% (36)
12	Heat shock cognate 71 kDa protein OS=Homo sapiens	sp|P11142|HSP7C_HUMAN	71 kDa	100% (24)	100% (16)
13	Myosin-9 OS=Homo sapiens GN=MYH9 PE=1 SV=4	sp|P35579|MYH9_HUMAN	227 kDa	100% (35)	13% (0)
14	DNA-dependent protein kinase catalytic subunit OS=Homo	sp|P78527|PRKDC_HUMAN	469 kDa	100% (26)	72% (0)
15	Polyadenylate-binding protein 1 OS=Homo sapiens	sp|P11940|PABP1_HUMAN	71 kDa	100% (16)	100% (24)
16	60S ribosomal protein L13 OS=Homo sapiens GN=RPL13	sp|P26373|RL13_HUMAN (+2)	24 kDa	100% (17)	100% (14)
17	Heterogeneous nuclear ribonucleoprotein U OS=Homo	sp|Q00839|HNRPU_HUMAN	91 kDa	100% (17)	100% (14)
18	ATP-dependent RNA helicase A OS=Homo sapiens	sp|Q08211|DHX9_HUMAN	141 kDa	100% (22)	100% (6)
19	Nucleolin OS=Homo sapiens GN=NCL PE=1 SV=3	sp|P19338|NUCL_HUMAN (+1)	77 kDa	100% (15)	100% (14)
20	Immunoglobulin kappa variable 2-29 OS=Homo sapiens	sp|A2NJV5|KV229_HUMAN	13 kDa	100% (10)	100% (22)
21	Insulin receptor substrate 4 OS=Homo sapiens GN=IRS4	sp|O14654|IRS4_HUMAN	134 kDa	100% (16)	100% (2)
22	40S ribosomal protein S9 OS=Homo sapiens GN=RPS9	sp|P46781|RS9_HUMAN (+1)	23 kDa	100% (12)	100% (14)
23	IgG L chain OS=Homo sapiens PE=2 SV=1	tr|S6B2A1|S6B2A1_HUMAN	20 kDa	100% (11)	100% (4)
24	Splicing factor, proline- and glutamine-rich OS=Homo	sp|P23246|SFPQ_HUMAN (+1)	76 kDa	100% (16)	100% (12)
25	Cytoplasmic dynein 1 heavy chain 1 OS=Homo sapiens	sp|Q14204|DYHC1_HUMAN	532 kDa	100% (19)	10% (0)
26	cDNA FLJ51907, highly similar to Stress-70 protein,	tr|B7Z4V2|B7Z4V2_HUMAN	72 kDa	100% (13)	100% (16)
27	Heat shock protein HSP 90-alpha OS=Homo sapiens	sp|P07900|HS90A_HUMAN (+1)	85 kDa	100% (13)	100% (2)
28	Isoform 2 of Heterogeneous nuclear ribonucleoprotein K	sp|P61978-2|HNRPK_HUMAN (+5)	51 kDa	100% (6)	100% (15)
29	Isoform 2 of ATP-dependent RNA helicase DDX3X	sp|O00571-2|DDX3X_HUMAN (+4)	71 kDa	100% (9)	100% (12)
30	Complement C3 OS=Homo sapiens GN=C3 PE=1 SV=2	sp|P01024|CO3_HUMAN (+1)	187 kDa	100% (20)	6% (0)
31	Isoform 2 of Heterogeneous nuclear ribonucleoprotein M	sp|P52272-2|HNRPM_HUMAN (+2)	74 kDa	100% (5)	100% (5)
32	Isoform 10 of Fibronectin OS=Homo sapiens GN=FN1	sp|P02751-10|FINC_HUMAN (+7)	240 kDa	100% (20)	6% (0)
33	T-complex protein 1 subunit alpha OS=Homo sapiens	sp|P17987|TCPA_HUMAN	60 kDa	100% (16)	100% (4)
34	78 kDa glucose-regulated protein OS=Homo sapiens	sp|P11021|GRP78_HUMAN	72 kDa	100% (11)	100% (8)
35	Probable ATP-dependent RNA helicase DDX5 OS=Homo	sp|P17844|DDX5_HUMAN (+2)	69 kDa	100% (8)	100% (11)
36	40S ribosomal protein S4, X isoform OS=Homo sapiens	sp|P62701|RS4X_HUMAN (+3)	30 kDa	100% (9)	100% (4)
37	40S ribosomal protein S3 OS=Homo sapiens GN=RPS3	sp|P23396|RS3_HUMAN	27 kDa	100% (13)	100% (6)
38	Isoform 2 of ATP synthase subunit alpha, mitochondrial	sp|P25705-2|ATPA_HUMAN (+2)	54 kDa	100% (10)	100% (3)
39	40S ribosomal protein S18 OS=Homo sapiens GN=RPS18	sp|P62269|RS18_HUMAN	18 kDa	100% (9)	100% (7)
40	40S ribosomal protein S16 OS=Homo sapiens GN=RPS16	sp|P62249|RS16_HUMAN	16 kDa	100% (12)	100% (6)
41	40S ribosomal protein S2 OS=Homo sapiens GN=RPS2	sp|P15880|RS2_HUMAN	31 kDa	100% (11)	100% (8)
42	Unconventional myosin-Id OS=Homo sapiens GN=MYO1D	sp|O94832|MYO1D_HUMAN (+1)	116 kDa	100% (5)	100% (6)
43	Heat shock protein beta-1 OS=Homo sapiens GN=HSPB1	sp|P04792|HSPB1_HUMAN (+1)	23 kDa	100% (13)	100% (3)
44	DNA replication licensing factor MCM7 OS=Homo sapiens	sp|P33993|MCM7_HUMAN (+2)	81 kDa	100% (14)	96% (0)
45	Neutral amino acid transporter B(0) OS=Homo sapiens	sp|Q15758|AAAT_HUMAN (+1)	57 kDa	100% (19)	99% (1)
46	Isoform 2 of Nesprin-2 OS=Homo sapiens GN=SYNE2	sp|Q8WXH0-2|SYNE2_HUMAN (+1)	799 kDa	86% (0)	100% (5)
47	Isoform 2 of Unconventional myosin-Ic OS=Homo sapiens	sp|O00159-2|MYO1C_HUMAN (+4)	118 kDa	100% (11)	100% (4)
48	Leucine-rich PPR motif-containing protein, mitochondrial	sp|P42704|LPPRC_HUMAN (+1)	158 kDa	100% (8)	100% (5)
49	Heterogeneous nuclear ribonucleoproteins A2/B1	sp|P22626|ROA2_HUMAN	37 kDa	100% (8)	100% (7)
50	Protein transport protein sec16 OS=Homo sapiens	tr|J3KNL6|J3KNL6_HUMAN	252 kDa	100% (16)	
51	Histone H1.3 OS=Homo sapiens GN=HIST1H1D PE=1	sp|P16402|H13_HUMAN	22 kDa	100% (7)	100% (4)
52	ADP/ATP translocase 3 OS=Homo sapiens GN=SLC25A6	sp|P12236|ADT3_HUMAN (+2)	33 kDa	100% (10)	100% (2)
53	ATP synthase subunit beta, mitochondrial OS=Homo	sp|P06576|ATPB_HUMAN (+1)	57 kDa	100% (16)	100% (4)
54	Ras GTPase-activating protein-binding protein 1 OS=Homo	sp|Q13283|G3BP1_HUMAN (+2)	52 kDa	100% (7)	100% (8)
55	Isoform 3 of 60S ribosomal protein L17 OS=Homo sapiens	sp|P18621-3|RL17_HUMAN (+3)	26 kDa	100% (7)	100% (7)
56	60S ribosomal protein L10 OS=Homo sapiens GN=RPL10	sp|P27635|RL10_HUMAN (+1)	25 kDa	100% (3)	100% (8)
57	Isoform Gamma-A of Fibrinogen gamma chain OS=Homo	sp|P02679-2|FIBG_HUMAN (+6)	49 kDa	100% (14)	
58	Pyruvate kinase PKM OS=Homo sapiens GN=PKM PE=1	sp|P14618|KPYM_HUMAN (+1)	58 kDa	100% (10)	100% (3)
59	Filaggrin OS=Homo sapiens GN=FLG PE=1 SV=3	sp|P20930|FILA_HUMAN	435 kDa	100% (8)	
60	GCT-A4 light chain variable region (Fragment) OS=Homo	tr|A0A0X9T7V9|A0A0X9T7V9_HUMAN	12 kDa	100% (8)	100% (8)
61	40S ribosomal protein S8 OS=Homo sapiens GN=RPS8	sp|P62241|RS8_HUMAN (+2)	24 kDa	100% (5)	100% (9)
62	Isoform A1-A of Heterogeneous nuclear ribonucleoprotein	sp|P09651-2|ROA1_HUMAN (+2)	34 kDa	100% (10)	100% (8)
63	Isoform 2 of Poly(rC)-binding protein 2 OS=Homo sapiens	sp|Q15366-2|PCBP2_HUMAN (+1)	39 kDa	100% (5)	100% (7)
64	Isoform 2 of Bcl-2-associated transcription factor 1	sp|Q9NYF8-2|BCLF1_HUMAN (+3)	106 kDa	100% (7)	100% (6)
65	Isoform 2 of Signal recognition particle subunit SRP68	sp|Q9UHB9-2|SRP68_HUMAN (+1)	67 kDa	100% (8)	23% (0)
66	Isoform 2 of 60S ribosomal protein L18 OS=Homo sapiens	sp|Q07020-2|RL18_HUMAN (+5)	18 kDa	100% (6)	100% (7)
67	60S ribosomal protein L4 OS=Homo sapiens GN=RPL4	sp|P36578|RL4_HUMAN (+1)	48 kDa	100% (6)	100% (6)
68	Junction plakoglobin OS=Homo sapiens GN=JUP PE=1	sp|P14923|PLAK_HUMAN (+2)	82 kDa	100% (10)	98% (0)
69	Isoform F of Constitutive coactivator of PPAR-gamma-like	sp|Q9NZB2-6|F120A_HUMAN (+1)	125 kDa	100% (7)	100% (6)
70	Pre-mRNA-processing factor 6 OS=Homo sapiens	sp|O94906|PRP6_HUMAN	107 kDa	100% (9)	
71	Immunoglobulin heavy variable 3-64D OS=Homo sapiens	sp|A0A0J9YX35|HV64D_HUMAN	13 kDa	100% (5)	100% (8)
72	Tubulin beta-4B chain OS=Homo sapiens GN=TUBB4B	sp|P68371|TBB4B_HUMAN	50 kDa	100% (9)	100% (4)
73	60S ribosomal protein L28 OS=Homo sapiens GN=RPL28	sp|P46779|RL28_HUMAN	16 kDa	100% (2)	100% (6)
74	Uncharacterized protein OS=Homo sapiens PE=2 SV=1	tr|A8K008|A8K008_HUMAN	52 kDa	99% (1)	100% (13)
75	Isoform 2 of Heterogeneous nuclear ribonucleoprotein Q	sp|O60506-2|HNRPQ_HUMAN (+3)	66 kDa	100% (4)	100% (6)
76	cDNA FLJ54333, highly similar to T-complex protein 1	tr|B4DXI1|B4DXI1_HUMAN	57 kDa	100% (7)	40% (0)
77	40S ribosomal protein S13 OS=Homo sapiens GN=RPS13	sp|P62277|RS13_HUMAN	17 kDa	100% (8)	100% (4)
78	60S ribosomal protein L7 OS=Homo sapiens GN=RPL7	sp|P18124|RL7_HUMAN (+2)	29 kDa	100% (6)	100% (5)
79	Protein-glutamine gamma-glutamyltransferase 2	sp|P21980|TGM2_HUMAN (+2)	77 kDa	100% (12)	

80	Non-POU domain-containing octamer-binding protein	sp|Q15233|NONO_HUMAN (+2)	54 kDa	100% (5)	100% (4)
81	C-1-tetrahydrofolate synthase, cytoplasmic OS=Homo	sp|P11586|C1TC_HUMAN (+3)	102 kDa	100% (11)	99% (1)
82	DNA replication licensing factor MCM6 OS=Homo sapiens	sp|Q14566|MCM6_HUMAN	93 kDa	100% (11)	99% (1)
83	Isoform 2 of DNA topoisomerase 2-alpha OS=Homo	sp|P11388-2|TOP2A_HUMAN (+3)	178 kDa	100% (9)	
84	Isoform 2 of Plectin OS=Homo sapiens GN=PLEC	sp|Q15149-2|PLEC_HUMAN (+8)	518 kDa	100% (4)	15% (0)
85	Fibrinogen beta chain OS=Homo sapiens GN=FGB PE=1	sp|P02675|FIBB_HUMAN (+4)	56 kDa	100% (8)	
86	Apolipoprotein B-100 OS=Homo sapiens GN=APOB PE=1	sp|P04114|APOB_HUMAN (+1)	516 kDa	100% (4)	100% (2)
87	40S ribosomal protein S3a OS=Homo sapiens GN=RPS3A	sp|P61247|RS3A_HUMAN (+1)	30 kDa	100% (4)	100% (7)
88	Isoform 4 of E3 ubiquitin-protein ligase UBR4 OS=Homo	sp|Q5T4S7-4|UBR4_HUMAN	574 kDa	100% (7)	9% (0)
89	T-complex protein 1 subunit gamma OS=Homo sapiens	sp|P49368|TCPG_HUMAN (+3)	61 kDa	100% (6)	100% (1)
90	Isoform 3 of 26S proteasome non-ATPase regulatory	sp|Q13200-3|PSMD2_HUMAN (+2)	86 kDa	100% (10)	100% (1)
91	Ras GTPase-activating-like protein IQGAP1 OS=Homo	sp|P46940|IQGA1_HUMAN (+2)	189 kDa	100% (8)	
92	Isoform 2 of Caprin-1 OS=Homo sapiens GN=CAPRIN1	sp|Q14444-2|CAPR1_HUMAN (+1)	77 kDa	100% (3)	100% (9)
93	60S ribosomal protein L13a (Fragment) OS=Homo sapiens	tr|M0QYS1|M0QYS1_HUMAN	24 kDa	100% (5)	100% (2)
94	Poly [ADP-ribose] polymerase 1 OS=Homo sapiens	sp|P09874|PARP1_HUMAN (+2)	113 kDa	100% (6)	100% (4)
95	Isoform Smooth muscle of Myosin light polypeptide 6	sp|P60660-2|MYL6_HUMAN (+4)	17 kDa	100% (6)	100% (4)
96	Thyroid hormone receptor-associated protein 3 OS=Homo	sp|Q9Y2W1|TR150_HUMAN	109 kDa	100% (6)	100% (5)
97	Polypyrimidine tract-binding protein 1 OS=Homo sapiens	tr|A0A0U1RRM4|A0A0U1RRM4_HUMAN	62 kDa	100% (3)	100% (4)
98	60S ribosomal protein L31 OS=Homo sapiens GN=RPL31	sp|P62899|RL31_HUMAN (+1)	14 kDa	100% (2)	100% (3)
99	CAD protein OS=Homo sapiens GN=CAD PE=1 SV=3	sp|P27708|PYR1_HUMAN (+1)	243 kDa	100% (10)	
100	Isoform 3 of Sodium/potassium-transporting ATPase	sp|P05023-3|AT1A1_HUMAN (+3)	110 kDa	100% (9)	100% (1)
101	Solute carrier family 2, facilitated glucose transporter	sp|P11166|GTR1_HUMAN (+3)	54 kDa	100% (5)	
102	Midasin OS=Homo sapiens GN=MDN1 PE=1 SV=2	sp|Q9NU22|MDN1_HUMAN	633 kDa	49% (0)	100% (4)
103	Nuclease-sensitive element-binding protein 1 OS=Homo	sp|P67809|YBOX1_HUMAN (+1)	36 kDa	100% (4)	100% (7)
104	Trifunctional purine biosynthetic protein adenosine-3	sp|P22102|PUR2_HUMAN (+1)	108 kDa	100% (6)	26% (0)
105	Isoform 2 of Complement C4-A OS=Homo sapiens	sp|P0C0L4-2|CO4A_HUMAN (+3)	188 kDa	100% (6)	
106	Filaggrin-2 OS=Homo sapiens GN=FLG2 PE=1 SV=1	sp|Q5D862|FILA2_HUMAN	248 kDa	100% (3)	100% (2)
107	60S ribosomal protein L7a OS=Homo sapiens GN=RPL7A	sp|P62424|RL7A_HUMAN	30 kDa	100% (3)	100% (5)
108	60S ribosomal protein L10a OS=Homo sapiens	sp|P62906|RL10A_HUMAN	25 kDa	100% (2)	100% (6)
109	40S ribosomal protein S19 OS=Homo sapiens GN=RPS19	sp|P39019|RS19_HUMAN	16 kDa	100% (2)	100% (4)
110	40S ribosomal protein S11 OS=Homo sapiens GN=RPS11	sp|P62280|RS11_HUMAN (+1)	18 kDa	100% (2)	100% (4)
111	Elongation factor Tu, mitochondrial OS=Homo sapiens	sp|P49411|EFTU_HUMAN	50 kDa	100% (4)	100% (1)
112	Receptor of activated protein C kinase 1 OS=Homo	sp|P63244|RACK1_HUMAN (+1)	35 kDa	100% (6)	100% (4)
113	40S ribosomal protein S7 OS=Homo sapiens GN=RPS7	sp|P62081|RS7_HUMAN	22 kDa	100% (3)	100% (3)
114	Isoform 3 of Exportin-2 OS=Homo sapiens GN=CSE1L	sp|P55060-3|XPO2_HUMAN (+1)	108 kDa	100% (4)	
115	Exportin-1 OS=Homo sapiens GN=XPO1 PE=1 SV=1	sp|O14980|XPO1_HUMAN	123 kDa	100% (8)	
116	60S ribosomal protein L8 OS=Homo sapiens GN=RPL8	sp|P62917|RL8_HUMAN (+1)	28 kDa	100% (5)	100% (4)
117	60S ribosomal protein L14 OS=Homo sapiens GN=RPL14	sp|P50914|RL14_HUMAN (+5)	23 kDa	100% (5)	100% (4)
118	Histone H1.5 OS=Homo sapiens GN=HIST1H1B PE=1	sp|P16401|H15_HUMAN	23 kDa	100% (3)	100% (4)
119	Isoform 2 of Heterogeneous nuclear ribonucleoprotein A/B	sp|Q99729-2|ROAA_HUMAN (+2)	36 kDa	99% (3)	100% (4)
120	Actin, alpha cardiac muscle 1 OS=Homo sapiens	sp|P68032|ACTC_HUMAN	42 kDa	100% (5)	99% (2)
121	60S ribosomal protein L6 OS=Homo sapiens PE=2 SV=1	tr|Q9HBB3|Q9HBB3_HUMAN	33 kDa	100% (3)	100% (4)
122	Hornerin OS=Homo sapiens GN=HRNR PE=1 SV=2	sp|Q86YZ3|HORN_HUMAN	282 kDa	100% (6)	
123	Isoform 3 of L-lactate dehydrogenase A chain OS=Homo	sp|P00338-3|LDHA_HUMAN (+3)	40 kDa	100% (4)	100% (2)
124	Isoform 2 of Polyadenylate-binding protein 4 OS=Homo	sp|Q13310-2|PABP4_HUMAN (+1)	70 kDa	100% (3)	100% (4)
125	40S ribosomal protein S14 OS=Homo sapiens GN=RPS14	sp|P62263|RS14_HUMAN	16 kDa	100% (3)	100% (4)
126	Myosin regulatory light chain 12B OS=Homo sapiens	sp|O14950|ML12B_HUMAN (+3)	20 kDa	100% (5)	100% (5)
127	60S ribosomal protein L12 OS=Homo sapiens GN=RPL12	sp|P30050|RL12_HUMAN (+1)	18 kDa	100% (3)	100% (4)
128	60S ribosomal protein L23 OS=Homo sapiens GN=RPL23	sp|P62829|RL23_HUMAN (+2)	15 kDa	100% (3)	100% (3)
129	ATPase family AAA domain-containing protein 3A	tr|H0Y2W2|H0Y2W2_HUMAN	64 kDa	100% (3)	100% (1)
130	Ras GTPase-activating protein-binding protein 2 OS=Homo	sp|Q9UN86|G3BP2_HUMAN	54 kDa	100% (3)	100% (2)
131	DNA replication licensing factor MCM4 OS=Homo sapiens	sp|P33991|MCM4_HUMAN (+2)	97 kDa	100% (5)	
132	Proliferating cell nuclear antigen OS=Homo sapiens	sp|P12004|PCNA_HUMAN	29 kDa	100% (4)	99% (1)
133	DNA topoisomerase 2-beta OS=Homo sapiens GN=TOP2B	sp|Q02880|TOP2B_HUMAN	183 kDa	100% (3)	
134	40S ribosomal protein S15a OS=Homo sapiens	sp|P62244|RS15A_HUMAN (+1)	15 kDa	100% (6)	75% (0)
135	Isoleucine--tRNA ligase, cytoplasmic OS=Homo sapiens	sp|P41252|SYIC_HUMAN (+1)	145 kDa	100% (5)	
136	Isoform 3 of Heterogeneous nuclear ribonucleoprotein D0	sp|Q14103-3|HNRPD_HUMAN (+1)	33 kDa	98% (1)	100% (5)
137	Elongation factor 2 OS=Homo sapiens GN=EEF2 PE=1	sp|P13639|EF2_HUMAN	95 kDa	100% (3)	99% (1)
138	Isoform 2 of Solute carrier family 2, facilitated glucose	sp|Q8TDB8-2|GTR14_HUMAN (+3)	54 kDa	100% (6)	
139	RuvB-like 1 OS=Homo sapiens GN=RUVBL1 PE=1 SV=1	sp|Q9Y265|RUVB1_HUMAN (+1)	50 kDa	100% (4)	100% (1)
140	Importin-7 OS=Homo sapiens GN=IPO7 PE=1 SV=1	sp|O95373|IPO7_HUMAN	120 kDa	100% (4)	100% (1)
141	Heterogeneous nuclear ribonucleoprotein H OS=Homo	sp|P31943|HNRH1_HUMAN (+2)	49 kDa	100% (3)	100% (4)
142	Isoform 2 of Regulator of nonsense transcripts 1	sp|Q92900-2|RENT1_HUMAN (+3)	123 kDa	100% (3)	100% (2)
143	Putative Ras-related protein Rab-1C OS=Homo sapiens	sp|Q92928|RAB1C_HUMAN	22 kDa	100% (3)	95% (1)
144	Histone H4 OS=Homo sapiens GN=HIST1H4A PE=1 SV=2	sp|P62805|H4_HUMAN (+2)	11 kDa	100% (5)	100% (3)
145	40S ribosomal protein S5 OS=Homo sapiens GN=RPS5	tr|M0R0R2|M0R0R2_HUMAN	25 kDa	100% (4)	100% (4)
146	60S ribosomal protein L24 OS=Homo sapiens GN=RPL24	sp|P83731|RL24_HUMAN (+3)	18 kDa	100% (3)	100% (4)
147	Histone H2B type 1-K OS=Homo sapiens GN=HIST1H2BK	sp|O60814|H2B1K_HUMAN (+7)	14 kDa	100% (4)	100% (3)
148	cDNA, FLJ92164, highly similar to Homo sapiens	tr|B2R4P2|B2R4P2_HUMAN	22 kDa	100% (4)	100% (1)
149	Galectin-7 OS=Homo sapiens GN=LGALS7 PE=1 SV=2	sp|P47929|LEG7_HUMAN	15 kDa	100% (5)	5% (0)
150	60S ribosomal protein L27a OS=Homo sapiens	sp|P46776|RL27A_HUMAN (+4)	17 kDa	100% (4)	100% (3)
151	Isoform 2 of Interleukin enhancer-binding factor 3	sp|Q12906-2|ILF3_HUMAN (+9)	76 kDa	100% (2)	100% (3)
152	Isoform 2 of 60S acidic ribosomal protein P0 OS=Homo	sp|P05388-2|RLA0_HUMAN (+3)	27 kDa	100% (4)	99% (1)
153	Tubulin beta-2B chain OS=Homo sapiens GN=TUBB2B	sp|Q9BVA1|TBB2B_HUMAN	50 kDa	100% (4)	100% (1)
154	Tubulin alpha-4A chain OS=Homo sapiens GN=TUBA4A	sp|P68366|TBA4A_HUMAN	50 kDa	100% (3)	96% (0)
155	Isoform 1b of Oxysterol-binding protein-related protein 3	sp|Q9H4L5-2|OSBL3_HUMAN (+3)	98 kDa	100% (1)	100% (5)
156	Peptidyl-prolyl cis-trans isomerase OS=Homo sapiens	tr|Q71V99|Q71V99_HUMAN	18 kDa	100% (5)	99% (1)
157	40S ribosomal protein S26 OS=Homo sapiens GN=RPS26	sp|P62854|RS26_HUMAN (+1)	13 kDa	100% (2)	100% (3)
158	Poly(rC)-binding protein 1 OS=Homo sapiens GN=PCBP1	sp|Q15365|PCBP1_HUMAN	37 kDa	100% (3)	100% (3)
159	Heterogeneous nuclear ribonucleoprotein U-like protein 2	sp|Q1KMD3|HNRL2_HUMAN (+1)	85 kDa	100% (3)	100% (2)
160	40S ribosomal protein S6 OS=Homo sapiens GN=RPS6	sp|P62753|RS6_HUMAN (+2)	29 kDa	100% (3)	100% (4)
161	Isoform 2 of 40S ribosomal protein S20 OS=Homo sapiens	sp|P60866-2|RS20_HUMAN (+1)	16 kDa	100% (5)	100% (3)
162	Isoform 3 of ATP-dependent RNA helicase DDX1	sp|Q92499-3|DDX1_HUMAN (+4)	69 kDa	100% (2)	100% (2)
163	60S ribosomal protein L18a OS=Homo sapiens	sp|Q02543|RL18A_HUMAN (+6)	21 kDa	100% (2)	100% (3)

164	Isoform 2 of Arginase-1 OS=Homo sapiens GN=ARG1	sp|P05089-2|ARGI1_HUMAN (+1)	36 kDa	100% (5)	
165	1-phosphatidylinositol 4,5-bisphosphate phosphodiesterase	sp|Q8N3E9|PLCD3_HUMAN	89 kDa	57% (0)	100% (2)
166	Isoform 2 of Coatomer subunit alpha OS=Homo sapiens	sp|P53621-2|COPA_HUMAN (+1)	139 kDa	100% (6)	
167	Cullin-associated NEDD8-dissociated protein 1 OS=Homo	sp|Q86VP6|CAND1_HUMAN (+1)	136 kDa	100% (3)	
168	RNA-binding protein 39 (Fragment) OS=Homo sapiens	tr|H0Y4X3|H0Y4X3_HUMAN	37 kDa	100% (3)	100% (1)
169	60S ribosomal protein L11 OS=Homo sapiens GN=RPL11	sp|P62913|RL11_HUMAN (+1)	20 kDa	100% (4)	99% (1)
170	Bifunctional glutamate/proline--tRNA ligase OS=Homo	sp|P07814|SYEP_HUMAN	171 kDa	100% (3)	14% (0)
171	Isoform 2 of Formin-like protein 3 OS=Homo sapiens	sp|Q8IVF7-2|FMNL3_HUMAN (+3)	111 kDa	100% (2)	99% (1)
172	Fus-like protein (Fragment) OS=Homo sapiens PE=2 SV=1	tr|Q13344|Q13344_HUMAN	53 kDa	100% (2)	100% (3)
173	60S ribosomal protein L30 OS=Homo sapiens GN=RPL30	sp|P62888|RL30_HUMAN (+2)	13 kDa	100% (2)	100% (4)
174	14-3-3 protein zeta/delta OS=Homo sapiens GN=YWHAZ	sp|P63104|1433Z_HUMAN (+2)	28 kDa	100% (7)	
175	60S ribosomal protein L23a OS=Homo sapiens	sp|P62750|RL23A_HUMAN	18 kDa	100% (2)	100% (3)
176	40S ribosomal protein S23 OS=Homo sapiens GN=RPS23	sp|P62266|RS23_HUMAN (+1)	16 kDa	100% (2)	100% (3)
177	40S ribosomal protein S25 OS=Homo sapiens GN=RPS25	sp|P62851|RS25_HUMAN	14 kDa	100% (2)	100% (3)
178	60S ribosomal protein L3 OS=Homo sapiens GN=RPL3	sp|P39023|RL3_HUMAN (+1)	46 kDa	100% (2)	100% (2)
179	Isoform 2 of Annexin A2 OS=Homo sapiens GN=ANXA2	sp|P07355-2|ANXA2_HUMAN (+5)	40 kDa	100% (4)	100% (2)
180	Delta 2-isopentenyl pyrophosphate transferase-like protein	tr|Q8TCX0|Q8TCX0_HUMAN	5 kDa	100% (2)	100% (5)
181	60S ribosomal protein L21 OS=Homo sapiens GN=RPL21	sp|P46778|RL21_HUMAN (+2)	19 kDa	100% (3)	100% (3)
182	RNA transcription, translation and transport factor protein	sp|Q9Y224|RTRAF_HUMAN (+2)	28 kDa	100% (1)	100% (2)
183	Isoform 2 of 5'-3' exoribonuclease 2 OS=Homo sapiens	sp|Q9H0D6-2|XRN2_HUMAN (+3)	100 kDa	100% (4)	100% (1)
184	Isoform Beta of Tripartite motif-containing protein 29	sp|Q14134-2|TRI29_HUMAN (+3)	64 kDa	99% (1)	100% (2)
185	Uncharacterized protein OS=Homo sapiens PE=2 SV=1	tr|A0A5E4|A0A5E4_HUMAN	25 kDa	100% (4)	
186	Electron transfer flavoprotein subunit alpha, mitochondrial	sp|P13804|ETFA_HUMAN (+3)	35 kDa	100% (4)	20% (0)
187	Isoform 4 of TRIO and F-actin-binding protein OS=Homo	sp|Q9H2D6-3|TARA_HUMAN (+1)	243 kDa	7% (0)	100% (2)
188	Pre-mRNA-processing-splicing factor 8 OS=Homo sapiens	sp|Q6P2Q9|PRP8_HUMAN	274 kDa	100% (2)	
189	Alpha-actinin-4 OS=Homo sapiens GN=ACTN4 PE=1 SV=2	sp|O43707|ACTN4_HUMAN (+4)	105 kDa	100% (2)	100% (3)
190	60S ribosomal protein L35 OS=Homo sapiens GN=RPL35	sp|P42766|RL35_HUMAN	15 kDa	100% (3)	100% (1)
191	Isoform 2 of Glyceraldehyde-3-phosphate dehydrogenase	sp|P04406-2|G3P_HUMAN (+2)	32 kDa	100% (4)	99% (1)
192	Dolichyl-diphosphooligosaccharide--protein	sp|P04843|RPN1_HUMAN (+3)	69 kDa	100% (4)	85% (1)
193	Glycine--tRNA ligase OS=Homo sapiens GN=GARS PE=1	sp|P41250|GARS_HUMAN	83 kDa	100% (2)	95% (1)
194	THO complex subunit 4 OS=Homo sapiens GN=ALYREF	sp|Q86V81|THOC4_HUMAN (+1)	27 kDa	100% (2)	100% (2)
195	Isoform 2 of La-related protein 1 OS=Homo sapiens	sp|Q6PKG0-3|LARP1_HUMAN (+1)	116 kDa	100% (2)	99% (1)
196	Isoform 4 of La-related protein 4 OS=Homo sapiens	sp|Q71RC2-4|LARP4_HUMAN (+2)	81 kDa	100% (2)	100% (4)
197	Isoform 2 of Asparagine synthetase [glutamine-	sp|P08243-2|ASNS_HUMAN (+1)	62 kDa	100% (4)	
198	Isoform 2 of Heterogeneous nuclear ribonucleoprotein H3	sp|P31942-2|HNRH3_HUMAN (+3)	35 kDa	100% (2)	100% (1)
199	Isoform 1B of Desmocollin-1 OS=Homo sapiens GN=DSC1	sp|Q08554-2|DSC1_HUMAN (+2)	94 kDa	100% (4)	
200	Isoform 2 of Adenine phosphoribosyltransferase OS=Homo	sp|P07741-2|APT_HUMAN (+2)	15 kDa	100% (5)	99% (1)
201	14-3-3 protein theta OS=Homo sapiens GN=YWHAQ PE=1	sp|P27348|1433T_HUMAN	28 kDa	100% (2)	
202	Isoform 1 of Protein POF1B OS=Homo sapiens GN=POF1B	sp|Q8WVV4-1|POF1B_HUMAN (+1)	69 kDa	100% (3)	16% (0)
203	Isoform 2 of Nucleoside diphosphate kinase A OS=Homo	sp|P15531-2|NDKA_HUMAN (+1)	20 kDa	100% (5)	
204	60S ribosomal protein L19 OS=Homo sapiens GN=RPL19	sp|P84098|RL19_HUMAN (+4)	23 kDa	83% (1)	100% (2)
205	Gelsolin OS=Homo sapiens GN=GSN PE=1 SV=1	tr|A0A0A0MS51|A0A0A0MS51_HUMAN	83 kDa	99% (1)	100% (2)
206	Isoform 2 of 40S ribosomal protein S24 OS=Homo sapiens	sp|P62847-2|RS24_HUMAN (+5)	15 kDa	100% (2)	100% (3)
207	ADP-ribosylation factor 3 OS=Homo sapiens GN=ARF3	sp|P61204|ARF3_HUMAN (+4)	21 kDa	100% (2)	100% (3)
208	Eukaryotic initiation factor 4A-I OS=Homo sapiens	sp|P60842|IF4A1_HUMAN (+1)	46 kDa	100% (3)	
209	Isoform 2 of Heterogeneous nuclear ribonucleoprotein L	sp|P14866-2|HNRPL_HUMAN (+3)	51 kDa	100% (3)	99% (1)
210	Isoform 2 of Insulin-like growth factor 2 mRNA-binding	sp|Q9Y6M1-1|IF2B2_HUMAN (+6)	62 kDa	99% (1)	100% (2)
211	Isoform B of Phosphate carrier protein, mitochondrial	sp|Q00325-2|MPCP_HUMAN (+4)	40 kDa	100% (2)	83% (1)
212	GTP-binding nuclear protein Ran OS=Homo sapiens	sp|P62826|RAN_HUMAN (+3)	24 kDa	100% (4)	
213	Mucin-4 (Fragment) OS=Homo sapiens GN=MUC4 PE=1	tr|A0A0G2JMX1|A0A0G2JMX1_HUMAN	533 kDa	68% (0)	100% (2)
214	Ceruloplasmin OS=Homo sapiens GN=CP PE=1 SV=1	sp|P00450|CERU_HUMAN (+5)	122 kDa	100% (2)	
215	D-3-phosphoglycerate dehydrogenase OS=Homo sapiens	sp|O43175|SERA_HUMAN (+4)	57 kDa	100% (2)	
216	Isoform 2 of Heterogeneous nuclear ribonucleoprotein A3	sp|P51991-2|ROA3_HUMAN (+2)	37 kDa		100% (4)
217	Puromycin-sensitive aminopeptidase OS=Homo sapiens	sp|P55786|PSA_HUMAN (+1)	103 kDa	100% (2)	
218	Isoform 2 of Pyruvate dehydrogenase E1 component	sp|P11177-2|ODPB_HUMAN (+1)	37 kDa	100% (3)	13% (0)
219	U5 small nuclear ribonucleoprotein 200 kDa helicase	sp|O75643|U520_HUMAN	245 kDa	100% (2)	
220	Isoform C1 of Heterogeneous nuclear ribonucleoproteins	sp|P07910-2|HNRPC_HUMAN (+2)	32 kDa	100% (3)	13% (0)
221	TNC protein OS=Homo sapiens GN=TNC PE=2 SV=1	tr|A7MBN2|A7MBN2_HUMAN	210 kDa	100% (2)	
222	Protein LYRIC OS=Homo sapiens GN=MTDH PE=1 SV=2	sp|Q86UE4|LYRIC_HUMAN (+2)	64 kDa	10% (0)	100% (2)
223	Ras-related protein Rab-11A (Fragment) OS=Homo sapiens	tr|H3BMH2|H3BMH2_HUMAN	18 kDa	100% (2)	7% (0)
224	Calmodulin-like protein 3 OS=Homo sapiens GN=CALML3	sp|P27482|CALL3_HUMAN	17 kDa	100% (4)	
225	Nicotinamide phosphoribosyltransferase OS=Homo	sp|P43490|NAMPT_HUMAN (+1)	56 kDa	100% (4)	
226	Coatomer subunit gamma-1 OS=Homo sapiens	sp|Q9Y678|COPG1_HUMAN (+2)	98 kDa	100% (4)	
227	Aspartate--tRNA ligase, cytoplasmic OS=Homo sapiens	sp|P14868|SYDC_HUMAN (+1)	57 kDa	100% (3)	
228	60S acidic ribosomal protein P2 OS=Homo sapiens	sp|P05387|RLA2_HUMAN (+1)	12 kDa	100% (2)	100% (2)
229	Desmoglein-1 OS=Homo sapiens GN=DSG1 PE=1 SV=2	sp|Q02413|DSG1_HUMAN	114 kDa	100% (3)	
230	Isoform 2 of Triosephosphate isomerase OS=Homo	sp|P60174-1|TPIS_HUMAN (+3)	27 kDa	100% (2)	100% (1)
231	Transmembrane emp24 domain-containing protein 10	sp|P49755|TMEDA_HUMAN (+2)	25 kDa	100% (2)	99% (1)
232	40S ribosomal protein S15 OS=Homo sapiens GN=RPS15	sp|P62841|RS15_HUMAN (+7)	17 kDa	100% (2)	99% (2)
233	60S ribosomal protein L37a OS=Homo sapiens	sp|P61513|RL37A_HUMAN (+1)	10 kDa	100% (2)	97% (1)
234	28S ribosomal protein S23, mitochondrial OS=Homo	sp|Q9Y3D9|RT23_HUMAN (+1)	22 kDa	75% (1)	100% (3)
235	Isoform 2 of Calcium-binding mitochondrial carrier protein	sp|Q9UJS0-2|CMC2_HUMAN (+3)	74 kDa	100% (2)	
236	RNA-binding motif protein, X chromosome OS=Homo	sp|P38159|RBMX_HUMAN	42 kDa	21% (0)	100% (2)
237	Histone H2A type 1 OS=Homo sapiens GN=HIST1H2AG	sp|P0C0S8|H2A1_HUMAN (+6)	14 kDa	100% (2)	20% (0)
238	Isoform Beta of E3 ubiquitin-protein ligase TRIM23	sp|P36406-2|TRI23_HUMAN (+3)	64 kDa	17% (0)	97% (1)
239	Little elongation complex subunit 2 OS=Homo sapiens	sp|Q659A1|ICE2_HUMAN (+2)	110 kDa	14% (0)	100% (2)
240	Proteasome-associated protein ECM29 homolog	sp|Q5VYK3|ECM29_HUMAN (+2)	204 kDa	100% (2)	9% (0)
241	Isoform 2 of CTP synthase 1 OS=Homo sapiens GN=CTPS1	sp|P17812-2|PYRG1_HUMAN (+1)	41 kDa	100% (2)	6% (0)
242	Kinesin-like protein KIF13B OS=Homo sapiens GN=KIF13B	sp|Q9NQT8|KI13B_HUMAN	203 kDa	7% (0)	100% (2)
243	DNA-directed RNA polymerase II subunit RPB1 OS=Homo	sp|P24928|RPB1_HUMAN	217 kDa	100% (2)	
244	DNA polymerase delta catalytic subunit OS=Homo sapiens	sp|P28340|DPOD1_HUMAN (+3)	124 kDa	100% (2)	
245	RuvB-like 2 OS=Homo sapiens GN=RUVBL2 PE=1 SV=3	sp|Q9Y230|RUVB2_HUMAN	51 kDa	100% (3)	7% (0)
246	Trifunctional enzyme subunit beta, mitochondrial	sp|P55084|ECHB_HUMAN (+1)	51 kDa	100% (2)	6% (0)
247	Isoform 2 of Glutamine--fructose-6-phosphate	sp|Q06210-2|GFPT1_HUMAN (+1)	77 kDa	100% (2)	

248	Isoform 2 of Dolichyl-diphosphooligosaccharide--protein	sp|P04844-2|RPN2_HUMAN (+2)	68 kDa	100% (2)	
249	Isoform 2 of Replication factor C subunit 3 OS=Homo	sp|P40938-2|RFC3_HUMAN (+4)	35 kDa	100% (3)	
250	Coatomer subunit beta OS=Homo sapiens GN=COPB1	sp|P53618|COPB_HUMAN (+1)	107 kDa	100% (3)	
251	Isoform Monomeric of Arginine--tRNA ligase, cytoplasmic	sp|P54136-2|SYRC_HUMAN (+1)	67 kDa	100% (4)	
252	Methionine--tRNA ligase, cytoplasmic OS=Homo sapiens	sp|P56192|SYMC_HUMAN (+1)	101 kDa	100% (4)	
253	Calmodulin-like protein 5 OS=Homo sapiens GN=CALML5	sp|Q9NZT1|CALL5_HUMAN	16 kDa	100% (3)	
254	Isoform 2 of Y-box-binding protein 3 OS=Homo sapiens	sp|P16989-2|YBOX3_HUMAN (+4)	32 kDa		100% (2)
255	Isoform 2 of Protein FAM98B OS=Homo sapiens	sp|Q52LJ0-2|FA98B_HUMAN	46 kDa		100% (2)
256	Isoform 2 of Serine/threonine-protein phosphatase PP1-	sp|P62136-2|PP1A_HUMAN (+1)	39 kDa	100% (2)	
257	N90-VRC38.10 light chain variable region (Fragment)	tr|A0A1W6IYK3|A0A1W6IYK3_HUMAN	12 kDa		100% (3)
258	60S ribosomal protein L36 OS=Homo sapiens GN=RPL36	sp|Q9Y3U8|RL36_HUMAN	12 kDa	99% (1)	100% (2)
259	40S ribosomal protein S10 OS=Homo sapiens GN=RPS10	sp|P46783|RS10_HUMAN (+1)	19 kDa	99% (1)	100% (2)
260	60S ribosomal protein L29 OS=Homo sapiens GN=RPL29	sp|P47914|RL29_HUMAN (+2)	18 kDa	99% (1)	100% (2)
261	Isoform 1 of RNA-binding protein Raly OS=Homo sapiens	sp|Q9UKM9-2|RALY_HUMAN (+3)	30 kDa	9% (0)	99% (1)
262	Isoform 2 of Ras-related protein Rab-5C OS=Homo	sp|P51148-2|RAB5C_HUMAN (+1)	27 kDa	100% (2)	
263	Glutathione S-transferase P OS=Homo sapiens GN=GSTP1	sp|P09211|GSTP1_HUMAN (+1)	23 kDa	100% (3)	
264	Protein S100-A14 OS=Homo sapiens GN=S100A14 PE=1	sp|Q9HCY8|S10AE_HUMAN	12 kDa	100% (3)	
265	Isoform A of Isocitrate dehydrogenase [NAD] subunit beta,	sp|O43837-2|IDH3B_HUMAN (+4)	42 kDa	100% (3)	
266	Proteasome subunit alpha type-5 OS=Homo sapiens	sp|P28066|PSA5_HUMAN (+2)	26 kDa	100% (3)	
267	Isoform Beta-4A of Integrin beta-4 OS=Homo sapiens	sp|P16144-2|ITB4_HUMAN (+9)	195 kDa	100% (2)	
268	Isoform Heart of ATP synthase subunit gamma,	sp|P36542-2|ATPG_HUMAN (+4)	33 kDa	100% (2)	
269	Serine/threonine-protein phosphatase 2A catalytic subunit	sp|P67775|PP2AA_HUMAN (+2)	36 kDa	100% (2)	
270	Peroxiredoxin-6 OS=Homo sapiens GN=PRDX6 PE=1 SV=3	sp|P30041|PRDX6_HUMAN (+1)	25 kDa	100% (2)	
271	X-ray repair cross-complementing protein 5 OS=Homo	sp|P13010|XRCC5_HUMAN	83 kDa		100% (2)
272	Isoform Non-brain of Clathrin light chain A OS=Homo	sp|P09496-2|CLCA_HUMAN (+3)	24 kDa	100% (2)	
273	rRNA 2'-O-methyltransferase fibrillarin OS=Homo sapiens	sp|P22087|FBRL_HUMAN (+6)	34 kDa		100% (1)
274	Isoform 2 of Eukaryotic translation initiation factor 4E type	sp|O60573-2|IF4E2_HUMAN (+6)	27 kDa	100% (2)	
275	Cystatin-A OS=Homo sapiens GN=CSTA PE=1 SV=1	sp|P01040|CYTA_HUMAN (+1)	11 kDa	100% (2)	
276	L-lactate dehydrogenase B chain OS=Homo sapiens	sp|P07195|LDHB_HUMAN (+1)	37 kDa	100% (2)	
277	Isoform 2 of Replication factor C subunit 4 OS=Homo	sp|P35249-2|RFC4_HUMAN (+5)	34 kDa	100% (2)	
278	Very-long-chain 3-oxoacyl-CoA reductase OS=Homo	sp|Q53GQ0|DHB12_HUMAN (+4)	34 kDa	100% (2)	
279	Isoform PKP3b of Plakophilin-3 OS=Homo sapiens	sp|Q9Y446-2|PKP3_HUMAN (+1)	89 kDa	100% (2)	
280	Isoform 2 of Replication factor C subunit 2 OS=Homo	sp|P35250-2|RFC2_HUMAN (+3)	35 kDa	100% (2)	
281	Mitochondrial import receptor subunit TOM22 homolog	sp|Q9NS69|TOM22_HUMAN (+1)	16 kDa	100% (2)	

Supplementary Table 8. List of dysadherin-binding ECM proteins identified by co-IP and subsequent LC-MS analysis.

			Probe identification probability (Spectral counts)	
	Identified proteins	Accession	IP M53	IP IgG
1	Isoform 10 of Fibronectin OS=Homo sapiens GN=FN1	FN1	100% (20)	6% (0)
2	Cytoplasmic dynein 1 heavy chain 1 OS=Homo sapiens	DYNC1H1	100% (19)	10% (0)
3	Myosin-9 OS=Homo sapiens GN=MYH9 PE=1 SV=4	MYH9	100% (35)	13% (0)
4	Isoform 2 of Plectin OS=Homo sapiens GN=PLEC	PLEC	100% (4)	15% (0)
5	DNA-dependent protein kinase catalytic subunit	PRKDC	100% (26)	72% (0)
6	40S ribosomal protein S15a OS=Homo sapiens	RPS15A	100% (6)	75% (0)
7	Dolichyl-diphosphooligosaccharide--protein	RPN1	100% (4)	85% (1)
8	Desmoplakin OS=Homo sapiens GN=DSP PE=1 SV=3	DSP	100% (36)	91% (1)
9	Junction plakoglobin OS=Homo sapiens GN=JUP PE=1	JUP	100% (10)	98% (0)
10	Isoform 2 of Glyceraldehyde-3-phosphate	GAPDH	100% (4)	99% (1)
11	Heat shock protein HSP 90-alpha OS=Homo sapiens	HSP90AA1	100% (13)	100% (2)
12	ADP/ATP translocase 3 OS=Homo sapiens GN=SLC25A6	SLC25A6	100% (10)	100% (2)
13	Isoform 2 of Annexin A2 OS=Homo sapiens GN=ANXA2	ANXA2	100% (4)	100% (2)
14	Isoform 2 of ATP synthase subunit alpha, mitochondrial	ATP5A1	100% (10)	100% (3)
15	Heat shock protein beta-1 OS=Homo sapiens	HSPB1	100% (13)	100% (3)
16	Histone H4 OS=Homo sapiens GN=HIST1H4A PE=1	HIST1H4A	100% (5)	100% (3)
17	Isoform 2 of 40S ribosomal protein S20 OS=Homo	RPS20	100% (5)	100% (3)
18	ATP synthase subunit beta, mitochondrial OS=Homo	ATP5B	100% (16)	100% (4)
19	Tubulin beta-4B chain OS=Homo sapiens GN=TUBB4B	TUBB4B	100% (9)	100% (4)
20	40S ribosomal protein S3 OS=Homo sapiens GN=RPS3	RPS3	100% (13)	100% (6)
21	Tubulin beta chain OS=Homo sapiens GN=TUBB PE=1	TUBB	100% (49)	100% (16)
22	Heat shock cognate 71 kDa protein OS=Homo sapiens	HSPA8	100% (24)	100% (16)

REFERENCES

1. Percie du Sert N, Hurst V, Ahluwalia A, Alam S, Avey MT, Baker M, et al. The ARRIVE guidelines 2.0: Updated guidelines for reporting animal research. J Cereb Blood Flow Metab. 2020; 40: 1769-77.
2. Sauerbrei W, Taube SE, McShane LM, Cavenagh MM, Altman DG. Reporting recommendations for tumor marker prognostic studies (REMARK): an abridged explanation and elaboration. J Natl Cancer Inst. 2018; 110: 803-11.
3. Aoki S, Shimamura T, Shibata T, Nakanishi Y, Moriya Y, Sato Y, et al. Prognostic significance of dysadherin expression in advanced colorectal carcinoma. Br J Cancer. 2003; 88: 726-32.
4. Xue X, Shah YM. In vitro organoid culture of primary mouse colon tumors. J Vis Exp. 2013.
5. Park S-Y, Lee C-J, Choi J-H, Kim J-H, Kim J-W, Kim J-Y, et al. The JAK2/STAT3/CCND2 Axis promotes colorectal Cancer stem cell persistence and radioresistance. J Exp Clin Cancer Res. 2019; 38: 1-18.
6. Ino Y, Gotoh M, Sakamoto M, Tsukagoshi K, Hirohashi S. Dysadherin, a cancer-associated cell membrane glycoprotein, down-regulates E-cadherin and promotes metastasis. Proc Natl Acad Sci. 2002; 99: 365-70.
7. Lockhead S, Moskaleva A, Kamenz J, Chen Y, Kang M, Reddy AR, et al. The apparent requirement for protein synthesis during g2 phase is due to checkpoint activation. Cell Rep. 2020; 32: 107901.
8. Lee EY, Jeon MJ, Yang W, Shin I. Effect of p27 on motility of MDA-MB-231 breast cancer cells. Oncol Rep. 2009; 21: 1621-5.
9. Hu Y, Smyth GK. ELDA: extreme limiting dilution analysis for comparing depleted and enriched populations in stem cell and other assays. J Immunol Methods. 2009; 347: 70-8.
10. Bouvet M, Tsuji K, Yang M, Jiang P, Moossa AR, Hoffman RM. In vivo color-coded imaging of the interaction of colon cancer cells and splenocytes in the formation of liver metastases. Cancer Res. 2006; 66: 11293-7.
11. Park S-Y, Choi J-H, Nam J-S. Targeting cancer stem cells in triple-negative breast cancer. Cancers. 2019; 11: 965.
12. Wang S, Englund E, Kjellman P, Li Z, Ahnlide JK, Rodriguez-Cupello C, et al. CCM3 is a gatekeeper in focal adhesions regulating mechanotransduction and YAP/TAZ signalling. Nat Cell Biol. 2021; 23: 758-70.
